# The CH^–^^3^Σ^+^ Anion: Inelastic Rate Coefficients from Collisions with He at Interstellar Conditions

**DOI:** 10.1021/acs.jpca.2c08021

**Published:** 2023-01-04

**Authors:** Jorge Alonso de la Fuente, Cristina Sanz-Sanz, Lola Gonzalez-Sanchez, Ersin Yurtsever, Roland Wester, Francesco A. Gianturco

**Affiliations:** † Departamento de Quimica Fisica Aplicada, Modulo 14, Universidad Autonoma de Madrid, 28049Madrid, Spain; ‡ Facultad de Ciencias Quimicas, Universidad de Salamanca, Plaza de los Caidos s/n, 37008Salamanca, Spain; ¶ Dept. of Chemistry, Koc University, Rumelifeneri Yolu, Sariyer,TR, 34450Istanbul,Turkey; § Institut fur Ionen Physik und Angewandte Physik, Leopold-Franzens-Universitat, Technikerstrasse 25, 6020Innsbruck, Austria

## Abstract

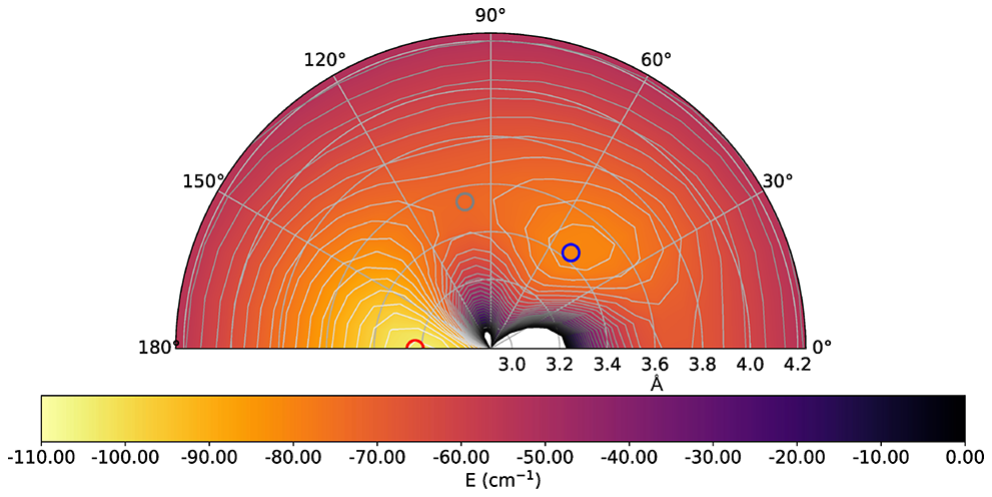

We present accurate *ab initio* calculations on several properties of a gas-phase system of interest in the interstellar medium (ISM), where the title molecular anion has been often surmised but not yet confirmed by observations. The CH^–^^3^Σ^+^ constitutes the smallest term in the series of longer anionic polyynes which have been observed in the ISM (e.g., C_4_H^–^ and several others). Hence, its dynamical behavior in collision with He atoms, one of the most abundant atoms in that environment, can provide quantitative indicators on the changes which can occur in the rotational state population of the title anion when driven by this collision dynamics. We therefore report an accurate evaluation of the full potential energy surface (PES) which acts between the molecular anion in its ground vibrational state and the He atom. The relevant inelastic scattering cross sections and the corresponding inelastic rate coefficients are then computed within a quantum treatment of the collisions. We find that the fairly small values of the final inelastic rate coefficients indicate state-changing processes by collisions to be inefficient paths for modifying the rotational state populations of this anion and therefore to aid its possible observation from direct radiative emission in the microwave region.

## Introduction

For many years the formation of interstellar anions and their possible detection have been an actively studied topic by the astrochemistry community, starting with Dalgarno and McCray^1^ who for the first time explored the role of anions, e.g., H^–^, O^–^, C^–^, CH^–^, C_2_H^–^, CN^–^, and S^–^, in forming simple molecules in interstellar clouds. They concluded at the time that interstellar anions were scarce and their likely contribution to forming molecular species would not be very significant. These results were attributed to the relatively slow rate of formation they had found for the radiative electron attachment (REA) rate coefficients: ∼10^–15^ cm^3^ s^–1^ molecule^–1^ was in fact the average value from their early calculations. A few years later, using a simple statistical model, Herbst^2^ suggested instead that anions could be efficiently formed in dense interstellar clouds. He showed that, for large neutral species with large electron affinity (C_4_H, C_3_N, C_5_N, C_9_N, etc.), the radiative attachment rate could be close to the collision limit ∼10^–7^ cm^3^ s^–1^ molecule^–1^. One of the most relevant results of this work was the prediction of an anion-to-neutral ratio between 1 and 10% which was later supported by astronomical observations. Further calculations with a more sophisticated scattering model were carried out in our group^3^ and found the REA rates for C-rich chains, albeit smaller than those found earlier by Herbst, to be ∼10^–9^ cm^3^ s^–1^ molecule^–1^. Finally, the definite observational proof that anions could exist in the ISM came in 2006, when McCarthy et al.^4^ detected for the first time C_6_H^–^ in the circumstellar envelope IRC+10216 and the dark cloud TMC-1. The anion-to-neutral ratios were in agreement with the early predictions of Herbst: 1–5% for IRC+10216 and 2.5% for TMC-1, thus supporting the prediction that anions could be efficiently formed in the interstellar medium (ISM) by radiative electron attachment or by some other chemical route. The detection of C_6_H^–^ led to the subsequent detection of five other anions, namely, C_4_H^–^, C_8_H^–^, CN^–^, C_3_N^–^, and C_5_N^–^, in several interstellar sources.^5−9^ These observations gave rise to new chemical modeling for the interstellar regions where the anions had been detected, as well as suggesting other possible sources of anionic molecules.^10−12^ These models yielded anion-to-neutral ratios which were reasonably close to observations for the largest anions (C_6_H^–^, C_8_H^–^, C_7_N^–^) but less successful for the smallest anions (CN^–^, C_4_H^–^, C_3_N^–^), hence leading to the preliminary conclusion that REA processes would be less efficient for molecules with fewer degrees of freedom. Further experimental studies carried out earlier in our group^13,14^ on the efficiency of photodetachment mechanisms for the destruction of C-bearing and N-bearing molecular anions have provided new evidence on the importance of starlight in driving their destruction outside chemical networks. Furthermore, the investigations of possible mechanisms of formation involving chemical reactions with other, simpler, and reasonably abundant atomic species like H, O, N, and C have yielded laboratory data on chemical processes which indicated that they could play a role in the formation of various smaller anions, some of them different from those of the earlier observations. They were still either not unequivocally detected or often predicted to be at borderline values of observable column densities.^12,15,16^

A case in point is that of the formation of CH^–^ from an initial neutral-ion reaction of CH_2_ with H^–^. Methylene, CH_2_, as the prototypical carbene is one of the most studied of all reactive intermediates, while the chemistry of its anion, CH_2_^–^ is still almost completely unknown. Early spectroscopic work established the existence of two low-lying electronic states: the ground X^3^B_1_ and the excited a^1^A_1_ states. Spectroscopic studies of the visible electronic band system b^1^B_1_ ← a^1^A_1_ lead to an accurate characterization of the molecule in the a^1^A_1_ state, but for many years the ground-state structure was uncertain.^17,18^ The astrochemical models dealing with anions in experiments at room temperature^19,20^ included the expected formation of C_2_H^–^ from reactions with *e*^–^ with several C-rich molecules but never were able to produce or observe CH^–^ among the anionic products. Thus, the CH^–^ has been often considered as a possible reaction intermediate^19^ but never actually observed or produced in laboratory reactions at room temperature. Additionally, the presence of CH_2_ has been found in the atmosphere of Titan, where CH_2_^–^ has also been detected.^21^ These findings were the reason why we have investigated in our earlier work the possible efficiency of the latter neutral radical to form the CH^–^ species through a different chemical reaction: a reaction with H^–^ that could become another path in the chemical networks for the Titan atmosphere. Our recent study of such a reaction^22^ has in fact suggested that the relevant reaction rates at the expected temperatures of the ISM environments, estimated from our modeling, were of the order of ∼10^–12^ cm^3^ s^–1^ molecule^–1^, which makes the reaction a very likely possibility for the formation of the CH^–^ anion.

In the present study we have therefore decided to further investigate another aspect of the problem: the dynamical evolution of the rotational populations of this interesting anion which is produced from its collisional interaction with He atoms, the latter being among the most abundant atoms in the ISM. We shall therefore present in the next section the details of our *ab initio* calculations of the interaction forces, and the strength of their angular anisotropy, that can drive rotational state-changing collisions from the encounters with He atoms. Quantum Treatment of Rotationally Inelastic Dynamics will briefly discuss the quantum dynamics machinery which we have applied here, while the state-to-state rotationally inelastic cross sections and the ensuing rates will be given and discussed in State-to-State Cross Sections and Rate Coefficients. Inelastic Collisions for CN^–^and CH with He: A Comparison with CH^–^ will show a comparison with the inelastic rates found for other, similar systems, and the final section will give our conclusions.

## Methods

### *Ab Initio* Calculations for Isolated Anion and the 3-Atom Complex

Calculations were carried out using a variety of post-Hartree–Fock *ab initio* methods. In our level of analysis, the molecular species involved are fully optimized using the coupled-cluster approach with full treatment of singles and doubles and an iterative treatment of triples: CCSD(T) as implemented in the MOLPRO suite of codes.^23^ The zero-point-energy (ZPE) corrections were included in all the calculations. We employed increasingly larger basis set expansions, starting with the AV5Z, then the AV6Z and up to complete-basis-set (CBS) with Davidson correction (see ref (24) for a more detailed description of the various acronyms), with differences in energy values never larger than about 10 cm^–1^. Calculations were also carried out at the MRCI level and extrapolating to the CBS expansion level. For the isolated anion we found the results to be identical over the region of the potential minimum, with small discrepancies of less than 3 cm^–1^ in the distance regions near the dissociation limit. Earlier calculations on the title system^25^ used MCSCF methods with a smaller basis set expansion, obtaining fairly similar results. The equilibrium geometry for the isolated anion was found to be about 1.135 Å  not far from an earlier experimental estimate of 1.10(±0.005) Å.^26^ The corresponding dipole moment was found to be 1.645 D when evaluated from the center of charges. In this molecule the charge center is defined as being located at a distance which is six times larger from the H atom than it is from the C atom. This is the same definition as the center of mass for which however the factors are 1 and 12. It is interesting to note here that earlier calculations of this quantity^25^ used the C atom as the center of the reference frame of the dipole, finding a value of 0.770 D. Once our value is shifted to the same reference frame, we found a value of 0.866 D, calculated with the MRCI method at the V6Z level. The value of the dipole moment shifted to the center-of-mass of this molecule turned out to be of 1.288 D at the equilibrium geometry mentioned above. These differences are of course due to the fact that the value of the dipole moment for a charged molecule depends on the definition of the origin of its frame of reference. Hence the different values mentioned above.

**Figure 1:**
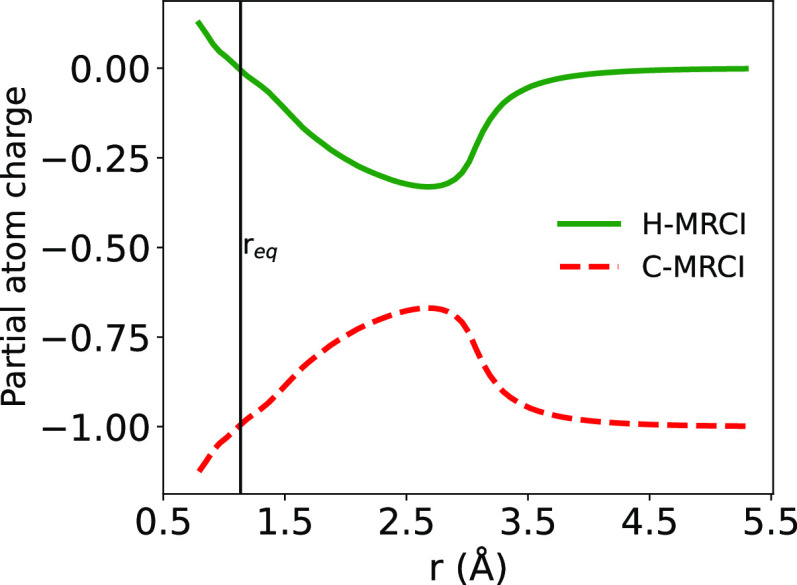
Behavior of the partial excess charge on each of the atoms in the anion as a function of the internuclear distance. Notice the asymptotic values where the negative charge moved entirely on the C atom

**Figure 2:**
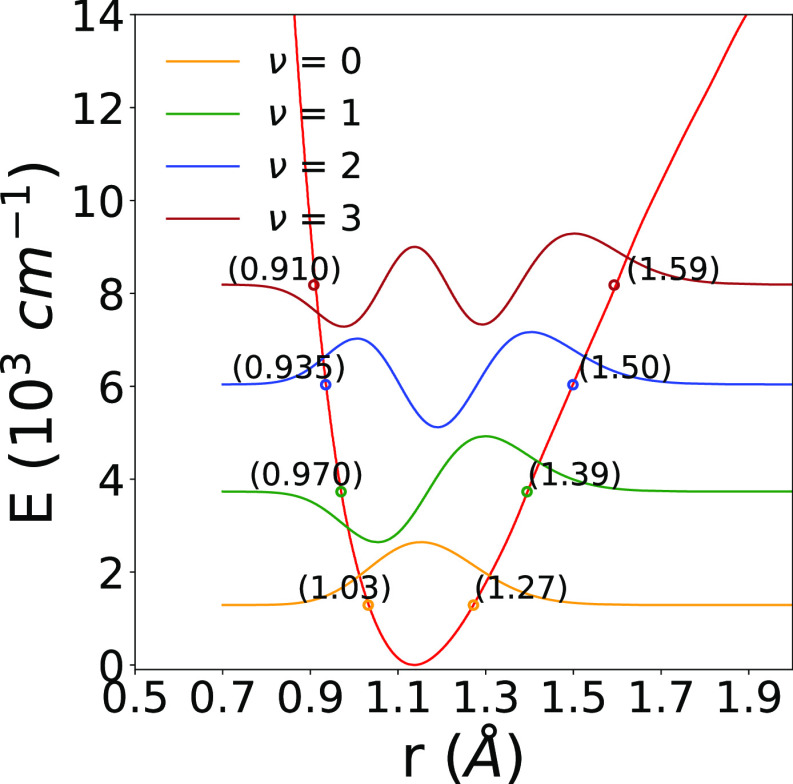
Locations and values of the lowest three vibrational w.f.s with their corresponding turning points.

The data in the Figure 1 report the excess charge, as a function of the intermolecular distance, for the ground electronic state of the present anion. One clearly sees that around the equilibrium distance (1.135 Å) the extra charge is largely located on the carbon atom, a feature that will be significant when discussing the strength of the interaction within the triatomic complex that we shall present below, since the closed-shell He partner will have a different interaction strength with regions containing an excess negative charge. It is also of interest to look at the shape of the potential curve (PEC) in the region of the equilibrium value, showing the lower-lying vibrational states as reported in Figure 2.

Hence, Figure 2 clearly indicates that in the present study of low-T dynamics the molecular anion will be in its ground vibrational state, for which the calculated rotational constant B is 13.679 cm^–1^. This is an unusually large value which shall play an important role in guiding the collision state-changing efficiency discussed below from the interaction with He atoms.

**Figure 3:**
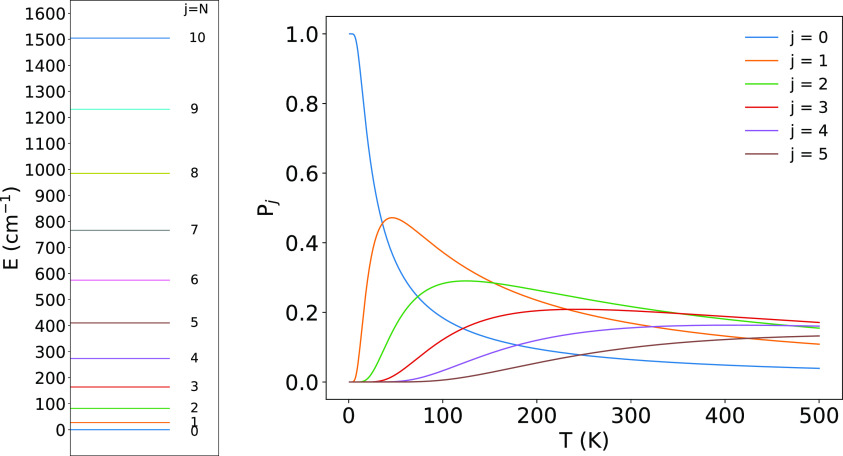
Energy for the first six rotational states of the ν = 0 vibrational states (right) and their Boltzmann distribution when treating the title anion as a pseudo-singlet rotor.

The additional data in Figure 3 show the corresponding spacing of the rotational levels for the ground vibrational state when the present anion is treated as a pseudo-singlet target, an issue which we shall further discuss and analyze in the next section. Figure 3 also reports the distributions of the rotational populations in the temperature range of interest for the present study.

The 3-atom system PES has been calculated at the MRCI level up to the CBS expansion but without including BSSE correction since the latter had very minor effects on the computed total energy values. Within the usual 2D representation of the radial and angular variables of the (*R*, θ) Jacobi space, the former being centered in the c.o.m of the diatomic anion and the latter angle rotated from the H atom side to the C atom side from 0° to 180°. The radial range covered from *R* = 1.59 Å to *R* = 31.75 Å  with steps of 0.102 Å for a total of 295 radial points. The angle values were a total of 19 with steps of 10°. The total number of computed raw points was therefore around 6000.

**Figure 4:**
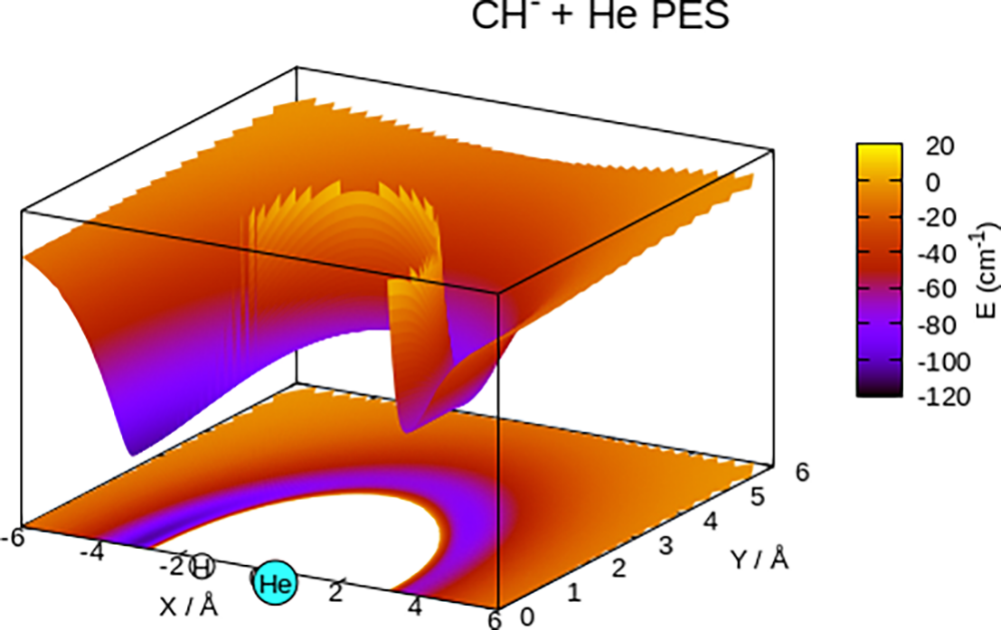
Spatial distribution of the interaction potential energy around the molecular anion. See main text for further details.

The pictorial view in Figure 4 reports the 3D spatial distribution of the interaction potential, where the target anion is placed along the *x*-coordinate. We clearly see that, at the equilibrium geometry, the presence of the excess negative charge largely around the C atom provides the stronger interaction with the neutral, closed-shell He atom on that side, with an intermediate, shallower well located around 50°. Specifically, we found that the global minimum of the PES was for θ = 180° and an energy value of −104.01 cm^–1^, while the saddle point was located at θ = 100° with a depth of −72.77 cm^–1^. There is also a local, shallower minimum at 50° of −81.35 cm^–1^.

**Figure 5:**
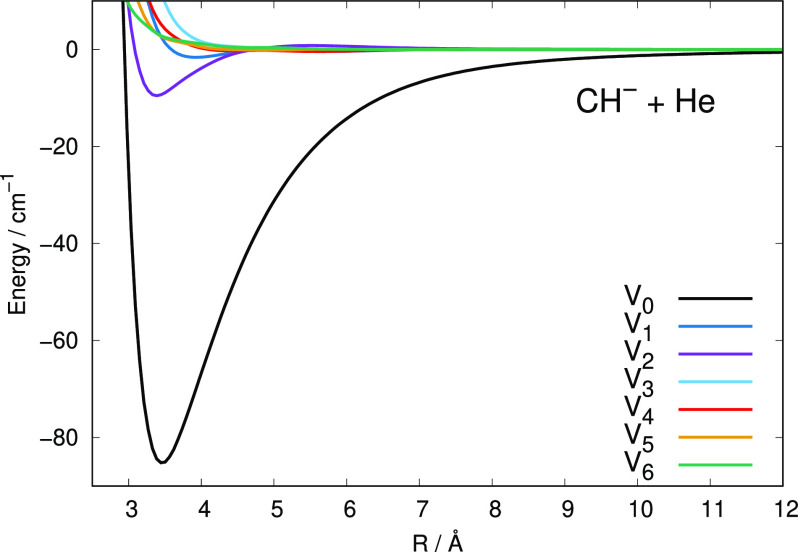
Multipolar expansion coefficients for the computed PES of the present study. See main text for further details.

The various curves given by Figure 5 report an additional representation of the interaction, showing the multipolar coefficients originating from the usual Legendre polynomial orthogonal expansion of the present PES:
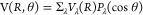
1The above expansion was carried out up to a maximum λ value of 16, and 500 interpolated points were used to describe each radial term, to be used below in the scattering calculations.

The different radial curves in the Figure 5 indicate a marked variation of their coupling strength acting during the quantum dynamics (as discussed in the following section). We see, in fact, that the spherical term *V*_0_ provides the strongest attractive interaction which is extending isotropically around the diatomic target, while the first anisotropic term of importance at short-range is the *V*_2_ that shows its attractive minimum close to that of the spherical term. As we shall discuss later, this term is responsible for the direct dynamical coupling of rotational levels with Δ*N* = 2, and therefore, we expect those inelastic cross sections to play an important role in the excitation/de-excitation processes involving the present system. An important role will be also played by the λ = 1 radial coefficient which is also attractive at short-range and extends further out via the second term in the following long-range expansion:
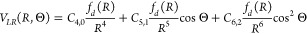
2where the radial expansion term associated with the *V*_λ_ = 1 via the coefficient *C*_5,1_ depends on the permanent dipole of CH^–^ and the polarizability of He:
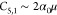
3This term will provide the nonspherical contribution that dies out the most slowly and therefore will be an important long-range driver of excitation probabilities, as we shall show below. We thus expect that the Δ*N* = 1 transitions will dominate the energy transfer processes at the lower temperatures, where long-range forces are important contributors to the dynamical torque driving rotational state-changing collisions.

### Quantum Treatment of Rotationally Inelastic Dynamics

We briefly report below the computational method employed to obtain inelastic cross sections and rate coefficients for the scattering of CH^–^^3^Σ^+^ with He atoms, using the PES discussed in the preceding section. As mentioned earlier, we had found in previous work that reducing the coupling between angular momenta to that of a pseudo-singlet rotor molecular target changes the rate coefficients of less than 5% over the temperature ranges of interest, while strongly reducing the computational times.^27^ We shall therefore employ such a simplification in the quantum dynamics calculations discussed in the following, and we will further make comparative numerical tests to make sure that such an assumption also holds for the present system. The same simplification will apply in the brief outline given next for our computational method, although we shall also be using the full angular momenta coupling as further discussed below in the presentation of our results.

The standard time-independent formulation of the coupled-channel (CC) approach to quantum scattering has been known for many years (for example, see Taylor^28^ for a general textbook formulation), and the more recent literature on the actual computational methods has been also very large. For some selected references produced over the years see refs (29−33). Since we have already discussed our specific computational methodology in many of our earlier publications,^34−36^ only a short outline of it will be given below.

For the case where no chemical modifications are induced in the molecule by the partner projectile, the total scattering wave function can be expanded in terms of asymptotic target rotational eigenfunctions (within the rigid rotor approximation) which are taken to be spherical harmonics and whose eigenvalues are given by *Bj*(*j*+1), where *B* is the rotational constant for the CH^–^ anion mentioned already in the previous section and obtained for the molecule described as a pseudo-singlet rotor: 13.679 cm^–1^ and *j* is the rotational quantum number corresponding to the final *N* quantum number of the triplet state. The channel components for the coupled channel (CC) equations are therefore expanded into products of total angular momentum *J* eigenfunctions and of radial functions to be determined via the solutions of the CC equations.^34,35^ The latter are the familiar set of coupled, second-order homogeneous differential equations:
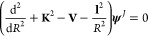
4In the above equations, the **K**^2^ matrix contains the wavevector values for all the coupled channels and the **V** matrix contains the full action of the anisotropic coupling potential. The required scattering observables are then obtained in the asymptotic region where the Log-derivative matrix has a known form in terms of free-particle solutions and unknown mixing coefficients. Therefore, at the end of the propagation one can use the Log-derivative matrix to obtain the K-matrix by solving the following linear system:
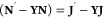
5where the prime signs indicate radial derivatives, and **J**(*R*) and **N**(*R*) are matrices of Riccati–Bessel and Riccati–Neumann functions.^35^ The matrix **Y**(*R*) collects the eigensolutions along the radial region of interest, out of which the Log-derivative matrix is then assembled.^35^ From the K-matrix produced by solving the coupled radial equations, the S-matrix is then directly obtained and from it the state-to-state cross sections.^35^ We have already published an algorithm that modifies the variable phase approach to solve that problem (i.e., the ASPIN code), specifically addressing the latter point, and we defer the interested reader to that reference for further details.^34,35^

In the present calculations we have generated a broad range of state-to-state rotationally inelastic cross sections considering the pseudosinglet states of the CH^–^ molecule. Specifically, the number of rotational states coupled within the dynamics started with *j* = 11 at the lowest collision energies up to 500 cm^–1^, went up to j = 18 between 500 and 1000 cm^–1^, and was extended up to *j* = 25 for the higher energies going to 1500 cm^–1^. The expansion over the *J* values required to converge the individual cross sections went up to *J* = 150 at the highest energies, was *J* = 100 between 500 and 1000 cm^–1^, and was sufficient to be up to *J* = 64 at the lowest energies. The radial range of integration during the propagation of the coupled equations covered values from 1.75 to 3,000.0 Å using a variable number of points which went up to 6,000 at the highest energies but was extended up to 90,000 steps at the lowest energies near the 10^–6^ cm^–1^ threshold. The extrapolation of the initial *ab initio* points was carried out by using the lowest expansion terms that were mentioned before in eqs 2 and 3, involving the He dipole polarizability and the permanent dipole of the CH^–^ target (e.g., see Hernández Vera et al.^27^). The range of *E*_trans_ went from 10^–6^ cm^–1^ to 2000 cm^–1^ using around 2,000 to 4,000 points depending on the considered transition. The maximum collision energy was 1,500 cm^–1^; hence, for *j* = 5 the relative translational energy was about 1,100 cm^–1^.

Once the state-to-state inelastic integral cross sections (σ_*j*→*j*′_) are known, the rotationally inelastic rate coefficients *k*_*j*→*j*′_(*T*) can be evaluated as the convolution of the cross sections over a Boltzmann distribution of the *E*_trans_ values:
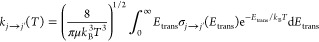
6

The individual rate coefficients were obtained at intervals of 1 K, starting from 5 K and going up to 500 K. The interplay between the number of coupled rotational states and the structural strength of the corresponding PES will be discussed in the following section when the dynamical outcomes will be analyzed.

As mentioned earlier, we have also performed the inelastic collision calculations considering the triplet state of the diatomic molecule. To study the fine structure of the ^3^Σ^+^ CH^–^ molecule, the coupling constants λ and γ were obtained from B3LYP/X2c-QZVP calculations following the method reported earlier in ref (37), which yielded λ = 0.3130 cm^–1^ and γ = −0.01524 cm^–1^. The convergence parameters and radial range of integration were the same as for the pseudo-singlet approach for each of the collision energy considered. In these calculations, the range of *E*_trans_ went from 0.1 cm^–1^ up to 1500 cm^–1^ with around 1000 points per each transition. A comparison between the scattering calculations performed within the two different coupling schemes will be reported in the next section.

## Results and Discussion

### State-to-State Cross Sections and Rate Coefficients

**Figure 6:**
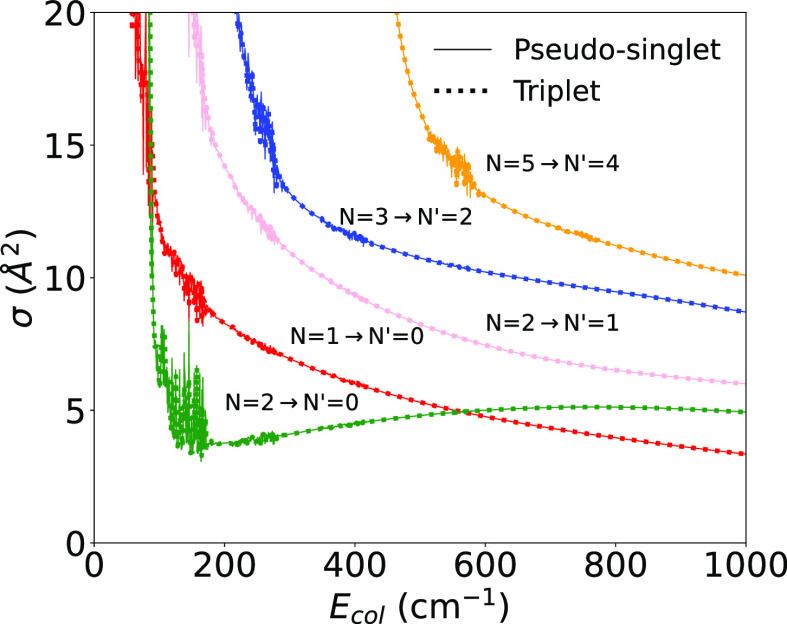
Computed state-to-state rotationally inelastic cross sections, treating the molecular anion either as a full triplet rotor (dotted lines) or as a pseudo-singlet rotor (continuous lines). We report for comparison five different de-excitation processes with Δ*j* = 1 from the lowest 5 levels. See main text for further comments.

The calculated inelastic cross sections involving both excitation and de-excitation (cooling) processes between rotational states of the anion are seen in Figure 6. As mentioned before, we treated the triplet-state of the target as a pseudo-singlet rotor since our earlier experience with calculations involving other molecular ions has shown that the final size of the derived rates did not change much when the proper additional coupling was included (e.g., see ref (27)). To specifically verify this statement we also report in Figure 6 the calculations of state-to-state cross sections which compare the exact triplet calculations (given by dots along the lines) with the pseudo-singlet approach (given by the continuous lines).

The comparison between the calculations reported in Figure 6 confirms very clearly the accuracy of the simplified treatment of rotational coupling when using the pseudo-singlet approach instead of the full triplet coupling. In fact, all the examined cross sections are essentially unchanged, in both value and energy dependence, when the angular momentum coupling is simplified. This finding thus bodes well for considering our final rate coefficients, as reported below, to be essentially exact within the pseudo-singlet treatment.

**Figure 7:**
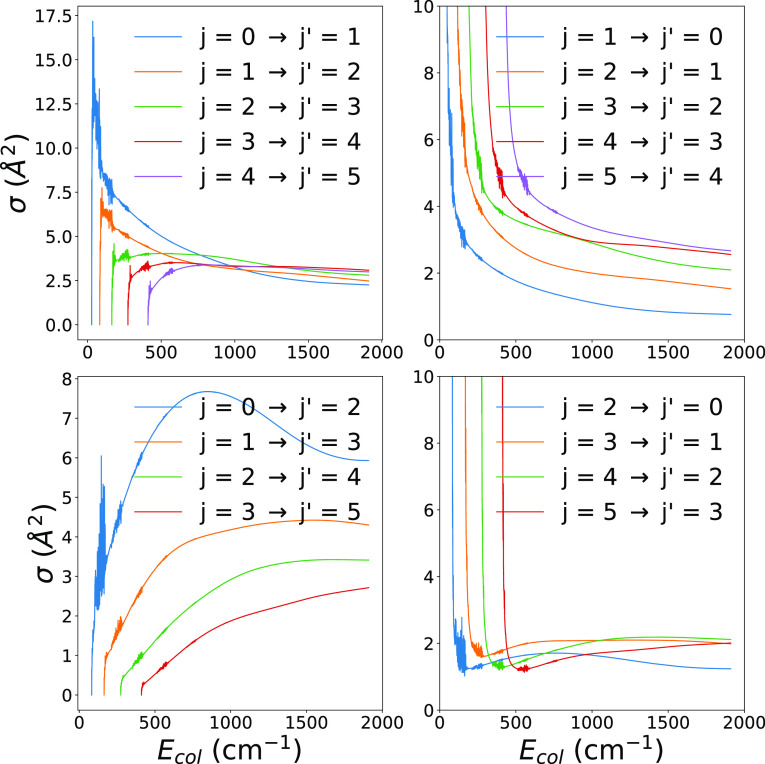
Computed state-to-state rotationally inelastic cross sections, treating the molecular anion as a pseudo-singlet rotor. The upper-left panel reports excitation processes with Δ*j* = 1 from the lowest 5 levels. The lower-left panel shows those with Δ*j* = 2 transitions. The de-excitation cross sections are presented in the upper- and lower-right panels.

If we now turn to the inelastic cross sections reported by the panels of Figure 7, it is interesting to note there that all the excitation cross sections show a marked presence of resonant structures in the lower-energy ranges, indicating that in this system both shape and Feshbach resonances are occurring and play a significant role in the enhancement of the corresponding low-energy rates, as we shall discuss below. As expected from the relative strength of the anisotropic coupling coefficients of the PES, we see that the transitions with Δ*j* = 2 of the lower-left panel become larger at the higher energies in comparison with the excitations with Δ*j* = 1 which are instead the larger ones at the lower energies. This effect is due to the fact that, while near the thresholds the differences in the energy gaps favor the Δ*j* = 1 processes, at the higher energies the strength of the short-range coupling is dominant, thereby favoring the transitions involving directly the *V*_λ_(*R*) with λ = 2. When looking at the corresponding cooling cross sections of the right-side panels, we see that all of them are much smaller than the excitation processes while dramatically increasing near thresholds, where the energy release into the relative motion of the partners, in qualitative classical terms, is much more significant than at the higher collision energies.

**Figure 8:**
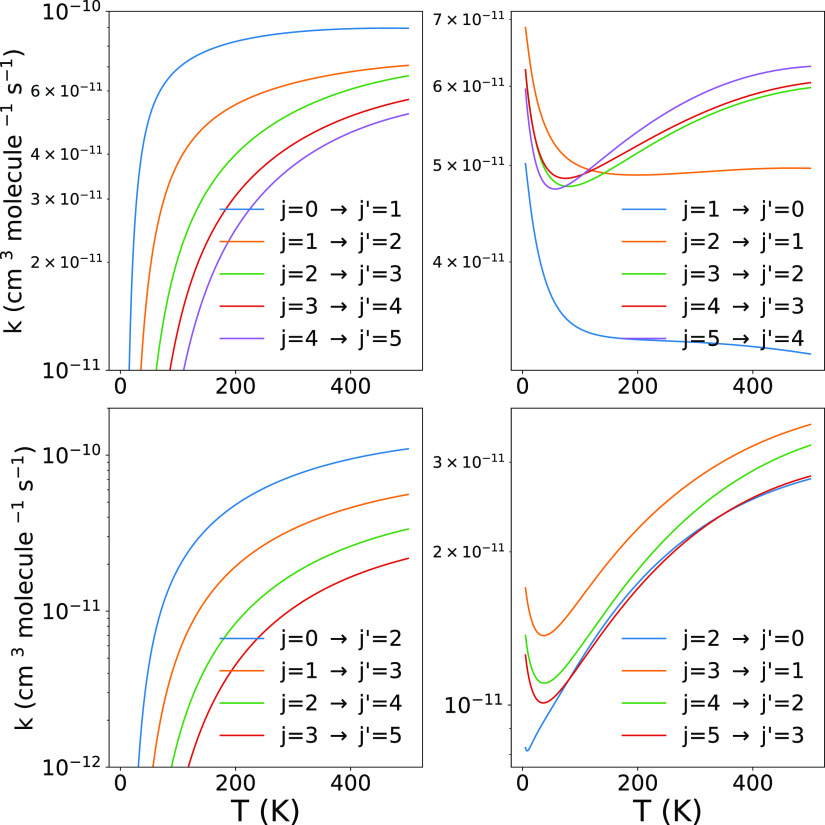
Computed state-to-state rotationally inelastic rate coefficients, treating the molecular anion as a pseudo-singlet rotor. The upper-left panel reports excitation processes with Δ*j* = 1 from the lowest 5 levels. The lower-left panel shows the excitations rates with Δ*j* = 2 transitions. The de-excitation rate coefficients are presented in the upper- and lower-right panels of the figure.

The data reported in Figure 8 present now the corresponding behavior as a function of temperature of the rate coefficients for the same set of processes given before by the inelastic cross sections. As noted earlier, we see that the excitation processes with Δ*j* = 1 are larger than those with Δ*j* = 2 at *T* values below 100 K, while the latter excitations become slightly larger as *T* increases. This effect is linked to the larger coupling strength of the multipolar potential term with λ = 2 in the short-range radial region that affects the higher collision energies, while the long-range coupling is dominated by the dipole term with λ = 1, hence affecting the state-changing collision efficiency at the lower temperatures.

The de-excitation rates reported in the two panels on the left side of the figure indicate the dominance of the inelastic processes which start from the higher rotational states, with those involving Δ*j* = 2 being invariably smaller than those with Δ*j* = 1.

### Inelastic Collisions for CN^–^ and CH with He: A Comparison with CH^–^

While the presence of the CH^–^ in the ISM has not been firmly confirmed, other very similar small species like CH and CN^–^ have been observed in that same environment. In the case of the neutral counterpart, for example, CH has been sighted in the interstellar space, interstellar comets, and stellar atmospheres.^38−40^ More recently, calculations have been carried out on the dynamics of its rotationally inelastic collisions with He atoms^41^ so a comparison of their results with those of its present anionic counterpart would be interesting, as we shall discuss below. In the case of CN^–^, the smallest cynopolyyne to be detected in interstellar environments, modeling and observation have happened in recent years,^42,43^ and the actual calculations of the rotationally inelastic dynamics in collision with He has been studied in detail in our group.^44,45^ Hence, it also becomes interesting to see the differences in behavior between the two smallest anions of the polyyne and cyanopolyyne sequences, the latter of which species has been searched for, and detected, in a variety of ISM environments.

**Figure 9:**
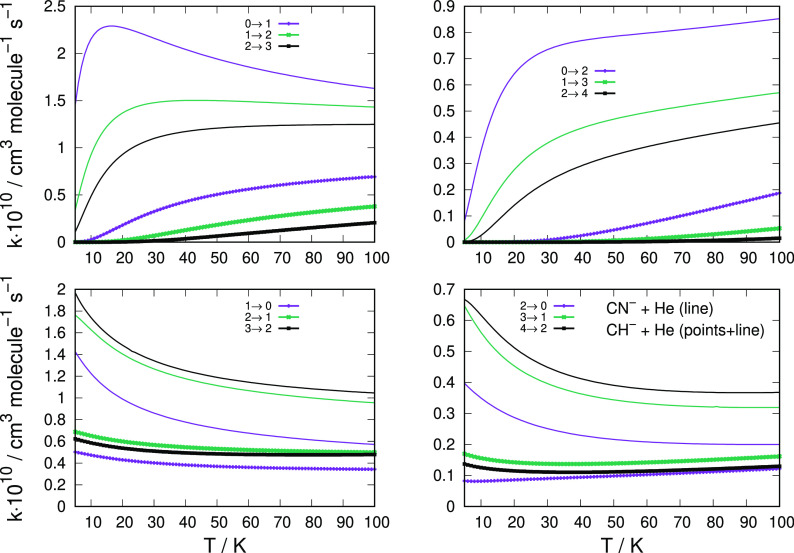
Comparing the computed state-changing rate coefficients between CH^–^ (thick lines) and CN^–^ (thin lines). The data of the former anion are from the present calculations while those of the latter are from our earlier work.^44^ The two upper panels report rotational excitation processes, while the lower two panels show de-excitation processes.

We report in Figure 9 a comparison of the computed inelastic rate coefficients for these two molecular anions, taking into consideration different transitions and a broad range of temperature values.

**Figure 10:**
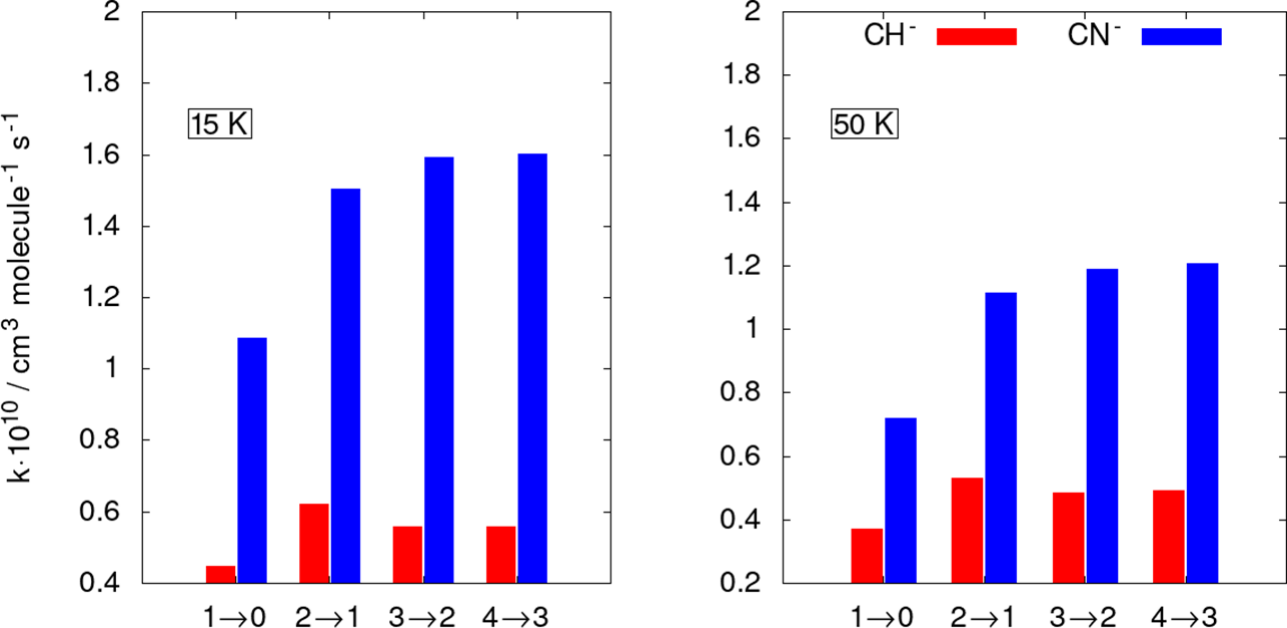
Computed state-changing rate coefficients for CH^–^ (red sticks) and CN^–^ (blue sticks). The data of the former anion are from the present calculations, while those of the latter are from our earlier work.^44^ The two panels report rotational de-excitation processes at two different temperatures and for the lowest four excited rotational states of the two molecular systems.

To further show pictorially the differences in size between the inelastic rates in the two different anions, we report in Figure 10 a “stick” view of the rate coefficients at two different temperatures.

The data in Figures 9 and 10 clearly show that the ISM-observed cynopolyyne, i.e., the CN^–^ anion, exhibits an excitation/cooling efficiency which is much larger than in the case of the CH^–^ anion: all the shown rate coefficients for the latter target are in fact nearly 1 order of magnitude smaller at all the considered temperatures. Such differences are linked to differences in the structural properties of the two polar rotors. In the case of the CN^–^, the rotational constant is nearly 1 order of magnitude smaller (1.872 cm^–1^) in comparison with that for CH^–^ (13.70 cm^–1^). This means that the markedly larger energy gaps for inelastic state-to-state collisions involving CH^–^ will make much less effective the role of the He atomic partners in changing rotational populations with respect to the case of the CN^–^ anion. If we also notice that the reduced mass values which appear in eq 6 for the derivation of the rate coefficients are very similar in the two systems, with a value of 3.061377 amu for the CH^–^/He and of 3.46860 amu for the CN^–^/He, we can conclude that the crucial difference in their dynamics is the large differences in the energy gaps between the states involved in the inelastic processes. Hence, the possible departure from local thermal equilibrium (LTE) conditions for the former polar anion is less likely to occur via collisions with He atoms than would be the case of the CN^–^ anion, a feature which we have discussed in detail for this molecule in our earlier work.^44^

The ground electronic state configuration of CH is 1σ^2^2σ^2^3σ^2^1π^1^, and therefore, the ground electronic state is of ^2^Π symmetry. The methylidene is a Hund’s case (b) radical in its lowest vibrational level of its ground electronic state, with the ^2^Π_1/2_ spin state being lower than the ^2^Π_3/2_. These two states are labeled F2 and F1, respectively. The electronic orbital angular momentum, L, is coupled with the rotational angular momentum of the bare nuclei, R, to form the total (excluding nuclear and electron spin) angular momentum, N. N is then coupled with the electron spin angular momentum, S, giving the total angular momentum, J. In Hund’s case (b), J = (*N* ± 1/2) for the F1 and F2 manifolds, respectively. J is coupled with the nuclear spin of H (I = 1/2) to give the grand total angular momentum, F. The calculations of the state-to-state rotationally inelastic rates have been carried out for the collisions of the CH neutral molecule with He atoms^41^ for a variety of changes of the lower quantum numbers and over a range of temperatures up to 300 K. It turned out that all such rates were practically negligible at the lowest temperatures and reached their largest values of ∼10^–13^ cm^3^s^–1^ only above about 200 K. Such values are therefore more than 2 orders of magnitude smaller than those we have obtained for the anion of the same molecule in temperature ranges relevant for the ISM conditions, thereby confirming the essentially marginal significance of the collision-driven paths to rotational state-changes induced in the neutral by the He present in these environments. The comparison also clearly confirms the much larger rate coefficients which occur in collisions involving charged molecular partners as opposed to the neutral ones.

## Present Conclusions

We have presented in this work extensive *ab initio* calculations involving the CH^–^ anion, known to be the smallest term of the polyyne anionic chains for which larger terms have been observed in the interstellar environments, as discussed in the Introduction. This small anion is of interest also for the study of its properties and behavior in cold traps, where its rotational population can be controlled by using He as a buffer gas. We have therefore carried out the full evaluation of the 2D PES describing its interaction with neutral He, also an important partner in the panoply of atoms identified in the ISM.

The interaction forces produced by our calculations are in turn employed to yield the low-energy behavior of the excitation/de-excitation probabilities involving its rotational states and during collisions with He atoms. The quantum evaluation of the relevant dynamics for these probabilities allows us to further get the corresponding rotational state-changing rate coefficients over a range of temperatures relevant for the ISM environments where this molecule is surmised to be present, albeit not yet uniquely detected. It turns out, in fact, that the very large energy spacing between rotational states makes energy-transfer processes by collision at low-*T* markedly inefficient in comparison with those involving other anions of similar size, like CN^–^. This difference could therefore provide a possible reason why only the latter anion has been so far detected in interstellar environments (see refs (42 and 43)). We can, in fact, argue that the out-of-equilibrium (i.e., away from LTE conditions) rotational populations of the present molecule via collisions with He is not a process that would be of primary importance within the kinetic modeling of such small anion in the ISM. These calculations therefore provide a quantitative estimate, from first-principles, of the collision efficiency of the title system in interaction with He, a species also present in the same environments. Our computed rate coefficients could be used in further modeling rotational population evolution of this specific species within larger chemical networks, while our study suggests the smallness of collision-driven probabilities as one of the possible reasons for the difficulty in detecting the present anion via microwave emission spectroscopy from the excited rotational states which would otherwise become populated via more efficient state-changing processes.

## Data Availability

All the data that support the findings of this study are available within the article itself and in its Supporting Information.

## Supporting Information Available

Raw *ab initio* PES of the rigid rotor CH^–^···He potential, the computed rotationally inelastic cross sections, and inelastic rate coefficients from the 2D-RR-PES employed in this work; the computed potential energy curve raw points for the isolated anion and the dipole moment values computed as a function of the bond values: (*i*) CHm: containing the PEC at aV5Z and aV6Z, with and without BSSE correction, and at CBS extrapolation level; (*ii*) CHmHe: containing the PES raw points at aV5Z, aV6Z and CBS extrapolation level; (*iii*) cross sections: containing the cross-section values for the inelastic collisions for the pseudo-singlet approach and for the triplet state; (*iv*) RATES: containing the rate coefficients (only for the pseudosinglet approach); (*v*) dipole-moment: containing computed DM values as a function of bond distances and using different reference frames (PDF)

## References

[ref1] Dalgarno A.; McCray R. A. The formation of interstellar molecules from negative ions. ApJ. 1973, 181, 95–100. 10.1086/152032.

[ref2] Herbst E. Can. negative molecular ions be detected in dense interstellar clouds?. Nature 1981, 289, 656–657. 10.1038/289656a0.

[ref3] Carelli F.; Satta M.; Grassi T.; Gianturco F. Carbon-rich molecular chains in protoplanetary and planetary atmospheres: quantum mechanisms and electron attachment rates for anion formation. ApJ. 2013, 774, 97–105. 10.1088/0004-637X/774/2/97.

[ref4] McCarthy M. C.; Gottlieb C. A.; Gupta H.; Thaddeus P. Laboratory and astronomical identification of the negative molecular ion C_6_H^–^. ApJ. 2006, 652, L141–145. 10.1086/510238.

[ref5] Sakai N.; Sakai T.; Osamura Y.; Yamamoto S. Detection of *C*_6_*H*^–^ toward the low-mass protostar IRAS 04368 + 2557 in L1527. ApJ. 2007, 667, L65–69. 10.1086/521979.

[ref6] Remijan A. J.; Hollis J. M.; Lovas F. J.; Cordiner M. A.; Millar T. J.; Markwick-Kemper A. J.; Jewell P. R. Detection of *C*_8_*H*^–^ and comparison with *C*_8_*H* toward IRC + 10216. ApJ. 2007, 664, L47–52. 10.1086/520704.

[ref7] Cernicharo J.; Guelin M.; Agundez M.; Kawaguchi K.; McCarthy M.; Thaddeus P. Astronomical detection of *C*_4_*H*^–^, the second interstellar anion. A&A 2007, 467, L37–L40. 10.1051/0004-6361:20077415.

[ref8] Cernicharo J.; Guelin M.; Agundez M.; McCarthy M. C.; Thaddeus P. Detection of *C*_5_*N*^–^ and vibrationally excited *C*_6_*H* in IRC + 10216. ApJ. 2008, 688, L83–86. 10.1086/595583.

[ref9] Agundez M.; Cernicharo J.; Guelin M.; Kahane C.; Roueff E.; Klos J.; Aoiz F. J.; Lique F.; Marcelino N.; Goicoechea J. R.; et al. Astronomical identification of *CN*^–^, the smallest observed molecular anion. A&A 2010, 517, L2–6. 10.1051/0004-6361/201015186.

[ref10] Millar T. J.; Herbst E.; Bettens R. P. A. Large molecules in the envelope surrounding IRC+10216. MNRAS 2000, 316, 195–203. 10.1046/j.1365-8711.2000.03560.x.

[ref11] Millar T. J.; Walsh C.; Cordiner M. A.; Chuimin R. N.; Herbst E. Hydrocarbon anions in interstellar clouds and circumstellar envelopes. ApJ. 2007, 662, L87–L90. 10.1086/519376.

[ref12] Harada N.; Herbst E. Modeling carbon chain anions in L1527. ApJ. 2008, 685, 272–279. 10.1086/590468.

[ref13] Best T.; Otto R.; Trippel S.; Hlavenka P.; von Zastrow A.; Eisenbach S.; Jezouin S.; Wester R.; et al. Absolute photodetachment cross-section measurements for hydrocarbon chain anions. ApJ. 2011, 742, 63–69. 10.1088/0004-637X/742/2/63.

[ref14] Kumar S.; Hauser D.; Jindra R.; Best T.; Rouchka S.; Geppert W.; Millar T.; Wester R. Absolute photodetachment cross-section measurements for hydrocarbon chain anions. ApJ. 2013, 776, 25–32. 10.1088/0004-637X/776/1/25.

[ref15] Yang Z.; Cole C. A.; Martinez J. O.; Carpenter M. Y.; Snow T. P.; Bierbaum V. M. Experimental And Theoretical Studies Of Reactions Between H Atoms And Nitrogen-Containing Carbanions. ApJ. 2011, 739, 19–25. 10.1088/0004-637X/739/1/19.

[ref16] Wang Z. C.; Cole C. A.; Demarais N. J.; Snow T. P.; Bierbaum V. M. Reactions of Azine Anions with Nitrogen and Oxygen Atoms: Implications for Titan’s Upper Atmosphere and Interstellar Chemistry. J. Am. Chem. Soc. 2015, 137, 10700–10709. 10.1021/jacs.5b06089. 26281019

[ref17] Leopold D. G.; Murray K. K.; Stevens Miller A. E.; Lineberger W. C. Methylene: A study of the *X*^3^*B*_1_ and *a*^1^*A*_1_ states by photoelectron spectroscopy of *CH*_2_ and *CD*_2_. J. Chem. Phys. 1985, 83, 4849–4865. 10.1063/1.449746.

[ref18] Bunker P. R.; Sears T. J. Analysis of the laser photoelectron spectrum of *CH*_2_^–^. J. Chem. Phys. 1985, 83, 4866–4871. 10.1063/1.449747.

[ref19] Martinez O. J.; Yang Z.; Demarais N. J.; Snow T. P.; Bierbaum V. M. Gas-Phase Reactions Of Hydride Anion *H*^–^. ApJ. 2010, 720, 173–181. 10.1088/0004-637X/720/1/173.

[ref20] Walsh C.; Harada N.; Herbst E.; Millar T. J. The Effects of Molecular Anions on the Chemistry of Dark Clouds. ApJ. 2009, 700, 752–759. 10.1088/0004-637X/700/1/752.

[ref21] Vuitton V.; Lavvas P.; Yelle R. V.; Galand M.; Wellbrock A.; Lewis G. R.; Coates A. J.; Wahlund J. E. Negative ion chemistry in Titan’s upper atmosphere. Planetary and Space Science 2009, 57, 1558–1565. 10.1016/j.pss.2009.04.004.

[ref22] Yurtsever E.; Satta M.; Wester R.; Gianturco F. On the Formation of Interstellar CH^–^ Anions: Exploring Mechanism and Rates for CH_2_ Reacting with H^–^. J.Phys.Chem.A 2020, 124, 5098–5105. 10.1021/acs.jpca.0c02412. 32463233 PMC7322726

[ref23] Werner H. J.; Knowles P. J.; Knizia G.; Manby F. R.; Schutz M. Molpro: a general-purpose quantum chemistry program package. WIREs Computational Molecular Science 2012, 2, 242–253. 10.1002/wcms.82.

[ref24] Sylvetsky N.; Kesharwani M.; Martin J. The aug-cc-pVnZ-F12 basis set family: Correlation consistent basis sets for explicitely correlated benchmark calculations on anions and noncovalent complexes. J. Chem. Phys. 2017, 147, 134106–134112. 10.1063/1.4998332. 28987100

[ref25] Maenz U.; Zilch A.; Rosmus P.; Werner H.-J. MCSCF-CI calculations of Infrared transition probabilities in the CH^–^ and NH^–^. J. Chem. Phys. 1986, 84, 5037–5044. 10.1063/1.450653.

[ref26] Kasdan A.; Herbst E.; Lineberger W. Laser Photoelectron Spectroscopy of CH^–^. Chem.Phys.Lett. 1975, 31, 78–86. 10.1016/0009-2614(75)80062-5.

[ref27] Hernández Vera M.; Gianturco F. A.; Wester R.; da Silva H. Jr.; Dulieu O.; Schiller S. Rotationally inelastic collisions of H_2_^+^ions with He buffer gas: Computing cross sections and rates. J. Chem. Phys. 2017, 146, 124310–124323. 10.1063/1.4978475. 28388146

[ref28] Taylor J. R. Scattering Theory The Quantum Theory of Nonrelativistic Collisions; Dover, 2006.

[ref29] Arthurs A. M.; Dalgarno A. The theory of scattering by a rigid rotator. Proc. R. Soc. A 1960, 256, 540–549. 10.1098/rspa.1960.0125.

[ref30] Secrest D. Rotational Excitation-I: The Quantal Treatment. Bernstein R. B., Ed.; Atom - Molecule Collision Theory; Plenum: New York, 1979.

[ref31] Kouri D.; Hoffman D. A Tutorial on Computational Approaches to Quantum Scattering. In Multiparticle Quantum Scattering With Applications to Nuclear, Atomic and Molecular Physics; Truhlar D. G., Simon B. Eds.; Springer: New York, NY, 1997; Vol. 89.

[ref32] Hutson J. Coupled channel methods for solving the bound-state Schroedinger equation. Comput. Phys. Commun. 1994, 84, 1–18. 10.1016/0010-4655(94)90200-3.

[ref33] Gianturco F. The transfer of molecular energies by collisions: recent quantum treatments. Lect. Notes Chem.; Springer Verlag: Berlin, 1979.

[ref34] Martinazzo R.; Bodo E.; Gianturco F. A. A Modified Variable-Phase Algorithm for Multichannel Scattering with Long-range Potentials. Comput. Phys. Commun. 2003, 151, 187–197. 10.1016/S0010-4655(02)00737-3.

[ref35] López-Durán D.; Bodo E.; Gianturco F. A. ASPIN: An All Spin Scattering Code for Atom-molecule Rovibrationally Inelastic Cross Sections. Comput. Phys. Commun. 2008, 179, 821 10.1016/j.cpc.2008.07.017.

[ref36] González-Sánchez L.; Gianturco F. A.; Carelli F.; Wester R. Computing Rotational Energy Transfers of OD^–^/OH^–^ in Collisions with Rb: Isotopic Effects and Inelastic Rates at Cold Ion-trap Conditions. New. J. Phys. 2015, 17, 123003–123011. 10.1088/1367-2630/17/12/123003.

[ref37] Franzke Y. J.; Spiske L.; Pollak P.; Weigend F. Segmented contracted error-consistent basis sets of quadruple-ζ valence quality for one-and two-component relativistic all-electron calculations. J. Chem. Theory Comput. 2020, 16, 5658–5674. 10.1021/acs.jctc.0c00546. 32786897

[ref38] Ziurys L.; Turner B. Detection of Interstellar Rotationally-excited CH. ApJ. 1985, 292, L25–33. 10.1086/184466.

[ref39] Masseron T.; Plez B.; Van Eck S.; Colin R.; Datoutidis I.; Godefroid M.; Coheur P.-F.; Bernath P.; Jorissen A.; Christlieb N. CH in interstellar atmospheres: an extensive linelist. A&A 2014, 571, A47 10.1051/0004-6361/201423956.

[ref40] Mendis D.; Ip W.-H. The neutral atmospheres of Comets. Astrophys. Space Sci. 1976, 39, 335 10.1007/BF00648334.

[ref41] Marinakis S.; Dean I.; Klos J.; Lique F. Collisonal excitation of CH(*X*^2^Π) by He: new ab initio potential energy surfaces and scattering calculations. Phys. Chem. Chem. Phys. 2015, 17, 21583–21595. 10.1039/C5CP03696H. 26220835

[ref42] Agúndez M.; Cernicharo J.; Guélin M.; Kahane C.; Roueff E.; Klos J.; Aoiz F. J.; Lique F.; Marcelino N.; Goicoechea J. R.; et al. Astronomical identification of CN-, the smallest observed molecular anion. A&A 2010, 517, L2–14. 10.1051/0004-6361/201015186.

[ref43] Cernicharo J.; Agúndez M.; Gúelin M. The Molecular Universe; Cambridge University Press: Cambridge, 2011; p 237.

[ref44] González-Sánchez L.; Mant B. P.; Wester R.; Gianturco F. A. Rotationally inelastic Collisions of CN^–^ with He: Computing Cross Sections and Rates in the Interstellar Medium. ApJ. 2020, 897, 75–88. 10.3847/1538-4357/ab94a0.

[ref45] González-Sánchez L.; Yurtsever E.; Mant B. P.; Wester R.; Gianturco F. A. Collision-driven state-changing efficiency of different buffer gases in cold traps: He(^1^*S*) Ar (^1^*S*) and p-H_2_(^1^ Σ) on trapped CN^–^(^1^ Σ). Phys. Chem. Chem. Phys. 2021, 23, 7703–7715. 10.1039/D0CP03440A. 32804174

