# Correction to “The CH^–^ ^3^Σ^–^ Anion: Inelastic Rate Coefficients from Collisions with He at Interstellar Conditions”

**DOI:** 10.1021/acs.jpca.3c03005

**Published:** 2023-06-06

**Authors:** Jorge Alonso de la Fuente, Cristina Sanz-Sanz, Lola Gonzalez-Sanchez, E. Yurtsever, Roland Wester, Francesco A. Gianturco

CH^–^ (^3^Σ^–^) constitutes the smallest term in the series of longer anionic polyynes that have been observed in the ISM (e.g., C_4_H^–^ and several others). We have just discovered a computational error in the results in the original paper regarding the potential energy surface between the CH^–^ (^3^Σ^–^) anion and the neutral He atom. The origin of this error is explained in the Introduction, and new, corrected calculations are presented and discussed. The relevant inelastic scattering cross sections and the corresponding inelastic rate coefficients are then computed again and compared with the earlier results. We find that we now obtain even smaller values for the final inelastic rate coefficients, thereby correctly confirming that possible state-changing processes induced by collisions would be a very inefficient path for modifying the rotational state populations of this anion and therefore be of marginal importance for aiding its possible observation.

## Introduction: Origin of the Computational Error

In the original manuscript, we included the calculations of an ab initio potential energy surface (PES) corresponding to the first triplet excited state of the triatomic system of HeCH^–^. This state dissociates into He(^1^S) + CH^–^(A ^3^Π) channel. The diatomic state, CH^–^(A ^3^Π), dissociates into C(^3^P) + H^–^(^1^S), the state dissociating into the complete dissociation with the negative charge in the H atom. On the other hand, the correct ground electronic state of the HeCH^–^ complex dissociates instead into He(^1^S) + CH^–^(X ^3^Σ^–^), whose diatomic fragment, CH^–^(X ^3^Σ^–^), asymptotically goes to C^–^(^4^S) + H(^2^S), which in turn yields a complete dissociation with the negative charge on the C atom. In the original work, we had calculated several electronic states of the triatomic system, including several whose complete dissociations yield the ground electronic states of the atomic fragments with the negative charge either in the C or in the H atom. A pictorial view of the calculated CH^–^ lower electronic roots is reported in Figure 1. Note that the lowest two electronic states of CH^–^ reported there have essentially the same value of their equilibrium bond length, a feature that has helped to confirm the properties of the isolated anion discussed in the earlier publication.

**Figure 1:**
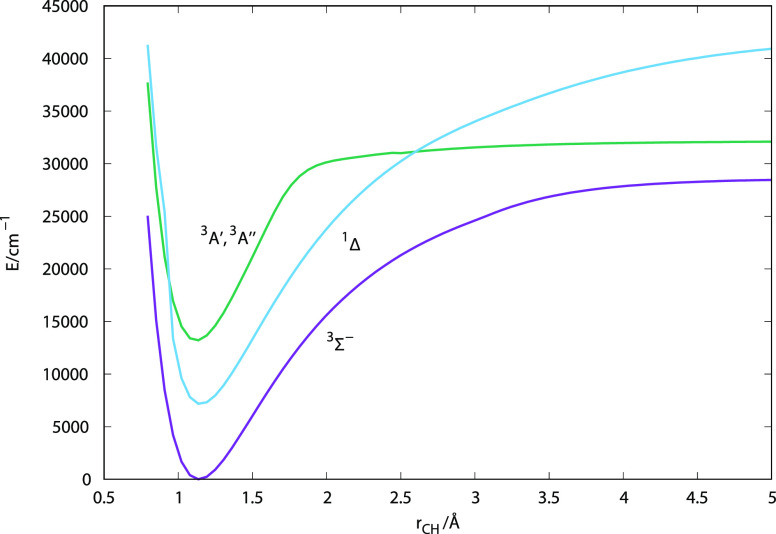
Pictorial view of the isolated CH^–^ molecular anion’s lower electronic states. See the main text for further details.

If we now consider that our triatomic calculations were done using *C*_*s*_ symmetry, the ground electronic state of HeCH^–^, a combination of He(^1^S) + CH^–^(X ^3^Σ^–^), belongs to the A″ irreducible representation. On the other hand, the state we had erroneously considered in the original manuscript was given by a combination of He(^1^*S*) + CH^–^(A ^3^Π), which instead belongs to both irreducible representations, A′ + A″. Our computational error was therefore that of using the ^3^A′ electronic state, instead of ^3^A″ one, for constructing the PES to be used in our dynamics calculations: that former PES, however, is clearly not the ground electronic state of the triatomic system which we wanted to generate.

## Methods

### The New Ab Initio Calculations for Lowest Root of the 3-Atom Complex

Calculations were carried out using a variety of post-Hartree–Fock ab initio methods. In our level of analysis, the molecular species involved are fully optimized using the coupled-cluster approach with full treatment of singles and doubles and an iterative treatment of triples: CCSD(T) as implemented in the MOLPRO suite of codes.^1^ We employed increasingly larger basis set expansions, starting with the AV5Z, then the AV6Z, and up to complete-basis-set (CBS) with Davidson correction (see ref (2) for the more detailed description of the various acronyms), with differences in energy values never larger than about 10 cm^–1^. Calculations were also carried out at the MRCI level and extrapolating to the CBS expansion level. For the isolated anion we found the results to be identical over the region of the potential minimum. However, the CCSD(T) calculations produced earlier the incorrect dissociation channel: an analysis of the partial charges on the asymptotic fragments always produced the negative excess charge on the H atom. In order to produce the PES with the correct asymptotic behavior we decided to perform the calculations via the MRCI method up to CBS expansion and Davidson correction. Earlier calculations on the title system^3^ used MCSCF-CI methods with a smaller basis set expansion, obtaining fairly similar results. The equilibrium geometry for the isolated anion was found to be about 1.135 Å, not far from an earlier experimental estimate of 1.10(±0.005) Å.^4^ The corresponding dipole moment was found to be 1.645 D when evaluated from the center of charges. In this molecule the charge center is defined as being located at a distance which is six times larger from the H atom than it is from the C atom. This is the same definition as the center of mass for which, however, the factors are 1 and 12. It is interesting to note here that earlier calculations of this quantity^3^ used the C atom as the center of the reference frame of the dipole, finding a value of 0.770 D. Once our value is shifted to the same reference frame, we found a value of 0.866 D, calculated with the MRCI method at the AV6Z level. The value of the dipole moment shifted to the center-of-mass of this molecule turned out to be of 1.288 D at the equilibrium geometry mentioned above. These differences are of course due to the fact that the value of the dipole moment for a charged molecule depends on the definition of the origin of its frame of reference. Hence the different values mentioned above.

None of the above calculations regarding the isolated molecular anion are involved in the present Correction. All the data reported in the original publication for the isolated CH^–^ partner are therefore correct and accurate.

The ground electronic state of the 3-atom system PES has been calculated at the MRCI level up to the CBS expansion but without including BSSE correction since the latter had very minor effects on the computed total energy values. Within the usual 2D representation of the radial and angular variables of the (*R*, θ) Jacobi space, the former is centered in the c.o.m. of the diatomic anion and the latter angle rotated from the H atom side to the C atom side from 0° to 180°. The radial range covered from *R* = 1.59 Å to *R* = 31.75 Å, with steps 0.102 Å for a total of 295 radial points. The angle values were a total of 19 with steps of 10°. The total number of computed raw points was therefore around 6000.

The pictorial view in Figure 2 reports the spatial distribution of the correct ground electronic state interaction potential in 3D, where the target anion is placed along the X-coordinate, the latter being centered at the center-of-mass of the diatom, with the He approaching the H atom at 0°. We clearly see that, at the equilibrium geometry, the presence of the excess negative change largely around the C atom provides the stronger interaction with the neutral, closed-shell He atom on that side. Specifically, we found two global minima of the PES were for θ = 170° and for θ = 20°, while the saddle point was located at θ = 80° with a much reduced depth of about −10 cm^–1^. The deeper attractive well on the C-end of the target is around −50 cm^–1^, while the one on the H-end of the molecule is around −30 cm^–1^. These values turn out to be smaller than those found in the earlier calculations which had chosen the incorrect root, thus indicating that we expect, on the whole, a markedly weaker interaction of the electronic ground state of the anion with the impinging He atom.

**Figure 2:**
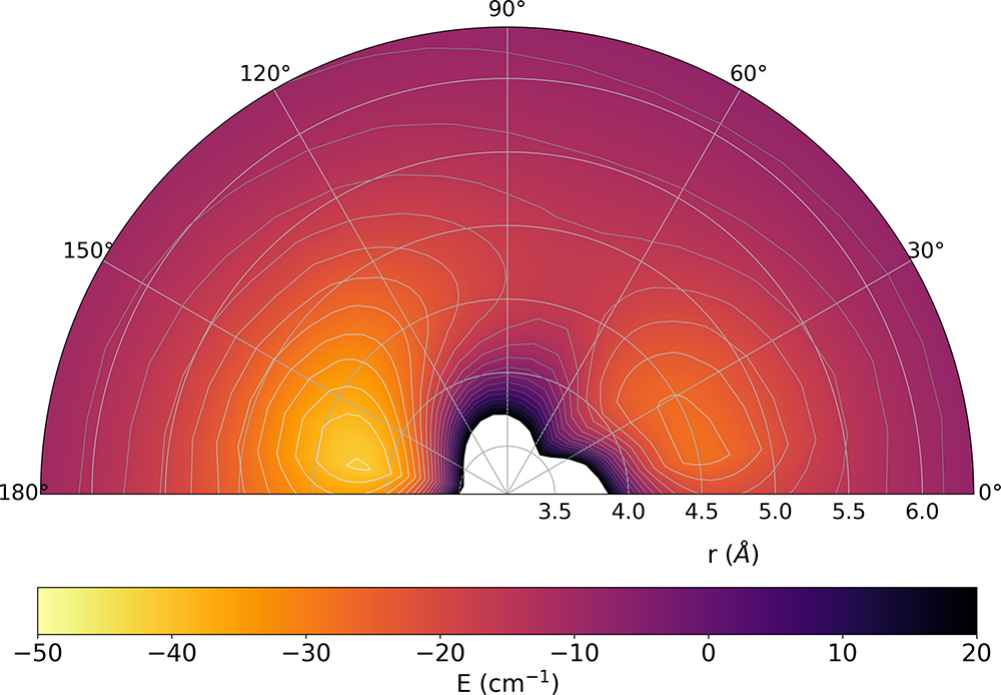
Spatial distribution of the triatomic PES given via energy isolines for the ^3^A″ electronic ground state. The He projectile approaches the H-end of the anion at 0°. Energy in cm^–1^ and distances in Å. See the main text for further details.

A qualitative comparison between the current, and correct, ground electronic state PES for the title system and the values we had obtained before, but belonging to its first excited electronic state, could also be had by looking at three significant angular cuts of the two systems, as reported in Figure 3.

**Figure 3:**
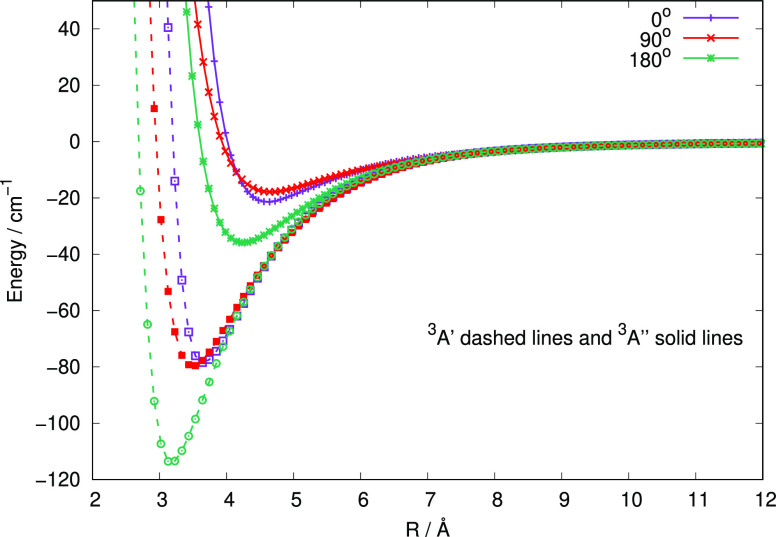
Comparison of three angular cuts of the two PESs related to the present ground state calculations (solid lines) in comparison with the first excited electronic state (dashed lines) we had computed before. See the main text for further details.

One clearly sees in Figure 3 how the overall strength of the interaction between the anion and the neutral atom now results in a very marked reduction when going from the excited electronic state A′ (dashed lines) to the correct ground electronic state ^3^A″ (solid lines). All the well depths located slightly further out along the radial distance are now much shallower, with minimum values which are uniformly a factor of 4 smaller than those for the excited electronic state. These differences, as we shall show below, have marked consequences on the quantum inelastic dynamics of the present work.

The various curves given by the Figure 4 additionally report a further comparison between a different representation of the interaction, i.e., the one showing the multipolar coefficients that are originating from the usual Legendre polynomial orthogonal expansion of the present PES:
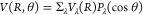
1The above expansion was carried out up to a maximum λ value of 16 and 500 interpolated points were used to describe each radial term, to be used below in the scattering calculations.

**Figure 4:**
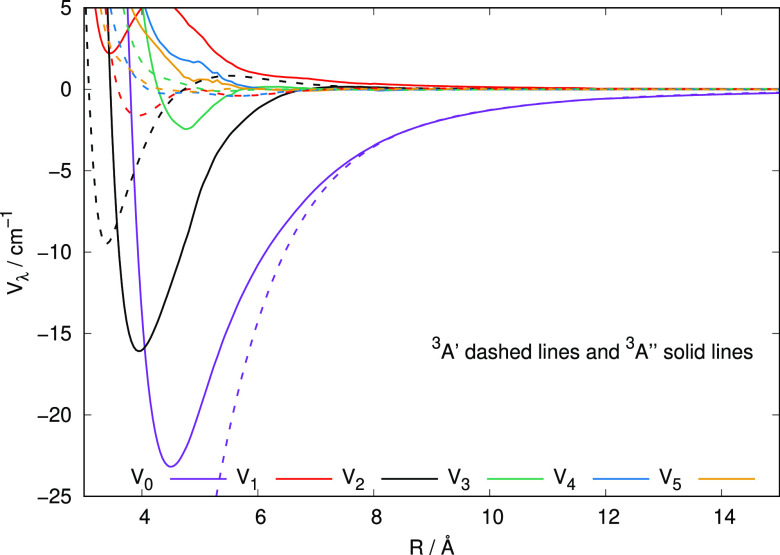
Comparison between the multipolar expansion coefficients for the computed HeCH^–^ PESs of the present study. The solid lines represent the ground electronic state roots while the dashed lines report the same coefficients obtained earlier for the first excited electronic state. See the main text for further details.

The different radial curves in the Figure 4 indicate a marked variation of their coupling strength acting during the quantum dynamics (as discussed in the following section). We see, in fact, that the spherical term *V*_0_ provides the strongest attractive interaction which is extending isotropically around the diatomic target, while the first anisotropic term of importance at short range is the *V*_2_ that shows its attractive minimum close to that of the spherical term. As we shall discuss later, this term is responsible for the direct dynamical coupling of rotational levels with Δ*N* = 2 and therefore we expect those inelastic cross sections to play an important role in the excitation/de-excitation processes involving the present system. When we now compare the correct results for the ground electronic state (given by the solid lines), we see once more that the latter are consistently producing weaker coupling strengths when compared with the radial coefficients for the first excited electronic state (dashed lines). Such differences will once more affect the outcomes of the dynamical calculations which we shall further discuss below.

An important role in the overall dynamics will also be played by the lower λ radial coefficients, which are attractive at short-range and extend further out via the various terms of the following long-range expansion into the asymptotic region:
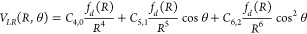
2where the radial expansion term associated with the *V*_λ_ = 1 via the coefficient *C*_5,1_ depends on the permanent dipole of CH^–^ and the polarizability of He:
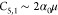
3This term provides the nonspherical contribution that dies out the most slowly and therefore will be an important long-range driver of excitation probabilities, as we shall show below. We thus expect that the Δ*N* = 1 transitions will dominate the energy transfer processes at the lower temperatures, where long-range forces are important contributors to the dynamical torque driving rotational state-changing collisions.

## Correct New Results and Discussion

### State-to-state Cross Sections and Rate Coefficients

As mentioned in the present Introduction, we are chiefly reporting in this Correction the results from the new, correct calculations and compare them with the older results in order to draw conclusions on the consequences of the changes and what is the final physics obtained with the revised calculations.

All methods employed here are the same as those reported in the original paper, and nothing was changed in that part of the original work, which was correctly carried out both there and in the present study.

A comparison between the old and new calculated inelastic cross sections involving both excitation and de-excitation (cooling) processes between rotational states of the anion is reported in the panels of Figure 5. As mentioned before, we treated the triplet-state of the target as a pseudo-singlet rotor since our earlier experience with calculations involving other molecular ions has shown that the final size of the derived rates did not change much when the proper additional coupling was included (e.g., see ref (5)). We had specifically verified this statement in the original paper where we reported calculations of state-to-state cross sections that compared the exact triplet calculations with the pseudo-singlet approach and found the two results to be nearly coincident.

**Figure 5:**
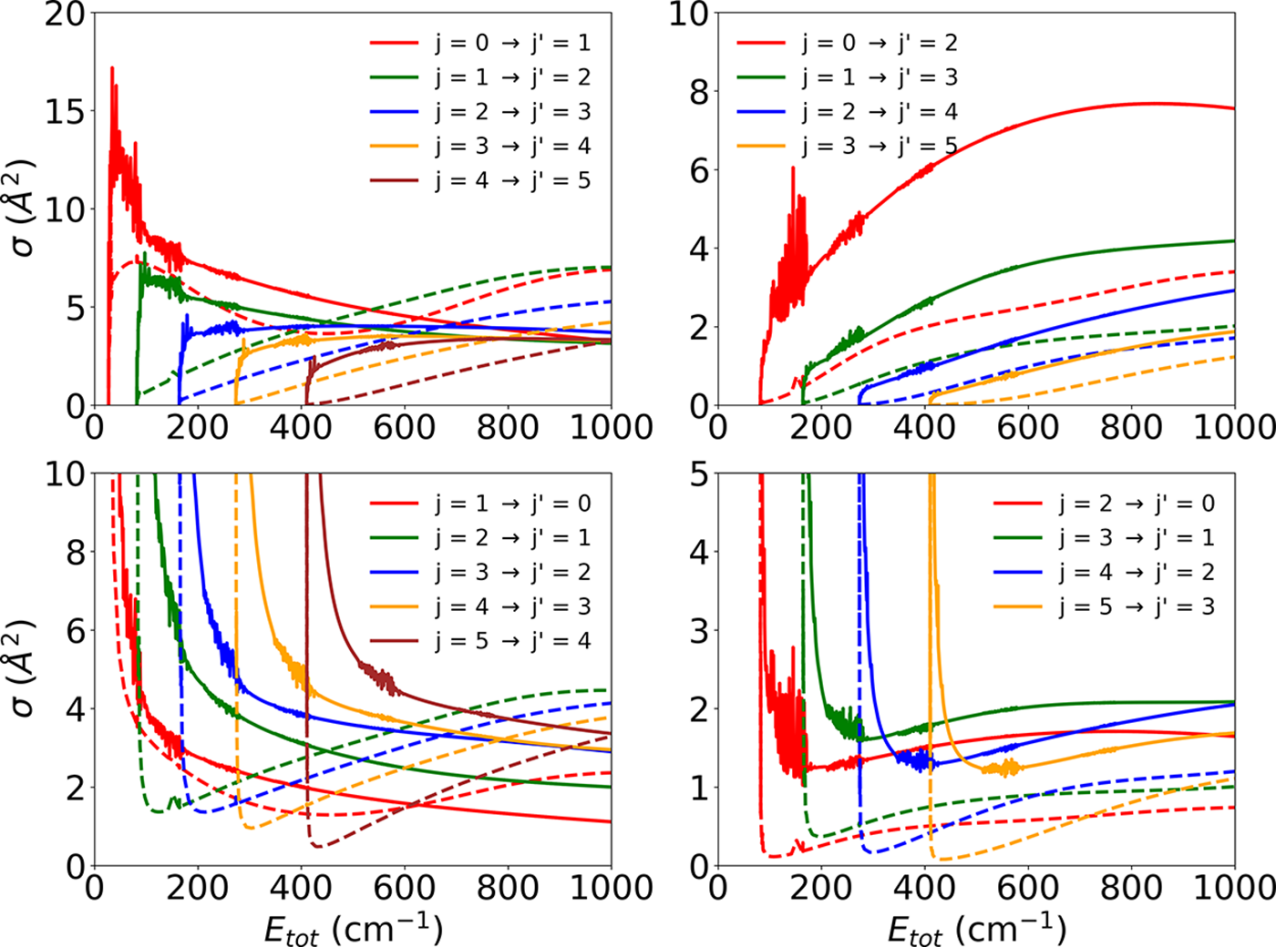
Comparison between computed state-to-state rotationally inelastic cross sections using the earlier PES (solid lines) and the new, correctly computed ground state PES (dotted lines). We report five different de-excitation processes with Δ*j* = 1, 2 from the lowest 4 and 5 levels (lower panels) and the corresponding excitation processes between the same levels (upper panels). See the main text for further comments.

The comparison between the calculations reported in Figure 5 confirms very clearly that all the examined cross sections are now markedly reduced in size, while keeping the same energy dependence as in the earlier data, when the correct PES for the ground electronic state of the system is employed (dashed lines in all panels). This finding thus suggests our final rate coefficients, as reported below, will also be markedly smaller when the correct interaction forces are employed and results are compared with the earlier calculations.

The data reported in Figure 6 show, as noted earlier, that the excitation processes with Δ*j* = 1 are larger than those with Δ*j* = 2 over the examined range of *T* values. This effect is linked to the larger coupling strength of the multipolar potential term with λ = 2 over the whole radial region, hence affecting the state-changing collision efficiency at the examined temperatures.

**Figure 6:**
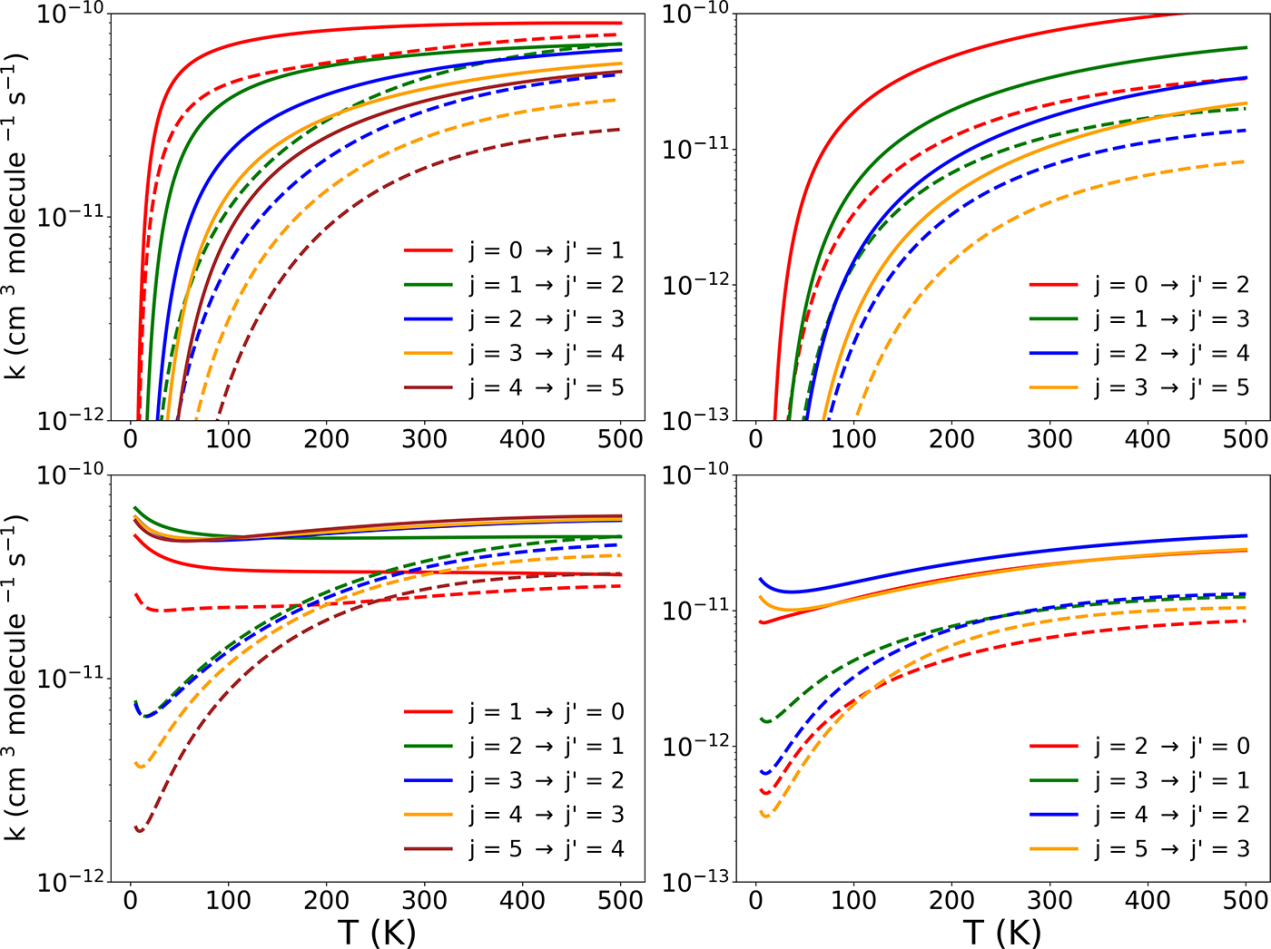
Computed state-to-state rotationally inelastic rate coefficients using both the new PES discussed in this work (dashed lines) and the earlier PES for the first excited electronic state employed earlier (solid lines). The upper-left panel reports excitation processes with Δ*j* = 1 from the lowest 5 levels, while the upper panel on the right shows those with Δ*j* = 2 transitions. The de-excitation cross sections involving the same Δ*j* = 1, 2 transitions are presented in the two lower panels.

The de-excitation rates reported in the two lower panels of the figure indicate once more the dominance of the inelastic processes which start from the higher rotational states, with those involving Δ*j* = 2 being invariably smaller than those with Δ*j* = 1.

On the whole, therefore, we can say that using the correct PES for the quantum dynamics uniformly produces smaller cross sections and smaller rate coefficients, while, however, the relative size relations and energy dependence of the quantum dynamics attributes remain very much the same as we had found in the previous analysis of the original paper.

### The Known Inelastic Collisions for CN^–^, C_2_H^–^, and CH with He: A Comparison with the Present CH^–^

While the presence of the CH^–^ in the ISM has not been firmly confirmed, other very similar small species like CH and CN^–^ have been observed in that same environment. In the case of the neutral counterpart, for example, CH has been sighted in the Interstellar Space, interstellar comets and stellar atmospheres.^6−8^ More recently, calculations have been carried out on the dynamics of its rotationally inelastic collisions with He atoms^9^ so a comparison of their results with those of its present anionic counterpart would be interesting, as we shall discuss below. In the case of the CN^–^, the smallest cynopolyyne to be detected in Interstellar environments, modeling and observation have happened in recent years^10,11^ and the actual calculations of the rotationally inelastic dynamics in collision with He has been studied in detail in our group.^12,13^ Hence, it also becomes interesting to see the differences in behavior between the two smallest anions of the polyyne and cyanopolyyne sequences, the latter of which species has been searched for, and detected, in a variety of ISM environments.

Additionally, another small negative anion of similar size and structure, the C_2_H^–^, has been often surmised to be present within the same ISM environments but never really confirmed. We have already studied its collisions with He and obtained accurate estimates of its efficiency in being rotationally excited/cooled by He scattering at the same low temperatures examined in the present study.^14^ We have therefore extended the comparison between the present results and those obtained for the three systems mentioned above. The results for such comparisons are reported in the following Figures 7 to 10.

We report in Figure 7 a comparison of the computed inelastic rate coefficients for these two molecular anions, taking into consideration different transitions and a broad range of temperature values.

**Figure 7:**
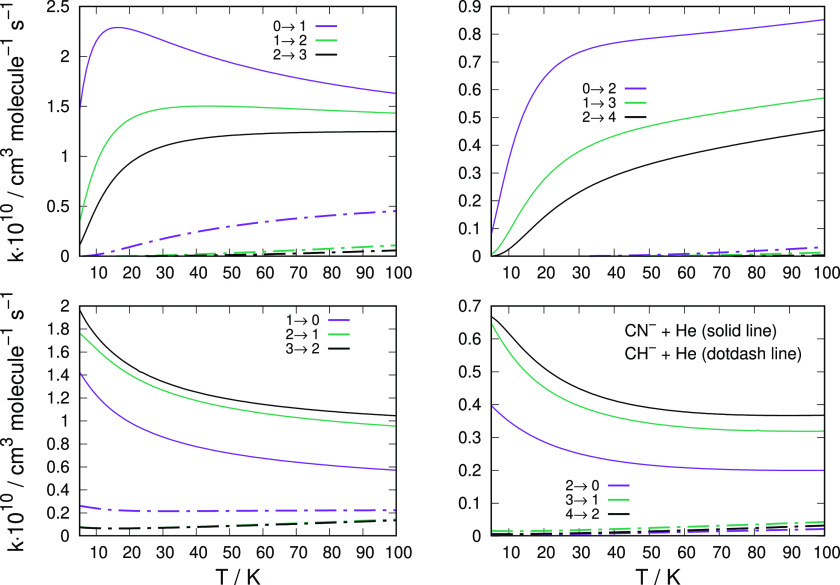
Comparing the computed state-changing rate coefficients between CH^–^ (dot-dash lines) and CN^–^ (solid lines). The data of the former anion are from the present calculations while those of the latter are from our earlier work.^12^ The two upper panels report rotational excitation processes, while the lower two panels show de-excitation processes.

To further show pictorially the differences in size between the inelastic rates in the two different anions, we report in Figure 8 a “stick” view of the rate coefficients at two different temperatures.

**Figure 8:**
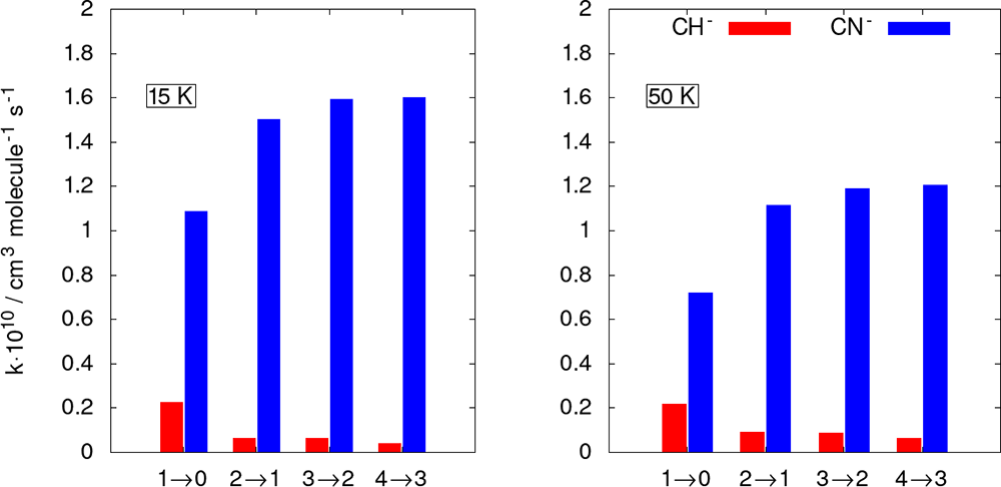
Computed state-changing rate coefficients for CH^–^ (red sticks) and CN^–^ (blue sticks). The data of the former anion are from the present calculations while those of the latter are from our earlier work.^12^ The two panels report rotational de-excitation processes at two different temperatures and for the lowest four excited rotational states of the two molecular systems.

The data in Figures 7 and 8 clearly show that the ISM-observed cynopolyyne, i.e., the CN^–^ anion, exhibits an excitation/cooling efficiency that is much larger than in the case of the CH^–^ anion: all the shown rate coefficients for the latter target are in fact nearly 1 order of magnitude smaller at all the considered temperatures. Such differences are linked to differences in the structural properties of the two polar rotors. In the case of the CN^–^ anion the rotational constant is nearly 1 order of magnitude smaller (1.872 cm^–1^) in comparison with that for CH^–^ (13.70 cm^–1^). This means that the markedly larger energy gaps for inelastic state-to-state collisions involving CH^–^ will make the role of the He atomic partners much less effective in changing rotational populations with respect to the case of the CN^–^ anion. If we also notice that the reduced mass values, which appear in eq 6 for the derivation of the rate coefficients, are very similar in the two systems, with a value of 3.061377 amu for the CH^–^/He and of 3.46860 amu for the CN^–^/He, we can conclude that the crucial difference in their dynamcs is the large differences in the energy gaps between the states involved in the inelastic processes. Hence, the possible departure from local thermal equilibrium (LTE) conditions for the former polar anion is less likely to occur via collisions with He atoms than in would be the case of the CN^–^anion, a feature we have discussed in detail for this molecule in our earlier work.^12^

The ground electronic state configuration of CH is 1σ^2^ 2σ^2^ 3σ^2^ 1π^1^, and therefore the ground electronic state is of ^2^Π symmetry. The methylidene is a Hund’s case (b) radical in its lowest vibrational level of its ground electronic state, with the ^2^Π_1/2_ spin state being lower than the ^2^Π_3/2_. These two states are labeled in the current literature as the F2 and F1 states, respectively.^9^ The electronic orbital angular momentum, L, is coupled with the rotational angular momentum of the bare nuclei, R, to form the total (excluding nuclear and electron spin) angular momentum, *N*. *N* is then coupled with the electron spin angular momentum, *S*, giving the total angular momentum, *J*. In Hund’s case (b), *J* = (*N* ± 1/2) for the F1 and F2 manifolds, respectively. *J* is coupled with the nuclear spin of H (*I* = 1/2) to give the grand total angular momentum, *F*. The calculations of the state-to-state rotationally inelastic rates have been carried out for the collisions of the CH neutral molecule with He atoms^9^ for a variety of changes of the lower quantum numbers and over a range of temperatures up to 300 K. It turned out that all such rates were practically negligible at the lowest temperatures and reached their largest values of ≈10^–13^ cm^3^ s^–1^ only above about 200 K. Such values are therefore more than 2 orders of magnitude smaller than those we have obtained for the present anion, the CH^–^ partner, in the same temperature range relevant for the ISM conditions. This finding thus confirms the essentially marginal significance of the collision-driven rotational state changes induced in CH by the He present in these environments. The comparison also clearly confirms the much larger rate coefficients which occur in collisions involving charged molecular partners as opposed to the neutral ones.

As mentioned earlier, we have extended the comparison of the present calculations, which now use the correct PES to describe the interaction between the partners of the present system, to another molecular anion of similar structure, the C_2_H^–^ negative ion, for which we had done earlier accurate calculations already reported in the literature.^14^ The results of the present comparisons are reported in the panels of Figures 9 and 10.

**Figure 9:**
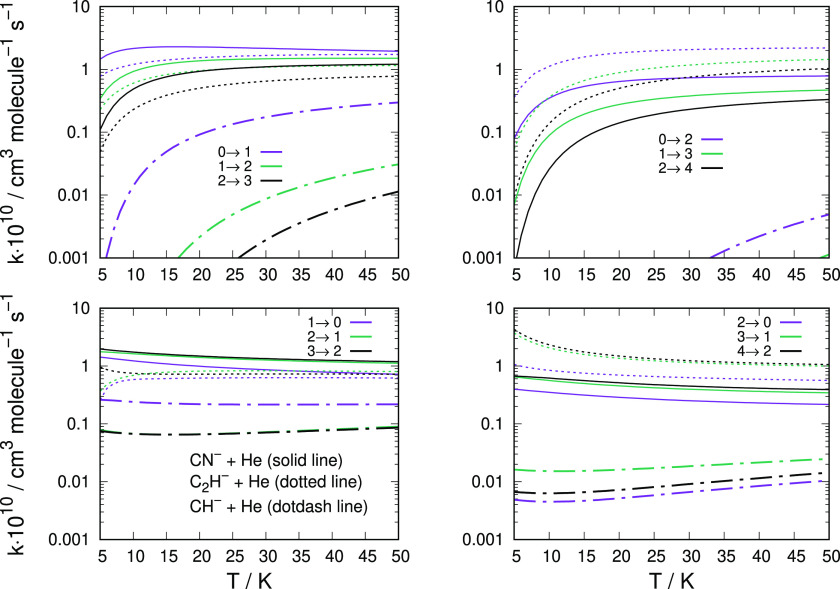
Comparing the computed state-changing rate coefficients between CH^–^ (dotdash lines), C_2_H^–^ (dotted lines), and CN^–^ (solid lines). The data of the first ion are from the present calculations while those of CN^–^ and of the C_2_H^–^ are from our earlier work in refs (12) and (14), respectively. The two upper panels report rotational excitation processes, while the lower two panels show de-excitation processes. See the main text for further details.

**Figure 10:**
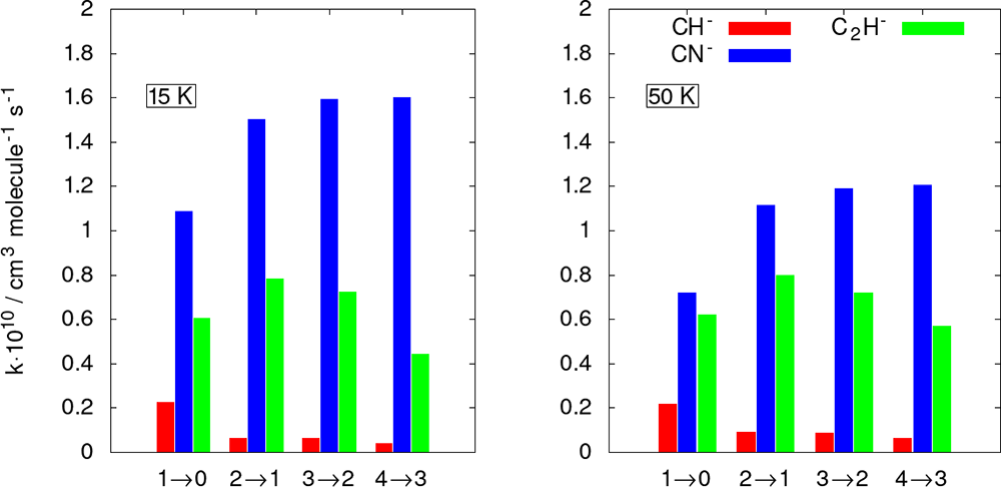
Computed state-changing rate coefficients for CH^–^ (red sticks), CN^–^ (blue sticks), and C_2_H^–^ (green sticks). The data of the last two anions are from our earlier work in refs (12) and (14), respectively. The two panels report rotational de-excitation processes at two different temperatures and for the lowest four excited rotational states of the two molecular systems.

The results reported by the two last figures essentially confirm what we had already found in the study we are correcting here: the CH^–^ anion exhibits the smallest excitation/de-excitation efficiency from collisions with the He partner in comparison with both CN^–^, which shows the largest excitation/cooling efficiency by collisions, and the C_2_H^–^ system, which is somewhere in between, although much closer to the behavior of the CN^–^ partner.

## Present Conclusions: Assessing the Changes from the Earlier Calculations

We have presented in this work extensive ab initio calculations involving the CH^–^ anion, known to be the smallest term of the polyyne anionic chains for which larger terms have been observed in the Interstellar environments, as discussed in the Introduction. The main scope of the present study is to correct an earlier error in the calculations that appeared in the original publication. In other words, we have discovered that the incorrect PES had been used before so we calculated the ground electronic state of the CH^–^/He system again and repeated all the quantum dynamics calculations relevant for the present study.

The new interaction forces produced by our corrected calculations were thus employed to yield the low-energy behavior of the excitation/de-excitation probabilities involving its rotational states and during collisions with He atoms. The quantum evaluation of the relevant dynamics for these probabilities allows us to correctly get the corresponding rotational state-changing rate coefficients over a range of temperatures relevant for the ISM environments where this molecule is surmised to be present, albeit not yet uniquely detected.

It turns out, in fact, that the very large energy spacings between rotational states are the crucial ingredients that make, in CH^–^, the energy-transfer processes by collision at low-*T* markedly inefficient in comparison with those involving other anions of similar size like CN^–^ and C_2_H^–^. Such differences are still confirmed, and even further enhanced, by the present revisions where the overall PES for the ground electronic state of the HeCH^–^ system is found to yield an even weaker interaction. They therefore still provide the reasons why only the CN^–^ anion has been so far detected in interstellar environments (see Agúndez et al.^10,11^). The present revisions therefore confirm that the out-of-equilibrium (i.e., away from LTE conditions) rotational populations of the present molecule via collisions with He is not a process that would be of significance within the kinetic modeling of such a small anion in the ISM. The new calculations provide now a correct quantitative estimate, from first-principles, of the very low collision efficiency of the title system in interaction with He. Our newly computed rate coefficients are found to be nearly 1 order of magnitude smaller than those in the earlier study but could be used in further modeling rotational population evolution of this specific species within larger chemical networks since our new findings confirm the smallness of collision-driven probabilities and suggest them to be one of the possible reasons for the difficulty in detecting the present anion via microwave emission spectroscopy from the excited rotational states.

## Supporting Information Available

The Supporting Information is available free of charge at https://pubs.acs.org/doi/10.1021/acs.jpca.3c03005. The raw *ab initio* PES of the rigid rotor CH^–^ · · · He potential, the computed rotationally inelastic cross sections and inelastic rate coefficients from the 2D-RR-PES employed in this work are available as Supporting Information to the present publication. We also include the computed potential energy curve raw points for the isolated anion and the dipole moment values computed as a function of the bond values. The different folders of the SI therefore contain: (*i*) CHm: containing the PEC at aV5Z level of expansion, for the X ^3^Σ^–^, the 3A′, the 3A′′ and the 3Δ. (*ii*) CHmHe: containing the PES raw points at aV5Z, aV6Z and CBS extrapolation level. (*iii*) Cross-Sections: containing the cross-section values for the inelastic collisions for the pseudo-Singlet approach and for the Triplet state. (*iv*) RATES: containing the rate coefficients (only for the pseudo-singlet approach). Data Availability All the data that support the findings of this study are available within the article itself and in its Supporting Information (ZIP)
