# Supplementary material for: The CH–3Σ+ Anion: Inelastic Rate Coefficients from Collisions with He at Interstellar Conditions
Source: J Phys Chem A. 2023 Jan 4;127(3):765–74. doi: 10.1021/acs.jpca.2c08021 (PMC9884083; doi:10.1021/acs.jpca.2c08021)
Supplement: Supplementary file 1 — jp2c08021_si_001.pdf [file jp2c08021_si_001.pdf]

The $CH^- \ ^3\Sigma_g^+$ anion : Inelastic Rate Coefficients from collisions with He under Interstellar Conditions

Jorge Alonso de la Fuente,[†] Cristina Sanz-Sanz,[†] Lola Gonzalez-Sanchez ,[‡] E. Yurtsever,[¶] R. Wester,[§] and F. A. Gianturco[*,§]

†*Departamento de Quimica Fisica Aplicada, Modulo 14, Universidad Autonoma de Madrid, 28049 Madrid, Spain*

‡*Facultad de Ciencias Quimicas, Universidad de Salamanca, Plaza de los Caidos s/n, 37008 Salamanca, Spain.*

¶*Dept. of Chemistry, Koc University, Rumelifeneri Yolu, Sariyer,TR, 34450 Istanbul,Turkey*

§*Institut fur Ionen Physik und Angewandte Physik, Leopold-Franzens-Universitat, Technikerstrasse 25, 6020, Innsbruck, Austria*

E-mail: francesco.gianturco@uibk.ac.at

Captions.

Figure 1. Behaviour of the partial excess charge on each of the atoms in the anion as a function of the internuclear distance.Notice the asymptotic values where the negative charge moved entirely on the C-atom.

Figure 2. Locations and values of the lowest three vibrational w.f.s with their corresponding turning points.

Figure 3. Energy for the first six rotational states of the ν=0 vibrational states (right) and their Boltzmann Distribution when treating the title anion as a pseudo-Singlet rotor.

Figure 4. Spatial distribution of the interaction potential energy around the molecular anion. See main text for further details.

Figure 5. Multipolar expansion coefficients for the computed PES of the present study. See main text for further details.

Figure 6. Computed state-to-state rotationally inelastic cross sections, treating the molecular anion either as a full Triplet rotor (filled squares) or as a pseudo-Singlet rotor (thin lines). We report for comparison five different de-excitation processes with Δj=1 from the lowest 5 levels. See main text for further comments.

Figure 7. Computed state-to-state rotationally inelastic cross sections, treating the molecular anion as a pseudo-singlet rotor. The upper-left panel reports excitation processes with Δj=1 from the lowest 5 levels. The lower-left panel shows excitations with Δj=2 transitions. The de-excitation cross sections are presented in the upper- and lower-right panels.

Figure 8. Computed state-to-state rotationally inelastic rate coefficients, treating the molecular anion as a pseudo-Singlet rotor. The upper-left panel reports excitation processes with Δj=1 from the lowest 5 levels. The lower-left panel shows excitations with Δj=2 transitions. The de-excitation cross sections are presented in the upper- and lower-right panels of the figure.

Figure 9. Comparing the computed state-changing rate coefficients between CH^- and CN^-. the data of the former anion are from the present calculations while those of the latter are from our earlier work[?] . The two upper panels report rotational excitation processes, while the lower two panels show de-excitation processes.

Figure 10. Computed state-changing rate coefficients for CH^- (red sticks) and CN^- (blue sticks). The data of the former anion are from the present calculations while those of the latter are from our earlier work[?] . The two panels report rotational de-excitation processes at two different temperatures and for the lowest four excited rotational states of the two molecular systems.

Method: MRCI – aV5Z

R(Å)	E (Eh)	R(Å)	E (Eh)
0.793766	-38.1973269247	3.06923	-38.2171434366
0.851975	-38.2420316047	3.12744	-38.2161682418
0.910184	-38.2717229097	3.18565	-38.215318411
0.963102	-38.2933666635	3.24386	-38.214582103
1.02131	-38.3055171313	3.29677	-38.2139439431
1.07952	-38.3120018635	3.35498	-38.2133893963
1.13773	-38.3144617281	3.41319	-38.2129058739
1.19065	-38.3141154552	3.4714	-38.2124828321
1.24886	-38.3118645782	3.52432	-38.2121114537
1.30707	-38.3083706119	3.58253	-38.2117843965
1.36528	-38.3041126962	3.64074	-38.2114955193
1.41819	-38.2994316645	3.69895	-38.2112396619
1.4764	-38.2945644305	3.75186	-38.2110124691
1.53461	-38.2896710616	3.81007	-38.2108102485
1.59282	-38.2848559665	3.86828	-38.2106298528
1.64574	-38.2801841749	3.92649	-38.2104685903
1.70395	-38.2756935386	3.9847	-38.210324147
1.76216	-38.2714036321	4.03762	-38.2101945276
1.82037	-38.2673220762	4.09583	-38.2100780048
1.87329	-38.2634489219	4.15404	-38.2099730783
1.9315	-38.2597796221	4.21225	-38.2098784412
1.98971	-38.2563070012	4.26517	-38.2097929515
2.04791	-38.2530225285	4.32338	-38.2097156089
2.10083	-38.2499171162	4.38159	-38.2096455348
2.15904	-38.2469816006	4.4398	-38.2095819558
2.21725	-38.2442070182	4.49271	-38.2095241897
2.27546	-38.2415847509	4.55092	-38.2094716335
2.33367	-38.2391065931	4.60913	-38.2094237531
2.38659	-38.2367647732	4.66734	-38.2093800749
2.4448	-38.2345519519	4.72026	-38.2093401778
2.50301	-38.2324612103	4.77847	-38.2093036874
2.56122	-38.2304860399	4.83668	-38.20927027
2.61413	-38.2286203368	4.89489	-38.209239627
2.67234	-38.22685842	4.9478	-38.2092114936
2.73055	-38.2251950768	5.00601	-38.2091856313
2.78876	-38.2236256778	5.06422	-38.2091618268
2.84168	-38.2221464297	5.12243	-38.209139889
2.89989	-38.2207549717	5.17535	-38.2091196464
2.9581	-38.2194517537	5.23356	-38.2091009448
3.01631	-38.2182430197	5.29177	-38.2090836456

Method: MRCI. AV5Z with BSSE correction

R(Å)	E (Eh)	R(Å)	E (Eh)
0.793766	-38.33836957	3.06923	-38.34097718
0.851975	-38.38137351	3.12744	-38.33963174
0.910184	-38.41475465	3.18565	-38.33834701
0.963102	-38.43419447	3.24386	-38.33716848
1.02131	-38.44583004	3.29677	-38.33610394
1.07952	-38.4517574	3.35498	-38.33514471
1.13773	-38.4536093	3.41319	-38.33428768
1.19065	-38.45259968	3.4714	-38.33351682
1.24886	-38.44962975	3.52432	-38.33282594
1.30707	-38.44536575	3.58253	-38.33220645
1.36528	-38.44029627	3.64074	-38.33164979
1.41819	-38.43477546	3.69895	-38.33114941
1.4764	-38.42905579	3.75186	-38.33069953
1.53461	-38.42331328	3.81007	-38.33029393
1.59282	-38.41766705	3.86828	-38.32992854
1.64574	-38.41219446	3.92649	-38.32959865
1.70395	-38.40694276	3.9847	-38.32930078
1.76216	-38.40193796	4.03762	-38.32903162
1.82037	-38.39719137	4.09583	-38.3287882
1.87329	-38.39270454	4.15404	-38.32856788
1.9315	-38.38847266	4.21225	-38.32836832
1.98971	-38.38448706	4.26517	-38.32818741
2.04791	-38.38073687	4.32338	-38.32802326
2.10083	-38.37721015	4.38159	-38.32787418
2.15904	-38.37389456	4.4398	-38.32773866
2.21725	-38.37077783	4.49271	-38.32761532
2.27546	-38.367848	4.55092	-38.32750296
2.33367	-38.36509357	4.60913	-38.32740047
2.38659	-38.36250358	4.66734	-38.32730688
2.4448	-38.36006773	4.72026	-38.3272213
2.50301	-38.35777638	4.77847	-38.32714297
2.56122	-38.35562054	4.83668	-38.32707118
2.61413	-38.35359192	4.89489	-38.32700549
2.67234	-38.35168289	4.9478	-38.32694499
2.73055	-38.34988647	5.00601	-38.32688938
2.78876	-38.34819621	5.06422	-38.32683824
2.84168	-38.34660593	5.12243	-38.32679121
2.89989	-38.3451087	5.17535	-38.32674793
2.9581	-38.34369295	5.23356	-38.32670814
3.01631	-38.34233091	5.29177	-38.32667155

Method: MRCI. AV6Z

R(Å)	E (Eh)	R(Å)	E (Eh)
0.793766	-38.1974030908	3.06923	-38.21718806
0.851975	-38.2421039053	3.12744	-38.21621236
0.910184	-38.2717927121	3.18565	-38.21536208
0.963102	-38.2934299864	3.24386	-38.21462547
1.02131	-38.3055773725	3.29677	-38.2139871
1.07952	-38.3120593589	3.35498	-38.21343241
1.13773	-38.3145168664	3.41319	-38.21294879
1.19065	-38.3141686916	3.4714	-38.21252569
1.24886	-38.3119163752	3.52432	-38.21215427
1.30707	-38.3084213424	3.58253	-38.21182719
1.36528	-38.3041625942	3.64074	-38.21153829
1.41819	-38.2994808676	3.69895	-38.21128241
1.4764	-38.2946130524	3.75186	-38.21105519
1.53461	-38.2897192305	3.81007	-38.21085295
1.59282	-38.2849038251	3.86828	-38.21067252
1.64574	-38.2802318626	3.92649	-38.21051123
1.70395	-38.2757411715	3.9847	-38.21036675
1.76216	-38.2714512911	4.03762	-38.2102371
1.82037	-38.2673698054	4.09583	-38.21012055
1.87329	-38.2634967349	4.15404	-38.21001559
1.9315	-38.2598275113	4.21225	-38.20992092
1.98971	-38.2563549454	4.26517	-38.20983541
2.04791	-38.2530704975	4.32338	-38.20975804
2.10083	-38.2499650746	4.38159	-38.20968794
2.15904	-38.2470295118	4.4398	-38.20962435
2.21725	-38.2442548464	4.49271	-38.20956657
2.27546	-38.2416324635	4.55092	-38.209514
2.33367	-38.2391541615	4.60913	-38.20946611
2.38659	-38.2368121749	4.66734	-38.20942242
2.4448	-38.2345991723	4.72026	-38.20938252
2.50301	-38.2325082442	4.77847	-38.20934602
2.56122	-38.230532889	4.83668	-38.2093126
2.61413	-38.2286670107	4.89489	-38.20928195
2.67234	-38.2269049309	4.9478	-38.20925382
2.73055	-38.2252414369	5.00601	-38.20922796
2.78876	-38.2236718933	5.06422	-38.20920415
2.84168	-38.2221924938	5.12243	-38.20918221
2.89989	-38.2208008488	5.17535	-38.20916197
2.9581	-38.2194973588	5.23356	-38.20914327
3.01631	-38.2182882044	5.29177	-38.20912598

Method: MRCI. AV6Z with BSSE correction

R(Å)	E(Eh)	R(Å)	E(Eh)
0.793766	-38.34037832	3.06923	-38.34152035
0.851975	-38.38327669	3.12744	-38.34016457
0.910184	-38.41656887	3.18565	-38.3388778
0.963102	-38.43590464	3.24386	-38.33769436
1.02131	-38.44744507	3.29677	-38.33664168
1.07952	-38.45328637	3.35498	-38.33568081
1.13773	-38.45506234	3.41319	-38.33482145
1.19065	-38.45398627	3.4714	-38.33404902
1.24886	-38.45095727	3.52432	-38.33335731
1.30707	-38.44663917	3.58253	-38.33273765
1.36528	-38.44151866	3.64074	-38.33218139
1.41819	-38.43594898	3.69895	-38.3316824
1.4764	-38.43018236	3.75186	-38.33127104
1.53461	-38.42439494	3.81007	-38.33086696
1.59282	-38.41870602	3.86828	-38.33050325
1.64574	-38.4131931	3.92649	-38.33017509
1.70395	-38.4079035	3.9847	-38.3298789
1.76216	-38.40286315	4.03762	-38.3296113
1.82037	-38.3980833	4.09583	-38.32936926
1.87329	-38.39356543	4.15404	-38.32915011
1.9315	-38.38930477	4.21225	-38.32895149
1.98971	-38.38529266	4.26517	-38.32877128
2.04791	-38.3815182	4.32338	-38.32860764
2.10083	-38.37796929	4.38159	-38.3284589
2.15904	-38.37463337	4.4398	-38.32828586
2.21725	-38.37149787	4.49271	-38.32816267
2.27546	-38.36855055	4.55092	-38.32805042
2.33367	-38.3657796	4.60913	-38.32794807
2.38659	-38.36317389	4.66734	-38.32785467
2.4448	-38.36072303	4.72026	-38.32776939
2.50301	-38.35841734	4.77847	-38.32769148
2.56122	-38.35624788	4.83668	-38.32762019
2.61413	-38.35420643	4.89489	-38.32755517
2.67234	-38.35228542	4.9478	-38.32749543
2.73055	-38.3504779	5.00601	-38.32744064
2.78876	-38.34877743	5.06422	-38.32739037
2.84168	-38.34717781	5.12243	-38.3273442
2.89989	-38.34567211	5.17535	-38.32729901
2.9581	-38.34424881	5.23356	-38.32725994
3.01631	-38.3428802	5.29177	-38.32722396

Method: MRCI. CBS

R(Å)	E (Eh)	R(Å)	E (Eh)
0.793766	-38.34240257	3.06923	-38.3424983
0.851975	-38.3851786	3.12744	-38.34113026
0.910184	-38.41839264	3.18565	-38.33983994
0.963102	-38.43764809	3.24386	-38.33864987
1.02131	-38.44912168	3.29677	-38.33757547
1.07952	-38.45490348	3.35498	-38.33660812
1.13773	-38.45662489	3.41319	-38.33574466
1.19065	-38.45549781	3.4714	-38.33496882
1.24886	-38.45242153	3.52432	-38.33427437
1.30707	-38.44806057	3.58253	-38.33365253
1.36528	-38.44290198	3.64074	-38.33309463
1.41819	-38.43729862	3.69895	-38.33259505
1.4764	-38.43150209	3.75186	-38.33214475
1.53461	-38.42568796	3.81007	-38.3317405
1.59282	-38.41997519	3.86828	-38.33137707
1.64574	-38.41444099	3.92649	-38.33104966
1.70395	-38.40913253	3.9847	-38.33075464
1.76216	-38.40407566	4.03762	-38.33048864
1.82037	-38.39928147	4.09583	-38.33024863
1.87329	-38.39475114	4.15404	-38.33003183
1.9315	-38.39047949	4.21225	-38.32983593
1.98971	-38.38645735	4.26517	-38.3296587
2.04791	-38.38267333	4.32338	-38.32949829
2.10083	-38.37911493	4.38159	-38.32935294
2.15904	-38.37576931	4.4398	-38.32922118
2.21725	-38.37262377	4.49271	-38.32910162
2.27546	-38.36966612	4.55092	-38.32899301
2.33367	-38.36688456	4.60913	-38.32889432
2.38659	-38.36426813	4.66734	-38.32880455
2.4448	-38.36180655	4.72026	-38.32872279
2.50301	-38.35949018	4.77847	-38.32864832
2.56122	-38.35731016	4.83668	-38.32858035
2.61413	-38.35525824	4.89489	-38.32851856
2.67234	-38.35332691	4.9478	-38.32846191
2.73055	-38.35150922	5.00601	-38.32841006
2.78876	-38.34979879	5.06422	-38.32836263
2.84168	-38.3481895	5.12243	-38.32831919
2.89989	-38.3466745	5.17535	-38.32827935
2.9581	-38.34524248	5.23356	-38.3282428
3.01631	-38.34386585	5.29177	-38.32820924

Dipole moment data. Method: MRCI - aV5Z

Three types of calculations depending on the frame of reference:

1) With respect to the C atom (C)

2) With respect the center of mass (CM)

3) With respect the center of charges (CC)

The data ara given both in atomic units (nothing) and in debye (-Deb).

R (Å)	DM (C)	DM (C) -Deb	DM (CC)	DM (CC) -Deb	DM (CM)	DM (CM) -Deb
1.5	0.7634	1.940369126	0.977686	2.4760972215	0.879532	2.2355461177
1.607595	0.71304	1.81235612	0.942692	2.3865607687	0.837498	2.1287061449
1.71519	0.65489	1.664551786	0.899912	2.2779354431	0.787677	2.0020748437
1.822785	0.58901	1.497117253	0.849409	2.1498788836	0.730133	1.8558133441
1.93038	0.51508	1.309200932	0.790848	2.0015162563	0.664531	1.6890700561
2.037975	0.43313	1.100908448	0.72427	1.8329146143	0.590913	1.5019506052
2.14557	0.34353	0.87316635	0.65004	1.6449504595	0.509642	1.2953815403
2.253165	0.24693	0.62762225	0.568806	1.4392243689	0.421368	1.0710104733
2.360759	0.14426	0.366667769	0.48151	1.2180702957	0.327031	0.831229026
2.468354	0.03671	0.093302982	0.38933	0.9844403283	0.227811	0.5790372718
2.575949	-0.07435	-0.18897036	0.293646	0.7418065375	0.125086	0.3179369634
2.683544	-0.18747	-0.476498571	0.195894	0.4938136672	0.020294	0.0515817855
2.791139	-0.30125	-0.765699094	0.097485	0.2440472139	-0.085156	-0.2164457046
2.898734	-0.41437	-1.053228132	-0.000267	-0.0041317907	-0.189949	-0.4828017088
3.006329	-0.52575	-1.336318818	-0.096273	-0.2479473418	-0.292995	-0.7447193622
3.113924	-0.6345	-1.612739465	-0.189654	-0.4851552003	-0.393417	-0.9999669764
3.221519	-0.73998	-1.880848376	-0.279766	-0.7141034394	-0.490569	-1.2469028531
3.329114	-0.84175	-2.139517681	-0.366163	-0.9336383981	-0.584008	-1.4843991251
3.436709	-0.93954	-2.388073314	-0.448582	-1.1430803738	-0.673467	-1.711781725
3.544304	-1.03322	-2.6261861	-0.526892	-1.3420862575	-0.758817	-1.9287214782
3.651899	-1.12276	-2.853772497	-0.601061	-1.5305680276	-0.840027	-2.1351348416
3.759494	-1.2082	-3.070929033	-0.671126	-1.708619428	-0.917133	-2.3311183447
3.867089	-1.28961	-3.27786477	-0.73717	-1.8764495426	-0.990217	-2.5168810485
3.974684	-1.36711	-3.474847258	-0.799298	-2.0343203113	-1.059386	-2.6926905036
4.082278	-1.44081	-3.662166095	-0.857624	-2.1825133967	-1.124753	-2.8588363072
4.189873	-1.51081	-3.84009374	-0.912256	-2.3213078178	-1.186425	-3.0155909191
4.297468	-1.57721	-4.008856179	-0.963281	-2.4503580892	-1.244491	-3.1631803248
4.405063	-1.63977	-4.167881841	-1.010476	-2.5708132292	-1.298726	-3.301032954
4.512658	-1.69902	-4.318475723	-1.054354	-2.6822476818	-1.349644	-3.4304538031
4.620253	-1.75467	-4.459929611	-1.094635	-2.7845170253	-1.396967	-3.5507346581
4.727848	-1.80659	-4.591885366	-1.13118	-2.8772952369	-1.440552	-3.6615173797
4.835443	-1.8545	-4.71366737	-1.163722	-2.9599063196	-1.480135	-3.7621263506
4.943038	-1.89795	-4.824112119	-1.191804	-3.0311970615	-1.515257	-3.8513980665
5.050633	-1.93619	-4.92129961	-1.214669	-3.0892607152	-1.545163	-3.9274125242
5.158228	-1.96797	-5.002080981	-1.231081	-3.1309579132	-1.568615	-3.9870208619
5.265823	-1.99122	-5.061186336	-1.238964	-3.1510331691	-1.583539	-4.0249531841
5.373418	-2.00237	-5.089517242	-1.234739	-3.1404010637	-1.586355	-4.0321110564
5.481013	-1.99503	-5.070850659	-1.212025	-3.082838922	-1.570681	-3.9922714403
5.588608	-1.95788	-4.976445134	-1.159512	-2.9494918962	-1.525208	-3.876692882
5.696203	-1.87472	-4.765060709	-1.060976	-2.6987647037	-1.433713	-3.6441354242
5.803797	-1.74155	-4.426582676	-0.912438	-2.3196215341	-1.292216	-3.2844843577

R (Å)	DM (C)	DM (C) -Deb	DM (CC)	DM (CC) -Deb	DM (CM)	DM (CM) -Deb
5.911392	-1.59028	-4.042099827	-0.7458	-1.8933738534	-1.132618	-2.8788284757
6.018987	-1.44895	-3.682874256	-0.589099	-1.4932869465	-0.982958	-2.4984298718
6.126582	-1.32488	-3.367505999	-0.449653	-1.1376715648	-0.850552	-2.1618885814
6.234177	-1.21601	-3.090799111	-0.325417	-0.8210905709	-0.733357	-1.8640086599
6.341772	-1.11933	-2.845059707	-0.213365	-0.5357540483	-0.628346	-1.5970962234
6.449367	-1.03246	-2.624259513	-0.111125	-0.2755630793	-0.533146	-1.3551229959
6.556962	-0.95467	-2.426540547	-0.017966	-0.0383026944	-0.447028	-1.1362309971
6.664557	-0.88289	-2.244077633	0.069191	0.1833397418	-0.366911	-0.9325950494
6.772152	-0.81711	-2.076889838	0.150339	0.3896207376	-0.292804	-0.7442342211
6.879747	-0.75666	-1.923248072	0.226157	0.5822608925	-0.224027	-0.5694194227
6.987342	-0.70101	-1.781777943	0.297186	0.7626418297	-0.160038	-0.4067762601
7.094937	-0.64971	-1.651400547	0.363851	0.931916985	-0.100414	-0.2552258309
7.202532	-0.60238	-1.5311046	0.42655	1.0910720762	-0.044755	-0.1137568507
7.310127	-0.55871	-1.420092541	0.485596	1.2409333465	0.00725	0.0184282415
7.417722	-0.5184	-1.317630553	0.541279	1.3822322882	0.055892	0.1420632626
7.525316	-0.48119	-1.22306875	0.593853	1.5156341453	0.101426	0.2577980984
7.632911	-0.44686	-1.135794046	0.64356	1.6417468111	0.144092	0.3662458358
7.740506	-0.41517	-1.055249901	0.690619	1.7611291445	0.184111	0.4679630141
7.848101	-0.38593	-0.980924067	0.735232	1.8742956284	0.221683	0.5634618812
7.955696	-0.35894	-0.912344665	0.777584	1.9817026287	0.256994	0.653214316
8.063291	-0.33405	-0.849068755	0.817849	2.0837984947	0.290219	0.7376632599
8.170886	-0.31108	-0.790688827	0.856188	2.1810130476	0.321518	0.817216221
8.278481	-0.28989	-0.736824405	0.892751	2.2737292821	0.35104	0.8922536756
8.386076	-0.27033	-0.687122814	0.927676	2.3622978989	0.378924	0.9631282999
8.493671	-0.25229	-0.641257732	0.961091	2.4470421863	0.405299	1.0301664152
8.601266	-0.23564	-0.598926141	0.993117	2.5282627429	0.430283	1.0936710392
8.708861	-0.22026	-0.559847743	1.023862	2.6062383277	0.453988	1.1539224704
8.816456	-0.20606	-0.523763908	1.053429	2.681226717	0.476515	1.2111793383
8.924051	-0.19295	-0.490436045	1.081912	2.7534670378	0.497957	1.2656802352
9.031646	-0.18084	-0.45964423	1.109397	2.8231790366	0.518402	1.3176450829
9.139241	-0.16964	-0.431185988	1.135964	2.8916227847	0.537928	1.3672763583
9.246835	-0.1588	-0.403623799	1.162179	2.956809763	0.557102	1.4160115804
9.35443	-0.14925	-0.379359601	1.187096	3.0200309614	0.574978	1.4614488111
9.462025	-0.14042	-0.356908452	1.211299	3.0814435678	0.592141	1.5050729933
9.56962	-0.13224	-0.33613029	1.234845	3.1411924031	0.608646	1.5470241885
9.677215	-0.12468	-0.316897802	1.257782	3.1994100602	0.624543	1.5874297101
9.78481	-0.11767	-0.299081114	1.280162	3.2562181461	0.639883	1.6264194314
9.892405	-0.11117	-0.28256632	1.302031	3.3117287393	0.65471	1.6641072578
10	-0.10514	-0.26724944	1.323427	3.3660450238	0.669066	1.7005971713

Dipole moment data. Method: MRCI – aV6Z

Three types of calculations depending on the frame of reference:

1) With respect to the C atom (C)

2) With respect the center of mass (CM)

3) With respect the center of charges (CC)

The data ara given both in atomic units (nothing) and in debye (-Deb).

R (Å)	DM (C)	DM (C) -Deb	DM (CC)	DM (CC) -Deb	DM (CM)	DM (CM) -Deb
1.5	0.75989	1.931437315	0.974172	2.4760972215	0.876018	2.2266143063
1.607595	0.70929	1.80283243	0.938945	2.3865607687	0.833751	2.1191824546
1.71519	0.65118	1.655138672	0.896209	2.2779354431	0.783974	1.9926617303
1.822785	0.58543	1.48801368	0.845828	2.1498788836	0.726552	1.8467097709
1.93038	0.51169	1.300582621	0.787457	2.0015162563	0.661141	1.6804517447
2.037975	0.42998	1.092912547	0.721124	1.8329146143	0.587767	1.4939547036
2.14557	0.34066	0.86587996	0.647173	1.6449504595	0.506776	1.2880951507
2.253165	0.24435	0.621085437	0.566234	1.4392243689	0.418796	1.0644736611
2.360759	0.14197	0.360862932	0.479226	1.2180702957	0.324747	0.825424189
2.468354	0.03469	0.088164533	0.387309	0.9844403283	0.225789	0.5738988227
2.575949	-0.07614	-0.19353769	0.291849	0.7418065375	0.123289	0.3133696332
2.683544	-0.18908	-0.480598993	0.194281	0.4938136672	0.018681	0.0474813635
2.791139	-0.30272	-0.769433878	0.096016	0.2440472139	-0.086626	-0.2201804888
2.898734	-0.41573	-1.056681315	-0.001626	-0.0041317907	-0.191307	-0.4862548922
3.006329	-0.52703	-1.339565298	-0.09755	-0.2479473418	-0.294272	-0.7479658424
3.113924	-0.63572	-1.615841589	-0.190875	-0.4851552003	-0.394638	-1.0030690996
3.221519	-0.74117	-1.88385826	-0.28095	-0.7141034394	-0.491754	-1.2499127377
3.329114	-0.84291	-2.142461651	-0.367322	-0.9336383981	-0.585166	-1.4873430953
3.436709	-0.94068	-2.390972059	-0.449722	-1.1430803738	-0.674607	-1.7146804702
3.544304	-1.03435	-2.629046374	-0.528017	-1.3420862575	-0.759943	-1.9315817528
3.651899	-1.12387	-2.856596577	-0.602172	-1.5305680276	-0.841138	-2.1379589219
3.759494	-1.20929	-3.073716409	-0.672223	-1.708619428	-0.918229	-2.3339057212
3.867089	-1.29069	-3.280614956	-0.738252	-1.8764495426	-0.991299	-2.519631235
3.974684	-1.36818	-3.477554157	-0.800363	-2.0343203113	-1.060451	-2.6953974024
4.082278	-1.44185	-3.664815674	-0.858667	-2.1825133967	-1.125795	-2.8614858867
4.189873	-1.51183	-3.842678528	-0.913273	-2.3213078178	-1.187442	-3.018175707
4.297468	-1.57797	-4.010797231	-0.964045	-2.4503580892	-1.245255	-3.1651213771
4.405063	-1.64073	-4.170320803	-1.011436	-2.5708132292	-1.299686	-3.3034719161
4.512658	-1.69994	-4.320823688	-1.055278	-2.6822476818	-1.350568	-3.4328017679
4.620253	-1.75555	-4.462161463	-1.095513	-2.7845170253	-1.397845	-3.55296651
4.727848	-1.80742	-4.594008107	-1.132015	-2.8772952369	-1.441387	-3.6636401209
4.835443	-1.85529	-4.715687622	-1.164517	-2.9599063196	-1.480929	-3.7641466022
4.943038	-1.89871	-4.826046796	-1.192565	-3.0311970615	-1.516018	-3.8533327434
5.050633	-1.93693	-4.923178882	-1.215409	-3.0892607152	-1.545902	-3.929291796
5.158228	-1.9687	-5.003944512	-1.231814	-3.1309579132	-1.569348	-3.9888843929
5.265823	-1.99197	-5.0630882	-1.239712	-3.1510331691	-1.584287	-4.0268550478
5.373418	-2.00316	-5.091524527	-1.235529	-3.1404010637	-1.587144	-4.0341183413
5.481013	-1.99588	-5.073030817	-1.212882	-3.082838922	-1.571538	-3.9944515985
5.588608	-1.95879	-4.978752224	-1.16042	-2.9494918962	-1.526116	-3.8789999719
5.696203	-1.87552	-4.767093463	-1.061776	-2.6987647037	-1.434513	-3.6461681784
5.803797	-1.74172	-4.427018726	-0.912609	-2.3196215341	-1.292387	-3.2849204075

R (Å)	DM (C)	DM (C) -Deb	DM (CC)	DM (CC) -Deb	DM (CM)	DM (CM) -Deb
5.911392	-1.5894	-4.039839477	-0.744911	-1.8933738534	-1.131729	-2.8765681257
6.018987	-1.44736	-3.678821002	-0.587504	-1.4932869465	-0.981363	-2.494376618
6.126582	-1.32282	-3.362274053	-0.447594	-1.1376715648	-0.848494	-2.1566566352
6.234177	-1.21364	-3.084761491	-0.323042	-0.8210905709	-0.730982	-1.8579710403
6.341772	-1.11675	-2.838493401	-0.210782	-0.5357540483	-0.625763	-1.5905299166
6.449367	-1.02975	-2.617370864	-0.108415	-0.2755630793	-0.530436	-1.3482343466
6.556962	-0.95178	-2.419178911	-0.015069	-0.0383026944	-0.444131	-1.1288693606
6.664557	-0.87995	-2.236604907	0.072131	0.1833397418	-0.363971	-0.9251223234
6.772152	-0.81416	-2.069392343	0.153289	0.3896207376	-0.289855	-0.7367367268
6.879747	-0.75374	-1.91582062	0.229079	0.5822608925	-0.221105	-0.5619919708
6.987342	-0.69815	-1.774508115	0.300046	0.7626418297	-0.157178	-0.3995064325
7.094937	-0.64692	-1.644301392	0.366644	0.931916985	-0.097621	-0.248126676
7.202532	-0.59967	-1.524214733	0.429261	1.0910720762	-0.042045	-0.1068669837
7.310127	-0.55608	-1.413421895	0.488221	1.2409333465	0.009875	0.0250988875
7.417722	-0.51586	-1.311191385	0.543812	1.3822322882	0.058425	0.1485024302
7.525316	-0.47875	-1.21685796	0.596296	1.5156341453	0.103869	0.2640088884
7.632911	-0.4445	-1.129813727	0.645913	1.6417468111	0.146445	0.3722261552
7.740506	-0.41291	-1.049499825	0.692882	1.7611291445	0.186373	0.4737130897
7.848101	-0.38375	-0.975401774	0.737405	1.8742956284	0.223856	0.5689841746
7.955696	-0.35687	-0.907063205	0.779662	1.9817026287	0.259072	0.658495776
8.063291	-0.33207	-0.844035772	0.819829	2.0837984947	0.292199	0.7426962428
8.170886	-0.30919	-0.785889651	0.858077	2.1810130476	0.323406	0.8220153967
8.278481	-0.28809	-0.732241848	0.894554	2.2737292821	0.352843	0.8968362323
8.386076	-0.26861	-0.682741664	0.9294	2.3622978989	0.380648	0.9675094504
8.493671	-0.25064	-0.637065808	0.962741	2.4470421863	0.406948	1.0343583388
8.601266	-0.23406	-0.594913684	0.994695	2.5282627429	0.431862	1.0976834962
8.708861	-0.21875	-0.556006531	1.025373	2.6062383277	0.455499	1.1577636821
8.816456	-0.20462	-0.520086574	1.054876	2.681226717	0.477961	1.2148566724
8.924051	-0.19157	-0.486914686	1.083297	2.7534670378	0.499342	1.2692015943
9.031646	-0.17951	-0.456271118	1.110724	2.8231790366	0.519729	1.3210181942
9.139241	-0.16795	-0.426895803	1.137652	2.8916227847	0.539616	1.3715665433
9.246835	-0.15768	-0.400777257	1.163299	2.956809763	0.558222	1.4188581227
9.35443	-0.14818	-0.37662449	1.188172	3.0200309614	0.576054	1.4641839222
9.462025	-0.13938	-0.354280316	1.212333	3.0814435678	0.593175	1.5077011296
9.56962	-0.13125	-0.333599913	1.23584	3.1411924031	0.609642	1.5495545657
9.677215	-0.12371	-0.314450688	1.258745	3.1994100602	0.625506	1.5898768241
9.78481	-0.11674	-0.296711034	1.281095	3.2562181461	0.640815	1.6287895109
9.892405	-0.11027	-0.280268873	1.302934	3.3117287393	0.655614	1.6664047051
10	-0.10427	-0.265021021	1.324304	3.3660450238	0.669943	1.7028255907

CH⁻ + He PES

Method: MRCI. AV5Z (120º – 180º)

R(Å) \ θ (º)	180	170	160	150	140	130	120
1.58753	10092.25636	10247.67949	10685.84517	10685.84517	10685.84517	12506.43306	12715.29826
1.68807	7199.8862	7322.129163	7667.397825	7667.397825	7667.397825	9099.116236	9325.466975
1.79391	5074.39541	5170.74037	5443.163218	5443.163218	5443.163218	6566.960959	6755.024355
1.89445	3524.812555	3600.928541	3816.283603	4118.704239	4118.704239	4700.489703	4852.846776
2.00029	2405.53599	2465.707146	2636.012852	2874.333736	2874.333736	3334.236176	3457.89036
2.10083	1605.36452	1652.716165	1787.089488	1975.124352	1975.124352	2340.180844	2441.09307
2.20138	1039.543638	1076.593145	1182.26797	1330.514283	1330.514283	1620.9121	1703.39285
2.30721	644.2325724	673.2383355	756.0768285	872.7100205	872.7100205	1103.270283	1170.442679
2.40776	372.0116417	394.6855627	459.4788541	550.9668411	550.9668411	733.0517482	787.3651275
2.5083	187.8548944	205.5664946	256.0280946	327.4626874	327.4626874	470.3647942	513.967813
2.61413	65.8709117	79.55515301	118.9574279	174.3462327	174.3462327	285.9358984	320.7731016
2.71468	-12.66368442	-2.049892764	28.40001324	71.25681838	117.4957271	158.0612174	185.8093911
2.82051	-61.15879204	-52.98994742	-29.55444964	3.456724951	39.12134745	70.74105307	92.76971867
2.92106	-89.26031982	-82.94384083	-65.07202415	-39.781966	-12.30813557	12.26863014	29.62468151
3.0216	-103.6271271	-98.82282814	-85.27027159	-66.01576493	-44.97473503	-25.96603993	-12.31691455
3.12744	-109.0393708	-105.4312083	-95.17516029	-80.67008398	-64.62429597	-50.02704032	-39.42861189
3.22798	-108.7847802	-106.0764637	-98.39046318	-87.46501759	-75.40269358	-64.27094186	-56.14599217
3.33382	-105.0910227	-103.0652722	-97.33479035	-89.16594574	-80.12578697	-71.82306285	-65.69972151
3.43436	-99.44613601	-97.92956652	-93.6629803	-87.60987083	-80.93564824	-74.7837752	-70.25162471
3.5349	-92.79825037	-91.67015093	-88.51849567	-84.10705621	-79.29397823	-74.7837752	-71.51140892
3.64074	-85.81017911	-84.96959139	-82.64974487	-79.45858418	-76.04575415	-72.93579907	-70.56766814
3.74128	-78.86161327	-78.2426949	-76.55493523	-74.29654159	-71.94157913	-69.8214545	-68.28074281
3.84712	-72.19397492	-71.74624674	-70.54133119	-68.98525627	-67.42040237	-66.0333229	-65.03032398
3.94766	-65.94772781	-65.62729489	-64.78670717	-63.75517655	-62.76754085	-61.90500568	-61.2860873
4.0482	-60.1887143	-59.96485021	-59.39202151	-58.73579245	-58.15418476	-57.66036691	-57.30481806
4.15404	-54.94327136	-54.78963914	-54.40336385	-54.00392008	-53.69446089	-53.44645459	-53.26868017
4.25458	-49.56833841	-50.0972722	-49.82731844	-49.59248062	-49.45421162	-49.36203229	-49.29399516
4.35513	-45.15031471	-45.12397776	-45.05155114	-45.05813538	-45.47294237	-45.46855288	-45.46635814
4.46096	-41.16026648	-41.15807174	-41.13831902	-41.21074564	-41.36876735	-41.8099113	-41.838443
4.56151	-37.5586883	-37.57185677	-37.57844101	-37.67939933	-37.86814748	-38.06347988	-38.20833311
4.66734	-34.31485371	-34.30387998	-34.34996965	-34.45531745	-34.65064985	-34.85695597	-35.01497768
4.76788	-31.39803628	-31.38267305	-31.42876272	-31.5231368	-31.70530072	-31.91160684	-32.07621279
4.86843	-28.7797043	-28.75775684	-28.79506752	-28.86529939	-29.02990534	-29.22304299	-29.38545419
4.97426	-26.44010506	-26.40498913	-26.42913133	-26.46863676	-26.60471101	-26.77809595	-26.93611766
5.07481	-24.35509636	-24.3046172	-24.31120144	-24.31559093	-24.418744	-24.55701299	-24.70406098
5.18064	-21.73237489	-21.73456964	-22.32056682	-22.3864092	-22.45664107	-22.48078328	-22.61685753
5.28119	-19.96340962	-19.96560436	-19.98535708	-20.66572834	-20.69645478	-20.69645478	-20.81716581
5.48757	-16.92368641	-16.92149166	-16.93027064	-16.97636031	-17.25948254	-17.29898797	-17.75110565
5.58811	-15.61781254	-15.61342304	-15.61781254	-15.65512322	-15.73852357	-15.98433512	-16.12260412
5.69394	-14.43045495	-14.4282602	-14.4282602	-14.45898665	-14.52921852	-14.542387	-14.92646755
5.79449	-13.35283466	-13.34844517	-13.34844517	-13.37478212	-13.43623501	-13.52402485	-13.53499858
5.89503	-12.3717832	-12.36739371	-12.36739371	-12.38714642	-12.43982033	-12.51883118	-12.60662102
6.00087	-11.47413209	-11.47193734	-11.4697426	-11.48730056	-11.53339023	-11.6036221	-11.68263296
6.10141	-10.65549183	-10.65329708	-10.65110234	-10.66646556	-10.70597099	-10.76742388	-10.83765575
6.20195	-9.904888697	-9.902693953	-9.902693953	-9.915862428	-9.950978364	-10.00365227	-10.06510516
6.30779	-9.2179332	-9.215738454	-9.215738454	-9.226712183	-9.257438628	-9.303528293	-9.354007452
6.40833	-8.583651607	-8.58145686	-8.583651607	-8.59243059	-8.620962289	-8.658272971	-8.69119416
6.51417	-7.999849171	-7.999849171	-7.999849171	-8.008628154	-8.03277036	-8.052523074	-8.006433408
6.61471	-7.464331146	-7.4621364	-7.4621364	-7.457746908	-7.477499622	-7.420436226	-7.457746908
6.71526	-6.966123803	-6.966123803	-6.952955328	-6.873944472	-6.895891932	-6.922228884	-6.952955328
6.82109	-6.494253413	-6.494253413	-6.410853066	-6.419632049	-6.450358494	-6.474500699	-6.500837652
6.92164	-5.985072341	-5.985072341	-6.002630309	-6.011409294	-6.114562357	-6.136509815	-6.158457276
7.02747	-5.607576029	-5.609770775	-5.699755363	-5.708534346	-5.721702821	-5.741455535	-5.761208249
7.12801	-5.335427526	-5.335427526	-5.339817018	-5.346401255	-5.359569733	-5.3771277	-5.392490922
7.22856	-4.999631388	-5.001826135	-5.004020879	-5.012799864	-5.023773593	-5.039136817	-5.054500038
7.33439	-4.690172202	-4.690172202	-4.694561694	-4.701145933	-4.712119662	-4.725288138	-4.740651359
7.43494	-4.396076238	-4.398270985	-4.402660475	-4.409244714	-4.420218443	-4.428997428	-4.448750142
7.53548	-4.091006544	-4.091006544	-4.095396036	-4.101980273	-4.112954004	-4.115148751	-4.172212147
7.64132	-3.893479403	-3.89567415	-3.900063642	-3.90664788	-3.915426864	-3.875921435	-3.875921435
7.74186	-3.667420565	-3.667420565	-3.671810058	-3.678394296	-3.687173279	-3.695952264	-3.65425209
7.84769	-3.456724951	-3.458919695	-3.461114441	-3.46769868	-3.476477665	-3.483061902	-3.48964614

R(Å) \ θ (º)	180	170	160	150	140	130	120
7.94824	-3.261392556	-3.263587302	-3.265782048	-3.272366285	-3.278950524	-3.287729509	-3.292118999
8.04878	-3.081423383	-3.081423383	-3.085812876	-3.090202368	-3.098981353	-3.10556559	-3.109955082
8.15462	-2.912427941	-2.914622687	-2.916817434	-2.923401672	-2.929985911	-2.934375402	-2.938764894
8.25516	-2.756600976	-2.758795723	-2.760990467	-2.76537996	-2.771964198	-2.778548437	-2.780743181
8.361	-2.611747739	-2.611747739	-2.616137232	-2.620526724	-2.627110963	-2.631500453	-2.6336952
8.46154	-2.475673489	-2.477868235	-2.480062979	-2.484452472	-2.49103671	-2.495426203	-2.497620947
8.56208	-2.350572966	-2.350572966	-2.354962458	-2.35935195	-2.363741443	-2.368130933	-2.37032568
8.66792	-2.232056683	-2.234251427	-2.236446173	-2.240835666	-2.245225158	-2.249614651	-2.251809397
8.76846	-2.122319381	-2.124514128	-2.126708874	-2.131098366	-2.135487858	-2.139877349	-2.139877349
8.8743	-2.021361066	-2.021361066	-2.023555813	-2.027945303	-2.032334796	-2.034529542	-2.036724288
8.97484	-1.924792242	-1.924792242	-1.926986988	-1.931376481	-1.935765972	-1.937960718	-1.940155464
9.07539	-1.834807657	-1.834807657	-1.837002403	-1.841391894	-1.84358664	-1.847976132	-1.847976132
9.18122	-1.749212562	-1.751407308	-1.753602054	-1.7557968	-1.760186293	-1.762381037	-1.762381037
9.28176	-1.670201705	-1.670201705	-1.672396452	-1.676785944	-1.67898069	-1.681175436	-1.681175436
9.38231	-1.595580341	-1.595580341	-1.597775088	-1.599969834	-1.604359326	-1.606554072	-1.606554072
9.48814	-1.52534847	-1.52534847	-1.527543216	-1.529737962	-1.531932708	-1.534127455	-1.534127455
9.58869	-1.459506091	-1.459506091	-1.461700835	-1.463895581	-1.466090328	-1.468285074	-1.468285074
9.69452	-1.395858456	-1.395858456	-1.398053202	-1.400247948	-1.402442695	-1.404637439	-1.404637439
9.79507	-1.336600314	-1.336600314	-1.33879506	-1.340989806	-1.343184552	-1.343184552	-1.343184552
9.89561	-1.279536918	-1.281731664	-1.281731664	-1.28392641	-1.286121156	-1.288315903	-1.288315903
10.0014	-1.226863014	-1.226863014	-1.22905776	-1.231252507	-1.231252507	-1.233447253	-1.233447253
10.102	-1.176383857	-1.176383857	-1.178578601	-1.180773347	-1.180773347	-1.182968094	-1.182968094
10.2078	-1.128099444	-1.13029419	-1.13029419	-1.132488936	-1.132488936	-1.134683682	-1.134683682
10.3084	-1.084204523	-1.084204523	-1.084204523	-1.086399269	-1.088594016	-1.088594016	-1.088594016
10.4089	-1.040309604	-1.040309604	-1.04250435	-1.04250435	-1.044699097	-1.044699097	-1.044699097
10.5147	-0.99860943	-1.000804176	-1.000804176	-1.002998922	-1.002998922	-1.002998922	-1.002998922
10.6153	-0.961298748	-0.961298748	-0.961298748	-0.963493494	-0.963493494	-0.963493494	-0.963493494
10.7211	-0.923988066	-0.923988066	-0.923988066	-0.926182812	-0.926182812	-0.926182812	-0.926182812
10.8217	-0.88887213	-0.88887213	-0.88887213	-0.891066876	-0.891066876	-0.891066876	-0.891066876
10.9222	-0.853756194	-0.853756194	-0.855950941	-0.855950941	-0.858145685	-0.858145685	-0.858145685
11.028	-0.823029749	-0.823029749	-0.823029749	-0.823029749	-0.825224496	-0.825224496	-0.825224496
11.1286	-0.792303306	-0.792303306	-0.792303306	-0.792303306	-0.794498052	-0.794498052	-0.794498052
11.2291	-0.761576863	-0.763771607	-0.763771607	-0.763771607	-0.763771607	-0.763771607	-0.763771607
11.335	-0.73523991	-0.73523991	-0.73523991	-0.73523991	-0.737434656	-0.737434656	-0.73523991
11.4355	-0.708902957	-0.708902957	-0.708902957	-0.708902957	-0.708902957	-0.708902957	-0.708902957
11.5414	-0.682566006	-0.682566006	-0.682566006	-0.682566006	-0.684760753	-0.684760753	-0.684760753
11.6419	-0.6584238	-0.6584238	-0.6584238	-0.6584238	-0.660618546	-0.660618546	-0.6584238
11.7424	-0.634281593	-0.634281593	-0.63647634	-0.63647634	-0.63647634	-0.63647634	-0.63647634
11.8483	-0.612334133	-0.612334133	-0.612334133	-0.614528879	-0.614528879	-0.614528879	-0.612334133
11.9488	-0.590386675	-0.590386675	-0.592581421	-0.592581421	-0.592581421	-0.592581421	-0.592581421
12.0547	-0.57063396	-0.57063396	-0.57063396	-0.57063396	-0.572828707	-0.572828707	-0.57063396
12.1552	-0.550881246	-0.550881246	-0.550881246	-0.550881246	-0.553075993	-0.553075993	-0.550881246
12.2557	-0.533323278	-0.533323278	-0.533323278	-0.533323278	-0.533323278	-0.533323278	-0.533323278
12.3616	-0.513570564	-0.513570564	-0.515765311	-0.515765311	-0.515765311	-0.515765311	-0.515765311
12.4621	-0.498207343	-0.498207343	-0.498207343	-0.498207343	-0.498207343	-0.498207343	-0.498207343
12.5627	-0.480649373	-0.480649373	-0.480649373	-0.480649373	-0.480649373	-0.480649373	-0.480649373
12.6685	-0.465286152	-0.465286152	-0.465286152	-0.465286152	-0.465286152	-0.465286152	-0.465286152
12.769	-0.44992293	-0.44992293	-0.44992293	-0.44992293	-0.44992293	-0.44992293	-0.44992293
12.8749	-0.434559708	-0.434559708	-0.434559708	-0.434559708	-0.434559708	-0.434559708	-0.434559708
12.9754	-0.421391233	-0.421391233	-0.421391233	-0.421391233	-0.421391233	-0.421391233	-0.421391233
13.076	-0.408222755	-0.408222755	-0.408222755	-0.408222755	-0.408222755	-0.408222755	-0.408222755
13.1818	-0.39505428	-0.39505428	-0.39505428	-0.39505428	-0.39505428	-0.39505428	-0.39505428
13.2823	-0.381885804	-0.381885804	-0.381885804	-0.381885804	-0.381885804	-0.381885804	-0.381885804
13.3882	-0.368717327	-0.370912074	-0.370912074	-0.370912074	-0.370912074	-0.370912074	-0.368717327
13.4887	-0.357743598	-0.357743598	-0.357743598	-0.357743598	-0.357743598	-0.357743598	-0.357743598
13.5893	-0.346769869	-0.346769869	-0.346769869	-0.346769869	-0.346769869	-0.346769869	-0.346769869
13.6951	-0.335796138	-0.335796138	-0.335796138	-0.335796138	-0.335796138	-0.335796138	-0.335796138
13.7956	-0.327017155	-0.327017155	-0.327017155	-0.327017155	-0.327017155	-0.327017155	-0.324822408
13.9015	-0.316043424	-0.316043424	-0.316043424	-0.316043424	-0.316043424	-0.316043424	-0.316043424
14.002	-0.30726444	-0.30726444	-0.30726444	-0.30726444	-0.30726444	-0.30726444	-0.305069694
14.1026	-0.29629071	-0.29629071	-0.29629071	-0.298485456	-0.298485456	-0.29629071	-0.29629071
14.2084	-0.287511726	-0.287511726	-0.287511726	-0.287511726	-0.287511726	-0.287511726	-0.287511726
14.3089	-0.278732742	-0.278732742	-0.280927488	-0.280927488	-0.280927488	-0.278732742	-0.278732742

R(Å) \ θ (º)	180	170	160	150	140	130	120
14.4095	-0.272148505	-0.272148505	-0.272148505	-0.272148505	-0.272148505	-0.272148505	-0.269953759
14.5153	-0.26336952	-0.26336952	-0.26336952	-0.26336952	-0.26336952	-0.26336952	-0.26336952
14.6159	-0.256785281	-0.256785281	-0.256785281	-0.256785281	-0.256785281	-0.254590535	-0.254590535
14.7217	-0.248006298	-0.248006298	-0.248006298	-0.248006298	-0.248006298	-0.248006298	-0.248006298
14.8222	-0.24142206	-0.24142206	-0.24142206	-0.24142206	-0.24142206	-0.24142206	-0.24142206
14.9228	-0.234837823	-0.234837823	-0.234837823	-0.234837823	-0.234837823	-0.234837823	-0.232643077
15.0286	-0.228253584	-0.228253584	-0.228253584	-0.228253584	-0.228253584	-0.228253584	-0.226058838
15.1292	-0.221669346	-0.221669346	-0.221669346	-0.221669346	-0.221669346	-0.221669346	-0.219474599
15.235	-0.215085109	-0.215085109	-0.215085109	-0.215085109	-0.215085109	-0.215085109	-0.215085109
15.3355	-0.20850087	-0.20850087	-0.20850087	-0.20850087	-0.20850087	-0.20850087	-0.20850087
15.4361	-0.201916632	-0.201916632	-0.201916632	-0.201916632	-0.201916632	-0.201916632	-0.201916632
15.5419	-0.197527139	-0.197527139	-0.197527139	-0.197527139	-0.197527139	-0.197527139	-0.197527139
15.6425	-0.190942902	-0.190942902	-0.190942902	-0.190942902	-0.190942902	-0.190942902	-0.190942902
15.7483	-0.18655341	-0.18655341	-0.18655341	-0.18655341	-0.18655341	-0.18655341	-0.18655341
15.8489	-0.182163917	-0.182163917	-0.182163917	-0.182163917	-0.182163917	-0.179969171	-0.179969171
15.9494	-0.17557968	-0.17557968	-0.17557968	-0.17557968	-0.17557968	-0.17557968	-0.17557968
16.0552	-0.171190188	-0.171190188	-0.171190188	-0.171190188	-0.171190188	-0.171190188	-0.171190188
16.1558	-0.166800696	-0.166800696	-0.166800696	-0.166800696	-0.166800696	-0.166800696	-0.166800696
16.2563	-0.162411203	-0.162411203	-0.162411203	-0.162411203	-0.162411203	-0.162411203	-0.162411203
16.3622	-0.158021713	-0.158021713	-0.158021713	-0.158021713	-0.158021713	-0.158021713	-0.158021713
16.4627	-0.15363222	-0.15363222	-0.15363222	-0.15363222	-0.15363222	-0.15363222	-0.15363222
16.5685	-0.149242728	-0.149242728	-0.149242728	-0.149242728	-0.149242728	-0.149242728	-0.149242728
16.6691	-0.144853236	-0.144853236	-0.144853236	-0.144853236	-0.144853236	-0.144853236	-0.144853236
16.7696	-0.142658489	-0.142658489	-0.142658489	-0.142658489	-0.142658489	-0.142658489	-0.140463745
16.8755	-0.138268999	-0.138268999	-0.138268999	-0.138268999	-0.138268999	-0.138268999	-0.138268999
16.976	-0.133879506	-0.133879506	-0.133879506	-0.133879506	-0.133879506	-0.133879506	-0.133879506
17.0818	-0.13168476	-0.13168476	-0.13168476	-0.13168476	-0.13168476	-0.13168476	-0.129490014
17.1824	-0.127295268	-0.127295268	-0.127295268	-0.127295268	-0.127295268	-0.127295268	-0.127295268
17.2829	-0.125100521	-0.125100521	-0.125100521	-0.125100521	-0.125100521	-0.122905775	-0.122905775
17.3888	-0.120711031	-0.120711031	-0.120711031	-0.120711031	-0.120711031	-0.120711031	-0.120711031
17.4893	-0.118516284	-0.118516284	-0.118516284	-0.118516284	-0.118516284	-0.118516284	-0.118516284
17.5898	-0.114126792	-0.114126792	-0.114126792	-0.114126792	-0.114126792	-0.114126792	-0.114126792
17.6957	-0.111932046	-0.111932046	-0.111932046	-0.111932046	-0.111932046	-0.111932046	-0.111932046
17.7962	-0.1097373	-0.1097373	-0.1097373	-0.1097373	-0.1097373	-0.1097373	-0.107542554
17.9021	-0.105347807	-0.105347807	-0.105347807	-0.105347807	-0.105347807	-0.105347807	-0.105347807
18.0026	-0.103153063	-0.103153063	-0.103153063	-0.103153063	-0.103153063	-0.103153063	-0.103153063
18.1031	-0.100958317	-0.100958317	-0.100958317	-0.100958317	-0.100958317	-0.100958317	-0.100958317
18.209	-0.09876357	-0.09876357	-0.09876357	-0.09876357	-0.09876357	-0.09876357	-0.09876357
18.3095	-0.096568824	-0.096568824	-0.096568824	-0.096568824	-0.096568824	-0.096568824	-0.096568824
18.4154	-0.094374078	-0.094374078	-0.094374078	-0.094374078	-0.094374078	-0.092179332	-0.092179332
18.5159	-0.092179332	-0.092179332	-0.092179332	-0.092179332	-0.089984586	-0.089984586	-0.089984586
18.6164	-0.087789839	-0.087789839	-0.087789839	-0.087789839	-0.087789839	-0.087789839	-0.087789839
18.7223	-0.085595093	-0.085595093	-0.085595093	-0.085595093	-0.085595093	-0.085595093	-0.085595093
18.8228	-0.083400349	-0.083400349	-0.083400349	-0.083400349	-0.083400349	-0.083400349	-0.083400349
18.9287	-0.083400349	-0.083400349	-0.083400349	-0.083400349	-0.081205602	-0.081205602	-0.081205602
19.0292	-0.081205602	-0.081205602	-0.081205602	-0.081205602	-0.081205602	-0.081205602	-0.079010856
19.1297	-0.079010856	-0.079010856	-0.079010856	-0.079010856	-0.079010856	-0.079010856	-0.079010856
19.2356	-0.07681611	-0.07681611	-0.07681611	-0.07681611	-0.07681611	-0.07681611	-0.07681611
19.3361	-0.074621364	-0.074621364	-0.074621364	-0.074621364	-0.074621364	-0.074621364	-0.074621364
19.4367	-0.072426618	-0.072426618	-0.072426618	-0.072426618	-0.072426618	-0.072426618	-0.072426618
19.5425	-0.070231872	-0.070231872	-0.070231872	-0.070231872	-0.070231872	-0.070231872	-0.070231872
19.6431	-0.070231872	-0.070231872	-0.070231872	-0.068037125	-0.068037125	-0.068037125	-0.068037125
19.7489	-0.068037125	-0.068037125	-0.068037125	-0.068037125	-0.068037125	-0.068037125	-0.068037125
19.8494	-0.065842379	-0.065842379	-0.065842379	-0.065842379	-0.065842379	-0.065842379	-0.065842379
19.95	-0.063647635	-0.063647635	-0.063647635	-0.063647635	-0.063647635	-0.063647635	-0.063647635
20.0558	-0.063647635	-0.063647635	-0.063647635	-0.063647635	-0.063647635	-0.061452888	-0.061452888
20.1564	-0.061452888	-0.061452888	-0.061452888	-0.061452888	-0.061452888	-0.061452888	-0.061452888
20.2622	-0.059258142	-0.059258142	-0.059258142	-0.059258142	-0.059258142	-0.059258142	-0.059258142
20.3627	-0.059258142	-0.059258142	-0.059258142	-0.057063396	-0.057063396	-0.057063396	-0.057063396
20.4633	-0.057063396	-0.057063396	-0.057063396	-0.057063396	-0.057063396	-0.057063396	-0.057063396
20.5691	-0.05486865	-0.05486865	-0.05486865	-0.05486865	-0.05486865	-0.05486865	-0.05486865
20.6697	-0.05486865	-0.05486865	-0.05486865	-0.05486865	-0.05486865	-0.05486865	-0.05486865
20.7755	-0.052673904	-0.052673904	-0.052673904	-0.052673904	-0.052673904	-0.052673904	-0.052673904

R(Å) \ θ (º)	180	170	160	150	140	130	120
20.876	-0.050479157	-0.050479157	-0.050479157	-0.050479157	-0.050479157	-0.050479157	-0.050479157
20.9766	-0.050479157	-0.050479157	-0.050479157	-0.050479157	-0.050479157	-0.050479157	-0.050479157
21.0824	-0.048284411	-0.048284411	-0.048284411	-0.048284411	-0.048284411	-0.048284411	-0.048284411
21.183	-0.048284411	-0.048284411	-0.048284411	-0.048284411	-0.048284411	-0.048284411	-0.048284411
21.2835	-0.046089667	-0.046089667	-0.046089667	-0.046089667	-0.046089667	-0.046089667	-0.046089667
21.3893	-0.046089667	-0.046089667	-0.046089667	-0.046089667	-0.046089667	-0.046089667	-0.046089667
21.4899	-0.043894921	-0.043894921	-0.043894921	-0.043894921	-0.043894921	-0.043894921	-0.043894921
21.5957	-0.043894921	-0.043894921	-0.043894921	-0.043894921	-0.043894921	-0.043894921	-0.043894921
21.6963	-0.041700174	-0.041700174	-0.041700174	-0.041700174	-0.041700174	-0.041700174	-0.041700174
21.7968	-0.041700174	-0.041700174	-0.041700174	-0.041700174	-0.041700174	-0.041700174	-0.041700174
21.9026	-0.039505428	-0.039505428	-0.039505428	-0.039505428	-0.039505428	-0.039505428	-0.039505428
22.0032	-0.039505428	-0.039505428	-0.039505428	-0.039505428	-0.039505428	-0.039505428	-0.039505428
22.109	-0.037310682	-0.037310682	-0.037310682	-0.037310682	-0.037310682	-0.037310682	-0.037310682
22.2096	-0.037310682	-0.037310682	-0.037310682	-0.037310682	-0.037310682	-0.037310682	-0.037310682
22.3101	-0.037310682	-0.037310682	-0.037310682	-0.037310682	-0.037310682	-0.037310682	-0.035115936
22.4159	-0.035115936	-0.035115936	-0.035115936	-0.035115936	-0.035115936	-0.035115936	-0.035115936
22.5165	-0.035115936	-0.035115936	-0.035115936	-0.035115936	-0.035115936	-0.035115936	-0.035115936
22.617	-0.03292119	-0.03292119	-0.03292119	-0.03292119	-0.03292119	-0.03292119	-0.03292119
22.7229	-0.03292119	-0.03292119	-0.03292119	-0.03292119	-0.03292119	-0.03292119	-0.03292119
22.8234	-0.03292119	-0.03292119	-0.03292119	-0.03292119	-0.03292119	-0.03292119	-0.03292119
22.9292	-0.030726443	-0.030726443	-0.030726443	-0.030726443	-0.030726443	-0.030726443	-0.030726443
23.0298	-0.030726443	-0.030726443	-0.030726443	-0.030726443	-0.030726443	-0.030726443	-0.030726443
23.1303	-0.030726443	-0.030726443	-0.030726443	-0.030726443	-0.030726443	-0.030726443	-0.028531697
23.2362	-0.028531697	-0.028531697	-0.028531697	-0.028531697	-0.028531697	-0.028531697	-0.028531697
23.3367	-0.028531697	-0.028531697	-0.028531697	-0.028531697	-0.028531697	-0.028531697	-0.028531697
23.4425	-0.028531697	-0.028531697	-0.028531697	-0.028531697	-0.028531697	-0.028531697	-0.026336953
23.5431	-0.026336953	-0.026336953	-0.026336953	-0.026336953	-0.026336953	-0.026336953	-0.026336953
23.6436	-0.026336953	-0.026336953	-0.026336953	-0.026336953	-0.026336953	-0.026336953	-0.026336953
23.7495	-0.026336953	-0.026336953	-0.026336953	-0.026336953	-0.026336953	-0.026336953	-0.026336953
23.85	-0.024142206	-0.024142206	-0.024142206	-0.024142206	-0.024142206	-0.024142206	-0.024142206
23.9558	-0.024142206	-0.024142206	-0.024142206	-0.024142206	-0.024142206	-0.024142206	-0.024142206
24.0564	-0.024142206	-0.024142206	-0.024142206	-0.024142206	-0.024142206	-0.024142206	-0.024142206
24.1569	-0.02194746	-0.02194746	-0.02194746	-0.02194746	-0.02194746	-0.02194746	-0.02194746
24.2628	-0.02194746	-0.02194746	-0.02194746	-0.02194746	-0.02194746	-0.02194746	-0.02194746
24.3633	-0.02194746	-0.02194746	-0.02194746	-0.02194746	-0.02194746	-0.02194746	-0.02194746
24.4639	-0.02194746	-0.02194746	-0.02194746	-0.02194746	-0.02194746	-0.02194746	-0.02194746
24.5697	-0.019752714	-0.019752714	-0.019752714	-0.019752714	-0.019752714	-0.019752714	-0.019752714
24.6702	-0.019752714	-0.019752714	-0.019752714	-0.019752714	-0.019752714	-0.019752714	-0.019752714
24.7761	-0.019752714	-0.019752714	-0.019752714	-0.019752714	-0.019752714	-0.019752714	-0.019752714
24.8766	-0.019752714	-0.019752714	-0.019752714	-0.019752714	-0.019752714	-0.019752714	-0.019752714
24.9772	-0.017557968	-0.017557968	-0.017557968	-0.017557968	-0.017557968	-0.017557968	-0.017557968
25.083	-0.017557968	-0.017557968	-0.017557968	-0.017557968	-0.017557968	-0.017557968	-0.017557968
25.1835	-0.017557968	-0.017557968	-0.017557968	-0.017557968	-0.017557968	-0.017557968	-0.017557968
25.2894	-0.017557968	-0.017557968	-0.017557968	-0.017557968	-0.017557968	-0.017557968	-0.017557968
25.3899	-0.017557968	-0.017557968	-0.017557968	-0.017557968	-0.017557968	-0.017557968	-0.015363222
25.4905	-0.015363222	-0.015363222	-0.015363222	-0.015363222	-0.015363222	-0.015363222	-0.015363222
25.5963	-0.015363222	-0.015363222	-0.015363222	-0.015363222	-0.015363222	-0.015363222	-0.015363222
25.6968	-0.015363222	-0.015363222	-0.015363222	-0.015363222	-0.015363222	-0.015363222	-0.015363222
25.8027	-0.015363222	-0.015363222	-0.015363222	-0.015363222	-0.015363222	-0.015363222	-0.015363222
25.9032	-0.015363222	-0.015363222	-0.015363222	-0.015363222	-0.013168476	-0.013168476	-0.013168476
26.0038	-0.013168476	-0.013168476	-0.013168476	-0.013168476	-0.013168476	-0.013168476	-0.013168476
26.1096	-0.013168476	-0.013168476	-0.013168476	-0.013168476	-0.013168476	-0.013168476	-0.013168476
26.2101	-0.013168476	-0.013168476	-0.013168476	-0.013168476	-0.013168476	-0.013168476	-0.013168476
26.3107	-0.013168476	-0.013168476	-0.013168476	-0.013168476	-0.013168476	-0.013168476	-0.013168476
26.4165	-0.013168476	-0.013168476	-0.013168476	-0.013168476	-0.013168476	-0.013168476	-0.013168476
26.5171	-0.010973729	-0.010973729	-0.010973729	-0.010973729	-0.010973729	-0.010973729	-0.010973729
26.6229	-0.010973729	-0.010973729	-0.010973729	-0.010973729	-0.010973729	-0.010973729	-0.010973729
26.7234	-0.010973729	-0.010973729	-0.010973729	-0.010973729	-0.010973729	-0.010973729	-0.010973729
26.824	-0.010973729	-0.010973729	-0.010973729	-0.010973729	-0.010973729	-0.010973729	-0.010973729
26.9298	-0.010973729	-0.010973729	-0.010973729	-0.010973729	-0.010973729	-0.010973729	-0.010973729
27.0304	-0.010973729	-0.010973729	-0.010973729	-0.010973729	-0.010973729	-0.010973729	-0.010973729
27.1362	-0.008778985	-0.008778985	-0.008778985	-0.008778985	-0.008778985	-0.008778985	-0.008778985
27.2367	-0.008778985	-0.008778985	-0.008778985	-0.008778985	-0.008778985	-0.008778985	-0.008778985

R(Å) \ θ (º)	180	170	160	150	140	130	120
27.3373	-0.008778985	-0.008778985	-0.008778985	-0.008778985	-0.008778985	-0.008778985	-0.008778985
27.4431	-0.008778985	-0.008778985	-0.008778985	-0.008778985	-0.008778985	-0.008778985	-0.008778985
27.5437	-0.008778985	-0.008778985	-0.008778985	-0.008778985	-0.008778985	-0.008778985	-0.008778985
27.6442	-0.008778985	-0.008778985	-0.008778985	-0.008778985	-0.008778985	-0.008778985	-0.008778985
27.75	-0.008778985	-0.008778985	-0.008778985	-0.008778985	-0.008778985	-0.008778985	-0.008778985
27.8506	-0.006584239	-0.006584239	-0.006584239	-0.006584239	-0.006584239	-0.006584239	-0.006584239
27.9564	-0.006584239	-0.006584239	-0.006584239	-0.006584239	-0.006584239	-0.006584239	-0.006584239
28.057	-0.006584239	-0.006584239	-0.006584239	-0.006584239	-0.006584239	-0.006584239	-0.006584239
28.1575	-0.006584239	-0.006584239	-0.006584239	-0.006584239	-0.006584239	-0.006584239	-0.006584239
28.2633	-0.006584239	-0.006584239	-0.006584239	-0.006584239	-0.006584239	-0.006584239	-0.006584239
28.3639	-0.006584239	-0.006584239	-0.006584239	-0.006584239	-0.006584239	-0.006584239	-0.006584239
28.4697	-0.006584239	-0.006584239	-0.006584239	-0.006584239	-0.006584239	-0.006584239	-0.006584239
28.5703	-0.006584239	-0.006584239	-0.006584239	-0.006584239	-0.006584239	-0.004389492	-0.004389492
28.6708	-0.004389492	-0.004389492	-0.004389492	-0.004389492	-0.004389492	-0.004389492	-0.004389492
28.7766	-0.004389492	-0.004389492	-0.004389492	-0.004389492	-0.004389492	-0.004389492	-0.004389492
28.8772	-0.004389492	-0.004389492	-0.004389492	-0.004389492	-0.004389492	-0.004389492	-0.004389492
28.983	-0.004389492	-0.004389492	-0.004389492	-0.004389492	-0.004389492	-0.004389492	-0.004389492
29.0836	-0.004389492	-0.004389492	-0.004389492	-0.004389492	-0.004389492	-0.004389492	-0.004389492
29.1841	-0.004389492	-0.004389492	-0.004389492	-0.004389492	-0.004389492	-0.004389492	-0.004389492
29.2899	-0.004389492	-0.004389492	-0.004389492	-0.004389492	-0.004389492	-0.004389492	-0.004389492
29.3905	-0.004389492	-0.004389492	-0.004389492	-0.004389492	-0.004389492	-0.004389492	-0.004389492
29.491	-0.004389492	-0.004389492	-0.004389492	-0.004389492	-0.004389492	-0.004389492	-0.002194746
29.5969	-0.002194746	-0.002194746	-0.002194746	-0.002194746	-0.002194746	-0.002194746	-0.002194746
29.6974	-0.002194746	-0.002194746	-0.002194746	-0.002194746	-0.002194746	-0.002194746	-0.002194746
29.8032	-0.002194746	-0.002194746	-0.002194746	-0.002194746	-0.002194746	-0.002194746	-0.002194746
29.9038	-0.002194746	-0.002194746	-0.002194746	-0.002194746	-0.002194746	-0.002194746	-0.002194746
30.0043	-0.002194746	-0.002194746	-0.002194746	-0.002194746	-0.002194746	-0.002194746	-0.002194746
30.1102	-0.002194746	-0.002194746	-0.002194746	-0.002194746	-0.002194746	-0.002194746	-0.002194746
30.2107	-0.002194746	-0.002194746	-0.002194746	-0.002194746	-0.002194746	-0.002194746	-0.002194746
30.3166	-0.002194746	-0.002194746	-0.002194746	-0.002194746	-0.002194746	-0.002194746	-0.002194746
30.4171	-0.002194746	-0.002194746	-0.002194746	-0.002194746	-0.002194746	-0.002194746	-0.002194746
30.5176	-0.002194746	-0.002194746	-0.002194746	-0.002194746	-0.002194746	-0.002194746	-0.002194746
30.6235	-0.002194746	-0.002194746	-0.002194746	0.0000000	0.0000000	0.0000000	0.0000000
30.724	0.0000000	0.0000000	0.0000000	0.0000000	0.0000000	0.0000000	0.0000000
30.8299	0.0000000	0.0000000	0.0000000	0.0000000	0.0000000	0.0000000	0.0000000
30.9304	0.0000000	0.0000000	0.0000000	0.0000000	0.0000000	0.0000000	0.0000000
31.0309	0.0000000	0.0000000	0.0000000	0.0000000	0.0000000	0.0000000	0.0000000
31.1368	0.0000000	0.0000000	0.0000000	0.0000000	0.0000000	0.0000000	0.0000000
31.2373	0.0000000	0.0000000	0.0000000	0.0000000	0.0000000	0.0000000	0.0000000
31.3379	0.0000000	0.0000000	0.0000000	0.0000000	0.0000000	0.0000000	0.0000000
31.4437	0.0000000	0.0000000	0.0000000	0.0000000	0.0000000	0.0000000	0.0000000
31.5442	-0.006584239	-0.006584239	-0.006584239	-0.006584239	-0.006584239	-0.006584239	-0.006584239
31.6501	-0.048284411	-0.048284411	-0.048284411	-0.048284411	-0.048284411	-0.048284411	-0.048284411
31.7506	-0.316043424	-0.316043424	-0.316043424	-0.316043424	-0.313848677	-0.316043424	-0.316043424

CH⁻ + He PES

Method: MRCI. AV5Z (60º – 110º)

R(Å) \ θ (º)	110	100	90	80	70	60
1.58753	6409.345275	4737.269256	4737.269256	3929.58956	5197.35825	8073.338045
1.68807	9224.655707	9224.655707	9224.655707	2847.935331	3742.781559	5891.229393
1.79391	6771.987547	6652.299268	6470.855227	2065.348165	6882.479839	7776.663255
1.89445	4876.563201	4799.490306	4694.574862	4671.685856	4871.90595	5469.291669
2.00029	3481.740665	3424.073714	3335.526686	3294.952417	3408.010368	3806.93179
2.10083	2463.878923	2420.480016	2346.881403	2300.190377	2358.23263	2619.260356
2.20138	1724.56337	1692.309382	1632.355506	1585.993691	1609.907644	1776.286949
2.30721	1189.758638	1166.173898	1118.187971	1075.557225	1079.376083	1182.182375
2.40776	804.539015	787.5824074	749.7976602	712.7086476	705.73155	766.9100948
2.5083	528.9667072	517.1436105	487.8547251	456.7332268	444.7608874	479.2798525
2.61413	333.7286873	325.6586062	303.272197	277.8833753	264.2671711	281.9897451
2.71468	196.8906637	191.6101048	174.6666657	154.2686963	140.8039296	148.145355
2.82051	102.1215314	98.79429644	86.05160117	69.83681772	57.43430807	58.5711865
2.92106	37.38969286	35.3639423	25.80143398	13.0016753	2.063061241	-0.278732742
3.0216	-5.99165658	-7.19876688	-14.38436528	-24.48897587	-33.84298332	-37.89448444
3.12744	-34.38728033	-35.11374125	-40.53476387	-48.46438117	-56.27548219	-60.91298048
3.22798	-52.22398107	-52.69585146	-56.8153897	-63.02432614	-69.42201073	-73.96513495
3.33382	-62.73461966	-63.09455801	-66.24182377	-71.05490175	-76.23669706	-80.31234038
3.43436	-68.07663143	-68.42559604	-70.81567444	-74.60380603	-78.67286512	-82.12520057
3.5349	-69.97289197	-70.2845459	-72.14349577	-75.04275523	-78.20538422	-81.01246435
3.64074	-69.52516379	-69.83242823	-71.24584465	-73.42742218	-75.84164278	-78.05614149
3.74128	-67.55428188	-67.84837784	-68.90624542	-70.49085203	-72.29712799	-74.00683512
3.84712	-64.58040105	-64.84816006	-65.6229054	-66.75319959	-68.07882617	-69.38031055
3.94766	-61.07319694	-61.25536086	-61.8106316	-62.59196117	-63.54448094	-64.52992189
4.0482	-57.20824924	-57.35749196	-57.74596201	-58.2705063	-58.94429332	-59.68611747
4.15404	-53.23795372	-53.35647001	-53.62203427	-53.97099889	-54.43409029	-54.99375052
4.25458	-49.30277414	-49.39275873	-49.57492265	-49.80317623	-50.12360915	-50.54719513
4.35513	-45.49050034	-45.51464255	-45.64193782	-45.84385445	-46.06991329	-46.39473569
4.46096	-41.86916944	-41.93501182	-42.0293859	-42.13692845	-42.30372915	-42.55393019
4.56151	-38.31368092	-38.41902873	-38.53973976	-38.66922977	-38.82505674	-39.03355761
4.66734	-35.12471498	-35.23006279	-35.35296857	-35.49343231	-35.63828555	-35.81605997
4.76788	-32.19253433	-32.30007688	-32.42737215	-32.58319912	-32.72585761	-32.88387932
4.86843	-29.50616522	-29.61809727	-29.75636627	-29.92755646	-30.07460444	-30.21726293
4.97426	-27.05902343	-27.17753972	-27.3245877	-27.50894636	-27.65818909	-27.78987385
5.07481	-24.84232997	-24.9674305	-25.12106272	-25.31200562	-25.46344309	-25.58195938
5.18064	-22.8253584	-22.96801689	-23.12384386	-23.3169815	-23.46622423	-23.57157204
5.28119	-20.93348735	-21.16174093	-21.3175679	-21.50631605	-21.64897454	-21.73895913
5.48757	-17.84986922	-18.06714907	-18.20541807	-18.36782927	-18.48634556	-18.4358664
5.58811	-16.27843108	-16.5132689	-16.67568011	-16.84906504	-16.97855506	-17.04659218
5.69394	-15.16130537	-15.29957437	-15.45320659	-15.60903355	-15.72316034	-15.41150641
5.79449	-13.72155199	-13.82909455	-13.94761083	-14.0749061	-14.17366967	-14.21756459
5.89503	-12.69660561	-12.79317443	-12.89852224	-13.00825954	-13.09165989	-13.12458108
6.00087	-11.76164381	-11.84723891	-11.93941824	-12.03159757	-12.0996347	-12.12158216
6.10141	-10.90788762	-10.98250898	-11.06371459	-11.13833595	-11.19100985	-11.20417833
6.20195	-10.12875279	-10.19240042	-10.2582428	-10.31750095	-10.35920112	-10.36139587
6.30779	-9.413265595	-9.465939498	-9.520808148	-9.566897813	-9.595429512	-9.59104002
6.40833	-8.754841793	-8.80093146	-8.84482638	-8.87774757	-8.897500284	-8.884331809
6.51417	-8.149091899	-8.188597327	-8.223713263	-8.245660721	-8.254439706	-8.236881738
6.61471	-7.578457938	-7.624547603	-7.653079302	-7.664053031	-7.666247778	-7.642105573
6.71526	-6.97929228	-7.091224326	-7.128535008	-7.132924501	-7.126340262	-7.10000331
6.82109	-6.509616637	-6.527174604	-6.641301397	-6.643496141	-6.632522412	-6.599601222
6.92164	-6.09042015	-6.088225404	-6.182599482	-6.193573211	-6.176015244	-6.143094054
7.02747	-5.776571471	-5.684392139	-5.684392139	-5.780960964	-5.759013503	-5.723897568
7.12801	-5.405659398	-5.324453795	-5.309090574	-5.399075161	-5.3771277	-5.339817018
7.22856	-5.063279021	-5.067668514	-4.977683928	-5.0479158	-5.023773593	-4.986462911
7.33439	-4.747235598	-4.749430344	-4.745040852	-4.725288138	-4.701145933	-4.661640505
7.43494	-4.455334381	-4.455334381	-4.448750142	-4.428997428	-4.402660475	-4.365349793
7.53548	-4.185380622	-4.185380622	-4.174406891	-4.154654179	-4.128317226	-4.091006544
7.64132	-3.926400595	-3.932984831	-3.922011102	-3.902258388	-3.875921435	-3.838610753
7.74186	-3.647667851	-3.691562772	-3.689368026	-3.669615312	-3.641083614	-3.608162425
7.84769	-3.441361727	-3.430387998	-3.474282919	-3.454530204	-3.428193252	-3.393077316

R(Å) \ θ (º)	110	100	90	80	70	60
7.94824	-3.292118999	-3.239445096	-3.267976795	-3.254808318	-3.228471366	-3.197744921
8.04878	-3.109955082	-3.103370843	-3.035333718	-3.0726444	-3.046307447	-3.015581004
8.15462	-2.938764894	-2.932180655	-2.872922515	-2.901454212	-2.877312005	-2.846585562
8.25516	-2.780743181	-2.774158944	-2.763185213	-2.743432499	-2.719290294	-2.690758596
8.361	-2.6336952	-2.627110963	-2.616137232	-2.598579264	-2.574437057	-2.548100106
8.46154	-2.497620947	-2.49103671	-2.480062979	-2.462505011	-2.438362807	-2.4142206
8.56208	-2.37032568	-2.363741443	-2.352767712	-2.335209744	-2.313262284	-2.289120079
8.66792	-2.249614651	-2.245225158	-2.234251427	-2.216693459	-2.196940745	-2.17279854
8.76846	-2.139877349	-2.133293112	-2.122319381	-2.10695616	-2.087203446	-2.065255985
8.8743	-2.034529542	-2.03014005	-2.01916632	-2.003803098	-1.986245131	-1.96429767
8.97484	-1.937960718	-1.931376481	-1.922597496	-1.907234274	-1.889676306	-1.869923592
9.07539	-1.845781386	-1.841391894	-1.830418164	-1.817249689	-1.801886465	-1.782133751
9.18122	-1.760186293	-1.7557968	-1.747017816	-1.73384934	-1.716291372	-1.698733404
9.28176	-1.681175436	-1.674591198	-1.665812215	-1.654838484	-1.639475262	-1.621917294
9.38231	-1.604359326	-1.599969834	-1.591190851	-1.58021712	-1.564853898	-1.54729593
9.48814	-1.534127455	-1.527543216	-1.520958978	-1.509985248	-1.494622026	-1.479258803
9.58869	-1.466090328	-1.461700835	-1.452921852	-1.441948121	-1.428779646	-1.413416424
9.69452	-1.402442695	-1.398053202	-1.389274218	-1.380495234	-1.367326757	-1.354158282
9.79507	-1.343184552	-1.33879506	-1.330016075	-1.321237092	-1.308068617	-1.29490014
9.89561	-1.286121156	-1.281731664	-1.275147425	-1.264173696	-1.253199965	-1.24003149
10.0014	-1.231252507	-1.226863014	-1.220278776	-1.211499792	-1.200526062	-1.189552332
10.102	-1.180773347	-1.176383857	-1.169799618	-1.16321538	-1.15224165	-1.139073175
10.2078	-1.132488936	-1.128099444	-1.123709951	-1.114930968	-1.103957237	-1.092983508
10.3084	-1.086399269	-1.082009779	-1.077620286	-1.068841302	-1.060062318	-1.049088587
10.4089	-1.04250435	-1.040309604	-1.033725366	-1.027141127	-1.018362144	-1.007388415
10.5147	-1.000804176	-0.99860943	-0.994219938	-0.985440954	-0.97666197	-0.967882987
10.6153	-0.963493494	-0.959104002	-0.954714509	-0.948130272	-0.939351288	-0.930572305
10.7211	-0.926182812	-0.92179332	-0.917403827	-0.910819591	-0.902040606	-0.893261623
10.8217	-0.88887213	-0.886677384	-0.882287892	-0.875703655	-0.869119416	-0.860340431
10.9222	-0.855950941	-0.851561448	-0.847171956	-0.842782464	-0.836198227	-0.827419242
11.028	-0.823029749	-0.820835003	-0.816445512	-0.809861274	-0.803277035	-0.796692798
11.1286	-0.792303306	-0.79010856	-0.785719068	-0.779134831	-0.774745338	-0.765966353
11.2291	-0.763771607	-0.759382116	-0.75718737	-0.750603132	-0.744018895	-0.737434656
11.335	-0.73523991	-0.733045164	-0.728655671	-0.724266181	-0.717681942	-0.711097704
11.4355	-0.708902957	-0.706708211	-0.70231872	-0.697929228	-0.69134499	-0.684760753
11.5414	-0.682566006	-0.68037126	-0.675981768	-0.671592275	-0.667202785	-0.660618546
11.6419	-0.6584238	-0.656229054	-0.651839561	-0.647450071	-0.643060578	-0.63647634
11.7424	-0.634281593	-0.632086847	-0.629892103	-0.62550261	-0.621113118	-0.614528879
11.8483	-0.612334133	-0.610139389	-0.607944642	-0.60355515	-0.599165658	-0.592581421
11.9488	-0.590386675	-0.588191928	-0.585997182	-0.58160769	-0.577218197	-0.572828707
12.0547	-0.57063396	-0.568439214	-0.566244468	-0.561854976	-0.557465483	-0.553075993
12.1552	-0.550881246	-0.5486865	-0.546491754	-0.542102262	-0.537712769	-0.533323278
12.2557	-0.531128532	-0.528933786	-0.52673904	-0.524544294	-0.520154801	-0.515765311
12.3616	-0.513570564	-0.511375818	-0.509181072	-0.506986326	-0.502596833	-0.498207343
12.4621	-0.496012596	-0.49381785	-0.491623104	-0.489428358	-0.485038866	-0.480649373
12.5627	-0.480649373	-0.478454629	-0.476259882	-0.47187039	-0.469675644	-0.465286152
12.6685	-0.463091405	-0.463091405	-0.460896661	-0.456507168	-0.454312422	-0.44992293
12.769	-0.447728184	-0.447728184	-0.445533437	-0.441143947	-0.4389492	-0.434559708
12.8749	-0.434559708	-0.432364962	-0.430170216	-0.42797547	-0.423585979	-0.421391233
12.9754	-0.419196486	-0.419196486	-0.41700174	-0.414806994	-0.410417502	-0.408222755
13.076	-0.406028009	-0.403833265	-0.403833265	-0.399443772	-0.397249026	-0.39505428
13.1818	-0.392859534	-0.392859534	-0.390664788	-0.388470041	-0.384080551	-0.381885804
13.2823	-0.379691058	-0.379691058	-0.377496312	-0.375301566	-0.37310682	-0.368717327
13.3882	-0.368717327	-0.366522583	-0.366522583	-0.364327837	-0.359938344	-0.357743598
13.4887	-0.357743598	-0.355548852	-0.353354106	-0.351159359	-0.348964613	-0.346769869
13.5893	-0.346769869	-0.344575122	-0.342380376	-0.34018563	-0.337990884	-0.335796138
13.6951	-0.335796138	-0.333601392	-0.333601392	-0.331406645	-0.329211901	-0.324822408
13.7956	-0.324822408	-0.324822408	-0.322627662	-0.320432916	-0.31823817	-0.316043424
13.9015	-0.316043424	-0.313848677	-0.311653931	-0.311653931	-0.309459187	-0.30726444
14.002	-0.305069694	-0.305069694	-0.302874948	-0.300680202	-0.298485456	-0.29629071
14.1026	-0.29629071	-0.294095963	-0.294095963	-0.291901217	-0.289706473	-0.287511726
14.2084	-0.287511726	-0.28531698	-0.28531698	-0.283122234	-0.280927488	-0.278732742
14.3089	-0.278732742	-0.278732742	-0.276537995	-0.274343249	-0.272148505	-0.272148505

R(Å) \ θ (º)	110	100	90	80	70	60
14.4095	-0.269953759	-0.269953759	-0.267759012	-0.267759012	-0.265564266	-0.26336952
14.5153	-0.26336952	-0.261174774	-0.261174774	-0.258980028	-0.256785281	-0.254590535
14.6159	-0.254590535	-0.254590535	-0.252395791	-0.250201044	-0.250201044	-0.248006298
14.7217	-0.248006298	-0.245811552	-0.245811552	-0.243616806	-0.24142206	-0.24142206
14.8222	-0.239227314	-0.239227314	-0.239227314	-0.237032567	-0.234837823	-0.232643077
14.9228	-0.232643077	-0.232643077	-0.23044833	-0.23044833	-0.228253584	-0.226058838
15.0286	-0.226058838	-0.226058838	-0.223864092	-0.223864092	-0.221669346	-0.219474599
15.1292	-0.219474599	-0.219474599	-0.217279853	-0.217279853	-0.215085109	-0.215085109
15.235	-0.212890362	-0.212890362	-0.212890362	-0.210695616	-0.20850087	-0.20850087
15.3355	-0.20850087	-0.206306124	-0.206306124	-0.204111378	-0.204111378	-0.201916632
15.4361	-0.201916632	-0.201916632	-0.199721885	-0.199721885	-0.197527139	-0.197527139
15.5419	-0.195332395	-0.195332395	-0.195332395	-0.193137648	-0.193137648	-0.190942902
15.6425	-0.190942902	-0.190942902	-0.188748156	-0.188748156	-0.18655341	-0.18655341
15.7483	-0.18655341	-0.184358664	-0.184358664	-0.182163917	-0.182163917	-0.179969171
15.8489	-0.179969171	-0.179969171	-0.179969171	-0.177774427	-0.177774427	-0.17557968
15.9494	-0.17557968	-0.17557968	-0.173384934	-0.173384934	-0.171190188	-0.171190188
16.0552	-0.171190188	-0.168995442	-0.168995442	-0.168995442	-0.166800696	-0.166800696
16.1558	-0.166800696	-0.16460595	-0.16460595	-0.16460595	-0.162411203	-0.162411203
16.2563	-0.162411203	-0.160216457	-0.160216457	-0.160216457	-0.158021713	-0.158021713
16.3622	-0.158021713	-0.155826966	-0.155826966	-0.155826966	-0.15363222	-0.15363222
16.4627	-0.15363222	-0.151437474	-0.151437474	-0.151437474	-0.149242728	-0.149242728
16.5685	-0.149242728	-0.149242728	-0.147047982	-0.147047982	-0.147047982	-0.144853236
16.6691	-0.144853236	-0.144853236	-0.142658489	-0.142658489	-0.142658489	-0.140463745
16.7696	-0.140463745	-0.140463745	-0.140463745	-0.138268999	-0.138268999	-0.138268999
16.8755	-0.138268999	-0.136074252	-0.136074252	-0.136074252	-0.133879506	-0.133879506
16.976	-0.133879506	-0.133879506	-0.13168476	-0.13168476	-0.13168476	-0.129490014
17.0818	-0.129490014	-0.129490014	-0.129490014	-0.129490014	-0.127295268	-0.127295268
17.1824	-0.127295268	-0.127295268	-0.125100521	-0.125100521	-0.125100521	-0.122905775
17.2829	-0.122905775	-0.122905775	-0.122905775	-0.122905775	-0.120711031	-0.120711031
17.3888	-0.120711031	-0.120711031	-0.118516284	-0.118516284	-0.118516284	-0.116321538
17.4893	-0.116321538	-0.116321538	-0.116321538	-0.116321538	-0.114126792	-0.114126792
17.5898	-0.114126792	-0.114126792	-0.114126792	-0.111932046	-0.111932046	-0.111932046
17.6957	-0.111932046	-0.1097373	-0.1097373	-0.1097373	-0.1097373	-0.107542554
17.7962	-0.107542554	-0.107542554	-0.107542554	-0.107542554	-0.105347807	-0.105347807
17.9021	-0.105347807	-0.105347807	-0.105347807	-0.105347807	-0.103153063	-0.103153063
18.0026	-0.103153063	-0.103153063	-0.103153063	-0.100958317	-0.100958317	-0.100958317
18.1031	-0.100958317	-0.100958317	-0.09876357	-0.09876357	-0.09876357	-0.09876357
18.209	-0.09876357	-0.096568824	-0.096568824	-0.096568824	-0.096568824	-0.094374078
18.3095	-0.094374078	-0.094374078	-0.094374078	-0.094374078	-0.094374078	-0.092179332
18.4154	-0.092179332	-0.092179332	-0.092179332	-0.092179332	-0.092179332	-0.089984586
18.5159	-0.089984586	-0.089984586	-0.089984586	-0.089984586	-0.089984586	-0.087789839
18.6164	-0.087789839	-0.087789839	-0.087789839	-0.087789839	-0.087789839	-0.085595093
18.7223	-0.085595093	-0.085595093	-0.085595093	-0.085595093	-0.085595093	-0.083400349
18.8228	-0.083400349	-0.083400349	-0.083400349	-0.083400349	-0.083400349	-0.081205602
18.9287	-0.081205602	-0.081205602	-0.081205602	-0.081205602	-0.081205602	-0.079010856
19.0292	-0.079010856	-0.079010856	-0.079010856	-0.079010856	-0.079010856	-0.079010856
19.1297	-0.07681611	-0.07681611	-0.07681611	-0.07681611	-0.07681611	-0.07681611
19.2356	-0.07681611	-0.074621364	-0.074621364	-0.074621364	-0.074621364	-0.074621364
19.3361	-0.074621364	-0.074621364	-0.072426618	-0.072426618	-0.072426618	-0.072426618
19.4367	-0.072426618	-0.072426618	-0.072426618	-0.070231872	-0.070231872	-0.070231872
19.5425	-0.070231872	-0.070231872	-0.070231872	-0.070231872	-0.070231872	-0.068037125
19.6431	-0.068037125	-0.068037125	-0.068037125	-0.068037125	-0.068037125	-0.068037125
19.7489	-0.068037125	-0.065842379	-0.065842379	-0.065842379	-0.065842379	-0.065842379
19.8494	-0.065842379	-0.065842379	-0.065842379	-0.063647635	-0.063647635	-0.063647635
19.95	-0.063647635	-0.063647635	-0.063647635	-0.063647635	-0.063647635	-0.061452888
20.0558	-0.061452888	-0.061452888	-0.061452888	-0.061452888	-0.061452888	-0.061452888
20.1564	-0.061452888	-0.061452888	-0.059258142	-0.059258142	-0.059258142	-0.059258142
20.2622	-0.059258142	-0.059258142	-0.059258142	-0.059258142	-0.059258142	-0.057063396
20.3627	-0.057063396	-0.057063396	-0.057063396	-0.057063396	-0.057063396	-0.057063396
20.4633	-0.057063396	-0.057063396	-0.05486865	-0.05486865	-0.05486865	-0.05486865
20.5691	-0.05486865	-0.05486865	-0.05486865	-0.05486865	-0.05486865	-0.052673904
20.6697	-0.052673904	-0.052673904	-0.052673904	-0.052673904	-0.052673904	-0.052673904
20.7755	-0.052673904	-0.052673904	-0.052673904	-0.052673904	-0.050479157	-0.050479157

R(Å) \ θ (º)	110	100	90	80	70	60
20.876	-0.050479157	-0.050479157	-0.050479157	-0.050479157	-0.050479157	-0.050479157
20.9766	-0.050479157	-0.050479157	-0.050479157	-0.048284411	-0.048284411	-0.048284411
21.0824	-0.048284411	-0.048284411	-0.048284411	-0.048284411	-0.048284411	-0.048284411
21.183	-0.048284411	-0.048284411	-0.046089667	-0.046089667	-0.046089667	-0.046089667
21.2835	-0.046089667	-0.046089667	-0.046089667	-0.046089667	-0.046089667	-0.046089667
21.3893	-0.046089667	-0.043894921	-0.043894921	-0.043894921	-0.043894921	-0.043894921
21.4899	-0.043894921	-0.043894921	-0.043894921	-0.043894921	-0.043894921	-0.043894921
21.5957	-0.043894921	-0.043894921	-0.041700174	-0.041700174	-0.041700174	-0.041700174
21.6963	-0.041700174	-0.041700174	-0.041700174	-0.041700174	-0.041700174	-0.041700174
21.7968	-0.041700174	-0.041700174	-0.039505428	-0.039505428	-0.039505428	-0.039505428
21.9026	-0.039505428	-0.039505428	-0.039505428	-0.039505428	-0.039505428	-0.039505428
22.0032	-0.039505428	-0.039505428	-0.039505428	-0.039505428	-0.037310682	-0.037310682
22.109	-0.037310682	-0.037310682	-0.037310682	-0.037310682	-0.037310682	-0.037310682
22.2096	-0.037310682	-0.037310682	-0.037310682	-0.037310682	-0.037310682	-0.035115936
22.3101	-0.035115936	-0.035115936	-0.035115936	-0.035115936	-0.035115936	-0.035115936
22.4159	-0.035115936	-0.035115936	-0.035115936	-0.035115936	-0.035115936	-0.035115936
22.5165	-0.035115936	-0.035115936	-0.035115936	-0.03292119	-0.03292119	-0.03292119
22.617	-0.03292119	-0.03292119	-0.03292119	-0.03292119	-0.03292119	-0.03292119
22.7229	-0.03292119	-0.03292119	-0.03292119	-0.03292119	-0.03292119	-0.03292119
22.8234	-0.03292119	-0.030726443	-0.030726443	-0.030726443	-0.030726443	-0.030726443
22.9292	-0.030726443	-0.030726443	-0.030726443	-0.030726443	-0.030726443	-0.030726443
23.0298	-0.030726443	-0.030726443	-0.030726443	-0.030726443	-0.030726443	-0.028531697
23.1303	-0.028531697	-0.028531697	-0.028531697	-0.028531697	-0.028531697	-0.028531697
23.2362	-0.028531697	-0.028531697	-0.028531697	-0.028531697	-0.028531697	-0.028531697
23.3367	-0.028531697	-0.028531697	-0.028531697	-0.028531697	-0.028531697	-0.026336953
23.4425	-0.026336953	-0.026336953	-0.026336953	-0.026336953	-0.026336953	-0.026336953
23.5431	-0.026336953	-0.026336953	-0.026336953	-0.026336953	-0.026336953	-0.026336953
23.6436	-0.026336953	-0.026336953	-0.026336953	-0.026336953	-0.026336953	-0.026336953
23.7495	-0.026336953	-0.024142206	-0.024142206	-0.024142206	-0.024142206	-0.024142206
23.85	-0.024142206	-0.024142206	-0.024142206	-0.024142206	-0.024142206	-0.024142206
23.9558	-0.024142206	-0.024142206	-0.024142206	-0.024142206	-0.024142206	-0.024142206
24.0564	-0.024142206	-0.024142206	-0.024142206	-0.024142206	-0.02194746	-0.02194746
24.1569	-0.02194746	-0.02194746	-0.02194746	-0.02194746	-0.02194746	-0.02194746
24.2628	-0.02194746	-0.02194746	-0.02194746	-0.02194746	-0.02194746	-0.02194746
24.3633	-0.02194746	-0.02194746	-0.02194746	-0.02194746	-0.02194746	-0.02194746
24.4639	-0.02194746	-0.02194746	-0.019752714	-0.019752714	-0.019752714	-0.019752714
24.5697	-0.019752714	-0.019752714	-0.019752714	-0.019752714	-0.019752714	-0.019752714
24.6702	-0.019752714	-0.019752714	-0.019752714	-0.019752714	-0.019752714	-0.019752714
24.7761	-0.019752714	-0.019752714	-0.019752714	-0.019752714	-0.019752714	-0.019752714
24.8766	-0.019752714	-0.019752714	-0.019752714	-0.017557968	-0.017557968	-0.017557968
24.9772	-0.017557968	-0.017557968	-0.017557968	-0.017557968	-0.017557968	-0.017557968
25.083	-0.017557968	-0.017557968	-0.017557968	-0.017557968	-0.017557968	-0.017557968
25.1835	-0.017557968	-0.017557968	-0.017557968	-0.017557968	-0.017557968	-0.017557968
25.2894	-0.017557968	-0.017557968	-0.017557968	-0.017557968	-0.017557968	-0.015363222
25.3899	-0.015363222	-0.015363222	-0.015363222	-0.015363222	-0.015363222	-0.015363222
25.4905	-0.015363222	-0.015363222	-0.015363222	-0.015363222	-0.015363222	-0.015363222
25.5963	-0.015363222	-0.015363222	-0.015363222	-0.015363222	-0.015363222	-0.015363222
25.6968	-0.015363222	-0.015363222	-0.015363222	-0.015363222	-0.015363222	-0.015363222
25.8027	-0.015363222	-0.015363222	-0.015363222	-0.015363222	-0.013168476	-0.013168476
25.9032	-0.013168476	-0.013168476	-0.013168476	-0.013168476	-0.013168476	-0.013168476
26.0038	-0.013168476	-0.013168476	-0.013168476	-0.013168476	-0.013168476	-0.013168476
26.1096	-0.013168476	-0.013168476	-0.013168476	-0.013168476	-0.013168476	-0.013168476
26.2101	-0.013168476	-0.013168476	-0.013168476	-0.013168476	-0.013168476	-0.013168476
26.3107	-0.013168476	-0.013168476	-0.013168476	-0.013168476	-0.013168476	-0.013168476
26.4165	-0.013168476	-0.010973729	-0.010973729	-0.010973729	-0.010973729	-0.010973729
26.5171	-0.010973729	-0.010973729	-0.010973729	-0.010973729	-0.010973729	-0.010973729
26.6229	-0.010973729	-0.010973729	-0.010973729	-0.010973729	-0.010973729	-0.010973729
26.7234	-0.010973729	-0.010973729	-0.010973729	-0.010973729	-0.010973729	-0.010973729
26.824	-0.010973729	-0.010973729	-0.010973729	-0.010973729	-0.010973729	-0.010973729
26.9298	-0.010973729	-0.010973729	-0.010973729	-0.010973729	-0.010973729	-0.010973729
27.0304	-0.010973729	-0.008778985	-0.008778985	-0.008778985	-0.008778985	-0.008778985
27.1362	-0.008778985	-0.008778985	-0.008778985	-0.008778985	-0.008778985	-0.008778985
27.2367	-0.008778985	-0.008778985	-0.008778985	-0.008778985	-0.008778985	-0.008778985

R(Å) \ θ (º)	110	100	90	80	70	60
27.3373	-0.008778985	-0.008778985	-0.008778985	-0.008778985	-0.008778985	-0.008778985
27.4431	-0.008778985	-0.008778985	-0.008778985	-0.008778985	-0.008778985	-0.008778985
27.5437	-0.008778985	-0.008778985	-0.008778985	-0.008778985	-0.008778985	-0.008778985
27.6442	-0.008778985	-0.008778985	-0.008778985	-0.008778985	-0.008778985	-0.006584239
27.75	-0.006584239	-0.006584239	-0.006584239	-0.006584239	-0.006584239	-0.006584239
27.8506	-0.006584239	-0.006584239	-0.006584239	-0.006584239	-0.006584239	-0.006584239
27.9564	-0.006584239	-0.006584239	-0.006584239	-0.006584239	-0.006584239	-0.006584239
28.057	-0.006584239	-0.006584239	-0.006584239	-0.006584239	-0.006584239	-0.006584239
28.1575	-0.006584239	-0.006584239	-0.006584239	-0.006584239	-0.006584239	-0.006584239
28.2633	-0.006584239	-0.006584239	-0.006584239	-0.006584239	-0.006584239	-0.006584239
28.3639	-0.006584239	-0.006584239	-0.006584239	-0.006584239	-0.006584239	-0.006584239
28.4697	-0.006584239	-0.006584239	-0.006584239	-0.006584239	-0.004389492	-0.004389492
28.5703	-0.004389492	-0.004389492	-0.004389492	-0.004389492	-0.004389492	-0.004389492
28.6708	-0.004389492	-0.004389492	-0.004389492	-0.004389492	-0.004389492	-0.004389492
28.7766	-0.004389492	-0.004389492	-0.004389492	-0.004389492	-0.004389492	-0.004389492
28.8772	-0.004389492	-0.004389492	-0.004389492	-0.004389492	-0.004389492	-0.004389492
28.983	-0.004389492	-0.004389492	-0.004389492	-0.004389492	-0.004389492	-0.004389492
29.0836	-0.004389492	-0.004389492	-0.004389492	-0.004389492	-0.004389492	-0.004389492
29.1841	-0.004389492	-0.004389492	-0.004389492	-0.004389492	-0.004389492	-0.004389492
29.2899	-0.004389492	-0.004389492	-0.004389492	-0.004389492	-0.004389492	-0.004389492
29.3905	-0.004389492	-0.004389492	-0.004389492	-0.004389492	-0.004389492	-0.002194746
29.491	-0.002194746	-0.002194746	-0.002194746	-0.002194746	-0.002194746	-0.002194746
29.5969	-0.002194746	-0.002194746	-0.002194746	-0.002194746	-0.002194746	-0.002194746
29.6974	-0.002194746	-0.002194746	-0.002194746	-0.002194746	-0.002194746	-0.002194746
29.8032	-0.002194746	-0.002194746	-0.002194746	-0.002194746	-0.002194746	-0.002194746
29.9038	-0.002194746	-0.002194746	-0.002194746	-0.002194746	-0.002194746	-0.002194746
30.0043	-0.002194746	-0.002194746	-0.002194746	-0.002194746	-0.002194746	-0.002194746
30.1102	-0.002194746	-0.002194746	-0.002194746	-0.002194746	-0.002194746	-0.002194746
30.2107	-0.002194746	-0.002194746	-0.002194746	-0.002194746	-0.002194746	-0.002194746
30.3166	-0.002194746	-0.002194746	-0.002194746	-0.002194746	-0.002194746	-0.002194746
30.4171	-0.002194746	-0.002194746	-0.002194746	-0.002194746	-0.002194746	-0.002194746
30.5176	-0.002194746	-0.002194746	-0.002194746	-0.002194746	0.000000000	0.000000000
30.6235	0.0000000	0.0000000	0.0000000	0.0000000	0.0000000	0.0000000
30.724	0.0000000	0.0000000	0.0000000	0.0000000	0.0000000	0.0000000
30.8299	0.0000000	0.0000000	0.0000000	0.0000000	0.0000000	0.0000000
30.9304	0.0000000	0.0000000	0.0000000	0.0000000	0.0000000	0.0000000
31.0309	0.0000000	0.0000000	0.0000000	0.0000000	0.0000000	0.0000000
31.1368	0.0000000	0.0000000	0.0000000	0.0000000	0.0000000	0.0000000
31.2373	0.0000000	0.0000000	0.0000000	0.0000000	0.0000000	0.0000000
31.3379	0.0000000	0.0000000	0.0000000	0.0000000	0.0000000	0.0000000
31.4437	0.0000000	0.0000000	0.0000000	0.0000000	0.0000000	0.0000000
31.5442	-0.006584239	-0.006584239	-0.006584239	0.000000000	0.000000000	0.000000000
31.6501	-0.048284411	-0.048284411	-0.048284411	0.000000000	0.000000000	0.000000000
31.7506	-0.316043424	-0.316043424	-0.316043424	0.000000000	0.000000000	0.000000000

CH^- + He PES
Method: MRCI. AV5Z (0º – 50º)

R(Å) \ θ (º)	50	40	30	20	10	0
1.58753	13163.46978	23418.00127	30337.5789	40294.84083	54240.6388	62950.76483
1.68807	13298.96023	16997.28502	22180.86904	28968.85629	36814.33141	41038.55026
1.79391	9464.587534	12187.13044	16016.047	20759.60421	25570.9637	27881.31136
1.89445	6665.237296	8638.83191	11428.67451	14781.19072	17923.59732	19319.73451
2.00029	4643.669924	6053.741555	8060.779708	10434.33605	12561.4751	13462.7234
2.10083	3196.180068	4190.430733	5617.77282	7291.409303	8752.554869	9356.353635
2.20138	2168.17421	2860.952369	3865.377876	5036.882812	6042.622972	6455.248388
2.30721	1444.51378	1921.802998	2621.141253	3433.928123	4123.732402	4407.59207
2.40776	939.921922	1264.78603	1745.979701	2304.393315	2774.367445	2968.203022
2.5083	591.4313731	809.4793882	1136.250731	1515.717925	1832.430746	1962.078782
2.61413	353.0643995	497.0616846	715.9656506	970.6330027	1180.573626	1264.606061
2.71468	191.8010477	285.1282318	429.8870938	598.3865232	734.6297706	787.5143702
2.82051	84.21679351	143.4068984	237.9806983	347.7750617	434.1097851	466.7346844
2.92106	13.81592607	50.40673138	111.4075017	181.8939642	235.2197078	254.8999952
3.0216	-30.98103454	-9.051132504	29.61809727	74.29654159	106.3508069	117.7239807
3.12744	-58.26831155	-45.63974307	-21.79821727	6.211131179	25.11228373	31.29707796
3.22798	-73.69957068	-66.81684722	-52.79461503	-35.50001655	-24.20146414	-20.90495565
3.33382	-81.24510743	-77.80155095	-70.15725064	-59.83316545	-52.62561959	-50.617427
3.43436	-83.56056446	-82.09666888	-78.49728544	-72.79314058	-67.7891197	-66.18476038
3.5349	-82.56195503	-82.19543245	-80.99051689	-78.33706898	-74.73988028	-73.22989504
3.64074	-79.52662131	-79.74609591	-79.78340659	-79.12498279	-76.65150405	-75.27100882
3.74128	-75.32148797	-75.84164278	-76.39910826	-76.82927848	-75.48170443	-74.38872092
3.84712	-70.51718898	-71.18439176	-71.88451574	-72.76460888	-72.29054375	-71.57944604
3.94766	-65.49122064	-66.22207106	-66.89805283	-67.81545665	-67.90983073	-67.55867137
4.0482	-60.48719976	-61.22463442	-61.8347738	-62.58318219	-62.92336782	-62.81363052
4.15404	-55.65436907	-56.35888253	-56.90976378	-57.43430807	-57.7788832	-57.77668845
4.25458	-51.0849079	-51.72796847	-52.22837056	-52.56197195	-52.79680978	-52.79022554
4.35513	-46.82710066	-47.38456614	-47.83887856	-48.04737943	-48.14833775	-48.09127435
4.46096	-42.87216836	-43.28039112	-43.61179777	-43.68202964	-43.63813472	-43.7851827
4.56151	-39.28375865	-39.59760733	-39.87195058	-39.9158455	-39.68759192	-39.64150225
4.66734	-36.01139237	-36.23745121	-36.44156258	-36.47228903	-36.213309	-36.1606351
4.76788	-33.03531679	-33.18017003	-33.30966004	-33.32282852	-33.05506951	-33.00678509
4.86843	-30.33358447	-30.41040058	-30.46307448	-30.45210075	-30.17556275	-30.13825207
4.97426	-27.87766369	-27.9018059	-27.88644268	-27.83815826	-27.55064654	-27.51992009
5.07481	-25.64560701	-25.63024379	-25.55342768	-25.23080002	-25.15617865	-25.1298417
5.18064	-23.61546696	-23.56718255	-23.44647152	-23.06458571	-22.97899062	-22.95265367
5.28119	-21.76310134	-21.69286946	-21.27367298	-21.10906703	-21.00591397	-20.97299278
5.48757	-18.44245064	-18.0495911	-17.86742719	-17.66551055	-17.5294363	-17.48334664
5.58811	-16.7086013	-16.61203247	-16.42986856	-16.22575718	-16.08090394	-16.03042478
5.69394	-15.39614319	-15.29737962	-15.11960519	-14.91768856	-14.76844583	-14.71577193
5.79449	-14.19561713	-14.09465881	-13.92127388	-13.72594148	-13.58108825	-13.52841434
5.89503	-13.09385464	-12.99289632	-12.82829037	-12.64173696	-12.50127322	-12.45079406
6.00087	-12.08427148	-11.98550791	-11.82748619	-11.65410126	-11.52022175	-11.47193734
6.10141	-11.16247816	-11.06151984	-10.91447186	-10.75206065	-10.62696013	-10.58087047
6.20195	-10.3153062	-10.21873738	-10.07827363	-9.929030904	-9.812709365	-9.771009193
6.30779	-9.540560862	-9.446186784	-9.31669677	-9.176233026	-9.070885218	-9.03137979
6.40833	-8.831657903	-8.741673318	-8.618767542	-8.491472273	-8.392708704	-8.357592769
6.51417	-8.184207834	-8.094223249	-7.982291201	-7.863774918	-7.775985077	-7.743063888
6.61471	-7.589431667	-7.503836574	-7.398488765	-7.290946212	-7.211935355	-7.181208912
6.71526	-7.04513466	-6.966123803	-6.865165488	-6.768596664	-6.696170046	-6.667638348
6.82109	-6.546927319	-6.470111208	-6.377931877	-6.290142036	-6.224299656	-6.20015745
6.92164	-6.09042015	-6.015798786	-5.932398438	-5.851192837	-5.791934694	-5.769987234
7.02747	-5.671223664	-5.603186538	-5.524175682	-5.451749064	-5.396880415	-5.3771277
7.12801	-5.287143113	-5.22349548	-5.151068862	-5.085226482	-5.03694207	-5.017189356
7.22856	-4.935983754	-4.874530865	-4.808688486	-4.749430344	-4.705535423	-4.687977456
7.33439	-4.613356092	-4.556292696	-4.494839807	-4.439971157	-4.400465731	-4.387297254
7.43494	-4.319260128	-4.264391478	-4.207328082	-4.159043669	-4.121732988	-4.110759258
7.53548	-4.047111624	-3.996632466	-3.943958562	-3.897868896	-3.867142452	-3.856168721
7.64132	-3.796910581	-3.748626168	-3.702536503	-3.660836328	-3.63230463	-3.6213309
7.74186	-3.56646225	-3.52256733	-3.478672409	-3.441361727	-3.415024776	-3.406245792
7.84769	-3.355766634	-3.31406646	-3.274561032	-3.239445096	-3.215302889	-3.206523906

R(Å) \ θ (º)	50	40	30	20	10	0
7.94824	-3.16043424	-3.123123558	-3.085812876	-3.052891686	-3.030944226	-3.024359989
8.04878	-2.980465068	-2.945349132	-2.910233197	-2.881701498	-2.861948784	-2.855364545
8.15462	-2.813664372	-2.780743181	-2.750016738	-2.723679785	-2.703927073	-2.69953758
8.25516	-2.660032152	-2.629305707	-2.598579264	-2.574437057	-2.559073836	-2.552489599
8.361	-2.517373661	-2.488841964	-2.460310265	-2.438362807	-2.422999583	-2.418610093
8.46154	-2.385688901	-2.357157204	-2.333014998	-2.311067537	-2.297899062	-2.29350957
8.56208	-2.262783126	-2.236446173	-2.212303969	-2.194746001	-2.181577524	-2.177188031
8.66792	-2.148656334	-2.124514128	-2.102566667	-2.082813953	-2.071840224	-2.067450732
8.76846	-2.04111378	-2.01916632	-1.99721886	-1.981855638	-1.970881907	-1.966492417
8.8743	-1.94235021	-1.92040275	-1.900650036	-1.885286814	-1.874313085	-1.872118339
8.97484	-1.847976132	-1.828223418	-1.81066545	-1.795302228	-1.786523244	-1.782133751
9.07539	-1.762381037	-1.742628325	-1.725070355	-1.71190188	-1.703122897	-1.698733404
9.18122	-1.67898069	-1.661422722	-1.646059501	-1.632891023	-1.62411204	-1.621917294
9.28176	-1.60216458	-1.584606612	-1.56924339	-1.558269659	-1.551685423	-1.54729593
9.38231	-1.529737962	-1.514374741	-1.499011517	-1.488037788	-1.481453549	-1.479258803
9.48814	-1.463895581	-1.446337614	-1.433169138	-1.422195409	-1.41561117	-1.413416424
9.58869	-1.398053202	-1.384884727	-1.37171625	-1.36074252	-1.354158282	-1.351963536
9.69452	-1.33879506	-1.323431838	-1.312458107	-1.301484378	-1.297094886	-1.29490014
9.79507	-1.281731664	-1.268563188	-1.255394711	-1.246615728	-1.242226236	-1.24003149
9.89561	-1.226863014	-1.213694539	-1.202720808	-1.196136571	-1.189552332	-1.187357586
10.0014	-1.176383857	-1.16321538	-1.154436396	-1.145657412	-1.141267919	-1.139073175
10.102	-1.128099444	-1.117125714	-1.106151984	-1.099567747	-1.092983508	-1.092983508
10.2078	-1.082009779	-1.071036048	-1.062257065	-1.05347808	-1.049088587	-1.049088587
10.3084	-1.038114858	-1.027141127	-1.018362144	-1.011777906	-1.007388415	-1.005193669
10.4089	-0.996414684	-0.987635701	-0.978856716	-0.972272477	-0.967882987	-0.96568824
10.5147	-0.956909256	-0.948130272	-0.939351288	-0.934961795	-0.930572305	-0.928377558
10.6153	-0.919598574	-0.910819591	-0.904235352	-0.897651113	-0.893261623	-0.893261623
10.7211	-0.884482638	-0.875703655	-0.869119416	-0.862535178	-0.860340431	-0.858145685
10.8217	-0.851561448	-0.842782464	-0.836198227	-0.829613988	-0.827419242	-0.825224496
10.9222	-0.818640259	-0.81205602	-0.805471782	-0.798887545	-0.796692798	-0.794498052
11.028	-0.787913814	-0.781329577	-0.774745338	-0.770355846	-0.765966353	-0.765966353
11.1286	-0.759382116	-0.752797878	-0.746213639	-0.741824149	-0.737434656	-0.737434656
11.2291	-0.730850418	-0.724266181	-0.717681942	-0.71329245	-0.711097704	-0.711097704
11.335	-0.704513467	-0.697929228	-0.693539736	-0.689150243	-0.684760753	-0.684760753
11.4355	-0.678176514	-0.673787022	-0.667202785	-0.662813292	-0.660618546	-0.660618546
11.5414	-0.654034308	-0.649644817	-0.643060578	-0.638671086	-0.63647634	-0.63647634
11.6419	-0.632086847	-0.62550261	-0.621113118	-0.616723626	-0.614528879	-0.614528879
11.7424	-0.607944642	-0.60355515	-0.599165658	-0.594776165	-0.592581421	-0.592581421
11.8483	-0.588191928	-0.58160769	-0.577218197	-0.575023451	-0.572828707	-0.572828707
11.9488	-0.566244468	-0.561854976	-0.557465483	-0.555270739	-0.553075993	-0.553075993
12.0547	-0.5486865	-0.542102262	-0.539907515	-0.535518025	-0.533323278	-0.533323278
12.1552	-0.528933786	-0.524544294	-0.520154801	-0.517960055	-0.515765311	-0.515765311
12.2557	-0.511375818	-0.506986326	-0.502596833	-0.500402087	-0.498207343	-0.498207343
12.3616	-0.49381785	-0.489428358	-0.487233612	-0.482844119	-0.482844119	-0.480649373
12.4621	-0.476259882	-0.474065136	-0.469675644	-0.467480898	-0.465286152	-0.465286152
12.5627	-0.460896661	-0.458701915	-0.454312422	-0.452117676	-0.44992293	-0.44992293
12.6685	-0.445533437	-0.443338691	-0.4389492	-0.436754454	-0.436754454	-0.434559708
12.769	-0.432364962	-0.42797547	-0.425780723	-0.423585979	-0.421391233	-0.421391233
12.8749	-0.41700174	-0.414806994	-0.412612248	-0.410417502	-0.408222755	-0.408222755
12.9754	-0.403833265	-0.401638518	-0.397249026	-0.397249026	-0.39505428	-0.39505428
13.076	-0.390664788	-0.388470041	-0.386275295	-0.384080551	-0.381885804	-0.381885804
13.1818	-0.379691058	-0.375301566	-0.37310682	-0.370912074	-0.370912074	-0.370912074
13.2823	-0.366522583	-0.364327837	-0.36213309	-0.359938344	-0.357743598	-0.357743598
13.3882	-0.355548852	-0.353354106	-0.351159359	-0.348964613	-0.346769869	-0.346769869
13.4887	-0.344575122	-0.342380376	-0.34018563	-0.337990884	-0.335796138	-0.335796138
13.5893	-0.333601392	-0.331406645	-0.329211901	-0.327017155	-0.327017155	-0.327017155
13.6951	-0.322627662	-0.320432916	-0.31823817	-0.31823817	-0.316043424	-0.316043424
13.7956	-0.313848677	-0.311653931	-0.309459187	-0.30726444	-0.30726444	-0.30726444
13.9015	-0.302874948	-0.300680202	-0.300680202	-0.298485456	-0.298485456	-0.29629071
14.002	-0.294095963	-0.291901217	-0.289706473	-0.289706473	-0.287511726	-0.287511726
14.1026	-0.28531698	-0.283122234	-0.283122234	-0.280927488	-0.280927488	-0.278732742
14.2084	-0.276537995	-0.274343249	-0.274343249	-0.272148505	-0.272148505	-0.272148505
14.3089	-0.269953759	-0.267759012	-0.265564266	-0.26336952	-0.26336952	-0.26336952

R(Å) \ θ (º)	50	40	30	20	10	0
14.4095	-0.261174774	-0.258980028	-0.256785281	-0.256785281	-0.256785281	-0.254590535
14.5153	-0.252395791	-0.252395791	-0.250201044	-0.250201044	-0.248006298	-0.248006298
14.6159	-0.245811552	-0.243616806	-0.243616806	-0.24142206	-0.24142206	-0.24142206
14.7217	-0.239227314	-0.237032567	-0.237032567	-0.234837823	-0.234837823	-0.234837823
14.8222	-0.232643077	-0.23044833	-0.228253584	-0.228253584	-0.228253584	-0.228253584
14.9228	-0.226058838	-0.223864092	-0.221669346	-0.221669346	-0.221669346	-0.221669346
15.0286	-0.219474599	-0.217279853	-0.217279853	-0.215085109	-0.215085109	-0.215085109
15.1292	-0.212890362	-0.210695616	-0.210695616	-0.20850087	-0.20850087	-0.20850087
15.235	-0.206306124	-0.206306124	-0.204111378	-0.204111378	-0.201916632	-0.201916632
15.3355	-0.201916632	-0.199721885	-0.197527139	-0.197527139	-0.197527139	-0.197527139
15.4361	-0.195332395	-0.193137648	-0.193137648	-0.193137648	-0.190942902	-0.190942902
15.5419	-0.190942902	-0.188748156	-0.18655341	-0.18655341	-0.18655341	-0.18655341
15.6425	-0.184358664	-0.184358664	-0.182163917	-0.182163917	-0.182163917	-0.179969171
15.7483	-0.179969171	-0.177774427	-0.177774427	-0.177774427	-0.17557968	-0.17557968
15.8489	-0.17557968	-0.173384934	-0.173384934	-0.171190188	-0.171190188	-0.171190188
15.9494	-0.168995442	-0.168995442	-0.166800696	-0.166800696	-0.166800696	-0.166800696
16.0552	-0.16460595	-0.16460595	-0.162411203	-0.162411203	-0.162411203	-0.162411203
16.1558	-0.160216457	-0.160216457	-0.158021713	-0.158021713	-0.158021713	-0.158021713
16.2563	-0.155826966	-0.155826966	-0.15363222	-0.15363222	-0.15363222	-0.15363222
16.3622	-0.151437474	-0.151437474	-0.151437474	-0.149242728	-0.149242728	-0.149242728
16.4627	-0.147047982	-0.147047982	-0.147047982	-0.144853236	-0.144853236	-0.144853236
16.5685	-0.144853236	-0.142658489	-0.142658489	-0.142658489	-0.142658489	-0.140463745
16.6691	-0.140463745	-0.140463745	-0.138268999	-0.138268999	-0.138268999	-0.138268999
16.7696	-0.136074252	-0.136074252	-0.136074252	-0.133879506	-0.133879506	-0.133879506
16.8755	-0.133879506	-0.13168476	-0.13168476	-0.13168476	-0.13168476	-0.13168476
16.976	-0.129490014	-0.129490014	-0.127295268	-0.127295268	-0.127295268	-0.127295268
17.0818	-0.125100521	-0.125100521	-0.125100521	-0.125100521	-0.125100521	-0.122905775
17.1824	-0.122905775	-0.122905775	-0.120711031	-0.120711031	-0.120711031	-0.120711031
17.2829	-0.120711031	-0.118516284	-0.118516284	-0.118516284	-0.118516284	-0.118516284
17.3888	-0.116321538	-0.116321538	-0.116321538	-0.114126792	-0.114126792	-0.114126792
17.4893	-0.114126792	-0.111932046	-0.111932046	-0.111932046	-0.111932046	-0.111932046
17.5898	-0.1097373	-0.1097373	-0.1097373	-0.1097373	-0.1097373	-0.1097373
17.6957	-0.107542554	-0.107542554	-0.107542554	-0.105347807	-0.105347807	-0.105347807
17.7962	-0.105347807	-0.105347807	-0.103153063	-0.103153063	-0.103153063	-0.103153063
17.9021	-0.103153063	-0.100958317	-0.100958317	-0.100958317	-0.100958317	-0.100958317
18.0026	-0.09876357	-0.09876357	-0.09876357	-0.09876357	-0.09876357	-0.09876357
18.1031	-0.096568824	-0.096568824	-0.096568824	-0.096568824	-0.096568824	-0.096568824
18.209	-0.094374078	-0.094374078	-0.094374078	-0.094374078	-0.094374078	-0.092179332
18.3095	-0.092179332	-0.092179332	-0.092179332	-0.092179332	-0.089984586	-0.089984586
18.4154	-0.089984586	-0.089984586	-0.089984586	-0.089984586	-0.087789839	-0.087789839
18.5159	-0.087789839	-0.087789839	-0.087789839	-0.087789839	-0.085595093	-0.085595093
18.6164	-0.085595093	-0.085595093	-0.085595093	-0.085595093	-0.083400349	-0.083400349
18.7223	-0.083400349	-0.083400349	-0.083400349	-0.083400349	-0.081205602	-0.081205602
18.8228	-0.081205602	-0.081205602	-0.081205602	-0.081205602	-0.081205602	-0.081205602
18.9287	-0.079010856	-0.079010856	-0.079010856	-0.079010856	-0.079010856	-0.079010856
19.0292	-0.07681611	-0.07681611	-0.07681611	-0.07681611	-0.07681611	-0.07681611
19.1297	-0.074621364	-0.074621364	-0.074621364	-0.074621364	-0.074621364	-0.074621364
19.2356	-0.074621364	-0.072426618	-0.072426618	-0.072426618	-0.072426618	-0.072426618
19.3361	-0.072426618	-0.072426618	-0.070231872	-0.070231872	-0.070231872	-0.070231872
19.4367	-0.070231872	-0.070231872	-0.070231872	-0.070231872	-0.068037125	-0.068037125
19.5425	-0.068037125	-0.068037125	-0.068037125	-0.068037125	-0.068037125	-0.068037125
19.6431	-0.065842379	-0.065842379	-0.065842379	-0.065842379	-0.065842379	-0.065842379
19.7489	-0.065842379	-0.065842379	-0.063647635	-0.063647635	-0.063647635	-0.063647635
19.8494	-0.063647635	-0.063647635	-0.063647635	-0.063647635	-0.061452888	-0.061452888
19.95	-0.061452888	-0.061452888	-0.061452888	-0.061452888	-0.061452888	-0.061452888
20.0558	-0.061452888	-0.059258142	-0.059258142	-0.059258142	-0.059258142	-0.059258142
20.1564	-0.059258142	-0.059258142	-0.059258142	-0.059258142	-0.057063396	-0.057063396
20.2622	-0.057063396	-0.057063396	-0.057063396	-0.057063396	-0.057063396	-0.057063396
20.3627	-0.057063396	-0.05486865	-0.05486865	-0.05486865	-0.05486865	-0.05486865
20.4633	-0.05486865	-0.05486865	-0.05486865	-0.05486865	-0.05486865	-0.05486865
20.5691	-0.052673904	-0.052673904	-0.052673904	-0.052673904	-0.052673904	-0.052673904
20.6697	-0.052673904	-0.052673904	-0.052673904	-0.050479157	-0.050479157	-0.050479157
20.7755	-0.050479157	-0.050479157	-0.050479157	-0.050479157	-0.050479157	-0.050479157

R(Å) \ θ (º)	50	40	30	20	10	0
20.876	-0.050479157	-0.048284411	-0.048284411	-0.048284411	-0.048284411	-0.048284411
20.9766	-0.048284411	-0.048284411	-0.048284411	-0.048284411	-0.048284411	-0.048284411
21.0824	-0.048284411	-0.046089667	-0.046089667	-0.046089667	-0.046089667	-0.046089667
21.183	-0.046089667	-0.046089667	-0.046089667	-0.046089667	-0.046089667	-0.046089667
21.2835	-0.043894921	-0.043894921	-0.043894921	-0.043894921	-0.043894921	-0.043894921
21.3893	-0.043894921	-0.043894921	-0.043894921	-0.043894921	-0.043894921	-0.043894921
21.4899	-0.041700174	-0.041700174	-0.041700174	-0.041700174	-0.041700174	-0.041700174
21.5957	-0.041700174	-0.041700174	-0.041700174	-0.041700174	-0.041700174	-0.041700174
21.6963	-0.041700174	-0.039505428	-0.039505428	-0.039505428	-0.039505428	-0.039505428
21.7968	-0.039505428	-0.039505428	-0.039505428	-0.039505428	-0.039505428	-0.039505428
21.9026	-0.039505428	-0.039505428	-0.037310682	-0.037310682	-0.037310682	-0.037310682
22.0032	-0.037310682	-0.037310682	-0.037310682	-0.037310682	-0.037310682	-0.037310682
22.109	-0.037310682	-0.037310682	-0.037310682	-0.037310682	-0.037310682	-0.037310682
22.2096	-0.035115936	-0.035115936	-0.035115936	-0.035115936	-0.035115936	-0.035115936
22.3101	-0.035115936	-0.035115936	-0.035115936	-0.035115936	-0.035115936	-0.035115936
22.4159	-0.035115936	-0.035115936	-0.03292119	-0.03292119	-0.03292119	-0.03292119
22.5165	-0.03292119	-0.03292119	-0.03292119	-0.03292119	-0.03292119	-0.03292119
22.617	-0.03292119	-0.03292119	-0.03292119	-0.03292119	-0.03292119	-0.03292119
22.7229	-0.030726443	-0.030726443	-0.030726443	-0.030726443	-0.030726443	-0.030726443
22.8234	-0.030726443	-0.030726443	-0.030726443	-0.030726443	-0.030726443	-0.030726443
22.9292	-0.030726443	-0.030726443	-0.030726443	-0.030726443	-0.030726443	-0.030726443
23.0298	-0.028531697	-0.028531697	-0.028531697	-0.028531697	-0.028531697	-0.028531697
23.1303	-0.028531697	-0.028531697	-0.028531697	-0.028531697	-0.028531697	-0.028531697
23.2362	-0.028531697	-0.028531697	-0.028531697	-0.028531697	-0.028531697	-0.028531697
23.3367	-0.026336953	-0.026336953	-0.026336953	-0.026336953	-0.026336953	-0.026336953
23.4425	-0.026336953	-0.026336953	-0.026336953	-0.026336953	-0.026336953	-0.026336953
23.5431	-0.026336953	-0.026336953	-0.026336953	-0.026336953	-0.026336953	-0.026336953
23.6436	-0.024142206	-0.024142206	-0.024142206	-0.024142206	-0.024142206	-0.024142206
23.7495	-0.024142206	-0.024142206	-0.024142206	-0.024142206	-0.024142206	-0.024142206
23.85	-0.024142206	-0.024142206	-0.024142206	-0.024142206	-0.024142206	-0.024142206
23.9558	-0.024142206	-0.024142206	-0.024142206	-0.02194746	-0.02194746	-0.02194746
24.0564	-0.02194746	-0.02194746	-0.02194746	-0.02194746	-0.02194746	-0.02194746
24.1569	-0.02194746	-0.02194746	-0.02194746	-0.02194746	-0.02194746	-0.02194746
24.2628	-0.02194746	-0.02194746	-0.02194746	-0.02194746	-0.02194746	-0.02194746
24.3633	-0.02194746	-0.019752714	-0.019752714	-0.019752714	-0.019752714	-0.019752714
24.4639	-0.019752714	-0.019752714	-0.019752714	-0.019752714	-0.019752714	-0.019752714
24.5697	-0.019752714	-0.019752714	-0.019752714	-0.019752714	-0.019752714	-0.019752714
24.6702	-0.019752714	-0.019752714	-0.019752714	-0.019752714	-0.019752714	-0.019752714
24.7761	-0.019752714	-0.019752714	-0.017557968	-0.017557968	-0.017557968	-0.017557968
24.8766	-0.017557968	-0.017557968	-0.017557968	-0.017557968	-0.017557968	-0.017557968
24.9772	-0.017557968	-0.017557968	-0.017557968	-0.017557968	-0.017557968	-0.017557968
25.083	-0.017557968	-0.017557968	-0.017557968	-0.017557968	-0.017557968	-0.017557968
25.1835	-0.017557968	-0.017557968	-0.017557968	-0.017557968	-0.017557968	-0.017557968
25.2894	-0.015363222	-0.015363222	-0.015363222	-0.015363222	-0.015363222	-0.015363222
25.3899	-0.015363222	-0.015363222	-0.015363222	-0.015363222	-0.015363222	-0.015363222
25.4905	-0.015363222	-0.015363222	-0.015363222	-0.015363222	-0.015363222	-0.015363222
25.5963	-0.015363222	-0.015363222	-0.015363222	-0.015363222	-0.015363222	-0.015363222
25.6968	-0.015363222	-0.015363222	-0.015363222	-0.015363222	-0.013168476	-0.013168476
25.8027	-0.013168476	-0.013168476	-0.013168476	-0.013168476	-0.013168476	-0.013168476
25.9032	-0.013168476	-0.013168476	-0.013168476	-0.013168476	-0.013168476	-0.013168476
26.0038	-0.013168476	-0.013168476	-0.013168476	-0.013168476	-0.013168476	-0.013168476
26.1096	-0.013168476	-0.013168476	-0.013168476	-0.013168476	-0.013168476	-0.013168476
26.2101	-0.013168476	-0.013168476	-0.013168476	-0.013168476	-0.013168476	-0.013168476
26.3107	-0.010973729	-0.010973729	-0.010973729	-0.010973729	-0.010973729	-0.010973729
26.4165	-0.010973729	-0.010973729	-0.010973729	-0.010973729	-0.010973729	-0.010973729
26.5171	-0.010973729	-0.010973729	-0.010973729	-0.010973729	-0.010973729	-0.010973729
26.6229	-0.010973729	-0.010973729	-0.010973729	-0.010973729	-0.010973729	-0.010973729
26.7234	-0.010973729	-0.010973729	-0.010973729	-0.010973729	-0.010973729	-0.010973729
26.824	-0.010973729	-0.010973729	-0.010973729	-0.010973729	-0.010973729	-0.010973729
26.9298	-0.008778985	-0.008778985	-0.008778985	-0.008778985	-0.008778985	-0.008778985
27.0304	-0.008778985	-0.008778985	-0.008778985	-0.008778985	-0.008778985	-0.008778985
27.1362	-0.008778985	-0.008778985	-0.008778985	-0.008778985	-0.008778985	-0.008778985
27.2367	-0.008778985	-0.008778985	-0.008778985	-0.008778985	-0.008778985	-0.008778985

R(Å) \ θ (º)	50	40	30	20	10	0
27.3373	-0.008778985	-0.008778985	-0.008778985	-0.008778985	-0.008778985	-0.008778985
27.4431	-0.008778985	-0.008778985	-0.008778985	-0.008778985	-0.008778985	-0.008778985
27.5437	-0.008778985	-0.008778985	-0.008778985	-0.008778985	-0.008778985	-0.008778985
27.6442	-0.006584239	-0.006584239	-0.006584239	-0.006584239	-0.006584239	-0.006584239
27.75	-0.006584239	-0.006584239	-0.006584239	-0.006584239	-0.006584239	-0.006584239
27.8506	-0.006584239	-0.006584239	-0.006584239	-0.006584239	-0.006584239	-0.006584239
27.9564	-0.006584239	-0.006584239	-0.006584239	-0.006584239	-0.006584239	-0.006584239
28.057	-0.006584239	-0.006584239	-0.006584239	-0.006584239	-0.006584239	-0.006584239
28.1575	-0.006584239	-0.006584239	-0.006584239	-0.006584239	-0.006584239	-0.006584239
28.2633	-0.006584239	-0.006584239	-0.006584239	-0.006584239	-0.006584239	-0.006584239
28.3639	-0.006584239	-0.006584239	-0.006584239	-0.006584239	-0.006584239	-0.004389492
28.4697	-0.004389492	-0.004389492	-0.004389492	-0.004389492	-0.004389492	-0.004389492
28.5703	-0.004389492	-0.004389492	-0.004389492	-0.004389492	-0.004389492	-0.004389492
28.6708	-0.004389492	-0.004389492	-0.004389492	-0.004389492	-0.004389492	-0.004389492
28.7766	-0.004389492	-0.004389492	-0.004389492	-0.004389492	-0.004389492	-0.004389492
28.8772	-0.004389492	-0.004389492	-0.004389492	-0.004389492	-0.004389492	-0.004389492
28.983	-0.004389492	-0.004389492	-0.004389492	-0.004389492	-0.004389492	-0.004389492
29.0836	-0.004389492	-0.004389492	-0.004389492	-0.004389492	-0.004389492	-0.004389492
29.1841	-0.004389492	-0.004389492	-0.004389492	-0.004389492	-0.004389492	-0.004389492
29.2899	-0.004389492	-0.004389492	-0.004389492	-0.004389492	-0.004389492	-0.004389492
29.3905	-0.002194746	-0.002194746	-0.002194746	-0.002194746	-0.002194746	-0.002194746
29.491	-0.002194746	-0.002194746	-0.002194746	-0.002194746	-0.002194746	-0.002194746
29.5969	-0.002194746	-0.002194746	-0.002194746	-0.002194746	-0.002194746	-0.002194746
29.6974	-0.002194746	-0.002194746	-0.002194746	-0.002194746	-0.002194746	-0.002194746
29.8032	-0.002194746	-0.002194746	-0.002194746	-0.002194746	-0.002194746	-0.002194746
29.9038	-0.002194746	-0.002194746	-0.002194746	-0.002194746	-0.002194746	-0.002194746
30.0043	-0.002194746	-0.002194746	-0.002194746	-0.002194746	-0.002194746	-0.002194746
30.1102	-0.002194746	-0.002194746	-0.002194746	-0.002194746	-0.002194746	-0.002194746
30.2107	-0.002194746	-0.002194746	-0.002194746	-0.002194746	-0.002194746	-0.002194746
30.3166	-0.002194746	-0.002194746	-0.002194746	-0.002194746	-0.002194746	-0.002194746
30.4171	-0.002194746	-0.002194746	-0.002194746	0.000000000	0.000000000	0.000000000
30.5176	0.000000000	0.000000000	0.000000000	0.000000000	0.000000000	0.000000000
30.6235	0.0000000	0.0000000	0.0000000	0.0000000	0.0000000	0.0000000
30.724	0.0000000	0.0000000	0.0000000	0.0000000	0.0000000	0.0000000
30.8299	0.0000000	0.0000000	0.0000000	0.0000000	0.0000000	0.0000000
30.9304	0.0000000	0.0000000	0.0000000	0.0000000	0.0000000	0.0000000
31.0309	0.0000000	0.0000000	0.0000000	0.0000000	0.0000000	0.0000000
31.1368	0.0000000	0.0000000	0.0000000	0.0000000	0.0000000	0.0000000
31.2373	0.0000000	0.0000000	0.0000000	0.0000000	0.0000000	0.0000000
31.3379	0.0000000	0.0000000	0.0000000	0.0000000	0.0000000	0.0000000
31.4437	0.0000000	0.0000000	0.0000000	0.0000000	0.0000000	0.0000000
31.5442	0.000000000	0.000000000	0.000000000	0.000000000	0.000000000	0.000000000
31.6501	0.000000000	0.000000000	0.000000000	0.000000000	0.000000000	0.000000000
31.7506	0.000000000	0.000000000	0.000000000	0.000000000	0.000000000	0.000000000

CH^- + He PES

Method: MRCI. AV6Z (120º - 180º)

R(Å) \ θ (º)	180	170	160	150	140	130	120
1.58753	10074.95737	10230.74483	10671.01966	11298.68409	11967.86215	12505.88438	6815.610318
1.68807	7169.611874	7291.723152	7637.187146	8127.230033	8647.343135	9072.698078	9279.111745
1.79391	5047.259571	5142.487405	5412.158041	5793.679517	6198.373122	6532.367373	6720.198125
1.89445	3504.636255	3578.899876	3789.419912	4086.880422	4402.994077	4665.850027	4820.373314
2.00029	2389.415581	2447.525871	2612.561991	2845.589147	3093.823699	3301.775882	3427.383391
2.10083	1591.526646	1637.100547	1766.781504	1949.818931	2145.318125	2310.431062	2412.519672
2.20138	1028.945209	1064.484731	1165.840296	1309.128678	1462.921115	1594.120836	1677.137103
2.30721	637.9775463	665.3921185	743.8674565	855.3583586	976.0035462	1080.185944	1147.632683
2.40776	370.0012544	390.9654682	451.30562	537.6644856	632.0429531	714.54565	769.0806986
2.5083	189.1234576	205.1055979	251.3905963	318.1196537	391.6765659	456.6520212	500.3735563
2.61413	69.3012997	81.51945068	117.0436094	168.4972347	225.532099	276.3229109	311.0306241
2.71468	-8.140312914	1.237836743	28.50536105	68.03273651	111.9693567	151.3562684	178.6545191
2.82051	-56.46423034	-49.28521618	-28.45927138	1.705317643	35.32443687	65.68435829	87.03484738
2.92106	-84.94764393	-79.52881606	-63.82540843	-41.01760799	-15.43345387	7.907669839	24.58773944
3.0216	-100.0101857	-96.01355326	-84.38139946	-67.31505457	-47.92886315	-29.99339884	-16.95441285
3.12744	-106.1225533	-103.2452413	-94.78010601	-82.13836905	-67.51916594	-53.76688751	-43.5942398
3.22798	-106.4561547	-104.4238199	-98.33998402	-89.05620844	-78.10881539	-67.6267085	-59.74098612
3.33382	-103.2408518	-101.8208512	-97.48622783	-90.71982591	-82.59926571	-74.69159587	-68.65604437
3.43436	-98.0480828	-97.04508388	-93.94390778	-89.02109251	-83.04699389	-77.14971139	-72.59780819
3.5349	-91.88084654	-91.15877511	-88.91135521	-85.33830872	-80.97515367	-76.64053032	-73.26720572
3.64074	-85.37781415	-84.84449087	-83.19843137	-80.59765736	-77.43063888	-74.28337312	-71.83403658
3.74128	-78.90989768	-78.51264866	-77.30553836	-75.41147256	-73.1135735	-70.85079037	-69.10377256
3.84712	-72.68998752	-72.40247579	-71.52896689	-70.14627691	-68.48265944	-66.85635265	-65.62948964
3.94766	-66.82562621	-66.63029381	-66.02454392	-65.03032398	-63.81223995	-62.64024559	-61.78648939
4.0482	-61.36948765	-61.25536086	-60.86689082	-60.16676684	-59.26472624	-58.40658055	-57.81399913
4.15404	-56.34351931	-56.29304015	-56.07356555	-55.59511093	-54.9213239	-54.28484756	-53.86784582
4.25458	-51.21439791	-51.23634537	-51.21000842	-51.32632996	-50.81934363	-50.33649951	-50.04240355
4.35513	-46.88855354	-46.95000643	-47.01804356	-46.9148905	-46.62737877	-46.3376723	-46.38595671
4.46096	-42.935816	-43.0170216	-43.14651161	-43.13553788	-42.95556871	-42.76462581	-42.6878097
4.56151	-39.32984832	-39.41763816	-39.57565987	-39.62613903	-39.52957021	-39.41324867	-39.38691172
4.66734	-36.0465083	-36.12771391	-36.29670935	-36.3801097	-36.34060427	-36.28354087	-36.29890409
4.76788	-33.06165374	-33.13188562	-33.29210207	-33.38647615	-33.3886709	-33.37550242	-33.42159209
4.86843	-30.35772667	-30.41259532	-30.55305907	-30.6452384	-30.6693806	-30.68693857	-30.7549757
4.97426	-27.91716912	-27.9544798	-28.06641185	-28.14103321	-28.1739544	-28.21126508	-28.29686018
5.07481	-25.16056814	-25.21763154	-25.78607075	-25.86727636	-25.8980028	-25.94628721	-26.04285604
5.18064	-23.1018964	-23.14798606	-23.25772336	-23.8129941	-23.83274681	-23.88542072	-23.98857378
5.28119	-21.23416755	-21.27367298	-21.36146282	-21.52167928	-21.97160221	-22.01769187	-22.12523443
5.38173	-19.54201838	-19.57274483	-19.64517145	-19.72857179	-19.90195673	-20.01388877	-20.44405899
5.48757	-18.00569618	-18.03203314	-18.09129128	-18.16152315	-18.24272875	-18.47537183	-18.63558829
5.58811	-16.61642197	-16.63397993	-16.68226435	-16.74152249	-16.82053334	-16.93246539	-17.25509305
5.69394	-15.35444302	-15.36761149	-15.40492217	-15.45759608	-15.5300227	-15.64195474	-15.79778171
5.79449	-14.20659086	-14.21975933	-14.24829103	-14.2943807	-14.36461257	-14.47215512	-14.62578734
5.89503	-13.16408651	-13.17286549	-13.1970077	-13.23651313	-13.30455025	-13.40989806	-13.55694604
6.00087	-12.21376149	-12.22254047	-12.24009844	-12.27740912	-12.34105676	-12.44420982	-12.58247882
6.10141	-11.34683682	-11.35342106	-11.36878428	-11.40170547	-11.46315836	-11.55972718	-11.69141194
6.20195	-10.55453351	-10.55892301	-10.57209148	-10.60281793	-10.66207607	-10.7542554	-10.87496643
6.30779	-9.825877843	-9.830267333	-9.84343581	-9.871967508	-9.926836158	-10.01243125	-10.12655804
6.40833	-9.156480312	-9.160869804	-9.17403828	-9.200375232	-9.25085439	-9.332059992	-9.435213053
6.51417	-8.541951432	-8.544146179	-8.557314654	-8.58145686	-8.629741272	-8.704362636	-8.798736714
6.61471	-7.973512218	-7.975706964	-7.986680694	-8.0108229	-8.056912565	-8.122754946	-8.208350039
6.71526	-7.446773178	-7.451162671	-7.459941654	-7.48408386	-7.525784034	-7.585042175	-7.661858285
6.82109	-6.959539566	-6.961734313	-6.972708042	-6.994655502	-7.031966184	-7.086834834	-7.154871959
6.92164	-6.50742189	-6.509616635	-6.520590366	-6.54034308	-6.575459016	-6.623743427	-6.683001569
7.02747	-6.088225404	-6.09042015	-6.099199133	-6.116757101	-6.149678292	-6.193573211	-6.246247115
7.12801	-5.697560616	-5.699755361	-5.708534346	-5.726092314	-5.754624013	-5.794129439	-5.83802436
7.22856	-5.335427526	-5.337622272	-5.346401255	-5.359569733	-5.385906684	-5.421022619	-5.460528048
7.33439	-4.997436642	-4.999631388	-5.008410372	-5.021578847	-5.045721054	-5.076447499	-5.111563434
7.43494	-4.685782709	-4.687977456	-4.694561694	-4.70773017	-4.727482884	-4.756014583	-4.78454628
7.53548	-4.393881492	-4.396076238	-4.402660475	-4.413634206	-4.43338692	-4.457529125	-4.481671332
7.64132	-4.123927734	-4.12612248	-4.132706718	-4.141485702	-4.159043669	-4.178796384	-4.200743844
7.74186	-3.873726689	-3.875921435	-3.880310928	-3.891284657	-3.904453134	-3.922011102	-3.941763816

R(Å) \ θ (º)	180	170	160	150	140	130	120
7.84769	-3.641083614	-3.64327836	-3.647667851	-3.656446836	-3.669615312	-3.684978533	-3.700341757
7.94824	-3.425998506	-3.425998506	-3.430387998	-3.439166981	-3.450140712	-3.463309188	-3.476477663
8.04878	-3.224081874	-3.22627662	-3.232860857	-3.23725035	-3.248224081	-3.25919781	-3.270171539
8.15462	-3.03972321	-3.041917955	-3.046307447	-3.052891686	-3.059475925	-3.070449654	-3.079228637
8.25516	-2.866338276	-2.868533022	-2.870727768	-2.879506752	-2.883896244	-2.894869973	-2.901454212
8.361	-2.706121817	-2.708316564	-2.71051131	-2.717095548	-2.723679785	-2.730264024	-2.736848262
8.46154	-2.559073836	-2.559073836	-2.563463328	-2.56785282	-2.572242311	-2.57882655	-2.585410788
8.56208	-2.422999583	-2.422999583	-2.425194329	-2.429583822	-2.433973314	-2.440557551	-2.442752297
8.66792	-2.29350957	-2.29350957	-2.295704316	-2.300093808	-2.3044833	-2.308872791	-2.311067537
8.76846	-2.17279854	-2.174993285	-2.177188031	-2.179382777	-2.18377227	-2.188161762	-2.190356508
8.8743	-2.063061239	-2.063061239	-2.065255985	-2.067450732	-2.071840224	-2.07403497	-2.076229716
8.97484	-1.959908178	-1.959908178	-1.962102924	-1.96429767	-1.968687161	-1.970881907	-1.970881907
9.07539	-1.863339354	-1.863339354	-1.8655341	-1.867728846	-1.869923592	-1.872118339	-1.874313083
9.18122	-1.773354768	-1.773354768	-1.775549514	-1.777744261	-1.779939005	-1.782133751	-1.782133751
9.28176	-1.689954419	-1.689954419	-1.692149166	-1.694343912	-1.694343912	-1.696538658	-1.696538658
9.38231	-1.610943563	-1.610943563	-1.613138309	-1.615333056	-1.615333056	-1.617527802	-1.617527802
9.48814	-1.536322199	-1.538516945	-1.538516945	-1.540711692	-1.542906438	-1.542906438	-1.542906438
9.58869	-1.468285074	-1.468285074	-1.47047982	-1.47047982	-1.472674566	-1.472674566	-1.472674566
9.69452	-1.402442693	-1.402442693	-1.404637439	-1.404637439	-1.406832185	-1.406832185	-1.406832185
9.79507	-1.340989806	-1.343184552	-1.343184552	-1.343184552	-1.345379297	-1.345379297	-1.345379297
9.89561	-1.28392641	-1.28392641	-1.286121156	-1.286121156	-1.286121156	-1.288315903	-1.286121156
10.0014	-1.22905776	-1.22905776	-1.231252507	-1.231252507	-1.231252507	-1.233447251	-1.231252507
10.102	-1.178578601	-1.178578601	-1.178578601	-1.180773347	-1.180773347	-1.180773347	-1.180773347
10.2078	-1.13029419	-1.13029419	-1.13029419	-1.13029419	-1.132488936	-1.132488936	-1.13029419
10.3084	-1.084204523	-1.084204523	-1.084204523	-1.084204523	-1.086399269	-1.086399269	-1.084204523
10.4089	-1.040309604	-1.040309604	-1.040309604	-1.04250435	-1.04250435	-1.04250435	-1.040309604
10.5147	-0.99860943	-0.99860943	-0.99860943	-1.000804176	-1.000804176	-1.000804176	-0.99860943
10.6153	-0.959104002	-0.959104002	-0.961298748	-0.961298748	-0.961298748	-0.961298748	-0.959104002
10.7211	-0.92179332	-0.92179332	-0.92179332	-0.923988066	-0.923988066	-0.923988066	-0.92179332
10.8217	-0.886677384	-0.886677384	-0.886677384	-0.886677384	-0.88887213	-0.886677384	-0.886677384
10.9222	-0.853756194	-0.853756194	-0.853756194	-0.853756194	-0.853756194	-0.853756194	-0.851561448
11.028	-0.820835003	-0.820835003	-0.820835003	-0.820835003	-0.820835003	-0.820835003	-0.820835003
11.1286	-0.79010856	-0.79010856	-0.79010856	-0.79010856	-0.79010856	-0.79010856	-0.79010856
11.2291	-0.761576861	-0.761576861	-0.761576861	-0.761576861	-0.761576861	-0.761576861	-0.759382116
11.335	-0.733045164	-0.733045164	-0.733045164	-0.733045164	-0.733045164	-0.733045164	-0.733045164
11.4355	-0.706708211	-0.706708211	-0.706708211	-0.706708211	-0.706708211	-0.706708211	-0.704513465
11.5414	-0.68037126	-0.68037126	-0.682566006	-0.682566006	-0.682566006	-0.68037126	-0.68037126
11.6419	-0.656229054	-0.656229054	-0.656229054	-0.656229054	-0.656229054	-0.656229054	-0.656229054
11.7424	-0.634281593	-0.634281593	-0.634281593	-0.634281593	-0.634281593	-0.634281593	-0.632086847
11.8483	-0.612334133	-0.612334133	-0.612334133	-0.612334133	-0.612334133	-0.610139387	-0.610139387
11.9488	-0.590386675	-0.590386675	-0.590386675	-0.590386675	-0.590386675	-0.590386675	-0.588191928
12.0547	-0.57063396	-0.57063396	-0.57063396	-0.57063396	-0.57063396	-0.568439214	-0.568439214
12.1552	-0.550881246	-0.550881246	-0.550881246	-0.550881246	-0.550881246	-0.550881246	-0.5486865
12.2557	-0.531128532	-0.531128532	-0.531128532	-0.531128532	-0.531128532	-0.531128532	-0.531128532
12.3616	-0.513570564	-0.513570564	-0.513570564	-0.513570564	-0.513570564	-0.513570564	-0.511375818
12.4621	-0.496012596	-0.496012596	-0.496012596	-0.496012596	-0.496012596	-0.496012596	-0.496012596
12.5627	-0.480649373	-0.480649373	-0.480649373	-0.480649373	-0.480649373	-0.478454627	-0.478454627
12.6685	-0.465286152	-0.465286152	-0.465286152	-0.465286152	-0.463091405	-0.463091405	-0.463091405
12.769	-0.44992293	-0.44992293	-0.44992293	-0.44992293	-0.44992293	-0.447728184	-0.447728184
12.8749	-0.434559708	-0.434559708	-0.434559708	-0.434559708	-0.434559708	-0.434559708	-0.432364962
12.9754	-0.421391233	-0.421391233	-0.421391233	-0.421391233	-0.419196486	-0.419196486	-0.419196486
13.076	-0.406028009	-0.406028009	-0.406028009	-0.406028009	-0.406028009	-0.406028009	-0.406028009
13.1818	-0.39505428	-0.39505428	-0.39505428	-0.392859534	-0.392859534	-0.392859534	-0.392859534
13.2823	-0.381885804	-0.381885804	-0.381885804	-0.381885804	-0.381885804	-0.381885804	-0.379691058
13.3882	-0.368717327	-0.368717327	-0.368717327	-0.368717327	-0.368717327	-0.368717327	-0.368717327
13.4887	-0.357743598	-0.357743598	-0.357743598	-0.357743598	-0.357743598	-0.357743598	-0.357743598
13.5893	-0.346769867	-0.346769867	-0.346769867	-0.346769867	-0.346769867	-0.346769867	-0.344575122
13.6951	-0.335796138	-0.335796138	-0.335796138	-0.335796138	-0.335796138	-0.335796138	-0.335796138
13.7956	-0.327017155	-0.327017155	-0.327017155	-0.324822408	-0.324822408	-0.324822408	-0.324822408
13.9015	-0.316043424	-0.316043424	-0.316043424	-0.316043424	-0.316043424	-0.316043424	-0.313848677
14.002	-0.30726444	-0.30726444	-0.30726444	-0.30726444	-0.30726444	-0.305069694	-0.305069694
14.1026	-0.29629071	-0.29629071	-0.29629071	-0.29629071	-0.29629071	-0.29629071	-0.29629071
14.2084	-0.287511726	-0.287511726	-0.287511726	-0.287511726	-0.287511726	-0.287511726	-0.287511726

R(Å) \ θ (º)	180	170	160	150	140	130	120
14.3089	-0.280927488	-0.280927488	-0.278732742	-0.278732742	-0.278732742	-0.278732742	-0.278732742
14.4095	-0.272148503	-0.272148503	-0.272148503	-0.272148503	-0.272148503	-0.269953759	-0.269953759
14.5153	-0.26336952	-0.26336952	-0.26336952	-0.26336952	-0.26336952	-0.26336952	-0.26336952
14.6159	-0.256785281	-0.256785281	-0.256785281	-0.256785281	-0.254590535	-0.254590535	-0.254590535
14.7217	-0.248006298	-0.248006298	-0.248006298	-0.248006298	-0.248006298	-0.248006298	-0.248006298
14.8222	-0.24142206	-0.24142206	-0.24142206	-0.24142206	-0.24142206	-0.24142206	-0.239227314
14.9228	-0.234837821	-0.234837821	-0.234837821	-0.234837821	-0.234837821	-0.234837821	-0.232643077
15.0286	-0.228253584	-0.228253584	-0.228253584	-0.228253584	-0.228253584	-0.226058838	-0.226058838
15.1292	-0.221669346	-0.221669346	-0.221669346	-0.221669346	-0.221669346	-0.221669346	-0.219474599
15.235	-0.215085107	-0.215085107	-0.215085107	-0.215085107	-0.215085107	-0.215085107	-0.215085107
15.3355	-0.20850087	-0.20850087	-0.20850087	-0.20850087	-0.20850087	-0.20850087	-0.20850087
15.4361	-0.204111378	-0.204111378	-0.204111378	-0.201916632	-0.201916632	-0.201916632	-0.201916632
15.5419	-0.197527139	-0.197527139	-0.197527139	-0.197527139	-0.197527139	-0.197527139	-0.197527139
15.6425	-0.190942902	-0.190942902	-0.190942902	-0.190942902	-0.190942902	-0.190942902	-0.190942902
15.7483	-0.18655341	-0.18655341	-0.18655341	-0.18655341	-0.18655341	-0.18655341	-0.18655341
15.8489	-0.182163917	-0.182163917	-0.182163917	-0.182163917	-0.182163917	-0.182163917	-0.179969171
15.9494	-0.17557968	-0.17557968	-0.17557968	-0.17557968	-0.17557968	-0.17557968	-0.17557968
16.0552	-0.171190188	-0.171190188	-0.171190188	-0.171190188	-0.171190188	-0.171190188	-0.171190188
16.1558	-0.166800696	-0.166800696	-0.166800696	-0.166800696	-0.166800696	-0.166800696	-0.166800696
16.2563	-0.162411203	-0.162411203	-0.162411203	-0.162411203	-0.162411203	-0.162411203	-0.162411203
16.3622	-0.158021711	-0.158021711	-0.158021711	-0.158021711	-0.158021711	-0.158021711	-0.158021711
16.4627	-0.15363222	-0.15363222	-0.15363222	-0.15363222	-0.15363222	-0.15363222	-0.15363222
16.5685	-0.149242728	-0.149242728	-0.149242728	-0.149242728	-0.149242728	-0.149242728	-0.149242728
16.6691	-0.147047982	-0.147047982	-0.147047982	-0.147047982	-0.144853236	-0.144853236	-0.144853236
16.7696	-0.142658489	-0.142658489	-0.142658489	-0.142658489	-0.142658489	-0.142658489	-0.140463743
16.8755	-0.138268999	-0.138268999	-0.138268999	-0.138268999	-0.138268999	-0.138268999	-0.138268999
16.976	-0.133879506	-0.133879506	-0.133879506	-0.133879506	-0.133879506	-0.133879506	-0.133879506
17.0818	-0.13168476	-0.13168476	-0.13168476	-0.13168476	-0.13168476	-0.13168476	-0.13168476
17.1824	-0.127295268	-0.127295268	-0.127295268	-0.127295268	-0.127295268	-0.127295268	-0.127295268
17.2829	-0.125100521	-0.125100521	-0.125100521	-0.125100521	-0.125100521	-0.125100521	-0.122905775
17.3888	-0.120711029	-0.120711029	-0.120711029	-0.120711029	-0.120711029	-0.120711029	-0.120711029
17.4893	-0.118516284	-0.118516284	-0.118516284	-0.118516284	-0.118516284	-0.118516284	-0.118516284
17.5898	-0.116321538	-0.116321538	-0.116321538	-0.114126792	-0.114126792	-0.114126792	-0.114126792
17.6957	-0.111932046	-0.111932046	-0.111932046	-0.111932046	-0.111932046	-0.111932046	-0.111932046
17.7962	-0.1097373	-0.1097373	-0.1097373	-0.1097373	-0.1097373	-0.1097373	-0.1097373
17.9021	-0.107542554	-0.107542554	-0.107542554	-0.107542554	-0.107542554	-0.105347807	-0.105347807
18.0026	-0.103153061	-0.103153061	-0.103153061	-0.103153061	-0.103153061	-0.103153061	-0.103153061
18.1031	-0.100958317	-0.100958317	-0.100958317	-0.100958317	-0.100958317	-0.100958317	-0.100958317
18.209	-0.09876357	-0.09876357	-0.09876357	-0.09876357	-0.09876357	-0.09876357	-0.09876357
18.3095	-0.096568824	-0.096568824	-0.096568824	-0.096568824	-0.096568824	-0.096568824	-0.096568824
18.4154	-0.094374078	-0.094374078	-0.094374078	-0.094374078	-0.094374078	-0.094374078	-0.094374078
18.5159	-0.092179332	-0.092179332	-0.092179332	-0.092179332	-0.092179332	-0.092179332	-0.092179332
18.6164	-0.089984586	-0.089984586	-0.089984586	-0.089984586	-0.089984586	-0.089984586	-0.087789839
18.7223	-0.087789839	-0.087789839	-0.087789839	-0.087789839	-0.087789839	-0.087789839	-0.085595093
18.8228	-0.085595093	-0.085595093	-0.085595093	-0.085595093	-0.085595093	-0.085595093	-0.085595093
18.9287	-0.083400347	-0.083400347	-0.083400347	-0.083400347	-0.083400347	-0.083400347	-0.083400347
19.0292	-0.081205602	-0.081205602	-0.081205602	-0.081205602	-0.081205602	-0.081205602	-0.081205602
19.1297	-0.079010856	-0.079010856	-0.079010856	-0.079010856	-0.079010856	-0.079010856	-0.079010856
19.2356	-0.07681611	-0.07681611	-0.07681611	-0.07681611	-0.07681611	-0.07681611	-0.07681611
19.3361	-0.074621364	-0.074621364	-0.074621364	-0.074621364	-0.074621364	-0.074621364	-0.074621364
19.4367	-0.072426618	-0.072426618	-0.072426618	-0.072426618	-0.072426618	-0.072426618	-0.072426618
19.5425	-0.072426618	-0.072426618	-0.072426618	-0.072426618	-0.070231872	-0.070231872	-0.070231872
19.6431	-0.070231872	-0.070231872	-0.070231872	-0.070231872	-0.070231872	-0.070231872	-0.070231872
19.7489	-0.068037125	-0.068037125	-0.068037125	-0.068037125	-0.068037125	-0.068037125	-0.068037125
19.8494	-0.065842379	-0.065842379	-0.065842379	-0.065842379	-0.065842379	-0.065842379	-0.065842379
19.95	-0.065842379	-0.065842379	-0.065842379	-0.063647633	-0.063647633	-0.063647633	-0.063647633
20.0558	-0.063647633	-0.063647633	-0.063647633	-0.063647633	-0.063647633	-0.063647633	-0.063647633
20.1564	-0.061452888	-0.061452888	-0.061452888	-0.061452888	-0.061452888	-0.061452888	-0.061452888
20.2622	-0.059258142	-0.059258142	-0.059258142	-0.059258142	-0.059258142	-0.059258142	-0.059258142
20.3627	-0.059258142	-0.059258142	-0.059258142	-0.059258142	-0.059258142	-0.059258142	-0.059258142
20.4633	-0.057063396	-0.057063396	-0.057063396	-0.057063396	-0.057063396	-0.057063396	-0.057063396
20.5691	-0.057063396	-0.057063396	-0.05486865	-0.05486865	-0.05486865	-0.05486865	-0.05486865
20.6697	-0.05486865	-0.05486865	-0.05486865	-0.05486865	-0.05486865	-0.05486865	-0.05486865

R(Å) \ θ (º)	180	170	160	150	140	130	120
20.7755	-0.052673904	-0.052673904	-0.052673904	-0.052673904	-0.052673904	-0.052673904	-0.052673904
20.876	-0.052673904	-0.052673904	-0.052673904	-0.052673904	-0.052673904	-0.052673904	-0.052673904
20.9766	-0.050479157	-0.050479157	-0.050479157	-0.050479157	-0.050479157	-0.050479157	-0.050479157
21.0824	-0.050479157	-0.050479157	-0.050479157	-0.050479157	-0.050479157	-0.048284411	-0.048284411
21.183	-0.048284411	-0.048284411	-0.048284411	-0.048284411	-0.048284411	-0.048284411	-0.048284411
21.2835	-0.048284411	-0.048284411	-0.048284411	-0.046089665	-0.046089665	-0.046089665	-0.046089665
21.3893	-0.046089665	-0.046089665	-0.046089665	-0.046089665	-0.046089665	-0.046089665	-0.046089665
21.4899	-0.046089665	-0.043894921	-0.043894921	-0.043894921	-0.043894921	-0.043894921	-0.043894921
21.5957	-0.043894921	-0.043894921	-0.043894921	-0.043894921	-0.043894921	-0.043894921	-0.043894921
21.6963	-0.043894921	-0.043894921	-0.043894921	-0.043894921	-0.041700174	-0.041700174	-0.041700174
21.7968	-0.041700174	-0.041700174	-0.041700174	-0.041700174	-0.041700174	-0.041700174	-0.041700174
21.9026	-0.041700174	-0.041700174	-0.041700174	-0.041700174	-0.041700174	-0.041700174	-0.039505428
22.0032	-0.039505428	-0.039505428	-0.039505428	-0.039505428	-0.039505428	-0.039505428	-0.039505428
22.109	-0.039505428	-0.039505428	-0.039505428	-0.039505428	-0.039505428	-0.039505428	-0.039505428
22.2096	-0.037310682	-0.037310682	-0.037310682	-0.037310682	-0.037310682	-0.037310682	-0.037310682
22.3101	-0.037310682	-0.037310682	-0.037310682	-0.037310682	-0.037310682	-0.037310682	-0.037310682
22.4159	-0.035115936	-0.035115936	-0.035115936	-0.035115936	-0.035115936	-0.035115936	-0.035115936
22.5165	-0.035115936	-0.035115936	-0.035115936	-0.035115936	-0.035115936	-0.035115936	-0.035115936
22.617	-0.035115936	-0.035115936	-0.035115936	-0.035115936	-0.035115936	-0.035115936	-0.035115936
22.7229	-0.03292119	-0.03292119	-0.03292119	-0.03292119	-0.03292119	-0.03292119	-0.03292119
22.8234	-0.03292119	-0.03292119	-0.03292119	-0.03292119	-0.03292119	-0.03292119	-0.03292119
22.9292	-0.03292119	-0.03292119	-0.03292119	-0.03292119	-0.03292119	-0.030726443	-0.030726443
23.0298	-0.030726443	-0.030726443	-0.030726443	-0.030726443	-0.030726443	-0.030726443	-0.030726443
23.1303	-0.030726443	-0.030726443	-0.030726443	-0.030726443	-0.030726443	-0.030726443	-0.030726443
23.2362	-0.030726443	-0.030726443	-0.030726443	-0.028531697	-0.028531697	-0.028531697	-0.028531697
23.3367	-0.028531697	-0.028531697	-0.028531697	-0.028531697	-0.028531697	-0.028531697	-0.028531697
23.4425	-0.028531697	-0.028531697	-0.028531697	-0.028531697	-0.028531697	-0.028531697	-0.028531697
23.5431	-0.028531697	-0.028531697	-0.028531697	-0.028531697	-0.028531697	-0.026336951	-0.026336951
23.6436	-0.026336951	-0.026336951	-0.026336951	-0.026336951	-0.026336951	-0.026336951	-0.026336951
23.7495	-0.026336951	-0.026336951	-0.026336951	-0.026336951	-0.026336951	-0.026336951	-0.026336951
23.85	-0.026336951	-0.026336951	-0.026336951	-0.026336951	-0.026336951	-0.026336951	-0.026336951
23.9558	-0.024142206	-0.024142206	-0.024142206	-0.024142206	-0.024142206	-0.024142206	-0.024142206
24.0564	-0.024142206	-0.024142206	-0.024142206	-0.024142206	-0.024142206	-0.024142206	-0.024142206
24.1569	-0.024142206	-0.024142206	-0.024142206	-0.024142206	-0.024142206	-0.024142206	-0.024142206
24.2628	-0.02194746	-0.02194746	-0.02194746	-0.02194746	-0.02194746	-0.02194746	-0.02194746
24.3633	-0.02194746	-0.02194746	-0.02194746	-0.02194746	-0.02194746	-0.02194746	-0.02194746
24.4639	-0.02194746	-0.02194746	-0.02194746	-0.02194746	-0.02194746	-0.02194746	-0.02194746
24.5697	-0.02194746	-0.02194746	-0.02194746	-0.02194746	-0.02194746	-0.02194746	-0.02194746
24.6702	-0.019752714	-0.019752714	-0.019752714	-0.019752714	-0.019752714	-0.019752714	-0.019752714
24.7761	-0.019752714	-0.019752714	-0.019752714	-0.019752714	-0.019752714	-0.019752714	-0.019752714
24.8766	-0.019752714	-0.019752714	-0.019752714	-0.019752714	-0.019752714	-0.019752714	-0.019752714
24.9772	-0.019752714	-0.019752714	-0.019752714	-0.019752714	-0.019752714	-0.019752714	-0.019752714
25.083	-0.017557968	-0.017557968	-0.017557968	-0.017557968	-0.017557968	-0.017557968	-0.017557968
25.1835	-0.017557968	-0.017557968	-0.017557968	-0.017557968	-0.017557968	-0.017557968	-0.017557968
25.2894	-0.017557968	-0.017557968	-0.017557968	-0.017557968	-0.017557968	-0.017557968	-0.017557968
25.3899	-0.017557968	-0.017557968	-0.017557968	-0.017557968	-0.017557968	-0.017557968	-0.017557968
25.4905	-0.017557968	-0.017557968	-0.017557968	-0.017557968	-0.017557968	-0.017557968	-0.017557968
25.5963	-0.015363222	-0.015363222	-0.015363222	-0.015363222	-0.015363222	-0.015363222	-0.015363222
25.6968	-0.015363222	-0.015363222	-0.015363222	-0.015363222	-0.015363222	-0.015363222	-0.015363222
25.8027	-0.015363222	-0.015363222	-0.015363222	-0.015363222	-0.015363222	-0.015363222	-0.015363222
25.9032	-0.015363222	-0.015363222	-0.015363222	-0.015363222	-0.015363222	-0.015363222	-0.015363222
26.0038	-0.015363222	-0.015363222	-0.015363222	-0.015363222	-0.015363222	-0.015363222	-0.015363222
26.1096	-0.013168476	-0.013168476	-0.013168476	-0.013168476	-0.013168476	-0.013168476	-0.013168476
26.2101	-0.013168476	-0.013168476	-0.013168476	-0.013168476	-0.013168476	-0.013168476	-0.013168476
26.3107	-0.013168476	-0.013168476	-0.013168476	-0.013168476	-0.013168476	-0.013168476	-0.013168476
26.4165	-0.013168476	-0.013168476	-0.013168476	-0.013168476	-0.013168476	-0.013168476	-0.013168476
26.5171	-0.013168476	-0.013168476	-0.013168476	-0.013168476	-0.013168476	-0.013168476	-0.013168476
26.6229	-0.013168476	-0.013168476	-0.013168476	-0.013168476	-0.013168476	-0.010973729	-0.010973729
26.7234	-0.010973729	-0.010973729	-0.010973729	-0.010973729	-0.010973729	-0.010973729	-0.010973729
26.824	-0.010973729	-0.010973729	-0.010973729	-0.010973729	-0.010973729	-0.010973729	-0.010973729
26.9298	-0.010973729	-0.010973729	-0.010973729	-0.010973729	-0.010973729	-0.010973729	-0.010973729
27.0304	-0.010973729	-0.010973729	-0.010973729	-0.010973729	-0.010973729	-0.010973729	-0.010973729
27.1362	-0.010973729	-0.010973729	-0.010973729	-0.010973729	-0.010973729	-0.010973729	-0.010973729

R(Å) \ θ (º)	180	170	160	150	140	130	120
27.2367	-0.010973729	-0.010973729	-0.010973729	-0.010973729	-0.010973729	-0.010973729	-0.010973729
27.3373	-0.008778983	-0.008778983	-0.008778983	-0.008778983	-0.008778983	-0.008778983	-0.008778983
27.4431	-0.008778983	-0.008778983	-0.008778983	-0.008778983	-0.008778983	-0.008778983	-0.008778983
27.5437	-0.008778983	-0.008778983	-0.008778983	-0.008778983	-0.008778983	-0.008778983	-0.008778983
27.6442	-0.008778983	-0.008778983	-0.008778983	-0.008778983	-0.008778983	-0.008778983	-0.008778983
27.75	-0.008778983	-0.008778983	-0.008778983	-0.008778983	-0.008778983	-0.008778983	-0.008778983
27.8506	-0.008778983	-0.008778983	-0.008778983	-0.008778983	-0.008778983	-0.008778983	-0.008778983
27.9564	-0.008778983	-0.008778983	-0.008778983	-0.008778983	-0.008778983	-0.008778983	-0.008778983
28.057	-0.006584239	-0.006584239	-0.006584239	-0.006584239	-0.006584239	-0.006584239	-0.006584239
28.1575	-0.006584239	-0.006584239	-0.006584239	-0.006584239	-0.006584239	-0.006584239	-0.006584239
28.2633	-0.006584239	-0.006584239	-0.006584239	-0.006584239	-0.006584239	-0.006584239	-0.006584239
28.3639	-0.006584239	-0.006584239	-0.006584239	-0.006584239	-0.006584239	-0.006584239	-0.006584239
28.4697	-0.006584239	-0.006584239	-0.006584239	-0.006584239	-0.006584239	-0.006584239	-0.006584239
28.5703	-0.006584239	-0.006584239	-0.006584239	-0.006584239	-0.006584239	-0.006584239	-0.006584239
28.6708	-0.006584239	-0.006584239	-0.006584239	-0.006584239	-0.006584239	-0.006584239	-0.006584239
28.7766	-0.006584239	-0.006584239	-0.006584239	-0.006584239	-0.006584239	-0.006584239	-0.006584239
28.8772	-0.004389492	-0.004389492	-0.004389492	-0.004389492	-0.004389492	-0.004389492	-0.004389492
28.983	-0.004389492	-0.004389492	-0.004389492	-0.004389492	-0.004389492	-0.004389492	-0.004389492
29.0836	-0.004389492	-0.004389492	-0.004389492	-0.004389492	-0.004389492	-0.004389492	-0.004389492
29.1841	-0.004389492	-0.004389492	-0.004389492	-0.004389492	-0.004389492	-0.004389492	-0.004389492
29.2899	-0.004389492	-0.004389492	-0.004389492	-0.004389492	-0.004389492	-0.004389492	-0.004389492
29.3905	-0.004389492	-0.004389492	-0.004389492	-0.004389492	-0.004389492	-0.004389492	-0.004389492
29.491	-0.004389492	-0.004389492	-0.004389492	-0.004389492	-0.004389492	-0.004389492	-0.004389492
29.5969	-0.004389492	-0.004389492	-0.004389492	-0.004389492	-0.004389492	-0.004389492	-0.004389492
29.6974	-0.004389492	-0.004389492	-0.004389492	-0.004389492	-0.004389492	-0.004389492	-0.004389492
29.8032	-0.004389492	-0.004389492	-0.004389492	-0.004389492	-0.004389492	-0.004389492	-0.002194746
29.9038	-0.002194746	-0.002194746	-0.002194746	-0.002194746	-0.002194746	-0.002194746	-0.002194746
30.0043	-0.002194746	-0.002194746	-0.002194746	-0.002194746	-0.002194746	-0.002194746	-0.002194746
30.1102	-0.002194746	-0.002194746	-0.002194746	-0.002194746	-0.002194746	-0.002194746	-0.002194746
30.2107	-0.002194746	-0.002194746	-0.002194746	-0.002194746	-0.002194746	-0.002194746	-0.002194746
30.3166	-0.002194746	-0.002194746	-0.002194746	-0.002194746	-0.002194746	-0.002194746	-0.002194746
30.4171	-0.002194746	-0.002194746	-0.002194746	-0.002194746	-0.002194746	-0.002194746	-0.002194746
30.5176	-0.002194746	-0.002194746	-0.002194746	-0.002194746	-0.002194746	-0.002194746	-0.002194746
30.6235	-0.002194746	-0.002194746	-0.002194746	-0.002194746	-0.002194746	-0.002194746	-0.002194746
30.724	-0.002194746	-0.002194746	-0.002194746	-0.002194746	-0.002194746	-0.002194746	-0.002194746
30.8299	-0.002194746	-0.002194746	-0.002194746	-0.002194746	-0.002194746	-0.002194746	-0.002194746
30.9304	-0.002194746	-0.002194746	-0.002194746	-0.002194746	-0.002194746	-0.002194746	-0.002194746
31.0309	0.000000000	0.000000000	0.000000000	0.000000000	0.000000000	0.000000000	0.000000000
31.1368	0.000000000	0.000000000	0.000000000	0.000000000	0.000000000	0.000000000	0.000000000
31.2373	0.000000000	0.000000000	0.000000000	0.000000000	0.000000000	0.000000000	0.000000000
31.3379	0.000000000	0.000000000	0.000000000	0.000000000	0.000000000	0.000000000	0.000000000
31.4437	0.000000000	0.000000000	0.000000000	0.000000000	0.000000000	0.000000000	0.000000000
31.5442	0.000000000	0.000000000	0.000000000	0.000000000	0.000000000	0.000000000	0.000000000
31.6501	0.000000000	0.000000000	0.000000000	0.000000000	0.000000000	0.000000000	0.000000000
31.7506	0.000000000	0.000000000	0.000000000	0.000000000	0.000000000	0.000000000	0.000000000

CH^- + He PES

Method: MRCI. AV6Z (60º – 110º)

R(Å) \ θ (º)	110	100	90	80	70	60
1.58753	4466.762422	2802.078308	1927.430327	1996.639447	3261.892958	6134.760603
1.68807	3104.196068	1729.369863	957.1748203	917.8054665	1811.361184	3957.570377
1.79391	6697.8183	888.7799507	238.2528468	144.1926175	764.5002637	2343.154725
1.89445	4844.879848	4759.984878	4622.339187	-397.4311899	4861.733302	5479.288737
2.00029	3454.861611	3401.410767	3318.370357	3285.978101	3407.161001	3810.250246
2.10083	2438.643733	2399.686992	2331.136295	2290.500573	2354.143818	2617.87986
2.20138	1701.035693	1672.5852	1616.829873	1575.450132	1604.361521	1773.543516
2.30721	1169.079741	1148.749809	1104.46203	1066.262475	1074.60251	1179.989824
2.40776	787.9555142	773.803792	739.3133586	705.9356614	702.6589056	765.5910524
2.5083	516.6278452	507.0280262	480.5549999	452.3634876	443.0906857	478.3668382
2.61413	324.7390077	318.3918022	298.2813446	275.0280108	263.2334457	281.083315
2.71468	190.0189139	185.9520496	170.8170812	152.0190817	139.8360466	147.203809
2.82051	96.36690737	93.89123388	82.61901842	67.71449834	56.35888253	57.76571472
2.92106	32.22765026	30.85373927	22.55101515	10.93641932	0.987635701	-0.75718737
3.0216	-10.71913946	-11.36658953	-17.43067273	-26.35889946	-34.7340502	-37.94935309
3.12744	-38.55290824	-38.76360385	-43.17723806	-49.97875591	-56.83733716	-60.55962638
3.22798	-55.72679569	-55.7180167	-58.93990383	-64.09316744	-69.62392736	-73.31768488
3.33382	-65.49999962	-65.41440453	-67.78692496	-71.70015707	-76.03478042	-79.4212735
3.43436	-70.22748251	-70.1506664	-71.91524218	-74.8408386	-78.20318947	-81.1529281
3.5349	-71.52457739	-71.49824044	-72.83484076	-75.02739201	-77.59743958	-80.05994459
3.64074	-70.59400509	-70.63351052	-71.65626215	-73.29354267	-75.22491915	-77.20896953
3.74128	-68.2566006	-68.35316942	-69.14547273	-70.35916727	-71.79233641	-73.33524284
3.84712	-65.0742189	-65.21468264	-65.83140627	-66.7202784	-67.76717224	-68.93477711
3.94766	-61.43971952	-61.59993598	-62.0827801	-62.72145119	-63.46985957	-64.33239475
4.0482	-57.60769301	-57.77229896	-58.14760052	-58.59313396	-59.11328876	-59.73440188
4.15404	-53.75371903	-53.91174074	-54.19486298	-54.49334843	-54.83353406	-55.27028852
4.25458	-49.9831454	-50.1214144	-50.33211002	-50.51646868	-50.71619057	-51.01248128
4.35513	-46.35961976	-46.4759413	-46.62957352	-46.72833709	-46.81832167	-47.00048559
4.46096	-42.7404836	-42.88972633	-43.04335855	-43.11578517	-43.15090111	-43.26063841
4.56151	-39.46153308	-39.60199682	-39.73148684	-39.76879752	-39.74685006	-39.79074498
4.66734	-36.38888868	-36.52496293	-36.63908972	-36.65884244	-36.60177904	-36.59300006
4.76788	-33.5269399	-33.66301415	-33.76836196	-33.78153043	-33.70690907	-33.65862466
4.86843	-30.87349198	-31.01176098	-31.11710879	-31.13027726	-31.05346115	-30.97883979
4.97426	-28.42635019	-28.57120343	-28.67435649	-28.6941092	-28.62387733	-28.53389275
5.07481	-26.18331978	-26.33256251	-26.43791032	-26.46424727	-26.40498913	-26.31061505
5.18064	-24.13562176	-24.28705924	-24.39460179	-24.42752298	-24.38362806	-24.29144873
5.28119	-22.2766719	-22.43030412	-22.53345718	-22.57296261	-22.54223617	-22.45664107
5.38173	-20.59549646	-20.74693394	-20.84789225	-20.88739768	-20.74912868	-20.69864953
5.48757	-18.83092068	-19.01527934	-19.14476936	-19.21280648	-19.23036445	-19.19085902
5.58811	-17.45042545	-17.63258936	-17.75768989	-17.82572701	-17.84547973	-17.54479952
5.69394	-15.97994563	-16.38158414	-16.50229517	-16.28501532	-16.28501532	-16.23892565
5.79449	-14.79917228	-14.95060975	-15.04278908	-15.08009977	-15.07790502	-15.03839959
5.89503	-13.72155199	-13.86421048	-13.94541608	-13.97394778	-13.96955829	-13.93444235
6.00087	-12.73830578	-12.8677958	-12.94022242	-12.95997513	-12.95119615	-12.91608021
6.10141	-11.83626518	-11.95258672	-12.0140396	-12.02720808	-12.01184486	-11.97672892
6.20195	-11.00665119	-11.111999	-11.16247816	-11.16906239	-11.14930968	-11.10980425
6.30779	-10.24507433	-10.33725366	-10.37895383	-10.37675909	-10.35042214	-10.31091671
6.40833	-9.542755608	-9.621766463	-9.656882401	-9.648103416	-9.615182226	-9.573482052
6.51417	-8.893110792	-8.961147917	-8.98748487	-8.974316394	-8.937005712	-8.890916046
6.61471	-8.291750388	-8.35100853	-8.368566498	-8.348813784	-8.309308356	-8.261023944
6.71526	-7.734284905	-7.784764062	-7.795737791	-7.771595587	-7.729895412	-7.677221509
6.82109	-7.218519594	-7.258025022	-7.264609259	-7.238272308	-7.192182641	-7.139508738
6.92164	-6.737870219	-6.77079141	-6.772986157	-6.744454458	-6.696170046	-6.643496141
7.02747	-6.292336782	-6.318673734	-6.316478988	-6.285752543	-6.237468132	-6.182599482
7.12801	-5.879724534	-5.899477248	-5.892893009	-5.862166566	-5.813882153	-5.759013503
7.22856	-5.495643983	-5.511007205	-5.502228222	-5.469307032	-5.421022619	-5.368348716
7.33439	-5.137900385	-5.151068862	-5.137900385	-5.104979196	-5.058889529	-5.006215625
7.43494	-4.808688486	-4.815272723	-4.802104248	-4.771377804	-4.725288138	-4.672614234
7.53548	-4.501424046	-4.505813538	-4.492645063	-4.459723871	-4.415828952	-4.36754454
7.64132	-4.216107066	-4.220496558	-4.205133336	-4.174406891	-4.130511972	-4.084422306
7.74186	-3.954932292	-3.954932292	-3.93956907	-3.908842625	-3.867142452	-3.823247532

R(Å) \ θ (º)	110	100	90	80	70	60
7.84769	-3.711315486	-3.70912074	-3.693757518	-3.663031075	-3.625720393	-3.581825472
7.94824	-3.485256648	-3.483061902	-3.465503934	-3.439166981	-3.401856299	-3.360156126
8.04878	-3.276755778	-3.272366285	-3.257003064	-3.230666111	-3.195550175	-3.156044747
8.15462	-3.083618129	-3.079228637	-3.063865415	-3.037528464	-3.004607273	-2.967296591
8.25516	-2.903648958	-2.899259466	-2.883896244	-2.859754038	-2.829027594	-2.793911658
8.361	-2.739043009	-2.73245877	-2.719290294	-2.695148088	-2.666616389	-2.6336952
8.46154	-2.585410788	-2.57882655	-2.565658074	-2.543710614	-2.515178915	-2.486647218
8.56208	-2.442752297	-2.43616806	-2.422999583	-2.403246869	-2.376909918	-2.348378219
8.66792	-2.311067537	-2.3044833	-2.291314823	-2.271562109	-2.247419904	-2.221082952
8.76846	-2.188161762	-2.181577524	-2.170603794	-2.15085108	-2.12890362	-2.104761413
8.8743	-2.076229716	-2.069645478	-2.056477002	-2.038919034	-2.01916632	-1.995024114
8.97484	-1.968687161	-1.96429767	-1.951129193	-1.935765972	-1.916013257	-1.894065797
9.07539	-1.872118339	-1.8655341	-1.854560369	-1.839197147	-1.821639179	-1.799691719
9.18122	-1.779939005	-1.773354768	-1.764575783	-1.749212562	-1.731654594	-1.71190188
9.28176	-1.694343912	-1.687759673	-1.67898069	-1.665812213	-1.648254245	-1.630696277
9.38231	-1.615333056	-1.608748819	-1.599969834	-1.586801358	-1.571438135	-1.553880167
9.48814	-1.540711692	-1.534127453	-1.52534847	-1.512179994	-1.499011517	-1.483648296
9.58869	-1.47047982	-1.463895581	-1.455116598	-1.444142867	-1.430974392	-1.41561117
9.69452	-1.404637439	-1.398053202	-1.391468964	-1.380495234	-1.367326757	-1.351963536
9.79507	-1.340989806	-1.336600314	-1.330016075	-1.319042346	-1.30587387	-1.292705393
9.89561	-1.28392641	-1.279536918	-1.270757933	-1.26197895	-1.251005219	-1.237836743
10.0014	-1.22905776	-1.224668268	-1.218084029	-1.2071103	-1.196136569	-1.18516284
10.102	-1.176383855	-1.171994364	-1.165410126	-1.156631143	-1.145657412	-1.134683682
10.2078	-1.128099444	-1.123709951	-1.117125714	-1.10834673	-1.099567747	-1.088594016
10.3084	-1.082009777	-1.077620286	-1.071036048	-1.064451809	-1.05347808	-1.044699095
10.4089	-1.038114858	-1.033725366	-1.027141127	-1.02055689	-1.011777906	-1.002998922
10.5147	-0.996414684	-0.992025191	-0.987635699	-0.978856716	-0.972272477	-0.963493494
10.6153	-0.956909256	-0.952519763	-0.948130272	-0.941546034	-0.932767049	-0.923988066
10.7211	-0.919598574	-0.915209081	-0.910819591	-0.904235352	-0.897651113	-0.88887213
10.8217	-0.884482638	-0.880093146	-0.875703653	-0.869119416	-0.862535178	-0.853756194
10.9222	-0.849366702	-0.847171956	-0.842782464	-0.836198227	-0.829613988	-0.823029749
11.028	-0.818640257	-0.814250766	-0.809861274	-0.805471782	-0.798887543	-0.79010856
11.1286	-0.787913814	-0.783524321	-0.779134831	-0.774745338	-0.7681611	-0.761576861
11.2291	-0.75718737	-0.754992624	-0.750603132	-0.746213639	-0.739629402	-0.733045164
11.335	-0.730850418	-0.726460925	-0.724266179	-0.717681942	-0.71329245	-0.706708211
11.4355	-0.704513465	-0.700123974	-0.697929228	-0.69134499	-0.686955497	-0.68037126
11.5414	-0.678176514	-0.675981768	-0.671592275	-0.667202783	-0.662813292	-0.656229054
11.6419	-0.654034308	-0.651839561	-0.647450071	-0.643060578	-0.638671086	-0.632086847
11.7424	-0.629892101	-0.627697356	-0.62550261	-0.621113118	-0.616723626	-0.610139387
11.8483	-0.607944642	-0.605749896	-0.60355515	-0.599165658	-0.594776165	-0.590386675
11.9488	-0.588191928	-0.585997182	-0.58160769	-0.577218197	-0.572828705	-0.568439214
12.0547	-0.566244468	-0.564049722	-0.561854976	-0.557465483	-0.553075993	-0.5486865
12.1552	-0.546491754	-0.544297008	-0.542102262	-0.539907515	-0.535518023	-0.531128532
12.2557	-0.528933786	-0.52673904	-0.524544294	-0.520154801	-0.515765311	-0.513570564
12.3616	-0.511375818	-0.509181072	-0.506986326	-0.502596833	-0.498207341	-0.496012596
12.4621	-0.49381785	-0.491623104	-0.489428358	-0.485038866	-0.482844119	-0.478454627
12.5627	-0.476259882	-0.476259882	-0.47187039	-0.469675644	-0.467480898	-0.463091405
12.6685	-0.460896659	-0.458701915	-0.456507168	-0.454312422	-0.452117676	-0.447728184
12.769	-0.445533437	-0.445533437	-0.443338691	-0.4389492	-0.436754454	-0.432364962
12.8749	-0.432364962	-0.430170216	-0.42797547	-0.425780723	-0.421391233	-0.419196486
12.9754	-0.41700174	-0.41700174	-0.414806994	-0.410417502	-0.408222755	-0.406028009
13.076	-0.403833263	-0.403833263	-0.401638518	-0.397249026	-0.39505428	-0.392859534
13.1818	-0.390664788	-0.390664788	-0.388470041	-0.386275295	-0.381885804	-0.379691058
13.2823	-0.379691058	-0.377496312	-0.375301566	-0.37310682	-0.370912074	-0.368717327
13.3882	-0.366522581	-0.366522581	-0.364327837	-0.36213309	-0.359938344	-0.355548852
13.4887	-0.355548852	-0.353354106	-0.353354106	-0.351159359	-0.348964613	-0.344575122
13.5893	-0.344575122	-0.342380376	-0.342380376	-0.34018563	-0.337990884	-0.335796138
13.6951	-0.333601392	-0.333601392	-0.331406645	-0.329211899	-0.327017155	-0.324822408
13.7956	-0.324822408	-0.322627662	-0.320432916	-0.31823817	-0.316043424	-0.313848677
13.9015	-0.313848677	-0.311653931	-0.311653931	-0.309459185	-0.30726444	-0.305069694
14.002	-0.305069694	-0.302874948	-0.300680202	-0.300680202	-0.298485456	-0.29629071
14.1026	-0.29629071	-0.294095963	-0.291901217	-0.291901217	-0.289706471	-0.287511726
14.2084	-0.28531698	-0.28531698	-0.283122234	-0.283122234	-0.280927488	-0.278732742

R(Å) \ θ (º)	110	100	90	80	70	60
14.3089	-0.278732742	-0.276537995	-0.274343249	-0.274343249	-0.272148503	-0.269953759
14.4095	-0.269953759	-0.267759012	-0.267759012	-0.265564266	-0.26336952	-0.261174774
14.5153	-0.261174774	-0.261174774	-0.258980028	-0.258980028	-0.256785281	-0.254590535
14.6159	-0.254590535	-0.252395789	-0.252395789	-0.250201044	-0.248006298	-0.248006298
14.7217	-0.245811552	-0.245811552	-0.243616806	-0.243616806	-0.24142206	-0.239227314
14.8222	-0.239227314	-0.239227314	-0.237032567	-0.237032567	-0.234837821	-0.232643077
14.9228	-0.232643077	-0.232643077	-0.23044833	-0.228253584	-0.228253584	-0.226058838
15.0286	-0.226058838	-0.226058838	-0.223864092	-0.223864092	-0.221669346	-0.219474599
15.1292	-0.219474599	-0.219474599	-0.217279853	-0.217279853	-0.215085107	-0.212890362
15.235	-0.212890362	-0.212890362	-0.210695616	-0.210695616	-0.20850087	-0.20850087
15.3355	-0.206306124	-0.206306124	-0.206306124	-0.204111378	-0.204111378	-0.201916632
15.4361	-0.201916632	-0.199721885	-0.199721885	-0.199721885	-0.197527139	-0.195332395
15.5419	-0.195332395	-0.195332395	-0.195332395	-0.193137648	-0.190942902	-0.190942902
15.6425	-0.190942902	-0.188748156	-0.188748156	-0.188748156	-0.18655341	-0.18655341
15.7483	-0.184358664	-0.184358664	-0.184358664	-0.182163917	-0.182163917	-0.179969171
15.8489	-0.179969171	-0.179969171	-0.177774425	-0.177774425	-0.17557968	-0.17557968
15.9494	-0.17557968	-0.17557968	-0.173384934	-0.173384934	-0.171190188	-0.171190188
16.0552	-0.171190188	-0.168995442	-0.168995442	-0.168995442	-0.166800696	-0.166800696
16.1558	-0.166800696	-0.16460595	-0.16460595	-0.16460595	-0.162411203	-0.162411203
16.2563	-0.162411203	-0.160216457	-0.160216457	-0.160216457	-0.158021711	-0.158021711
16.3622	-0.158021711	-0.155826966	-0.155826966	-0.155826966	-0.15363222	-0.15363222
16.4627	-0.15363222	-0.151437474	-0.151437474	-0.151437474	-0.149242728	-0.149242728
16.5685	-0.149242728	-0.149242728	-0.147047982	-0.147047982	-0.144853236	-0.144853236
16.6691	-0.144853236	-0.144853236	-0.142658489	-0.142658489	-0.142658489	-0.140463743
16.7696	-0.140463743	-0.140463743	-0.140463743	-0.138268999	-0.138268999	-0.138268999
16.8755	-0.138268999	-0.136074252	-0.136074252	-0.136074252	-0.133879506	-0.133879506
16.976	-0.133879506	-0.133879506	-0.13168476	-0.13168476	-0.13168476	-0.129490014
17.0818	-0.129490014	-0.129490014	-0.129490014	-0.129490014	-0.127295268	-0.127295268
17.1824	-0.127295268	-0.127295268	-0.125100521	-0.125100521	-0.125100521	-0.122905775
17.2829	-0.122905775	-0.122905775	-0.122905775	-0.122905775	-0.120711029	-0.120711029
17.3888	-0.120711029	-0.120711029	-0.118516284	-0.118516284	-0.118516284	-0.118516284
17.4893	-0.116321538	-0.116321538	-0.116321538	-0.116321538	-0.114126792	-0.114126792
17.5898	-0.114126792	-0.114126792	-0.114126792	-0.111932046	-0.111932046	-0.111932046
17.6957	-0.111932046	-0.111932046	-0.1097373	-0.1097373	-0.1097373	-0.1097373
17.7962	-0.107542554	-0.107542554	-0.107542554	-0.107542554	-0.107542554	-0.105347807
17.9021	-0.105347807	-0.105347807	-0.105347807	-0.105347807	-0.103153061	-0.103153061
18.0026	-0.103153061	-0.103153061	-0.103153061	-0.100958317	-0.100958317	-0.100958317
18.1031	-0.100958317	-0.100958317	-0.09876357	-0.09876357	-0.09876357	-0.09876357
18.209	-0.09876357	-0.096568824	-0.096568824	-0.096568824	-0.096568824	-0.096568824
18.3095	-0.096568824	-0.094374078	-0.094374078	-0.094374078	-0.094374078	-0.092179332
18.4154	-0.092179332	-0.092179332	-0.092179332	-0.092179332	-0.092179332	-0.089984586
18.5159	-0.089984586	-0.089984586	-0.089984586	-0.089984586	-0.089984586	-0.087789839
18.6164	-0.087789839	-0.087789839	-0.087789839	-0.087789839	-0.087789839	-0.085595093
18.7223	-0.085595093	-0.085595093	-0.085595093	-0.085595093	-0.085595093	-0.083400347
18.8228	-0.083400347	-0.083400347	-0.083400347	-0.083400347	-0.083400347	-0.081205602
18.9287	-0.081205602	-0.081205602	-0.081205602	-0.081205602	-0.081205602	-0.081205602
19.0292	-0.081205602	-0.079010856	-0.079010856	-0.079010856	-0.079010856	-0.079010856
19.1297	-0.079010856	-0.07681611	-0.07681611	-0.07681611	-0.07681611	-0.07681611
19.2356	-0.07681611	-0.07681611	-0.074621364	-0.074621364	-0.074621364	-0.074621364
19.3361	-0.074621364	-0.074621364	-0.074621364	-0.072426618	-0.072426618	-0.072426618
19.4367	-0.072426618	-0.072426618	-0.072426618	-0.072426618	-0.070231872	-0.070231872
19.5425	-0.070231872	-0.070231872	-0.070231872	-0.070231872	-0.070231872	-0.070231872
19.6431	-0.068037125	-0.068037125	-0.068037125	-0.068037125	-0.068037125	-0.068037125
19.7489	-0.068037125	-0.068037125	-0.065842379	-0.065842379	-0.065842379	-0.065842379
19.8494	-0.065842379	-0.065842379	-0.065842379	-0.065842379	-0.063647633	-0.063647633
19.95	-0.063647633	-0.063647633	-0.063647633	-0.063647633	-0.063647633	-0.063647633
20.0558	-0.063647633	-0.061452888	-0.061452888	-0.061452888	-0.061452888	-0.061452888
20.1564	-0.061452888	-0.061452888	-0.061452888	-0.059258142	-0.059258142	-0.059258142
20.2622	-0.059258142	-0.059258142	-0.059258142	-0.059258142	-0.059258142	-0.059258142
20.3627	-0.059258142	-0.057063396	-0.057063396	-0.057063396	-0.057063396	-0.057063396
20.4633	-0.057063396	-0.057063396	-0.057063396	-0.057063396	-0.05486865	-0.05486865
20.5691	-0.05486865	-0.05486865	-0.05486865	-0.05486865	-0.05486865	-0.05486865
20.6697	-0.05486865	-0.05486865	-0.052673904	-0.052673904	-0.052673904	-0.052673904

R(Å) \ θ (º)	110	100	90	80	70	60
20.7755	-0.052673904	-0.052673904	-0.052673904	-0.052673904	-0.052673904	-0.052673904
20.876	-0.050479157	-0.050479157	-0.050479157	-0.050479157	-0.050479157	-0.050479157
20.9766	-0.050479157	-0.050479157	-0.050479157	-0.050479157	-0.050479157	-0.048284411
21.0824	-0.048284411	-0.048284411	-0.048284411	-0.048284411	-0.048284411	-0.048284411
21.183	-0.048284411	-0.048284411	-0.048284411	-0.048284411	-0.046089665	-0.046089665
21.2835	-0.046089665	-0.046089665	-0.046089665	-0.046089665	-0.046089665	-0.046089665
21.3893	-0.046089665	-0.046089665	-0.046089665	-0.046089665	-0.043894921	-0.043894921
21.4899	-0.043894921	-0.043894921	-0.043894921	-0.043894921	-0.043894921	-0.043894921
21.5957	-0.043894921	-0.043894921	-0.043894921	-0.043894921	-0.041700174	-0.041700174
21.6963	-0.041700174	-0.041700174	-0.041700174	-0.041700174	-0.041700174	-0.041700174
21.7968	-0.041700174	-0.041700174	-0.041700174	-0.041700174	-0.041700174	-0.039505428
21.9026	-0.039505428	-0.039505428	-0.039505428	-0.039505428	-0.039505428	-0.039505428
22.0032	-0.039505428	-0.039505428	-0.039505428	-0.039505428	-0.039505428	-0.039505428
22.109	-0.039505428	-0.037310682	-0.037310682	-0.037310682	-0.037310682	-0.037310682
22.2096	-0.037310682	-0.037310682	-0.037310682	-0.037310682	-0.037310682	-0.037310682
22.3101	-0.037310682	-0.037310682	-0.037310682	-0.035115936	-0.035115936	-0.035115936
22.4159	-0.035115936	-0.035115936	-0.035115936	-0.035115936	-0.035115936	-0.035115936
22.5165	-0.035115936	-0.035115936	-0.035115936	-0.035115936	-0.035115936	-0.035115936
22.617	-0.035115936	-0.03292119	-0.03292119	-0.03292119	-0.03292119	-0.03292119
22.7229	-0.03292119	-0.03292119	-0.03292119	-0.03292119	-0.03292119	-0.03292119
22.8234	-0.03292119	-0.03292119	-0.03292119	-0.03292119	-0.030726443	-0.030726443
22.9292	-0.030726443	-0.030726443	-0.030726443	-0.030726443	-0.030726443	-0.030726443
23.0298	-0.030726443	-0.030726443	-0.030726443	-0.030726443	-0.030726443	-0.030726443
23.1303	-0.030726443	-0.030726443	-0.030726443	-0.030726443	-0.028531697	-0.028531697
23.2362	-0.028531697	-0.028531697	-0.028531697	-0.028531697	-0.028531697	-0.028531697
23.3367	-0.028531697	-0.028531697	-0.028531697	-0.028531697	-0.028531697	-0.028531697
23.4425	-0.028531697	-0.028531697	-0.028531697	-0.028531697	-0.026336951	-0.026336951
23.5431	-0.026336951	-0.026336951	-0.026336951	-0.026336951	-0.026336951	-0.026336951
23.6436	-0.026336951	-0.026336951	-0.026336951	-0.026336951	-0.026336951	-0.026336951
23.7495	-0.026336951	-0.026336951	-0.026336951	-0.026336951	-0.026336951	-0.026336951
23.85	-0.024142206	-0.024142206	-0.024142206	-0.024142206	-0.024142206	-0.024142206
23.9558	-0.024142206	-0.024142206	-0.024142206	-0.024142206	-0.024142206	-0.024142206
24.0564	-0.024142206	-0.024142206	-0.024142206	-0.024142206	-0.024142206	-0.024142206
24.1569	-0.024142206	-0.024142206	-0.024142206	-0.02194746	-0.02194746	-0.02194746
24.2628	-0.02194746	-0.02194746	-0.02194746	-0.02194746	-0.02194746	-0.02194746
24.3633	-0.02194746	-0.02194746	-0.02194746	-0.02194746	-0.02194746	-0.02194746
24.4639	-0.02194746	-0.02194746	-0.02194746	-0.02194746	-0.02194746	-0.02194746
24.5697	-0.02194746	-0.02194746	-0.02194746	-0.019752714	-0.019752714	-0.019752714
24.6702	-0.019752714	-0.019752714	-0.019752714	-0.019752714	-0.019752714	-0.019752714
24.7761	-0.019752714	-0.019752714	-0.019752714	-0.019752714	-0.019752714	-0.019752714
24.8766	-0.019752714	-0.019752714	-0.019752714	-0.019752714	-0.019752714	-0.019752714
24.9772	-0.019752714	-0.019752714	-0.019752714	-0.017557968	-0.017557968	-0.017557968
25.083	-0.017557968	-0.017557968	-0.017557968	-0.017557968	-0.017557968	-0.017557968
25.1835	-0.017557968	-0.017557968	-0.017557968	-0.017557968	-0.017557968	-0.017557968
25.2894	-0.017557968	-0.017557968	-0.017557968	-0.017557968	-0.017557968	-0.017557968
25.3899	-0.017557968	-0.017557968	-0.017557968	-0.017557968	-0.017557968	-0.017557968
25.4905	-0.017557968	-0.015363222	-0.015363222	-0.015363222	-0.015363222	-0.015363222
25.5963	-0.015363222	-0.015363222	-0.015363222	-0.015363222	-0.015363222	-0.015363222
25.6968	-0.015363222	-0.015363222	-0.015363222	-0.015363222	-0.015363222	-0.015363222
25.8027	-0.015363222	-0.015363222	-0.015363222	-0.015363222	-0.015363222	-0.015363222
25.9032	-0.015363222	-0.015363222	-0.015363222	-0.015363222	-0.015363222	-0.015363222
26.0038	-0.015363222	-0.013168476	-0.013168476	-0.013168476	-0.013168476	-0.013168476
26.1096	-0.013168476	-0.013168476	-0.013168476	-0.013168476	-0.013168476	-0.013168476
26.2101	-0.013168476	-0.013168476	-0.013168476	-0.013168476	-0.013168476	-0.013168476
26.3107	-0.013168476	-0.013168476	-0.013168476	-0.013168476	-0.013168476	-0.013168476
26.4165	-0.013168476	-0.013168476	-0.013168476	-0.013168476	-0.013168476	-0.013168476
26.5171	-0.013168476	-0.013168476	-0.013168476	-0.013168476	-0.010973729	-0.010973729
26.6229	-0.010973729	-0.010973729	-0.010973729	-0.010973729	-0.010973729	-0.010973729
26.7234	-0.010973729	-0.010973729	-0.010973729	-0.010973729	-0.010973729	-0.010973729
26.824	-0.010973729	-0.010973729	-0.010973729	-0.010973729	-0.010973729	-0.010973729
26.9298	-0.010973729	-0.010973729	-0.010973729	-0.010973729	-0.010973729	-0.010973729
27.0304	-0.010973729	-0.010973729	-0.010973729	-0.010973729	-0.010973729	-0.010973729
27.1362	-0.010973729	-0.010973729	-0.010973729	-0.010973729	-0.010973729	-0.008778983

R(Å) \ θ (º)	110	100	90	80	70	60
27.2367	-0.008778983	-0.008778983	-0.008778983	-0.008778983	-0.008778983	-0.008778983
27.3373	-0.008778983	-0.008778983	-0.008778983	-0.008778983	-0.008778983	-0.008778983
27.4431	-0.008778983	-0.008778983	-0.008778983	-0.008778983	-0.008778983	-0.008778983
27.5437	-0.008778983	-0.008778983	-0.008778983	-0.008778983	-0.008778983	-0.008778983
27.6442	-0.008778983	-0.008778983	-0.008778983	-0.008778983	-0.008778983	-0.008778983
27.75	-0.008778983	-0.008778983	-0.008778983	-0.008778983	-0.008778983	-0.008778983
27.8506	-0.008778983	-0.008778983	-0.008778983	-0.008778983	-0.008778983	-0.008778983
27.9564	-0.008778983	-0.006584239	-0.006584239	-0.006584239	-0.006584239	-0.006584239
28.057	-0.006584239	-0.006584239	-0.006584239	-0.006584239	-0.006584239	-0.006584239
28.1575	-0.006584239	-0.006584239	-0.006584239	-0.006584239	-0.006584239	-0.006584239
28.2633	-0.006584239	-0.006584239	-0.006584239	-0.006584239	-0.006584239	-0.006584239
28.3639	-0.006584239	-0.006584239	-0.006584239	-0.006584239	-0.006584239	-0.006584239
28.4697	-0.006584239	-0.006584239	-0.006584239	-0.006584239	-0.006584239	-0.006584239
28.5703	-0.006584239	-0.006584239	-0.006584239	-0.006584239	-0.006584239	-0.006584239
28.6708	-0.006584239	-0.006584239	-0.006584239	-0.006584239	-0.006584239	-0.006584239
28.7766	-0.006584239	-0.006584239	-0.004389492	-0.004389492	-0.004389492	-0.004389492
28.8772	-0.004389492	-0.004389492	-0.004389492	-0.004389492	-0.004389492	-0.004389492
28.983	-0.004389492	-0.004389492	-0.004389492	-0.004389492	-0.004389492	-0.004389492
29.0836	-0.004389492	-0.004389492	-0.004389492	-0.004389492	-0.004389492	-0.004389492
29.1841	-0.004389492	-0.004389492	-0.004389492	-0.004389492	-0.004389492	-0.004389492
29.2899	-0.004389492	-0.004389492	-0.004389492	-0.004389492	-0.004389492	-0.004389492
29.3905	-0.004389492	-0.004389492	-0.004389492	-0.004389492	-0.004389492	-0.004389492
29.491	-0.004389492	-0.004389492	-0.004389492	-0.004389492	-0.004389492	-0.004389492
29.5969	-0.004389492	-0.004389492	-0.004389492	-0.004389492	-0.004389492	-0.004389492
29.6974	-0.004389492	-0.004389492	-0.004389492	-0.004389492	-0.002194746	-0.002194746
29.8032	-0.002194746	-0.002194746	-0.002194746	-0.002194746	-0.002194746	-0.002194746
29.9038	-0.002194746	-0.002194746	-0.002194746	-0.002194746	-0.002194746	-0.002194746
30.0043	-0.002194746	-0.002194746	-0.002194746	-0.002194746	-0.002194746	-0.002194746
30.1102	-0.002194746	-0.002194746	-0.002194746	-0.002194746	-0.002194746	-0.002194746
30.2107	-0.002194746	-0.002194746	-0.002194746	-0.002194746	-0.002194746	-0.002194746
30.3166	-0.002194746	-0.002194746	-0.002194746	-0.002194746	-0.002194746	-0.002194746
30.4171	-0.002194746	-0.002194746	-0.002194746	-0.002194746	-0.002194746	-0.002194746
30.5176	-0.002194746	-0.002194746	-0.002194746	-0.002194746	-0.002194746	-0.002194746
30.6235	-0.002194746	-0.002194746	-0.002194746	-0.002194746	-0.002194746	-0.002194746
30.724	-0.002194746	-0.002194746	-0.002194746	-0.002194746	-0.002194746	-0.002194746
30.8299	-0.002194746	-0.002194746	-0.002194746	-0.002194746	-0.002194746	-0.002194746
30.9304	-0.002194746	0.000000000	0.000000000	0.000000000	0.000000000	0.000000000
31.0309	0.000000000	0.000000000	0.000000000	0.000000000	0.000000000	0.000000000
31.1368	0.000000000	0.000000000	0.000000000	0.000000000	0.000000000	0.000000000
31.2373	0.000000000	0.000000000	0.000000000	0.000000000	0.000000000	0.000000000
31.3379	0.000000000	0.000000000	0.000000000	0.000000000	0.000000000	0.000000000
31.4437	0.000000000	0.000000000	0.000000000	0.000000000	0.000000000	0.000000000
31.5442	0.000000000	0.000000000	0.000000000	0.000000000	0.000000000	0.000000000
31.6501	0.000000000	0.000000000	0.000000000	0.000000000	0.000000000	0.000000000
31.7506	0.000000000	0.000000000	0.000000000	0.000000000	0.000000000	0.000000000

CH⁻ + He PES

Method: MRCI. AV6Z (0º – 50º)

R(Å) \ θ (º)	50	40	30	20	10	0
1.58753	11217.75503	23497.6925	30414.85372	40343.05062	54251.73104	62919.63236
1.68807	7820.389179	17043.81583	22206.67048	28966.44426	36788.57607	40984.84922
1.79391	9492.401551	12207.21456	16012.41909	20729.25526	25519.46179	27807.54156
1.89445	6678.322372	8641.737754	11411.02217	14739.56078	17858.72502	19238.76594
2.00029	4646.226803	6047.734535	8038.746653	10391.82821	12499.24308	13392.10745
2.10083	3193.895338	4181.489337	5595.864865	7254.267617	8705.093486	9304.309624
2.20138	2165.073034	2851.945132	3844.988686	5007.69708	6011.670469	6418.655388
2.30721	1441.994212	1913.419068	2603.10483	3412.625919	4104.561295	4380.851285
2.40776	937.8369133	1257.22513	1731.393419	2289.872876	2763.411273	2951.900449
2.5083	589.3902593	803.1782724	1125.652302	1506.769945	1829.147406	1958.299429
2.61413	351.0452332	492.461497	709.0719534	966.2610687	1184.227878	1271.984797
2.71468	190.0562246	282.3277359	425.9233825	597.6468938	743.2836541	801.8504511
2.82051	82.99870948	142.1273615	236.0954115	349.6581537	445.507101	483.8646769
2.92106	13.2496816	50.19164627	110.9070996	185.3748314	247.4268851	272.1243618
3.0216	-30.89763419	-8.634130764	29.99998307	78.46655899	118.0268556	133.482257
3.12744	-57.64939318	-44.87377672	-20.8786187	10.39212231	35.38369501	44.67844432
3.22798	-72.73388244	-65.86652221	-51.51727286	-31.65482156	-15.83289764	-10.38992756
3.33382	-80.12798171	-76.7590466	-68.58800725	-56.31279287	-46.17526109	-42.89192108
3.43436	-82.48294417	-81.14195437	-76.71734643	-69.41542649	-62.82021476	-60.63424774
3.5349	-81.63796696	-81.42288185	-79.16887771	-74.98569184	-70.66643171	-69.0532934
3.64074	-78.80674462	-79.22155162	-78.15929455	-75.84603227	-73.05212061	-71.82745234
3.74128	-74.80791741	-75.56510478	-75.17882948	-73.92562952	-72.19397492	-71.31388178
3.84712	-70.19236657	-71.09879667	-71.12732837	-70.4798783	-69.46810039	-68.89966118
3.94766	-65.34197791	-66.25060276	-66.53811448	-66.25060276	-65.71069524	-65.38806758
4.0482	-60.50475773	-61.31242426	-61.73162074	-61.66358362	-61.40679833	-61.25097137
4.15404	-55.83214349	-56.48398306	-56.92732175	-56.9821904	-56.8724531	-56.81100021
4.25458	-51.41850929	-51.90135341	-52.28982345	-52.39078177	-52.3446921	-52.32054989
4.35513	-47.27043935	-47.55575633	-47.76645194	-47.77962042	-47.99690027	-47.97714756
4.46096	-43.4559708	-43.64471896	-43.76104049	-43.74567727	-43.65788743	-43.60521353
4.56151	-39.93559822	-40.06728298	-40.10898315	-40.05630925	-39.92681923	-39.89609279
4.66734	-36.69615312	-36.79930618	-36.79711144	-36.71590583	-36.56446836	-36.53593666
4.76788	-33.72446704	-33.81664637	-33.7990884	-33.70251958	-33.52913464	-33.50060294
4.86843	-31.00737149	-31.09077184	-31.07979811	-30.98322928	-30.78570214	-30.75936519
4.97426	-28.531698	-28.60192987	-28.60412462	-28.38026053	-28.29686018	-28.27052323
5.07481	-26.2798886	-26.33036776	-26.34792573	-26.11089316	-26.03188231	-26.00554535
5.18064	-24.23658008	-24.26511178	-24.10050583	-24.03685819	-23.96443157	-23.94028937
5.28119	-22.3864092	-22.31178784	-22.10328697	-22.04402882	-21.96940746	-21.94087576
5.38173	-20.63500189	-20.38480085	-20.36504813	-20.31456898	-20.25311609	-20.22458439
5.48757	-18.87262085	-18.80897322	-18.77824678	-18.73435186	-18.67948321	-18.655341
5.58811	-17.45262019	-17.37360934	-17.33190916	-17.28801424	-17.24411932	-17.22217186
5.69394	-16.15113581	-16.06554072	-16.00847732	-15.96677715	-15.92727172	-15.90971375
5.79449	-14.95719399	-14.8672094	-14.80136702	-14.7574721	-14.72235617	-14.70699295
5.89503	-13.85982099	-13.7698364	-13.69740979	-13.64912537	-13.61620418	-13.60303571
6.00087	-12.84804308	-12.76025324	-12.68343713	-12.63295798	-12.60223153	-12.58906306
6.10141	-11.91308129	-11.82968094	-11.75286483	-11.69799618	-11.66726974	-11.65629601
6.20195	-11.05054611	-10.97153525	-10.89471914	-10.83985049	-10.8069293	-10.79595557
6.30779	-10.25385331	-10.1770372	-10.10241584	-10.04754719	-10.014626	-10.00365227
6.40833	-9.51422391	-9.443992038	-9.37156542	-9.31669677	-9.283775579	-9.27280185
6.51417	-8.83385265	-8.765815524	-8.695583653	-8.640715001	-8.607793812	-8.596820082
6.61471	-8.201765802	-8.138118168	-8.072275788	-8.017407137	-7.984485947	-7.973512218
6.71526	-7.620158112	-7.556510478	-7.495057589	-7.442383686	-7.409462496	-7.400683511
6.82109	-7.082445342	-7.020992453	-6.961734313	-6.911255154	-6.88052871	-6.869554979
6.92164	-6.584238001	-6.524979858	-6.467916462	-6.421826796	-6.391100352	-6.382321367
7.02747	-6.125536086	-6.06847269	-6.01360404	-5.96970912	-5.941177422	-5.932398438
7.12801	-5.704144853	-5.647081457	-5.5966023	-5.554902126	-5.526370428	-5.517591444
7.22856	-5.313480066	-5.258611416	-5.210327003	-5.173016321	-5.14667937	-5.137900385
7.33439	-4.953541722	-4.903062564	-4.856972898	-4.819662216	-4.795520009	-4.786741026
7.43494	-4.622135076	-4.573850663	-4.529955745	-4.494839807	-4.472892348	-4.464113364
7.53548	-4.317065382	-4.270975715	-4.229275541	-4.198549098	-4.176601637	-4.1700174
7.64132	-4.036137894	-3.992242974	-3.952737546	-3.924205848	-3.904453134	-3.897868896
7.74186	-3.777157865	-3.735457692	-3.700341757	-3.671810058	-3.65425209	-3.647667851

R(Å) \ θ (º)	50	40	30	20	10	0
7.84769	-3.540125298	-3.50061987	-3.465503934	-3.439166981	-3.423803759	-3.417219522
7.94824	-3.320650698	-3.283340016	-3.252613571	-3.228471366	-3.213108143	-3.206523906
8.04878	-3.118734065	-3.083618129	-3.055086432	-3.030944226	-3.01777575	-3.011191512
8.15462	-2.932180655	-2.899259466	-2.872922513	-2.850975054	-2.837806577	-2.833417087
8.25516	-2.760990467	-2.730264024	-2.703927071	-2.684174357	-2.671005882	-2.668811135
8.361	-2.602968756	-2.574437057	-2.550294852	-2.530542138	-2.519568408	-2.515178915
8.46154	-2.455920774	-2.429583822	-2.407636362	-2.390078394	-2.379104664	-2.374715172
8.56208	-2.319846522	-2.295704316	-2.275951602	-2.258393634	-2.249614651	-2.245225158
8.66792	-2.196940745	-2.17279854	-2.153045826	-2.137682603	-2.12890362	-2.126708874
8.76846	-2.080619207	-2.058671748	-2.04111378	-2.025750557	-2.01916632	-2.014776828
8.8743	-1.973076654	-1.953323939	-1.935765972	-1.922597496	-1.916013257	-1.911623765
8.97484	-1.874313083	-1.854560369	-1.839197147	-1.826028672	-1.819444433	-1.817249687
9.07539	-1.779939005	-1.762381037	-1.747017816	-1.736044086	-1.729459848	-1.727265101
9.18122	-1.694343912	-1.676785944	-1.663617468	-1.652643738	-1.646059501	-1.643864754
9.28176	-1.613138309	-1.597775088	-1.584606612	-1.575827627	-1.56924339	-1.567048644
9.38231	-1.538516945	-1.523153724	-1.509985248	-1.501206263	-1.496816771	-1.494622026
9.48814	-1.468285074	-1.452921852	-1.441948121	-1.433169138	-1.428779646	-1.4265849
9.58869	-1.400247948	-1.387079471	-1.376105742	-1.369521503	-1.362937266	-1.362937266
9.69452	-1.33879506	-1.327821329	-1.3168476	-1.308068615	-1.303679124	-1.301484378
9.79507	-1.281731664	-1.268563188	-1.259784204	-1.251005219	-1.246615728	-1.246615728
9.89561	-1.224668268	-1.213694537	-1.204915554	-1.198331315	-1.193941825	-1.191747078
10.0014	-1.17418911	-1.16321538	-1.154436396	-1.147852158	-1.143462665	-1.143462665
10.102	-1.123709951	-1.114930968	-1.106151984	-1.099567747	-1.097373	-1.095178254
10.2078	-1.077620286	-1.068841302	-1.062257065	-1.055672826	-1.051283334	-1.051283334
10.3084	-1.033725366	-1.024946381	-1.018362144	-1.011777906	-1.009583159	-1.007388413
10.4089	-0.992025191	-0.985440954	-0.97666197	-0.972272477	-0.967882987	-0.967882987
10.5147	-0.954714509	-0.945935526	-0.939351288	-0.932767049	-0.930572303	-0.930572303
10.6153	-0.915209081	-0.908624844	-0.902040606	-0.897651113	-0.893261621	-0.893261621
10.7211	-0.880093146	-0.873508909	-0.86692467	-0.862535178	-0.860340431	-0.858145685
10.8217	-0.847171956	-0.840587717	-0.83400348	-0.829613988	-0.827419242	-0.825224496
10.9222	-0.814250766	-0.807666528	-0.803277035	-0.798887543	-0.796692798	-0.794498052
11.028	-0.783524321	-0.776940084	-0.772550592	-0.7681611	-0.765966353	-0.765966353
11.1286	-0.754992624	-0.748408386	-0.744018893	-0.739629402	-0.737434656	-0.737434656
11.2291	-0.726460925	-0.722071434	-0.717681942	-0.71329245	-0.711097704	-0.708902957
11.335	-0.700123974	-0.695734482	-0.69134499	-0.686955497	-0.684760753	-0.684760753
11.4355	-0.675981768	-0.669397529	-0.665008038	-0.662813292	-0.660618546	-0.660618546
11.5414	-0.651839561	-0.645255324	-0.640865832	-0.638671086	-0.63647634	-0.63647634
11.6419	-0.627697356	-0.623307864	-0.618918372	-0.616723626	-0.614528879	-0.614528879
11.7424	-0.605749896	-0.601360404	-0.596970911	-0.594776165	-0.592581419	-0.592581419
11.8483	-0.583802436	-0.579412944	-0.577218197	-0.572828705	-0.572828705	-0.57063396
11.9488	-0.564049722	-0.55966023	-0.557465483	-0.553075993	-0.553075993	-0.550881246
12.0547	-0.544297008	-0.539907515	-0.537712769	-0.535518023	-0.533323278	-0.533323278
12.1552	-0.52673904	-0.522349548	-0.520154801	-0.517960055	-0.515765311	-0.515765311
12.2557	-0.509181072	-0.50479158	-0.502596833	-0.500402087	-0.498207341	-0.498207341
12.3616	-0.491623104	-0.487233612	-0.485038866	-0.482844119	-0.480649373	-0.480649373
12.4621	-0.474065136	-0.47187039	-0.469675644	-0.467480898	-0.465286152	-0.465286152
12.5627	-0.458701915	-0.456507168	-0.454312422	-0.452117676	-0.44992293	-0.44992293
12.6685	-0.443338691	-0.441143945	-0.4389492	-0.436754454	-0.434559708	-0.434559708
12.769	-0.430170216	-0.425780723	-0.423585977	-0.421391233	-0.421391233	-0.421391233
12.8749	-0.414806994	-0.412612248	-0.410417502	-0.408222755	-0.408222755	-0.408222755
12.9754	-0.401638518	-0.399443772	-0.397249026	-0.39505428	-0.39505428	-0.39505428
13.076	-0.390664788	-0.386275295	-0.384080549	-0.384080549	-0.381885804	-0.381885804
13.1818	-0.377496312	-0.375301566	-0.37310682	-0.370912074	-0.370912074	-0.368717327
13.2823	-0.364327837	-0.36213309	-0.359938344	-0.359938344	-0.357743598	-0.357743598
13.3882	-0.353354106	-0.351159359	-0.348964613	-0.348964613	-0.346769867	-0.346769867
13.4887	-0.342380376	-0.34018563	-0.337990884	-0.337990884	-0.335796138	-0.335796138
13.5893	-0.331406645	-0.329211899	-0.329211899	-0.327017155	-0.327017155	-0.324822408
13.6951	-0.322627662	-0.320432916	-0.31823817	-0.316043424	-0.316043424	-0.316043424
13.7956	-0.311653931	-0.309459185	-0.309459185	-0.30726444	-0.30726444	-0.30726444
13.9015	-0.302874948	-0.300680202	-0.298485456	-0.298485456	-0.298485456	-0.29629071
14.002	-0.294095963	-0.291901217	-0.289706471	-0.289706471	-0.287511726	-0.287511726
14.1026	-0.28531698	-0.283122234	-0.280927488	-0.280927488	-0.280927488	-0.278732742
14.2084	-0.276537995	-0.274343249	-0.274343249	-0.272148503	-0.272148503	-0.272148503

R(Å) \ θ (º)	50	40	30	20	10	0
14.3089	-0.267759012	-0.267759012	-0.265564266	-0.26336952	-0.26336952	-0.26336952
14.4095	-0.261174774	-0.258980028	-0.256785281	-0.256785281	-0.256785281	-0.254590535
14.5153	-0.252395789	-0.252395789	-0.250201044	-0.248006298	-0.248006298	-0.248006298
14.6159	-0.245811552	-0.243616806	-0.243616806	-0.24142206	-0.24142206	-0.24142206
14.7217	-0.239227314	-0.237032567	-0.234837821	-0.234837821	-0.234837821	-0.234837821
14.8222	-0.232643077	-0.23044833	-0.228253584	-0.228253584	-0.228253584	-0.228253584
14.9228	-0.223864092	-0.223864092	-0.221669346	-0.221669346	-0.221669346	-0.221669346
15.0286	-0.219474599	-0.217279853	-0.217279853	-0.215085107	-0.215085107	-0.215085107
15.1292	-0.212890362	-0.210695616	-0.210695616	-0.20850087	-0.20850087	-0.20850087
15.235	-0.206306124	-0.204111378	-0.204111378	-0.204111378	-0.201916632	-0.201916632
15.3355	-0.199721885	-0.199721885	-0.197527139	-0.197527139	-0.197527139	-0.197527139
15.4361	-0.195332395	-0.193137648	-0.193137648	-0.193137648	-0.190942902	-0.190942902
15.5419	-0.188748156	-0.188748156	-0.18655341	-0.18655341	-0.18655341	-0.18655341
15.6425	-0.184358664	-0.184358664	-0.182163917	-0.182163917	-0.182163917	-0.182163917
15.7483	-0.179969171	-0.177774425	-0.177774425	-0.177774425	-0.17557968	-0.17557968
15.8489	-0.173384934	-0.173384934	-0.173384934	-0.171190188	-0.171190188	-0.171190188
15.9494	-0.168995442	-0.168995442	-0.168995442	-0.166800696	-0.166800696	-0.166800696
16.0552	-0.16460595	-0.16460595	-0.162411203	-0.162411203	-0.162411203	-0.162411203
16.1558	-0.160216457	-0.160216457	-0.158021711	-0.158021711	-0.158021711	-0.158021711
16.2563	-0.155826966	-0.155826966	-0.155826966	-0.15363222	-0.15363222	-0.15363222
16.3622	-0.151437474	-0.151437474	-0.151437474	-0.149242728	-0.149242728	-0.149242728
16.4627	-0.147047982	-0.147047982	-0.147047982	-0.147047982	-0.144853236	-0.144853236
16.5685	-0.144853236	-0.142658489	-0.142658489	-0.142658489	-0.142658489	-0.142658489
16.6691	-0.140463743	-0.140463743	-0.138268999	-0.138268999	-0.138268999	-0.138268999
16.7696	-0.136074252	-0.136074252	-0.136074252	-0.133879506	-0.133879506	-0.133879506
16.8755	-0.133879506	-0.13168476	-0.13168476	-0.13168476	-0.13168476	-0.13168476
16.976	-0.129490014	-0.129490014	-0.129490014	-0.127295268	-0.127295268	-0.127295268
17.0818	-0.127295268	-0.125100521	-0.125100521	-0.125100521	-0.125100521	-0.125100521
17.1824	-0.122905775	-0.122905775	-0.122905775	-0.120711029	-0.120711029	-0.120711029
17.2829	-0.120711029	-0.118516284	-0.118516284	-0.118516284	-0.118516284	-0.118516284
17.3888	-0.116321538	-0.116321538	-0.116321538	-0.116321538	-0.114126792	-0.114126792
17.4893	-0.114126792	-0.114126792	-0.111932046	-0.111932046	-0.111932046	-0.111932046
17.5898	-0.111932046	-0.1097373	-0.1097373	-0.1097373	-0.1097373	-0.1097373
17.6957	-0.107542554	-0.107542554	-0.107542554	-0.107542554	-0.107542554	-0.105347807
17.7962	-0.105347807	-0.105347807	-0.105347807	-0.103153061	-0.103153061	-0.103153061
17.9021	-0.103153061	-0.103153061	-0.100958317	-0.100958317	-0.100958317	-0.100958317
18.0026	-0.100958317	-0.09876357	-0.09876357	-0.09876357	-0.09876357	-0.09876357
18.1031	-0.096568824	-0.096568824	-0.096568824	-0.096568824	-0.096568824	-0.096568824
18.209	-0.094374078	-0.094374078	-0.094374078	-0.094374078	-0.094374078	-0.094374078
18.3095	-0.092179332	-0.092179332	-0.092179332	-0.092179332	-0.092179332	-0.092179332
18.4154	-0.089984586	-0.089984586	-0.089984586	-0.089984586	-0.089984586	-0.089984586
18.5159	-0.087789839	-0.087789839	-0.087789839	-0.087789839	-0.087789839	-0.087789839
18.6164	-0.085595093	-0.085595093	-0.085595093	-0.085595093	-0.085595093	-0.085595093
18.7223	-0.083400347	-0.083400347	-0.083400347	-0.083400347	-0.083400347	-0.083400347
18.8228	-0.081205602	-0.081205602	-0.081205602	-0.081205602	-0.081205602	-0.081205602
18.9287	-0.079010856	-0.079010856	-0.079010856	-0.079010856	-0.079010856	-0.079010856
19.0292	-0.07681611	-0.07681611	-0.07681611	-0.07681611	-0.07681611	-0.07681611
19.1297	-0.07681611	-0.074621364	-0.074621364	-0.074621364	-0.074621364	-0.074621364
19.2356	-0.074621364	-0.074621364	-0.072426618	-0.072426618	-0.072426618	-0.072426618
19.3361	-0.072426618	-0.072426618	-0.072426618	-0.072426618	-0.070231872	-0.070231872
19.4367	-0.070231872	-0.070231872	-0.070231872	-0.070231872	-0.070231872	-0.070231872
19.5425	-0.068037125	-0.068037125	-0.068037125	-0.068037125	-0.068037125	-0.068037125
19.6431	-0.068037125	-0.065842379	-0.065842379	-0.065842379	-0.065842379	-0.065842379
19.7489	-0.065842379	-0.065842379	-0.065842379	-0.063647633	-0.063647633	-0.063647633
19.8494	-0.063647633	-0.063647633	-0.063647633	-0.063647633	-0.063647633	-0.063647633
19.95	-0.061452888	-0.061452888	-0.061452888	-0.061452888	-0.061452888	-0.061452888
20.0558	-0.061452888	-0.061452888	-0.059258142	-0.059258142	-0.059258142	-0.059258142
20.1564	-0.059258142	-0.059258142	-0.059258142	-0.059258142	-0.059258142	-0.059258142
20.2622	-0.057063396	-0.057063396	-0.057063396	-0.057063396	-0.057063396	-0.057063396
20.3627	-0.057063396	-0.057063396	-0.057063396	-0.05486865	-0.05486865	-0.05486865
20.4633	-0.05486865	-0.05486865	-0.05486865	-0.05486865	-0.05486865	-0.05486865
20.5691	-0.05486865	-0.052673904	-0.052673904	-0.052673904	-0.052673904	-0.052673904
20.6697	-0.052673904	-0.052673904	-0.052673904	-0.052673904	-0.052673904	-0.052673904

R(Å) \ θ (º)	50	40	30	20	10	0
20.7755	-0.050479157	-0.050479157	-0.050479157	-0.050479157	-0.050479157	-0.050479157
20.876	-0.050479157	-0.050479157	-0.050479157	-0.050479157	-0.050479157	-0.048284411
20.9766	-0.048284411	-0.048284411	-0.048284411	-0.048284411	-0.048284411	-0.048284411
21.0824	-0.048284411	-0.048284411	-0.048284411	-0.046089665	-0.046089665	-0.046089665
21.183	-0.046089665	-0.046089665	-0.046089665	-0.046089665	-0.046089665	-0.046089665
21.2835	-0.046089665	-0.046089665	-0.046089665	-0.043894921	-0.043894921	-0.043894921
21.3893	-0.043894921	-0.043894921	-0.043894921	-0.043894921	-0.043894921	-0.043894921
21.4899	-0.043894921	-0.043894921	-0.043894921	-0.041700174	-0.041700174	-0.041700174
21.5957	-0.041700174	-0.041700174	-0.041700174	-0.041700174	-0.041700174	-0.041700174
21.6963	-0.041700174	-0.041700174	-0.041700174	-0.041700174	-0.041700174	-0.041700174
21.7968	-0.039505428	-0.039505428	-0.039505428	-0.039505428	-0.039505428	-0.039505428
21.9026	-0.039505428	-0.039505428	-0.039505428	-0.039505428	-0.039505428	-0.039505428
22.0032	-0.037310682	-0.037310682	-0.037310682	-0.037310682	-0.037310682	-0.037310682
22.109	-0.037310682	-0.037310682	-0.037310682	-0.037310682	-0.037310682	-0.037310682
22.2096	-0.037310682	-0.037310682	-0.035115936	-0.035115936	-0.035115936	-0.035115936
22.3101	-0.035115936	-0.035115936	-0.035115936	-0.035115936	-0.035115936	-0.035115936
22.4159	-0.035115936	-0.035115936	-0.035115936	-0.035115936	-0.035115936	-0.035115936
22.5165	-0.03292119	-0.03292119	-0.03292119	-0.03292119	-0.03292119	-0.03292119
22.617	-0.03292119	-0.03292119	-0.03292119	-0.03292119	-0.03292119	-0.03292119
22.7229	-0.03292119	-0.03292119	-0.03292119	-0.03292119	-0.03292119	-0.030726443
22.8234	-0.030726443	-0.030726443	-0.030726443	-0.030726443	-0.030726443	-0.030726443
22.9292	-0.030726443	-0.030726443	-0.030726443	-0.030726443	-0.030726443	-0.030726443
23.0298	-0.030726443	-0.030726443	-0.030726443	-0.028531697	-0.028531697	-0.028531697
23.1303	-0.028531697	-0.028531697	-0.028531697	-0.028531697	-0.028531697	-0.028531697
23.2362	-0.028531697	-0.028531697	-0.028531697	-0.028531697	-0.028531697	-0.028531697
23.3367	-0.028531697	-0.028531697	-0.028531697	-0.028531697	-0.026336951	-0.026336951
23.4425	-0.026336951	-0.026336951	-0.026336951	-0.026336951	-0.026336951	-0.026336951
23.5431	-0.026336951	-0.026336951	-0.026336951	-0.026336951	-0.026336951	-0.026336951
23.6436	-0.026336951	-0.026336951	-0.026336951	-0.026336951	-0.026336951	-0.026336951
23.7495	-0.024142206	-0.024142206	-0.024142206	-0.024142206	-0.024142206	-0.024142206
23.85	-0.024142206	-0.024142206	-0.024142206	-0.024142206	-0.024142206	-0.024142206
23.9558	-0.024142206	-0.024142206	-0.024142206	-0.024142206	-0.024142206	-0.024142206
24.0564	-0.024142206	-0.024142206	-0.02194746	-0.02194746	-0.02194746	-0.02194746
24.1569	-0.02194746	-0.02194746	-0.02194746	-0.02194746	-0.02194746	-0.02194746
24.2628	-0.02194746	-0.02194746	-0.02194746	-0.02194746	-0.02194746	-0.02194746
24.3633	-0.02194746	-0.02194746	-0.02194746	-0.02194746	-0.02194746	-0.02194746
24.4639	-0.02194746	-0.019752714	-0.019752714	-0.019752714	-0.019752714	-0.019752714
24.5697	-0.019752714	-0.019752714	-0.019752714	-0.019752714	-0.019752714	-0.019752714
24.6702	-0.019752714	-0.019752714	-0.019752714	-0.019752714	-0.019752714	-0.019752714
24.7761	-0.019752714	-0.019752714	-0.019752714	-0.019752714	-0.019752714	-0.019752714
24.8766	-0.019752714	-0.019752714	-0.019752714	-0.017557968	-0.017557968	-0.017557968
24.9772	-0.017557968	-0.017557968	-0.017557968	-0.017557968	-0.017557968	-0.017557968
25.083	-0.017557968	-0.017557968	-0.017557968	-0.017557968	-0.017557968	-0.017557968
25.1835	-0.017557968	-0.017557968	-0.017557968	-0.017557968	-0.017557968	-0.017557968
25.2894	-0.017557968	-0.017557968	-0.017557968	-0.017557968	-0.017557968	-0.017557968
25.3899	-0.015363222	-0.015363222	-0.015363222	-0.015363222	-0.015363222	-0.015363222
25.4905	-0.015363222	-0.015363222	-0.015363222	-0.015363222	-0.015363222	-0.015363222
25.5963	-0.015363222	-0.015363222	-0.015363222	-0.015363222	-0.015363222	-0.015363222
25.6968	-0.015363222	-0.015363222	-0.015363222	-0.015363222	-0.015363222	-0.015363222
25.8027	-0.015363222	-0.015363222	-0.015363222	-0.015363222	-0.015363222	-0.015363222
25.9032	-0.013168476	-0.013168476	-0.013168476	-0.013168476	-0.013168476	-0.013168476
26.0038	-0.013168476	-0.013168476	-0.013168476	-0.013168476	-0.013168476	-0.013168476
26.1096	-0.013168476	-0.013168476	-0.013168476	-0.013168476	-0.013168476	-0.013168476
26.2101	-0.013168476	-0.013168476	-0.013168476	-0.013168476	-0.013168476	-0.013168476
26.3107	-0.013168476	-0.013168476	-0.013168476	-0.013168476	-0.013168476	-0.013168476
26.4165	-0.013168476	-0.013168476	-0.013168476	-0.013168476	-0.010973729	-0.010973729
26.5171	-0.010973729	-0.010973729	-0.010973729	-0.010973729	-0.010973729	-0.010973729
26.6229	-0.010973729	-0.010973729	-0.010973729	-0.010973729	-0.010973729	-0.010973729
26.7234	-0.010973729	-0.010973729	-0.010973729	-0.010973729	-0.010973729	-0.010973729
26.824	-0.010973729	-0.010973729	-0.010973729	-0.010973729	-0.010973729	-0.010973729
26.9298	-0.010973729	-0.010973729	-0.010973729	-0.010973729	-0.010973729	-0.010973729
27.0304	-0.010973729	-0.010973729	-0.010973729	-0.010973729	-0.010973729	-0.010973729
27.1362	-0.008778983	-0.008778983	-0.008778983	-0.008778983	-0.008778983	-0.008778983

R(Å) \ θ (º)	50	40	30	20	10	0
27.2367	-0.008778983	-0.008778983	-0.008778983	-0.008778983	-0.008778983	-0.008778983
27.3373	-0.008778983	-0.008778983	-0.008778983	-0.008778983	-0.008778983	-0.008778983
27.4431	-0.008778983	-0.008778983	-0.008778983	-0.008778983	-0.008778983	-0.008778983
27.5437	-0.008778983	-0.008778983	-0.008778983	-0.008778983	-0.008778983	-0.008778983
27.6442	-0.008778983	-0.008778983	-0.008778983	-0.008778983	-0.008778983	-0.008778983
27.75	-0.008778983	-0.008778983	-0.008778983	-0.008778983	-0.008778983	-0.008778983
27.8506	-0.006584239	-0.006584239	-0.006584239	-0.006584239	-0.006584239	-0.006584239
27.9564	-0.006584239	-0.006584239	-0.006584239	-0.006584239	-0.006584239	-0.006584239
28.057	-0.006584239	-0.006584239	-0.006584239	-0.006584239	-0.006584239	-0.006584239
28.1575	-0.006584239	-0.006584239	-0.006584239	-0.006584239	-0.006584239	-0.006584239
28.2633	-0.006584239	-0.006584239	-0.006584239	-0.006584239	-0.006584239	-0.006584239
28.3639	-0.006584239	-0.006584239	-0.006584239	-0.006584239	-0.006584239	-0.006584239
28.4697	-0.006584239	-0.006584239	-0.006584239	-0.006584239	-0.006584239	-0.006584239
28.5703	-0.006584239	-0.006584239	-0.006584239	-0.006584239	-0.006584239	-0.006584239
28.6708	-0.006584239	-0.004389492	-0.004389492	-0.004389492	-0.004389492	-0.004389492
28.7766	-0.004389492	-0.004389492	-0.004389492	-0.004389492	-0.004389492	-0.004389492
28.8772	-0.004389492	-0.004389492	-0.004389492	-0.004389492	-0.004389492	-0.004389492
28.983	-0.004389492	-0.004389492	-0.004389492	-0.004389492	-0.004389492	-0.004389492
29.0836	-0.004389492	-0.004389492	-0.004389492	-0.004389492	-0.004389492	-0.004389492
29.1841	-0.004389492	-0.004389492	-0.004389492	-0.004389492	-0.004389492	-0.004389492
29.2899	-0.004389492	-0.004389492	-0.004389492	-0.004389492	-0.004389492	-0.004389492
29.3905	-0.004389492	-0.004389492	-0.004389492	-0.004389492	-0.004389492	-0.004389492
29.491	-0.004389492	-0.004389492	-0.004389492	-0.004389492	-0.004389492	-0.004389492
29.5969	-0.004389492	-0.004389492	-0.004389492	-0.004389492	-0.004389492	-0.004389492
29.6974	-0.002194746	-0.002194746	-0.002194746	-0.002194746	-0.002194746	-0.002194746
29.8032	-0.002194746	-0.002194746	-0.002194746	-0.002194746	-0.002194746	-0.002194746
29.9038	-0.002194746	-0.002194746	-0.002194746	-0.002194746	-0.002194746	-0.002194746
30.0043	-0.002194746	-0.002194746	-0.002194746	-0.002194746	-0.002194746	-0.002194746
30.1102	-0.002194746	-0.002194746	-0.002194746	-0.002194746	-0.002194746	-0.002194746
30.2107	-0.002194746	-0.002194746	-0.002194746	-0.002194746	-0.002194746	-0.002194746
30.3166	-0.002194746	-0.002194746	-0.002194746	-0.002194746	-0.002194746	-0.002194746
30.4171	-0.002194746	-0.002194746	-0.002194746	-0.002194746	-0.002194746	-0.002194746
30.5176	-0.002194746	-0.002194746	-0.002194746	-0.002194746	-0.002194746	-0.002194746
30.6235	-0.002194746	-0.002194746	-0.002194746	-0.002194746	-0.002194746	-0.002194746
30.724	-0.002194746	-0.002194746	-0.002194746	-0.002194746	-0.002194746	-0.002194746
30.8299	0.000000000	0.000000000	0.000000000	0.000000000	0.000000000	0.000000000
30.9304	0.000000000	0.000000000	0.000000000	0.000000000	0.000000000	0.000000000
31.0309	0.000000000	0.000000000	0.000000000	0.000000000	0.000000000	0.000000000
31.1368	0.000000000	0.000000000	0.000000000	0.000000000	0.000000000	0.000000000
31.2373	0.000000000	0.000000000	0.000000000	0.000000000	0.000000000	0.000000000
31.3379	0.000000000	0.000000000	0.000000000	0.000000000	0.000000000	0.000000000
31.4437	0.000000000	0.000000000	0.000000000	0.000000000	0.000000000	0.000000000
31.5442	0.000000000	0.000000000	0.000000000	0.000000000	0.000000000	0.000000000
31.6501	0.000000000	0.000000000	0.000000000	0.000000000	0.000000000	0.000000000
31.7506	0.000000000	0.000000000	0.000000000	0.000000000	0.000000000	0.000000000

CH^- + He PES
Method: MRCI. CBS extrapolation (140º – 180º)

R(Å) \ θ(º)	180	170	160	150	140
1.89445	3483.47	3555.79	3761.24	4053.49	4366.67
2.00029	2372.5	2428.45	2587.96	2815.43	3060.32
2.10083	1577.01	1620.72	1745.48	1923.27	2115.32
2.20138	1017.83	1051.78	1148.61	1286.69	1436.64
2.30721	631.415	657.16	731.059	837.153	953.714
2.40776	367.894	387.064	442.732	523.708	614.243
2.5083	190.454	204.625	246.524	308.318	378.536
2.61413	72.9002	83.5821	115.039	162.36	216.567
2.71468	-3.3952	4.68772	28.6176	64.6484	106.12
2.82051	-51.5392	-45.3969	-27.3092	-0.131089	31.297
2.92106	-80.4229	-75.9466	-62.5163	-42.3129	-18.709
3.0216	-96.2144	-93.0664	-83.4472	-68.6794	-51.0223
3.12744	-103.062	-100.952	-94.3637	-83.6797	-70.5513
3.22798	-104.012	-102.689	-98.2887	-90.727	-80.9773
3.33382	-101.3	-100.517	-97.6469	-92.3488	-85.1547
3.43436	-96.5805	-96.116	-94.2387	-90.502	-85.2288
3.5349	-90.919	-90.6201	-89.3234	-86.6286	-82.7784
3.64074	-84.9236	-84.7097	-83.7754	-81.792	-78.9068
3.74128	-78.96	-78.7964	-78.093	-76.579	-74.3563
3.84712	-73.2068	-73.0894	-72.5637	-71.3664	-69.5979
3.94766	-67.7456	-67.6831	-67.3225	-66.3704	-64.9095
4.0482	-62.6092	-62.6079	-62.4114	-61.6645	-60.4237
4.15404	-57.8124	-57.8704	-57.8273	-57.262	-56.1984
4.25458	-52.9416	-52.4318	-52.6568	-53.1444	-52.2394
4.35513	-48.7128	-48.8634	-49.0812	-48.8646	-47.8265
4.46096	-44.7984	-44.9661	-45.2519	-45.1568	-44.6085
4.56151	-41.1866	-41.3545	-41.6718	-41.6693	-41.2543
4.66734	-37.8609	-38.0391	-38.3375	-38.3973	-38.0968
4.76788	-34.8075	-34.9678	-35.2469	-35.3435	-35.1351
4.86843	-32.0131	-32.1513	-32.3972	-32.5098	-32.3679
4.97426	-29.465	-29.5811	-29.7841	-29.8931	-29.7985
5.07481	-26.0029	-26.1746	-27.3336	-27.4935	-27.4268
5.18064	-24.5381	-24.6322	-24.2408	-25.3087	-25.2571
5.28119	-22.5667	-22.6447	-22.8077	-22.4221	-23.2881
5.38173	-20.7728	-20.8358	-20.9712	-21.0823	-20.6975
5.48757	-19.141	-19.1955	-19.3072	-19.403	-19.259
5.58811	-17.6625	-17.7056	-17.7966	-17.881	-17.945
5.69394	-16.3205	-16.3553	-16.4289	-16.5037	-16.5742
5.79449	-15.1025	-15.1303	-15.1905	-15.2573	-15.3339
5.89503	-13.9965	-14.0191	-14.068	-14.1279	-14.2089
6.00087	-12.9885	-13.0081	-13.0486	-13.1041	-13.1867
6.10141	-12.0723	-12.0865	-12.1208	-12.1735	-12.2569
6.20195	-11.2332	-11.245	-11.2752	-11.3247	-11.4077
6.30779	-10.4647	-10.4748	-10.5017	-10.5486	-10.6313
6.40833	-9.75899	-9.76715	-9.79193	-9.83682	-9.91723
6.51417	-9.10847	-9.11612	-9.1387	-9.18322	-9.25867
6.61471	-8.50722	-8.51431	-8.53821	-8.59264	-8.64948
6.71526	-7.95158	-7.9582	-7.9923	-8.12275	-8.08301
6.82109	-7.4476	-7.45378	-7.56204	-7.59696	-7.55529
6.92164	-7.05485	-7.06033	-7.06175	-7.09422	-7.0627
7.02747	-6.58924	-6.59428	-6.51666	-6.54596	-6.6022
7.12801	-6.07647	-6.08114	-6.09477	-6.12142	-6.17277
7.22856	-5.68457	-5.68863	-5.70216	-5.7252	-5.77035
7.33439	-5.32016	-5.32398	-5.33587	-5.35638	-5.39561
7.43494	-4.98602	-4.99041	-5.00003	-5.01841	-5.0455
7.53548	-4.71157	-4.71459	-4.72319	-4.74041	-4.72
7.64132	-4.36445	-4.36723	-4.37577	-4.38976	-4.41651
7.74186	-4.08929	-4.09159	-4.09826	-4.11212	-4.13452

R(Å) \ θ(º)	180	170	160	150	140
7.84769	-3.83352	-3.83617	-3.84191	-3.85337	-3.87225
7.94824	-3.59647	-3.5982	-3.6031	-3.61283	-3.62929
8.04878	-3.37562	-3.37833	-3.38456	-3.39003	-3.40439
8.15462	-3.17123	-3.17355	-3.17888	-3.18695	-3.19584
8.25516	-2.98192	-2.98307	-2.98649	-2.99528	-3.00278
8.361	-2.80663	-2.80766	-2.81049	-2.81571	-2.82432
8.46154	-2.64445	-2.6453	-2.64777	-2.6521	-2.65937
8.56208	-2.49669	-2.49531	-2.49726	-2.50102	-2.50721
8.66792	-2.35416	-2.35472	-2.35642	-2.35954	-2.36447
8.76846	-2.22549	-2.22596	-2.22737	-2.22998	-2.23393
8.8743	-2.10627	-2.10668	-2.10787	-2.11001	-2.11311
8.97484	-1.99573	-1.99607	-1.99706	-1.9988	-2.00119
9.07539	-1.89314	-1.89342	-1.89423	-1.89563	-1.89743
9.18122	-1.79782	-1.79806	-1.79871	-1.79988	-1.80111
9.28176	-1.70915	-1.70953	-1.71004	-1.71076	-1.71175
9.38231	-1.6267	-1.62685	-1.62724	-1.62778	-1.62839
9.48814	-1.5496	-1.54972	-1.55001	-1.5504	-1.55069
9.58869	-1.47758	-1.47767	-1.47784	-1.47813	-1.47817
9.69452	-1.4102	-1.41026	-1.41039	-1.41055	-1.41037
9.79507	-1.34706	-1.3471	-1.34716	-1.34722	-1.34689
9.89561	-1.2878	-1.28782	-1.28784	-1.2878	-1.28734
10.0014	-1.23211	-1.23211	-1.23208	-1.23197	-1.23141
10.102	-1.17968	-1.17968	-1.17961	-1.17943	-1.1788
10.2078	-1.13028	-1.13026	-1.13016	-1.12993	-1.12928
10.3084	-1.08363	-1.0836	-1.08348	-1.08321	-1.08252
10.4089	-1.03954	-1.0395	-1.03935	-1.03905	-1.03832
10.5147	-0.997808	-0.99777	-0.997607	-0.997273	-0.996525
10.6153	-0.958272	-0.958233	-0.958056	-0.957701	-0.956937
10.7211	-0.92077	-0.920727	-0.920542	-0.92017	-0.919399
10.8217	-0.885166	-0.885114	-0.884922	-0.884539	-0.883764
10.9222	-0.851322	-0.851267	-0.851069	-0.850679	-0.849901
11.028	-0.819124	-0.819069	-0.818864	-0.818467	-0.817702
11.1286	-0.78847	-0.788412	-0.788203	-0.787809	-0.787048
11.2291	-0.759259	-0.759201	-0.758991	-0.758594	-0.757842
11.335	-0.731406	-0.731347	-0.731138	-0.730741	-0.729999
11.4355	-0.704826	-0.704769	-0.704558	-0.704163	-0.703434
11.5414	-0.679446	-0.679392	-0.679183	-0.67879	-0.678075
11.6419	-0.655203	-0.655148	-0.654941	-0.654551	-0.653848
11.7424	-0.632027	-0.631972	-0.631766	-0.631382	-0.630695
11.8483	-0.609862	-0.609807	-0.609603	-0.609223	-0.60855
11.9488	-0.588651	-0.588596	-0.588395	-0.58802	-0.587363
12.0547	-0.568345	-0.568287	-0.568093	-0.567724	-0.567082
12.1552	-0.548896	-0.548839	-0.548647	-0.548284	-0.547655
12.2557	-0.530259	-0.530204	-0.530015	-0.529659	-0.529046
12.3616	-0.512395	-0.512341	-0.512154	-0.511804	-0.511205
12.4621	-0.495263	-0.495208	-0.495025	-0.494682	-0.494097
12.5627	-0.478828	-0.478774	-0.478593	-0.478258	-0.477688
12.6685	-0.463055	-0.463003	-0.462825	-0.462495	-0.46194
12.769	-0.447912	-0.44786	-0.447686	-0.447363	-0.44682
12.8749	-0.433368	-0.433318	-0.433147	-0.432832	-0.432301
12.9754	-0.419396	-0.419346	-0.419179	-0.418869	-0.418353
13.076	-0.405968	-0.405919	-0.405755	-0.405453	-0.404961
13.1818	-0.39306	-0.393027	-0.392868	-0.392572	-0.39208
13.2823	-0.380646	-0.380599	-0.380441	-0.380151	-0.379674
13.3882	-0.368705	-0.368659	-0.368502	-0.36822	-0.367754
13.4887	-0.357214	-0.357169	-0.357017	-0.356739	-0.356285
13.5893	-0.346154	-0.34611	-0.345961	-0.34569	-0.345246
13.6951	-0.335505	-0.335462	-0.335316	-0.335051	-0.334618
13.7956	-0.325249	-0.325208	-0.325064	-0.324804	-0.324383
13.9015	-0.31537	-0.315328	-0.315188	-0.314935	-0.314524

R(Å) \ θ(º)	180	170	160	150	140
14.002	-0.305849	-0.305807	-0.30567	-0.305422	-0.305023
14.1026	-0.296672	-0.296632	-0.296497	-0.296254	-0.295864
14.2084	-0.287824	-0.287784	-0.287652	-0.287415	-0.287034
14.3089	-0.279291	-0.279252	-0.279121	-0.27889	-0.278519
14.4095	-0.271058	-0.27102	-0.270895	-0.270668	-0.270306
14.5153	-0.263114	-0.263077	-0.262954	-0.262733	-0.262379
14.6159	-0.255447	-0.255411	-0.255291	-0.255074	-0.254729
14.7217	-0.248046	-0.24801	-0.247893	-0.247681	-0.247345
14.8222	-0.2409	-0.240865	-0.240749	-0.240542	-0.240215
14.9228	-0.233997	-0.233961	-0.233848	-0.233646	-0.233327
15.0286	-0.227327	-0.227293	-0.227183	-0.226984	-0.226674
15.1292	-0.220883	-0.220849	-0.220742	-0.220548	-0.220243
15.235	-0.214653	-0.214621	-0.214515	-0.214325	-0.214029
15.3355	-0.208631	-0.2086	-0.208495	-0.20831	-0.208022
15.4361	-0.202807	-0.202776	-0.202675	-0.202494	-0.202211
15.5419	-0.197174	-0.197145	-0.197045	-0.196869	-0.196593
15.6425	-0.191726	-0.191696	-0.191598	-0.191426	-0.191157
15.7483	-0.186452	-0.186423	-0.186329	-0.18616	-0.185898
15.8489	-0.181348	-0.18132	-0.181227	-0.181061	-0.180806
15.9494	-0.176407	-0.176379	-0.176289	-0.176127	-0.175878
16.0552	-0.171623	-0.171597	-0.171508	-0.17135	-0.171106
16.1558	-0.16699	-0.166964	-0.166876	-0.166722	-0.166484
16.2563	-0.162502	-0.162476	-0.162392	-0.16224	-0.162009
16.3622	-0.158155	-0.158129	-0.158046	-0.157899	-0.157671
16.4627	-0.153941	-0.153917	-0.153834	-0.153691	-0.153469
16.5685	-0.149857	-0.149832	-0.149753	-0.149612	-0.149396
16.6691	-0.145898	-0.145874	-0.145796	-0.145658	-0.145447
16.7696	-0.14206	-0.142035	-0.141958	-0.141824	-0.141617
16.8755	-0.138336	-0.138314	-0.138239	-0.138107	-0.137906
16.976	-0.134725	-0.134703	-0.13463	-0.134501	-0.134304
17.0818	-0.131222	-0.131201	-0.131129	-0.131002	-0.130811
17.1824	-0.127822	-0.127801	-0.127731	-0.127608	-0.12742
17.2829	-0.124524	-0.124503	-0.124434	-0.124314	-0.124131
17.3888	-0.121321	-0.121299	-0.121233	-0.121116	-0.120937
17.4893	-0.118212	-0.118191	-0.118125	-0.11801	-0.117835
17.5898	-0.115193	-0.115174	-0.115109	-0.114994	-0.114824
17.6957	-0.112259	-0.11224	-0.112177	-0.112067	-0.1119
17.7962	-0.109412	-0.109393	-0.109331	-0.109222	-0.109061
17.9021	-0.106645	-0.106626	-0.106565	-0.10646	-0.106301
18.0026	-0.103956	-0.103939	-0.10388	-0.103775	-0.10362
18.1031	-0.101344	-0.101326	-0.101268	-0.101167	-0.101015
18.209	-0.0988037	-0.0987866	-0.09873	-0.0986315	-0.0984818
18.3095	-0.0963358	-0.0963189	-0.0962631	-0.0961657	-0.0960212
18.4154	-0.0939349	-0.0939178	-0.0938634	-0.0937683	-0.0936266
18.5159	-0.0916002	-0.0915839	-0.0915312	-0.0914377	-0.0912995
18.6164	-0.0893306	-0.0893139	-0.0892623	-0.0891703	-0.0890351
18.7223	-0.0871213	-0.0871057	-0.0870546	-0.0869666	-0.0868334
18.8228	-0.0849725	-0.0849571	-0.0849075	-0.0848197	-0.0846909
18.9287	-0.0828828	-0.0828668	-0.0828181	-0.0827325	-0.0826061
19.0292	-0.0808476	-0.0808316	-0.080784	-0.0807004	-0.0805777
19.1297	-0.0788673	-0.0788528	-0.0788057	-0.0787238	-0.0786026
19.2356	-0.0769381	-0.0769239	-0.0768785	-0.0767981	-0.0766794
19.3361	-0.0750621	-0.0750485	-0.0750028	-0.0749247	-0.074809
19.4367	-0.0732341	-0.0732211	-0.0731759	-0.0730987	-0.0729858
19.5425	-0.071453	-0.0714394	-0.0713964	-0.0713211	-0.0712096
19.6431	-0.0697196	-0.0697071	-0.0696641	-0.0695908	-0.0694817
19.7489	-0.0680312	-0.0680185	-0.0679761	-0.0679048	-0.0677983
19.8494	-0.0663851	-0.0663726	-0.0663312	-0.0662603	-0.0661567
19.95	-0.0647817	-0.0647696	-0.0647299	-0.0646605	-0.0645587
20.0558	-0.0632188	-0.0632058	-0.0631668	-0.0630994	-0.063

R(Å) \ θ(º)	180	170	160	150	140
20.1564	-0.0616954	-0.061684	-0.0616454	-0.0615791	-0.0614821
20.2622	-0.0602107	-0.0601984	-0.0601613	-0.0600963	-0.0600002
20.3627	-0.0587624	-0.0587516	-0.0587143	-0.0586511	-0.0585578
20.4633	-0.0573507	-0.0573397	-0.0573044	-0.0572414	-0.0571501
20.5691	-0.0559746	-0.0559647	-0.0559285	-0.0558679	-0.0557784
20.6697	-0.0546329	-0.0546217	-0.0545875	-0.0545272	-0.0544394
20.7755	-0.0533227	-0.0533124	-0.0532781	-0.0532195	-0.0531335
20.876	-0.0520449	-0.0520352	-0.0520028	-0.0519437	-0.0518599
20.9766	-0.0507985	-0.0507891	-0.0507564	-0.0507	-0.0506174
21.0824	-0.049583	-0.0495732	-0.0495403	-0.0494854	-0.0494051
21.183	-0.0483963	-0.0483854	-0.0483549	-0.0483007	-0.0482212
21.2835	-0.0472384	-0.0472279	-0.0471974	-0.0471445	-0.0470661
21.3893	-0.0461072	-0.0460976	-0.0460671	-0.0460144	-0.0459393
21.4899	-0.0450028	-0.0449943	-0.0449644	-0.0449133	-0.0448382
21.5957	-0.0439254	-0.0439162	-0.0438864	-0.043837	-0.0437641
21.6963	-0.042872	-0.042863	-0.0428349	-0.0427855	-0.0427142
21.7968	-0.0418453	-0.0418367	-0.0418084	-0.0417603	-0.0416903
21.9026	-0.0408409	-0.0408324	-0.0408056	-0.0407578	-0.0406895
22.0032	-0.0398608	-0.039852	-0.0398245	-0.0397782	-0.0397109
22.109	-0.0389028	-0.0388938	-0.0388681	-0.0388224	-0.0387568
22.2096	-0.0379665	-0.0379592	-0.0379333	-0.0378868	-0.0378223
22.3101	-0.0370521	-0.0370442	-0.0370183	-0.0369747	-0.0369123
22.4159	-0.0361591	-0.036151	-0.0361266	-0.0360825	-0.0360202
22.5165	-0.0352841	-0.0352768	-0.0352525	-0.0352099	-0.03515
22.617	-0.0344321	-0.034425	-0.0344002	-0.034359	-0.0342984
22.7229	-0.0335976	-0.0335902	-0.0335664	-0.0335254	-0.0334672
22.8234	-0.0327816	-0.0327746	-0.0327511	-0.0327105	-0.032653
22.9292	-0.0319845	-0.0319768	-0.0319544	-0.0319156	-0.0318583
23.0298	-0.0312042	-0.0311977	-0.0311755	-0.0311364	-0.0310813
23.1303	-0.030442	-0.0304348	-0.0304119	-0.0303755	-0.0303204
23.2362	-0.0296949	-0.0296881	-0.0296666	-0.0296304	-0.029576
23.3367	-0.0289658	-0.0289588	-0.0289379	-0.0289015	-0.0288495
23.4425	-0.0282527	-0.0282462	-0.0282244	-0.0281891	-0.028138
23.5431	-0.0275535	-0.0275474	-0.0275274	-0.0274923	-0.027442
23.6436	-0.0268716	-0.026865	-0.026845	-0.0268106	-0.0267603
23.7495	-0.0262018	-0.0261963	-0.0261763	-0.0261429	-0.0260942
23.85	-0.025549	-0.0255431	-0.0255225	-0.0254904	-0.0254419
23.9558	-0.024908	-0.0249031	-0.024884	-0.0248516	-0.0248048
24.0564	-0.0242818	-0.0242752	-0.0242563	-0.0242256	-0.0241797
24.1569	-0.0236688	-0.0236638	-0.0236447	-0.0236128	-0.0235678
24.2628	-0.0230688	-0.0230624	-0.0230442	-0.0230143	-0.0229698
24.3633	-0.0224801	-0.0224744	-0.0224569	-0.0224261	-0.0223825
24.4639	-0.0219051	-0.0218994	-0.0218816	-0.0218524	-0.0218088
24.5697	-0.0213411	-0.0213362	-0.0213198	-0.0212893	-0.0212482
24.6702	-0.0207893	-0.020784	-0.0207667	-0.0207386	-0.0206967
24.7761	-0.0202487	-0.0202435	-0.0202263	-0.0201996	-0.0201594
24.8766	-0.0197207	-0.0197139	-0.0196983	-0.0196706	-0.0196311
24.9772	-0.0192023	-0.0191957	-0.0191794	-0.0191525	-0.0191136
25.083	-0.0186929	-0.018688	-0.0186727	-0.0186457	-0.0186077
25.1835	-0.0181953	-0.0181907	-0.0181751	-0.018149	-0.0181106
25.2894	-0.0177081	-0.0177039	-0.0176886	-0.0176622	-0.0176253
25.3899	-0.0172292	-0.0172246	-0.0172094	-0.0171853	-0.0171484
25.4905	-0.0167619	-0.0167564	-0.0167424	-0.0167178	-0.0166818
25.5963	-0.0163026	-0.0162984	-0.0162839	-0.0162591	-0.0162249
25.6968	-0.0158522	-0.0158491	-0.0158349	-0.0158096	-0.0157758
25.8027	-0.0154117	-0.0154078	-0.0153937	-0.01537	-0.0153364
25.9032	-0.0149796	-0.0149756	-0.0149618	-0.0149394	-0.0149052
26.0038	-0.0145573	-0.0145525	-0.0145387	-0.0145156	-0.0144831
26.1096	-0.0141412	-0.0141366	-0.0141232	-0.0141019	-0.0140694
26.2101	-0.0137338	-0.0137292	-0.0137165	-0.0136952	-0.0136636

R(Å) \ θ(º)	180	170	160	150	140
26.3107	-0.0133344	-0.0133307	-0.0133179	-0.0132967	-0.0132659
26.4165	-0.0129431	-0.0129389	-0.0129268	-0.0129047	-0.0128755
26.5171	-0.0125588	-0.0125548	-0.0125428	-0.0125221	-0.0124914
26.6229	-0.0121817	-0.0121786	-0.0121668	-0.0121453	-0.0121161
26.7234	0.0118126	-0.0118084	-0.0117968	-0.011777	-0.0117485
26.824	-0.0114509	-0.0114465	-0.0114346	-0.0114144	-0.0113857
26.9298	-0.0110942	-0.0110903	-0.0110789	-0.0110606	-0.0110321
27.0304	-0.0107459	-0.0107415	-0.0107303	-0.0107115	-0.010684
27.1362	-0.0104033	-0.0104002	-0.010389	-0.0103697	-0.0103427
27.2367	-0.0100677	-0.0100636	-0.0100537	-0.010035	-0.0100072
27.3373	-0.00973765	-0.00973436	-0.0097236	-0.00970517	-0.00967883
27.4431	-0.00941414	-0.00941129	-0.00940032	-0.00938188	-0.00935642
27.5437	-0.009097	-0.00909415	-0.00908318	-0.0090654	-0.00904016
27.6442	-0.00878623	-0.00878293	-0.00877196	-0.00875484	-0.00872982
27.75	-0.00847918	-0.00847633	-0.00846601	-0.00844977	-0.00842453
27.8506	-0.00817872	-0.00817719	-0.00816621	-0.00814931	-0.00812583
27.9564	-0.00788506	-0.00788221	-0.0078719	-0.007855	-0.00783195
28.057	-0.00759624	-0.00759272	-0.00758329	-0.00756617	-0.00754334
28.1575	-0.0073107	-0.00730807	-0.00729907	-0.00728261	-0.00725978
28.2633	-0.00703197	-0.00702977	-0.00702033	-0.00700453	-0.00698193
28.3639	-0.0067585	-0.00675653	-0.00674577	-0.00673173	-0.00670868
28.4697	-0.00648943	-0.00648635	-0.00647801	-0.00646287	-0.00644114
28.5703	-0.00622496	-0.00622232	-0.00621442	-0.00619862	-0.00617777
28.6708	-0.00596532	-0.00596247	-0.00595588	-0.00593964	-0.00591813
28.7766	-0.00571073	-0.00570832	-0.005698	-0.00568395	-0.00566354
28.8772	-0.00545943	-0.00545789	-0.0054489	-0.00543485	-0.00541378
28.983	-0.00521406	-0.00521208	-0.00520352	-0.00518904	-0.00516972
29.0836	-0.00497264	-0.00496978	-0.00496232	-0.00494849	-0.00492896
29.1841	-0.0047356	-0.00473319	-0.00472617	-0.00471168	-0.00469281
29.2899	-0.00450186	-0.00449923	-0.00449177	-0.00447772	-0.00445907
29.3905	-0.00427251	-0.00427076	-0.00426242	-0.00424881	-0.00422972
29.491	-0.00404777	-0.00404514	-0.00403702	-0.0040256	-0.00400629
29.5969	-0.0038272	-0.00382478	-0.00381644	-0.00380371	-0.00378638
29.6974	-0.0036097	-0.00360663	-0.0035996	-0.00358621	-0.00356931
29.8032	-0.00339615	-0.00339373	-0.00338649	-0.00337464	-0.0033562
29.9038	-0.00318633	-0.00318348	-0.00317668	-0.00316504	-0.00314749
30.0043	0.00297915	-0.00297651	-0.00296905	-0.00295698	-0.00294052
30.1102	0.00277613	-0.00277372	-0.0027667	-0.00275441	-0.00273882
30.2107	-0.00257641	-0.00257356	-0.00256697	-0.00255622	-0.00253888
30.3166	-0.00238108	-0.00237932	-0.00237142	-0.00235869	-0.00234377
30.4171	-0.00218794	-0.00218619	-0.00217872	-0.00216775	-0.00215129
30.5176	-0.00199722	-0.00199568	-0.00198954	-0.00197725	-0.0019621
30.6235	-0.00181132	-0.00180913	-0.00180276	-0.00179179	-0.00177643
30.724	-0.00162872	-0.00162609	-0.00161863	-0.00160809	-0.0015947
30.8299	-0.00144722	-0.00144568	-0.00143866	-0.00142922	-0.00141386
30.9304	-0.00127054	-0.00126834	-0.0012622	-0.00125188	-0.0012374
31.0309	-0.00109496	-0.0010921	-0.00108662	-0.00107586	-0.00106226
31.1368	-0.000919379	-0.000916525	-0.000909941	-0.000900505	-0.000891505
31.2373	-0.000725363	-0.000722291	-0.000717461	-0.000706707	-0.000722509
31.3379	-0.000391762	-0.000388689	-0.00038408	-0.000373326	-0.000558343
31.4437	0.000841247	0.000845197	0.000846295	0.000857488	-0.000395273
31.5442	0.00784776	0.00786751	0.00783327	0.00786246	-0.000235716
31.6501	0.0518074	0.0519281	0.051679	0.0518125	-0.0000821
31.7506	0.331699	0.332463	0.330841	0.331644	-0.000319774

CH^- + He PES

Method: MRCI. CBS extrapolation (90º – 130º)

R(Å) \ θ(º)	130	120	110	100	90
1.89445	4629.51	4786.3	4811.64	4718.54	4546.55
2.00029	3267.72	3395.37	3426.66	3377.63	3300.37
2.10083	2279.22	2382.54	2412.17	2377.88	2314.62
2.20138	1566.01	1649.59	1676.35	1651.89	1600.54
2.30721	1055.97	1123.7	1147.39	1130.47	1090.06
2.40776	695.128	749.899	770.559	759.346	728.317
2.5083	442.268	486.111	503.683	496.418	472.899
2.61413	266.235	300.809	315.305	310.77	293.048
2.71468	144.322	171.148	182.811	180.018	166.78
2.82051	60.3783	81.0178	90.3289	88.7473	79.0213
2.92106	3.32987	19.303	26.8126	26.1211	19.1412
3.0216	-34.2193	-21.8204	-15.6806	-15.7402	-20.6268
3.12744	-57.6914	-47.9646	-42.9218	-42.5912	-45.9488
3.22798	-71.1448	-63.5117	-59.4028	-58.8859	-61.1683
3.33382	-77.6992	-71.7559	-68.4011	-67.8474	-69.4075
3.43436	-79.6325	-75.0568	-72.4846	-71.9601	-73.0692
3.5349	-78.5865	-75.1104	-73.1499	-72.773	-73.5585
3.64074	-75.6964	-73.1625	-71.7144	-71.473	-72.0862
3.74128	-71.9281	-69.9666	-68.9912	-68.8819	-69.3968
3.84712	-67.7203	-66.2596	-65.5925	-65.598	-66.051
3.94766	-63.4107	-62.3098	-61.8216	-61.9614	-62.3668
4.0482	-59.1905	-58.3467	-58.0279	-58.2096	-58.5673
4.15404	-55.1618	-54.4984	-54.2962	-54.4934	-54.7954
4.25458	-51.3579	-50.8254	-50.6951	-50.8856	-51.1289
4.35513	-47.2496	-47.3502	-47.2681	-47.484	-47.6656
4.46096	-43.7662	-43.5759	-43.6561	-43.8924	-44.1089
4.56151	-40.8281	-40.623	-40.6674	-40.8441	-40.9811
4.66734	-37.7801	-37.6424	-37.7154	-37.8849	-37.9881
4.76788	-34.9117	-34.8307	-34.9269	-35.0929	-35.1749
4.86843	-32.2215	-32.1897	-32.3088	-32.4755	-32.5428
4.97426	-29.714	-29.7234	-29.8617	-30.0311	-30.0909
5.07481	-27.4034	-27.4471	-27.5917	-27.7633	-27.8192
5.18064	-25.3568	-25.4282	-25.5119	-25.6725	-25.7264
5.28119	-23.406	-23.4987	-23.6876	-23.7584	-23.8107
5.38173	-20.9753	-21.7492	-21.9459	-22.0176	-22.0685
5.48757	-19.7093	-19.5656	-19.8585	-20.0083	-20.1268
5.58811	-17.9264	-18.4429	-18.6794	-18.8062	-18.893
5.69394	-16.7966	-16.7123	-16.8358	-17.5167	-17.6033
5.79449	-15.4687	-15.7685	-15.9285	-16.1289	-16.1923
5.89503	-14.3463	-14.5534	-14.7976	-14.9865	-15.0441
6.00087	-13.3248	-13.5283	-13.7603	-13.936	-13.9869
6.10141	-12.3925	-12.5891	-12.8072	-12.9687	-13.0124
6.20195	-11.5394	-11.7268	-11.9288	-12.0754	-12.1123
6.30779	-10.7562	-10.9344	-11.1177	-11.2491	-11.2789
6.40833	-10.0373	-10.2155	-10.3669	-10.483	-10.5057
6.51417	-9.38544	-9.62789	-9.67193	-9.77144	-9.7872
6.61471	-8.85955	-8.99548	-9.03819	-9.11116	-9.11836
6.71526	-8.2816	-8.40464	-8.52596	-8.50899	-8.49571
6.82109	-7.72964	-7.83954	-7.95952	-8.02545	-7.91678
6.92164	-7.13577	-7.23316	-7.4173	-7.4877	-7.39007
7.02747	-6.66805	-6.75353	-6.83247	-6.98412	-6.9773
7.12801	-6.23058	-6.3057	-6.37381	-6.50151	-6.50427
7.22856	-5.82221	-5.88844	-5.94574	-5.97368	-6.05125
7.33439	-5.44317	-5.49951	-5.54692	-5.56884	-5.55197
7.43494	-5.09521	-5.13638	-5.17621	-5.19281	-5.17381
7.53548	-4.81414	-4.80592	-4.83276	-4.84228	-4.82331
7.64132	-4.49791	-4.54148	-4.52008	-4.52066	-4.49987
7.74186	-4.16093	-4.2423	-4.27382	-4.22872	-4.1999

R(Å) \ θ(º)	130	120	110	100	90
7.84769	-3.89535	-3.92074	-3.99117	-4.00176	-3.92362
7.94824	-3.64896	-3.67004	-3.6853	-3.73679	-3.67529
8.04878	-3.42092	-3.43824	-3.45004	-3.44849	-3.48896
8.15462	-3.2099	-3.22399	-3.23293	-3.2302	-3.2626
8.25516	-3.01469	-3.02599	-3.03254	-3.02822	-3.01029
8.361	-2.8342	-2.8432	-2.8477	-2.84262	-2.82548
8.46154	-2.66748	-2.67519	-2.67724	-2.67148	-2.65483
8.56208	-2.51461	-2.51931	-2.52005	-2.51377	-2.49791
8.66792	-2.36944	-2.37357	-2.37377	-2.36713	-2.35209
8.76846	-2.23791	-2.24085	-2.24013	-2.23331	-2.21903
8.8743	-2.11624	-2.11818	-2.11675	-2.10983	-2.09628
8.97484	-2.0036	-2.00472	-2.00271	-1.99577	-1.98305
9.07539	-1.89922	-1.89967	-1.89721	-1.89031	-1.87815
9.18122	-1.80239	-1.80229	-1.79949	-1.79268	-1.78117
9.28176	-1.71259	-1.71201	-1.70889	-1.70225	-1.69132
9.38231	-1.62886	-1.62792	-1.62468	-1.61812	-1.60776
9.48814	-1.55087	-1.54965	-1.54627	-1.53988	-1.53006
9.58869	-1.4781	-1.47666	-1.47321	-1.467	-1.45768
9.69452	-1.41009	-1.40848	-1.405	-1.39898	-1.39014
9.79507	-1.34644	-1.3447	-1.34121	-1.33539	-1.327
9.89561	-1.28677	-1.28494	-1.28147	-1.27585	-1.26787
10.0014	-1.23073	-1.22887	-1.22541	-1.21998	-1.21239
10.102	-1.17803	-1.1761	-1.17277	-1.1675	-1.16028
10.2078	-1.12844	-1.12646	-1.12314	-1.11811	-1.11122
10.3084	-1.08158	-1.07963	-1.07639	-1.07154	-1.06498
10.4089	-1.03735	-1.0354	-1.03224	-1.02763	-1.02131
10.5147	-0.99553	-0.993598	-0.990511	-0.986019	-0.980041
10.6153	-0.955923	-0.954015	-0.951013	-0.946695	-0.940978
10.7211	-0.918374	-0.91649	-0.913586	-0.909424	-0.903962
10.8217	-0.882732	-0.88089	-0.878067	-0.874063	-0.868838
10.9222	-0.848876	-0.847068	-0.844329	-0.840478	-0.835477
11.028	-0.816678	-0.814908	-0.812255	-0.808548	-0.80376
11.1286	-0.786032	-0.784315	-0.781734	-0.778165	-0.773573
11.2291	-0.756837	-0.755151	-0.752663	-0.749227	-0.744826
11.335	-0.729007	-0.727362	-0.724954	-0.721647	-0.717427
11.4355	-0.702456	-0.700856	-0.698525	-0.69534	-0.691292
11.5414	-0.677113	-0.675555	-0.673298	-0.67023	-0.666346
11.6419	-0.652904	-0.651388	-0.649204	-0.646251	-0.642519
11.7424	-0.629767	-0.628293	-0.62618	-0.623333	-0.61975
11.8483	-0.607642	-0.606208	-0.604164	-0.601422	-0.597978
11.9488	-0.586473	-0.585079	-0.583103	-0.580459	-0.577148
12.0547	-0.56621	-0.564856	-0.562943	-0.560393	-0.557211
12.1552	-0.546804	-0.545488	-0.543637	-0.541179	-0.538119
12.2557	-0.528212	-0.526934	-0.525142	-0.522772	-0.519826
12.3616	-0.510392	-0.509148	-0.507415	-0.505124	-0.502289
12.4621	-0.493301	-0.492096	-0.490418	-0.488212	-0.485482
12.5627	-0.476911	-0.475739	-0.474116	-0.471985	-0.469358
12.6685	-0.461182	-0.460042	-0.458471	-0.456416	-0.453885
12.769	-0.44608	-0.444974	-0.443454	-0.44147	-0.43903
12.8749	-0.431579	-0.430506	-0.429033	-0.427116	-0.424777
12.9754	-0.417649	-0.416617	-0.415192	-0.413342	-0.411076
13.076	-0.404273	-0.403262	-0.401882	-0.400096	-0.397913
13.1818	-0.391409	-0.390424	-0.389085	-0.387356	-0.385248
13.2823	-0.379019	-0.378063	-0.376767	-0.375098	-0.373066
13.3882	-0.367116	-0.366187	-0.364932	-0.363318	-0.361357
13.4887	-0.355662	-0.35476	-0.353545	-0.351984	-0.350091
13.5893	-0.34464	-0.343763	-0.342585	-0.341076	-0.33925
13.6951	-0.334027	-0.333176	-0.332034	-0.330573	-0.32881
13.7956	-0.323807	-0.322979	-0.321872	-0.32046	-0.318757
13.9015	-0.313961	-0.313157	-0.312083	-0.310717	-0.309072

R(Å) \ θ(º)	130	120	110	100	90
14.002	-0.304474	-0.303692	-0.302651	-0.301329	-0.29974
14.1026	-0.29533	-0.29457	-0.29356	-0.292281	-0.290745
14.2084	-0.286514	-0.285775	-0.284796	-0.283557	-0.282073
14.3089	-0.278011	-0.277293	-0.276343	-0.275145	-0.27371
14.4095	-0.26981	-0.269112	-0.26819	-0.267028	-0.265641
14.5153	-0.261896	-0.261218	-0.260323	-0.259199	-0.257856
14.6159	-0.25426	-0.253599	-0.252731	-0.251641	-0.250344
14.7217	-0.246885	-0.246244	-0.245402	-0.244347	-0.243089
14.8222	-0.239767	-0.239143	-0.238325	-0.237302	-0.236086
14.9228	-0.232891	-0.232283	-0.231488	-0.230498	-0.229322
15.0286	-0.226248	-0.225655	-0.224885	-0.223923	-0.222785
15.1292	-0.219828	-0.219253	-0.218504	-0.217573	-0.21647
15.235	-0.213625	-0.213064	-0.212338	-0.211434	-0.210366
15.3355	-0.207627	-0.207082	-0.206376	-0.205499	-0.204464
15.4361	-0.201826	-0.201296	-0.20061	-0.19976	-0.198758
15.5419	-0.196217	-0.195701	-0.195035	-0.194211	-0.193238
15.6425	-0.19079	-0.190289	-0.18964	-0.18884	-0.187899
15.7483	-0.18554	-0.185052	-0.184421	-0.183644	-0.182731
15.8489	-0.180457	-0.179982	-0.179368	-0.178616	-0.177732
15.9494	-0.175537	-0.175074	-0.174479	-0.173747	-0.172889
16.0552	-0.170774	-0.170323	-0.169744	-0.169033	-0.168201
16.1558	-0.16616	-0.165722	-0.165158	-0.164468	-0.16366
16.2563	-0.161692	-0.161265	-0.160717	-0.160047	-0.159263
16.3622	-0.157364	-0.156946	-0.156414	-0.155762	-0.155002
16.4627	-0.153169	-0.152762	-0.152245	-0.151612	-0.150873
16.5685	-0.149103	-0.148707	-0.148202	-0.147588	-0.146872
16.6691	-0.145161	-0.144776	-0.144285	-0.143687	-0.142991
16.7696	-0.141339	-0.140963	-0.140486	-0.139905	-0.139229
16.8755	-0.137633	-0.137267	-0.136802	-0.136238	-0.135581
16.976	-0.134038	-0.133681	-0.133229	-0.132679	-0.132042
17.0818	-0.130551	-0.130203	-0.129763	-0.129229	-0.128608
17.1824	-0.127166	-0.126827	-0.126399	-0.125879	-0.125277
17.2829	-0.123882	-0.123552	-0.123133	-0.122628	-0.122044
17.3888	-0.120695	-0.120372	-0.119965	-0.119474	-0.118905
17.4893	-0.1176	-0.117286	-0.116889	-0.11641	-0.115858
17.5898	-0.114593	-0.114286	-0.113899	-0.113432	-0.112895
17.6957	-0.111674	-0.111375	-0.110998	-0.110545	-0.110022
17.7962	-0.10884	-0.108547	-0.10818	-0.107738	-0.10723
17.9021	-0.106085	-0.105801	-0.105443	-0.105013	-0.104518
18.0026	-0.103409	-0.103131	-0.102782	-0.102364	-0.101883
18.1031	-0.100809	-0.100538	-0.100198	-0.0997901	-0.0993217
18.209	-0.0982805	-0.0980163	-0.0976862	-0.0972881	-0.0968326
18.3095	-0.0958244	-0.0955663	-0.0952434	-0.0948558	-0.0944125
18.4154	-0.0934356	-0.093183	-0.0928678	-0.0924912	-0.0920593
18.5159	-0.0911125	-0.0908658	-0.0905583	-0.0901909	-0.0897708
18.6164	-0.0888514	-0.088612	-0.088312	-0.087954	-0.0875443
18.7223	-0.0866543	-0.0864186	-0.0861271	-0.0857781	-0.0853796
18.8228	-0.0845157	-0.0842864	-0.0840011	-0.0836618	-0.0832733
18.9287	-0.0824345	-0.0822104	-0.0819332	-0.0816009	-0.0812223
19.0292	-0.0804089	-0.080191	-0.0799195	-0.0795973	-0.0792288
19.1297	-0.0784383	-0.0782245	-0.0779598	-0.0776453	-0.0772858
19.2356	-0.07652	-0.0763107	-0.0760534	-0.0757453	-0.0753961
19.3361	-0.0746508	-0.0744473	-0.0741952	-0.0738962	-0.0735558
19.4367	-0.0728316	-0.0726325	-0.0723865	-0.0720943	-0.0717621
19.5425	-0.0710613	-0.0708662	-0.0706252	-0.070341	-0.0700168
19.6431	-0.0693353	-0.0691455	-0.0689106	-0.0686334	-0.0683167
19.7489	-0.0676546	-0.0674685	-0.0672387	-0.0669679	-0.0666597
19.8494	-0.0660167	-0.0658354	-0.0656111	-0.0653473	-0.065047
19.95	-0.0644215	-0.0642442	-0.064024	-0.0637664	-0.0634732
20.0558	-0.0628663	-0.0626918	-0.0624789	-0.0622265	-0.061941

R(Å) \ θ(º)	130	120	110	100	90
20.1564	-0.0613506	-0.0611812	-0.0609716	-0.0607256	-0.0604462
20.2622	-0.0598722	-0.0597063	-0.0595022	-0.0592628	-0.058991
20.3627	-0.0584316	-0.0582701	-0.0580706	-0.0578364	-0.0575708
20.4633	-0.0570263	-0.0568685	-0.0566743	-0.0564449	-0.0561855
20.5691	-0.0556577	-0.0555021	-0.055312	-0.0550884	-0.0548353
20.6697	-0.0543204	-0.0541688	-0.0539829	-0.0537651	-0.0535182
20.7755	-0.0530178	-0.0528697	-0.0526884	-0.0524753	-0.0522341
20.876	-0.0517479	-0.051602	-0.0514238	-0.0512155	-0.0509802
20.9766	-0.0505062	-0.0503644	-0.0501914	-0.049988	-0.0497578
21.0824	-0.0492969	-0.0491581	-0.0489881	-0.0487892	-0.0485649
21.183	-0.0481143	-0.0479789	-0.0478132	-0.0476188	-0.0474002
21.2835	-0.0469621	-0.0468289	-0.0466671	-0.0464779	-0.0462631
21.3893	-0.0458382	-0.0457071	-0.045548	-0.0453617	-0.0451525
21.4899	-0.044739	-0.0446111	-0.0444557	-0.0442744	-0.0440703
21.5957	-0.0436658	-0.0435422	-0.0433895	-0.0432119	-0.0430122
21.6963	-0.0426187	-0.0424971	-0.0423472	-0.0421751	-0.0419791
21.7968	-0.0415964	-0.0414761	-0.0413306	-0.0411616	-0.0409711
21.9026	-0.0405971	-0.0404795	-0.0403368	-0.0401685	-0.0399817
22.0032	-0.0396211	-0.0395061	-0.0393663	-0.039205	-0.0390226
22.109	-0.0386673	-0.0385547	-0.0384182	-0.0382579	-0.0380804
22.2096	-0.037736	-0.0376263	-0.0374933	-0.0373372	-0.0371643
22.3101	-0.0368265	-0.0367179	-0.0365866	-0.0364345	-0.0362642
22.4159	-0.0359381	-0.0358325	-0.0357041	-0.0355555	-0.0353885
22.5165	-0.0350679	-0.0349634	-0.0348381	-0.0346913	-0.0345277
22.617	-0.0342176	-0.0341165	-0.0339927	-0.0338507	-0.0336902
22.7229	-0.033388	-0.0332886	-0.0331666	-0.0330272	-0.0328705
22.8234	-0.0325762	-0.032479	-0.0323602	-0.0322226	-0.0320703
22.9292	-0.031783	-0.0316862	-0.0315708	-0.0314373	-0.0312879
23.0298	-0.0310072	-0.0309123	-0.0307984	-0.0306674	-0.0305215
23.1303	-0.030248	-0.0301563	-0.0300443	-0.0299164	-0.0297715
23.2362	-0.0295051	-0.029414	-0.0293058	-0.02918	-0.0290396
23.3367	-0.028778	-0.0286913	-0.0285839	-0.0284608	-0.0283243
23.4425	-0.0280686	-0.0279828	-0.0278779	-0.0277578	-0.0276237
23.5431	-0.0273749	-0.0272906	-0.0271872	-0.0270691	-0.0269383
23.6436	-0.0266943	-0.0266111	-0.0265108	-0.0263953	-0.0262667
23.7495	-0.0260297	-0.0259483	-0.0258497	-0.0257363	-0.0256112
23.85	-0.0253792	-0.0252991	-0.0252029	-0.0250917	-0.0249681
23.9558	-0.0247423	-0.0246635	-0.024568	-0.0244593	-0.0243393
24.0564	-0.0241181	-0.0240413	-0.0239482	-0.0238422	-0.0237235
24.1569	-0.0235084	-0.023432	-0.0233411	-0.0232369	-0.0231221
24.2628	-0.022911	-0.022837	-0.0227477	-0.022645	-0.0225324
24.3633	-0.0223261	-0.0222528	-0.022165	-0.0220653	-0.0219549
24.4639	-0.021753	-0.0216808	-0.0215965	-0.021498	-0.0213896
24.5697	-0.0211923	-0.0211229	-0.0210395	-0.0209423	-0.0208354
24.6702	-0.0206427	-0.020574	-0.0204917	-0.0203975	-0.0202937
24.7761	-0.0201048	-0.0200376	-0.019957	-0.0198649	-0.0197628
24.8766	-0.0195789	-0.0195126	-0.0194327	-0.0193421	-0.0192431
24.9772	-0.0190622	-0.0189966	-0.0189194	-0.0188311	-0.0187326
25.083	-0.0185572	-0.0184932	-0.0184168	-0.0183301	-0.0182333
25.1835	-0.0180619	-0.0179998	-0.0179241	-0.0178387	-0.0177458
25.2894	-0.0175773	-0.0175154	-0.0174421	-0.0173574	-0.0172645
25.3899	-0.0171006	-0.0170409	-0.0169682	-0.016887	-0.0167957
25.4905	-0.0166355	-0.016576	-0.0165054	-0.0164253	-0.0163364
25.5963	-0.0161788	-0.0161209	-0.0160519	-0.0159725	-0.015884
25.6968	-0.0157302	-0.0156738	-0.0156055	-0.015528	-0.0154427
25.8027	-0.0152921	-0.0152357	-0.0151686	-0.0150937	-0.015009
25.9032	-0.0148619	-0.0148071	-0.0147417	-0.0146677	-0.014585
26.0038	-0.0144403	-0.0143866	-0.0143216	-0.0142496	-0.0141675
26.1096	-0.0140266	-0.0139735	-0.013911	-0.0138392	-0.0137595
26.2101	-0.0136219	-0.0135697	-0.0135078	-0.0134378	-0.0133596

R(Å) \ θ(º)	130	120	110	100	90
26.3107	-0.0132242	-0.0131733	-0.0131127	-0.0130438	-0.0129674
26.4165	-0.0128336	-0.0127842	-0.0127247	-0.012656	-0.0125825
26.5171	-0.0124519	-0.0124032	-0.0123448	-0.0122772	-0.0122043
26.6229	-0.012077	-0.0120285	-0.0119719	-0.0119069	-0.0118345
26.7234	-0.0117098	-0.0116629	-0.011606	-0.0115422	-0.0114711
26.824	-0.0113484	-0.0113021	-0.0112465	-0.0111838	-0.0111135
26.9298	-0.010995	-0.0109489	-0.0108947	-0.0108326	-0.0107646
27.0304	-0.010648	-0.0106028	-0.0105495	-0.0104889	-0.0104222
27.1362	-0.0103067	-0.0102635	-0.01021	-0.0101509	-0.0100855
27.2367	-0.0099725	-0.00992881	-0.00987745	-0.00981929	-0.00975455
27.3373	-0.009645	-0.00960114	-0.00955132	-0.00949403	-0.00943039
27.4431	-0.0093235	-0.00928092	-0.0092322	-0.00917426	-0.00911259
27.5437	-0.0090079	-0.00896598	-0.00891616	-0.00886151	-0.00880115
27.6442	-0.0086971	-0.00865652	-0.00860735	-0.0085538	-0.00849323
27.75	-0.0083921	-0.00835211	-0.00830514	-0.00825137	-0.00819343
27.8506	-0.008094	-0.00805494	-0.00800907	-0.00795596	-0.00789845
27.9564	-0.0078012	-0.0077626	-0.00771629	-0.00766515	-0.00760897
28.057	-0.0075139	-0.00747421	-0.00742943	-0.00737896	-0.00732255
28.1575	-0.0072302	-0.00719262	-0.00714851	-0.00709803	-0.00704382
28.2633	-0.0069523	-0.00691521	-0.00687219	-0.00682369	-0.00676969
28.3639	-0.0066802	-0.00664437	-0.00660136	-0.00655329	-0.00650106
28.4697	-0.0064128	-0.00637705	-0.00633448	-0.00628707	-0.00623483
28.5703	-0.0061488	-0.00611325	-0.00607242	-0.00602611	-0.00597498
28.6708	-0.0058909	-0.00585712	-0.0058163	-0.00577043	-0.00572017
28.7766	-0.0056372	-0.00560319	-0.00556302	-0.00551803	-0.00546887
28.8772	-0.0053879	-0.0053543	-0.00531546	-0.00527068	-0.00522262
28.983	-0.0051427	-0.00511091	-0.00507184	-0.00502838	-0.00498076
29.0836	-0.0049024	-0.0048708	-0.00483195	-0.00478937	-0.00474285
29.1841	-0.0046671	-0.00463552	-0.00459711	-0.0045552	-0.00450933
29.2899	-0.0044341	-0.00440288	-0.00436579	-0.00432497	-0.00428041
29.3905	-0.0042054	-0.00417594	-0.00413951	-0.00409957	-0.00405479
29.491	-0.0039819	-0.00395208	-0.0039174	-0.0038768	-0.00383312
29.5969	-0.0037625	-0.00373217	-0.00369705	-0.00365842	-0.00361584
29.6974	-0.0035465	-0.0035162	-0.00348306	-0.00344356	-0.00340186
29.8032	-0.0033338	-0.00330529	-0.00327105	-0.00323242	-0.00319094
29.9038	-0.0031244	-0.00309569	-0.00306211	-0.00302458	-0.00298464
30.0043	-0.0029184	-0.0028896	-0.00285822	-0.00282113	-0.00278052
30.1102	-0.0027158	-0.00268856	-0.00265652	-0.00261987	-0.00258124
30.2107	-0.0025185	-0.00249126	-0.00245833	-0.00242322	-0.00238547
30.3166	-0.0023236	-0.00229592	-0.00226476	-0.0022303	-0.00219211
30.4171	-0.0021315	-0.00210454	-0.00207316	-0.00203826	-0.00200161
30.5176	-0.001941	-0.00191601	-0.00188485	-0.00185215	-0.00181418
30.6235	-0.0017558	-0.00173034	-0.00170071	-0.00166735	-0.00163201
30.724	-0.0015734	-0.00154905	-0.0015192	-0.00148738	-0.00145029
30.8299	-0.001395	-0.0013693	-0.00134033	-0.0013096	-0.00127339
30.9304	-0.0012172	-0.00119416	-0.00116475	-0.00113205	-0.00109891
31.0309	-0.0010425	-0.00101836	-0.000991147	-0.00096064	-0.000926403
31.1368	-0.0008685	-0.000845197	-0.000817543	-0.000785939	-0.000752797
31.2373	-0.000674	-0.000650523	-0.000623965	-0.000593459	-0.000561415
31.3379	-0.0003419	-0.000319116	-0.000291462	-0.00026293	-0.000230448
31.4437	0.0008917	0.000913454	0.000942644	0.000972712	0.00100256
31.5442	0.0079055	0.00792655	0.00796847	0.00800117	0.00802399
31.6501	0.0519257	0.0519327	0.052064	0.0521164	0.052096
31.7506	0.3322	0.332132	0.332835	0.333018	0.332722

CH^- + He PES

Method: MRCI. CBS extrapolation (40º – 80º)

R(Å) \ θ(º)	80	70	60	50	40
1.89445	-5715.85	4851.06	5489.78	6692.05	8644.79
2.00029	3276.57	3406.27	3813.74	4648.91	6041.43
2.10083	2280.34	2349.85	2616.43	3191.5	4172.11
2.20138	1564.39	1598.54	1770.67	2161.82	2842.5
2.30721	1056.51	1069.59	1177.69	1439.35	1904.62
2.40776	698.83	699.438	764.208	935.652	1249.29
2.5083	447.782	441.34	477.409	587.249	796.569
2.61413	272.031	262.148	280.134	348.927	487.639
2.71468	149.662	138.821	146.215	188.226	279.39
2.82051	65.4899	55.2318	56.9205	81.7197	140.787
2.92106	8.77218	-0.141939	-1.25749	12.6559	49.9662
3.0216	-28.3189	-35.6674	-38.0064	-30.8084	-8.19339
3.12744	-51.566	-57.4237	-60.1873	-56.9988	-44.0684
3.22798	-65.2128	-69.8379	-72.6373	-71.7198	-64.8702
3.33382	-72.3747	-75.8212	-78.4868	-78.9532	-75.666
3.43436	-75.0872	-77.7116	-80.1326	-81.3501	-80.1392
3.5349	-75.0097	-76.9569	-79.0607	-80.6696	-80.6114
3.64074	-73.1511	-74.5791	-76.322	-78.0525	-78.671
3.74128	-70.2192	-71.2608	-72.6321	-74.2678	-75.275
3.84712	-66.6846	-67.4387	-68.4662	-69.852	-71.0097
3.94766	-62.8557	-63.3922	-64.1237	-65.1837	-66.2812
4.0482	-58.933	-59.2886	-59.7856	-60.5211	-61.4059
4.15404	-55.0399	-55.25	-55.5594	-56.0185	-56.6141
4.25458	-51.2662	-51.339	-51.5016	-51.7653	-52.0857
4.35513	-47.6549	-47.6014	-47.6355	-47.7327	-47.7336
4.46096	-44.1455	-44.0391	-44.0009	-44.0687	-44.0251
4.56151	-40.9207	-40.7159	-40.5849	-40.6191	-40.5566
4.66734	-37.8783	-37.6138	-37.4076	-37.4147	-37.3885
4.76788	-35.036	-34.7367	-34.4721	-34.4479	-34.4812
4.86843	-32.3902	-32.0796	-31.7768	-31.7168	-31.8022
4.97426	-29.9375	-29.6362	-29.3151	-29.2169	-29.3334
5.07481	-27.6728	-27.3946	-27.0756	-26.9446	-27.0657
5.18064	-25.5931	-25.3459	-25.0465	-24.8906	-24.9969
5.28119	-23.6923	-23.4779	-23.2093	-23.0398	-22.9625
5.38173	-21.9636	-21.5405	-21.358	-21.2244	-20.8911
5.48757	-20.0998	-20.0085	-19.9807	-19.3258	-19.6034
5.58811	-18.8492	-18.7555	-18.068	-18.2328	-18.1707
5.69394	-16.9943	-16.8743	-17.108	-16.9444	-16.8692
5.79449	-16.1329	-16.028	-15.9	-15.7589	-15.6777
5.89503	-14.9874	-14.8914	-14.7829	-14.6634	-14.5837
6.00087	-13.9328	-13.8425	-13.748	-13.6469	-13.5734
6.10141	-12.9601	-12.873	-12.7877	-12.7017	-12.635
6.20195	-12.0608	-11.9754	-11.8958	-11.8207	-11.7603
6.30779	-11.2278	-11.1432	-11.0662	-10.9986	-10.9443
6.40833	-10.4546	-10.3705	-10.2946	-10.231	-10.1811
6.51417	-9.73623	-9.65229	-9.57616	-9.51451	-9.46739
6.61471	-9.06749	-8.98377	-8.9072	-8.84625	-8.80041
6.71526	-8.4443	-8.36095	-8.28457	-8.22333	-8.177
6.82109	-7.86308	-7.78168	-7.70492	-7.64325	-7.59777
6.92164	-7.32177	-7.24229	-7.16596	-7.10359	-7.05806
7.02747	-6.81567	-6.73996	-6.66475	-6.60281	-6.55663
7.12801	-6.34728	-6.27293	-6.19913	-6.13821	-6.09227
7.22856	-5.91042	-5.83889	-5.76803	-5.7073	-5.66164
7.33439	-5.50458	-5.43634	-5.36742	-5.30879	-5.26389
7.43494	-5.12854	-5.06324	-4.99706	-4.94028	-4.89678
7.53548	-4.78034	-4.7182	-4.65538	-4.60071	-4.55788
7.64132	-4.45796	-4.39903	-4.33993	-4.28697	-4.24588
7.74186	-4.15924	-4.10424	-4.04817	-3.99817	-3.95879

R(Å) \ θ(º)	80	70	60	50	40
7.84769	-3.88369	-3.83221	-3.77954	-3.73255	-3.69471
7.94824	-3.62943	-3.58146	-3.53184	-3.48798	-3.45204
8.04878	-3.39495	-3.35043	-3.30393	-3.2627	-3.22936
8.15462	-3.17898	-3.13761	-3.09461	-3.05587	-3.02464
8.25516	-2.98011	-2.94203	-2.90191	-2.8658	-2.83626
8.361	-2.79728	-2.76154	-2.72468	-2.69104	-2.66345
8.46154	-2.62846	-2.59569	-2.56161	-2.52909	-2.50347
8.56208	-2.47331	-2.44176	-2.41033	-2.38155	-2.35773
8.66792	-2.32928	-2.30137	-2.27238	-2.24574	-2.22364
8.76846	-2.19774	-2.17201	-2.14525	-2.12061	-2.1001
8.8743	-2.07639	-2.05265	-2.028	-2.00522	-1.9862
8.97484	-1.96444	-1.94255	-1.91971	-1.89865	-1.88101
9.07539	-1.86085	-1.84059	-1.81957	-1.80007	-1.78369
9.18122	-1.76499	-1.74625	-1.7268	-1.70871	-1.69348
9.28176	-1.67612	-1.65873	-1.64071	-1.62393	-1.60977
9.38231	-1.59358	-1.57743	-1.5607	-1.54514	-1.53192
9.48814	-1.51677	-1.50174	-1.48624	-1.47176	-1.45947
9.58869	-1.44521	-1.4312	-1.41681	-1.40332	-1.39184
9.69452	-1.37842	-1.36534	-1.35194	-1.33935	-1.32863
9.79507	-1.31597	-1.30373	-1.29124	-1.27943	-1.26936
9.89561	-1.25748	-1.246	-1.2342	-1.22329	-1.21386
10.0014	-1.20258	-1.19181	-1.18088	-1.17038	-1.1616
10.102	-1.151	-1.14086	-1.13046	-1.12073	-1.11252
10.2078	-1.10244	-1.09287	-1.08309	-1.07392	-1.06604
10.3084	-1.05665	-1.04761	-1.03839	-1.02975	-1.02229
10.4089	-1.01342	-1.00487	-0.996157	-0.988067	-0.981017
10.5147	-0.972586	-0.964501	-0.956258	-0.948529	-0.941874
10.6153	-0.933889	-0.926218	-0.918402	-0.911078	-0.904769
10.7211	-0.897211	-0.889929	-0.882513	-0.875558	-0.869543
10.8217	-0.862406	-0.855473	-0.848435	-0.841831	-0.836111
10.9222	-0.829344	-0.822747	-0.816049	-0.809777	-0.804319
11.028	-0.797918	-0.791622	-0.785241	-0.779257	-0.774065
11.1286	-0.767991	-0.761992	-0.755913	-0.750207	-0.745254
11.2291	-0.739489	-0.733763	-0.727964	-0.722519	-0.717793
11.335	-0.712322	-0.706853	-0.701315	-0.696119	-0.691603
11.4355	-0.686407	-0.68118	-0.675889	-0.670924	-0.666606
11.5414	-0.661669	-0.65667	-0.651611	-0.646864	-0.642731
11.6419	-0.63804	-0.633256	-0.628418	-0.623877	-0.619922
11.7424	-0.615458	-0.610877	-0.606248	-0.6019	-0.598113
11.8483	-0.593863	-0.589473	-0.585041	-0.580878	-0.577249
11.9488	-0.573201	-0.568995	-0.564748	-0.56076	-0.557282
12.0547	-0.553423	-0.549391	-0.54532	-0.541498	-0.538162
12.1552	-0.534484	-0.530616	-0.526712	-0.523046	-0.519845
12.2557	-0.516336	-0.512621	-0.508875	-0.505364	-0.502293
12.3616	-0.498941	-0.495379	-0.491786	-0.488411	-0.485464
12.4621	-0.482263	-0.478842	-0.475393	-0.472154	-0.469322
12.5627	-0.466263	-0.462977	-0.459666	-0.456554	-0.453833
12.6685	-0.450908	-0.447751	-0.444569	-0.441581	-0.438967
12.769	-0.436169	-0.433133	-0.430087	-0.427215	-0.424704
12.8749	-0.422024	-0.419105	-0.416169	-0.413409	-0.410996
12.9754	-0.408428	-0.405622	-0.402798	-0.400142	-0.397815
13.076	-0.39536	-0.392656	-0.389939	-0.387374	-0.385139
13.1818	-0.382781	-0.380182	-0.377569	-0.375113	-0.372961
13.2823	-0.3707	-0.368198	-0.365682	-0.36332	-0.361248
13.3882	-0.359078	-0.35667	-0.354248	-0.351973	-0.34998
13.4887	-0.347896	-0.345574	-0.343244	-0.341053	-0.339133
13.5893	-0.337132	-0.334896	-0.332652	-0.330541	-0.328692
13.6951	-0.326769	-0.324615	-0.322452	-0.32042	-0.318637
13.7956	-0.316788	-0.314712	-0.312627	-0.31067	-0.308951
13.9015	-0.307174	-0.305171	-0.303163	-0.301274	-0.299618

R(Å) \ θ(º)	80	70	60	50	40
14.002	-0.297908	-0.295977	-0.29404	-0.29222	-0.290622
14.1026	-0.288977	-0.287114	-0.285247	-0.283492	-0.28195
14.2084	-0.280367	-0.278569	-0.276767	-0.275074	-0.273587
14.3089	-0.272062	-0.270326	-0.268588	-0.266955	-0.26552
14.4095	-0.26405	-0.262374	-0.260697	-0.259122	-0.257736
14.5153	-0.256319	-0.254701	-0.253083	-0.251561	-0.250224
14.6159	-0.248857	-0.247296	-0.245733	-0.244263	-0.242971
14.7217	-0.241654	-0.240146	-0.238636	-0.237217	-0.235971
14.8222	-0.234696	-0.233239	-0.231782	-0.230411	-0.229207
14.9228	-0.227978	-0.226569	-0.225161	-0.223837	-0.222673
15.0286	-0.221487	-0.220124	-0.218763	-0.217485	-0.216358
15.1292	-0.215215	-0.213899	-0.212582	-0.211346	-0.210256
15.235	-0.209151	-0.207878	-0.206605	-0.20541	-0.204358
15.3355	-0.203289	-0.202058	-0.200827	-0.199671	-0.198652
15.4361	-0.197619	-0.196427	-0.195237	-0.19412	-0.193135
15.5419	-0.192137	-0.190983	-0.189834	-0.18875	-0.187797
15.6425	-0.186832	-0.185716	-0.184602	-0.183555	-0.182633
15.7483	-0.181698	-0.180618	-0.179539	-0.178526	-0.177634
15.8489	-0.176729	-0.175682	-0.174639	-0.173659	-0.172794
15.9494	-0.17192	-0.170906	-0.169896	-0.168945	-0.168108
16.0552	-0.167262	-0.166279	-0.165301	-0.164381	-0.16357
16.1558	-0.16275	-0.161799	-0.160851	-0.15996	-0.159176
16.2563	-0.15838	-0.157459	-0.15654	-0.155678	-0.154916
16.3622	-0.154146	-0.153253	-0.152363	-0.151527	-0.150791
16.4627	-0.150043	-0.149177	-0.148315	-0.147504	-0.146789
16.5685	-0.146066	-0.145226	-0.14439	-0.143605	-0.142912
16.6691	-0.14221	-0.141395	-0.140586	-0.139824	-0.139152
16.7696	-0.138471	-0.137681	-0.136896	-0.136158	-0.135505
16.8755	-0.134846	-0.134081	-0.133316	-0.1326	-0.131969
16.976	-0.131328	-0.130585	-0.129847	-0.129151	-0.128538
17.0818	-0.127916	-0.127194	-0.126476	-0.125803	-0.125207
17.1824	-0.124605	-0.123905	-0.123208	-0.122553	-0.121976
17.2829	-0.121391	-0.120711	-0.120035	-0.1194	-0.118839
17.3888	-0.11827	-0.117611	-0.116954	-0.116332	-0.11579
17.4893	-0.115237	-0.114596	-0.113961	-0.113363	-0.112834
17.5898	-0.112298	-0.111675	-0.111056	-0.110475	-0.109962
17.6957	-0.109441	-0.108835	-0.108235	-0.10767	-0.107172
17.7962	-0.106666	-0.106077	-0.105495	-0.104947	-0.104462
17.9021	-0.103969	-0.103399	-0.102831	-0.102298	-0.101828
18.0026	-0.10135	-0.100794	-0.100244	-0.099726	-0.0992693
18.1031	-0.0988029	-0.0982623	-0.0977274	-0.0972246	-0.0967808
18.209	-0.0963283	-0.0958035	-0.0952832	-0.0947948	-0.0943636
18.3095	-0.0939211	-0.0934113	-0.0929058	-0.0924302	-0.0920103
18.4154	-0.0915817	-0.0910859	-0.0905943	-0.0901327	-0.0897228
18.5159	-0.0893058	-0.0888231	-0.0883453	-0.0878967	-0.0874988
18.6164	-0.0870928	-0.0866233	-0.0861592	-0.0857211	-0.085335
18.7223	-0.08494	-0.0844826	-0.0840303	-0.0836062	-0.08323
18.8228	-0.082846	-0.0824007	-0.0819597	-0.0815471	-0.0811813
18.9287	-0.0808057	-0.0803738	-0.0799449	-0.0795433	-0.0791875
19.0292	-0.0788232	-0.0784018	-0.0779842	-0.0775924	-0.0772467
19.1297	-0.0768914	-0.0764805	-0.0760754	-0.075695	-0.0753579
19.2356	-0.0750112	-0.0746122	-0.0742162	-0.0738462	-0.0735187
19.3361	-0.0731799	-0.0727916	-0.0724062	-0.0720461	-0.0717263
19.4367	-0.0713975	-0.0710185	-0.0706445	-0.0702929	-0.0699826
19.5425	-0.0696617	-0.0692914	-0.0689278	-0.0685852	-0.0682838
19.6431	-0.0679702	-0.0676114	-0.0672562	-0.0669224	-0.0666268
19.7489	-0.0663219	-0.0659725	-0.0656262	-0.0653014	-0.0650154
19.8494	-0.0647165	-0.0643763	-0.0640392	-0.0637231	-0.0634429
19.95	-0.0631525	-0.0628196	-0.0624914	-0.0621835	-0.0619107
20.0558	-0.0616274	-0.0613045	-0.0609841	-0.0606839	-0.0604185

R(Å) \ θ(º)	80	70	60	50	40
20.1564	-0.0601415	-0.0598257	-0.0595132	-0.0592215	-0.0589621
20.2622	-0.0586923	-0.0583853	-0.0580813	-0.057796	-0.0575438
20.3627	-0.05728	-0.0569798	-0.0566833	-0.0564052	-0.0561598
20.4633	-0.0559019	-0.0556098	-0.0553206	-0.0550499	-0.0548098
20.5691	-0.0545596	-0.0542737	-0.0539912	-0.0537272	-0.0534936
20.6697	-0.0532494	-0.0529698	-0.0526959	-0.0524384	-0.0522099
20.7755	-0.0519712	-0.0516997	-0.051431	-0.0511804	-0.050957
20.876	-0.0507234	-0.0504583	-0.0501974	-0.0499522	-0.0497358
20.9766	-0.0495065	-0.0492484	-0.048994	-0.0487552	-0.048543
21.0824	-0.0483187	-0.0480669	-0.0478183	-0.0475847	-0.0473786
21.183	-0.0471609	-0.0469147	-0.0466722	-0.0464441	-0.0462429
21.2835	-0.04603	-0.0457896	-0.0455526	-0.0453301	-0.045133
21.3893	-0.0449256	-0.044691	-0.0444601	-0.0442428	-0.0440514
21.4899	-0.0438477	-0.0436193	-0.0433932	-0.0431825	-0.042994
21.5957	-0.0427943	-0.0425711	-0.0423507	-0.0421444	-0.0419609
21.6963	-0.0417671	-0.0415481	-0.041333	-0.0411322	-0.0409465
21.7968	-0.0407639	-0.0405446	-0.0403344	-0.0401465	-0.0399681
21.9026	-0.0397844	-0.0395739	-0.0393687	-0.0391714	-0.0390033
22.0032	-0.0388205	-0.038619	-0.0384193	-0.0382364	-0.0380672
22.109	-0.0378905	-0.0376899	-0.0374935	-0.0373052	-0.0371456
22.2096	-0.0369712	-0.036778	-0.0365873	-0.0364128	-0.0362517
22.3101	-0.0360834	-0.0358913	-0.0357052	-0.0355222	-0.035371
22.4159	-0.0352031	-0.0350196	-0.0348368	-0.0346658	-0.0345144
22.5165	-0.0343526	-0.0341711	-0.033992	-0.0338254	-0.0336773
22.617	-0.0335186	-0.033341	-0.033167	-0.0330035	-0.032858
22.7229	-0.0327026	-0.032529	-0.0323582	-0.0321998	-0.0320569
22.8234	-0.0319061	-0.0317371	-0.0315692	-0.0314138	-0.0312754
22.9292	-0.0311276	-0.0309608	-0.030798	-0.0306448	-0.0305098
23.0298	-0.0303648	-0.0302021	-0.0300428	-0.0298938	-0.029761
23.1303	-0.0296181	-0.0294596	-0.029304	-0.0291585	-0.029029
23.2362	-0.0288894	-0.0287336	-0.0285824	-0.0284395	-0.0283131
23.3367	-0.0281766	-0.0280249	-0.0278764	-0.0277368	-0.0276134
23.4425	-0.0274795	-0.0273305	-0.0271848	-0.0270494	-0.0269278
23.5431	-0.0267972	-0.0266515	-0.0265088	-0.0263754	-0.0262566
23.6436	-0.0261285	-0.0259869	-0.0258469	-0.0257156	-0.0256015
23.7495	-0.0254749	-0.0253364	-0.0252001	-0.0250721	-0.0249595
23.85	-0.024836	-0.0247001	-0.0245671	-0.0244414	-0.0243305
23.9558	-0.0242091	-0.0240768	-0.0239458	-0.023824	-0.0237151
24.0564	-0.0235977	-0.0234673	-0.0233394	-0.0232198	-0.0231144
24.1569	-0.0229979	-0.0228706	-0.0227457	-0.0226283	-0.0225231
24.2628	-0.0224106	-0.0222852	-0.0221628	-0.0220486	-0.0219472
24.3633	-0.0218357	-0.0217124	-0.0215935	-0.0214813	-0.0213821
24.4639	-0.0212732	-0.0211532	-0.0210355	-0.0209258	-0.0208279
24.5697	-0.0207217	-0.0206036	-0.0204886	-0.0203811	-0.020286
24.6702	-0.0201824	-0.0200663	-0.0199549	-0.0198491	-0.0197553
24.7761	-0.0196533	-0.01954	-0.0194301	-0.0193283	-0.019235
24.8766	-0.0191349	-0.0190238	-0.0189161	-0.0188167	-0.0187262
24.9772	-0.0186277	-0.0185201	-0.0184141	-0.0183152	-0.0182282
25.083	-0.0181299	-0.0180246	-0.0179208	-0.0178257	-0.0177395
25.1835	-0.0176444	-0.0175402	-0.0174397	-0.0173446	-0.0172595
25.2894	-0.0171666	-0.0170646	-0.0169658	-0.0168734	-0.0167907
25.3899	-0.0166992	-0.0165991	-0.016503	-0.016411	-0.0163307
25.4905	-0.0162411	-0.0161435	-0.0160475	-0.0159593	-0.0158812
25.5963	-0.0157916	-0.0156957	-0.0156027	-0.0155151	-0.0154378
25.6968	-0.0153512	-0.0152583	-0.0151659	-0.015081	-0.0150048
25.8027	-0.0149203	-0.0148284	-0.0147386	-0.0146548	-0.0145808
25.9032	-0.014497	-0.0144076	-0.0143187	-0.014238	-0.0141649
26.0038	-0.0140817	-0.0139939	-0.0139081	-0.0138278	-0.013756
26.1096	-0.0136759	-0.0135894	-0.0135056	-0.0134264	-0.0133566
26.2101	-0.0132769	-0.0131924	-0.0131099	-0.0130326	-0.0129635

R(Å) \ θ(º)	80	70	60	50	40
26.3107	-0.0128865	-0.0128028	-0.0127212	-0.0126459	-0.0125783
26.4165	-0.0125022	-0.0124207	-0.0123417	-0.0122671	-0.0122015
26.5171	-0.0121255	-0.0120467	-0.011968	-0.0118957	-0.011831
26.6229	-0.0117573	-0.0116791	-0.0116027	-0.0115314	-0.0114682
26.7234	-0.0113962	-0.011319	-0.0112432	-0.0111726	-0.0111111
26.824	-0.0110413	-0.0109647	-0.0108912	-0.0108232	-0.0107628
26.9298	-0.0106921	-0.0106182	-0.0105471	-0.0104788	-0.0104193
27.0304	-0.0103515	-0.0102789	-0.0102082	-0.0101413	-0.0100835
27.1362	-0.0100162	-0.00994352	-0.00987438	-0.0098096	-0.00975257
27.2367	-0.00968651	-0.00961606	-0.0095478	-0.0094853	-0.00942885
27.3373	-0.00936432	-0.00929431	-0.00922869	-0.0091661	-0.00911149
27.4431	-0.00904806	-0.0089798	-0.00891418	-0.008853	-0.00879961
27.5437	-0.00873685	-0.00866969	-0.00860582	-0.0085455	-0.00849279
27.6442	-0.00843046	-0.00836659	-0.00830207	-0.0082435	-0.00819189
27.75	-0.00813175	-0.00806701	-0.0080049	-0.0079485	-0.00789736
27.8506	-0.00783722	-0.00777533	-0.00771409	-0.0076577	-0.00760699
27.9564	-0.00754839	-0.00748716	-0.00742702	-0.0073717	-0.00732277
28.057	-0.00726439	-0.00720447	-0.00714565	-0.0070914	-0.00704338
28.1575	-0.00698632	-0.00692684	-0.00686999	-0.0068164	-0.00676882
28.2633	-0.00671307	-0.00665513	-0.00659828	-0.0065467	-0.00649952
28.3639	-0.00644421	-0.00638715	-0.00633316	-0.0062809	-0.00623483
28.4697	-0.00618128	-0.00612466	-0.00606979	-0.0060187	-0.00597432
28.5703	-0.0059223	-0.00586699	-0.00581388	-0.0057638	-0.00571951
28.6708	-0.0056664	-0.00561284	-0.00556105	-0.0055123	-0.00546887
28.7766	-0.00541707	-0.00536418	-0.00531304	-0.0052652	-0.0052224
28.8772	-0.00517258	-0.00512012	-0.00506877	-0.005022	-0.00498032
28.983	-0.00493181	-0.0048798	-0.00482998	-0.0047848	-0.00474306
29.0836	-0.00469434	-0.00464386	-0.00459448	-0.0045502	-0.00450954
29.1841	-0.00446148	-0.00441144	-0.00436403	-0.0043197	-0.00427932
29.2899	-0.00423279	-0.00418406	-0.00413644	-0.0040939	-0.00405457
29.3905	-0.00400783	-0.0039602	-0.00391411	-0.0038715	-0.00383312
29.491	-0.00378747	-0.00374094	-0.00369507	-0.0036529	-0.00361562
29.5969	-0.00357107	-0.00352476	-0.00348087	-0.0034381	-0.00340207
29.6974	-0.00335796	-0.00331209	-0.00326885	-0.0032289	-0.00319094
29.8032	-0.00314814	-0.00310403	-0.00306035	-0.0030209	-0.00298595
29.9038	-0.00294118	-0.00289772	-0.00285515	-0.002817	-0.00278096
30.0043	-0.00273817	-0.00269537	-0.00265433	-0.0026155	-0.00258234
30.1102	-0.00253998	-0.0024974	-0.00245746	-0.0024195	-0.00238547
30.2107	-0.00234465	-0.00230317	-0.00226322	-0.0022255	-0.00219211
30.3166	-0.00215195	-0.00211113	-0.00207184	-0.0020354	-0.00200227
30.4171	-0.00196232	-0.00192238	-0.00188331	-0.0018464	-0.00181484
30.5176	-0.00177577	-0.00173582	-0.00169851	-0.001663	-0.00163157
30.6235	-0.00159295	-0.00155476	-0.00151723	-0.0014821	-0.00145182
30.724	-0.0014132	-0.00137523	-0.00133923	-0.0013041	-0.00127449
30.8299	-0.00123696	-0.00119965	-0.00116321	-0.0011301	-0.00109913
30.9304	-0.00106226	-0.00102538	-0.000989171	-0.0009573	-0.00092728
31.0309	-0.000891067	-0.000854415	-0.000819957	-0.0007881	-0.000758943
31.1368	-0.000723387	-0.000686955	-0.000652717	-0.0006211	-0.000591483
31.2373	-0.000557465	-0.000522569	-0.000488989	-0.0004578	-0.000429072
31.3379	-0.000394834	-0.000360816	-0.000328114	-0.0002965	-0.000268636
31.4437	-0.00023374	-0.0002006	-0.000167458	-0.0001374	-0.000110176
31.5442	-0.0000786	-0.0000454	-0.0000125	0.0000171	0.0000435
31.6501	0.0000731	0.000106226	0.000137831	0.0001668	0.000192041
31.7506	-0.000167458	-0.000134756	-0.00010425	-0.0000764	-0.0000503

CH^- + He PES

Method: MRCI. CBS extrapolation (0º – 30º)

R(Å) \ θ(º)	30	20	10	0
1.89445	11392.5	14695.9	17790.7	19153.8
2.00029	8015.63	10347.2	12434	13318
2.10083	5572.88	7215.3	8655.3	9249.71
2.20138	3823.6	4977.08	5979.2	6380.27
2.30721	2584.18	3390.28	4084.45	4352.8
2.40776	1716.09	2274.64	2751.92	2934.8
2.5083	1114.53	1497.38	1825.7	1954.34
2.61413	701.839	961.676	1188.06	1279.73
2.71468	421.767	596.871	752.367	816.891
2.82051	234.118	351.632	457.463	501.836
2.92106	110.381	189.03	260.237	290.195
3.0216	30.3996	82.8441	130.277	150.016
3.12744	-19.9151	14.7777	46.1624	58.7179
3.22798	-50.177	-27.6173	-7.05315	0.645625
3.33382	-66.939	-52.6213	-39.4076	-34.7853
3.43436	-74.8478	-65.8734	-57.6068	-54.81
3.5349	-77.2583	-71.4692	-66.392	-64.669
3.64074	-76.4517	-72.4049	-69.2739	-68.2129
3.74128	-73.8968	-70.8785	-68.7423	-68.0876
3.84712	-70.3356	-68.0802	-66.5049	-66.0876
3.94766	-66.16	-64.6058	-63.4014	-63.1112
4.0482	-61.6211	-60.6966	-59.8139	-59.6122
4.15404	-56.9466	-56.5067	-55.9215	-55.7997
4.25458	-52.3564	-52.2116	-51.8727	-51.8275
4.35513	-47.6914	-47.4971	-47.8385	-47.8568
4.46096	-43.9175	-43.8104	-43.6796	-43.4146
4.56151	-40.355	-40.2022	-40.1769	-40.1626
4.66734	-37.17	-36.9709	-36.933	-36.928
4.76788	-34.3126	-34.0995	-34.0251	-34.0189
4.86843	-31.7255	-31.54	-31.4227	-31.4097
4.97426	-29.3591	-28.9495	-29.0798	-29.0592
5.07481	-27.1819	-27.0336	-26.9507	-26.9259
5.18064	-24.7861	-25.0536	-24.9992	-24.9755
5.28119	-22.9746	-23.023	-22.9819	-22.9554
5.38173	-21.3284	-21.4262	-21.4266	-21.4124
5.48757	-19.7315	-19.855	-19.888	-19.8854
5.58811	-18.2745	-18.4043	-18.4627	-18.471
5.69394	-16.9423	-17.0682	-17.1424	-17.1597
5.79449	-15.7246	-15.8385	-15.9194	-15.9429
5.89503	-14.6086	-14.7043	-14.7857	-14.8127
6.00087	-13.5817	-13.6593	-13.7347	-13.7618
6.10141	-12.6319	-12.6921	-12.7579	-12.7835
6.20195	-11.7495	-11.7943	-11.8494	-11.872
6.30779	-10.9285	-10.9589	-11.0031	-11.0221
6.40833	-10.1618	-10.1814	-10.2151	-10.2304
6.51417	-9.44575	-9.45607	-9.48056	-9.49234
6.61471	-8.7768	-8.7799	-8.79645	-8.80507
6.71526	-8.15253	-8.15001	-8.16006	-8.16716
6.82109	-7.57096	-7.56394	-7.56876	-7.57235
6.92164	-7.02954	-7.01963	-7.02017	-7.02188
7.02747	-6.52759	-6.5143	-6.51189	-6.51239
7.12801	-6.06205	-6.0471	-6.04231	-6.04149
7.22856	-5.63179	-5.61631	-5.60872	-5.60686
7.33439	-5.23373	-5.21644	-5.20878	-5.2063
7.43494	-4.86647	-4.84863	-4.84028	-4.83738
7.53548	-4.5283	-4.51033	-4.50115	-4.49832
7.64132	-4.21681	-4.19889	-4.18966	-4.18654
7.74186	-3.93056	-3.91268	-3.90345	-3.90035

R(Å) \ θ(º)	30	20	10	0
7.84769	-3.66751	-3.65012	-3.64076	-3.6378
7.94824	-3.42603	-3.41027	-3.40033	-3.39703
8.04878	-3.20444	-3.18814	-3.17931	-3.17634
8.15462	-3.00119	-2.98563	-2.97673	-2.97388
8.25516	-2.8144	-2.79948	-2.78964	-2.78699
8.361	-2.64136	-2.62749	-2.61967	-2.61713
8.46154	-2.48418	-2.47113	-2.46373	-2.4613
8.56208	-2.33972	-2.3275	-2.3205	-2.31819
8.66792	-2.20686	-2.1954	-2.18882	-2.18666
8.76846	-2.08449	-2.07379	-2.06765	-2.0656
8.8743	-1.97169	-1.96172	-1.95594	-1.95401
8.97484	-1.86752	-1.85822	-1.85279	-1.85098
9.07539	-1.77116	-1.76244	-1.75737	-1.75568
9.18122	-1.6818	-1.67369	-1.66895	-1.66736
9.28176	-1.59888	-1.59131	-1.58687	-1.58539
9.38231	-1.52179	-1.5147	-1.51055	-1.50917
9.48814	-1.44998	-1.44334	-1.43945	-1.43815
9.58869	-1.38292	-1.37673	-1.37308	-1.37185
9.69452	-1.32028	-1.31444	-1.311	-1.30985
9.79507	-1.26158	-1.25608	-1.25284	-1.25176
9.89561	-1.20649	-1.20131	-1.19825	-1.19723
10.0014	-1.15472	-1.14982	-1.14692	-1.14595
10.102	-1.10596	-1.10132	-1.09859	-1.09767
10.2078	-1.05998	-1.0556	-1.053	-1.05213
10.3084	-1.01642	-1.01241	-1.00994	-1.00912
10.4089	-0.975448	-0.971639	-0.969293	-0.96851
10.5147	-0.936594	-0.932821	-0.930585	-0.929978
10.6153	-0.899752	-0.896158	-0.894025	-0.893312
10.7211	-0.864765	-0.861352	-0.859317	-0.858632
10.8217	-0.83156	-0.828321	-0.826376	-0.825708
10.9222	-0.799979	-0.796871	-0.795013	-0.794387
11.028	-0.769925	-0.766954	-0.765178	-0.764578
11.1286	-0.7413	-0.738458	-0.736758	-0.736183
11.2291	-0.714014	-0.711296	-0.709667	-0.709117
11.335	-0.687988	-0.685385	-0.683824	-0.683296
11.4355	-0.663146	-0.66065	-0.659153	-0.658647
11.5414	-0.63942	-0.637029	-0.635593	-0.63511
11.6419	-0.616748	-0.614458	-0.61308	-0.612614
11.7424	-0.595072	-0.592874	-0.591552	-0.591104
11.8483	-0.574334	-0.572223	-0.570954	-0.570525
11.9488	-0.554486	-0.55246	-0.55124	-0.550828
12.0547	-0.535478	-0.533534	-0.532362	-0.531965
12.1552	-0.517262	-0.515403	-0.514275	-0.513895
12.2557	-0.49982	-0.498025	-0.496942	-0.496575
12.3616	-0.483087	-0.481363	-0.48032	-0.479967
12.4621	-0.467039	-0.465379	-0.464379	-0.464039
12.5627	-0.451639	-0.450045	-0.44908	-0.448753
12.6685	-0.436857	-0.435323	-0.434394	-0.43408
12.769	-0.422674	-0.421186	-0.420292	-0.419989
12.8749	-0.409045	-0.407622	-0.406758	-0.406455
12.9754	-0.395925	-0.394557	-0.393728	-0.393448
13.076	-0.383331	-0.382013	-0.381215	-0.380945
13.1818	-0.37122	-0.369952	-0.369183	-0.368921
13.2823	-0.359572	-0.35835	-0.357608	-0.357357
13.3882	-0.348365	-0.347186	-0.346471	-0.34623
13.4887	-0.337578	-0.336442	-0.335753	-0.335518
13.5893	-0.327192	-0.326097	-0.325432	-0.325207
13.6951	-0.317192	-0.316136	-0.315493	-0.315276
13.7956	-0.307557	-0.306538	-0.305918	-0.305709
13.9015	-0.298274	-0.297291	-0.296693	-0.296491

R(Å) \ θ(º)	30	20	10	0
14.002	-0.289326	-0.288377	-0.2878	-0.287604
14.1026	-0.280699	-0.279783	-0.279226	-0.279037
14.2084	-0.272379	-0.271495	-0.270957	-0.270774
14.3089	-0.264354	-0.2635	-0.26298	-0.262803
14.4095	-0.25661	-0.255785	-0.255283	-0.255112
14.5153	-0.249136	-0.248339	-0.247853	-0.247689
14.6159	-0.241921	-0.241151	-0.240682	-0.240522
14.7217	-0.234955	-0.234211	-0.233757	-0.233602
14.8222	-0.228224	-0.227505	-0.227066	-0.226918
14.9228	-0.221723	-0.221027	-0.220603	-0.220459
15.0286	-0.215441	-0.214768	-0.214356	-0.214218
15.1292	-0.209368	-0.208718	-0.20832	-0.208185
15.235	-0.203498	-0.202869	-0.202485	-0.202353
15.3355	-0.197822	-0.197213	-0.19684	-0.196714
15.4361	-0.192331	-0.191741	-0.191381	-0.191257
15.5419	-0.18702	-0.186448	-0.186099	-0.18598
15.6425	-0.18188	-0.181325	-0.180987	-0.180871
15.7483	-0.176904	-0.176369	-0.176041	-0.17593
15.8489	-0.172088	-0.171569	-0.171251	-0.171143
15.9494	-0.167424	-0.166921	-0.166614	-0.166509
16.0552	-0.162908	-0.16242	-0.162122	-0.16202
16.1558	-0.158534	-0.158061	-0.157772	-0.157672
16.2563	-0.154295	-0.153836	-0.153556	-0.15346
16.3622	-0.150188	-0.149742	-0.14947	-0.149377
16.4627	-0.146205	-0.145773	-0.14551	-0.14542
16.5685	-0.142345	-0.141927	-0.141672	-0.141584
16.6691	-0.138602	-0.138196	-0.137948	-0.137864
16.7696	-0.134973	-0.134579	-0.134339	-0.134256
16.8755	-0.131452	-0.131069	-0.130836	-0.130756
16.976	-0.128036	-0.127665	-0.127439	-0.127361
17.0818	-0.124721	-0.124361	-0.124141	-0.124065
17.1824	-0.121504	-0.121154	-0.12094	-0.120866
17.2829	-0.118374	-0.118035	-0.117826	-0.117755
17.3888	-0.115345	-0.115015	-0.114813	-0.114744
17.4893	-0.112401	-0.11208	-0.111884	-0.111817
17.5898	-0.109541	-0.10923	-0.109039	-0.108974
17.6957	-0.106763	-0.106461	-0.106276	-0.106212
17.7962	-0.104065	-0.103771	-0.103591	-0.103529
17.9021	-0.101442	-0.101157	-0.100981	-0.100923
18.0026	-0.0988944	-0.0986165	-0.0984469	-0.0983878
18.1031	-0.0964161	-0.0961468	-0.0959806	-0.095924
18.209	-0.0940087	-0.0937455	-0.0935846	-0.0935287
18.3095	-0.0916658	-0.091411	-0.0912547	-0.0912005
18.4154	-0.0893878	-0.0891401	-0.088988	-0.0889362
18.5159	-0.0871731	-0.0869324	-0.0867831	-0.0867329
18.6164	-0.0850185	-0.0847833	-0.0846382	-0.0845895
18.7223	-0.0829215	-0.0826939	-0.0825527	-0.0825045
18.8228	-0.0808814	-0.0806587	-0.0805224	-0.0804752
18.9287	-0.0788959	-0.0786799	-0.0785469	-0.0785004
19.0292	-0.0769616	-0.076752	-0.0766217	-0.076578
19.1297	-0.0750823	-0.0748755	-0.0747498	-0.0747063
19.2356	-0.0732483	-0.0730493	-0.0729257	-0.0728838
19.3361	-0.0714638	-0.0712691	-0.0711499	-0.0711089
19.4367	-0.0697267	-0.0695372	-0.0694205	-0.0693814
19.5425	-0.0680347	-0.0678499	-0.0677362	-0.0676969
19.6431	-0.0663847	-0.066205	-0.0660952	-0.066057
19.7489	-0.064779	-0.0646035	-0.0644955	-0.0644579
19.8494	-0.063212	-0.0630417	-0.0629363	-0.0629008
19.95	-0.0616873	-0.0615216	-0.061418	-0.0613833
20.0558	-0.0601997	-0.0600375	-0.0599381	-0.0599027

R(Å) \ θ(º)	30	20	10	0
20.1564	-0.0587498	-0.0585907	-0.0584942	-0.0584604
20.2622	-0.0573366	-0.0571821	-0.0570875	-0.0570555
20.3627	-0.0559575	-0.0558065	-0.0557141	-0.0556827
20.4633	-0.0546132	-0.0544668	-0.0543766	-0.0543454
20.5691	-0.0533014	-0.0531583	-0.0530707	-0.05304
20.6697	-0.0520225	-0.0518831	-0.0517971	-0.0517675
20.7755	-0.0507737	-0.0506372	-0.0505542	-0.0505255
20.876	-0.0495569	-0.0494237	-0.0493416	-0.0493144
20.9766	-0.0483694	-0.048239	-0.0481595	-0.0481328
21.0824	-0.047209	-0.0470819	-0.0470051	-0.0469777
21.183	-0.0460776	-0.0459534	-0.0458777	-0.045852
21.2835	-0.0449717	-0.044851	-0.0447768	-0.0447522
21.3893	-0.0438927	-0.0437753	-0.0437033	-0.0436785
21.4899	-0.0428395	-0.0427253	-0.0426547	-0.0426301
21.5957	-0.0418101	-0.0416984	-0.0416304	-0.0416067
21.6963	-0.0407984	-0.0406921	-0.0406335	-0.0406103
21.7968	-0.0398248	-0.0397181	-0.0396441	-0.0396213
21.9026	-0.0388633	-0.0387577	-0.0387019	-0.0386789
22.0032	-0.03793	-0.037828	-0.0377586	-0.0377358
22.109	-0.0370115	-0.0369121	-0.0368575	-0.0368364
22.2096	-0.0361203	-0.0360232	-0.0359541	-0.0359337
22.3101	-0.0352432	-0.0351486	-0.0350889	-0.0350688
22.4159	-0.0343886	-0.0342958	-0.034238	-0.0342183
22.5165	-0.0335542	-0.0334633	-0.0334075	-0.0333876
22.617	-0.0327384	-0.032649	-0.0325942	-0.0325764
22.7229	-0.031941	-0.0318537	-0.0317999	-0.0317812
22.8234	-0.0311612	-0.0310756	-0.0310243	-0.0310056
22.9292	-0.0303979	-0.0303154	-0.0302638	-0.0302458
23.0298	-0.0296523	-0.0295718	-0.0295211	-0.0295038
23.1303	-0.0289215	-0.0288431	-0.0287938	-0.0287773
23.2362	-0.028208	-0.0281316	-0.0280831	-0.0280666
23.3367	-0.0275114	-0.0274354	-0.027388	-0.027372
23.4425	-0.0268279	-0.026754	-0.0267083	-0.0266918
23.5431	-0.0261594	-0.0260865	-0.0260411	-0.026026
23.6436	-0.025506	-0.0254347	-0.0253908	-0.0253754
23.7495	-0.0248658	-0.0247962	-0.0247534	-0.024739
23.85	-0.0242386	-0.0241716	-0.0241288	-0.0241137
23.9558	-0.0236264	-0.0235602	-0.0235187	-0.0235042
24.0564	-0.0230255	-0.0229612	-0.0229213	-0.022907
24.1569	-0.0224384	-0.0223739	-0.022334	-0.022321
24.2628	-0.0218632	-0.0218	-0.0217624	-0.0217497
24.3633	-0.0212998	-0.0212392	-0.0212015	-0.0211881
24.4639	-0.0207491	-0.0206886	-0.0206517	-0.0206383
24.5697	-0.0202077	-0.0201491	-0.0201131	-0.0201001
24.6702	-0.019679	-0.0196217	-0.0195861	-0.0195741
24.7761	-0.0191604	-0.0191044	-0.0190704	-0.0190585
24.8766	-0.0186534	-0.0185978	-0.0185638	-0.0185511
24.9772	-0.0181554	-0.0181012	-0.0180689	-0.0180571
25.083	-0.0176684	-0.0176161	-0.0175828	-0.0175714
25.1835	-0.017191	-0.0171386	-0.0171063	-0.0170955
25.2894	-0.0167231	-0.016672	-0.0166401	-0.01663
25.3899	-0.0162642	-0.0162148	-0.0161838	-0.0161735
25.4905	-0.0158153	-0.0157664	-0.0157368	-0.0157265
25.5963	-0.0153746	-0.0153274	-0.0152974	-0.0152871
25.6968	-0.0149421	-0.0148962	-0.014867	-0.0148565
25.8027	-0.0145191	-0.0144744	-0.0144456	-0.0144359
25.9032	-0.0141052	-0.0140593	-0.0140312	-0.0140211
26.0038	-0.0136972	-0.0136533	-0.0136263	-0.0136169
26.1096	-0.0132991	-0.013256	-0.0132288	-0.0132194
26.2101	-0.0129066	-0.0128649	-0.0128377	-0.0128305

R(Å) \ θ(º)	30	20	10	0
26.3107	-0.0125232	-0.012482	-0.0124569	-0.0124475
26.4165	-0.0121475	-0.0121064	-0.0120814	-0.0120737
26.5171	-0.0117781	-0.0117375	-0.011714	-0.0117059
26.6229	-0.0114169	-0.0113776	-0.011353	-0.0113449
26.7234	-0.0110598	-0.0110216	-0.0109981	-0.0109893
26.824	-0.0107126	-0.0106741	-0.0106507	-0.0106428
26.9298	-0.0103706	-0.0103335	-0.0103105	-0.0103026
27.0304	-0.0100346	-0.00999882	-0.00997644	-0.00996963
27.1362	-0.00970561	-0.00966961	-0.0096481	-0.00964086
27.2367	-0.00938276	-0.00934655	-0.00932701	-0.00931867
27.3373	-0.00906496	-0.00903182	-0.00901009	-0.00900285
27.4431	-0.00875484	-0.00872082	-0.00869975	-0.00869251
27.5437	-0.00844802	-0.00841531	-0.00839534	-0.00838832
27.6442	-0.00814843	-0.00811617	-0.0080973	-0.00809049
27.75	-0.00785478	-0.00782251	-0.00780342	-0.00779662
27.8506	-0.00756507	-0.00753369	-0.00751525	-0.00750888
27.9564	-0.00728151	-0.00725122	-0.00723191	-0.00722554
28.057	-0.007003	-0.00697359	-0.00695449	-0.00694813
28.1575	-0.00673041	-0.00670056	-0.0066819	-0.0066762
28.2633	-0.00646155	-0.00643236	-0.00641436	-0.00640734
28.3639	-0.00619665	-0.00616943	-0.00615056	-0.00614397
28.4697	-0.00593767	-0.00591023	-0.00589245	-0.00588675
28.5703	-0.00568439	-0.00565608	-0.00563918	-0.00563369
28.6708	-0.00543309	-0.00540588	-0.00538942	-0.00538349
28.7766	-0.00518706	-0.00516051	-0.00514514	-0.00513878
28.8772	-0.00494674	-0.0049215	-0.00490526	-0.00489955
28.983	-0.00470883	-0.00468381	-0.00466844	-0.00466318
29.0836	-0.00447531	-0.0044516	-0.00443624	-0.0044301
29.1841	-0.00424771	-0.00422225	-0.00420777	-0.00420294
29.2899	-0.00402319	-0.00399817	-0.00398434	-0.00397973
29.3905	-0.00380174	-0.00377825	-0.00376267	-0.00375872
29.491	-0.00358534	-0.00356119	-0.00354803	-0.0035421
29.5969	-0.00337179	-0.0033494	-0.00333448	-0.00332965
29.6974	-0.00316197	-0.00313915	-0.00312488	-0.00312071
29.8032	-0.00295457	-0.00293262	-0.00291901	-0.0029144
29.9038	-0.00275353	-0.00273048	-0.00271753	-0.00271271
30.0043	-0.00255271	-0.00253252	-0.00251869	-0.00251496
30.1102	-0.00235781	-0.00233762	-0.00232336	-0.00231897
30.2107	-0.00216534	-0.00214383	-0.00213198	-0.00212693
30.3166	-0.00197549	-0.0019553	-0.00194235	-0.00193818
30.4171	-0.0017885	-0.00176984	-0.00175711	-0.0017525
30.5176	-0.00160633	-0.00158746	-0.00157385	-0.00156968
30.6235	-0.00142615	-0.00140661	-0.00139608	-0.00139059
30.724	-0.00124969	-0.00122994	-0.00121808	-0.00121523
30.8299	-0.00107433	-0.00105589	-0.00104426	-0.00103987
30.9304	-0.000902919	-0.000883825	-0.000874387	-0.000869119
31.0309	-0.000734801	-0.000717023	-0.000705611	-0.000702319
31.1368	-0.000569755	-0.000552417	-0.000540345	-0.000537273
31.2373	-0.000406466	-0.000388689	-0.000378374	-0.000375082
31.3379	-0.00024603	-0.00022979	-0.000219036	-0.000215084
31.4437	-0.0000884	-0.0000702	-0.000061	-0.0000575
31.5442	0.0000654	0.0000819	0.0000924	0.0000961
31.6501	0.00021333	0.000230229	0.000240106	0.000243398
31.7506	-0.0000288	-0.0000127	-0.00000395	0

Inelastic collisions' Cross-Section: Triplet State

Initial rotational state: j=5

**Final State: j'=4**		**Final State: j'=3**		**Final State: j'=2**	
E(cm⁻¹)	σ (Å²)	E(cm⁻¹)	σ (Å²)	E(cm⁻¹)	σ (Å²)
410.96970977	378.674482618	410.96970977	378.674482618	410.96970977	19.096740852
411.06970977	769.502833462	411.06970977	769.502833462	411.06970977	36.529529437
411.16970977	307.152203705	411.16970977	307.152203705	411.16970977	15.201436057
411.26970977	261.09935268	411.26970977	261.09935268	411.26970977	12.728666642
411.36970977	240.371069604	411.36970977	240.371069604	411.36970977	11.467683976
411.46970977	263.604523555	411.46970977	263.604523555	411.46970977	11.863644132
411.56970977	264.884622753	411.56970977	264.884622753	411.56970977	12.033785215
411.66970977	227.460595346	411.66970977	227.460595346	411.66970977	10.922412931
411.76970977	197.82757261	411.76970977	197.82757261	411.76970977	9.480053033
411.86970977	228.50826795	411.86970977	228.50826795	411.86970977	10.404311604
411.96970977	176.898642951	411.96970977	176.898642951	411.96970977	8.606642468
412.06970977	159.370129873	412.06970977	159.370129873	412.06970977	7.780841547
412.16970977	149.929593542	412.16970977	149.929593542	412.16970977	7.293473365
412.26970977	142.755178326	412.26970977	142.755178326	412.26970977	6.916673493
412.36970977	136.681565553	412.36970977	136.681565553	412.36970977	6.59691665
412.46970977	131.2986313	412.46970977	131.2986313	412.46970977	6.313125105
412.57032977	126.425255645	412.57032977	126.425255645	412.57032977	6.054270337
412.85376577	116.256421881	412.85376577	116.256421881	412.85376577	5.451669441
414.07830077	116.57945195	414.07830077	116.57945195	414.07830077	5.357680151
414.64517477	92.830679593	414.64517477	92.830679593	414.64517477	4.303623836
415.27157777	82.819498246	415.27157777	82.819498246	415.27157777	3.81554356
415.86970977	79.0612456039	415.86970977	79.0612456039	415.86970977	3.599920239
415.96970977	78.4240034487	415.96970977	78.4240034487	415.96970977	3.56511745
416.06970977	77.7315779471	416.06970977	77.7315779471	416.06970977	3.528428169
416.16970977	76.9833127868	416.16970977	76.9833127868	416.16970977	3.489686231
416.26970977	76.1856720804	416.26970977	76.1856720804	416.26970977	3.448921162
416.36970977	75.3510851942	416.36970977	75.3510851942	416.36970977	3.406201125
416.46970977	74.5008586897	416.46970977	74.5008586897	416.46970977	3.361357338
416.56970977	73.7114773428	416.56970977	73.7114773428	416.56970977	3.3142036892
416.66970977	73.5302858757	416.66970977	73.5302858757	416.66970977	3.2831472399
416.76970977	73.510294235	416.76970977	73.510294235	416.76970977	3.2629516422
416.86970977	73.1230138	416.86970977	73.1230138	416.86970977	3.2375721136
416.96970977	73.552055978	416.96970977	73.552055978	416.96970977	3.2496476317
417.57032977	98.810111685	417.57032977	98.810111685	417.57032977	3.942575158
417.85376577	100.565014002	417.85376577	100.565014002	417.85376577	4.073825281
419.07830077	61.2248970559	419.07830077	61.2248970559	419.07830077	2.7244775972
419.64517477	57.500611249	419.64517477	57.500611249	419.64517477	2.5657584636
420.27157777	54.5564200319	420.27157777	54.5564200319	420.27157777	2.4346230511
420.86970977	52.685953939	420.86970977	52.685953939	420.86970977	2.343996375
422.57032977	54.2514475885	422.57032977	54.2514475885	422.57032977	2.3258805693
422.85376577	53.0800647078	422.85376577	53.0800647078	422.85376577	2.2793792025
424.07830077	46.9848871795	424.07830077	46.9848871795	424.07830077	2.0477411566
424.64517477	45.3150845219	424.64517477	45.3150845219	424.64517477	1.9779590533
425.27157777	54.358294901	425.27157777	54.358294901	425.27157777	2.2212094235
425.86970977	55.697778739	425.86970977	55.697778739	425.86970977	2.2084508404
427.57032977	39.7583279839	427.57032977	39.7583279839	427.57032977	1.7420325465
427.85376577	39.3387088749	427.85376577	39.3387088749	427.85376577	1.7240431583
429.07830077	37.5022996045	429.07830077	37.5022996045	429.07830077	1.6474031733
429.64517477	36.6858876882	429.64517477	36.6858876882	429.64517477	1.6138722403

Final State: j’=4		Final State: j’=3		Final State: j’=2	
E(cm⁻¹)	σ (Å²)	E(cm⁻¹)	σ (Å²)	E(cm⁻¹)	σ (Å²)
430.27157777	35.8433143113	430.27157777	35.8433143113	430.27157777	1.5792340779
430.86970977	35.1105261232	430.86970977	35.1105261232	430.86970977	1.548869062
432.57032977	33.8355965785	432.57032977	33.8355965785	432.57032977	1.4886717285
432.85376577	34.0106364103	432.85376577	34.0106364103	432.85376577	1.490142115
434.07830077	34.5142687164	434.07830077	34.5142687164	434.07830077	1.4894576879
434.64517477	33.0675532205	434.64517477	33.0675532205	434.64517477	1.442280928
435.27157777	31.8927657253	435.27157777	31.8927657253	435.27157777	1.4029975414
435.86970977	31.0933978369	435.86970977	31.0933978369	435.86970977	1.3748676199
437.57032977	29.4716097511	437.57032977	29.4716097511	437.57032977	1.3146108583
437.85376577	29.2482296711	437.85376577	29.2482296711	437.85376577	1.3060866098
439.07830077	28.3853590631	439.07830077	28.3853590631	439.07830077	1.2727238382
439.64517477	28.0413748079	439.64517477	28.0413748079	439.64517477	1.2591347348
440.27157777	27.7053264425	440.27157777	27.7053264425	440.27157777	1.245552846
440.86970977	27.4260717445	440.86970977	27.4260717445	440.86970977	1.2339380339
442.57032977	26.6950278686	442.57032977	26.6950278686	442.57032977	1.203712782
442.85376577	26.5599658963	442.85376577	26.5599658963	442.85376577	1.1984569146
444.07830077	25.9416015389	444.07830077	25.9416015389	444.07830077	1.1752272222
444.64517477	25.6545153016	444.64517477	25.6545153016	444.64517477	1.1646717223
445.27157777	25.3465762674	445.27157777	25.3465762674	445.27157777	1.1534097155
445.86970977	25.0643974862	445.86970977	25.0643974862	445.86970977	1.1431153563
447.57032977	24.3307012628	447.57032977	24.3307012628	447.57032977	1.1163715099
447.85376577	24.218108389	447.85376577	24.218108389	447.85376577	1.1122650511
449.07830077	23.7610405922	449.07830077	23.7610405922	449.07830077	1.0955848945
449.64517477	23.5641796879	449.64517477	23.5641796879	449.64517477	1.0884017106
450.27157777	23.3559111785	450.27157777	23.3559111785	450.27157777	1.0808134989
450.86970977	23.1647986708	450.86970977	23.1647986708	450.86970977	1.0738714636
452.57032977	22.6524798807	452.57032977	22.6524798807	452.57032977	1.055449722
452.85376577	22.5706187722	452.85376577	22.5706187722	452.85376577	1.0525396875
454.07830077	22.226734723	454.07830077	22.226734723	454.07830077	1.0404290415
454.64517477	22.0728049034	454.64517477	22.0728049034	454.64517477	1.0350675752
455.27157777	21.9066336686	455.27157777	21.9066336686	455.27157777	1.0293197839
455.86970977	21.7518832976	455.86970977	21.7518832976	455.86970977	1.0240033456
457.57032977	21.3332670149	457.57032977	21.3332670149	457.57032977	1.0097916862
457.85376577	21.2665812673	457.85376577	21.2665812673	457.85376577	1.0075505412
459.07830077	20.9883777773	459.07830077	20.9883777773	459.07830077	0.9982706474
459.64517477	20.8648708219	459.64517477	20.8648708219	459.64517477	0.9941886527
460.27157777	20.7321027657	460.27157777	20.7321027657	460.27157777	0.9898283119
460.86970977	20.6088091068	460.86970977	20.6088091068	460.86970977	0.9858063844
462.57032977	20.2754490146	462.57032977	20.2754490146	462.57032977	0.9750767407
462.85376577	20.2221885247	462.85376577	20.2221885247	462.85376577	0.9733837527
464.07830077	19.9989987148	464.07830077	19.9989987148	464.07830077	0.9663589868
464.64517477	19.8992839186	464.64517477	19.8992839186	464.64517477	0.9632587905
465.27157777	19.7916054192	465.27157777	19.7916054192	465.27157777	0.959938944
465.86970977	19.691153881	465.86970977	19.691153881	465.86970977	0.9568689942
467.57032977	19.4175138342	467.57032977	19.4175138342	467.57032977	0.9486459006
467.85376577	19.373554924	467.85376577	19.373554924	467.85376577	0.9473448151
469.07830077	19.1887831373	469.07830077	19.1887831373	469.07830077	0.9419396138
469.64517477	19.1059890488	469.64517477	19.1059890488	469.64517477	0.93955211
470.27157777	19.0164468046	470.27157777	19.0164468046	470.27157777	0.9369950748
470.86970977	18.9328008963	470.86970977	18.9328008963	470.86970977	0.9346306843
472.57032977	18.7044360084	472.57032977	18.7044360084	472.57032977	0.9283012051

Final State: j'=4		Final State: j'=3		Final State: j'=2	
E(cm⁻¹)	σ (Å²)	E(cm⁻¹)	σ (Å²)	E(cm⁻¹)	σ (Å²)
472.85376577	18.6676800998	472.85376577	18.6676800998	472.85376577	0.9273004411
474.07830077	18.5129467523	474.07830077	18.5129467523	474.07830077	0.9231452628
474.64517477	18.4434743439	474.64517477	18.4434743439	474.64517477	0.9213111093
475.27157777	18.368231304	475.27157777	18.368231304	475.27157777	0.9193474689
475.86970977	18.2978336216	475.86970977	18.2978336216	475.86970977	0.9175324074
477.57032977	18.1050461393	477.57032977	18.1050461393	477.57032977	0.9126761892
477.85376577	18.0739303939	477.85376577	18.0739303939	477.85376577	0.9119087017
479.07830077	17.9426663727	479.07830077	17.9426663727	479.07830077	0.9087231159
479.64517477	17.8835851668	479.64517477	17.8835851668	479.64517477	0.9073175377
480.27157777	17.8194938345	480.27157777	17.8194938345	480.27157777	0.9058131775
480.86970977	17.759433493	480.86970977	17.759433493	480.86970977	0.9044231095
482.57032977	17.5944828336	482.57032977	17.5944828336	482.57032977	0.9007065611
482.85376577	17.5677955214	482.85376577	17.5677955214	482.85376577	0.9001195592
484.07830077	17.4550160909	484.07830077	17.4550160909	484.07830077	0.8976843021
484.64517477	17.4041500293	484.64517477	17.4041500293	484.64517477	0.8966103657
485.27157777	17.3488963008	485.27157777	17.3488963008	485.27157777	0.8954613212
485.86970977	17.2970463054	485.86970977	17.2970463054	485.86970977	0.8943998437
487.57032977	17.1542787817	487.57032977	17.1542787817	487.57032977	0.8915623217
487.85376577	17.1311283274	487.85376577	17.1311283274	487.85376577	0.8911140891
489.07830077	17.0331260784	489.07830077	17.0331260784	489.07830077	0.8892535004
489.64517477	16.9888318833	489.64517477	16.9888318833	489.64517477	0.888432107
490.27157777	16.9406480035	490.27157777	16.9406480035	490.27157777	0.8875523133
490.86970977	16.8953648527	490.86970977	16.8953648527	490.86970977	0.8867383865
492.57032977	16.7703176347	492.57032977	16.7703176347	492.57032977	0.8845537984
492.85376577	16.749987671	492.85376577	16.749987671	492.85376577	0.884207056
494.07830077	16.6637482956	494.07830077	16.6637482956	494.07830077	0.8827605627
494.64517477	16.6246713481	494.64517477	16.6246713481	494.64517477	0.882117271
495.27157777	16.5820872239	495.27157777	16.5820872239	495.27157777	0.8814240106
495.86970977	16.5419909939	495.86970977	16.5419909939	495.86970977	0.8807779107
497.57032977	16.4308435279	497.57032977	16.4308435279	497.57032977	0.8790127945
497.85376577	16.4127082081	497.85376577	16.4127082081	497.85376577	0.8787271982
499.07830077	16.3355464212	499.07830077	16.3355464212	499.07830077	0.8775135851
499.64517477	16.3004456055	499.64517477	16.3004456055	499.64517477	0.8769596957
500.27157777	16.2620846646	500.27157777	16.2620846646	500.27157777	0.8763504372
500.86970977	16.2258485543	500.86970977	16.2258485543	500.86970977	0.8757688933
502.57032977	16.124693609	502.57032977	16.124693609	502.57032977	0.8740906677
502.85376577	16.1080710437	502.85376577	16.1080710437	502.85376577	0.8738034789
504.07830077	16.0368749694	504.07830077	16.0368749694	504.07830077	0.8725180049
504.64517477	16.004187375	504.64517477	16.004187375	504.64517477	0.8718890942
505.27157777	15.9681960525	505.27157777	15.9681960525	505.27157777	0.8711594956
505.86970977	15.9338918519	505.86970977	15.9338918519	505.86970977	0.8704198316
507.57032977	15.835925097	507.57032977	15.835925097	507.57032977	0.867975781
507.85376577	15.8193997493	507.85376577	15.8193997493	507.85376577	0.8674993981
509.07830077	15.7464714489	509.07830077	15.7464714489	509.07830077	0.8650832295
509.64517477	15.711329018	509.64517477	15.711329018	509.64517477	0.8636883165
510.27157777	15.6707313027	510.27157777	15.6707313027	510.27157777	0.8618345443
510.86970977	15.6292330048	510.86970977	15.6292330048	510.86970977	0.8596157281
512.57032977	15.4683632248	512.57032977	15.4683632248	512.57032977	0.8472866227
512.85376577	15.4216664415	512.85376577	15.4216664415	512.85376577	0.8429100752
514.07830077	15.2041366207	514.07830077	15.2041366207	514.07830077	0.8538939965
515.27157777	15.2937576925	515.27157777	15.2937576925	514.64517477	0.8850968337

Final State: j'=4		Final State: j'=3		Final State: j'=2	
E(cm⁻¹)	σ (Å²)	E(cm⁻¹)	σ (Å²)	E(cm⁻¹)	σ (Å²)
515.86970977	15.2338559943	515.86970977	15.2338559943	515.27157777	0.8587433343
517.57032977	15.1442979858	517.57032977	15.1442979858	515.86970977	0.8782881469
517.85376577	15.0711756981	517.85376577	15.0711756981	517.57032977	0.8450118741
519.07830077	15.3752550415	519.07830077	15.3752550415	517.85376577	0.8553310639
519.64517477	15.2058546851	519.64517477	15.2058546851	519.07830077	0.8950583394
520.27157777	15.3275250466	520.27157777	15.3275250466	519.64517477	0.8727425014
520.86970977	15.1050670893	520.86970977	15.1050670893	520.27157777	0.8887184885
522.57032977	15.1929340441	522.57032977	15.1929340441	520.86970977	0.8608712046
522.85376577	15.1049583126	522.85376577	15.1049583126	522.57032977	0.8710024666
524.07830077	15.203804352	524.07830077	15.203804352	522.85376577	0.906665643
524.64517477	15.0554223051	524.64517477	15.0554223051	524.07830077	0.8857985792
525.27157777	14.7661512555	525.27157777	14.7661512555	524.64517477	0.8686899791
525.86970977	15.0860631034	525.86970977	15.0860631034	525.27157777	0.8690872699
527.57032977	15.241987386	527.57032977	15.241987386	525.86970977	0.8989608668
527.85376577	15.1896783802	527.85376577	15.1896783802	527.57032977	0.9121421506
529.07830077	14.9883606117	529.07830077	14.9883606117	527.85376577	0.9040609167
529.64517477	14.7845252122	529.64517477	14.7845252122	529.07830077	0.8797842404
530.27157777	14.7210474806	530.27157777	14.7210474806	529.64517477	0.861005527
530.86970977	15.0635602157	530.86970977	15.0635602157	530.27157777	0.904021529
532.57032977	15.0919272773	532.57032977	15.0919272773	530.86970977	0.9006282427
532.85376577	15.0523404659	532.85376577	15.0523404659	532.57032977	0.9127231012
534.07830077	14.903641849	534.07830077	14.903641849	532.85376577	0.9074786502
534.64517477	14.8047633786	534.64517477	14.8047633786	534.07830077	0.8908384535
535.27157777	14.5258561314	535.27157777	14.5258561314	534.64517477	0.8806319642
535.86970977	14.6222536561	535.86970977	14.6222536561	535.27157777	0.8593026333
537.57032977	15.0477730319	537.57032977	15.0477730319	535.86970977	0.907802451
537.85376577	14.9834664807	537.85376577	14.9834664807	537.57032977	0.9301499881
539.07830077	14.8388138606	539.07830077	14.8388138606	537.85376577	0.9199364902
539.64517477	14.7840739518	539.64517477	14.7840739518	539.07830077	0.9026014654
540.27157777	14.7143580736	540.27157777	14.7143580736	539.64517477	0.8970727816
540.86970977	14.6130236303	540.86970977	14.6130236303	540.27157777	0.8901159546
542.57032977	14.6154123492	542.57032977	14.6154123492	540.86970977	0.8799978482
542.85376577	14.5583470585	542.85376577	14.5583470585	542.57032977	0.9022597539
544.07830077	14.7724886867	544.07830077	14.7724886867	542.85376577	0.9211911062
544.64517477	14.6949164222	544.64517477	14.6949164222	544.07830077	0.9114949559
545.27157777	14.6235430169	545.27157777	14.6235430169	544.64517477	0.9018154569
545.86970977	14.5506541306	545.86970977	14.5506541306	545.27157777	0.8934160673
547.57032977	14.1831702451	547.57032977	14.1831702451	545.86970977	0.8847694817
547.85376577	14.22519545	547.85376577	14.22519545	547.57032977	0.8723733481
549.07830077	14.382357389	549.07830077	14.382357389	547.85376577	0.8849643242
549.64517477	14.428457137	549.64517477	14.428457137	549.07830077	0.9184865729
550.27157777	14.5783356439	550.27157777	14.5783356439	549.64517477	0.9485590547
550.86970977	14.345261082	550.86970977	14.345261082	550.27157777	0.9229178916
552.57032977	14.334984828	552.57032977	14.334984828	550.86970977	0.9186581507
552.85376577	14.1122343909	552.85376577	14.1122343909	552.57032977	0.8934276793
554.07830077	14.5754457002	554.07830077	14.5754457002	552.85376577	0.8788342765
554.64517477	14.4337456292	554.64517477	14.4337456292	554.07830077	0.9525903261
555.27157777	14.2026801407	555.27157777	14.2026801407	554.64517477	0.9210451168
555.86970977	13.4918084762	555.86970977	13.4918084762	555.27157777	0.8882393164
557.57032977	14.772582708	557.57032977	14.772582708	555.86970977	0.8474925981
557.85376577	14.6255996566	557.85376577	14.6255996566	557.57032977	0.9759163417

Final State: j'=4		Final State: j'=3		Final State: j'=2	
E(cm⁻¹)	σ (Å²)	E(cm⁻¹)	σ (Å²)	E(cm⁻¹)	σ (Å²)
559.07830077	14.1616437535	559.07830077	14.1616437535	557.85376577	0.9528271196
559.64517477	13.9886843909	559.64517477	13.9886843909	559.07830077	0.8921228445
560.27157777	14.6506224722	560.27157777	14.6506224722	559.64517477	0.9217466796
560.86970977	14.5080459971	560.86970977	14.5080459971	560.27157777	0.9983704123
562.57032977	14.1715183836	562.57032977	14.1715183836	560.86970977	0.9558366705
562.85376577	14.0482139857	562.85376577	14.0482139857	562.57032977	0.9053122932
564.07830077	14.3093674957	564.07830077	14.3093674957	562.85376577	0.8911053339
564.64517477	14.2979222136	564.64517477	14.2979222136	564.07830077	0.9866866484
565.27157777	14.2773974271	565.27157777	14.2773974271	564.64517477	0.9422791928
565.86970977	14.1788737553	565.86970977	14.1788737553	565.27157777	0.9429384852
567.57032977	13.6805944942	567.57032977	13.6805944942	565.86970977	0.931020921
567.85376577	13.5381711503	567.85376577	13.5381711503	567.57032977	0.8793271334
569.07830077	14.3038103431	569.07830077	14.3038103431	567.85376577	0.8972806425
569.64517477	14.0417048948	569.64517477	14.0417048948	569.07830077	0.9505366527
570.27157777	14.3230769978	570.27157777	14.3230769978	569.64517477	0.957206667
570.86970977	14.1198825603	570.86970977	14.1198825603	570.27157777	0.9736465614
572.57032977	13.5508057128	572.57032977	13.5508057128	570.86970977	0.9308437821
572.85376577	14.0843641259	572.85376577	14.0843641259	572.57032977	0.9519581306
574.07830077	14.4137819227	574.07830077	14.4137819227	572.85376577	0.9784892533
574.64517477	14.1114429825	574.64517477	14.1114429825	574.07830077	0.9942303587
575.27157777	13.9383945079	575.27157777	13.9383945079	574.64517477	0.9489126503
575.86970977	13.8639399588	575.86970977	13.8639399588	575.27157777	0.9296564775
577.57032977	13.5075944687	577.57032977	13.5075944687	575.86970977	0.9447676449
577.85376577	13.8124011762	577.85376577	13.8124011762	577.57032977	0.9359546979
579.07830077	13.6576447593	579.07830077	13.6576447593	577.85376577	0.9481885049
579.64517477	13.5775469356	579.64517477	13.5775469356	579.07830077	0.9233298511
580.27157777	13.4689450235	580.27157777	13.4689450235	579.64517477	0.9160859165
580.86970977	13.253339896	580.86970977	13.253339896	580.27157777	0.907619566
582.57032977	13.5485105401	582.57032977	13.5485105401	580.86970977	0.901482525
582.85376577	13.547570565	582.85376577	13.547570565	582.57032977	0.9286074331
584.07830077	13.4762691277	584.07830077	13.4762691277	582.85376577	0.9273655446
584.64517477	13.4449410211	584.64517477	13.4449410211	584.07830077	0.9222843056
585.27157777	13.4034246202	585.27157777	13.4034246202	584.64517477	0.9207955225
585.86970977	13.3480986072	585.86970977	13.3480986072	585.27157777	0.9187510062
587.57032977	13.3007815805	587.57032977	13.3007815805	585.86970977	0.9158712487
587.85376577	13.2989062786	587.85376577	13.2989062786	587.57032977	0.9178970944
589.07830077	13.2195608806	589.07830077	13.2195608806	587.85376577	0.9184499047
589.64517477	13.1208768079	589.64517477	13.1208768079	589.07830077	0.9150818684
590.27157777	13.282126903	590.27157777	13.282126903	589.64517477	0.9131690882
590.86970977	13.2982396985	590.86970977	13.2982396985	590.27157777	0.9256036678
592.57032977	13.2374300025	592.57032977	13.2374300025	590.86970977	0.926557693
592.85376577	13.2308650273	592.85376577	13.2308650273	592.57032977	0.9261254222
594.07830077	13.2049273495	594.07830077	13.2049273495	592.85376577	0.9263597651
594.64517477	13.1930528823	594.64517477	13.1930528823	594.07830077	0.9275541096
595.27157777	13.1793399462	595.27157777	13.1793399462	594.64517477	0.9281144516
595.86970977	13.1648868903	595.86970977	13.1648868903	595.27157777	0.9286890152
597.57032977	13.0964384261	597.57032977	13.0964384261	595.86970977	0.9291379605
597.85376577	13.092812715	597.85376577	13.092812715	597.57032977	0.9285465533
599.07830077	13.1072191158	599.07830077	13.1072191158	597.85376577	0.9290743319
599.64517477	13.1028791461	599.64517477	13.1028791461	599.07830077	0.9332255447
600.27157777	13.0949968637	600.27157777	13.0949968637	599.64517477	0.9343081535

Final State: j'=4		Final State: j'=3		Final State: j'=2	
E(cm⁻¹)	σ (Å²)	E(cm⁻¹)	σ (Å²)	E(cm⁻¹)	σ (Å²)
600.86970977	13.0863300596	600.86970977	13.0863300596	600.27157777	0.9352930416
602.57032977	13.0596746432	602.57032977	13.0596746432	600.86970977	0.9361576623
602.85376577	13.0550594498	602.85376577	13.0550594498	602.57032977	0.9384839514
604.07830077	13.0344732697	604.07830077	13.0344732697	602.85376577	0.9388599936
604.64517477	13.0245804186	604.64517477	13.0245804186	604.07830077	0.9404388528
605.27157777	13.0135936318	605.27157777	13.0135936318	604.64517477	0.9411436835
605.86970977	13.0034473597	605.86970977	13.0034473597	605.27157777	0.9419181594
607.57032977	12.9782674877	607.57032977	12.9782674877	605.86970977	0.9426817289
607.85376577	12.9744173725	607.85376577	12.9744173725	607.57032977	0.9451109593
609.07830077	12.9579091134	609.07830077	12.9579091134	607.85376577	0.9455399396
609.64517477	12.9502056905	609.64517477	12.9502056905	609.07830077	0.9474004224
610.27157777	12.9416196629	610.27157777	12.9416196629	609.64517477	0.9482565134
610.86970977	12.9333528604	610.86970977	12.9333528604	610.27157777	0.9491967537
612.57032977	12.9095717968	612.57032977	12.9095717968	610.86970977	0.9500892227
612.85376577	12.9055817599	612.85376577	12.9055817599	612.57032977	0.9526043282
614.07830077	12.8883283014	614.07830077	12.8883283014	612.85376577	0.9530211944
614.64517477	12.8803639338	614.64517477	12.8803639338	614.07830077	0.9548195019
615.27157777	12.8716058564	615.27157777	12.8716058564	614.64517477	0.9556526932
615.86970977	12.8632991399	615.86970977	12.8632991399	615.27157777	0.9565757602
617.57032977	12.8400303602	617.57032977	12.8400303602	615.86970977	0.9574605484
617.85376577	12.8362012204	617.85376577	12.8362012204	617.57032977	0.9599983113
619.07830077	12.819794792	619.07830077	12.819794792	617.85376577	0.9604244265
619.64517477	12.8122645721	619.64517477	12.8122645721	619.07830077	0.9622741704
620.27157777	12.8039834745	620.27157777	12.8039834745	619.64517477	0.963134611
620.86970977	12.7961108528	620.86970977	12.7961108528	620.27157777	0.9640879173
622.57032977	12.7738876667	622.57032977	12.7738876667	620.86970977	0.9650003504
622.85376577	12.7702051072	622.85376577	12.7702051072	622.57032977	0.9676042946
624.07830077	12.7543633672	624.07830077	12.7543633672	622.85376577	0.9680395534
624.64517477	12.7470678244	624.64517477	12.7470678244	624.07830077	0.9699240298
625.27157777	12.7390350447	625.27157777	12.7390350447	624.64517477	0.9707986705
625.86970977	12.7313940251	625.86970977	12.7313940251	625.27157777	0.9717668865
627.57032977	12.7098313556	627.57032977	12.7098313556	625.86970977	0.9726931713
627.85376577	12.7062615394	627.85376577	12.7062615394	627.57032977	0.9753367911
629.07830077	12.6909185524	629.07830077	12.6909185524	627.85376577	0.9757788861
629.64517477	12.68385956	629.64517477	12.68385956	629.07830077	0.9776939075
630.27157777	12.6760911349	630.27157777	12.6760911349	629.64517477	0.9785832062
630.86970977	12.6687041156	630.86970977	12.6687041156	630.27157777	0.9795679344
632.57032977	12.6478619851	632.57032977	12.6478619851	630.86970977	0.9805102066
632.85376577	12.6444108922	632.85376577	12.6444108922	632.57032977	0.9831996901
634.07830077	12.6295731062	634.07830077	12.6295731062	632.85376577	0.9836494066
634.64517477	12.6227431964	634.64517477	12.6227431964	634.07830077	0.985597039
635.27157777	12.6152242536	635.27157777	12.6152242536	634.64517477	0.9865012112
635.86970977	12.6080719817	635.86970977	12.6080719817	635.27157777	0.9875021912
637.57032977	12.5878799763	637.57032977	12.5878799763	635.86970977	0.9884598036
637.85376577	12.5845350386	637.85376577	12.5845350386	637.57032977	0.991192047
639.07830077	12.570150014	639.07830077	12.570150014	637.85376577	0.9916487869
639.64517477	12.563526847	639.64517477	12.563526847	639.07830077	0.9936264895
640.27157777	12.556234429	640.27157777	12.556234429	639.64517477	0.9945444705
640.86970977	12.5492968129	640.86970977	12.5492968129	640.27157777	0.9955606443
642.57032977	12.5297069041	642.57032977	12.5297069041	640.86970977	0.9965327105
642.85376577	12.5264611861	642.85376577	12.5264611861	642.57032977	0.9993058334

Final State: j’=4		Final State: j’=3		Final State: j’=2	
E(cm⁻¹)	σ (Å²)	E(cm⁻¹)	σ (Å²)	E(cm⁻¹)	σ (Å²)
644.07830077	12.512501124	644.07830077	12.512501124	642.85376577	0.9997693577
644.64517477	12.5060726292	644.64517477	12.5060726292	644.07830077	1.0017763037
645.27157777	12.4989938193	645.27157777	12.4989938193	644.64517477	1.002707772
645.86970977	12.4922585865	645.86970977	12.4922585865	645.27157777	1.003738817
647.57032977	12.4732357793	647.57032977	12.4732357793	645.86970977	1.0047250439
647.85376577	12.4700833511	647.85376577	12.4700833511	647.57032977	1.007538217
649.07830077	12.4565223778	649.07830077	12.4565223778	647.85376577	1.0080083884
649.64517477	12.4502764879	649.64517477	12.4502764879	649.07830077	1.0100439377
650.27157777	12.4433978973	650.27157777	12.4433978973	649.64517477	1.0109885937
650.86970977	12.4368523299	650.86970977	12.4368523299	650.27157777	1.01203417
652.57032977	12.4183609893	652.57032977	12.4183609893	650.86970977	1.0130342381
652.85376577	12.4152960739	652.85376577	12.4152960739	652.57032977	1.0158865941
654.07830077	12.4021097495	654.07830077	12.4021097495	652.85376577	1.0163632716
654.64517477	12.3960354759	654.64517477	12.3960354759	654.07830077	1.0184268841
655.27157777	12.3893452629	655.27157777	12.3893452629	654.64517477	1.0193845018
655.86970977	12.3829783328	655.86970977	12.3829783328	655.27157777	1.020444388
657.57032977	12.3649886077	657.57032977	12.3649886077	655.86970977	1.0214581028
657.85376577	12.362006413	657.85376577	12.362006413	657.57032977	1.0243492168
659.07830077	12.3491746439	659.07830077	12.3491746439	657.85376577	1.0248323477
659.64517477	12.3432630015	659.64517477	12.3432630015	659.07830077	1.0269238376
660.27157777	12.3367514047	660.27157777	12.3367514047	659.64517477	1.0278943705
660.86970977	12.3305540471	660.86970977	12.3305540471	660.27157777	1.0289685277
662.57032977	12.3130412151	662.57032977	12.3130412151	660.86970977	1.0299958841
662.85376577	12.3101377426	662.85376577	12.3101377426	662.57032977	1.0329258547
664.07830077	12.2976439107	664.07830077	12.2976439107	662.85376577	1.0334154769
664.64517477	12.2918874768	664.64517477	12.2918874768	664.07830077	1.0355350724
665.27157777	12.2855465708	665.27157777	12.2855465708	664.64517477	1.0365186533
665.86970977	12.2795113634	665.86970977	12.2795113634	665.27157777	1.0376072666
667.57032977	12.2624555029	667.57032977	12.2624555029	665.86970977	1.0386484631
667.85376577	12.2596276985	667.85376577	12.2596276985	667.57032977	1.0416180418
669.07830077	12.2474590764	669.07830077	12.2474590764	667.85376577	1.0421143062
669.64517477	12.2418523918	669.64517477	12.2418523918	669.07830077	1.0442627648
670.27157777	12.2356763657	670.27157777	12.2356763657	669.64517477	1.0452598007
670.86970977	12.229798145	670.86970977	12.229798145	670.27157777	1.0463633609
672.57032977	12.2131863538	672.57032977	12.2131863538	670.86970977	1.047418917
672.85376577	12.2104322588	672.85376577	12.2104322588	672.57032977	1.050429816
674.07830077	12.1985815017	674.07830077	12.1985815017	672.85376577	1.0509330482
674.64517477	12.1931216892	674.64517477	12.1931216892	674.07830077	1.0531119066
675.27157777	12.1871078637	675.27157777	12.1871078637	674.64517477	1.0541231997
675.86970977	12.1813844685	675.86970977	12.1813844685	675.27157777	1.0552426615
677.57032977	12.1652131914	677.57032977	12.1652131914	675.86970977	1.0563135481
677.85376577	12.1625326473	677.85376577	12.1625326473	677.57032977	1.0593689635
679.07830077	12.1510004229	679.07830077	12.1510004229	677.85376577	1.0598797609
679.64517477	12.1456886211	679.64517477	12.1456886211	679.07830077	1.0620918409
680.27157777	12.1398389091	680.27157777	12.1398389091	679.64517477	1.0631188324
680.86970977	12.1342728436	680.86970977	12.1342728436	680.27157777	1.0642559065
682.57032977	12.1185534652	682.57032977	12.1185534652	680.86970977	1.0653438853
682.85376577	12.1159490456	682.85376577	12.1159490456	682.57032977	1.0684495645
684.07830077	12.1047490832	684.07830077	12.1047490832	682.85376577	1.0689690091
684.64517477	12.0995932747	684.64517477	12.0995932747	684.07830077	1.0712194548
685.27157777	12.0939177754	685.27157777	12.0939177754	684.64517477	1.0722648168

Final State: j’=4		Final State: j’=3		Final State: j’=2	
E(cm⁻¹)	σ (Å²)	E(cm⁻¹)	σ (Å²)	E(cm⁻¹)	σ (Å²)
685.86970977	12.0885201316	685.86970977	12.0885201316	685.27157777	1.0734226873
687.57032977	12.0732929572	687.57032977	12.0732929572	685.86970977	1.0745310659
687.85376577	12.0707728585	687.85376577	12.0707728585	687.57032977	1.0776980287
689.07830077	12.0599463181	689.07830077	12.0599463181	687.85376577	1.078228226
689.64517477	12.0549692118	689.64517477	12.0549692118	689.07830077	1.0805272235
690.27157777	12.0494962407	690.27157777	12.0494962407	689.64517477	1.0815963635
690.86970977	12.0442975796	690.86970977	12.0442975796	690.27157777	1.0827816062
692.57032977	12.0296733602	692.57032977	12.0296733602	690.86970977	1.0839173181
692.85376577	12.0272602229	692.85376577	12.0272602229	692.57032977	1.0871697209
694.07830077	12.0169230302	694.07830077	12.0169230302	692.85376577	1.0877154813
694.64517477	12.0121902504	694.64517477	12.0121902504	694.07830077	1.0900871582
695.27157777	12.0070032153	695.27157777	12.0070032153	694.64517477	1.0911934593
695.86970977	12.0020965216	695.86970977	12.0020965216	695.27157777	1.0924226402
697.57032977	11.9884358345	697.57032977	11.9884358345	695.86970977	1.0936044289
697.85376577	11.986208632	697.85376577	11.986208632	697.57032977	1.0970125846
699.07830077	11.9767962297	699.07830077	11.9767962297	697.85376577	1.097589073
699.64517477	11.9725800162	699.64517477	11.9725800162	699.07830077	1.1001160332
700.27157777	11.9680563873	700.27157777	11.9680563873	699.64517477	1.1013104346
700.86970977	11.9639050625	700.86970977	11.9639050625	700.27157777	1.1026539462
702.57032977	11.9536163583	702.57032977	11.9536163583	700.86970977	1.1039661919
702.85376577	11.95228064	702.85376577	11.95228064	702.57032977	1.1079571255
704.07830077	11.9500097565	704.07830077	11.9500097565	702.85376577	1.108685392
704.64517477	11.9553315815	704.64517477	11.9553315815	704.07830077	1.1123524214
705.27157777	11.9612049551	705.27157777	11.9612049551	704.64517477	1.1147172325
705.86970977	11.9379728789	705.86970977	11.9379728789	705.27157777	1.113654325
707.57032977	11.907169699	707.57032977	11.907169699	705.86970977	1.1081081314
707.85376577	11.8927041258	707.85376577	11.8927041258	707.57032977	1.1092454649
709.07830077	11.9208559088	709.07830077	11.9208559088	707.85376577	1.1116754436
709.64517477	11.9117075513	709.64517477	11.9117075513	709.07830077	1.1204265457
710.27157777	11.8747297353	710.27157777	11.8747297353	709.64517477	1.1163834947
710.86970977	11.8687837555	710.86970977	11.8687837555	710.27157777	1.116129245
712.57032977	11.8903171835	712.57032977	11.8903171835	710.86970977	1.1174166997
712.85376577	11.8942533666	712.85376577	11.8942533666	712.57032977	1.127184769
714.07830077	11.851116163	714.07830077	11.851116163	712.85376577	1.1250368831
714.64517477	11.8252412888	714.64517477	11.8252412888	714.07830077	1.126723681
715.27157777	11.8272262443	715.27157777	11.8272262443	714.64517477	1.1200423804
715.86970977	11.8362314847	715.86970977	11.8362314847	715.27157777	1.1273613986
717.57032977	11.8030971904	717.57032977	11.8030971904	715.86970977	1.1310743579
717.85376577	11.8122520719	717.85376577	11.8122520719	717.57032977	1.1281421944
719.07830077	11.7831025044	719.07830077	11.7831025044	717.85376577	1.1282329431
719.64517477	11.7897157291	719.64517477	11.7897157291	719.07830077	1.1323214514
720.27157777	11.7946720743	720.27157777	11.7946720743	719.64517477	1.1357857988
720.86970977	11.8043354641	720.86970977	11.8043354641	720.27157777	1.1388170249
722.57032977	11.7783907579	722.57032977	11.7783907579	720.86970977	1.142111325
722.85376577	11.7681556728	722.85376577	11.7681556728	722.57032977	1.1412288783
724.07830077	11.7441618769	724.07830077	11.7441618769	722.85376577	1.130739656
724.64517477	11.7477774905	724.64517477	11.7477774905	724.07830077	1.1428004141
725.27157777	11.7502364032	725.27157777	11.7502364032	724.64517477	1.1454973992
725.86970977	11.7540706962	725.86970977	11.7540706962	725.27157777	1.148063393
727.57032977	11.7202654282	727.57032977	11.7202654282	725.86970977	1.1506323771
727.85376577	11.7266920746	727.85376577	11.7266920746	727.57032977	1.1455211291

Final State: j'=4		Final State: j'=3		Final State: j'=2	
E(cm⁻¹)	σ (Å²)	E(cm⁻¹)	σ (Å²)	E(cm⁻¹)	σ (Å²)
729.07830077	11.691735184	729.07830077	11.691735184	727.85376577	1.1486847678
729.64517477	11.7013791441	729.64517477	11.7013791441	729.07830077	1.1503586457
730.27157777	11.7041468623	730.27157777	11.7041468623	729.64517477	1.1543562142
730.86970977	11.7053143423	730.86970977	11.7053143423	730.27157777	1.1570214471
732.57032977	11.7188393545	732.57032977	11.7188393545	730.86970977	1.159243166
732.85376577	11.7319858866	732.85376577	11.7319858866	732.57032977	1.1666011315
734.07830077	11.6930206592	734.07830077	11.6930206592	732.85376577	1.1686985508
734.64517477	11.6660586701	734.64517477	11.6660586701	734.07830077	1.1638026904
735.27157777	11.6540243105	735.27157777	11.6540243105	734.64517477	1.1601522386
735.86970977	11.6632401152	735.86970977	11.6632401152	735.27157777	1.16541933
737.57032977	11.6743895017	737.57032977	11.6743895017	735.86970977	1.1691648358
737.85376577	11.6781078808	737.85376577	11.6781078808	737.57032977	1.1767917974
739.07830077	11.7018398289	739.07830077	11.7018398289	737.85376577	1.1782889328
739.64517477	11.6863283534	739.64517477	11.6863283534	739.07830077	1.1777593737
740.27157777	11.6993057196	740.27157777	11.6993057196	739.64517477	1.1788443918
740.86970977	11.6520198054	740.86970977	11.6520198054	740.27157777	1.1751717323
742.57032977	11.6258462365	742.57032977	11.6258462365	740.86970977	1.1720192697
742.85376577	11.5945150162	742.85376577	11.5945150162	742.57032977	1.1701173242
744.07830077	11.6300997449	744.07830077	11.6300997449	742.85376577	1.1697228619
744.64517477	11.6529607788	744.64517477	11.6529607788	744.07830077	1.1884264497
745.27157777	11.5726128144	745.27157777	11.5726128144	744.64517477	1.1839075917
745.86970977	11.5820554171	745.86970977	11.5820554171	745.27157777	1.1706438103
747.57032977	11.673787954	747.57032977	11.673787954	745.86970977	1.1851829246
747.85376577	11.593014706	747.85376577	11.593014706	747.57032977	1.1866255475
749.07830077	11.5124481792	749.07830077	11.5124481792	747.85376577	1.170897668
749.64517477	11.545433991	749.64517477	11.545433991	749.07830077	1.185349879
750.27157777	11.5682524838	750.27157777	11.5682524838	749.64517477	1.1935971226
750.86970977	11.6210407214	750.86970977	11.6210407214	750.27157777	1.19955015
752.57032977	11.5227143665	752.57032977	11.5227143665	750.86970977	1.2023204642
752.85376577	11.5282349099	752.85376577	11.5282349099	752.57032977	1.1986984427
754.07830077	11.555255194	754.07830077	11.555255194	752.85376577	1.2009282367
754.64517477	11.6039314957	754.64517477	11.6039314957	754.07830077	1.2098858196
755.27157777	11.566889239	755.27157777	11.566889239	754.64517477	1.2168267291
755.86970977	11.4740613177	755.86970977	11.4740613177	755.27157777	1.1964241449
757.57032977	11.5132017107	757.57032977	11.5132017107	755.86970977	1.1974005245
757.85376577	11.5106734861	757.85376577	11.5106734861	757.57032977	1.208662235
759.07830077	11.5751857526	759.07830077	11.5751857526	757.85376577	1.2101344876
759.64517477	11.4822438263	759.64517477	11.4822438263	759.07830077	1.2235883744
760.27157777	11.4449969368	760.27157777	11.4449969368	759.64517477	1.1908970738
760.86970977	11.5158471983	760.86970977	11.5158471983	760.27157777	1.2087367966
762.57032977	11.5003456755	762.57032977	11.5003456755	760.86970977	1.2230788971
762.85376577	11.5458951524	762.85376577	11.5458951524	762.57032977	1.227978612
764.07830077	11.4909605958	764.07830077	11.4909605958	762.85376577	1.224866312
764.64517477	11.4214487624	764.64517477	11.4214487624	764.07830077	1.2124059952
765.27157777	11.4230742817	765.27157777	11.4230742817	764.64517477	1.2168556288
765.86970977	11.4126563905	765.86970977	11.4126563905	765.27157777	1.2092483577
767.57032977	11.4235390699	767.57032977	11.4235390699	765.86970977	1.2228463999
767.85376577	11.4297819993	767.85376577	11.4297819993	767.57032977	1.2144115242
769.07830077	11.4229291837	769.07830077	11.4229291837	767.85376577	1.2278829593
769.64517477	11.4024513626	769.64517477	11.4024513626	769.07830077	1.2212397575
770.27157777	11.4094085169	770.27157777	11.4094085169	769.64517477	1.2264830194

Final State: j’=4		Final State: j’=3		Final State: j’=2	
E(cm⁻¹)	σ (Å²)	E(cm⁻¹)	σ (Å²)	E(cm⁻¹)	σ (Å²)
770.86970977	11.4141095929	770.86970977	11.4141095929	770.27157777	1.2304277509
772.57032977	11.4206152965	772.57032977	11.4206152965	770.86970977	1.233047236
772.85376577	11.4198074759	772.85376577	11.4198074759	772.57032977	1.2362938448
774.07830077	11.3710702991	774.07830077	11.3710702991	772.85376577	1.2362640747
774.64517477	11.3754095606	774.64517477	11.3754095606	774.07830077	1.2345574476
775.27157777	11.3755648353	775.27157777	11.3755648353	774.64517477	1.237426081
775.86970977	11.3742393879	775.86970977	11.3742393879	775.27157777	1.2393392311
777.57032977	11.368362844	777.57032977	11.368362844	775.86970977	1.2408684794
777.85376577	11.3660767918	777.85376577	11.3660767918	777.57032977	1.2444513042
779.07830077	11.3549398899	779.07830077	11.3549398899	777.85376577	1.2447808135
779.64517477	11.3519219076	779.64517477	11.3519219076	779.07830077	1.2467064622
780.27157777	11.3504114286	780.27157777	11.3504114286	779.64517477	1.2479803565
780.86970977	11.351756771	780.86970977	11.351756771	780.27157777	1.2495563665
782.57032977	11.3257715579	782.57032977	11.3257715579	780.86970977	1.2503257451
782.85376577	11.3246309218	782.85376577	11.3246309218	782.57032977	1.2528538898
784.07830077	11.3175301684	784.07830077	11.3175301684	782.85376577	1.2534805572
784.64517477	11.3138745975	784.64517477	11.3138745975	784.07830077	1.2559315041
785.27157777	11.3097863542	785.27157777	11.3097863542	784.64517477	1.2570309557
785.86970977	11.3058791154	785.86970977	11.3058791154	785.27157777	1.2582434809
787.57032977	11.2949676369	787.57032977	11.2949676369	785.86970977	1.2594015056
787.85376577	11.2932161687	787.85376577	11.2932161687	787.57032977	1.2627043321
789.07830077	11.2846326026	789.07830077	11.2846326026	787.85376577	1.2632568311
789.64517477	11.2789435862	789.64517477	11.2789435862	789.07830077	1.2654234864
790.27157777	11.2740246806	790.27157777	11.2740246806	789.64517477	1.266263631
790.86970977	11.2700392348	790.86970977	11.2700392348	790.27157777	1.2674336346
792.57032977	11.2587977319	792.57032977	11.2587977319	790.86970977	1.2686155224
792.85376577	11.2569042868	792.85376577	11.2569042868	792.57032977	1.2719158722
794.07830077	11.2486978796	794.07830077	11.2486978796	792.85376577	1.2724599825
794.64517477	11.2448926519	794.64517477	11.2448926519	794.07830077	1.2748029889
795.27157777	11.2406840022	795.27157777	11.2406840022	794.64517477	1.2758848834
795.86970977	11.2366547098	795.86970977	11.2366547098	795.27157777	1.27707848
797.57032977	11.2250275542	797.57032977	11.2250275542	795.86970977	1.2782153937
797.85376577	11.223062742	797.85376577	11.223062742	797.57032977	1.2814211118
799.07830077	11.2145816282	799.07830077	11.2145816282	797.85376577	1.2819522012
799.64517477	11.210684496	799.64517477	11.210684496	799.07830077	1.2842503397
800.27157777	11.2064021928	800.27157777	11.2064021928	799.64517477	1.2853186099
800.86970977	11.2023323442	800.86970977	11.2023323442	800.27157777	1.2865019289
802.57032977	11.1908286778	802.57032977	11.1908286778	800.86970977	1.2876338294
802.85376577	11.1889180966	802.85376577	11.1889180966	802.57032977	1.2908575113
804.07830077	11.1806778182	804.07830077	11.1806778182	802.85376577	1.2913951811
804.64517477	11.1768687118	804.64517477	11.1768687118	804.07830077	1.2937183919
805.27157777	11.1726621953	805.27157777	11.1726621953	804.64517477	1.2947938103
805.86970977	11.1686473752	805.86970977	11.1686473752	805.27157777	1.2959819636
807.57032977	11.1572415794	807.57032977	11.1572415794	805.86970977	1.2971162351
807.85376577	11.1553423931	807.85376577	11.1553423931	807.57032977	1.3003399935
809.07830077	11.1471462033	809.07830077	11.1471462033	807.85376577	1.3008771941
809.64517477	11.1433578287	809.64517477	11.1433578287	809.07830077	1.3031981965
810.27157777	11.1391765899	810.27157777	11.1391765899	809.64517477	1.3042728619
810.86970977	11.1351891206	810.86970977	11.1351891206	810.27157777	1.3054606059
812.57032977	11.1238795354	812.57032977	11.1238795354	810.86970977	1.3065949954
812.85376577	11.1219985389	812.85376577	11.1219985389	812.57032977	1.3098216796

Final State: j'=4		Final State: j'=3		Final State: j'=2	
E(cm⁻¹)	σ (Å²)	E(cm⁻¹)	σ (Å²)	E(cm⁻¹)	σ (Å²)
814.07830077	11.1138844662	814.07830077	11.1138844662	812.85376577	1.310359649
814.64517477	11.1101348399	814.64517477	11.1101348399	814.07830077	1.3126843933
815.27157777	11.1059961268	815.27157777	11.1059961268	814.64517477	1.3137608678
815.86970977	11.1020487135	815.86970977	11.1020487135	815.27157777	1.3149505692
817.57032977	11.0908486613	817.57032977	11.0908486613	815.86970977	1.3160867398
817.85376577	11.0889852807	817.85376577	11.0889852807	817.57032977	1.3193179351
819.07830077	11.0809457388	819.07830077	11.0809457388	817.85376577	1.319856581
819.64517477	11.0772299317	819.64517477	11.0772299317	819.07830077	1.3221840591
820.27157777	11.0731283025	820.27157777	11.0731283025	819.64517477	1.3232617218
820.86970977	11.0692161309	820.86970977	11.0692161309	820.27157777	1.3244527087
822.57032977	11.0581162189	822.57032977	11.0581162189	820.86970977	1.3255901037
822.85376577	11.0562695983	822.85376577	11.0562695983	822.57032977	1.3288248384
824.07830077	11.0483026785	824.07830077	11.0483026785	822.85376577	1.3293640888
824.64517477	11.0446206555	824.64517477	11.0446206555	824.07830077	1.3316942659
825.27157777	11.0405564487	825.27157777	11.0405564487	824.64517477	1.3327732154
825.86970977	11.0366800475	825.86970977	11.0366800475	825.27157777	1.3339656557
827.57032977	11.0256819547	827.57032977	11.0256819547	825.86970977	1.335104452
827.85376577	11.0238522901	827.85376577	11.0238522901	827.57032977	1.3383432604
829.07830077	11.0159584768	829.07830077	11.0159584768	827.85376577	1.338883199
829.64517477	11.012310228	829.64517477	11.012310228	829.07830077	1.3412163585
830.27157777	11.0082832281	830.27157777	11.0082832281	829.64517477	1.3422966845
830.86970977	11.0044423215	830.86970977	11.0044423215	830.27157777	1.3434906654
832.57032977	10.9935447422	832.57032977	10.9935447422	830.86970977	1.3446309265
832.85376577	10.9917317763	832.85376577	10.9917317763	832.57032977	1.3478739198
834.07830077	10.9839100263	834.07830077	10.9839100263	832.85376577	1.3484145554
834.64517477	10.9802950724	834.64517477	10.9802950724	834.07830077	1.3507507501
835.27157777	10.9763048778	835.27157777	10.9763048778	834.64517477	1.3518324984
835.86970977	10.9724990453	835.86970977	10.9724990453	835.27157777	1.3530280368
837.57032977	10.9617010847	837.57032977	10.9617010847	835.86970977	1.3541698047
837.85376577	10.9599047095	837.85376577	10.9599047095	837.57032977	1.3574171176
839.07830077	10.9521546062	839.07830077	10.9521546062	837.85376577	1.3579584809
839.64517477	10.9485727884	839.64517477	10.9485727884	839.07830077	1.3602978346
840.27157777	10.9446191489	840.27157777	10.9446191489	839.64517477	1.3613810591
840.86970977	10.9408482509	840.86970977	10.9408482509	840.27157777	1.362578237
842.57032977	10.9301494528	842.57032977	10.9301494528	840.86970977	1.363721579
842.85376577	10.9283696043	842.85376577	10.9283696043	842.57032977	1.366973407
844.07830077	10.9206907804	844.07830077	10.9206907804	842.85376577	1.3675155304
844.64517477	10.9171419248	844.64517477	10.9171419248	844.07830077	1.3698581812
845.27157777	10.9132246915	845.27157777	10.9132246915	844.64517477	1.3709429448
845.86970977	10.9094885396	845.86970977	10.9094885396	845.27157777	1.3721418272
847.57032977	10.898888437	847.57032977	10.898888437	845.86970977	1.3732868086
847.85376577	10.8971250007	847.85376577	10.8971250007	847.57032977	1.3765433328
849.07830077	10.8895171085	849.07830077	10.8895171085	847.85376577	1.3770862458
849.64517477	10.8860010695	849.64517477	10.8860010695	849.07830077	1.3794323229
850.27157777	10.8821200997	850.27157777	10.8821200997	849.64517477	1.3805186816
850.86970977	10.8784185187	850.86970977	10.8784185187	850.27157777	1.381719339
852.57032977	10.8679167001	852.57032977	10.8679167001	850.86970977	1.3828660182
852.85376577	10.8661696297	852.85376577	10.8661696297	852.57032977	1.3861274184
854.07830077	10.858632415	854.07830077	10.858632415	852.85376577	1.3866711488
854.64517477	10.8551490722	854.64517477	10.8551490722	854.07830077	1.3890207841
855.27157777	10.8513042268	855.27157777	10.8513042268	854.64517477	1.3901088018

Final State: j'=4		Final State: j'=3		Final State: j'=2	
E(cm⁻¹)	σ (Å²)	E(cm⁻¹)	σ (Å²)	E(cm⁻¹)	σ (Å²)
855.86970977	10.8476371275	855.86970977	10.8476371275	855.27157777	1.3913112979
857.57032977	10.8372332699	857.57032977	10.8372332699	855.86970977	1.3924597416
857.85376577	10.8355025278	857.85376577	10.8355025278	857.57032977	1.3957262031
859.07830077	10.8280357803	859.07830077	10.8280357803	857.85376577	1.3962707842
859.64517477	10.824585056	859.64517477	10.824585056	859.07830077	1.3986241103
860.27157777	10.8207762544	860.27157777	10.8207762544	859.64517477	1.3997138459
860.86970977	10.8171435496	860.86970977	10.8171435496	860.27157777	1.4009182509
862.57032977	10.8068374297	862.57032977	10.8068374297	860.86970977	1.4020685266
862.85376577	10.8051229762	862.85376577	10.8051229762	862.57032977	1.4053402357
864.07830077	10.7977265756	864.07830077	10.7977265756	862.85376577	1.4058856999
864.64517477	10.7943083888	864.64517477	10.7943083888	864.07830077	1.4082428541
865.27157777	10.7905355313	865.27157777	10.7905355313	864.64517477	1.4093343732
865.86970977	10.786937166	865.86970977	10.786937166	865.27157777	1.4105407601
867.57032977	10.7767286143	867.57032977	10.7767286143	865.86970977	1.4116929295
867.85376577	10.7750304107	867.85376577	10.7750304107	867.57032977	1.4149700844
869.07830077	10.7677042256	869.07830077	10.7677042256	867.85376577	1.4155164582
869.64517477	10.7643185296	869.64517477	10.7643185296	869.07830077	1.417877579
870.27157777	10.7605815792	870.27157777	10.7605815792	869.64517477	1.4189709481
870.86970977	10.7570174768	870.86970977	10.7570174768	870.27157777	1.4201793821
872.57032977	10.7469063241	872.57032977	10.7469063241	870.86970977	1.4213335235
872.85376577	10.7452243455	872.85376577	10.7452243455	872.57032977	1.4246163076
874.07830077	10.7379682607	874.07830077	10.7379682607	872.85376577	1.4251636296
874.64517477	10.7346149895	874.64517477	10.7346149895	874.07830077	1.4275288562
875.27157777	10.7309138669	875.27157777	10.7309138669	874.64517477	1.4286241333
875.86970977	10.7273839678	875.86970977	10.7273839678	875.27157777	1.4298346945
877.57032977	10.7173699889	877.57032977	10.7173699889	875.86970977	1.4309908661
877.85376577	10.715704188	877.85376577	10.715704188	877.57032977	1.4342794837
879.07830077	10.7085179771	879.07830077	10.7085179771	877.85376577	1.4348277815
879.64517477	10.7051970638	879.64517477	10.7051970638	879.07830077	1.4371972564
880.27157777	10.7015316595	880.27157777	10.7015316595	879.64517477	1.4382945161
880.86970977	10.698035855	880.86970977	10.698035855	880.27157777	1.4395072753
882.57032977	10.6881187213	882.57032977	10.6881187213	880.86970977	1.4406655532
882.85376577	10.6864690624	882.85376577	10.6864690624	882.57032977	1.4439602095
884.07830077	10.6793525166	884.07830077	10.6793525166	882.85376577	1.4445095253
884.64517477	10.6760640017	884.64517477	10.6760640017	884.07830077	1.4468834112
885.27157777	10.6724339909	885.27157777	10.6724339909	884.64517477	1.4479827393
885.86970977	10.6689721514	885.86970977	10.6689721514	885.27157777	1.4491977631
887.57032977	10.6591514668	887.57032977	10.6591514668	885.86970977	1.4503582335
887.85376577	10.6575178726	887.85376577	10.6575178726	887.57032977	1.4536591833
889.07830077	10.6504706458	889.07830077	10.6504706458	887.85376577	1.4542095525
889.64517477	10.6472139878	889.64517477	10.6472139878	889.07830077	1.4565880342
890.27157777	10.643619561	890.27157777	10.643619561	889.64517477	1.4576894977
890.86970977	10.6401914955	890.86970977	10.6401914955	890.27157777	1.4589069208
892.57032977	10.6304666243	892.57032977	10.6304666243	890.86970977	1.460069675
892.85376577	10.6288489643	892.85376577	10.6288489643	892.57032977	1.4633772271
894.07830077	10.6218705195	894.07830077	10.6218705195	892.85376577	1.4639287108
894.64517477	10.6186456611	894.64517477	10.6186456611	894.07830077	1.4663120408
895.27157777	10.6150862988	895.27157777	10.6150862988	894.64517477	1.4674157685
895.86970977	10.6116916621	895.86970977	10.6116916621	895.27157777	1.4686357174
897.57032977	10.6020616412	897.57032977	10.6020616412	895.86970977	1.4698009112
897.85376577	10.6004597478	897.85376577	10.6004597478	897.57032977	1.473115482

Final State: j’=4		Final State: j’=3		Final State: j’=2	
E(cm⁻¹)	σ (Å²)	E(cm⁻¹)	σ (Å²)	E(cm⁻¹)	σ (Å²)
899.07830077	10.5935492052	899.07830077	10.5935492052	897.85376577	1.473668152
899.64517477	10.5903556734	899.64517477	10.5903556734	899.07830077	1.4760566821
900.27157777	10.5868308722	900.27157777	10.5868308722	899.64517477	1.4771628572
900.86970977	10.5834691465	900.86970977	10.5834691465	900.27157777	1.4783855397
902.57032977	10.5739321834	902.57032977	10.5739321834	900.86970977	1.4795533741
902.85376577	10.5723457133	902.85376577	10.5723457133	902.57032977	1.4828756471
904.07830077	10.5655015374	904.07830077	10.5655015374	902.85376577	1.4834296306
904.64517477	10.5623385464	904.64517477	10.5623385464	904.07830077	1.4858239551
905.27157777	10.558847331	905.27157777	10.558847331	904.64517477	1.4869328785
905.86970977	10.5555174927	905.86970977	10.5555174927	905.27157777	1.4881586579
907.57032977	10.5460702369	907.57032977	10.5460702369	905.86970977	1.489329515
907.85376577	10.5444985387	907.85376577	10.5444985387	907.57032977	1.4926607455
909.07830077	10.5377175498	909.07830077	10.5377175498	907.85376577	1.4932162875
909.64517477	10.5345834113	909.64517477	10.5345834113	909.07830077	1.4956175803
910.27157777	10.5311237251	910.27157777	10.5311237251	909.64517477	1.4967298875
910.86970977	10.5278236147	910.86970977	10.5278236147	910.27157777	1.4979595354
912.57032977	10.5184583153	912.57032977	10.5184583153	910.86970977	1.4991342306
912.85376577	10.5168998684	912.85376577	10.5168998684	912.57032977	1.5024773443
914.07830077	10.5101741725	914.07830077	10.5101741725	912.85376577	1.5030350367
914.64517477	10.5070643997	914.64517477	10.5070643997	914.07830077	1.5054463293
915.27157777	10.5036305592	915.27157777	10.5036305592	914.64517477	1.5065637389
915.86970977	10.5003538718	915.86970977	10.5003538718	915.27157777	1.5077994642
917.57032977	10.4910456589	917.57032977	10.4910456589	915.86970977	1.5089804756
917.85376577	10.489494862	917.85376577	10.489494862	917.57032977	1.5123454321
919.07830077	10.4827932236	919.07830077	10.4827932236	917.85376577	1.5129075319
919.64517477	10.4796876462	919.64517477	10.4796876462	919.07830077	1.5153418265
920.27157777	10.4762507684	920.27157777	10.4762507684	919.64517477	1.516472996
920.86970977	10.4729604138	920.86970977	10.4729604138	920.27157777	1.5177274899
922.57032977	10.4634828709	922.57032977	10.4634828709	920.86970977	1.5189316457
922.85376577	10.4618596436	922.85376577	10.4618596436	922.57032977	1.5224365403
924.07830077	10.4554483317	924.07830077	10.4554483317	922.85376577	1.5230526893
924.64517477	10.4541727204	924.64517477	10.4541727204	924.07830077	1.5268781702
925.27157777	10.450254186	925.27157777	10.450254186	924.64517477	1.5284511622
925.86970977	10.4462732053	925.86970977	10.4462732053	925.27157777	1.5278495926
927.57032977	10.4372441483	927.57032977	10.4372441483	925.86970977	1.5301142272
927.85376577	10.4351338914	927.85376577	10.4351338914	927.57032977	1.5323653951
929.07830077	10.4320596192	929.07830077	10.4320596192	927.85376577	1.533612297
929.64517477	10.4274592863	929.64517477	10.4274592863	929.07830077	1.535622608
930.27157777	10.4245244633	930.27157777	10.4245244633	929.64517477	1.5370400599
930.86970977	10.4203287236	930.86970977	10.4203287236	930.27157777	1.537943757
932.57032977	10.413200673	932.57032977	10.413200673	930.86970977	1.538704433
932.85376577	10.4110330971	932.85376577	10.4110330971	932.57032977	1.5420385649
934.07830077	10.4041600207	934.07830077	10.4041600207	932.85376577	1.543098794
934.64517477	10.4005330976	934.64517477	10.4005330976	934.07830077	1.5446871503
935.27157777	10.3965296814	935.27157777	10.3965296814	934.64517477	1.5460410612
935.86970977	10.3971082015	935.86970977	10.3971082015	935.27157777	1.5479800985
937.57032977	10.3886553081	937.57032977	10.3886553081	935.86970977	1.55129768
937.85376577	10.3852961986	937.85376577	10.3852961986	937.57032977	1.5519909963
939.07830077	10.3776730951	939.07830077	10.3776730951	937.85376577	1.5518950863
939.64517477	10.3742378369	939.64517477	10.3742378369	939.07830077	1.5546761002
940.27157777	10.3709861936	940.27157777	10.3709861936	939.64517477	1.5560836873

Final State: j’=4		Final State: j’=3		Final State: j’=2	
E(cm⁻¹)	σ (Å²)	E(cm⁻¹)	σ (Å²)	E(cm⁻¹)	σ (Å²)
940.86970977	10.3730991768	940.86970977	10.3730991768	940.27157777	1.5591465119
942.57032977	10.3612205079	942.57032977	10.3612205079	940.86970977	1.5600593217
942.85376577	10.3592945161	942.85376577	10.3592945161	942.57032977	1.5611015126
944.07830077	10.3521528376	944.07830077	10.3521528376	942.85376577	1.5617568631
944.64517477	10.348913886	944.64517477	10.348913886	944.07830077	1.5644584443
945.27157777	10.3451711888	945.27157777	10.3451711888	944.64517477	1.5657366135
945.86970977	10.3436803073	945.86970977	10.3436803073	945.27157777	1.5673460015
947.57032977	10.3376044648	947.57032977	10.3376044648	945.86970977	1.5708179642
947.85376577	10.334855134	947.85376577	10.334855134	947.57032977	1.5717707187
949.07830077	10.3270374059	949.07830077	10.3270374059	947.85376577	1.5714581814
949.64517477	10.323899678	949.64517477	10.323899678	949.07830077	1.5742310905
950.27157777	10.3204525035	950.27157777	10.3204525035	949.64517477	1.5754555499
950.86970977	10.3171136815	950.86970977	10.3171136815	950.27157777	1.576817508
952.57032977	10.3130810571	952.57032977	10.3130810571	950.86970977	1.5781646672
952.85376577	10.3089624519	952.85376577	10.3089624519	952.57032977	1.5827814011
954.07830077	10.302630335	954.07830077	10.302630335	952.85376577	1.5831225849
954.64517477	10.298969611	954.64517477	10.298969611	954.07830077	1.5839846781
955.27157777	10.2953964321	955.27157777	10.2953964321	954.64517477	1.5853429372
955.86970977	10.292048005	955.86970977	10.292048005	955.27157777	1.5867592651
957.57032977	10.2826278547	957.57032977	10.2826278547	955.86970977	1.5881147314
957.85376577	10.2817545674	957.85376577	10.2817545674	957.57032977	1.5935118838
959.07830077	10.2803647444	959.07830077	10.2803647444	957.85376577	1.595022378
959.64517477	10.277054332	959.64517477	10.277054332	959.07830077	1.5988865016
960.27157777	10.2743761043	960.27157777	10.2743761043	959.64517477	1.5985760247
960.86970977	10.2685069529	960.86970977	10.2685069529	960.27157777	1.5973863231
962.57032977	10.2598679895	962.57032977	10.2598679895	960.86970977	1.5998985688
962.85376577	10.25775867	962.85376577	10.25775867	962.57032977	1.601385983
964.07830077	10.256382217	964.07830077	10.256382217	962.85376577	1.6023902043
964.64517477	10.2507971013	964.64517477	10.2507971013	964.07830077	1.606521391
965.27157777	10.2462438043	965.27157777	10.2462438043	964.64517477	1.6051308863
965.86970977	10.2417454809	965.86970977	10.2417454809	965.27157777	1.6068671159
967.57032977	10.2404376306	967.57032977	10.2404376306	965.86970977	1.609251845
967.85376577	10.2381318071	967.85376577	10.2381318071	967.57032977	1.6107403192
969.07830077	10.2278903855	969.07830077	10.2278903855	967.85376577	1.6116349206
969.64517477	10.2248163675	969.64517477	10.2248163675	969.07830077	1.6143731134
970.27157777	10.2300742189	970.27157777	10.2300742189	969.64517477	1.6182858682
970.86970977	10.2223066635	970.86970977	10.2223066635	970.27157777	1.6207044953
972.57032977	10.2110192385	972.57032977	10.2110192385	970.86970977	1.616920654
972.85376577	10.2092380402	972.85376577	10.2092380402	972.57032977	1.621250474
974.07830077	10.2099850213	974.07830077	10.2099850213	972.85376577	1.6220547913
974.64517477	10.2081603828	974.64517477	10.2081603828	974.07830077	1.6300862672
975.27157777	10.2011614776	975.27157777	10.2011614776	974.64517477	1.6264105925
975.86970977	10.1971878099	975.86970977	10.1971878099	975.27157777	1.6275720289
977.57032977	10.1883896465	977.57032977	10.1883896465	975.86970977	1.6281185916
977.85376577	10.1851758855	977.85376577	10.1851758855	977.57032977	1.6336852752
979.07830077	10.1837671283	979.07830077	10.1837671283	977.85376577	1.6336639439
979.64517477	10.1777171474	979.64517477	10.1777171474	979.07830077	1.6332693739
980.27157777	10.1788037068	980.27157777	10.1788037068	979.64517477	1.6358879259
980.86970977	10.1731083155	980.86970977	10.1731083155	980.27157777	1.6402278631
982.57032977	10.1659379393	982.57032977	10.1659379393	980.86970977	1.6373544219
982.85376577	10.1694856438	982.85376577	10.1694856438	982.57032977	1.6438995276

**Final State: j'=4**		**Final State: j'=3**		**Final State: j'=2**	
E(cm^{-1})	σ (Å^2)	E(cm^{-1})	σ (Å^2)	E(cm^{-1})	σ (Å^2)
984.07830077	10.1641762843	984.07830077	10.1641762843	982.85376577	1.6475825016
984.64517477	10.158527158	984.64517477	10.158527158	984.07830077	1.6477992094
985.27157777	10.1524369544	985.27157777	10.1524369544	984.64517477	1.6447472734
985.86970977	10.148177107	985.86970977	10.148177107	985.27157777	1.6465180371
987.57032977	10.1449158389	987.57032977	10.1449158389	985.86970977	1.6495772567
987.85376577	10.146254869	987.85376577	10.146254869	987.57032977	1.6559543552
989.07830077	10.1363693129	989.07830077	10.1363693129	987.85376577	1.6558758577
989.64517477	10.1334035166	989.64517477	10.1334035166	989.07830077	1.6557141325
990.27157777	10.1303803078	990.27157777	10.1303803078	989.64517477	1.6570199001
990.86970977	10.127469457	990.86970977	10.127469457	990.27157777	1.6585352295
992.57032977	10.1226553035	992.57032977	10.1226553035	990.86970977	1.6600705587
992.85376577	10.1207261343	992.85376577	10.1207261343	992.57032977	1.6635546684
994.07830077	10.1141727934	994.07830077	10.1141727934	992.85376577	1.6637025318
994.64517477	10.1115470138	994.64517477	10.1115470138	994.07830077	1.666236194
995.27157777	10.1087002589	995.27157777	10.1087002589	994.64517477	1.6674471277
995.86970977	10.1060546332	995.86970977	10.1060546332	995.27157777	1.66878736
997.57032977	10.098946431	997.57032977	10.098946431	995.86970977	1.6700890915
997.85376577	10.0976634719	997.85376577	10.0976634719	997.57032977	1.6736657733
999.07830077	10.0921008059	999.07830077	10.0921008059	997.85376577	1.6742253292
999.64517477	10.0900073625	999.64517477	10.0900073625	999.07830077	1.6767435475
1000.27157777	10.0885446127	1000.27157777	10.0885446127	999.64517477	1.6782525312
1000.86970777	10.0846000379	1000.86970777	10.0846000379	1000.27157777	1.6793090407
1002.57032777	10.0769825155	1002.57032777	10.0769825155	1000.86970777	1.680222697
1002.85376777	10.0757482326	1002.85376777	10.0757482326	1002.57032777	1.6837980156
1004.07829777	10.0704222807	1004.07829777	10.0704222807	1002.85376777	1.6843816622
1004.64517777	10.0679619546	1004.64517777	10.0679619546	1004.07829777	1.6868951017
1005.27157777	10.0652490405	1005.27157777	10.0652490405	1004.64517777	1.6880575611
1005.86970777	10.062665776	1005.86970777	10.062665776	1005.27157777	1.689342763
1007.57032777	10.0554633631	1007.57032777	10.0554633631	1005.86970777	1.6905714319
1007.85376777	10.0543251115	1007.85376777	10.0543251115	1007.57032777	1.6940969389
1009.07829777	10.049087991	1009.07829777	10.049087991	1007.85376777	1.6946887896
1009.64517777	10.0466063774	1009.64517777	10.0466063774	1009.07829777	1.6971483672
1010.27157777	10.0438980425	1010.27157777	10.0438980425	1009.64517777	1.6982954871
1010.86970777	10.0413309445	1010.86970777	10.0413309445	1010.27157777	1.6995719475
1012.57032777	10.0340849414	1012.57032777	10.0340849414	1010.86970777	1.7007938232
1012.85376777	10.0328827173	1012.85376777	10.0328827173	1012.57032777	1.7042724564
1014.07829777	10.0277052124	1014.07829777	10.0277052124	1012.85376777	1.7048525547
1014.64517777	10.0253180678	1014.64517777	10.0253180678	1014.07829777	1.7073597475
1015.27157777	10.0226878931	1015.27157777	10.0226878931	1014.64517777	1.7085209886
1015.86970777	10.0201831656	1015.86970777	10.0201831656	1015.27157777	1.7098044877
1017.57032777	10.0130772923	1017.57032777	10.0130772923	1015.86970777	1.7110299766
1017.85376777	10.0118937716	1017.85376777	10.0118937716	1017.57032777	1.7145102919
1019.07829777	10.0067866484	1019.07829777	10.0067866484	1017.85376777	1.7150898256
1019.64517777	10.0044273654	1019.64517777	10.0044273654	1019.07829777	1.7175933714
1020.27157777	10.0018247229	1020.27157777	10.0018247229	1019.64517777	1.7187524894
1020.86970777	9.9993440699	1020.86970777	9.9993440699	1020.27157777	1.7200335104
1022.57032777	9.9923160107	1022.57032777	9.9923160107	1020.86970777	1.7212568808
1022.85376777	9.9911482856	1022.85376777	9.9911482856	1022.57032777	1.7247359246
1024.07829777	9.9861151567	1024.07829777	9.9861151567	1022.85376777	1.7253158448
1024.64517777	9.9837915522	1024.64517777	9.9837915522	1024.07829777	1.7278214455
1025.27157777	9.981228505	1025.27157777	9.981228505	1024.64517777	1.7289814079

Final State: j'=4		Final State: j'=3		Final State: j'=2	
E(cm⁻¹)	σ (Å²)	E(cm⁻¹)	σ (Å²)	E(cm⁻¹)	σ (Å²)
1025.86970777	9.9787854968	1025.86970777	9.9787854968	1025.27157777	1.7302631767
1027.57032777	9.9718619198	1027.57032777	9.9718619198	1025.86970777	1.7314870627
1029.07829777	9.965749803	1029.07829777	9.965749803	1027.57032777	1.7349665491
1029.64517777	9.9634587646	1029.64517777	9.9634587646	1029.07829777	1.7380514473
1030.27157777	9.9609313442	1030.27157777	9.9609313442	1029.64517777	1.7392110134
1030.86970777	9.9585221525	1030.86970777	9.9585221525	1030.27157777	1.7404922964
1032.57032777	9.9516946718	1032.57032777	9.9516946718	1030.86970777	1.7417156903
1034.07829777	9.9456684303	1034.07829777	9.9456684303	1032.57032777	1.7451937609
1034.64517777	9.9434098132	1034.64517777	9.9434098132	1034.07829777	1.7482774119
1035.27157777	9.9409183345	1035.27157777	9.9409183345	1034.64517777	1.7494364965
1035.86970777	9.9385434981	1035.86970777	9.9385434981	1035.27157777	1.7507172164
1037.57032777	9.9318136515	1037.57032777	9.9318136515	1035.86970777	1.7519400474
1039.07829777	9.9258736633	1039.07829777	9.9258736633	1037.57032777	1.7554163164
1039.64517777	9.9236473983	1039.64517777	9.9236473983	1039.07829777	1.7584980853
1040.27157777	9.9211915318	1040.27157777	9.9211915318	1039.64517777	1.7596563862
1040.86970777	9.918850642	1040.86970777	9.918850642	1040.27157777	1.7609361995
1042.57032777	9.9122169728	1042.57032777	9.9122169728	1040.86970777	1.7621581247
1044.07829777	9.9063619357	1044.07829777	9.9063619357	1042.57032777	1.7656315889
1044.64517777	9.9041674989	1044.64517777	9.9041674989	1044.07829777	1.7687106098
1045.27157777	9.901746812	1045.27157777	9.901746812	1044.64517777	1.7698678203
1045.86970777	9.8994394513	1045.86970777	9.8994394513	1045.27157777	1.7711463895
1047.57032777	9.8929009282	1047.57032777	9.8929009282	1045.86970777	1.7723670855
1049.07829777	9.8871299779	1049.07829777	9.8871299779	1047.57032777	1.7758368613
1049.64517777	9.8849671007	1049.64517777	9.8849671007	1049.07829777	1.7789123612
1050.27157777	9.8825812416	1050.27157777	9.8825812416	1049.64517777	1.7800681854
1050.86970777	9.8803070592	1050.86970777	9.8803070592	1050.27157777	1.781345178
1052.57032777	9.8738626857	1052.57032777	9.8738626857	1050.86970777	1.7825643315
1054.07829777	9.8681748897	1054.07829777	9.8681748897	1052.57032777	1.7860295143
1054.64517777	9.8660431806	1054.64517777	9.8660431806	1054.07829777	1.7891006754
1055.27157777	9.8636916989	1055.27157777	9.8636916989	1054.64517777	1.7902548024
1055.86970777	9.8614503325	1055.86970777	9.8614503325	1055.27157777	1.7915298806
1057.57032777	9.8550989313	1057.57032777	9.8550989313	1055.86970777	1.7927471649
1059.07829777	9.8494932542	1059.07829777	9.8494932542	1057.57032777	1.7962068149
1059.64517777	9.8473923144	1059.64517777	9.8473923144	1059.07829777	1.7992727955
1060.27157777	9.8450748045	1060.27157777	9.8450748045	1059.64517777	1.8004249107
1060.86970777	9.8428658285	1060.86970777	9.8428658285	1060.27157777	1.8016977264
1062.57032777	9.8366062044	1062.57032777	9.8366062044	1060.86970777	1.8029128034
1064.07829777	9.831081545	1064.07829777	9.831081545	1062.57032777	1.8063659674
1064.64517777	9.8290110163	1064.64517777	9.8290110163	1064.07829777	1.8094259237
1065.27157777	9.826726997	1065.27157777	9.826726997	1064.64517777	1.8105757087
1065.86970777	9.8245499836	1065.86970777	9.8245499836	1065.27157777	1.8118459051
1067.57032777	9.8183808869	1067.57032777	9.8183808869	1065.86970777	1.8130584442
1069.07829777	9.8129361582	1069.07829777	9.8129361582	1067.57032777	1.8165041516
1069.64517777	9.8108955713	1069.64517777	9.8108955713	1069.07829777	1.8195572236
1070.27157777	9.8086445958	1070.27157777	9.8086445958	1069.64517777	1.8207043522
1070.86970777	9.8064990661	1070.86970777	9.8064990661	1070.27157777	1.8219715682
1072.57032777	9.800419202	1072.57032777	9.800419202	1070.86970777	1.8231812192
1074.07829777	9.7950532407	1074.07829777	9.7950532407	1072.57032777	1.8266184984
1074.64517777	9.793042163	1074.64517777	9.793042163	1074.07829777	1.8296638084
1075.27157777	9.7908237503	1075.27157777	9.7908237503	1074.64517777	1.830807953
1075.86970777	9.788709228	1075.86970777	9.788709228	1075.27157777	1.8320718278

Final State: j’=4		Final State: j’=3		Final State: j’=2	
E(cm⁻¹)	σ (Å²)	E(cm⁻¹)	σ (Å²)	E(cm⁻¹)	σ (Å²)
1077.57032777	9.7827172714	1077.57032777	9.7827172714	1075.86970777	1.8332782441
1079.07829777	9.7774288466	1079.07829777	9.7774288466	1077.57032777	1.8367060951
1079.64517777	9.7754468249	1079.64517777	9.7754468249	1079.07829777	1.8397427649
1080.27157777	9.7732604579	1080.27157777	9.7732604579	1079.64517777	1.8408835902
1080.86970777	9.7711764656	1080.86970777	9.7711764656	1080.27157777	1.842143756
1084.07829777	9.7600589118	1084.07829777	9.7600589118	1080.86970777	1.8433465527
1084.64517777	9.7581054732	1084.64517777	9.7581054732	1084.07829777	1.8497911377
1085.27157777	9.7559506322	1085.27157777	9.7559506322	1084.64517777	1.8509283057
1085.86970777	9.7538966907	1085.86970777	9.7538966907	1085.27157777	1.852184384
1089.07829777	9.7429392046	1089.07829777	9.7429392046	1085.86970777	1.8533832697
1089.64517777	9.7410139508	1089.64517777	9.7410139508	1089.07829777	1.8598059291
1090.27157777	9.7388900144	1090.27157777	9.7388900144	1089.64517777	1.8609386583
1090.86970777	9.7368655908	1090.86970777	9.7368655908	1090.27157777	1.8621907147
1094.07829777	9.7260653894	1094.07829777	9.7260653894	1090.86970777	1.8633852978
1094.64517777	9.7241676332	1094.64517777	9.7241676332	1094.07829777	1.8697841169
1095.27157777	9.7220742067	1095.27157777	9.7220742067	1094.64517777	1.8709129457
1095.86970777	9.7200787845	1095.86970777	9.7200787845	1095.27157777	1.8721597204
1099.07829777	9.7094329635	1099.07829777	9.7094329635	1095.86970777	1.8733496323
1099.64517777	9.7075622938	1099.64517777	9.7075622938	1099.07829777	1.8797226616
1100.86970777	9.7035317127	1100.86970777	9.7035317127	1099.64517777	1.8808468032
1104.07829777	9.6930372954	1104.07829777	9.6930372954	1100.86970777	1.8832732264
1104.64517777	9.6911931614	1104.64517777	9.6911931614	1104.07829777	1.8896184938
1105.86970777	9.6872197363	1105.86970777	9.6872197363	1104.64517777	1.8907375967
1109.07829777	9.6768736592	1109.07829777	9.6768736592	1105.86970777	1.8931530079
1109.64517777	9.6750555358	1109.64517777	9.6750555358	1109.07829777	1.8994685323
1110.86970777	9.6711380539	1110.86970777	9.6711380539	1109.64517777	1.9005822493
1114.07829777	9.6609371991	1114.07829777	9.6609371991	1110.86970777	1.9029858835
1114.64517777	9.6591445289	1114.64517777	9.6591445289	1114.07829777	1.9092696717
1115.86970777	9.6552818098	1115.86970777	9.6552818098	1114.64517777	1.9103776469
1119.07829777	9.6452229874	1119.07829777	9.6452229874	1115.86970777	1.9127687465
1119.64517777	9.6434551821	1119.64517777	9.6434551821	1119.07829777	1.9190187942
1120.86970777	9.6396459829	1120.86970777	9.6396459829	1119.64517777	1.9201206765
1124.07829777	9.6297259646	1124.07829777	9.6297259646	1120.86970777	1.9224984734
1124.64517777	9.6279824936	1124.64517777	9.6279824936	1124.07829777	1.928712771
1125.86970777	9.6242255568	1125.86970777	9.6242255568	1124.64517777	1.9298081999
1129.07829777	9.6144411077	1129.07829777	9.6144411077	1125.86970777	1.9321719204
1129.64517777	9.6127213513	1129.64517777	9.6127213513	1129.07829777	1.9383484312
1130.86970777	9.6090154533	1130.86970777	9.6090154533	1129.64517777	1.9394370483
1134.07829777	9.5993632665	1134.07829777	9.5993632665	1130.86970777	1.9417859145
1134.64517777	9.5976666749	1134.64517777	9.5976666749	1134.07829777	1.9479225774
1135.86970777	9.5940105642	1135.86970777	9.5940105642	1134.64517777	1.9490040116
1139.07829777	9.5844873627	1139.07829777	9.5844873627	1135.86970777	1.9513372219
1139.64517777	9.5828133203	1139.64517777	9.5828133203	1139.07829777	1.9574319129
1140.86970777	9.5792057673	1140.86970777	9.5792057673	1139.64517777	1.958505792
1144.07829777	9.56980834	1144.07829777	9.56980834	1140.86970777	1.960822518
1144.64517777	9.5681563366	1144.64517777	9.5681563366	1144.07829777	1.96687302
1145.86970777	9.5645961105	1145.86970777	9.5645961105	1144.64517777	1.9679389365
1149.07829777	9.5553214202	1149.07829777	9.5553214202	1145.86970777	1.9702382964
1149.64517777	9.5536908918	1149.64517777	9.5536908918	1149.07829777	1.976242162
1150.86970777	9.550176923	1150.86970777	9.550176923	1149.64517777	1.9772996575
1154.07829777	9.5410223747	1154.07829777	9.5410223747	1150.86970777	1.9795806333

Final State: j’=4		Final State: j’=3		Final State: j’=2	
E(cm⁻¹)	σ (Å²)	E(cm⁻¹)	σ (Å²)	E(cm⁻¹)	σ (Å²)
1154.64517777	9.5394129379	1154.64517777	9.5394129379	1154.07829777	1.9855349222
1155.86970777	9.5359444192	1155.86970777	9.5359444192	1154.64517777	1.9865834212
1159.07829777	9.5269086834	1159.07829777	9.5269086834	1155.86970777	1.9888446823
1159.64517777	9.5253202653	1159.64517777	9.5253202653	1159.07829777	1.9947450717
1160.86970777	9.5218973588	1160.86970777	9.5218973588	1159.64517777	1.9957836218
1164.07829777	9.512984357	1164.07829777	9.512984357	1160.86970777	1.9980228
1165.86970777	9.5080472126	1165.86970777	9.5080472126	1164.07829777	2.0038594421
1169.07829777	9.4993328863	1169.07829777	9.4993328863	1165.86970777	2.007094168
1170.86970777	9.4946475088	1170.86970777	9.4946475088	1169.07829777	2.0127864418
1174.07829777	9.4859346513	1174.07829777	9.4859346513	1170.86970777	2.0160149506
1175.86970777	9.4808658362	1175.86970777	9.4808658362	1174.07829777	2.0216467897
1179.07829777	9.472199289	1179.07829777	9.472199289	1175.86970777	2.0251711265
1180.86970777	9.467254792	1180.86970777	9.467254792	1179.07829777	2.0307730135
1184.07829777	9.458506922	1184.07829777	9.458506922	1180.86970777	2.034019874
1185.86970777	9.454034103	1185.86970777	9.454034103	1184.07829777	2.0399155613
1189.07829777	9.445363676	1189.07829777	9.445363676	1185.86970777	2.0427305059
1190.86970777	9.440840569	1190.86970777	9.440840569	1189.07829777	2.0485987823
1194.07829777	9.432230152	1194.07829777	9.432230152	1190.86970777	2.051491198
1195.86970777	9.427810019	1195.86970777	9.427810019	1194.07829777	2.0573669193
1199.07829777	9.419634086	1199.07829777	9.419634086	1195.86970777	2.0601453622
1200.86970777	9.414988809	1200.86970777	9.414988809	1199.07829777	2.0656671727
1204.07829777	9.407699762	1204.07829777	9.407699762	1200.86970777	2.0686365869
1205.86970777	9.402539329	1205.86970777	9.402539329	1204.07829777	2.0734383991
1209.07829777	9.394285726	1209.07829777	9.394285726	1205.86970777	2.07723201
1210.86970777	9.389339252	1210.86970777	9.389339252	1209.07829777	2.0822106165
1214.07829777	9.381113722	1214.07829777	9.381113722	1210.86970777	2.0856043237
1215.86970777	9.377714336	1215.86970777	9.377714336	1214.07829777	2.0911832594
1219.07829777	9.369193843	1219.07829777	9.369193843	1215.86970777	2.0929004237
1220.86970777	9.364391851	1220.86970777	9.364391851	1219.07829777	2.0986024429
1224.07829777	9.357137156	1224.07829777	9.357137156	1220.86970777	2.1022007417
1225.86970777	9.352600047	1225.86970777	9.352600047	1224.07829777	2.1063271508
1229.07829777	9.344920717	1229.07829777	9.344920717	1225.86970777	2.1094564373
1230.86970777	9.339620591	1230.86970777	9.339620591	1229.07829777	2.114280597
1234.07829777	9.332525799	1234.07829777	9.332525799	1230.86970777	2.118296742
1235.86970777	9.328218455	1235.86970777	9.328218455	1234.07829777	2.122561248
1239.07829777	9.320467832	1239.07829777	9.320467832	1235.86970777	2.12537602
1240.86970777	9.316205961	1240.86970777	9.316205961	1239.07829777	2.130482335
1244.07829777	9.308506536	1244.07829777	9.308506536	1240.86970777	2.133098667
1245.86970777	9.304151224	1245.86970777	9.304151224	1244.07829777	2.137981517
1249.07829777	9.296617343	1249.07829777	9.296617343	1245.86970777	2.140654394
1250.86970777	9.292367484	1250.86970777	9.292367484	1249.07829777	2.14551392
1254.07829777	9.284767671	1254.07829777	9.284767671	1250.86970777	2.148181399
1255.86970777	9.280560477	1255.86970777	9.280560477	1254.07829777	2.152930834
1259.07829777	9.273018237	1259.07829777	9.273018237	1255.86970777	2.155568914
1260.86970777	9.268816763	1260.86970777	9.268816763	1259.07829777	2.160243654
1264.07829777	9.261312162	1264.07829777	9.261312162	1260.86970777	2.162835946
1265.86970777	9.25713336	1265.86970777	9.25713336	1264.07829777	2.167446576
1269.07829777	9.249664407	1269.07829777	9.249664407	1265.86970777	2.170000796
1270.86970777	9.24550316	1270.86970777	9.24550316	1269.07829777	2.174540271
1275.86970777	9.233921448	1275.86970777	9.233921448	1270.86970777	2.177055352
1280.86970777	9.22238375	1280.86970777	9.22238375	1275.86970777	2.184001737

Final State: j’=4		Final State: j’=3		Final State: j’=2	
E(cm⁻¹)	σ (Å²)	E(cm⁻¹)	σ (Å²)	E(cm⁻¹)	σ (Å²)
1285.86970777	9.210885358	1285.86970777	9.210885358	1280.86970777	2.190839465
1290.86970777	9.19942202	1290.86970777	9.19942202	1285.86970777	2.197568868
1295.86970777	9.187989579	1295.86970777	9.187989579	1290.86970777	2.204190303
1300.86970777	9.176584029	1300.86970777	9.176584029	1295.86970777	2.210704253
1305.86970777	9.165201552	1305.86970777	9.165201552	1300.86970777	2.21711136
1310.86970777	9.153838495	1310.86970777	9.153838495	1305.86970777	2.223412382
1315.86970777	9.142491415	1315.86970777	9.142491415	1310.86970777	2.229608205
1320.86970777	9.131157054	1320.86970777	9.131157054	1315.86970777	2.235699853
1325.86970777	9.119832336	1325.86970777	9.119832336	1320.86970777	2.241688467
1330.86970777	9.108514397	1330.86970777	9.108514397	1325.86970777	2.247575306
1335.86970777	9.097200569	1335.86970777	9.097200569	1330.86970777	2.253361748
1340.86970777	9.085888351	1340.86970777	9.085888351	1335.86970777	2.25904929
1345.86970777	9.074575513	1345.86970777	9.074575513	1340.86970777	2.264639525
1350.86970777	9.063259956	1350.86970777	9.063259956	1345.86970777	2.270134156
1355.86970777	9.051939837	1355.86970777	9.051939837	1350.86970777	2.275534994
1360.86970777	9.040613494	1360.86970777	9.040613494	1355.86970777	2.280843933
1365.86970777	9.029279465	1365.86970777	9.029279465	1360.86970777	2.286062968
1370.86970777	9.01793654	1370.86970777	9.01793654	1365.86970777	2.291194166
1375.86970777	9.006583655	1375.86970777	9.006583655	1370.86970777	2.296239682
1380.86970777	8.99521997	1380.86970777	8.99521997	1375.86970777	2.301201752
1385.86970777	8.983844865	1385.86970777	8.983844865	1380.86970777	2.306082663
1390.86970777	8.972457887	1390.86970777	8.972457887	1385.86970777	2.310884767
1395.86970777	8.961058819	1395.86970777	8.961058819	1390.86970777	2.315610487
1400.86970777	8.949647444	1400.86970777	8.949647444	1395.86970777	2.320262282
1405.86970777	8.938223987	1405.86970777	8.938223987	1400.86970777	2.32484263
				1405.86970777	2.329354112

		Initial rotational state: j=5	
**Final State: j'=1**		**Final State: j'=0**	
E(cm⁻¹)	σ (Å²)	E(cm⁻¹)	σ (Å²)
410.9697098	12.04810558	410.9697098	6.29888663
411.0697098	22.59747714	411.0697098	11.44125867
411.1697098	9.59443203	411.1697098	4.9667795
411.2697098	8.00207519	411.2697098	4.1078637
411.4697098	7.194176913	411.3697098	3.63327931
411.5697098	7.376052308	411.4697098	3.5254322
411.6697098	6.920759264	411.5697098	3.64317517
411.7697098	5.981386359	411.6697098	3.545161205
411.8697098	6.435888872	411.7697098	3.047211065
411.9697098	5.487553665	411.8697098	3.187378399
412.0697098	4.956210087	411.9697098	2.823401825
412.1697098	4.638450531	412.0697098	2.548538825
412.2697098	4.393111082	412.1697098	2.378890067
412.3697098	4.185166231	412.2697098	2.24733449
412.4697098	4.000517947	412.3697098	2.135894398
412.5703298	3.831543862	412.4697098	2.037004876
412.8537658	3.424976231	412.5703298	1.94634453
414.0783008	3.414327246	412.8537658	1.721058801
414.6451748	2.742644434	414.0783008	1.693558447
415.2715778	2.428625056	414.6451748	1.365475577
415.8697098	2.288773955	415.2715778	1.204044659
415.9697098	2.266260637	415.8697098	1.126509257
416.0697098	2.242548732	415.9697098	1.11428954
416.1697098	2.217510863	416.0697098	1.101600361
416.2697098	2.19113029	416.1697098	1.088359774
416.3697098	2.163381933	416.2697098	1.07452425
416.4697098	2.133975122	416.3697098	1.060024005
416.5697098	2.10194749	416.4697098	1.044614559
416.6697098	2.076683682	416.5697098	1.027648724
416.7697098	2.061679266	416.6697098	1.013746017
416.8697098	2.048984774	416.7697098	1.003714977
416.9697098	2.045806878	416.8697098	0.99257254
417.5703298	2.398019808	416.9697098	0.99298071
417.8537658	2.578013158	417.5703298	1.116553711
419.0783008	1.736064028	417.8537658	1.200263699
419.6451748	1.634823364	419.0783008	0.841929322
420.2715778	1.55096952	419.6451748	0.793508815
420.8697098	1.492565499	420.2715778	0.752409793
422.5703298	1.475619579	420.8697098	0.722549338
422.8537658	1.446693423	422.5703298	0.699231262
424.0783008	1.301930905	422.8537658	0.68603486
424.6451748	1.257521242	424.0783008	0.62190638
425.2715778	1.401519243	424.6451748	0.600948849
425.8697098	1.372143525	425.2715778	0.638409761
427.5703298	1.107520235	425.8697098	0.63281001
427.8537658	1.096122881	427.5703298	0.528755094
429.0783008	1.047663999	427.8537658	0.5232331
429.6451748	1.026477798	429.0783008	0.500201411
430.2715778	1.004565992	429.6451748	0.490240256

Final State: j’=1		Final State: j’=0	
E(cm⁻¹)	σ (Å²)	E(cm⁻¹)	σ (Å²)
430.8697098	0.985306862	430.2715778	0.47992854
432.5703298	0.945802025	430.8697098	0.47081336
432.8537658	0.945708689	432.5703298	0.450511817
434.0783008	0.940852182	432.8537658	0.449161487
434.6451748	0.913338798	434.0783008	0.442712115
435.2715778	0.890164229	434.6451748	0.432410312
435.8697098	0.873201571	435.2715778	0.423205274
437.5703298	0.836049587	435.8697098	0.41607346
437.8537658	0.83073408	437.5703298	0.399682104
439.0783008	0.809801951	437.8537658	0.397285248
439.6451748	0.801193215	439.0783008	0.387742687
440.2715778	0.792506242	439.6451748	0.383748536
442.5703298	0.765419723	440.2715778	0.37964471
442.8537658	0.762084702	440.8697098	0.376016042
444.0783008	0.747528632	442.5703298	0.36658356
444.6451748	0.740960276	442.8537658	0.365046121
445.2715778	0.73395766	444.0783008	0.358519531
445.8697098	0.727552873	444.6451748	0.355622277
447.5703298	0.710863398	445.2715778	0.352542643
447.8537658	0.708291736	445.8697098	0.349727228
449.0783008	0.697815361	447.5703298	0.342374466
449.6451748	0.693288697	447.8537658	0.341237976
450.2715778	0.688498563	449.0783008	0.336596396
450.8697098	0.684111314	449.6451748	0.334585817
452.5703298	0.672467777	450.2715778	0.332456928
452.8537658	0.670630507	450.8697098	0.330508316
454.0783008	0.662993838	452.5703298	0.325363313
454.6451748	0.659617657	452.8537658	0.32455709
455.2715778	0.656000661	454.0783008	0.321226502
455.8697098	0.6526567	454.6451748	0.319764851
457.5703298	0.643719762	455.2715778	0.318206168
457.8537658	0.642310079	455.8697098	0.316771592
459.0783008	0.636470198	457.5703298	0.312966698
459.6451748	0.633899587	457.8537658	0.312370323
460.2715778	0.631152315	459.0783008	0.309911049
460.8697098	0.628616999	459.6451748	0.308834621
462.5703298	0.62184804	460.2715778	0.307688723
462.8537658	0.620779408	460.8697098	0.306636019
464.0783008	0.616344129	462.5703298	0.303848605
464.6451748	0.614386284	462.8537658	0.303412288
465.2715778	0.612289508	464.0783008	0.301613942
465.8697098	0.610350416	464.6451748	0.300827165
467.5703298	0.605155628	465.2715778	0.299989862
467.8537658	0.604333563	465.8697098	0.299220757
469.0783008	0.600917824	467.5703298	0.29718788
469.6451748	0.599408725	467.8537658	0.29687021
470.2715778	0.597792166	469.0783008	0.295563465
470.8697098	0.596297076	469.6451748	0.294993417
472.5703298	0.592292951	470.2715778	0.294388173
472.8537658	0.591659588	470.8697098	0.293833706
474.0783008	0.589029037	472.5703298	0.292376654

Final State: j’=1		Final State: j’=0	
E(cm⁻¹)	σ (Å²)	E(cm⁻¹)	σ (Å²)
474.6451748	0.587867448	472.8537658	0.292150284
475.2715778	0.586623571	474.0783008	0.291223667
475.8697098	0.585473553	474.6451748	0.290822038
477.5703298	0.582395569	475.2715778	0.29039759
477.8537658	0.581908983	475.8697098	0.290010728
479.0783008	0.579888992	477.5703298	0.28900477
479.6451748	0.578997572	477.8537658	0.288850093
480.2715778	0.578043437	479.0783008	0.288222399
480.8697098	0.577161753	479.6451748	0.287953431
482.5703298	0.57480445	480.2715778	0.287671546
482.8537658	0.574432172	480.8697098	0.287416997
484.0783008	0.572887954	482.5703298	0.286767888
484.6451748	0.572207146	482.8537658	0.286670025
485.2715778	0.571478919	484.0783008	0.286279505
485.8697098	0.570806413	484.6451748	0.286115918
487.8537658	0.568726787	485.2715778	0.285947361
489.0783008	0.56755137	485.8697098	0.285798041
489.6451748	0.56703321	487.5703298	0.285432842
490.2715778	0.566478849	487.8537658	0.285380174
490.8697098	0.565966688	489.0783008	0.28517817
492.5703298	0.564596385	489.6451748	0.285098234
492.8537658	0.564379616	490.2715778	0.285019499
494.0783008	0.563478134	490.8697098	0.284953427
494.6451748	0.563078943	492.5703298	0.284811701
495.2715778	0.562650167	492.8537658	0.2847944
495.8697098	0.562252077	494.0783008	0.284739084
497.5703298	0.561173636	494.6451748	0.284723691
497.8537658	0.561000621	495.2715778	0.284713776
499.0783008	0.560270922	495.8697098	0.284710952
499.6451748	0.559941176	497.5703298	0.284735503
500.2715778	0.559581117	497.8537658	0.284743894
500.8697098	0.559240192	499.0783008	0.284792568
502.5703298	0.558272646	499.6451748	0.284821251
502.8537658	0.558109586	500.2715778	0.284856848
504.0783008	0.557388678	500.8697098	0.284894141
504.6451748	0.557041097	502.5703298	0.285012586
505.2715778	0.556641744	502.8537658	0.285033331
505.8697098	0.556240723	504.0783008	0.285122257
507.5703298	0.554937053	504.6451748	0.285161225
507.8537658	0.554685745	505.2715778	0.285200742
509.6451748	0.55268939	505.8697098	0.285233221
510.2715778	0.551719399	507.5703298	0.285275281
510.8697098	0.550552901	507.8537658	0.285271029
512.5703298	0.543786611	509.0783008	0.285188584
512.8537658	0.541172467	509.6451748	0.285098308
514.0783008	0.535609844	510.2715778	0.284936334
514.6451748	0.55509565	510.8697098	0.28468802
515.2715778	0.544620554	512.5703298	0.282517439
515.8697098	0.546673402	512.8537658	0.281452599
517.5703298	0.536538572	514.0783008	0.274373786
517.8537658	0.537299883	514.6451748	0.283799296

Final State: j'=1		Final State: j'=0	
E(cm⁻¹)	σ (Å²)	E(cm⁻¹)	σ (Å²)
519.0783008	0.560506675	515.2715778	0.282067855
519.6451748	0.550653398	515.8697098	0.279414752
520.2715778	0.561418499	517.5703298	0.278205062
520.8697098	0.546416819	517.8537658	0.277367516
522.5703298	0.551209962	519.0783008	0.286319442
522.8537658	0.558437023	519.6451748	0.284731917
524.0783008	0.561958032	520.2715778	0.290160971
524.6451748	0.552281249	520.8697098	0.285020831
525.2715778	0.540527783	522.5703298	0.285159959
525.8697098	0.559351385	522.8537658	0.282695049
527.5703298	0.575225894	524.0783008	0.292722404
527.8537658	0.571646327	524.6451748	0.289099222
529.6451748	0.546585007	525.2715778	0.280878453
530.2715778	0.555472149	525.8697098	0.288058921
530.8697098	0.565810407	527.5703298	0.298628607
532.5703298	0.576604898	527.8537658	0.297706145
532.8537658	0.574166556	529.0783008	0.293799416
534.0783008	0.565769819	529.6451748	0.288533504
534.6451748	0.559920827	530.2715778	0.287202626
535.2715778	0.543528721	530.8697098	0.293859034
535.8697098	0.559923343	532.5703298	0.301444406
537.5703298	0.585329311	532.8537658	0.300803146
537.8537658	0.580899703	534.0783008	0.298437005
539.0783008	0.572637834	534.6451748	0.296445227
539.6451748	0.569813821	535.2715778	0.289035712
540.2715778	0.566069988	535.8697098	0.291484343
540.8697098	0.560138206	537.5703298	0.306292593
542.5703298	0.565306825	537.8537658	0.305193348
542.8537658	0.567302031	539.0783008	0.303072085
544.0783008	0.57772015	539.6451748	0.302342728
544.6451748	0.572980536	540.2715778	0.301272766
545.2715778	0.568703575	540.8697098	0.299289962
545.8697098	0.56412221	542.5703298	0.300534287
547.5703298	0.547093414	542.8537658	0.29515094
547.8537658	0.553613279	544.0783008	0.30687493
549.0783008	0.563656735	544.6451748	0.305536418
549.6451748	0.578939859	545.2715778	0.304284308
550.2715778	0.577748681	545.8697098	0.30280328
550.8697098	0.565897533	547.5703298	0.29124889
552.5703298	0.56542398	547.8537658	0.294657777
552.8537658	0.551575987	549.0783008	0.293932439
554.0783008	0.594798914	549.6451748	0.301668333
554.6451748	0.581051296	550.2715778	0.306297149
555.2715778	0.563057298	550.8697098	0.2958545
555.8697098	0.52002648	552.5703298	0.304476897
557.5703298	0.609551161	552.8537658	0.296461506
557.8537658	0.599248533	554.0783008	0.31526568
559.0783008	0.564895093	554.6451748	0.312151935
559.6451748	0.561006876	555.2715778	0.305887399
560.2715778	0.616225038	555.8697098	0.284067933
560.8697098	0.600373157	557.5703298	0.322984554

Final State: j’=1		Final State: j’=0	
E(cm⁻¹)	σ (Å²)	E(cm⁻¹)	σ (Å²)
562.5703298	0.57453691	557.8537658	0.320252552
562.8537658	0.565815474	559.0783008	0.307405385
564.0783008	0.60102528	559.6451748	0.296153714
564.6451748	0.589090319	560.2715778	0.324386553
565.2715778	0.588681839	560.8697098	0.322154164
565.8697098	0.582025416	562.5703298	0.314226665
567.5703298	0.550890294	562.8537658	0.31086879
567.8537658	0.542548148	564.0783008	0.318524223
569.0783008	0.598337184	564.6451748	0.316749451
569.6451748	0.585948367	565.2715778	0.317671194
570.2715778	0.608610498	565.8697098	0.313031872
570.8697098	0.588457048	567.5703298	0.304056176
572.5703298	0.567334118	567.8537658	0.29205087
572.8537658	0.595501156	569.0783008	0.324937532
574.0783008	0.619074489	569.6451748	0.310699689
574.6451748	0.594434715	570.2715778	0.328605963
575.2715778	0.58469563	570.8697098	0.323011654
575.8697098	0.586059821	572.5703298	0.306288164
577.5703298	0.569433408	572.8537658	0.317878405
577.8537658	0.58706646	574.0783008	0.332777019
579.0783008	0.576764508	574.6451748	0.323600658
579.6451748	0.572446455	575.2715778	0.322546477
580.2715778	0.566882607	575.8697098	0.32106486
580.8697098	0.556727849	577.5703298	0.313961535
582.5703298	0.576780459	577.8537658	0.323896539
582.8537658	0.576793484	579.0783008	0.321743756
584.0783008	0.574143475	579.6451748	0.320591446
584.6451748	0.573167369	580.2715778	0.319039877
585.2715778	0.571745053	580.8697098	0.315861868
585.8697098	0.569611153	582.5703298	0.323779029
587.5703298	0.569991998	582.8537658	0.323854496
587.8537658	0.57031775	584.0783008	0.323539729
589.0783008	0.567789463	584.6451748	0.32350792
589.6451748	0.565660777	585.2715778	0.32339147
590.2715778	0.573769244	585.8697098	0.323104463
590.8697098	0.574701289	587.5703298	0.324245429
592.5703298	0.574009927	587.8537658	0.324473452
592.8537658	0.574082877	589.0783008	0.324308013
594.0783008	0.574518037	589.6451748	0.324768159
594.6451748	0.574723462	590.2715778	0.327053431
595.2715778	0.574918929	590.8697098	0.327463661
595.8697098	0.575036092	592.5703298	0.328152667
597.5703298	0.574017542	592.8537658	0.328325746
597.8537658	0.574259466	594.0783008	0.329108241
599.0783008	0.576679058	594.6451748	0.329473
599.6451748	0.577237417	595.2715778	0.329869332
600.2715778	0.577706165	595.8697098	0.330232672
600.8697098	0.578098978	597.5703298	0.331105515
602.5703298	0.57911598	597.8537658	0.331378427
602.8537658	0.579276531	599.0783008	0.332613613
604.0783008	0.579936272	599.6451748	0.333046904

Final State: j’=1		Final State: j’=0	
E(cm^{-1})	σ (Å^2)	E(cm^{-1})	σ (Å^2)
604.6451748	0.580222363	600.2715778	0.333502103
605.2715778	0.580534235	600.8697098	0.333930166
605.8697098	0.580847187	602.5703298	0.335140048
607.5703298	0.581908409	602.8537658	0.335341143
607.8537658	0.582101246	604.0783008	0.336207486
609.0783008	0.582937828	604.6451748	0.336608092
609.6451748	0.583320869	605.2715778	0.337053367
610.2715778	0.5837394	605.8697098	0.337484507
610.8697098	0.58413489	607.5703298	0.338744201
612.5703298	0.585240865	607.8537658	0.338956137
612.8537658	0.585423151	609.0783008	0.339870024
614.0783008	0.586207308	609.6451748	0.340291681
614.6451748	0.586569922	610.2715778	0.340756887
615.2715778	0.586971569	610.8697098	0.341200476
615.8697098	0.587356716	612.5703298	0.342460312
617.5703298	0.588463088	612.8537658	0.342670282
617.8537658	0.588649085	614.0783008	0.343578196
619.0783008	0.589456882	614.6451748	0.343999174
619.6451748	0.589832697	615.2715778	0.344465029
620.2715778	0.590248997	615.8697098	0.344910566
620.8697098	0.590647311	617.5703298	0.346180951
622.5703298	0.591782943	617.8537658	0.346393139
622.8537658	0.591972589	619.0783008	0.347311057
624.0783008	0.592793067	619.6451748	0.347736556
624.6451748	0.593173557	620.2715778	0.348207096
625.2715778	0.593594542	620.8697098	0.348656724
625.8697098	0.593997098	622.5703298	0.349936721
627.5703298	0.595145072	622.8537658	0.350150277
627.8537658	0.595336932	624.0783008	0.351073655
629.0783008	0.596167639	624.6451748	0.351501528
629.6451748	0.596553206	625.2715778	0.351974641
630.2715778	0.596980006	625.8697098	0.352426708
630.8697098	0.597388262	627.5703298	0.35371366
632.5703298	0.598552745	627.8537658	0.353928385
632.8537658	0.598747344	629.0783008	0.354856797
634.0783008	0.599589719	629.6451748	0.355286979
634.6451748	0.599980563	630.2715778	0.355762618
635.2715778	0.600413088	630.8697098	0.356217056
635.8697098	0.600826707	632.5703298	0.357510479
637.5703298	0.602005959	632.8537658	0.357726237
637.8537658	0.602202962	634.0783008	0.358658955
639.0783008	0.603055585	634.6451748	0.359091046
639.6451748	0.603451113	635.2715778	0.359568732
640.2715778	0.603888782	635.8697098	0.360025068
640.8697098	0.604307292	637.5703298	0.3613236
642.5703298	0.605500358	637.8537658	0.36154017
642.8537658	0.605699653	639.0783008	0.362476281
644.0783008	0.60656215	639.6451748	0.362909881
644.6451748	0.606962233	640.2715778	0.363389187
645.2715778	0.607404922	640.8697098	0.363847025
645.8697098	0.60782821	642.5703298	0.3651496

Final State: j'=1		Final State: j'=0	
E(cm⁻¹)	σ (Å²)	E(cm⁻¹)	σ (Å²)
647.5703298	0.609034773	642.8537658	0.36536681
647.8537658	0.609236308	644.0783008	0.366305581
649.0783008	0.610108432	644.6451748	0.366740353
649.6451748	0.610512948	645.2715778	0.367220911
650.2715778	0.610960523	645.8697098	0.367679903
650.8697098	0.611388462	647.5703298	0.368985532
652.5703298	0.612608194	647.8537658	0.369203221
652.8537658	0.612811913	649.0783008	0.370143956
654.0783008	0.613693471	649.6451748	0.37057958
654.6451748	0.614102351	650.2715778	0.371061045
655.2715778	0.614554745	650.8697098	0.371520861
655.8697098	0.614987284	652.5703298	0.372828641
657.5703298	0.616220107	652.8537658	0.37304666
657.8537658	0.616426014	654.0783008	0.373988733
659.0783008	0.617317029	654.6451748	0.37442493
659.6451748	0.617730302	655.2715778	0.374906993
660.2715778	0.61818756	655.8697098	0.37536735
660.8697098	0.618624757	657.5703298	0.376676493
662.5703298	0.619870915	657.8537658	0.376894715
662.8537658	0.620079058	659.0783008	0.377837594
664.0783008	0.620979792	659.6451748	0.378274126
664.6451748	0.621397595	660.2715778	0.378756529
665.2715778	0.621859888	660.8697098	0.379217185
665.8697098	0.622301925	662.5703298	0.380527045
667.5703298	0.62356202	662.8537658	0.380745368
667.8537658	0.623772515	664.0783008	0.38168863
669.0783008	0.624683526	664.6451748	0.382125308
669.6451748	0.625106152	665.2715778	0.382607856
670.2715778	0.625573825	665.8697098	0.38306863
670.8697098	0.626021055	667.5703298	0.384378751
672.5703298	0.627296237	667.8537658	0.38459711
672.8537658	0.627509297	669.0783008	0.385540489
674.0783008	0.628431577	669.6451748	0.385977211
674.6451748	0.628859533	670.2715778	0.386459799
675.2715778	0.629333186	670.8697098	0.386920611
675.8697098	0.629786221	672.5703298	0.388230825
677.5703298	0.631078454	672.8537658	0.3884492
677.8537658	0.631294443	674.0783008	0.389392673
679.0783008	0.632229699	674.6451748	0.389829453
679.6451748	0.63266385	675.2715778	0.390312121
680.2715778	0.633144503	675.8697098	0.390773025
680.8697098	0.633604378	677.5703298	0.392083629
682.5703298	0.634917032	677.8537658	0.39230209
682.8537658	0.635136581	679.0783008	0.393246031
684.0783008	0.636087779	679.6451748	0.393683086
684.6451748	0.636529658	680.2715778	0.394166106
685.2715778	0.637019132	680.8697098	0.394627404
685.8697098	0.637487734	682.5703298	0.395939486
687.5703298	0.638827039	682.8537658	0.396158254
687.8537658	0.63905133	684.0783008	0.397103759
689.0783008	0.640024185	684.6451748	0.397541685

Final State: j’=1		Final State: j’=0	
E(cm⁻¹)	σ (Å²)	E(cm⁻¹)	σ (Å²)
689.6451748	0.640476809	685.2715778	0.398025797
690.2715778	0.640978763	685.8697098	0.398488273
690.8697098	0.641459937	687.5703298	0.399804573
692.5703298	0.642839251	687.8537658	0.400024189
692.8537658	0.643070935	689.0783008	0.400973945
694.0783008	0.644078717	689.6451748	0.401414208
694.6451748	0.644549433	690.2715778	0.401901214
695.2715778	0.645073308	690.8697098	0.402366799
695.8697098	0.645576976	692.5703298	0.403694219
697.5703298	0.647034786	692.8537658	0.403916085
697.8537658	0.647282164	694.0783008	0.404877209
699.0783008	0.648370113	694.6451748	0.405323809
699.6451748	0.648886861	695.2715778	0.405818788
700.2715778	0.649470617	695.8697098	0.406293106
700.8697098	0.650043964	697.5703298	0.407653377
702.5703298	0.651817845	697.8537658	0.40788223
702.8537658	0.652149088	699.0783008	0.408880759
704.0783008	0.653890596	699.6451748	0.409349914
704.6451748	0.655170124	700.2715778	0.409875263
705.2715778	0.655335086	700.8697098	0.410385751
705.8697098	0.652830954	702.5703298	0.411919693
707.5703298	0.65289263	702.8537658	0.412196255
707.8537658	0.653313562	704.0783008	0.413578649
709.0783008	0.65798477	704.6451748	0.414515491
709.6451748	0.65663051	705.2715778	0.414887
710.2715778	0.65540273	705.8697098	0.413583752
710.8697098	0.655671565	707.5703298	0.413936484
712.5703298	0.660819453	707.8537658	0.414231086
712.8537658	0.660656644	709.0783008	0.417477401
714.0783008	0.660193176	709.6451748	0.416716831
714.6451748	0.656529627	710.2715778	0.416166368
715.2715778	0.659580152	710.8697098	0.41658274
715.8697098	0.661471718	712.5703298	0.419858022
717.5703298	0.660323277	712.8537658	0.419852339
717.8537658	0.660315654	714.0783008	0.420228217
719.0783008	0.661338472	714.6451748	0.417940068
719.6451748	0.663022581	715.2715778	0.419931752
720.2715778	0.664539881	715.8697098	0.421165562
720.8697098	0.666333235	717.5703298	0.421331958
722.5703298	0.666323007	717.8537658	0.421337671
722.8537658	0.661436305	719.0783008	0.421986339
724.0783008	0.665702073	719.6451748	0.423092596
724.6451748	0.667013206	720.2715778	0.42410874
725.2715778	0.668271599	720.8697098	0.425243866
725.8697098	0.669579994	722.5703298	0.426133263
727.5703298	0.6676929	722.8537658	0.422940225
727.8537658	0.668638141	724.0783008	0.425819966
729.0783008	0.668571885	724.6451748	0.426703586
729.6451748	0.670523936	725.2715778	0.427564603
730.2715778	0.671816831	725.8697098	0.42843134
730.8697098	0.672891463	727.5703298	0.428044446

Final State: j’=1		Final State: j’=0	
E(cm^{-1})	σ (Å^2)	E(cm^{-1})	σ (Å^2)
732.5703298	0.676743024	727.8537658	0.428550568
732.8537658	0.678142416	729.0783008	0.428783804
734.0783008	0.675584999	729.6451748	0.430020564
734.6451748	0.673435611	730.2715778	0.430896954
735.2715778	0.675079947	730.8697098	0.43164003
735.8697098	0.676945076	732.5703298	0.434088455
737.5703298	0.680752433	732.8537658	0.434805051
737.8537658	0.681542073	734.0783008	0.434191359
739.0783008	0.682746583	734.6451748	0.433030917
739.6451748	0.682834179	735.2715778	0.434079749
740.2715778	0.682236487	735.8697098	0.435264889
740.8697098	0.68017377	737.5703298	0.437844042
742.5703298	0.678917396	737.8537658	0.438380041
742.8537658	0.677515726	739.0783008	0.439692839
744.0783008	0.685921642	739.6451748	0.439216522
744.6451748	0.685967062	740.2715778	0.439202397
745.2715778	0.677899689	740.8697098	0.438757743
745.8697098	0.683628971	742.5703298	0.438674768
747.5703298	0.689155585	742.8537658	0.437477135
747.8537658	0.680732121	744.0783008	0.442197171
749.0783008	0.68267128	744.6451748	0.442839833
749.6451748	0.686870715	745.2715778	0.437983613
750.2715778	0.690127195	745.8697098	0.441016391
750.8697098	0.693659726	747.5703298	0.44427494
752.5703298	0.688916361	747.8537658	0.440449501
752.8537658	0.690044164	749.0783008	0.441406837
754.0783008	0.694811406	749.6451748	0.44383573
754.6451748	0.699410138	750.2715778	0.445788295
755.2715778	0.691853665	750.8697098	0.448174079
755.8697098	0.688092248	752.5703298	0.445456533
757.5703298	0.694268764	752.8537658	0.446148891
757.8537658	0.694740444	754.0783008	0.449017946
759.0783008	0.702658384	754.6451748	0.451539278
759.6451748	0.688370987	755.2715778	0.446725104
760.2715778	0.692623927	755.8697098	0.446132956
760.8697098	0.70082692	757.5703298	0.450204019
762.5703298	0.702598597	757.8537658	0.450301349
762.8537658	0.703656435	759.0783008	0.454334388
764.0783008	0.698305223	759.6451748	0.447107159
764.6451748	0.696503253	760.2715778	0.449079067
765.2715778	0.694293688	760.8697098	0.454168496
765.8697098	0.698651901	762.5703298	0.454979678
767.5703298	0.697458923	762.8537658	0.456289142
767.8537658	0.702127456	764.0783008	0.452393003
769.0783008	0.700907575	764.6451748	0.45239879
769.6451748	0.701504103	765.2715778	0.451794488
770.2715778	0.703369485	765.8697098	0.453533478
770.8697098	0.704734203	767.5703298	0.453561327
772.5703298	0.706601914	767.8537658	0.456060586
772.8537658	0.707161351	769.0783008	0.455257154
774.0783008	0.705127256	769.6451748	0.456252216

Final State: j’=1		Final State: j’=0	
E(cm⁻¹)	σ (Å²)	E(cm⁻¹)	σ (Å²)
774.6451748	0.706488262	770.2715778	0.457310231
775.2715778	0.707421303	770.8697098	0.458102803
775.8697098	0.708157528	772.5703298	0.459244592
777.5703298	0.709943418	772.8537658	0.459746383
777.8537658	0.71010458	774.0783008	0.459021455
779.0783008	0.71086075	774.6451748	0.459813161
779.6451748	0.711417917	775.2715778	0.460387286
780.2715778	0.712167	775.8697098	0.46085761
780.8697098	0.712769148	777.5703298	0.462006669
782.5703298	0.713375879	777.8537658	0.462129226
782.8537658	0.713666867	779.0783008	0.46274595
784.0783008	0.714761958	779.6451748	0.46312443
784.6451748	0.715243316	780.2715778	0.463579098
785.2715778	0.715772516	780.8697098	0.463708795
785.8697098	0.716277832	782.5703298	0.464697783
787.5703298	0.717726375	782.8537658	0.464885492
787.8537658	0.717971407	784.0783008	0.465630265
789.0783008	0.718927629	784.6451748	0.465964926
789.6451748	0.719252442	785.2715778	0.466333086
790.2715778	0.719734669	785.8697098	0.46668361
790.8697098	0.720242	787.5703298	0.467675361
792.5703298	0.721672445	787.8537658	0.467839431
792.8537658	0.721908454	789.0783008	0.468487966
794.0783008	0.722924904	789.6451748	0.468762378
794.6451748	0.723394439	790.2715778	0.469120182
795.2715778	0.723912607	790.8697098	0.469471085
795.8697098	0.724406207	792.5703298	0.470444056
797.5703298	0.725795442	792.8537658	0.47060386
797.8537658	0.726024998	794.0783008	0.471289628
799.0783008	0.727017907	794.6451748	0.471604876
799.6451748	0.727479842	795.2715778	0.471951597
800.2715778	0.727991989	795.8697098	0.472280844
800.8697098	0.728482298	797.5703298	0.473205682
802.5703298	0.729880409	797.8537658	0.473358574
802.8537658	0.730113787	799.0783008	0.47401818
804.0783008	0.731122671	799.6451748	0.474323252
804.6451748	0.731589906	800.2715778	0.474659913
805.2715778	0.732106253	800.8697098	0.474980716
805.8697098	0.732599298	802.5703298	0.475888124
807.5703298	0.734001151	802.8537658	0.476038601
807.8537658	0.734234836	804.0783008	0.476686041
809.0783008	0.735244799	804.6451748	0.476984252
809.6451748	0.735712616	805.2715778	0.477312655
810.2715778	0.736229813	805.8697098	0.477625132
810.8697098	0.736723924	807.5703298	0.478507788
812.5703298	0.738130215	807.8537658	0.478654089
812.8537658	0.738364797	809.0783008	0.479283591
814.0783008	0.739378897	809.6451748	0.479573616
814.6451748	0.739848684	810.2715778	0.479893076
815.2715778	0.740368037	810.8697098	0.480197121
815.8697098	0.740864171	812.5703298	0.481056225

Final State: j’=1		Final State: j’=0	
E(cm⁻¹)	σ (Å²)	E(cm⁻¹)	σ (Å²)
817.5703298	0.742275923	812.8537658	0.481198631
817.8537658	0.742511374	814.0783008	0.481811296
819.0783008	0.743529126	814.6451748	0.482093492
819.6451748	0.744000563	815.2715778	0.482404266
820.2715778	0.744521725	815.8697098	0.482699979
820.8697098	0.745019577	817.5703298	0.483535198
822.5703298	0.746436235	817.8537658	0.4836736
822.8537658	0.746672515	819.0783008	0.484268912
824.0783008	0.747693863	819.6451748	0.484543052
824.6451748	0.748166977	820.2715778	0.484844912
825.2715778	0.748690005	820.8697098	0.485132103
825.8697098	0.749189647	822.5703298	0.485943067
827.5703298	0.750611416	822.8537658	0.486077423
827.8537658	0.750848547	824.0783008	0.486655238
829.0783008	0.751873575	824.6451748	0.486921268
829.6451748	0.752348392	825.2715778	0.487214164
830.2715778	0.752873298	825.8697098	0.487492787
830.8697098	0.753374729	827.5703298	0.488279341
832.5703298	0.754801571	827.8537658	0.488409623
832.8537658	0.755039543	829.0783008	0.48896981
834.0783008	0.756068207	829.6451748	0.489227664
834.6451748	0.756544708	830.2715778	0.489511523
835.2715778	0.757071465	830.8697098	0.489781504
835.8697098	0.757574668	832.5703298	0.490543446
837.5703298	0.75900653	832.8537658	0.49066962
837.8537658	0.75924534	834.0783008	0.491212039
839.0783008	0.760277613	834.6451748	0.491461663
839.6451748	0.760755777	835.2715778	0.491736412
840.2715778	0.761284377	835.8697098	0.491997691
840.8697098	0.761789333	837.5703298	0.492734858
842.5703298	0.763226167	837.8537658	0.492856898
842.8537658	0.763465804	839.0783008	0.493381445
844.0783008	0.764501637	839.6451748	0.493622787
844.6451748	0.764981448	840.2715778	0.493888378
845.2715778	0.765511857	840.8697098	0.494140907
845.8697098	0.766018539	842.5703298	0.49485317
847.5703298	0.767460264	842.8537658	0.494971055
847.8537658	0.767700708	844.0783008	0.495477639
849.0783008	0.768740035	844.6451748	0.49571066
849.6451748	0.769221456	845.2715778	0.49596705
850.2715778	0.76975364	845.8697098	0.496210791
850.8697098	0.770262012	847.5703298	0.49689804
852.5703298	0.77170851	847.8537658	0.497011751
852.8537658	0.77194975	849.0783008	0.497500303
854.0783008	0.772992483	849.6451748	0.497724968
854.6451748	0.773475472	850.2715778	0.497972125
855.2715778	0.774009386	850.8697098	0.498207043
855.8697098	0.774519401	852.5703298	0.498869195
857.5703298	0.775970541	852.8537658	0.498978723
857.8537658	0.776212548	854.0783008	0.499449186
859.0783008	0.777258585	854.6451748	0.499665475

Final State: j’=1		Final State: j’=0	
E(cm^{-1})	σ (Å^2)	E(cm^{-1})	σ (Å^2)
859.6451748	0.777743095	855.2715778	0.499903371
860.2715778	0.778278683	855.8697098	0.500129447
860.8697098	0.778790287	857.5703298	0.500766444
862.5703298	0.780245909	857.8537658	0.500871777
862.8537658	0.780488658	859.0783008	0.501324117
864.0783008	0.781537879	859.6451748	0.501532014
864.6451748	0.782023852	860.2715778	0.501760637
865.2715778	0.782561049	860.8697098	0.501977856
865.8697098	0.783074182	862.5703298	0.502589664
867.5703298	0.784534106	862.8537658	0.502690799
867.8537658	0.784777565	864.0783008	0.503124996
869.0783008	0.785829834	864.6451748	0.503324497
869.6451748	0.786317207	865.2715778	0.503543836
870.2715778	0.786855939	865.8697098	0.503752192
870.8697098	0.787370531	867.5703298	0.504338802
872.5703298	0.788834551	867.8537658	0.504435738
872.8537658	0.789078684	869.0783008	0.504851793
874.0783008	0.790133847	869.6451748	0.505042892
874.6451748	0.790622544	870.2715778	0.505252953
875.2715778	0.791162733	870.8697098	0.505452449
875.8697098	0.791678702	872.5703298	0.506013871
877.5703298	0.793146588	872.8537658	0.506106609
877.8537658	0.79339136	874.0783008	0.506504532
879.0783008	0.794449245	874.6451748	0.506687238
879.6451748	0.794939189	875.2715778	0.506888027
880.2715778	0.795480743	875.8697098	0.507078672
880.8697098	0.795998009	877.5703298	0.507614933
882.5703298	0.797469521	877.8537658	0.507703478
882.8537658	0.797714888	879.0783008	0.508083295
884.0783008	0.798775322	879.6451748	0.508257622
884.6451748	0.799266426	880.2715778	0.508449154
885.2715778	0.799809263	880.8697098	0.50863096
885.8697098	0.800327735	882.5703298	0.509142105
887.5703298	0.801802627	882.8537658	0.509226467
887.8537658	0.80204855	884.0783008	0.509588212
889.0783008	0.803111359	884.6451748	0.509754144
889.6451748	0.803603552	885.2715778	0.509936473
890.2715778	0.80414757	885.8697098	0.510109461
890.8697098	0.804667167	887.5703298	0.510595548
892.5703298	0.806145203	887.8537658	0.510675735
892.8537658	0.806391642	889.0783008	0.511019456
894.0783008	0.807456658	889.6451748	0.511177079
894.6451748	0.807949862	890.2715778	0.51135016
895.2715778	0.808494988	890.8697098	0.51151435
895.8697098	0.809015638	892.5703298	0.511975445
897.5703298	0.810496615	892.8537658	0.512051468
897.8537658	0.810743538	894.0783008	0.512377204
899.0783008	0.811810637	894.6451748	0.512526508
899.6451748	0.812304797	895.2715778	0.512690396
900.2715778	0.812850976	895.8697098	0.512845811
900.8697098	0.813372626	897.5703298	0.513281965

Final State: j'=1		Final State: j'=0	
E(cm⁻¹)	σ (Å²)	E(cm⁻¹)	σ (Å²)
902.5703298	0.814856445	897.8537658	0.513353832
902.8537658	0.815103842	899.0783008	0.513661621
904.0783008	0.816172993	899.6451748	0.513802615
904.6451748	0.816668109	900.2715778	0.513957322
905.2715778	0.817215352	900.8697098	0.514103974
905.8697098	0.817738027	902.5703298	0.514515202
907.5703298	0.819224841	902.8537658	0.514582916
907.8537658	0.819472752	904.0783008	0.514872745
909.0783008	0.82054419	904.6451748	0.515005423
909.6451748	0.821040413	905.2715778	0.515150934
910.2715778	0.821588919	905.8697098	0.515288799
910.8697098	0.822112852	907.5703298	0.515675008
912.5703298	0.823603586	907.8537658	0.515738545
912.8537658	0.823852216	909.0783008	0.516010294
914.0783008	0.824927047	909.6451748	0.516134582
914.6451748	0.825425041	910.2715778	0.516270802
915.2715778	0.825975694	910.8697098	0.516399772
915.8697098	0.826501902	912.5703298	0.516760532
917.5703298	0.828000863	912.8537658	0.516819796
917.8537658	0.828251218	914.0783008	0.517072971
919.0783008	0.829335374	914.6451748	0.51718857
919.6451748	0.829839159	915.2715778	0.517315112
920.2715778	0.830397926	915.8697098	0.517434744
920.8697098	0.830934402	917.5703298	0.517768232
922.5703298	0.832498459	917.8537658	0.51782281
922.8537658	0.832774504	919.0783008	0.518055013
924.0783008	0.8344905	919.6451748	0.518160361
924.6451748	0.835159119	920.2715778	0.518274972
925.2715778	0.834864662	920.8697098	0.518382358
925.8697098	0.835904363	922.5703298	0.51867185
927.5703298	0.836897739	922.8537658	0.518716452
927.8537658	0.837472574	924.0783008	0.519112562
929.0783008	0.838307111	924.6451748	0.519511922
929.6451748	0.838986164	925.2715778	0.519345385
930.2715778	0.839311838	925.8697098	0.519435459
930.8697098	0.839672719	927.5703298	0.519618082
932.5703298	0.841141623	927.8537658	0.519678171
932.8537658	0.841651002	929.0783008	0.520187664
934.0783008	0.842320019	929.6451748	0.52023504
934.6451748	0.842916744	930.2715778	0.520336438
935.2715778	0.843751831	930.8697098	0.52023897
935.8697098	0.845221071	932.5703298	0.520639829
937.5703298	0.845558596	932.8537658	0.520605396
937.8537658	0.84550973	934.0783008	0.5207766
939.0783008	0.846730774	934.6451748	0.520814504
939.6451748	0.847338429	935.2715778	0.5209061
940.2715778	0.848645363	935.8697098	0.521659271
940.8697098	0.849202206	937.5703298	0.521609953
942.5703298	0.84958206	937.8537658	0.521384467
942.8537658	0.849869997	939.0783008	0.521459009
944.0783008	0.851047012	939.6451748	0.521522525

Final State: j'=1		Final State: j'=0	
E(cm⁻¹)	σ (Å²)	E(cm⁻¹)	σ (Å²)
944.6451748	0.851598706	940.2715778	0.521869302
945.2715778	0.852279675	940.8697098	0.522249518
945.8697098	0.853783147	942.5703298	0.522018867
947.5703298	0.854268543	942.8537658	0.522016227
947.8537658	0.854153299	944.0783008	0.522117925
949.0783008	0.855358529	944.6451748	0.52217696
949.6451748	0.855889016	945.2715778	0.522256004
950.2715778	0.856477107	945.8697098	0.522775651
950.8697098	0.857053804	947.5703298	0.522794584
952.5703298	0.859266805	947.8537658	0.522682514
952.8537658	0.859324003	949.0783008	0.522704235
954.0783008	0.859666809	949.6451748	0.522754475
954.6451748	0.860252708	950.2715778	0.522812893
955.2715778	0.860866902	950.8697098	0.522871545
955.8697098	0.861457534	952.5703298	0.523479922
957.5703298	0.863866903	952.8537658	0.523243599
957.8537658	0.864536231	954.0783008	0.523232576
959.0783008	0.866181431	954.6451748	0.523238155
959.6451748	0.866090788	955.2715778	0.523273085
960.2715778	0.865502648	955.8697098	0.523307575
960.8697098	0.866636127	957.5703298	0.523571943
962.5703298	0.867178748	957.8537658	0.523802326
962.8537658	0.867625888	959.0783008	0.524352307
964.0783008	0.869444965	959.6451748	0.524441727
964.6451748	0.868740839	960.2715778	0.524076793
965.2715778	0.869494438	960.8697098	0.523970628
965.8697098	0.870494441	962.5703298	0.524052791
967.5703298	0.871260792	962.8537658	0.524017974
967.8537658	0.871621303	964.0783008	0.525044954
969.0783008	0.872792938	964.6451748	0.524373899
969.6451748	0.874479295	965.2715778	0.5242788
970.2715778	0.875606943	965.8697098	0.524289225
970.8697098	0.873882144	967.5703298	0.52473903
972.5703298	0.875722945	967.8537658	0.524730025
972.8537658	0.876058987	969.0783008	0.524452346
974.0783008	0.879463193	969.6451748	0.524653161
974.6451748	0.878210724	970.2715778	0.525923468
975.2715778	0.87853167	970.8697098	0.524830386
975.8697098	0.878854309	972.5703298	0.524669023
977.5703298	0.881124949	972.8537658	0.524669823
977.8537658	0.880984001	974.0783008	0.526232847
979.0783008	0.880901721	974.6451748	0.525255387
979.6451748	0.882071274	975.2715778	0.524960129
980.2715778	0.884031208	975.8697098	0.524873367
980.8697098	0.882575208	977.5703298	0.525400302
982.5703298	0.885351293	977.8537658	0.525080296
982.8537658	0.887215504	979.0783008	0.525297063
984.0783008	0.887314941	979.6451748	0.524971563
984.6451748	0.885789441	980.2715778	0.525987386
985.2715778	0.886558101	980.8697098	0.525206216
985.8697098	0.887851511	982.5703298	0.525617311

Final State: j’=1		Final State: j’=0	
E(cm⁻¹)	σ (Å²)	E(cm⁻¹)	σ (Å²)
987.5703298	0.890611862	982.8537658	0.526099538
987.8537658	0.890652829	984.0783008	0.526137791
989.0783008	0.890502127	984.6451748	0.525701526
989.6451748	0.891031897	985.2715778	0.525268779
990.2715778	0.891652191	985.8697098	0.525333512
990.8697098	0.892266102	987.5703298	0.526269278
992.5703298	0.893874747	987.8537658	0.52629241
992.8537658	0.893929328	989.0783008	0.525729547
994.0783008	0.894937678	989.6451748	0.525739933
994.6451748	0.895438059	990.2715778	0.525785373
995.2715778	0.89599365	990.8697098	0.52585882
995.8697098	0.896534965	992.5703298	0.52601865
997.5703298	0.898044398	992.8537658	0.525924095
997.8537658	0.898277308	994.0783008	0.525902342
999.0783008	0.899316359	994.6451748	0.52591822
999.6451748	0.89994379	995.2715778	0.52593792
1000.271578	0.900477499	995.8697098	0.525962935
1000.869708	0.900793014	997.5703298	0.526027045
1002.570328	0.902266538	997.8537658	0.526026649
1002.853768	0.902508365	999.0783008	0.526042434
1004.078298	0.903548859	999.6451748	0.526138759
1004.645178	0.904029488	1000.271578	0.526100729
1005.271578	0.904560438	1000.869708	0.526027506
1005.869708	0.905067642	1002.570328	0.526049316
1007.570328	0.906525329	1002.853768	0.526051264
1007.853768	0.906772975	1004.078298	0.5260561
1009.078298	0.907787868	1004.645178	0.526057103
1009.645178	0.908256631	1005.271578	0.526057676
1010.271578	0.908778946	1005.869708	0.526057932
1010.869708	0.909279095	1007.570328	0.526064031
1012.570328	0.91070183	1007.853768	0.526065711
1012.853768	0.91093882	1009.078298	0.526045682
1014.078298	0.911962139	1009.645178	0.526037274
1014.645178	0.912435611	1010.271578	0.526029578
1015.271578	0.912958606	1010.869708	0.526022385
1015.869708	0.913457627	1012.570328	0.525999739
1017.570328	0.914872067	1012.853768	0.525995555
1017.853768	0.915107107	1014.078298	0.52597611
1019.078298	0.916120971	1014.645178	0.52596637
1019.645178	0.916589624	1015.271578	0.525955024
1020.271578	0.917107046	1015.869708	0.525943506
1020.869708	0.917600683	1017.570328	0.525906169
1022.570328	0.91900187	1017.853768	0.525899318
1022.853768	0.919235056	1019.078298	0.525868092
1024.078298	0.920241309	1019.645178	0.525852819
1024.645178	0.920706456	1020.271578	0.525835363
1025.271578	0.921219928	1020.869708	0.525818123
1025.869708	0.921709698	1022.570328	0.525766047
1027.570328	0.923099296	1022.853768	0.525756925
1029.078298	0.924327769	1024.078298	0.525716042
1029.645178	0.924788667	1024.645178	0.525696298

Final State: j'=1		Final State: j'=0	
E(cm⁻¹)	σ (Å²)	E(cm⁻¹)	σ (Å²)
1030.271578	0.925297385	1025.271578	0.525673877
1030.869708	0.925782581	1025.869708	0.525651868
1032.570328	0.927159064	1027.570328	0.525586109
1034.078298	0.928375831	1029.078298	0.525523902
1034.645178	0.928832304	1029.645178	0.525499587
1035.271578	0.929336112	1030.271578	0.525472132
1035.869708	0.929816593	1030.869708	0.525445347
1037.570328	0.93117951	1032.570328	0.525366184
1039.078298	0.932384025	1034.078298	0.525292309
1039.645178	0.932835833	1034.645178	0.525263652
1040.271578	0.933334453	1035.271578	0.525231425
1040.869708	0.933809944	1035.869708	0.525200106
1042.570328	0.935158491	1037.570328	0.525108163
1044.078298	0.936350048	1039.078298	0.525023085
1044.645178	0.936796935	1039.645178	0.524990249
1045.271578	0.937290087	1040.271578	0.524953424
1045.869708	0.937760325	1040.869708	0.524917736
1047.570328	0.939093778	1042.570328	0.524813487
1049.078298	0.940271748	1044.078298	0.524717641
1049.645178	0.94071348	1044.645178	0.524680791
1050.271578	0.941200902	1045.271578	0.524639556
1050.869708	0.941665636	1045.869708	0.52459968
1052.570328	0.942983284	1047.570328	0.524483638
1054.078298	0.944147036	1049.078298	0.524377482
1054.645178	0.944583371	1049.645178	0.524336791
1055.271578	0.9450648	1050.271578	0.524291336
1055.869708	0.945523785	1050.869708	0.524247448
1057.570328	0.946824914	1052.570328	0.524120125
1059.078298	0.947973816	1054.078298	0.524004109
1059.645178	0.948404525	1054.645178	0.523959748
1060.271578	0.948879703	1055.271578	0.523910257
1060.869708	0.949332686	1055.869708	0.52386254
1062.570328	0.950616603	1057.570328	0.523724439
1064.078298	0.951750046	1059.078298	0.523599013
1064.645178	0.952174895	1059.645178	0.523551148
1065.271578	0.95264357	1060.271578	0.523497808
1065.869708	0.953090315	1060.869708	0.523446438
1067.570328	0.954356331	1062.570328	0.523298067
1069.078298	0.955473717	1064.078298	0.523163675
1069.645178	0.955892483	1064.645178	0.523112475
1070.271578	0.956354406	1065.271578	0.523055473
1070.869708	0.956794676	1065.869708	0.523000626
1072.570328	0.958042134	1067.570328	0.522842489
1074.078298	0.959142869	1069.078298	0.522699576
1074.645178	0.959555332	1069.645178	0.52264521
1075.271578	0.960010263	1070.271578	0.522584729
1075.869708	0.960443827	1070.869708	0.522526581
1077.570328	0.96167207	1072.570328	0.522359175
1079.078298	0.962755593	1074.078298	0.52220819
1079.645178	0.963161539	1074.645178	0.522150822
1080.271578	0.963609242	1075.271578	0.522087044

Final State: j'=1		Final State: j'=0	
E(cm⁻¹)	σ (Å²)	E(cm⁻¹)	σ (Å²)
1080.869708	0.964035879	1075.869708	0.522025773
1084.078298	0.966310028	1077.570328	0.521849595
1084.645178	0.966709252	1079.078298	0.521690971
1085.271578	0.967149497	1079.645178	0.521630767
1085.869708	0.967568987	1080.271578	0.521563876
1089.078298	0.969804367	1080.869708	0.521499661
1089.645178	0.970196903	1084.078298	0.521149372
1090.271578	0.970629232	1084.645178	0.521086493
1090.869708	0.971041365	1085.271578	0.52101667
1094.078298	0.97323686	1085.869708	0.520949668
1094.645178	0.973622038	1089.078298	0.520584822
1095.271578	0.974046713	1089.645178	0.520519378
1095.869708	0.974451286	1090.271578	0.520446855
1099.078298	0.976605817	1090.869708	0.520377242
1099.645178	0.976983689	1094.078298	0.519998741
1100.869708	0.977797083	1094.645178	0.519930998
1104.078298	0.979909622	1095.271578	0.519855842
1104.645178	0.980280008	1095.869708	0.519783787
1105.869708	0.981077159	1099.078298	0.519392525
1109.078298	0.983146714	1099.645178	0.519322585
1109.645178	0.983509439	1100.869708	0.519170693
1110.869708	0.984289985	1104.078298	0.518767548
1114.078298	0.986315607	1104.645178	0.518695569
1114.645178	0.986670512	1105.869708	0.518539328
1115.869708	0.987434097	1109.078298	0.518125171
1119.078298	0.989414887	1109.645178	0.518051302
1119.645178	0.989761815	1110.869708	0.51789104
1120.869708	0.990508106	1114.078298	0.517466721
1124.078298	0.992443208	1114.645178	0.517391115
1124.645178	0.992782007	1115.869708	0.517227152
1125.869708	0.993510686	1119.078298	0.516793509
1129.078298	0.99539928	1119.645178	0.51671631
1129.645178	0.995729809	1120.869708	0.516548963
1130.869708	0.996440569	1124.078298	0.516106816
1134.078298	0.998281865	1124.645178	0.516028167
1134.645178	0.998603985	1125.869708	0.515857747
1135.869708	0.999296529	1129.078298	0.515407892
1139.078298	1.001089751	1129.645178	0.515327935
1139.645178	1.001403326	1130.869708	0.515154742
1140.869708	1.002077358	1134.078298	0.514697963
1144.078298	1.003821714	1134.645178	0.514616834
1144.645178	1.0041266	1135.869708	0.514441168
1145.869708	1.004781804	1139.078298	0.513978215
1149.078298	1.006476422	1139.645178	0.513896046
1149.645178	1.006772452	1140.869708	0.513718174
1150.869708	1.007408454	1144.078298	0.513249799
1154.078298	1.00905219	1144.645178	0.513166718
1154.645178	1.009339132	1145.869708	0.512986924
1155.869708	1.009955377	1149.078298	0.512513819
1159.078298	1.011546272	1149.645178	0.512429947
1159.645178	1.011823664	1150.869708	0.512248491

Final State: j’=1		Final State: j’=0	
E(cm⁻¹)	σ (Å²)	E(cm⁻¹)	σ (Å²)
1160.869708	1.012418959	1154.078298	0.511771315
1164.078298	1.013951623	1154.645178	0.511686765
1165.869708	1.014787331	1155.869708	0.511503888
1169.078298	1.016211033	1159.078298	0.511023227
1170.869708	1.017054031	1159.645178	0.510938095
1174.078298	1.018404619	1160.869708	0.510753986
1175.869708	1.019404519	1164.078298	0.51027022
1179.078298	1.020745014	1165.869708	0.509999198
1180.869708	1.021569924	1169.078298	0.5095102
1184.078298	1.023063311	1170.869708	0.509181768
1185.869708	1.023619786	1174.078298	0.508728524
1189.078298	1.0250677	1175.869708	0.508456998
1190.869708	1.025662489	1179.078298	0.507970795
1194.078298	1.027098122	1180.869708	0.507716397
1195.869708	1.027613394	1184.078298	0.507246119
1199.078298	1.028861709	1185.869708	0.506949909
1200.869708	1.02944126	1189.078298	0.506492903
1204.078298	1.030240459	1190.869708	0.50620204
1205.869708	1.031357303	1194.078298	0.505751156
1209.078298	1.032236086	1195.869708	0.505459524
1210.869708	1.033113865	1199.078298	0.504941902
1214.078298	1.034325319	1200.869708	0.504720726
1215.869708	1.034212985	1204.078298	0.504141444
1219.078298	1.035475346	1205.869708	0.503885495
1220.869708	1.036349522	1209.078298	0.503458332
1224.078298	1.036841605	1210.869708	0.503192167
1225.869708	1.037436645	1214.078298	0.502747885
1229.078298	1.038221802	1215.869708	0.502376371
1230.869708	1.039419151	1219.078298	0.50196843
1234.078298	1.039851935	1220.869708	0.501815326
1235.869708	1.040335631	1224.078298	0.50117156
1239.078298	1.041228186	1225.869708	0.501000073
1240.869708	1.041616981	1229.078298	0.500400723
1244.078298	1.042394505	1230.869708	0.500298718
1245.869708	1.042795801	1234.078298	0.499732955
1249.078298	1.043551857	1235.869708	0.499506944
1250.869708	1.043948083	1239.078298	0.499072129
1254.078298	1.044638274	1240.869708	0.498791999
1255.869708	1.045015971	1244.078298	0.498330936
1259.078298	1.045663599	1245.869708	0.498063784
1260.869708	1.046014379	1249.078298	0.497633303
1264.078298	1.046623607	1250.869708	0.497383039
1265.869708	1.046952563	1254.078298	0.496938725
1269.078298	1.047521902	1255.869708	0.496695379
1270.869708	1.047829006	1259.078298	0.496256493
1275.869708	1.048646315	1260.869708	0.49601345
1280.869708	1.049406147	1264.078298	0.495581929
1285.869708	1.05011042	1265.869708	0.495342588
1290.869708	1.050761111	1269.078298	0.494916749
1295.869708	1.051360213	1270.869708	0.494680739
1300.869708	1.051909784	1275.869708	0.494028789

Final State: j’=1		Final State: j’=0	
E(cm⁻¹)	σ (Å²)	E(cm⁻¹)	σ (Å²)
1305.869708	1.052411916	1280.869708	0.493386778
1310.869708	1.052868736	1285.869708	0.492754917
1315.869708	1.053282409	1290.869708	0.492133359
1320.869708	1.053655122	1295.869708	0.491522217
1325.869708	1.05398908	1300.869708	0.490921582
1330.869708	1.05428651	1305.869708	0.490331514
1335.869708	1.054549648	1310.869708	0.489752041
1340.869708	1.054780738	1315.869708	0.489183171
1345.869708	1.054982026	1320.869708	0.488624879
1350.869708	1.05515576	1325.869708	0.488077122
1355.869708	1.055304178	1330.869708	0.487539827
1360.869708	1.055429514	1335.869708	0.487012902
1365.869708	1.055533988	1340.869708	0.48649623
1370.869708	1.0556198	1345.869708	0.485989681
1375.869708	1.055689136	1350.869708	0.485493096
1380.869708	1.055744153	1355.869708	0.485006308
1385.869708	1.055786987	1360.869708	0.484529126
1390.869708	1.055819733	1365.869708	0.484061347
1395.869708	1.055844463	1370.869708	0.483602754
1400.869708	1.055863218	1375.869708	0.483153116
1405.869708	1.055877996	1380.869708	0.482712192
		1385.869708	0.482279729
		1390.869708	0.481855465
		1395.869708	0.48143913
		1400.869708	0.481030452
		1405.869708	0.480629146

Initial rotational state: j=4

Final State: j'=5		Final State: j'=3		Final State: j'=2	
E(cm⁻¹)	σ (Å²)	E(cm⁻¹)	σ (Å²)	E(cm⁻¹)	σ (Å²)
410.9948863	0.899734354	274.2034773	397.6832522	274.2034773	98.39592658
411.0948863	1.847456036	274.3034773	774.6367226	274.3034773	179.4722718
411.1948863	1.28178792	274.4034773	318.6303324	274.4034773	76.91633416
411.2948863	1.390695753	274.5034773	277.6365305	274.5034773	65.75921829
411.3948863	1.521401465	274.6034773	256.5772698	274.6034773	59.17060566
411.4948863	1.93531087	274.6703513	257.3737719	274.6703513	56.92701496
411.5948863	2.15367134	274.7034773	275.2471392	274.7034773	58.91979823
411.6948863	2.026503278	274.8034773	294.981811	274.8034773	64.00690716
411.7948863	1.917861512	274.9034773	226.6651908	274.9034773	53.73591565
411.8948863	2.352819206	275.0034773	209.4172794	275.0034773	48.78444872
411.9948863	2.025176779	275.1034773	235.6625232	275.1034773	52.03837176
412.0948863	1.97606308	275.2034773	181.5181344	275.2034773	43.35450019
412.1948863	1.993846405	275.2967543	167.4542704	275.2967543	40.07288712
412.2948863	2.024810999	275.3034773	166.7335246	275.3034773	39.89086274
412.3948863	2.058819616	275.4034773	157.7523082	275.4034773	37.56814546
412.4948863	2.092767135	275.5034773	150.6099145	275.5034773	35.68733771
412.5955063	2.126367201	277.5955063	108.4491836	277.5955063	24.53329275
412.8789423	2.244017438	277.8789423	98.67789488	277.8789423	22.35990888
414.1034773	3.467458274	279.1034773	84.60233947	279.1034773	18.71848459
414.6703513	3.211752082	279.2034773	83.92808311	279.2034773	18.53019286
415.2967543	3.304148226	279.3034773	83.18717867	279.3034773	18.33035993
415.8948863	3.548554064	279.4034773	82.38032483	279.4034773	18.11787655
415.9948863	3.58482947	279.5034773	81.51613469	279.5034773	17.89231443
416.0948863	3.617377026	279.6034773	80.6107342	279.6034773	17.65286286
416.1948863	3.646050712	279.6703513	79.99618674	279.6703513	17.48375568
416.2948863	3.671041469	279.7034773	79.69517668	279.7034773	17.39685289
416.3948863	3.692843841	279.8034773	78.87437626	279.8034773	17.12431924
416.4948863	3.712386391	279.9034773	78.57609369	279.9034773	16.92106155
416.5948863	3.733007718	280.0034773	78.98836973	280.0034773	16.95956853
416.6948863	3.77970033	280.2967543	81.28097565	280.2967543	16.9608432
416.7948863	3.843712726	282.5955063	63.33772155	282.5955063	13.68340004
416.8948863	3.888934781	282.8789423	61.54573734	282.8789423	13.304426
416.9948863	3.968765425	284.1034773	56.4591884	284.1034773	12.13675676
417.5955063	5.815079568	284.6703513	56.31259235	284.6703513	11.94507268
417.8789423	6.199691049	285.2967543	58.54574147	285.2967543	12.09802144
419.1034773	4.339950418	287.5955063	49.4937507	287.5955063	10.34685607
419.6703513	4.333776796	287.8789423	48.75307526	287.8789423	10.18258796
420.2967543	4.379756692	289.1034773	60.02155601	289.1034773	11.11490557
420.8948863	4.474615608	289.6703513	45.29343251	289.6703513	9.396663227
422.5955063	5.313268603	290.2967543	43.50136556	290.2967543	9.097237038
422.8789423	5.311655813	292.5955063	39.72883082	292.5955063	8.32733569
424.1034773	5.131562355	292.8789423	39.28554274	292.8789423	8.239423681
424.6703513	5.138952439	294.1034773	37.56079436	294.1034773	7.89286811
425.2967543	6.41548358	294.6703513	36.90116044	294.6703513	7.753713648

Final State: j'=5		Final State: j'=3		Final State: j'=2	
E(cm⁻¹)	σ (Å²)	E(cm⁻¹)	σ (Å²)	E(cm⁻¹)	σ (Å²)
425.8948863	6.8241747	295.2967543	36.36974238	295.2967543	7.624469514
427.5955063	5.347850563	297.5955063	36.51976668	297.5955063	7.394739338
427.8789423	5.370161851	297.8789423	35.65085212	297.8789423	7.282516032
429.1034773	5.440681431	299.1034773	33.27328183	299.1034773	6.94083321
429.6703513	5.466034472	299.6703513	32.56069997	299.6703513	6.82422113
430.2967543	5.494559263	300.2967543	31.88958766	300.2967543	6.709139499
430.8948863	5.525208409	302.5955063	29.99269813	302.5955063	6.360804815
432.5955063	5.710692331	302.8789423	29.810049	302.8789423	6.324564189
432.8789423	5.804138143	304.1034773	29.15737385	304.1034773	6.184829438
434.1034773	6.167367343	304.6703513	28.90925718	304.6703513	6.127470073
434.6703513	6.030456508	305.2967543	28.63046354	305.2967543	6.065478182
435.2967543	5.944966959	307.5955063	27.35064222	307.5955063	5.829111434
435.8948863	5.914892329	307.8789423	27.18550922	307.8789423	5.800606939
437.5955063	5.922342754	309.1034773	26.50494057	309.1034773	5.683156263
437.8789423	5.929081419	309.6703513	26.21137493	309.6703513	5.632118851
439.1034773	5.968651238	310.2967543	25.90336812	310.2967543	5.57814855
439.6703513	5.993349872	312.5955063	24.90980511	312.5955063	5.400191211
440.2967543	6.026699414	312.8789423	24.80026365	312.8789423	5.380209551
440.8948863	6.064639843	314.1034773	24.35111066	314.1034773	5.297956004
442.5955063	6.172370431	314.6703513	24.15358253	314.6703513	5.261836972
442.8789423	6.185286517	315.2967543	23.94092118	315.2967543	5.223155108
444.1034773	6.225910994	317.5955063	23.19899533	317.5955063	5.090657217
444.6703513	6.240649377	317.8789423	23.11144574	317.8789423	5.075265851
445.2967543	6.256417553	319.1034773	22.74384196	319.1034773	5.011046729
445.8948863	6.271776683	319.6703513	22.57977915	319.6703513	4.982547454
447.5955063	6.319710041	320.2967543	22.40313278	320.2967543	4.951938826
447.8789423	6.328435586	322.5955063	21.79645632	322.5955063	4.847179461
449.1034773	6.36856309	322.8789423	21.72600611	322.8789423	4.835040524
449.6703513	6.388307648	324.1034773	21.43172944	324.1034773	4.784402924
450.2967543	6.410722937	324.6703513	21.30072428	324.6703513	4.761905209
450.8948863	6.432450815	325.2967543	21.15952603	325.2967543	4.737697609
452.5955063	6.493793595	327.5955063	20.67016771	327.5955063	4.654223015
452.8789423	6.50376312	327.8789423	20.6126889	327.8789423	4.644467408
454.1034773	6.545737927	329.1034773	20.37092736	329.1034773	4.603550016
454.6703513	6.564614353	329.6703513	20.26246776	329.6703513	4.585251032
455.2967543	6.585147793	330.2967543	20.14505026	330.2967543	4.565476403
455.8948863	6.604508357	332.5955063	19.73474656	332.5955063	4.496589486
457.5955063	6.658808104	332.8789423	19.68627497	332.8789423	4.488464068
457.8789423	6.667807765	334.1034773	19.48184341	334.1034773	4.454191848
459.1034773	6.706669093	334.6703513	19.38983172	334.6703513	4.438753917
459.6703513	6.724670148	335.2967543	19.28998917	335.2967543	4.421982766
460.2967543	6.744573089	337.5955063	18.93868913	337.5955063	4.362649714
460.8948863	6.763581517	337.8789423	18.8968718	337.8789423	4.355533216
462.5955063	6.817529623	339.1034773	18.71951957	339.1034773	4.325146644
462.8789423	6.826489969	339.6703513	18.63908463	339.6703513	4.311225559

Final State: j’=5		Final State: j’=3		Final State: j’=2	
E(cm⁻¹)	σ (Å²)	E(cm⁻¹)	σ (Å²)	E(cm⁻¹)	σ (Å²)
464.1034773	6.865037825	340.2967543	18.5512846	340.2967543	4.295898799
464.6703513	6.882776858	342.5955063	18.23644157	342.5955063	4.239247208
465.2967543	6.90229159	342.8789423	18.19811875	342.8789423	4.23209409
465.8948863	6.920837264	344.1034773	18.03246666	344.1034773	4.200194669
467.5955063	6.973095595	344.6703513	17.95509216	344.6703513	4.184599426
467.8789423	6.981739972	345.2967543	17.86827734	345.2967543	4.166403179
469.1034773	7.018886617	347.5955063	17.51004646	347.5955063	4.079160085
469.6703513	7.035978853	347.8789423	17.4541319	347.8789423	4.063207324
470.2967543	7.054794037	349.1034773	17.06041703	349.1034773	3.948671155
470.8948863	7.072691794	349.6703513	16.92567629	349.6703513	3.975736063
472.5955063	7.123230032	350.2967543	17.16117538	350.2967543	4.138881949
472.8789423	7.131603797	352.5955063	17.19240515	352.5955063	4.162267943
474.1034773	7.167620182	352.8789423	17.11456031	352.8789423	4.084381373
474.6703513	7.18420406	354.1034773	16.79504585	354.1034773	4.094594315
475.2967543	7.202462994	354.6703513	16.89486227	354.6703513	4.182950626
475.8948863	7.21983177	355.2967543	16.97029194	355.2967543	4.116892176
477.5955063	7.26885561	357.5955063	16.62244405	357.5955063	4.186622588
477.8789423	7.27697369	357.8789423	16.80679208	357.8789423	4.264015192
479.1034773	7.311871434	359.1034773	16.91685824	359.1034773	4.241126957
479.6703513	7.327929816	359.6703513	16.75594715	359.6703513	4.118420318
480.2967543	7.345603015	360.2967543	16.50871694	360.2967543	4.018955683
480.8948863	7.362408194	362.5955063	16.27567336	362.5955063	4.042664226
482.5955063	7.409812598	362.8789423	16.58678983	362.8789423	4.244184183
482.8789423	7.417659018	364.1034773	16.47521171	364.1034773	4.089287968
484.1034773	7.451378953	364.6703513	16.31406523	364.6703513	4.026673747
484.6703513	7.466890051	365.2967543	16.04700289	365.2967543	3.935145648
485.2967543	7.483957116	367.5955063	16.33576651	367.5955063	4.238947983
485.8948863	7.500181869	367.8789423	16.5613768	367.8789423	4.253996124
487.5955063	7.54592515	369.1034773	16.20242706	369.1034773	4.069090681
487.8789423	7.553492719	369.6703513	16.08144593	369.6703513	4.023117594
489.1034773	7.58599883	370.2967543	15.91864337	370.2967543	3.964404169
489.6703513	7.600942092	372.5955063	15.63074218	372.5955063	3.980803262
490.2967543	7.617376315	372.8789423	16.02717304	372.8789423	4.210302605
490.8948863	7.632990939	374.1034773	16.01929197	374.1034773	4.084504619
492.5955063	7.676961267	374.6703513	15.90955225	374.6703513	4.040033641
492.8789423	7.684227017	375.2967543	15.8015531	375.2967543	3.999694488
494.1034773	7.715404665	377.5955063	15.00161872	377.5955063	3.8647607
494.6703513	7.729717958	377.8789423	15.31298731	377.8789423	4.069858042
495.2967543	7.74544331	379.1034773	15.71229529	379.1034773	4.151712412
495.8948863	7.760367499	379.6703513	15.79822264	379.6703513	4.083724529
497.5955063	7.802288192	380.2967543	15.61842988	380.2967543	3.999869652
497.8789423	7.809197765	382.5955063	15.03944695	382.5955063	3.774681676
499.1034773	7.838778521	382.8789423	14.91302459	382.8789423	3.749654564
499.6703513	7.852315815	384.1034773	14.6560761	384.1034773	3.75675544
500.2967543	7.867152104	384.6703513	14.68316843	384.6703513	4.001693084

Final State: j'=5		Final State: j'=3		Final State: j'=2	
E(cm⁻¹)	σ (Å²)	E(cm⁻¹)	σ (Å²)	E(cm⁻¹)	σ (Å²)
500.8948863	7.881192012	385.2967543	15.08242933	385.2967543	3.948805552
502.5955063	7.920363439	387.5955063	15.39441384	387.5955063	4.07693817
502.8789423	7.926773349	387.8789423	15.27817245	387.8789423	4.007888184
504.1034773	7.954016743	389.1034773	14.81880527	389.1034773	3.963509026
504.6703513	7.966352937	389.6703513	15.30655487	389.6703513	4.137763028
505.2967543	7.979751155	390.2967543	15.15569629	390.2967543	3.997453485
505.8948863	7.992286961	392.5955063	15.35127798	392.5955063	4.285458609
507.5955063	8.026187064	392.8789423	15.1482778	392.8789423	4.207458229
507.8789423	8.031519736	394.1034773	15.17401546	394.1034773	4.03592207
509.1034773	8.053067467	394.6703513	14.88004787	394.6703513	3.909170808
509.6703513	8.061944588	395.2967543	14.30616996	395.2967543	3.858178517
510.2967543	8.070556467	397.5955063	14.91706762	397.5955063	3.967496855
510.8948863	8.077080938	397.8789423	14.83390393	397.8789423	3.930132477
512.5955063	8.071716389	399.1034773	13.89128123	399.1034773	3.683536931
512.8789423	8.06019055	399.6703513	14.30249782	399.6703513	4.021726981
514.1034773	8.000613816	400.2967543	15.02168399	400.2967543	4.209945854
514.6703513	8.142906356	402.5955063	14.44904915	402.5955063	3.883362325
515.2967543	8.100380093	402.8789423	14.45576252	402.8789423	3.889050838
515.8948863	8.093995279	404.1034773	14.70333847	404.1034773	4.226158369
517.5955063	8.120505255	404.6703513	15.00094658	404.6703513	4.142705267
517.8789423	8.093188507	405.2967543	14.274175	405.2967543	3.858215683
519.1034773	8.308361187	407.5955063	14.10272297	407.5955063	3.885358146
519.6703513	8.241604103	407.8789423	14.04723025	407.8789423	3.807666136
520.2967543	8.333777342	409.1034773	14.61760451	409.1034773	4.149060599
520.8948863	8.23787249	409.6703513	14.67229902	409.6703513	4.174519726
522.5955063	8.356233737	410.2967543	14.94123533	410.2967543	4.203766349
522.8789423	8.317913973	410.9948863	14.36502874	410.9948863	3.932041086
524.1034773	8.423458676	411.0948863	14.03078654	411.0948863	3.86472931
524.6703513	8.36413862	411.1948863	14.20659985	411.1948863	3.891208302
525.2967543	8.227586916	411.2948863	14.08745713	411.2948863	3.843118462
525.8948863	8.429997935	411.3948863	13.90927911	411.3948863	3.782432182
527.5955063	8.584709907	411.4948863	13.58106425	411.4948863	3.750799391
527.8789423	8.566422693	411.5948863	13.73749183	411.5948863	3.888573129
529.1034773	8.500290062	411.6948863	14.16745512	411.6948863	4.021591776
529.6703513	8.406472388	411.7948863	14.04113295	411.7948863	3.91285626
530.2967543	8.393121358	411.8948863	13.80316854	411.8948863	3.917702471
530.8948863	8.611530915	411.9948863	14.24036706	411.9948863	4.031535505
532.5955063	8.692407594	412.0948863	14.19051606	412.0948863	3.974361259
532.8789423	8.680252581	412.1948863	14.1269549	412.1948863	3.936655019
534.1034773	8.639795021	412.2948863	14.06776986	412.2948863	3.9078501
534.6703513	8.603208183	412.3948863	14.00997229	412.3948863	3.882621426
535.2967543	8.46397384	412.4948863	13.94947911	412.4948863	3.858040839
535.8948863	8.539832291	412.5955063	13.88065709	412.5955063	3.831661689
537.5955063	8.851359925	412.8789423	13.53456655	412.8789423	3.724460407
537.8789423	8.823736369	414.1034773	13.92636595	414.1034773	3.964133105

Final State: j'=5		Final State: j'=3		Final State: j'=2	
E(cm⁻¹)	σ (Å²)	E(cm⁻¹)	σ (Å²)	E(cm⁻¹)	σ (Å²)
539.1034773	8.781938548	414.6703513	13.87088363	414.6703513	3.922125412
539.6703513	8.769425045	415.2967543	13.75162097	415.2967543	3.879297741
540.2967543	8.749853418	415.8948863	13.60814677	415.8948863	3.839992291
540.8948863	8.71021	415.9948863	13.58126985	415.9948863	3.832671876
542.5955063	8.768966949	416.0948863	13.55206812	416.0948863	3.824565727
542.8789423	8.745108064	416.1948863	13.51893671	416.1948863	3.815231132
544.1034773	8.915009436	416.2948863	13.47908651	416.2948863	3.804015754
544.6703513	8.887226944	416.3948863	13.42723213	416.3948863	3.789953778
545.2967543	8.864903969	416.4948863	13.35206749	416.4948863	3.771936916
545.8948863	8.84044265	416.5948863	13.2301703	416.5948863	3.753667665
547.5955063	8.6713309	416.6948863	13.15108918	416.6948863	3.796952946
547.8789423	8.705331097	416.7948863	13.21751142	416.7948863	3.868096679
549.1034773	8.84013799	416.8948863	13.42343728	416.8948863	3.958798972
549.6703513	8.88850663	416.9948863	13.37543181	416.9948863	3.869538418
550.2967543	9.00044802	417.5955063	13.39541241	417.5955063	3.958233669
550.8948863	8.874235373	417.8789423	13.46255459	417.8789423	3.968267326
552.5955063	8.921601611	419.1034773	13.62630871	419.1034773	3.927298723
552.8789423	8.791905423	419.6703513	13.5750846	419.6703513	3.910490202
554.1034773	9.117932853	420.2967543	13.5228034	420.2967543	3.897469946
554.6703513	9.046745628	420.8948863	13.46835625	420.8948863	3.886181398
555.2967543	8.92099526	422.5955063	13.28024434	422.5955063	3.871990059
555.8948863	8.492103523	422.8789423	13.28242588	422.8789423	3.87623422
557.5955063	9.349676282	424.1034773	13.27229729	424.1034773	3.877076124
557.8789423	9.265337869	424.6703513	13.2253136	424.6703513	3.865913382
559.1034773	9.007810286	425.2967543	12.91431199	425.2967543	3.858811961
559.6703513	8.911866591	425.8948863	13.16720623	425.8948863	3.935434848
560.2967543	9.353963321	427.5955063	13.2268885	427.5955063	3.904796069
560.8948863	9.280581657	427.8789423	13.213828	427.8789423	3.903717653
562.5955063	9.114074049	429.1034773	13.16962748	429.1034773	3.902597183
562.8789423	9.042950175	429.6703513	13.15186006	429.6703513	3.90275473
564.1034773	9.244251498	430.2967543	13.13240414	430.2967543	3.902996849
564.6703513	9.253940348	430.8948863	13.1132912	430.8948863	3.903158433
565.2967543	9.259144423	432.5955063	13.04219748	432.5955063	3.901442536
565.8948863	9.212265588	432.8789423	13.02226091	432.8789423	3.900747368
567.5955063	8.935313088	434.1034773	12.98510991	434.1034773	3.913389917
567.8789423	8.845967053	434.6703513	12.99751612	434.6703513	3.917970378
569.1034773	9.381873477	435.2967543	12.9949123	435.2967543	3.919840471
569.6703513	9.219685294	435.8948863	12.98534446	435.8948863	3.921056472
570.2967543	9.42694583	437.5955063	12.94894539	437.5955063	3.924691365
570.8948863	9.309427148	437.8789423	12.94245432	437.8789423	3.92534413
572.5955063	8.97565585	439.1034773	12.91362662	439.1034773	3.928243125
572.8789423	9.339956709	439.6703513	12.89977204	439.6703513	3.929620852
574.1034773	9.590307408	440.2967543	12.88403716	440.2967543	3.931199502
574.6703513	9.404923887	440.8948863	12.86876973	440.8948863	3.932829541
575.2967543	9.304638011	442.5955063	12.82873845	442.5955063	3.938597106

Final State: j'=5		Final State: j'=3		Final State: j'=2	
E(cm⁻¹)	σ (Å²)	E(cm⁻¹)	σ (Å²)	E(cm⁻¹)	σ (Å²)
575.8948863	9.269200625	442.8789423	12.82291699	442.8789423	3.939670529
577.5955063	9.075690649	444.1034773	12.79921183	444.1034773	3.944257251
577.8789423	9.284968401	444.6703513	12.78847188	444.6703513	3.94630815
579.1034773	9.21127754	445.2967543	12.77654832	445.2967543	3.948528209
579.6703513	9.171062696	445.8948863	12.76507069	445.8948863	3.950617524
580.2967543	9.112813403	447.5955063	12.73198184	447.5955063	3.956496477
580.8948863	8.978885194	447.8789423	12.72641747	447.8789423	3.957476998
582.5955063	9.221603082	449.1034773	12.70230183	449.1034773	3.96174571
582.8789423	9.227676331	449.6703513	12.69113772	449.6703513	3.963748758
584.1034773	9.20789674	450.2967543	12.6788427	450.2967543	3.965988031
584.6703513	9.199713388	450.8948863	12.66717388	450.8948863	3.968153894
585.2967543	9.185826091	452.5955063	12.63456476	452.5955063	3.974455693
585.8948863	9.161660856	452.8789423	12.62922175	452.8789423	3.975524104
587.5955063	9.167766122	454.1034773	12.60642479	454.1034773	3.980186227
587.8789423	9.172878132	454.6703513	12.59601306	454.6703513	3.982365985
589.1034773	9.145677251	455.2967543	12.5845976	455.2967543	3.984788172
589.6703513	9.089741023	455.8948863	12.57377569	455.8948863	3.987113349
590.2967543	9.215557093	457.5955063	12.54336309	457.5955063	3.993787092
590.8948863	9.239797304	457.8789423	12.53834041	457.8789423	3.99490848
592.5955063	9.234788445	459.1034773	12.5167815	459.1034773	3.999784497
592.8789423	9.236375205	459.6703513	12.50687788	459.6703513	4.002059527
594.1034773	9.244734577	460.2967543	12.49599121	460.2967543	4.004587066
594.6703513	9.248594566	460.8948863	12.48565282	460.8948863	4.007014128
595.2967543	9.252369865	462.5955063	12.45657269	462.5955063	4.013988625
595.8948863	9.254946255	462.8789423	12.45177241	462.8789423	4.01516169
597.5955063	9.242540936	464.1034773	12.4311889	464.1034773	4.020264342
597.8789423	9.245876062	464.6703513	12.42174574	464.6703513	4.022645298
599.1034773	9.28153734	465.2967543	12.41137361	465.2967543	4.025289845
599.6703513	9.290188027	465.8948863	12.40153052	465.8948863	4.027828162
600.2967543	9.297497456	467.5955063	12.3738628	467.5955063	4.03511358
600.8948863	9.303605065	467.8789423	12.36929639	467.8789423	4.036337476
602.5955063	9.319200498	469.1034773	12.34971138	469.1034773	4.041656311
602.8789423	9.321628052	469.6703513	12.34072242	469.6703513	4.04413555
604.1034773	9.331493528	470.2967543	12.33084553	470.2967543	4.046887548
604.6703513	9.335712498	470.8948863	12.32146872	470.8948863	4.049527461
605.2967543	9.340269747	472.5955063	12.29509351	472.5955063	4.057097537
605.8948863	9.344805206	472.8789423	12.29073818	472.8789423	4.058368428
607.5955063	9.360014625	474.1034773	12.27205299	474.1034773	4.063889069
607.8789423	9.362754267	474.6703513	12.26347477	474.6703513	4.066461217
609.1034773	9.374536695	475.2967543	12.25404803	475.2967543	4.069315582
609.6703513	9.379867877	475.8948863	12.24509776	475.8948863	4.072052924
610.2967543	9.385647686	477.5955063	12.21992006	477.5955063	4.079898749
610.8948863	9.391060604	477.8789423	12.2157623	477.8789423	4.081215402
612.5955063	9.405949597	479.1034773	12.19792456	479.1034773	4.086933293
612.8789423	9.408368883	479.6703513	12.18973532	479.6703513	4.08959648

Final State: j’=5		Final State: j’=3		Final State: j’=2	
E(cm⁻¹)	σ (Å²)	E(cm⁻¹)	σ (Å²)	E(cm⁻¹)	σ (Å²)
614.1034773	9.418669998	480.2967543	12.18073601	480.2967543	4.092551241
614.6703513	9.423378953	480.8948863	12.17219151	480.8948863	4.095384337
615.2967543	9.428557855	482.5955063	12.14815527	482.5955063	4.103501897
615.8948863	9.433489836	482.8789423	12.14418604	482.8789423	4.10486383
617.5955063	9.447482509	484.1034773	12.12715755	484.1034773	4.110777461
617.8789423	9.449809901	484.6703513	12.1193402	484.6703513	4.113531453
619.1034773	9.459833573	485.2967543	12.11074998	485.2967543	4.116586784
619.6703513	9.464449563	485.8948863	12.10259448	485.8948863	4.119516197
620.2967543	9.469527236	487.5955063	12.07965662	487.5955063	4.127909635
620.8948863	9.474350218	487.8789423	12.07586954	487.8789423	4.129317933
622.5955063	9.487911457	489.1034773	12.05962615	489.1034773	4.135433589
622.8789423	9.4901489	489.6703513	12.05217152	489.6703513	4.138282201
624.1034773	9.499740759	490.2967543	12.04398198	490.2967543	4.141443037
624.6703513	9.504141028	490.8948863	12.03620912	490.8948863	4.144474219
625.2967543	9.508975008	492.5955063	12.01436221	492.5955063	4.153163833
625.8948863	9.513563989	492.8789423	12.01075777	492.8789423	4.154622631
627.5955063	9.526475803	494.1034773	11.99530748	494.1034773	4.160960996
627.8789423	9.528609076	494.6703513	11.98822298	494.6703513	4.163915529
629.1034773	9.5377662	495.2967543	11.98044529	495.2967543	4.167195827
629.6703513	9.541973153	495.8948863	11.97306904	495.8948863	4.170343704
630.2967543	9.546598394	497.5955063	11.95237334	497.5955063	4.179382008
630.8948863	9.550991878	497.8789423	11.94896496	497.8789423	4.180901774
632.5955063	9.563360516	499.1034773	11.93438001	499.1034773	4.187515005
632.8789423	9.565404179	499.6703513	11.92770795	499.6703513	4.190604027
634.1034773	9.574174848	500.2967543	11.92039652	500.2967543	4.194039187
634.6703513	9.578202764	500.8948863	11.91347757	500.8948863	4.197341954
635.2967543	9.582629914	502.5955063	11.8941646	502.5955063	4.206867103
635.8948863	9.586834053	502.8789423	11.89100157	502.8789423	4.208476163
637.5955063	9.598664137	504.1034773	11.87754166	504.1034773	4.215510226
637.8789423	9.600618205	504.6703513	11.87143422	504.6703513	4.21881718
639.1034773	9.609003204	505.2967543	11.86478786	505.2967543	4.222514553
639.6703513	9.612853587	505.8948863	11.8585527	505.8948863	4.226092727
640.2967543	9.61708548	507.5955063	11.8415556	507.5955063	4.236588
640.8948863	9.621104168	507.8789423	11.8388528	507.8789423	4.238395496
642.5955063	9.63241264	509.1034773	11.82776888	509.1034773	4.24647384
642.8789423	9.63428062	509.6703513	11.82306551	509.6703513	4.250407912
644.1034773	9.642296282	510.2967543	11.81832332	510.2967543	4.254959943
644.6703513	9.645977134	510.8948863	11.81442646	510.8948863	4.25958486
645.2967543	9.65002269	512.5955063	11.81157794	512.5955063	4.275892459
645.8948863	9.653864366	512.8789423	11.81436726	512.8789423	4.279502301
647.5955063	9.664674285	514.1034773	11.80112258	514.1034773	4.258269384
647.8789423	9.666459812	514.6703513	11.75334009	514.6703513	4.248971384
649.1034773	9.674121373	515.2967543	11.77350396	515.2967543	4.274041672
649.6703513	9.677639429	515.8948863	11.75040162	515.8948863	4.24928275
650.2967543	9.681505938	517.5955063	11.76790754	517.5955063	4.293836626

Final State: j’=5		Final State: j’=3		Final State: j’=2	
E(cm⁻¹)	σ (Å²)	E(cm⁻¹)	σ (Å²)	E(cm⁻¹)	σ (Å²)
650.8948863	9.685177421	517.8789423	11.75781507	517.8789423	4.280393075
652.5955063	9.695507904	519.1034773	11.68821887	519.1034773	4.265623886
652.8789423	9.697214173	519.6703513	11.71222281	519.6703513	4.287935116
654.1034773	9.704535469	520.2967543	11.68352677	520.2967543	4.286856812
654.6703513	9.707897221	520.8948863	11.71410896	520.8948863	4.306934675
655.2967543	9.711591906	522.5955063	11.67401866	522.5955063	4.306286784
655.8948863	9.715100253	522.8789423	11.65367817	522.8789423	4.266046259
657.5955063	9.7249717	524.1034773	11.64702432	524.1034773	4.314756585
657.8789423	9.726602168	524.6703513	11.66537336	524.6703513	4.32775167
659.1034773	9.733598246	525.2967543	11.67605728	525.2967543	4.308161377
659.6703513	9.736810732	525.8948863	11.60224359	525.8948863	4.291182141
660.2967543	9.740341431	527.5955063	11.57767079	527.5955063	4.316907873
660.8948863	9.74369411	527.8789423	11.58572951	527.8789423	4.325274138
662.5955063	9.753128073	529.1034773	11.60803447	529.1034773	4.349352057
662.8789423	9.754686337	529.6703513	11.63207372	529.6703513	4.359742239
664.1034773	9.761372998	530.2967543	11.5826883	530.2967543	4.305308283
664.6703513	9.764443591	530.8948863	11.54995374	530.8948863	4.333014332
665.2967543	9.767818488	532.5955063	11.52983009	532.5955063	4.348519034
665.8948863	9.771023425	532.8789423	11.5345961	532.8789423	4.354461747
667.5955063	9.780042708	534.1034773	11.54766965	534.1034773	4.374121956
667.8789423	9.781532693	534.6703513	11.55812576	534.6703513	4.383719556
669.1034773	9.787927029	535.2967543	11.58912624	535.2967543	4.391295523
669.6703513	9.790863818	535.8948863	11.51252145	535.8948863	4.337299285
670.2967543	9.794092006	537.5955063	11.45941129	537.5955063	4.365818092
670.8948863	9.797158014	537.8789423	11.47177063	537.8789423	4.376725218
672.5955063	9.805788675	539.1034773	11.48688436	539.1034773	4.398359132
672.8789423	9.807214818	539.6703513	11.49045731	539.6703513	4.406036566
674.1034773	9.813336614	540.2967543	11.49591664	540.2967543	4.414804599
674.6703513	9.81614909	540.8948863	11.50690756	540.8948863	4.424606627
675.2967543	9.819241322	542.5955063	11.46125492	542.5955063	4.404259764
675.8948863	9.822178952	542.8789423	11.44220151	542.8789423	4.375614937
677.5955063	9.830452584	544.1034773	11.43032052	544.1034773	4.424182855
677.8789423	9.831820414	544.6703513	11.44044375	544.6703513	4.435560501
679.1034773	9.837694594	545.2967543	11.44805167	545.2967543	4.445953989
679.6703513	9.840394821	545.8948863	11.45623542	545.8948863	4.4559655
680.2967543	9.843364924	547.5955063	11.48301985	547.5955063	4.452821026
680.8948863	9.84618786	547.8789423	11.46277066	547.8789423	4.444956875
682.5955063	9.854146415	549.1034773	11.37501656	549.1034773	4.400189163
682.8789423	9.85546347	549.6703513	11.35905926	549.6703513	4.391791092
684.1034773	9.861124376	550.2967543	11.36840971	550.2967543	4.441021818
684.6703513	9.863729537	550.8948863	11.38706282	550.8948863	4.423735049
685.2967543	9.866597482	552.5955063	11.40170931	552.5955063	4.486598629
685.8948863	9.869325863	552.8789423	11.42896029	552.8789423	4.484431577
687.5955063	9.877033727	554.1034773	11.31316065	554.1034773	4.449923728
687.8789423	9.878311881	554.6703513	11.34921434	554.6703513	4.483308503

Final State: j’=5		Final State: j’=3		Final State: j’=2	
E(cm⁻¹)	σ (Å²)	E(cm⁻¹)	σ (Å²)	E(cm⁻¹)	σ (Å²)
689.1034773	9.883815595	555.2967543	11.39354485	555.2967543	4.510289054
689.6703513	9.886354609	555.8948863	11.46612301	555.8948863	4.492935992
690.2967543	9.889154943	557.5955063	11.23364333	557.5955063	4.458523687
690.8948863	9.891824703	557.8789423	11.27047052	557.8789423	4.48289146
692.5955063	9.89940351	559.1034773	11.35800272	559.1034773	4.531248421
692.8789423	9.900666493	559.6703513	11.32658805	559.6703513	4.463071854
694.1034773	9.90613049	560.2967543	11.20119609	560.2967543	4.448974358
694.6703513	9.908667655	560.8948863	11.25168555	560.8948863	4.500423571
695.2967543	9.911480502	562.5955063	11.31568813	562.5955063	4.552558846
695.8948863	9.914179246	562.8789423	11.33752338	562.8789423	4.562802014
697.5955063	9.921958243	564.1034773	11.17965749	564.1034773	4.452386581
697.8789423	9.92327668	564.6703513	11.24009473	564.6703513	4.523693644
699.1034773	9.929084718	565.2967543	11.2340724	565.2967543	4.525993396
699.6703513	9.931856686	565.8948863	11.2603849	565.8948863	4.538545147
700.2967543	9.935007526	567.5955063	11.33533259	567.5955063	4.581965921
700.8948863	9.938131987	567.8789423	11.30878449	567.8789423	4.521148733
702.5955063	9.948149662	569.1034773	11.20373068	569.1034773	4.560666907
702.8789423	9.950117	569.6703513	11.21526388	569.6703513	4.518520143
704.1034773	9.961472296	570.2967543	11.17270609	570.2967543	4.547869234
704.6703513	9.971989343	570.8948863	11.22695279	570.8948863	4.59099652
705.2967543	9.983710425	572.5955063	11.19519872	572.5955063	4.500310193
705.8948863	9.970875826	572.8789423	11.13403406	572.8789423	4.516630907
707.5955063	9.963029505	574.1034773	11.10932513	574.1034773	4.554280017
707.8789423	9.953988017	574.6703513	11.17340417	574.6703513	4.589950144
709.1034773	9.990276453	575.2967543	11.17030724	575.2967543	4.595439527
709.6703513	9.988868845	575.8948863	11.13658003	575.8948863	4.562728962
710.2967543	9.964067309	577.5955063	11.11154934	577.5955063	4.533855616
710.8948863	9.965407235	577.8789423	11.07978176	577.8789423	4.553704893
712.5955063	10.00108424	579.1034773	11.12206737	579.1034773	4.593236432
712.8789423	10.00747477	579.6703513	11.12855747	579.6703513	4.600159273
714.1034773	9.98344782	580.2967543	11.13619571	580.2967543	4.606322774
714.6703513	9.967494878	580.8948863	11.1384642	580.8948863	4.595410816
715.2967543	9.975669093	582.5955063	11.06237535	582.5955063	4.589680404
715.8948863	9.989318666	582.8789423	11.0687724	582.8789423	4.597227136
717.5955063	9.978358101	584.1034773	11.0733736	584.1034773	4.610679425
717.8789423	9.989023903	584.6703513	11.07213553	584.6703513	4.614737353
719.1034773	9.97661462	585.2967543	11.07023146	585.2967543	4.618571477
719.6703513	9.987846015	585.8948863	11.06706104	585.8948863	4.620736766
720.2967543	9.998244723	587.5955063	11.04750372	587.5955063	4.625150832
720.8948863	10.01231616	587.8789423	11.04581421	587.8789423	4.627060307
722.5955063	10.00685414	589.1034773	11.04563404	589.1034773	4.636044472
722.8789423	10.00118464	589.6703513	11.01981355	589.6703513	4.625129607
724.1034773	9.992635723	590.2967543	11.00489957	590.2967543	4.629412909
724.6703513	10.00120011	590.8948863	11.0122485	590.8948863	4.639550803
725.2967543	10.00933883	592.5955063	11.00228221	592.5955063	4.648893041

Final State: j'=5		Final State: j'=3		Final State: j'=2	
E(cm⁻¹)	σ (Å²)	E(cm⁻¹)	σ (Å²)	E(cm⁻¹)	σ (Å²)
725.8948863	10.0183568	592.8789423	11.00011971	592.8789423	4.650306578
727.5955063	10.00570196	594.1034773	10.99064255	594.1034773	4.656417342
727.8789423	10.01388816	594.6703513	10.98625248	594.6703513	4.659240129
729.1034773	9.995709043	595.2967543	10.98142769	595.2967543	4.662343604
729.6703513	10.00930806	595.8948863	10.97686792	595.8948863	4.665275619
730.2967543	10.01757087	597.5955063	10.96147156	597.5955063	4.671526724
730.8948863	10.02418223	597.8789423	10.95756074	597.8789423	4.672191889
732.5955063	10.05161827	599.1034773	10.94655315	599.1034773	4.678730897
732.8789423	10.06545546	599.6703513	10.9424096	599.6703513	4.681841477
734.1034773	10.04332168	600.2967543	10.93767641	600.2967543	4.685102319
734.6703513	10.02581312	600.8948863	10.9330498	600.8948863	4.688122693
735.2967543	10.02091814	602.5955063	10.9196391	602.5955063	4.696488307
735.8948863	10.0343216	602.8789423	10.91738585	602.8789423	4.697863273
737.5955063	10.05939889	604.1034773	10.9075973	604.1034773	4.703741984
737.8789423	10.06516496	604.6703513	10.90301455	604.6703513	4.706419993
739.1034773	10.09676523	605.2967543	10.89788781	605.2967543	4.709342671
739.6703513	10.08827667	605.8948863	10.8929323	605.8948863	4.712110001
740.2967543	10.10554912	607.5955063	10.87884924	607.5955063	4.720050493
740.8948863	10.06973985	607.8789423	10.8765351	607.8789423	4.721393079
742.5955063	10.06256924	609.1034773	10.86663639	609.1034773	4.727222907
742.8789423	10.03765149	609.6703513	10.86209071	609.6703513	4.729926588
744.1034773	10.07915666	610.2967543	10.85708402	610.2967543	4.732912282
744.6703513	10.10431925	610.8948863	10.85231426	610.8948863	4.735759451
745.2967543	10.03977709	612.5955063	10.83878681	612.5955063	4.743826512
745.8948863	10.05324941	612.8789423	10.83653497	612.8789423	4.745166469
747.5955063	10.14863538	614.1034773	10.82680987	614.1034773	4.750940321
747.8789423	10.08045395	614.6703513	10.82230952	614.6703513	4.753605594
749.1034773	10.02036145	615.2967543	10.81733861	615.2967543	4.756546173
749.6703513	10.05390801	615.8948863	10.81259526	615.8948863	4.759350411
750.2967543	10.07907822	617.5955063	10.79913776	617.5955063	4.76731048
750.8948863	10.13015836	617.8789423	10.7969002	617.8789423	4.76863574
752.5955063	10.05889198	619.1034773	10.78725308	619.1034773	4.774357191
752.8789423	10.06609386	619.6703513	10.78279824	619.6703513	4.777003376
754.1034773	10.09993229	620.2967543	10.77788357	620.2967543	4.779925399
754.6703513	10.14691901	620.8948863	10.77319833	620.8948863	4.782713379
755.2967543	10.12012665	622.5955063	10.75991496	622.5955063	4.790626966
755.8948863	10.04383809	622.8789423	10.75770621	622.8789423	4.791943858
757.5955063	10.09265298	624.1034773	10.7481796	624.1034773	4.797626133
757.8789423	10.09213232	624.6703513	10.7437779	624.6703513	4.800252637
759.1034773	10.15830368	625.2967543	10.73892008	625.2967543	4.803152026
759.6703513	10.0826992	625.8948863	10.73428741	625.8948863	4.80591766
760.2967543	10.05427236	627.5955063	10.72114724	627.5955063	4.813765838
760.8948863	10.12086971	627.8789423	10.71896179	627.8789423	4.815071712
762.5955063	10.12137695	629.1034773	10.70953498	629.1034773	4.820706608
762.8789423	10.16318893	629.6703513	10.70517937	629.6703513	4.823311394

Final State: j'=5		Final State: j'=3		Final State: j'=2	
E(cm⁻¹)	σ (Å²)	E(cm⁻¹)	σ (Å²)	E(cm⁻¹)	σ (Å²)
764.1034773	10.12609296	630.2967543	10.70037255	630.2967543	4.826186964
764.6703513	10.06842453	630.8948863	10.6957887	630.8948863	4.828930062
765.2967543	10.07444677	632.5955063	10.68278805	632.5955063	4.83671489
765.8948863	10.07101482	632.8789423	10.68062588	632.8789423	4.838010281
767.5955063	10.09342071	634.1034773	10.67129967	634.1034773	4.843599948
767.8789423	10.1012443	634.6703513	10.66699051	634.6703513	4.846183806
769.1034773	10.10425505	635.2967543	10.66223483	635.2967543	4.849036187
769.6703513	10.09107212	635.8948863	10.65769962	635.8948863	4.851757098
770.2967543	10.10207954	637.5955063	10.64483598	637.5955063	4.859478567
770.8948863	10.11086798	637.8789423	10.64269646	637.8789423	4.860763355
772.5955063	10.12957693	639.1034773	10.63346748	639.1034773	4.86630709
772.8789423	10.13107848	639.6703513	10.629203	639.6703513	4.868869609
774.1034773	10.09728188	640.2967543	10.62449645	640.2967543	4.871698413
774.6703513	10.10544485	640.8948863	10.62000798	640.8948863	4.874396787
775.2967543	10.11032987	642.5955063	10.60727626	642.5955063	4.882054088
775.8948863	10.11367144	642.8789423	10.60515858	642.8789423	4.883328173
777.5955063	10.12123334	644.1034773	10.59602364	644.1034773	4.888825698
777.8789423	10.12132332	644.6703513	10.5918025	644.6703513	4.891366835
779.1034773	10.12053253	645.2967543	10.58714371	645.2967543	4.894172016
779.6703513	10.12205074	645.8948863	10.58270068	645.8948863	4.896847825
780.2967543	10.12533774	647.5955063	10.57009741	647.5955063	4.904441013
780.8948863	10.13103287	647.8789423	10.56800103	647.8789423	4.905704431
782.5955063	10.1202621	649.1034773	10.55895776	649.1034773	4.911155817
782.8789423	10.12131638	649.6703513	10.55477883	649.6703513	4.91367561
784.1034773	10.12389386	650.2967543	10.55016658	650.2967543	4.916457208
784.6703513	10.12473857	650.8948863	10.54576785	650.8948863	4.919110491
785.2967543	10.1256145	652.5955063	10.53328969	652.5955063	4.926639708
785.8948863	10.12643432	652.8789423	10.53121406	652.8789423	4.927892463
787.5955063	10.12887089	654.1034773	10.52226008	654.1034773	4.933297847
787.8789423	10.12932693	654.6703513	10.5181223	654.6703513	4.935796355
789.1034773	10.13035727	655.2967543	10.51355535	655.2967543	4.938554427
789.6703513	10.12927101	655.8948863	10.50919975	655.8948863	4.941185313
790.2967543	10.12928465	657.5955063	10.49684356	657.5955063	4.948650849
790.8948863	10.12992499	657.8789423	10.49478819	657.8789423	4.949893002
792.5955063	10.13175795	659.1034773	10.48592128	659.1034773	4.955252663
792.8789423	10.13203565	659.6703513	10.48182363	659.6703513	4.957730034
794.1034773	10.13317936	660.2967543	10.47730093	660.2967543	4.960464787
794.6703513	10.13368541	660.8948863	10.47298744	660.8948863	4.963073416
795.2967543	10.13422811	662.5955063	10.46075041	662.5955063	4.970475848
795.8948863	10.13472406	662.8789423	10.45871479	662.8789423	4.971707523
797.5955063	10.1359122	664.1034773	10.44993299	664.1034773	4.977021928
797.8789423	10.13607614	664.6703513	10.44587461	664.6703513	4.979478392
799.1034773	10.13675928	665.2967543	10.44139515	665.2967543	4.98219011
799.6703513	10.13708435	665.8948863	10.43712285	665.8948863	4.984776744
800.2967543	10.13745256	667.5955063	10.42500231	667.5955063	4.992116926

Final State: j'=5		Final State: j'=3		Final State: j'=2	
E(cm⁻¹)	σ (Å²)	E(cm⁻¹)	σ (Å²)	E(cm⁻¹)	σ (Å²)
800.8948863	10.13780911	667.8789423	10.42298601	667.8789423	4.993338242
802.5955063	10.13881841	669.1034773	10.41428744	669.1034773	4.998608085
802.8789423	10.1389833	669.6703513	10.41026743	669.6703513	5.001043986
804.1034773	10.13967758	670.2967543	10.40583027	670.2967543	5.003733027
804.6703513	10.13998723	670.8948863	10.40159825	670.8948863	5.00629807
805.2967543	10.14031941	672.5955063	10.38959162	672.5955063	5.013577166
805.8948863	10.14062627	672.8789423	10.38759424	672.8789423	5.014788364
807.5955063	10.14144322	674.1034773	10.37897706	674.1034773	5.020014608
807.8789423	10.14157186	674.6703513	10.37499458	674.6703513	5.022430426
809.1034773	10.14210589	675.2967543	10.37059875	675.2967543	5.02509735
809.6703513	10.14234212	675.8948863	10.3664061	675.8948863	5.027641357
810.2967543	10.14259574	677.5955063	10.35451077	677.5955063	5.034861114
810.8948863	10.14283083	677.8789423	10.35253183	677.8789423	5.03606248
812.5955063	10.14346249	679.1034773	10.3439941	679.1034773	5.041246549
812.8789423	10.14356249	679.6703513	10.34004823	679.6703513	5.04364298
814.1034773	10.14397691	680.2967543	10.33569272	680.2967543	5.046288618
814.6703513	10.14415895	680.8948863	10.33153842	680.8948863	5.048812425
815.2967543	10.14435279	682.5955063	10.31975147	682.5955063	5.055975438
815.8948863	10.14453067	682.8789423	10.31779051	682.8789423	5.057167458
817.5955063	10.1449976	684.1034773	10.30932997	684.1034773	5.062311581
817.8789423	10.14506982	684.6703513	10.30541959	684.6703513	5.06468978
819.1034773	10.14536373	685.2967543	10.30110317	685.2967543	5.067315452
819.6703513	10.14548985	685.8948863	10.29698603	685.8948863	5.069820403
820.2967543	10.14562197	687.5955063	10.28530368	687.5955063	5.076931099
820.8948863	10.1457411	687.8789423	10.28335999	687.8789423	5.078114627
822.5955063	10.14604278	689.1034773	10.27497351	689.1034773	5.083222753
822.8789423	10.14608781	689.6703513	10.27109711	689.6703513	5.085584773
824.1034773	10.14626518	690.2967543	10.26681791	690.2967543	5.088192995
824.6703513	10.14633797	690.8948863	10.26273603	690.8948863	5.090681708
825.2967543	10.14641161	692.5955063	10.25115206	692.5955063	5.09774908
825.8948863	10.14647524	692.8789423	10.24922444	692.8789423	5.098925843
827.5955063	10.146621	694.1034773	10.24090627	694.1034773	5.10400684
827.8789423	10.14664027	694.6703513	10.23706071	694.6703513	5.106357592
829.1034773	10.1467071	695.2967543	10.23281367	695.2967543	5.108952902
829.6703513	10.14672909	695.8948863	10.22876421	695.8948863	5.111433753
830.2967543	10.14674682	697.5955063	10.21726373	697.5955063	5.11848371
830.8948863	10.14675736	697.8789423	10.21534911	697.8789423	5.119659389
832.5955063	10.1467535	699.1034773	10.20708285	699.1034773	5.124744444
832.8789423	10.14674805	699.6703513	10.20325842	699.6703513	5.127103547
834.1034773	10.14670871	700.2967543	10.19903316	700.2967543	5.129716542
834.6703513	10.14668191	700.8948863	10.19499851	700.8948863	5.132220462
835.2967543	10.14664604	702.5955063	10.18351505	702.5955063	5.139439837
835.8948863	10.14660567	702.8789423	10.18159747	702.8789423	5.140672355
837.5955063	10.14645861	704.1034773	10.17331416	704.1034773	5.146342657
837.8789423	10.14642949	704.6703513	10.16970695	704.6703513	5.149852379

Final State: j’=5		Final State: j’=3		Final State: j’=2	
E(cm⁻¹)	σ (Å²)	E(cm⁻¹)	σ (Å²)	E(cm⁻¹)	σ (Å²)
839.1034773	10.14628871	705.2967543	10.16883241	705.2967543	5.156828176
839.6703513	10.14621534	705.8948863	10.16824873	705.8948863	5.160667795
840.2967543	10.1461283	707.5955063	10.15769165	707.5955063	5.165848129
840.8948863	10.14603936	707.8789423	10.15307676	707.8789423	5.162955264
842.5955063	10.14575562	709.1034773	10.14275822	709.1034773	5.170811767
842.8789423	10.14570394	709.6703513	10.144015	709.6703513	5.176901895
844.1034773	10.14546634	710.2967543	10.13822293	710.2967543	5.173864922
844.6703513	10.14534853	710.8948863	10.13176617	710.8948863	5.174652972
845.2967543	10.14521263	712.5955063	10.12085906	712.5955063	5.184707438
845.8948863	10.14507733	712.8789423	10.12246754	712.8789423	5.190415885
847.5955063	10.14466319	714.1034773	10.11057703	714.1034773	5.188646947
847.8789423	10.14458998	714.6703513	10.1104041	714.6703513	5.190614444
849.1034773	10.14426001	715.2967543	10.10164282	715.2967543	5.190027934
849.6703513	10.14409983	715.8948863	10.09727285	715.8948863	5.193350484
850.2967543	10.14391736	717.5955063	10.09000408	717.5955063	5.202440575
850.8948863	10.14373785	717.8789423	10.08785912	717.8789423	5.20463423
852.5955063	10.14319942	719.1034773	10.07754167	719.1034773	5.204254879
852.8789423	10.14310568	719.6703513	10.07317588	719.6703513	5.206947282
854.1034773	10.14268774	720.2967543	10.06880798	720.2967543	5.210121058
854.6703513	10.14248718	720.8948863	10.06495762	720.8948863	5.213943158
855.2967543	10.14226037	722.5955063	10.0572679	722.5955063	5.223201115
855.8948863	10.14203876	722.8789423	10.06115225	722.8789423	5.228401285
857.5955063	10.14138201	724.1034773	10.04503629	724.1034773	5.223605761
857.8789423	10.14126876	724.6703513	10.04100785	724.6703513	5.226262718
859.1034773	10.14076709	725.2967543	10.03676109	725.2967543	5.229233045
859.6703513	10.14052814	725.8948863	10.03281448	725.8948863	5.232314737
860.2967543	10.14025915	727.5955063	10.03053612	727.5955063	5.244031073
860.8948863	10.1399975	727.8789423	10.02339094	727.8789423	5.242233668
862.5955063	10.13922828	729.1034773	10.0138908	729.1034773	5.242396638
862.8789423	10.13909648	729.6703513	10.0093792	729.6703513	5.245133076
864.1034773	10.13851527	730.2967543	10.00509318	730.2967543	5.24802982
864.6703513	10.13823981	730.8948863	10.00112962	730.8948863	5.250763907
865.2967543	10.13793076	732.5955063	9.990393747	732.5955063	5.260004225
865.8948863	10.13763109	732.8789423	9.98942731	732.8789423	5.263305441
867.5955063	10.13675506	734.1034773	9.984299504	734.1034773	5.26825505
867.8789423	10.13660564	734.6703513	9.98579104	734.6703513	5.270401876
869.1034773	10.13594893	735.2967543	9.974035212	735.2967543	5.266592315
869.6703513	10.13563884	735.8948863	9.969646908	735.8948863	5.269674636
870.2967543	10.13529177	737.5955063	9.958405009	737.5955063	5.278196329
870.8948863	10.13495604	737.8789423	9.956588873	737.8789423	5.279902356
872.5955063	10.13397871	739.1034773	9.954559709	739.1034773	5.294525154
872.8789423	10.13381258	739.6703513	9.950502509	739.6703513	5.293696405
874.1034773	10.13308425	740.2967543	9.95768355	740.2967543	5.306211666
874.6703513	10.13274133	740.8948863	9.948434225	740.8948863	5.3024637
875.2967543	10.13235821	742.5955063	9.939000821	742.5955063	5.309511097

Final State: j’=5		Final State: j’=3		Final State: j’=2	
E(cm⁻¹)	σ (Å²)	E(cm⁻¹)	σ (Å²)	E(cm⁻¹)	σ (Å²)
875.8948863	10.13198828	742.8789423	9.934494757	742.8789423	5.303582897
877.5955063	10.13091487	744.1034773	9.919949088	744.1034773	5.30525655
877.8789423	10.13073288	744.6703513	9.926611301	744.6703513	5.320943215
879.1034773	10.12993667	745.2967543	9.922170023	745.2967543	5.314317411
879.6703513	10.12956262	745.8948863	9.910069171	745.8948863	5.308944363
880.2967543	10.12914531	747.5955063	9.923256944	747.5955063	5.346334398
880.8948863	10.12874295	747.8789423	9.922373002	747.8789423	5.343025335
882.5955063	10.12757845	749.1034773	9.891456618	749.1034773	5.317486322
882.8789423	10.12738145	749.6703513	9.885870124	749.6703513	5.321341939
884.1034773	10.12652086	750.2967543	9.881591786	750.2967543	5.326258053
884.6703513	10.12611746	750.8948863	9.885463489	750.8948863	5.34222004
885.2967543	10.12566756	752.5955063	9.868739208	752.5955063	5.332243905
885.8948863	10.12523446	752.8789423	9.866615335	752.8789423	5.333759527
887.5955063	10.12398359	754.1034773	9.858460668	754.1034773	5.341565228
887.8789423	10.12377235	754.6703513	9.857616126	754.6703513	5.351476057
889.1034773	10.1228507	755.2967543	9.873025444	755.2967543	5.366915628
889.6703513	10.12241911	755.8948863	9.854846977	755.8948863	5.347281159
890.2967543	10.12193865	757.5955063	9.842134923	757.5955063	5.358749843
890.8948863	10.12147636	757.8789423	9.839218348	757.8789423	5.358256006
892.5955063	10.12014346	759.1034773	9.834679627	759.1034773	5.371661411
892.8789423	10.11991867	759.6703513	9.849644456	759.6703513	5.383529282
894.1034773	10.11893898	760.2967543	9.824654984	760.2967543	5.360381038
894.6703513	10.11848076	760.8948863	9.820376079	760.8948863	5.370724388
895.2967543	10.117971	762.5955063	9.808314529	762.5955063	5.374114709
895.8948863	10.11748091	762.8789423	9.815145131	762.8789423	5.390708151
897.5955063	10.11606982	764.1034773	9.826012369	764.1034773	5.401511385
897.8789423	10.11583212	764.6703513	9.80520549	764.6703513	5.381680046
899.1034773	10.11479693	765.2967543	9.803987554	765.2967543	5.391530302
899.6703513	10.11431321	765.8948863	9.790425037	765.8948863	5.380839628
900.2967543	10.1137754	767.5955063	9.793254571	767.5955063	5.404087763
900.8948863	10.11325864	767.8789423	9.78118064	767.8789423	5.395001526
902.5955063	10.11177228	769.1034773	9.787503141	769.1034773	5.411253039
902.8789423	10.11152211	769.6703513	9.773461195	769.6703513	5.403074065
904.1034773	10.11043323	770.2967543	9.768922021	770.2967543	5.405586616
904.6703513	10.10992474	770.8948863	9.765462647	770.8948863	5.408695633
905.2967543	10.10935962	772.5955063	9.761145534	772.5955063	5.419565361
905.8948863	10.10881681	772.8789423	9.760552444	772.8789423	5.422768236
907.5955063	10.10725642	774.1034773	9.749230936	774.1034773	5.420675126
907.8789423	10.10699387	774.6703513	9.745331155	774.6703513	5.422997952
909.1034773	10.10585145	775.2967543	9.741773101	775.2967543	5.425754599
909.6703513	10.10531804	775.8948863	9.738486065	775.8948863	5.428327518
910.2967543	10.10472523	777.5955063	9.729895552	777.5955063	5.435991303
910.8948863	10.1041558	777.8789423	9.728584749	777.8789423	5.437231356
912.5955063	10.10251826	779.1034773	9.721803514	779.1034773	5.441572759
912.8789423	10.10224259	779.6703513	9.718580382	779.6703513	5.443705312

Final State: j’=5		Final State: j’=3		Final State: j’=2	
E(cm⁻¹)	σ (Å²)	E(cm⁻¹)	σ (Å²)	E(cm⁻¹)	σ (Å²)
914.1034773	10.10104208	780.2967543	9.715222155	780.2967543	5.446363947
914.6703513	10.10048087	780.8948863	9.715100327	780.8948863	5.450893757
915.2967543	10.09985646	782.5955063	9.702215734	782.5955063	5.454001792
915.8948863	10.09925579	782.8789423	9.700714406	782.8789423	5.455166101
917.5955063	10.0975213	784.1034773	9.694149402	784.1034773	5.459947825
917.8789423	10.09722786	784.6703513	9.691084309	784.6703513	5.462104612
919.1034773	10.09594211	785.2967543	9.68769411	785.2967543	5.464475837
919.6703513	10.095335	785.8948863	9.684461352	785.8948863	5.466737129
920.2967543	10.09465266	787.5955063	9.675347212	787.5955063	5.473202274
920.8948863	10.09398652	787.8789423	9.673855361	787.8789423	5.474297401
922.5955063	10.09194147	789.1034773	9.667697374	789.1034773	5.479087506
922.8789423	10.09155406	789.6703513	9.66459916	789.6703513	5.481014447
924.1034773	10.09042893	790.2967543	9.661004561	790.2967543	5.48316039
924.6703513	10.09151107	790.8948863	9.65768574	790.8948863	5.485325814
925.2967543	10.09035849	792.5955063	9.648509415	792.5955063	5.491609574
925.8948863	10.08896901	792.8789423	9.646993942	792.8789423	5.492659282
927.5955063	10.08721647	794.1034773	9.640471025	794.1034773	5.497193464
927.8789423	10.0863433	794.6703513	9.637463867	794.6703513	5.499292077
929.1034773	10.08836483	795.2967543	9.634150527	795.2967543	5.501611004
929.6703513	10.08620922	795.8948863	9.630996573	795.8948863	5.503824858
930.2967543	10.08591773	797.5955063	9.622064159	797.5955063	5.510098219
930.8948863	10.0842513	797.8789423	9.620575508	797.8789423	5.511138001
932.5955063	10.08419003	799.1034773	9.614137872	799.1034773	5.515615894
932.8789423	10.08324546	799.6703513	9.611159145	799.6703513	5.517685544
934.1034773	10.08146887	800.2967543	9.607872372	800.2967543	5.519972211
934.6703513	10.08021437	800.8948863	9.604739815	800.8948863	5.522155585
935.2967543	10.07883194	802.5955063	9.595867336	802.5955063	5.528361574
935.8948863	10.08171411	802.8789423	9.594393502	802.8789423	5.529395384
937.5955063	10.08029067	804.1034773	9.588041791	804.1034773	5.533859251
937.8789423	10.07814491	804.6703513	9.585109815	804.6703513	5.535924017
939.1034773	10.07555595	805.2967543	9.581875837	805.2967543	5.53820411
939.6703513	10.0744435	805.8948863	9.578793398	805.8948863	5.540379678
940.2967543	10.07369738	807.5955063	9.570057209	807.5955063	5.546555559
940.8948863	10.07814856	807.8789423	9.568604998	807.8789423	5.547583432
942.5955063	10.07318323	809.1034773	9.562343342	809.1034773	5.552019546
942.8789423	10.07240666	809.6703513	9.559451489	809.6703513	5.554070718
944.1034773	10.07018894	810.2967543	9.556261075	810.2967543	5.556335594
944.6703513	10.069221	810.8948863	9.553219743	810.8948863	5.558496616
945.2967543	10.0679898	812.5955063	9.544600347	812.5955063	5.564632545
945.8948863	10.06874143	812.8789423	9.543167808	812.8789423	5.565654011
947.5955063	10.06944847	814.1034773	9.536992286	814.1034773	5.570063186
947.8789423	10.06780715	814.6703513	9.534140854	814.6703513	5.572102194
949.1034773	10.06483348	815.2967543	9.530995409	815.2967543	5.57435372
949.6703513	10.06391976	815.8948863	9.527997272	815.8948863	5.576502063
950.2967543	10.06292434	817.5955063	9.519501271	817.5955063	5.582601754

Final State: j'=5		Final State: j'=3		Final State: j'=2	
E(cm⁻¹)	σ (Å²)	E(cm⁻¹)	σ (Å²)	E(cm⁻¹)	σ (Å²)
950.8948863	10.06192296	817.8789423	9.51808934	817.8789423	5.583617154
952.5955063	10.06438058	819.1034773	9.512002724	819.1034773	5.587999861
952.8789423	10.06142913	819.6703513	9.509192359	819.6703513	5.590026491
954.1034773	10.05980193	820.2967543	9.506092271	820.2967543	5.592264298
954.6703513	10.05833318	820.8948863	9.503137372	820.8948863	5.594399483
955.2967543	10.05716749	822.5955063	9.494764155	822.5955063	5.600461613
955.8948863	10.0561117	822.8789423	9.493372677	822.8789423	5.601470701
957.5955063	10.05315934	824.1034773	9.487374451	824.1034773	5.605826256
957.8789423	10.05336035	824.6703513	9.484605031	824.6703513	5.607840322
959.1034773	10.05649323	825.2967543	9.481550234	825.2967543	5.61006424
959.6703513	10.05533411	825.8948863	9.478638616	825.8948863	5.612186151
960.2967543	10.05500701	827.5955063	9.470388653	827.5955063	5.618210524
960.8948863	10.05142487	827.8789423	9.469017751	827.8789423	5.619213328
962.5955063	10.04915087	829.1034773	9.463108465	829.1034773	5.623541646
962.8789423	10.04811394	829.6703513	9.460380288	829.6703513	5.625543063
964.1034773	10.05122787	830.2967543	9.457371078	830.2967543	5.627752999
964.6703513	10.0477393	830.8948863	9.454503006	830.8948863	5.629861559
965.2967543	10.04552289	832.5955063	9.44637699	832.5955063	5.635847796
965.8948863	10.04326999	832.8789423	9.445026776	832.8789423	5.636844233
967.5955063	10.04802707	834.1034773	9.439206865	834.1034773	5.641144964
967.8789423	10.04677714	834.6703513	9.436520074	834.6703513	5.6431336
969.1034773	10.04107351	835.2967543	9.433556609	835.2967543	5.645329365
969.6703513	10.04003975	835.8948863	9.430732272	835.8948863	5.647424362
970.2967543	10.04746417	837.5955063	9.422730659	837.5955063	5.653371983
970.8948863	10.04190974	837.8789423	9.421401169	837.8789423	5.654361965
972.5955063	10.03677863	839.1034773	9.415670973	839.1034773	5.658634769
972.8789423	10.03602086	839.6703513	9.41302575	839.6703513	5.660610434
974.1034773	10.04105488	840.2967543	9.410108228	840.2967543	5.662791852
974.6703513	10.04119414	840.8948863	9.407327768	840.8948863	5.664873124
975.2967543	10.0364477	842.5955063	9.399451071	842.5955063	5.670781579
975.8948863	10.03461146	842.8789423	9.398142436	842.8789423	5.671765014
977.5955063	10.0319089	844.1034773	9.392502312	844.1034773	5.676009473
977.8789423	10.02971517	844.6703513	9.389898804	844.6703513	5.677971989
979.1034773	10.03249729	845.2967543	9.387027412	845.2967543	5.680138845
979.6703513	10.02850028	845.8948863	9.384291031	845.8948863	5.682206177
980.2967543	10.03173056	847.5955063	9.376539747	847.5955063	5.688074873
980.8948863	10.02809833	847.8789423	9.375252039	847.8789423	5.689051662
982.5955063	10.02672402	849.1034773	9.369702318	849.1034773	5.693267325
982.8789423	10.03113339	849.6703513	9.367140699	849.6703513	5.695216464
984.1034773	10.03044948	850.2967543	9.364315611	850.2967543	5.697368521
984.6703513	10.02634962	850.8948863	9.361623459	850.8948863	5.699421682
985.2967543	10.02252631	852.5955063	9.353998047	852.5955063	5.705249939
985.8948863	10.02041266	852.8789423	9.352731328	852.8789423	5.706219969
987.5955063	10.02263418	854.1034773	9.347272363	854.1034773	5.71040633
987.8789423	10.0252654	854.6703513	9.34475278	854.6703513	5.712341859

Final State: j'=5		Final State: j'=3		Final State: j'=2	
E(cm⁻¹)	σ (Å²)	E(cm⁻¹)	σ (Å²)	E(cm⁻¹)	σ (Å²)
989.1034773	10.01938227	855.2967543	9.341974163	855.2967543	5.714478847
989.6703513	10.01832563	855.8948863	9.339326395	855.8948863	5.716517595
990.2967543	10.01740524	857.5955063	9.33182731	857.5955063	5.722304659
990.8948863	10.01650258	857.8789423	9.330581647	857.8789423	5.723267797
992.5955063	10.0173341	859.1034773	9.325213724	859.1034773	5.727424297
992.8789423	10.01634404	859.6703513	9.322736331	859.6703513	5.729345962
994.1034773	10.01385403	860.2967543	9.320004346	860.2967543	5.73146759
994.6703513	10.01310021	860.8948863	9.317401126	860.8948863	5.733491638
995.2967543	10.01231707	862.5955063	9.310028766	862.5955063	5.73923672
995.8948863	10.01163659	862.8789423	9.308804222	862.8789423	5.740192816
997.5955063	10.01009041	864.1034773	9.303527669	864.1034773	5.744318871
997.8789423	10.00973217	864.6703513	9.301092581	864.6703513	5.746226368
999.1034773	10.00815367	865.2967543	9.298407376	865.2967543	5.748332313
999.6703513	10.00788739	865.8948863	9.295848813	865.8948863	5.750341347
1000.296754	10.00844401	867.5955063	9.288603566	867.5955063	5.75604354
1000.894884	10.00644418	867.8789423	9.287400241	867.8789423	5.756992453
1002.595504	10.00429347	869.1034773	9.282215287	869.1034773	5.761087379
1002.878944	10.00396691	869.6703513	9.279822649	869.6703513	5.762980423
1004.103474	10.00255212	870.2967543	9.277184335	870.2967543	5.765070335
1004.670354	10.00189658	870.8948863	9.274670598	870.8948863	5.767064029
1005.296754	10.00117367	872.5955063	9.267552823	872.5955063	5.772722349
1005.894884	10.00048642	872.8789423	9.266370741	872.8789423	5.773663934
1007.595504	9.998651422	874.1034773	9.261277681	874.1034773	5.777726998
1007.878944	9.998404248	874.6703513	9.258927571	874.6703513	5.779605256
1009.103474	9.997009646	875.2967543	9.256336296	875.2967543	5.781678753
1009.670354	9.99630104	875.8948863	9.253867482	875.8948863	5.783656741
1010.296754	9.995547595	877.5955063	9.246877483	877.5955063	5.789270137
1010.894884	9.994843048	877.8789423	9.245716717	877.8789423	5.790204192
1012.595504	9.992870983	879.1034773	9.240715745	879.1034773	5.794234649
1012.878944	9.992544667	879.6703513	9.238408281	879.6703513	5.796097705
1014.103474	9.991141148	880.2967543	9.235864151	880.2967543	5.798154387
1014.670354	9.990495536	880.8948863	9.233440355	880.8948863	5.800116252
1015.296754	9.989785714	882.5955063	9.226578444	882.5955063	5.805683568
1015.894884	9.98911072	882.8789423	9.225439027	882.8789423	5.806609907
1017.595504	9.987186183	884.1034773	9.220530333	884.1034773	5.810606864
1017.878944	9.986863206	884.6703513	9.218265618	884.6703513	5.812454523
1019.103474	9.985464088	885.2967543	9.21576869	885.2967543	5.81449374
1019.670354	9.984815946	885.8948863	9.213390016	885.8948863	5.816439066
1020.296754	9.984100199	887.5955063	9.206656413	887.5955063	5.821958989
1020.894884	9.983417395	887.8789423	9.20553838	887.8789423	5.822877394
1022.595504	9.981480501	889.1034773	9.200722126	889.1034773	5.826839872
1022.878944	9.981158357	889.6703513	9.198500218	889.6703513	5.828671314
1024.103474	9.979768816	890.2967543	9.196050632	890.2967543	5.830692928
1024.670354	9.979126687	890.8948863	9.193717154	890.8948863	5.832621156
1025.296754	9.978417821	892.5955063	9.187112029	892.5955063	5.838092259

Final State: j'=5		Final State: j'=3		Final State: j'=2	
E(cm⁻¹)	σ (Å²)	E(cm⁻¹)	σ (Å²)	E(cm⁻¹)	σ (Å²)
1025.894884	9.977741525	892.8789423	9.186015432	892.8789423	5.839002462
1027.595504	9.975821144	894.1034773	9.18129175	894.1034773	5.84292935
1029.103474	9.974120839	894.6703513	9.179112703	894.6703513	5.844744208
1029.670354	9.973482304	895.2967543	9.176710483	895.2967543	5.846747445
1030.296754	9.972777186	895.8948863	9.174422222	895.8948863	5.848658045
1030.894884	9.972104367	897.5955063	9.167945768	897.5955063	5.854078517
1032.595504	9.970194261	897.8789423	9.166870632	897.8789423	5.854980224
1034.103474	9.968504334	899.1034773	9.162239639	899.1034773	5.85887016
1034.670354	9.967870024	899.6703513	9.160103488	899.6703513	5.86066779
1035.296754	9.967169732	900.2967543	9.157748677	900.2967543	5.862651867
1035.894884	9.966501654	900.8948863	9.155505704	900.8948863	5.864544087
1037.595504	9.964605479	902.5955063	9.149158022	902.5955063	5.869911697
1039.103474	9.962928177	902.8789423	9.148104332	902.8789423	5.870804508
1039.670354	9.962298652	904.1034773	9.14356606	904.1034773	5.874655693
1040.296754	9.961603642	904.6703513	9.141472868	904.6703513	5.876435197
1040.894884	9.960940648	905.2967543	9.139165511	905.2967543	5.878399083
1042.595504	9.959058988	905.8948863	9.136967844	905.8948863	5.880271894
1044.103474	9.957394764	907.5955063	9.130749007	907.5955063	5.885583394
1044.670354	9.956770217	907.8789423	9.129716811	907.8789423	5.886466693
1045.296754	9.956080787	909.1034773	9.125271338	909.1034773	5.890276318
1045.894884	9.955423135	909.6703513	9.123221101	909.6703513	5.892036251
1047.595504	9.95355695	910.2967543	9.120961225	910.2967543	5.893978243
1049.103474	9.951906739	910.8948863	9.118808898	910.8948863	5.895829854
1049.670354	9.951287547	912.5955063	9.112719048	912.5955063	5.901079253
1050.296754	9.950604041	912.8789423	9.111708343	912.8789423	5.901951889
1050.894884	9.949952074	914.1034773	9.10735583	914.1034773	5.905714174
1052.595504	9.948102281	914.6703513	9.105348651	914.6703513	5.907451362
1054.103474	9.946466845	915.2967543	9.10313638	915.2967543	5.909367479
1054.670354	9.945853222	915.8948863	9.101029555	915.8948863	5.911193533
1055.296754	9.945175917	917.5955063	9.095069315	917.5955063	5.916364036
1055.894884	9.944529923	917.8789423	9.094080269	917.8789423	5.917222324
1057.595504	9.942697245	919.1034773	9.089821639	919.1034773	5.920916673
1059.103474	9.941077147	919.6703513	9.087858155	919.6703513	5.922617859
1059.670354	9.940469344	920.2967543	9.085694459	920.2967543	5.924489205
1060.296754	9.939798509	920.8948863	9.083634414	920.8948863	5.926265288
1060.894884	9.939158718	922.5955063	9.077812562	922.5955063	5.931202824
1062.595504	9.937343771	922.8789423	9.076848312	922.8789423	5.931988934
1064.103474	9.935739556	924.1034773	9.072554546	924.1034773	5.935090403
1064.670354	9.935137769	924.6703513	9.070411334	924.6703513	5.93734319
1065.296754	9.934473587	925.2967543	9.068458883	925.2967543	5.939817161
1065.894884	9.933840163	925.8948863	9.066488273	925.8948863	5.940724455
1067.595504	9.932043464	927.5955063	9.060926648	927.5955063	5.946405433
1069.103474	9.930455572	927.8789423	9.060002029	927.8789423	5.946732497
1069.670354	9.929859942	929.1034773	9.055636163	929.1034773	5.951767496
1070.296754	9.929202593	929.6703513	9.053888257	929.6703513	5.953049047

Final State: j’=5		Final State: j’=3		Final State: j’=2	
E(cm⁻¹)	σ (Å²)	E(cm⁻¹)	σ (Å²)	E(cm⁻¹)	σ (Å²)
1070.894884	9.928575701	930.2967543	9.05158525	930.2967543	5.954990664
1072.595504	9.926797669	930.8948863	9.049850959	930.8948863	5.956538157
1074.103474	9.925226442	932.5955063	9.044161025	932.5955063	5.962189263
1074.670354	9.924637098	932.8789423	9.04337953	932.8789423	5.96245529
1075.296754	9.923986681	934.1034773	9.039376801	934.1034773	5.966504963
1075.894884	9.923366466	934.6703513	9.037537908	934.6703513	5.967771552
1077.595504	9.921607425	935.2967543	9.035449878	935.2967543	5.968898257
1079.103474	9.920053104	935.8948863	9.033071662	935.8948863	5.971095441
1079.670354	9.919470137	937.5955063	9.028028975	937.5955063	5.977651165
1080.296754	9.91882679	937.8789423	9.027255345	937.8789423	5.978065075
1080.894884	9.918213286	939.1034773	9.023348906	939.1034773	5.980909747
1084.103474	9.914936242	939.6703513	9.021525172	939.6703513	5.982188505
1084.670354	9.914359706	940.2967543	9.019262146	940.2967543	5.983060268
1085.296754	9.913723473	940.8948863	9.017620793	940.8948863	5.986902977
1085.894884	9.913116786	942.5955063	9.012441403	942.5955063	5.991957466
1089.103474	9.909876237	942.8789423	9.011549672	942.8789423	5.992531785
1089.670354	9.909306234	944.1034773	9.007712273	944.1034773	5.995547354
1090.296754	9.908677066	944.6703513	9.005934395	944.6703513	5.996945922
1090.894884	9.908077206	945.2967543	9.003938841	945.2967543	5.998300516
1094.103474	9.904873222	945.8948863	9.001753551	945.8948863	5.999382872
1094.670354	9.904309602	947.5955063	8.996771591	947.5955063	6.006386615
1095.296754	9.903687648	947.8789423	8.996281455	947.8789423	6.007430813
1095.894884	9.903094582	949.1034773	8.992497707	949.1034773	6.010071033
1099.103474	9.899927047	949.6703513	8.990768908	949.6703513	6.011510485
1099.670354	9.899369845	950.2967543	8.988861486	950.2967543	6.013098044
1100.894884	9.898168666	950.8948863	8.98703346	950.8948863	6.014560105
1104.103474	9.895037266	952.5955063	8.982248337	952.5955063	6.02059423
1104.670354	9.894486425	952.8789423	8.981186577	952.8789423	6.020381364
1105.894884	9.89329897	954.1034773	8.977774034	954.1034773	6.024594376
1109.103474	9.890203272	954.6703513	8.976045887	954.6703513	6.025718997
1109.670354	9.889658697	955.2967543	8.9741875	955.2967543	6.02718898
1110.894884	9.888484724	955.8948863	8.972433832	955.8948863	6.028611954
1114.103474	9.885424123	957.5955063	8.967440264	957.5955063	6.032008183
1114.670354	9.884885713	957.8789423	8.96651171	957.8789423	6.032669724
1115.894884	9.883724992	959.1034773	8.962560227	959.1034773	6.036548616
1119.103474	9.880698726	959.6703513	8.961209026	959.6703513	6.03948073
1119.670354	9.880166323	960.2967543	8.95953528	960.2967543	6.042070899
1120.894884	9.87901853	960.8948863	8.958084656	960.8948863	6.041951796
1124.103474	9.876025773	962.5955063	8.953012888	962.5955063	6.047639451
1124.670354	9.875499225	962.8789423	8.952260414	962.8789423	6.04784765
1125.894884	9.874364016	964.1034773	8.948243534	964.1034773	6.052932183
1129.103474	9.871403784	964.6703513	8.946939916	964.6703513	6.054008802
1129.670354	9.870882918	965.2967543	8.945263614	965.2967543	6.054739105
1130.894884	9.86975991	965.8948863	8.943524892	965.8948863	6.054965777
1134.103474	9.866831164	967.5955063	8.93907857	967.5955063	6.06339834

Final State: j’=5		Final State: j’=3		Final State: j’=2	
E(cm⁻¹)	σ (Å²)	E(cm⁻¹)	σ (Å²)	E(cm⁻¹)	σ (Å²)
1134.670354	9.866315775	967.8789423	8.938187451	967.8789423	6.063582304
1135.894884	9.865204559	969.1034773	8.935040321	969.1034773	6.065145899
1139.103474	9.862306155	969.6703513	8.933319266	969.6703513	6.064790316
1139.670354	9.861796061	970.2967543	8.931117168	970.2967543	6.070097852
1140.894884	9.860696193	970.8948863	8.929996363	970.8948863	6.071896847
1144.103474	9.857827031	972.5955063	8.925463047	972.5955063	6.074473963
1144.670354	9.857322032	972.8789423	8.924681444	972.8789423	6.074899132
1145.894884	9.856233102	974.1034773	8.920370261	974.1034773	6.077990561
1149.103474	9.853392175	974.6703513	8.920462938	974.6703513	6.082249131
1149.670354	9.852892113	975.2967543	8.917991773	975.2967543	6.081967343
1150.894884	9.851813777	975.8948863	8.917387952	975.8948863	6.084032981
1154.103474	9.849000481	977.5955063	8.912108449	977.5955063	6.087368342
1154.670354	9.848505279	977.8789423	8.911289184	977.8789423	6.087056748
1155.894884	9.84743751	979.1034773	8.908283446	979.1034773	6.093987751
1159.103474	9.844652484	979.6703513	8.907143007	979.6703513	6.093017695
1159.670354	9.84416245	980.2967543	8.9050659	980.2967543	6.095526773
1160.894884	9.843106173	980.8948863	8.903551068	980.8948863	6.097270499
1164.103474	9.840355431	982.5955063	8.898553613	982.5955063	6.099762164
1165.894884	9.838834651	982.8789423	8.897867297	982.8789423	6.100570852
1169.103474	9.836198671	984.1034773	8.89611873	984.1034773	6.106384518
1170.894884	9.834887205	984.6703513	8.894021079	984.6703513	6.108878738
1174.103474	9.832166827	985.2967543	8.892752	985.2967543	6.108461612
1175.894884	9.830401298	985.8948863	8.891153864	985.8948863	6.10832174
1179.103474	9.827645903	987.5955063	8.886228105	987.5955063	6.113051577
1180.894884	9.825969301	987.8789423	8.88555181	987.8789423	6.115041399
1184.103474	9.823035987	989.1034773	8.883130094	989.1034773	6.118183039
1185.894884	9.821802007	989.6703513	8.881667866	989.6703513	6.119341411
1189.103474	9.818864911	990.2967543	8.880038577	990.2967543	6.12068949
1190.894884	9.817532243	990.8948863	8.878387477	990.8948863	6.121885821
1194.103474	9.814574327	992.5955063	8.874784917	992.5955063	6.127465569
1195.894884	9.813303469	992.8789423	8.874143125	992.8789423	6.128186679
1199.103474	9.810724196	994.1034773	8.87106517	994.1034773	6.130763766
1200.894884	9.809166553	994.6703513	8.869679772	994.6703513	6.132105299
1204.103474	9.807433842	995.2967543	8.868158215	995.2967543	6.133604326
1205.894884	9.805292288	995.8948863	8.866707786	995.8948863	6.135044347
1209.103474	9.802467388	997.5955063	8.86271437	997.5955063	6.139379988
1210.894884	9.800515088	997.8789423	8.862056019	997.8789423	6.140085425
1214.103474	9.797642312	999.1034773	8.859156159	999.1034773	6.143030557
1215.894884	9.797280719	999.6703513	8.857636219	999.6703513	6.144286978
1219.103474	9.794006266	1000.296754	8.856638707	1000.296754	6.146522694
1220.894884	9.792123651	1000.894884	8.85526419	1000.894884	6.147751099
1224.103474	9.790111821	1002.595504	8.851277764	1002.595504	6.151767755
1225.894884	9.788459334	1002.878944	8.8506323	1002.878944	6.152457119
1229.103474	9.785956655	1004.103474	8.847861624	1004.103474	6.155433077
1230.894884	9.78346051	1004.670354	8.846586608	1004.670354	6.156807224

Final State: j’=5		Final State: j’=3		Final State: j’=2	
E(cm⁻¹)	σ (Å²)	E(cm⁻¹)	σ (Å²)	E(cm⁻¹)	σ (Å²)
1234.103474	9.781432456	1005.296754	8.84518236	1005.296754	6.158322566
1235.894884	9.779973632	1005.894884	8.843845509	1005.894884	6.159766199
1239.103474	9.777232037	1007.595504	8.840063716	1007.595504	6.163870171
1240.894884	9.775751081	1007.878944	8.839447027	1007.878944	6.164570857
1244.103474	9.772994255	1009.103474	8.836821551	1009.103474	6.167557135
1245.894884	9.771380492	1009.670354	8.835588091	1009.670354	6.168903024
1249.103474	9.768725576	1010.296754	8.834227446	1010.296754	6.170389805
1250.894884	9.767180921	1010.894884	8.832933279	1010.894884	6.17180913
1254.103474	9.764393717	1012.595504	8.829281288	1012.595504	6.175835542
1255.894884	9.762856809	1012.878944	8.828676447	1012.878944	6.176504802
1259.103474	9.760064284	1014.103474	8.826075522	1014.103474	6.17938983
1260.894884	9.758496966	1014.670354	8.824878308	1014.670354	6.180722033
1264.103474	9.755679217	1015.296754	8.823560727	1015.296754	6.182192041
1265.894884	9.754099907	1015.894884	8.822308222	1015.894884	6.183593772
1269.103474	9.751255975	1017.595504	8.818776136	1017.595504	6.187564974
1270.894884	9.749659816	1017.878944	8.818191098	1017.878944	6.188224141
1275.894884	9.74517336	1019.103474	8.815674204	1019.103474	6.191062767
1280.894884	9.740637334	1019.670354	8.81451501	1019.670354	6.192372105
1285.894884	9.736048344	1020.296754	8.813238556	1020.296754	6.19381564
1290.894884	9.731403385	1020.894884	8.812024164	1020.894884	6.195190898
1295.894884	9.72669948	1022.595504	8.808595553	1022.595504	6.199084784
1300.894884	9.721933787	1022.878944	8.808027616	1022.878944	6.199731449
1305.894884	9.717103662	1024.103474	8.80558556	1024.103474	6.20251777
1310.894884	9.712206576	1024.670354	8.804461466	1024.670354	6.20380351
1315.894884	9.707240159	1025.296754	8.80322401	1025.296754	6.20522123
1320.894884	9.702202201	1025.894884	8.802046992	1025.894884	6.206571961
1325.894884	9.697090726	1027.595504	8.798724885	1027.595504	6.210396141
1330.894884	9.691903866	1029.103474	8.795808889	1029.103474	6.213766752
1335.894884	9.686639967	1029.670354	8.794719874	1029.670354	6.21502883
1340.894884	9.681297526	1030.296754	8.793521019	1030.296754	6.216420302
1345.894884	9.675875278	1030.894884	8.792380708	1030.894884	6.217745856
1350.894884	9.670372101	1032.595504	8.789162076	1032.595504	6.221498196
1355.894884	9.664787082	1034.103474	8.786337056	1034.103474	6.224805017
1360.894884	9.65911952	1034.670354	8.785282125	1034.670354	6.226043153
1365.894884	9.653368878	1035.296754	8.784120849	1035.296754	6.227408141
1370.894884	9.64753485	1035.894884	8.783016312	1035.894884	6.228708448
1375.894884	9.641617294	1037.595504	8.779899074	1037.595504	6.232388972
1380.894884	9.635616295	1039.103474	8.777163417	1039.103474	6.235632087
1385.894884	9.62953214	1039.670354	8.776141914	1039.670354	6.236846247
1390.894884	9.623365238	1040.296754	8.775017483	1040.296754	6.238184749
1395.894884	9.617116268	1040.894884	8.773948062	1040.894884	6.239459727
1400.894884	9.610785925	1042.595504	8.770930036	1042.595504	6.24306815
1405.894884	9.604375304	1044.103474	8.768281685	1044.103474	6.246247267
		1044.670354	8.767292837	1044.670354	6.247437346
		1045.296754	8.766204384	1045.296754	6.248749233

Final State: j'=5		Final State: j'=3		Final State: j'=2	
E(cm⁻¹)	σ (Å²)	E(cm⁻¹)	σ (Å²)	E(cm⁻¹)	σ (Å²)
		1045.894884	8.765169212	1045.894884	6.24999878
		1047.595504	8.762248061	1047.595504	6.253534883
		1049.103474	8.759684924	1049.103474	6.256649785
		1049.670354	8.758727939	1049.670354	6.257815739
		1050.296754	8.757674607	1050.296754	6.259100936
		1050.894884	8.756672863	1050.894884	6.260324986
		1052.595504	8.753846182	1052.595504	6.263788617
		1054.103474	8.75136613	1054.103474	6.266839213
		1054.670354	8.750440215	1054.670354	6.267980976
		1055.296754	8.749421104	1055.296754	6.269239439
		1055.894884	8.748451926	1055.894884	6.270437969
		1057.595504	8.745717239	1057.595504	6.273828994
		1059.103474	8.743318068	1059.103474	6.276815214
		1059.670354	8.742422391	1059.670354	6.277932751
		1060.296754	8.741436553	1060.296754	6.279164469
		1060.894884	8.740499051	1060.894884	6.280337466
		1062.595504	8.73785387	1062.595504	6.283655848
		1064.103474	8.735533295	1064.103474	6.286577636
		1064.670354	8.734666971	1064.670354	6.287670957
		1065.296754	8.733713489	1065.296754	6.288875912
		1065.894884	8.732806752	1065.894884	6.290023365
		1067.595504	8.730248433	1067.595504	6.293269098
		1069.103474	8.728004132	1069.103474	6.296126495
		1069.670354	8.727166278	1069.670354	6.297195623
		1070.296754	8.726244172	1070.296754	6.298373826
		1070.894884	8.725367267	1070.894884	6.299495731
		1072.595504	8.722893148	1072.595504	6.302668888
		1074.103474	8.720722739	1074.103474	6.305461976
		1074.670354	8.719912496	1074.670354	6.306506943
		1075.296754	8.719020749	1075.296754	6.307658444
		1075.894884	8.718172725	1075.894884	6.308754863
		1077.595504	8.715780075	1077.595504	6.311855578
		1079.103474	8.71368112	1079.103474	6.314584464
		1079.670354	8.712897551	1079.670354	6.315605297
		1080.296754	8.712035137	1080.296754	6.316730169
		1080.894884	8.71121502	1080.894884	6.317801136
		1084.103474	8.706871076	1084.103474	6.323494485
		1084.670354	8.706113237	1084.670354	6.324491271
		1085.296754	8.705279136	1085.296754	6.32558957
		1085.894884	8.704485914	1085.894884	6.326635212
		1089.103474	8.700284258	1089.103474	6.332192755
		1089.670354	8.699550385	1089.670354	6.333166019
		1090.296754	8.698744331	1090.296754	6.334237427
		1090.894884	8.697977015	1090.894884	6.335257829
		1094.103474	8.69391216	1094.103474	6.340680159

Final State: j'=5		Final State: j'=3		Final State: j'=2	
E(cm⁻¹)	σ (Å²)	E(cm⁻¹)	σ (Å²)	E(cm⁻¹)	σ (Å²)
		1094.670354	8.693202908	1094.670354	6.34162914
		1095.296754	8.69242222	1095.296754	6.342674665
		1095.894884	8.691679757	1095.894884	6.343669935
		1099.103474	8.687746182	1099.103474	6.348957753
		1099.670354	8.687059713	1099.670354	6.349883026
		1100.894884	8.685585485	1100.894884	6.351872642
		1104.103474	8.681777529	1104.103474	6.357026778
		1104.670354	8.681112891	1104.670354	6.357928498
		1105.894884	8.679685399	1105.894884	6.35986726
		1109.103474	8.675997365	1109.103474	6.364888609
		1109.670354	8.675353545	1109.670354	6.365766913
		1110.894884	8.673970605	1110.894884	6.367655236
		1114.103474	8.670396732	1114.103474	6.372544801
		1114.670354	8.66977269	1114.670354	6.373399917
		1115.894884	8.66843207	1115.894884	6.375238179
		1119.103474	8.664966566	1119.103474	6.379997116
		1119.670354	8.664361269	1119.670354	6.380829241
		1120.894884	8.663060765	1120.894884	6.382617894
		1124.103474	8.659697723	1124.103474	6.387247454
		1124.670354	8.659110152	1124.670354	6.388056796
		1125.894884	8.65784752	1125.894884	6.389796361
		1129.103474	8.654581061	1129.103474	6.39429787
		1129.670354	8.654010151	1129.670354	6.395084685
		1130.894884	8.652783111	1130.894884	6.396775675
		1134.103474	8.649607262	1134.103474	6.401150605
		1134.670354	8.649051973	1134.670354	6.401915159
		1135.894884	8.64785823	1135.894884	6.403558171
		1139.103474	8.644767013	1139.103474	6.407808073
		1139.670354	8.644226253	1139.670354	6.408550649
		1140.894884	8.643063542	1140.894884	6.4101463
		1144.103474	8.64005084	1144.103474	6.414272891
		1144.670354	8.63952355	1144.670354	6.414993807
		1145.894884	8.638389502	1145.894884	6.416542778
		1149.103474	8.635449139	1149.103474	6.420547939
		1149.670354	8.634934212	1149.670354	6.421247533
		1150.894884	8.633826403	1150.894884	6.422750605
		1154.103474	8.630951915	1154.103474	6.426636482
		1154.670354	8.630448178	1154.670354	6.427315163
		1155.894884	8.629364033	1155.894884	6.428773254
		1159.103474	8.626548273	1159.103474	6.432542648
		1159.670354	8.626054336	1159.670354	6.433200985
		1160.894884	8.624990747	1160.894884	6.434615442
		1164.103474	8.622223612	1164.103474	6.438273263
		1165.894884	8.620687973	1165.894884	6.440286915
		1169.103474	8.61792318	1169.103474	6.443863746

Final State: j’=5		Final State: j’=3		Final State: j’=2	
E(cm⁻¹)	σ (Å²)	E(cm⁻¹)	σ (Å²)	E(cm⁻¹)	σ (Å²)
		1170.894884	8.616680073	1170.894884	6.445741184
		1174.103474	8.613756743	1174.103474	6.449243873
		1175.894884	8.61254032	1175.894884	6.450991496
		1179.103474	8.609832881	1179.103474	6.454333902
		1180.894884	8.608339235	1180.894884	6.456161122
		1184.103474	8.60586517	1184.103474	6.459265407
		1185.894884	8.604283669	1185.894884	6.461145111
		1189.103474	8.601829562	1189.103474	6.464134431
		1190.894884	8.600320412	1190.894884	6.465912281
		1194.103474	8.597906504	1194.103474	6.468770124
		1195.894884	8.596374109	1195.894884	6.470523232
		1199.103474	8.594023056	1199.103474	6.473336531
		1200.894884	8.592400877	1200.894884	6.475004351
		1204.103474	8.589876378	1204.103474	6.477942413
		1205.894884	8.589002012	1205.894884	6.47911784
		1209.103474	8.585975765	1209.103474	6.482070456
		1210.894884	8.584854884	1210.894884	6.483367986
		1214.103474	8.58245883	1214.103474	6.485834868
		1215.894884	8.580581286	1215.894884	6.487678326
		1219.103474	8.578174976	1219.103474	6.490054476
		1220.894884	8.577033232	1220.894884	6.491093436
		1224.103474	8.574229842	1224.103474	6.493925947
		1225.894884	8.572789294	1225.894884	6.495174904
		1229.103474	8.5704969	1229.103474	6.497300574
		1230.894884	8.56947437	1230.894884	6.498405927
		1234.103474	8.566807771	1234.103474	6.500724725
		1235.894884	8.565232825	1235.894884	6.502067767
		1239.103474	8.562740061	1239.103474	6.504145719
		1240.894884	8.561283031	1240.894884	6.505364769
		1244.103474	8.558728229	1244.103474	6.507402585
		1245.894884	8.557288128	1245.894884	6.508484001
		1249.103474	8.554693064	1249.103474	6.510510385
		1250.894884	8.553237846	1250.894884	6.511590801
		1254.103474	8.55061527	1254.103474	6.513478985
		1255.894884	8.549140854	1255.894884	6.514524118
		1259.103474	8.546484166	1259.103474	6.516347773
		1260.894884	8.544990722	1260.894884	6.517343662
		1264.103474	8.542296572	1264.103474	6.519090763
		1265.894884	8.540781575	1265.894884	6.520046736
		1269.103474	8.538047776	1269.103474	6.521722631
		1270.894884	8.536509683	1270.894884	6.522638321
		1275.894884	8.532170426	1275.894884	6.525120542
		1280.894884	8.527760046	1280.894884	6.527496696
		1285.894884	8.523274898	1285.894884	6.529769509
		1290.894884	8.518711697	1290.894884	6.53194177

Final State: j’=5		Final State: j’=3		Final State: j’=2	
E(cm⁻¹)	σ (Å²)	E(cm⁻¹)	σ (Å²)	E(cm⁻¹)	σ (Å²)
		1295.894884	8.514067522	1295.894884	6.534016141
		1300.894884	8.509339751	1300.894884	6.535995186
		1305.894884	8.504526108	1305.894884	6.537881344
		1310.894884	8.499624612	1310.894884	6.539676951
		1315.894884	8.494633634	1315.894884	6.54138422
		1320.894884	8.489551915	1320.894884	6.543005268
		1325.894884	8.484378429	1325.894884	6.544542038
		1330.894884	8.479112581	1330.894884	6.545996398
		1335.894884	8.473753956	1335.894884	6.547370078
		1340.894884	8.468302577	1340.894884	6.548664677
		1345.894884	8.462758674	1345.894884	6.549881697
		1350.894884	8.457122833	1350.894884	6.551022475
		1355.894884	8.451395894	1355.894884	6.55208825
		1360.894884	8.445578988	1360.894884	6.553080173
		1365.894884	8.439673498	1365.894884	6.553999219
		1370.894884	8.433681059	1370.894884	6.554846298
		1375.894884	8.427603559	1375.894884	6.555622165
		1380.894884	8.421443127	1380.894884	6.556327522
		1385.894884	8.415202083	1385.894884	6.556962915
		1390.894884	8.408883	1390.894884	6.557528842
		1395.894884	8.402488577	1395.894884	6.558025605
		1400.894884	8.396021782	1400.894884	6.558453656
		1405.894884	8.389485684	1405.894884	6.558813061

	Initial rotational state: j=4		
**Final State: j’=1**		**Final State: j’=0**	
E(cm⁻¹)	σ (Å²)	E(cm⁻¹)	σ (Å²)
274.2034773	30.31950034	274.2034773	15.57240874
274.3034773	56.53691588	274.3034773	27.94248935
274.4034773	23.86443203	274.4034773	12.22338601
274.5034773	20.56953935	274.5034773	10.38582319
274.6034773	18.70627496	274.6034773	9.25307731
274.6703513	18.28948923	274.6703513	8.71550582
274.7034773	19.22173621	274.7034773	8.82950666
274.8034773	20.60203813	274.8034773	9.61772957
274.9034773	16.61866174	274.9034773	8.51715106
275.0034773	15.26236594	275.0034773	7.6871391
275.1034773	16.60491784	275.1034773	7.9674609
275.2034773	13.37526988	275.2034773	6.92290777
275.2967543	12.39896754	275.2967543	6.42063024
275.3034773	12.34570852	275.3034773	6.39172939
275.4034773	11.66751848	275.4034773	6.01830156
275.5034773	11.11778442	275.5034773	5.71256167
277.5955063	7.774072296	277.5955063	3.94385732
277.8789423	7.091454437	277.8789423	3.59491111
279.1034773	6.021922853	279.1034773	3.009707793
279.2034773	5.968541892	279.2034773	2.979385075
279.3034773	5.911090627	279.3034773	2.947118116
279.4034773	5.849414857	279.4034773	2.912674742
279.5034773	5.783717342	279.5034773	2.875875279
279.6034773	5.714315106	279.6034773	2.836319748
279.6703513	5.665933557	279.6703513	2.807789445
279.7034773	5.641399619	279.7034773	2.792789957
279.8034773	5.566258956	279.8034773	2.742731878
279.9034773	5.504764717	279.9034773	2.698040843
280.0034773	5.494197593	280.0034773	2.699502693
280.2967543	5.58721827	280.2967543	2.665416857
282.5955063	4.471361569	282.5955063	2.212882952
282.8789423	4.350180133	282.8789423	2.151675291
284.1034773	3.991754063	284.1034773	1.964090319
284.6703513	3.957913122	284.6703513	1.934715941
285.2967543	4.062063618	285.2967543	1.962481888
287.5955063	3.471178725	287.5955063	1.679712023
287.8789423	3.419391005	287.8789423	1.653128672
289.1034773	3.819857933	289.1034773	1.799307911
289.6703513	3.172350996	289.6703513	1.527432393
290.2967543	3.076399228	290.2967543	1.480275148
292.5955063	2.831357796	292.5955063	1.356725522
292.8789423	2.802727576	292.8789423	1.342572018
294.1034773	2.690831646	294.1034773	1.286833523
294.6703513	2.647175741	294.6703513	1.264527757

Final State: j'=1		Final State: j'=0	
E(cm⁻¹)	σ (Å²)	E(cm⁻¹)	σ (Å²)
295.2967543	2.609654727	295.2967543	1.244001552
297.5955063	2.584210733	297.5955063	1.209624816
297.8789423	2.537021355	297.8789423	1.191014589
299.1034773	2.404542361	299.1034773	1.135042491
299.6703513	2.362934905	299.6703513	1.116118415
300.2967543	2.323030283	300.2967543	1.097499532
302.5955063	2.207240072	302.5955063	1.041408921
302.8789423	2.195749612	302.8789423	1.035604609
304.1034773	2.153540798	304.1034773	1.013344215
304.6703513	2.137158988	304.6703513	1.004258946
305.2967543	2.119243141	305.2967543	0.994424303
307.5955063	2.043353587	307.5955063	0.956514409
307.8789423	2.033803747	307.8789423	0.951926001
309.1034773	1.994474396	309.1034773	0.933032086
309.6703513	1.977486556	309.6703513	0.924832119
310.2967543	1.959634731	310.2967543	0.916169596
312.5955063	1.901883965	312.5955063	0.887682739
312.8789423	1.895521291	312.8789423	0.884492226
314.1034773	1.86954898	314.1034773	0.871374819
314.6703513	1.858219789	314.6703513	0.865621733
315.2967543	1.846105146	315.2967543	0.859464117
317.5955063	1.804522113	317.5955063	0.838396293
317.8789423	1.799676894	317.8789423	0.835951656
319.1034773	1.779447666	319.1034773	0.825759801
319.6703513	1.770474311	319.6703513	0.821241929
320.2967543	1.760848712	320.2967543	0.816393905
322.5955063	1.728089941	322.5955063	0.799842783
322.8789423	1.724319593	322.8789423	0.797929902
324.1034773	1.70866057	324.1034773	0.78996367
324.6703513	1.701740425	324.6703513	0.786431964
325.2967543	1.694319978	325.2967543	0.782637607
327.5955063	1.668930834	327.5955063	0.769606257
327.8789423	1.665983238	327.8789423	0.768089343
329.1034773	1.653665309	329.1034773	0.761743167
329.6703513	1.648180864	329.6703513	0.758914252
330.2967543	1.642272158	330.2967543	0.755864331
332.5955063	1.621854202	332.5955063	0.7453072
332.8789423	1.619465087	332.8789423	0.744069976
334.1034773	1.609440049	334.1034773	0.738873758
334.6703513	1.604953922	334.6703513	0.736546089
335.2967543	1.60010313	335.2967543	0.734027651
337.5955063	1.583155347	337.5955063	0.725219133
337.8789423	1.581147259	337.8789423	0.724174963
339.1034773	1.572639367	339.1034773	0.719752026
339.6703513	1.568779765	339.6703513	0.717746908

Final State: j’=1		Final State: j’=0	
E(cm^{-1})	σ (Å^2)	E(cm^{-1})	σ (Å^2)
340.2967543	1.56456011	340.2967543	0.715556471
342.5955063	1.54926249	342.5955063	0.707641622
342.8789423	1.547367667	342.8789423	0.706665208
344.1034773	1.53902912	344.1034773	0.702380765
344.6703513	1.535022355	344.6703513	0.700328719
345.2967543	1.530408533	345.2967543	0.697968916
347.5955063	1.509249163	347.5955063	0.686947954
347.8789423	1.505555425	347.8789423	0.684899298
349.1034773	1.48151579	349.1034773	0.665634369
349.6703513	1.493338535	349.6703513	0.65442588
350.2967543	1.533077132	350.2967543	0.667288859
352.5955063	1.511251664	352.5955063	0.680884107
352.8789423	1.501646439	352.8789423	0.676297856
354.1034773	1.516343925	354.1034773	0.652813414
354.6703513	1.52411353	354.6703513	0.663416551
355.2967543	1.518515205	355.2967543	0.676508722
357.5955063	1.538860984	357.5955063	0.665441239
357.8789423	1.513106164	357.8789423	0.672688396
359.1034773	1.544452024	359.1034773	0.678855161
359.6703513	1.516571055	359.6703513	0.67846951
360.2967543	1.494167974	360.2967543	0.667877664
362.5955063	1.518589619	362.5955063	0.63716806
362.8789423	1.560167942	362.8789423	0.674131475
364.1034773	1.509063289	364.1034773	0.675791514
364.6703513	1.495070754	364.6703513	0.669416156
365.2967543	1.475761364	365.2967543	0.656303695
367.5955063	1.556061805	367.5955063	0.668840786
367.8789423	1.544115692	367.8789423	0.683425824
369.1034773	1.503622523	369.1034773	0.673273646
369.6703513	1.493575974	369.6703513	0.668891888
370.2967543	1.48112435	370.2967543	0.662219286
372.5955063	1.500968951	372.5955063	0.640422066
372.8789423	1.536960119	372.8789423	0.66192884
374.1034773	1.506649002	374.1034773	0.673648139
374.6703513	1.496983246	374.6703513	0.670033893
375.2967543	1.488352032	375.2967543	0.666372359
377.5955063	1.460038837	377.5955063	0.614786776
377.8789423	1.506948218	377.8789423	0.641188345
379.1034773	1.513541347	379.1034773	0.660989571
379.6703513	1.505048949	379.6703513	0.671581277
380.2967543	1.487290354	380.2967543	0.665466422
382.5955063	1.440053122	382.5955063	0.639473919
382.8789423	1.438180865	382.8789423	0.630761327
384.1034773	1.440853536	384.1034773	0.620324123
384.6703513	1.493268537	384.6703513	0.609286417

Final State: j'=1		Final State: j'=0	
E(cm⁻¹)	σ (Å²)	E(cm⁻¹)	σ (Å²)
385.2967543	1.475267982	385.2967543	0.623135936
387.5955063	1.519202443	387.5955063	0.66246356
387.8789423	1.499884242	387.8789423	0.659251442
389.1034773	1.480406042	389.1034773	0.620447243
389.6703513	1.543394129	389.6703513	0.653426829
390.2967543	1.500560028	390.2967543	0.658983701
392.5955063	1.55873244	392.5955063	0.650598656
392.8789423	1.583223985	392.8789423	0.639651536
394.1034773	1.504894135	394.1034773	0.665082492
394.6703513	1.47738748	394.6703513	0.651070169
395.2967543	1.456356737	395.2967543	0.610831635
397.5955063	1.494117342	397.5955063	0.660654972
397.8789423	1.485778301	397.8789423	0.657252512
399.1034773	1.434353088	399.1034773	0.606692823
399.6703513	1.506823779	399.6703513	0.610147016
400.2967543	1.539278253	400.2967543	0.658377405
402.5955063	1.474654772	402.5955063	0.635282214
402.8789423	1.483110125	402.8789423	0.636619493
404.1034773	1.563702283	404.1034773	0.629275563
404.6703513	1.539799067	404.6703513	0.670553575
405.2967543	1.47042242	405.2967543	0.6299322
407.5955063	1.491039603	407.5955063	0.623266321
407.8789423	1.469947721	407.8789423	0.630409061
409.1034773	1.530178696	409.1034773	0.651361139
409.6703513	1.545652751	409.6703513	0.643647467
410.2967543	1.562465321	410.2967543	0.673499605
410.9948863	1.480373294	410.9948863	0.64597144
411.0948863	1.45473315	411.0948863	0.621400861
411.1948863	1.467611869	411.1948863	0.635744215
411.2948863	1.455402472	411.2948863	0.629575838
411.3948863	1.440809737	411.3948863	0.619613667
411.4948863	1.425149801	411.4948863	0.595613835
411.5948863	1.456916617	411.5948863	0.59846241
411.6948863	1.493038238	411.6948863	0.622200042
411.7948863	1.467183376	411.7948863	0.621303825
411.8948863	1.464202472	411.8948863	0.599743761
411.9948863	1.494934359	411.9948863	0.628880339
412.0948863	1.481030666	412.0948863	0.630495808
412.1948863	1.471823221	412.1948863	0.628665054
412.2948863	1.464870851	412.2948863	0.626374006
412.3948863	1.458889691	412.3948863	0.623907114
412.4948863	1.453196503	412.4948863	0.621194195
412.5955063	1.447238833	412.5955063	0.617996201
412.8789423	1.423499645	412.8789423	0.600405449
414.1034773	1.45758627	414.1034773	0.613018008

Final State: j’=1		Final State: j’=0	
E(cm⁻¹)	σ (Å²)	E(cm⁻¹)	σ (Å²)
414.6703513	1.452794056	414.6703513	0.613751104
415.2967543	1.443294295	415.2967543	0.6094192
415.8948863	1.432691986	415.8948863	0.603079236
415.9948863	1.430843897	415.9948863	0.601917313
416.0948863	1.428885324	416.0948863	0.600673134
416.1948863	1.426721778	416.1948863	0.599274203
416.2948863	1.424202369	416.2948863	0.597587185
416.3948863	1.4210801	416.3948863	0.595344915
416.4948863	1.416970413	416.4948863	0.591927845
416.5948863	1.411977808	416.5948863	0.585776145
416.6948863	1.41554107	416.6948863	0.578757153
416.7948863	1.423661281	416.7948863	0.578784153
416.8948863	1.430631257	416.8948863	0.58546723
416.9948863	1.428435319	416.9948863	0.588917373
417.5955063	1.428871411	417.5955063	0.588179602
417.8789423	1.425006002	417.8789423	0.584700031
419.1034773	1.441421862	419.1034773	0.602266977
419.6703513	1.438696055	419.6703513	0.600934739
420.2967543	1.436086221	420.2967543	0.599330895
420.8948863	1.433178236	420.8948863	0.597413269
422.5955063	1.421117738	422.5955063	0.588548393
422.8789423	1.422159001	422.8789423	0.588843191
424.1034773	1.425548056	424.1034773	0.590119755
424.6703513	1.423743431	424.6703513	0.58886415
425.2967543	1.402719993	425.2967543	0.57275194
425.8948863	1.421044107	425.8948863	0.583314084
427.5955063	1.430687876	427.5955063	0.590059789
427.8789423	1.43041751	427.8789423	0.589711731
429.1034773	1.430256194	429.1034773	0.588715692
429.6703513	1.430406342	429.6703513	0.588372394
430.2967543	1.430582522	430.2967543	0.587998531
430.8948863	1.430698695	430.8948863	0.587614451
432.5955063	1.429426009	432.5955063	0.585740985
432.8789423	1.428362008	432.8789423	0.585020742
434.1034773	1.427512285	434.1034773	0.583629789
434.6703513	1.430476102	434.6703513	0.584614786
435.2967543	1.432279237	435.2967543	0.585016551
435.8948863	1.43329503	435.8948863	0.585057498
437.5955063	1.435174663	437.5955063	0.584672904
437.8789423	1.435436478	437.8789423	0.584582801
439.1034773	1.436469573	439.1034773	0.584145391
439.6703513	1.436884832	439.6703513	0.583913459
440.2967543	1.437286749	440.2967543	0.583631634
440.8948863	1.437625057	440.8948863	0.583342374
442.5955063	1.438816006	442.5955063	0.582613513

Final State: j’=1		Final State: j’=0	
E(cm⁻¹)	σ (Å²)	E(cm⁻¹)	σ (Å²)
442.8789423	1.439096195	442.8789423	0.582525906
444.1034773	1.440474731	444.1034773	0.582215534
444.6703513	1.441146709	444.6703513	0.58208452
445.2967543	1.441887315	445.2967543	0.581937741
445.8948863	1.442586526	445.8948863	0.581793091
447.5955063	1.444523572	447.5955063	0.581354747
447.8789423	1.444839681	447.8789423	0.581278107
449.1034773	1.446189052	449.1034773	0.580937843
449.6703513	1.446808279	449.6703513	0.580776706
450.2967543	1.44749253	450.2967543	0.580597542
450.8948863	1.448149177	450.8948863	0.580426615
452.5955063	1.450056646	452.5955063	0.579950005
452.8789423	1.450382007	452.8789423	0.579872514
454.1034773	1.451812467	454.1034773	0.579544238
454.6703513	1.452487054	454.6703513	0.579395384
455.2967543	1.453240257	455.2967543	0.579232704
455.8948863	1.453966129	455.8948863	0.579078785
457.5955063	1.456058801	457.5955063	0.578646301
457.8789423	1.456411135	457.8789423	0.57857474
459.1034773	1.457943621	459.1034773	0.578266764
459.6703513	1.45865858	459.6703513	0.578124782
460.2967543	1.459452728	460.2967543	0.577968314
460.8948863	1.460215161	460.8948863	0.577819231
462.5955063	1.462406349	462.5955063	0.577398551
462.8789423	1.462775087	462.8789423	0.57732886
464.1034773	1.464380329	464.1034773	0.577029365
464.6703513	1.46513026	464.6703513	0.576891624
465.2967543	1.465964003	465.2967543	0.576740101
465.8948863	1.466765079	465.8948863	0.576596078
467.5955063	1.469068873	467.5955063	0.576189997
467.8789423	1.46945655	467.8789423	0.576122786
469.1034773	1.471143336	469.1034773	0.57583386
469.6703513	1.471930666	469.6703513	0.575700869
470.2967543	1.472805362	470.2967543	0.575554433
470.8948863	1.47364514	470.8948863	0.575415117
472.5955063	1.476056872	472.5955063	0.575021585
472.8789423	1.476462268	472.8789423	0.574956355
474.1034773	1.478224945	474.1034773	0.574675681
474.6703513	1.479047117	474.6703513	0.574546373
475.2967543	1.479960171	475.2967543	0.574403932
475.8948863	1.48083647	475.8948863	0.574268363
477.5955063	1.483351796	477.5955063	0.573885242
477.8789423	1.483774443	477.8789423	0.573821727
479.1034773	1.485611667	479.1034773	0.573548411
479.6703513	1.48646838	479.6703513	0.573422491

Final State: j’=1		Final State: j’=0	
E(cm⁻¹)	σ (Å²)	E(cm⁻¹)	σ (Å²)
480.2967543	1.487419613	480.2967543	0.573283793
480.8948863	1.488332393	480.8948863	0.573151797
482.5955063	1.490951572	482.5955063	0.572778872
482.8789423	1.491391557	482.8789423	0.572717065
484.1034773	1.493303809	484.1034773	0.572451202
484.6703513	1.494195327	484.6703513	0.572328779
485.2967543	1.495185115	485.2967543	0.572193991
485.8948863	1.496134805	485.8948863	0.57206578
487.5955063	1.498859609	487.5955063	0.571703999
487.8789423	1.499317315	487.8789423	0.571644119
489.1034773	1.501306662	489.1034773	0.571386851
489.6703513	1.502234208	489.6703513	0.57126859
490.2967543	1.503264094	490.2967543	0.571138562
490.8948863	1.504252386	490.8948863	0.571015066
492.5955063	1.507088971	492.5955063	0.570667807
492.8789423	1.507565648	492.8789423	0.570610533
494.1034773	1.509638287	494.1034773	0.570365268
494.6703513	1.510605215	494.6703513	0.57025302
495.2967543	1.511679322	495.2967543	0.570130026
495.8948863	1.512710603	495.8948863	0.570013677
497.5955063	1.515674277	497.5955063	0.569689453
497.8789423	1.516172952	497.8789423	0.569636478
499.1034773	1.518343901	499.1034773	0.569411643
499.6703513	1.5193584	499.6703513	0.569310024
500.2967543	1.520486849	500.2967543	0.569199785
500.8948863	1.521572	500.8948863	0.569096743
502.5955063	1.52470196	502.5955063	0.56881791
502.8789423	1.525230654	502.8789423	0.568773828
504.1034773	1.527541125	504.1034773	0.568593154
504.6703513	1.528626713	504.6703513	0.568515796
505.2967543	1.529839716	505.2967543	0.568435913
505.8948863	1.531012634	505.8948863	0.568366051
507.5955063	1.534444561	507.5955063	0.568213338
507.8789423	1.535033873	507.8789423	0.568196624
509.1034773	1.537658644	509.1034773	0.568167353
509.6703513	1.538929998	509.6703513	0.568186243
510.2967543	1.54039363	510.2967543	0.568243299
510.8948863	1.541870656	510.8948863	0.568349757
512.5955063	1.546971013	512.5955063	0.56938601
512.8789423	1.548080677	512.8789423	0.569875959
514.1034773	1.542845857	514.1034773	0.571101134
514.6703513	1.540591445	514.6703513	0.566775553
515.2967543	1.548640812	515.2967543	0.568604379
515.8948863	1.542577196	515.8948863	0.568191286
517.5955063	1.553209355	517.5955063	0.570214996

Final State: j’=1		Final State: j’=0	
E(cm⁻¹)	σ (Å²)	E(cm⁻¹)	σ (Å²)
517.8789423	1.550896283	517.8789423	0.569832508
519.1034773	1.545552246	519.1034773	0.565030163
519.6703513	1.553075854	519.6703513	0.566722851
520.2967543	1.552702121	520.2967543	0.564268002
520.8948863	1.559326916	520.8948863	0.567208727
522.5955063	1.557948959	522.5955063	0.56652489
522.8789423	1.545359755	522.8789423	0.564910122
524.1034773	1.561897148	524.1034773	0.563424693
524.6703513	1.565938407	524.6703513	0.565385281
525.2967543	1.56241997	525.2967543	0.567449607
525.8948863	1.559118609	525.8948863	0.563644961
527.5955063	1.563763897	527.5955063	0.559949562
527.8789423	1.566185672	527.8789423	0.560679068
529.1034773	1.573546627	529.1034773	0.563176176
529.6703513	1.577401188	529.6703513	0.565721693
530.2967543	1.564948806	530.2967543	0.562982524
530.8948863	1.570066837	530.8948863	0.561837321
532.5955063	1.57445424	532.5955063	0.558801625
532.8789423	1.576224455	532.8789423	0.559300577
534.1034773	1.582310515	534.1034773	0.560970336
534.6703513	1.585432045	534.6703513	0.562151348
535.2967543	1.588927696	535.2967543	0.565434458
535.8948863	1.576686701	535.8948863	0.561282161
537.5955063	1.581385122	537.5955063	0.556054929
537.8789423	1.584476133	537.8789423	0.557026903
539.1034773	1.591029255	539.1034773	0.558731918
539.6703513	1.593456717	539.6703513	0.559288613
540.2967543	1.59627749	540.2967543	0.560034923
540.8948863	1.599565661	540.8948863	0.561240725
542.5955063	1.597866645	542.5955063	0.559415063
542.8789423	1.585480578	542.8789423	0.559219418
544.1034773	1.600276511	544.1034773	0.556906602
544.6703513	1.603694976	544.6703513	0.557935931
545.2967543	1.606883581	545.2967543	0.558851534
545.8948863	1.609955461	545.8948863	0.559849553
547.5955063	1.609461169	547.5955063	0.564451047
547.8789423	1.608784799	547.8789423	0.562623043
549.1034773	1.59844984	549.1034773	0.560331024
549.6703513	1.59596941	549.6703513	0.555724644
550.2967543	1.608510667	550.2967543	0.556361454
550.8948863	1.603359004	550.8948863	0.560157197
552.5955063	1.621806186	552.5955063	0.558996183
552.8789423	1.62220019	552.8789423	0.562453293
554.1034773	1.611997241	554.1034773	0.55199576
554.6703513	1.622018757	554.6703513	0.554959924

Final State: j'=1		Final State: j'=0	
E(cm⁻¹)	σ (Å²)	E(cm⁻¹)	σ (Å²)
555.2967543	1.630549116	555.2967543	0.559000127
555.8948863	1.633954568	555.8948863	0.567984878
557.5955063	1.616214468	557.5955063	0.547848831
557.8789423	1.622882387	557.8789423	0.550312098
559.1034773	1.637919989	559.1034773	0.558368509
559.6703513	1.620474531	559.6703513	0.560106057
560.2967543	1.615062609	560.2967543	0.545976295
560.8948863	1.629623327	560.8948863	0.549559192
562.5955063	1.64561664	562.5955063	0.555498323
562.8789423	1.649083202	562.8789423	0.557507742
564.1034773	1.623349056	564.1034773	0.547426655
564.6703513	1.635920168	564.6703513	0.551998212
565.2967543	1.640765723	565.2967543	0.551618465
565.8948863	1.642047122	565.8948863	0.554457175
567.5955063	1.659736502	567.5955063	0.560728103
567.8789423	1.645387597	567.8789423	0.56336651
569.1034773	1.65067604	569.1034773	0.549316321
569.6703513	1.638164374	569.6703513	0.553988903
570.2967543	1.647470125	570.2967543	0.546789299
570.8948863	1.660493139	570.8948863	0.551417489
572.5955063	1.647237157	572.5955063	0.554347156
572.8789423	1.642587317	572.8789423	0.549275798
574.1034773	1.650797138	574.1034773	0.543950851
574.6703513	1.661611554	574.6703513	0.550070389
575.2967543	1.662368672	575.2967543	0.549578965
575.8948863	1.654559387	575.8948863	0.548002044
577.5955063	1.652126013	577.5955063	0.548608799
577.8789423	1.652408013	577.8789423	0.544539666
579.1034773	1.664026793	579.1034773	0.547691741
579.6703513	1.66658674	579.6703513	0.548416278
580.2967543	1.669318444	580.2967543	0.549233276
580.8948863	1.671969754	580.8948863	0.549944684
582.5955063	1.664999448	582.5955063	0.54494515
582.8789423	1.667137993	582.8789423	0.54536034
584.1034773	1.671783874	584.1034773	0.545973855
584.6703513	1.673361117	584.6703513	0.546045435
585.2967543	1.674983721	585.2967543	0.546106212
585.8948863	1.676244142	585.8948863	0.546115055
587.5955063	1.678632856	587.5955063	0.545208786
587.8789423	1.6793182	587.8789423	0.54513773
589.1034773	1.683292002	589.1034773	0.545356142
589.6703513	1.681669473	589.6703513	0.544142114
590.2967543	1.681087801	590.2967543	0.543163007
590.8948863	1.684069582	590.8948863	0.543520929
592.5955063	1.687943883	592.5955063	0.543196842

Final State: j'=1		Final State: j'=0	
E(cm⁻¹)	σ (Å²)	E(cm⁻¹)	σ (Å²)
592.8789423	1.688522437	592.8789423	0.543107472
594.1034773	1.691007436	594.1034773	0.542707238
594.6703513	1.692158441	594.6703513	0.542520241
595.2967543	1.693434785	595.2967543	0.542314865
595.8948863	1.694662794	595.8948863	0.542122087
597.5955063	1.69789028	597.5955063	0.541468362
597.8789423	1.698216963	597.8789423	0.541278076
599.1034773	1.700382722	599.1034773	0.540726947
599.6703513	1.701546502	599.6703513	0.54053426
600.2967543	1.702817356	600.2967543	0.540316434
600.8948863	1.704018104	600.8948863	0.540103349
602.5955063	1.707399153	602.5955063	0.539481305
602.8789423	1.707960351	602.8789423	0.539376123
604.1034773	1.710379074	604.1034773	0.538917126
604.6703513	1.711492763	604.6703513	0.538700985
605.2967543	1.712714595	605.2967543	0.538457929
605.8948863	1.713870841	605.8948863	0.538221396
607.5955063	1.717138089	607.5955063	0.537540439
607.8789423	1.717685015	607.8789423	0.537427568
609.1034773	1.720059334	609.1034773	0.53694249
609.6703513	1.721163049	609.6703513	0.53671856
610.2967543	1.722384438	610.2967543	0.536471108
610.8948863	1.723552018	610.8948863	0.53623452
612.5955063	1.726874468	612.5955063	0.535558993
612.8789423	1.727428241	612.8789423	0.535445876
614.1034773	1.72981963	614.1034773	0.534955118
614.6703513	1.730925825	614.6703513	0.534726764
615.2967543	1.732147482	615.2967543	0.534473594
615.8948863	1.733313377	615.8948863	0.534231081
617.5955063	1.736626178	617.5955063	0.533538026
617.8789423	1.737178178	617.8789423	0.533422072
619.1034773	1.739563073	619.1034773	0.532919794
619.6703513	1.74066724	619.6703513	0.532686571
620.2967543	1.741887482	620.2967543	0.532428342
620.8948863	1.743052773	620.8948863	0.532181256
622.5955063	1.746366298	622.5955063	0.53147596
622.8789423	1.746918569	622.8789423	0.531357997
624.1034773	1.749304449	624.1034773	0.530846993
624.6703513	1.750408832	624.6703513	0.530609668
625.2967543	1.751629064	625.2967543	0.530346856
625.8948863	1.752794086	625.8948863	0.53009535
627.5955063	1.756105608	627.5955063	0.529377342
627.8789423	1.756657395	627.8789423	0.529257261
629.1034773	1.759040804	629.1034773	0.528737112
629.6703513	1.760143899	629.6703513	0.528495589

Final State: j’=1		Final State: j’=0	
E(cm⁻¹)	σ (Å²)	E(cm⁻¹)	σ (Å²)
630.2967543	1.761362637	630.2967543	0.52822817
630.8948863	1.762526183	630.8948863	0.527972303
632.5955063	1.765833343	632.5955063	0.527242087
632.8789423	1.766384386	632.8789423	0.527119996
634.1034773	1.768764501	634.1034773	0.526591251
634.6703513	1.769866022	634.6703513	0.52634579
635.2967543	1.771082984	635.2967543	0.526074044
635.8948863	1.772244776	635.8948863	0.525814071
637.5955063	1.775546642	637.5955063	0.525072298
637.8789423	1.776096747	637.8789423	0.524948297
639.1034773	1.778472661	639.1034773	0.524411365
639.6703513	1.77957213	639.6703513	0.524162143
640.2967543	1.78078675	640.2967543	0.523886266
640.8948863	1.781946239	640.8948863	0.523622371
642.5955063	1.785241186	642.5955063	0.522869582
642.8789423	1.785790087	642.8789423	0.522743767
644.1034773	1.788160622	644.1034773	0.522199052
644.6703513	1.789257517	644.6703513	0.521946264
645.2967543	1.790469226	645.2967543	0.521666473
645.8948863	1.791625873	645.8948863	0.521398869
647.5955063	1.794912414	647.5955063	0.520635677
647.8789423	1.795459865	647.8789423	0.520508146
649.1034773	1.797823993	649.1034773	0.519956096
649.6703513	1.79891784	649.6703513	0.519699948
650.2967543	1.80012612	650.2967543	0.519416475
650.8948863	1.801279429	650.8948863	0.51914538
652.5955063	1.804556151	652.5955063	0.518372403
652.8789423	1.805101922	652.8789423	0.518243264
654.1034773	1.80745863	654.1034773	0.517684325
654.6703513	1.80854896	654.6703513	0.517425027
655.2967543	1.809753288	655.2967543	0.5171381
655.8948863	1.810902769	655.8948863	0.516863733
657.5955063	1.814168285	657.5955063	0.516081605
657.8789423	1.814712145	657.8789423	0.51595096
659.1034773	1.817060448	659.1034773	0.515385593
659.6703513	1.818146803	659.6703513	0.515123356
660.2967543	1.819346689	660.2967543	0.51483321
660.8948863	1.820491871	660.8948863	0.514555799
662.5955063	1.823744875	662.5955063	0.51376516
662.8789423	1.824286605	662.8789423	0.513633117
664.1034773	1.826625588	664.1034773	0.513061787
664.6703513	1.827707557	664.6703513	0.512796827
665.2967543	1.828902544	665.2967543	0.512503702
665.8948863	1.830043	665.8948863	0.512223471
667.5955063	1.833282309	667.5955063	0.511424968

Final State: j’=1		Final State: j’=0	
E(cm⁻¹)	σ (Å²)	E(cm⁻¹)	σ (Å²)
667.8789423	1.833821722	667.8789423	0.511291636
669.1034773	1.836150577	669.1034773	0.5107148
669.6703513	1.837227801	669.6703513	0.510447332
670.2967543	1.838417499	670.2967543	0.510151458
670.8948863	1.839552863	670.8948863	0.509868633
672.5955063	1.842777507	672.5955063	0.50906289
672.8789423	1.843314448	672.8789423	0.508928372
674.1034773	1.845632551	674.1034773	0.508346476
674.6703513	1.846704756	674.6703513	0.508076699
675.2967543	1.847888881	675.2967543	0.507778304
675.8948863	1.849018905	675.8948863	0.507493095
677.5955063	1.852228266	677.5955063	0.506680702
677.8789423	1.852762648	677.8789423	0.506545094
679.1034773	1.855069688	679.1034773	0.505958546
679.6703513	1.85613677	679.6703513	0.505686648
680.2967543	1.857315245	680.2967543	0.505385929
680.8948863	1.858439875	680.8948863	0.505098522
682.5955063	1.861634029	682.5955063	0.50427999
682.8789423	1.862165903	682.8789423	0.504143372
684.1034773	1.864462214	684.1034773	0.503552509
684.6703513	1.865524401	684.6703513	0.50327863
685.2967543	1.866697547	685.2967543	0.502975741
685.8948863	1.867817173	685.8948863	0.502686271
687.5955063	1.870997669	687.5955063	0.501861924
687.8789423	1.871527366	687.8789423	0.501724341
689.1034773	1.873814714	689.1034773	0.501129309
689.6703513	1.874873037	689.6703513	0.5008535
690.2967543	1.876042152	690.2967543	0.500548466
690.8948863	1.87715821	690.8948863	0.500256935
692.5955063	1.880330456	692.5955063	0.499426608
692.8789423	1.880859112	692.8789423	0.499288004
694.1034773	1.883143407	694.1034773	0.498688435
694.6703513	1.884201274	694.6703513	0.498410436
695.2967543	1.885370835	695.2967543	0.49810265
695.8948863	1.886488199	695.8948863	0.49780887
697.5955063	1.889671849	697.5955063	0.496970652
697.8789423	1.890203846	697.8789423	0.496830578
699.1034773	1.892509573	699.1034773	0.496223896
699.6703513	1.893582533	699.6703513	0.495942044
700.2967543	1.89477419	700.2967543	0.49562966
700.8948863	1.895920159	700.8948863	0.495330245
702.5955063	1.899261218	702.5955063	0.494469396
702.8789423	1.899840713	702.8789423	0.494323718
704.1034773	1.902582204	704.1034773	0.49367894
704.6703513	1.904366427	704.6703513	0.493373582

Final State: j'=1		Final State: j'=0	
E(cm^{-1})	σ ($Å^2$)	E(cm^{-1})	σ ($Å^2$)
705.2967543	1.907192537	705.2967543	0.493309809
705.8948863	1.907822303	705.8948863	0.493399186
707.5955063	1.909500219	707.5955063	0.492647385
707.8789423	1.908487273	707.8789423	0.492274613
709.1034773	1.913524891	709.1034773	0.491457612
709.6703513	1.914982578	709.6703513	0.491580986
710.2967543	1.913523679	710.2967543	0.491170157
710.8948863	1.91414919	710.8948863	0.490768174
712.5955063	1.919067233	712.5955063	0.489757269
712.8789423	1.921406126	712.8789423	0.48998912
714.1034773	1.921363489	714.1034773	0.489094171
714.6703513	1.92001008	714.6703513	0.489208449
715.2967543	1.921015658	715.2967543	0.488491841
715.8948863	1.922803291	715.8948863	0.488110369
717.5955063	1.92704529	717.5955063	0.487691444
717.8789423	1.927492045	717.8789423	0.487549318
719.1034773	1.927052096	719.1034773	0.486716978
719.6703513	1.928545613	719.6703513	0.486338867
720.2967543	1.930153564	720.2967543	0.48597213
720.8948863	1.932026941	720.8948863	0.485643294
722.5955063	1.936824236	722.5955063	0.485101891
722.8789423	1.93620411	722.8789423	0.485771722
724.1034773	1.935786114	724.1034773	0.484223082
724.6703513	1.937145097	724.6703513	0.483884036
725.2967543	1.938594174	725.2967543	0.483534393
725.8948863	1.940069925	725.8948863	0.483209274
727.5955063	1.945031032	727.5955063	0.483120484
727.8789423	1.943980886	727.8789423	0.482584089
729.1034773	1.943906841	729.1034773	0.481867102
729.6703513	1.945454325	729.6703513	0.48146456
730.2967543	1.946880352	730.2967543	0.481107298
730.8948863	1.948181804	730.8948863	0.480783044
732.5955063	1.952437167	732.5955063	0.479903402
732.8789423	1.953804622	732.8789423	0.479825125
734.1034773	1.956368271	734.1034773	0.479537778
734.6703513	1.956062612	734.6703513	0.479515536
735.2967543	1.95507369	735.2967543	0.478713713
735.8948863	1.956705621	735.8948863	0.478324634
737.5955063	1.960955019	737.5955063	0.477363795
737.8789423	1.96183925	737.8789423	0.477200063
739.1034773	1.968055548	739.1034773	0.476980869
739.6703513	1.966661054	739.6703513	0.476775713
740.2967543	1.971775657	740.2967543	0.477248356
740.8948863	1.971298236	740.8948863	0.476763752
742.5955063	1.97346452	742.5955063	0.476004927

Final State: j’=1		Final State: j’=0	
E(cm⁻¹)	σ (Å²)	E(cm⁻¹)	σ (Å²)
742.8789423	1.970059755	742.8789423	0.475737396
744.1034773	1.972394971	744.1034773	0.474367092
744.6703513	1.978830415	744.6703513	0.474730652
745.2967543	1.973676466	745.2967543	0.474877134
745.8948863	1.972729881	745.8948863	0.473777909
747.5955063	1.987365347	747.5955063	0.474444787
747.8789423	1.986149853	747.8789423	0.474794196
749.1034773	1.976223453	749.1034773	0.472397063
749.6703513	1.978621806	749.6703513	0.471838715
750.2967543	1.981248887	750.2967543	0.471415879
750.8948863	1.988474841	750.8948863	0.471541235
752.5955063	1.982933637	752.5955063	0.47052527
752.8789423	1.98378463	752.8789423	0.470327729
754.1034773	1.987767104	754.1034773	0.469577069
754.6703513	1.992180961	754.6703513	0.469392958
755.2967543	1.994831616	755.2967543	0.471035949
755.8948863	1.989736806	755.8948863	0.469512463
757.5955063	1.995193796	757.5955063	0.468224161
757.8789423	1.994851649	757.8789423	0.468050397
759.1034773	2.000601144	759.1034773	0.467514504
759.6703513	2.002325094	759.6703513	0.469111918
760.2967543	1.994345473	760.2967543	0.467112502
760.8948863	2.000732969	760.8948863	0.466441266
762.5955063	2.001631034	762.5955063	0.465590099
762.8789423	2.009053409	762.8789423	0.466008918
764.1034773	2.010419476	764.1034773	0.467119622
764.6703513	2.003671301	764.6703513	0.465361423
765.2967543	2.00764074	765.2967543	0.465479981
765.8948863	2.003453779	765.8948863	0.464364569
767.5955063	2.011942535	767.5955063	0.464694968
767.8789423	2.009586237	767.8789423	0.463558741
769.1034773	2.01476756	769.1034773	0.464153012
769.6703513	2.01269367	769.6703513	0.462992576
770.2967543	2.013892546	770.2967543	0.462596083
770.8948863	2.015213328	770.8948863	0.462306252
772.5955063	2.019346198	772.5955063	0.461814861
772.8789423	2.020878296	772.8789423	0.461774569
774.1034773	2.019948717	774.1034773	0.461070159
774.6703513	2.020977884	774.6703513	0.460742777
775.2967543	2.022094726	775.2967543	0.460455864
775.8948863	2.023121941	775.8948863	0.460194016
777.5955063	2.026083511	777.5955063	0.459518786
777.8789423	2.026548185	777.8789423	0.459418348
779.1034773	2.028280685	779.1034773	0.458888617
779.6703513	2.029131556	779.6703513	0.458634391

Final State: j’=1		Final State: j’=0	
E(cm⁻¹)	σ (Å²)	E(cm⁻¹)	σ (Å²)
780.2967543	2.03014557	780.2967543	0.458371502
780.8948863	2.031558865	780.8948863	0.45836803
782.5955063	2.033333074	782.5955063	0.457348217
782.8789423	2.033783242	782.8789423	0.457229693
784.1034773	2.035649114	784.1034773	0.456713792
784.6703513	2.036494662	784.6703513	0.456473267
785.2967543	2.037424121	785.2967543	0.456207334
785.8948863	2.038309135	785.8948863	0.455953858
787.5955063	2.04082353	787.5955063	0.455239876
787.8789423	2.041244977	787.8789423	0.4551231
789.1034773	2.043077204	789.1034773	0.454639418
789.6703513	2.043859059	789.6703513	0.454395971
790.2967543	2.044719818	790.2967543	0.45411635
790.8948863	2.04556722	790.8948863	0.453857914
792.5955063	2.047997835	792.5955063	0.453142516
792.8789423	2.048402117	792.8789423	0.453024406
794.1034773	2.050143854	794.1034773	0.452516326
794.6703513	2.050947472	794.6703513	0.45228226
795.2967543	2.051833564	795.2967543	0.452024487
795.8948863	2.052677743	795.8948863	0.451779226
797.5955063	2.055064049	797.5955063	0.45108526
797.8789423	2.055459155	797.8789423	0.450969734
799.1034773	2.057158046	799.1034773	0.45047071
799.6703513	2.057940968	799.6703513	0.450240113
800.2967543	2.058803981	800.2967543	0.449985868
800.8948863	2.059626039	800.8948863	0.449743738
802.5955063	2.061952465	802.5955063	0.449058877
802.8789423	2.062338591	802.8789423	0.448945249
804.1034773	2.064001335	804.1034773	0.448456011
804.6703513	2.064768005	804.6703513	0.448230431
805.2967543	2.065612876	805.2967543	0.447981813
805.8948863	2.066417318	805.8948863	0.44774504
807.5955063	2.06869197	807.5955063	0.447075064
807.8789423	2.069069251	807.8789423	0.446963855
809.1034773	2.070693188	809.1034773	0.446484897
809.6703513	2.071441651	809.6703513	0.446264004
810.2967543	2.072266287	810.2967543	0.446020534
810.8948863	2.073051331	810.8948863	0.445788669
812.5955063	2.075270744	812.5955063	0.445132764
812.8789423	2.075638829	812.8789423	0.445023934
814.1034773	2.077223091	814.1034773	0.444555367
814.6703513	2.077953199	814.6703513	0.444339345
815.2967543	2.07875754	815.2967543	0.444101299
815.8948863	2.079523199	815.8948863	0.443874646
817.5955063	2.081687308	817.5955063	0.443233703

Final State: j’=1		Final State: j’=0	
E(cm⁻¹)	σ (Å²)	E(cm⁻¹)	σ (Å²)
817.8789423	2.082046145	817.8789423	0.443127381
819.1034773	2.083590334	819.1034773	0.442669694
819.6703513	2.084301819	819.6703513	0.442458731
820.2967543	2.085085543	820.2967543	0.442226288
820.8948863	2.085831458	820.8948863	0.442005003
822.5955063	2.087939233	822.5955063	0.441379401
822.8789423	2.088288646	822.8789423	0.441275648
824.1034773	2.089792048	824.1034773	0.440829115
824.6703513	2.09048461	824.6703513	0.44062334
825.2967543	2.091247387	825.2967543	0.44039665
825.8948863	2.091973277	825.8948863	0.440180875
827.5955063	2.094023933	827.5955063	0.439571049
827.8789423	2.094363807	827.8789423	0.439469941
829.1034773	2.095825912	829.1034773	0.439034872
829.6703513	2.096499306	829.6703513	0.438834432
830.2967543	2.097240889	830.2967543	0.438613655
830.8948863	2.097946501	830.8948863	0.438403542
832.5955063	2.099939356	832.5955063	0.437809904
832.8789423	2.100269574	832.8789423	0.437711508
834.1034773	2.101689891	834.1034773	0.43728819
834.6703513	2.102343911	834.6703513	0.437093212
835.2967543	2.103064044	835.2967543	0.436878489
835.8948863	2.103749145	835.8948863	0.436674172
837.5955063	2.105683563	837.5955063	0.436097095
837.8789423	2.106004021	837.8789423	0.436001471
839.1034773	2.107382106	839.1034773	0.435590168
839.6703513	2.108016543	839.6703513	0.435400773
840.2967543	2.108715009	840.2967543	0.435192234
840.8948863	2.109379403	840.8948863	0.434993836
842.5955063	2.111254809	842.5955063	0.434433668
842.8789423	2.111565417	842.8789423	0.434340872
844.1034773	2.112900885	844.1034773	0.433941826
844.6703513	2.113515565	844.6703513	0.433758126
845.2967543	2.114192172	845.2967543	0.43355589
845.8948863	2.114835674	845.8948863	0.433363527
847.5955063	2.116651579	847.5955063	0.432820584
847.8789423	2.116952253	847.8789423	0.432730667
849.1034773	2.118244759	849.1034773	0.432344101
849.6703513	2.118839518	849.6703513	0.432166191
850.2967543	2.119494103	850.2967543	0.431970371
850.8948863	2.120116562	850.8948863	0.431784145
852.5955063	2.121872523	852.5955063	0.431258711
852.8789423	2.122163195	852.8789423	0.431171724
854.1034773	2.123412446	854.1034773	0.430797832
854.6703513	2.123987159	854.6703513	0.430625807

Final State: j’=1		Final State: j’=0	
E(cm⁻¹)	σ (Å²)	E(cm⁻¹)	σ (Å²)
855.2967543	2.124619574	855.2967543	0.430436499
855.8948863	2.125220846	855.8948863	0.430256501
857.5955063	2.126916502	857.5955063	0.429748838
857.8789423	2.127197108	857.8789423	0.429664818
859.1034773	2.128402848	859.1034773	0.429303781
859.6703513	2.128957401	859.6703513	0.429137718
860.2967543	2.129567528	860.2967543	0.42895501
860.8948863	2.130147498	860.8948863	0.428781326
862.5955063	2.131782527	862.5955063	0.428291654
862.8789423	2.132053022	862.8789423	0.428210641
864.1034773	2.133215037	864.1034773	0.427862611
864.6703513	2.133749336	864.6703513	0.427702583
865.2967543	2.134337063	865.2967543	0.42752655
865.8948863	2.134895641	865.8948863	0.427359246
867.5955063	2.136469783	867.5955063	0.426887765
867.8789423	2.136730124	867.8789423	0.42680979
869.1034773	2.137848242	869.1034773	0.426474901
869.6703513	2.138362206	869.6703513	0.426320967
870.2967543	2.138927458	870.2967543	0.426151675
870.8948863	2.139464563	870.8948863	0.425990816
872.5955063	2.14097761	872.5955063	0.42553769
872.8789423	2.141227761	872.8789423	0.425462778
874.1034773	2.142301841	874.1034773	0.425141145
874.6703513	2.142795408	874.6703513	0.424993354
875.2967543	2.143338113	875.2967543	0.424830856
875.8948863	2.143853683	875.8948863	0.424676492
877.5955063	2.145305469	877.5955063	0.424241858
877.8789423	2.145545404	877.8789423	0.424170032
879.1034773	2.146575337	879.1034773	0.423861741
879.6703513	2.147048458	879.6703513	0.423720135
880.2967543	2.14756856	880.2967543	0.423564474
880.8948863	2.148062548	880.8948863	0.423416642
882.5955063	2.149452936	882.5955063	0.423000606
882.8789423	2.149682633	882.8789423	0.422931882
884.1034773	2.150668323	884.1034773	0.422637005
884.6703513	2.151120964	884.6703513	0.422501642
885.2967543	2.151618416	885.2967543	0.422352821
885.8948863	2.152090775	885.8948863	0.422211552
887.5955063	2.153419635	887.5955063	0.421814188
887.8789423	2.153639079	887.8789423	0.421748577
889.1034773	2.154580435	889.1034773	0.42146716
889.6703513	2.155012538	889.6703513	0.421338
890.2967543	2.155487309	890.2967543	0.421196099
890.8948863	2.155937995	890.8948863	0.42106141
892.5955063	2.157205173	892.5955063	0.420682766

Final State: j’=1		Final State: j’=0	
E(cm⁻¹)	σ (Å²)	E(cm⁻¹)	σ (Å²)
892.8789423	2.157414328	892.8789423	0.420620276
894.1034773	2.15831123	894.1034773	0.420352343
894.6703513	2.158722739	894.6703513	0.420229426
895.2967543	2.159174737	895.2967543	0.420094427
895.8948863	2.159603665	895.8948863	0.419966326
897.5955063	2.160808946	897.5955063	0.419606413
897.8789423	2.16100777	897.8789423	0.419547046
899.1034773	2.161859998	899.1034773	0.4192926
899.6703513	2.1622508	899.6703513	0.419175927
900.2967543	2.162679898	900.2967543	0.419047823
900.8948863	2.163086937	900.8948863	0.418926309
902.5955063	2.164229828	902.5955063	0.418585115
902.8789423	2.164418232	902.8789423	0.418528868
904.1034773	2.165225347	904.1034773	0.418287891
904.6703513	2.165595204	904.6703513	0.418177451
905.2967543	2.166001112	905.2967543	0.418056234
905.8948863	2.166385955	905.8948863	0.417941292
907.5955063	2.167465401	907.5955063	0.417618773
907.8789423	2.167643175	907.8789423	0.417565638
909.1034773	2.168404138	909.1034773	0.417338092
909.6703513	2.168752483	909.6703513	0.417233868
910.2967543	2.169134498	910.2967543	0.417119517
910.8948863	2.169496384	910.8948863	0.417011128
912.5955063	2.170509624	912.5955063	0.416707222
912.8789423	2.170676201	912.8789423	0.416657186
914.1034773	2.171388065	914.1034773	0.416443033
914.6703513	2.171713217	914.6703513	0.416345001
915.2967543	2.172069163	915.2967543	0.416237494
915.8948863	2.172405649	915.8948863	0.416135639
917.5955063	2.173342836	917.5955063	0.415850331
917.8789423	2.173495982	917.8789423	0.415803398
919.1034773	2.174145991	919.1034773	0.415602686
919.6703513	2.174439571	919.6703513	0.415510916
920.2967543	2.174757313	920.2967543	0.41541037
920.8948863	2.175052578	920.8948863	0.415315272
922.5955063	2.17581267	922.5955063	0.415050335
922.8789423	2.175914612	922.8789423	0.415007399
924.1034773	2.176343774	924.1034773	0.414853897
924.6703513	2.177208248	924.6703513	0.414781791
925.2967543	2.177667207	925.2967543	0.414638096
925.8948863	2.17740886	925.8948863	0.414570214
927.5955063	2.178483385	927.5955063	0.414295553
927.8789423	2.17831601	927.8789423	0.414266885
929.1034773	2.179958817	929.1034773	0.414086829
929.6703513	2.17992563	929.6703513	0.414034928

Final State: j’=1		Final State: j’=0	
E(cm⁻¹)	σ (Å²)	E(cm⁻¹)	σ (Å²)
930.2967543	2.180411606	930.2967543	0.413922612
930.8948863	2.180356935	930.8948863	0.413823238
932.5955063	2.181557773	932.5955063	0.413613516
932.8789423	2.181212926	932.8789423	0.413575921
934.1034773	2.182098313	934.1034773	0.413393437
934.6703513	2.182108635	934.6703513	0.413315567
935.2967543	2.182044672	935.2967543	0.413224851
935.8948863	2.183098896	935.8948863	0.413184774
937.5955063	2.184291996	937.5955063	0.412979476
937.8789423	2.184015308	937.8789423	0.41292126
939.1034773	2.184087586	939.1034773	0.41276218
939.6703513	2.184138937	939.6703513	0.41268521
940.2967543	2.184201392	940.2967543	0.412602678
940.8948863	2.185435755	940.8948863	0.412591022
942.5955063	2.185994394	942.5955063	0.412372955
942.8789423	2.185951603	942.8789423	0.412337954
944.1034773	2.186137296	944.1034773	0.412191413
944.6703513	2.186241083	944.6703513	0.412122286
945.2967543	2.186292982	945.2967543	0.412037484
945.8948863	2.186595027	945.8948863	0.411945736
947.5955063	2.188009592	947.5955063	0.411861213
947.8789423	2.188089508	947.8789423	0.411817254
949.1034773	2.188023137	949.1034773	0.411678988
949.6703513	2.188132418	949.6703513	0.411618011
950.2967543	2.188256211	950.2967543	0.411550341
950.8948863	2.188357233	950.8948863	0.411483127
952.5955063	2.189569807	952.5955063	0.411417947
952.8789423	2.189032938	952.8789423	0.411350728
954.1034773	2.189797564	954.1034773	0.411229496
954.6703513	2.189718805	954.6703513	0.411170437
955.2967543	2.18975604	955.2967543	0.411110002
955.8948863	2.189795463	955.8948863	0.411054557
957.5955063	2.189631808	957.5955063	0.410952683
957.8789423	2.189791678	957.8789423	0.410956207
959.1034773	2.190780111	959.1034773	0.410825537
959.6703513	2.191632748	959.6703513	0.410839608
960.2967543	2.191927631	960.2967543	0.410754274
960.8948863	2.191022141	960.8948863	0.410710032
962.5955063	2.192157489	962.5955063	0.410520244
962.8789423	2.191874454	962.8789423	0.410497859
964.1034773	2.19386666	964.1034773	0.410515456
964.6703513	2.193286839	964.6703513	0.410356263
965.2967543	2.192818954	965.2967543	0.410291788
965.8948863	2.192279017	965.8948863	0.410210948
967.5955063	2.194667069	967.5955063	0.410246397

Final State: j'=1		Final State: j'=0	
E(cm⁻¹)	σ (Å²)	E(cm⁻¹)	σ (Å²)
967.8789423	2.194468624	967.8789423	0.410199739
969.1034773	2.193579665	969.1034773	0.410079046
969.6703513	2.192864839	969.6703513	0.410044088
970.2967543	2.195851968	970.2967543	0.410131836
970.8948863	2.195201484	970.8948863	0.410001344
972.5955063	2.19437099	972.5955063	0.409858126
972.8789423	2.194265702	972.8789423	0.409832458
974.1034773	2.195893664	974.1034773	0.409752665
974.6703513	2.195818607	974.6703513	0.409886791
975.2967543	2.195053161	975.2967543	0.409806522
975.8948863	2.195100614	975.8948863	0.409806124
977.5955063	2.195462847	977.5955063	0.409708709
977.8789423	2.194873587	977.8789423	0.409569389
979.1034773	2.196947291	979.1034773	0.409627631
979.6703513	2.195411887	979.6703513	0.409571178
980.2967543	2.196696134	980.2967543	0.409693157
980.8948863	2.196511113	980.8948863	0.409488518
982.5955063	2.196145431	982.5955063	0.409331066
982.8789423	2.196148928	982.8789423	0.409480709
984.1034773	2.197541778	984.1034773	0.40956741
984.6703513	2.198200105	984.6703513	0.409468875
985.2967543	2.196820654	985.2967543	0.409382318
985.8948863	2.196121996	985.8948863	0.409342622
987.5955063	2.197314278	987.5955063	0.409296761
987.8789423	2.197848264	987.8789423	0.409375716
989.1034773	2.197552592	989.1034773	0.409320818
989.6703513	2.197511734	989.6703513	0.409290721
990.2967543	2.197524634	990.2967543	0.409259969
990.8948863	2.197544629	990.8948863	0.409214747
992.5955063	2.198218251	992.5955063	0.409288487
992.8789423	2.198176954	992.8789423	0.409288079
994.1034773	2.198104836	994.1034773	0.409232932
994.6703513	2.198138306	994.6703513	0.409218866
995.2967543	2.198181922	995.2967543	0.4092052
995.8948863	2.198230555	995.8948863	0.409193193
997.5955063	2.198437951	997.5955063	0.409182062
997.8789423	2.198458807	997.8789423	0.409179995
999.1034773	2.198530454	999.1034773	0.409162341
999.6703513	2.198585936	999.6703513	0.409134386
1000.296754	2.198787788	1000.296754	0.409214349
1000.894884	2.198718875	1000.894884	0.40918669
1002.595504	2.198785822	1002.595504	0.40917175
1002.878944	2.198801573	1002.878944	0.409171546
1004.103474	2.198865244	1004.103474	0.40917277
1004.670354	2.198892189	1004.670354	0.409174235

Final State: j’=1		Final State: j’=0	
E(cm⁻¹)	σ (Å²)	E(cm⁻¹)	σ (Å²)
1005.296754	2.19892022	1005.296754	0.409176428
1005.894884	2.19894539	1005.894884	0.409179035
1007.595504	2.19901531	1007.595504	0.409190231
1007.878944	2.199030368	1007.878944	0.409194606
1009.103474	2.199072733	1009.103474	0.409213413
1009.670354	2.199082858	1009.670354	0.409219204
1010.296754	2.199093964	1010.296754	0.409226302
1010.894884	2.199103874	1010.894884	0.409233868
1012.595504	2.199125359	1012.595504	0.409259028
1012.878944	2.199127811	1012.878944	0.409263699
1014.103474	2.199134549	1014.103474	0.409285403
1014.670354	2.199135603	1014.670354	0.409296303
1015.296754	2.199135311	1015.296754	0.409309022
1015.894884	2.19913359	1015.894884	0.409321857
1017.595504	2.199119895	1017.595504	0.409361673
1017.878944	2.19911623	1017.878944	0.409368703
1019.103474	2.199095949	1019.103474	0.409400271
1019.670354	2.199084227	1019.670354	0.40941557
1020.296754	2.199069614	1020.296754	0.409433001
1020.894884	2.199054062	1020.894884	0.409450167
1022.595504	2.199001451	1022.595504	0.409501803
1022.878944	2.198991487	1022.878944	0.409510819
1024.103474	2.198944564	1024.103474	0.409551117
1024.670354	2.198920718	1024.670354	0.409570514
1025.296754	2.198892807	1025.296754	0.40959249
1025.894884	2.198864631	1025.894884	0.409614001
1027.595504	2.198776398	1027.595504	0.409677939
1029.103474	2.198688165	1029.103474	0.409738007
1029.670354	2.198652586	1029.670354	0.409761396
1030.296754	2.198611749	1030.296754	0.409787752
1030.894884	2.198571272	1030.894884	0.409813416
1032.595504	2.198448355	1032.595504	0.409889027
1034.103474	2.198329776	1034.103474	0.409959319
1034.670354	2.198282888	1034.670354	0.409986523
1035.296754	2.198229629	1035.296754	0.410017082
1035.894884	2.198177357	1035.894884	0.410046742
1037.595504	2.198021226	1037.595504	0.410133629
1039.103474	2.197873591	1039.103474	0.410213802
1039.670354	2.197815879	1039.670354	0.410244694
1040.296754	2.197750715	1040.296754	0.410279304
1040.894884	2.197687134	1040.894884	0.410312815
1042.595504	2.197499163	1042.595504	0.410410544
1044.103474	2.197323682	1044.103474	0.410500202
1044.670354	2.197255603	1044.670354	0.410534628
1045.296754	2.19717904	1045.296754	0.410573126

Final State: j’=1		Final State: j’=0	
E(cm^{-1})	σ (Å^2)	E(cm^{-1})	σ (Å^2)
1045.894884	2.197104631	1045.894884	0.410610329
1047.595504	2.196886201	1047.595504	0.410718455
1049.103474	2.196684105	1049.103474	0.410817209
1049.670354	2.196606116	1049.670354	0.410855025
1050.296754	2.196518665	1050.296754	0.410897248
1050.894884	2.19643392	1050.894884	0.410937989
1052.595504	2.196186421	1052.595504	0.41105608
1054.103474	2.195958949	1054.103474	0.411163543
1054.670354	2.195871513	1054.670354	0.411204602
1055.296754	2.195773687	1055.296754	0.41125039
1055.894884	2.195679093	1055.894884	0.411294518
1057.595504	2.195403911	1057.595504	0.411422131
1059.103474	2.195152288	1059.103474	0.411537917
1059.670354	2.19505587	1059.670354	0.411582073
1060.296754	2.194948179	1060.296754	0.411631264
1060.894884	2.194844226	1060.894884	0.411678624
1062.595504	2.194542753	1062.595504	0.411815325
1064.103474	2.194268208	1064.103474	0.411939046
1064.670354	2.19416327	1064.670354	0.411986155
1065.296754	2.194046224	1065.296754	0.412038591
1065.894884	2.1939334	1065.894884	0.41208903
1067.595504	2.19360702	1067.595504	0.412234388
1069.103474	2.19331078	1069.103474	0.412365659
1069.670354	2.193197782	1069.670354	0.412415578
1070.296754	2.193071889	1070.296754	0.412471099
1070.894884	2.19295068	1070.894884	0.41252446
1072.595504	2.192600771	1072.595504	0.412678048
1074.103474	2.19228406	1074.103474	0.412816492
1074.670354	2.192163464	1074.670354	0.412869079
1075.296754	2.192029235	1075.296754	0.412927528
1075.894884	2.191900119	1075.894884	0.412983674
1077.595504	2.191528053	1077.595504	0.413145056
1079.103474	2.19119209	1079.103474	0.413290298
1079.670354	2.191064344	1079.670354	0.413345411
1080.296754	2.19092228	1080.296754	0.413406633
1080.894884	2.190785749	1080.894884	0.413465406
1084.103474	2.190038878	1084.103474	0.413785844
1084.670354	2.189904445	1084.670354	0.413843344
1085.296754	2.189755047	1085.296754	0.413907188
1085.894884	2.189611564	1085.894884	0.413968446
1089.103474	2.188828414	1089.103474	0.414301914
1089.670354	2.188688204	1089.670354	0.414362051
1090.296754	2.188531502	1090.296754	0.41442798
1090.894884	2.188381554	1090.894884	0.414491579
1094.103474	2.187564649	1094.103474	0.41483732

Final State: j'=1		Final State: j'=0	
E(cm⁻¹)	σ (Å²)	E(cm⁻¹)	σ (Å²)
1094.670354	2.187418183	1094.670354	0.414899189
1095.296754	2.187255603	1095.296754	0.414967824
1095.894884	2.18709965	1095.894884	0.415033623
1099.103474	2.186251495	1099.103474	0.415390886
1099.670354	2.186099668	1099.670354	0.415454744
1100.894884	2.185769732	1100.894884	0.415593421
1104.103474	2.184892797	1104.103474	0.415961476
1104.670354	2.184736051	1104.670354	0.416027192
1105.894884	2.18439565	1105.894884	0.416169835
1109.103474	2.183492365	1109.103474	0.416547973
1109.670354	2.183331126	1109.670354	0.416615423
1110.894884	2.182981182	1110.894884	0.416761766
1114.103474	2.182053942	1114.103474	0.417149293
1114.670354	2.181888632	1114.670354	0.417218356
1115.894884	2.181530049	1115.894884	0.417368141
1119.103474	2.180581219	1119.103474	0.417764385
1119.670354	2.180412248	1119.670354	0.417834945
1120.894884	2.180045917	1120.894884	0.417987916
1124.103474	2.179077809	1124.103474	0.418392234
1124.670354	2.178905583	1124.670354	0.418464175
1125.894884	2.178532384	1125.894884	0.418620095
1129.103474	2.177547272	1129.103474	0.419031855
1129.670354	2.177372192	1129.670354	0.419105072
1130.894884	2.176992981	1130.894884	0.419263702
1134.103474	2.175993101	1134.103474	0.419682303
1134.670354	2.17581556	1134.670354	0.419756689
1135.894884	2.175431178	1135.894884	0.419917801
1139.103474	2.174418729	1139.103474	0.420342658
1139.670354	2.17423911	1139.670354	0.42041811
1140.894884	2.173850393	1140.894884	0.420581492
1144.103474	2.172827544	1144.103474	0.421012033
1144.670354	2.172646232	1144.670354	0.421088451
1145.894884	2.172254011	1145.894884	0.421253885
1149.103474	2.171222961	1149.103474	0.421689552
1149.670354	2.171040351	1149.670354	0.42176684
1150.894884	2.170645476	1150.894884	0.421934107
1154.103474	2.169608521	1154.103474	0.422374319
1154.670354	2.169425029	1154.670354	0.422452368
1155.894884	2.169028446	1155.894884	0.422621234
1159.103474	2.167988296	1159.103474	0.423065296
1159.670354	2.16780447	1159.670354	0.423143968
1160.894884	2.167407434	1160.894884	0.4233141
1164.103474	2.166368699	1164.103474	0.423760784
1165.894884	2.16579157	1165.894884	0.424010211
1169.103474	2.164783934	1169.103474	0.424450246

Final State: j’=1		Final State: j’=0	
E(cm⁻¹)	σ (Å²)	E(cm⁻¹)	σ (Å²)
1170.894884	2.164068908	1170.894884	0.424665074
1174.103474	2.163152859	1174.103474	0.42513786
1175.894884	2.162430134	1175.894884	0.425410575
1179.103474	2.161421057	1179.103474	0.425868998
1180.894884	2.160836147	1180.894884	0.426154185
1184.103474	2.15972735	1184.103474	0.426636783
1185.894884	2.159219202	1185.894884	0.426870709
1189.103474	2.158137091	1189.103474	0.427354562
1190.894884	2.157597595	1190.894884	0.42759944
1194.103474	2.156505964	1194.103474	0.428089616
1195.894884	2.156001838	1195.894884	0.428327864
1199.103474	2.154850774	1199.103474	0.428772758
1200.894884	2.15444974	1200.894884	0.429052499
1204.103474	2.153470972	1204.103474	0.42941422
1205.894884	2.15258805	1205.894884	0.429721088
1209.103474	2.151851536	1209.103474	0.430242107
1210.894884	2.151113008	1210.894884	0.430547684
1214.103474	2.150057419	1214.103474	0.431028821
1215.894884	2.1497467	1215.894884	0.431192197
1219.103474	2.148728346	1219.103474	0.431732507
1220.894884	2.148122055	1220.894884	0.431994163
1224.103474	2.147171809	1224.103474	0.432450707
1225.894884	2.146753519	1225.894884	0.432720847
1229.103474	2.145536726	1229.103474	0.433129353
1230.894884	2.144938778	1230.894884	0.433526276
1234.103474	2.143997453	1234.103474	0.433900857
1235.894884	2.143571508	1235.894884	0.434203089
1239.103474	2.142622214	1239.103474	0.434678917
1240.894884	2.142092287	1240.894884	0.434947555
1244.103474	2.141143874	1244.103474	0.435427148
1245.894884	2.140596185	1245.894884	0.435693598
1249.103474	2.139723677	1249.103474	0.436177981
1250.894884	2.139216355	1250.894884	0.436447678
1254.103474	2.138314164	1254.103474	0.436930797
1255.894884	2.137822553	1255.894884	0.437201973
1259.103474	2.136940065	1259.103474	0.437688233
1260.894884	2.136451732	1260.894884	0.437960149
1264.103474	2.135586034	1264.103474	0.438448196
1265.894884	2.135107377	1265.894884	0.438721432
1269.103474	2.134257851	1269.103474	0.439212025
1270.894884	2.133788067	1270.894884	0.439486613
1275.894884	2.132494335	1275.894884	0.440255848
1280.894884	2.131226289	1280.894884	0.441029587
1285.894884	2.129983967	1285.894884	0.4418082
1290.894884	2.128767387	1290.894884	0.442592117

Final State: j'=1		Final State: j'=0	
E(cm⁻¹)	σ (Å²)	E(cm⁻¹)	σ (Å²)
1295.894884	2.127576451	1295.894884	0.443381779
1300.894884	2.126411009	1300.894884	0.444177637
1305.894884	2.125270851	1305.894884	0.444980159
1310.894884	2.12415569	1310.894884	0.445789825
1315.894884	2.123065203	1315.894884	0.446607117
1320.894884	2.121998996	1320.894884	0.447432532
1325.894884	2.120956635	1325.894884	0.448266557
1330.894884	2.119937649	1330.894884	0.44910969
1335.894884	2.118941521	1335.894884	0.449962417
1340.894884	2.117967687	1340.894884	0.450825221
1345.894884	2.117015574	1345.894884	0.45169858
1350.894884	2.116084564	1350.894884	0.452582956
1355.894884	2.115174022	1355.894884	0.4534788
1360.894884	2.114283286	1360.894884	0.454386545
1365.894884	2.113411688	1365.894884	0.455306609
1370.894884	2.112558538	1370.894884	0.45623939
1375.894884	2.111723126	1375.894884	0.457185259
1380.894884	2.110904761	1380.894884	0.458144569
1385.894884	2.110102715	1385.894884	0.459117648
1390.894884	2.109316274	1390.894884	0.460104792
1395.894884	2.10854472	1395.894884	0.461106274
1400.894884	2.107787331	1400.894884	0.462122328
1405.894884	2.107043385	1405.894884	0.463153171

Initial rotational state: j=3

Final State: j'=5		Final State: j'=4		Final State: j'=2	
E(cm⁻¹)	σ (Å²)	E(cm⁻¹)	σ (Å²)	E(cm⁻¹)	σ (Å²)
411.028012	0.149260567	274.236603	1.242189437	164.803477	391.3558987
411.128012	0.290435368	274.336603	2.471107468	164.903477	739.7331363
411.228012	0.20958338	274.436603	1.76675627	165.003477	313.1721865
411.328012	0.224166301	274.536603	1.960109273	165.103477	280.0206549
411.428012	0.240445762	274.636603	2.14762151	165.203477	263.3365605
411.528012	0.286244301	274.703477	2.365208132	165.303477	297.687691
411.628012	0.32392461	274.736603	2.644653759	165.32988	323.0255529
411.728012	0.325764719	274.836603	3.167217032	165.403477	263.9674067
411.828012	0.306436712	274.936603	2.635992465	165.503477	218.7653154
411.928012	0.358611443	275.036603	2.655932764	165.603477	212.7392672
412.028012	0.329728036	275.136603	3.19910793	165.703477	221.4345161
412.128012	0.321809292	275.236603	2.739348602	165.803477	173.2220494
412.228012	0.323306473	275.32988	2.716734227	165.903477	161.6625315
412.328012	0.326965449	275.336603	2.718282756	167.628632	133.1437007
412.428012	0.331178077	275.436603	2.756113487	167.912068	120.4287015
412.528012	0.335374074	275.536603	2.80513444	169.703477	83.69390341
412.628632	0.339385441	277.628632	4.560471183	169.803477	83.03975631
412.912068	0.350740427	277.912068	4.459638716	169.903477	82.31802343
414.136603	0.536868106	279.136603	4.949177011	170.003477	81.53437045
414.703477	0.501753053	279.236603	4.999341168	170.103477	80.70599667
415.32988	0.513386974	279.336603	5.043854239	170.203477	79.86783052
415.928012	0.545575961	279.436603	5.082577485	170.303477	79.10238375
416.028012	0.550368237	279.536603	5.115862153	170.32988	78.93648109
416.128012	0.554649671	279.636603	5.144544714	170.403477	78.66634485
416.228012	0.558379301	279.703477	5.161930079	172.628632	67.146284
416.328012	0.561546673	279.736603	5.170349901	172.912068	64.40828879
416.428012	0.564150682	279.836603	5.199105804	174.703477	55.75697257
416.528012	0.56616123	279.936603	5.257521933	175.32988	55.91108513
416.628012	0.567552818	280.036603	5.373536608	177.628632	51.24371897
416.728012	0.571498225	280.32988	5.784472896	177.912068	50.00408689
416.828012	0.579064556	282.628632	6.001908288	179.703477	57.92911248
416.928012	0.586348356	282.912068	6.007691601	180.32988	44.46806414
417.028012	0.594677273	284.136603	6.198210789	182.628632	40.1861866
417.628632	0.780723851	284.703477	6.495004773	182.912068	39.72386049
417.912068	0.854001319	285.32988	7.108263878	184.703477	37.12621118
419.136603	0.657680524	287.628632	7.083682575	185.32988	36.42503417
419.703477	0.659286054	287.912068	7.105743075	187.628632	38.11413505
420.32988	0.667122659	289.136603	9.42291599	187.912068	37.33313978
420.928012	0.680129837	289.703477	7.339528271	189.703477	32.91894873
422.628632	0.779241639	290.32988	7.293055334	190.32988	32.11867825
422.912068	0.780927437	292.628632	7.4573324	192.628632	29.9946553
424.136603	0.768256139	292.912068	7.469320767	192.912068	29.78925981
424.703477	0.771289736	294.136603	7.530053877	194.703477	28.77454533
425.32988	0.8939548	294.703477	7.572149509	195.32988	28.50711994

Final State: j'=5		Final State: j'=4		Final State: j'=2	
E(cm⁻¹)	σ (Å²)	E(cm⁻¹)	σ (Å²)	E(cm⁻¹)	σ (Å²)
425.928012	0.92097608	295.32988	7.651257191	197.628632	27.28916184
427.628632	0.8089952	297.912068	8.241668183	197.912068	27.11682146
427.912068	0.812966086	299.136603	8.011239829	199.703477	26.07668706
429.136603	0.82740686	299.703477	7.982314359	200.32988	25.74460329
429.703477	0.833316114	300.32988	7.970799596	202.628632	24.66953932
430.32988	0.839867911	302.628632	8.01358298	202.912068	24.55083779
430.928012	0.846426383	302.912068	8.026947316	204.703477	23.85052796
432.628632	0.873504091	304.136603	8.111057997	205.32988	23.61966303
432.912068	0.883333558	304.703477	8.159781844	207.628632	22.79688615
434.136603	0.921914957	305.32988	8.208851594	207.912068	22.69637119
434.703477	0.914310918	307.628632	8.280646849	209.703477	22.05113022
435.32988	0.911598911	307.912068	8.283454924	210.32988	21.81179965
435.928012	0.91330211	309.136603	8.296183398	212.628632	20.57764231
437.628632	0.925848207	309.703477	8.30381428	212.912068	20.56719099
437.912068	0.928396124	310.32988	8.314024946	214.703477	20.39754484
439.136603	0.940264632	312.628632	8.367999619	215.32988	20.77591479
439.703477	0.946274017	312.912068	8.376177718	217.628632	20.07657522
440.32988	0.953397168	314.136603	8.41334935	217.912068	20.1630769
440.928012	0.960687219	314.703477	8.430793529	219.703477	19.70178884
442.628632	0.981517983	315.32988	8.449677318	220.32988	19.30622634
442.912068	0.984614928	317.628632	8.512612793	222.628632	19.73898788
444.136603	0.996860273	317.912068	8.519711605	222.912068	19.55880683
444.703477	1.002233305	319.136603	8.54933884	224.703477	18.94459824
445.32988	1.008141943	319.703477	8.562688573	225.32988	19.23792953
445.928012	1.013816907	320.32988	8.577321248	227.628632	18.80088183
447.628632	1.030322873	322.628632	8.631073586	227.912068	18.65442892
447.912068	1.033135866	322.912068	8.637756488	229.703477	18.29650066
449.136603	1.045492998	324.136603	8.666740393	230.32988	18.38291479
449.703477	1.051314379	324.703477	8.680178528	232.628632	18.25340026
450.32988	1.057803479	325.32988	8.695001672	232.912068	18.14896073
450.928012	1.064036959	327.628632	8.748671689	234.703477	17.28868628
452.628632	1.08180654	327.912068	8.755173447	235.32988	17.9283597
452.912068	1.084760914	329.136603	8.782912424	237.628632	17.8501579
454.136603	1.097479747	329.703477	8.795555771	237.912068	17.76532721
454.703477	1.103345222	330.32988	8.809381187	239.703477	17.18766409
455.32988	1.109815092	332.628632	8.858840426	240.32988	16.75656782
455.928012	1.115986211	332.912068	8.864802307	242.628632	17.66522576
457.628632	1.133532205	334.136603	8.890210473	242.912068	17.47850163
457.912068	1.136460303	334.703477	8.901773864	244.703477	16.84031905
459.136603	1.149132721	335.32988	8.914395848	245.32988	16.5993849
459.703477	1.155012932	337.628632	8.959117418	247.628632	15.46911901
460.32988	1.161521203	337.912068	8.964431018	247.912068	16.001946
460.928012	1.167745636	339.136603	8.986773721	249.703477	16.38170036
462.628632	1.18548886	339.703477	8.996736382	250.32988	16.70036084
462.912068	1.188451544	340.32988	9.007420389	252.628632	16.35448688

Final State: j’=5		Final State: j’=4		Final State: j’=2	
E(cm⁻¹)	σ (Å²)	E(cm⁻¹)	σ (Å²)	E(cm⁻¹)	σ (Å²)
464.136603	1.20126494	342.628632	9.04283321	252.912068	16.61527181
464.703477	1.207202914	342.912068	9.04666141	254.703477	15.11465096
465.32988	1.21376823	344.136603	9.061231914	255.32988	15.63814747
465.928012	1.220040559	344.703477	9.066581877	257.628632	16.16290315
467.628632	1.237890183	345.32988	9.07106178	257.912068	15.92243218
467.912068	1.240867369	347.628632	9.060186646	259.703477	16.42817364
469.136603	1.253737245	347.912068	9.051975884	260.32988	16.16803583
469.703477	1.259699308	349.136603	8.934736594	262.628632	14.84005216
470.32988	1.266290697	349.703477	8.903715356	262.912068	15.12306073
470.928012	1.272587719	350.32988	9.071315142	264.703477	15.91266411
472.628632	1.290508106	352.628632	9.246099491	265.32988	15.67943451
472.912068	1.293497141	352.912068	9.224044871	267.628632	16.24960245
474.136603	1.306417751	354.136603	9.132722021	267.912068	15.98789252
474.703477	1.312402698	354.703477	9.225316414	269.703477	15.5360808
475.32988	1.319018574	355.32988	9.306866215	270.32988	14.74691793
475.928012	1.325337981	357.628632	9.263464862	272.628632	15.33699685
477.628632	1.343314996	357.912068	9.381693589	272.912068	15.51987294
477.912068	1.346312273	359.136603	9.521856189	274.236603	15.119942
479.136603	1.359264122	359.703477	9.466081951	274.336603	14.71996073
479.703477	1.365260997	360.32988	9.364556675	274.436603	14.90179898
480.32988	1.371888117	362.628632	9.367565355	274.536603	14.73331225
480.928012	1.378216348	362.912068	9.563960695	274.636603	14.49853129
482.628632	1.396207924	364.136603	9.571170512	274.703477	14.21010922
482.912068	1.399206075	364.703477	9.510126258	274.736603	14.04256219
484.136603	1.412156327	365.32988	9.389728028	274.836603	14.44800492
484.703477	1.418149395	367.628632	9.689599089	274.936603	14.88835832
485.32988	1.424769954	367.912068	9.837598087	275.036603	14.64707103
485.928012	1.431089509	369.136603	9.691674119	275.136603	14.46986374
487.628632	1.449042509	369.703477	9.649863254	275.236603	14.94290056
487.912068	1.452032086	370.32988	9.585414525	275.32988	14.87191188
489.136603	1.464937424	372.628632	9.529017695	275.336603	14.86605519
489.703477	1.470905075	372.912068	9.784523242	275.436603	14.78211851
490.32988	1.477493834	374.136603	9.844782403	275.536603	14.70365525
490.928012	1.483779149	374.703477	9.806151524	277.628632	14.46748972
492.628632	1.501611665	375.32988	9.771013354	277.912068	14.42056477
492.912068	1.504577498	377.628632	9.381995435	279.136603	14.06468666
494.136603	1.517366581	377.912068	9.593522883	279.236603	14.02808517
494.703477	1.523272231	379.136603	9.902015332	279.336603	13.98825866
495.32988	1.52978573	379.703477	9.983094495	279.436603	13.9430068
495.928012	1.535992038	380.32988	9.899189316	279.536603	13.88859396
497.628632	1.55355576	382.628632	9.635728493	279.636603	13.81826938
497.912068	1.556469533	382.912068	9.567098498	279.703477	13.75634556
499.136603	1.56900539	384.136603	9.455828389	279.736603	13.71913341
499.703477	1.574776193	384.703477	9.496442018	279.836603	13.57222156
500.32988	1.581125715	385.32988	9.780748289	279.936603	13.44578948

Final State: j'=5		Final State: j'=4		Final State: j'=2	
E(cm^{-1})	σ (Å^2)	E(cm^{-1})	σ (Å^2)	E(cm^{-1})	σ (Å^2)
500.928012	1.587158931	387.628632	10.08535469	280.036603	13.62010798
502.628632	1.604122187	387.912068	10.02152198	280.32988	13.62615772
502.912068	1.606917023	389.136603	9.769413407	282.628632	14.03789049
504.136603	1.618858907	389.703477	10.11740624	282.912068	14.0051722
504.703477	1.6243018	390.32988	10.04325968	284.136603	13.86245461
505.32988	1.630240148	392.628632	10.27032273	284.703477	13.76942876
505.928012	1.635823379	392.912068	10.14397553	285.32988	13.64608709
507.628632	1.65107566	394.136603	10.2121205	287.628632	13.56891861
507.912068	1.65349973	394.703477	10.03687615	287.912068	13.52945763
509.136603	1.663394944	395.32988	9.670927063	289.136603	13.44437171
509.703477	1.667541294	397.628632	10.17608179	289.703477	13.63697185
510.32988	1.671640912	397.912068	10.13023733	290.32988	13.56626751
510.928012	1.674866169	399.136603	9.530813245	292.628632	13.44035117
512.628632	1.674438505	399.703477	9.835126659	292.912068	13.42868547
512.912068	1.670277933	400.32988	10.35054165	294.136603	13.37768549
514.136603	1.665080701	402.628632	10.03841462	294.703477	13.35198812
514.703477	1.72339383	402.912068	10.0557672	295.32988	13.31865909
515.32988	1.704455193	404.136603	10.27438336	297.628632	13.21320452
515.928012	1.71592565	404.703477	10.49975516	297.912068	13.22004568
517.628632	1.706009621	405.32988	10.01571553	299.136603	13.20770823
517.912068	1.708988928	407.628632	9.974315291	299.703477	13.19372479
519.136603	1.789873585	407.912068	9.941851317	300.32988	13.17669401
519.703477	1.765073292	409.136603	10.38229029	302.628632	13.1087005
520.32988	1.801013494	409.703477	10.44410392	302.912068	13.09978636
520.928012	1.764845186	410.32988	10.65423963	304.136603	13.05995891
522.628632	1.805826134	411.028012	10.27076133	304.703477	13.04166474
522.912068	1.811566563	411.128012	10.0375769	305.32988	13.02281257
524.136603	1.841548443	411.228012	10.16443716	307.628632	12.96606188
524.703477	1.819103869	411.328012	10.08251948	307.912068	12.95952455
525.32988	1.787091369	411.428012	9.959008379	309.136603	12.93107292
525.928012	1.871658063	411.528012	9.732142558	309.703477	12.91774897
527.628632	1.915964862	411.628012	9.829909114	310.32988	12.90292662
527.912068	1.907833172	411.728012	10.14828399	312.628632	12.84807801
529.136603	1.882291522	411.828012	10.06709288	312.912068	12.84132519
529.703477	1.850026808	411.928012	9.902148692	314.136603	12.81240661
530.32988	1.881735959	412.028012	10.21431085	314.703477	12.79922446
530.928012	1.935518033	412.128012	10.18174537	315.32988	12.78484669
532.628632	1.969194376	412.228012	10.1394594	317.628632	12.73381607
532.912068	1.964241441	412.328012	10.1002663	317.912068	12.72769299
534.136603	1.949588468	412.428012	10.06203043	319.136603	12.70158627
534.703477	1.936719626	412.528012	10.02183409	319.703477	12.68967167
535.32988	1.892066739	412.628632	9.975669262	320.32988	12.67661873
535.928012	1.957930173	412.912068	9.736955486	322.628632	12.6296538
537.628632	2.046497406	414.136603	10.05604023	322.912068	12.62396216
537.912068	2.034244291	414.703477	10.0337371	324.136603	12.59962689

Final State: j'=5		Final State: j'=4		Final State: j'=2	
E(cm⁻¹)	σ (Å²)	E(cm⁻¹)	σ (Å²)	E(cm⁻¹)	σ (Å²)
539.136603	2.018474927	415.32988	9.966808737	324.703477	12.58850443
539.703477	2.014501976	415.928012	9.88099379	325.32988	12.576322
540.32988	2.008091038	416.028012	9.864509176	327.628632	12.53261355
540.928012	1.994469711	416.128012	9.846328352	327.912068	12.52733435
542.628632	2.032731254	416.228012	9.825289316	329.136603	12.50480506
542.912068	2.038820908	416.328012	9.799375834	329.703477	12.49452821
544.136603	2.082965199	416.428012	9.76477828	330.32988	12.48328403
544.703477	2.071996628	416.528012	9.713391888	332.628632	12.44302279
545.32988	2.063397449	416.628012	9.628633176	332.912068	12.43816775
545.928012	2.053680592	416.728012	9.573566271	334.136603	12.41747088
547.628632	2.004371101	416.828012	9.619620196	334.703477	12.40804462
547.912068	2.028951229	416.928012	9.777878018	335.32988	12.39774468
549.136603	2.109511963	417.028012	9.744833207	337.628632	12.36103581
549.703477	2.13044461	417.628632	9.780709142	337.912068	12.35663418
550.32988	2.143213578	417.912068	9.832303344	339.136603	12.33795704
550.928012	2.105791586	419.136603	9.990209549	339.703477	12.32950795
552.628632	2.113395242	419.703477	9.969325202	340.32988	12.32032792
552.912068	2.069546117	420.32988	9.949191518	342.628632	12.28826885
554.136603	2.217731763	420.928012	9.92642126	342.912068	12.28452569
554.703477	2.179849142	422.628632	9.835751062	344.136603	12.26904
555.32988	2.126097278	422.912068	9.84531126	344.703477	12.26232727
555.928012	1.99658808	424.136603	9.871899596	345.32988	12.25534498
557.628632	2.31387221	424.703477	9.852601909	347.628632	12.23686223
557.912068	2.275468083	425.32988	9.637419432	347.912068	12.23612199
559.136603	2.16781388	425.928012	9.841519032	349.136603	12.24080934
559.703477	2.166237479	427.628632	9.933235825	349.703477	12.20674435
560.32988	2.346732626	427.912068	9.931029665	350.32988	12.14191124
560.928012	2.29938909	429.136603	9.930357035	352.628632	12.11502446
562.628632	2.225372154	429.703477	9.931905014	352.912068	12.13398074
562.912068	2.197286529	430.32988	9.933628951	354.136603	12.0831779
564.136603	2.349508011	430.928012	9.934752657	354.703477	12.052347
564.703477	2.30208478	432.628632	9.924588472	355.32988	12.07754381
565.32988	2.305407914	432.912068	9.91664097	357.628632	12.00215086
565.928012	2.279750952	434.136603	9.91921678	357.912068	11.97753644
567.628632	2.186052639	434.703477	9.94293191	359.136603	11.96411394
567.912068	2.178331123	435.32988	9.95659209	359.703477	12.01013649
569.136603	2.363274747	435.928012	9.96412728	360.32988	12.03309608
569.703477	2.317134234	437.628632	9.977988896	362.628632	11.95202517
570.32988	2.400730811	437.912068	9.979898343	362.912068	11.89549971
570.928012	2.338800491	439.136603	9.987297983	364.136603	11.95426971
572.628632	2.30406274	439.703477	9.990195085	364.703477	11.96730312
572.912068	2.404138301	440.32988	9.992967484	365.32988	11.98548754
574.136603	2.477572578	440.928012	9.995328674	367.628632	11.82415223
574.703477	2.396453187	442.628632	10.00415726	367.912068	11.83462169
575.32988	2.376391078	442.912068	10.00622076	369.136603	11.89123521

Final State: j'=5		Final State: j'=4		Final State: j'=2	
E(cm⁻¹)	σ (Å²)	E(cm⁻¹)	σ (Å²)	E(cm⁻¹)	σ (Å²)
575.928012	2.393406286	444.136603	10.01604501	369.703477	11.89984464
577.628632	2.375321666	444.703477	10.02065709	370.32988	11.91114654
577.912068	2.433384321	445.32988	10.02562333	372.628632	11.84000256
579.136603	2.395609879	445.928012	10.03020863	372.912068	11.75688791
579.703477	2.384815581	447.628632	10.04244	374.136603	11.81989522
580.32988	2.370317005	447.912068	10.04437554	374.703477	11.82943167
580.928012	2.347057442	449.136603	10.0524696	375.32988	11.83639638
582.628632	2.442178741	449.703477	10.05610265	377.628632	11.81606934
582.912068	2.440112035	450.32988	10.06006716	377.912068	11.74869041
584.136603	2.436639379	450.928012	10.06382865	379.136603	11.70788647
584.703477	2.43733379	452.628632	10.07455061	379.703477	11.75163483
585.32988	2.437526816	452.912068	10.0763505	380.32988	11.77698886
585.928012	2.436504534	454.136603	10.08416089	382.628632	11.82906711
587.628632	2.454588154	454.703477	10.08778393	382.912068	11.82766021
587.912068	2.457412181	455.32988	10.09178224	384.136603	11.80162307
589.136603	2.456108723	455.928012	10.09558808	384.703477	11.67191069
589.703477	2.466998598	457.628632	10.10630107	385.32988	11.69878726
590.32988	2.497608143	457.912068	10.10806758	387.628632	11.65418216
590.928012	2.499243876	459.136603	10.11563132	387.912068	11.6825306
592.628632	2.509532658	459.703477	10.1190957	389.136603	11.65184286
592.912068	2.51200805	460.32988	10.12289785	389.703477	11.58880758
594.136603	2.523084064	460.928012	10.12650437	390.32988	11.66109317
594.703477	2.528223225	462.628632	10.13664372	392.628632	11.48018134
595.32988	2.53380524	462.912068	10.13831874	392.912068	11.50274539
595.928012	2.538929043	464.136603	10.1455114	394.136603	11.61539397
597.628632	2.551677257	464.703477	10.14881803	394.703477	11.65781205
597.912068	2.555655607	465.32988	10.15245548	395.32988	11.63515168
599.136603	2.572677205	465.928012	10.15591281	397.628632	11.60865102
599.703477	2.578566442	467.628632	10.16565695	397.912068	11.62114989
600.32988	2.584787141	467.912068	10.16726847	399.136603	11.68550652
600.928012	2.590651269	469.136603	10.17418882	399.703477	11.50883286
602.628632	2.607222004	469.703477	10.17736916	400.32988	11.47061666
602.912068	2.609971482	470.32988	10.18086623	402.628632	11.58529617
604.136603	2.621796493	470.928012	10.18418855	402.912068	11.57698618
604.703477	2.627255473	472.628632	10.19354488	404.136603	11.37670191
605.32988	2.633320772	472.912068	10.1950915	404.703477	11.4681919
605.928012	2.639194341	474.136603	10.20173214	405.32988	11.56717985
607.628632	2.656338145	474.703477	10.20478396	407.628632	11.5234571
607.912068	2.65921435	475.32988	10.20814016	407.912068	11.56737876
609.136603	2.671581732	475.928012	10.21132928	409.136603	11.40604342
609.703477	2.677270474	477.628632	10.22031572	409.703477	11.3877503
610.32988	2.683533217	477.912068	10.22180207	410.32988	11.39374234
610.928012	2.689494643	479.136603	10.22818696	411.028012	11.48996055
612.628632	2.706372493	479.703477	10.23112299	411.128012	11.48539793
612.912068	2.709178384	480.32988	10.23435305	411.228012	11.49370644

Final State: j'=5		Final State: j'=4		Final State: j'=2	
E(cm⁻¹)	σ (Å²)	E(cm⁻¹)	σ (Å²)	E(cm⁻¹)	σ (Å²)
614.136603	2.721290477	480.928012	10.23742358	411.328012	11.51036612
614.703477	2.726896127	482.628632	10.24608226	411.428012	11.52964384
615.32988	2.733092197	482.912068	10.24751535	411.528012	11.51794202
615.928012	2.739011263	484.136603	10.25367486	411.628012	11.44424625
617.628632	2.755852437	484.703477	10.25650918	411.728012	11.39396124
617.912068	2.758660015	485.32988	10.25962892	411.828012	11.45262908
619.136603	2.770787138	485.928012	10.26259619	411.928012	11.41997311
619.703477	2.776398384	487.628632	10.27097331	412.028012	11.39426493
620.32988	2.782596019	487.912068	10.27236131	412.128012	11.4264327
620.928012	2.788510751	489.136603	10.2783329	412.228012	11.44357949
622.628632	2.80530957	489.703477	10.28108425	412.328012	11.45521442
622.912068	2.808106796	490.32988	10.28411553	412.428012	11.4646633
624.136603	2.820183847	490.928012	10.28700163	412.528012	11.47346868
624.703477	2.825770648	492.628632	10.29516777	412.628632	11.48268661
625.32988	2.831941407	492.912068	10.29652377	412.912068	11.51712269
625.928012	2.837831155	494.136603	10.30236849	414.136603	11.38097725
627.628632	2.854564537	494.703477	10.30506799	414.703477	11.40177392
627.912068	2.857351715	495.32988	10.3080476	415.32988	11.41327549
629.136603	2.869387567	495.928012	10.31089036	415.928012	11.4189061
629.703477	2.874956196	497.628632	10.31896989	416.028012	11.42008483
630.32988	2.881107277	497.912068	10.32031743	416.128012	11.42157886
630.928012	2.886978393	499.136603	10.32614897	416.228012	11.42352883
632.628632	2.903658433	499.703477	10.3288568	416.328012	11.42610992
632.912068	2.906436548	500.32988	10.33185781	416.428012	11.42950757
634.136603	2.918432552	500.928012	10.33473448	416.528012	11.43359972
634.703477	2.923982335	502.628632	10.34299821	416.628012	11.43467471
635.32988	2.930112331	502.912068	10.34439168	416.728012	11.40280751
635.928012	2.935963123	504.136603	10.3504862	416.828012	11.36761346
637.628632	2.952584589	504.703477	10.35335823	416.928012	11.34321211
637.912068	2.95535288	505.32988	10.35657978	417.028012	11.37849644
639.136603	2.967306372	505.928012	10.35971278	417.628632	11.31421414
639.703477	2.972836483	507.628632	10.36904806	417.912068	11.30147695
640.32988	2.978944748	507.912068	10.37068779	419.136603	11.34461062
640.928012	2.984774813	509.136603	10.37819542	419.703477	11.34736369
642.628632	3.001337499	509.703477	10.38199406	420.32988	11.34702667
642.912068	3.004096008	510.32988	10.3865538	420.928012	11.34527263
644.136603	3.016007274	510.928012	10.39142656	422.628632	11.32238933
644.703477	3.021517854	512.628632	10.41233861	422.912068	11.31850972
645.32988	3.027604545	512.912068	10.41866868	424.136603	11.31210496
645.928012	3.033413991	514.136603	10.42377847	424.703477	11.3118028
647.628632	3.049918053	514.703477	10.38916004	425.32988	11.28150636
647.912068	3.052666794	515.32988	10.41527371	425.928012	11.25104059
649.136603	3.064535855	515.928012	10.40324762	427.628632	11.27380956
649.703477	3.070026907	517.628632	10.44108286	427.912068	11.27175882
650.32988	3.076092036	517.912068	10.43591332	429.136603	11.26216671

Final State: j'=5		Final State: j'=4		Final State: j'=2	
E(cm⁻¹)	σ (Å²)	E(cm⁻¹)	σ (Å²)	E(cm⁻¹)	σ (Å²)
650.928012	3.081880913	519.136603	10.39046774	429.703477	11.25765865
652.628632	3.098326634	519.703477	10.41900459	430.32988	11.25267785
652.912068	3.101065677	520.32988	10.40165895	430.928012	11.24791865
654.136603	3.112892974	520.928012	10.43657732	432.628632	11.23375532
654.703477	3.118364755	522.628632	10.42246875	432.912068	11.23061494
655.32988	3.124408662	522.912068	10.40915375	434.136603	11.21262855
655.928012	3.130177334	524.136603	10.417872	434.703477	11.20925148
657.628632	3.146566004	524.703477	10.44140359	435.32988	11.2055747
657.912068	3.149295597	525.32988	10.45910677	435.928012	11.20151079
659.136603	3.161082288	525.928012	10.39997872	437.628632	11.18865545
659.703477	3.16653542	527.628632	10.39945727	437.912068	11.18643388
660.32988	3.172558801	527.912068	10.41019057	439.136603	11.17671375
660.928012	3.178308003	529.136603	10.44529344	439.703477	11.17214232
662.628632	3.194641967	529.703477	10.47378187	440.32988	11.16701147
662.912068	3.197362546	530.32988	10.43736059	440.928012	11.16201169
664.136603	3.209110655	530.928012	10.41478819	442.628632	11.14754918
664.703477	3.21454614	532.628632	10.41714924	442.912068	11.14518586
665.32988	3.220550206	532.912068	10.4248464	444.136603	11.13523818
665.928012	3.226281138	534.136603	10.4512652	444.703477	11.13073355
667.628632	3.242564244	534.703477	10.46744526	445.32988	11.12579456
667.912068	3.245276523	535.32988	10.50282026	445.928012	11.12110127
669.136603	3.256989372	535.928012	10.44063315	447.628632	11.10781426
669.703477	3.262408896	537.628632	10.41221684	447.912068	11.10560357
670.32988	3.268395604	537.912068	10.42672117	449.136603	11.09605604
670.928012	3.274110269	539.136603	10.45460469	449.703477	11.09163732
672.628632	3.290348839	539.703477	10.46437422	450.32988	11.08675664
672.912068	3.293053961	540.32988	10.47652379	450.928012	11.08210054
674.136603	3.304736916	540.928012	10.49335852	452.628632	11.06891115
674.703477	3.31014318	542.628632	10.47105898	452.912068	11.06672282
675.32988	3.316115714	542.912068	10.45724924	454.136603	11.05730788
675.928012	3.321817325	544.136603	10.45965742	454.703477	11.05297144
677.628632	3.338021658	544.703477	10.47524241	455.32988	11.04819544
677.912068	3.340721521	545.32988	10.48917412	455.928012	11.04364983
679.136603	3.352383403	545.928012	10.50330149	457.628632	11.03079855
679.703477	3.357780887	547.628632	10.54676171	457.912068	11.02866635
680.32988	3.363744512	547.912068	10.53138935	459.136603	11.01948412
680.928012	3.369438438	549.136603	10.46386777	459.703477	11.01524894
682.628632	3.385625848	549.703477	10.45570117	460.32988	11.01058007
682.912068	3.388323682	550.32988	10.470555	460.928012	11.00613279
684.136603	3.399979755	550.928012	10.49426464	462.628632	10.99354556
684.703477	3.405376328	552.628632	10.52576883	462.912068	10.99145602
685.32988	3.411340391	552.912068	10.55392798	464.136603	10.98245629
685.928012	3.41703628	554.136603	10.46006977	464.703477	10.97830542
687.628632	3.433238767	554.703477	10.49929628	465.32988	10.97373001
687.912068	3.435940664	555.32988	10.54677998	465.928012	10.96937226

Final State: j’=5		Final State: j’=4		Final State: j’=2	
E(cm⁻¹)	σ (Å²)	E(cm⁻¹)	σ (Å²)	E(cm⁻¹)	σ (Å²)
689.136603	3.447620282	555.928012	10.62050277	467.628632	10.95704159
689.703477	3.453031463	557.628632	10.4226459	467.912068	10.95499497
690.32988	3.459014794	557.912068	10.4596437	469.136603	10.94618049
690.928012	3.464732491	559.136603	10.55324198	469.703477	10.94211505
692.628632	3.481018946	559.703477	10.53013998	470.32988	10.93763364
692.912068	3.48373859	560.32988	10.4199443	470.928012	10.93336521
694.136603	3.495510324	560.928012	10.4728336	472.628632	10.92128534
694.703477	3.500974126	562.628632	10.5495004	472.912068	10.91928006
695.32988	3.507024771	562.912068	10.57263472	474.136603	10.91064277
695.928012	3.512816438	564.136603	10.43817896	474.703477	10.90665862
697.628632	3.529386292	564.703477	10.49949781	475.32988	10.90226656
697.912068	3.532166709	565.32988	10.49974293	475.928012	10.89808291
699.136603	3.544265044	565.928012	10.53066291	477.628632	10.88624229
699.703477	3.549926212	567.628632	10.61701673	477.912068	10.88427661
700.32988	3.556242405	567.912068	10.59558447	479.136603	10.87580978
700.928012	3.562350605	569.136603	10.50871699	479.703477	10.87190413
702.628632	3.580429388	569.703477	10.52699528	480.32988	10.86759855
702.912068	3.583620898	570.32988	10.49118269	480.928012	10.86349729
704.136603	3.598964593	570.928012	10.54772026	482.628632	10.85188958
704.703477	3.608367823	572.628632	10.53463372	482.912068	10.84996258
705.32988	3.611619431	572.912068	10.47904589	484.136603	10.84166216
705.928012	3.601281954	574.136603	10.46707474	484.703477	10.83783329
707.628632	3.609030878	574.703477	10.53270612	485.32988	10.83361239
707.912068	3.613303027	575.32988	10.53598862	485.928012	10.82959178
709.136603	3.645235499	575.928012	10.50908469	487.628632	10.81821255
709.703477	3.638550753	577.628632	10.50119441	487.912068	10.81632348
710.32988	3.634750073	577.912068	10.4744841	489.136603	10.80818694
710.928012	3.643250361	579.136603	10.52552339	489.703477	10.80443382
712.628632	3.675947588	579.703477	10.53681754	490.32988	10.80029658
712.912068	3.673988917	580.32988	10.54971502	490.928012	10.79635587
714.136603	3.679251025	580.928012	10.55812894	492.628632	10.78520403
714.703477	3.665621794	582.628632	10.50040603	492.912068	10.78335297
715.32988	3.685254166	582.912068	10.50899055	494.136603	10.77538088
715.928012	3.698025836	584.136603	10.5242238	494.703477	10.77170425
717.628632	3.697592305	584.703477	10.52805501	495.32988	10.76765178
717.912068	3.703720583	585.32988	10.53176085	495.928012	10.76379238
719.136603	3.713228102	585.928012	10.53399808	497.628632	10.7528742
719.703477	3.724959519	587.628632	10.53021155	497.912068	10.75106255
720.32988	3.735907862	587.912068	10.53105854	499.136603	10.74326262
720.928012	3.747847214	589.136603	10.54143491	499.703477	10.73966699
722.628632	3.752246894	589.703477	10.52149414	500.32988	10.73570517
722.912068	3.734506434	590.32988	10.51282299	500.928012	10.73193366
724.136603	3.760509326	590.928012	10.52493666	502.628632	10.72127462
724.703477	3.77019847	592.628632	10.52983443	502.912068	10.71950782
725.32988	3.779794928	592.912068	10.53015665	504.136603	10.71190909

Final State: j'=5		Final State: j'=4		Final State: j'=2	
E(cm⁻¹)	σ (Å²)	E(cm⁻¹)	σ (Å²)	E(cm⁻¹)	σ (Å²)
725.928012	3.78939526	594.136603	10.53137252	504.703477	10.70841158
727.628632	3.779920706	594.703477	10.53190674	505.32988	10.704563
727.912068	3.794719835	595.32988	10.53250332	505.928012	10.70090531
729.136603	3.799180623	595.928012	10.53309976	507.628632	10.69061414
729.703477	3.812521204	597.628632	10.53236693	507.912068	10.68891766
730.32988	3.822362539	597.912068	10.53093621	509.136603	10.68167145
730.928012	3.830843881	599.136603	10.53036635	509.703477	10.67837638
732.628632	3.858443892	599.703477	10.53099413	510.32988	10.6747988
732.912068	3.86616682	600.32988	10.53152127	510.928012	10.6714727
734.136603	3.858700465	600.928012	10.53190382	512.628632	10.66336833
734.703477	3.846428146	602.628632	10.53264997	512.912068	10.66269365
735.32988	3.862540951	602.912068	10.53274327	514.136603	10.66601861
735.928012	3.875514863	604.136603	10.53305008	514.703477	10.65427446
737.628632	3.903657901	604.703477	10.53311828	515.32988	10.65153099
737.912068	3.909155361	605.32988	10.53311522	515.928012	10.65387554
739.136603	3.92113526	605.928012	10.53303665	517.628632	10.64017526
739.703477	3.921861916	607.628632	10.53272658	517.912068	10.64268772
740.32988	3.915359269	607.912068	10.53269363	519.136603	10.62599554
740.928012	3.910471335	609.136603	10.53260413	519.703477	10.624474
742.628632	3.914595912	609.703477	10.53257475	520.32988	10.61525642
742.912068	3.909082156	610.32988	10.53254099	520.928012	10.6170805
744.136603	3.959908856	610.928012	10.53250263	522.628632	10.60512965
744.703477	3.956614842	612.628632	10.53233775	522.912068	10.61135734
745.32988	3.921582588	612.912068	10.53230023	524.136603	10.59137012
745.928012	3.955468016	614.136603	10.53210016	524.703477	10.59182862
747.628632	3.975520265	614.703477	10.53198654	525.32988	10.60258926
747.912068	3.935299939	615.32988	10.53184638	525.928012	10.59001409
749.136603	3.96299059	615.928012	10.53169945	527.628632	10.56754796
749.703477	3.98927125	617.628632	10.53122411	527.912068	10.56660689
750.32988	4.009228915	617.912068	10.53113783	529.136603	10.56362963
750.928012	4.026616924	619.136603	10.53074447	529.703477	10.56706967
752.628632	4.012815238	619.703477	10.53055133	530.32988	10.57355297
752.912068	4.020086498	620.32988	10.53032971	530.928012	10.554305
754.136603	4.05049012	620.928012	10.53010991	532.628632	10.53824896
754.703477	4.075414992	622.628632	10.5294392	532.912068	10.53726905
755.32988	4.023365033	622.912068	10.52932061	534.136603	10.53314753
755.928012	4.019629741	624.136603	10.52878527	534.703477	10.53246233
757.628632	4.062848323	624.703477	10.52852462	535.32988	10.53939451
757.912068	4.066833152	625.32988	10.52822715	535.928012	10.54025056
759.136603	4.109255767	625.928012	10.52793381	537.628632	10.50812613
759.703477	4.028052032	627.628632	10.52705088	537.912068	10.50742175
760.32988	4.064615003	627.912068	10.52689678	539.136603	10.50312504
760.928012	4.110616327	629.136603	10.5262086	539.703477	10.50104014
762.628632	4.127627117	629.703477	10.52587786	540.32988	10.499117
762.912068	4.132743457	630.32988	10.52550354	540.928012	10.49857748

Final State: j'=5		Final State: j'=4		Final State: j'=2	
E(cm⁻¹)	σ (Å²)	E(cm⁻¹)	σ (Å²)	E(cm⁻¹)	σ (Å²)
764.136603	4.090633336	630.928012	10.52513749	542.628632	10.49511089
764.703477	4.099670203	632.628632	10.52405134	542.912068	10.49812912
765.32988	4.091927943	632.912068	10.52386383	544.136603	10.47431017
765.928012	4.121178105	634.136603	10.52303256	544.703477	10.47284322
767.628632	4.115444742	634.703477	10.52263615	545.32988	10.47105628
767.912068	4.146658104	635.32988	10.52218966	545.928012	10.46968218
769.136603	4.131993652	635.928012	10.521755	547.628632	10.47744468
769.703477	4.149136674	637.628632	10.52047524	547.912068	10.47429747
770.32988	4.162081612	637.912068	10.52025568	549.136603	10.47039049
770.928012	4.171608424	639.136603	10.51928653	549.703477	10.46392311
772.628632	4.184774495	639.703477	10.51882667	550.32988	10.44780409
772.912068	4.187562548	640.32988	10.51831032	550.928012	10.45901275
774.136603	4.184649766	640.928012	10.51780929	552.628632	10.43834772
774.703477	4.194712137	642.628632	10.51634244	552.912068	10.44855368
775.32988	4.202125646	642.912068	10.51609192	554.136603	10.42183465
775.928012	4.208285423	644.136603	10.51498993	554.703477	10.42132634
777.628632	4.223446366	644.703477	10.51446905	555.32988	10.4256072
777.912068	4.225131267	645.32988	10.5138856	555.928012	10.46116731
779.136603	4.233798441	645.928012	10.51332081	557.628632	10.39521585
779.703477	4.238988816	647.628632	10.51167446	557.912068	10.39643281
780.32988	4.245228427	647.912068	10.51139426	559.136603	10.40633123
780.928012	4.247664115	649.136603	10.51016489	559.703477	10.42442209
782.628632	4.260412701	649.703477	10.50958546	560.32988	10.38570374
782.912068	4.262981163	650.32988	10.50893766	560.928012	10.38246026
784.136603	4.27322291	650.928012	10.50831172	562.628632	10.38341025
784.703477	4.277840447	652.628632	10.50649308	562.912068	10.38662955
785.32988	4.282927591	652.912068	10.5061844	564.136603	10.38390423
785.928012	4.28777786	654.136603	10.50483275	564.703477	10.37291485
787.628632	4.301543703	654.703477	10.50419712	565.32988	10.37108717
787.912068	4.303828451	655.32988	10.50348749	565.928012	10.37097956
789.136603	4.312816032	655.928012	10.50280286	567.628632	10.3800587
789.703477	4.31656361	657.628632	10.50081888	567.912068	10.3998565
790.32988	4.321571011	657.912068	10.50048289	569.136603	10.34343382
790.928012	4.326503257	659.136603	10.49901391	569.703477	10.36236546
792.628632	4.340190048	659.703477	10.49832437	570.32988	10.33600451
792.912068	4.342441952	660.32988	10.49755548	570.928012	10.33868601
794.136603	4.352123008	660.928012	10.49681454	572.628632	10.37411681
794.703477	4.356583422	662.628632	10.49467206	572.912068	10.34553248
795.32988	4.361496442	662.912068	10.49430987	574.136603	10.31414854
795.928012	4.366168666	664.136603	10.49272842	574.703477	10.32233919
797.628632	4.379322256	664.703477	10.49198724	575.32988	10.31794562
797.912068	4.38150129	665.32988	10.49116156	575.928012	10.31985378
799.136603	4.390924202	665.928012	10.49036666	577.628632	10.32797572
799.703477	4.395295839	667.628632	10.48807227	577.912068	10.30577669
800.32988	4.400129851	667.912068	10.48768498	579.136603	10.30336994

Final State: j'=5		Final State: j'=4		Final State: j'=2	
E(cm⁻¹)	σ (Å²)	E(cm⁻¹)	σ (Å²)	E(cm⁻¹)	σ (Å²)
800.928012	4.404745324	669.136603	10.48599575	579.703477	10.30289674
802.628632	4.417846939	669.703477	10.48520503	580.32988	10.30320338
802.912068	4.420026156	670.32988	10.48432492	580.928012	10.31184289
804.136603	4.429423558	670.928012	10.48347831	582.628632	10.2862133
804.703477	4.433763651	672.628632	10.48103828	582.912068	10.28505645
805.32988	4.438551628	672.912068	10.48062691	584.136603	10.28119081
805.928012	4.443115754	674.136603	10.47883435	584.703477	10.27925078
807.628632	4.456053304	674.703477	10.47799614	585.32988	10.27725986
807.912068	4.458204331	675.32988	10.47706381	585.928012	10.27566246
809.136603	4.467481982	675.928012	10.47616759	587.628632	10.26789289
809.703477	4.471768746	677.628632	10.47358777	587.912068	10.26656703
810.32988	4.476499886	677.912068	10.47315328	589.136603	10.26329985
810.928012	4.481011832	679.136603	10.47126144	589.703477	10.2610489
812.628632	4.493809654	679.703477	10.47037762	590.32988	10.25315247
812.912068	4.495938135	680.32988	10.46939505	590.928012	10.25067257
814.136603	4.505118783	680.928012	10.4684511	592.628632	10.24399788
814.703477	4.509360376	682.628632	10.46573671	592.912068	10.24276648
815.32988	4.514041118	682.912068	10.46527994	594.136603	10.23739196
815.928012	4.518504427	684.136603	10.46329234	594.703477	10.23490864
817.628632	4.531161408	684.703477	10.46236442	595.32988	10.2321876
817.912068	4.533266131	685.32988	10.46133333	595.928012	10.22963281
819.136603	4.542343486	685.928012	10.46034319	597.628632	10.22275565
819.703477	4.546537039	687.628632	10.45749822	597.912068	10.22127836
820.32988	4.551164674	687.912068	10.45701979	599.136603	10.2148998
820.928012	4.555577272	689.136603	10.45493879	599.703477	10.21231379
822.628632	4.568090391	689.703477	10.45396775	600.32988	10.20951543
822.912068	4.570171177	690.32988	10.45288903	600.928012	10.20686299
824.136603	4.579145337	690.928012	10.45185344	602.628632	10.19936698
824.703477	4.583291211	692.628632	10.44887904	602.912068	10.19812348
825.32988	4.587866177	692.912068	10.44837898	604.136603	10.1927718
825.928012	4.592228498	694.136603	10.44620404	604.703477	10.19030261
827.628632	4.604598601	694.703477	10.44518914	605.32988	10.18757155
827.912068	4.606655537	695.32988	10.44406037	605.928012	10.18495217
829.136603	4.615526562	695.928012	10.44297895	607.628632	10.17744661
829.703477	4.619624631	697.628632	10.43986714	607.912068	10.17619593
830.32988	4.624146755	697.912068	10.43934344	609.136603	10.17081522
830.928012	4.628458559	699.136603	10.43706294	609.703477	10.16833724
832.628632	4.64068492	699.703477	10.43599662	610.32988	10.16560744
832.912068	4.64271789	700.32988	10.43480968	610.928012	10.16300846
834.136603	4.651485285	700.928012	10.43366699	612.628632	10.15565349
834.703477	4.65553537	702.628632	10.43035697	612.912068	10.15443194
835.32988	4.660004441	702.912068	10.42979461	614.136603	10.14916594
835.928012	4.664265597	704.136603	10.4273448	614.703477	10.14673368
837.628632	4.676347877	704.703477	10.42643272	615.32988	10.14404957
837.912068	4.678356833	705.32988	10.42862549	615.928012	10.14148995

Final State: j'=5		Final State: j'=4		Final State: j'=2	
E(cm⁻¹)	σ (Å²)	E(cm⁻¹)	σ (Å²)	E(cm⁻¹)	σ (Å²)
839.136603	4.687020447	705.928012	10.43095404	617.628632	10.13423107
839.703477	4.69102248	707.628632	10.42842293	617.912068	10.13302416
840.32988	4.695438437	707.912068	10.42497514	619.136603	10.12782018
840.928012	4.699648875	709.136603	10.4202492	619.703477	10.125417
842.628632	4.711586898	709.703477	10.42426593	620.32988	10.12276591
842.912068	4.713571805	710.32988	10.42145425	620.928012	10.12023901
844.136603	4.722131489	710.928012	10.41761088	622.628632	10.11307715
844.703477	4.726085398	712.628632	10.41443463	622.912068	10.11188685
845.32988	4.73044817	712.912068	10.41741414	624.136603	10.10675501
845.928012	4.734607825	714.136603	10.41104559	624.703477	10.10438506
847.628632	4.74640138	714.703477	10.41355825	625.32988	10.10177044
847.912068	4.748362189	715.32988	10.40741156	625.928012	10.09927785
849.136603	4.756817826	715.928012	10.40570798	627.628632	10.09221208
849.703477	4.760723549	717.628632	10.40612051	627.912068	10.09103746
850.32988	4.765033079	717.912068	10.4052599	629.136603	10.08597266
850.928012	4.769141893	719.136603	10.40024993	629.703477	10.08363341
852.628632	4.780790842	719.703477	10.39835191	630.32988	10.08105246
852.912068	4.782727553	720.32988	10.39671985	630.928012	10.07859186
854.136603	4.791079053	720.928012	10.39548743	632.628632	10.0716164
854.703477	4.794936564	722.628632	10.39530844	632.912068	10.07045675
855.32988	4.799192808	722.912068	10.40055226	634.136603	10.06545631
855.928012	4.803250734	724.136603	10.38946639	634.703477	10.0631467
857.628632	4.814755044	724.703477	10.38785351	635.32988	10.0605984
857.912068	4.816667629	725.32988	10.38627608	635.928012	10.05816882
859.136603	4.824914946	725.928012	10.38487536	637.628632	10.05128077
859.703477	4.828724226	727.628632	10.39005717	637.912068	10.05013558
860.32988	4.832927188	727.912068	10.38396837	639.136603	10.04519714
860.928012	4.836934213	729.136603	10.37955245	639.703477	10.04291601
862.628632	4.848293829	729.703477	10.37737421	640.32988	10.04039901
862.912068	4.850182304	730.32988	10.37568675	640.928012	10.03799914
864.136603	4.858325435	730.928012	10.3742023	642.628632	10.03119466
864.703477	4.862086505	732.628632	10.37049854	642.912068	10.03006326
865.32988	4.86623617	732.912068	10.37072599	644.136603	10.02518408
865.928012	4.870192316	734.136603	10.37074287	644.703477	10.02293014
867.628632	4.881407274	734.703477	10.37480694	645.32988	10.020443
867.912068	4.883271643	735.32988	10.3652104	645.928012	10.01807151
869.136603	4.891310625	735.928012	10.36321921	647.628632	10.01134686
869.703477	4.895023467	737.628632	10.35881143	647.912068	10.01022864
870.32988	4.899119875	737.912068	10.35813247	649.136603	10.005406
870.928012	4.903025148	739.136603	10.36111596	649.703477	10.00317799
872.628632	4.914095505	739.703477	10.35942194	650.32988	10.00071936
872.912068	4.915935791	740.32988	10.36959689	650.928012	9.998374939
874.136603	4.923870654	740.928012	10.36235185	652.628632	9.991726358
874.703477	4.927535324	742.628632	10.35972567	652.912068	9.9906207
875.32988	4.931578468	742.912068	10.35621037	654.136603	9.985851856

Final State: j'=5		Final State: j'=4		Final State: j'=2	
E(cm⁻¹)	σ (Å²)	E(cm⁻¹)	σ (Å²)	E(cm⁻¹)	σ (Å²)
875.928012	4.93543292	744.136603	10.34616799	654.703477	9.983648539
877.628632	4.946358748	744.703477	10.35546036	655.32988	9.981217013
877.912068	4.948174935	745.32988	10.35340263	655.928012	9.978898291
879.136603	4.95600576	745.928012	10.34327127	657.628632	9.972321886
879.703477	4.959622269	747.628632	10.3641082	657.912068	9.971228135
880.32988	4.963612204	747.912068	10.36412347	659.136603	9.966510298
880.928012	4.967415844	749.136603	10.33697647	659.703477	9.964330324
882.628632	4.978197225	749.703477	10.33344807	660.32988	9.961924451
882.912068	4.979989344	750.32988	10.3315239	660.928012	9.959630054
884.136603	4.987716204	750.928012	10.33775354	662.628632	9.953121905
884.703477	4.991284588	752.628632	10.32733434	662.912068	9.952039396
885.32988	4.995221317	752.912068	10.32624838	664.136603	9.947369649
885.928012	4.998974179	754.136603	10.32261694	664.703477	9.945211735
887.628632	5.009611191	754.703477	10.32396592	665.32988	9.94283
887.912068	5.011379262	755.32988	10.34276914	665.928012	9.940558504
889.136603	5.019002178	755.928012	10.32594796	667.628632	9.93411446
889.703477	5.022522427	757.628632	10.31941123	667.912068	9.933042489
890.32988	5.026406031	757.912068	10.3174104	669.136603	9.928417856
890.928012	5.030108121	759.136603	10.31734203	669.703477	9.926280553
892.628632	5.040600813	759.703477	10.33572437	670.32988	9.923921429
892.912068	5.042344818	760.32988	10.31161408	670.928012	9.921671297
894.136603	5.049863817	760.928012	10.30943149	672.628632	9.915287034
894.703477	5.053335953	762.628632	10.30337509	672.912068	9.914224887
895.32988	5.057166365	762.912068	10.31120839	674.136603	9.909642138
895.928012	5.060817693	764.136603	10.32760416	674.703477	9.907523961
897.628632	5.071165992	764.703477	10.30786711	675.32988	9.905185763
897.912068	5.072885928	765.32988	10.30906918	675.928012	9.902955439
899.136603	5.080300899	765.928012	10.29700973	677.628632	9.896626284
899.703477	5.083724873	767.628632	10.30647101	677.912068	9.895573137
900.32988	5.087502035	767.912068	10.29498441	679.136603	9.891028838
900.928012	5.091102495	769.136603	10.30622016	679.703477	9.888928144
902.628632	5.101306058	769.703477	10.29360162	680.32988	9.886609039
902.912068	5.103001853	770.32988	10.29116445	680.928012	9.884396686
904.136603	5.110312446	770.928012	10.28975512	682.628632	9.878117397
904.703477	5.113688036	772.628632	10.29170787	682.912068	9.877072384
905.32988	5.117411712	772.912068	10.29181702	684.136603	9.872562505
905.928012	5.120961057	774.136603	10.28455775	684.703477	9.87047738
907.628632	5.131018924	774.703477	10.28253281	685.32988	9.868175151
907.912068	5.132690386	775.32988	10.28108136	685.928012	9.865978665
909.136603	5.139895635	775.928012	10.2798065	687.628632	9.859742833
909.703477	5.143222318	777.628632	10.27694665	687.912068	9.858704816
910.32988	5.146891831	777.912068	10.27659412	689.136603	9.854224203
910.928012	5.150389317	779.136603	10.2738757	689.703477	9.852152091
912.628632	5.160298863	779.703477	10.27251967	690.32988	9.849863863
912.912068	5.161945456	780.32988	10.27123019	690.928012	9.847680255

Final State: j’=5		Final State: j’=4		Final State: j’=2	
E(cm⁻¹)	σ (Å²)	E(cm⁻¹)	σ (Å²)	E(cm⁻¹)	σ (Å²)
914.136603	5.169042529	780.928012	10.27314742	692.628632	9.841478455
914.703477	5.172318695	782.628632	10.26572343	692.912068	9.840445674
915.32988	5.175931964	782.912068	10.26514686	694.136603	9.835986095
915.928012	5.179375274	784.136603	10.26255876	694.703477	9.833922708
917.628632	5.189127324	784.703477	10.26132541	695.32988	9.831641408
917.912068	5.190746992	785.32988	10.25995335	695.928012	9.829467028
919.136603	5.197724588	785.928012	10.25864229	697.628632	9.823279677
919.703477	5.200943089	787.628632	10.25496665	697.912068	9.822248138
920.32988	5.204490226	787.912068	10.25437851	699.136603	9.81778854
920.928012	5.207867238	789.136603	10.25212838	699.703477	9.815721376
922.628632	5.217406166	789.703477	10.25081793	700.32988	9.813433935
922.912068	5.218989363	790.32988	10.24918136	700.928012	9.811245297
924.136603	5.228008708	790.928012	10.24773369	702.628632	9.804976898
924.703477	5.233591529	792.628632	10.24386435	702.912068	9.803920282
925.32988	5.234041247	792.912068	10.24323008	704.136603	9.799254116
925.928012	5.237964918	794.136603	10.24050214	704.703477	9.796974096
927.628632	5.245979107	794.703477	10.23924511	705.32988	9.795232801
927.912068	5.247954987	795.32988	10.23786085	705.928012	9.794433824
929.136603	5.257239104	795.928012	10.23654419	707.628632	9.788758042
929.703477	5.259704911	797.628632	10.23280965	707.912068	9.787367621
930.32988	5.26315252	797.912068	10.23218326	709.136603	9.781899493
930.928012	5.264690874	799.136603	10.22945731	709.703477	9.780771188
932.628632	5.275470474	799.703477	10.22818969	710.32988	9.779163768
932.912068	5.276876428	800.32988	10.22678868	710.928012	9.776752038
934.136603	5.282425694	800.928012	10.22545196	712.628632	9.769131945
934.703477	5.285258658	802.628632	10.22166011	712.912068	9.769000108
935.32988	5.288988692	802.912068	10.22102943	714.136603	9.765471945
935.928012	5.298526352	804.136603	10.21830855	714.703477	9.764084565
937.628632	5.304219249	804.703477	10.21705087	715.32988	9.760798872
937.912068	5.303270419	805.32988	10.21566226	715.928012	9.758063159
939.136603	5.309274208	805.928012	10.21433723	717.628632	9.754537753
939.703477	5.31230694	807.628632	10.21057275	717.912068	9.752707314
940.32988	5.318265832	807.912068	10.20994559	719.136603	9.74798765
940.928012	5.324799534	809.136603	10.20723668	719.703477	9.745406385
942.628632	5.328551389	809.703477	10.20598308	720.32988	9.742738612
942.912068	5.329798135	810.32988	10.2045983	720.928012	9.740059544
944.136603	5.335980561	810.928012	10.20327651	722.628632	9.736402015
944.703477	5.338935084	812.628632	10.19952201	722.912068	9.735928283
945.32988	5.342355511	812.912068	10.19889682	724.136603	9.730306206
945.928012	5.349913316	814.136603	10.19619804	724.703477	9.727904633
947.628632	5.357492243	814.703477	10.19494994	725.32988	9.725349737
947.912068	5.356652764	815.32988	10.19357166	725.928012	9.722863852
949.136603	5.362265972	815.928012	10.19225653	727.628632	9.720580952
949.703477	5.365150788	817.628632	10.18852225	727.912068	9.717372865
950.32988	5.368360962	817.912068	10.18790059	729.136603	9.713509057

Final State: j'=5		Final State: j'=4		Final State: j'=2	
E(cm⁻¹)	σ (Å²)	E(cm⁻¹)	σ (Å²)	E(cm⁻¹)	σ (Å²)
950.928012	5.371454979	819.136603	10.18521725	729.703477	9.710750718
952.628632	5.3847692	819.703477	10.18397637	730.32988	9.708169555
952.912068	5.38429279	820.32988	10.18260619	730.928012	9.705789295
954.136603	5.388259949	820.928012	10.18129885	732.628632	9.698633005
954.703477	5.390861936	822.628632	10.1775873	732.912068	9.697092544
955.32988	5.393941135	822.912068	10.17696951	734.136603	9.695597326
955.928012	5.396903531	824.136603	10.17430329	734.703477	9.695028663
957.628632	5.407516719	824.703477	10.17307054	735.32988	9.691375476
957.912068	5.410933399	825.32988	10.17170953	735.928012	9.688566378
959.136603	5.422613121	825.928012	10.17041112	737.628632	9.681524577
959.703477	5.423747251	827.628632	10.16672582	737.912068	9.680310475
960.32988	5.423376148	827.912068	10.16611256	739.136603	9.676677223
960.928012	5.425414755	829.136603	10.16346621	739.703477	9.674169955
962.628632	5.431965221	829.703477	10.16224292	740.32988	9.674171965
962.912068	5.43321011	830.32988	10.1608925	740.928012	9.673954909
964.136603	5.447126328	830.928012	10.15960437	742.628632	9.66875516
964.703477	5.443233481	832.628632	10.15594906	742.912068	9.667626797
965.32988	5.445491298	832.912068	10.15534089	744.136603	9.659102621
965.928012	5.448682567	834.136603	10.152717	744.703477	9.659063724
967.628632	5.459699724	834.703477	10.15150429	745.32988	9.659754674
967.912068	5.460890382	835.32988	10.15016573	745.928012	9.654339773
969.136603	5.463837909	835.928012	10.14888902	747.628632	9.649244692
969.703477	5.46976253	837.628632	10.14526709	747.912068	9.653972358
970.32988	5.48297801	837.912068	10.14466459	749.136603	9.645645963
970.928012	5.474270962	839.136603	10.14206555	749.703477	9.64151437
972.628632	5.480883165	839.703477	10.14086456	750.32988	9.638026595
972.912068	5.482224304	840.32988	10.13953906	750.928012	9.635781749
974.136603	5.502501396	840.928012	10.13827501	752.628632	9.631387373
974.703477	5.496661784	842.628632	10.13468974	752.912068	9.629965554
975.32988	5.497068442	842.912068	10.13409348	754.136603	9.623877161
975.928012	5.497739895	844.136603	10.13152169	754.703477	9.620006457
977.628632	5.510079243	844.703477	10.1303335	755.32988	9.623351965
977.912068	5.508490832	845.32988	10.12902231	755.928012	9.623500914
979.136603	5.514462872	845.928012	10.12777206	757.628632	9.614560954
979.703477	5.515368368	847.628632	10.12422675	757.912068	9.613221209
980.32988	5.527559874	847.912068	10.12363725	759.136603	9.605487022
980.928012	5.521738801	849.136603	10.121095	759.703477	9.613038261
982.628632	5.534477696	849.703477	10.11992068	760.32988	9.606374197
982.912068	5.542887371	850.32988	10.11862495	760.928012	9.601019677
984.136603	5.547089779	850.928012	10.11738959	762.628632	9.594246887
984.703477	5.5421889	852.628632	10.11388737	762.912068	9.594603377
985.32988	5.542241483	852.912068	10.11330514	764.136603	9.595916026
985.928012	5.54644055	854.136603	10.11079469	764.703477	9.592580291
987.628632	5.562478768	854.703477	10.10963528	765.32988	9.592397984
987.912068	5.565502084	855.32988	10.10835615	765.928012	9.586397537

Final State: j'=5		Final State: j'=4		Final State: j'=2	
E(cm⁻¹)	σ (Å²)	E(cm⁻¹)	σ (Å²)	E(cm⁻¹)	σ (Å²)
989.136603	5.56392046	855.928012	10.10713676	767.628632	9.582991452
989.703477	5.566553632	857.628632	10.10368065	767.912068	9.578642601
990.32988	5.569791611	857.912068	10.10310623	769.136603	9.577408065
990.928012	5.573109014	859.136603	10.10062975	769.703477	9.573892278
992.628632	5.582721224	859.703477	10.09948621	770.32988	9.570891235
992.912068	5.582942587	860.32988	10.09822478	770.928012	9.568317142
994.136603	5.588082648	860.928012	10.09702243	772.628632	9.56263631
994.703477	5.590773265	862.628632	10.09361538	772.912068	9.5620685
995.32988	5.593775989	862.912068	10.09304921	774.136603	9.558906391
995.928012	5.596715918	864.136603	10.09060879	774.703477	9.556324214
997.628632	5.605065065	864.703477	10.08948213	775.32988	9.553889267
997.912068	5.606332714	865.32988	10.08823944	775.928012	9.551671076
999.136603	5.61197488	865.928012	10.08705511	777.628632	9.545619609
999.703477	5.615508664	867.628632	10.08369999	777.912068	9.544710353
1000.32988	5.618363009	867.912068	10.08314256	779.136603	9.540652538
1000.92801	5.620003156	869.136603	10.0807402	779.703477	9.538624425
1002.62863	5.627923158	869.703477	10.07963134	780.32988	9.536297824
1002.91207	5.629230055	870.32988	10.07840843	780.928012	9.534501487
1004.1366	5.634845388	870.928012	10.0772431	782.628632	9.528907825
1004.70348	5.637435578	872.628632	10.07394263	782.912068	9.527889913
1005.32988	5.640294916	872.912068	10.0733944	784.136603	9.523611815
1005.92801	5.643024995	874.136603	10.07103206	784.703477	9.521648669
1007.62863	5.650879019	874.703477	10.06994189	785.32988	9.519480759
1007.91207	5.652213499	875.32988	10.06873974	785.928012	9.517410564
1009.1366	5.657628039	875.928012	10.06759437	787.628632	9.511518583
1009.70348	5.66012832	877.628632	10.06435116	787.912068	9.510535989
1010.32988	5.662916699	877.912068	10.06381258	789.136603	9.506416518
1010.92801	5.66558658	879.136603	10.06149217	789.703477	9.50458028
1012.62863	5.67317404	879.703477	10.06042152	790.32988	9.502418932
1012.91207	5.674436633	880.32988	10.0592411	790.928012	9.500327796
1014.1366	5.679884321	880.928012	10.05811656	792.628632	9.494419519
1014.70348	5.682402606	882.628632	10.0549332	792.912068	9.493437818
1015.32988	5.68518249	882.912068	10.05440468	794.136603	9.48919964
1015.92801	5.687832861	884.136603	10.05212794	794.703477	9.487238576
1017.62863	5.695332051	884.703477	10.05107769	795.32988	9.48507232
1017.91207	5.696576502	885.32988	10.04991987	795.928012	9.483005157
1019.1366	5.701940331	885.928012	10.04881704	797.628632	9.47714006
1019.70348	5.704417603	887.628632	10.04569593	797.912068	9.476163491
1020.32988	5.707151154	887.912068	10.04517786	799.136603	9.471938921
1020.92801	5.709757606	889.136603	10.04294655	799.703477	9.469979315
1022.62863	5.717148211	889.703477	10.04191747	800.32988	9.46781155
1022.91207	5.718377066	890.32988	10.04078315	800.928012	9.465739875
1024.1366	5.723676253	890.928012	10.03970287	802.628632	9.459843378
1024.70348	5.726123901	892.628632	10.03664635	802.912068	9.458859945
1025.32988	5.728824415	892.912068	10.0361391	804.136603	9.454609347

Final State: j'=5		Final State: j'=4		Final State: j'=2	
E(cm⁻¹)	σ (Å²)	E(cm⁻¹)	σ (Å²)	E(cm⁻¹)	σ (Å²)
1025.92801	5.731398881	894.136603	10.03395486	804.703477	9.452640736
1027.62863	5.738696162	894.703477	10.03294771	805.32988	9.450464764
1029.1366	5.74513902	895.32988	10.03183771	805.928012	9.448386381
1029.70348	5.747554369	895.928012	10.03078072	807.628632	9.442473459
1030.32988	5.750219188	897.628632	10.02779103	807.912068	9.441487341
1030.92801	5.752759684	897.912068	10.027295	809.136603	9.437224669
1032.62863	5.759961456	899.136603	10.02515941	809.703477	9.435249951
1034.1366	5.766321179	899.703477	10.0241749	810.32988	9.433066803
1034.70348	5.768705578	900.32988	10.02309002	810.928012	9.430981105
1035.32988	5.771336357	900.928012	10.0220571	812.628632	9.425045298
1035.92801	5.773844469	902.628632	10.01913633	812.912068	9.42405519
1037.62863	5.780954712	902.912068	10.01865184	814.136603	9.419774868
1039.1366	5.787233758	904.136603	10.01656641	814.703477	9.417791929
1039.70348	5.789587932	904.703477	10.01560523	815.32988	9.415599675
1040.32988	5.792185393	905.32988	10.01454623	815.928012	9.413505292
1040.92801	5.794661767	905.928012	10.01353811	817.628632	9.407544771
1042.62863	5.801682206	907.628632	10.01068829	817.912068	9.40655052
1044.1366	5.807882255	907.912068	10.0102157	819.136603	9.402252223
1044.70348	5.810206894	909.136603	10.00818188	819.703477	9.400260868
1045.32988	5.81277181	909.703477	10.00724469	820.32988	9.398059228
1045.92801	5.81521723	910.32988	10.00621232	820.928012	9.395955814
1047.62863	5.822150188	910.928012	10.0052297	822.628632	9.389969054
1049.1366	5.828273329	912.628632	10.00245289	822.912068	9.388970363
1049.70348	5.83056922	912.912068	10.00199254	824.136603	9.384652628
1050.32988	5.833102476	914.136603	10.00001182	824.703477	9.382652121
1050.92801	5.835517734	914.703477	9.999099374	825.32988	9.380440313
1052.62863	5.842365545	915.32988	9.998094436	825.928012	9.378327089
1054.1366	5.848413805	915.928012	9.997138125	827.628632	9.372312067
1054.70348	5.850681703	917.628632	9.994436863	827.912068	9.37130861
1055.32988	5.853184134	917.912068	9.993989233	829.136603	9.366970117
1055.92801	5.855570073	919.136603	9.992063985	829.703477	9.364959947
1057.62863	5.862334985	919.703477	9.991177598	830.32988	9.362737304
1059.1366	5.868310432	920.32988	9.990201848	830.928012	9.360613724
1059.70348	5.87055111	920.928012	9.989273951	832.628632	9.354568867
1060.32988	5.873023574	922.628632	9.986659874	832.912068	9.353560394
1060.92801	5.875380994	922.912068	9.986228853	834.136603	9.349200019
1062.62863	5.882065366	924.136603	9.984205346	834.703477	9.347179591
1064.1366	5.887970058	924.703477	9.983088555	835.32988	9.34494563
1064.70348	5.890184306	925.32988	9.982346635	835.928012	9.342811118
1065.32988	5.892627659	925.928012	9.981508603	837.628632	9.336734893
1065.92801	5.894957386	927.628632	9.979103815	837.912068	9.33572114
1067.62863	5.901563572	927.912068	9.978715707	839.136603	9.331337819
1069.1366	5.907399619	929.136603	9.976564249	839.703477	9.329306698
1069.70348	5.909588221	929.703477	9.975869234	840.32988	9.327060832
1070.32988	5.91200334	930.32988	9.97468518	840.928012	9.324914897

Final State: j’=5		Final State: j’=4		Final State: j’=2	
E(cm⁻¹)	σ (Å²)	E(cm⁻¹)	σ (Å²)	E(cm⁻¹)	σ (Å²)
1070.92801	5.914306215	930.928012	9.974089075	842.628632	9.318805933
1072.62863	5.920836613	932.628632	9.971501554	842.912068	9.317786658
1074.1366	5.926606088	932.912068	9.971241594	844.136603	9.313379413
1074.70348	5.928769846	934.136603	9.969462868	844.703477	9.311337146
1075.32988	5.931157618	934.703477	9.968651447	845.32988	9.3090789
1075.92801	5.933434472	935.32988	9.967696685	845.928012	9.306921117
1077.62863	5.939891443	935.928012	9.966309772	847.628632	9.300778126
1079.1366	5.945596513	937.628632	9.964405696	847.912068	9.299753152
1079.70348	5.947736227	937.912068	9.964150893	849.136603	9.295321138
1080.32988	5.950097536	939.136603	9.962434449	849.703477	9.293267297
1080.92801	5.95234924	939.703477	9.961620571	850.32988	9.290996264
1084.1366	5.964377993	940.32988	9.960472851	850.928012	9.288826207
1084.70348	5.966494471	940.928012	9.959892482	852.628632	9.282648069
1085.32988	5.968830198	942.628632	9.957738398	852.912068	9.281617213
1085.92801	5.971057566	942.912068	9.957346419	854.136603	9.277159621
1089.1366	5.982957623	944.136603	9.955663834	854.703477	9.275093914
1089.70348	5.985051507	944.703477	9.954880205	855.32988	9.272809692
1090.32988	5.987362694	945.32988	9.953979465	855.928012	9.270627004
1090.92801	5.989566605	945.928012	9.952824429	857.628632	9.26441276
1094.1366	6.00134252	947.628632	9.950808197	857.912068	9.263375846
1094.70348	6.003414929	947.912068	9.950855557	859.136603	9.25889202
1095.32988	6.005702178	949.136603	9.949190591	859.703477	9.256814126
1095.92801	6.007883473	949.703477	9.948441919	860.32988	9.254516399
1099.1366	6.019539809	950.32988	9.947615524	860.928012	9.25232078
1099.70348	6.021591404	950.928012	9.946816901	862.628632	9.24606955
1100.92801	6.0260153	952.628632	9.945006811	862.912068	9.245026443
1104.1366	6.037556654	952.912068	9.944393828	864.136603	9.240515816
1104.70348	6.039588238	954.136603	9.9430957	864.703477	9.238425468
1105.92801	6.04396921	954.703477	9.942328948	865.32988	9.236113936
1109.1366	6.055400163	955.32988	9.941536267	865.928012	9.233905113
1109.70348	6.057412533	955.928012	9.940800255	867.628632	9.22761622
1110.92801	6.061752334	957.628632	9.938672363	867.912068	9.226566799
1114.1366	6.073077427	957.912068	9.938251782	869.136603	9.222028877
1114.70348	6.075071393	959.136603	9.936252526	869.703477	9.219925871
1115.92801	6.079371762	959.703477	9.935944719	870.32988	9.217600311
1119.1366	6.090595505	960.32988	9.935307457	870.928012	9.215378086
1119.70348	6.092571886	960.928012	9.934925351	872.628632	9.209050909
1120.92801	6.096834531	962.628632	9.932649166	872.912068	9.207995098
1124.1366	6.107961475	962.912068	9.932377677	874.136603	9.203429517
1124.70348	6.109921043	964.136603	9.93033169	874.703477	9.201313657
1125.92801	6.114147698	964.703477	9.930000471	875.32988	9.198973907
1129.1366	6.125182284	965.32988	9.929371635	875.928012	9.196738078
1129.70348	6.12712583	965.928012	9.928624918	877.628632	9.190372186
1130.92801	6.131318195	967.628632	9.926978903	877.912068	9.189309921
1134.1366	6.142264863	967.912068	9.926567645	879.136603	9.184716368

Final State: j'=5		Final State: j'=4		Final State: j'=2	
E(cm⁻¹)	σ (Å²)	E(cm⁻¹)	σ (Å²)	E(cm⁻¹)	σ (Å²)
1134.70348	6.144193177	969.136603	9.925452022	879.703477	9.18258753
1135.92801	6.148352894	969.703477	9.924693758	880.32988	9.180233436
1139.1366	6.159215989	970.32988	9.923323635	880.928012	9.177983897
1139.70348	6.161129811	970.928012	9.923315631	882.628632	9.171578974
1140.92801	6.16525856	972.628632	9.921556098	882.912068	9.170510202
1144.1366	6.176042319	972.912068	9.921233062	884.136603	9.165888439
1144.70348	6.177942409	974.136603	9.918739253	884.703477	9.163746492
1145.92801	6.182041753	974.703477	9.920000648	885.32988	9.161378013
1149.1366	6.19275026	975.32988	9.918447286	885.928012	9.159114674
1149.70348	6.194637321	975.928012	9.918903864	887.628632	9.152670482
1150.92801	6.198708767	977.628632	9.916259317	887.912068	9.151595149
1154.1366	6.209345806	977.912068	9.915911269	889.136603	9.146945086
1154.70348	6.211220466	979.136603	9.914853232	889.703477	9.144790089
1155.92801	6.21526535	979.703477	9.914667869	890.32988	9.142407069
1159.1366	6.225833988	980.32988	9.913518054	890.928012	9.140129904
1159.70348	6.227696703	980.928012	9.912959453	892.628632	9.133646347
1160.92801	6.231715864	982.628632	9.910637125	892.912068	9.132564458
1164.1366	6.242216659	982.912068	9.91031748	894.136603	9.127886054
1165.92801	6.248058752	984.136603	9.910646534	894.703477	9.125717928
1169.1366	6.258458482	984.703477	9.909426021	895.32988	9.123320408
1170.92801	6.263729504	985.32988	9.909157506	895.928012	9.121029377
1174.1366	6.27452216	985.928012	9.90850509	897.628632	9.114506449
1175.92801	6.280287902	987.628632	9.906215076	897.912068	9.113418012
1179.1366	6.290631995	987.912068	9.905823255	899.136603	9.108711272
1180.92801	6.296603288	989.136603	9.905461802	899.703477	9.106530031
1184.1366	6.307066487	989.703477	9.904872836	900.32988	9.104118008
1185.92801	6.312596818	990.32988	9.904204658	900.928012	9.101813152
1189.1366	6.323098776	990.928012	9.90346168	902.628632	9.095250907
1190.92801	6.328657139	992.628632	9.902537126	902.912068	9.094155893
1194.1366	6.33918833	992.912068	9.902341805	904.136603	9.089420853
1195.92801	6.344680122	994.136603	9.901130905	904.703477	9.087226525
1199.1366	6.354641083	994.703477	9.900611856	905.32988	9.084800033
1200.92801	6.360660568	995.32988	9.900046822	905.928012	9.082481351
1204.1366	6.36993032	995.928012	9.899508152	907.628632	9.075879775
1205.92801	6.375716881	997.628632	9.898110593	907.912068	9.074778213
1209.1366	6.386363528	997.912068	9.897884503	909.136603	9.070014804
1210.92801	6.392070726	999.136603	9.896841059	909.703477	9.067807321
1214.1366	6.402450943	999.703477	9.896170729	910.32988	9.06536627
1215.92801	6.407104155	1000.32988	9.896154248	910.928012	9.063033681
1219.1366	6.417835652	1000.92801	9.895690445	912.628632	9.056392382
1220.92801	6.42408752	1002.62863	9.894256718	912.912068	9.055284169
1224.1366	6.433027424	1002.91207	9.894037504	914.136603	9.050491875
1225.92801	6.439288582	1004.1366	9.893105913	914.703477	9.04827092
1229.1366	6.448068675	1004.70348	9.892680565	915.32988	9.045814875
1230.92801	6.455205695	1005.32988	9.892213875	915.928012	9.043467835

Final State: j'=5		Final State: j'=4		Final State: j'=2	
E(cm⁻¹)	σ (Å²)	E(cm⁻¹)	σ (Å²)	E(cm⁻¹)	σ (Å²)
1234.1366	6.46392932	1005.92801	9.891770892	917.628632	9.036784436
1235.92801	6.470173409	1007.62863	9.890522963	917.912068	9.035668996
1239.1366	6.480346627	1007.91207	9.89032853	919.136603	9.030844399
1240.92801	6.485759198	1009.1366	9.889528443	919.703477	9.028607562
1244.1366	6.495692169	1009.70348	9.889135341	920.32988	9.026132954
1245.92801	6.501101743	1010.32988	9.888701715	920.928012	9.023766643
1249.1366	6.511301982	1010.92801	9.888291487	922.628632	9.017007681
1250.92801	6.516870873	1012.62863	9.887146409	922.912068	9.015871342
1254.1366	6.526845824	1012.91207	9.886958444	924.136603	9.01081972
1255.92801	6.532454447	1014.1366	9.886155567	924.703477	9.008652222
1259.1366	6.54243681	1014.70348	9.885789058	925.32988	9.006427422
1260.92801	6.548012066	1015.32988	9.885388253	925.928012	9.003779319
1264.1366	6.558005594	1015.92801	9.88501003	927.628632	8.99720644
1265.92801	6.56358564	1017.62863	9.8839578	927.912068	8.995926053
1269.1366	6.573578873	1017.91207	9.883785154	929.136603	8.991336607
1270.92801	6.579159062	1019.1366	9.883046849	929.703477	8.988951285
1275.92801	6.594739298	1019.70348	9.882709307	930.32988	8.98663201
1280.92801	6.610327329	1020.32988	9.882339556	930.928012	8.984197661
1285.92801	6.625924963	1020.92801	9.881989764	932.628632	8.977444337
1290.92801	6.641533877	1022.62863	9.881013077	932.912068	8.976076723
1295.92801	6.657155251	1022.91207	9.88085291	934.136603	8.971467107
1300.92801	6.672790107	1024.1366	9.88016961	934.703477	8.969144628
1305.92801	6.688439171	1024.70348	9.879858088	935.32988	8.966548293
1310.92801	6.704102854	1025.32988	9.879517396	935.928012	8.96417438
1315.92801	6.719781361	1025.92801	9.879195542	937.628632	8.957489494
1320.92801	6.735474596	1027.62863	9.878298784	937.912068	8.956404736
1325.92801	6.751182198	1029.1366	9.877525952	939.136603	8.95139532
1330.92801	6.766903549	1029.70348	9.877240788	939.703477	8.949081065
1335.92801	6.782637754	1030.32988	9.87692906	940.32988	8.946443152
1340.92801	6.79838367	1030.92801	9.876634732	940.928012	8.943980814
1345.92801	6.814139915	1032.62863	9.875815484	942.628632	8.937440118
1350.92801	6.829904828	1034.1366	9.875110787	942.912068	8.936265446
1355.92801	6.845676545	1034.70348	9.87485117	944.136603	8.931268634
1360.92801	6.861452946	1035.32988	9.874567613	944.703477	8.92896176
1365.92801	6.877231665	1035.92801	9.874300113	945.32988	8.926407938
1370.92801	6.893010143	1037.62863	9.873556987	945.928012	8.92391808
1375.92801	6.908785622	1039.1366	9.872919426	947.628632	8.917107402
1380.92801	6.92455511	1039.70348	9.872684929	947.912068	8.916097862
1385.92801	6.940315452	1040.32988	9.872429066	949.136603	8.911037375
1390.92801	6.956063305	1040.92801	9.872187967	949.703477	8.908722948
1395.92801	6.971795127	1042.62863	9.871519432	950.32988	8.906167074
1400.92801	6.987507333	1044.1366	9.870947525	950.928012	8.903726462
1405.92801	7.003196052	1044.70348	9.870737577	952.628632	8.896636596
		1045.32988	9.870508782	952.912068	8.895311704
		1045.92801	9.870293433	954.136603	8.890710921

Final State: j’=5		Final State: j’=4		Final State: j’=2	
E(cm⁻¹)	σ (Å²)	E(cm⁻¹)	σ (Å²)	E(cm⁻¹)	σ (Å²)
		1047.62863	9.869697726	954.703477	8.888349742
		1049.1366	9.869189903	955.32988	8.88576349
		1049.70348	9.869003909	955.928012	8.883291385
		1050.32988	9.868801525	957.628632	8.876023631
		1050.92801	9.8686113	957.912068	8.874734787
		1052.62863	9.86808662	959.136603	8.869809119
		1054.1366	9.867641227	959.703477	8.867510491
		1054.70348	9.867478589	960.32988	8.86522152
		1055.32988	9.86730188	960.928012	8.862572552
		1055.92801	9.867136122	962.628632	8.855997751
		1057.62863	9.866680535	962.912068	8.854743727
		1059.1366	9.866295814	964.136603	8.849749278
		1059.70348	9.866155848	964.703477	8.847657792
		1060.32988	9.866004128	965.32988	8.844965948
		1060.92801	9.865862111	965.928012	8.842383263
		1062.62863	9.865473559	967.628632	8.835600053
		1064.1366	9.865147693	967.912068	8.834396503
		1064.70348	9.865029695	969.136603	8.829233149
		1065.32988	9.864902149	969.703477	8.826532437
		1065.92801	9.864783142	970.32988	8.824325659
		1067.62863	9.864459458	970.928012	8.822108677
		1069.1366	9.864190508	972.628632	8.814878445
		1069.70348	9.864093739	972.912068	8.81368197
		1070.32988	9.863989568	974.136603	8.808744567
		1070.92801	9.863892782	974.703477	8.806108326
		1072.62863	9.863631751	975.32988	8.80343432
		1074.1366	9.863417676	975.928012	8.801072987
		1074.70348	9.863341393	977.628632	8.793918003
		1075.32988	9.863259746	977.912068	8.792889382
		1075.92801	9.863184347	979.136603	8.788134194
		1077.62863	9.862983622	979.703477	8.78543248
		1079.1366	9.862822357	980.32988	8.782573392
		1079.70348	9.862765733	980.928012	8.780651476
		1080.32988	9.862705706	982.628632	8.7734035
		1080.92801	9.862650857	982.912068	8.771517199
		1084.1366	9.862397336	984.136603	8.766783739
		1084.70348	9.862359581	984.703477	8.765053907
		1085.32988	9.862320276	985.32988	8.762202924
		1085.92801	9.862285104	985.928012	8.75947449
		1089.1366	9.862135281	987.628632	8.752412141
		1089.70348	9.862114614	987.912068	8.751130134
		1090.32988	9.86209599	989.136603	8.746209138
		1090.92801	9.86207959	989.703477	8.743840854
		1094.1366	9.862028532	990.32988	8.741224849
		1094.70348	9.862025886	990.928012	8.738741035

Final State: j'=5		Final State: j'=4		Final State: j'=2	
E(cm⁻¹)	σ (Å²)	E(cm⁻¹)	σ (Å²)	E(cm⁻¹)	σ (Å²)
		1095.32988	9.862025134	992.628632	8.731591501
		1095.92801	9.862026563	992.912068	8.730404467
		1099.1366	9.86206923	994.136603	8.725310359
		1099.70348	9.862082796	994.703477	8.722941613
		1100.92801	9.862118153	995.32988	8.720322352
		1104.1366	9.862249312	995.928012	8.717818712
		1104.70348	9.862278161	997.628632	8.710691497
		1105.92801	9.862346175	997.912068	8.709506279
		1109.1366	9.862560516	999.136603	8.704384528
		1109.70348	9.862603714	999.703477	8.702000327
		1110.92801	9.862702317	1000.32988	8.699307343
		1114.1366	9.862994412	1000.92801	8.696875085
		1114.70348	9.863050969	1002.62863	8.689742525
		1115.92801	9.863178076	1002.91207	8.688553613
		1119.1366	9.863542393	1004.1366	8.683416284
		1119.70348	9.863611318	1004.70348	8.681037446
		1120.92801	9.863764784	1005.32988	8.67840821
		1124.1366	9.864195664	1005.92801	8.675896892
		1124.70348	9.864275991	1007.62863	8.668748244
		1125.92801	9.864453665	1007.91207	8.667553536
		1129.1366	9.864945407	1009.1366	8.662410641
		1129.70348	9.865036085	1009.70348	8.660031481
		1130.92801	9.865235768	1010.32988	8.657400277
		1134.1366	9.865782526	1010.92801	8.654886622
		1134.70348	9.865882545	1012.62863	8.647736375
		1135.92801	9.86610198	1012.91207	8.646544271
		1139.1366	9.866697883	1014.1366	8.641392887
		1139.70348	9.866806169	1014.70348	8.639007522
		1140.92801	9.867043066	1015.32988	8.636371152
		1144.1366	9.867682046	1015.92801	8.633853392
		1144.70348	9.867797565	1017.62863	8.626693976
		1145.92801	9.8680496	1017.91207	8.625500665
		1149.1366	9.868725488	1019.1366	8.620344802
		1149.70348	9.868847077	1019.70348	8.61795767
		1150.92801	9.869111832	1020.32988	8.615319626
		1154.1366	9.869818054	1020.92801	8.612800368
		1154.70348	9.869944563	1022.62863	8.605636364
		1155.92801	9.870219389	1022.91207	8.604442184
		1159.1366	9.870948591	1024.1366	8.599282515
		1159.70348	9.871078551	1024.70348	8.596893732
		1160.92801	9.871360224	1025.32988	8.59425389
		1164.1366	9.872101618	1025.92801	8.591733092
		1165.92801	9.872514218	1027.62863	8.584565264
		1169.1366	9.873215369	1029.1366	8.57820886
		1170.92801	9.87394589	1029.70348	8.57581926

Final State: j’=5		Final State: j’=4		Final State: j’=2	
E(cm⁻¹)	σ (Å²)	E(cm⁻¹)	σ (Å²)	E(cm⁻¹)	σ (Å²)
		1174.1366	9.874415979	1030.32988	8.573178654
		1175.92801	9.875148163	1030.92801	8.570657206
		1179.1366	9.875834029	1032.62863	8.563487973
		1180.92801	9.876223121	1034.1366	8.557130828
		1184.1366	9.877133759	1034.70348	8.554741091
		1185.92801	9.877401259	1035.32988	8.552100422
		1189.1366	9.878295301	1035.92801	8.549578958
		1190.92801	9.878623668	1037.62863	8.542410194
		1194.1366	9.879523568	1039.1366	8.536054043
		1195.92801	9.879804138	1039.70348	8.533664793
		1199.1366	9.880738284	1040.32988	8.531024777
		1200.92801	9.880893002	1040.92801	8.528504022
		1204.1366	9.881588547	1042.62863	8.521337759
		1205.92801	9.882574386	1044.1366	8.514984402
		1209.1366	9.882662867	1044.70348	8.512596343
		1210.92801	9.883353045	1045.32988	8.509957727
		1214.1366	9.884119645	1045.92801	8.507438396
		1215.92801	9.883918482	1047.62863	8.500276625
		1219.1366	9.884641875	1049.1366	8.49392777
		1220.92801	9.885267366	1049.70348	8.491541558
		1224.1366	9.885484314	1050.32988	8.488905052
		1225.92801	9.885749074	1050.92801	8.486387845
		1229.1366	9.886558471	1052.62863	8.479232484
		1230.92801	9.887266853	1054.1366	8.472889915
		1234.1366	9.887577548	1054.70348	8.470506185
		1235.92801	9.887640574	1055.32988	8.467872498
		1239.1366	9.888120394	1055.92801	8.46535807
		1240.92801	9.88830425	1057.62863	8.458211111
		1244.1366	9.888678761	1059.1366	8.451876482
		1245.92801	9.888860908	1059.70348	8.449495904
		1249.1366	9.889156735	1060.32988	8.446865767
		1250.92801	9.889304309	1060.92801	8.444354799
		1254.1366	9.889533485	1062.62863	8.437218102
		1255.92801	9.889640274	1064.1366	8.430893122
		1259.1366	9.889797029	1064.70348	8.428516284
		1260.92801	9.889863681	1065.32988	8.425890382
		1264.1366	9.889944583	1065.92801	8.423383526
		1265.92801	9.88996825	1067.62863	8.416258965
		1269.1366	9.889971139	1069.1366	8.409945274
		1270.92801	9.889950267	1069.70348	8.407572806
		1275.92801	9.889804955	1070.32988	8.404951806
		1280.92801	9.889528494	1070.92801	8.402449717
		1285.92801	9.88911719	1072.62863	8.395339114
		1290.92801	9.888567753	1074.1366	8.389038303
		1295.92801	9.887877288	1074.70348	8.386670816

Final State: j'=5		Final State: j'=4		Final State: j'=2	
E(cm⁻¹)	σ (Å²)	E(cm⁻¹)	σ (Å²)	E(cm⁻¹)	σ (Å²)
		1300.92801	9.887043211	1075.32988	8.384055371
		1305.92801	9.886063369	1075.92801	8.381558682
		1310.92801	9.88493594	1077.62863	8.374463823
		1315.92801	9.883659462	1079.1366	8.368177469
		1320.92801	9.882232898	1079.70348	8.365815518
		1325.92801	9.8806555	1080.32988	8.363206297
		1330.92801	9.878926994	1080.92801	8.360715616
		1335.92801	9.877047318	1084.1366	8.347367811
		1340.92801	9.875016864	1084.70348	8.345011975
		1345.92801	9.872836359	1085.32988	8.342409588
		1350.92801	9.870506858	1085.92801	8.339925497
		1355.92801	9.868029709	1089.1366	8.326614301
		1360.92801	9.865406632	1089.70348	8.32426508
		1365.92801	9.862639612	1090.32988	8.321670199
		1370.92801	9.859730947	1090.92801	8.319193284
		1375.92801	9.856683221	1094.1366	8.305921681
		1380.92801	9.85349926	1094.70348	8.303579715
		1385.92801	9.850182176	1095.32988	8.300992806
		1390.92801	9.846735301	1095.92801	8.298523605
		1395.92801	9.843162203	1099.1366	8.285294585
		1400.92801	9.839466688	1099.70348	8.282960327
		1405.92801	9.835652705	1100.92801	8.277921043
				1104.1366	8.264737367
				1104.70348	8.262411307
				1105.92801	8.257389939
				1109.1366	8.244254319
				1109.70348	8.241936915
				1110.92801	8.236934408
				1114.1366	8.223849392
				1114.70348	8.221541088
				1115.92801	8.216558443
				1119.1366	8.203526466
				1119.70348	8.2012277
				1120.92801	8.196265788
				1124.1366	8.183289153
				1124.70348	8.18100032
				1125.92801	8.176059976
				1129.1366	8.163140851
				1129.70348	8.16086232
				1130.92801	8.155944377
				1134.1366	8.143084806
				1134.70348	8.140816896
				1135.92801	8.135922098
				1139.1366	8.123123955
				1139.70348	8.120867031

Final State: j’=5		Final State: j’=4		Final State: j’=2	
E(cm⁻¹)	σ (Å²)	E(cm⁻¹)	σ (Å²)	E(cm⁻¹)	σ (Å²)
				1140.92801	8.115996075
				1144.1366	8.10326115
				1144.70348	8.101015511
				1145.92801	8.096169029
				1149.1366	8.083498978
				1149.70348	8.081264924
				1150.92801	8.076443543
				1154.1366	8.063840009
				1154.70348	8.06161781
				1155.92801	8.056822158
				1159.1366	8.044286857
				1159.70348	8.042076836
				1160.92801	8.037307695
				1164.1366	8.024843294
				1165.92801	8.017905409
				1169.1366	8.00552994
				1170.92801	7.998652909
				1174.1366	7.986327436
				1175.92801	7.979421767
				1179.1366	7.96717002
				1180.92801	7.96029419
				1184.1366	7.948076064
				1185.92801	7.941322856
				1189.1366	7.929191799
				1190.92801	7.922463302
				1194.1366	7.910395984
				1195.92801	7.903727006
				1199.1366	7.891731653
				1200.92801	7.885117107
				1204.1366	7.873361597
				1205.92801	7.86662795
				1209.1366	7.854777233
				1210.92801	7.848114505
				1214.1366	7.83634969
				1215.92801	7.829939763
				1219.1366	7.818191489
				1220.92801	7.811722515
				1224.1366	7.800069022
				1225.92801	7.79368709
				1229.1366	7.782143899
				1230.92801	7.775624696
				1234.1366	7.76425569
				1235.92801	7.757848963
				1239.1366	7.746467071
				1240.92801	7.740111175

Final State: j’=5		Final State: j’=4		Final State: j’=2	
E(cm⁻¹)	σ (Å²)	E(cm⁻¹)	σ (Å²)	E(cm⁻¹)	σ (Å²)
				1244.1366	7.728780558
				1245.92801	7.72245966
				1249.1366	7.711228098
				1250.92801	7.70496391
				1254.1366	7.693778643
				1255.92801	7.687555225
				1259.1366	7.676438764
				1260.92801	7.670251283
				1264.1366	7.659202851
				1265.92801	7.653053018
				1269.1366	7.642071152
				1270.92801	7.635958256
				1275.92801	7.61896594
				1280.92801	7.602074785
				1285.92801	7.585283589
				1290.92801	7.568591023
				1295.92801	7.551995962
				1300.92801	7.53549725
				1305.92801	7.519093705
				1310.92801	7.502784287
				1315.92801	7.486567967
				1320.92801	7.470443862
				1325.92801	7.454411092
				1330.92801	7.438468878
				1335.92801	7.422616556
				1340.92801	7.406853566
				1345.92801	7.391179402
				1350.92801	7.37559369
				1355.92801	7.360096206
				1360.92801	7.34468679
				1365.92801	7.329365393
				1370.92801	7.314132092
				1375.92801	7.298987092
				1380.92801	7.283930714
				1385.92801	7.26896337
				1390.92801	7.254085619
				1395.92801	7.239297976
				1400.92801	7.224601489
				1405.92801	7.209996723

Final State: j’=1		Initial rotational state: j=3<br>Final State: j’=0	
E(cm⁻¹)	σ (Å²)	E(cm⁻¹)	σ (Å²)
164.903477	214.8688056	164.803477	31.16337568
165.003477	94.79899408	164.903477	57.67006761
165.103477	81.1650833	165.003477	25.19649964
165.203477	71.66124494	165.103477	21.96490657
165.303477	70.06717958	165.203477	19.67599837
165.32988	74.34427165	165.303477	19.53311282
165.403477	75.55445771	165.32988	20.84702082
165.503477	65.92352986	165.403477	20.60724013
165.603477	60.03808374	165.503477	18.09521594
165.703477	61.56911181	165.603477	16.7598833
165.803477	53.86803897	165.703477	17.31699071
165.903477	49.98078989	165.803477	14.8438115
167.628632	37.94829229	165.903477	13.87830518
167.912068	34.63159011	167.628632	11.74624395
169.703477	23.18312278	167.912068	10.81551085
169.803477	22.91419918	169.703477	7.4704704
169.903477	22.62578782	169.803477	7.40598238
170.003477	22.31390309	169.903477	7.3316592
170.103477	21.97342113	170.003477	7.24631774
170.203477	21.59537516	170.103477	7.14834358
170.303477	21.16514234	170.203477	7.03427695
170.32988	21.04173266	170.303477	6.8964674
170.403477	20.69502043	170.32988	6.85470404
172.628632	18.27244451	170.403477	6.72766924
172.912068	17.55846878	172.628632	6.31969069
174.703477	15.0294675	172.912068	6.04077527
175.32988	14.77279874	174.703477	5.22126787
177.628632	13.2926222	175.32988	5.278954
177.912068	12.9997541	177.628632	4.90883334
179.703477	13.43272572	177.912068	4.78146094
180.32988	11.54967362	179.703477	5.46245406
182.628632	10.4759645	180.32988	4.27938158
182.912068	10.35969335	182.628632	3.90042612
184.703477	9.700398777	182.912068	3.85668161
185.32988	9.508593012	184.703477	3.6104141
187.628632	9.365888079	185.32988	3.54535739
187.912068	9.238561451	187.628632	3.78959812
189.703477	8.509839377	187.912068	3.70196045
190.32988	8.347365001	189.703477	3.24295716
192.628632	7.871871345	190.32988	3.16375454
192.912068	7.822280361	192.628632	2.958004988
194.703477	7.55216663	192.912068	2.938556465
195.32988	7.472224602	194.703477	2.847315043
197.628632	7.171652843	195.32988	2.825645137

Final State: j'=1		Final State: j'=0	
E(cm⁻¹)	σ (Å²)	E(cm⁻¹)	σ (Å²)
197.912068	7.13377187	197.628632	2.716218857
199.703477	6.906245361	197.912068	2.699723593
200.32988	6.832560316	199.703477	2.599723585
202.628632	6.586888448	200.32988	2.567988203
202.912068	6.558977838	202.628632	2.467212517
204.703477	6.39161755	202.912068	2.456358012
205.32988	6.33584239	204.703477	2.393751908
207.628632	6.136418802	205.32988	2.373633485
207.912068	6.111904648	207.628632	2.304090544
209.703477	5.952136013	207.912068	2.295917469
210.32988	5.891592008	209.703477	2.246843499
212.628632	5.782205876	210.32988	2.231023116
212.912068	5.887571094	212.628632	2.164071788
214.703477	6.054549564	212.912068	2.145599174
215.32988	6.032987876	214.703477	2.088762418
217.628632	5.941355874	215.32988	2.069448344
217.912068	6.159915155	217.628632	2.004066687
219.703477	5.629268718	217.912068	1.999029404
220.32988	5.856448186	219.703477	1.993086079
222.628632	5.721524981	220.32988	1.950933086
222.912068	5.627618915	222.628632	1.926776713
224.703477	5.957815226	222.912068	1.931185363
225.32988	5.687766906	224.703477	1.858576758
227.628632	5.432758963	225.32988	1.886962699
227.912068	5.390395783	227.628632	1.865135393
229.703477	6.02782094	227.912068	1.86325125
230.32988	5.371714896	229.703477	1.797724588
232.628632	5.326261029	230.32988	1.820256125
232.912068	5.292205256	232.628632	1.800331926
234.703477	5.551339961	232.912068	1.798662852
235.32988	5.793131255	234.703477	1.730805219
237.628632	5.27509714	235.32988	1.740382821
237.912068	5.241498426	237.628632	1.743460009
239.703477	5.070506873	237.912068	1.742609837
240.32988	5.053620015	239.703477	1.731136373
242.628632	5.447000257	240.32988	1.716916785
242.912068	5.26527519	242.628632	1.679385723
244.703477	4.98947056	242.912068	1.691453814
245.32988	4.917288419	244.703477	1.695736084
247.628632	5.46000426	245.32988	1.696083521
247.912068	5.599324012	247.628632	1.610610369
249.703477	5.307184903	247.912068	1.606270299
250.32988	5.254653989	249.703477	1.627964581
252.628632	5.315574611	250.32988	1.610326049
252.912068	5.292068044	252.628632	1.588021646

Final State: j'=1		Final State: j'=0	
E(cm^{-1})	σ (Å^2)	E(cm^{-1})	σ (Å^2)
254.703477	4.896975332	252.912068	1.578190696
255.32988	5.761585821	254.703477	1.596737313
257.628632	5.005091118	255.32988	1.528610084
257.912068	4.948494956	257.628632	1.585529427
259.703477	5.20065881	257.912068	1.587974538
260.32988	5.045939064	259.703477	1.5448814
262.628632	5.349729133	260.32988	1.557096918
262.912068	5.548835709	262.628632	1.521358685
264.703477	5.10112482	262.912068	1.480993482
265.32988	5.035944061	264.703477	1.546109967
267.628632	5.378117932	265.32988	1.544368245
267.912068	5.140903884	267.628632	1.482753107
269.703477	4.936938965	267.912068	1.506298049
270.32988	4.787507954	269.703477	1.50920856
272.628632	5.870404192	270.32988	1.527478052
272.912068	5.401169832	272.628632	1.450482949
274.236603	4.851994667	272.912068	1.476939091
274.336603	4.844460153	274.236603	1.466560384
274.436603	4.816808688	274.336603	1.436102508
274.536603	4.775103839	274.436603	1.45199393
274.636603	4.748114754	274.536603	1.451333276
274.703477	4.780894513	274.636603	1.450711345
274.736603	4.880101417	274.703477	1.447721645
274.836603	5.174366113	274.736603	1.438919397
274.936603	5.015596915	274.836603	1.393186469
275.036603	4.899970705	274.936603	1.404003043
275.136603	5.094128308	275.036603	1.415968581
275.236603	5.01270259	275.136603	1.388730136
275.32988	4.924870541	275.236603	1.393317584
275.336603	4.920658228	275.32988	1.401953567
275.436603	4.873053262	275.336603	1.402304164
275.536603	4.840727354	275.436603	1.405589022
277.628632	4.879237942	275.536603	1.406914841
277.912068	4.852842782	277.628632	1.343852452
279.136603	4.774106283	277.912068	1.347373809
279.236603	4.76955324	279.136603	1.332312999
279.336603	4.765301694	279.236603	1.330243218
279.436603	4.76164646	279.336603	1.328127794
279.536603	4.759442222	279.436603	1.325868293
279.636603	4.761163489	279.536603	1.323245229
279.703477	4.767926059	279.636603	1.319770641
279.736603	4.774773539	279.703477	1.316427195
279.836603	4.830111385	279.736603	1.314221111
279.936603	5.011172669	279.836603	1.303150305
280.036603	5.231352057	279.936603	1.281583116

Final State: j'=1		Final State: j'=0	
E(cm^{-1})	σ (Å^2)	E(cm^{-1})	σ (Å^2)
280.32988	4.949067353	280.036603	1.26938521
282.628632	4.844771323	280.32988	1.302952459
282.912068	4.835929861	282.628632	1.289782443
284.136603	4.810034143	282.912068	1.288750511
284.703477	4.80112492	284.136603	1.27961643
285.32988	4.799819904	284.703477	1.270301702
287.628632	4.804333509	285.32988	1.252978736
287.912068	4.800584887	287.628632	1.241947748
289.136603	4.895908501	287.912068	1.239613592
289.703477	4.851890878	289.136603	1.19240558
290.32988	4.83272921	289.703477	1.230738513
292.628632	4.825345072	290.32988	1.228198913
292.912068	4.825280665	292.628632	1.214651828
294.136603	4.825265307	292.912068	1.213222921
294.703477	4.825305501	294.136603	1.206923706
295.32988	4.825358414	294.703477	1.203734785
297.628632	4.841484416	295.32988	1.199543741
297.912068	4.841683003	297.628632	1.177947737
299.136603	4.841897629	297.912068	1.17914103
299.703477	4.842490516	299.136603	1.178633976
300.32988	4.84341172	299.703477	1.176968248
302.628632	4.848130367	300.32988	1.17477639
302.912068	4.848829232	302.628632	1.165374014
304.136603	4.852282072	302.912068	1.164089071
304.703477	4.85416793	304.136603	1.158130566
305.32988	4.856408976	304.703477	1.15524166
307.628632	4.864489228	305.32988	1.152169056
307.912068	4.865438333	307.628632	1.142951221
309.136603	4.86953442	307.912068	1.141926756
309.703477	4.871453174	309.136603	1.137511754
310.32988	4.873603545	309.703477	1.135452978
312.628632	4.881854205	310.32988	1.133160549
312.912068	4.882915506	312.628632	1.124595813
314.136603	4.887611267	312.912068	1.123528273
314.703477	4.889841295	314.136603	1.118919695
315.32988	4.892341823	314.703477	1.116800255
317.628632	4.901789537	315.32988	1.114477266
317.912068	4.902980793	317.628632	1.106164347
319.136603	4.908191462	317.912068	1.105161631
319.703477	4.910639323	319.136603	1.100875346
320.32988	4.913371123	319.703477	1.098912946
322.628632	4.923645954	320.32988	1.096758136
322.912068	4.924940598	322.628632	1.088954603
324.136603	4.930604591	322.912068	1.088002799
324.703477	4.933265493	324.136603	1.083916673

Final State: j'=1		Final State: j'=0	
E(cm⁻¹)	σ (Å²)	E(cm⁻¹)	σ (Å²)
325.32988	4.93623431	324.703477	1.082039789
327.628632	4.947382832	325.32988	1.079977091
327.912068	4.948784858	327.628632	1.072515009
329.136603	4.954911065	327.912068	1.071607092
329.703477	4.957785102	329.136603	1.067715895
330.32988	4.960989197	329.703477	1.065931806
332.628632	4.973006122	330.32988	1.063973088
332.912068	4.974516639	332.628632	1.056898644
334.136603	4.981118086	332.912068	1.056038704
334.703477	4.984216726	334.136603	1.052354802
335.32988	4.987673244	334.703477	1.050666807
337.628632	5.000669429	335.32988	1.048814574
337.912068	5.002308242	337.628632	1.042139231
339.136603	5.009490057	337.912068	1.04133011
339.703477	5.012874625	339.136603	1.03787263
340.32988	5.016662934	339.703477	1.036294278
342.628632	5.031079794	340.32988	1.034567881
342.912068	5.032924593	342.628632	1.028419129
344.136603	5.041115111	342.912068	1.027685417
344.703477	5.045051636	344.136603	1.024598029
345.32988	5.0495358	344.703477	1.023225255
347.628632	5.067793111	345.32988	1.021764695
347.912068	5.07027527	347.628632	1.017494643
349.136603	5.072229572	347.912068	1.017260673
349.703477	5.036328078	349.136603	1.021113951
350.32988	5.009080514	349.703477	1.026265196
352.628632	5.066982366	350.32988	1.021004488
352.912068	5.07974987	352.628632	1.004907502
354.136603	5.021724603	352.912068	1.005210128
354.703477	5.027918676	354.136603	1.015518217
355.32988	5.072418374	354.703477	1.007766587
357.628632	5.029832358	355.32988	0.998554274
357.912068	5.056949126	357.628632	0.998923612
359.136603	5.056408762	357.912068	0.993372339
359.703477	5.102542352	359.136603	0.987807731
360.32988	5.116986925	359.703477	0.983827993
362.628632	5.045919963	360.32988	0.986276163
362.912068	5.048466828	362.628632	0.998073643
364.136603	5.133569951	362.912068	0.980119481
364.703477	5.144116578	364.136603	0.971525458
365.32988	5.148887635	364.703477	0.972691462
367.628632	5.070671753	365.32988	0.9774642
367.912068	5.118002664	367.628632	0.968892365
369.136603	5.167044739	367.912068	0.958743128
369.703477	5.176253195	369.136603	0.958285624

Final State: j'=1		Final State: j'=0	
E(cm⁻¹)	σ (Å²)	E(cm⁻¹)	σ (Å²)
370.32988	5.18447745	369.703477	0.95876117
372.628632	5.125389421	370.32988	0.960449711
372.912068	5.109866579	372.628632	0.969426045
374.136603	5.195904385	372.912068	0.957311446
374.703477	5.206540283	374.136603	0.944427991
375.32988	5.215841023	374.703477	0.944575611
377.628632	5.159769299	375.32988	0.944734616
377.912068	5.147222029	377.628632	0.970870484
379.136603	5.179119066	377.912068	0.954487392
379.703477	5.231657061	379.136603	0.939918518
380.32988	5.250623373	379.703477	0.930550697
382.628632	5.282818447	380.32988	0.931636931
382.912068	5.26719012	382.628632	0.938367642
384.136603	5.23793679	382.912068	0.942982254
384.703477	5.137882586	384.136603	0.951937488
385.32988	5.207753142	384.703477	0.957004678
387.628632	5.245542318	385.32988	0.946285637
387.912068	5.270595402	387.628632	0.920742945
389.136603	5.203867825	387.912068	0.9203796
389.703477	5.198712819	389.136603	0.941377749
390.32988	5.283764294	389.703477	0.925402736
392.628632	5.1766865	390.32988	0.917173694
392.912068	5.147248956	392.628632	0.922088215
394.136603	5.322087863	392.912068	0.931519649
394.703477	5.338971	394.136603	0.903291819
395.32988	5.262543319	394.703477	0.909143992
397.628632	5.353772652	395.32988	0.933052857
397.912068	5.361560538	397.628632	0.899596249
399.136603	5.328389562	397.912068	0.900719349
399.703477	5.211940119	399.136603	0.929213759
400.32988	5.29232133	399.703477	0.934582775
402.628632	5.358128237	400.32988	0.897075558
402.912068	5.350596636	402.628632	0.902838754
404.136603	5.196128677	402.912068	0.903311652
404.703477	5.354735719	404.136603	0.912566821
405.32988	5.372352119	404.703477	0.882731291
407.628632	5.330082451	405.32988	0.900779647
407.912068	5.390280638	407.628632	0.909345192
409.136603	5.354501957	407.912068	0.905431261
409.703477	5.307843185	409.136603	0.882116482
410.32988	5.368043352	409.703477	0.889608614
411.028012	5.433395148	410.32988	0.872725923
411.128012	5.400077406	411.028012	0.874684523
411.228012	5.425696407	411.128012	0.882066178
411.328012	5.430597982	411.228012	0.87754899

Final State: j’=1		Final State: j’=0	
E(cm⁻¹)	σ (Å²)	E(cm⁻¹)	σ (Å²)
411.428012	5.429885126	411.328012	0.879990827
411.528012	5.383380963	411.428012	0.884894502
411.628012	5.325734131	411.528012	0.898811812
411.728012	5.335585704	411.628012	0.897778535
411.828012	5.382168338	411.728012	0.883709353
411.928012	5.320960263	411.828012	0.882549461
412.028012	5.353679275	411.928012	0.895113734
412.128012	5.383798733	412.028012	0.877936138
412.228012	5.395590404	412.128012	0.876010555
412.328012	5.402046846	412.228012	0.876462974
412.428012	5.40630398	412.328012	0.877335613
412.528012	5.409419825	412.428012	0.878414659
412.628632	5.411753525	412.528012	0.879736267
412.912068	5.407975648	412.628632	0.881455605
414.136603	5.372610354	412.912068	0.892590273
414.703477	5.390350004	414.136603	0.872076178
415.32988	5.39825275	414.703477	0.872586482
415.928012	5.399755043	415.32988	0.874145035
416.028012	5.399798998	415.928012	0.876371384
416.128012	5.399798023	416.028012	0.876901314
416.228012	5.399696991	416.128012	0.877544582
416.328012	5.399354182	416.228012	0.878366566
416.428012	5.398404923	416.328012	0.879483263
416.528012	5.395746222	416.428012	0.881123525
416.628012	5.387000182	416.528012	0.883805388
416.728012	5.360969383	416.628012	0.888751585
416.828012	5.348551458	416.728012	0.89413842
416.928012	5.362432762	416.828012	0.893103953
417.028012	5.377979991	416.928012	0.884259608
417.628632	5.350963345	417.028012	0.880884174
417.912068	5.348992232	417.628632	0.873877502
419.136603	5.399262882	417.912068	0.870480394
419.703477	5.404396614	419.136603	0.865548964
420.32988	5.408381518	419.703477	0.865727421
420.928012	5.411165897	420.32988	0.865722899
422.628632	5.408513483	420.928012	0.865757972
422.912068	5.409684168	422.628632	0.865445232
424.136603	5.418587478	422.912068	0.86473162
424.703477	5.421517385	424.136603	0.862817564
425.32988	5.396573227	424.703477	0.863141974
425.928012	5.40560485	425.32988	0.870300182
427.628632	5.434985809	425.928012	0.858503652
427.912068	5.436232673	427.628632	0.855827033
429.136603	5.441849602	427.912068	0.855557765
429.703477	5.444531241	429.136603	0.854103895

Final State: j’=1		Final State: j’=0	
E(cm⁻¹)	σ (Å²)	E(cm⁻¹)	σ (Å²)
430.32988	5.447489252	429.703477	0.853377587
430.928012	5.450272604	430.32988	0.852577167
432.628632	5.457140645	430.928012	0.851830291
432.912068	5.457668548	432.628632	0.850066155
434.136603	5.460682901	432.912068	0.849893409
434.703477	5.464888038	434.136603	0.847625973
435.32988	5.468863466	434.703477	0.84635832
435.928012	5.472166155	435.32988	0.845336333
437.628632	5.480663422	435.928012	0.844475344
437.912068	5.482025185	437.628632	0.84214379
439.136603	5.487802765	437.912068	0.841761236
439.703477	5.490415998	439.136603	0.840126472
440.32988	5.49324964	439.703477	0.839380882
440.928012	5.495903856	440.32988	0.838563866
442.628632	5.503453576	440.928012	0.83778427
442.912068	5.504750447	442.628632	0.835488778
444.136603	5.510454503	442.912068	0.835094085
444.703477	5.513115586	444.136603	0.833390343
445.32988	5.516052164	444.703477	0.832611237
445.928012	5.518846669	445.32988	0.831759288
447.628632	5.52672534	445.928012	0.830953984
447.912068	5.528028378	447.628632	0.828700942
449.136603	5.533626257	447.912068	0.828329886
449.703477	5.536201786	449.136603	0.82673851
450.32988	5.539038081	449.703477	0.826007185
450.928012	5.541738663	450.32988	0.825202135
452.628632	5.549389781	450.928012	0.824435929
452.912068	5.550662183	452.628632	0.82226877
454.136603	5.556151538	452.912068	0.82190922
454.703477	5.558687966	454.136603	0.820362132
455.32988	5.561486693	454.703477	0.819649752
455.928012	5.564154622	455.32988	0.818865648
457.628632	5.571712797	455.928012	0.818120089
457.912068	5.572968203	457.628632	0.816017774
459.136603	5.578377102	457.912068	0.815669931
459.703477	5.580872725	459.136603	0.814175415
460.32988	5.583624265	459.703477	0.813488047
460.928012	5.586245627	460.32988	0.812731729
462.628632	5.593667233	460.928012	0.81201265
462.912068	5.594899726	462.628632	0.809984451
464.136603	5.600210116	462.912068	0.809648712
464.703477	5.602660641	464.136603	0.808205663
465.32988	5.605362813	464.703477	0.807541702
465.928012	5.607937433	465.32988	0.806810997
467.628632	5.615227865	465.928012	0.806116194

Final State: j’=1		Final State: j’=0	
E(cm⁻¹)	σ (Å²)	E(cm⁻¹)	σ (Å²)
467.912068	5.616438641	467.628632	0.804156309
469.136603	5.621655358	467.912068	0.803831905
469.703477	5.624062508	469.136603	0.802437736
470.32988	5.626716658	469.703477	0.801796371
470.928012	5.629245333	470.32988	0.801090625
472.628632	5.636404542	470.928012	0.800419639
472.912068	5.637593372	472.628632	0.798527296
474.136603	5.642715158	472.912068	0.79821411
474.703477	5.645078309	474.136603	0.796868296
475.32988	5.647683823	474.703477	0.796249232
475.928012	5.650166093	475.32988	0.795568054
477.628632	5.657193649	475.928012	0.794920453
477.912068	5.658360597	477.628632	0.793094201
479.136603	5.663388074	477.912068	0.792791981
479.703477	5.665707735	479.136603	0.791493337
480.32988	5.66826532	479.703477	0.790896012
480.928012	5.67070196	480.32988	0.790238789
482.628632	5.67760061	480.928012	0.789614007
482.912068	5.678746204	482.628632	0.787852321
484.136603	5.683681934	482.912068	0.787560819
484.703477	5.685959408	484.136603	0.78630833
485.32988	5.688470634	484.703477	0.785732298
485.928012	5.690863267	485.32988	0.785098557
487.628632	5.697638329	485.928012	0.784496132
487.912068	5.698763586	487.628632	0.782797748
489.136603	5.703612385	487.912068	0.782516756
489.703477	5.705850196	489.136603	0.781309546
490.32988	5.708318086	489.703477	0.780754406
490.928012	5.710669831	490.32988	0.780143701
492.628632	5.717331811	490.928012	0.779563219
492.912068	5.718438719	492.628632	0.777926953
494.136603	5.723210241	492.912068	0.777656282
494.703477	5.72541349	494.136603	0.776493522
495.32988	5.727844163	494.703477	0.775958902
495.928012	5.730161458	495.32988	0.775370814
497.628632	5.736732183	495.928012	0.774811893
497.912068	5.737825025	497.628632	0.7732367
499.136603	5.74254016	497.912068	0.772976175
499.703477	5.744720106	499.136603	0.771857165
500.32988	5.747127442	499.703477	0.771342741
500.928012	5.749425128	500.32988	0.770776942
502.628632	5.755957916	500.928012	0.770239263
502.912068	5.757047551	502.628632	0.768724345
504.136603	5.761762321	502.912068	0.768473839
504.703477	5.76395103	504.136603	0.76739811

Final State: j’=1		Final State: j’=0	
E(cm⁻¹)	σ (Å²)	E(cm⁻¹)	σ (Å²)
505.32988	5.766376386	504.703477	0.76690371
505.928012	5.768701103	505.32988	0.766360035
507.628632	5.775385812	505.928012	0.765843496
507.912068	5.776515832	507.628632	0.764388828
509.136603	5.781484795	507.912068	0.764148412
509.703477	5.783854714	509.136603	0.763116547
510.32988	5.786555816	509.703477	0.762642692
510.928012	5.789258414	510.32988	0.762121993
512.628632	5.798885165	510.928012	0.761627777
512.912068	5.801441987	512.628632	0.760238412
514.136603	5.817965244	512.912068	0.76000634
514.703477	5.807257181	514.136603	0.758682262
515.32988	5.81133829	514.703477	0.758276656
515.928012	5.818605609	515.32988	0.757910663
517.628632	5.826110175	515.928012	0.757216342
517.912068	5.829249986	517.628632	0.756091044
519.136603	5.821756027	517.912068	0.755721432
519.703477	5.82606975	519.136603	0.754808985
520.32988	5.82071618	519.703477	0.754409049
520.928012	5.830116063	520.32988	0.753969196
522.628632	5.836198707	520.928012	0.75354984
522.912068	5.844182388	522.628632	0.75228611
524.136603	5.83207197	522.912068	0.751742584
524.703477	5.839513529	524.136603	0.751166175
525.32988	5.856105563	524.703477	0.750750233
525.928012	5.844215375	525.32988	0.750111339
527.628632	5.837553789	525.928012	0.750136619
527.912068	5.839955988	527.628632	0.748659698
529.136603	5.850583068	527.912068	0.748460293
529.703477	5.860830996	529.136603	0.747573539
530.32988	5.8662315	529.703477	0.747178022
530.928012	5.856107065	530.32988	0.746882898
532.628632	5.85310843	530.928012	0.746518952
532.912068	5.855201806	532.628632	0.745217767
534.136603	5.863772825	532.912068	0.745016369
534.703477	5.869182087	534.136603	0.744153138
535.32988	5.88351677	534.703477	0.74375506
535.928012	5.880968879	535.32988	0.74337687
537.628632	5.865242994	535.928012	0.743362965
537.912068	5.86806616	537.628632	0.742000621
539.136603	5.876375551	537.912068	0.741793874
539.703477	5.879844569	539.136603	0.740955927
540.32988	5.884121263	539.703477	0.740579854
540.928012	5.889772576	540.32988	0.740166648
542.628632	5.895096199	540.928012	0.739776191

Final State: j'=1		Final State: j'=0	
E(cm⁻¹)	σ (Å²)	E(cm⁻¹)	σ (Å²)
542.912068	5.903776509	542.628632	0.738742892
544.136603	5.889691753	542.912068	0.738988516
544.703477	5.894167329	544.136603	0.738025316
545.32988	5.898699053	544.703477	0.737666101
545.928012	5.903454318	545.32988	0.737296898
547.628632	5.93166174	545.928012	0.736967152
547.912068	5.92751736	547.628632	0.735484234
549.136603	5.930300774	547.912068	0.735126063
549.703477	5.920463733	549.136603	0.735042615
550.32988	5.915516848	549.703477	0.734774346
550.928012	5.936983733	550.32988	0.734422075
552.628632	5.928456359	550.928012	0.733589289
552.912068	5.943512915	552.628632	0.73289942
554.136603	5.918513202	552.912068	0.732771442
554.703477	5.925086539	554.136603	0.731389479
555.32988	5.937750586	554.703477	0.73137089
555.928012	5.977112913	555.32988	0.731234741
557.628632	5.91685578	555.928012	0.731547709
557.912068	5.922768561	557.628632	0.730110116
559.136603	5.949277648	557.912068	0.72990213
559.703477	5.973746367	559.136603	0.729501487
560.32988	5.926678081	559.703477	0.728754905
560.928012	5.932089948	560.32988	0.728286584
562.628632	5.952236084	560.928012	0.72821449
562.912068	5.959217976	562.628632	0.727569648
564.136603	5.947344166	562.912068	0.727490403
564.703477	5.95791514	564.136603	0.727841206
565.32988	5.955660654	564.703477	0.726465572
565.928012	5.967111856	565.32988	0.726799862
567.628632	5.989909101	565.928012	0.726178681
567.912068	6.013179703	567.628632	0.725448516
569.136603	5.958529323	567.912068	0.725472412
569.703477	5.989539445	569.136603	0.724432982
570.32988	5.958833599	569.703477	0.72342865
570.928012	5.969812683	570.32988	0.723223514
572.628632	6.007834865	570.928012	0.723487789
572.912068	5.985501078	572.628632	0.723390611
574.136603	5.963602646	572.912068	0.723158353
574.703477	5.982361186	574.136603	0.722151879
575.32988	5.983490488	574.703477	0.722054062
575.928012	5.986617704	575.32988	0.721383687
577.628632	6.002282949	575.928012	0.721044349
577.912068	5.984735794	577.628632	0.721096566
579.136603	5.995455872	577.912068	0.720513842
579.703477	5.999693104	579.136603	0.71973692

Final State: j’=1		Final State: j’=0	
E(cm⁻¹)	σ (Å²)	E(cm⁻¹)	σ (Å²)
580.32988	6.004805571	579.703477	0.719494087
580.928012	6.011121528	580.32988	0.719250122
582.628632	6.001498882	580.928012	0.719698972
582.912068	6.003143216	582.628632	0.718513304
584.136603	6.008918279	582.912068	0.718343215
584.703477	6.011132812	584.136603	0.717805312
585.32988	6.013616214	584.703477	0.717581344
585.928012	6.016044952	585.32988	0.717345946
587.628632	6.019595505	585.928012	0.717146423
587.912068	6.020303074	587.628632	0.716575061
589.136603	6.025347088	587.912068	0.716467383
589.703477	6.024561587	589.136603	0.716080311
590.32988	6.022972671	589.703477	0.716020604
590.928012	6.025433173	590.32988	0.715681354
592.628632	6.030372336	590.928012	0.71539378
592.912068	6.031070677	592.628632	0.71481599
594.136603	6.034018086	592.912068	0.714721551
594.703477	6.035370754	594.136603	0.714317484
595.32988	6.036869205	594.703477	0.714133437
595.928012	6.038314905	595.32988	0.713933065
597.628632	6.042256582	595.928012	0.713745808
597.912068	6.042631629	597.628632	0.7132757
599.136603	6.044840421	597.912068	0.71318985
599.703477	6.046100317	599.136603	0.712769733
600.32988	6.047494506	599.703477	0.712590709
600.928012	6.048814832	600.32988	0.712399512
602.628632	6.052515146	600.928012	0.712220875
602.912068	6.053125692	602.628632	0.71172747
604.136603	6.05574387	602.912068	0.711647096
604.703477	6.056941522	604.136603	0.711306071
605.32988	6.058248456	604.703477	0.711151636
605.928012	6.059477289	605.32988	0.710983141
607.628632	6.062908003	605.928012	0.710823627
607.912068	6.063477242	607.628632	0.710374096
609.136603	6.065934702	607.912068	0.710300057
609.703477	6.067070119	609.136603	0.709984737
610.32988	6.06832022	609.703477	0.709841501
610.928012	6.069510188	610.32988	0.709685238
612.628632	6.072866652	610.928012	0.709538025
612.912068	6.073421735	612.628632	0.709129898
614.136603	6.075804414	612.912068	0.709063346
614.703477	6.076898632	614.136603	0.70878048
615.32988	6.078101284	614.703477	0.708652029
615.928012	6.079243388	615.32988	0.708511867
617.628632	6.082458923	615.928012	0.708379753

Final State: j'=1		Final State: j'=0	
E(cm⁻¹)	σ (Å²)	E(cm⁻¹)	σ (Å²)
617.912068	6.08299047	617.628632	0.708013239
619.136603	6.085273194	617.912068	0.707953469
619.703477	6.086322457	619.136603	0.707699579
620.32988	6.087476451	619.703477	0.707584448
620.928012	6.088572921	620.32988	0.707459005
622.628632	6.091661948	620.928012	0.707340959
622.912068	6.09217261	622.628632	0.70701462
624.136603	6.094365086	622.912068	0.706961561
624.703477	6.095372445	624.136603	0.706736689
625.32988	6.096480004	624.703477	0.706634965
625.928012	6.097532086	625.32988	0.706524308
627.628632	6.100494166	625.928012	0.706420339
627.912068	6.100983666	627.628632	0.706133777
629.136603	6.103084735	627.912068	0.70608731
629.703477	6.104049879	629.136603	0.705890787
630.32988	6.105110853	629.703477	0.705802127
630.928012	6.106118585	630.32988	0.705705859
632.628632	6.108955138	630.928012	0.705615599
632.912068	6.10942378	632.628632	0.705367825
634.136603	6.111435068	632.912068	0.705327794
634.703477	6.112358787	634.136603	0.705159009
635.32988	6.113374066	634.703477	0.70508315
635.928012	6.114338242	635.32988	0.705000989
637.628632	6.117051351	635.928012	0.70492417
637.912068	6.117499485	637.628632	0.704714414
639.136603	6.119422298	637.912068	0.704680698
639.703477	6.120305152	639.136603	0.704539087
640.32988	6.121275366	639.703477	0.704475755
640.928012	6.122196562	640.32988	0.704407403
642.628632	6.124787909	640.928012	0.704343727
642.912068	6.125215802	642.628632	0.704171163
644.136603	6.127051392	642.912068	0.704143619
644.703477	6.127893969	644.136603	0.704028569
645.32988	6.128819778	644.703477	0.703977485
645.928012	6.129698621	645.32988	0.70392263
647.628632	6.132170017	645.928012	0.703871807
647.912068	6.132577992	647.628632	0.703735589
649.136603	6.134327667	647.912068	0.703714072
649.703477	6.135130586	649.136603	0.703624987
650.32988	6.136012632	649.703477	0.703585866
650.928012	6.136849792	650.32988	0.703544199
652.628632	6.139203058	650.928012	0.703505932
652.912068	6.139591396	652.628632	0.703405212
654.136603	6.141256465	652.912068	0.703389585
654.703477	6.142020335	654.136603	0.703325847

Final State: j’=1		Final State: j’=0	
E(cm⁻¹)	σ (Å²)	E(cm⁻¹)	σ (Å²)
655.32988	6.142859279	654.703477	0.703298411
655.928012	6.143655392	655.32988	0.703269612
657.628632	6.145892292	655.928012	0.703243598
657.912068	6.146261288	657.628632	0.703177519
659.136603	6.147843006	657.912068	0.703167636
659.703477	6.148568363	659.136603	0.703128619
660.32988	6.149364878	659.703477	0.703112578
660.928012	6.150120515	660.32988	0.703096332
662.628632	6.152242723	660.928012	0.703082267
662.912068	6.152592658	662.628632	0.70304996
664.136603	6.154092194	662.912068	0.703045673
664.703477	6.154779607	664.136603	0.703030754
665.32988	6.155534228	664.703477	0.703025814
665.928012	6.156249935	665.32988	0.703021801
667.628632	6.158258962	665.928012	0.703019376
667.912068	6.158590082	667.628632	0.703019963
669.136603	6.160008421	667.912068	0.703021131
669.703477	6.160658318	669.136603	0.703029676
670.32988	6.161371556	669.703477	0.703035549
670.928012	6.162047786	670.32988	0.703043444
672.628632	6.163944759	670.928012	0.703052351
672.912068	6.164257245	672.628632	0.703084954
674.136603	6.165595139	672.912068	0.703091431
674.703477	6.166207822	674.136603	0.703122814
675.32988	6.166879962	674.703477	0.703139208
675.928012	6.167516972	675.32988	0.703158685
677.628632	6.169302505	675.928012	0.703178623
677.912068	6.169596399	677.628632	0.703242389
679.136603	6.170854008	677.912068	0.703254027
679.703477	6.171429524	679.136603	0.703307632
680.32988	6.172060542	679.703477	0.703334262
680.928012	6.172658249	680.32988	0.70336502
682.628632	6.174331738	680.928012	0.703395687
682.912068	6.17460692	682.628632	0.70348978
684.136603	6.175783412	682.912068	0.703506451
684.703477	6.176321192	684.136603	0.703581698
685.32988	6.17691038	684.703477	0.703618301
685.928012	6.177467997	685.32988	0.70366005
687.628632	6.179026437	685.928012	0.703701178
687.912068	6.17928224	687.628632	0.70382487
689.136603	6.18037428	687.912068	0.703846453
689.703477	6.180872489	689.136603	0.703942865
690.32988	6.18141752	689.703477	0.703989237
690.928012	6.181932476	690.32988	0.704041756
692.628632	6.183366392	690.928012	0.704093158

Final State: j’=1		Final State: j’=0	
E(cm⁻¹)	σ (Å²)	E(cm⁻¹)	σ (Å²)
692.912068	6.183600877	692.628632	0.704246006
694.136603	6.184598362	692.912068	0.704272443
694.703477	6.185051198	694.136603	0.704389863
695.32988	6.185545539	694.703477	0.704445982
695.928012	6.186008639	695.32988	0.704509521
697.628632	6.187285479	695.928012	0.704571072
697.912068	6.187491461	697.628632	0.704753788
699.136603	6.188354704	697.912068	0.704785297
699.703477	6.188737295	699.136603	0.704925117
700.32988	6.189144649	699.703477	0.704991999
700.928012	6.189515289	700.32988	0.705067653
702.628632	6.190416048	700.928012	0.705141801
702.912068	6.190529985	702.628632	0.70536652
704.136603	6.19071616	702.912068	0.705407015
704.703477	6.19032383	704.136603	0.705609213
705.32988	6.190921932	704.703477	0.705764472
705.928012	6.194136345	705.32988	0.706123302
707.628632	6.196944081	705.928012	0.706254274
707.912068	6.196958077	707.628632	0.706299267
709.136603	6.193950255	707.912068	0.706102591
709.703477	6.196477855	709.136603	0.706555839
710.32988	6.198486607	709.703477	0.706761694
710.928012	6.198628547	710.32988	0.706509962
712.628632	6.196897577	710.928012	0.706591453
712.912068	6.197688873	712.628632	0.706939678
714.136603	6.198849258	712.912068	0.707210291
714.703477	6.203549164	714.136603	0.707177224
715.32988	6.201484225	714.703477	0.707103397
715.928012	6.200635121	715.32988	0.707078488
717.628632	6.203236788	715.928012	0.707223384
717.912068	6.203467119	717.628632	0.707741727
719.136603	6.204967671	717.912068	0.707883088
719.703477	6.204210552	719.136603	0.707649344
720.32988	6.203718088	719.703477	0.707772667
720.928012	6.203034785	720.32988	0.707912081
722.628632	6.204407105	720.928012	0.708072334
722.912068	6.210106747	722.628632	0.708672787
724.136603	6.20749818	722.912068	0.708777864
724.703477	6.207060577	724.136603	0.70851115
725.32988	6.206769788	724.703477	0.708636633
725.928012	6.206440718	725.32988	0.708772743
727.628632	6.210106566	725.928012	0.70890848
727.912068	6.209222359	727.628632	0.709548158
729.136603	6.211137555	727.912068	0.709513088
729.703477	6.210075534	729.136603	0.709413135

Final State: j’=1		Final State: j’=0	
E(cm⁻¹)	σ (Å²)	E(cm⁻¹)	σ (Å²)
730.32988	6.209710287	729.703477	0.709556388
730.928012	6.20951516	730.32988	0.709699302
732.628632	6.208560289	730.928012	0.709832006
732.912068	6.208076214	732.628632	0.710234184
734.136603	6.210466069	732.912068	0.710334939
734.703477	6.213640686	734.136603	0.710868154
735.32988	6.21264107	734.703477	0.710860924
735.928012	6.211674665	735.32988	0.710683734
737.628632	6.210234799	735.928012	0.710847739
737.912068	6.209821325	737.628632	0.71129473
739.136603	6.209511697	737.912068	0.711386993
739.703477	6.21164766	739.136603	0.712343324
740.32988	6.213086429	739.703477	0.712058675
740.928012	6.213888276	740.32988	0.712743662
742.628632	6.216453729	740.928012	0.712890851
742.912068	6.218819356	742.628632	0.713415459
744.136603	6.213217943	742.912068	0.71298142
744.703477	6.213364536	744.136603	0.712839651
745.32988	6.221809748	744.703477	0.713832427
745.928012	6.217840118	745.32988	0.713551265
747.628632	6.217082868	745.928012	0.713064403
747.912068	6.22267709	747.628632	0.714793141
749.136603	6.221824198	747.912068	0.715156149
749.703477	6.218664828	749.136603	0.71369849
750.32988	6.21649717	749.703477	0.713896221
750.928012	6.213734316	750.32988	0.714132663
752.628632	6.220315808	750.928012	0.715003259
752.912068	6.219605212	752.628632	0.714545129
754.136603	6.216841573	752.912068	0.714624654
754.703477	6.214009562	754.136603	0.715002805
755.32988	6.224810591	754.703477	0.715371527
755.928012	6.22350878	755.32988	0.71591082
757.628632	6.219356796	755.928012	0.715744076
757.912068	6.219571234	757.628632	0.716457053
759.136603	6.215527182	757.912068	0.716374097
759.703477	6.22884832	759.136603	0.716715962
760.32988	6.224971069	759.703477	0.717806935
760.928012	6.217522968	760.32988	0.716544627
762.628632	6.21870058	760.928012	0.717026705
762.912068	6.21677504	762.628632	0.717184006
764.136603	6.227056178	762.912068	0.71831151
764.703477	6.225563896	764.136603	0.718528226
765.32988	6.22706869	764.703477	0.71793142
765.928012	6.224986568	765.32988	0.718881849
767.628632	6.227793475	765.928012	0.718020783

Final State: j’=1		Final State: j’=0	
E(cm^{-1})	σ (Å^2)	E(cm^{-1})	σ (Å^2)
767.912068	6.223444909	767.628632	0.719361955
769.136603	6.227811854	767.912068	0.718865598
769.703477	6.225390926	769.136603	0.71953855
770.32988	6.224464591	769.703477	0.719487003
770.928012	6.223993635	770.32988	0.719632651
772.628632	6.224499263	770.928012	0.719802158
772.912068	6.224073489	772.628632	0.720479294
774.136603	6.22641442	772.912068	0.720659419
774.703477	6.22581465	774.136603	0.72078911
775.32988	6.225676296	774.703477	0.720931896
775.928012	6.225666017	775.32988	0.721107853
777.628632	6.225992077	775.928012	0.721278598
777.912068	6.226158248	777.628632	0.721776685
779.136603	6.22658315	777.912068	0.721862404
779.703477	6.226637623	779.136603	0.722217804
780.32988	6.226688478	779.703477	0.722378069
780.928012	6.227661587	780.32988	0.722550588
782.628632	6.227366995	780.928012	0.722730784
782.912068	6.227399901	782.628632	0.723257723
784.136603	6.227613881	782.912068	0.72334084
784.703477	6.227717854	784.136603	0.723703559
785.32988	6.227830866	784.703477	0.72387248
785.928012	6.22793737	785.32988	0.724059602
787.628632	6.228241768	785.928012	0.724238686
787.912068	6.228296356	787.628632	0.724749427
789.136603	6.228642926	787.912068	0.72483464
789.703477	6.228782465	789.136603	0.725210365
790.32988	6.228845415	789.703477	0.725391183
790.928012	6.228909949	790.32988	0.725583304
792.628632	6.229146674	790.928012	0.725765291
792.912068	6.229188703	792.628632	0.726286416
794.136603	6.229371534	792.912068	0.726373717
794.703477	6.229456361	794.136603	0.726752024
795.32988	6.229550338	794.703477	0.726927729
795.928012	6.229640644	795.32988	0.727122318
797.628632	6.229898889	795.928012	0.727308569
797.912068	6.229940811	797.628632	0.727841098
799.136603	6.23011382	797.912068	0.727930275
799.703477	6.230190076	799.136603	0.728316331
800.32988	6.230272493	799.703477	0.728495365
800.928012	6.230349816	800.32988	0.728693424
802.628632	6.230564213	800.928012	0.728882804
802.912068	6.23059925	802.628632	0.72942276
804.136603	6.230748612	802.912068	0.72951298
804.703477	6.230816633	804.136603	0.729903547

Final State: j'=1		Final State: j'=0	
E(cm⁻¹)	σ (Å²)	E(cm⁻¹)	σ (Å²)
805.32988	6.23089095	804.703477	0.73008478
805.928012	6.230961054	805.32988	0.730285367
807.628632	6.231155501	805.928012	0.730477211
807.912068	6.231187155	807.628632	0.731024294
809.136603	6.231321398	807.912068	0.731115698
809.703477	6.231382142	809.136603	0.731511294
810.32988	6.23144829	809.703477	0.7316948
810.928012	6.231510504	810.32988	0.731897836
812.628632	6.231682555	810.928012	0.732091963
812.912068	6.231710558	812.628632	0.732645213
814.136603	6.231829474	812.912068	0.732737607
814.703477	6.231883408	814.136603	0.733137366
815.32988	6.231942195	814.703477	0.733322746
815.928012	6.231997557	815.32988	0.733527824
817.628632	6.232150954	815.928012	0.733723869
817.912068	6.232175955	817.628632	0.73428243
819.136603	6.232282137	817.912068	0.734375687
819.703477	6.232330323	819.136603	0.734779097
820.32988	6.23238286	819.703477	0.734966124
820.928012	6.23243234	820.32988	0.735172986
822.628632	6.232569495	820.928012	0.735370706
822.912068	6.232591871	822.628632	0.73593384
824.136603	6.232686935	822.912068	0.736027829
824.703477	6.232730108	824.136603	0.736434334
825.32988	6.232777215	824.703477	0.736622743
825.928012	6.232821623	825.32988	0.736831099
827.628632	6.232944907	825.928012	0.737030211
827.912068	6.232965045	827.628632	0.737597131
829.136603	6.233050746	827.912068	0.737691729
829.703477	6.233089712	829.136603	0.738100769
830.32988	6.233132279	829.703477	0.738290314
830.928012	6.233172454	830.32988	0.738499894
832.628632	6.233284242	830.928012	0.738700138
832.912068	6.233302539	832.628632	0.739270114
834.136603	6.233380517	832.912068	0.7393652
834.703477	6.233416054	834.136603	0.739776256
835.32988	6.233454923	834.703477	0.73996669
835.928012	6.233491655	835.32988	0.740177222
837.628632	6.233594178	835.928012	0.740378352
837.912068	6.233611006	837.628632	0.740950669
839.136603	6.233682852	837.912068	0.741046123
839.703477	6.233715672	839.136603	0.741458696
840.32988	6.233751632	839.703477	0.741649791
840.928012	6.233785702	840.32988	0.741861021
842.628632	6.233881084	840.928012	0.742062784

Final State: j'=1		Final State: j'=0	
E(cm⁻¹)	σ (Å²)	E(cm⁻¹)	σ (Å²)
842.912068	6.233896784	842.628632	0.742636758
844.136603	6.233964021	842.912068	0.742732463
844.703477	6.233994849	844.136603	0.743146058
845.32988	6.234028681	844.703477	0.743337586
845.928012	6.23406079	845.32988	0.74354927
847.628632	6.234151109	845.928012	0.743751432
847.912068	6.234166032	847.628632	0.744326403
849.136603	6.234230109	847.912068	0.744422249
849.703477	6.234259583	849.136603	0.744836399
850.32988	6.234292025	849.703477	0.74502814
850.928012	6.234322882	850.32988	0.745240037
852.628632	6.23441006	850.928012	0.745442378
852.912068	6.234424521	852.628632	0.746017703
854.136603	6.234486838	852.912068	0.746113591
854.703477	6.234515591	854.136603	0.746527837
855.32988	6.234547337	854.703477	0.746719593
855.928012	6.234577595	855.32988	0.746931474
857.628632	6.23466351	855.928012	0.747133776
857.912068	6.234677823	857.628632	0.747708852
859.136603	6.23473967	857.912068	0.747804678
859.703477	6.234768319	859.136603	0.748218597
860.32988	6.234800013	859.703477	0.748410167
860.928012	6.234830318	860.32988	0.748621816
862.628632	6.234916718	860.928012	0.748823872
862.912068	6.234931172	862.628632	0.749398114
864.136603	6.234993793	862.912068	0.749493784
864.703477	6.235022896	864.136603	0.749906962
865.32988	6.235055166	864.703477	0.750098156
865.928012	6.235086098	865.32988	0.750309366
867.628632	6.235174653	865.928012	0.750510975
867.912068	6.235189508	867.628632	0.751083831
869.136603	6.235254074	867.912068	0.75117925
869.703477	6.235284184	869.136603	0.751591294
870.32988	6.235317616	869.703477	0.751781928
870.928012	6.235349714	870.32988	0.751992497
872.628632	6.235441964	870.928012	0.752193476
872.912068	6.235457498	872.628632	0.752764413
874.136603	6.235525106	872.912068	0.752859493
874.703477	6.235556703	874.136603	0.753270031
875.32988	6.235591857	874.703477	0.753459936
875.928012	6.235625673	875.32988	0.753669673
877.628632	6.235723097	875.928012	0.75386984
877.912068	6.235739528	877.628632	0.75443835
879.136603	6.235811222	877.912068	0.754533012
879.703477	6.235844782	879.136603	0.754941675

Final State: j’=1		Final State: j’=0	
E(cm⁻¹)	σ (Å²)	E(cm⁻¹)	σ (Å²)
880.32988	6.235882178	879.703477	0.755130686
880.928012	6.23591817	880.32988	0.755339417
882.628632	6.236022152	880.928012	0.755538596
882.912068	6.236039727	882.628632	0.756104198
884.136603	6.23611647	882.912068	0.75619836
884.703477	6.236152598	884.136603	0.756604809
885.32988	6.236192576	884.703477	0.756792797
885.928012	6.236231229	885.32988	0.75700031
887.628632	6.236343064	885.928012	0.757198341
887.912068	6.236361984	887.628632	0.757760565
889.136603	6.236444702	887.912068	0.757854145
889.703477	6.23648353	889.136603	0.758258049
890.32988	6.236526841	889.703477	0.758444801
890.928012	6.236568593	890.32988	0.758650994
892.628632	6.236689536	890.928012	0.75884771
892.912068	6.236710018	892.628632	0.759406104
894.136603	6.236799584	892.912068	0.759499033
894.703477	6.236841655	894.136603	0.759900062
895.32988	6.236888608	894.703477	0.760085458
895.928012	6.236933889	895.32988	0.760290133
897.628632	6.237065126	895.928012	0.760485379
897.912068	6.237087348	897.628632	0.761039478
899.136603	6.237184642	897.912068	0.76113168
899.703477	6.237230365	899.136603	0.761529505
900.32988	6.237281401	899.703477	0.761713386
900.928012	6.237330644	900.32988	0.761916363
902.628632	6.237473403	900.928012	0.762109969
902.912068	6.237497602	902.628632	0.762659295
904.136603	6.237603551	902.912068	0.76275068
904.703477	6.237653349	904.136603	0.763144927
905.32988	6.23770896	904.703477	0.763327119
905.928012	6.237762633	905.32988	0.763528204
907.628632	6.237918403	905.928012	0.763719979
907.912068	6.237944829	907.628632	0.764263925
909.136603	6.238060566	907.912068	0.76435439
909.703477	6.23811504	909.136603	0.764744574
910.32988	6.238175896	909.703477	0.76492483
910.928012	6.238234697	910.32988	0.765123736
912.628632	6.238405673	910.928012	0.765313382
912.912068	6.238434746	912.628632	0.765851002
914.136603	6.238562343	912.912068	0.765940362
914.703477	6.238622594	914.136603	0.766325588
915.32988	6.238690084	914.703477	0.766503426
915.928012	6.2387555	915.32988	0.766699546
917.628632	6.238947388	915.928012	0.766886403

Final State: j’=1		Final State: j’=0	
E(cm⁻¹)	σ (Å²)	E(cm⁻¹)	σ (Å²)
917.912068	6.238980355	917.628632	0.767415158
919.136603	6.239126744	917.912068	0.76750286
919.703477	6.239197225	919.136603	0.767880006
920.32988	6.239277705	919.703477	0.768053401
920.928012	6.239357976	920.32988	0.768243823
922.628632	6.239620862	920.928012	0.768424089
922.912068	6.239675559	922.628632	0.768919094
924.136603	6.239714895	922.912068	0.768995391
924.703477	6.239287803	924.136603	0.769220944
925.32988	6.239582471	924.703477	0.769438007
925.928012	6.239855331	925.32988	0.769818431
927.628632	6.240094835	925.928012	0.769828591
927.912068	6.24022481	927.628632	0.770481612
929.136603	6.23972991	927.912068	0.770465334
929.703477	6.239989197	929.136603	0.771047066
930.32988	6.239932837	929.703477	0.771113322
930.928012	6.240312531	930.32988	0.771380631
932.628632	6.240311578	930.928012	0.771551554
932.912068	6.240566336	932.628632	0.772122158
934.136603	6.240625946	932.912068	0.772098305
934.703477	6.24080577	934.136603	0.772592673
935.32988	6.240943114	934.703477	0.772714047
935.928012	6.240096476	935.32988	0.772814924
937.628632	6.240612677	935.928012	0.77297487
937.912068	6.241027801	937.628632	0.773705904
939.136603	6.241394115	937.912068	0.773788829
939.703477	6.241510198	939.136603	0.774069385
940.32988	6.241286192	939.703477	0.774198924
940.928012	6.240998207	940.32988	0.774243973
942.628632	6.241719568	940.928012	0.774584778
942.912068	6.241837171	942.628632	0.775213149
944.136603	6.242122194	942.912068	0.775267705
944.703477	6.242227304	944.136603	0.775572949
945.32988	6.242315983	944.703477	0.775717821
945.928012	6.241785309	945.32988	0.775863487
947.628632	6.242270467	945.928012	0.775948816
947.912068	6.242513456	947.628632	0.776656256
949.136603	6.242925078	947.912068	0.77679728
949.703477	6.24303774	949.136603	0.777058125
950.32988	6.243154199	949.703477	0.7772063
950.928012	6.243255828	950.32988	0.777371146
952.628632	6.243192803	950.928012	0.777525648
952.912068	6.243499985	952.628632	0.778001951
954.136603	6.243790945	952.912068	0.777997674
954.703477	6.243981767	954.136603	0.778535727

Final State: j’=1		Final State: j’=0	
E(cm⁻¹)	σ (Å²)	E(cm⁻¹)	σ (Å²)
955.32988	6.244140696	954.703477	0.778646028
955.928012	6.244294182	955.32988	0.778794324
957.628632	6.244668659	955.928012	0.778936844
957.912068	6.24449326	957.628632	0.779163991
959.136603	6.243951917	957.912068	0.779165651
959.703477	6.2439296	959.136603	0.779607325
960.32988	6.244495065	959.703477	0.779832451
960.928012	6.24506793	960.32988	0.780246871
962.628632	6.245135751	960.928012	0.780187413
962.912068	6.245340946	962.628632	0.780906529
964.136603	6.244158912	962.912068	0.78091283
964.703477	6.24511037	964.136603	0.781286197
965.32988	6.245504381	964.703477	0.781602977
965.928012	6.245718452	965.32988	0.781677891
967.628632	6.245516772	965.928012	0.781708084
967.912068	6.245582764	967.628632	0.782499554
969.136603	6.246459011	967.912068	0.78254438
969.703477	6.246535476	969.136603	0.782716164
970.32988	6.244672101	969.703477	0.782598174
970.928012	6.246235973	970.32988	0.783043744
972.628632	6.247052848	970.928012	0.783436354
972.912068	6.247137738	972.628632	0.783706738
974.136603	6.2450704	972.912068	0.783753994
974.703477	6.247099677	974.136603	0.784021023
975.32988	6.247503583	974.703477	0.784335022
975.928012	6.247975533	975.32988	0.784379225
977.628632	6.247556998	975.928012	0.784567733
977.912068	6.247946985	977.628632	0.784852865
979.136603	6.24782908	977.912068	0.784991417
979.703477	6.248723729	979.136603	0.785674817
980.32988	6.247508375	979.703477	0.785553007
980.928012	6.248401527	980.32988	0.785552898
982.628632	6.248286961	980.928012	0.786066736
982.912068	6.24812628	982.628632	0.786354395
984.136603	6.248345545	982.912068	0.78612809
984.703477	6.248708477	984.136603	0.786714719
985.32988	6.249635951	984.703477	0.787155671
985.928012	6.249804532	985.32988	0.787157261
987.628632	6.248809002	985.928012	0.787109688
987.912068	6.248871494	987.628632	0.78754586
989.136603	6.24997465	987.912068	0.787693964
989.703477	6.250075522	989.136603	0.788080632
990.32988	6.250134189	989.703477	0.788213186
990.928012	6.250109506	990.32988	0.788364374
992.628632	6.250502441	990.928012	0.78851483

Final State: j'=1		Final State: j'=0	
E(cm⁻¹)	σ (Å²)	E(cm⁻¹)	σ (Å²)
992.912068	6.250701915	992.628632	0.788993404
994.136603	6.25095761	992.912068	0.78906771
994.703477	6.251057152	994.136603	0.789369004
995.32988	6.251165635	994.703477	0.78951278
995.928012	6.251260386	995.32988	0.78967218
997.628632	6.25157414	995.928012	0.789824478
997.912068	6.251640488	997.628632	0.790266214
999.136603	6.251879918	997.912068	0.790339505
999.703477	6.251835123	999.136603	0.790653529
1000.32988	6.252186463	999.703477	0.79079925
1000.92801	6.252377457	1000.32988	0.79095405
1002.62863	6.25270124	1000.92801	0.791117412
1002.91207	6.252760982	1002.62863	0.791544166
1004.1366	6.253023931	1002.91207	0.791616026
1004.70348	6.25314674	1004.1366	0.79192607
1005.32988	6.253282552	1004.70348	0.792069298
1005.92801	6.253411617	1005.32988	0.792227328
1007.62863	6.253771616	1005.92801	0.792378038
1007.91207	6.253835052	1007.62863	0.79280473
1009.1366	6.254138616	1007.91207	0.792875388
1009.70348	6.254269236	1009.1366	0.793182307
1010.32988	6.254411128	1009.70348	0.793324068
1010.92801	6.254546363	1010.32988	0.793480284
1012.62863	6.254932099	1010.92801	0.793629172
1012.91207	6.254996605	1012.62863	0.794051257
1014.1366	6.255275942	1012.91207	0.794121432
1014.70348	6.255405652	1014.1366	0.794424043
1015.32988	6.255549426	1014.70348	0.794563815
1015.92801	6.255687375	1015.32988	0.794718033
1017.62863	6.256083404	1015.92801	0.794865065
1017.91207	6.256149725	1017.62863	0.795282029
1019.1366	6.256436633	1017.91207	0.795351379
1019.70348	6.256569601	1019.1366	0.795650482
1020.32988	6.256716677	1019.70348	0.795788661
1020.92801	6.256857266	1020.32988	0.795941137
1022.62863	6.257258005	1020.92801	0.796086527
1022.91207	6.257324955	1022.62863	0.796498803
1024.1366	6.257614749	1022.91207	0.796567358
1024.70348	6.257749227	1024.1366	0.796863032
1025.32988	6.257898065	1024.70348	0.796999631
1025.92801	6.258040424	1025.32988	0.797150372
1027.62863	6.258446402	1025.92801	0.797294118
1029.1366	6.258807726	1027.62863	0.797701787
1029.70348	6.258943844	1029.1366	0.798062031
1030.32988	6.259094442	1029.70348	0.798197153

Final State: j’=1		Final State: j’=0	
E(cm⁻¹)	σ (Å²)	E(cm⁻¹)	σ (Å²)
1030.92801	6.259238411	1030.32988	0.798346277
1032.62863	6.259648594	1030.92801	0.798488482
1034.1366	6.260013324	1032.62863	0.798891844
1034.70348	6.260150663	1034.1366	0.799248324
1035.32988	6.260302571	1034.70348	0.799382047
1035.92801	6.260447759	1035.32988	0.799529634
1037.62863	6.260861253	1035.92801	0.799670385
1039.1366	6.261228699	1037.62863	0.800069657
1039.70348	6.261367004	1039.1366	0.80042259
1040.32988	6.261519942	1039.70348	0.800554996
1040.92801	6.261666082	1040.32988	0.800701142
1042.62863	6.262082054	1040.92801	0.800840526
1044.1366	6.262451444	1042.62863	0.801235981
1044.70348	6.262590423	1044.1366	0.801585595
1045.32988	6.262744053	1044.70348	0.801716775
1045.92801	6.262890822	1045.32988	0.801861575
1047.62863	6.263308374	1045.92801	0.801999685
1049.1366	6.263678916	1047.62863	0.802391573
1049.70348	6.263818257	1049.1366	0.802738106
1050.32988	6.263972277	1049.70348	0.802868144
1050.92801	6.264119349	1050.32988	0.803011693
1052.62863	6.264537614	1050.92801	0.803148625
1054.1366	6.264908533	1052.62863	0.803537218
1054.70348	6.265047955	1054.1366	0.803880904
1055.32988	6.265202019	1054.70348	0.804009892
1055.92801	6.265349109	1055.32988	0.804152294
1057.62863	6.265767194	1055.92801	0.804288139
1059.1366	6.266137701	1057.62863	0.804673711
1059.70348	6.266276916	1059.1366	0.805014793
1060.32988	6.266430694	1059.70348	0.805142819
1060.92801	6.266577484	1060.32988	0.80528417
1062.62863	6.266994518	1060.92801	0.805419027
1064.1366	6.267363834	1062.62863	0.805801843
1064.70348	6.267502551	1064.1366	0.806140562
1065.32988	6.267655733	1064.70348	0.80626772
1065.92801	6.267801907	1065.32988	0.806408121
1067.62863	6.268217016	1065.92801	0.806542083
1069.1366	6.268584377	1067.62863	0.806922424
1069.70348	6.268722305	1069.1366	0.807259023
1070.32988	6.268874574	1069.70348	0.807385403
1070.92801	6.26901984	1070.32988	0.807524958
1072.62863	6.269432173	1070.92801	0.807658123
1074.1366	6.269796812	1072.62863	0.808036257
1074.70348	6.269933655	1074.1366	0.808370978
1075.32988	6.27008469	1074.70348	0.80849667

Final State: j'=1		Final State: j'=0	
E(cm^{-1})	σ (Å^2)	E(cm^{-1})	σ (Å^2)
1075.92801	6.270228758	1075.32988	0.808635477
1077.62863	6.270637443	1075.92801	0.80876794
1079.1366	6.27099864	1077.62863	0.80914414
1079.70348	6.271134122	1079.1366	0.809477222
1080.32988	6.271283636	1079.70348	0.809602316
1080.92801	6.27142615	1080.32988	0.809740476
1084.1366	6.272187432	1080.92801	0.809872332
1084.70348	6.272321274	1084.1366	0.810578547
1085.32988	6.272468939	1084.70348	0.810703132
1085.92801	6.272609716	1085.32988	0.810840743
1089.1366	6.273360787	1085.92801	0.810972083
1089.70348	6.273492309	1089.1366	0.811675733
1090.32988	6.273638274	1089.70348	0.811800766
1090.92801	6.27377698	1090.32988	0.811937053
1094.1366	6.274516405	1090.92801	0.812067968
1094.70348	6.274646216	1094.1366	0.812769533
1095.32988	6.274789337	1094.70348	0.812893365
1095.92801	6.274925698	1095.32988	0.813030158
1099.1366	6.275652063	1095.92801	0.813160744
1099.70348	6.275779448	1099.1366	0.813860705
1100.92801	6.276053658	1099.70348	0.813984281
1104.1366	6.276765558	1100.92801	0.814251147
1104.70348	6.276890311	1104.1366	0.814949958
1105.92801	6.277158722	1104.70348	0.815073365
1109.1366	6.277854852	1105.92801	0.815339891
1109.70348	6.277976724	1109.1366	0.816038002
1110.92801	6.278238849	1109.70348	0.816161313
1114.1366	6.278917934	1110.92801	0.816427662
1114.70348	6.27903672	1114.1366	0.817125501
1115.92801	6.279292088	1114.70348	0.817248792
1119.1366	6.279952946	1115.92801	0.817515124
1119.70348	6.280068446	1119.1366	0.818213096
1120.92801	6.280316598	1119.70348	0.818336434
1124.1366	6.280958117	1120.92801	0.8186029
1124.70348	6.281070111	1124.1366	0.819301393
1125.92801	6.281310641	1124.70348	0.819424847
1129.1366	6.281931728	1125.92801	0.819691592
1129.70348	6.282040041	1129.1366	0.820390969
1130.92801	6.282272575	1129.70348	0.820514605
1134.1366	6.282872229	1130.92801	0.820781759
1134.70348	6.282976708	1134.1366	0.821482368
1135.92801	6.283200854	1134.70348	0.821606243
1139.1366	6.28377816	1135.92801	0.821873963
1139.70348	6.283878633	1139.1366	0.822576105
1140.92801	6.284094067	1139.70348	0.822700276

Final State: j’=1		Final State: j’=0	
E(cm⁻¹)	σ (Å²)	E(cm⁻¹)	σ (Å²)
1144.1366	6.284648169	1140.92801	0.822968637
1144.70348	6.284744476	1144.1366	0.82367268
1145.92801	6.284950887	1144.70348	0.823797206
1149.1366	6.285480954	1145.92801	0.824066349
1149.70348	6.285572986	1149.1366	0.824772594
1150.92801	6.285770053	1149.70348	0.824897529
1154.1366	6.286275311	1150.92801	0.825167584
1154.70348	6.286362876	1154.1366	0.825876396
1155.92801	6.286550312	1154.70348	0.826001815
1159.1366	6.287029853	1155.92801	0.826272952
1159.70348	6.287112803	1159.1366	0.826984879
1160.92801	6.287290137	1159.70348	0.827110906
1164.1366	6.287742332	1160.92801	0.827383433
1165.92801	6.287986192	1164.1366	0.828099869
1169.1366	6.288398539	1165.92801	0.828502073
1170.92801	6.288719256	1169.1366	0.829236358
1174.1366	6.289043969	1170.92801	0.829676251
1175.92801	6.289322716	1174.1366	0.830382683
1179.1366	6.289683084	1175.92801	0.830752743
1180.92801	6.289888822	1179.1366	0.831476065
1184.1366	6.290252995	1180.92801	0.831843784
1185.92801	6.290431746	1184.1366	0.832533849
1189.1366	6.290743529	1185.92801	0.832976472
1190.92801	6.29091893	1189.1366	0.833671405
1194.1366	6.291204998	1190.92801	0.834100552
1195.92801	6.291351285	1194.1366	0.834789808
1199.1366	6.291677689	1195.92801	0.835233699
1200.92801	6.291725353	1199.1366	0.83597133
1204.1366	6.292012456	1200.92801	0.836380679
1205.92801	6.292243116	1204.1366	0.837252612
1209.1366	6.29234402	1205.92801	0.837574972
1210.92801	6.292537839	1209.1366	0.838280002
1214.1366	6.292713235	1210.92801	0.838628186
1215.92801	6.292814775	1214.1366	0.839338874
1219.1366	6.292931642	1215.92801	0.839902712
1220.92801	6.29285946	1219.1366	0.84056005
1224.1366	6.293230497	1220.92801	0.840943239
1225.92801	6.293148154	1224.1366	0.841742319
1229.1366	6.293442327	1225.92801	0.842154616
1230.92801	6.29341907	1229.1366	0.8429414
1234.1366	6.293584613	1230.92801	0.843215571
1235.92801	6.293573485	1234.1366	0.844063255
1239.1366	6.2936076	1235.92801	0.844461303
1240.92801	6.293675041	1239.1366	0.845202101
1244.1366	6.293728484	1240.92801	0.845620052

Final State: j'=1		Final State: j'=0	
E(cm^{-1})	σ (Å^2)	E(cm^{-1})	σ (Å^2)
1245.92801	6.29375514	1244.1366	0.84636146
1249.1366	6.293759118	1245.92801	0.846770913
1250.92801	6.293764901	1249.1366	0.8475218
1254.1366	6.293759907	1250.92801	0.847936815
1255.92801	6.293748989	1254.1366	0.848679628
1259.1366	6.293724807	1255.92801	0.849094971
1260.92801	6.293704317	1259.1366	0.849837206
1264.1366	6.293655588	1260.92801	0.850251224
1265.92801	6.293622861	1264.1366	0.850992088
1269.1366	6.293553923	1265.92801	0.851405229
1270.92801	6.293509596	1269.1366	0.852144135
1275.92801	6.293364252	1270.92801	0.85255604
1280.92801	6.293188113	1275.92801	0.853702964
1285.92801	6.292982018	1280.92801	0.854845231
1290.92801	6.292746869	1285.92801	0.855982073
1295.92801	6.292483523	1290.92801	0.857112699
1300.92801	6.29219285	1295.92801	0.858236353
1305.92801	6.291875616	1300.92801	0.859352243
1310.92801	6.291532568	1305.92801	0.860459591
1315.92801	6.2911644	1310.92801	0.861557637
1320.92801	6.290771738	1315.92801	0.862645596
1325.92801	6.290355147	1320.92801	0.86372273
1330.92801	6.289915127	1325.92801	0.864788301
1335.92801	6.289452074	1330.92801	0.865841586
1340.92801	6.288966351	1335.92801	0.866881884
1345.92801	6.288458204	1340.92801	0.86790852
1350.92801	6.287927802	1345.92801	0.868920842
1355.92801	6.287375232	1350.92801	0.869918219
1360.92801	6.286800509	1355.92801	0.870900058
1365.92801	6.286203503	1360.92801	0.871865793
1370.92801	6.285584063	1365.92801	0.872814902
1375.92801	6.284941865	1370.92801	0.873746886
1380.92801	6.284276567	1375.92801	0.874661301
1385.92801	6.28358766	1380.92801	0.875557704
1390.92801	6.282874608	1385.92801	0.87643574
1395.92801	6.282136672	1390.92801	0.87729507
1400.92801	6.281373293	1395.92801	0.878135393
1405.92801	6.280583464	1400.92801	0.878956455
		1405.92801	0.879758048

Initial rotational state: j=2					
Final State: j'=5		Final State: j'=4		Final State: j'=3	
E(cm⁻¹)	σ (Å²)	E(cm⁻¹)	σ (Å²)	E(cm⁻¹)	σ (Å²)
411.0849853	0.033720655	274.2935763	0.243811958	164.8604503	1.765642501
411.1849853	0.066342085	274.3935763	0.458794226	164.9604503	3.456974289
411.2849853	0.047663906	274.4935763	0.340900075	165.0604503	2.550224967
411.4849853	0.054380046	274.5935763	0.371336841	165.1604503	2.897163761
411.5849853	0.065001426	274.6935763	0.397036736	165.2604503	3.224797488
411.6849853	0.073267106	274.7604503	0.419713405	165.3604503	4.15482607
411.7849853	0.073259037	274.7935763	0.45239006	165.3868533	4.660120964
411.8849853	0.069412242	274.8935763	0.546311961	165.4604503	4.067603621
411.9849853	0.081203022	274.9935763	0.500731452	165.5604503	3.655728894
412.0849853	0.074288456	275.0935763	0.496445774	165.6604503	3.868951465
412.2849853	0.073134931	275.1935763	0.568987194	165.7604503	4.407229774
412.3849853	0.074009843	275.2935763	0.526444413	165.8604503	3.801623282
412.4849853	0.074999394	275.3868533	0.522715777	165.9604503	3.822620211
412.6856053	0.07692438	275.3935763	0.522886508	167.6856053	6.879676633
412.9690413	0.079579674	275.4935763	0.527812271	167.9690413	6.738251186
414.1935763	0.121196158	275.5935763	0.534696723	169.7604503	7.003847813
415.3868533	0.116346047	277.6856053	0.836820796	169.8604503	7.073988792
415.9849853	0.123795367	277.9690413	0.820485298	169.9604503	7.136012102
416.0849853	0.124908537	279.1935763	0.893070949	170.0604503	7.190131188
416.1849853	0.125907352	279.2935763	0.900561604	170.1604503	7.237581468
416.2849853	0.126782821	279.3935763	0.907127138	170.2604503	7.281159792
416.3849853	0.127532653	279.4935763	0.912686903	170.3604503	7.327626094
416.4849853	0.12815586	279.5935763	0.917191099	170.3868533	7.342336373
416.5849853	0.128641122	279.6935763	0.920567338	170.4604503	7.398057375
416.6849853	0.128957857	279.7604503	0.922110455	172.6856053	8.483630062
416.7849853	0.129692815	279.7935763	0.922628229	172.9690413	8.394715773
416.8849853	0.131099687	279.8935763	0.92317282	174.7604503	8.641003441
416.9849853	0.132404685	279.9935763	0.92600154	175.3868533	9.13410547
417.0849853	0.13482537	280.0935763	0.943659824	177.6856053	9.901386834
417.6856053	0.17892903	280.3868533	0.988706815	177.9690413	9.840987532
417.9690413	0.193839972	282.6856053	1.070823916	179.7604503	12.70040337
419.1935763	0.149873911	282.9690413	1.073564926	180.3868533	10.07301024
419.7604503	0.150405965	284.1935763	1.106056705	182.6856053	10.1833202
420.3868533	0.152388658	284.7604503	1.145871003	182.9690413	10.19458001
420.9849853	0.155576884	285.3868533	1.224272634	184.7604503	10.27106876
422.6856053	0.179182046	287.6856053	1.243699991	185.3868533	10.32604519
424.1935763	0.176915918	287.9690413	1.247566619	187.6856053	11.73279335
424.7604503	0.17781712	289.1935763	1.471721093	187.9690413	11.60284373
425.3868533	0.208241436	289.7604503	1.287376231	189.7604503	10.83000374
425.9849853	0.215248662	290.3868533	1.291995628	190.3868533	10.7662516
427.6856053	0.187751688	292.6856053	1.333575121	192.6856053	10.72006702
427.9690413	0.188771072	292.9690413	1.337683562	192.9690413	10.72628764

Final State: j'=5		Final State: j'=4		Final State: j'=3	
E(cm⁻¹)	σ (Å²)	E(cm⁻¹)	σ (Å²)	E(cm⁻¹)	σ (Å²)
429.1935763	0.192538592	294.1935763	1.356154456	194.7604503	10.83804225
429.7604503	0.194105931	294.7604503	1.36594626	195.3868533	10.8990617
430.3868533	0.195852031	295.3868533	1.379594818	197.6856053	10.98734687
432.6856053	0.204906477	297.6856053	1.465841187	197.9690413	10.98431215
432.9690413	0.207597641	297.9690413	1.458936279	199.7604503	10.95910672
434.1935763	0.218170164	299.1935763	1.453209891	200.3868533	10.95348235
434.7604503	0.216020118	299.7604503	1.45710424	202.6856053	10.95560716
435.3868533	0.215245907	300.3868533	1.463117868	202.9690413	10.95807139
435.9849853	0.215692383	302.6856053	1.492154545	204.7604503	10.97855006
437.6856053	0.21909696	302.9690413	1.496375255	205.3868533	10.98534867
437.9690413	0.219793273	304.1935763	1.516698952	207.6856053	10.99381331
439.1935763	0.223053531	304.7604503	1.526919517	207.9690413	10.9923669
439.7604503	0.224716099	305.3868533	1.537894769	209.7604503	10.96393188
440.9849853	0.228737904	307.6856053	1.569941067	210.3868533	10.94133397
442.6856053	0.234580919	307.9690413	1.573420143	212.6856053	10.64968233
442.9690413	0.235445338	309.1935763	1.588469562	212.9690413	10.68390832
444.1935763	0.238850177	309.7604503	1.595554884	214.7604503	10.83784238
444.7604503	0.24034272	310.3868533	1.603509602	215.3868533	11.1247936
445.3868533	0.241986636	312.6856053	1.633884261	217.6856053	11.049449
445.9849853	0.243569307	312.9690413	1.637742008	217.9690413	11.12738667
447.6856053	0.248197162	314.1935763	1.654561537	219.7604503	11.09080244
447.9690413	0.248989609	314.7604503	1.662378136	220.3868533	10.94255431
449.1935763	0.252483222	315.3868533	1.670996981	222.6856053	11.45511815
449.7604503	0.25413554	317.6856053	1.702217974	222.9690413	11.38333449
450.3868533	0.255981554	317.9690413	1.706020879	224.7604503	11.221475
450.9849853	0.257758324	319.1935763	1.722366193	225.3868533	11.45998683
452.6856053	0.262836807	319.7604503	1.729898877	227.6856053	11.44187565
452.9690413	0.263682667	320.3868533	1.738206776	227.9690413	11.38157921
454.1935763	0.267328649	322.6856053	1.768623404	229.7604503	11.33870122
454.7604503	0.269012677	322.9690413	1.772369136	230.3868533	11.45086146
455.3868533	0.270872406	324.1935763	1.788537525	232.6856053	11.5895041
457.6856053	0.277712704	324.7604503	1.796011167	232.9690413	11.54936313
457.9690413	0.27855998	325.3868533	1.804257922	234.7604503	11.15604366
459.1935763	0.282234396	327.6856053	1.834366961	235.3868533	11.62091728
459.7604503	0.283943555	327.9690413	1.838058031	237.6856053	11.77324824
460.3868533	0.285838322	329.1935763	1.853938952	237.9690413	11.74131242
462.6856053	0.292842779	329.7604503	1.861251506	239.7604503	11.50429123
462.9690413	0.293711405	330.3868533	1.869300598	240.3868533	11.26502436
464.1935763	0.297474875	332.6856053	1.898521412	242.6856053	12.05750423
464.7604503	0.299222562	332.9690413	1.90208577	242.9690413	11.95351772
465.3868533	0.301157513	334.1935763	1.917372496	244.7604503	11.65079228
465.9849853	0.303008658	334.7604503	1.92438165	245.3868533	11.52946079
467.6856053	0.308290164	335.3868533	1.932071346	247.6856053	10.90010966

Final State: j'=5		Final State: j'=4		Final State: j'=3	
E(cm⁻¹)	σ (Å²)	E(cm⁻¹)	σ (Å²)	E(cm⁻¹)	σ (Å²)
467.9690413	0.30917303	337.6856053	1.959692178	247.9690413	11.29098045
469.1935763	0.312995947	337.9690413	1.963020201	249.7604503	11.68511698
469.7604503	0.314770483	339.1935763	1.977152986	250.3868533	11.95447776
470.3868533	0.316734933	339.7604503	1.983540755	252.6856053	11.85189009
470.9849853	0.318614216	340.3868533	1.990464671	252.9690413	12.0585909
472.6856053	0.323976012	342.6856053	2.014257549	254.7604503	11.08037237
472.9690413	0.324872288	342.9690413	2.01695748	255.3868533	11.49189753
474.1935763	0.328752948	344.1935763	2.027753643	257.6856053	12.02577941
474.7604503	0.330553973	344.7604503	2.032133681	257.9690413	11.86422323
475.3868533	0.332547386	345.3868533	2.036340078	259.7604503	12.34825024
475.9849853	0.334453928	347.6856053	2.040044724	260.3868533	12.19041685
477.6856053	0.339890464	347.9690413	2.037704136	262.6856053	11.30201055
477.9690413	0.34079873	349.1935763	2.003855687	262.9690413	11.54016328
479.1935763	0.344729484	349.7604503	2.028849497	264.7604503	12.24419743
479.7604503	0.346552718	350.3868533	2.124536446	265.3868533	12.09898627
480.3868533	0.348569922	352.6856053	2.18187706	267.6856053	12.66242063
480.9849853	0.350498438	352.9690413	2.146232149	267.9690413	12.4755642
482.6856053	0.355993376	354.1935763	2.175842171	269.7604503	12.21555201
482.9690413	0.356910771	354.7604503	2.233943584	270.3868533	11.63013773
484.1935763	0.360878897	355.3868533	2.209776285	272.6856053	12.18868762
484.7604503	0.362718249	357.6856053	2.290896626	272.9690413	12.36682683
485.3868533	0.364752356	357.9690413	2.340161169	274.2935763	12.10870263
485.9849853	0.36669608	359.1935763	2.34955021	274.3935763	11.79517074
487.6856053	0.372228866	359.7604503	2.292476403	274.4935763	11.94363124
489.1935763	0.377140439	360.3868533	2.248479757	274.5935763	11.81375616
489.7604503	0.378987508	362.6856053	2.303406874	274.6935763	11.63249352
490.3868533	0.381028695	362.9690413	2.423102697	274.7604503	11.41234745
490.9849853	0.382977675	364.1935763	2.356354364	274.7935763	11.28577511
492.6856053	0.388516397	364.7604503	2.330185435	274.8935763	11.56437249
492.9690413	0.38943883	365.3868533	2.287891131	274.9935763	11.94815352
494.1935763	0.393420335	367.6856053	2.505595915	275.0935763	11.76830863
494.7604503	0.395260882	367.9690413	2.519554305	275.1935763	11.63062684
495.3868533	0.397292246	369.1935763	2.431574794	275.2935763	12.01043703
495.9849853	0.399229065	369.7604503	2.413658326	275.3868533	11.95906454
497.6856053	0.404716111	370.3868533	2.388788512	275.3935763	11.95472423
497.9690413	0.405627126	372.6856053	2.435062377	275.4935763	11.89237944
499.1935763	0.409548385	372.9690413	2.584041381	275.5935763	11.83408131
499.7604503	0.41135427	374.1935763	2.524926528	277.6856053	11.73655748
500.3868533	0.413341575	374.7604503	2.506726042	277.9690413	11.71090381
500.9849853	0.41522999	375.3868533	2.49179333	279.1935763	11.47352861
502.6856053	0.420537968	377.6856053	2.442430657	279.2935763	11.44783281
502.9690413	0.421411939	377.9690413	2.578067294	279.3935763	11.41948587
504.1935763	0.425142696	379.1935763	2.650180394	279.4935763	11.38670098

Final State: j’=5		Final State: j’=4		Final State: j’=3	
E(cm⁻¹)	σ (Å²)	E(cm⁻¹)	σ (Å²)	E(cm⁻¹)	σ (Å²)
504.7604503	0.426840322	379.7604503	2.614633411	279.5935763	11.34645587
505.3868533	0.428689552	380.3868533	2.570868142	279.6935763	11.29333201
505.9849853	0.430424505	382.6856053	2.459721026	279.7604503	11.24585737
507.6856053	0.43513456	382.9690413	2.447459066	279.7935763	11.21715402
507.9690413	0.435877005	384.1935763	2.468834033	279.8935763	11.10380275
509.1935763	0.438875406	384.7604503	2.639223262	279.9935763	11.00725784
509.7604503	0.440106286	385.3868533	2.61248339	280.0935763	11.1375848
510.3868533	0.441292971	387.6856053	2.732935427	280.3868533	11.1677203
510.9849853	0.442180468	387.9690413	2.691118651	282.6856053	11.59464122
512.9690413	0.440106455	389.1935763	2.681890822	282.9690413	11.57900255
514.1935763	0.449869992	389.7604503	2.804339978	284.1935763	11.5092888
514.7604503	0.468323657	390.3868533	2.719150651	284.7604503	11.45408937
515.3868533	0.456478145	392.6856053	2.948622462	285.3868533	11.37543794
515.9849853	0.469302259	392.9690413	2.899455426	287.6856053	11.39707356
517.6856053	0.456206284	394.1935763	2.800681287	287.9690413	11.37438863
517.9690413	0.46325359	394.7604503	2.720676977	289.1935763	11.34785833
519.1935763	0.488997828	395.3868533	2.691777491	289.7604503	11.53057338
519.7604503	0.478123769	397.6856053	2.801882845	290.3868533	11.4935449
520.3868533	0.489259394	397.9690413	2.779371922	292.6856053	11.46810905
520.9849853	0.475808476	399.1935763	2.618473729	292.9690413	11.46803911
522.6856053	0.486694375	399.7604503	2.870225398	294.1935763	11.46672243
524.1935763	0.500282411	400.3868533	3.010607061	294.7604503	11.46404743
524.7604503	0.492412931	402.6856053	2.808724879	295.3868533	11.45664607
525.3868533	0.494692473	402.9690413	2.818157429	297.6856053	11.44206808
525.9849853	0.513275145	404.1935763	3.080761205	297.9690413	11.45728185
527.6856053	0.526803362	404.7604503	3.02458246	299.1935763	11.48639562
527.9690413	0.523083231	405.3868533	2.82403248	299.7604503	11.4924871
529.1935763	0.512938919	407.6856053	2.873912987	300.3868533	11.49768419
529.7604503	0.503644121	407.9690413	2.819494593	302.6856053	11.51051371
530.3868533	0.531613106	409.1935763	3.089600731	302.9690413	11.51147354
530.9849853	0.530727502	409.7604503	3.115176349	304.1935763	11.5140654
532.6856053	0.543614915	410.3868533	3.14926108	304.7604503	11.51517302
532.9690413	0.541411974	411.0849853	2.953335529	305.3868533	11.51744348
534.1935763	0.53535393	411.1849853	2.901790603	307.6856053	11.5354713
534.7604503	0.530966742	411.2849853	2.923645153	307.9690413	11.53796648
535.3868533	0.519797909	411.3849853	2.889113131	309.1935763	11.54822615
535.9849853	0.551802851	411.4849853	2.845272318	309.7604503	11.55265628
537.6856053	0.570350491	411.5849853	2.819236647	310.3868533	11.55732928
537.9690413	0.565023643	411.6849853	2.922150832	312.6856053	11.57288366
539.1935763	0.558227233	411.7849853	3.036681056	312.9690413	11.574683
539.7604503	0.55656492	411.8849853	2.952992412	314.1935763	11.58237139
540.3868533	0.554165065	411.9849853	2.950349401	314.7604503	11.58594425
540.9849853	0.549652055	412.0849853	3.037286221	315.3868533	11.58993865

Final State: j'=5		Final State: j'=4		Final State: j'=3	
E(cm⁻¹)	σ (Å²)	E(cm⁻¹)	σ (Å²)	E(cm⁻¹)	σ (Å²)
542.6856053	0.568721091	412.1849853	2.996976819	317.6856053	11.60507525
542.9690413	0.582389589	412.2849853	2.970269114	317.9690413	11.6069787
544.1935763	0.579323833	412.3849853	2.949978046	319.1935763	11.61523125
544.7604503	0.574908369	412.4849853	2.932267266	319.7604503	11.61905158
545.3868533	0.571441355	412.5849853	2.914985684	320.3868533	11.62326303
545.9849853	0.567684865	412.6856053	2.896316465	322.6856053	11.63857615
547.6856053	0.564667189	412.9690413	2.818553177	322.9690413	11.64044839
547.9690413	0.574074039	414.1935763	3.015166381	324.1935763	11.64850706
549.1935763	0.598873821	414.7604503	2.990255809	324.7604503	11.65222587
549.7604503	0.621451426	415.3868533	2.965224473	325.3868533	11.65633067
550.3868533	0.605466354	415.9849853	2.942338733	327.6856053	11.67139763
550.9849853	0.604704894	416.0849853	2.937916801	327.9690413	11.67325905
552.6856053	0.592945234	416.1849853	2.932884323	329.1935763	11.68131687
552.9690413	0.583801262	416.2849853	2.926899726	329.7604503	11.68505701
554.1935763	0.636971526	416.3849853	2.91945748	330.3868533	11.68919831
555.3868533	0.597343282	416.4849853	2.909800267	332.6856053	11.70448555
555.9849853	0.570745447	416.5849853	2.897008925	332.9690413	11.70638215
557.6856053	0.663391714	416.6849853	2.883514855	334.1935763	11.71461371
557.9690413	0.648690694	416.7849853	2.914498551	334.7604503	11.71844833
559.1935763	0.610678717	416.8849853	2.977047508	335.3868533	11.72270673
559.7604503	0.632940821	416.9849853	3.048761553	337.6856053	11.73858273
560.3868533	0.68723504	417.0849853	2.97908669	337.9690413	11.74057476
560.9849853	0.65993241	417.6856053	3.054666896	339.1935763	11.74929692
562.6856053	0.629865556	417.9690413	3.06495294	339.7604503	11.75341007
562.9690413	0.620706756	419.1935763	3.04788124	340.3868533	11.75802275
564.1935763	0.691479341	419.7604503	3.041597223	342.6856053	11.77578982
564.7604503	0.661309876	420.3868533	3.038862918	342.9690413	11.77810478
565.3868533	0.66368705	420.9849853	3.037065294	344.1935763	11.78857602
565.9849853	0.65696155	422.6856053	3.045735575	344.7604503	11.79375956
567.6856053	0.624714966	422.9690413	3.052328895	345.3868533	11.79983244
567.9690413	0.63819225	424.1935763	3.067020306	347.6856053	11.82834675
569.1935763	0.68035047	424.7604503	3.064636323	347.9690413	11.83327298
569.7604503	0.688168193	425.3868533	3.063478713	349.1935763	11.86177735
570.3868533	0.700464544	425.9849853	3.134502496	349.7604503	11.8402184
570.9849853	0.671386812	427.6856053	3.128796014	350.3868533	11.79022475
572.6856053	0.691614744	427.9690413	3.131132085	352.6856053	11.80749538
572.9690413	0.711919196	429.1935763	3.143986731	352.9690413	11.8314583
574.1935763	0.726851091	429.7604503	3.150446565	354.1935763	11.8052633
574.7604503	0.695022913	430.3868533	3.157616211	354.7604503	11.78544863
575.3868533	0.682717348	430.9849853	3.164383081	355.3868533	11.82192628
575.9849853	0.696612806	432.6856053	3.181720674	357.6856053	11.79212198
577.6856053	0.694163287	432.9690413	3.184253328	357.9690413	11.77095048
577.9690413	0.703577612	434.1935763	3.208009856	359.1935763	11.78124226

Final State: j’=5		Final State: j’=4		Final State: j’=3	
E(cm⁻¹)	σ (Å²)	E(cm⁻¹)	σ (Å²)	E(cm⁻¹)	σ (Å²)
579.1935763	0.688611297	434.7604503	3.217919516	359.7604503	11.83585079
579.7604503	0.68472102	435.3868533	3.226261127	360.3868533	11.86954154
580.3868533	0.680038448	435.9849853	3.233742499	362.6856053	11.83042636
580.9849853	0.677069489	437.6856053	3.255062055	362.9690413	11.78020562
582.6856053	0.702014778	437.9690413	3.258640393	364.1935763	11.85876124
582.9690413	0.701806947	439.1935763	3.274121555	364.7604503	11.88145416
584.1935763	0.7012081	439.7604503	3.281294173	365.3868533	11.91007928
584.7604503	0.701571631	440.3868533	3.289249857	367.6856053	11.79101944
585.3868533	0.701657021	440.9849853	3.29693284	367.9690413	11.80404285
585.9849853	0.701017304	442.6856053	3.319646824	369.1935763	11.88076156
587.6856053	0.707012926	442.9690413	3.323515591	369.7604503	11.89875622
587.9690413	0.708172929	444.1935763	3.340151483	370.3868533	11.92034067
589.1935763	0.708709607	444.7604503	3.347772094	372.6856053	11.88737072
589.7604503	0.70854943	445.3868533	3.356139481	372.9690413	11.80760708
590.3868533	0.720078265	445.9849853	3.364089197	374.1935763	11.89069187
590.9849853	0.722269199	447.6856053	3.386564585	374.7604503	11.90933322
592.6856053	0.726320566	447.9690413	3.390300337	375.3868533	11.92629195
592.9690413	0.727232544	449.1935763	3.40643295	377.6856053	11.94188195
594.1935763	0.731310339	449.7604503	3.413905346	377.9690413	11.87965708
594.7604503	0.733201432	450.3868533	3.422170945	379.1935763	11.85818646
595.3868533	0.735254014	450.9849853	3.430073969	379.7604503	11.9086532
595.9849853	0.737132969	452.6856053	3.452598197	380.3868533	11.94393731
597.6856053	0.740972483	452.9690413	3.456358004	382.6856053	12.03118767
597.9690413	0.742110807	454.1935763	3.472609679	382.9690413	12.03411883
599.1935763	0.748527895	454.7604503	3.48013435	384.1935763	12.02574087
599.7604503	0.750826267	455.3868533	3.488448338	384.7604503	11.90398434
600.3868533	0.753196624	455.9849853	3.496385498	385.3868533	11.93962386
600.9849853	0.75539858	457.6856053	3.518942537	387.6856053	11.92682487
602.6856053	0.761548592	457.9690413	3.522700785	387.9690413	11.95962192
602.9690413	0.762563466	459.1935763	3.538935166	389.1935763	11.94518254
604.1935763	0.766908389	459.7604503	3.54644991	389.7604503	11.89120092
604.7604503	0.768897114	460.3868533	3.554753947	390.3868533	11.97255514
605.3868533	0.771090022	460.9849853	3.562683698	392.6856053	11.82100096
605.9849853	0.773202619	462.6856053	3.585234444	392.9690413	11.84756058
607.6856053	0.779416812	462.9690413	3.588993743	394.1935763	11.97842972
607.9690413	0.780471845	464.1935763	3.605238008	394.7604503	12.02963622
609.1935763	0.785034418	464.7604503	3.612759513	395.3868533	12.01412606
609.7604503	0.787141531	465.3868533	3.621071952	397.6856053	12.01889191
610.3868533	0.789464551	465.9849853	3.629010215	397.9690413	12.0355951
610.9849853	0.791677733	467.6856053	3.651585193	399.1935763	12.11780922
612.6856053	0.797948478	467.9690413	3.655348331	399.7604503	11.9441084
612.9690413	0.798991199	469.1935763	3.671608315	400.3868533	11.91322277
614.1935763	0.803492292	469.7604503	3.679136685	402.6856053	12.05912158

Final State: j’=5		Final State: j’=4		Final State: j’=3	
E(cm⁻¹)	σ (Å²)	E(cm⁻¹)	σ (Å²)	E(cm⁻¹)	σ (Å²)
614.7604503	0.805575726	470.3868533	3.687456534	402.9690413	12.05668714
615.3868533	0.807879341	470.9849853	3.695401805	404.1935763	11.86848032
615.9849853	0.81008125	472.6856053	3.717997338	404.7604503	11.96490577
617.6856053	0.816357449	472.9690413	3.721764106	405.3868533	12.07399945
617.9690413	0.817405738	474.1935763	3.738040747	407.6856053	12.06454135
619.1935763	0.821940842	474.7604503	3.745577463	407.9690413	12.10820268
619.7604503	0.824043097	475.3868533	3.753907052	409.1935763	11.95353095
620.3868533	0.826367755	475.9849853	3.761862128	409.7604503	11.94769444
620.9849853	0.828588849	477.6856053	3.784488483	410.3868533	11.95663366
622.6856053	0.834909682	477.9690413	3.788260795	411.0849853	12.06629636
622.9690413	0.835963887	479.1935763	3.804562858	411.1849853	12.06522915
624.1935763	0.840520707	479.7604503	3.812112121	411.2849853	12.07246621
624.7604503	0.842631525	480.3868533	3.820456157	411.3849853	12.09078163
625.3868533	0.84496506	480.9849853	3.828425649	411.4849853	12.11119883
625.9849853	0.847194374	482.6856053	3.851096406	411.5849853	12.09864314
627.6856053	0.853539251	482.9690413	3.854876668	411.6849853	12.02966994
627.9690413	0.854597718	484.1935763	3.871214966	411.7849853	11.97305331
629.1935763	0.859174018	484.7604503	3.878782172	411.8849853	12.03782021
629.7604503	0.861294415	485.3868533	3.887146959	411.9849853	12.01020638
630.3868533	0.863638894	485.9849853	3.895137226	412.0849853	11.9793983
630.9849853	0.865878931	487.6856053	3.917872709	412.1849853	12.01350395
632.6856053	0.87225509	487.9690413	3.921664657	412.2849853	12.03257519
632.9690413	0.873318811	489.1935763	3.93805679	412.3849853	12.04596335
634.1935763	0.877917744	489.7604503	3.945650899	412.4849853	12.05706535
634.7604503	0.880048531	490.3868533	3.954047019	412.5849853	12.06747245
635.3868533	0.882404407	490.9849853	3.962068893	412.6856053	12.07827867
635.9849853	0.884655248	492.6856053	3.984904332	412.9690413	12.11689033
637.6856053	0.891061764	492.9690413	3.988714554	414.1935763	11.98995041
637.9690413	0.892130508	494.1935763	4.005191713	414.7604503	12.01847006
639.1935763	0.896751086	494.7604503	4.012828848	415.3868533	12.03796764
639.7604503	0.898891895	495.3868533	4.02127555	415.9849853	12.05095839
640.3868533	0.901258855	495.9849853	4.029348945	416.0849853	12.05337407
640.9849853	0.903520304	497.6856053	4.052350951	416.1849853	12.05611599
642.6856053	0.909957153	497.9690413	4.056192239	416.2849853	12.05932907
642.9690413	0.911030982	499.1935763	4.072816595	416.3849853	12.0631919
644.1935763	0.915673627	499.7604503	4.080529957	416.4849853	12.06788828
644.7604503	0.917824701	500.3868533	4.089067725	416.5849853	12.07326702
645.3868533	0.920203044	500.9849853	4.09723569	416.6849853	12.07543859
645.9849853	0.92247539	502.6856053	4.120556259	416.7849853	12.04406921
647.6856053	0.928943385	502.9690413	4.124459278	416.8849853	12.00537595
647.9690413	0.930022427	504.1935763	4.141386855	416.9849853	11.98221351
649.1935763	0.934687664	504.7604503	4.149264585	417.0849853	12.02249032
649.7604503	0.936849234	505.3868533	4.158006038	417.6856053	11.9620955

Final State: j'=5		Final State: j'=4		Final State: j'=3	
E(cm⁻¹)	σ (Å²)	E(cm⁻¹)	σ (Å²)	E(cm⁻¹)	σ (Å²)
650.3868533	0.939239201	505.9849853	4.166394193	417.9690413	11.9500808
650.9849853	0.941522679	507.6856053	4.190531668	419.1935763	12.00990925
652.6856053	0.948022499	507.9690413	4.19460815	419.7604503	12.01933438
652.9690413	0.94910687	509.1935763	4.212474248	420.3868533	12.02614518
654.1935763	0.953795242	509.7604503	4.220931363	420.9849853	12.03109635
654.7604503	0.955967577	510.3868533	4.230475756	422.6856053	12.02609284
655.3868533	0.95836949	510.9849853	4.23986228	422.9690413	12.02517099
655.9849853	0.96066442	512.6856053	4.269678889	424.1935763	12.03201667
657.6856053	0.967197075	512.9690413	4.275536987	424.7604503	12.03795876
657.9690413	0.96828696	514.1935763	4.264343748	425.3868533	12.01293096
659.1935763	0.972999288	514.7604503	4.259154337	425.9849853	11.98682531
659.7604503	0.975182806	515.3868533	4.289229765	427.6856053	12.02963739
660.3868533	0.977597134	515.9849853	4.268887877	427.9690413	12.03053072
660.9849853	0.979903988	517.6856053	4.327632118	429.1935763	12.03353019
662.6856053	0.986470922	517.9690413	4.315710318	429.7604503	12.03480514
662.9690413	0.987566579	519.1935763	4.310235612	430.3868533	12.03618529
664.1935763	0.992304049	519.7604503	4.337778306	430.9849853	12.03747124
664.7604503	0.994499316	520.3868533	4.341052633	432.6856053	12.04029397
665.3868533	0.996926723	520.9849853	4.366018064	432.9690413	12.03990398
665.9849853	0.999246157	522.6856053	4.378423248	434.1935763	12.03346593
667.6856053	1.005849409	522.9690413	4.339671212	434.7604503	12.03572594
667.9690413	1.006951202	524.1935763	4.398508238	435.3868533	12.03825699
669.1935763	1.011715476	524.7604503	4.416064921	435.9849853	12.04006004
669.7604503	1.013923326	525.3868533	4.401231132	437.6856053	12.0436559
670.3868533	1.016364765	525.9849853	4.387991772	437.9690413	12.0441503
670.9849853	1.018697741	527.6856053	4.427237726	439.1935763	12.04608867
672.6856053	1.025340273	527.9690413	4.437933693	439.7604503	12.04687258
672.9690413	1.026448735	529.1935763	4.471838819	440.3868533	12.04762675
674.1935763	1.031242256	529.7604503	4.48682621	440.9849853	12.04821267
674.7604503	1.0334639	530.3868533	4.434917947	442.6856053	12.04947586
675.3868533	1.035920792	530.9849853	4.468433007	442.9690413	12.04971761
675.9849853	1.038268723	532.6856053	4.49701046	444.1935763	12.0509816
677.6856053	1.044954996	532.9690413	4.505253351	444.7604503	12.05164047
677.9690413	1.046070933	534.1935763	4.534667381	445.3868533	12.05238504
679.1935763	1.050897445	534.7604503	4.548807088	445.9849853	12.05309608
679.7604503	1.053134762	535.3868533	4.56153251	447.6856053	12.05504937
680.3868533	1.055609293	535.9849853	4.508864919	447.9690413	12.05536049
680.9849853	1.057974404	537.6856053	4.551625567	449.1935763	12.05664773
682.6856053	1.06471151	537.9690413	4.565033527	449.7604503	12.05721178
682.9690413	1.065836239	539.1935763	4.596523664	450.3868533	12.05781315
684.1935763	1.070701896	539.7604503	4.608669378	450.9849853	12.05836849
684.7604503	1.072958039	540.3868533	4.622390791	452.6856053	12.05987596
685.3868533	1.075453937	540.9849853	4.636993064	452.9690413	12.06012019

Final State: j'=5		Final State: j'=4		Final State: j'=3	
E(cm⁻¹)	σ (Å²)	E(cm⁻¹)	σ (Å²)	E(cm⁻¹)	σ (Å²)
685.9849853	1.07784006	542.6856053	4.627949652	454.1935763	12.0611602
687.6856053	1.084640603	542.9690413	4.599765875	454.7604503	12.061634
687.9690413	1.085776504	544.1935763	4.659667176	455.3868533	12.06215167
689.1935763	1.090692737	544.7604503	4.675708335	455.9849853	12.06263967
689.7604503	1.092973721	545.3868533	4.691146924	457.6856053	12.06398745
690.3868533	1.095498262	545.9849853	4.705983834	457.9690413	12.06420564
690.9849853	1.097913038	547.6856053	4.715256448	459.1935763	12.06512525
692.6856053	1.10480338	547.9690413	4.708165627	459.7604503	12.06553771
692.9690413	1.10595567	549.1935763	4.669184952	460.3868533	12.06598351
694.1935763	1.11094852	549.7604503	4.663938429	460.9849853	12.06639952
694.7604503	1.113268719	550.3868533	4.721479607	462.6856053	12.06753118
695.3868533	1.115839566	550.9849853	4.707049978	462.9690413	12.06771264
695.9849853	1.118303087	552.6856053	4.785806599	464.1935763	12.06847409
697.6856053	1.125357937	552.9690413	4.786066393	464.7604503	12.06881448
697.9690413	1.126542671	554.1935763	4.757212972	465.3868533	12.06918196
699.1935763	1.131699384	554.7604503	4.79675133	465.9849853	12.06952448
699.7604503	1.134112459	555.3868533	4.829920429	467.6856053	12.07045445
700.3868533	1.136803827	555.9849853	4.817697355	467.9690413	12.07060319
700.9849853	1.139404557	557.6856053	4.790641198	469.1935763	12.07122545
702.6856053	1.147071342	557.9690413	4.818521045	469.7604503	12.07150237
702.9690413	1.148415173	559.1935763	4.878846876	470.3868533	12.07180017
704.1935763	1.154765244	559.7604503	4.809075242	470.9849853	12.07207657
704.7604503	1.158404791	560.3868533	4.798678843	472.6856053	12.07281994
705.3868533	1.158556631	560.9849853	4.857891207	472.9690413	12.07293777
705.9849853	1.153972403	562.6856053	4.925743689	474.1935763	12.07342712
707.6856053	1.15873333	562.9690413	4.938737374	474.7604503	12.07364288
707.9690413	1.161849321	564.1935763	4.826661028	475.3868533	12.07387347
709.1935763	1.173592398	564.7604503	4.908773852	475.9849853	12.07408602
709.7604503	1.170521928	565.3868533	4.914936652	477.6856053	12.07465028
710.3868533	1.171528559	565.9849853	4.93392935	477.9690413	12.07473859
710.9849853	1.174059809	567.6856053	4.992019381	479.1935763	12.07510186
712.6856053	1.187834066	567.9690413	4.927859612	479.7604503	12.07525997
712.9690413	1.186148443	569.1935763	4.978124011	480.3868533	12.0754274
714.1935763	1.190435963	569.7604503	4.935735273	480.9849853	12.07558021
714.7604503	1.184511429	570.3868533	4.972229481	482.6856053	12.07597748
715.3868533	1.193508063	570.9849853	5.023158043	482.9690413	12.07603843
715.9849853	1.198656719	572.6856053	4.935871712	484.1935763	12.07628468
717.6856053	1.199032314	572.9690413	4.954764944	484.7604503	12.0763894
717.9690413	1.19964353	574.1935763	5.003466591	485.3868533	12.07649847
719.1935763	1.206482783	574.7604503	5.047613913	485.9849853	12.07659608
719.7604503	1.211315801	575.3868533	5.057410079	487.6856053	12.0768396
720.3868533	1.21581435	575.9849853	5.02140017	487.9690413	12.07687535
720.9849853	1.220542064	577.6856053	5.00413623	489.1935763	12.07701459

Final State: j'=5		Final State: j'=4		Final State: j'=3	
E(cm⁻¹)	σ (Å²)	E(cm⁻¹)	σ (Å²)	E(cm⁻¹)	σ (Å²)
722.6856053	1.223118607	577.9690413	5.028100676	489.7604503	12.0770707
722.9690413	1.212261049	579.1935763	5.079384106	490.3868533	12.07712673
724.1935763	1.2277552	579.7604503	5.090678267	490.9849853	12.07717444
724.7604503	1.231787577	580.3868533	5.101514614	492.6856053	12.07728027
725.3868533	1.23580358	580.9849853	5.0938723	492.9690413	12.07729372
725.9849853	1.2397705	582.6856053	5.097749058	494.1935763	12.07733881
727.6856053	1.237655474	582.9690413	5.107880015	494.7604503	12.07735271
727.9690413	1.24161282	584.1935763	5.130563315	495.3868533	12.07736311
729.1935763	1.245887279	584.7604503	5.138657826	495.9849853	12.07736837
729.7604503	1.251337499	585.3868533	5.146878011	497.6856053	12.07735998
730.3868533	1.25547324	585.9849853	5.153057495	497.9690413	12.07735546
730.9849853	1.259075429	587.6856053	5.16863339	499.1935763	12.07732672
732.6856053	1.270466836	587.9690413	5.172538241	499.7604503	12.07730883
732.9690413	1.273314831	589.1935763	5.190241922	500.3868533	12.07728613
734.1935763	1.270454174	589.7604503	5.181304121	500.9849853	12.07726195
734.7604503	1.267661035	590.3868533	5.190314113	502.6856053	12.07718383
735.3868533	1.274540541	590.9849853	5.205269807	502.9690413	12.07717008
735.9849853	1.279814565	592.6856053	5.226252846	504.1935763	12.07711155
737.6856053	1.291538804	592.9690413	5.229586505	504.7604503	12.07708644
737.9690413	1.293744666	594.1935763	5.243983335	505.3868533	12.07706185
739.1935763	1.2956338	594.7604503	5.250635735	505.9849853	12.07704299
739.7604503	1.297897881	595.3868533	5.257965004	507.6856053	12.07703381
740.3868533	1.295143371	595.9849853	5.264924288	507.9690413	12.07704229
740.9849853	1.292766843	597.6856053	5.282327578	509.1935763	12.07713688
742.6856053	1.293901122	597.9690413	5.284793671	509.7604503	12.0772283
742.9690413	1.293976981	599.1935763	5.299612906	510.3868533	12.07738708
744.1935763	1.317195436	599.7604503	5.306559217	510.9849853	12.07762706
744.7604503	1.31329922	600.3868533	5.314031526	512.6856053	12.07977772
745.3868533	1.299782767	600.9849853	5.321057105	512.9690413	12.08089451
745.9849853	1.317094652	602.6856053	5.340760116	514.1935763	12.09248546
747.6856053	1.322113738	602.9690413	5.344019047	514.7604503	12.08317029
747.9690413	1.304918002	604.1935763	5.358018763	515.3868533	12.0841276
749.1935763	1.323511174	604.7604503	5.364444715	515.9849853	12.09053631
749.7604503	1.333788193	605.3868533	5.371499657	517.6856053	12.08590572
750.3868533	1.341655703	605.9849853	5.378205369	517.9690413	12.0906928
750.9849853	1.346069028	607.6856053	5.397332681	519.1935763	12.07970905
752.6856053	1.345168877	607.9690413	5.400539478	519.7604503	12.08138704
752.9690413	1.348213913	609.1935763	5.41441782	520.3868533	12.07522248
754.1935763	1.360641905	609.7604503	5.420842795	520.9849853	12.08102169
754.7604503	1.369531147	610.3868533	5.427936363	522.6856053	12.0781839
755.3868533	1.347557879	610.9849853	5.434701659	522.9690413	12.08683859
755.9849853	1.350085726	612.6856053	5.453884409	524.1935763	12.07197278
757.6856053	1.365937195	612.9690413	5.457073383	524.7604503	12.07602722

Final State: j’=5		Final State: j’=4		Final State: j’=3	
E(cm⁻¹)	σ (Å²)	E(cm⁻¹)	σ (Å²)	E(cm⁻¹)	σ (Å²)
757.9690413	1.368221285	614.1935763	5.470823778	525.3868533	12.09184364
759.1935763	1.385750363	614.7604503	5.47717524	525.9849853	12.08161821
759.7604503	1.349431912	615.3868533	5.484184547	527.6856053	12.06638568
760.3868533	1.371245416	615.9849853	5.490869569	527.9690413	12.06702924
760.9849853	1.388646134	617.6856053	5.509841546	529.1935763	12.07109947
762.6856053	1.397467875	617.9690413	5.512999058	529.7604503	12.07844677
762.9690413	1.394813714	619.1935763	5.526626316	530.3868533	12.08956432
764.1935763	1.382472718	619.7604503	5.532926785	530.9849853	12.0713293
764.7604503	1.38888872	620.3868533	5.539882784	532.6856053	12.06313107
765.3868533	1.381199845	620.9849853	5.546518645	532.9690413	12.06369191
765.9849853	1.397989681	622.6856053	5.565350902	534.1935763	12.06625038
767.6856053	1.391586959	622.9690413	5.568484439	534.7604503	12.0688307
767.9690413	1.407421526	624.1935763	5.582004823	535.3868533	12.0803317
769.1935763	1.401849378	624.7604503	5.588254124	535.9849853	12.08507912
769.7604503	1.409168701	625.3868533	5.595152585	537.6856053	12.0581794
770.3868533	1.414877746	625.9849853	5.601732725	537.9690413	12.05898353
770.9849853	1.419012894	627.6856053	5.620404589	539.1935763	12.06112726
772.6856053	1.425892987	627.9690413	5.62351132	539.7604503	12.0620084
772.9690413	1.426482308	629.1935763	5.6369162	540.3868533	12.0634135
774.1935763	1.426763005	629.7604503	5.643112377	540.9849853	12.06624269
774.7604503	1.431130235	630.3868533	5.649952376	542.6856053	12.07203893
775.3868533	1.434510066	630.9849853	5.656476957	542.9690413	12.07679278
775.9849853	1.437395138	632.6856053	5.674992065	544.1935763	12.05660898
777.6856053	1.444714326	632.9690413	5.678072774	544.7604503	12.05810437
777.9690413	1.44562321	634.1935763	5.691365523	545.3868533	12.05957018
779.1935763	1.450135849	634.7604503	5.697509837	545.9849853	12.06135114
779.7604503	1.452669565	635.3868533	5.704292538	547.6856053	12.07943353
780.3868533	1.455665153	635.9849853	5.710762425	547.9690413	12.07772308
780.9849853	1.457626736	637.6856053	5.729121871	549.1935763	12.0801285
782.6856053	1.463763897	637.9690413	5.732176638	549.7604503	12.07570739
782.9690413	1.46501883	639.1935763	5.745357222	550.3868533	12.06063626
784.1935763	1.470142657	639.7604503	5.751449617	550.9849853	12.07611048
784.7604503	1.472473908	640.3868533	5.758174955	552.6856053	12.06223384
785.3868533	1.475047356	640.9849853	5.764590103	552.9690413	12.07516682
785.9849853	1.477505078	642.6856053	5.782794065	554.1935763	12.05135199
787.6856053	1.484505789	642.9690413	5.785822932	554.7604503	12.05374858
787.9690413	1.485675031	644.1935763	5.798891847	555.3868533	12.06208787
789.1935763	1.490467831	644.7604503	5.804932594	555.9849853	12.10504871
789.7604503	1.492494335	645.3868533	5.811600949	557.6856053	12.03916537
790.3868533	1.495018905	645.9849853	5.817961687	557.9690413	12.04200175
790.9849853	1.497505654	647.6856053	5.836011306	559.1935763	12.05993475
792.6856053	1.504505954	647.9690413	5.839014493	559.7604503	12.0830235
792.9690413	1.505665762	649.1935763	5.851972538	560.3868533	12.04240806

Final State: j'=5		Final State: j'=4		Final State: j'=3	
E(cm⁻¹)	σ (Å²)	E(cm⁻¹)	σ (Å²)	E(cm⁻¹)	σ (Å²)
794.1935763	1.510667692	649.7604503	5.85796204	560.9849853	12.04163048
794.7604503	1.51298015	650.3868533	5.864573806	562.6856053	12.05155266
795.3868533	1.515533304	650.9849853	5.87088059	562.9690413	12.0567772
795.9849853	1.517967999	652.6856053	5.888777005	564.1935763	12.06051514
797.6856053	1.524859158	652.9690413	5.891754707	564.7604503	12.04994544
797.9690413	1.526003956	654.1935763	5.904602777	565.3868533	12.05112665
799.1935763	1.530954432	654.7604503	5.910541446	565.9849853	12.05337137
799.7604503	1.533251512	655.3868533	5.917097095	567.6856053	12.07297391
800.3868533	1.535793335	655.9849853	5.923350356	567.9690413	12.09695027
800.9849853	1.53822291	657.6856053	5.941094962	569.1935763	12.03849249
802.6856053	1.545137786	657.9690413	5.944047399	569.7604503	12.06297308
802.9690413	1.546290811	659.1935763	5.956786547	570.3868533	12.03576019
804.1935763	1.551272886	659.7604503	5.96267487	570.9849853	12.0418384
804.7604503	1.553579307	660.3868533	5.969174984	572.6856053	12.09047977
805.3868533	1.556127805	660.9849853	5.975375283	572.9690413	12.05916297
805.9849853	1.558561077	662.6856053	5.992969722	574.1935763	12.02911988
807.6856053	1.565478491	662.9690413	5.9958972	574.7604503	12.04067198
807.9690413	1.566631362	664.1935763	6.008528661	575.3868533	12.03932713
809.1935763	1.571612539	664.7604503	6.014367282	575.9849853	12.04504288
809.7604503	1.57391886	665.3868533	6.020812519	577.6856053	12.06113177
810.3868533	1.576467757	665.9849853	6.026960513	577.9690413	12.03759169
810.9849853	1.578902017	667.6856053	6.04440671	579.1935763	12.0405588
812.6856053	1.585825361	667.9690413	6.047309552	579.7604503	12.04271257
812.9690413	1.586979558	669.1935763	6.05983484	580.3868533	12.04606748
814.1935763	1.591966986	669.7604503	6.065624438	580.9849853	12.05889185
814.7604503	1.594276295	670.3868533	6.07201564	582.6856053	12.03712952
815.3868533	1.596828444	670.9849853	6.078112108	582.9690413	12.03710862
815.9849853	1.59926571	672.6856053	6.09541246	584.1935763	12.03833096
817.6856053	1.606196936	672.9690413	6.098291082	584.7604503	12.03871545
817.9690413	1.607352354	674.1935763	6.110712038	585.3868533	12.03931067
819.1935763	1.612344834	674.7604503	6.116453513	585.9849853	12.04022355
819.7604503	1.614656399	675.3868533	6.122791657	587.6856053	12.03901543
820.3868533	1.617211001	675.9849853	6.128837629	587.9690413	12.0387714
820.9849853	1.619650604	677.6856053	6.145995122	589.1935763	12.04057484
822.6856053	1.626588557	677.9690413	6.148850045	589.7604503	12.04051909
822.9690413	1.627745122	679.1935763	6.161169053	590.3868533	12.03410999
824.1935763	1.632742637	679.7604503	6.166863579	590.9849853	12.03396165
824.7604503	1.635056578	680.3868533	6.173150008	592.6856053	12.03383951
825.3868533	1.637613845	680.9849853	6.179146798	592.9690413	12.03367417
825.9849853	1.640056018	682.6856053	6.196165541	594.1935763	12.03287613
827.6856053	1.64700138	682.9690413	6.198997525	594.7604503	12.03250136
827.9690413	1.648159187	684.1935763	6.211217971	595.3868533	12.03210636
829.1935763	1.653162108	684.7604503	6.216867228	595.9849853	12.03177242

Final State: j'=5		Final State: j'=4		Final State: j'=3	
E(cm⁻¹)	σ (Å²)	E(cm⁻¹)	σ (Å²)	E(cm⁻¹)	σ (Å²)
829.7604503	1.655478542	685.3868533	6.223103939	597.6856053	12.03123605
830.3868533	1.658038601	685.9849853	6.22905352	597.9690413	12.03075208
830.9849853	1.660483425	687.6856053	6.245939902	599.1935763	12.02864332
832.6856053	1.667436363	687.9690413	6.248750103	599.7604503	12.02808772
832.9690413	1.668595438	689.1935763	6.260877528	600.3868533	12.02753567
834.1935763	1.673603848	689.7604503	6.266484366	600.9849853	12.02702411
834.7604503	1.675922854	690.3868533	6.27267477	602.6856053	12.02558313
835.3868533	1.678485729	690.9849853	6.278580727	602.9690413	12.0253441
835.9849853	1.68093327	692.6856053	6.295346774	604.1935763	12.02431702
837.6856053	1.687893988	692.9690413	6.29813754	604.7604503	12.02384098
837.9690413	1.68905437	694.1935763	6.310183619	605.3868533	12.02330447
839.1935763	1.694068452	694.7604503	6.31575447	605.9849853	12.02277119
839.7604503	1.696390097	695.3868533	6.321904434	607.6856053	12.02114739
840.3868533	1.698955906	695.9849853	6.327777644	607.9690413	12.02087147
840.9849853	1.701406263	697.6856053	6.34445659	609.1935763	12.01968784
842.6856053	1.708375039	697.9690413	6.347235144	609.7604503	12.01914523
842.9690413	1.709536774	699.1935763	6.359239469	610.3868533	12.01854812
844.1935763	1.714556729	699.7604503	6.364799111	610.9849853	12.01797991
844.7604503	1.71688111	700.3868533	6.370947488	612.6856053	12.01636731
845.3868533	1.719449946	700.9849853	6.376826683	612.9690413	12.01609819
845.9849853	1.721903211	702.6856053	6.393652962	614.1935763	12.01493146
847.6856053	1.728880309	702.9690413	6.396491821	614.7604503	12.01438821
847.9690413	1.730043439	704.1935763	6.40918012	615.3868533	12.01378507
849.1935763	1.735069451	704.7604503	6.416138913	615.9849853	12.01320621
849.7604503	1.737396653	705.3868533	6.427795687	617.6856053	12.01154585
850.3868533	1.739968621	705.9849853	6.435429796	617.9690413	12.01126731
850.9849853	1.742424884	707.6856053	6.449534738	619.1935763	12.01005899
852.6856053	1.74941057	707.9690413	6.447183457	619.7604503	12.00949722
852.9690413	1.75057514	709.1935763	6.462273451	620.3868533	12.0088749
854.1935763	1.755607409	709.7604503	6.472668988	620.9849853	12.0082793
854.7604503	1.757937522	710.3868533	6.471762777	622.6856053	12.00657733
855.3868533	1.76051272	710.9849853	6.475467343	622.9690413	12.00629255
855.9849853	1.762972074	712.6856053	6.495594429	624.1935763	12.00505818
857.6856053	1.76996663	712.9690413	6.504071925	624.7604503	12.00448447
857.9690413	1.771132689	714.1935763	6.507324409	625.3868533	12.00384883
859.1935763	1.776171408	714.7604503	6.512415957	625.9849853	12.00324018
859.7604503	1.778504525	715.3868533	6.514497308	627.6856053	12.00150029
860.3868533	1.781083056	715.9849853	6.521331136	627.9690413	12.00120897
860.9849853	1.78354561	717.6856053	6.54014947	629.1935763	11.99994597
862.6856053	1.790549316	717.9690413	6.544416892	629.7604503	11.99935887
862.9690413	1.791716908	719.1935763	6.549308535	630.3868533	11.99870838
864.1935763	1.79676229	719.7604503	6.55519619	630.9849853	11.99808551
864.7604503	1.799098508	720.3868533	6.561956627	632.6856053	11.99630556

Final State: j’=5		Final State: j’=4		Final State: j’=3	
E(cm⁻¹)	σ (Å²)	E(cm⁻¹)	σ (Å²)	E(cm⁻¹)	σ (Å²)
865.3868533	1.801680481	720.9849853	6.569400342	632.9690413	11.9960076
865.9849853	1.80414633	722.6856053	6.588387968	634.1935763	11.99471611
867.6856053	1.811159481	722.9690413	6.596553395	634.7604503	11.99411591
867.9690413	1.812328656	724.1935763	6.595687994	635.3868533	11.99345099
869.1935763	1.817380914	724.7604503	6.601517732	635.9849853	11.99281437
869.7604503	1.819720334	725.3868533	6.60800364	637.6856053	11.99099538
870.3868533	1.822305854	725.9849853	6.614504927	637.9690413	11.99069091
870.9849853	1.824775103	727.6856053	6.636453938	639.1935763	11.98937128
872.6856053	1.831797992	727.9690413	6.63566144	639.7604503	11.98875804
872.9690413	1.832968801	729.1935763	6.641205271	640.3868533	11.98807869
874.1935763	1.838028147	729.7604503	6.647106296	640.9849853	11.9874283
874.7604503	1.840370867	730.3868533	6.6534766	642.6856053	11.98556998
875.3868533	1.842960049	730.9849853	6.659518708	642.9690413	11.98525894
875.9849853	1.845432806	732.6856053	6.678539772	644.1935763	11.98391091
877.6856053	1.852465741	732.9690413	6.683903552	644.7604503	11.98328452
877.9690413	1.853638232	734.1935763	6.695390966	645.3868533	11.9825906
879.1935763	1.85870489	734.7604503	6.700859394	645.9849853	11.98192625
879.7604503	1.861051014	735.3868533	6.698489726	647.6856053	11.98002827
880.3868533	1.863643975	735.9849853	6.704946595	647.9690413	11.97971062
880.9849853	1.86612035	737.6856053	6.723016055	649.1935763	11.97833392
882.6856053	1.873163656	737.9690413	6.726389807	649.7604503	11.97769417
882.9690413	1.874337888	739.1935763	6.749941805	650.3868533	11.97698552
884.1935763	1.879412106	739.7604503	6.751509125	650.9849853	11.9763071
884.7604503	1.881761766	740.3868533	6.770205038	652.6856053	11.97436891
885.3868533	1.884358617	740.9849853	6.767523693	652.9690413	11.97404454
885.9849853	1.886838741	742.6856053	6.784153579	654.1935763	11.97263869
887.6856053	1.893892797	742.9690413	6.777646143	654.7604503	11.97198544
887.9690413	1.895068836	744.1935763	6.784922515	655.3868533	11.97126177
889.1935763	1.900150897	744.7604503	6.807490481	655.9849853	11.97056899
889.7604503	1.902504199	745.3868533	6.801376768	657.6856053	11.96858974
890.3868533	1.905105129	745.9849853	6.797155693	657.9690413	11.96825847
890.9849853	1.907589149	747.6856053	6.852796271	659.1935763	11.96682283
892.6856053	1.914654421	747.9690413	6.848807461	659.7604503	11.96615569
892.9690413	1.915832346	749.1935763	6.82140312	660.3868533	11.96541666
894.1935763	1.920922621	749.7604503	6.828658195	660.9849853	11.96470916
894.7604503	1.923279761	750.3868533	6.837537519	662.6856053	11.96268777
895.3868533	1.925884959	750.9849853	6.859889706	662.9690413	11.96234945
895.9849853	1.928373091	752.6856053	6.85466233	664.1935763	11.96088311
897.6856053	1.935450192	752.9690413	6.857768004	664.7604503	11.96020173
897.9690413	1.936630118	754.1935763	6.872806606	665.3868533	11.95944682
899.1935763	1.941729116	754.7604503	6.88766744	665.9849853	11.95872413
899.7604503	1.944090353	755.3868533	6.910652145	667.6856053	11.95665915
900.3868533	1.946700117	755.9849853	6.887399761	667.9690413	11.95631351

Final State: j'=5		Final State: j'=4		Final State: j'=3	
E(cm⁻¹)	σ (Å²)	E(cm⁻¹)	σ (Å²)	E(cm⁻¹)	σ (Å²)
900.9849853	1.949192653	757.6856053	6.909676391	669.1935763	11.95481536
902.6856053	1.956282551	757.9690413	6.90958663	669.7604503	11.95411913
902.9690413	1.957464652	759.1935763	6.931451939	670.3868533	11.95334778
904.1935763	1.962573211	759.7604503	6.950688098	670.9849853	11.95260924
904.7604503	1.964938965	760.3868533	6.92225058	672.6856053	11.95049884
905.3868533	1.967553805	760.9849853	6.937652315	672.9690413	11.95014557
905.9849853	1.970051273	762.6856053	6.949143026	674.1935763	11.94861415
907.6856053	1.977155719	762.9690413	6.970012262	674.7604503	11.94790237
907.9690413	1.978340325	764.1935763	6.990516445	675.3868533	11.94711373
909.1935763	1.983460067	764.7604503	6.966677455	675.9849853	11.94635862
909.7604503	1.985831213	765.3868533	6.981214433	677.6856053	11.94420039
910.3868533	1.988452186	765.9849853	6.97095591	677.9690413	11.94383904
910.9849853	1.990955712	767.6856053	7.007395915	679.1935763	11.94227247
912.6856053	1.998078681	767.9690413	6.997231268	679.7604503	11.94154426
912.9690413	1.99926661	769.1935763	7.022458949	680.3868533	11.94073728
914.1935763	2.00440165	769.7604503	7.01477492	680.9849853	11.93996451
914.7604503	2.006780516	770.3868533	7.020492213	682.6856053	11.93775522
915.3868533	2.009410619	770.9849853	7.026879118	682.9690413	11.93738524
915.9849853	2.011923557	772.6856053	7.047761546	684.1935763	11.93578089
917.6856053	2.019078513	772.9690413	7.052643901	684.7604503	11.93503489
917.9690413	2.020272801	774.1935763	7.055036595	685.3868533	11.93420806
919.1935763	2.025440686	774.7604503	7.060273191	685.9849853	11.93341611
919.7604503	2.027838939	775.3868533	7.066303273	687.6856053	11.93115099
920.3868533	2.030495264	775.9849853	7.071983025	687.9690413	11.9307715
920.9849853	2.033040267	777.6856053	7.088576149	689.1935763	11.92912523
922.6856053	2.040386043	777.9690413	7.091293836	689.7604503	11.92835936
922.9690413	2.041653365	779.1935763	7.101698062	690.3868533	11.9275102
924.1935763	2.048718297	779.7604503	7.106670802	690.9849853	11.9266965
924.7604503	2.051726549	780.3868533	7.11255596	692.6856053	11.92436702
925.3868533	2.051853994	780.9849853	7.12071882	692.9690413	11.92397636
925.9849853	2.055788566	782.6856053	7.131379638	694.1935763	11.92228022
927.6856053	2.061486572	782.9690413	7.133988744	694.7604503	11.92149019
927.9690413	2.06357939	784.1935763	7.144931734	695.3868533	11.92061072
929.1935763	2.068200523	784.7604503	7.149920491	695.9849853	11.91977228
929.7604503	2.07099972	785.3868533	7.155415245	697.6856053	11.91735739
930.3868533	2.073197426	785.9849853	7.160656143	697.9690413	11.91695119
930.9849853	2.075128692	787.6856053	7.175593076	699.1935763	11.9151816
932.6856053	2.082252093	787.9690413	7.178104009	699.7604503	11.91435315
932.9690413	2.084109393	789.1935763	7.189026406	700.3868533	11.91342943
934.1935763	2.088150823	789.7604503	7.193697172	700.9849853	11.91253774
934.7604503	2.090852065	790.3868533	7.198878604	702.6856053	11.90992453
935.3868533	2.09443212	790.9849853	7.203977033	702.9690413	11.90947142
935.9849853	2.09989214	792.6856053	7.21862892	704.1935763	11.90738313

Final State: j’=5		Final State: j’=4		Final State: j’=3	
E(cm⁻¹)	σ (Å²)	E(cm⁻¹)	σ (Å²)	E(cm⁻¹)	σ (Å²)
937.6856053	2.103388799	792.9690413	7.221072536	704.7604503	11.90628138
937.9690413	2.103704026	794.1935763	7.231623441	705.3868533	11.90594743
939.1935763	2.109347406	794.7604503	7.236504449	705.9849853	11.90660961
939.7604503	2.112124796	795.3868533	7.241895925	707.6856053	11.90466088
940.3868533	2.117182417	795.9849853	7.247041516	707.9690413	11.90381756
940.9849853	2.11936365	797.6856053	7.261633252	709.1935763	11.90086043
942.6856053	2.123410762	797.9690413	7.264056045	709.7604503	11.90097045
942.9690413	2.124732365	799.1935763	7.274499473	710.3868533	11.90080862
944.1935763	2.130272805	799.7604503	7.279326933	710.9849853	11.89955692
944.7604503	2.132876208	800.3868533	7.284658891	712.6856053	11.89520195
945.3868533	2.136021894	800.9849853	7.28974811	712.9690413	11.89580882
945.9849853	2.14162357	802.6856053	7.304204906	714.1935763	11.89502837
947.6856053	2.145532219	802.9690413	7.306612186	714.7604503	11.89480579
947.9690413	2.145554585	804.1935763	7.317004129	715.3868533	11.89257146
949.1935763	2.151191701	804.7604503	7.321809925	715.9849853	11.8909201
949.7604503	2.153723038	805.3868533	7.327116424	717.6856053	11.8914607
950.3868533	2.156532839	805.9849853	7.332179327	717.9690413	11.88987538
950.9849853	2.159280226	807.6856053	7.346551091	719.1935763	11.88754563
952.6856053	2.168165995	807.9690413	7.348942995	719.7604503	11.88596669
952.9690413	2.169040859	809.1935763	7.359265769	720.3868533	11.8844427
954.1935763	2.17209493	809.7604503	7.364038597	720.9849853	11.88283512
954.7604503	2.174808775	810.3868533	7.369308429	722.6856053	11.88315087
955.3868533	2.177694296	810.9849853	7.37433634	722.9690413	11.88307051
955.9849853	2.180454521	812.6856053	7.388610545	724.1935763	11.87967261
957.6856053	2.190457598	812.9690413	7.390986534	724.7604503	11.87827957
957.9690413	2.192851463	814.1935763	7.401241641	725.3868533	11.87685727
959.1935763	2.200050096	814.7604503	7.405983568	725.9849853	11.87544123
959.7604503	2.200484431	815.3868533	7.41121942	727.6856053	11.87732359
960.3868533	2.199822538	815.9849853	7.41621498	727.9690413	11.87407543
960.9849853	2.204108161	817.6856053	7.430397137	729.1935763	11.87261608
962.6856053	2.208763068	817.9690413	7.432757752	729.7604503	11.87076009
962.9690413	2.210569121	819.1935763	7.442946196	730.3868533	11.86927195
964.1935763	2.218077917	819.7604503	7.447657144	730.9849853	11.86795065
964.7604503	2.217028746	820.3868533	7.452858713	732.6856053	11.86371535
965.3868533	2.220356386	820.9849853	7.457821493	732.9690413	11.86261674
965.9849853	2.224543759	822.6856053	7.471910305	734.1935763	11.86401147
967.6856053	2.229202224	822.9690413	7.474255359	734.7604503	11.86455559
967.9690413	2.230767576	824.1935763	7.484376639	735.3868533	11.86190729
969.1935763	2.236396688	824.7604503	7.489056504	735.9849853	11.86003141
969.7604503	2.242564591	825.3868533	7.494223742	737.6856053	11.85583623
970.3868533	2.24702893	825.9849853	7.499153769	737.9690413	11.85508834
970.9849853	2.242588488	827.6856053	7.513149562	739.1935763	11.85390925
972.6856053	2.251103165	827.9690413	7.515479124	739.7604503	11.85219926

Final State: j’=5		Final State: j’=4		Final State: j’=3	
E(cm⁻¹)	σ (Å²)	E(cm⁻¹)	σ (Å²)	E(cm⁻¹)	σ (Å²)
972.9690413	2.252639522	829.1935763	7.525533434	740.3868533	11.85387523
974.1935763	2.265732522	829.7604503	7.530182299	740.9849853	11.85518739
974.7604503	2.261309576	830.3868533	7.535315307	742.6856053	11.85285096
975.3868533	2.263796455	830.9849853	7.540212628	742.9690413	11.85228907
975.9849853	2.265463114	832.6856053	7.55411538	744.1935763	11.84509491
977.6856053	2.275759955	832.9690413	7.556429422	744.7604503	11.84643506
977.9690413	2.276172614	834.1935763	7.566416695	745.3868533	11.84873964
979.1935763	2.277433453	834.7604503	7.571034542	745.9849853	11.84372432
979.7604503	2.281898814	835.3868533	7.576133218	747.6856053	11.84156239
980.3868533	2.288775388	835.9849853	7.580997759	747.9690413	11.84807006
980.9849853	2.28574538	837.6856053	7.594807281	749.1935763	11.84109697
982.6856053	2.297330103	837.9690413	7.597105796	749.7604503	11.83745544
982.9690413	2.30288075	839.1935763	7.607025886	750.3868533	11.83475116
984.1935763	2.305105735	839.7604503	7.611612613	750.9849853	11.83378601
984.7604503	2.301454961	840.3868533	7.616676896	752.6856053	11.83225541
985.3868533	2.304958466	840.9849853	7.621508588	752.9690413	11.8312089
985.9849853	2.310140025	842.6856053	7.635224639	754.1935763	11.8267547
987.6856053	2.321409549	842.9690413	7.637507553	754.7604503	11.8235624
987.9690413	2.321975155	844.1935763	7.647360262	755.3868533	11.82880061
989.1935763	2.323412297	844.7604503	7.651915767	755.9849853	11.83066846
989.7604503	2.326068447	845.3868533	7.656945547	757.6856053	11.82356005
990.3868533	2.329106514	845.9849853	7.661744274	757.9690413	11.82279486
990.9849853	2.332135882	847.6856053	7.675366477	759.1935763	11.81663573
992.6856053	2.339497045	847.9690413	7.677633735	759.7604503	11.82640845
992.9690413	2.340116959	849.1935763	7.68741875	760.3868533	11.82022386
994.1935763	2.34545579	849.7604503	7.691942896	760.9849853	11.8153771
994.7604503	2.347981095	850.3868533	7.696938002	762.6856053	11.81088258
995.3868533	2.35077489	850.9849853	7.701703592	762.9690413	11.81285386
995.9849853	2.353473699	852.6856053	7.715231466	764.1935763	11.81641384
997.6856053	2.360972928	852.9690413	7.71748298	764.7604503	11.81394864
997.9690413	2.36217149	854.1935763	7.727199961	765.3868533	11.81545845
999.1935763	2.367492338	854.7604503	7.731692556	765.9849853	11.80889029
999.7604503	2.370444641	855.3868533	7.736652774	767.6856053	11.80863645
1000.386853	2.372828828	855.9849853	7.741385036	767.9690413	11.8040995
1000.984983	2.374987029	857.6856053	7.754817998	769.1935763	11.80554149
1002.685603	2.382487204	857.9690413	7.757053672	769.7604503	11.8024166
1002.969043	2.383720157	859.1935763	7.766702116	770.3868533	11.80018349
1004.193573	2.389035831	859.7604503	7.771162975	770.9849853	11.79840343
1004.760453	2.39149528	860.3868533	7.776088067	772.6856053	11.79542266
1005.386853	2.394214093	860.9849853	7.780786752	772.9690413	11.79543382
1005.984983	2.396812424	862.6856053	7.794124081	774.1935763	11.79419339
1007.685603	2.404246319	862.9690413	7.796343796	774.7604503	11.79231299
1007.969043	2.405491322	864.1935763	7.8059232	775.3868533	11.79074271

Final State: j’=5		Final State: j’=4		Final State: j’=3	
E(cm⁻¹)	σ (Å²)	E(cm⁻¹)	σ (Å²)	E(cm⁻¹)	σ (Å²)
1009.193573	2.410732995	864.7604503	7.810352023	775.9849853	11.78937212
1009.760453	2.413171744	865.3868533	7.815241699	777.6856053	11.78577447
1010.386853	2.415879302	865.9849853	7.819906523	777.9690413	11.78529323
1010.984983	2.41846907	867.6856053	7.833147401	779.1935763	11.7830538
1012.685603	2.425839571	867.9690413	7.835350999	779.7604503	11.78182974
1012.969043	2.427068553	869.1935763	7.844860713	780.3868533	11.7803701
1014.193573	2.432379878	869.7604503	7.84925722	780.9849853	11.77948333
1014.760453	2.434839666	870.3868533	7.854111148	782.6856053	11.77639186
1015.386853	2.437558345	870.9849853	7.858741787	782.9690413	11.77576656
1015.984983	2.440154332	872.6856053	7.87188524	784.1935763	11.7732062
1017.685603	2.447530271	872.9690413	7.874072565	784.7604503	11.77203845
1017.969043	2.448758953	874.1935763	7.883511857	785.3868533	11.77074692
1019.193573	2.454067261	874.7604503	7.8878757	785.9849853	11.76951065
1019.760453	2.456525031	875.3868533	7.892693486	787.6856053	11.76597304
1020.386853	2.459241286	875.9849853	7.897289573	787.9690413	11.76538057
1020.984983	2.461835313	877.6856053	7.910334492	789.1935763	11.76296753
1022.685603	2.46921243	877.9690413	7.912505369	789.7604503	11.7619346
1022.969043	2.470442162	879.1935763	7.921873415	790.3868533	11.76062713
1024.193573	2.475755543	879.7604503	7.926204201	790.9849853	11.75934178
1024.760453	2.478215501	880.3868533	7.930985408	792.6856053	11.75571786
1025.386853	2.480933915	880.9849853	7.935546507	792.9690413	11.75511542
1025.984983	2.483529719	882.6856053	7.948491638	794.1935763	11.75250942
1027.685603	2.490910412	882.9690413	7.95064584	794.7604503	11.75130031
1029.193573	2.497455222	884.1935763	7.95994169	795.3868533	11.74996231
1029.760453	2.499915588	884.7604503	7.964239201	795.9849853	11.74868352
1030.386853	2.502634375	885.3868533	7.968983065	797.6856053	11.74504811
1030.984983	2.505230496	885.9849853	7.97350869	797.9690413	11.74444128
1032.685603	2.512612047	887.6856053	7.98635257	799.1935763	11.74180585
1034.193573	2.519157613	887.9690413	7.988489842	799.7604503	11.7405772
1034.760453	2.521618237	889.1935763	7.997712373	800.3868533	11.73921378
1035.386853	2.524337267	889.7604503	8.00197562	800.9849853	11.73790703
1035.984983	2.526933575	890.3868533	8.006682022	802.6856053	11.73416936
1037.685603	2.534315362	890.9849853	8.01117155	802.9690413	11.73354348
1039.193573	2.54086071	892.6856053	8.023912486	804.1935763	11.73083048
1039.760453	2.543321139	892.9690413	8.026032525	804.7604503	11.7295697
1040.386853	2.54603989	894.1935763	8.035180404	805.3868533	11.72817305
1040.984983	2.548635869	894.7604503	8.039408961	805.9849853	11.72683601
1042.685603	2.556016374	895.3868533	8.044076966	807.6856053	11.72301577
1044.193573	2.562560193	895.9849853	8.048529724	807.9690413	11.72237627
1044.760453	2.565019955	897.6856053	8.061165505	809.1935763	11.7196038
1045.386853	2.567737907	897.9690413	8.063267953	809.7604503	11.71831496
1045.984983	2.570333063	899.1935763	8.072339507	810.3868533	11.71688681
1047.685603	2.577710929	899.7604503	8.076532572	810.9849853	11.71551914

Final State: j’=5		Final State: j’=4		Final State: j’=3	
E(cm⁻¹)	σ (Å²)	E(cm⁻¹)	σ (Å²)	E(cm⁻¹)	σ (Å²)
1049.193573	2.584252014	900.3868533	8.081161219	812.6856053	11.71160959
1049.760453	2.586710654	900.9849853	8.08557626	812.9690413	11.710955
1050.386853	2.589427302	902.6856053	8.09810409	814.1935763	11.70811705
1050.984983	2.592021149	902.9690413	8.100188415	814.7604503	11.70679791
1052.685603	2.599394972	904.1935763	8.109181321	815.3868533	11.70533626
1054.193573	2.605932057	904.7604503	8.113337744	815.9849853	11.70393669
1054.760453	2.608389092	905.3868533	8.11792568	817.6856053	11.69993669
1055.386853	2.611103901	905.9849853	8.122301697	817.9690413	11.69926703
1055.984983	2.613695932	907.6856053	8.134717272	819.1935763	11.696364
1057.685603	2.621064249	907.9690413	8.136782705	819.7604503	11.69501469
1059.193573	2.62759602	909.1935763	8.145693235	820.3868533	11.69351964
1059.760453	2.630050955	909.7604503	8.149811073	820.9849853	11.69208815
1060.386853	2.632763382	910.3868533	8.154356042	822.6856053	11.68799692
1060.984983	2.635353071	910.9849853	8.158690585	822.9690413	11.68731199
1062.685603	2.642714381	912.6856053	8.170985844	824.1935763	11.68434289
1064.193573	2.64923951	912.9690413	8.173030799	824.7604503	11.68296286
1064.760453	2.651691843	914.1935763	8.181851108	825.3868533	11.68143385
1065.386853	2.654401326	914.7604503	8.185926034	825.9849853	11.67996985
1065.984983	2.656988141	915.3868533	8.190422572	827.6856053	11.67578596
1067.685603	2.664340913	915.9849853	8.194709706	827.9690413	11.67508558
1069.193573	2.670858038	917.6856053	8.206861556	829.1935763	11.67204952
1069.760453	2.673307253	917.9690413	8.208880942	829.7604503	11.67063846
1070.386853	2.676013218	919.1935763	8.217582585	830.3868533	11.66907507
1070.984983	2.678596607	919.7604503	8.221596341	830.9849853	11.66757824
1072.685603	2.685939287	920.3868533	8.226018278	832.6856053	11.66330081
1074.193573	2.692447006	920.9849853	8.23022427	832.9690413	11.66258478
1074.760453	2.694892578	922.6856053	8.242020375	834.1935763	11.65948113
1075.386853	2.697594447	922.9690413	8.243934983	834.7604503	11.65803871
1075.984983	2.700173859	924.1935763	8.25172504	835.3868533	11.65644067
1077.685603	2.707504849	924.7604503	8.256415212	835.9849853	11.65491069
1079.193573	2.714001748	925.3868533	8.261806606	837.6856053	11.6505388
1079.760453	2.716443139	925.9849853	8.264862246	837.9690413	11.64980703
1080.386853	2.71914032	927.6856053	8.277595237	839.1935763	11.64663517
1080.984983	2.721715133	927.9690413	8.278928264	839.7604503	11.64516111
1084.193573	2.73551749	929.1935763	8.289387063	840.3868533	11.6435281
1084.760453	2.737954152	929.7604503	8.292770051	840.9849853	11.64196469
1085.386853	2.740646038	930.3868533	8.297271357	842.6856053	11.63749762
1085.984983	2.743215769	930.9849853	8.301146279	842.9690413	11.63674993
1089.193573	2.756989371	932.6856053	8.313875382	844.1935763	11.63350937
1089.760453	2.759420107	932.9690413	8.315066354	844.7604503	11.6320035
1090.386853	2.762106725	934.1935763	8.32416443	845.3868533	11.63033526
1090.984983	2.76467074	934.7604503	8.327541954	845.9849853	11.6287382
1094.193573	2.778412457	935.3868533	8.33091901	847.6856053	11.62417535

Final State: j’=5		Final State: j’=4		Final State: j’=3	
E(cm⁻¹)	σ (Å²)	E(cm⁻¹)	σ (Å²)	E(cm⁻¹)	σ (Å²)
1094.760453	2.780837991	935.9849853	8.335472185	847.9690413	11.62341169
1095.386853	2.783517429	937.6856053	8.349595277	849.1935763	11.62010203
1095.984983	2.78607513	937.9690413	8.350972695	849.7604503	11.61856411
1099.193573	2.799781745	939.1935763	8.358394438	850.3868533	11.61686049
1099.760453	2.802200865	939.7604503	8.36178057	850.9849853	11.6152296
1100.984983	2.807423914	940.3868533	8.364841992	852.6856053	11.61057048
1104.193573	2.821092175	940.9849853	8.37184415	852.9690413	11.60979078
1104.760453	2.823504292	942.6856053	8.383623059	854.1935763	11.6064117
1105.984983	2.828712008	942.9690413	8.385217894	854.7604503	11.60484165
1109.193573	2.842338611	944.1935763	8.39285215	855.3868533	11.60310248
1109.760453	2.844743146	944.7604503	8.396389756	855.9849853	11.60143765
1110.984983	2.849934253	945.3868533	8.400039197	857.6856053	11.59668196
1114.193573	2.863515863	945.9849853	8.403191981	857.9690413	11.59588614
1114.760453	2.865912227	947.6856053	8.417810512	859.1935763	11.59243745
1115.984983	2.871085448	947.9690413	8.419971549	859.7604503	11.59083518
1119.193573	2.884618693	949.1935763	8.427056009	860.3868533	11.58906041
1119.760453	2.887006287	949.7604503	8.430637718	860.9849853	11.58736155
1120.984983	2.892160329	950.3868533	8.434590378	862.6856053	11.58250908
1124.193573	2.905641805	950.9849853	8.438289053	862.9690413	11.58169714
1124.760453	2.908020023	952.6856053	8.451428353	864.1935763	11.57817876
1125.984983	2.913153576	952.9690413	8.45197778	864.7604503	11.57654423
1129.193573	2.926579823	954.1935763	8.461171253	865.3868533	11.57473378
1129.760453	2.928948049	954.7604503	8.464298934	865.9849853	11.57300089
1130.984983	2.934059783	955.3868533	8.468073483	867.6856053	11.56805161
1134.193573	2.947427282	955.9849853	8.471704302	867.9690413	11.56722352
1134.760453	2.949784889	957.6856053	8.481029043	869.1935763	11.56363546
1135.984983	2.954873438	957.9690413	8.482935403	869.7604503	11.56196867
1139.193573	2.96817856	959.1935763	8.491593132	870.3868533	11.5601226
1139.760453	2.9705249	959.7604503	8.497274939	870.9849853	11.55835569
1140.984983	2.975588852	960.3868533	8.502521122	872.6856053	11.55330971
1144.193573	2.988827796	960.9849853	8.504150679	872.9690413	11.5524655
1144.760453	2.991162182	962.6856053	8.516628429	874.1935763	11.54880789
1145.984983	2.996200018	962.9690413	8.517701612	874.7604503	11.5471089
1149.193573	3.009368628	964.1935763	8.528146378	875.3868533	11.54522726
1149.760453	3.01169028	964.7604503	8.531165584	875.9849853	11.54342639
1150.984983	3.016700286	965.3868533	8.533882662	877.6856053	11.53828398
1154.193573	3.02979362	965.9849853	8.535849418	877.9690413	11.53742371
1154.760453	3.032101581	967.6856053	8.552078968	879.1935763	11.53369672
1155.984983	3.037081549	967.9690413	8.553256585	879.7604503	11.53196565
1159.193573	3.050092517	969.1935763	8.558727216	880.3868533	11.53004856
1159.760453	3.052385242	969.7604503	8.559945037	880.9849853	11.52821386
1160.984983	3.057331354	970.3868533	8.568612368	882.6856053	11.52297536
1164.193573	3.070244389	970.9849853	8.572996681	882.9690413	11.52209911

Final State: j'=5		Final State: j'=4		Final State: j'=3	
E(cm⁻¹)	σ (Å²)	E(cm⁻¹)	σ (Å²)	E(cm⁻¹)	σ (Å²)
1165.984983	3.077417033	972.6856053	8.581147192	884.1935763	11.51830304
1169.193573	3.090108285	972.9690413	8.582501809	884.7604503	11.51653993
1170.984983	3.097227553	974.1935763	8.589922865	885.3868533	11.51458763
1174.193573	3.109891454	974.7604503	8.59761616	885.9849853	11.51271928
1175.984983	3.117467094	975.3868533	8.598921071	887.6856053	11.50738521
1179.193573	3.130058335	975.9849853	8.603394301	887.9690413	11.50649304
1180.984983	3.137223323	977.6856053	8.612559569	889.1935763	11.50262829
1184.193573	3.15022299	977.9690413	8.612951837	889.7604503	11.50083351
1185.984983	3.156746822	979.1935763	8.625839558	890.3868533	11.49884609
1189.193573	3.169702716	979.7604503	8.626045517	890.9849853	11.49694428
1190.984983	3.176342848	980.3868533	8.631201766	892.6856053	11.49151526
1194.193573	3.189309197	980.9849853	8.635167107	892.9690413	11.4906073
1195.984983	3.195771532	982.6856053	8.643203082	894.1935763	11.48667434
1199.193573	3.208192336	982.9690413	8.644906516	894.7604503	11.48484804
1200.984983	3.214943902	984.1935763	8.656787245	895.3868533	11.48282583
1204.193573	3.226292412	984.7604503	8.661437833	895.9849853	11.48089079
1205.984983	3.234256014	985.3868533	8.662425845	897.6856053	11.47536754
1209.193573	3.245843777	985.9849853	8.66390516	897.9690413	11.4744439
1210.984983	3.253251038	987.6856053	8.674957857	899.1935763	11.4704433
1214.193573	3.265745077	987.9690413	8.678432113	899.7604503	11.46858572
1215.984983	3.270619545	989.1935763	8.686036488	900.3868533	11.46652896
1219.193573	3.28323211	989.7604503	8.689136411	900.9849853	11.46456102
1220.984983	3.29096453	990.3868533	8.69265578	902.6856053	11.45894428
1224.193573	3.301197017	990.9849853	8.695893087	902.9690413	11.45800508
1225.984983	3.308191716	992.6856053	8.708157583	904.1935763	11.45393738
1229.193573	3.319499583	992.9690413	8.709901028	904.7604503	11.45204878
1230.984983	3.327872149	994.1935763	8.716683098	905.3868533	11.44995777
1234.193573	3.338257901	994.7604503	8.720031641	905.9849853	11.44795716
1235.984983	3.344784451	995.3868533	8.723754375	907.6856053	11.44224765
1239.193573	3.356520709	995.9849853	8.727320146	907.9690413	11.441293
1240.984983	3.362704296	997.6856053	8.737792527	909.1935763	11.43715861
1244.193573	3.374084559	997.9690413	8.739513102	909.7604503	11.43523915
1245.984983	3.380356971	999.1935763	8.746797594	910.3868533	11.43311404
1249.193573	3.391690932	999.7604503	8.750029014	910.9849853	11.43108087
1250.984983	3.397947356	1000.386853	8.754761796	912.6856053	11.42527878
1254.193573	3.409105628	1000.984983	8.758028848	912.9690413	11.42430868
1255.984983	3.415312002	1002.685603	8.768018833	914.1935763	11.42010741
1259.193573	3.426345661	1002.969043	8.769711318	914.7604503	11.41815691
1260.984983	3.432476078	1004.193573	8.777016917	915.3868533	11.41599738
1264.193573	3.443401507	1004.760453	8.780392166	915.9849853	11.41393122
1265.984983	3.449467766	1005.386853	8.784116142	917.6856053	11.40803414
1269.193573	3.460273367	1005.984983	8.78766608	917.9690413	11.40704796
1270.984983	3.46627353	1007.685603	8.7977515	919.1935763	11.40277592

Final State: j'=5		Final State: j'=4		Final State: j'=3	
E(cm⁻¹)	σ (Å²)	E(cm⁻¹)	σ (Å²)	E(cm⁻¹)	σ (Å²)
1275.984983	3.48289603	1007.969043	8.799454997	919.7604503	11.4007915
1280.984983	3.499333811	1009.193573	8.806754621	920.3868533	11.39859323
1285.984983	3.515586719	1009.760453	8.810079879	920.9849853	11.39648808
1290.984983	3.531654666	1010.386853	8.813752386	922.6856053	11.39045323
1295.984983	3.547537779	1010.984983	8.817257345	922.9690413	11.38943314
1300.984983	3.563236445	1012.685603	8.827203094	924.1935763	11.3848677
1305.984983	3.578751263	1012.969043	8.828857197	924.7604503	11.38299453
1310.984983	3.594083066	1014.193573	8.835991062	925.3868533	11.38103117
1315.984983	3.609232908	1014.760453	8.839287022	925.9849853	11.37853922
1320.984983	3.624202062	1015.386853	8.842924815	927.6856053	11.37271609
1325.984983	3.638992013	1015.984983	8.846394425	927.9690413	11.37148544
1330.984983	3.653604458	1017.685603	8.856232345	929.1935763	11.36746564
1335.984983	3.668041302	1017.969043	8.857867179	929.7604503	11.36528818
1340.984983	3.682304639	1019.193573	8.86491393	930.3868533	11.36325116
1345.984983	3.696396759	1019.760453	8.868167554	930.9849853	11.3610261
1350.984983	3.710320152	1020.386853	8.871756894	932.6856053	11.35491583
1355.984983	3.724077467	1020.984983	8.875178517	932.9690413	11.35358551
1360.984983	3.737671536	1022.685603	8.884877015	934.1935763	11.34951064
1365.984983	3.751105348	1022.969043	8.88648917	934.7604503	11.3473773
1370.984983	3.764382048	1024.193573	8.893440366	935.3868533	11.34496922
1375.984983	3.777504936	1024.760453	8.89665064	935.9849853	11.34289774
1380.984983	3.790477435	1025.386853	8.900192429	937.6856053	11.33676366
1385.984983	3.803303096	1025.984983	8.903568844	937.9690413	11.33579093
1390.984983	3.815985588	1027.685603	8.913138865	939.1935763	11.33117224
1395.984983	3.828528699	1029.193573	8.92158749	939.7604503	11.32903372
1400.984983	3.84093626	1029.760453	8.924754362	940.3868533	11.32656071
1405.984983	3.853212289	1030.386853	8.928248006	940.9849853	11.32428122
		1030.984983	8.931578308	942.6856053	11.31837412
		1032.685603	8.941016813	942.9690413	11.31728195
		1034.193573	8.949348723	944.1935763	11.31265081
		1034.760453	8.952471742	944.7604503	11.31051103
		1035.386853	8.955916966	945.3868533	11.30813491
		1035.984983	8.959201031	945.9849853	11.30584961
		1037.685603	8.968508122	947.6856053	11.29948937
		1039.193573	8.976723444	947.9690413	11.2986191
		1039.760453	8.979802618	949.1935763	11.29388537
		1040.386853	8.983199373	949.7604503	11.29172465
		1040.984983	8.986437134	950.3868533	11.289337
		1042.685603	8.995612428	950.9849853	11.28705458
		1044.193573	9.003710783	952.6856053	11.28037268
		1044.760453	9.006745964	952.9690413	11.27904316
		1045.386853	9.010094077	954.1935763	11.27488387
		1045.984983	9.013285383	954.7604503	11.27265477

Final State: j’=5		Final State: j’=4		Final State: j’=3	
E(cm⁻¹)	σ (Å²)	E(cm⁻¹)	σ (Å²)	E(cm⁻¹)	σ (Å²)
		1047.685603	9.022328558	955.3868533	11.27021578
		1049.193573	9.030309702	955.9849853	11.26788113
		1049.760453	9.033300812	957.6856053	11.2609585
		1050.386853	9.036600204	957.9690413	11.25963815
		1050.984983	9.03974499	959.1935763	11.2550319
		1052.685603	9.048655869	959.7604503	11.2528902
		1054.193573	9.056519663	960.3868533	11.25082111
		1054.760453	9.059466636	960.9849853	11.2481948
		1055.386853	9.062717259	962.6856053	11.2421252
		1055.984983	9.065815475	962.9690413	11.24089861
		1057.685603	9.074593929	964.1935763	11.23618707
		1059.193573	9.082340251	964.7604503	11.23425897
		1059.760453	9.085243074	965.3868533	11.23165788
		1060.386853	9.088444898	965.9849853	11.22913762
		1060.984983	9.091496515	967.6856053	11.22279414
		1062.685603	9.100142482	967.9690413	11.22159328
		1064.193573	9.107771348	969.1935763	11.21661343
		1064.760453	9.110630007	969.7604503	11.2138501
		1065.386853	9.113783044	970.3868533	11.21200679
		1065.984983	9.116788078	970.9849853	11.20987039
		1067.685603	9.125301603	972.6856053	11.20286696
		1069.193573	9.132813044	972.9690413	11.20170615
		1069.760453	9.135627571	974.1935763	11.19705843
		1070.386853	9.138731851	974.7604503	11.19437002
		1070.984983	9.141690324	975.3868533	11.19178133
		1072.685603	9.150071519	975.9849853	11.18950746
		1074.193573	9.157465691	977.6856053	11.18257033
		1074.760453	9.160236131	977.9690413	11.18156332
		1075.386853	9.163291737	979.1935763	11.17710093
		1075.984983	9.166203726	979.7604503	11.17434236
		1077.685603	9.174452812	980.3868533	11.17157318
		1079.193573	9.181729881	980.9849853	11.16983714
		1079.760453	9.184456357	982.6856053	11.16274069
		1080.386853	9.187463352	982.9690413	11.16077978
		1080.984983	9.190328915	984.1935763	11.15600935
		1084.193573	9.20560651	984.7604503	11.15472011
		1084.760453	9.20828909	985.3868533	11.15185418
		1085.386853	9.211247614	985.9849853	11.14905429
		1085.984983	9.214066955	987.6856053	11.14229709
		1089.193573	9.229096649	987.9690413	11.14089632
		1089.760453	9.231736082	989.1935763	11.13616633
		1090.386853	9.234645731	989.7604503	11.13384423
		1090.984983	9.237418968	990.3868533	11.131278

Final State: j’=5		Final State: j’=4		Final State: j’=3	
E(cm⁻¹)	σ (Å²)	E(cm⁻¹)	σ (Å²)	E(cm⁻¹)	σ (Å²)
		1094.193573	9.252201717	990.9849853	11.12884034
		1094.760453	9.254797022	992.6856053	11.12180026
		1095.386853	9.257659116	992.9690413	11.12063346
		1095.984983	9.260386436	994.1935763	11.11562706
		1099.193573	9.274923316	994.7604503	11.11329315
		1099.760453	9.277475244	995.3868533	11.11071035
		1100.984983	9.28297109	995.9849853	11.10823955
		1104.193573	9.297263318	997.6856053	11.10119575
		1104.760453	9.299772121	997.9690413	11.10002383
		1105.984983	9.305174882	999.1935763	11.094956
		1109.193573	9.31922386	999.7604503	11.09259021
		1109.760453	9.32168978	1000.386853	11.08990812
		1110.984983	9.327000035	1000.984983	11.08751106
		1114.193573	9.340807318	1002.685603	11.08042941
		1114.760453	9.343230658	1002.969043	11.07924772
		1115.984983	9.348449026	1004.193573	11.07413784
		1119.193573	9.362016345	1004.760453	11.07176966
		1119.760453	9.364397415	1005.386853	11.06915067
		1120.984983	9.3695246	1005.984983	11.06664759
		1124.193573	9.382853863	1007.685603	11.05951312
		1124.760453	9.385192997	1007.969043	11.05831897
		1125.984983	9.390229761	1009.193573	11.0531791
		1129.193573	9.403322995	1009.760453	11.05080016
		1129.760453	9.405620581	1010.386853	11.04816725
		1130.984983	9.41056773	1010.984983	11.04565032
		1134.193573	9.423427185	1012.685603	11.0384827
		1134.760453	9.425683617	1012.969043	11.0372866
		1135.984983	9.430542024	1014.193573	11.03211426
		1139.193573	9.443170087	1014.760453	11.0297172
		1139.760453	9.4453858	1015.386853	11.02706645
		1140.984983	9.450156459	1015.984983	11.02453355
		1144.193573	9.462555715	1017.685603	11.01732386
		1144.760453	9.464731199	1017.969043	11.01612117
		1145.984983	9.469415129	1019.193573	11.01092134
		1149.193573	9.481588462	1019.760453	11.00851201
		1149.760453	9.483724228	1020.386853	11.00584803
		1150.984983	9.488322611	1020.984983	11.00330267
		1154.193573	9.500273324	1022.685603	10.99605723
		1154.760453	9.502370016	1022.969043	10.99484848
		1155.984983	9.506884246	1024.193573	10.9896224
		1159.193573	9.518616542	1024.760453	10.98720104
		1159.760453	9.520675006	1025.386853	10.9845239
		1160.984983	9.525107124	1025.984983	10.98196615

Final State: j'=5		Final State: j'=4		Final State: j'=3	
E(cm⁻¹)	σ (Å²)	E(cm⁻¹)	σ (Å²)	E(cm⁻¹)	σ (Å²)
		1164.193573	9.536628411	1027.685603	10.97468645
		1165.984983	9.543005805	1029.193573	10.96822236
		1169.193573	9.554360134	1029.760453	10.96579026
		1170.984983	9.560573294	1030.386853	10.96310142
		1174.193573	9.571681032	1030.984983	10.9605326
		1175.984983	9.57765242	1032.685603	10.95322215
		1179.193573	9.588533239	1034.193573	10.94673159
		1180.984983	9.594584621	1034.760453	10.94428974
		1184.193573	9.605104238	1035.386853	10.94159022
		1185.984983	9.611181422	1035.984983	10.93901135
		1189.193573	9.621487455	1037.685603	10.93167296
		1190.984983	9.627389983	1039.193573	10.92515851
		1194.193573	9.637451527	1039.760453	10.92270788
		1195.984983	9.643295778	1040.386853	10.9199988
		1199.193573	9.653271099	1040.984983	10.91741097
		1200.984983	9.658938297	1042.685603	10.91004772
		1204.193573	9.668986024	1044.193573	10.9035121
		1205.984983	9.673992203	1044.760453	10.9010537
		1209.193573	9.684028255	1045.386853	10.89833618
		1210.984983	9.689124802	1045.984983	10.89574041
		1214.193573	9.698428273	1047.685603	10.88835528
		1215.984983	9.704253618	1049.193573	10.88180108
		1219.193573	9.713440037	1049.760453	10.87933591
		1220.984983	9.718102022	1050.386853	10.87661105
		1224.193573	9.72782292	1050.984983	10.87400837
		1225.984983	9.732771399	1052.685603	10.8666043
		1229.193573	9.741518629	1054.193573	10.86003414
		1230.984983	9.746214758	1054.760453	10.85756316
		1234.193573	9.755096052	1055.386853	10.85483201
		1235.984983	9.760141232	1055.984983	10.85222347
		1239.193573	9.768654247	1057.685603	10.84480337
		1240.984983	9.773485853	1059.193573	10.83821982
		1244.193573	9.781898188	1059.760453	10.83574401
		1245.984983	9.786502441	1060.386853	10.83300764
		1249.193573	9.794852898	1060.984983	10.83039423
		1250.984983	9.79942928	1062.685603	10.82296097
		1254.193573	9.807535327	1064.193573	10.81636653
		1255.984983	9.812035976	1064.760453	10.81388682
		1259.193573	9.820004718	1065.386853	10.81114628
		1260.984983	9.82440911	1065.984983	10.80852902
		1264.193573	9.832222717	1067.685603	10.80108538
		1265.984983	9.836544814	1069.193573	10.79448256
		1269.193573	9.844211629	1069.760453	10.79199989

Final State: j’=5		Final State: j’=4		Final State: j’=3	
E(cm⁻¹)	σ (Å²)	E(cm⁻¹)	σ (Å²)	E(cm⁻¹)	σ (Å²)
		1270.984983	9.848451042	1070.386853	10.78925619
		1275.984983	9.860131381	1070.984983	10.78663601
		1280.984983	9.87159115	1072.685603	10.77918479
		1285.984983	9.882834836	1074.193573	10.77257598
		1290.984983	9.893867061	1074.760453	10.77009128
		1295.984983	9.904692239	1075.386853	10.76734541
		1300.984983	9.915314634	1075.984983	10.7647233
		1305.984983	9.925738327	1077.685603	10.75726717
		1310.984983	9.935967239	1079.193573	10.75065476
		1315.984983	9.946005155	1079.760453	10.74816887
		1320.984983	9.955855652	1080.386853	10.74542183
		1325.984983	9.965522112	1080.984983	10.74279871
		1330.984983	9.975007735	1084.193573	10.72872658
		1335.984983	9.984315568	1084.760453	10.72624037
		1340.984983	9.993448447	1085.386853	10.72349311
		1345.984983	10.00240901	1085.984983	10.72086988
		1350.984983	10.01119975	1089.193573	10.70679898
		1355.984983	10.01982293	1089.760453	10.70431306
		1360.984983	10.02828064	1090.386853	10.70156674
		1365.984983	10.03657481	1090.984983	10.69894426
		1370.984983	10.04470718	1094.193573	10.68487921
		1375.984983	10.0526793	1094.760453	10.68239488
		1380.984983	10.06049261	1095.386853	10.67964989
		1385.984983	10.06814834	1095.984983	10.67702904
		1390.984983	10.07564755	1099.193573	10.66297439
		1395.984983	10.08299114	1099.760453	10.6604922
		1400.984983	10.09018012	1100.984983	10.65513122
		1405.984983	10.09721495	1104.193573	10.64109129
				1104.760453	10.63861197
				1105.984983	10.63325752
				1109.193573	10.61923644
				1109.760453	10.61676073
				1110.984983	10.61141434
				1114.193573	10.59741616
				1114.760453	10.59494474
				1115.984983	10.58960793
				1119.193573	10.57563646
				1119.760453	10.57317001
				1120.984983	10.5678442
				1124.193573	10.55390312
				1124.760453	10.55144226
				1125.984983	10.54612876
				1129.193573	10.53222151

Final State: j'=5		Final State: j'=4		Final State: j'=3	
E(cm⁻¹)	σ (Å²)	E(cm⁻¹)	σ (Å²)	E(cm⁻¹)	σ (Å²)
				1129.760453	10.52976687
				1130.984983	10.524467
				1134.193573	10.51059688
				1134.760453	10.50814901
				1135.984983	10.50286399
				1139.193573	10.4890341
				1139.760453	10.48659353
				1140.984983	10.48132449
				1144.193573	10.4675378
				1144.760453	10.46510506
				1145.984983	10.4598531
				1149.193573	10.44611239
				1149.760453	10.44368795
				1150.984983	10.43845411
				1154.193573	10.4247621
				1154.760453	10.42234644
				1155.984983	10.41713176
				1159.193573	10.40349129
				1159.760453	10.40108498
				1160.984983	10.39589072
				1164.193573	10.38230587
				1165.984983	10.3747387
				1169.193573	10.36123385
				1170.984983	10.3537084
				1174.193573	10.34025839
				1175.984983	10.33268165
				1179.193573	10.31927887
				1180.984983	10.31172656
				1184.193573	10.29832183
				1185.984983	10.29091684
				1189.193573	10.27759103
				1190.984983	10.27019946
				1194.193573	10.25692504
				1195.984983	10.24959118
				1199.193573	10.23636643
				1200.984983	10.22909707
				1204.193573	10.21617439
				1205.984983	10.20869881
				1209.193573	10.19562277
				1210.984983	10.18824371
				1214.193573	10.17523631
				1215.984983	10.1681796
				1219.193573	10.15514898

Final State: j’=5		Final State: j’=4		Final State: j’=3	
E(cm⁻¹)	σ (Å²)	E(cm⁻¹)	σ (Å²)	E(cm⁻¹)	σ (Å²)
				1220.984983	10.14798951
				1224.193573	10.13507153
				1225.984983	10.12801029
				1229.193573	10.11518499
				1230.984983	10.10792498
				1234.193573	10.09532509
				1235.984983	10.08818861
				1239.193573	10.07552958
				1240.984983	10.0684516
				1244.193573	10.05583074
				1245.984983	10.0487779
				1249.193573	10.03626341
				1250.984983	10.02927435
				1254.193573	10.01678753
				1255.984983	10.00983639
				1259.193573	9.997412227
				1260.984983	9.990492953
				1264.193573	9.978130894
				1265.984983	9.971245944
				1269.193573	9.958944479
				1270.984983	9.952093194
				1275.984983	9.933033921
				1280.984983	9.9140671
				1285.984983	9.895191759
				1290.984983	9.876406784
				1295.984983	9.857711289
				1300.984983	9.839104297
				1305.984983	9.820584918
				1310.984983	9.802152298
				1315.984983	9.783805664
				1320.984983	9.76554435
				1325.984983	9.747367768
				1330.984983	9.729275425
				1335.984983	9.711266959
				1340.984983	9.693342103
				1345.984983	9.675500726
				1350.984983	9.657742811
				1355.984983	9.640068528
				1360.984983	9.622478116
				1365.984983	9.604971998
				1370.984983	9.587550734
				1375.984983	9.570215046
				1380.984983	9.552965759

Final State: j’=5		Final State: j’=4		Final State: j’=3	
E(cm⁻¹)	σ (Å²)	E(cm⁻¹)	σ (Å²)	E(cm⁻¹)	σ (Å²)
				1385.984983	9.535803858
				1390.984983	9.518730507
				1395.984983	9.501746799
				1400.984983	9.48485454
				1405.984983	9.468054883

Final State: j'=5		Final State: j'=4		Final State: j'=3	
E(cm^{-1})	σ ($Å^2$)	E(cm^{-1})	σ ($Å^2$)	E(cm^{-1})	σ ($Å^2$)

	Initial rotational state: j=2		
**Final State: j’=1**		**Final State: j’=0**	
E(cm⁻¹)	σ (Å²)	E(cm⁻¹)	σ (Å²)
82.78560503	447.9157007	82.78560503	58.4613694
82.88560503	714.148397	82.88560503	90.5619387
82.96904163	370.319026	82.96904163	46.7923285
82.98560503	355.6584056	82.98560503	44.7655807
83.08560503	306.3468825	83.08560503	37.7823077
83.18560503	276.3547599	83.18560503	33.6800098
83.28560503	314.6571706	83.28560503	38.9293575
83.38560503	288.0535731	83.38560503	36.8253708
83.48560503	253.9278836	83.48560503	32.1538318
83.58560503	243.3139743	83.58560503	30.11752117
87.68560503	94.47325766	87.68560503	11.0313681
87.88560503	92.00559576	87.78560503	10.89076799
87.96904163	90.78620709	87.88560503	10.7400857
87.98560503	90.52829309	87.96904163	10.60589547
88.18560503	86.89382748	87.98560503	10.57831878
92.68560503	63.98771132	88.08560503	10.40568886
92.96904163	63.62599172	88.18560503	10.22669813
97.68560503	64.52025608	92.68560503	7.45552716
97.96904163	59.36502616	92.96904163	7.39822775
100.3868533	46.12802109	97.68560503	7.40179081
102.6856053	41.06757087	97.96904163	6.63844202
102.9690413	40.26183591	100.3868533	5.84695406
105.3868533	41.8048233	102.6856053	6.76974735
107.9690413	34.97665825	102.9690413	7.41804422
112.6856053	31.62850288	105.3868533	8.00045478
112.9690413	31.7924277	107.6856053	7.89427425
115.3868533	30.19696901	107.9690413	7.87575941
117.9690413	29.73998987	110.3868533	5.95402128
120.3868533	27.79769333	112.6856053	5.92916822
122.9690413	27.51395747	112.9690413	5.96182285
127.6856053	25.98508818	115.3868533	6.18811608
127.9690413	25.8664764	117.6856053	4.32170828
130.3868533	24.54209316	117.9690413	4.31553077
132.9690413	23.09746163	120.3868533	5.18065205
135.3868533	23.49706839	122.6856053	3.9015817
137.9690413	21.25915944	122.9690413	3.90154534
142.9690413	20.94481865	125.3868533	6.28849872
147.9690413	21.28185184	127.6856053	3.69293471
152.6856053	19.6112246	127.9690413	3.64382422
152.9690413	19.05313912	130.3868533	3.99625801
155.3868533	20.00841767	132.6856053	4.86185123
157.9690413	18.38482801	132.9690413	5.17647423

Final State: j'=1		Final State: j'=0	
E(cm⁻¹)	σ (Å²)	E(cm⁻¹)	σ (Å²)
162.6856053	18.43390516	135.3868533	3.63372229
162.9690413	18.43462037	137.6856053	5.07097451
164.9604503	18.0403874	137.9690413	5.90634895
165.0604503	18.41475432	140.3868533	4.79204627
165.1604503	18.22762972	142.6856053	5.0639682
165.2604503	17.94039036	142.9690413	4.99575869
165.3604503	17.22657184	145.3868533	6.20761117
165.3868533	16.98292732	147.6856053	3.39053961
165.4604503	17.1953093	147.9690413	3.42682094
165.5604503	17.88958424	150.3868533	3.41885321
165.6604503	17.68777231	152.6856053	4.50814177
165.7604503	17.18504143	152.9690413	5.49618268
165.8604503	17.90643592	155.3868533	3.90727447
165.9604503	17.96130555	157.6856053	6.60427191
167.6856053	17.14328395	157.9690413	5.72434362
167.9690413	17.12775714	160.3868533	3.40661095
169.7604503	16.7483592	162.6856053	4.96318735
169.8604503	16.70277398	162.9690413	5.07157116
169.9604503	16.65409484	164.8604503	3.28706456
170.0604503	16.59961105	164.9604503	3.7101728
170.1604503	16.53431216	165.0604503	3.43791108
170.2604503	16.44835049	165.1604503	3.57193982
170.3604503	16.3205489	165.2604503	3.88928964
170.3868533	16.2748504	165.3604503	4.86311422
170.4604503	16.10648428	165.3868533	5.1549041
172.6856053	16.42101418	165.4604503	4.71199382
172.9690413	16.39913275	165.5604503	3.80115391
174.7604503	16.13741352	165.6604503	4.00818126
175.3868533	15.9764505	165.7604503	4.61322012
177.6856053	15.66141564	165.8604503	3.68455053
177.9690413	15.64357285	165.9604503	3.53876371
179.7604503	15.16693248	167.6856053	3.6760058
180.3868533	15.54338709	167.9690413	3.60580166
182.6856053	15.3050486	169.7604503	3.74708732
182.9690413	15.28254729	169.8604503	3.78015487
184.7604503	15.14454708	169.9604503	3.82193862
185.3868533	15.09325941	170.0604503	3.87791697
187.6856053	14.78751545	170.1604503	3.95854939
187.9690413	14.79338571	170.2604503	4.0859028
189.7604503	14.77918936	170.3604503	4.31232362
190.3868533	14.74816445	170.3868533	4.40222399
192.6856053	14.61814059	170.4604503	4.76813556
192.9690413	14.60130186	172.6856053	3.59530411

Final State: j’=1		Final State: j’=0	
E(cm⁻¹)	σ (Å²)	E(cm⁻¹)	σ (Å²)
194.7604503	14.49018621	172.9690413	3.59457859
195.3868533	14.4505893	174.7604503	3.651374
197.6856053	14.32439248	175.3868533	3.69362864
197.9690413	14.31051887	177.6856053	3.73297966
199.7604503	14.22467851	177.9690413	3.74110629
200.3868533	14.19497559	179.7604503	3.84676669
202.6856053	14.08721312	180.3868533	3.66225754
202.9690413	14.07412584	182.6856053	3.7034268
204.7604503	13.99324575	182.9690413	3.70570314
205.3868533	13.96599407	184.7604503	3.72018589
207.6856053	13.87281872	185.3868533	3.72728044
207.9690413	13.8622682	187.6856053	3.76261329
209.7604503	13.80299011	187.9690413	3.74984502
210.3868533	13.78693314	189.7604503	3.72863847
212.6856053	13.74097758	190.3868533	3.72993429
212.9690413	13.6906472	192.6856053	3.73821805
214.7604503	13.53540817	192.9690413	3.73947367
215.3868533	13.44621967	194.7604503	3.74814245
217.6856053	13.35271605	195.3868533	3.75063244
217.9690413	13.28142663	197.6856053	3.75374547
219.7604503	13.34181529	197.9690413	3.75397435
220.3868533	13.26244514	199.7604503	3.75630646
222.6856053	13.11182674	200.3868533	3.75740964
222.9690413	13.14149778	202.6856053	3.76188123
224.7604503	12.97450906	202.9690413	3.762415
225.3868533	13.04790545	204.7604503	3.7652193
227.6856053	12.98940052	205.3868533	3.76584563
227.9690413	13.00102396	207.6856053	3.76552065
229.7604503	12.76286861	207.9690413	3.76506945
230.3868533	12.92119832	209.7604503	3.75794613
232.6856053	12.80191778	210.3868533	3.75216612
232.9690413	12.80873712	212.6856053	3.63835353
234.7604503	12.73637173	212.9690413	3.63345524
235.3868533	12.58461112	214.7604503	3.68734377
237.6856053	12.60998457	215.3868533	3.76789861
237.9690413	12.61646559	217.6856053	3.71486329
239.7604503	12.64938475	217.9690413	3.76751923
240.3868533	12.66862039	219.7604503	3.76093229
242.6856053	12.35208873	220.3868533	3.70070705
242.9690413	12.41758406	222.6856053	3.85544361
244.7604503	12.50761662	222.9690413	3.84650077
245.3868533	12.53737048	224.7604503	3.78327513
247.6856053	12.38869554	225.3868533	3.83059918

Final State: j’=1		Final State: j’=0	
E(cm⁻¹)	σ (Å²)	E(cm⁻¹)	σ (Å²)
247.9690413	12.28447698	227.6856053	3.86995137
249.7604503	12.26726994	227.9690413	3.85643842
250.3868533	12.19792471	229.7604503	3.84604994
252.6856053	12.12550152	230.3868533	3.80649286
252.9690413	12.05979054	232.6856053	3.91017207
254.7604503	12.33740742	232.9690413	3.90190217
255.3868533	11.99163923	234.7604503	3.75658813
257.6856053	12.07272024	235.3868533	3.89554785
257.9690413	12.11671676	237.6856053	3.9552229
259.7604503	11.86564472	237.9690413	3.94960133
260.3868533	11.94133062	239.7604503	3.90037322
262.6856053	11.95990137	240.3868533	3.84092858
262.9690413	11.82100255	242.6856053	3.99805018
264.7604503	11.85153125	242.9690413	3.99001816
265.3868533	11.87533924	244.7604503	3.92998999
267.6856053	11.55723572	245.3868533	3.89608329
267.9690413	11.68332111	247.6856053	3.70333222
269.7604503	11.7574825	247.9690413	3.77025256
270.3868533	11.93034984	249.7604503	3.85214787
272.6856053	11.44048943	250.3868533	3.9063389
272.9690413	11.55406586	252.6856053	3.88381008
274.2935763	11.67213785	252.9690413	3.97499702
274.3935763	11.67512577	254.7604503	3.77181736
274.4935763	11.68309116	255.3868533	3.75364127
274.5935763	11.71518751	257.6856053	4.02520204
274.6935763	11.75387007	257.9690413	3.9818706
274.7604503	11.78118401	259.7604503	4.10141918
274.7935763	11.76477738	260.3868533	4.08020607
274.8935763	11.54776456	262.6856053	3.78821944
274.9935763	11.5398466	262.9690413	3.83545746
275.0935763	11.62187435	264.7604503	4.03209784
275.1935763	11.55102077	265.3868533	4.01407401
275.2935763	11.50750159	267.6856053	4.14468612
275.3868533	11.55152443	267.9690413	4.13633215
275.3935763	11.55384858	269.7604503	4.10138419
275.4935763	11.58110044	270.3868533	3.92473967
275.5935763	11.60054065	272.6856053	3.89639201
277.6856053	11.47479668	272.9690413	3.9827775
277.9690413	11.48750816	274.2935763	4.09113159
279.1935763	11.50556837	274.3935763	4.0037038
279.2935763	11.50627561	274.4935763	4.05621616
279.3935763	11.50733923	274.5935763	4.0251591
279.4935763	11.5088732	274.6935763	3.97424683

Final State: j’=1		Final State: j’=0	
E(cm⁻¹)	σ (Å²)	E(cm⁻¹)	σ (Å²)
279.5935763	11.5109241	274.7604503	3.90127729
279.6935763	11.51324091	274.7935763	3.84996243
279.7604503	11.514298	274.8935763	3.89107284
279.7935763	11.51422847	274.9935763	4.03936306
279.8935763	11.5051587	275.0935763	4.00246282
279.9935763	11.4500707	275.1935763	3.92971821
280.0935763	11.36121495	275.2935763	4.06923189
280.3868533	11.46180164	275.3868533	4.07197416
282.6856053	11.35403175	275.3935763	4.07151684
282.9690413	11.3530544	275.4935763	4.06249333
284.1935763	11.33902755	275.5935763	4.05196381
284.7604503	11.32746643	277.6856053	4.05338349
285.3868533	11.3042423	279.1935763	4.01216306
287.6856053	11.25073839	279.2935763	4.00808264
287.9690413	11.24782127	279.3935763	4.00346581
289.1935763	11.1392817	279.4935763	3.99796433
289.7604503	11.17514742	279.5935763	3.99097141
290.3868533	11.17230964	279.6935763	3.98135639
292.6856053	11.12569454	279.7604503	3.97238914
292.9690413	11.11971871	279.7935763	3.96677454
294.1935763	11.09388423	279.8935763	3.94264842
294.7604503	11.08184864	279.9935763	3.91561151
295.3868533	11.06824101	280.0935763	3.93900637
297.6856053	11.0040024	280.3868533	3.93344823
297.9690413	10.99975656	282.6856053	4.05031714
299.1935763	10.97854742	282.9690413	4.04839878
299.7604503	10.96759304	284.1935763	4.0408893
300.3868533	10.95515625	284.7604503	4.03517832
302.6856053	10.90835764	285.3868533	4.02850426
302.9690413	10.90249369	287.6856053	4.03915778
304.1935763	10.87676584	287.9690413	4.03643659
304.7604503	10.86465599	289.1935763	4.04788002
305.3868533	10.851292	289.7604503	4.06724679
307.6856053	10.80412901	290.3868533	4.06314492
307.9690413	10.79847518	292.6856053	4.06696777
309.1935763	10.77420703	292.9690413	4.06785239
309.7604503	10.7630276	294.7604503	4.07312949
310.3868533	10.75070043	295.3868533	4.07441366
312.6856053	10.70562612	297.6856053	4.08402192
312.9690413	10.70008462	297.9690413	4.08621076
314.1935763	10.67620025	299.1935763	4.09241225
314.7604503	10.66518442	299.7604503	4.09476244
317.6856053	10.608929	300.3868533	4.09728315

Final State: j’=1		Final State: j’=0	
E(cm⁻¹)	σ (Å²)	E(cm⁻¹)	σ (Å²)
317.9690413	10.60353483	302.6856053	4.10625677
319.1935763	10.58034102	302.9690413	4.10733355
319.7604503	10.5696621	304.1935763	4.11195276
320.3868533	10.55790225	304.7604503	4.11414742
322.6856053	10.51508863	305.3868533	4.11668794
322.9690413	10.50984546	307.6856053	4.12665757
324.1935763	10.48728122	307.9690413	4.12788798
324.7604503	10.47688391	309.1935763	4.13314427
325.3868533	10.46543055	309.7604503	4.13554912
327.6856053	10.42372412	310.3868533	4.13819038
327.9690413	10.41861735	312.6856053	4.14779481
329.7604503	10.38652328	312.9690413	4.14897508
330.3868533	10.37537515	314.1935763	4.15408089
332.6856053	10.33479384	314.7604503	4.15645157
332.9690413	10.32982602	315.3868533	4.15907789
334.1935763	10.30845415	317.6856053	4.16876785
334.7604503	10.29861055	317.9690413	4.1699666
335.3868533	10.28777044	319.1935763	4.17515089
337.6856053	10.24833058	319.7604503	4.177553
337.9690413	10.24350571	320.3868533	4.18020861
339.1935763	10.22276086	322.6856053	4.18996619
339.7604503	10.21321448	322.9690413	4.19117102
340.3868533	10.20270955	324.1935763	4.19638222
342.6856053	10.16459774	324.7604503	4.19879857
342.9690413	10.15995276	325.3868533	4.20147205
344.1935763	10.14005573	327.6856053	4.21131914
344.7604503	10.13095681	327.9690413	4.21253779
345.3868533	10.12100934	329.1935763	4.21781557
347.6856053	10.08647259	329.7604503	4.22026643
347.9690413	10.08273973	330.3868533	4.22298085
349.1935763	10.07627786	332.6856053	4.23300545
349.7604503	10.08262429	332.9690413	4.23424936
350.3868533	10.068244	334.1935763	4.23964703
352.6856053	10.00800417	334.7604503	4.24216009
352.9690413	10.00484809	335.3868533	4.24494902
354.1935763	10.01343083	337.6856053	4.25531398
354.7604503	9.994226644	337.9690413	4.25660908
355.3868533	9.968437507	339.1935763	4.2622589
357.6856053	9.946148866	339.7604503	4.26490902
357.9690413	9.928445111	340.3868533	4.26786779
359.1935763	9.908954597	342.6856053	4.2790943
359.7604503	9.892815331	342.9690413	4.28053233
360.3868533	9.889760961	344.1935763	4.28694686

Final State: j’=1		Final State: j’=0	
E(cm⁻¹)	σ (Å²)	E(cm⁻¹)	σ (Å²)
362.6856053	9.892306767	344.7604503	4.29006079
362.9690413	9.857053316	345.3868533	4.29365078
364.1935763	9.821584635	347.6856053	4.30961271
364.7604503	9.817276738	347.9690413	4.31224966
365.3868533	9.819169998	349.1935763	4.32919017
367.6856053	9.7848327	349.7604503	4.32614872
367.9690413	9.758687293	350.3868533	4.30624647
369.1935763	9.742984809	352.6856053	4.31703145
369.7604503	9.737708198	352.9690413	4.32469043
370.3868533	9.734123186	354.1935763	4.32244736
372.6856053	9.731777017	354.7604503	4.31837405
372.9690413	9.703581903	355.3868533	4.32417146
374.1935763	9.663904232	357.6856053	4.31923538
374.7604503	9.658159328	357.9690413	4.32411208
375.3868533	9.651896021	359.1935763	4.31885502
377.6856053	9.672778174	359.7604503	4.33611457
377.9690413	9.640337545	360.3868533	4.35067144
379.1935763	9.602799202	362.6856053	4.35100281
379.7604503	9.578759191	362.9690413	4.3249687
380.3868533	9.574262237	364.1935763	4.35335202
382.6856053	9.564202226	364.7604503	4.36305326
382.9690413	9.572348019	365.3868533	4.3763447
384.1935763	9.577582715	367.6856053	4.34187875
384.7604503	9.577876769	367.9690413	4.3449949
385.3868533	9.553592155	369.1935763	4.3725373
387.6856053	9.48476163	369.7604503	4.38022522
387.9690413	9.47948657	370.3868533	4.38955108
389.1935763	9.51118026	372.6856053	4.39098293
389.7604503	9.477951304	372.9690413	4.36620613
390.3868533	9.450677171	374.1935763	4.38835494
392.6856053	9.437939041	374.7604503	4.39605756
392.9690413	9.453451595	375.3868533	4.4033986
394.1935763	9.382975847	377.6856053	4.42851647
394.7604503	9.388354242	377.9690413	4.40403692
395.3868533	9.432195293	379.1935763	4.40141637
397.6856053	9.343192796	379.7604503	4.41097287
397.9690413	9.34248437	380.3868533	4.42436136
399.1935763	9.388203348	382.6856053	4.46707276
399.7604503	9.384536833	382.9690413	4.47156682
400.3868533	9.309481143	384.1935763	4.46726452
402.6856053	9.303621074	384.7604503	4.44526782
402.9690413	9.302837553	385.3868533	4.45504265
404.1935763	9.312715093	387.6856053	4.43245782

Final State: j'=1		Final State: j'=0	
E(cm⁻¹)	σ (Å²)	E(cm⁻¹)	σ (Å²)
404.7604503	9.243523978	387.9690413	4.44586271
405.3868533	9.274410292	389.1935763	4.46684967
407.6856053	9.274646097	389.7604503	4.42505837
407.9690413	9.257807271	390.3868533	4.45167415
409.1935763	9.191851145	392.6856053	4.4218918
409.7604503	9.212751933	392.9690413	4.4180424
410.3868533	9.171039416	394.1935763	4.46665274
411.0849853	9.16985076	394.7604503	4.48904769
411.1849853	9.188859034	395.3868533	4.50802343
411.2849853	9.176061738	397.9690413	4.49343125
411.3849853	9.179945702	399.1935763	4.53966934
411.4849853	9.188768896	399.7604503	4.48417107
411.5849853	9.219092471	400.3868533	4.47442042
411.6849853	9.219350715	402.6856053	4.53167135
411.7849853	9.185988173	402.9690413	4.52594532
411.8849853	9.182601824	404.1935763	4.4790025
411.9849853	9.210417133	404.7604503	4.48577095
412.0849853	9.175057572	405.3868533	4.54894018
412.1849853	9.169274072	407.6856053	4.5365546
412.2849853	9.168961863	407.9690413	4.54088518
412.3849853	9.169709531	409.1935763	4.52118548
412.4849853	9.170948764	409.7604503	4.51542904
412.5849853	9.172691801	410.3868533	4.49466359
412.6856053	9.175198558	411.0849853	4.54400906
412.9690413	9.193633939	411.1849853	4.55381511
414.1935763	9.144919564	411.2849853	4.54895763
414.7604503	9.141075027	411.3849853	4.55781859
415.3868533	9.139142686	411.4849853	4.56953136
415.9849853	9.139065208	411.5849853	4.58241077
416.0849853	9.139374113	411.6849853	4.54616012
416.1849853	9.139902989	411.7849853	4.52151794
416.2849853	9.140771502	411.8849853	4.54306986
416.3849853	9.142181871	411.9849853	4.54452069
416.4849853	9.144504833	412.0849853	4.5197885
416.5849853	9.148482591	412.1849853	4.530056
416.6849853	9.155381696	412.2849853	4.53680212
416.7849853	9.158151741	412.3849853	4.54185788
416.8849853	9.145487556	412.4849853	4.54618835
416.9849853	9.120586837	412.5849853	4.55031452
417.0849853	9.131551288	412.6856053	4.55464975
417.6856053	9.112584797	412.9690413	4.57203569
417.9690413	9.102429994	414.1935763	4.53530273
419.1935763	9.087012059	414.7604503	4.54411924

Final State: j’=1		Final State: j’=0	
E(cm⁻¹)	σ (Å²)	E(cm⁻¹)	σ (Å²)
419.7604503	9.082856269	415.3868533	4.55193542
420.3868533	9.07768611	415.9849853	4.55791553
420.9849853	9.072752451	416.0849853	4.55892116
422.6856053	9.056882538	416.1849853	4.55999497
422.9690413	9.052942263	416.2849853	4.56117828
424.1935763	9.038870835	416.3849853	4.56253161
424.7604503	9.03494147	416.4849853	4.56414204
425.3868533	9.034937636	416.5849853	4.56612354
425.9849853	9.009264053	416.6849853	4.56825802
427.6856053	8.993370275	416.7849853	4.56448998
427.9690413	8.990433612	416.8849853	4.55847256
429.1935763	8.977085879	416.9849853	4.55748074
429.7604503	8.970793502	417.0849853	4.56563704
430.3868533	8.9638461	417.6856053	4.54953565
430.9849853	8.95724835	417.9690413	4.54429294
432.6856053	8.939041477	419.1935763	4.55812919
432.9690413	8.936058868	419.7604503	4.56217106
434.1935763	8.919696316	420.3868533	4.56580862
434.7604503	8.912712762	420.9849853	4.56899119
435.3868533	8.905647787	422.6856053	4.57420379
435.9849853	8.899012119	422.9690413	4.57470661
437.6856053	8.88015143	424.1935763	4.57927251
437.9690413	8.877008612	424.7604503	4.58216809
439.1935763	8.863451917	425.3868533	4.58209433
439.7604503	8.857187582	425.9849853	4.57727653
440.3868533	8.850263642	427.6856053	4.58934217
440.9849853	8.843632994	427.9690413	4.59038634
442.6856053	8.824605353	429.1935763	4.59465565
442.9690413	8.821428324	429.7604503	4.59659319
444.1935763	8.80778155	430.3868533	4.59872738
444.7604503	8.80151188	430.9849853	4.60076148
445.3868533	8.794613645	432.6856053	4.60649641
445.9849853	8.788051454	432.9690413	4.60737061
447.6856053	8.769496724	434.1935763	4.61020569
447.9690413	8.766416525	434.7604503	4.61208436
449.1935763	8.75314133	435.3868533	4.61427134
449.7604503	8.747011468	435.9849853	4.61631121
450.3868533	8.740248153	437.6856053	4.62192731
450.9849853	8.733799872	437.9690413	4.62284861
452.6856053	8.715522429	439.1935763	4.62679886
452.9690413	8.712485114	439.7604503	4.62861114
454.1935763	8.699395984	440.3868533	4.63059857
454.7604503	8.693355494	440.9849853	4.63247792

Final State: j’=1		Final State: j’=0	
E(cm⁻¹)	σ (Å²)	E(cm⁻¹)	σ (Å²)
455.3868533	8.686694849	442.6856053	4.63774399
455.9849853	8.680348752	442.9690413	4.63861995
457.6856053	8.662378903	444.1935763	4.64241853
457.9690413	8.659394279	444.7604503	4.64418149
459.1935763	8.646532945	445.3868533	4.6461293
459.7604503	8.640596894	445.9849853	4.64798708
460.3868533	8.634050445	447.6856053	4.65324896
460.9849853	8.627812063	447.9690413	4.65412241
462.6856053	8.610141048	449.1935763	4.65788298
462.9690413	8.607205384	449.7604503	4.65961646
464.1935763	8.594553322	450.3868533	4.66152656
464.7604503	8.588713384	450.9849853	4.66334531
465.3868533	8.582272672	452.6856053	4.66849125
465.9849853	8.576134963	452.9690413	4.66934557
467.6856053	8.558749479	454.1935763	4.67302638
467.9690413	8.555861276	454.7604503	4.67472487
469.1935763	8.543413946	455.3868533	4.6765977
469.7604503	8.537668496	455.9849853	4.67838204
470.3868533	8.531331963	457.6856053	4.68343362
470.9849853	8.525293365	457.9690413	4.68427237
472.6856053	8.508187852	459.1935763	4.68788539
472.9690413	8.505345999	459.7604503	4.68955206
474.1935763	8.493097916	460.3868533	4.69138934
474.7604503	8.487444063	460.9849853	4.69313946
475.3868533	8.48120835	462.6856053	4.69809205
475.9849853	8.475265615	462.9690413	4.69891416
477.6856053	8.458430476	464.1935763	4.70245512
477.9690413	8.455633361	464.7604503	4.70408834
479.1935763	8.443577583	465.3868533	4.70588869
479.7604503	8.438012242	465.9849853	4.70760347
480.3868533	8.431873888	467.6856053	4.71245603
480.9849853	8.426023726	467.9690413	4.71326148
482.6856053	8.409449503	469.1935763	4.7167304
482.9690413	8.406695591	469.7604503	4.71833029
484.1935763	8.394825223	470.3868533	4.72009375
484.7604503	8.389345109	470.9849853	4.72177329
485.3868533	8.38330043	472.6856053	4.72652545
485.9849853	8.377539263	472.9690413	4.72731411
487.6856053	8.361215361	474.1935763	4.73071048
487.9690413	8.358502719	474.7604503	4.7322767
489.1935763	8.346809454	475.3868533	4.73400292
489.7604503	8.341410477	475.9849853	4.73564687
490.3868533	8.335454875	477.6856053	4.74029755

Final State: j'=1		Final State: j'=0	
E(cm⁻¹)	σ (Å²)	E(cm⁻¹)	σ (Å²)
490.9849853	8.329778126	477.9690413	4.74106929
492.6856053	8.313690695	479.1935763	4.74439233
492.9690413	8.311016899	479.7604503	4.74592455
494.1935763	8.299489509	480.3868533	4.74761317
494.7604503	8.294166192	480.9849853	4.74922114
495.3868533	8.288293227	482.6856053	4.75376932
495.9849853	8.282694398	482.9690413	4.75452394
497.6856053	8.266822746	484.1935763	4.75777285
497.9690413	8.264184035	484.7604503	4.75927068
499.1935763	8.252804376	485.3868533	4.76092123
499.7604503	8.247547303	485.9849853	4.76249277
500.3868533	8.241745726	487.6856053	4.76693702
500.9849853	8.23621313	487.9690413	4.76767426
502.6856053	8.220516987	489.1935763	4.77084787
502.9690413	8.21790535	489.7604503	4.7723107
504.1935763	8.206633673	490.3868533	4.77392248
504.7604503	8.201420607	490.9849853	4.7754569
505.3868533	8.195662293	492.6856053	4.77979492
505.9849853	8.190164643	492.9690413	4.78051437
507.6856053	8.174520952	494.1935763	4.78361068
507.9690413	8.1719088	494.7604503	4.78503751
509.1935763	8.160587432	495.3868533	4.7866093
509.7604503	8.155314252	495.9849853	4.78810538
510.3868533	8.149446727	497.6856053	4.79233307
510.9849853	8.143781588	497.9690413	4.79303391
512.6856053	8.126736148	499.1935763	4.79604913
512.9690413	8.123489031	499.7604503	4.79743791
514.1935763	8.111471526	500.3868533	4.79896726
514.7604503	8.112249961	500.9849853	4.80042234
515.3868533	8.10365505	502.6856053	4.80453054
515.9849853	8.099935254	502.9690413	4.80521094
517.6856053	8.080537397	504.1935763	4.80813577
517.9690413	8.07908818	504.7604503	4.80948135
519.1935763	8.074768669	505.3868533	4.81096168
519.7604503	8.066763408	505.9849853	4.81236848
520.3868533	8.064437686	507.6856053	4.81632903
520.9849853	8.055003017	507.9690413	4.81698285
522.6856053	8.041372787	509.1935763	4.81978333
522.9690413	8.04187764	509.7604503	4.8210645
524.1935763	8.031520825	510.3868533	4.82246674
524.7604503	8.0237671	510.9849853	4.82379037
525.3868533	8.016958592	512.6856053	4.82749557
525.9849853	8.019473527	512.9690413	4.82817617

Final State: j'=1		Final State: j'=0	
E(cm⁻¹)	σ (Å²)	E(cm⁻¹)	σ (Å²)
527.6856053	8.006097287	514.1935763	4.8373751
527.9690413	8.002390248	514.7604503	4.83769966
529.1935763	7.987809688	515.3868533	4.83635092
529.7604503	7.979669745	515.9849853	4.8427236
530.3868533	7.982471395	517.6856053	4.84234177
530.9849853	7.976695929	517.9690413	4.84533289
532.6856053	7.96420785	519.1935763	4.84870415
532.9690413	7.960899411	519.7604503	4.84778488
534.1935763	7.947574104	520.3868533	4.84835403
534.7604503	7.94093552	520.9849853	4.84881215
535.3868533	7.931852612	522.6856053	4.85386762
535.9849853	7.93753829	522.9690413	4.86276966
537.6856053	7.925962974	524.1935763	4.8555104
537.9690413	7.921760445	524.7604503	4.8565681
539.1935763	7.908309258	525.3868533	4.86424547
539.7604503	7.902545218	525.9849853	4.86364336
540.3868533	7.896017491	527.6856053	4.86403838
540.9849853	7.889161401	527.9690413	4.86406913
542.6856053	7.879586508	529.1935763	4.86582783
542.9690413	7.881392502	529.7604503	4.86776362
544.1935763	7.869433959	530.3868533	4.87724222
544.7604503	7.862891588	530.9849853	4.87208125
545.3868533	7.856150854	532.6856053	4.87395492
545.9849853	7.849618679	532.9690413	4.87423979
547.6856053	7.829097185	534.1935763	4.87615397
547.9690413	7.829539218	534.7604503	4.87728259
549.1935763	7.829238221	535.3868533	4.88056993
549.7604503	7.831165498	535.9849853	4.88715744
550.3868533	7.820603861	537.6856053	4.88467734
550.9849853	7.811086601	537.9690413	4.88449419
552.6856053	7.795806031	539.1935763	4.88608267
552.9690413	7.789476113	539.7604503	4.88702865
554.1935763	7.793292277	540.3868533	4.88813298
554.7604503	7.783863034	540.9849853	4.88933868
555.3868533	7.772947428	542.6856053	4.89551829
555.9849853	7.761934168	542.9690413	4.90306632
557.6856053	7.773010795	544.1935763	4.89550837
557.9690413	7.765982015	544.7604503	4.89614711
559.1935763	7.744261943	545.3868533	4.89699376
559.7604503	7.74188956	545.9849853	4.89782385
560.3868533	7.754527409	547.6856053	4.90834032
560.9849853	7.742455127	547.9690413	4.90939979
562.6856053	7.719228601	549.1935763	4.91612738

Final State: j'=1		Final State: j'=0	
E(cm^{-1})	σ (Å^2)	E(cm^{-1})	σ (Å^2)
562.9690413	7.714150191	549.7604503	4.91781677
564.1935763	7.731326426	550.3868533	4.9108381
564.7604503	7.709545091	550.9849853	4.91978458
565.3868533	7.707865181	552.6856053	4.9129912
565.9849853	7.696928604	552.9690413	4.91657121
567.6856053	7.675036894	554.1935763	4.9206199
567.9690413	7.675181005	554.7604503	4.91808756
569.1935763	7.678222298	555.3868533	4.91797715
569.7604503	7.668642108	555.9849853	4.92785617
570.3868533	7.671644835	557.6856053	4.92472272
570.9849853	7.659786348	557.9690413	4.92347801
572.6856053	7.655943651	559.1935763	4.92429573
572.9690413	7.657339083	559.7604503	4.94043845
574.1935763	7.649277433	560.3868533	4.9346751
574.7604503	7.634718183	560.9849853	4.92961401
575.3868533	7.628610901	562.6856053	4.92973435
575.9849853	7.62950877	562.9690413	4.93024537
577.6856053	7.622175922	564.1935763	4.94272671
577.9690413	7.620128479	564.7604503	4.93908192
579.1935763	7.60331466	565.3868533	4.93763683
579.7604503	7.59776733	565.9849853	4.93992738
580.3868533	7.591820086	567.6856053	4.941909
580.9849853	7.591960951	567.9690413	4.95468403
582.6856053	7.583175017	569.1935763	4.94246448
584.1935763	7.56903229	569.7604503	4.95766345
584.7604503	7.564612917	570.3868533	4.94825624
585.3868533	7.559858799	570.9849853	4.94472257
585.9849853	7.555618458	572.6856053	4.96620392
587.6856053	7.544815143	572.9690413	4.95949514
587.9690413	7.542755133	574.1935763	4.95404637
589.1935763	7.533413388	574.7604503	4.95328427
589.7604503	7.533342823	575.3868533	4.95475972
590.3868533	7.528893374	575.9849853	4.96105948
590.9849853	7.523101922	577.6856053	4.96906783
592.6856053	7.511103565	577.9690413	4.9644967
592.9690413	7.509170297	579.1935763	4.96450018
594.1935763	7.500851298	579.7604503	4.96557546
595.3868533	7.492787874	580.3868533	4.96707757
595.9849853	7.488766254	580.9849853	4.96987648
597.6856053	7.478011815	582.6856053	4.97178061
597.9690413	7.476346295	582.9690413	4.97186992
599.1935763	7.468081429	584.1935763	4.97378338
599.7604503	7.464230967	584.7604503	4.97478048

Final State: j'=1		Final State: j'=0	
E(cm^{-1})	σ (Å^2)	E(cm^{-1})	σ (Å^2)
600.3868533	7.460032322	585.3868533	4.97594548
600.9849853	7.456057462	585.9849853	4.97719998
602.6856053	7.444862402	587.6856053	4.98027282
602.9690413	7.443008293	587.9690413	4.98070464
604.1935763	7.435036753	589.1935763	4.98290545
604.7604503	7.431371266	589.7604503	4.98419004
605.3868533	7.427340657	590.3868533	4.98478282
605.9849853	7.423507049	590.9849853	4.9853405
607.6856053	7.41262101	592.6856053	4.98819431
607.9690413	7.410805866	592.9690413	4.98865362
609.1935763	7.402972863	594.1935763	4.99061644
610.3868533	7.395365985	594.7604503	4.99151935
610.9849853	7.391565267	595.3868533	4.99251631
612.6856053	7.380807007	595.9849853	4.9934708
612.9690413	7.379021016	597.6856053	4.99625439
614.1935763	7.371328164	597.9690413	4.99668485
614.7604503	7.367779287	599.1935763	4.99844191
615.3868533	7.363866445	599.7604503	4.99928968
617.6856053	7.349580041	600.3868533	5.0002351
617.9690413	7.34782609	600.9849853	5.00113897
619.1935763	7.340267434	602.6856053	5.00369879
619.7604503	7.33677875	602.9690413	5.00412346
620.3868533	7.332931482	604.1935763	5.0059515
620.9849853	7.32926609	604.7604503	5.00679373
622.6856053	7.318883784	605.3868533	5.00772044
624.1935763	7.309729631	605.9849853	5.00860021
624.7604503	7.306300968	607.6856053	5.01107293
625.3868533	7.302520218	607.9690413	5.01148178
625.9849853	7.298917912	609.1935763	5.0132401
627.6856053	7.288716922	609.7604503	5.01404986
627.9690413	7.287022652	610.3868533	5.01494163
629.1935763	7.279722197	610.9849853	5.01579001
629.7604503	7.276353178	612.6856053	5.01818507
630.3868533	7.272638093	612.9690413	5.01858172
630.9849853	7.269098312	614.1935763	5.02028683
632.6856053	7.259074219	614.7604503	5.02107138
632.9690413	7.257409325	615.3868533	5.02193476
634.1935763	7.250235479	615.9849853	5.02275566
634.7604503	7.246924935	617.6856053	5.02507098
635.3868533	7.243274397	617.9690413	5.0254542
635.9849853	7.239796091	619.1935763	5.02710116
637.6856053	7.229946458	619.7604503	5.02785882
637.9690413	7.228310563	620.3868533	5.02869257

Final State: j’=1		Final State: j’=0	
E(cm⁻¹)	σ (Å²)	E(cm⁻¹)	σ (Å²)
639.1935763	7.22126182	620.9849853	5.02948526
639.7604503	7.218009009	622.6856053	5.03172094
640.3868533	7.214422183	622.9690413	5.03209092
640.9849853	7.21100464	624.1935763	5.03368079
642.6856053	7.201327109	624.7604503	5.03441204
642.9690413	7.199719831	625.3868533	5.03521659
644.7604503	7.189598506	625.9849853	5.0359814
645.3868533	7.186074485	627.6856053	5.03813767
645.9849853	7.182716778	627.9690413	5.03849442
647.6856053	7.173208839	629.1935763	5.04002706
647.9690413	7.171629753	629.7604503	5.04073182
649.1935763	7.164825809	630.3868533	5.04150708
649.7604503	7.161686043	630.9849853	5.04224396
650.3868533	7.158223904	632.6856053	5.04432078
650.9849853	7.154925187	632.9690413	5.0446643
652.6856053	7.145584425	634.1935763	5.04613975
652.9690413	7.144033131	634.7604503	5.04681805
654.1935763	7.13734893	635.3868533	5.0475641
654.7604503	7.134264487	635.9849853	5.04827305
655.3868533	7.130863343	637.6856053	5.05027051
655.9849853	7.127622784	637.9690413	5.0506008
657.6856053	7.118446797	639.1935763	5.05201911
657.9690413	7.116922873	639.7604503	5.05267097
659.1935763	7.110356732	640.3868533	5.05338777
659.7604503	7.107326759	640.9849853	5.05406881
660.3868533	7.103985758	642.6856053	5.05598691
660.9849853	7.100802505	642.9690413	5.05630398
662.6856053	7.091788917	644.1935763	5.05766517
664.1935763	7.083842188	644.7604503	5.05829056
664.7604503	7.080865962	645.3868533	5.05897813
665.3868533	7.077584192	645.9849853	5.05963127
665.9849853	7.074457439	647.6856053	5.06147003
667.6856053	7.065603945	647.9690413	5.06177385
667.9690413	7.064133621	649.1935763	5.0630779
669.1935763	7.057798545	649.7604503	5.06367683
669.7604503	7.054875277	650.3868533	5.0643352
670.3868533	7.051651963	650.9849853	5.0649604
670.9849853	7.048580904	652.6856053	5.06671978
672.6856053	7.039885334	652.9690413	5.06701038
672.9690413	7.038441266	654.1935763	5.06825725
674.1935763	7.032219364	654.7604503	5.06882971
674.7604503	7.029348385	655.3868533	5.06945882
675.3868533	7.026182732	655.9849853	5.07005611

Final State: j’=1		Final State: j’=0	
E(cm⁻¹)	σ (Å²)	E(cm⁻¹)	σ (Å²)
675.9849853	7.023166692	657.6856053	5.07173602
677.6856053	7.014627036	657.9690413	5.07201339
677.9690413	7.013208875	659.1935763	5.07320303
679.1935763	7.007098794	659.7604503	5.07374901
679.7604503	7.004279473	660.3868533	5.07434883
680.3868533	7.001170858	660.9849853	5.07491814
680.9849853	6.998209169	662.6856053	5.07651853
682.6856053	6.989823831	662.9690413	5.07678262
682.9690413	6.988431353	664.1935763	5.07791495
684.1935763	6.982432081	664.7604503	5.07843439
684.7604503	6.979664018	665.3868533	5.07900487
685.3868533	6.97661195	665.9849853	5.07954615
685.9849853	6.973704305	667.6856053	5.08106683
687.6856053	6.965472445	667.9690413	5.08131762
687.9690413	6.96410554	669.1935763	5.08239249
689.1935763	6.958216864	669.7604503	5.08288531
689.7604503	6.955500029	670.3868533	5.08342634
690.3868533	6.952504687	670.9849853	5.08393952
690.9849853	6.94965124	672.6856053	5.08538015
692.6856053	6.941574187	672.9690413	5.0856176
692.9690413	6.940233213	674.1935763	5.08663473
694.1935763	6.934457167	674.7604503	5.08710079
694.7604503	6.931792903	675.3868533	5.08761224
695.3868533	6.928856595	675.9849853	5.08809714
695.9849853	6.926058973	677.6856053	5.08945721
697.6856053	6.918145832	677.9690413	5.08968121
697.9690413	6.916832903	679.1935763	5.09064013
699.7604503	6.90857777	679.7604503	5.09107919
700.3868533	6.905710541	680.3868533	5.09156076
700.9849853	6.90298369	680.9849853	5.09201709
702.6856053	6.895308769	682.6856053	5.09329568
702.9690413	6.894045985	682.9690413	5.09350605
704.1935763	6.888715265	684.1935763	5.09440595
704.7604503	6.886451277	684.7604503	5.09481757
705.3868533	6.88381086	685.3868533	5.09526875
705.9849853	6.880299853	685.9849853	5.09569596
707.6856053	6.87190378	687.6856053	5.0968912
707.9690413	6.870507218	687.9690413	5.09708759
709.1935763	6.866483488	689.1935763	5.09792672
709.7604503	6.863303245	689.7604503	5.09830998
710.3868533	6.859751682	690.3868533	5.09872965
710.9849853	6.857297753	690.9849853	5.09912655
712.6856053	6.850513714	692.6856053	5.10023425

Final State: j'=1		Final State: j'=0	
E(cm⁻¹)	σ (Å²)	E(cm⁻¹)	σ (Å²)
712.9690413	6.849152636	692.9690413	5.10041581
714.1935763	6.843746182	694.1935763	5.10118991
714.7604503	6.839727642	694.7604503	5.10154244
715.3868533	6.83765708	695.3868533	5.1019264
715.9849853	6.835417234	695.9849853	5.10229082
717.6856053	6.827833651	697.6856053	5.1032981
717.9690413	6.826756606	697.9690413	5.10346202
719.1935763	6.820736805	699.1935763	5.10415561
720.3868533	6.816181171	699.7604503	5.10446772
720.9849853	6.813895483	700.3868533	5.10480482
722.6856053	6.806897809	700.9849853	5.10511781
722.9690413	6.803897998	702.6856053	5.10593709
724.1935763	6.799428989	702.9690413	5.1060573
724.7604503	6.797250492	704.1935763	5.10643168
725.3868533	6.794780327	704.7604503	5.10632114
725.9849853	6.792426703	705.3868533	5.10589462
727.6856053	6.784637835	705.9849853	5.10667086
727.9690413	6.783916277	707.6856053	5.10839602
729.1935763	6.778106542	707.9690413	5.10919445
729.7604503	6.776158096	709.1935763	5.10813532
730.3868533	6.773752111	709.7604503	5.10842102
730.9849853	6.77140675	710.3868533	5.11018999
732.6856053	6.764767191	710.9849853	5.11050109
734.1935763	6.758704617	712.6856053	5.11021707
734.7604503	6.755353056	712.9690413	5.10972251
735.3868533	6.753047345	714.1935763	5.11123651
735.9849853	6.750983312	714.7604503	5.11296339
737.6856053	6.744737088	715.3868533	5.11304236
737.9690413	6.743764133	715.9849853	5.1128526
739.1935763	6.739733989	717.6856053	5.11336169
739.7604503	6.736151986	717.9690413	5.1132577
740.3868533	6.733565331	719.1935763	5.11513284
740.9849853	6.731547145	719.7604503	5.1150162
742.6856053	6.724602361	720.3868533	5.11494468
742.9690413	6.722344232	720.9849853	5.11470894
744.1935763	6.718667125	722.6856053	5.11465978
744.7604503	6.717143846	722.9690413	5.11593606
745.3868533	6.712032151	724.1935763	5.11708418
745.9849853	6.710255254	724.7604503	5.11703093
747.6856053	6.704009322	725.3868533	5.11701823
747.9690413	6.702335603	725.9849853	5.11695897
749.1935763	6.697012757	727.6856053	5.11736085
749.7604503	6.695772145	727.9690413	5.11756556

Final State: j’=1		Final State: j’=0	
E(cm^{-1})	σ (Å^2)	E(cm^{-1})	σ (Å^2)
750.3868533	6.694003314	729.1935763	5.11922875
750.9849853	6.692893653	729.7604503	5.11897101
752.6856053	6.684137971	730.3868533	5.11892283
752.9690413	6.683270513	730.9849853	5.11892277
754.1935763	6.679383251	732.6856053	5.11863095
754.7604503	6.677859192	732.9690413	5.11829103
755.3868533	6.671608337	734.1935763	5.11857289
757.6856053	6.666705889	734.7604503	5.11982976
757.9690413	6.665345635	735.3868533	5.1207026
759.1935763	6.661201643	735.9849853	5.12041501
759.7604503	6.656289943	737.6856053	5.12000099
760.3868533	6.654453347	737.9690413	5.11984065
762.6856053	6.647556237	739.1935763	5.11793386
762.9690413	6.648072648	739.7604503	5.11937938
764.1935763	6.63933869	740.3868533	5.1184371
764.7604503	6.638587412	740.9849853	5.11868796
765.3868533	6.636779841	742.6856053	5.11948589
765.9849853	6.634257319	742.9690413	5.12143764
767.6856053	6.627462528	744.1935763	5.12114947
767.9690413	6.627517656	744.7604503	5.11870456
769.1935763	6.62133339	745.3868533	5.12220118
769.7604503	6.620685758	745.9849853	5.12290078
770.3868533	6.618657446	747.6856053	5.11836188
770.9849853	6.616585857	747.9690413	5.11954493
772.6856053	6.610416122	749.1935763	5.12475819
772.9690413	6.60955166	749.7604503	5.1237307
774.1935763	6.604739824	750.3868533	5.12286657
774.7604503	6.602864114	750.9849853	5.11984115
775.3868533	6.60065453	752.6856053	5.12434658
775.9849853	6.598523297	752.9690413	5.12412283
777.6856053	6.592354503	754.1935763	5.12305547
777.9690413	6.591305032	754.7604503	5.12134604
779.1935763	6.586954291	755.3868533	5.12247483
779.7604503	6.584960582	755.9849853	5.1245605
780.3868533	6.582712301	757.6856053	5.12259764
780.9849853	6.580080597	757.9690413	5.12302445
782.6856053	6.57479613	759.1935763	5.12114794
782.9690413	6.573798664	759.7604503	5.12234655
784.1935763	6.569514287	760.3868533	5.12557287
784.7604503	6.567542342	760.9849853	5.12262178
785.3868533	6.565368924	762.6856053	5.12340689
785.9849853	6.563297686	762.9690413	5.11974733
787.6856053	6.557419659	764.1935763	5.12210335

Final State: j’=1		Final State: j’=0	
E(cm⁻¹)	σ (Å²)	E(cm⁻¹)	σ (Å²)
787.9690413	6.556438481	764.7604503	5.12497299
789.1935763	6.55218405	765.3868533	5.12335627
789.7604503	6.55028025	765.9849853	5.12540427
790.3868533	6.548183201	767.6856053	5.12310851
790.9849853	6.546162778	767.9690413	5.12374014
792.6856053	6.54040791	769.1935763	5.12351423
792.9690413	6.539450636	769.7604503	5.12383923
794.1935763	6.535323333	770.3868533	5.12352188
794.7604503	6.533417357	770.9849853	5.12323069
795.3868533	6.531314622	772.6856053	5.12269917
795.9849853	6.529310132	772.9690413	5.12185254
797.6856053	6.523633347	774.1935763	5.12345819
797.9690413	6.52269119	774.7604503	5.12319457
799.1935763	6.518632981	775.3868533	5.12301945
799.7604503	6.516759909	775.9849853	5.12288992
800.3868533	6.514693702	777.6856053	5.12257032
800.9849853	6.512724136	777.9690413	5.1225514
802.6856053	6.507142548	779.1935763	5.12249056
802.9690413	6.506214933	779.7604503	5.12240669
804.1935763	6.502216221	780.3868533	5.12228304
804.7604503	6.500370037	780.9849853	5.12230632
805.3868533	6.498333628	782.6856053	5.12211463
805.9849853	6.496392765	782.9690413	5.12205528
807.6856053	6.490894012	784.1935763	5.12183958
807.9690413	6.48998038	784.7604503	5.12174326
809.1935763	6.486042252	785.3868533	5.12163479
809.7604503	6.484224144	785.9849853	5.12152846
810.3868533	6.482218753	787.6856053	5.12120986
810.9849853	6.480307387	787.9690413	5.12115463
812.6856053	6.474891586	789.1935763	5.12093888
812.9690413	6.473991612	789.7604503	5.12084893
814.1935763	6.470112101	790.3868533	5.12072054
814.7604503	6.468320902	790.9849853	5.12058785
815.3868533	6.466345104	792.6856053	5.12020128
815.9849853	6.464461893	792.9690413	5.12013534
817.6856053	6.459125567	794.1935763	5.11984416
817.9690413	6.458238773	794.7604503	5.11970575
819.1935763	6.454416007	795.3868533	5.11955018
819.7604503	6.452650972	795.9849853	5.11939918
820.3868533	6.45070399	797.6856053	5.11895759
820.9849853	6.448848178	797.9690413	5.118882
822.6856053	6.443589303	799.1935763	5.11854706
822.9690413	6.44271534	799.7604503	5.11838736

Final State: j’=1		Final State: j’=0	
E(cm⁻¹)	σ (Å²)	E(cm⁻¹)	σ (Å²)
824.1935763	6.43894773	800.3868533	5.11820764
824.7604503	6.437208087	800.9849853	5.11803297
825.3868533	6.43528906	802.6856053	5.11752051
825.9849853	6.433459857	802.9690413	5.11743287
827.6856053	6.428276037	804.1935763	5.11704708
827.9690413	6.427414494	804.7604503	5.11686458
829.1935763	6.423700379	805.3868533	5.11666001
829.7604503	6.421985348	805.9849853	5.11646186
830.3868533	6.420093396	807.6856053	5.11588336
830.9849853	6.418289957	807.9690413	5.11578476
832.6856053	6.413178906	809.1935763	5.11535159
832.9690413	6.412329434	809.7604503	5.11514708
834.1935763	6.408667152	810.3868533	5.11491819
834.7604503	6.406975993	810.9849853	5.1146968
835.3868533	6.405110322	812.6856053	5.11405216
835.9849853	6.403331883	812.9690413	5.11394254
837.6856053	6.398291403	814.1935763	5.11346191
837.9690413	6.397453613	814.7604503	5.1132355
839.1935763	6.39384159	815.3868533	5.11298247
839.7604503	6.39217356	815.9849853	5.11273808
840.3868533	6.390333373	817.6856053	5.11202831
840.9849853	6.388579156	817.9690413	5.1119079
842.6856053	6.383607073	819.1935763	5.11138063
842.9690413	6.382780612	819.7604503	5.11113271
844.1935763	6.379217292	820.3868533	5.11085592
844.7604503	6.377571685	820.9849853	5.11058888
845.3868533	6.375756181	822.6856053	5.10981494
845.9849853	6.37402545	822.9690413	5.10968384
847.6856053	6.369119648	824.1935763	5.10911058
847.9690413	6.368304182	824.7604503	5.10884142
849.1935763	6.364788053	825.3868533	5.1085412
849.7604503	6.363164189	825.9849853	5.10825184
850.3868533	6.361372582	827.6856053	5.10741461
850.9849853	6.359664606	827.9690413	5.107273
852.6856053	6.354823015	829.1935763	5.10665438
852.9690413	6.354018169	829.7604503	5.10636428
854.1935763	6.350547798	830.3868533	5.106041
854.7604503	6.348944977	830.9849853	5.10572961
855.3868533	6.34717656	832.6856053	5.10483004
855.9849853	6.345490618	832.9690413	5.10467808
857.6856053	6.340711268	834.1935763	5.10401482
857.9690413	6.339916713	834.7604503	5.10370411
859.1935763	6.336490648	835.3868533	5.10335807

Final State: j’=1		Final State: j’=0	
E(cm⁻¹)	σ (Å²)	E(cm⁻¹)	σ (Å²)
859.7604503	6.334908206	835.9849853	5.10302503
860.3868533	6.333162258	837.6856053	5.10206406
860.9849853	6.331497676	837.9690413	5.10190189
862.6856053	6.326778585	839.1935763	5.10119466
862.9690413	6.325994023	839.7604503	5.10086366
864.1935763	6.322610881	840.3868533	5.10049523
864.7604503	6.321048209	840.9849853	5.10014084
865.3868533	6.319323987	842.6856053	5.09911943
865.9849853	6.317680097	842.9690413	5.09894722
867.6856053	6.313019407	844.1935763	5.0981967
867.9690413	6.312244505	844.7604503	5.09784573
869.1935763	6.308902935	845.3868533	5.09745528
869.7604503	6.307359421	845.9849853	5.0970799
870.3868533	6.305656286	847.6856053	5.09599902
870.9849853	6.304032445	847.9690413	5.09581692
872.6856053	6.2994283	849.1935763	5.09502385
872.9690413	6.29866278	849.7604503	5.09465321
874.1935763	6.295361508	850.3868533	5.09424109
874.7604503	6.293836528	850.9849853	5.09384505
875.3868533	6.292153802	852.6856053	5.09270569
875.9849853	6.290549376	852.9690413	5.09251388
877.6856053	6.286000055	854.1935763	5.09167895
877.9690413	6.285243619	854.7604503	5.09128899
879.1935763	6.28198138	855.3868533	5.09085556
879.7604503	6.280474375	855.9849853	5.09043923
880.3868533	6.278811435	857.6856053	5.0892424
880.9849853	6.277225829	857.9690413	5.08904103
882.6856053	6.272729618	859.1935763	5.08816496
882.9690413	6.271981993	859.7604503	5.08775603
884.1935763	6.268757588	860.3868533	5.08730166
884.7604503	6.267267999	860.9849853	5.08686539
885.3868533	6.265624224	862.6856053	5.08561208
885.9849853	6.264056854	862.9690413	5.08540136
887.6856053	6.259612141	864.1935763	5.08448487
887.9690413	6.258873037	864.7604503	5.08405729
889.1935763	6.255685287	865.3868533	5.08358237
889.7604503	6.254212578	865.9849853	5.08312652
890.3868533	6.252587398	867.6856053	5.08181776
890.9849853	6.251037715	867.9690413	5.08159781
892.6856053	6.246642868	869.1935763	5.08064166
892.9690413	6.245912034	869.7604503	5.08019578
894.1935763	6.242759817	870.3868533	5.07970069
894.7604503	6.241303459	870.9849853	5.0792256

Final State: j'=1		Final State: j'=0	
E(cm⁻¹)	σ (Å²)	E(cm⁻¹)	σ (Å²)
895.3868533	6.239696272	872.6856053	5.07786244
895.9849853	6.238163708	872.9690413	5.07763345
897.6856053	6.233817228	874.1935763	5.07663841
897.9690413	6.233094374	874.7604503	5.07617457
899.1935763	6.229976564	875.3868533	5.07565968
899.7604503	6.228536059	875.9849853	5.07516575
900.3868533	6.226946317	877.6856053	5.07374919
900.9849853	6.225430315	877.9690413	5.07351137
902.6856053	6.221130585	879.1935763	5.07247813
902.9690413	6.220415475	879.7604503	5.0719967
904.1935763	6.217330936	880.3868533	5.07146239
904.7604503	6.215905713	880.9849853	5.07094997
905.3868533	6.214332785	882.6856053	5.06948113
905.9849853	6.212832785	882.9690413	5.06923461
907.6856053	6.208578127	884.1935763	5.068164
907.9690413	6.207870479	884.7604503	5.06766515
909.1935763	6.204817907	885.3868533	5.06711201
909.7604503	6.203407355	885.9849853	5.06658149
910.3868533	6.201850558	887.6856053	5.06506142
910.9849853	6.200365846	887.9690413	5.0648064
912.6856053	6.196154091	889.1935763	5.06369919
912.9690413	6.195453504	889.7604503	5.06318362
914.1935763	6.192431045	890.3868533	5.06261172
914.7604503	6.191034221	890.9849853	5.0620635
915.3868533	6.189492384	892.6856053	5.06049335
915.9849853	6.18802176	892.9690413	5.06023002
917.6856053	6.183848465	894.1935763	5.05908704
917.9690413	6.18315399	894.7604503	5.058555
919.1935763	6.180156559	895.3868533	5.05796494
919.7604503	6.178770247	895.9849853	5.05739941
920.3868533	6.177238823	897.6856053	5.0557804
920.9849853	6.175776388	897.9690413	5.05550897
922.6856053	6.171604094	899.1935763	5.05433112
922.9690413	6.170901212	899.7604503	5.05378302
924.1935763	6.167742092	900.3868533	5.05317528
924.7604503	6.166469298	900.9849853	5.05259293
925.3868533	6.1652064	902.6856053	5.0509264
925.9849853	6.163517248	902.9690413	5.0506471
927.6856053	6.159608536	904.1935763	5.04943543
927.9690413	6.158785996	904.7604503	5.04887178
929.1935763	6.156200241	905.3868533	5.04824692
929.7604503	6.154651363	905.9849853	5.04764831
930.3868533	6.15330383	907.6856053	5.04593598

Final State: j'=1		Final State: j'=0	
E(cm⁻¹)	σ (Å²)	E(cm⁻¹)	σ (Å²)
930.9849853	6.151837454	907.9690413	5.04564912
932.6856053	6.147856899	909.1935763	5.04440502
932.9690413	6.147021951	909.7604503	5.04382651
934.1935763	6.144313541	910.3868533	5.04318534
934.7604503	6.142910786	910.9849853	5.04257129
935.3868533	6.141351021	912.6856053	5.04081584
935.9849853	6.140057063	912.9690413	5.04052193
937.6856053	6.136189243	914.1935763	5.03924788
937.9690413	6.135493395	914.7604503	5.03865582
939.1935763	6.13251389	915.3868533	5.03799999
939.7604503	6.13115431	915.9849853	5.03737228
940.3868533	6.129617149	917.6856053	5.03558043
940.9849853	6.128386281	917.9690413	5.03528092
942.6856053	6.124483849	919.1935763	5.03398497
942.9690413	6.123784064	919.7604503	5.03338456
944.1935763	6.120877215	920.3868533	5.03272143
944.7604503	6.119546918	920.9849853	5.03208965
945.3868533	6.118084373	922.6856053	5.03032172
945.9849853	6.116787914	922.9690413	5.03003947
947.6856053	6.112891885	924.1935763	5.02891436
947.9690413	6.112281633	924.7604503	5.0280424
949.1935763	6.109340857	925.3868533	5.02712623
949.7604503	6.108024413	925.9849853	5.02682887
950.3868533	6.10657627	927.6856053	5.02472653
950.9849853	6.105199348	927.9690413	5.02461819
952.6856053	6.101259517	929.1935763	5.02266908
952.9690413	6.10048417	929.7604503	5.02225283
954.1935763	6.097915595	930.3868533	5.02138825
954.7604503	6.096563951	930.9849853	5.02084395
955.3868533	6.095107419	932.6856053	5.01872602
955.9849853	6.09371882	932.9690413	5.01866945
957.6856053	6.089535728	934.1935763	5.01701849
957.9690413	6.088799157	934.7604503	5.01649509
959.1935763	6.086368004	935.3868533	5.01596301
959.7604503	6.084994584	935.9849853	5.01498446
960.3868533	6.083827394	937.6856053	5.01267133
960.9849853	6.082204904	937.9690413	5.01248622
962.6856053	6.078733501	939.1935763	5.01128223
962.9690413	6.078010751	939.7604503	5.0107092
964.1935763	6.075363267	940.3868533	5.01014896
964.7604503	6.074291664	940.9849853	5.0089593
965.3868533	6.072788333	942.6856053	5.00687967
965.9849853	6.07137833	942.9690413	5.00662368

Final State: j'=1		Final State: j'=0	
E(cm^{-1})	σ (Å^2)	E(cm^{-1})	σ (Å^2)
967.6856053	6.067801202	944.1935763	5.00530764
967.9690413	6.067184953	944.7604503	5.00468183
969.1935763	6.064251938	945.3868533	5.00401428
969.7604503	6.062772344	945.9849853	5.00329136
970.3868533	6.061866433	947.6856053	5.0008269
970.9849853	6.060583609	947.9690413	5.00044604
972.6856053	6.056630746	949.1935763	4.9992185
972.9690413	6.055989522	949.7604503	4.99856831
974.1935763	6.05377933	950.3868533	4.99784358
974.7604503	6.052090829	950.9849853	4.99715442
975.3868533	6.050567317	952.6856053	4.9948904
975.9849853	6.049212187	952.9690413	4.99483072
977.6856053	6.045420655	954.1935763	4.99298642
977.9690413	6.045016395	954.7604503	4.99241893
979.1935763	6.042582955	955.3868533	4.99171828
979.7604503	6.041039668	955.9849853	4.99104409
980.3868533	6.039511062	957.6856053	4.98938267
980.9849853	6.038673768	957.9690413	4.9890757
982.6856053	6.03510685	959.1935763	4.98714492
982.9690413	6.03404163	959.7604503	4.98620939
984.1935763	6.031537474	960.3868533	4.98514944
984.7604503	6.030529898	960.9849853	4.98496704
985.3868533	6.029016206	962.6856053	4.98231601
985.9849853	6.027569275	962.9690413	4.98214253
987.6856053	6.024188348	964.1935763	4.9799617
987.9690413	6.023579585	964.7604503	4.9793157
989.1935763	6.020851695	965.3868533	4.97880053
989.7604503	6.019654039	965.9849853	4.9783507
990.3868533	6.018347158	967.6856053	4.9752494
990.9849853	6.017142773	967.9690413	4.97496772
992.6856053	6.013472709	969.1935763	4.97392792
992.9690413	6.012828555	969.7604503	4.97372056
994.1935763	6.010268878	970.3868533	4.97163726
994.7604503	6.00908408	970.9849853	4.97094197
995.3868533	6.007776295	972.6856053	4.96925835
995.9849853	6.006531094	972.9690413	4.96895826
997.6856053	6.00297844	974.1935763	4.96672586
997.9690413	6.00238474	974.7604503	4.96614488
999.1935763	5.999842878	975.3868533	4.96570633
999.7604503	5.998725622	975.9849853	4.9649138
1000.386853	5.997283476	977.6856053	4.96272963
1000.984983	5.996065399	977.9690413	4.96246502
1002.685603	5.992554719	979.1935763	4.95991718

Final State: j’=1		Final State: j’=0	
E(cm^{-1})	σ (Å^2)	E(cm^{-1})	σ (Å^2)
1002.969043	5.991968692	979.7604503	4.9599535
1004.193573	5.989439317	980.3868533	4.95884389
1004.760453	5.988270269	980.9849853	4.95777687
1005.386853	5.986980041	982.6856053	4.95569785
1005.984983	5.985749005	982.9690413	4.95572507
1007.685603	5.982260433	984.1935763	4.95338409
1007.969043	5.981676963	984.7604503	4.95220129
1009.193573	5.979158473	985.3868533	4.95196198
1009.760453	5.978000398	985.9849853	4.95156461
1010.386853	5.976722443	987.6856053	4.94878379
1010.984983	5.97550348	987.9690413	4.94818305
1012.685603	5.972044979	989.1935763	4.94664565
1012.969043	5.971469655	989.7604503	4.94589087
1014.193573	5.968987705	990.3868533	4.94502653
1014.760453	5.967840689	990.9849853	4.94416843
1015.386853	5.966574593	992.6856053	4.94163325
1015.984983	5.9653669	992.9690413	4.94127789
1017.685603	5.961940369	994.1935763	4.93960322
1017.969043	5.961370433	994.7604503	4.93880157
1019.193573	5.95891217	995.3868533	4.93791096
1019.760453	5.957776335	995.9849853	4.9370546
1020.386853	5.956522783	997.6856053	4.93458996
1020.984983	5.955327295	997.9690413	4.93418393
1022.685603	5.951936298	999.1935763	4.93242003
1022.969043	5.951372261	999.7604503	4.93154566
1024.193573	5.948939182	1000.386853	4.93067896
1024.760453	5.94781489	1000.984983	4.929835
1025.386853	5.946574027	1002.685603	4.92737249
1025.984983	5.945390645	1002.969043	4.9269595
1027.685603	5.942033928	1004.193573	4.92517229
1029.193573	5.939067285	1004.760453	4.92434308
1029.760453	5.937954474	1005.386853	4.92342524
1030.386853	5.936726317	1005.984983	4.92254646
1030.984983	5.935555109	1007.685603	4.92003818
1032.685603	5.932233037	1007.969043	4.91961883
1034.193573	5.929297176	1009.193573	4.91781543
1034.760453	5.92819593	1009.760453	4.91697747
1035.386853	5.92698056	1010.386853	4.9160491
1035.984983	5.925821541	1010.984983	4.91516084
1037.685603	5.922534191	1012.685603	4.91262729
1039.193573	5.919629113	1012.969043	4.91220391
1039.760453	5.91853943	1014.193573	4.91037128
1040.386853	5.917336882	1014.760453	4.90952092

Final State: j’=1		Final State: j’=0	
E(cm^{-1})	σ (Å^2)	E(cm^{-1})	σ (Å^2)
1040.984983	5.916190094	1015.386853	4.90857988
1042.685603	5.912937534	1015.984983	4.90768
1044.193573	5.910063354	1017.685603	4.90511503
1044.760453	5.90898531	1017.969043	4.90468658
1045.386853	5.907795608	1019.193573	4.9028321
1045.984983	5.90666108	1019.760453	4.90197171
1047.685603	5.90344346	1020.386853	4.90101959
1049.193573	5.900600263	1020.984983	4.90010907
1049.760453	5.899533879	1022.685603	4.89751309
1050.386853	5.898357055	1022.969043	4.8970794
1050.984983	5.897234853	1024.193573	4.89520239
1052.685603	5.894052227	1024.760453	4.89433163
1054.193573	5.891240142	1025.386853	4.89336811
1054.760453	5.890185447	1025.984983	4.89244675
1055.386853	5.889021539	1027.685603	4.88982023
1055.984983	5.887911686	1029.193573	4.88748266
1057.685603	5.884764203	1029.760453	4.88660186
1059.193573	5.881983256	1030.386853	4.88562722
1059.760453	5.880940291	1030.984983	4.8846953
1060.386853	5.879789359	1032.685603	4.88203873
1060.984983	5.878691861	1034.193573	4.87967465
1062.685603	5.87557959	1034.760453	4.8787839
1064.193573	5.872829909	1035.386853	4.87779829
1064.760453	5.871798702	1035.984983	4.87685591
1065.386853	5.870660749	1037.685603	4.87416974
1065.984983	5.869575676	1039.193573	4.87177955
1067.685603	5.866498694	1039.760453	4.87087901
1069.193573	5.863780317	1040.386853	4.86988262
1069.760453	5.86276089	1040.984983	4.86892996
1070.386853	5.861635957	1042.685603	4.86621467
1070.984983	5.860563311	1044.193573	4.86379875
1072.685603	5.857521693	1044.760453	4.86288859
1074.193573	5.854834678	1045.386853	4.86188159
1074.760453	5.853827041	1045.984983	4.8609188
1075.386853	5.852715137	1047.685603	4.85817482
1075.984983	5.851654945	1049.193573	4.85573359
1077.685603	5.848648723	1049.760453	4.85481394
1079.193573	5.845993114	1050.386853	4.85379646
1079.760453	5.844997273	1050.984983	4.85282371
1080.386853	5.843898422	1052.685603	4.85005147
1080.984983	5.842850652	1054.193573	4.84758534
1084.193573	5.837255669	1054.760453	4.84665635
1084.760453	5.83627163	1055.386853	4.84562858

Final State: j'=1		Final State: j'=0	
E(cm⁻¹)	σ (Å²)	E(cm⁻¹)	σ (Å²)
1085.386853	5.835185808	1055.984983	4.84464599
1085.984983	5.834150526	1057.685603	4.84184595
1089.193573	5.828622308	1059.193573	4.83935528
1089.760453	5.827654158	1059.760453	4.8384171
1090.386853	5.826577272	1060.386853	4.83737917
1090.984983	5.825554443	1060.984983	4.83638692
1094.193573	5.820092911	1062.685603	4.8335595
1094.760453	5.819132456	1064.193573	4.83104466
1095.386853	5.818072677	1064.760453	4.83009741
1095.984983	5.817062261	1065.386853	4.82904952
1099.193573	5.811667291	1065.984983	4.82804775
1099.760453	5.810718565	1067.685603	4.82519339
1100.984983	5.808673707	1069.193573	4.82265475
1104.193573	5.803345061	1069.760453	4.82169861
1104.760453	5.802408042	1070.386853	4.82064087
1105.984983	5.800388445	1070.984983	4.81962975
1109.193573	5.795125781	1072.685603	4.81674883
1109.760453	5.794200407	1074.193573	4.81418681
1110.984983	5.792205921	1074.760453	4.81322188
1114.193573	5.787008869	1075.386853	4.81215449
1114.760453	5.786095043	1075.984983	4.81113415
1115.984983	5.784125508	1077.685603	4.80822713
1119.193573	5.77899358	1079.193573	4.80564206
1119.760453	5.77809122	1079.760453	4.80466852
1120.984983	5.776146421	1080.386853	4.8035916
1124.193573	5.771079039	1080.984983	4.8025622
1124.760453	5.770188042	1084.193573	4.79702177
1125.984983	5.768267706	1084.760453	4.79603975
1129.193573	5.763264169	1085.386853	4.79495347
1129.760453	5.762384394	1085.984983	4.79391515
1130.984983	5.760488282	1089.193573	4.78832721
1134.193573	5.755547794	1089.760453	4.7873355
1134.760453	5.754679125	1090.386853	4.78624136
1135.984983	5.752806874	1090.984983	4.78519426
1139.193573	5.74792857	1094.193573	4.77955962
1139.760453	5.747070804	1094.760453	4.77856104
1140.984983	5.745222084	1095.386853	4.77745653
1144.193573	5.740404975	1095.984983	4.77640081
1144.760453	5.73955795	1099.193573	4.77072027
1145.984983	5.737732356	1099.760453	4.76971364
1149.193573	5.732975339	1100.984983	4.7675361
1149.760453	5.732138859	1104.193573	4.7618105
1150.984983	5.73033597	1104.760453	4.76079598

Final State: j’=1		Final State: j’=0	
E(cm^{-1})	σ (Å^2)	E(cm^{-1})	σ (Å^2)
1154.193573	5.72563791	1105.984983	4.7586014
1154.760453	5.724811764	1109.193573	4.75283162
1155.984983	5.723031133	1109.760453	4.75180935
1159.193573	5.718390919	1110.984983	4.74959809
1159.760453	5.717574923	1114.193573	4.74378498
1160.984983	5.715816173	1114.760453	4.74275512
1164.193573	5.711233177	1115.984983	4.74052751
1165.984983	5.708690791	1119.193573	4.73467198
1169.193573	5.704174115	1119.760453	4.73363467
1170.984983	5.701764194	1120.984983	4.73139106
1174.193573	5.69721559	1124.193573	4.72549401
1175.984983	5.694740225	1124.760453	4.72444942
1179.193573	5.690299414	1125.984983	4.72219018
1180.984983	5.687774114	1129.193573	4.71625252
1184.193573	5.683377238	1129.760453	4.71520083
1185.984983	5.680958435	1130.984983	4.7129263
1189.193573	5.676618346	1134.193573	4.706949
1190.984983	5.674217998	1134.760453	4.70589037
1194.193573	5.669916142	1135.984983	4.70360092
1195.984983	5.667553276	1139.193573	4.69758495
1199.193573	5.663341202	1139.760453	4.69651955
1200.984983	5.660957777	1140.984983	4.69421554
1204.193573	5.656926135	1144.193573	4.68816187
1205.984983	5.654582234	1144.760453	4.68708988
1209.193573	5.65026381	1145.984983	4.68477169
1210.984983	5.647915583	1149.193573	4.67868127
1214.193573	5.643782502	1149.760453	4.67760284
1215.984983	5.641536956	1150.984983	4.67527081
1219.193573	5.637380114	1154.193573	4.66914449
1220.984983	5.635166986	1154.760453	4.66805976
1224.193573	5.631029115	1155.984983	4.66571417
1225.984983	5.628807824	1159.193573	4.65955249
1229.193573	5.624800532	1159.760453	4.65846152
1230.984983	5.622541832	1160.984983	4.65610239
1234.193573	5.618568629	1164.193573	4.64990447
1235.984983	5.616307164	1165.984983	4.64643267
1239.193573	5.612333239	1169.193573	4.64017839
1240.984983	5.610094177	1170.984983	4.63672353
1244.193573	5.606117073	1174.193573	4.63041881
1245.984983	5.603891334	1175.984983	4.62700129
1249.193573	5.599952662	1179.193573	4.62069818
1250.984983	5.597748114	1180.984983	4.61721158
1254.193573	5.593807329	1184.193573	4.61095294

Final State: j'=1		Final State: j'=0	
E(cm⁻¹)	σ (Å²)	E(cm⁻¹)	σ (Å²)
1255.984983	5.591611647	1185.984983	4.60736397
1259.193573	5.587680954	1189.193573	4.60106774
1260.984983	5.585489219	1190.984983	4.59748994
1264.193573	5.581567696	1194.193573	4.59118254
1265.984983	5.579379772	1195.984983	4.58756694
1269.193573	5.575463181	1199.193573	4.58121936
1270.984983	5.573277397	1200.984983	4.57758737
1275.984983	5.567178015	1204.193573	4.57107152
1280.984983	5.561077184	1205.984983	4.56767061
1285.984983	5.554970627	1209.193573	4.56116119
1290.984983	5.548854037	1210.984983	4.55768938
1295.984983	5.542723274	1214.193573	4.55128858
1300.984983	5.536574293	1215.984983	4.54746701
1305.984983	5.530403119	1219.193573	4.54108231
1310.984983	5.524205943	1220.984983	4.53747164
1315.984983	5.517979086	1224.193573	4.53098681
1320.984983	5.511718972	1225.984983	4.52731218
1325.984983	5.505422243	1229.193573	4.52085456
1330.984983	5.499085677	1230.984983	4.51740374
1335.984983	5.492706241	1234.193573	4.51080216
1340.984983	5.486281077	1235.984983	4.50715739
1345.984983	5.479807522	1239.193573	4.50064651
1350.984983	5.473283137	1240.984983	4.49701518
1355.984983	5.466705636	1244.193573	4.49050539
1360.984983	5.460073013	1245.984983	4.48687508
1365.984983	5.453383428	1249.193573	4.48033641
1370.984983	5.446635265	1250.984983	4.47669306
1375.984983	5.439827126	1254.193573	4.47016449
1380.984983	5.432957924	1255.984983	4.46651636
1385.984983	5.426026604	1259.193573	4.45998492
1390.984983	5.419032544	1260.984983	4.45633766
1395.984983	5.411975188	1264.193573	4.44980415
1400.984983	5.40485428	1265.984983	4.44615659
1405.984983	5.397669752	1269.193573	4.43962419
		1270.984983	4.43597763
		1275.984983	4.42580317
		1280.984983	4.41563622
		1285.984983	4.4054796
		1290.984983	4.39533616
		1295.984983	4.38520874
		1300.984983	4.37510015
		1305.984983	4.36501316
		1310.984983	4.35495055

Final State: j’=1		Final State: j’=0	
E(cm⁻¹)	σ (Å²)	E(cm⁻¹)	σ (Å²)
		1315.984983	4.34491503
		1320.984983	4.3349093
		1325.984983	4.324936
		1330.984983	4.31499775
		1335.984983	4.30509709
		1340.984983	4.29523653
		1345.984983	4.28541854
		1350.984983	4.27564548
		1355.984983	4.26591973
		1360.984983	4.25624353
		1365.984983	4.24661912
		1370.984983	4.23704863
		1375.984983	4.22753413
		1380.984983	4.21807765
		1385.984983	4.20868112
		1390.984983	4.19934643
		1395.984983	4.19007532
		1400.984983	4.18086967
		1405.984983	4.17173102

Initial rotational state: j=1

Final State: j'=5		Final State: j'=4		Final State: j'=3	
E(cm⁻¹)	σ (Å²)	E(cm⁻¹)	σ (Å²)	E(cm⁻¹)	σ (Å²)
411.308892	0.031498935	274.517483	0.102105319	165.084357	0.567935782
411.408892	0.054806019	274.617483	0.174666327	165.184357	0.942772001
411.508892	0.042363372	274.717483	0.139200081	165.384357	0.851988997
411.608892	0.045701343	274.817483	0.154336602	165.484357	0.892771087
411.708892	0.049093666	274.917483	0.166446766	165.584357	0.98794545
411.808892	0.05593948	275.017483	0.196910813	165.61076	1.072830605
411.908892	0.064085755	275.117483	0.229349465	165.784357	1.121091089
412.008892	0.067842353	275.217483	0.201977553	165.984357	1.213444185
412.108892	0.062962093	275.317483	0.201507315	168.192948	1.957299485
412.208892	0.070479693	275.417483	0.229534863	169.984357	1.988574736
412.308892	0.067238903	275.517483	0.210425112	170.184357	2.011157932
412.408892	0.066034676	275.61076	0.21031894	170.284357	2.017980095
412.508892	0.066394457	278.192948	0.338026164	170.61076	2.008552355
412.608892	0.067130775	279.617483	0.381640597	172.909512	2.389356223
412.708892	0.067957658	279.817483	0.386703849	173.192948	2.373332308
412.808892	0.068768812	280.017483	0.390158819	174.984357	2.436407819
412.909512	0.069528923	280.61076	0.425206537	175.61076	2.533885284
413.192948	0.071410098	282.909512	0.457400559	178.192948	2.707380549
414.417483	0.110057961	283.192948	0.459066621	179.984357	3.127446443
414.984357	0.103241685	284.417483	0.476665757	180.61076	2.793093242
415.61076	0.105860197	285.61076	0.540155588	182.909512	2.859530851
416.208892	0.112593354	288.192948	0.550827304	183.192948	2.866693841
416.308892	0.113585797	289.417483	0.666540809	184.984357	2.91168756
416.408892	0.114470839	292.909512	0.598950129	188.192948	3.146520729
416.508892	0.115240468	295.61076	0.625494038	193.192948	3.137143334
416.608892	0.115892016	297.909512	0.679471894	197.909512	3.260322103
416.708892	0.11642218	299.417483	0.669081276	198.192948	3.265379415
416.808892	0.116812339	303.192948	0.692942928	200.61076	3.306787839
416.908892	0.116989799	304.417483	0.70522167	203.192948	3.352981421
417.008892	0.117258217	305.61076	0.71829236	208.192948	3.433006924
417.108892	0.118347366	307.909512	0.737108531	209.984357	3.446787794
417.208892	0.119722093	309.984357	0.751691993	212.909512	3.510102026
417.308892	0.121202268	313.192948	0.776330247	213.192948	3.591171035
417.909512	0.153217014	314.417483	0.786368505	214.984357	3.787407187
418.192948	0.176980101	317.909512	0.81499367	218.192948	4.022511898
419.417483	0.136669445	320.61076	0.836628902	222.909512	3.97484351
419.984357	0.13721378	322.909512	0.855022996	223.192948	3.923641465
420.61076	0.139044384	325.61076	0.876782644	225.61076	4.073969028
421.208892	0.141940524	329.984357	0.912074886	228.192948	3.978524203
422.909512	0.163046034	332.909512	0.935514921	233.192948	4.112096821
423.192948	0.163445838	334.984357	0.952010759	235.61076	4.601300467
424.417483	0.161337835	335.61076	0.956963196	238.192948	4.266847519

Final State: j'=5		Final State: j'=4		Final State: j'=3	
E(cm⁻¹)	σ (Å²)	E(cm⁻¹)	σ (Å²)	E(cm⁻¹)	σ (Å²)
424.984357	0.162211653	338.192948	0.977189192	240.61076	4.197621579
425.61076	0.188593167	339.417483	0.986635306	242.909512	4.606848774
426.208892	0.192702306	340.61076	0.995710401	243.192948	4.469443416
427.909512	0.171492995	343.192948	1.014669056	248.192948	4.972376543
428.192948	0.172448455	345.61076	1.03077779	249.984357	4.733512032
429.417483	0.176015203	348.192948	1.042031311	253.192948	4.843618431
429.984357	0.1775111	349.984357	1.053927431	254.984357	4.522395109
430.61076	0.179173957	350.61076	1.089438812	255.61076	5.37171967
431.208892	0.180830661	353.192948	1.091606971	260.61076	4.844191649
432.909512	0.187420703	354.984357	1.13033834	262.909512	5.222768924
433.192948	0.18971505	357.909512	1.171673643	263.192948	5.427932531
434.984357	0.197037534	364.417483	1.212039967	264.984357	5.027660056
435.61076	0.196775446	365.61076	1.196327592	267.909512	5.373997811
436.208892	0.197438399	369.984357	1.252802966	268.192948	5.149685406
437.909512	0.200961488	370.61076	1.247996547	269.984357	4.999062175
438.192948	0.201648007	374.984357	1.302351914	270.61076	4.845595652
439.417483	0.204808817	379.984357	1.354094453	274.517483	5.034350228
439.984357	0.206390346	382.909512	1.320093534	274.617483	5.029818563
440.61076	0.208249649	384.417483	1.331821044	274.717483	4.991623311
441.208892	0.210138587	385.61076	1.378574114	274.817483	4.9489355
442.909512	0.215544993	389.417483	1.414441455	274.917483	4.915762276
443.192948	0.216363058	392.909512	1.517718225	274.984357	4.924018021
444.417483	0.219651749	394.984357	1.455050579	275.017483	4.993373448
444.984357	0.221113387	395.61076	1.443414796	275.117483	5.436596099
445.61076	0.222725905	398.192948	1.489920868	275.217483	5.285628813
446.208892	0.224276523	399.417483	1.445005128	275.317483	5.138884645
447.909512	0.228786045	400.61076	1.563105559	275.417483	5.301726676
448.192948	0.229554066	402.909512	1.514881912	275.517483	5.212774719
449.417483	0.2329264	403.192948	1.534208098	275.61076	5.129818948
449.984357	0.234514853	408.192948	1.545619616	275.617483	5.125853509
450.61076	0.236286137	409.417483	1.621940361	275.717483	5.081166758
451.208892	0.237989024	410.61076	1.669677556	275.817483	5.050991714
452.909512	0.242857059	411.308892	1.584993482	277.909512	5.148479854
453.192948	0.243668975	411.408892	1.562065694	278.192948	5.127243162
454.417483	0.24717372	411.508892	1.570639129	279.417483	5.073074134
454.984357	0.248795073	411.608892	1.557601498	279.517483	5.070577027
455.61076	0.250587043	411.708892	1.53980351	279.617483	5.068303188
456.208892	0.25229955	411.808892	1.51966158	279.717483	5.066496796
457.909512	0.25718455	411.908892	1.58922764	279.817483	5.065918514
458.192948	0.258001884	412.008892	1.610354642	279.917483	5.068812138
459.417483	0.261545675	412.108892	1.584245163	279.984357	5.075798462
459.984357	0.263193528	412.208892	1.581694406	280.017483	5.08243327
460.61076	0.265019903	412.308892	1.604890652	280.117483	5.134787127

Final State: j’=5		Final State: j’=4		Final State: j’=3	
E(cm⁻¹)	σ (Å²)	E(cm⁻¹)	σ (Å²)	E(cm⁻¹)	σ (Å²)
461.208892	0.266769108	412.408892	1.592320088	280.217483	5.327209759
462.909512	0.271768813	412.508892	1.583959134	280.317483	5.660983712
463.192948	0.272605614	412.608892	1.577568326	280.61076	5.281316395
464.417483	0.276231332	412.708892	1.571969872	282.909512	5.233573097
464.984357	0.277915238	412.808892	1.566493119	283.192948	5.230808375
465.61076	0.279779778	412.909512	1.560541683	284.417483	5.231391536
466.208892	0.281563799	413.192948	1.534341012	284.984357	5.234530328
467.909512	0.286655082	414.417483	1.584546527	285.61076	5.247853535
468.192948	0.287506329	414.984357	1.583360299	287.909512	5.305748171
469.417483	0.291192844	415.61076	1.577463549	288.192948	5.307687207
469.984357	0.292904326	416.208892	1.570046439	289.417483	5.452725903
470.61076	0.294799147	416.308892	1.568680352	289.984357	5.404630903
471.208892	0.296611975	416.408892	1.567165403	290.61076	5.398107966
472.909512	0.301784962	416.508892	1.565386365	292.909512	5.440994651
473.192948	0.302649788	416.608892	1.563159762	293.192948	5.447132823
474.417483	0.306394688	416.708892	1.560188566	294.417483	5.473777074
474.984357	0.308132948	416.808892	1.556045636	294.984357	5.486068051
475.61076	0.31005709	416.908892	1.551310136	295.61076	5.499564815
476.208892	0.311897592	417.008892	1.561234578	297.909512	5.567470741
477.909512	0.317147104	417.108892	1.569335566	298.192948	5.57360189
478.192948	0.318024329	417.208892	1.57721096	299.417483	5.59950773
479.417483	0.321821488	417.308892	1.571889066	299.984357	5.612044145
479.984357	0.323583196	417.909512	1.581465199	300.61076	5.626158367
480.61076	0.325532677	418.192948	1.575162899	302.909512	5.679061197
481.208892	0.327396804	419.417483	1.602387537	303.192948	5.685672446
482.909512	0.332710348	419.984357	1.603370531	304.417483	5.714633001
483.192948	0.33359778	420.61076	1.604811044	304.984357	5.728323885
484.417483	0.337437436	421.208892	1.605657284	305.61076	5.743599225
484.984357	0.339217897	422.909512	1.60387735	307.909512	5.799075614
485.61076	0.341187399	423.192948	1.606965212	308.192948	5.805815577
486.208892	0.343069921	424.417483	1.618853279	309.417483	5.8348239
487.909512	0.348431608	424.984357	1.62043703	309.984357	5.848222705
488.192948	0.34932642	425.61076	1.604555974	310.61076	5.863023339
489.417483	0.353195624	426.208892	1.624958817	312.909512	5.917406876
489.984357	0.354988388	427.909512	1.647994374	313.192948	5.924126788
490.61076	0.356970384	428.192948	1.649567679	314.417483	5.953197781
491.208892	0.358863695	429.417483	1.657454986	314.984357	5.966672165
492.909512	0.364249241	429.984357	1.661341407	315.61076	5.981568166
493.192948	0.365146953	430.61076	1.665634387	317.909512	6.036231586
494.417483	0.36902467	431.208892	1.669660326	318.192948	6.042968253
494.984357	0.370818971	432.909512	1.679152186	319.417483	6.072063464
495.61076	0.372800693	433.192948	1.679728976	319.984357	6.085528553
496.208892	0.374691656	434.417483	1.686680072	320.61076	6.100406123

Final State: j'=5		Final State: j'=4		Final State: j'=3	
E(cm⁻¹)	σ (Å²)	E(cm⁻¹)	σ (Å²)	E(cm⁻¹)	σ (Å²)
497.909512	0.380057663	434.984357	1.693753314	322.909512	6.155006134
498.192948	0.380950008	435.61076	1.699855982	323.192948	6.161740042
499.417483	0.384796324	436.208892	1.70486012	324.417483	6.190839967
499.984357	0.386570989	437.909512	1.717865473	324.984357	6.204315764
500.61076	0.388526681	438.192948	1.719965725	325.61076	6.219210472
501.208892	0.390388011	439.417483	1.728900533	327.909512	6.273907338
502.909512	0.395638507	439.984357	1.732949064	328.192948	6.280655887
503.192948	0.396506164	440.61076	1.737345359	329.417483	6.309824548
504.417483	0.400222756	441.208892	1.74147994	329.984357	6.323335291
504.984357	0.401922142	442.909512	1.753468468	330.61076	6.338271361
505.61076	0.403780619	443.192948	1.755559094	332.909512	6.393153525
506.208892	0.405532633	444.417483	1.764775313	333.192948	6.39992966
507.909512	0.410348361	444.984357	1.769072669	334.417483	6.429233198
508.192948	0.411118966	445.61076	1.773811734	334.984357	6.442816345
509.417483	0.414288387	446.208892	1.778320134	335.61076	6.457840858
509.984357	0.415634045	447.909512	1.791038325	337.909512	6.513143066
510.61076	0.416984693	448.192948	1.793144298	338.192948	6.519983775
511.208892	0.418078104	449.417483	1.802204731	339.417483	6.549608108
512.909512	0.418426452	449.984357	1.806382543	339.984357	6.56336663
513.192948	0.417309474	450.61076	1.810991909	340.61076	6.578608988
514.417483	0.417285091	451.208892	1.815390341	342.909512	6.635009699
514.984357	0.434097351	452.909512	1.82790983	343.192948	6.642030345
515.61076	0.427770338	453.192948	1.830000524	344.417483	6.672600693
516.208892	0.431755792	454.417483	1.839047314	344.984357	6.686917192
517.909512	0.428945525	454.984357	1.843241796	345.61076	6.702896985
518.192948	0.430968391	455.61076	1.847879946	347.909512	6.763833503
519.417483	0.45302447	456.208892	1.852310747	348.192948	6.771660941
519.984357	0.446620358	457.909512	1.864911212	349.417483	6.7937823
520.61076	0.457165088	458.192948	1.86701099	349.984357	6.752499425
521.208892	0.446960059	459.417483	1.876080217	350.61076	6.725856159
522.909512	0.456570225	459.984357	1.880277186	352.909512	6.842120015
523.192948	0.463144851	460.61076	1.884913961	353.192948	6.863071388
524.417483	0.470087371	461.208892	1.88934073	354.417483	6.797365704
524.984357	0.463810249	462.909512	1.901925388	354.984357	6.819447361
525.61076	0.456441383	463.192948	1.904022874	355.61076	6.889902036
526.208892	0.474267937	464.417483	1.91308583	357.909512	6.866044501
527.909512	0.492369901	464.984357	1.917282212	358.192948	6.908392386
528.192948	0.490288318	465.61076	1.921920046	359.417483	6.92815763
529.417483	0.483456802	466.208892	1.926349383	359.984357	6.996292531
529.984357	0.47428263	467.909512	1.938947422	360.61076	7.025153
530.61076	0.484790639	468.192948	1.94104773	362.909512	6.954711612
531.208892	0.494879943	469.417483	1.95012368	363.192948	6.967434893
532.909512	0.509693168	469.984357	1.954326266	364.417483	7.103185075

Final State: j'=5		Final State: j'=4		Final State: j'=3	
E(cm⁻¹)	σ (Å²)	E(cm⁻¹)	σ (Å²)	E(cm⁻¹)	σ (Å²)
533.192948	0.508469314	470.61076	1.958970943	364.984357	7.125901671
534.417483	0.504857957	471.208892	1.963406736	365.61076	7.141718364
534.984357	0.501345019	472.909512	1.976022775	367.909512	7.066759416
535.61076	0.488654562	473.192948	1.978126066	368.192948	7.137999161
536.208892	0.506275658	474.417483	1.987215126	369.417483	7.221298081
537.909512	0.533140131	474.984357	1.991423992	369.984357	7.242146801
538.192948	0.530148159	475.61076	1.996075854	370.61076	7.26259342
539.417483	0.526491505	476.208892	2.0005188	372.909512	7.206632193
539.984357	0.525627331	477.909512	2.01315707	373.192948	7.193391783
540.61076	0.524052309	478.192948	2.015264366	374.417483	7.331757869
541.208892	0.520301018	479.417483	2.024371793	374.984357	7.354430401
542.909512	0.529312115	479.984357	2.028589768	375.61076	7.376166442
543.192948	0.533896693	480.61076	2.033252129	377.909512	7.323272646
544.417483	0.54642975	481.208892	2.037705536	378.192948	7.307487229
544.984357	0.543708054	482.909512	2.050375868	379.417483	7.378260951
545.61076	0.541522112	483.192948	2.052488855	379.984357	7.459134651
546.208892	0.538901799	484.417483	2.061622027	380.61076	7.493922553
547.909512	0.527863608	484.984357	2.065852572	382.909512	7.571394348
548.192948	0.534986302	485.61076	2.07052935	383.192948	7.550893026
549.417483	0.548120999	486.208892	2.074997041	384.417483	7.529440091
549.984357	0.565591864	487.909512	2.087711007	384.984357	7.377972996
550.61076	0.564446155	488.192948	2.089831725	385.61076	7.498413047
551.208892	0.556575503	489.417483	2.099000019	387.909512	7.585442711
552.909512	0.559453675	489.984357	2.103247819	388.192948	7.624470509
553.192948	0.546950124	490.61076	2.10794446	389.417483	7.537331513
554.417483	0.592855063	491.208892	2.112431941	389.984357	7.548907245
554.984357	0.581088213	492.909512	2.125207068	390.61076	7.675073882
555.61076	0.564841296	493.192948	2.127338759	392.909512	7.555599238
556.208892	0.524400271	494.417483	2.136557281	393.192948	7.501172707
557.909512	0.617918772	494.984357	2.140830041	394.417483	7.778151421
558.192948	0.608796079	495.61076	2.14555565	394.984357	7.810268821
559.417483	0.577400541	496.208892	2.150072271	395.61076	7.698483605
559.984357	0.577224332	497.909512	2.162939288	397.909512	7.868204753
560.61076	0.632870902	498.192948	2.165087762	398.192948	7.883204581
561.208892	0.619042873	499.417483	2.174384529	399.417483	7.852820473
562.909512	0.597273375	499.984357	2.178697045	399.984357	7.680200434
563.192948	0.588930135	500.61076	2.183469567	400.61076	7.814285048
564.417483	0.627700569	501.208892	2.188034272	402.909512	7.936623607
564.984357	0.617909138	502.909512	2.201059344	403.192948	7.919345699
565.61076	0.620782526	503.192948	2.203237855	404.417483	7.706407838
566.208892	0.615367422	504.417483	2.212679918	404.984357	7.960146947
567.909512	0.586145086	504.984357	2.217069867	405.61076	8.000012655
568.192948	0.577730658	505.61076	2.221937284	407.909512	7.961222549

Final State: j'=5		Final State: j'=4		Final State: j'=3	
E(cm⁻¹)	σ (Å²)	E(cm⁻¹)	σ (Å²)	E(cm⁻¹)	σ (Å²)
569.417483	0.640336348	506.208892	2.226603445	408.192948	8.053503008
569.984357	0.62671408	507.909512	2.239997329	409.417483	8.002441706
570.61076	0.654799737	508.192948	2.242253001	409.984357	7.946753723
571.208892	0.635084828	509.417483	2.25210796	410.61076	8.02575811
572.909512	0.619621386	509.984357	2.256750038	411.308892	8.146848074
573.192948	0.651621913	510.61076	2.261964625	411.408892	8.108086685
574.417483	0.678720512	511.208892	2.267060154	411.508892	8.143629737
574.984357	0.655502266	512.909512	2.282878915	411.608892	8.151433746
575.61076	0.643183874	513.192948	2.285882994	411.708892	8.153534788
576.208892	0.64802228	514.417483	2.283011066	411.808892	8.098119316
577.909512	0.638543193	514.984357	2.284260526	411.908892	7.965987623
578.192948	0.652266941	515.61076	2.298283073	412.008892	7.981785995
579.417483	0.645112002	516.208892	2.291655909	412.108892	8.070893005
579.984357	0.641780226	517.909512	2.314772875	412.208892	8.002918406
580.61076	0.637157178	518.192948	2.313596606	412.308892	8.050819274
581.208892	0.627063562	519.417483	2.312194213	412.408892	8.091292763
582.909512	0.654485775	519.984357	2.325212097	412.508892	8.108315036
583.192948	0.65516548	520.61076	2.328834125	412.608892	8.118428835
584.417483	0.65539049	521.208892	2.340959275	412.708892	8.125680156
584.984357	0.65575145	522.909512	2.346145966	412.808892	8.131458292
585.61076	0.655740535	523.192948	2.329885913	412.909512	8.136258575
586.208892	0.654826619	524.417483	2.360171939	413.192948	8.136207392
587.909512	0.659660895	524.984357	2.368801706	414.417483	8.094018415
588.192948	0.660752267	525.61076	2.364425417	414.984357	8.126868381
589.417483	0.66087543	526.208892	2.364563427	415.61076	8.145677167
589.984357	0.660371491	527.909512	2.379434843	416.208892	8.154678403
590.61076	0.671306875	528.192948	2.384302708	416.308892	8.155914528
591.208892	0.673505682	529.417483	2.401068362	416.408892	8.157102712
592.909512	0.677035007	529.984357	2.409240044	416.508892	8.158165186
593.192948	0.677837401	530.61076	2.392843588	416.608892	8.158899377
594.417483	0.681437135	531.208892	2.404069258	416.708892	8.158780511
594.984357	0.68310455	532.909512	2.418578327	416.808892	8.156201373
595.61076	0.684906971	533.192948	2.422534356	416.908892	8.144555765
596.208892	0.686542186	534.417483	2.437409508	417.008892	8.103177721
597.909512	0.689549555	534.984357	2.444770214	417.108892	8.076912408
598.192948	0.690561277	535.61076	2.452226084	417.208892	8.106243968
599.417483	0.696514942	536.208892	2.437095629	417.308892	8.137743905
599.984357	0.698590326	537.909512	2.452277162	417.909512	8.106401142
600.61076	0.700707163	538.192948	2.458089679	418.192948	8.098276618
601.208892	0.702662016	539.417483	2.473599403	419.417483	8.190390757
602.909512	0.708093041	539.984357	2.479909426	419.984357	8.204410455
603.192948	0.708986283	540.61076	2.487111236	420.61076	8.217437679
604.417483	0.712800216	541.208892	2.494901635	421.208892	8.228333461

Final State: j'=5		Final State: j'=4		Final State: j'=3	
E(cm⁻¹)	σ (Å²)	E(cm⁻¹)	σ (Å²)	E(cm⁻¹)	σ (Å²)
604.984357	0.71454005	542.909512	2.500522127	422.909512	8.242983604
605.61076	0.716455981	543.192948	2.480140448	423.192948	8.248022579
606.208892	0.718302969	544.417483	2.510457447	424.417483	8.275244515
607.909512	0.723759847	544.984357	2.518260847	424.984357	8.285998106
608.192948	0.724688139	545.61076	2.526031021	425.61076	8.25088008
609.417483	0.728700208	546.208892	2.533518873	426.208892	8.27451564
609.984357	0.730550769	547.909512	2.538772497	427.909512	8.338426616
610.61076	0.732588827	548.192948	2.540682236	428.192948	8.34340809
611.208892	0.734528639	549.417483	2.530355751	429.417483	8.365269582
612.909512	0.740015174	549.984357	2.527981488	429.984357	8.375498305
613.192948	0.740926212	550.61076	2.551236848	430.61076	8.386779431
614.417483	0.744855249	551.208892	2.54204564	431.208892	8.397474781
614.984357	0.746672154	552.909512	2.58169344	432.909512	8.426176972
615.61076	0.748679994	553.192948	2.581777028	433.192948	8.429970072
616.208892	0.750598245	554.417483	2.57361382	434.417483	8.447666496
617.909512	0.75606121	554.984357	2.591331265	434.984357	8.460178153
618.192948	0.756972976	555.61076	2.607462128	435.61076	8.472901918
619.417483	0.76091501	556.208892	2.610019128	436.208892	8.484285994
619.984357	0.762740926	557.909512	2.595399207	437.909512	8.515231703
620.61076	0.76475891	558.192948	2.6065455	438.192948	8.5202969
621.208892	0.766685889	559.417483	2.635321996	439.417483	8.541986178
622.909512	0.772163692	559.984357	2.604045881	439.984357	8.551914324
623.192948	0.773076419	560.61076	2.606027065	440.61076	8.562788066
624.417483	0.777018822	561.208892	2.630180507	441.208892	8.573078379
624.984357	0.778843459	562.909512	2.662920921	442.909512	8.602284278
625.61076	0.780859471	563.192948	2.669627851	443.192948	8.607204106
626.208892	0.782784331	564.417483	2.635921069	444.417483	8.628586486
627.909512	0.78825678	564.984357	2.655281886	444.984357	8.638500123
628.192948	0.789168863	565.61076	2.666492191	445.61076	8.649436562
629.417483	0.793109514	566.208892	2.669212013	446.208892	8.659852763
629.984357	0.794933872	567.909512	2.705394653	447.909512	8.689302324
630.61076	0.79694991	568.192948	2.67873063	448.192948	8.694186037
631.208892	0.798875045	569.417483	2.69936518	449.417483	8.715206753
632.909512	0.804349015	569.984357	2.677532618	449.984357	8.724897219
633.192948	0.805261387	570.61076	2.699529631	450.61076	8.73557861
634.417483	0.809203198	571.208892	2.722515472	451.208892	8.745754588
634.984357	0.81102801	572.909512	2.700888053	452.909512	8.774584939
635.61076	0.81304447	573.192948	2.703996864	453.192948	8.779377092
636.208892	0.81496993	574.417483	2.72054193	454.417483	8.800040535
637.909512	0.820444482	574.984357	2.739458902	454.984357	8.809583467
638.192948	0.821356914	575.61076	2.744382297	455.61076	8.820110927
639.417483	0.825298954	576.208892	2.732412258	456.208892	8.830145232
639.984357	0.827123866	577.909512	2.73485288	457.909512	8.858573549

Final State: j’=5		Final State: j’=4		Final State: j’=3	
E(cm⁻¹)	σ (Å²)	E(cm⁻¹)	σ (Å²)	E(cm⁻¹)	σ (Å²)
640.61076	0.829140449	578.192948	2.738143027	458.192948	8.863296483
641.208892	0.83106605	579.417483	2.761447938	459.417483	8.883650356
642.909512	0.836541166	579.984357	2.767897135	459.984357	8.893044681
643.192948	0.837453718	580.61076	2.774938759	460.61076	8.90340482
644.417483	0.841396366	581.208892	2.781219235	461.208892	8.913277213
644.984357	0.843221611	582.909512	2.777356126	462.909512	8.94123988
645.61076	0.845238594	583.192948	2.781857518	463.192948	8.945885128
646.208892	0.847164607	584.417483	2.794366776	464.417483	8.96590474
647.909512	0.852641045	584.984357	2.799251465	464.984357	8.975145477
648.192948	0.853553843	585.61076	2.80443844	465.61076	8.985336846
649.417483	0.85749761	586.208892	2.808879916	466.208892	8.995048923
649.984357	0.859323409	587.909512	2.819713663	467.909512	9.022559682
650.61076	0.861341034	588.192948	2.821985425	468.192948	9.027130023
651.208892	0.863267691	589.417483	2.833398215	469.417483	9.046826729
652.909512	0.868746136	589.984357	2.832077595	469.984357	9.055918227
653.192948	0.869659291	590.61076	2.834728418	470.61076	9.065944811
654.417483	0.873604713	591.208892	2.841865875	472.909512	9.102563217
654.984357	0.875431339	592.909512	2.855048905	473.192948	9.10705907
655.61076	0.877449924	593.192948	2.857135309	474.417483	9.126434301
656.208892	0.879377541	594.417483	2.866119886	474.984357	9.135377151
657.909512	0.88485898	594.984357	2.870277442	475.61076	9.145239671
658.192948	0.885772677	595.61076	2.874877131	476.208892	9.154638056
659.417483	0.88972057	596.208892	2.879282305	477.909512	9.181258546
659.984357	0.891548423	597.909512	2.891330949	479.417483	9.20473889
660.61076	0.893568426	598.192948	2.893000024	479.984357	9.213535475
661.208892	0.895497462	599.417483	2.901429668	480.61076	9.223236751
662.909512	0.90098331	599.984357	2.905591902	481.208892	9.232481589
663.192948	0.901897796	600.61076	2.910166021	482.909512	9.258667895
664.417483	0.905849299	601.208892	2.91451148	483.192948	9.263018125
664.984357	0.907678931	602.909512	2.926803612	484.417483	9.281766438
665.61076	0.909700989	603.192948	2.928847168	484.984357	9.290420334
666.208892	0.911632077	604.417483	2.937661737	485.61076	9.299964566
667.909512	0.917124264	604.984357	2.941729619	486.208892	9.309060025
668.192948	0.918039886	605.61076	2.946208073	487.909512	9.334825193
669.417483	0.921996569	606.208892	2.950465235	488.192948	9.339105796
669.984357	0.923828762	607.909512	2.962526854	489.417483	9.357555328
670.61076	0.925853766	608.192948	2.96453987	489.984357	9.366072121
671.208892	0.9277878	609.417483	2.973251895	490.61076	9.375465844
672.909512	0.93328908	609.984357	2.97729041	491.208892	9.384418584
673.192948	0.934206327	610.61076	2.981754523	492.909512	9.409784169
674.417483	0.938170436	611.208892	2.986017763	493.192948	9.413999166
674.984357	0.940006297	612.909512	2.998135372	494.417483	9.432168884
675.61076	0.942035541	613.192948	3.000153814	494.984357	9.440558399

Final State: j'=5		Final State: j'=4		Final State: j'=3	
E(cm⁻¹)	σ (Å²)	E(cm⁻¹)	σ (Å²)	E(cm⁻¹)	σ (Å²)
676.208892	0.943973809	614.417483	3.008868298	495.61076	9.449813287
677.909512	0.949488212	614.984357	3.012898858	496.208892	9.458635469
678.192948	0.950407815	615.61076	3.017349883	497.909512	9.483641924
679.417483	0.954382711	616.208892	3.021597387	498.192948	9.487799082
679.984357	0.95622392	617.909512	3.033662074	499.417483	9.505726775
680.61076	0.958259358	618.192948	3.035671437	499.984357	9.514009189
681.208892	0.96020383	619.417483	3.044348759	500.61076	9.523149921
682.909512	0.965737633	619.984357	3.048363841	501.208892	9.531867762
683.192948	0.966660744	620.61076	3.052799213	502.909512	9.556608267
684.417483	0.970651814	621.208892	3.05703307	503.192948	9.560726459
684.984357	0.972501118	622.909512	3.069063443	504.417483	9.578508879
685.61076	0.974545995	623.192948	3.071067369	504.984357	9.586739213
686.208892	0.976500003	624.417483	3.079721	505.61076	9.595836628
687.909512	0.982064074	624.984357	3.083724743	506.208892	9.604529749
688.192948	0.982992743	625.61076	3.0881472	507.909512	9.629326941
689.417483	0.987009791	626.208892	3.092368335	508.192948	9.633480003
689.984357	0.98887233	627.909512	3.104360474	509.417483	9.651547537
690.61076	0.990932844	628.192948	3.106357794	509.984357	9.660016939
691.208892	0.99290288	629.417483	3.114982309	510.61076	9.669505649
692.909512	0.998519571	629.984357	3.118972367	511.208892	9.678765392
693.192948	0.999458205	630.61076	3.123379608	512.909512	9.708326213
694.417483	1.003523199	631.208892	3.127586146	513.192948	9.714861226
694.984357	1.005411083	632.909512	3.139536682	514.417483	9.752772678
695.61076	1.007502862	633.192948	3.141527062	514.984357	9.737634261
696.208892	1.009505066	634.417483	3.150121515	515.61076	9.750505789
697.909512	1.015237127	634.984357	3.154097618	516.208892	9.767596033
698.192948	1.016199216	635.61076	3.158489394	517.909512	9.793914943
699.417483	1.020385483	636.208892	3.162681098	518.192948	9.799787502
699.984357	1.022343875	637.909512	3.174589046	519.417483	9.796979243
700.61076	1.024527951	638.192948	3.17657225	519.984357	9.809482494
701.208892	1.026638605	639.417483	3.185135519	520.61076	9.804639586
702.909512	1.032867728	639.984357	3.189097025	521.208892	9.825358969
703.192948	1.033962519	640.61076	3.193472571	522.909512	9.84860257
704.417483	1.039182746	641.208892	3.197648685	523.192948	9.864776977
704.984357	1.042308676	642.909512	3.209511775	524.984357	9.869706531
705.61076	1.043813451	643.192948	3.211487431	525.61076	9.903760683
706.208892	1.041241347	644.417483	3.220017833	526.208892	9.885200895
707.909512	1.044667052	644.984357	3.223964014	527.909512	9.888476555
708.192948	1.04593063	645.61076	3.228322522	528.192948	9.894669427
709.417483	1.055249669	646.208892	3.232482281	529.417483	9.92182951
709.984357	1.054685122	647.909512	3.244298404	529.984357	9.943571625
710.61076	1.053942441	648.192948	3.24626617	530.61076	9.95704543
711.208892	1.055726366	649.417483	3.254762246	531.208892	9.944100727

Final State: j'=5		Final State: j'=4		Final State: j'=3	
E(cm⁻¹)	σ (Å²)	E(cm⁻¹)	σ (Å²)	E(cm⁻¹)	σ (Å²)
712.909512	1.066995965	649.984357	3.258692416	532.909512	9.951852427
713.192948	1.06731431	650.61076	3.263033152	533.192948	9.957487677
714.417483	1.06883688	651.208892	3.267175837	534.417483	9.981046819
714.984357	1.064557611	652.909512	3.278942952	534.984357	9.994439439
715.61076	1.070558227	653.192948	3.280902481	536.208892	10.02129815
716.208892	1.074768363	654.417483	3.289362745	537.909512	10.00883793
717.909512	1.075802496	654.984357	3.293276213	538.192948	10.01570173
718.192948	1.077080813	655.61076	3.297598397	539.417483	10.03867701
719.417483	1.080862491	656.208892	3.301723292	539.984357	10.04867343
719.984357	1.08467436	657.909512	3.313439324	540.61076	10.06048347
720.61076	1.08835789	658.192948	3.315390271	541.208892	10.07446962
721.208892	1.092415514	659.417483	3.323813243	542.909512	10.09648138
722.909512	1.0952943	659.984357	3.327709324	543.192948	10.11332523
723.192948	1.088765644	660.61076	3.332012215	544.417483	10.09707368
724.417483	1.097701717	661.208892	3.336118595	544.984357	10.10872218
724.984357	1.100937525	662.909512	3.347781556	545.61076	10.12090019
725.61076	1.104215767	663.192948	3.349723591	546.208892	10.13327185
726.208892	1.107526173	664.417483	3.358107873	547.909512	10.19473448
727.909512	1.107074286	664.984357	3.361985932	548.192948	10.18786839
728.192948	1.110008358	665.61076	3.366268827	549.417483	10.2001565
729.417483	1.112105304	666.208892	3.370356044	549.984357	10.18718665
729.984357	1.116355712	667.909512	3.381964122	550.61076	10.18485375
730.61076	1.119693322	668.192948	3.383896954	551.208892	10.22659491
731.208892	1.12262413	669.417483	3.392241304	552.909512	10.22293149
732.909512	1.132279693	669.984357	3.396100789	553.192948	10.25169807
733.192948	1.135053394	670.61076	3.400363091	554.417483	10.2156459
734.417483	1.133360807	671.208892	3.404430582	554.984357	10.23114103
734.984357	1.131737446	672.909512	3.41598229	555.61076	10.25736121
735.61076	1.134734459	673.192948	3.41790569	556.208892	10.33198364
736.208892	1.138951193	674.417483	3.426209175	557.909512	10.23703223
737.909512	1.148573177	674.984357	3.43004968	558.192948	10.24912108
738.192948	1.150434085	675.61076	3.434290974	559.417483	10.30332016
739.417483	1.154440624	676.208892	3.438338382	559.984357	10.35197316
739.984357	1.156165464	677.909512	3.449832862	560.61076	10.27117295
740.61076	1.155383022	678.192948	3.451746714	561.208892	10.28533081
741.208892	1.153223131	679.417483	3.460008953	562.909512	10.33170482
742.909512	1.155178912	679.984357	3.463830383	563.192948	10.34578191
743.192948	1.153063092	680.61076	3.468050624	564.417483	10.3331854
744.417483	1.169528384	681.208892	3.472077947	564.984357	10.35669732
744.984357	1.170449605	682.909512	3.48351562	565.61076	10.3526709
745.61076	1.158664345	683.192948	3.485420061	566.208892	10.37947773
746.208892	1.169170486	684.417483	3.493641882	567.909512	10.43032177
747.909512	1.181801853	684.984357	3.497444752	568.192948	10.47805702

Final State: j'=5		Final State: j'=4		Final State: j'=3	
E(cm⁻¹)	σ (Å²)	E(cm⁻¹)	σ (Å²)	E(cm⁻¹)	σ (Å²)
748.192948	1.168044326	685.61076	3.501644644	569.417483	10.38520058
749.417483	1.173669937	686.208892	3.505652713	569.984357	10.45327973
749.984357	1.181651916	687.909512	3.517036782	570.61076	10.3931608
750.61076	1.188345368	688.192948	3.518932486	571.208892	10.41644594
751.208892	1.194432982	689.417483	3.527117433	572.909512	10.4979049
752.909512	1.190641496	689.984357	3.530903788	573.192948	10.44884919
753.192948	1.193087966	690.61076	3.535085908	574.417483	10.42354756
754.417483	1.203548639	691.208892	3.539077552	574.984357	10.4574756
754.984357	1.212042013	692.909512	3.550418609	575.61076	10.46845387
755.61076	1.200723758	693.192948	3.552307795	576.208892	10.47506461
756.208892	1.195446302	694.417483	3.560467377	577.909512	10.50780207
757.909512	1.209883425	694.984357	3.564243847	578.192948	10.48794935
758.192948	1.210634401	695.61076	3.568417487	579.417483	10.51378481
759.417483	1.225501069	696.208892	3.572401561	579.984357	10.5248147
759.984357	1.20347908	697.909512	3.583737708	580.61076	10.53774496
760.61076	1.211444226	698.192948	3.585628862	581.208892	10.55394909
761.208892	1.225704248	699.417483	3.593810546	582.909512	10.54617342
762.909512	1.2326535	699.984357	3.597607295	583.192948	10.55090261
763.192948	1.231835838	700.61076	3.601813263	584.417483	10.56863664
764.417483	1.22662558	701.208892	3.605843999	584.984357	10.57604294
764.984357	1.225349029	702.909512	3.617460024	585.61076	10.58429488
765.61076	1.220092602	703.192948	3.619439689	586.208892	10.5922752
766.208892	1.232597194	704.417483	3.628465774	587.909512	10.60893448
767.909512	1.233756398	704.984357	3.633711037	588.192948	10.61192875
768.192948	1.24160676	705.61076	3.640831142	589.417483	10.62839762
769.417483	1.240166632	706.208892	3.643305966	589.984357	10.62980755
769.984357	1.243665126	707.909512	3.651761036	590.61076	10.63119029
770.61076	1.248039589	708.192948	3.651135419	591.208892	10.63941322
771.208892	1.251502626	709.417483	3.665207901	592.909512	10.65839533
772.909512	1.257414845	709.984357	3.669234625	593.192948	10.66133915
773.192948	1.25926141	710.61076	3.668356637	594.417483	10.67392194
774.417483	1.258004133	711.208892	3.671243035	594.984357	10.67971841
774.984357	1.261377397	712.909512	3.686262413	595.61076	10.68612456
775.61076	1.264144551	713.192948	3.691560568	596.208892	10.6922626
776.208892	1.266519711	714.417483	3.695438166	597.909512	10.70936701
777.909512	1.2727191	714.984357	3.693673781	598.192948	10.71170052
778.192948	1.273511236	715.61076	3.69781384	599.417483	10.72289577
779.417483	1.27705386	716.208892	3.703081377	599.984357	10.72848296
779.984357	1.279057449	717.909512	3.716661947	600.61076	10.73465236
780.61076	1.281509262	718.192948	3.717885913	601.208892	10.7405176
782.909512	1.287740905	719.417483	3.720834695	602.909512	10.75706681
783.192948	1.2887625	719.984357	3.725391238	603.192948	10.75980952
784.417483	1.292890791	720.61076	3.730374144	604.417483	10.77160915

Final State: j'=5		Final State: j'=4		Final State: j'=3	
E(cm⁻¹)	σ (Å²)	E(cm⁻¹)	σ (Å²)	E(cm⁻¹)	σ (Å²)
784.984357	1.294756241	721.208892	3.735851443	604.984357	10.77703759
785.61076	1.296812513	722.909512	3.750766858	605.61076	10.7830005
786.208892	1.29877576	723.192948	3.749112155	606.208892	10.78865426
787.909512	1.30437781	724.417483	3.752548394	607.909512	10.80458643
788.192948	1.305317624	724.984357	3.756843132	608.192948	10.80723291
789.417483	1.309187746	725.61076	3.761502753	609.417483	10.81864846
789.984357	1.310770934	726.208892	3.766150502	609.984357	10.82392073
790.61076	1.312742797	727.909512	3.780890262	610.61076	10.82973304
791.208892	1.314707337	728.192948	3.779574308	611.208892	10.8352704
792.909512	1.320270713	729.417483	3.783041134	612.909512	10.85093531
793.192948	1.321193528	729.984357	3.787659329	613.192948	10.85353398
794.417483	1.325174173	730.61076	3.792251355	614.417483	10.86471881
794.984357	1.32701482	731.208892	3.796528845	614.984357	10.86987295
795.61076	1.329047314	732.909512	3.809877884	615.61076	10.87555088
796.208892	1.330985676	733.192948	3.813533073	616.208892	10.88095569
797.909512	1.336469742	734.417483	3.822003706	617.909512	10.89623556
798.192948	1.337379988	734.984357	3.822116099	618.192948	10.89877002
799.417483	1.341314195	735.61076	3.822806678	619.417483	10.90968083
799.984357	1.343139351	736.208892	3.827681683	619.984357	10.91471056
800.61076	1.345159074	737.909512	3.840924673	620.61076	10.92025294
801.208892	1.34708976	738.192948	3.84350321	621.208892	10.92552989
802.909512	1.352585484	739.417483	3.859514453	622.909512	10.94045213
803.192948	1.353501943	739.984357	3.858087824	623.192948	10.94292739
804.417483	1.357461974	740.61076	3.869962143	624.417483	10.9535826
804.984357	1.359295268	741.208892	3.870795989	624.984357	10.95849387
805.61076	1.36132097	742.909512	3.878530815	625.61076	10.96390518
806.208892	1.363255052	743.192948	3.872937899	626.208892	10.96905688
807.909512	1.368753065	744.417483	3.882295037	627.909512	10.98362235
808.192948	1.369669332	744.984357	3.896435824	628.192948	10.98603817
809.417483	1.373628099	745.61076	3.886726239	629.417483	10.99643682
809.984357	1.375460979	746.208892	3.887771751	629.984357	11.00122956
810.61076	1.377486608	747.909512	3.921580878	630.61076	11.00651013
811.208892	1.379421109	748.192948	3.917961242	631.208892	11.01153726
812.909512	1.384922972	749.417483	3.903820233	632.909512	11.02574991
813.192948	1.385840184	749.984357	3.910045438	633.192948	11.02810712
814.417483	1.389803529	750.61076	3.917094535	634.417483	11.0382532
814.984357	1.391638641	751.208892	3.93431437	634.984357	11.04292936
815.61076	1.393666718	752.909512	3.926727103	635.61076	11.04808132
816.208892	1.395603496	753.192948	3.929196173	636.208892	11.05298582
817.909512	1.401111355	754.417483	3.940597871	637.909512	11.06685093
818.192948	1.402029496	754.984357	3.951640022	638.192948	11.06915036
819.417483	1.405996703	755.61076	3.956330913	639.417483	11.07904734
819.984357	1.407833545	756.208892	3.94951906	639.984357	11.08360841

Final State: j’=5		Final State: j’=4		Final State: j’=3	
E(cm⁻¹)	σ (Å²)	E(cm⁻¹)	σ (Å²)	E(cm⁻¹)	σ (Å²)
820.61076	1.409863512	757.909512	3.964501464	640.61076	11.0886334
821.208892	1.411802087	758.192948	3.964619007	641.208892	11.09341689
823.192948	1.418234198	759.417483	3.981341111	642.909512	11.10693893
824.984357	1.42404404	759.984357	3.982675619	643.192948	11.10918133
825.61076	1.426076097	760.61076	3.970868908	644.417483	11.11883245
826.208892	1.428016699	761.208892	3.986318976	644.984357	11.12328
828.192948	1.434455631	762.909512	3.991927082	645.61076	11.12817976
829.984357	1.440271728	763.192948	4.010045043	646.208892	11.13284383
830.61076	1.442305988	764.417483	4.012395672	647.909512	11.14602751
831.208892	1.444248689	764.984357	4.002330287	648.192948	11.14821372
832.909512	1.449773616	765.61076	4.010271479	649.417483	11.15762239
833.192948	1.450694637	766.208892	4.004082032	649.984357	11.16195798
834.417483	1.4546744	767.909512	4.024375317	650.61076	11.16673428
834.984357	1.456517115	768.192948	4.02194642	651.208892	11.17128062
835.61076	1.458553615	769.417483	4.034021891	652.909512	11.18413068
836.208892	1.460498467	769.984357	4.032813968	653.192948	11.18626143
837.909512	1.466029537	770.61076	4.036935752	654.417483	11.19543103
838.192948	1.466951588	771.208892	4.041208879	654.984357	11.19965626
839.417483	1.470935817	772.909512	4.054272699	655.61076	11.20431073
839.984357	1.472780605	773.192948	4.05861828	656.208892	11.20874101
840.61076	1.474819403	774.417483	4.059309968	657.909512	11.22126205
841.208892	1.476766455	774.984357	4.0629014	658.192948	11.2233381
843.192948	1.4832269	775.61076	4.066814573	659.417483	11.23227191
844.417483	1.487215663	776.208892	4.070477387	659.984357	11.23638824
844.984357	1.48906256	777.909512	4.080972981	660.61076	11.2409226
845.61076	1.491103681	778.192948	4.082651502	661.208892	11.24523835
846.208892	1.493052952	779.417483	4.089397827	662.909512	11.25743477
847.909512	1.498596621	779.984357	4.092626772	663.192948	11.25945686
848.192948	1.499520778	780.61076	4.096350337	664.417483	11.26815803
849.417483	1.503514097	781.208892	4.100690331	664.984357	11.27216689
849.984357	1.5053631	782.909512	4.108852183	665.61076	11.27658265
850.61076	1.507406551	783.192948	4.110505898	666.208892	11.28078537
851.208892	1.50935805	784.417483	4.11749829	667.909512	11.29266128
852.909512	1.514908037	784.984357	4.120698777	668.192948	11.29463005
853.192948	1.515833241	785.61076	4.124224763	669.417483	11.30310132
854.417483	1.519831103	786.208892	4.127585618	669.984357	11.30700397
854.984357	1.521682203	787.909512	4.137132417	670.61076	11.31130254
855.61076	1.523727969	788.192948	4.138727818	671.208892	11.31539347
856.208892	1.525681673	789.417483	4.145630707	672.909512	11.32695225
857.909512	1.531237913	789.984357	4.148691617	673.192948	11.32886829
858.192948	1.532164157	790.61076	4.152073981	674.417483	11.33711195
859.417483	1.53616649	791.208892	4.155350779	674.984357	11.34090938
859.984357	1.538019658	792.909512	4.164700053	675.61076	11.34509176

Final State: j’=5		Final State: j’=4		Final State: j’=3	
E(cm⁻¹)	σ (Å²)	E(cm⁻¹)	σ (Å²)	E(cm⁻¹)	σ (Å²)
860.61076	1.540067707	793.192948	4.166255646	676.208892	11.34907186
861.208892	1.542023574	794.417483	4.172963444	677.909512	11.36031589
862.909512	1.547585946	794.984357	4.176061631	678.192948	11.3621795
863.192948	1.548513206	795.61076	4.179480083	679.417483	11.3701968
864.417483	1.552519918	796.208892	4.182739135	679.984357	11.37388946
865.61076	1.556425362	797.909512	4.191970547	680.61076	11.37795608
866.208892	1.558383345	798.192948	4.193502911	681.208892	11.38182562
867.909512	1.563951672	799.417483	4.200103992	682.909512	11.39275505
868.192948	1.564879918	799.984357	4.203151049	683.192948	11.39456621
869.417483	1.568890861	800.61076	4.206512556	684.417483	11.40235644
869.984357	1.570747988	801.208892	4.209717081	684.984357	11.40594373
870.61076	1.572800392	802.909512	4.218799748	685.61076	11.40989374
871.208892	1.574760405	803.192948	4.22030933	686.208892	11.41365169
872.909512	1.580334446	804.417483	4.226817091	687.909512	11.42426214
873.192948	1.581263636	804.984357	4.229821824	688.192948	11.42601977
874.417483	1.585278623	805.61076	4.233136176	689.417483	11.43357764
874.984357	1.587137603	806.208892	4.236295049	689.984357	11.43705657
875.61076	1.589192044	807.909512	4.245244427	690.61076	11.44088604
876.208892	1.591153988	808.192948	4.246731341	691.208892	11.44452803
877.909512	1.596733446	809.417483	4.253139939	692.909512	11.45480301
879.417483	1.601682335	809.984357	4.25609825	693.192948	11.45650374
879.984357	1.603543064	810.61076	4.259361053	694.417483	11.46381123
881.208892	1.607563181	811.208892	4.26247053	694.984357	11.46717134
882.909512	1.61314772	812.909512	4.271279189	695.61076	11.47086911
883.192948	1.614078636	813.192948	4.272742656	696.208892	11.47437781
884.417483	1.618101017	814.417483	4.279050011	697.909512	11.48425769
885.61076	1.622021522	814.984357	4.281961438	698.192948	11.48588828
886.208892	1.623986974	815.61076	4.285172386	699.417483	11.49287204
887.909512	1.629576241	816.208892	4.288232319	699.984357	11.49606717
888.192948	1.630507937	817.909512	4.296899617	700.61076	11.49956458
889.417483	1.634533629	818.192948	4.298339442	701.208892	11.50286553
890.61076	1.638457305	819.417483	4.304544364	702.909512	11.51194098
891.208892	1.640424322	819.984357	4.307408204	703.192948	11.5133823
892.909512	1.646017972	820.61076	4.310566462	704.417483	11.51902032
893.192948	1.646950384	821.208892	4.313575948	704.984357	11.52070533
894.417483	1.650979151	822.909512	4.322099282	705.61076	11.52475769
894.984357	1.65284443	823.192948	4.323515034	706.208892	11.53360109
895.61076	1.65490577	824.417483	4.329615714	707.909512	11.54602735
896.208892	1.65687425	824.984357	4.332431191	708.192948	11.54694531
897.909512	1.66247199	825.61076	4.335535898	709.417483	11.54621846
898.192948	1.663405076	826.208892	4.338494186	709.984357	11.55343377
899.984357	1.669303357	827.909512	4.346871465	710.61076	11.55995726
900.61076	1.671366171	828.192948	4.348262811	711.208892	11.56280087

Final State: j'=5		Final State: j'=4		Final State: j'=3	
E(cm⁻¹)	σ (Å²)	E(cm⁻¹)	σ (Å²)	E(cm⁻¹)	σ (Å²)
901.208892	1.673336053	829.417483	4.35425786	712.909512	11.56667496
902.909512	1.678937794	829.984357	4.357024308	713.192948	11.56936163
903.192948	1.679871552	830.61076	4.36007477	714.417483	11.57673955
904.417483	1.683906144	831.208892	4.362981165	714.984357	11.58797182
904.984357	1.685774133	832.909512	4.371210499	715.61076	11.58672723
906.208892	1.68980987	833.192948	4.372577126	716.208892	11.58765158
909.417483	1.700388677	834.417483	4.37846516	717.909512	11.59947595
909.984357	1.702258395	834.984357	4.381181968	718.192948	11.6011635
910.61076	1.704324749	835.61076	4.384177498	719.417483	11.60910914
911.208892	1.706298153	836.208892	4.387031363	719.984357	11.61007473
912.909512	1.711910856	837.909512	4.395110934	720.61076	11.61175185
913.192948	1.71284662	838.192948	4.396452539	721.208892	11.61293652
914.417483	1.716890656	839.417483	4.402232316	722.909512	11.62234711
914.984357	1.718763557	840.61076	4.407838864	723.192948	11.63469161
915.61076	1.720833839	841.208892	4.410639598	724.417483	11.63443542
916.208892	1.722811484	842.909512	4.418567752	724.984357	11.63595025
917.909512	1.728440042	843.192948	4.419884073	725.61076	11.63796556
918.192948	1.729379214	844.417483	4.425554422	726.208892	11.63978062
919.417483	1.733442038	844.984357	4.42817028	727.909512	11.65336634
919.984357	1.73532684	845.61076	4.431054095	728.192948	11.65289968
920.61076	1.737414017	846.208892	4.433801152	729.417483	11.66152018
921.208892	1.73941333	847.909512	4.441576373	729.984357	11.6618848
922.909512	1.74518364	848.192948	4.442867164	730.61076	11.66373186
923.192948	1.746180344	849.417483	4.44842703	731.208892	11.66577049
924.417483	1.751508932	849.984357	4.450991641	732.909512	11.67076037
926.208892	1.75730623	850.61076	4.453818797	733.192948	11.67096408
927.909512	1.761746034	851.208892	4.4565117	734.417483	11.68013083
928.192948	1.763498422	852.909512	4.464132595	734.984357	11.68799125
929.417483	1.76686681	853.192948	4.465397616	735.61076	11.68914718
929.984357	1.769006562	854.417483	4.470846056	736.208892	11.68974978
930.61076	1.770671189	854.984357	4.473359012	737.909512	11.69375226
931.208892	1.772372377	855.61076	4.476129023	738.192948	11.69407975
932.909512	1.777918039	856.208892	4.478767302	739.417483	11.6979368
933.192948	1.779487702	857.909512	4.486232656	739.984357	11.70454745
934.417483	1.78258151	858.192948	4.4874717	740.61076	11.71011379
934.984357	1.784665985	859.417483	4.492807838	741.208892	11.71348454
935.61076	1.787405505	859.984357	4.495268737	742.909512	11.72557255
939.417483	1.799113429	860.61076	4.497981178	743.192948	11.73124936
939.984357	1.801235412	861.208892	4.500564436	744.417483	11.72468827
940.61076	1.805056453	862.909512	4.507873093	744.984357	11.72723684
941.208892	1.806802652	863.192948	4.509086013	745.61076	11.74585472
942.909512	1.810179872	864.417483	4.514309056	746.208892	11.74035955
943.192948	1.811189184	864.984357	4.516717543	747.909512	11.74549101

Final State: j'=5		Final State: j'=4		Final State: j'=3	
E(cm⁻¹)	σ (Å²)	E(cm⁻¹)	σ (Å²)	E(cm⁻¹)	σ (Å²)
944.417483	1.815438886	865.61076	4.519372026	748.192948	11.75717838
944.984357	1.817428717	866.208892	4.521899903	749.417483	11.7599584
945.61076	1.819803303	867.909512	4.529050873	749.984357	11.75642634
947.909512	1.827311265	868.192948	4.530237472	750.61076	11.75471875
948.192948	1.827460599	869.417483	4.535346735	751.208892	11.75102926
949.417483	1.831750618	869.984357	4.537702499	752.909512	11.77060953
949.984357	1.833688866	870.61076	4.540298692	753.192948	11.77035648
950.61076	1.835837072	871.208892	4.542770854	754.417483	11.7697463
951.208892	1.837925251	872.909512	4.549763248	754.984357	11.76639445
952.909512	1.844911442	873.192948	4.550923394	755.61076	11.79070844
953.192948	1.845756212	874.417483	4.555918258	756.208892	11.7885158
954.417483	1.848077009	874.984357	4.558221031	757.909512	11.78771117
954.984357	1.850119246	875.61076	4.560758607	758.192948	11.78920873
955.61076	1.852319585	876.208892	4.563174778	759.417483	11.78556778
956.208892	1.854434328	877.909512	4.570007807	759.984357	11.81502251
957.909512	1.861957207	878.192948	4.571141356	760.61076	11.8081159
958.192948	1.864021064	879.417483	4.576021288	761.208892	11.7961266
959.417483	1.869258335	879.984357	4.578270796	762.909512	11.80484018
959.984357	1.869600566	880.61076	4.580749489	763.192948	11.80067751
960.61076	1.869257397	881.208892	4.583109426	764.417483	11.82705011
962.909512	1.876215589	882.909512	4.589782351	764.984357	11.82449066
963.192948	1.877619817	883.192948	4.590889195	765.61076	11.83183709
964.417483	1.882724018	884.417483	4.595653676	766.208892	11.82807585
964.984357	1.882466508	884.984357	4.597849686	767.909512	11.83976251
965.61076	1.884981041	885.61076	4.600269256	768.192948	11.83311662
966.208892	1.8881018	886.208892	4.602572696	769.417483	11.84671874
968.192948	1.893035939	887.909512	4.609084824	769.984357	11.84343426
969.417483	1.897436999	888.192948	4.610164858	770.61076	11.84394339
970.61076	1.904445205	889.417483	4.614813394	771.208892	11.84521272
971.208892	1.902262947	889.984357	4.616955699	772.909512	11.85258169
972.909512	1.90860391	890.61076	4.619315871	773.192948	11.8519681
973.192948	1.90974132	891.208892	4.621562571	774.417483	11.86128429
974.417483	1.918302462	892.909512	4.627913171	774.984357	11.86221341
974.984357	1.916658769	893.192948	4.628966244	775.61076	11.86419233
975.61076	1.918351781	894.417483	4.633498274	776.208892	11.86630343
976.208892	1.920072597	894.984357	4.635586589	777.909512	11.87296755
977.909512	1.926995934	895.61076	4.637887057	778.192948	11.87429143
978.192948	1.927687432	896.208892	4.640076683	779.417483	11.87941258
979.417483	1.928925226	897.909512	4.646264869	779.984357	11.88151152
979.984357	1.9324604	898.192948	4.647290821	780.61076	11.88381032
980.61076	1.936525436	899.417483	4.651705575	781.208892	11.88789636
981.208892	1.935005596	899.984357	4.65373952	782.909512	11.89315829
982.909512	1.943665217	900.61076	4.65597985	783.192948	11.89421276

Final State: j'=5		Final State: j'=4		Final State: j'=3	
E(cm⁻¹)	σ (Å²)	E(cm⁻¹)	σ (Å²)	E(cm⁻¹)	σ (Å²)
983.192948	1.947875527	901.208892	4.658111995	784.417483	11.89888998
984.417483	1.950512573	902.909512	4.664136284	784.984357	11.90105916
984.984357	1.947117377	903.192948	4.66513488	785.61076	11.90344921
985.61076	1.949716716	904.417483	4.669431103	786.208892	11.9057254
986.208892	1.953661304	904.984357	4.671410034	787.909512	11.91218306
988.192948	1.962120691	905.61076	4.673589446	788.192948	11.91326447
989.417483	1.963531052	906.208892	4.675663305	789.417483	11.91814496
989.984357	1.965469222	907.909512	4.681521007	789.984357	11.92035008
990.61076	1.967684291	908.192948	4.682491701	790.61076	11.92261465
991.208892	1.969865043	909.417483	4.68666686	791.208892	11.92478493
993.192948	1.976181038	909.984357	4.688589394	792.909512	11.93103877
994.984357	1.981942175	910.61076	4.690706212	793.192948	11.93208341
996.208892	1.986019389	911.208892	4.692719967	794.417483	11.93659094
999.417483	1.996492433	912.909512	4.698404675	794.984357	11.93867355
999.984357	1.998675828	913.192948	4.699346162	795.61076	11.94097211
1000.61076	2.000639774	914.417483	4.703393514	796.208892	11.94316485
1001.20889	2.002171625	914.984357	4.705255849	797.909512	11.94938524
1003.19295	2.008631318	915.61076	4.707305153	798.192948	11.95041736
1005.61076	2.016423013	916.208892	4.709253296	799.417483	11.95485287
1006.20889	2.018349467	917.909512	4.714742952	799.984357	11.95689458
1007.90951	2.023861349	918.192948	4.715650257	800.61076	11.95914398
1009.41748	2.028679563	919.417483	4.719541411	801.208892	11.96128614
1011.20889	2.034385719	919.984357	4.721324882	802.909512	11.96734978
1012.90951	2.039817128	920.61076	4.723279682	803.192948	11.96835671
1014.98436	2.046438173	921.208892	4.725127019	804.417483	11.97269519
1016.20889	2.05034248	922.909512	4.730194922	804.984357	11.97469719
1017.90951	2.055753004	923.192948	4.730982272	805.61076	11.97690468
1018.19295	2.056653118	924.417483	4.734484273	806.208892	11.97900788
1019.41748	2.06053811	924.984357	4.737672785	807.909512	11.98496193
1020.61076	2.064319254	925.61076	4.739579735	808.192948	11.98595052
1021.20889	2.066212981	926.208892	4.740189363	809.417483	11.99020894
1023.19295	2.07248701	927.909512	4.745965142	809.984357	11.99217345
1024.98436	2.078141223	928.192948	4.746087691	810.61076	11.99433928
1025.61076	2.080115784	929.417483	4.752095722	811.208892	11.99640251
1026.20889	2.081999929	929.984357	4.753365145	812.909512	12.00224352
1027.90951	2.087349785	930.61076	4.755679202	813.192948	12.00321345
1029.41748	2.092084487	931.208892	4.756593436	814.417483	12.00739231
1031.20889	2.097697895	932.909512	4.762544567	814.984357	12.00932057
1036.20889	2.113300016	933.192948	4.762324824	815.61076	12.01144675
1037.90951	2.118584055	934.417483	4.766733655	816.208892	12.01347254
1040.61076	2.126952637	934.984357	4.767872774	817.909512	12.01920885
1041.20889	2.128801523	935.61076	4.768922128	818.192948	12.02016156
1044.98436	2.14043626	936.208892	4.772839971	819.417483	12.02426669

Final State: j'=5		Final State: j'=4		Final State: j'=3	
E(cm⁻¹)	σ (Å²)	E(cm⁻¹)	σ (Å²)	E(cm⁻¹)	σ (Å²)
1045.61076	2.142360582	937.909512	4.778292606	819.984357	12.02616116
1046.20889	2.144196421	939.417483	4.780870514	820.61076	12.02825023
1047.90951	2.149407307	939.984357	4.782072173	821.208892	12.03024081
1049.41748	2.154016871	940.61076	4.783419859	822.909512	12.03587826
1050.61076	2.15765702	941.208892	4.787285237	823.192948	12.03681466
1051.20889	2.159479144	942.909512	4.79179039	824.417483	12.04085
1054.41748	2.169224669	943.192948	4.792264095	824.984357	12.04271249
1056.20889	2.174644102	944.417483	4.795044687	825.61076	12.04476649
1059.98436	2.186013609	944.984357	4.79636001	826.208892	12.04672384
1060.61076	2.187893002	945.61076	4.797660235	827.909512	12.05226814
1061.20889	2.189685693	946.208892	4.799656744	828.192948	12.05318919
1062.90951	2.194772602	947.909512	4.80590499	829.417483	12.05715892
1064.41748	2.199270608	948.192948	4.806501037	829.984357	12.05899138
1066.20889	2.204598395	949.417483	4.808762792	830.61076	12.06101245
1069.41748	2.214097711	949.984357	4.810084726	831.208892	12.06293857
1069.98436	2.215770132	950.61076	4.811547991	832.909512	12.06839542
1070.61076	2.217616109	951.208892	4.812902376	833.192948	12.06930212
1071.20889	2.219376733	952.909512	4.818762758	834.417483	12.07321027
1072.90951	2.224371633	953.192948	4.81806127	834.984357	12.07501454
1074.98436	2.23044344	954.417483	4.822111853	835.61076	12.07700473
1075.61076	2.232271698	954.984357	4.823037789	836.208892	12.07890158
1076.20889	2.234015322	955.61076	4.824303763	837.909512	12.08427645
1079.41748	2.243333093	956.208892	4.825513804	838.192948	12.08516964
1079.98436	2.244972984	957.909512	4.828447818	839.417483	12.08902009
1080.61076	2.246782858	958.192948	4.829115408	839.984357	12.09079796
1081.20889	2.248508854	959.417483	4.833716194	840.61076	12.09275919
1084.98436	2.259353519	959.984357	4.836638836	841.208892	12.09462867
1086.20889	2.262852109	960.61076	4.838431476	842.909512	12.0999268
1089.41748	2.271974895	962.909512	4.843174514	843.192948	12.1008074
1090.61076	2.275351068	963.192948	4.843052487	844.417483	12.10460387
1091.20889	2.277039959	964.417483	4.849955281	844.984357	12.10635711
1094.41748	2.286060378	964.984357	4.849510453	845.61076	12.10829131
1094.98436	2.287647085	965.61076	4.849651628	846.208892	12.11013517
1096.20889	2.291067404	966.208892	4.849489184	847.909512	12.11536167
1104.41748	2.313735878	967.909512	4.858017179	848.192948	12.11623051
1106.20889	2.318621665	968.192948	4.858009011	849.417483	12.11997666
1109.41748	2.327316429	969.417483	4.858326502	849.984357	12.12170687
1111.20889	2.332139123	969.984357	4.857593857	850.61076	12.12361585
1114.98436	2.342227606	970.61076	4.865962606	851.208892	12.12543586
1116.20889	2.345477462	971.208892	4.865153545	852.909512	12.13059561
1119.41748	2.353940561	972.909512	4.866483122	853.192948	12.13145346
1121.20889	2.358632369	973.192948	4.86675497	854.417483	12.13515277
1124.41748	2.36697555	974.417483	4.873152984	854.984357	12.13686155

Final State: j'=5		Final State: j'=4		Final State: j'=3	
E(cm⁻¹)	σ (Å²)	E(cm⁻¹)	σ (Å²)	E(cm⁻¹)	σ (Å²)
1126.20889	2.371599679	974.984357	4.873394086	855.61076	12.13874709
1129.41748	2.379820335	975.61076	4.873078802	856.208892	12.14054489
1134.41748	2.392470919	976.208892	4.87398943	857.909512	12.14564258
1141.20889	2.409335904	977.909512	4.877994073	858.192948	12.14649023
1144.41748	2.417173746	978.192948	4.876870313	859.417483	12.15014601
1144.98436	2.418549685	979.417483	4.883776885	859.984357	12.15183491
1146.20889	2.421512857	979.984357	4.881332517	860.61076	12.15369867
1149.41748	2.429217764	980.61076	4.8860741	861.208892	12.15547588
1154.98436	2.442378208	981.208892	4.886127724	862.909512	12.16051601
1156.20889	2.445236968	982.909512	4.888417881	863.192948	12.16135421
1159.41748	2.452663719	983.192948	4.889249969	864.417483	12.16496961
1166.20889	2.468046202	984.417483	4.894183899	864.984357	12.16664011
1171.20889	2.479160514	984.984357	4.89672127	865.61076	12.16848368
1176.20889	2.490279706	985.61076	4.894718224	866.208892	12.1702418
1179.41748	2.496945195	986.208892	4.89402351	867.909512	12.17522866
1181.20889	2.500946896	987.909512	4.900379796	868.192948	12.17605811
1186.20889	2.511352502	988.192948	4.901923934	869.417483	12.17963619
1191.20889	2.521721911	989.417483	4.90309495	869.984357	12.18128965
1194.41748	2.528646009	989.984357	4.90400747	870.61076	12.1831146
1196.20889	2.531837389	990.61076	4.905141476	871.208892	12.18485508
1201.20889	2.541617585	991.208892	4.906228396	872.909512	12.18979271
1204.41748	2.546901332	992.909512	4.910712377	873.192948	12.19061411
1209.41748	2.557082159	994.417483	4.91309249	874.417483	12.19415777
1211.20889	2.561127585	994.984357	4.914155019	874.984357	12.19579552
1216.20889	2.568972808	995.61076	4.915341874	875.61076	12.19760326
1221.20889	2.579467039	996.208892	4.916490479	876.208892	12.19932752
1224.41748	2.583884993	997.909512	4.919899428	877.909512	12.20421985
1226.20889	2.587203293	998.192948	4.920434906	878.192948	12.2050338
1234.41748	2.60172052	999.417483	4.922707023	879.417483	12.20854579
1236.20889	2.604637982	999.984357	4.923838059	879.984357	12.21016911
1239.41748	2.610077306	1001.20889	4.926202785	880.61076	12.21196106
1241.20889	2.612845069	1002.90951	4.929277666	881.208892	12.21367037
1244.41748	2.617982662	1003.19295	4.92979787	882.909512	12.21852113
1246.20889	2.620778127	1004.41748	4.932032216	883.192948	12.21932826
1249.41748	2.625827571	1004.98436	4.933059154	884.417483	12.22281122
1251.20889	2.62858461	1005.61076	4.93418876	884.984357	12.22442147
1254.41748	2.633464479	1006.20889	4.935262555	885.61076	12.22619875
1256.20889	2.636163622	1008.19295	4.938821252	886.208892	12.22789442
1259.41748	2.64091661	1009.41748	4.940983921	887.909512	12.23270713
1261.20889	2.643537552	1009.98436	4.941963677	888.192948	12.23350807
1264.41748	2.648174006	1010.61076	4.943044822	889.417483	12.23696446
1266.20889	2.650728875	1011.20889	4.944074215	889.984357	12.23856248
1269.41748	2.655244848	1013.19295	4.947459879	890.61076	12.24032676

Final State: j’=5		Final State: j’=4		Final State: j’=3	
E(cm⁻¹)	σ (Å²)	E(cm⁻¹)	σ (Å²)	E(cm⁻¹)	σ (Å²)
1271.20889	2.657733424	1014.41748	4.949524692	891.208892	12.24200997
1276.20889	2.664557324	1014.98436	4.950474203	892.909512	12.24678805
1281.20889	2.671204371	1015.61076	4.951518879	893.192948	12.24758332
1286.20889	2.677679042	1016.20889	4.952511909	894.417483	12.25101564
1291.20889	2.683986008	1017.90951	4.955308746	894.984357	12.25260269
1296.20889	2.690130031	1018.19295	4.955770821	895.61076	12.25435504
1301.20889	2.696116068	1019.41748	4.957754017	896.208892	12.25602695
1306.20889	2.701949218	1019.98436	4.958665144	897.909512	12.26077373
1311.20889	2.707634699	1020.61076	4.959666947	898.192948	12.26156389
1316.20889	2.71317787	1021.20889	4.960618692	899.417483	12.26497451
1321.20889	2.71858418	1022.90951	4.963299235	899.984357	12.26655171
1326.20889	2.723859185	1023.19295	4.963742359	900.61076	12.26829333
1331.20889	2.72900851	1024.41748	4.965644903	901.208892	12.26995515
1336.20889	2.734037864	1024.98436	4.966519157	902.909512	12.27467403
1341.20889	2.738952995	1025.61076	4.967480435	903.192948	12.27545965
1346.20889	2.743759716	1026.20889	4.96839367	904.417483	12.27885108
1351.20889	2.748463862	1029.41748	4.973214895	904.984357	12.28041962
1356.20889	2.753071295	1029.98436	4.97405316	905.61076	12.28215182
1361.20889	2.757587885	1030.61076	4.974974758	906.208892	12.28380482
1366.20889	2.762019509	1031.20889	4.975850206	907.909512	12.28849961
1371.20889	2.766372034	1032.90951	4.978315045	908.192948	12.28928138
1376.20889	2.770651305	1034.41748	4.980470918	909.417483	12.29265658
1381.20889	2.774863138	1034.98436	4.981274178	909.984357	12.29421788
1386.20889	2.779013305	1035.61076	4.98215727	910.61076	12.29594236
1391.20889	2.783107529	1036.20889	4.982996074	911.208892	12.29758825
1396.20889	2.787151478	1037.90951	4.985357514	912.909512	12.30226423
1401.20889	2.791150779	1039.41748	4.987422633	913.192948	12.30304309
1406.20889	2.795110958	1039.98436	4.988191987	914.417483	12.30640679
		1040.61076	4.989037759	914.984357	12.30796339
		1041.20889	4.989841055	915.61076	12.30968321
		1042.90951	4.992102283	916.208892	12.31132525
		1044.41748	4.994079432	917.909512	12.31599469
		1044.98436	4.994815944	918.192948	12.3167733
		1045.61076	4.995625562	919.417483	12.32014
		1047.90951	4.998558689	919.984357	12.32170108
		1049.41748	5.000450737	920.61076	12.32342925
		1049.98436	5.001155493	921.208892	12.32508435
		1050.61076	5.001930151	922.909512	12.32984912
		1051.20889	5.002665831	923.192948	12.33066415
		1052.90951	5.004736281	924.417483	12.33370365
		1054.41748	5.006546139	924.984357	12.33418645
		1054.98436	5.007220212	925.61076	12.33650093
		1055.61076	5.007961107	926.208892	12.3385887

Final State: j’=5		Final State: j’=4		Final State: j’=3	
E(cm⁻¹)	σ (Å²)	E(cm⁻¹)	σ (Å²)	E(cm⁻¹)	σ (Å²)
		1057.90951	5.010644649	927.909512	12.34318179
		1059.41748	5.012375192	928.192948	12.3441959
		1059.98436	5.01301967	929.417483	12.34620469
		1060.61076	5.013728024	929.984357	12.34795918
		1061.20889	5.014400674	930.61076	12.34933918
		1062.90951	5.016293421	931.208892	12.35170846
		1064.41748	5.017947561	932.909512	12.3558764
		1064.98436	5.018563549	933.192948	12.35706351
		1065.61076	5.019240564	934.417483	12.36014067
		1066.20889	5.019883427	934.984357	12.36187132
		1067.90951	5.021692255	935.61076	12.36368048
		1070.61076	5.024508392	936.208892	12.3631691
		1071.20889	5.025122638	937.909512	12.3685976
		1072.90951	5.026850847	938.192948	12.37006971
		1074.41748	5.028360948	939.417483	12.37373247
		1074.98436	5.028923249	939.984357	12.37532633
		1075.61076	5.029541218	940.61076	12.37650288
		1076.20889	5.030127995	941.208892	12.37735617
		1077.90951	5.031778868	942.909512	12.38274084
		1079.41748	5.033221343	943.192948	12.38364278
		1079.98436	5.033758444	944.417483	12.38711716
		1080.61076	5.034348718	944.984357	12.38867158
		1084.41748	5.0378638	945.61076	12.39033513
		1084.98436	5.038376814	946.208892	12.39073402
		1085.61076	5.038940595	947.909512	12.39557116
		1089.41748	5.042297963	948.192948	12.39685917
		1089.98436	5.04278887	949.417483	12.40053159
		1090.61076	5.043326481	949.984357	12.40208624
		1091.20889	5.043837814	950.61076	12.40378694
		1094.41748	5.046533489	951.208892	12.40539063
		1094.98436	5.047001581	952.909512	12.40933036
		1095.61076	5.047516014	953.192948	12.4105253
		1099.41748	5.050579924	954.417483	12.41395291
		1099.98436	5.051027176	954.984357	12.41564028
		1101.20889	5.051985493	955.61076	12.41740944
		1104.98436	5.054874242	956.208892	12.41910202
		1109.41748	5.058143389	957.909512	12.42371049
		1109.98436	5.058552165	958.192948	12.42424872
		1111.20889	5.059428199	959.417483	12.42582905
		1114.41748	5.061679137	959.984357	12.42710213
		1114.98436	5.062070259	960.61076	12.4295953
		1116.20889	5.062908547	961.208892	12.4323178
		1119.98436	5.065437705	962.909512	12.43620347

Final State: j'=5		Final State: j'=4		Final State: j'=3	
E(cm⁻¹)	σ (Å²)	E(cm⁻¹)	σ (Å²)	E(cm⁻¹)	σ (Å²)
		1121.20889	5.066240483	963.192948	12.43726927
		1124.41748	5.068304664	964.417483	12.4375537
		1124.98436	5.068663562	964.984357	12.44088008
		1129.41748	5.071412499	965.61076	12.44308293
		1129.98436	5.071756814	966.208892	12.44488363
		1131.20889	5.072495171	967.909512	12.44816764
		1134.41748	5.074395571	968.192948	12.44916701
		1134.98436	5.074726291	969.417483	12.45359886
		1136.20889	5.075435663	969.984357	12.45523691
		1139.41748	5.077262602	970.61076	12.4523804
		1139.98436	5.077580714	971.208892	12.4571941
		1141.20889	5.078263232	972.909512	12.46261201
		1144.41748	5.080022284	973.192948	12.46341621
		1144.98436	5.080328776	974.417483	12.46150876
		1149.98436	5.082979226	974.984357	12.46749628
		1151.20889	5.083614487	975.61076	12.46963743
		1154.41748	5.085254905	976.208892	12.47197272
		1154.98436	5.085541232	977.909512	12.47465511
		1156.20889	5.08615638	978.192948	12.47623164
		1159.41748	5.087747311	979.417483	12.47866455
		1159.98436	5.088025464	979.984357	12.48172437
		1161.20889	5.088623645	980.61076	12.48030827
		1164.41748	5.090176534	981.208892	12.48372728
		1169.41748	5.092626276	982.909512	12.48727975
		1171.20889	5.09305436	983.192948	12.48770596
		1174.41748	5.094931825	984.417483	12.49008531
		1176.20889	5.09533425	984.984357	12.49291145
		1179.41748	5.096901252	985.61076	12.49589732
		1184.41748	5.098885226	986.208892	12.49750399
		1189.41748	5.101067343	987.909512	12.4993209
		1191.20889	5.101905244	988.192948	12.49957645
		1194.41748	5.103107708	989.417483	12.50474968
		1196.20889	5.103997313	989.984357	12.50617535
		1199.41748	5.10496744	990.61076	12.50764238
		1206.20889	5.107468704	991.208892	12.50887672
		1209.41748	5.109417625	992.909512	12.51333905
		1211.20889	5.109698598	993.192948	12.51434147
		1214.41748	5.110867617	994.417483	12.51746818
		1216.20889	5.112139241	994.984357	12.51887978
		1219.41748	5.113275797	995.61076	12.52043495
		1224.41748	5.115175333	996.208892	12.52190034
		1229.41748	5.116801113	997.909512	12.52614947
		1231.20889	5.117304243	998.192948	12.52688437

Final State: j’=5		Final State: j’=4		Final State: j’=3	
E(cm⁻¹)	σ (Å²)	E(cm⁻¹)	σ (Å²)	E(cm⁻¹)	σ (Å²)
		1236.20889	5.119487125	999.417483	12.52995966
		1239.41748	5.12069606	999.984357	12.53105379
		1241.20889	5.121356848	1000.61076	12.53308324
		1249.41748	5.124457981	1001.20889	12.53474613
		1251.20889	5.125139488	1002.90951	12.53897796
		1254.41748	5.126359757	1003.19295	12.53969379
		1259.41748	5.128296139	1004.41748	12.5427922
		1264.41748	5.130231939	1004.98436	12.54422659
		1266.20889	5.130929237	1005.61076	12.54581024
		1271.20889	5.132883	1006.20889	12.5473198
		1276.20889	5.134850953	1007.90951	12.55158887
		1281.20889	5.136834114	1008.19295	12.55230661
		1286.20889	5.138833289	1009.41748	12.55546181
		1291.20889	5.140849277	1009.98436	12.5569
		1296.20889	5.142882542	1010.61076	12.55848296
		1301.20889	5.144933431	1011.20889	12.55999257
		1306.20889	5.147002127	1012.90951	12.56427929
		1316.20889	5.151192763	1013.19295	12.56499304
		1326.20889	5.155452795	1014.41748	12.56807427
		1346.20889	5.164164224	1014.98436	12.56949941
		1351.20889	5.166377514	1015.61076	12.57107363
		1366.20889	5.173086966	1016.20889	12.57257668
		1371.20889	5.175342952	1017.90951	12.57685007
		1376.20889	5.177606714	1018.19295	12.57756181
		1381.20889	5.179877048	1019.41748	12.58063384
		1386.20889	5.18215267	1019.98436	12.58205429
		1391.20889	5.184432305	1020.61076	12.58362271
		1396.20889	5.186714657	1021.20889	12.5851192
		1401.20889	5.188998401	1022.90951	12.58936847
		1406.20889	5.191282221	1023.19295	12.5900759
				1024.41748	12.59312974
				1024.98436	12.59454214
				1025.61076	12.59610189
				1026.20889	12.59759031
				1027.90951	12.60181709
				1029.41748	12.60555845
				1029.98436	12.60696324
				1030.61076	12.60851449
				1031.20889	12.60999466
				1032.90951	12.61419742
				1034.41748	12.61791696
				1034.98436	12.61931344
				1035.61076	12.6208555

Final State: j'=5		Final State: j'=4		Final State: j'=3	
E(cm⁻¹)	σ (Å²)	E(cm⁻¹)	σ (Å²)	E(cm⁻¹)	σ (Å²)
				1036.20889	12.62232683
				1037.90951	12.62650423
				1039.41748	12.630201
				1039.98436	12.63158886
				1040.61076	12.63312129
				1041.20889	12.63458339
				1042.90951	12.63873429
				1044.41748	12.64240715
				1044.98436	12.64378593
				1045.61076	12.64530826
				1046.20889	12.64676067
				1047.90951	12.65088369
				1049.41748	12.65453144
				1049.98436	12.65590072
				1050.61076	12.65741247
				1051.20889	12.65885472
				1052.90951	12.66294854
				1054.41748	12.66657007
				1054.98436	12.66792937
				1055.61076	12.66943008
				1056.20889	12.67086171
				1057.90951	12.67492501
				1059.41748	12.67851908
				1059.98436	12.67986796
				1060.61076	12.68135711
				1061.20889	12.68277763
				1062.90951	12.68680907
				1064.41748	12.69037451
				1064.98436	12.69171254
				1065.61076	12.69318961
				1066.20889	12.69459857
				1067.90951	12.69859682
				1069.41748	12.70213242
				1069.98436	12.70345915
				1070.61076	12.70492367
				1071.20889	12.70632059
				1072.90951	12.71028436
				1074.41748	12.71378891
				1074.98436	12.71510388
				1075.61076	12.71655538
				1076.20889	12.7179398
				1077.90951	12.72186765
				1079.41748	12.7253401

Final State: j’=5		Final State: j’=4		Final State: j’=3	
E(cm⁻¹)	σ (Å²)	E(cm⁻¹)	σ (Å²)	E(cm⁻¹)	σ (Å²)
				1079.98436	12.7266429
				1080.61076	12.72808088
				1081.20889	12.72945228
				1084.41748	12.73678219
				1084.98436	12.73807238
				1085.61076	12.73949637
				1086.20889	12.74085441
				1089.41748	12.74811142
				1089.98436	12.74938725
				1090.61076	12.75079815
				1091.20889	12.75214235
				1094.41748	12.7593242
				1094.98436	12.76058798
				1095.61076	12.76198261
				1096.20889	12.76331255
				1099.41748	12.77041702
				1099.98436	12.77166695
				1101.20889	12.77436154
				1104.41748	12.78138646
				1104.98436	12.78262219
				1106.20889	12.78528599
				1109.41748	12.79222933
				1109.98436	12.7934505
				1111.20889	12.79608271
				1114.41748	12.80294249
				1114.98436	12.80414879
				1116.20889	12.80674874
				1119.41748	12.81352312
				1119.98436	12.81471421
				1121.20889	12.81728117
				1124.41748	12.82396844
				1124.98436	12.82514402
				1126.20889	12.82767738
				1129.41748	12.83427591
				1129.98436	12.83543571
				1131.20889	12.83793489
				1134.41748	12.84444321
				1134.98436	12.84558698
				1136.20889	12.84805145
				1139.41748	12.85446818
				1139.98436	12.85559569
				1144.41748	12.86434888
				1144.98436	12.86545991

Final State: j'=5		Final State: j'=4		Final State: j'=3	
E(cm⁻¹)	σ (Å²)	E(cm⁻¹)	σ (Å²)	E(cm⁻¹)	σ (Å²)
				1146.20889	12.86785347
				1149.41748	12.87408346
				1149.98436	12.87517783
				1151.20889	12.87753531
				1154.41748	12.8836702
				1154.98436	12.88474767
				1156.20889	12.88706862
				1159.41748	12.89310705
				1159.98436	12.89416734
				1161.20889	12.896451
				1164.41748	12.90239008
				1166.20889	12.90567632
				1169.41748	12.91148981
				1171.20889	12.91497781
				1174.41748	12.92047034
				1176.20889	12.92386655
				1179.41748	12.92940718
				1181.20889	12.93257451
				1184.41748	12.93816442
				1186.20889	12.94122426
				1189.41748	12.94666686
				1191.20889	12.94969717
				1194.41748	12.95504136
				1196.20889	12.95799372
				1199.41748	12.96337861
				1201.20889	12.96610606
				1204.41748	12.97129867
				1206.20889	12.97446932
				1209.41748	12.97929745
				1211.20889	12.9822822
				1214.41748	12.98722893
				1216.20889	12.98992555
				1219.41748	12.99481915
				1221.20889	12.99721083
				1224.41748	13.00248581
				1226.20889	13.00482755
				1229.41748	13.00982508
				1231.20889	13.01234041
				1234.41748	13.01721098
				1236.20889	13.01963393
				1239.41748	13.02411945
				1241.20889	13.02671289
				1244.41748	13.03120153

Final State: j'=5		Final State: j'=4		Final State: j'=3	
E(cm⁻¹)	σ (Å²)	E(cm⁻¹)	σ (Å²)	E(cm⁻¹)	σ (Å²)
				1246.20889	13.03369471
				1249.41748	13.03804165
				1254.41748	13.04476448
				1256.20889	13.04713696
				1259.41748	13.05135882
				1261.20889	13.05369179
				1264.41748	13.05782819
				1266.20889	13.06011657
				1269.41748	13.06417682
				1271.20889	13.06642216
				1286.20889	13.08463536
				1291.20889	13.09048056
				1296.20889	13.09621672
				1301.20889	13.10184608
				1306.20889	13.10737083
				1311.20889	13.112793
				1316.20889	13.11811455
				1321.20889	13.12333724
				1326.20889	13.12846276
				1331.20889	13.13349261
				1336.20889	13.13842813
				1341.20889	13.14327046
				1346.20889	13.14802068
				1356.20889	13.15724769
				1361.20889	13.16172559
				1366.20889	13.16611344
				1371.20889	13.17041127
				1376.20889	13.17461894
				1381.20889	13.17873605
				1386.20889	13.18276198
				1391.20889	13.18669598
				1396.20889	13.19053695
				1401.20889	13.19428402

		Initial rotational state: j=1	
**Final State: j’=2**		**Final State: j’=0**	
E(cm⁻¹)	σ (Å²)	E(cm⁻¹)	σ (Å²)
83.0095117	3.794676575	28.2929483	278.5189405
83.1095117	5.903877577	28.3929483	663.199311
83.1929483	5.140869404	28.4929483	256.4938428
83.2095117	5.220217941	28.5929483	255.0379768
83.3095117	5.791265281	28.6929483	203.7541268
83.4095117	6.216336096	28.7929483	167.5766639
83.5095117	8.02568896	28.8929483	162.5558799
83.6095117	7.956724838	28.9929483	140.6719551
83.7095117	7.553223532	29.0929483	136.7137287
83.8095117	7.825996708	38.1929483	41.6619436
87.9095117	13.7315992	43.1929483	34.6251486
88.0095117	13.79552463	48.1929483	32.3693085
88.1095117	13.83069927	53.1929483	27.11146241
88.1929483	13.83455495	58.1929483	19.28778028
88.2095117	13.83223381	63.1929483	22.23947457
88.3095117	13.79331583	68.1929483	18.37766862
88.4095117	13.70421011	73.1929483	16.83175009
92.9095117	16.78327506	78.1929483	17.75535316
93.1929483	17.08300996	83.0095117	16.60971215
97.9095117	22.75875044	83.1095117	15.75669957
98.1929483	21.46794093	83.1929483	16.35399708
100.61076	18.94902615	83.2095117	16.36760363
102.909512	18.43287357	83.3095117	16.32767915
103.192948	18.25452192	83.4095117	15.93946078
105.61076	20.58764955	83.5095117	14.18875055
107.909512	18.50587889	83.6095117	13.39561986
108.192948	18.51820206	83.7095117	14.90896154
110.61076	18.83527854	83.8095117	15.07200752
112.909512	18.76308904	87.9095117	14.62576232
113.192948	18.9649141	88.0095117	14.68037157
115.61076	18.90024931	88.1095117	14.75584044
117.909512	19.53455539	88.1929483	14.84116702
118.192948	19.47746461	88.2095117	14.86120771
120.61076	18.97096127	88.3095117	15.01124646
122.909512	19.51828879	88.4095117	15.23152873
123.192948	19.48076782	92.9095117	13.18711011
125.61076	18.37737864	93.1929483	13.17078192
127.909512	19.57162397	97.9095117	12.10580601
128.192948	19.54959495	98.1929483	11.97820956
130.61076	19.06590609	100.61076	12.16071376
132.909512	18.63970236	102.909512	11.68065812
133.192948	18.52140321	103.192948	11.50117138

Final State: j'=2		Final State: j'=0	
E(cm^{-1})	σ (Å^2)	E(cm^{-1})	σ (Å^2)
135.61076	19.22822923	105.61076	11.26479032
137.909512	18.2657858	107.909512	10.90036349
138.192948	17.78095534	108.192948	10.90902877
140.61076	18.52537062	110.61076	11.15984826
142.909512	18.09109222	112.909512	10.99835362
143.192948	18.29589074	113.192948	11.02852498
145.61076	17.32410652	115.61076	10.76459811
147.909512	19.39358218	117.909512	11.13679261
148.192948	19.29282306	118.192948	11.1088197
150.61076	19.27716472	120.61076	10.69544902
152.909512	18.30138591	122.909512	10.97479111
153.192948	17.78361345	123.192948	10.9519402
155.61076	19.01696235	125.61076	10.09996795
157.909512	17.18566023	127.909512	10.8077593
158.192948	17.99260134	128.192948	10.79957306
160.61076	19.04275941	130.61076	10.52082215
162.909512	18.33466172	132.909512	10.11005014
163.192948	18.24630652	133.192948	10.05727296
165.084357	18.73181555	135.61076	10.42040254
165.184357	18.15326241	137.909512	9.78239285
165.284357	18.4098733	138.192948	9.47540409
165.384357	18.20981915	140.61076	9.76820805
165.484357	17.90127649	142.909512	9.51256607
165.584357	17.11521874	143.192948	9.57076217
165.61076	16.85601599	145.61076	9.02759289
165.684357	17.16312289	147.909512	10.14665346
165.784357	17.97360023	148.192948	10.10083817
165.884357	17.85602883	150.61076	10.06211898
165.984357	17.4475807	152.909512	9.47218682
166.084357	18.0034489	153.192948	9.06070977
166.184357	18.05408821	155.61076	9.63954039
167.909512	17.37189698	157.909512	8.36851222
168.192948	17.40106942	158.192948	8.93152027
169.984357	17.13528342	160.61076	9.76820092
170.084357	17.09553676	162.909512	9.17455154
170.184357	17.05208438	163.192948	8.9833394
170.284357	17.00180032	165.084357	9.60551263
170.384357	16.93897204	165.184357	9.35206912
170.484357	16.85249284	165.284357	9.47772483
170.584357	16.71918713	165.384357	9.38212589
170.61076	16.67104921	165.484357	9.19877529
170.684357	16.49624484	165.584357	8.68540349
172.909512	17.02350096	165.61076	8.53190151

Final State: j'=2		Final State: j'=0	
E(cm⁻¹)	σ (Å²)	E(cm⁻¹)	σ (Å²)
173.192948	17.01978978	165.684357	8.77493821
174.984357	16.86995555	165.784357	9.26953455
175.61076	16.74062547	165.884357	9.20379616
177.909512	16.56578564	165.984357	8.96311237
178.192948	16.56379269	166.084357	9.32692411
179.984357	16.18877435	166.184357	9.37198326
180.61076	16.60534837	167.909512	9.13269082
182.909512	16.48849248	168.192948	9.15345846
183.192948	16.48074254	169.984357	9.03749011
184.984357	16.4334029	170.084357	9.02091951
185.61076	16.41238394	170.184357	9.00223022
187.909512	16.20505773	170.284357	8.97974494
188.192948	16.22802247	170.384357	8.95025767
189.984357	16.30277923	170.484357	8.90702522
190.61076	16.30034743	170.584357	8.83421308
192.909512	16.27070924	170.61076	8.80602189
193.192948	16.26580027	170.684357	8.69423257
194.984357	16.22771059	172.909512	8.95290452
195.61076	16.21290243	173.192948	8.94911688
197.909512	16.17709937	174.984357	8.87346349
198.192948	16.17423434	175.61076	8.82097651
199.984357	16.15661975	177.909512	8.72948865
200.61076	16.15019786	178.192948	8.72451713
202.909512	16.12542545	179.984357	8.55983136
203.192948	16.12232187	180.61076	8.69318484
204.984357	16.10345755	182.909512	8.62367491
205.61076	16.09750771	183.192948	8.61696791
207.909512	16.08129875	184.984357	8.57537214
208.192948	16.08015236	185.61076	8.55965582
209.984357	16.08048291	187.909512	8.46200699
210.61076	16.08571784	188.192948	8.46412357
212.909512	16.11625448	189.984357	8.45979556
213.192948	16.06552066	190.61076	8.45002717
214.984357	15.95030704	192.909512	8.40856277
215.61076	15.86457056	193.192948	8.4031455
217.909512	15.83534724	194.984357	8.3670847
218.192948	15.75980826	195.61076	8.35404727
219.984357	15.89762318	197.909512	8.3117337
220.61076	15.81442382	198.192948	8.30704607
222.909512	15.71369791	199.984357	8.27802482
223.192948	15.75765358	200.61076	8.2679691
224.984357	15.59360928	202.909512	8.23137329
225.61076	15.72042183	203.192948	8.22691454

Final State: j’=2		Final State: j’=0	
E(cm⁻¹)	σ (Å²)	E(cm⁻¹)	σ (Å²)
227.909512	15.71915902	204.984357	8.19929287
228.192948	15.742102	205.61076	8.18997368
229.984357	15.49850127	207.909512	8.1583525
230.61076	15.71473296	208.192948	8.15484252
232.909512	15.63399247	209.984357	8.13617177
233.192948	15.65036768	210.61076	8.13208639
234.984357	15.59280508	212.909512	8.15242798
235.61076	15.45134177	213.192948	8.13831385
237.909512	15.53312884	214.984357	8.07535201
238.192948	15.54834091	215.61076	8.01635355
239.984357	15.63684508	217.909512	8.00307021
240.61076	15.68119982	218.192948	7.96818355
242.909512	15.34684654	219.984357	7.98234711
243.192948	15.43136085	220.61076	7.9777686
244.984357	15.58506686	222.909512	7.87603182
245.61076	15.63884396	223.192948	7.88694481
247.909512	15.50327665	224.984357	7.84787011
248.192948	15.3545308	225.61076	7.85748199
249.984357	15.40998258	227.909512	7.82322922
250.61076	15.31736842	228.192948	7.83063576
252.909512	15.26049528	229.984357	7.76154051
253.192948	15.20823333	230.61076	7.8166783
254.984357	15.61989231	232.909512	7.74226219
255.61076	15.13842066	233.192948	7.74643757
257.909512	15.33352498	234.984357	7.7583585
258.192948	15.39828488	235.61076	7.68384177
259.984357	15.11086423	237.909512	7.65758923
260.61076	15.21824025	238.192948	7.66076948
262.909512	15.26749177	239.984357	7.68349444
263.192948	15.07864371	240.61076	7.70906183
264.984357	15.19136474	242.909512	7.55210497
265.61076	15.22776403	243.192948	7.57440356
267.909512	14.87836738	244.984357	7.61800202
268.192948	15.04302893	245.61076	7.63741857
269.984357	15.16619741	247.909512	7.65196894
270.61076	15.42663365	248.192948	7.58302077
272.909512	14.77238425	249.984357	7.56360463
273.192948	14.92589281	250.61076	7.51261413
274.517483	15.13124521	252.909512	7.48879016
274.617483	15.13886009	253.192948	7.44570194
274.717483	15.16346813	254.984357	7.60339325
274.817483	15.2091644	255.61076	7.47946012
274.917483	15.2735703	257.909512	7.42477851

Final State: j’=2		Final State: j’=0	
E(cm⁻¹)	σ (Å²)	E(cm⁻¹)	σ (Å²)
274.984357	15.3373892	258.192948	7.45329235
275.017483	15.33618722	259.984357	7.33130181
275.117483	14.88367201	260.61076	7.36010613
275.217483	14.92771368	262.909512	7.45877544
275.317483	15.04438446	263.192948	7.37619505
275.417483	14.97294523	264.984357	7.33420226
275.517483	14.94894217	265.61076	7.3454239
275.61076	15.00350794	267.909512	7.20159911
275.617483	15.00642362	268.192948	7.24457022
275.717483	15.04134499	269.984357	7.27562334
275.817483	15.06743904	270.61076	7.39803386
277.909512	14.93426634	272.909512	7.23344628
278.192948	14.95543206	273.192948	7.22784309
279.417483	14.99936866	274.517483	7.23741896
279.517483	15.00221604	274.617483	7.26246727
279.617483	15.00569275	274.717483	7.25649212
279.717483	15.01003973	274.817483	7.27785942
279.817483	15.01550959	274.917483	7.31124554
279.917483	15.02220061	274.984357	7.35628717
279.984357	15.02698152	275.017483	7.38049038
280.017483	15.02904515	275.117483	7.24223911
280.117483	15.02600362	275.217483	7.19333418
280.217483	14.94487313	275.317483	7.23317362
280.317483	14.77614539	275.417483	7.23431608
280.61076	14.97432585	275.517483	7.19179044
282.909512	14.85705742	275.61076	7.20511275
283.192948	14.86014088	275.617483	7.20597353
284.417483	14.86103753	275.717483	7.2171775
284.984357	14.85477607	275.817483	7.22652491
285.61076	14.833333	277.909512	7.17747041
287.909512	14.79922515	278.192948	7.18266937
288.192948	14.80018316	279.417483	7.19852032
289.417483	14.66646604	279.517483	7.19995032
289.984357	14.73176439	279.617483	7.20170865
290.61076	14.7361507	279.717483	7.20397899
292.909512	14.70792118	279.817483	7.20701778
293.192948	14.70411706	279.917483	7.21122541
294.417483	14.68752221	279.984357	7.21496665
294.984357	14.67966788	280.017483	7.21715208
295.61076	14.67053778	280.117483	7.22423491
297.909512	14.61719609	280.217483	7.21317765
298.192948	14.61553914	280.317483	7.15187989
299.417483	14.60411871	280.61076	7.21421266

Final State: j’=2		Final State: j’=0	
E(cm^{-1})	σ (Å^2)	E(cm^{-1})	σ (Å^2)
299.984357	14.59722909	282.909512	7.12620786
300.61076	14.58912187	283.192948	7.12598529
302.909512	14.55734624	284.417483	7.12140588
303.192948	14.55324978	284.984357	7.11819748
304.417483	14.53488366	285.61076	7.11070919
304.984357	14.52603676	287.909512	7.08454853
305.61076	14.51623317	289.417483	7.03568211
307.909512	14.48233715	289.984357	7.04449816
308.192948	14.4783237	290.61076	7.04371415
309.417483	14.46106907	292.909512	7.02185513
309.984357	14.45308622	293.192948	7.01894185
310.61076	14.44425022	294.417483	7.00635999
312.909512	14.41160078	294.984357	7.00056367
313.192948	14.40754929	295.61076	6.99416971
314.417483	14.39000389	297.909512	6.96337043
314.984357	14.38187263	298.192948	6.96070758
315.61076	14.37289275	299.417483	6.94902025
317.909512	14.34007511	299.984357	6.94332167
318.192948	14.3360463	300.61076	6.93691454
319.417483	14.31867958	302.909512	6.91298627
319.984357	14.31065828	303.192948	6.9100023
320.61076	14.30180548	304.417483	6.89694348
322.909512	14.26939103	304.984357	6.89078731
323.192948	14.26540082	305.61076	6.88394621
324.417483	14.24817705	307.909512	6.85933273
324.984357	14.24021243	308.192948	6.85635619
325.61076	14.23141867	309.417483	6.84356307
327.909512	14.19922149	309.984357	6.8376632
328.192948	14.19526052	310.61076	6.83115307
329.417483	14.17817326	312.909512	6.80729748
329.984357	14.17027747	313.192948	6.80435711
330.61076	14.16156335	314.417483	6.7916576
332.909512	14.12968785	314.984357	6.78578342
333.192948	14.12576935	315.61076	6.77929897
334.417483	14.10887149	317.909512	6.75559061
334.984357	14.10106698	318.192948	6.75267834
335.61076	14.09245692	319.417483	6.74012313
337.909512	14.06099992	319.984357	6.73432491
338.192948	14.05713829	320.61076	6.72792725
339.417483	14.04050489	322.909512	6.70452431
339.984357	14.0328354	323.192948	6.70164602
340.61076	14.02438615	324.417483	6.68922768
342.909512	13.99367385	324.984357	6.68348784

Final State: j’=2		Final State: j’=0	
E(cm⁻¹)	σ (Å²)	E(cm⁻¹)	σ (Å²)
343.192948	13.98992846	325.61076	6.67715165
344.417483	13.97389974	327.909512	6.6539552
344.984357	13.9665889	328.192948	6.65110103
345.61076	13.95862504	329.417483	6.63878425
347.909512	13.9318271	329.984357	6.63308987
348.192948	13.92921933	330.61076	6.6268028
349.417483	13.93145394	332.909512	6.6037716
349.984357	13.94544266	333.192948	6.60093565
350.61076	13.93024665	334.417483	6.58869054
352.909512	13.86678282	334.984357	6.58302505
353.192948	13.86536777	335.61076	6.57676608
354.417483	13.88923816	337.909512	6.55379688
354.984357	13.86551221	338.192948	6.55096316
355.61076	13.83575261	339.417483	6.53871056
357.909512	13.82287951	339.984357	6.53303045
358.192948	13.80106177	340.61076	6.52674548
359.417483	13.78291547	342.909512	6.5035579
359.984357	13.76701667	343.192948	6.50067879
360.61076	13.76800781	344.417483	6.48815839
362.909512	13.79088323	344.984357	6.48230233
363.192948	13.74179779	347.909512	6.45069902
364.417483	13.70361496	348.192948	6.44738749
364.984357	13.70214379	349.417483	6.43421477
365.61076	13.70982918	349.984357	6.44145676
367.909512	13.67783271	350.61076	6.44953143
368.192948	13.64444733	352.909512	6.41446268
369.417483	13.63263199	353.192948	6.40771454
369.984357	13.62958943	354.417483	6.41836843
370.61076	13.62935919	354.984357	6.41166347
372.909512	13.64631834	355.61076	6.39326324
373.192948	13.60334557	357.909512	6.3911211
374.417483	13.55930273	358.192948	6.3798048
374.984357	13.55542317	359.417483	6.37319378
375.61076	13.55122105	359.984357	6.352796
377.909512	13.59962652	360.61076	6.34128561
378.192948	13.55327113	362.909512	6.35044005
379.417483	13.50797227	363.192948	6.34993585
379.984357	13.47969949	364.417483	6.31098294
380.61076	13.47787325	364.984357	6.30210537
382.909512	13.47984598	365.61076	6.29393802
383.192948	13.49413098	367.909512	6.30922489
384.417483	13.50832672	368.192948	6.29274754
384.984357	13.51628708	369.417483	6.26474682

Final State: j’=2		Final State: j’=0	
E(cm^{-1})	σ (Å^2)	E(cm^{-1})	σ (Å^2)
385.61076	13.48383	369.984357	6.25674943
387.909512	13.40077808	370.61076	6.24837471
388.192948	13.39563591	372.909512	6.252915
389.417483	13.45063726	373.192948	6.26021099
389.984357	13.40213102	374.417483	6.22112181
390.61076	13.37106982	375.61076	6.20448404
392.909512	13.3643128	377.909512	6.20576319
393.192948	13.3954875	378.192948	6.21118815
394.417483	13.30023885	379.417483	6.19212449
394.984357	13.31152321	379.984357	6.17070884
395.61076	13.38166541	380.61076	6.15831877
397.909512	13.26573418	382.909512	6.12449982
398.192948	13.26651523	383.192948	6.12651285
399.417483	13.33957728	384.417483	6.13015402
399.984357	13.33415586	384.984357	6.16460174
400.61076	13.23390256	385.61076	6.13712639
402.909512	13.24198303	387.909512	6.11447146
403.192948	13.24175433	388.192948	6.10224845
404.417483	13.25510859	389.417483	6.11263585
404.984357	13.16610788	389.984357	6.1182081
405.61076	13.21326893	390.61076	6.08326625
407.909512	13.22135025	392.909512	6.10992461
408.192948	13.20550001	393.192948	6.12435593
409.417483	13.12057618	394.417483	6.04592419
409.984357	13.14887889	394.984357	6.03249
410.61076	13.10115079	395.61076	6.04905399
411.308892	13.09912199	397.909512	6.01221786
411.808892	13.1683755	398.192948	6.00663984
412.008892	13.13139189	399.417483	5.99935686
412.208892	13.15170015	399.984357	6.05101698
412.308892	13.10927939	400.61076	6.01799254
412.608892	13.10593096	402.909512	5.97019196
412.708892	13.10833557	403.192948	5.97569514
416.208892	13.08250548	404.417483	6.03000003
416.308892	13.08350793	404.984357	5.9716603
416.408892	13.08482644	405.61076	5.94584047
416.508892	13.08663253	407.909512	5.95293374
416.608892	13.08921442	408.192948	5.93374148
416.708892	13.0930927	409.417483	5.93969041
416.808892	13.09928633	409.984357	5.95267465
419.984357	13.02253578	410.61076	5.93918454
421.208892	13.01463587	411.308892	5.89864833
425.61076	12.98475352	411.408892	5.90261296

Final State: j’=2		Final State: j’=0	
E(cm⁻¹)	σ (Å²)	E(cm⁻¹)	σ (Å²)
427.909512	12.93576036	411.508892	5.89679157
428.192948	12.93298514	411.608892	5.89275496
429.417483	12.92002593	411.708892	5.88951951
429.984357	12.91384817	411.808892	5.89827271
434.417483	12.86236278	411.908892	5.92975034
434.984357	12.85511128	412.008892	5.93589911
436.208892	12.84135482	412.108892	5.91236842
437.909512	12.82238455	412.208892	5.92527113
439.417483	12.80551274	412.308892	5.9196448
441.208892	12.78539273	412.408892	5.90855157
443.192948	12.76261002	412.508892	5.90319986
446.208892	12.72827013	412.608892	5.89967351
451.208892	12.67223892	412.708892	5.89692467
452.909512	12.65325063	412.808892	5.89456261
454.984357	12.63015284	412.909512	5.89241483
466.208892	12.50686029	413.192948	5.88898134
467.909512	12.48840599	414.417483	5.89894069
468.192948	12.48533605	414.984357	5.88879415
471.208892	12.45277202	415.61076	5.88138329
478.192948	12.37806671	416.208892	5.87649892
479.417483	12.3650677	416.308892	5.87577646
481.208892	12.34610348	416.408892	5.87507542
482.909512	12.32815735	416.508892	5.8744154
484.417483	12.31229002	416.608892	5.87384983
484.984357	12.30633622	416.708892	5.87351978
485.61076	12.29976408	416.808892	5.8738558
486.208892	12.29349529	416.908892	5.87653342
489.417483	12.25997717	417.008892	5.88635169
492.909512	12.22369939	417.108892	5.89268841
495.61076	12.19576881	417.208892	5.88720485
499.417483	12.15657321	417.308892	5.87597542
499.984357	12.15074971	417.909512	5.88230517
501.208892	12.13817837	418.192948	5.88343891
502.909512	12.12073117	419.417483	5.85823846
503.192948	12.11782369	419.984357	5.85282845
504.417483	12.10525958	420.61076	5.84751595
504.984357	12.09943971	421.208892	5.84282101
505.61076	12.09300399	422.909512	5.83346861
507.909512	12.06930118	423.192948	5.8314455
508.192948	12.06636293	424.417483	5.82126783
509.417483	12.05359611	424.984357	5.81691794
510.61076	12.04096462	425.61076	5.82316562
511.208892	12.03450195	426.208892	5.81624841

Final State: j’=2		Final State: j’=0	
E(cm⁻¹)	σ (Å²)	E(cm⁻¹)	σ (Å²)
513.192948	12.01076906	427.909512	5.79533691
514.984357	12.00000144	428.192948	5.79325255
516.208892	11.98552112	429.417483	5.78421113
517.909512	11.96159924	429.984357	5.78001139
518.192948	11.96093436	430.61076	5.77537966
520.61076	11.94629729	431.208892	5.77097798
524.417483	11.90902071	432.909512	5.75887738
524.984357	11.89921012	433.192948	5.75709205
525.61076	11.8907621	434.417483	5.74908907
526.208892	11.89665556	434.984357	5.74439542
528.192948	11.87688228	435.61076	5.73945755
529.984357	11.84833729	436.208892	5.73492143
531.208892	11.84745387	437.909512	5.72237071
533.192948	11.82972337	438.192948	5.72030279
534.417483	11.81340332	439.417483	5.7114212
535.61076	11.79332103	439.984357	5.7073398
539.417483	11.76901787	440.61076	5.70285506
541.208892	11.74543096	441.208892	5.69859664
544.417483	11.72474677	442.909512	5.68651231
544.984357	11.7165486	443.192948	5.68448807
545.61076	11.70819064	444.417483	5.67572044
549.417483	11.67823493	444.984357	5.6716622
551.208892	11.65578019	445.61076	5.66718491
552.909512	11.63730662	446.208892	5.66291861
554.984357	11.62489293	447.909512	5.65084146
559.984357	11.57470301	448.192948	5.64883629
560.61076	11.59506985	449.417483	5.64019803
565.61076	11.53869167	449.984357	5.63621206
566.208892	11.52252342	450.61076	5.63181615
568.192948	11.49376022	451.208892	5.62762652
571.208892	11.47925222	452.909512	5.61575119
572.909512	11.47723249	453.192948	5.61377685
573.192948	11.48154301	454.417483	5.60526263
575.61076	11.44271388	454.984357	5.60132983
576.208892	11.44787932	455.61076	5.59699067
577.909512	11.44023979	456.208892	5.59285398
578.192948	11.43559594	457.909512	5.58112902
579.417483	11.41368173	458.192948	5.57918023
579.984357	11.40665762	459.417483	5.57077867
580.61076	11.39917096	459.984357	5.56689915
581.208892	11.40028265	460.61076	5.56261949
582.909512	11.3912953	461.208892	5.55853993
583.192948	11.38686567	462.909512	5.5469784

Final State: j’=2		Final State: j’=0	
E(cm⁻¹)	σ (Å²)	E(cm⁻¹)	σ (Å²)
584.417483	11.37351768	463.192948	5.54505675
584.984357	11.36814494	464.417483	5.53677206
585.61076	11.36239684	464.984357	5.5329463
586.208892	11.35735822	465.61076	5.52872567
587.909512	11.34486646	466.208892	5.52470236
588.192948	11.34239053	467.909512	5.51329916
589.417483	11.33105554	468.192948	5.51140383
589.984357	11.33250019	469.417483	5.5032323
590.61076	11.32674974	469.984357	5.49945881
591.208892	11.31939903	470.61076	5.49529588
592.909512	11.30502992	471.208892	5.49132755
593.192948	11.30272955	472.909512	5.4800805
594.417483	11.29283033	473.192948	5.4782111
594.984357	11.28826487	474.417483	5.47015165
595.61076	11.28323281	474.984357	5.4664299
596.208892	11.2784468	475.61076	5.46232409
597.909512	11.26585121	476.208892	5.45841022
598.192948	11.26393167	477.909512	5.44731743
599.417483	11.25403455	478.192948	5.44547369
599.984357	11.24941735	479.417483	5.43752469
600.61076	11.24439475	479.984357	5.43385394
601.208892	11.23964595	480.61076	5.42980437
602.909512	11.22628224	481.208892	5.4259441
603.192948	11.22406956	482.909512	5.4150032
604.417483	11.21455885	483.192948	5.41318469
604.984357	11.21018805	484.417483	5.40534445
605.61076	11.20538405	484.984357	5.40172392
606.208892	11.20081553	485.61076	5.39772973
607.909512	11.18782573	486.208892	5.39392222
608.192948	11.18565669	487.909512	5.38313082
609.417483	11.1762899	488.192948	5.38133717
609.984357	11.17196174	489.417483	5.37360396
610.61076	11.16718699	489.984357	5.37003281
611.208892	11.16263713	490.61076	5.36609314
612.909512	11.14975346	491.208892	5.36233753
613.192948	11.14761399	492.909512	5.3516931
614.417483	11.13839648	493.192948	5.34992384
614.984357	11.13414302	494.417483	5.34229576
615.61076	11.12945227	494.984357	5.33877314
616.208892	11.124982	495.61076	5.3348869
617.909512	11.1123152	496.208892	5.33118223
618.192948	11.11021003	497.909512	5.32068183
619.417483	11.10113467	498.192948	5.31893647

Final State: j'=2		Final State: j'=0	
E(cm^{-1})	σ (Å^2)	E(cm^{-1})	σ (Å^2)
619.984357	11.0969443	499.417483	5.31141123
620.61076	11.09232203	499.984357	5.30793596
621.208892	11.0879172	500.61076	5.30410186
622.909512	11.0754343	501.208892	5.3004468
623.192948	11.0733603	502.909512	5.29008627
624.417483	11.06442115	503.192948	5.28836399
624.984357	11.06029456	504.417483	5.28093785
625.61076	11.05574316	504.984357	5.27750792
626.208892	11.05140559	505.61076	5.27372351
627.909512	11.03911709	506.208892	5.27011531
628.192948	11.03707534	507.909512	5.25988382
629.417483	11.02827512	508.192948	5.2581822
629.984357	11.02421263	509.417483	5.25084046
630.61076	11.01973183	509.984357	5.24744552
631.208892	11.01546147	510.61076	5.2436944
632.909512	11.00336345	511.208892	5.24010911
633.192948	11.00135337	512.909512	5.22974224
634.417483	10.99268979	513.192948	5.2278804
634.984357	10.98869049	514.417483	5.21637591
635.61076	10.98427954	514.984357	5.21505728
636.208892	10.98007576	515.61076	5.21227095
637.909512	10.96816705	516.208892	5.20591853
638.192948	10.96618848	517.909512	5.19765852
639.417483	10.95766108	518.192948	5.19481379
639.984357	10.9537247	519.417483	5.18877309
640.61076	10.94938324	519.984357	5.18604982
641.208892	10.94524584	520.61076	5.18371242
642.909512	10.93352535	521.208892	5.17974648
643.192948	10.93157812	522.909512	5.16921488
644.417483	10.92318586	523.192948	5.16341673
644.984357	10.91931199	524.417483	5.16246244
645.61076	10.91503956	524.984357	5.15864334
646.208892	10.91096793	525.61076	5.15061433
647.909512	10.89943421	526.208892	5.15001137
648.192948	10.8975181	527.909512	5.14288156
649.417483	10.88926001	528.192948	5.14133873
649.984357	10.88544814	529.417483	5.13396429
650.61076	10.88124417	529.984357	5.12946046
651.208892	10.87723785	530.61076	5.12234482
652.909512	10.86588955	531.208892	5.12290935
653.192948	10.86400429	532.909512	5.11507968
654.417483	10.8558793	533.192948	5.11347366
654.984357	10.85212902	534.417483	5.10631219

Final State: j'=2		Final State: j'=0	
E(cm⁻¹)	σ (Å²)	E(cm⁻¹)	σ (Å²)
655.61076	10.84799298	534.984357	5.10272016
656.208892	10.84405149	535.61076	5.09696519
657.909512	10.83288715	536.208892	5.09245033
658.192948	10.8310325	537.909512	5.08762868
659.417483	10.82303961	538.192948	5.08612178
659.984357	10.81935035	539.417483	5.07916412
660.61076	10.81528173	539.984357	5.07590945
661.208892	10.81140455	540.61076	5.07223519
662.909512	10.80042268	541.208892	5.0684713
663.192948	10.79859838	542.909512	5.05792027
664.417483	10.79073654	543.192948	5.05282382
664.984357	10.78710788	544.417483	5.05242304
665.61076	10.78310605	544.984357	5.04919667
666.208892	10.77929267	545.61076	5.0456432
667.909512	10.76849185	546.208892	5.04220598
668.192948	10.76669768	547.909512	5.02689069
669.417483	10.75896581	548.192948	5.02566651
669.984357	10.75539723	549.417483	5.01780888
670.61076	10.7514618	549.984357	5.01598624
671.208892	10.74771171	550.61076	5.01682012
672.909512	10.73709058	551.208892	5.00749813
673.192948	10.7353263	552.909512	5.00448056
674.417483	10.72772354	553.192948	4.99996887
674.984357	10.72421465	554.417483	4.99584285
675.61076	10.72034509	554.984357	4.99382031
676.208892	10.7166579	555.61076	4.98979086
677.909512	10.70621533	556.208892	4.97878499
678.192948	10.70448081	557.909512	4.98022608
679.417483	10.69700637	558.192948	4.97875072
679.984357	10.69355686	559.417483	4.97017123
680.61076	10.68975292	559.984357	4.95740562
681.208892	10.68612832	560.61076	4.96361283
682.909512	10.67586372	561.208892	4.96249906
683.192948	10.67415884	562.909512	4.95302166
684.417483	10.66681257	563.192948	4.95079552
684.984357	10.66342242	564.417483	4.94290034
685.61076	10.65968404	564.984357	4.93974777
686.208892	10.65612217	565.61076	4.93890401
687.909512	10.64603608	566.208892	4.93378285
688.192948	10.644361	567.909512	4.92302006
689.417483	10.63714381	568.192948	4.91329581
689.984357	10.63381358	569.417483	4.92040177
690.61076	10.63014166	569.984357	4.90671677

Final State: j’=2		Final State: j’=0	
E(cm^{-1})	σ (Å^2)	E(cm^{-1})	σ (Å^2)
691.208892	10.62664337	570.61076	4.91274952
692.909512	10.61673958	571.208892	4.91063736
694.417483	10.60801144	572.909512	4.89025464
694.984357	10.60474378	573.192948	4.89571043
695.61076	10.60114257	574.417483	4.894858
696.208892	10.5977109	574.984357	4.89092456
697.909512	10.58800508	575.61076	4.88697657
698.192948	10.58639482	576.208892	4.88114273
699.417483	10.57946448	578.192948	4.87246405
699.984357	10.57627203	580.61076	4.85885853
700.61076	10.5727575	581.208892	4.85504377
701.208892	10.56941623	582.909512	4.84841213
702.909512	10.56002502	583.192948	4.84697267
703.192948	10.55848346	585.61076	4.83423558
704.417483	10.55201353	587.909512	4.82262499
704.984357	10.54934454	588.192948	4.82122862
705.61076	10.54608566	589.417483	4.81482528
706.208892	10.54139317	589.984357	4.81237532
707.909512	10.53088829	592.909512	4.79827224
708.192948	10.52923853	594.984357	4.7881158
709.417483	10.52490086	596.208892	4.78213993
709.984357	10.52069501	597.909512	4.77389286
710.61076	10.51609618	599.417483	4.76676195
711.208892	10.51309136	599.984357	4.76403597
712.909512	10.50503934	600.61076	4.76102552
713.192948	10.50331127	603.192948	4.74866775
714.417483	10.49667088	604.984357	4.7401428
714.984357	10.49116655	605.61076	4.73717338
715.61076	10.48884615	606.208892	4.73434437
716.208892	10.48620094	608.192948	4.72499251
717.909512	10.47690065	609.417483	4.71923709
718.192948	10.47550256	610.61076	4.71364138
719.417483	10.46788243	611.208892	4.7108417
719.984357	10.46537112	612.909512	4.70290152
720.61076	10.46243508	614.984357	4.6932555
721.208892	10.45970812	615.61076	4.69035234
722.909512	10.45124768	616.208892	4.68758405
723.192948	10.44679639	617.909512	4.67973322
724.417483	10.44163405	619.984357	4.67019401
724.984357	10.43900968	620.61076	4.66732246
725.61076	10.43601495	621.208892	4.6645841
726.208892	10.43316335	625.61076	4.64453969
727.909512	10.4235038	626.208892	4.64183065

Final State: j’=2		Final State: j’=0	
E(cm^{-1})	σ (Å^2)	E(cm^{-1})	σ (Å^2)
728.192948	10.42263389	629.984357	4.62481048
729.417483	10.41524464	630.61076	4.62199975
729.984357	10.41294596	632.909512	4.61171682
730.61076	10.41003184	633.192948	4.61045237
731.208892	10.40717602	634.417483	4.60499813
732.909512	10.39909723	634.984357	4.60247795
733.192948	10.3977303	635.61076	4.59969656
734.417483	10.39167324	639.417483	4.58287128
734.984357	10.38727979	640.61076	4.57762429
735.61076	10.38443159	642.909512	4.56755252
736.208892	10.38198191	645.61076	4.55577739
737.909512	10.37446591	647.909512	4.54580733
738.192948	10.37332163	648.192948	4.54458122
739.417483	10.36874037	650.61076	4.53415025
739.984357	10.36374245	651.208892	4.53157753
740.61076	10.36061865	652.909512	4.52427939
741.208892	10.35818953	655.61076	4.51273745
742.909512	10.34930207	657.909512	4.50296335
743.192948	10.346172	659.417483	4.49657533
744.417483	10.34227365	660.61076	4.49153367
744.984357	10.34061788	663.192948	4.48066338
745.61076	10.33323505	664.417483	4.47552724
746.208892	10.33141609	664.984357	4.47315367
747.909512	10.32396219	665.61076	4.47053379
748.192948	10.3214672	669.984357	4.45232811
749.417483	10.31487774	670.61076	4.44973291
749.984357	10.31357038	672.909512	4.4402352
750.61076	10.31158914	674.984357	4.43169757
751.208892	10.31092352	675.61076	4.42912648
752.909512	10.2990696	676.208892	4.42667424
753.192948	10.29805859	679.984357	4.41125789
754.417483	10.29349075	680.61076	4.40871048
754.984357	10.29188477	681.208892	4.40628077
755.61076	10.28244216	683.192948	4.39824039
756.208892	10.28349322	684.417483	4.3932926
757.909512	10.27783873	684.984357	4.39100587
758.192948	10.27609058	685.61076	4.38848182
759.417483	10.27142996	687.909512	4.37924408
759.984357	10.26374894	690.61076	4.36843932
760.61076	10.2621688	691.208892	4.36605428
761.208892	10.26318769	693.192948	4.35816248
762.909512	10.25414575	694.984357	4.35106348
763.192948	10.25606222	695.61076	4.34858792

Final State: j’=2		Final State: j’=0	
E(cm⁻¹)	σ (Å²)	E(cm⁻¹)	σ (Å²)
764.417483	10.24303148	697.909512	4.33952946
764.984357	10.24295442	698.192948	4.33841599
765.61076	10.24042457	700.61076	4.32895375
766.208892	10.23737016	705.61076	4.31031664
767.909512	10.2287481	706.208892	4.30766517
768.192948	10.22909189	709.417483	4.29539012
769.417483	10.22088869	710.61076	4.28994689
769.984357	10.22048821	711.208892	4.28765282
770.61076	10.21804732	712.909512	4.28166486
771.208892	10.21550384	713.192948	4.28081899
772.909512	10.20779097	714.417483	4.27575199
773.192948	10.20691389	714.984357	4.27267585
774.417483	10.20071165	715.61076	4.27044241
774.984357	10.19842137	716.208892	4.26839779
775.61076	10.19568452	717.909512	4.26201304
776.208892	10.1930372	720.61076	4.25145996
777.909512	10.1853329	725.61076	4.2324103
778.192948	10.18401495	726.208892	4.23029112
779.417483	10.17860189	727.909512	4.22391429
779.984357	10.17612379	729.417483	4.2175805
780.61076	10.17331149	729.984357	4.21569675
781.208892	10.16991638	730.61076	4.21347938
782.909512	10.16351233	734.984357	4.19726619
783.192948	10.1622693	735.61076	4.19463283
784.417483	10.15693372	737.909512	4.18678356
784.984357	10.15447923	740.61076	4.17775733
785.61076	10.15177416	743.192948	4.16705239
786.208892	10.14919603	745.61076	4.15792862
787.909512	10.14187496	746.208892	4.15553359
788.192948	10.14065126	747.909512	4.15153855
789.417483	10.1353397	750.61076	4.13984504
789.984357	10.13298056	752.909512	4.13082553
790.61076	10.13038022	753.192948	4.12992908
791.208892	10.12786884	754.417483	4.12608392
792.909512	10.1207063	754.984357	4.12485084
793.192948	10.1195144	755.61076	4.12148621
794.417483	10.11437465	757.909512	4.11397281
794.984357	10.11200066	760.61076	4.10272627
795.61076	10.10938123	761.208892	4.10215122
796.208892	10.10688386	763.192948	4.09641054
797.909512	10.09981103	765.61076	4.08577705
798.192948	10.09863736	766.208892	4.08279806
799.417483	10.09358216	767.909512	4.07764492

Final State: j’=2		Final State: j’=0	
E(cm⁻¹)	σ (Å²)	E(cm⁻¹)	σ (Å²)
799.984357	10.09124874	768.192948	4.07653667
800.61076	10.08867446	769.417483	4.07188854
801.208892	10.08622033	769.984357	4.07005128
802.909512	10.07926398	770.61076	4.06797727
803.192948	10.07810772	771.208892	4.06597097
804.417483	10.07312272	772.909512	4.06020185
804.984357	10.07082086	773.192948	4.05955768
805.61076	10.06828163	774.417483	4.05440179
806.208892	10.06586136	774.984357	4.05250506
807.909512	10.05900344	775.61076	4.0503451
808.192948	10.05786385	776.208892	4.04826467
809.417483	10.05295133	777.909512	4.04230796
809.984357	10.05068313	778.192948	4.0412977
810.61076	10.04818109	779.417483	4.03695902
811.208892	10.04579617	779.984357	4.03497412
812.909512	10.03903754	780.61076	4.03278229
813.192948	10.03791426	781.208892	4.03052854
814.417483	10.03307169	782.909512	4.02469828
814.984357	10.03083558	783.192948	4.02371192
815.61076	10.02836879	784.417483	4.01943782
816.208892	10.02601741	784.984357	4.01745925
817.909512	10.01935344	785.61076	4.01527429
818.192948	10.01824587	786.208892	4.01318932
819.417483	10.01347089	787.909512	4.00726713
819.984357	10.01126593	788.192948	4.00628022
820.61076	10.00883347	789.417483	4.00200481
821.208892	10.00651471	789.984357	4.00003439
822.909512	9.999942959	790.61076	3.99786725
823.192948	9.998850652	791.208892	3.99579826
824.417483	9.994141294	792.909512	3.98991571
824.984357	9.991966532	793.192948	3.98893591
825.61076	9.989567321	794.417483	3.98470584
826.208892	9.9872802	794.984357	3.98274929
827.909512	9.980797616	795.61076	3.9805885
828.192948	9.979720078	796.208892	3.97852639
829.417483	9.975074234	797.909512	3.97267016
829.984357	9.972928707	798.192948	3.97169534
830.61076	9.970561609	799.417483	3.9674882
831.208892	9.968305064	799.984357	3.96554283
832.909512	9.961908807	800.61076	3.96339465
833.192948	9.960845559	801.208892	3.96134483
834.417483	9.956261151	802.909512	3.95552404
834.984357	9.954143905	803.192948	3.95455495

Final State: j’=2		Final State: j’=0	
E(cm⁻¹)	σ (Å²)	E(cm⁻¹)	σ (Å²)
835.61076	9.951807961	804.417483	3.95037156
836.208892	9.949581004	804.984357	3.94843681
837.909512	9.943268287	805.61076	3.94630027
838.192948	9.942218863	806.208892	3.94426153
839.417483	9.937693907	807.909512	3.93847224
839.984357	9.935603992	808.192948	3.93750842
840.61076	9.933298161	809.417483	3.93334788
841.208892	9.931099836	809.984357	3.93142375
842.909512	9.924867862	810.61076	3.92929893
843.192948	9.923831825	811.208892	3.92727139
844.417483	9.919364353	812.909512	3.92151381
844.984357	9.917300886	813.192948	3.92055525
845.61076	9.915024164	814.417483	3.91641732
846.208892	9.912853526	814.984357	3.91450359
847.909512	9.906699662	815.61076	3.91239024
848.192948	9.905676572	816.208892	3.91037362
849.417483	9.901264648	817.909512	3.90464695
849.984357	9.899226761	818.192948	3.90369352
850.61076	9.896978155	819.417483	3.89957773
851.208892	9.894834287	819.984357	3.89767422
852.909512	9.888755872	820.61076	3.89557215
853.192948	9.887745245	821.208892	3.89356626
854.417483	9.88338706	822.909512	3.88787005
854.984357	9.88137388	823.192948	3.88692168
855.61076	9.879152465	824.417483	3.88282769
856.208892	9.87703445	824.984357	3.88093424
857.909512	9.871028994	825.61076	3.87884326
858.192948	9.870030445	826.208892	3.87684797
859.417483	9.86572412	827.909512	3.87118173
859.984357	9.863734785	828.192948	3.87023832
860.61076	9.861539668	829.417483	3.86616581
861.208892	9.859446619	829.984357	3.86428229
862.909512	9.853511606	830.61076	3.86220226
863.192948	9.852524726	831.208892	3.86021736
864.417483	9.848268533	832.909512	3.85458067
864.984357	9.846302288	833.192948	3.85364219
865.61076	9.844132493	834.417483	3.84959083
866.208892	9.842063596	834.984357	3.84771709
867.909512	9.836196631	835.61076	3.84564785
868.192948	9.835221007	836.208892	3.84367325
869.417483	9.831013208	837.909512	3.83806571
869.984357	9.829069244	838.192948	3.83713209
870.61076	9.826924017	839.417483	3.83310165

Final State: j'=2		Final State: j'=0	
E(cm⁻¹)	σ (Å²)	E(cm⁻¹)	σ (Å²)
871.208892	9.824878409	839.984357	3.83123756
872.909512	9.819077158	840.61076	3.82917899
873.192948	9.818112403	841.208892	3.82721456
874.417483	9.813951387	842.909512	3.82163583
874.984357	9.812028921	843.192948	3.820707
875.61076	9.809907358	844.417483	3.81669722
876.208892	9.807884254	844.984357	3.8148427
877.909512	9.802146508	845.61076	3.81279466
878.192948	9.801192281	846.208892	3.81084029
879.417483	9.797076433	847.909512	3.8052901
879.984357	9.795174777	848.192948	3.804366
880.61076	9.793076055	849.417483	3.80037671
881.208892	9.791074715	849.984357	3.79853161
882.909512	9.785398312	850.61076	3.79649402
883.192948	9.784454225	851.208892	3.79454961
884.417483	9.780382016	852.909512	3.78902769
884.984357	9.778500452	853.192948	3.78810829
885.61076	9.776423825	854.417483	3.78413928
886.208892	9.774443469	854.984357	3.78230359
887.909512	9.768826325	855.61076	3.78027635
888.192948	9.767892068	856.208892	3.77834182
889.417483	9.763861978	857.909512	3.77284793
889.984357	9.761999781	858.192948	3.7719332
890.61076	9.759944518	859.417483	3.76798434
891.208892	9.75798449	859.984357	3.76615796
892.909512	9.752424557	860.61076	3.764141
893.192948	9.751499764	861.208892	3.76221628
894.417483	9.747510403	862.909512	3.75675025
894.984357	9.745666912	863.192948	3.75584017
895.61076	9.743632252	864.417483	3.75191133
896.208892	9.741691774	864.984357	3.75009421
897.909512	9.736187123	865.61076	3.74808747
898.192948	9.735271453	866.208892	3.7461725
899.417483	9.731321348	867.909512	3.7407342
899.984357	9.729495935	868.192948	3.73982872
900.61076	9.727481156	869.417483	3.73591979
901.208892	9.725559569	869.984357	3.73411188
902.909512	9.720108092	870.61076	3.73211534
903.192948	9.719201232	871.208892	3.73021009
904.417483	9.715288939	872.909512	3.72479936
904.984357	9.713480863	873.192948	3.72389847
905.61076	9.711485158	874.417483	3.72000939
906.208892	9.709581673	874.984357	3.71821066

Final State: j'=2		Final State: j'=0	
E(cm⁻¹)	σ (Å²)	E(cm⁻¹)	σ (Å²)
907.909512	9.704181206	875.61076	3.71622424
908.192948	9.703282773	876.208892	3.71432866
909.417483	9.699406512	877.909512	3.70894545
909.984357	9.697614966	878.192948	3.70804915
910.61076	9.695637393	879.417483	3.70417984
911.208892	9.693751097	879.984357	3.70239027
912.909512	9.68839861	880.61076	3.70041396
913.192948	9.687508055	881.208892	3.69852805
914.417483	9.683665231	882.909512	3.69317226
914.984357	9.681888853	883.192948	3.69228054
915.61076	9.679927725	884.417483	3.68843098
916.208892	9.678056845	884.984357	3.68665041
917.909512	9.67274577	885.61076	3.68468433
918.192948	9.671861648	886.208892	3.68280806
919.417483	9.668044534	887.909512	3.67747966
919.984357	9.666278365	888.192948	3.6765925
920.61076	9.664326641	889.417483	3.67276267
921.208892	9.662461955	889.984357	3.67099136
922.909512	9.657132957	890.61076	3.66903525
923.192948	9.656231981	891.208892	3.66716859
924.417483	9.65216609	892.909512	3.66186756
924.984357	9.650601245	893.192948	3.66098496
925.61076	9.649012647	894.417483	3.65717483
926.208892	9.646744821	894.984357	3.65541264
927.909512	9.641851867	895.61076	3.6534666
928.192948	9.640731093	896.208892	3.65160956
929.417483	9.637564951	897.909512	3.64633586
929.984357	9.635581516	898.192948	3.64545782
930.61076	9.633909694	899.417483	3.64166735
931.208892	9.631970744	899.984357	3.63991427
932.909512	9.626906615	900.61076	3.63797829
933.192948	9.625785994	901.208892	3.63613083
934.417483	9.622394541	902.909512	3.63088443
934.984357	9.620584619	903.192948	3.63001091
935.61076	9.618558056	904.417483	3.62624006
936.208892	9.617038833	904.984357	3.62449605
937.909512	9.612061414	905.61076	3.62257006
938.192948	9.611146649	906.208892	3.62073217
939.417483	9.607309118	907.909512	3.61551285
939.984357	9.605559367	908.192948	3.61464385
940.61076	9.603518304	909.417483	3.6108924
941.208892	9.602016768	909.984357	3.60915734
942.909512	9.597070231	910.61076	3.60724125

Final State: j’=2		Final State: j’=0	
E(cm⁻¹)	σ (Å²)	E(cm⁻¹)	σ (Å²)
943.192948	9.596165509	911.208892	3.60541277
944.417483	9.592428644	912.909512	3.60022001
944.984357	9.590720794	913.192948	3.59935539
945.61076	9.58884252	914.417483	3.59562278
946.208892	9.587183948	914.984357	3.59389632
947.909512	9.582272601	915.61076	3.59198965
948.192948	9.581449552	916.208892	3.59017005
949.417483	9.57765513	917.909512	3.5850017
949.984357	9.575964058	918.192948	3.58414099
950.61076	9.574104651	919.417483	3.58042433
951.208892	9.572337384	919.984357	3.5787046
952.909512	9.56725922	920.61076	3.57680454
953.192948	9.566224213	921.208892	3.57499021
954.417483	9.563002521	922.909512	3.56982157
954.984357	9.561254879	923.192948	3.56895514
955.61076	9.559376834	924.417483	3.56515271
956.208892	9.557585511	924.984357	3.56353334
957.909512	9.552128198	925.61076	3.56176833
958.192948	9.551090145	926.208892	3.55981091
959.417483	9.548090692	927.909512	3.55480927
959.984357	9.546368079	928.192948	3.55386551
960.61076	9.54493007	929.417483	3.55042676
961.208892	9.542663454	929.984357	3.54862981
962.909512	9.538366864	930.61076	3.54683717
963.192948	9.537406325	931.208892	3.54500047
964.417483	9.534175715	932.909512	3.53997335
964.984357	9.532715246	933.192948	3.53901844
965.61076	9.530750477	934.417483	3.53549219
966.208892	9.528903656	934.984357	3.53375739
967.909512	9.524422908	935.61076	3.53182804
968.192948	9.52358597	936.208892	3.53016229
969.417483	9.519740722	937.909512	3.52521464
969.984357	9.517676383	938.192948	3.52433119
970.61076	9.516912075	939.417483	3.52062011
971.208892	9.515099868	939.984357	3.51891273
972.909512	9.509953749	940.61076	3.51700601
973.192948	9.509121478	941.208892	3.51538404
974.417483	9.5066051	942.909512	3.51039774
974.984357	9.504086518	943.192948	3.50953183
975.61076	9.502083456	944.417483	3.50587324
976.208892	9.500270009	944.984357	3.50418897
977.909512	9.495525994	945.61076	3.50232706
978.192948	9.494939431	946.208892	3.50061727

Final State: j’=2		Final State: j’=0	
E(cm^{-1})	σ (Å^2)	E(cm^{-1})	σ (Å^2)
979.417483	9.491891352	947.909512	3.49570961
979.984357	9.489784071	948.192948	3.49488987
980.61076	9.48801379	949.417483	3.49120793
981.208892	9.486840463	949.984357	3.48953592
982.909512	9.482202913	950.61076	3.48769242
983.192948	9.480634216	951.208892	3.48593439
984.417483	9.477649393	952.909512	3.4809789
984.984357	9.476222772	953.192948	3.4800507
985.61076	9.474291966	954.417483	3.47663672
986.208892	9.472380999	954.984357	3.47493861
988.192948	9.467416157	955.61076	3.4730907
989.417483	9.463773162	956.208892	3.47132874
989.984357	9.462227234	957.909512	3.46619509
990.61076	9.460545388	958.192948	3.46533583
991.208892	9.459008562	959.417483	3.46197101
992.909512	9.454240413	959.984357	3.460351
993.192948	9.453398811	960.61076	3.4586653
994.417483	9.450094269	961.208892	3.45673464
994.984357	9.448564344	962.909512	3.45203815
995.61076	9.446875415	963.192948	3.45115586
996.208892	9.445267663	964.417483	3.44781319
997.909512	9.440674493	964.984357	3.44620207
998.192948	9.439906007	965.61076	3.44431436
999.417483	9.436620901	966.208892	3.44251524
999.984357	9.435191192	967.909512	3.43786948
1000.61076	9.433297229	968.192948	3.43705866
1001.20889	9.431716999	969.417483	3.43337152
1002.90951	9.427176233	969.984357	3.4315741
1003.19295	9.426417511	970.61076	3.43021291
1004.41748	9.423141984	971.208892	3.42847902
1004.98436	9.421627792	972.909512	3.42346457
1005.61076	9.41995653	973.192948	3.42263905
1006.20889	9.41836177	974.417483	3.41948824
1007.90951	9.413841814	974.984357	3.41762264
1008.19295	9.413084764	975.61076	3.41574306
1009.41748	9.409817116	976.208892	3.41403897
1010.61076	9.406658904	977.909512	3.40919788
1011.20889	9.405078292	978.192948	3.40842295
1012.90951	9.40059283	979.417483	3.40520525
1013.19295	9.399846533	979.984357	3.40335193
1014.41748	9.39662663	980.61076	3.40164987
1014.98436	9.395138335	981.208892	3.4001036
1015.61076	9.393495359	982.909512	3.39531808

Final State: j’=2		Final State: j’=0	
E(cm⁻¹)	σ (Å²)	E(cm⁻¹)	σ (Å²)
1016.20889	9.391927947	983.192948	3.39425382
1017.90951	9.387479863	984.417483	3.39107721
1018.19295	9.386739923	984.984357	3.38959781
1019.41748	9.383548047	985.61076	3.38766623
1019.98436	9.382073109	986.208892	3.38586079
1020.61076	9.380445175	987.909512	3.38131864
1021.20889	9.378892492	988.192948	3.3805432
1022.90951	9.374487637	989.417483	3.37705969
1023.19295	9.373754856	989.984357	3.37548159
1024.41748	9.37059357	990.61076	3.37375046
1024.98436	9.369132611	991.208892	3.37212226
1025.61076	9.367520015	992.909512	3.36740501
1026.20889	9.365981987	993.192948	3.36659883
1027.90951	9.361618669	994.417483	3.36321585
1029.41748	9.357761596	994.984357	3.36165507
1029.98436	9.356314581	995.61076	3.35993244
1031.20889	9.353194273	996.208892	3.35829075
1032.90951	9.348873243	997.909512	3.35362633
1034.41748	9.34505385	998.192948	3.35284786
1034.98436	9.343620985	999.417483	3.34949742
1035.61076	9.342039561	999.984357	3.34798261
1036.20889	9.340531346	1000.61076	3.34620988
1037.90951	9.336252976	1001.20889	3.34457774
1039.41748	9.332471451	1002.90951	3.33994218
1039.98436	9.331052887	1003.19295	3.33917028
1040.61076	9.329487247	1004.41748	3.33583858
1041.20889	9.327994114	1004.98436	3.33429816
1042.90951	9.323758736	1005.61076	3.33259752
1044.41748	9.320015483	1006.20889	3.33097467
1044.98436	9.318611331	1007.90951	3.3263716
1045.61076	9.317061625	1008.19295	3.32560412
1046.20889	9.315583745	1009.41748	3.3222902
1049.41748	9.30768713	1009.98436	3.32076056
1049.98436	9.30629751	1010.61076	3.319072
1050.61076	9.304763909	1011.20889	3.31746115
1051.20889	9.30330139	1012.90951	3.31288888
1052.90951	9.299153218	1013.19295	3.31212797
1054.41748	9.295487538	1014.41748	3.3088443
1054.98436	9.294112569	1014.98436	3.30732622
1055.61076	9.292595184	1015.61076	3.3056502
1056.20889	9.291148161	1016.20889	3.30405118
1057.90951	9.287044186	1017.90951	3.29951241
1059.41748	9.283417747	1018.19295	3.29875709

Final State: j’=2		Final State: j’=0	
E(cm⁻¹)	σ (Å²)	E(cm⁻¹)	σ (Å²)
1059.98436	9.282057569	1019.41748	3.29549778
1060.61076	9.280556515	1019.98436	3.29399108
1061.20889	9.279125136	1020.61076	3.29232772
1062.90951	9.275065672	1021.20889	3.29074094
1064.41748	9.27147879	1022.90951	3.28623747
1064.98436	9.270133522	1023.19295	3.28548805
1065.61076	9.268648967	1024.41748	3.28225415
1066.20889	9.267233322	1024.98436	3.28075917
1067.90951	9.263218705	1025.61076	3.27910874
1069.41748	9.259671686	1026.20889	3.27753433
1069.98436	9.258341427	1027.90951	3.27306601
1070.61076	9.256873439	1029.41748	3.26911396
1071.20889	9.255473677	1029.98436	3.26763078
1072.90951	9.251504212	1030.61076	3.26599341
1074.41748	9.247997294	1031.20889	3.26443148
1074.98436	9.246682136	1032.90951	3.2599988
1075.61076	9.245230846	1034.41748	3.25607846
1076.20889	9.243847013	1034.98436	3.25460723
1077.90951	9.239922964	1035.61076	3.25298307
1079.41748	9.236456401	1036.20889	3.25143377
1079.98436	9.235156406	1037.90951	3.24703707
1080.61076	9.233721911	1039.41748	3.24314874
1081.20889	9.232354101	1039.98436	3.24168956
1084.41748	9.22504964	1040.61076	3.24007874
1084.98436	9.223764888	1041.20889	3.23854221
1085.61076	9.222347236	1042.90951	3.23418185
1086.20889	9.220995555	1044.41748	3.23032584
1089.41748	9.213777546	1044.98436	3.22887883
1089.98436	9.212513554	1045.61076	3.22728149
1090.61076	9.211107328	1046.20889	3.22575782
1091.20889	9.209771781	1047.90951	3.22143415
1094.41748	9.202640442	1049.41748	3.21761077
1094.98436	9.201386326	1049.98436	3.21617605
1095.61076	9.200002516	1050.61076	3.21459229
1096.20889	9.198683142	1051.20889	3.21308162
1099.41748	9.19163859	1052.90951	3.20879497
1099.98436	9.190399783	1054.41748	3.20500453
1101.20889	9.187729715	1054.98436	3.20358222
1104.41748	9.180771923	1055.61076	3.20201219
1104.98436	9.179548432	1056.20889	3.20051461
1106.20889	9.176911466	1057.90951	3.19626533
1109.41748	9.170040242	1059.41748	3.1925081
1109.98436	9.168832043	1059.98436	3.19109828

Final State: j’=2		Final State: j’=0	
E(cm⁻¹)	σ (Å²)	E(cm⁻¹)	σ (Å²)
1111.20889	9.166228049	1060.61076	3.18954208
1114.41748	9.159443122	1061.20889	3.18805773
1114.98436	9.158250146	1062.90951	3.18384609
1116.20889	9.155679018	1064.41748	3.18012234
1119.41748	9.148979921	1064.98436	3.17872513
1119.98436	9.147802069	1065.61076	3.17718287
1121.20889	9.14526359	1066.20889	3.17571186
1124.41748	9.138649724	1067.90951	3.17153819
1124.98436	9.137486894	1069.41748	3.16784815
1126.20889	9.134980736	1069.98436	3.16646367
1129.41748	9.128451319	1070.61076	3.16493545
1129.98436	9.127303341	1071.20889	3.16347787
1131.20889	9.124829244	1072.90951	3.15934248
1134.41748	9.118383326	1074.41748	3.15568641
1136.20889	9.114807576	1074.98436	3.15431469
1139.41748	9.108444042	1075.61076	3.15280063
1139.98436	9.107325229	1076.20889	3.15135655
1141.20889	9.104913918	1077.90951	3.14725963
1144.41748	9.098631545	1079.41748	3.14363781
1144.98436	9.097526937	1079.98436	3.14227898
1146.20889	9.095146313	1080.61076	3.14077915
1149.41748	9.088943585	1081.20889	3.13934871
1149.98436	9.087853005	1084.41748	3.13170313
1151.20889	9.085502447	1084.98436	3.13035726
1154.41748	9.079377884	1085.61076	3.12887176
1154.98436	9.078300973	1086.20889	3.127455
1156.20889	9.075979936	1089.41748	3.11988302
1159.41748	9.069932007	1089.98436	3.11855488
1159.98436	9.068868538	1090.61076	3.11707908
1161.20889	9.066576497	1091.20889	3.11567608
1164.41748	9.060604518	1094.41748	3.1081781
1166.20889	9.05729213	1094.98436	3.10685836
1169.41748	9.051411064	1095.61076	3.10540175
1171.20889	9.048293189	1096.20889	3.10401259
1174.41748	9.042365902	1099.41748	3.09658894
1176.20889	9.039122071	1099.98436	3.09528234
1179.41748	9.033354557	1101.20889	3.09246501
1181.20889	9.030033723	1104.41748	3.085116
1184.41748	9.024300085	1104.98436	3.08382263
1186.20889	9.02115213	1106.20889	3.08103384
1189.41748	9.015495019	1109.41748	3.07375974
1191.20889	9.012368624	1109.98436	3.07247962
1194.41748	9.006760415	1111.20889	3.06971948

Final State: j’=2		Final State: j’=0	
E(cm⁻¹)	σ (Å²)	E(cm⁻¹)	σ (Å²)
1196.20889	9.003684675	1114.41748	3.06252051
1199.41748	8.998208019	1114.98436	3.06125367
1201.20889	8.995090149	1116.20889	3.05852223
1204.41748	8.989902299	1119.41748	3.05139859
1206.20889	8.986797752	1119.98436	3.05014506
1209.41748	8.981149775	1121.20889	3.04744238
1211.20889	8.978075228	1124.41748	3.04039418
1214.41748	8.972679835	1124.98436	3.03915399
1216.20889	8.96979585	1126.20889	3.03648014
1219.41748	8.964322159	1129.41748	3.02950746
1221.20889	8.961460935	1129.98436	3.02828062
1224.41748	8.956018203	1131.20889	3.02563559
1226.20889	8.953133316	1134.41748	3.01873848
1229.41748	8.94796608	1134.98436	3.01752499
1231.20889	8.944962401	1136.20889	3.01490881
1234.41748	8.939757112	1139.41748	3.00808726
1236.20889	8.936787591	1139.98436	3.00688711
1239.41748	8.931585861	1141.20889	3.00429976
1241.20889	8.928646795	1144.41748	2.997553708
1244.41748	8.923428815	1144.98436	2.996366888
1246.20889	8.920502441	1146.20889	2.99380834
1249.41748	8.915335571	1149.41748	2.98713771
1251.20889	8.912438034	1149.98436	2.985964213
1254.41748	8.907255095	1151.20889	2.983434412
1256.20889	8.904365296	1154.41748	2.976839087
1259.41748	8.899187164	1154.98436	2.975678882
1261.20889	8.896297526	1156.20889	2.973177807
1264.41748	8.891122918	1159.41748	2.966657704
1266.20889	8.88823325	1159.98436	2.965510789
1269.41748	8.883055592	1161.20889	2.963038427
1271.20889	8.880163271	1164.41748	2.956593771
1276.20889	8.872081328	1166.20889	2.953016994
1281.20889	8.863980515	1169.41748	2.946653129
1286.20889	8.855854218	1171.20889	2.943140929
1291.20889	8.84769578	1174.41748	2.936829469
1296.20889	8.839498775	1176.20889	2.933328007
1301.20889	8.831256918	1179.41748	2.927102613
1306.20889	8.822964047	1181.20889	2.92362421
1311.20889	8.81461422	1184.41748	2.917462487
1316.20889	8.806201765	1186.20889	2.914057101
1321.20889	8.797721088	1189.41748	2.907974003
1326.20889	8.789167006	1191.20889	2.904604304
1331.20889	8.780534484	1194.41748	2.898589303

Final State: j'=2		Final State: j'=0	
E(cm⁻¹)	σ (Å²)	E(cm⁻¹)	σ (Å²)
1336.20889	8.77181885	1196.20889	2.895267231
1341.20889	8.763015603	1199.41748	2.889332283
1346.20889	8.754120694	1201.20889	2.886044386
1351.20889	8.745130272	1204.41748	2.880229301
1356.20889	8.736040891	1206.20889	2.876956025
1361.20889	8.726849353	1209.41748	2.87113453
1366.20889	8.717552899	1211.20889	2.867896041
1371.20889	8.708149035	1214.41748	2.862165825
1376.20889	8.698635643	1216.20889	2.859014508
1381.20889	8.689011002	1219.41748	2.853344173
1386.20889	8.679273652	1221.20889	2.85024239
1391.20889	8.669422595	1224.41748	2.844607982
1396.20889	8.659457067	1226.20889	2.841536758
1401.20889	8.649376756	1229.41748	2.836010541
1406.20889	8.639181654	1231.20889	2.832937537
		1234.41748	2.827497373
		1236.20889	2.82445969
		1239.41748	2.819076918
		1241.20889	2.816077283
		1244.41748	2.8107481
		1246.20889	2.807787424
		1249.41748	2.802531923
		1251.20889	2.799611125
		1254.41748	2.794411676
		1256.20889	2.791526682
		1259.41748	2.786388399
		1261.20889	2.783536833
		1264.41748	2.77845991
		1266.20889	2.77564207
		1269.41748	2.770624759
		1271.20889	2.767840042
		1276.20889	2.760129522
		1281.20889	2.752508995
		1286.20889	2.744976998
		1291.20889	2.737532091
		1296.20889	2.730172875
		1301.20889	2.72289794
		1306.20889	2.715705933
		1311.20889	2.708595531
		1316.20889	2.701565442
		1321.20889	2.694614409
		1326.20889	2.687741241
		1331.20889	2.680944799

Final State: j'=0

E(cm^{-1})	σ (Å^2)
1336.20889	2.674223979
1341.20889	2.667577741
1351.20889	2.654505162
1356.20889	2.648077034
1361.20889	2.641719932
1366.20889	2.635433146
1371.20889	2.629216006
1376.20889	2.623067943
1381.20889	2.616988301
1386.20889	2.610976732
1391.20889	2.60503281
1396.20889	2.599156189
1401.20889	2.593346526
1406.20889	2.587603674

Initial rotational state: j=0					
Final State: j’=5		Final State: j’=4		Final State: j’=3	
E(cm⁻¹)	σ (Å²)	E(cm⁻¹)	σ (Å²)	E(cm⁻¹)	σ (Å²)
410.698132	0.014824375	273.906723	0.045160084	164.473597	0.11890336
410.798132	0.027937671	274.006723	0.08412958	164.573597	0.224034441
410.898132	0.020969514	274.106723	0.064010443	164.673597	0.172349952
410.998132	0.022095676	274.206723	0.069168854	164.773597	0.191525214
411.098132	0.0231088	274.306723	0.072933244	164.873597	0.20337035
411.198132	0.026372866	274.373597	0.075985877	164.973597	0.232548967
411.298132	0.02983276	274.406723	0.081182696	165	0.25628053
411.398132	0.031242116	274.506723	0.095019345	165.073597	0.268917221
411.498132	0.029630063	274.606723	0.092271593	165.173597	0.25541521
411.598132	0.033238402	274.706723	0.09137975	165.273597	0.258263038
411.698132	0.032625629	274.806723	0.101902075	165.373597	0.288107399
411.798132	0.031855112	274.906723	0.098539084	165.473597	0.2742081
411.898132	0.03189711	275	0.098136178	165.573597	0.276326225
411.998132	0.032151087	275.006723	0.098171348	167.298752	0.51706039
412.098132	0.032464095	275.106723	0.099069926	167.582188	0.51479318
412.198132	0.032775722	275.206723	0.100285443	169.373597	0.537614276
412.298752	0.033060746	277.298752	0.157907566	169.473597	0.542833041
412.582188	0.033607716	277.582188	0.154967935	169.573597	0.547139573
413.806723	0.051173129	278.806723	0.169060922	169.673597	0.550413229
414.373597	0.04816477	278.906723	0.170489699	169.773597	0.552496525
415	0.049144585	279.006723	0.171738847	169.873597	0.553085488
415.598132	0.051865806	279.106723	0.172790063	169.973597	0.551533801
415.698132	0.052269774	279.206723	0.173627003	170	0.550642935
415.798132	0.052629407	279.306723	0.174219167	170.073597	0.547108115
415.898132	0.052939946	279.373597	0.174441974	172.298752	0.697124607
415.998132	0.05319811	279.406723	0.174484581	172.582188	0.688518838
416.098132	0.053398999	279.506723	0.174252837	174.373597	0.714383562
416.198132	0.053528341	279.606723	0.174199344	175	0.764018834
416.298132	0.053549432	279.706723	0.177096938	177.298752	0.849349926
416.398132	0.053717211	280	0.183832311	177.582188	0.843846878
416.498132	0.054070509	282.298752	0.204843206	179.373597	1.083351018
416.598132	0.054347934	282.582188	0.205465498	180	0.87941074
416.698132	0.055273548	283.806723	0.212230853	182.298752	0.906085609
417.298752	0.068144292	284.373597	0.220294841	182.582188	0.908502496
417.582188	0.076083042	285	0.235926922	184.373597	0.924097739
418.806723	0.062097936	287.298752	0.240515217	185	0.932401022
419.373597	0.06239153	287.582188	0.241391905	187.298752	1.092634079
420	0.063193789	288.806723	0.283301489	187.582188	1.078765013
420.598132	0.064379118	289.373597	0.249997645	189.373597	1.007898151
422.298752	0.072364112	290	0.251378211	190	1.004391803
422.582188	0.07262482	292.298752	0.26059273	192.298752	1.010375752
423.806723	0.072243572	292.582188	0.261527186	192.582188	1.012349417

Final State: j'=5		Final State: j'=4		Final State: j'=3	
E(cm⁻¹)	σ (Å²)	E(cm⁻¹)	σ (Å²)	E(cm⁻¹)	σ (Å²)
424.373597	0.072670658	293.806723	0.265721101	194.373597	1.033123789
425	0.081010723	294.373597	0.267922726	195	1.043145966
425.598132	0.082606482	295	0.270951387	197.298752	1.064890425
427.298752	0.076778545	297.298752	0.289422608	197.582188	1.06594204
427.582188	0.077197905	297.582188	0.28811509	199.373597	1.071745952
428.806723	0.078826547	298.806723	0.287433762	200	1.074116403
429.373597	0.079528548	299.373597	0.288451506	202.298752	1.085661122
430	0.080306741	300	0.289925731	202.582188	1.087392203
430.598132	0.081071108	302.298752	0.296782808	204.373597	1.099349859
432.298752	0.083779612	302.582188	0.297763731	205	1.103703155
432.582188	0.084560055	303.806723	0.30244741	207.298752	1.119103054
433.806723	0.087664312	304.373597	0.3047896	207.582188	1.12092531
434.373597	0.087586166	305	0.307311374	209.373597	1.13243389
435	0.087837616	307.298752	0.314849244	210	1.136631316
435.598132	0.088336037	307.582188	0.315682169	212.298752	1.145244059
437.298752	0.090228217	308.806723	0.319291112	212.582188	1.140674715
437.582188	0.090572941	309.373597	0.320989945	214.373597	1.14171303
438.806723	0.092119638	310	0.322895778	215	1.142426938
439.373597	0.092869184	312.298752	0.330158967	217.298752	1.144339918
440	0.093727814	312.582188	0.331080518	217.582188	1.146394773
440.598132	0.094578318	313.806723	0.335100204	219.373597	1.170648566
442.298752	0.097019892	314.373597	0.336970828	220	1.155727848
442.582188	0.097408312	315	0.3390365	222.298752	1.176062623
443.806723	0.099030263	317.298752	0.346554005	222.582188	1.182967611
444.373597	0.099768391	317.582188	0.347473617	224.373597	1.164646075
445	0.100585185	318.806723	0.351435321	225	1.189776865
445.598132	0.101369654	319.373597	0.353265764	227.298752	1.209068769
447.298752	0.103635142	320	0.355287829	227.582188	1.211742829
447.582188	0.104018247	322.298752	0.362718812	229.373597	1.194137333
448.806723	0.105691582	322.582188	0.363636977	230	1.214840711
449.373597	0.106475664	323.806723	0.367608451	232.298752	1.232383247
450	0.107348143	324.373597	0.369449018	232.582188	1.234848964
450.598132	0.108186265	325	0.371483774	234.373597	1.209628403
452.298752	0.110588154	327.298752	0.37895036	235	1.222926579
452.582188	0.110990458	327.582188	0.379870162	237.298752	1.254032719
453.806723	0.112733841	328.806723	0.383840064	237.582188	1.2567776
454.373597	0.113543899	329.373597	0.385675252	239.373597	1.269333997
455	0.11444145	330	0.387700923	240	1.265823718
455.598132	0.115301086	332.298752	0.395110343	242.298752	1.263959722
457.298752	0.117760811	332.582188	0.396020814	242.582188	1.276079897
457.582188	0.118173195	333.806723	0.39994473	244.373597	1.298943916
458.806723	0.119963292	334.373597	0.401755253	245	1.305997728
459.373597	0.120796732	335	0.4037508	247.298752	1.263042512

Final State: j'=5		Final State: j'=4		Final State: j'=3	
E(cm⁻¹)	σ (Å²)	E(cm⁻¹)	σ (Å²)	E(cm⁻¹)	σ (Å²)
460	0.121721225	337.298752	0.411015544	247.582188	1.264135082
460.598132	0.122607563	337.582188	0.411903335	249.373597	1.298202982
462.298752	0.125145187	338.806723	0.415712428	250	1.290801626
462.582188	0.125570694	339.373597	0.41745864	252.298752	1.294027123
463.806723	0.127417177	340	0.419372881	252.582188	1.289419137
464.373597	0.12827639	342.298752	0.426205379	254.373597	1.320688693
465	0.129229041	342.582188	0.427019004	255	1.269300365
465.598132	0.130141814	343.806723	0.430422302	257.298752	1.339263362
467.298752	0.132753534	344.373597	0.431915957	257.582188	1.343852307
467.582188	0.133191186	345	0.433479513	259.373597	1.323359715
468.806723	0.135089729	347.298752	0.437434145	260	1.339383875
469.373597	0.135972875	347.582188	0.437442481	262.298752	1.324966042
470	0.136951897	348.806723	0.430532795	262.582188	1.293070124
470.598132	0.137889793	349.373597	0.425933435	264.373597	1.367655321
472.298752	0.140572728	350	0.437323958	265	1.370981317
472.582188	0.141022217	352.298752	0.456058375	267.298752	1.335125185
473.806723	0.142971775	352.582188	0.454495878	267.582188	1.35853601
474.373597	0.143878453	353.806723	0.444375303	269.373597	1.375496058
475	0.144883396	354.373597	0.454124871	270	1.396709202
475.598132	0.145845952	355	0.465286487	272.298752	1.341719823
477.298752	0.148598415	357.298752	0.468144481	272.582188	1.369289655
477.582188	0.149059397	357.582188	0.47380948	273.906723	1.371095696
478.806723	0.151058248	358.806723	0.483868871	274.006723	1.34336197
479.373597	0.15198754	359.373597	0.485886223	274.106723	1.358460699
480	0.15301731	360	0.480893074	274.206723	1.358554322
480.598132	0.154003412	362.298752	0.468341596	274.306723	1.358626432
482.298752	0.156821907	362.582188	0.496663692	274.373597	1.355269251
482.582188	0.157293756	363.806723	0.502799965	274.406723	1.345612115
483.806723	0.159339068	364.373597	0.500388734	274.506723	1.306051111
484.373597	0.160289582	365	0.493009727	274.606723	1.317758065
485	0.161342585	367.298752	0.512652903	274.706723	1.329585713
485.598132	0.162350643	367.582188	0.524231653	274.806723	1.305533006
487.298752	0.16523026	368.806723	0.521498613	274.906723	1.30964738
487.582188	0.165712094	369.373597	0.520381913	275	1.31842312
488.806723	0.167799795	370	0.517641013	275.006723	1.318801568
489.373597	0.168769495	372.298752	0.509233058	275.106723	1.322612738
490	0.169843347	372.582188	0.528471807	275.206723	1.324572437
490.598132	0.170870944	373.806723	0.541847809	277.298752	1.279502831
492.298752	0.173803912	374.373597	0.541176048	277.582188	1.284698441
492.582188	0.174294289	375	0.540657431	278.806723	1.278303059
493.806723	0.176417594	377.298752	0.507394842	278.906723	1.276964385
494.373597	0.177402993	377.582188	0.530678512	279.006723	1.275569922
495	0.178493544	378.806723	0.551648778	279.106723	1.274017316

Final State: j’=5		Final State: j’=4		Final State: j’=3	
E(cm⁻¹)	σ (Å²)	E(cm⁻¹)	σ (Å²)	E(cm⁻¹)	σ (Å²)
495.598132	0.179536403	379.373597	0.561784852	279.206723	1.272079247
497.298752	0.182508503	380	0.559071962	279.306723	1.269247022
497.582188	0.183004699	382.298752	0.545374039	279.373597	1.266282218
498.806723	0.185150405	382.582188	0.539104708	279.406723	1.264235737
499.373597	0.186144462	383.806723	0.53382693	279.506723	1.253468023
500	0.187243125	384.373597	0.527819636	279.606723	1.232993395
500.598132	0.188292118	385	0.540977366	279.706723	1.22518027
502.298752	0.191271057	387.298752	0.583418322	280	1.259644133
502.582188	0.191766541	387.582188	0.58155026	282.298752	1.259527228
503.806723	0.193901261	388.806723	0.551148721	282.582188	1.260268534
504.373597	0.194884989	389.373597	0.583175453	283.806723	1.258778978
505	0.195967374	390	0.590076609	284.373597	1.252967923
505.598132	0.196995088	392.298752	0.5908007	285	1.239592722
507.298752	0.199869979	392.582188	0.581736541	287.298752	1.242121798
507.582188	0.200339405	393.806723	0.609056333	287.582188	1.24136286
508.806723	0.202315567	394.373597	0.598022534	288.806723	1.20233
509.373597	0.203189328	395	0.563509737	289.373597	1.242683025
510	0.204106868	397.298752	0.617108741	290	1.243626334
510.598132	0.204911611	397.582188	0.614880226	292.298752	1.242541476
512.298752	0.206095447	398.806723	0.570703655	292.582188	1.242619673
512.582188	0.205762095	399.373597	0.575778324	293.806723	1.242749256
513.806723	0.202574768	400	0.623869837	294.373597	1.242482887
514.373597	0.210419914	402.298752	0.609751593	295	1.241457087
515	0.210037367	402.582188	0.612267468	297.298752	1.23101057
515.598132	0.209347211	403.806723	0.609015049	297.582188	1.233687648
517.298752	0.210717523	404.373597	0.650400402	298.806723	1.239343221
517.582188	0.210605182	405	0.611601876	299.373597	1.240444385
518.806723	0.21959949	407.298752	0.613673188	300	1.241270099
519.373597	0.218943541	407.582188	0.620624683	302.298752	1.242643564
520	0.224273444	408.806723	0.647636185	302.582188	1.242654081
520.598132	0.221168429	409.373597	0.640760907	303.806723	1.2421988
522.298752	0.223795126	410	0.672314644	304.373597	1.241812968
522.582188	0.223214354	410.698132	0.646674342	305	1.241489952
523.806723	0.232410344	410.798132	0.622326861	307.298752	1.242299011
524.373597	0.230380755	410.898132	0.636750568	307.582188	1.242496942
525	0.224976663	410.998132	0.630727305	308.806723	1.243309064
525.598132	0.231379408	411.098132	0.620443778	309.373597	1.243638789
527.298752	0.242804709	411.198132	0.594458077	310	1.243962137
527.582188	0.242514866	411.298132	0.606658589	312.298752	1.244785667
528.806723	0.241251073	411.398132	0.625885625	312.582188	1.244853319
529.373597	0.237699034	411.498132	0.624331734	313.806723	1.245096623
530	0.238044197	411.598132	0.603422934	314.373597	1.24519597
530.598132	0.244053297	411.698132	0.632788842	315	1.245305939

Final State: j'=5		Final State: j'=4		Final State: j'=3	
E(cm⁻¹)	σ (Å²)	E(cm⁻¹)	σ (Å²)	E(cm⁻¹)	σ (Å²)
532.298752	0.253180343	411.798132	0.634689914	317.298752	1.245761117
532.582188	0.253097421	411.898132	0.633148044	317.582188	1.245822429
533.806723	0.253037353	411.998132	0.63113526	318.806723	1.246089993
534.373597	0.252213927	412.098132	0.628930594	319.373597	1.246211944
535	0.246710177	412.198132	0.626455694	320	1.246342856
535.598132	0.250111189	412.298752	0.623456536	322.298752	1.246770601
537.298752	0.265289991	412.582188	0.605638138	322.582188	1.246816839
537.582188	0.264799817	413.806723	0.623250731	323.806723	1.247000865
538.806723	0.264897943	414.373597	0.625590842	324.373597	1.247078169
539.373597	0.265146453	415	0.622960699	325	1.247158549
540	0.265175898	415.598132	0.618185678	327.298752	1.247418161
540.598132	0.264326406	415.698132	0.617273035	327.582188	1.247447158
542.298752	0.268054861	415.798132	0.616268391	328.806723	1.24756645
542.582188	0.264009901	415.898132	0.615092774	329.373597	1.247618722
543.806723	0.276091188	415.998132	0.613599982	330	1.247674567
544.373597	0.275772202	416.098132	0.611493564	332.298752	1.247864213
545	0.275606978	416.198132	0.608076664	332.582188	1.247886199
545.598132	0.275172953	416.298132	0.601599356	333.806723	1.247978743
547.298752	0.267253633	416.398132	0.594982992	334.373597	1.248020704
547.582188	0.270906999	416.498132	0.597435107	335	1.248066915
548.806723	0.271856197	416.598132	0.60348759	337.298752	1.248243604
549.373597	0.28001509	416.698132	0.607075979	337.582188	1.248267269
550	0.284896677	417.298752	0.607340029	338.806723	1.248378719
550.598132	0.276471357	417.582188	0.605563778	339.373597	1.248437124
552.298752	0.286708762	418.806723	0.626538195	340	1.248508544
552.582188	0.279428045	419.373597	0.626744424	342.298752	1.248869197
553.806723	0.299362307	420	0.62681886	342.582188	1.24892975
554.373597	0.297252755	420.598132	0.626462904	343.806723	1.249258607
555	0.292122566	422.298752	0.621837706	344.373597	1.249461885
555.598132	0.27185839	422.582188	0.622923488	345	1.249742721
557.298752	0.31217246	423.806723	0.627556728	347.298752	1.252009127
557.582188	0.309991266	424.373597	0.627707056	347.582188	1.2526411
558.806723	0.299142027	425	0.612084241	348.806723	1.261456133
559.373597	0.289597986	425.598132	0.625523822	349.373597	1.269531377
560	0.317783725	427.298752	0.636831472	350	1.264792638
560.598132	0.31652864	427.582188	0.637208498	352.298752	1.252364189
562.298752	0.311243364	428.806723	0.639362738	352.582188	1.253548348
562.582188	0.308256449	429.373597	0.640476885	353.806723	1.269899531
563.806723	0.318110996	430	0.641706957	354.373597	1.26211992
564.373597	0.316764093	430.598132	0.642845058	355	1.252645978
565	0.318426711	432.298752	0.645173608	357.298752	1.259790903
565.598132	0.314814978	432.582188	0.645100848	357.582188	1.253892808
567.298752	0.307620246	433.806723	0.646738127	358.806723	1.250351534

Final State: j’=5		Final State: j’=4		Final State: j’=3	
E(cm⁻¹)	σ (Å²)	E(cm⁻¹)	σ (Å²)	E(cm⁻¹)	σ (Å²)
567.582188	0.296515605	434.373597	0.649261771	359.373597	1.247062183
568.806723	0.331677145	435	0.651293499	360	1.252052365
569.373597	0.319928964	435.598132	0.652852626	362.298752	1.273859926
570	0.3373668	437.298752	0.656705892	362.582188	1.251143169
570.598132	0.332439082	437.582188	0.657315232	363.806723	1.244068839
572.298752	0.318490088	438.806723	0.659880307	364.373597	1.247174733
572.582188	0.330124596	439.373597	0.661027043	365	1.255170126
573.806723	0.34732259	440	0.662259717	367.298752	1.249781068
574.373597	0.338548133	440.598132	0.663408531	367.582188	1.238089897
575	0.33843432	442.298752	0.666753989	368.806723	1.240797189
575.598132	0.337899616	442.582188	0.667346222	369.373597	1.242949971
577.298752	0.33268857	443.806723	0.669970031	370	1.246863016
577.582188	0.343816981	444.373597	0.671192433	372.298752	1.264900864
578.806723	0.343306601	445	0.672536053	372.582188	1.248858155
579.373597	0.342889885	445.598132	0.673809131	373.806723	1.235970382
580	0.342111282	447.298752	0.6773723	374.373597	1.237625477
580.598132	0.340106734	447.582188	0.677958387	375	1.239451288
582.298752	0.350541363	448.806723	0.680467915	377.298752	1.279275107
582.582188	0.351028519	449.373597	0.681618907	377.582188	1.258316894
583.806723	0.352444938	450	0.682884701	378.806723	1.242183893
584.373597	0.353218755	450.598132	0.68408888	379.373597	1.231825074
585	0.353980524	452.298752	0.687498982	380	1.234794447
585.598132	0.354514673	452.582188	0.688066105	382.298752	1.249539982
587.298752	0.358191364	453.806723	0.69051252	382.582188	1.256338081
587.582188	0.358842213	454.373597	0.691642485	383.806723	1.271801147
588.806723	0.360345712	455	0.692888701	384.373597	1.27859118
589.373597	0.361635669	455.598132	0.694075912	385	1.266383179
590	0.365113193	457.298752	0.697434033	387.298752	1.237861153
590.598132	0.366426612	457.582188	0.697990981	387.582188	1.238093279
592.298752	0.369603224	458.806723	0.70038765	388.806723	1.269174022
592.582188	0.370197299	459.373597	0.701491857	389.373597	1.248884243
593.806723	0.37280314	460	0.702708154	390	1.239377021
594.373597	0.374012534	460.598132	0.703865681	392.298752	1.25096022
595	0.375341447	462.298752	0.707137971	392.582188	1.264855047
595.598132	0.376593173	462.582188	0.70768063	393.806723	1.229198075
597.298752	0.37997419	463.806723	0.710016491	394.373597	1.238553789
597.582188	0.380685902	464.373597	0.711093168	395	1.272012496
598.806723	0.383813104	465	0.712279531	397.298752	1.231836137
599.373597	0.385104154	465.598132	0.713409046	397.582188	1.234010191
600	0.386504468	467.298752	0.716602901	398.806723	1.276571653
600.598132	0.387834547	467.582188	0.717132674	399.373597	1.285010357
602.298752	0.391610527	468.806723	0.719413099	400	1.23433647
602.582188	0.392239572	469.373597	0.720464166	402.298752	1.247242394

Final State: j'=5		Final State: j'=4		Final State: j'=3	
E(cm⁻¹)	σ (Å²)	E(cm⁻¹)	σ (Å²)	E(cm⁻¹)	σ (Å²)
603.806723	0.394955535	470	0.721622194	402.582188	1.248074398
604.373597	0.396212975	470.598132	0.722724632	403.806723	1.263259765
605	0.397606054	472.298752	0.725841311	404.373597	1.223695912
605.598132	0.398943813	472.582188	0.726358204	405	1.251282099
607.298752	0.402790064	473.806723	0.728582989	407.298752	1.266960362
607.582188	0.403433857	474.373597	0.729608335	407.582188	1.262880704
608.806723	0.406214564	475	0.730738007	408.806723	1.230535761
609.373597	0.407500921	475.598132	0.731813428	409.373597	1.243318037
610	0.408922064	477.298752	0.734853776	410	1.220017766
610.598132	0.410278862	477.582188	0.735358033	410.698132	1.225337442
612.298752	0.414137764	478.806723	0.73752853	410.798132	1.235982187
612.582188	0.414781324	479.373597	0.738528924	410.898132	1.22989122
613.806723	0.417563998	480	0.739631163	410.998132	1.233646701
614.373597	0.418853746	480.598132	0.740680532	411.098132	1.241269392
615	0.420280298	482.298752	0.743647604	411.198132	1.262755591
615.598132	0.421643864	482.582188	0.74413977	411.298132	1.254594837
617.298752	0.425528095	483.806723	0.746258462	411.398132	1.237987721
617.582188	0.426176442	484.373597	0.747235127	411.498132	1.237867546
618.806723	0.428980353	485	0.748311344	411.598132	1.255756713
619.373597	0.430279818	485.598132	0.749336073	411.698132	1.231853127
620	0.431716744	487.298752	0.752234314	411.798132	1.229344988
620.598132	0.433089762	487.582188	0.752715201	411.898132	1.230181464
622.298752	0.436998457	488.806723	0.754785878	411.998132	1.231616654
622.582188	0.437650602	489.373597	0.755740744	412.098132	1.233353261
623.806723	0.440470378	490	0.756793225	412.198132	1.235448085
624.373597	0.44177701	490.598132	0.757795649	412.298752	1.238133603
625	0.443221796	492.298752	0.76063269	412.582188	1.255160698
625.598132	0.444602302	492.582188	0.761103732	413.806723	1.227662004
627.298752	0.448532337	493.806723	0.763133211	414.373597	1.229497517
627.582188	0.449188055	494.373597	0.764069812	415	1.232920939
628.806723	0.452023253	495	0.765102775	415.598132	1.237248505
629.373597	0.453337001	495.598132	0.766087284	415.698132	1.238204386
630	0.454789624	497.298752	0.768877828	415.798132	1.239327729
630.598132	0.456177566	497.582188	0.769341854	415.898132	1.240715944
632.298752	0.460128411	498.806723	0.771343929	415.998132	1.242543533
632.582188	0.460787539	499.373597	0.772269649	416.098132	1.245159063
633.806723	0.463637286	500	0.773292134	416.198132	1.249362554
634.373597	0.464957663	500.598132	0.774268335	416.298132	1.25704022
635	0.46641753	502.298752	0.777046519	416.398132	1.264453087
635.598132	0.467812319	502.582188	0.777510445	416.498132	1.260207386
637.298752	0.471782256	503.806723	0.77952047	416.598132	1.249883364
637.582188	0.472444516	504.373597	0.780455417	416.698132	1.245628377
638.806723	0.475307635	505	0.781493217	417.298752	1.237142174

Final State: j'=5		Final State: j'=4		Final State: j'=3	
E(cm⁻¹)	σ (Å²)	E(cm⁻¹)	σ (Å²)	E(cm⁻¹)	σ (Å²)
639.373597	0.476634119	505.598132	0.782490079	417.582188	1.232356329
640	0.478100673	507.298752	0.785372494	418.806723	1.228029251
640.598132	0.479501785	507.582188	0.785862845	419.373597	1.229346824
642.298752	0.483489388	508.806723	0.788034197	420	1.230518399
642.582188	0.484154547	509.373597	0.789080962	420.598132	1.231694148
643.806723	0.487030036	510	0.790285692	422.298752	1.234390185
644.373597	0.488362164	510.598132	0.791506544	422.582188	1.233901844
645	0.489834892	512.298752	0.796002977	423.806723	1.233455029
645.598132	0.49124184	512.582188	0.797202601	424.373597	1.234996899
647.298752	0.495245714	513.806723	0.800970602	425	1.245841255
647.582188	0.495913537	514.373597	0.796037151	425.598132	1.230445485
648.806723	0.498800397	515	0.799745542	427.298752	1.229773116
649.373597	0.500137704	515.598132	0.799954565	427.582188	1.229893084
650	0.5016161	517.298752	0.805984894	428.806723	1.229985672
650.598132	0.503028399	517.582188	0.805977715	429.373597	1.229945989
652.298752	0.507047187	518.806723	0.801156455	430	1.229900468
652.582188	0.507717455	519.373597	0.804701952	430.598132	1.229877485
653.806723	0.510614729	520	0.802274194	432.298752	1.230311211
654.373597	0.511956783	520.598132	0.8075117	432.582188	1.230554983
655	0.51344037	522.298752	0.809547253	433.806723	1.229369618
655.598132	0.514857574	522.582188	0.807081335	434.373597	1.228515075
657.298752	0.518890033	523.806723	0.807524835	435	1.228107582
657.582188	0.519562538	524.373597	0.811326976	435.598132	1.227878294
658.806723	0.522469362	525	0.815089186	437.298752	1.227368123
659.373597	0.523815773	525.598132	0.810961059	437.582188	1.227288159
660	0.525304122	527.298752	0.80833605	438.806723	1.226956964
660.598132	0.526725829	527.582188	0.809850983	439.373597	1.226813425
662.298752	0.530770854	528.806723	0.815501444	440	1.226659992
662.582188	0.531445421	529.373597	0.820186923	440.598132	1.226509438
663.806723	0.534361052	530	0.816922232	442.298752	1.225940432
664.373597	0.535711481	530.598132	0.816547482	442.582188	1.225824367
665	0.537204239	532.298752	0.814841602	443.806723	1.225313612
665.598132	0.538630119	532.582188	0.816023496	444.373597	1.225084891
667.298752	0.542686842	533.806723	0.820443775	445	1.224840648
667.582188	0.543363337	534.373597	0.823104919	445.598132	1.224614912
668.806723	0.546287232	535	0.828988499	447.298752	1.224002935
669.373597	0.547641459	535.598132	0.823706044	447.582188	1.223904075
670	0.549138388	537.298752	0.818843645	448.806723	1.223483123
670.598132	0.550568239	537.582188	0.820705955	449.373597	1.223290114
672.298752	0.554636183	538.806723	0.825141356	450	1.223076946
672.582188	0.555314542	539.373597	0.826859726	450.598132	1.222872797
673.806723	0.558246493	540	0.828957888	452.298752	1.222286318
674.373597	0.559604456	540.598132	0.831708582	452.582188	1.222187753

Final State: j'=5		Final State: j'=4		Final State: j'=3	
E(cm⁻¹)	σ (Å²)	E(cm⁻¹)	σ (Å²)	E(cm⁻¹)	σ (Å²)
675	0.561105526	542.298752	0.83165495	453.806723	1.221760658
675.598132	0.562539345	542.582188	0.831514346	454.373597	1.221562821
677.298752	0.566618698	543.806723	0.830258285	455	1.221344575
677.582188	0.567298984	544.373597	0.832662478	455.598132	1.221136755
678.806723	0.570239366	545	0.834997861	457.298752	1.220550129
679.373597	0.571601303	545.598132	0.837420442	457.582188	1.220453043
680	0.57310683	547.298752	0.846787366	458.806723	1.220035852
680.598132	0.574544976	547.582188	0.844558867	459.373597	1.219843908
682.298752	0.578637119	548.806723	0.843092741	460	1.219632615
682.582188	0.579319618	549.373597	0.836992558	460.598132	1.219431574
683.806723	0.582269902	550	0.83907557	462.298752	1.218863624
684.373597	0.583636631	550.598132	0.845229918	462.582188	1.218769444
685	0.585147637	552.298752	0.846474055	463.806723	1.218364099
685.598132	0.58659121	552.582188	0.852102253	464.373597	1.218177303
687.298752	0.59070005	553.806723	0.838037175	465	1.217971528
687.582188	0.591385548	554.373597	0.843372602	465.598132	1.217775697
688.806723	0.594349646	555	0.850520543	467.298752	1.217222564
689.373597	0.595723318	555.598132	0.864756534	467.582188	1.217130932
690	0.597242465	557.298752	0.836832604	468.806723	1.216736957
690.598132	0.598694331	557.582188	0.840977837	469.373597	1.216555654
692.298752	0.602830199	558.806723	0.855235966	470	1.216356129
692.582188	0.603520801	559.373597	0.858001246	470.598132	1.216166433
693.806723	0.606509467	560	0.837865053	472.298752	1.215631521
694.373597	0.607896154	560.598132	0.84416467	472.582188	1.21554301
695	0.609431167	562.298752	0.855777332	473.806723	1.215162843
695.598132	0.610899905	562.582188	0.859342245	474.373597	1.214988059
697.298752	0.615096053	563.806723	0.845425647	475	1.214795823
697.582188	0.615799028	564.373597	0.853433701	475.598132	1.214613152
698.806723	0.618852272	565	0.853917989	477.298752	1.214098599
699.373597	0.620276933	565.598132	0.858836017	477.582188	1.214013547
700	0.621862314	567.298752	0.871434164	478.806723	1.213648454
700.598132	0.623390218	567.582188	0.875079471	479.373597	1.213480749
702.298752	0.627864136	568.806723	0.855550531	480	1.213296409
702.582188	0.628642467	569.373597	0.862049068	480.598132	1.213121377
703.806723	0.632297703	570	0.853182232	482.298752	1.212628975
704.373597	0.634453693	570.598132	0.861277298	482.582188	1.212547681
705	0.635792858	572.298752	0.867243868	483.806723	1.212199031
705.598132	0.634507752	572.582188	0.860855131	484.373597	1.212039056
707.298752	0.637256177	573.806723	0.854141864	485	1.21186336
707.582188	0.638037071	574.373597	0.864427279	485.598132	1.211696647
708.806723	0.644485867	575	0.864460962	487.298752	1.211228385
709.373597	0.643913958	575.598132	0.863039705	487.582188	1.211151178
710	0.644089565	577.298752	0.866218192	488.806723	1.210820395

Final State: j'=5		Final State: j'=4		Final State: j'=3	
E(cm⁻¹)	σ (Å²)	E(cm⁻¹)	σ (Å²)	E(cm⁻¹)	σ (Å²)
710.598132	0.645447871	577.582188	0.859889772	489.373597	1.210668812
712.298752	0.652604536	578.806723	0.866494693	490	1.210502473
712.582188	0.652788453	579.373597	0.86839785	490.598132	1.210344774
713.806723	0.655122169	580	0.870547006	492.298752	1.209902555
714.373597	0.65225773	580.598132	0.871774924	492.582188	1.20982976
715	0.656075623	582.298752	0.866787764	493.806723	1.209518212
715.598132	0.658745658	582.582188	0.867812222	494.373597	1.209375653
717.298752	0.661091372	583.806723	0.870389617	495	1.209219358
717.582188	0.661523682	584.373597	0.871247596	495.598132	1.209071344
718.806723	0.663990581	585	0.872165401	497.298752	1.208657107
719.373597	0.666422209	585.598132	0.872957041	497.582188	1.208589042
720	0.668796322	587.298752	0.873721585	498.806723	1.208298157
720.598132	0.671336457	587.582188	0.87397655	499.373597	1.208165283
722.298752	0.674865815	588.806723	0.875937845	500	1.20801979
722.582188	0.670168339	589.373597	0.874767269	500.598132	1.207882172
723.806723	0.676169434	590	0.873944627	502.298752	1.207498076
724.373597	0.678263176	590.598132	0.875263577	502.582188	1.207435099
725	0.68039939	592.298752	0.876904017	503.806723	1.207166557
725.598132	0.68251554	592.582188	0.87711959	504.373597	1.207044201
727.298752	0.68395745	593.806723	0.87802307	505	1.206910471
727.582188	0.685173412	594.373597	0.878435667	505.598132	1.206784246
728.806723	0.687004983	595	0.878891519	507.298752	1.206433506
729.373597	0.689661495	595.598132	0.879330146	507.582188	1.20637627
730	0.691826298	597.298752	0.880387908	508.806723	1.206133306
730.598132	0.693747005	597.582188	0.880432602	509.373597	1.206023358
732.298752	0.699772899	598.806723	0.881059365	510	1.205903905
732.582188	0.701285856	599.373597	0.881445922	510.598132	1.20579205
733.806723	0.701779314	600	0.88186294	512.298752	1.205485412
734.373597	0.700492824	600.598132	0.882250782	512.582188	1.205431061
735	0.703040334	602.298752	0.883315977	513.806723	1.204671961
735.598132	0.705673519	602.582188	0.883489438	514.373597	1.204668643
737.298752	0.711924828	603.806723	0.884226024	515	1.204762195
737.582188	0.713145284	604.373597	0.884558064	515.598132	1.204331702
738.806723	0.716605063	605	0.884915888	517.298752	1.204347038
739.373597	0.71675209	605.598132	0.88524817	517.582188	1.204090594
740	0.71734915	607.298752	0.886168092	518.806723	1.203969946
740.598132	0.717342948	607.582188	0.886320837	519.373597	1.203911109
742.298752	0.719260045	608.806723	0.886979679	520	1.203896898
742.582188	0.717723871	609.373597	0.887282818	520.598132	1.203858748
743.806723	0.726952791	610	0.887615669	522.298752	1.203628953
744.373597	0.728429394	610.598132	0.887930929	522.582188	1.20311332
745	0.721481447	612.298752	0.888811584	523.806723	1.203441559
745.598132	0.727128704	612.582188	0.888955911	524.373597	1.203367646

Final State: j'=5		Final State: j'=4		Final State: j'=3	
E(cm⁻¹)	σ (Å²)	E(cm⁻¹)	σ (Å²)	E(cm⁻¹)	σ (Å²)
747.298752	0.734111222	613.806723	0.889570889	525	1.203000023
747.582188	0.72821706	614.373597	0.88985082	525.598132	1.203635017
748.806723	0.731604792	615	0.890156687	527.298752	1.203049501
749.373597	0.73628194	615.598132	0.890445445	527.582188	1.203021024
750	0.740286909	617.298752	0.891249789	528.806723	1.202852364
750.598132	0.744732091	617.582188	0.891381559	529.373597	1.202792448
752.298752	0.742434201	618.806723	0.891943593	530	1.202979462
752.582188	0.743921297	619.373597	0.892199827	530.598132	1.20297798
753.806723	0.750180597	620	0.892480073	532.298752	1.202604429
754.373597	0.755136302	620.598132	0.892744822	532.582188	1.202564703
755	0.747829961	622.298752	0.893482332	533.806723	1.202398837
755.598132	0.747487761	622.582188	0.893603042	534.373597	1.202321369
757.298752	0.756167741	623.806723	0.894117275	535	1.202330062
757.582188	0.756883459	624.373597	0.894351316	535.598132	1.202891324
758.806723	0.7651437	625	0.894606989	537.298752	1.20238321
759.373597	0.75355257	625.598132	0.894848235	537.582188	1.202326841
760	0.757656935	627.298752	0.895518827	538.806723	1.202168665
760.598132	0.767067458	627.582188	0.895628404	539.373597	1.202111836
762.298752	0.7703427	628.806723	0.896094644	540	1.202050799
762.582188	0.772461516	629.373597	0.896306569	540.598132	1.201997188
763.806723	0.767070674	630	0.896537887	542.298752	1.201954929
764.373597	0.768194979	630.598132	0.896755969	542.582188	1.202645227
765	0.767857143	632.298752	0.897361187	543.806723	1.202238376
765.598132	0.771346659	632.582188	0.897459936	544.373597	1.20219475
767.298752	0.773743899	633.806723	0.897879602	545	1.20218969
767.582188	0.778289331	634.373597	0.89807008	545.598132	1.202220139
768.806723	0.778514637	635	0.898277769	547.298752	1.201373027
769.373597	0.78067603	635.598132	0.898473361	547.582188	1.201112794
770	0.783205816	637.298752	0.899014998	548.806723	1.202123754
770.598132	0.785252901	637.582188	0.8991032	549.373597	1.202214654
772.298752	0.789359255	638.806723	0.899477484	550	1.202189921
772.582188	0.790315178	639.373597	0.899647044	550.598132	1.201336267
773.806723	0.790552182	640	0.899831691	552.298752	1.201827084
774.373597	0.792559746	640.598132	0.900005349	552.582188	1.201834035
775	0.794269461	642.298752	0.900485012	553.806723	1.200744009
775.598132	0.795769951	642.582188	0.900562941	554.373597	1.201225112
777.298752	0.799709399	643.806723	0.900893027	555	1.201571506
777.582188	0.800246799	644.373597	0.901042231	555.598132	1.20260191
778.806723	0.802727717	645	0.901204459	557.298752	1.201794704
779.373597	0.804035736	645.598132	0.901356788	557.582188	1.20170693
780	0.805545662	647.298752	0.901776207	558.806723	1.202143143
780.598132	0.806326173	647.582188	0.901844151	559.373597	1.201390453
782.298752	0.810121239	648.806723	0.902131299	560	1.201219621

Final State: j'=5		Final State: j'=4		Final State: j'=3	
E(cm⁻¹)	σ (Å²)	E(cm⁻¹)	σ (Å²)	E(cm⁻¹)	σ (Å²)
782.582188	0.810771792	649.373597	0.902260728	560.598132	1.20162406
783.806723	0.813468754	650	0.902401185	562.298752	1.202061807
784.373597	0.814699846	650.598132	0.902532798	562.582188	1.202180282
785	0.81605736	652.298752	0.902893718	563.806723	1.203799567
785.598132	0.817351803	652.582188	0.902951973	564.373597	1.20203334
787.298752	0.821023552	653.806723	0.903197443	565	1.203131062
787.582188	0.821633283	654.373597	0.903307683	565.598132	1.2025752
788.806723	0.824159419	655	0.903427014	567.298752	1.202916765
789.373597	0.825286447	655.598132	0.903538535	567.582188	1.203132691
790	0.826625925	657.298752	0.903842729	568.806723	1.202540926
790.598132	0.827920567	657.582188	0.903891587	569.373597	1.2013557
792.298752	0.83155795	658.806723	0.904096662	570	1.201556417
792.582188	0.832160005	659.373597	0.90418831	570.598132	1.202499626
793.806723	0.83475275	660	0.904287175	572.298752	1.203719188
794.373597	0.835949006	660.598132	0.904379228	572.582188	1.203647626
795	0.837267914	662.298752	0.904628495	573.806723	1.203020272
795.598132	0.838523977	662.582188	0.904668256	574.373597	1.203293826
797.298752	0.842074776	663.806723	0.904834234	575	1.202700605
797.582188	0.842664307	664.373597	0.904907887	575.598132	1.202667698
798.806723	0.845209386	665	0.904986952	577.298752	1.204116812
799.373597	0.846386943	665.598132	0.905060173	577.582188	1.203391266
800	0.847687253	667.298752	0.905256315	578.806723	1.203106966
800.598132	0.848927591	667.582188	0.905287281	579.373597	1.203167012
802.298752	0.852445176	668.806723	0.905415452	580	1.203274159
802.582188	0.853030002	669.373597	0.905471713	580.598132	1.204535707
803.806723	0.855551577	670	0.905531632	582.298752	1.203912456
804.373597	0.856716031	670.598132	0.905586651	582.582188	1.203857994
805	0.858000627	672.298752	0.905731435	583.806723	1.203947342
805.598132	0.85922514	672.582188	0.905753902	584.373597	1.204028626
807.298752	0.862695646	673.806723	0.905845534	585	1.204137533
807.582188	0.86327251	674.373597	0.905884982	585.598132	1.204282935
808.806723	0.865759787	675	0.905926399	587.298752	1.204680023
809.373597	0.86690852	675.598132	0.905963824	587.582188	1.20472454
810	0.868175907	677.298752	0.906058966	588.806723	1.205050245
810.598132	0.869384146	677.582188	0.906073213	589.373597	1.205384851
812.298752	0.872808953	678.806723	0.906129508	590	1.205314559
812.582188	0.873378233	679.373597	0.906152693	590.598132	1.205301298
813.806723	0.875832631	680	0.906176206	592.298752	1.205659345
814.373597	0.87696604	680.598132	0.906196601	592.582188	1.205721177
815	0.878216393	682.298752	0.906243671	593.806723	1.205991915
815.598132	0.879408266	682.582188	0.90624995	594.373597	1.206120639
817.298752	0.882786018	683.806723	0.906271982	595	1.206266747
817.582188	0.883347388	684.373597	0.906279377	595.598132	1.206411985

Final State: j’=5		Final State: j’=4		Final State: j’=3	
E(cm⁻¹)	σ (Å²)	E(cm⁻¹)	σ (Å²)	E(cm⁻¹)	σ (Å²)
818.806723	0.885767435	685	0.906285503	597.298752	1.206923699
819.373597	0.886884856	685.598132	0.906289338	597.582188	1.206994661
820	0.888117487	687.298752	0.906289566	598.806723	1.207218548
820.598132	0.88929239	687.582188	0.906288067	599.373597	1.207346658
822.298752	0.89262167	688.806723	0.906276568	600	1.207498186
822.582188	0.893174928	689.373597	0.906268475	600.598132	1.207648346
823.806723	0.895559829	690	0.906257492	602.298752	1.208093714
824.373597	0.896660921	690.598132	0.906245	602.582188	1.208170226
825	0.897875464	692.298752	0.90619872	603.806723	1.208508486
825.598132	0.89903305	692.582188	0.906189447	604.373597	1.208669356
827.298752	0.902312841	693.806723	0.906144194	605	1.208849639
827.582188	0.902857815	694.373597	0.906120348	605.598132	1.209023
828.806723	0.905206801	695	0.906091405	607.298752	1.209516858
829.373597	0.906291191	695.598132	0.906062431	607.582188	1.209599837
830	0.90748725	697.298752	0.905966767	608.806723	1.209963376
830.598132	0.908627129	697.582188	0.905948997	609.373597	1.210134836
832.298752	0.911856331	698.806723	0.905865703	610	1.21032665
832.582188	0.912392839	699.373597	0.905823255	610.598132	1.210512146
833.806723	0.914705135	700	0.905773127	612.298752	1.211051719
834.373597	0.915772491	700.598132	0.905721708	612.582188	1.211143352
835	0.916949661	702.298752	0.90555011	613.806723	1.211544577
835.598132	0.918071469	702.582188	0.905516327	614.373597	1.21173315
837.298752	0.921249071	703.806723	0.905338408	615	1.211943522
837.582188	0.921776946	704.373597	0.905243165	615.598132	1.212146323
838.806723	0.924051851	705	0.90562879	617.298752	1.212733139
839.373597	0.925101836	705.598132	0.906266398	617.582188	1.212832422
840	0.926259773	707.298752	0.906261871	618.806723	1.213266285
840.598132	0.927363175	707.582188	0.90581631	619.373597	1.21346988
842.298752	0.93048823	708.806723	0.905342419	620	1.213696898
842.582188	0.93100732	709.373597	0.906023033	620.598132	1.213915678
843.806723	0.93324416	710	0.90572277	622.298752	1.214548469
844.373597	0.934276474	710.598132	0.905460977	622.582188	1.214655481
845	0.935414839	712.298752	0.904973618	623.806723	1.215122892
845.598132	0.936499519	712.582188	0.905649384	624.373597	1.215342038
847.298752	0.939571141	713.806723	0.904927688	625	1.215586248
847.582188	0.940081293	714.373597	0.905566102	625.598132	1.21582141
848.806723	0.942279444	715	0.904741685	627.298752	1.216500631
849.373597	0.943293795	715.598132	0.904504836	627.582188	1.216615355
850	0.944412278	717.298752	0.905068876	628.806723	1.217115972
850.598132	0.945477938	717.582188	0.904993991	629.373597	1.217350453
852.298752	0.948495293	718.806723	0.904412387	630	1.217611575
852.582188	0.948996376	719.373597	0.904148745	630.598132	1.217862881
853.806723	0.951155252	720	0.90394987	632.298752	1.218587919

Final State: j’=5		Final State: j’=4		Final State: j’=3	
E(cm⁻¹)	σ (Å²)	E(cm⁻¹)	σ (Å²)	E(cm⁻¹)	σ (Å²)
854.373597	0.952151376	720.598132	0.903799865	632.582188	1.218710265
855	0.953249684	722.298752	0.904107538	633.806723	1.219243807
855.598132	0.954296046	722.582188	0.905524728	634.373597	1.219493525
857.298752	0.957258366	723.806723	0.903598661	635	1.219771463
857.582188	0.957750252	724.373597	0.903395966	635.598132	1.220038816
858.806723	0.959869316	725	0.903216792	637.298752	1.220809389
859.373597	0.960846967	725.598132	0.903060802	637.582188	1.220939317
860	0.961924834	727.298752	0.904183228	638.806723	1.221505552
860.598132	0.962951646	727.582188	0.903375193	639.373597	1.221770373
862.298752	0.965858216	728.806723	0.902948455	640	1.222064981
862.582188	0.96634079	729.373597	0.902617889	640.598132	1.222348232
863.806723	0.968419545	730	0.902413133	642.298752	1.223163901
864.373597	0.969378499	730.598132	0.902247631	642.582188	1.223301331
865	0.970435671	732.298752	0.901851949	643.806723	1.223899905
865.598132	0.971442696	732.582188	0.901923996	644.373597	1.224179673
867.298752	0.974292873	733.806723	0.902275253	645	1.224490771
867.582188	0.974766025	734.373597	0.902598107	645.598132	1.224789747
868.806723	0.976804015	735	0.90158269	647.298752	1.225650005
869.373597	0.977744059	735.598132	0.901285724	647.582188	1.225794846
870	0.978780316	737.298752	0.900704032	648.806723	1.226425399
870.598132	0.979767341	737.582188	0.900599336	649.373597	1.226719926
872.298752	0.98256052	738.806723	0.901100531	650	1.227047324
872.582188	0.983024155	739.373597	0.901087037	650.598132	1.227361834
873.806723	0.985020964	740	0.902373991	652.298752	1.22826612
874.373597	0.985941909	740.598132	0.90195666	652.582188	1.228418283
875	0.986957038	742.298752	0.901674085	653.806723	1.229080393
875.598132	0.987923868	742.582188	0.901382598	654.373597	1.229389492
877.298752	0.990659502	743.806723	0.899672975	655	1.229732958
877.582188	0.991113528	744.373597	0.900776976	655.598132	1.230062785
878.806723	0.993068771	745	0.901452471	657.298752	1.231010483
879.373597	0.993970447	745.598132	0.899804946	657.582188	1.231169857
880	0.994964257	747.298752	0.902252	658.806723	1.231863049
880.598132	0.995910712	747.582188	0.903111886	659.373597	1.232186503
882.298752	0.998588299	748.806723	0.899407887	660	1.232545798
882.582188	0.999032635	749.373597	0.898740671	660.598132	1.232890713
883.806723	1.000945964	750	0.898368769	662.298752	1.233881137
884.373597	1.001828145	750.598132	0.89911046	662.582188	1.234047602
885	1.002800529	752.298752	0.898242456	663.806723	1.234771382
885.598132	1.003726443	752.582188	0.898059232	664.373597	1.235108948
887.298752	1.006345523	753.806723	0.897459845	665	1.235483811
887.582188	1.006780094	754.373597	0.897528553	665.598132	1.235843555
888.806723	1.008651187	755	0.901022502	667.298752	1.23687597
889.373597	1.009513853	755.598132	0.898539844	667.582188	1.237049416

Final State: j’=5		Final State: j’=4		Final State: j’=3	
E(cm⁻¹)	σ (Å²)	E(cm⁻¹)	σ (Å²)	E(cm⁻¹)	σ (Å²)
890	1.010464521	757.298752	0.897201491	668.806723	1.237803234
890.598132	1.011369734	757.582188	0.897071384	669.373597	1.238154666
892.298752	1.013929878	758.806723	0.896951888	670	1.238544818
892.582188	1.01435461	759.373597	0.900151953	670.598132	1.238919124
893.806723	1.016183156	760	0.896883387	672.298752	1.239992754
894.373597	1.017026099	760.598132	0.89602542	672.582188	1.240173042
895	1.017954951	762.298752	0.895486404	673.806723	1.24095635
895.598132	1.018839319	762.582188	0.89666057	674.373597	1.241321384
897.298752	1.021340083	763.806723	0.899455346	675	1.241726523
897.582188	1.021754906	764.373597	0.896495548	675.598132	1.242115118
898.806723	1.023540577	765	0.897074926	677.298752	1.243229193
899.373597	1.024363643	765.598132	0.895302352	677.582188	1.24341619
900	1.025270511	767.298752	0.897012438	678.806723	1.244228415
900.598132	1.026133864	767.582188	0.895013858	679.373597	1.244606789
902.298752	1.02857475	768.806723	0.896922514	680	1.245026645
902.582188	1.028979572	769.373597	0.895070466	680.598132	1.245429249
903.806723	1.030721972	770	0.894709037	682.298752	1.246582997
904.373597	1.03152497	770.598132	0.894532691	682.582188	1.246776588
905	1.032409626	772.298752	0.894625105	683.806723	1.247617203
905.598132	1.033251739	772.582188	0.894832131	684.373597	1.248008684
907.298752	1.035632034	773.806723	0.894173628	685	1.248442992
907.582188	1.036026727	774.373597	0.893898787	685.598132	1.248859371
908.806723	1.037725241	775	0.893736958	687.298752	1.250052172
909.373597	1.038507849	775.598132	0.893604723	687.582188	1.250252257
910	1.039369912	777.298752	0.893358849	688.806723	1.251120899
910.598132	1.040190384	777.582188	0.893339709	689.373597	1.251525338
912.298752	1.042508706	778.806723	0.893069891	690	1.251973955
912.582188	1.042892994	779.373597	0.892927104	690.598132	1.252404003
913.806723	1.044546253	780	0.892804141	692.298752	1.253635723
914.373597	1.045307695	780.598132	0.893213112	692.582188	1.253842309
915	1.046146192	782.298752	0.892224135	693.806723	1.254739159
915.598132	1.046943942	782.582188	0.892166603	694.373597	1.255156732
917.298752	1.049196077	783.806723	0.891908116	695	1.255620257
917.582188	1.049569027	784.373597	0.891783818	695.598132	1.256064058
918.806723	1.05117177	785	0.891645199	697.298752	1.257336613
919.373597	1.051908689	785.598132	0.891512757	697.582188	1.257550191
920	1.05271883	787.298752	0.891144254	698.806723	1.258478207
920.598132	1.053487723	787.582188	0.891086504	699.373597	1.258910961
922.298752	1.055638928	788.806723	0.890874793	700	1.259391768
922.582188	1.055989647	789.373597	0.890736529	700.598132	1.259853793
923.806723	1.057910745	790	0.890563147	702.298752	1.261189781
924.373597	1.059243722	790.598132	0.890413257	702.582188	1.26141757
925	1.059479843	792.298752	0.890019602	703.806723	1.262450403

Final State: j'=5		Final State: j'=4		Final State: j'=3	
E(cm⁻¹)	σ (Å²)	E(cm⁻¹)	σ (Å²)	E(cm⁻¹)	σ (Å²)
925.598132	1.060238819	792.582188	0.889955456	704.373597	1.263043075
927.298752	1.062134383	793.806723	0.889680242	705	1.264010387
927.582188	1.062528571	794.373597	0.889553778	705.598132	1.264541198
928.806723	1.064656127	795	0.889414794	707.298752	1.265574761
929.373597	1.065258733	795.598132	0.889282879	707.582188	1.265379862
930	1.066027079	797.298752	0.888909449	708.806723	1.266883067
930.598132	1.066396394	797.582188	0.888846761	709.373597	1.267507042
932.298752	1.068751143	798.806723	0.888573807	710	1.26743343
932.582188	1.068943168	799.373597	0.888447012	710.598132	1.267887054
933.806723	1.070391068	800	0.888307095	712.298752	1.269437182
934.373597	1.070979173	800.598132	0.888173847	712.582188	1.270044154
935	1.071736493	802.298752	0.887797217	713.806723	1.270682127
935.598132	1.073769182	802.582188	0.887734772	714.373597	1.270830453
937.298752	1.075230137	803.806723	0.887466045	715	1.271105972
937.582188	1.075022446	804.373597	0.887342196	715.598132	1.271684637
938.806723	1.076269683	805	0.887205724	717.298752	1.273543051
939.373597	1.076906276	805.598132	0.887075762	717.582188	1.273909181
940	1.078236023	807.298752	0.886707941	718.806723	1.274140295
940.598132	1.079489991	807.582188	0.886646867	719.373597	1.274659013
942.298752	1.080523641	808.806723	0.886383785	720	1.275238793
942.582188	1.08076988	809.373597	0.886262444	720.598132	1.275844214
943.806723	1.082062591	810	0.886128712	722.298752	1.277849484
944.373597	1.082684894	810.598132	0.88600138	722.582188	1.278120331
945	1.083402508	812.298752	0.885641419	723.806723	1.278299994
945.598132	1.085078308	812.582188	0.885581739	724.373597	1.278819442
947.298752	1.086516726	813.806723	0.885324993	725	1.279389057
947.582188	1.086549715	814.373597	0.885206743	725.598132	1.27994373
948.806723	1.08766904	815	0.885076538	727.298752	1.281992688
949.373597	1.088268668	815.598132	0.884952666	727.582188	1.282059437
950	1.088936735	817.298752	0.884602971	728.806723	1.282505078
950.598132	1.089580305	817.582188	0.884545055	729.373597	1.283052219
952.298752	1.092329293	818.806723	0.884296073	730	1.283630081
952.582188	1.092091029	819.373597	0.8841815	730.598132	1.284175024
953.806723	1.093116417	820	0.88405541	732.298752	1.28577063
954.373597	1.093619432	820.598132	0.88393553	732.582188	1.286103642
955	1.094233677	822.298752	0.883597496	733.806723	1.28768645
955.598132	1.094821811	822.582188	0.883541567	734.373597	1.287903705
957.298752	1.09683404	823.806723	0.883301348	735	1.287950437
957.582188	1.09760497	824.373597	0.88319092	735.598132	1.288547686
958.806723	1.099726367	825	0.883069478	737.298752	1.29021426
959.373597	1.100455557	825.598132	0.8829541	737.582188	1.290525533
960	1.100197201	827.298752	0.882629215	738.806723	1.292878924
960.598132	1.100543102	827.582188	0.882575531	739.373597	1.29264992

Final State: j'=5		Final State: j'=4		Final State: j'=3	
E(cm⁻¹)	σ (Å²)	E(cm⁻¹)	σ (Å²)	E(cm⁻¹)	σ (Å²)
962.298752	1.102123476	828.806723	0.882345151	740	1.294214369
962.582188	1.102294004	829.373597	0.882239368	740.598132	1.294775473
963.806723	1.105456267	830	0.882123123	742.298752	1.296493082
964.373597	1.104560918	830.598132	0.882012766	742.582188	1.295888005
965	1.104895208	832.298752	0.881702479	743.806723	1.296276092
965.598132	1.105432981	832.582188	0.881651275	744.373597	1.298301616
967.298752	1.107782933	833.806723	0.881431754	745	1.298139073
967.582188	1.108021429	834.373597	0.881331079	745.598132	1.29755041
968.806723	1.108474871	835	0.881220538	747.298752	1.301494461
969.373597	1.109442754	835.598132	0.881115687	747.582188	1.302207997
970	1.112464117	837.298752	0.880821361	748.806723	1.300277811
970.598132	1.110772179	837.582188	0.88077286	749.373597	1.300912735
972.298752	1.111859077	838.806723	0.880565163	750	1.301654667
972.582188	1.11209795	839.373597	0.880470042	750.598132	1.303593196
973.806723	1.116329849	840	0.880365693	752.298752	1.303514271
974.373597	1.114855669	840.598132	0.880266809	752.582188	1.30379599
975	1.114777973	842.298752	0.879989745	753.806723	1.305080941
975.598132	1.115074791	842.582188	0.879944165	754.373597	1.306069254
977.298752	1.117542287	843.806723	0.879749215	755	1.307253182
977.582188	1.117139298	844.373597	0.879660069	755.598132	1.307297315
978.806723	1.118582726	845	0.879562377	757.298752	1.30930556
979.373597	1.118368184	845.598132	0.879469901	757.582188	1.309410993
980	1.12099148	847.298752	0.879211337	758.806723	1.310699023
980.598132	1.119871276	847.582188	0.879168876	759.373597	1.312659371
982.298752	1.122216764	848.806723	0.878987545	760	1.310867731
982.582188	1.123428596	849.373597	0.87890477	760.598132	1.312129092
983.806723	1.12434098	850	0.878814175	762.298752	1.313143977
984.373597	1.124120044	850.598132	0.878728523	762.582188	1.315528961
985	1.123635631	852.298752	0.878489623	763.806723	1.316188466
985.598132	1.124249156	852.582188	0.878450479	764.373597	1.315514029
987.298752	1.127817602	853.806723	0.878283588	765	1.317615678
987.582188	1.127757656	854.373597	0.878207566	765.598132	1.316147068
988.806723	1.127689007	855	0.878124477	767.298752	1.319474462
989.373597	1.128171019	855.598132	0.878046042	767.582188	1.31868789
990	1.128775179	857.298752	0.877827904	768.806723	1.320515256
990.598132	1.129419573	857.582188	0.877792254	769.373597	1.320657642
992.298752	1.131115641	858.806723	0.87764058	770	1.321217986
992.582188	1.131148467	859.373597	0.877571663	770.598132	1.321807358
993.806723	1.132086258	860	0.877496469	772.298752	1.323826861
994.373597	1.13257441	860.598132	0.87742562	772.582188	1.324306788
995	1.133118018	862.298752	0.87722927	773.806723	1.325105033
995.598132	1.133650186	862.582188	0.877197287	774.373597	1.325628789
997.298752	1.13514259	863.806723	0.877061548	775	1.326240609

Final State: j'=5		Final State: j'=4		Final State: j'=3	
E(cm⁻¹)	σ (Å²)	E(cm⁻¹)	σ (Å²)	E(cm⁻¹)	σ (Å²)
997.582188	1.135367335	864.373597	0.877000066	775.598132	1.326829602
998.806723	1.136375179	865	0.876933131	777.298752	1.328525913
999.373597	1.137052718	865.598132	0.876870205	777.582188	1.328813197
1000	1.137441867	867.298752	0.876696606	778.806723	1.330026871
1000.59813	1.137761128	867.582188	0.876668446	779.373597	1.330580516
1002.29875	1.139150157	868.806723	0.876549315	780	1.331183351
1002.58219	1.139377599	869.373597	0.876495574	780.598132	1.331800185
1003.80672	1.140351094	870	0.876437235	782.298752	1.333530411
1004.3736	1.140798302	870.598132	0.876382551	782.582188	1.333811954
1005	1.141290738	872.298752	0.876232592	783.806723	1.335034318
1005.59813	1.141759761	872.582188	0.876208396	784.373597	1.335601653
1007.29875	1.143102131	873.806723	0.876106501	785	1.33622913
1007.58219	1.143326541	874.373597	0.876060788	785.598132	1.336828753
1008.80672	1.144236265	875	0.876011349	787.298752	1.338534992
1009.3736	1.144658701	875.598132	0.875965208	787.582188	1.338819321
1010	1.145128298	877.298752	0.875839702	788.806723	1.340061269
1010.59813	1.14557644	877.582188	0.875819606	789.373597	1.340648131
1012.29875	1.146842732	878.806723	0.875735511	790	1.341282421
1012.58219	1.147052443	879.373597	0.875698085	790.598132	1.341885146
1013.80672	1.147954038	880	0.875657835	792.298752	1.343604166
1014.3736	1.148369005	880.598132	0.875620494	792.582188	1.343891283
1015	1.148825689	882.298752	0.87552019	793.806723	1.345133145
1015.59813	1.149259686	882.582188	0.875504319	794.373597	1.345708731
1017.29875	1.150480525	883.806723	0.875438545	795	1.346345286
1017.58219	1.150682184	884.373597	0.875409698	795.598132	1.346953667
1018.80672	1.151548416	885	0.875378835	797.298752	1.348687526
1019.3736	1.151946834	885.598132	0.875350546	797.582188	1.348977077
1020	1.152385229	887.298752	0.87527611	798.806723	1.350228817
1020.59813	1.152802014	887.582188	0.87526457	799.373597	1.350808508
1022.29875	1.153977237	888.806723	0.875217585	800	1.351449223
1022.58219	1.154171695	889.373597	0.875197412	800.598132	1.352061244
1023.80672	1.155007119	890	0.875176283	802.298752	1.353802764
1024.3736	1.15539127	890.598132	0.875157258	802.582188	1.354093243
1025	1.15581384	892.298752	0.875109299	803.806723	1.35534901
1025.59813	1.156215448	892.582188	0.875102188	804.373597	1.355930782
1027.29875	1.157347228	893.806723	0.875074402	805	1.356573983
1028.80672	1.158338413	894.373597	0.87506315	805.598132	1.357188482
1029.3736	1.15870804	895	0.875051905	807.298752	1.358937302
1030	1.159114605	895.598132	0.875042333	807.582188	1.359228993
1030.59813	1.159500987	897.298752	0.875021369	808.806723	1.360489867
1032.29875	1.160589853	897.582188	0.875018779	809.373597	1.361073911
1033.80672	1.161543436	898.806723	0.875010553	810	1.361719515
1034.3736	1.161899017	899.373597	0.875008383	810.598132	1.362336207

Final State: j’=5		Final State: j’=4		Final State: j’=3	
E(cm⁻¹)	σ (Å²)	E(cm⁻¹)	σ (Å²)	E(cm⁻¹)	σ (Å²)
1035	1.162290118	900	0.875007191	812.298752	1.364090708
1035.59813	1.162661782	900.598132	0.875007242	812.582188	1.364383281
1037.29875	1.163709021	902.298752	0.875013734	813.806723	1.365647771
1038.80672	1.164625958	902.582188	0.87501573	814.373597	1.366233398
1039.3736	1.164967835	903.806723	0.875027373	815	1.366880706
1040	1.165343828	904.373597	0.875034426	815.598132	1.367498973
1040.59813	1.16570111	905	0.875043442	817.298752	1.369257719
1042.29875	1.166707702	905.598132	0.875053254	817.582188	1.369550961
1043.80672	1.1675889	907.298752	0.875087581	818.806723	1.37081822
1044.3736	1.167917415	907.582188	0.875094231	819.373597	1.371405057
1045	1.168278695	908.806723	0.875126003	820	1.372053644
1045.59813	1.168621977	909.373597	0.875142396	820.598132	1.372673081
1047.29875	1.169589005	910	0.875161747	822.298752	1.374434839
1048.80672	1.170435435	910.598132	0.875181442	822.582188	1.374728535
1049.3736	1.170750955	912.298752	0.87524394	823.806723	1.375997632
1050	1.171097922	912.582188	0.875255293	824.373597	1.37658523
1050.59813	1.171427582	913.806723	0.875307439	825	1.377234604
1052.29875	1.17235614	914.373597	0.875333277	825.598132	1.377854734
1053.80672	1.173168766	915	0.875363089	827.298752	1.379618171
1054.3736	1.173471654	915.598132	0.875392791	827.582188	1.379912109
1055	1.173804713	917.298752	0.875483877	828.806723	1.381182085
1055.59813	1.174121138	917.582188	0.87550002	829.373597	1.381770033
1057.29875	1.175012319	918.806723	0.875572977	830	1.382419738
1058.80672	1.175792118	919.373597	0.875608555	830.598132	1.383040118
1059.3736	1.17608274	920	0.875649246	832.298752	1.384803994
1060	1.176402294	920.598132	0.875689608	832.582188	1.385097971
1060.59813	1.176705875	922.298752	0.875813601	833.806723	1.386367952
1062.29875	1.177560786	922.582188	0.875836583	834.373597	1.386955822
1063.80672	1.178308742	923.806723	0.875997139	835	1.387605392
1064.3736	1.178587472	924.373597	0.876064446	835.598132	1.388225606
1065	1.178893933	925	0.876022092	837.298752	1.389988698
1065.59813	1.17918506	925.598132	0.876121655	837.582188	1.390282505
1067.29875	1.180004821	927.298752	0.87621017	838.806723	1.391551631
1068.80672	1.180721923	927.582188	0.87626676	839.373597	1.392139038
1069.3736	1.180989135	928.806723	0.876364161	840	1.392788036
1070	1.181282915	929.373597	0.876473425	840.598132	1.393407649
1070.59813	1.181561983	930	0.876483523	842.298752	1.395168783
1072.29875	1.182347715	930.598132	0.876509636	842.582188	1.39546222
1073.80672	1.183034967	932.298752	0.876731509	843.806723	1.396729642
1074.3736	1.183291035	932.582188	0.876765214	844.373597	1.397316191
1075	1.18357255	933.806723	0.87685203	845	1.397964196
1075.59813	1.183839956	934.373597	0.876907297	845.598132	1.398582804
1077.29875	1.184592786	935	0.876958761	847.298752	1.400340843

Final State: j'=5		Final State: j'=4		Final State: j'=3	
E(cm⁻¹)	σ (Å²)	E(cm⁻¹)	σ (Å²)	E(cm⁻¹)	σ (Å²)
1078.80672	1.18525119	935.598132	0.877100552	847.582188	1.400633719
1079.3736	1.185496491	937.298752	0.877327162	848.806723	1.401898628
1080	1.185766157	937.582188	0.877312273	849.373597	1.402483938
1080.59813	1.186022313	938.806723	0.877445542	850	1.403130536
1083.80672	1.187373919	939.373597	0.877499616	850.598132	1.403747755
1084.3736	1.187608832	940	0.877568769	852.298752	1.40550158
1085	1.18786707	940.598132	0.877770009	852.582188	1.405793724
1085.59813	1.188112343	942.298752	0.877954606	853.806723	1.407055324
1088.80672	1.189406489	942.582188	0.877988045	854.373597	1.40763905
1089.3736	1.189631232	943.806723	0.878141688	855	1.408283841
1090	1.189878614	944.373597	0.87820949	855.598132	1.408899293
1090.59813	1.190113419	945	0.878265792	857.298752	1.410647852
1093.80672	1.191352225	945.598132	0.87829825	857.582188	1.410939085
1094.3736	1.191567491	947.298752	0.878761601	858.806723	1.412196638
1095	1.191804117	947.582188	0.878772154	859.373597	1.412778427
1095.59813	1.192028853	948.806723	0.878937241	860	1.413421034
1098.80672	1.193214444	949.373597	0.879019688	860.598132	1.414034357
1099.3736	1.193420448	950	0.87910977	862.298752	1.415776631
1100.59813	1.193861954	950.598132	0.879189773	862.582188	1.416066784
1103.80672	1.194996449	952.298752	0.879681525	863.806723	1.417319561
1104.3736	1.195193567	952.582188	0.879647684	864.373597	1.417899084
1105.59813	1.195616022	953.806723	0.879842884	865	1.418539142
1108.80672	1.196701529	954.373597	0.879926761	865.598132	1.419149986
1109.3736	1.196890132	955	0.880029448	867.298752	1.420885
1110.59813	1.197294334	955.598132	0.880132039	867.582188	1.421173908
1113.80672	1.198332943	957.298752	0.880533268	868.806723	1.422421218
1114.3736	1.198513398	957.582188	0.88066521	869.373597	1.42299815
1115.59813	1.198900143	958.806723	0.880851866	870	1.423635304
1118.80672	1.199893924	959.373597	0.881058684	870.598132	1.424243341
1119.3736	1.200066598	960	0.881104671	872.298752	1.425970155
1120.59813	1.20043667	960.598132	0.881240682	872.582188	1.426257668
1123.80672	1.201387677	962.298752	0.881444596	873.806723	1.427498852
1124.3736	1.201552925	962.582188	0.881500684	874.373597	1.428072891
1125.59813	1.201907107	963.806723	0.881979682	875	1.428706805
1128.80672	1.202817355	964.373597	0.881844854	875.598132	1.429311717
1129.3736	1.20297554	965	0.881932861	877.298752	1.431029433
1130.59813	1.203314591	965.598132	0.881974869	877.582188	1.4313154
1133.80672	1.204186077	967.298752	0.882660923	878.806723	1.432549811
1134.3736	1.204337545	967.582188	0.882667124	879.373597	1.433120663
1135.59813	1.204662219	968.806723	0.8828468	880	1.433751026
1138.80672	1.205496899	969.373597	0.88298948	880.598132	1.434352499
1139.3736	1.205641994	970	0.883364717	882.298752	1.436060259
1140.59813	1.205953031	970.598132	0.883320383	882.582188	1.436344541

Final State: j’=5		Final State: j’=4		Final State: j’=3	
E(cm⁻¹)	σ (Å²)	E(cm⁻¹)	σ (Å²)	E(cm⁻¹)	σ (Å²)
1143.80672	1.206752816	972.298752	0.883618002	883.806723	1.437571566
1144.3736	1.20689187	972.582188	0.883663388	884.373597	1.438139012
1145.59813	1.207189987	973.806723	0.883906307	885	1.43876544
1148.80672	1.207956729	974.373597	0.884415753	885.598132	1.439363187
1149.3736	1.208090064	975	0.884467281	887.298752	1.44106015
1150.59813	1.20837595	975.598132	0.884682032	887.582188	1.441342598
1153.80672	1.209111413	977.298752	0.885063806	888.806723	1.442561648
1154.3736	1.209239333	977.582188	0.884865808	889.373597	1.443125292
1155.59813	1.209513637	978.806723	0.885421366	890	1.443747612
1158.80672	1.210219416	979.373597	0.885505549	890.598132	1.444341336
1159.3736	1.210342189	980	0.885976892	892.298752	1.446026691
1160.59813	1.210605445	980.598132	0.885748177	892.582188	1.446307182
1163.80672	1.211282658	982.298752	0.886029345	893.806723	1.447517663
1165.59813	1.211652679	982.582188	0.886439897	894.373597	1.448077293
1168.80672	1.212296397	983.806723	0.887073726	895	1.448695145
1170.59813	1.212524753	984.373597	0.887026959	895.598132	1.449284561
1173.80672	1.213222255	985	0.887049867	897.298752	1.45095747
1175.59813	1.21357598	985.598132	0.887172377	897.582188	1.451235862
1178.80672	1.214176024	987.298752	0.887685892	898.806723	1.452437176
1180.59813	1.214555954	987.582188	0.887907849	899.373597	1.452992504
1183.80672	1.215183444	988.806723	0.88822958	900	1.453605554
1185.59813	1.215446703	989.373597	0.888359092	900.598132	1.454190361
1188.80672	1.216086665	990	0.88850704	902.298752	1.455849941
1190.59813	1.216352096	990.598132	0.888611416	902.582188	1.456126078
1193.80672	1.216986522	992.298752	0.889353884	903.806723	1.457317535
1195.59813	1.217239658	992.582188	0.889450502	904.373597	1.457868241
1198.80672	1.217684506	993.806723	0.889748684	905	1.458476141
1200.59813	1.218105785	994.373597	0.889910878	905.598132	1.459055974
1203.80672	1.218383757	995	0.890093879	907.298752	1.460701109
1205.59813	1.218710686	995.598132	0.890270654	907.582188	1.460974792
1208.80672	1.219342717	997.298752	0.890821942	908.806723	1.46215549
1210.59813	1.21962589	997.582188	0.890913082	909.373597	1.462701114
1213.80672	1.220200653	998.806723	0.891287071	910	1.463303321
1215.59813	1.220202958	999.373597	0.891415751	910.598132	1.46387763
1218.80672	1.220860944	1000	0.891801979	912.298752	1.465506528
1220.59813	1.221396677	1000.59813	0.891942371	912.582188	1.46577741
1223.80672	1.22144173	1002.29875	0.892479815	913.806723	1.466945656
1225.59813	1.221931119	1002.58219	0.892574129	914.373597	1.467485278
1228.80672	1.222085292	1003.80672	0.892985551	915	1.468080635
1230.59813	1.222714845	1004.3736	0.893177632	915.598132	1.468648156
1233.80672	1.222927672	1005	0.89339089	917.298752	1.470255966
1235.59813	1.223255423	1005.59813	0.8935954	917.582188	1.470522986
1238.80672	1.223764505	1007.29875	0.894184051	918.806723	1.471672776

Final State: j’=5		Final State: j’=4		Final State: j’=3	
E(cm⁻¹)	σ (Å²)	E(cm⁻¹)	σ (Å²)	E(cm⁻¹)	σ (Å²)
1240.59813	1.223953158	1007.58219	0.894287563	919.373597	1.472202503
1243.80672	1.224381159	1008.80672	0.894733086	920	1.472785396
1245.59813	1.224590935	1009.3736	0.89493279	920.598132	1.47333883
1248.80672	1.22507748	1010	0.895154761	922.298752	1.474877233
1250.59813	1.225319852	1010.59813	0.895368204	922.582188	1.475121372
1253.80672	1.225755557	1012.29875	0.895981809	923.806723	1.475990885
1255.59813	1.226005607	1012.58219	0.896084951	924.373597	1.476622718
1258.80672	1.22643819	1013.80672	0.896533301	925	1.47753248
1260.59813	1.226680116	1014.3736	0.896742414	925.598132	1.477752201
1263.80672	1.227114788	1015	0.896974734	927.298752	1.47959642
1265.59813	1.227356943	1015.59813	0.897197862	927.582188	1.479651704
1268.80672	1.227789828	1017.29875	0.89783834	928.806723	1.481193748
1270.59813	1.228031462	1017.58219	0.897945782	929.373597	1.481522298
1275.59813	1.228706286	1018.80672	0.898412034	930	1.48224643
1280.59813	1.229381948	1019.3736	0.898629079	930.598132	1.482781768
1285.59813	1.230059374	1020	0.898869846	932.298752	1.484456242
1290.59813	1.230739371	1020.59813	0.89910068	932.582188	1.484503597
1295.59813	1.231422624	1022.29875	0.89976206	933.806723	1.485876766
1300.59813	1.232109765	1022.58219	0.899873027	934.373597	1.48630235
1305.59813	1.23280134	1023.80672	0.900354867	935	1.48670406
1310.59813	1.233497819	1024.3736	0.900579262	935.598132	1.48724487
1315.59813	1.234199601	1025	0.900828201	937.298752	1.489195087
1320.59813	1.234907011	1025.59813	0.901066855	937.582188	1.489455148
1325.59813	1.235620308	1027.29875	0.901750406	938.806723	1.490408613
1330.59813	1.236339679	1028.80672	0.902362592	939.373597	1.490847507
1335.59813	1.237065253	1029.3736	0.902594177	940	1.491145176
1340.59813	1.237797091	1030	0.902851001	940.598132	1.491998351
1345.59813	1.238535206	1030.59813	0.903097127	942.298752	1.493782099
1350.59813	1.239279538	1032.29875	0.903801675	942.582188	1.493982798
1355.59813	1.240029987	1033.80672	0.904432257	943.806723	1.494980481
1360.59813	1.240786405	1034.3736	0.904670712	944.373597	1.49544838
1365.59813	1.241548585	1035	0.904935104	945	1.495936076
1370.59813	1.242316285	1035.59813	0.90518843	945.598132	1.496323587
1375.59813	1.243089219	1037.29875	0.9059133	947.298752	1.498212631
1380.59813	1.24386706	1038.80672	0.9065617	947.582188	1.498594456
1385.59813	1.244649454	1039.3736	0.906806803	948.806723	1.499505538
1390.59813	1.245436009	1040	0.907078502	949.373597	1.499978902
1395.59813	1.246226302	1040.59813	0.907338772	950	1.500503786
1400.59813	1.247019902	1042.29875	0.908083199	950.598132	1.500998966
1405.59813	1.247816326	1043.80672	0.908748707	952.298752	1.502481913
		1044.3736	0.909000186	952.582188	1.502552952
		1045	0.909278896	953.806723	1.504002805
		1045.59813	0.909545828	954.373597	1.504402805

Final State: j’=5		Final State: j’=4		Final State: j’=3	
E(cm⁻¹)	σ (Å²)	E(cm⁻¹)	σ (Å²)	E(cm⁻¹)	σ (Å²)
		1047.29875	0.910309008	955	1.504894502
		1048.80672	0.910990925	955.598132	1.505365241
		1049.3736	0.911248516	957.298752	1.506376615
		1050	0.911533945	957.582188	1.506436341
		1050.59813	0.911807257	958.806723	1.507683928
		1052.29875	0.912588406	959.373597	1.508326476
		1053.80672	0.913286023	960	1.509333837
		1054.3736	0.913549464	960.598132	1.509391877
		1055	0.913841319	962.298752	1.511346879
		1055.59813	0.914120733	962.582188	1.511447583
		1057.29875	0.91491904	963.806723	1.512555521
		1058.80672	0.915631644	964.373597	1.51336553
		1059.3736	0.91590066	965	1.513712194
		1060	0.91619864	965.598132	1.513955624
		1060.59813	0.916483872	967.298752	1.516060336
		1062.29875	0.917298525	967.582188	1.516215083
		1063.80672	0.918025395	968.806723	1.516942565
		1064.3736	0.918299717	969.373597	1.516869334
		1065	0.918603525	970	1.517988874
		1065.59813	0.918894287	970.598132	1.518910435
		1067.29875	0.919724478	972.298752	1.519977818
		1068.80672	0.920464887	972.582188	1.520158582
		1069.3736	0.920744244	973.806723	1.521079398
		1070	0.921053581	974.373597	1.521855497
		1070.59813	0.921349581	975	1.522148801
		1072.29875	0.922194493	975.598132	1.522693091
		1073.80672	0.922947727	977.298752	1.523767015
		1074.3736	0.923231847	977.582188	1.524117322
		1075	0.923546412	978.806723	1.525845552
		1075.59813	0.923847374	979.373597	1.525770485
		1077.29875	0.92470619	980	1.525969405
		1078.80672	0.925471524	980.598132	1.527172639
		1079.3736	0.925760139	982.298752	1.528247554
		1080	0.926079632	982.582188	1.52795587
		1080.59813	0.926385262	983.806723	1.52931933
		1083.80672	0.928033904	984.373597	1.530494165
		1084.3736	0.928326742	985	1.530679597
		1085	0.92865087	985.598132	1.530741463
		1085.59813	0.928960891	987.298752	1.53216833
		1088.80672	0.930632503	987.582188	1.532506056
		1089.3736	0.930929936	988.806723	1.533645874
		1090	0.931257769	989.373597	1.534079049
		1090.59813	0.931571899	990	1.534566727

Final State: j'=5		Final State: j'=4		Final State: j'=3	
E(cm⁻¹)	σ (Å²)	E(cm⁻¹)	σ (Å²)	E(cm⁻¹)	σ (Å²)
		1093.80672	0.933264989	990.598132	1.535043595
		1094.3736	0.933565483	992.298752	1.536498112
		1095	0.933897998	992.582188	1.536729427
		1095.59813	0.934215963	993.806723	1.537692252
		1098.80672	0.935929053	994.373597	1.538145848
		1099.3736	0.936232983	995	1.53864791
		1100.59813	0.936890792	995.598132	1.539127376
		1103.80672	0.938622432	997.298752	1.540506696
		1104.3736	0.938929547	997.582188	1.540735779
		1105.59813	0.939594128	998.806723	1.541720106
		1108.80672	0.941342903	999.373597	1.542174732
		1109.3736	0.941652947	1000	1.542669687
		1110.59813	0.942323772	1000.59813	1.543167611
		1113.80672	0.944088287	1002.29875	1.544514389
		1114.3736	0.944401021	1002.58219	1.544740134
		1115.59813	0.945077562	1003.80672	1.545714223
		1118.80672	0.946856464	1004.3736	1.546164366
		1119.3736	0.947171652	1005	1.54666116
		1120.59813	0.947853402	1005.59813	1.547135016
		1123.80672	0.949645378	1007.29875	1.548477941
		1124.3736	0.949962788	1007.58219	1.548700778
		1125.59813	0.950649257	1008.80672	1.549666294
		1128.80672	0.952453026	1009.3736	1.550112412
		1129.3736	0.952772437	1010	1.550604395
		1130.59813	0.95346314	1010.59813	1.551073481
		1133.80672	0.955277469	1012.29875	1.552403995
		1134.3736	0.955598665	1012.58219	1.552625296
		1135.59813	0.956293141	1013.80672	1.553579914
		1138.80672	0.958116824	1014.3736	1.554021012
		1139.3736	0.958439595	1015	1.554507829
		1140.59813	0.959137392	1015.59813	1.55497209
		1143.80672	0.960969259	1017.29875	1.556289191
		1144.3736	0.961293396	1017.58219	1.556508311
		1145.59813	0.96199407	1018.80672	1.557453623
		1148.80672	0.963832955	1019.3736	1.557890477
		1149.3736	0.964158253	1020	1.558372642
		1150.59813	0.964861347	1020.59813	1.5588325
		1153.80672	0.966706023	1022.29875	1.560137048
		1154.3736	0.967032256	1022.58219	1.560354055
		1155.59813	0.967737278	1023.80672	1.56129023
		1158.80672	0.969586256	1024.3736	1.561722874
		1159.3736	0.969913122	1025	1.562200406
		1160.59813	0.970619334	1025.59813	1.562655867

Final State: j’=5		Final State: j’=4		Final State: j’=3	
E(cm⁻¹)	σ (Å²)	E(cm⁻¹)	σ (Å²)	E(cm⁻¹)	σ (Å²)
		1163.80672	0.972469882	1027.29875	1.563948053
		1165.59813	0.973501912	1028.80672	1.565090472
		1168.80672	0.97533247	1029.3736	1.565519113
		1170.59813	0.976297961	1030	1.565992252
		1173.80672	0.978177669	1030.59813	1.566443527
		1175.59813	0.979268309	1032.29875	1.567723958
		1178.80672	0.981123922	1033.80672	1.568856075
		1180.59813	0.982237005	1034.3736	1.569280872
		1183.80672	0.984153305	1035	1.569749787
		1185.59813	0.985140245	1035.59813	1.570197052
		1188.80672	0.987055757	1037.29875	1.571466184
		1190.59813	0.988065771	1038.80672	1.572588443
		1193.80672	0.989990968	1039.3736	1.573009568
		1195.59813	0.990984125	1040	1.573474453
		1198.80672	0.992808738	1040.59813	1.573917892
		1200.59813	0.993887252	1042.29875	1.575176285
		1203.80672	0.995496133	1043.80672	1.576289156
		1205.59813	0.996659469	1044.3736	1.5767068
		1208.80672	0.998637999	1045	1.577167853
		1210.59813	0.999781483	1045.59813	1.577607657
		1213.80672	1.001669632	1047.29875	1.578855827
		1215.59813	1.002469378	1048.80672	1.579959806
		1218.80672	1.004504896	1049.3736	1.580374138
		1220.59813	1.00554254	1050	1.580831556
		1223.80672	1.007359304	1050.59813	1.581267923
		1225.59813	1.008411208	1052.29875	1.582506435
		1228.80672	1.010128039	1053.80672	1.583602007
		1230.59813	1.011483081	1054.3736	1.584013219
		1233.80672	1.013113179	1055	1.58446722
		1235.59813	1.014228155	1055.59813	1.584900339
		1238.80672	1.016087656	1057.29875	1.586129758
		1240.59813	1.017131826	1058.80672	1.587217425
		1243.80672	1.018996616	1059.3736	1.587625702
		1245.59813	1.020032325	1060	1.588076482
		1248.80672	1.021906279	1060.59813	1.58850656
		1250.59813	1.022949097	1062.29875	1.589727443
		1253.80672	1.024815335	1063.80672	1.590807706
		1255.59813	1.025859799	1064.3736	1.591213241
		1258.80672	1.027730139	1065	1.591661013
		1260.59813	1.028774474	1065.59813	1.592088243
		1263.80672	1.030645761	1067.29875	1.593301173
		1265.59813	1.031691403	1068.80672	1.594374538
		1268.80672	1.033565474	1069.3736	1.59477752

Final State: j'=5		Final State: j'=4		Final State: j'=3	
E(cm⁻¹)	σ (Å²)	E(cm⁻¹)	σ (Å²)	E(cm⁻¹)	σ (Å²)
		1270.59813	1.034612525	1070	1.595222499
		1275.59813	1.037538301	1070.59813	1.595647082
		1280.59813	1.040469875	1072.29875	1.59685262
		1285.59813	1.043408216	1073.80672	1.597919594
		1290.59813	1.046354432	1074.3736	1.598320214
		1295.59813	1.049309654	1075	1.598762607
		1300.59813	1.052275053	1075.59813	1.599184745
		1305.59813	1.055251829	1077.29875	1.60038346
		1310.59813	1.058241227	1078.80672	1.601444545
		1315.59813	1.06124449	1079.3736	1.601842988
		1320.59813	1.064262895	1080	1.602283004
		1325.59813	1.067297719	1080.59813	1.602702897
		1330.59813	1.070350242	1083.80672	1.604951045
		1335.59813	1.073421741	1084.3736	1.605347499
		1340.59813	1.076513477	1085	1.605785345
		1345.59813	1.0796267	1085.59813	1.606203184
		1350.59813	1.082762629	1088.80672	1.608440739
		1355.59813	1.085922457	1089.3736	1.608837649
		1360.59813	1.089107347	1090	1.609271263
		1365.59813	1.092318407	1090.59813	1.609687243
		1370.59813	1.095556711	1093.80672	1.611915221
		1375.59813	1.098823278	1094.3736	1.612308255
		1380.59813	1.10211907	1095	1.612742353
		1385.59813	1.105444993	1095.59813	1.613156671
		1390.59813	1.108801889	1098.80672	1.615376088
		1395.59813	1.112190523	1099.3736	1.61576767
		1400.59813	1.115611607	1100.59813	1.616613032
		1405.59813	1.119065759	1103.80672	1.61882486
				1104.3736	1.61921517
				1105.59813	1.620057845
				1108.80672	1.622263042
				1109.3736	1.622652241
				1110.59813	1.623492579
				1113.80672	1.625692062
				1114.3736	1.626080312
				1115.59813	1.626918654
				1118.80672	1.629113299
				1119.3736	1.629500751
				1120.59813	1.630337426
				1123.80672	1.632528078
				1124.3736	1.632914872
				1125.59813	1.633750198
				1128.80672	1.635937645

Final State: j’=5		Final State: j’=4		Final State: j’=3	
E(cm⁻¹)	σ (Å²)	E(cm⁻¹)	σ (Å²)	E(cm⁻¹)	σ (Å²)
				1129.3736	1.636323928
				1130.59813	1.637158192
				1133.80672	1.639343196
				1134.3736	1.639729097
				1135.59813	1.640562584
				1138.80672	1.64274587
				1139.3736	1.643131514
				1140.59813	1.643964502
				1143.80672	1.646146777
				1144.3736	1.646532292
				1145.59813	1.647365042
				1148.80672	1.649547023
				1149.3736	1.649932533
				1150.59813	1.650765327
				1153.80672	1.65294782
				1154.3736	1.653333486
				1155.59813	1.654166699
				1158.80672	1.656350869
				1159.3736	1.656736952
				1160.59813	1.657571221
				1163.80672	1.659759949
				1165.59813	1.660985151
				1168.80672	1.663205601
				1170.59813	1.664487077
				1173.80672	1.666671164
				1175.59813	1.667815804
				1178.80672	1.670015881
				1180.59813	1.671158015
				1183.80672	1.673276383
				1185.59813	1.67457317
				1188.80672	1.676696779
				1190.59813	1.677964048
				1193.80672	1.680073046
				1195.59813	1.681367025
				1198.80672	1.683560507
				1200.59813	1.684791884
				1203.80672	1.687273436
				1205.59813	1.688300001
				1208.80672	1.690436169
				1210.59813	1.6915298
				1213.80672	1.693666348
				1215.59813	1.695204522
				1218.80672	1.697216847

Final State: j'=5		Final State: j'=4		Final State: j'=3	
E(cm⁻¹)	σ (Å²)	E(cm⁻¹)	σ (Å²)	E(cm⁻¹)	σ (Å²)
				1220.59813	1.698376631
				1223.80672	1.700690667
				1225.59813	1.701910879
				1228.80672	1.704177376
				1230.59813	1.705111068
				1233.80672	1.707509447
				1235.59813	1.708698767
				1238.80672	1.710876165
				1240.59813	1.712099458
				1243.80672	1.714274435
				1245.59813	1.715478682
				1248.80672	1.717669269
				1250.59813	1.718882792
				1253.80672	1.721053531
				1255.59813	1.722265735
				1258.80672	1.724431796
				1260.59813	1.725639398
				1263.80672	1.727799223
				1265.59813	1.729003127
				1268.80672	1.731155561
				1270.59813	1.732355055
				1275.59813	1.735693827
				1280.59813	1.739017921
				1285.59813	1.742325818
				1290.59813	1.745615944
				1295.59813	1.748886803
				1300.59813	1.752136808
				1305.59813	1.755364407
				1310.59813	1.758568073
				1315.59813	1.76174623
				1320.59813	1.76489739
				1325.59813	1.768020065
				1330.59813	1.771112793
				1335.59813	1.774174149
				1340.59813	1.777202769
				1345.59813	1.780197322
				1350.59813	1.783156522
				1355.59813	1.78607916
				1360.59813	1.788964078
				1365.59813	1.791810202
				1370.59813	1.794616511
				1375.59813	1.79738207
				1380.59813	1.800106021

Final State: j’=5		Final State: j’=4		Final State: j’=3	
E(cm⁻¹)	σ (Å²)	E(cm⁻¹)	σ (Å²)	E(cm⁻¹)	σ (Å²)
				1385.59813	1.802787576
				1390.59813	1.805426051
				1395.59813	1.808020817
				1400.59813	1.810571344
				1405.59813	1.8130772

	Initial rotational state: j=0		
**Final State: j’=2**		**Final State: j’=1**	
E(cm^{-1})	σ (Å^2)	E(cm^{-1})	σ (Å^2)
82.3987517	0.316672833	27.6821883	2.999795315
82.4987517	0.521875895	27.7821883	6.325840389
82.5821883	0.427898769	27.8821883	4.404951593
82.5987517	0.431200531	27.9821883	6.166922887
82.6987517	0.464228747	28.0821883	5.646817069
82.7987517	0.489309089	28.1821883	5.18845999
82.8987517	0.664893794	28.2821883	5.752077659
82.9987517	0.681127226	28.3821883	5.357845426
83.0987517	0.641616897	28.4821883	5.567257833
83.1987517	0.653956308	37.5821883	11.3747703
87.2987517	1.101840884	42.5821883	12.51818423
87.3987517	1.107427643	47.5821883	13.81204932
87.4987517	1.111427284	52.5821883	13.05206929
87.5821883	1.113432991	57.5821883	10.16210166
87.5987517	1.113681333	62.5821883	12.55967662
87.6987517	1.114144301	67.5821883	10.92216016
87.7987517	1.11318533	72.5821883	10.49879702
92.2987517	1.377592169	77.5821883	11.48646768
92.5821883	1.400242898	82.3987517	11.1337056
97.2987517	1.950249926	82.4987517	10.43267638
97.5821883	1.759738601	82.5821883	10.94074938
100	1.748678578	82.5987517	10.95648203
102.298752	2.235995756	82.6987517	10.95595584
102.582188	2.477814605	82.7987517	10.74874747
105	2.91515805	82.8987517	9.675142823
107.298752	3.092607192	82.9987517	9.092366823
107.582188	3.111369442	83.0987517	10.10066633
110	2.521721409	83.1987517	10.14671543
112.298752	2.664191792	87.2987517	10.05282953
112.582188	2.683243148	87.3987517	10.09352787
115	2.955219576	87.4987517	10.14782872
117.298752	2.164156448	87.5821883	10.20772132
117.582188	2.173572122	87.5987517	10.2216328
120	2.722798424	87.6987517	10.32450455
122.298752	2.139788303	87.7987517	10.47291493
122.582188	2.14999993	92.2987517	9.29320994
125	3.596636551	92.5821883	9.29431585
127.298752	2.187233134	97.2987517	8.715778703
127.582188	2.167127767	97.5821883	8.616115035
130	2.458415544	100	8.847859392
132.298752	3.086019163	102.298752	8.576855654
132.582188	3.285714691	102.582188	8.455980842

Final State: j’=2		Final State: j’=1	
E(cm^{-1})	σ (Å^2)	E(cm^{-1})	σ (Å^2)
135	2.376456095	105	8.343223512
137.298752	3.403361414	107.298752	8.136671055
137.582188	3.991631971	107.582188	8.146303353
140	3.299351151	110	8.399623226
142.298752	3.578383571	112.298752	8.320448288
142.582188	3.503126965	112.582188	8.350676311
145	4.511447441	115	8.209866839
147.298752	2.503628827	117.298752	8.551032327
147.582188	2.537340843	117.582188	8.536408816
150	2.580862321	120	8.264422343
152.298752	3.4768621	122.298752	8.530854649
152.582188	4.255583343	122.582188	8.519013099
155	3.068322726	125	7.89903691
157.298752	5.17459529	127.298752	8.495026621
157.582188	4.536285308	127.582188	8.494063279
160	2.767910679	130	8.320033899
162.298752	4.059170676	132.298752	8.034191847
162.582188	4.22335151	132.582188	7.963418927
164.473597	2.746064808	135	8.319772291
164.573597	3.092721955	137.298752	7.840223442
164.673597	2.879921956	137.582188	7.612977955
164.773597	2.996561907	140	7.8747336
164.873597	3.27171474	142.298752	7.699341647
164.973597	4.111701381	142.582188	7.743812391
165	4.35284738	145	7.362383854
165.073597	3.916730632	147.298752	8.272412864
165.173597	3.169009664	147.582188	8.23977956
165.273597	3.340231361	150	8.233089798
165.373597	3.844224716	152.298752	7.794549149
165.473597	3.093271418	152.582188	7.463438135
165.573597	2.976304037	155	7.949736254
167.298752	3.121454	157.298752	6.940767319
167.582188	3.066751194	157.582188	7.275039612
169.373597	3.221030886	160	8.108161592
169.473597	3.251485816	162.298752	7.576491026
169.573597	3.289623385	162.582188	7.494131143
169.673597	3.340301819	164.473597	8.02029405
169.773597	3.412835168	164.573597	7.769638223
169.873597	3.526972357	164.673597	7.917676459
169.973597	3.729707958	164.773597	7.845345238
170	3.810168266	164.873597	7.701701012
170.073597	4.136086162	164.973597	7.282741832
172.298752	3.138801348	165	7.156401554

Final State: j'=2		Final State: j'=1	
E(cm^{-1})	σ (Å^2)	E(cm^{-1})	σ (Å^2)
172.582188	3.1429193	165.073597	7.381742869
174.373597	3.222365736	165.173597	7.744579367
175	3.270137012	165.273597	7.650172675
177.298752	3.342490138	165.373597	7.399708717
177.582188	3.354468611	165.473597	7.779657631
179.373597	3.473711946	165.573597	7.829320858
180	3.321749355	167.298752	7.645990387
182.298752	3.394370739	167.582188	7.662606995
182.582188	3.400770426	169.373597	7.586317567
184.373597	3.441144918	169.473597	7.573492694
185	3.45708178	169.573597	7.559031448
187.298752	3.523758143	169.673597	7.541631188
187.582188	3.515954512	169.773597	7.518786938
189.373597	3.521968369	169.873597	7.485193738
190	3.532078956	169.973597	7.428237431
192.298752	3.572097584	170	7.406031105
192.582188	3.577212589	170.073597	7.316944778
194.373597	3.610038866	172.298752	7.536862182
195	3.620910451	172.582188	7.536439331
197.298752	3.654582954	174.373597	7.487707169
197.582188	3.658539505	175	7.449088088
199.373597	3.684179074	177.298752	7.387850447
200	3.693334685	177.582188	7.386035882
202.298752	3.72696156	179.373597	7.25469438
202.582188	3.731048817	180	7.377640042
204.373597	3.756106279	182.298752	7.334842098
205	3.76442898	182.582188	7.331123634
207.298752	3.791945906	184.373597	7.308184682
207.582188	3.794880169	185	7.299120743
209.373597	3.808850237	187.298752	7.23072044
210	3.810277489	187.582188	7.234018999
212.298752	3.720675508	189.373597	7.242511544
212.582188	3.718654668	190	7.238198693
214.373597	3.793984463	192.298752	7.217139487
215	3.883101577	192.582188	7.214246679
217.298752	3.853463787	194.373597	7.19422635
217.582188	3.912473992	195	7.18677046
219.373597	3.923414048	197.298752	7.163828586
220	3.869305536	197.582188	7.161431507
222.298752	4.054086049	199.373597	7.146667339
222.582188	4.047712515	200	7.141525504
224.373597	4.000570028	202.298752	7.122658747
225	4.055925851	202.582188	7.12035048

Final State: j’=2		Final State: j’=1	
E(cm^{-1})	σ (Å^2)	E(cm^{-1})	σ (Å^2)
227.298752	4.121578812	204.373597	7.106103989
227.582188	4.110018206	205	7.101359547
229.373597	4.117817947	207.298752	7.085939792
230	4.08057004	207.582188	7.084349679
232.298752	4.215166078	209.373597	7.077203715
232.582188	4.209029109	210	7.076750812
234.373597	4.071957892	212.298752	7.104895612
235	4.224506166	212.582188	7.095123032
237.298752	4.312825577	214.373597	7.047351968
237.582188	4.309421657	215	7.00253445
239.373597	4.272324407	217.298752	6.999624527
240	4.212388367	217.582188	6.971729062
242.298752	4.406668279	219.373597	6.988742216
242.582188	4.400646477	220	6.987057803
244.373597	4.350745011	222.298752	6.910117668
245	4.31859628	222.582188	6.920726697
247.298752	4.126201942	224.373597	6.900402658
247.582188	4.2040101	225	6.905211715
249.373597	4.307339979	227.298752	6.884656761
250	4.373640456	227.582188	6.892154047
252.298752	4.367478635	229.373597	6.842863055
252.582188	4.472999991	230	6.889471808
254.373597	4.256443654	232.298752	6.833518469
255	4.247376835	232.582188	6.838247255
257.298752	4.568859313	234.373597	6.858153367
257.582188	4.521549734	235	6.788569938
259.373597	4.673097458	237.298752	6.777785721
260	4.654262473	237.582188	6.781670283
262.298752	4.341311802	239.373597	6.80789269
262.582188	4.397126394	240	6.830951816
264.373597	4.634544009	242.298752	6.702035902
265	4.617683424	242.582188	6.722702348
267.298752	4.786077862	244.373597	6.767851591
267.582188	4.77919347	245	6.786780123
269.373597	4.753175745	247.298752	6.803578497
270	4.548137704	247.582188	6.755618666
272.298752	4.552690754	249.373597	6.732356032
272.582188	4.646935977	250	6.698393608
273.906723	4.775228432	252.298752	6.69119079
274.006723	4.672573882	252.582188	6.645745815
274.106723	4.73497818	254.373597	6.780611065
274.206723	4.698980637	255	6.686403918
274.306723	4.637631746	257.298752	6.636562931

Final State: j'=2		Final State: j'=1	
E(cm^{-1})	σ (Å^2)	E(cm^{-1})	σ (Å^2)
274.373597	4.5474114	257.582188	6.661823206
274.406723	4.486376656	259.373597	6.560391235
274.506723	4.577058718	260	6.58787937
274.606723	4.721637511	262.298752	6.679615104
274.706723	4.676752159	262.582188	6.618727594
274.806723	4.592057104	264.373597	6.576526363
274.906723	4.756718254	265	6.593075025
275	4.761088398	267.298752	6.466671948
275.006723	4.760619953	267.582188	6.504226545
275.106723	4.750942739	269.373597	6.537908138
275.206723	4.739383179	270	6.640464211
277.298752	4.75679546	272.298752	6.497531268
277.582188	4.754076407	272.582188	6.508755633
278.806723	4.719078683	273.906723	6.517188462
278.906723	4.714949939	274.006723	6.544413328
279.006723	4.710166801	274.106723	6.530869025
279.106723	4.704306953	274.206723	6.549266885
279.206723	4.696631345	274.306723	6.576084365
279.306723	4.685757633	274.373597	6.606617711
279.373597	4.675389685	274.406723	6.616065794
279.406723	4.668829045	274.506723	6.530064116
279.506723	4.640572897	274.606723	6.496048478
279.606723	4.612773005	274.706723	6.530064603
279.706723	4.645471826	274.806723	6.531889495
280	4.632884694	274.906723	6.477196083
282.298752	4.788704264	275	6.488966279
282.582188	4.788413533	275.006723	6.489765928
283.806723	4.787968815	275.106723	6.500259169
284.373597	4.78507027	275.206723	6.509005392
285	4.781492322	277.298752	6.471586175
287.298752	4.809569161	277.582188	6.476864659
287.582188	4.808154379	278.806723	6.494023786
288.806723	4.830109917	278.906723	6.495512271
289.373597	4.85688065	279.006723	6.497251568
290	4.856191284	279.106723	6.49937921
292.298752	4.875869595	279.206723	6.502058492
292.582188	4.878774086	279.306723	6.505484434
293.806723	4.891194638	279.373597	6.5082248
294.373597	4.896686375	279.406723	6.50965674
295	4.902244737	279.506723	6.512537548
297.298752	4.928469358	279.606723	6.499559477
297.582188	4.93289415	279.706723	6.467067282
298.806723	4.948083401	280	6.51100574

Final State: j’=2		Final State: j’=1	
E(cm⁻¹)	σ (Å²)	E(cm⁻¹)	σ (Å²)
299.373597	4.954473904	282.298752	6.437795478
300	4.961432299	282.582188	6.438306389
302.298752	4.986533343	283.806723	6.437108025
302.582188	4.989581611	284.373597	6.435491501
303.806723	5.00268516	285	6.430334147
304.373597	5.00880556	287.298752	6.412077086
305	5.01569852	287.582188	6.412139881
307.298752	5.04168703	288.806723	6.37501745
307.582188	5.044883	289.373597	6.380371952
308.806723	5.05859391	290	6.381360683
309.373597	5.06489305	292.298752	6.366825874
310	5.07182422	292.582188	6.364817365
312.298752	5.09706469	293.806723	6.356118365
312.582188	5.10016261	294.373597	6.352101601
313.806723	5.11353131	295	6.347655258
314.373597	5.11971594	297.298752	6.324769556
315	5.12654894	297.582188	6.32292451
317.298752	5.15160546	298.806723	6.314878014
317.582188	5.15469093	299.373597	6.310894635
318.806723	5.16800538	300	6.30638634
319.373597	5.17415954	302.298752	6.289395874
320	5.18095268	302.582188	6.287262919
322.298752	5.20581809	303.806723	6.277879401
322.582188	5.20887763	304.373597	6.27342669
323.806723	5.22208214	305	6.268464856
324.373597	5.22818825	307.298752	6.250628483
325	5.23493135	307.582188	6.248474808
327.298752	5.25964699	308.806723	6.239213702
327.582188	5.26269199	309.373597	6.234937586
328.806723	5.2758433	310	6.230214092
329.373597	5.28192999	312.298752	6.212851237
330	5.28865562	312.582188	6.210704789
332.298752	5.31334554	313.806723	6.201418621
332.582188	5.31639204	314.373597	6.197115188
333.806723	5.32956465	315	6.19235927
334.373597	5.3356703	317.298752	6.174928072
335	5.34242463	317.582188	6.172782509
337.298752	5.36730886	318.806723	6.163521719
337.582188	5.37039149	319.373597	6.159238835
338.806723	5.38376066	320	6.15450859
339.373597	5.38998381	322.298752	6.137162731
340	5.39689188	322.582188	6.135024693
342.298752	5.4226482	323.806723	6.125788156

Final State: j’=2		Final State: j’=1	
E(cm⁻¹)	σ (Å²)	E(cm⁻¹)	σ (Å²)
342.582188	5.42588566	324.373597	6.121512389
343.806723	5.44011277	325	6.116787527
344.373597	5.44687392	327.298752	6.099446803
345	5.45452858	327.582188	6.097308469
347.298752	5.48611769	328.806723	6.088068915
347.582188	5.4908594	329.373597	6.083790709
348.806723	5.51827173	330	6.079062435
349.373597	5.51723156	332.298752	6.061697977
350	5.4952629	332.582188	6.059554991
352.298752	5.51967137	333.806723	6.050289661
352.582188	5.53082096	334.373597	6.045995936
353.806723	5.53391101	335	6.0412473
354.373597	5.53114469	337.298752	6.023772701
355	5.54170506	337.582188	6.021611435
357.298752	5.54604385	338.806723	6.012251955
357.582188	5.55349812	339.373597	6.007904782
358.806723	5.55249531	340	6.00308826
359.373597	5.57719425	342.298752	5.985254035
360	5.59877574	342.582188	5.983031715
362.298752	5.60893601	343.806723	5.973343521
362.582188	5.57805875	344.373597	5.968796961
363.806723	5.61957313	345	5.963710742
364.373597	5.63464252	347.298752	5.944010673
365	5.65458769	347.582188	5.941379388
367.298752	5.62087491	348.806723	5.931176335
367.582188	5.62565689	349.373597	5.938149622
368.806723	5.66663671	350	5.946213738
369.373597	5.6790864	352.298752	5.918161955
370	5.69392118	352.582188	5.912102461
372.298752	5.70538804	353.806723	5.922350667
372.582188	5.67515717	354.373597	5.918074935
373.806723	5.70890423	355	5.902055691
374.373597	5.72135083	357.298752	5.903033174
375	5.73358838	357.582188	5.89353472
377.298752	5.77541449	358.806723	5.889397733
377.582188	5.74591412	359.373597	5.870754186
378.806723	5.74758434	360	5.86092191
379.373597	5.76204046	362.298752	5.871958252
380	5.78213167	362.582188	5.871230676
382.298752	5.84750943	363.806723	5.837769243
382.582188	5.85436279	364.373597	5.830274347
383.806723	5.85385772	365	5.823600857
384.373597	5.82785981	367.298752	5.839933179

Final State: j'=2		Final State: j'=1	
E(cm^{-1})	σ (Å^2)	E(cm^{-1})	σ (Å^2)
385	5.84335719	367.582188	5.826221192
387.298752	5.82297391	368.806723	5.801331736
387.582188	5.84167967	369.373597	5.794626215
388.806723	5.87303444	370	5.787673677
389.373597	5.82154211	372.298752	5.794114653
390	5.85884497	372.582188	5.801386623
392.298752	5.82900387	373.806723	5.767073844
392.582188	5.82399398	374.373597	5.759956895
393.806723	5.89376106	375	5.753039097
394.373597	5.925534	377.298752	5.756640744
395	5.95169845	377.582188	5.760461885
397.298752	5.93350853	378.806723	5.745922307
397.582188	5.94392471	379.373597	5.727071698
398.806723	6.00838919	380	5.716144852
399.373597	5.93822473	382.298752	5.687606379
400	5.92765518	382.582188	5.689621431
402.298752	6.0118026	383.806723	5.695181844
402.582188	6.00710159	384.373597	5.724351687
403.806723	5.95036334	385	5.701882604
404.373597	5.95995424	387.298752	5.684087532
405	6.04418082	387.582188	5.672890802
407.298752	6.03826053	388.806723	5.682830543
407.582188	6.04472309	389.373597	5.690686446
408.806723	6.02362447	390	5.658107113
409.373597	6.0148006	392.298752	5.686424524
410	5.99560917	392.582188	5.698268589
410.698132	6.06102955	393.806723	5.627159491
410.798132	6.07402273	394.373597	5.615337505
410.898132	6.06800072	395	5.630906142
410.998132	6.08019463	397.298752	5.599345917
411.098132	6.09615808	397.582188	5.594447576
411.198132	6.10969924	398.806723	5.590848109
411.298132	6.06371016	399.373597	5.63668237
411.398132	6.03732743	400	5.608387797
411.498132	6.06357795	402.298752	5.566502389
411.598132	6.06451832	402.582188	5.567615622
411.698132	6.03196959	403.806723	5.617756417
411.798132	6.04613957	404.373597	5.569404004
411.898132	6.0555203	405	5.54868704
411.998132	6.06263293	407.298752	5.55404683
412.098132	6.06877649	407.582188	5.537994143
412.198132	6.07464704	408.806723	5.542477578
412.298752	6.08079505	409.373597	5.556803228

Final State: j'=2		Final State: j'=1	
E(cm⁻¹)	σ (Å²)	E(cm⁻¹)	σ (Å²)
412.582188	6.10470585	410	5.538146366
413.806723	6.06047501	410.698132	5.505983585
414.373597	6.07426419	410.798132	5.511403628
415	6.08695164	410.898132	5.506053361
415.598132	6.09710126	410.998132	5.502262501
415.698132	6.09880625	411.098132	5.499789374
415.798132	6.1006009	411.198132	5.513565157
415.898132	6.10253823	411.298132	5.533271623
415.998132	6.10469442	411.398132	5.532780132
416.098132	6.10717144	411.498132	5.516293612
416.198132	6.11006855	411.598132	5.533808816
416.298132	6.11283889	411.698132	5.529447249
416.398132	6.10695557	411.798132	5.517887921
416.498132	6.10200859	411.898132	5.512566792
416.598132	6.10117227	411.998132	5.509195783
416.698132	6.11109915	412.098132	5.506648949
417.298752	6.09192997	412.198132	5.504517651
417.582188	6.08622058	412.298752	5.502632786
418.806723	6.10889503	412.582188	5.500491796
419.373597	6.11631834	413.806723	5.509916796
420	6.12341133	414.373597	5.500936104
420.598132	6.12978952	415	5.49455779
422.298752	6.14277338	415.598132	5.490523617
422.582188	6.14444722	415.698132	5.489943941
423.806723	6.15485828	415.798132	5.48938892
424.373597	6.16071542	415.898132	5.4888792
425	6.16218814	415.998132	5.48847071
425.598132	6.15872582	416.098132	5.488312646
427.298752	6.18048988	416.198132	5.488869559
427.582188	6.18286981	416.298132	5.491978129
428.806723	6.19281623	416.398132	5.502022425
429.373597	6.19736269	416.498132	5.503930643
430	6.20237322	416.598132	5.49967979
430.598132	6.20714846	416.698132	5.491380524
432.298752	6.22063385	417.298752	5.498513559
432.582188	6.22276531	417.582188	5.498435803
433.806723	6.2307247	418.806723	5.476400677
434.373597	6.23515754	419.373597	5.471832431
435	6.24020377	420	5.467423317
435.598132	6.24495398	420.598132	5.463569914
437.298752	6.25819244	422.298752	5.456296544
437.582188	6.26037604	422.582188	5.454673036
438.806723	6.26975956	423.806723	5.446260325

Final State: j’=2		Final State: j’=1	
E(cm⁻¹)	σ (Å²)	E(cm⁻¹)	σ (Å²)
439.373597	6.2740759	424.373597	5.442699626
440	6.27882105	425	5.449280328
440.598132	6.2833234	425.598132	5.442712517
442.298752	6.29600009	427.298752	5.42502806
442.582188	6.29810762	427.582188	5.423320081
443.806723	6.30722236	428.806723	5.415905285
444.373597	6.31144269	429.373597	5.412456763
445	6.31610197	430	5.408652203
445.598132	6.32054435	430.598132	5.405036761
447.298752	6.33312791	432.298752	5.395133434
447.582188	6.33521753	432.582188	5.393696316
448.806723	6.34421847	433.806723	5.387218713
449.373597	6.34837017	434.373597	5.383297283
450	6.35294676	435	5.379184239
450.598132	6.35730619	435.598132	5.375423209
452.298752	6.36964741	437.298752	5.365046333
452.582188	6.37169699	437.582188	5.363337739
453.806723	6.38052898	438.806723	5.35600012
454.373597	6.38460517	439.373597	5.35262867
455	6.38910032	440	5.348924692
455.598132	6.39338364	440.598132	5.345408188
457.298752	6.40551365	442.298752	5.335421608
457.582188	6.40752828	442.582188	5.33374617
458.806723	6.41620879	443.806723	5.326481691
459.373597	6.42021439	444.373597	5.32311633
460	6.42463105	445	5.319402155
460.598132	6.42883912	445.598132	5.315861972
462.298752	6.4407533	447.298752	5.305835437
462.582188	6.44273182	447.582188	5.304170048
463.806723	6.45125627	448.806723	5.296993348
464.373597	6.45518958	449.373597	5.293680482
465	6.4595265	450	5.290025899
465.598132	6.46365839	450.598132	5.286541722
467.298752	6.4753569	452.298752	5.276659838
467.582188	6.47729955	452.582188	5.275015986
468.806723	6.48566913	453.806723	5.267923942
469.373597	6.48953083	454.373597	5.264646394
470	6.49378863	455	5.261029003
470.598132	6.49784504	455.598132	5.257579255
472.298752	6.50932894	457.298752	5.247795455
472.582188	6.51123582	457.582188	5.246168461
473.806723	6.51945087	458.806723	5.239151561
474.373597	6.52324105	459.373597	5.235909959

Final State: j'=2		Final State: j'=1	
E(cm⁻¹)	σ (Å²)	E(cm⁻¹)	σ (Å²)
475	6.52741979	460	5.232332956
475.598132	6.53140074	460.598132	5.228922129
477.298752	6.54267002	462.298752	5.219250369
477.582188	6.54454116	462.582188	5.217642023
478.806723	6.55260167	463.806723	5.210705453
479.373597	6.55632034	464.373597	5.207500822
480	6.56042005	465	5.203964398
480.598132	6.56432551	465.598132	5.200592274
482.298752	6.5753802	467.298752	5.191029395
482.582188	6.57721555	467.582188	5.189439169
483.806723	6.58512146	468.806723	5.182580643
484.373597	6.5887685	469.373597	5.179412099
485	6.59278905	470	5.175915563
485.598132	6.59661886	470.598132	5.172581503
487.298752	6.60745829	472.298752	5.163127015
487.582188	6.60925774	472.582188	5.161554823
488.806723	6.61700832	473.806723	5.154774364
489.373597	6.62058333	474.373597	5.151641928
490	6.62452426	475	5.148185299
490.598132	6.6282779	475.598132	5.144889357
492.298752	6.63890019	477.298752	5.135542954
492.582188	6.64066336	477.582188	5.133988793
493.806723	6.64825674	478.806723	5.127285942
494.373597	6.65175879	479.373597	5.124189402
495	6.65561879	480	5.12077239
495.598132	6.65929502	480.598132	5.117514237
497.298752	6.66969566	482.298752	5.10827513
497.582188	6.67142164	482.582188	5.106738821
498.806723	6.67885341	483.806723	5.100113022
499.373597	6.68227998	484.373597	5.097052087
500	6.68605613	485	5.0936744
500.598132	6.68965166	485.598132	5.090453719
502.298752	6.69981904	487.298752	5.081321023
502.582188	6.70150542	487.582188	5.07980243
503.806723	6.70876349	488.806723	5.073252985
504.373597	6.7121078	489.373597	5.070227351
505	6.7157913	490	5.066888633
505.598132	6.71929641	490.598132	5.0637051
507.298752	6.7291924	492.298752	5.054677718
507.582188	6.73083087	492.582188	5.053176624
508.806723	6.73786881	493.806723	5.046702681
509.373597	6.74110187	494.373597	5.043711923
510	6.74465298	495	5.040411654

Final State: j’=2		Final State: j’=1	
E(cm⁻¹)	σ (Å²)	E(cm⁻¹)	σ (Å²)
510.598132	6.74802005	495.598132	5.037264773
512.298752	6.75750953	497.298752	5.028341179
512.582188	6.75918798	497.582188	5.026857337
513.806723	6.77514167	498.806723	5.020457593
514.373597	6.77698991	499.373597	5.017501007
515	6.77675945	500	5.014238395
515.598132	6.7867613	500.598132	5.011127348
517.298752	6.79102088	502.298752	5.002304778
517.582188	6.795711	502.582188	5.00083757
518.806723	6.80318861	503.806723	4.994509327
519.373597	6.80374408	504.373597	4.991585451
520	6.8058909	505	4.988358525
520.598132	6.80807561	505.598132	4.98528111
522.298752	6.81949195	507.298752	4.976550378
522.582188	6.83171425	507.582188	4.975097718
523.806723	6.82519929	508.806723	4.968827717
524.373597	6.82810375	509.373597	4.965926927
525	6.84029563	510	4.96272049
525.598132	6.84122257	510.598132	4.959654135
527.298752	6.84559419	512.298752	4.9507632
527.582188	6.8463087	512.582188	4.949152147
528.806723	6.85171879	513.806723	4.938976666
529.373597	6.8559069	514.373597	4.937855668
530	6.87019373	515	4.935649453
530.598132	6.86482272	515.598132	4.930157058
532.298752	6.87140541	517.298752	4.923048605
532.582188	6.87247035	517.582188	4.920458072
533.806723	6.8780429	518.806723	4.915491677
534.373597	6.88097732	519.373597	4.913101476
535	6.88728487	520	4.911212518
535.598132	6.89748697	520.598132	4.90779183
537.298752	6.89821379	522.298752	4.898690398
537.582188	6.89859675	522.582188	4.893649914
538.806723	6.90365515	523.806723	4.893083192
539.373597	6.90629614	524.373597	4.889742951
540	6.90929974	525	4.882618661
540.598132	6.91239596	525.598132	4.881923527
542.298752	6.92496727	527.298752	4.876277903
542.582188	6.93602643	527.582188	4.87496741
543.806723	6.92840151	528.806723	4.86859255
544.373597	6.93058278	529.373597	4.864578622
545	6.93319612	530	4.858367339
545.598132	6.93572592	530.598132	4.858976633

Final State: j’=2		Final State: j’=1	
E(cm⁻¹)	σ (Å²)	E(cm⁻¹)	σ (Å²)
547.298752	6.95446485	532.298752	4.85241198
547.582188	6.95625765	532.582188	4.8510304
548.806723	6.96864536	533.806723	4.844838386
549.373597	6.97221319	534.373597	4.841705478
550	6.96423877	535	4.83649096
550.598132	6.97786199	535.598132	4.832472851
552.298752	6.97229973	537.298752	4.828765493
552.582188	6.97831888	537.582188	4.827487277
553.806723	6.98630776	538.806723	4.821474413
554.373597	6.98395255	539.373597	4.818654442
555	6.98524823	540	4.815462543
555.598132	7.00125703	540.598132	4.8121681
557.298752	6.9998067	542.298752	4.803059922
557.582188	6.99860354	542.582188	4.798509251
558.806723	7.00263325	543.806723	4.798432041
559.373597	7.02604973	544.373597	4.79563228
560	7.0197968	545	4.792546347
560.598132	7.01380011	545.598132	4.78955541
562.298752	7.01764181	547.298752	4.775861445
562.582188	7.01904383	547.582188	4.774864724
563.806723	7.03892285	548.806723	4.767812164
564.373597	7.03546314	549.373597	4.766477346
565	7.0348505	550	4.767332894
565.598132	7.03958745	550.598132	4.759103236
567.298752	7.04643675	552.298752	4.756697818
567.582188	7.06453134	552.582188	4.752488534
568.806723	7.04960725	553.806723	4.749115215
569.373597	7.07013109	554.373597	4.747511825
570	7.06027575	555	4.743947917
570.598132	7.05651075	555.598132	4.733640387
572.298752	7.0900512	557.298752	4.735818972
572.582188	7.08172031	557.582188	4.734588621
573.806723	7.07639445	558.806723	4.726907585
574.373597	7.07674741	559.373597	4.715769397
575	7.080041	560	4.72103309
575.598132	7.08942578	560.598132	4.720438679
577.298752	7.10530993	562.298752	4.712163691
577.582188	7.09904129	562.582188	4.710151082
578.806723	7.10150396	563.806723	4.703366973
579.373597	7.10418959	564.373597	4.700454057
580	7.10761441	565	4.699536612
580.598132	7.11239748	565.598132	4.69504934
582.298752	7.11895937	567.298752	4.68536862

Final State: j'=2		Final State: j'=1	
E(cm⁻¹)	σ (Å²)	E(cm⁻¹)	σ (Å²)
582.582188	7.11964732	567.582188	4.677051345
583.806723	7.12482828	568.806723	4.683780982
584.373597	7.12738664	569.373597	4.672810241
585	7.13030358	570	4.676946337
585.598132	7.13328799	570.598132	4.675254822
587.298752	7.14105192	572.298752	4.657225858
587.582188	7.14223164	572.582188	4.661050615
588.806723	7.14782488	573.806723	4.661474453
589.373597	7.15080887	574.373597	4.657545632
590	7.1528683	575	4.65437073
590.598132	7.1548046	575.598132	4.649611099
592.298752	7.16221119	577.298752	4.638961434
592.582188	7.16342122	577.582188	4.641781199
593.806723	7.1686135	578.806723	4.636044926
594.373597	7.17100631	579.373597	4.633139004
595	7.17364738	580	4.62974994
595.598132	7.17617106	580.598132	4.626635776
597.298752	7.18343223	582.298752	4.620678875
597.582188	7.18459283	582.582188	4.619431172
598.806723	7.18946079	583.806723	4.613734544
599.373597	7.19175954	584.373597	4.611128211
600	7.19431002	585	4.608225618
600.598132	7.1967451	585.598132	4.605409062
602.298752	7.20364398	587.298752	4.598032572
602.582188	7.20478952	587.582188	4.596809103
603.806723	7.20972424	588.806723	4.591159512
604.373597	7.21200026	589.373597	4.58897121
605	7.21450764	590	4.586777215
605.598132	7.21689263	590.598132	4.584176695
607.298752	7.22362263	592.298752	4.576688735
607.582188	7.22473816	592.582188	4.575465303
608.806723	7.22954143	593.806723	4.570199603
609.373597	7.23175634	594.373597	4.567767685
610	7.23419764	595	4.56508164
610.598132	7.23652238	595.598132	4.562516111
612.298752	7.24309772	597.298752	4.555267906
612.582188	7.24418856	597.582188	4.554108349
613.806723	7.24888445	598.806723	4.549009221
614.373597	7.25104889	599.373597	4.546612215
615	7.25343367	600	4.543964395
615.598132	7.2557039	600.598132	4.54143969
617.298752	7.26212218	602.298752	4.534281509
617.582188	7.26318668	602.582188	4.53309116

Final State: j'=2		Final State: j'=1	
E(cm⁻¹)	σ (Å²)	E(cm⁻¹)	σ (Å²)
618.806723	7.26776856	603.806723	4.527957378
619.373597	7.26988032	604.373597	4.52558648
620	7.27220699	605	4.522971857
620.598132	7.2744219	605.598132	4.520480692
622.298752	7.28068385	607.298752	4.513418494
622.582188	7.28172235	607.582188	4.512243296
623.806723	7.28619217	608.806723	4.507171335
624.373597	7.2882521	609.373597	4.504826731
625	7.29052152	610	4.502238428
625.598132	7.29268181	610.598132	4.499769756
627.298752	7.29878829	612.298752	4.492766324
627.582188	7.29980092	612.582188	4.491601383
628.806723	7.30415888	613.806723	4.486576299
629.373597	7.30616704	614.373597	4.48425431
630	7.30837929	615	4.481691608
630.598132	7.31048504	615.598132	4.479247583
632.298752	7.31643659	617.298752	4.472314435
632.582188	7.3174234	617.582188	4.471161121
633.806723	7.32166996	618.806723	4.466185646
634.373597	7.32362661	619.373597	4.463886281
635	7.32578197	620	4.461348323
635.598132	7.3278334	620.598132	4.45892773
637.298752	7.33363063	622.298752	4.452060507
637.582188	7.33459174	622.582188	4.450918114
638.806723	7.33872734	623.806723	4.445989832
639.373597	7.34063265	624.373597	4.443712256
640	7.34273129	625	4.441198398
640.598132	7.34472859	625.598132	4.438800768
642.298752	7.35037204	627.298752	4.431998652
642.582188	7.35130755	627.582188	4.430867081
643.806723	7.35533255	628.806723	4.425985338
644.373597	7.3571867	629.373597	4.42372922
645	7.3592288	630	4.421238973
645.598132	7.36117215	630.598132	4.418863856
647.298752	7.36666233	632.298752	4.412125298
647.582188	7.36757231	632.582188	4.411004275
648.806723	7.37148705	633.806723	4.406167873
649.373597	7.37329019	634.373597	4.403932687
650	7.37527597	635	4.401465487
650.598132	7.37716551	635.598132	4.39911229
652.298752	7.38250285	637.298752	4.392435783
652.582188	7.38338737	637.582188	4.39132506
653.806723	7.38719216	638.806723	4.38653301

Final State: j’=2		Final State: j’=1	
E(cm⁻¹)	σ (Å²)	E(cm⁻¹)	σ (Å²)
654.373597	7.38894443	639.373597	4.384318255
655	7.39087399	640	4.381873563
655.598132	7.39270991	640.598132	4.379541826
657.298752	7.3978948	642.298752	4.372925951
657.582188	7.39875391	642.582188	4.371825294
658.806723	7.40244902	643.806723	4.367076513
659.373597	7.40415051	644.373597	4.364881705
660	7.40602401	645	4.36245901
660.598132	7.40780638	645.598132	4.360148192
662.298752	7.41283912	647.298752	4.353591511
662.582188	7.41367288	647.582188	4.352500665
663.806723	7.41725849	648.806723	4.347794158
664.373597	7.4189093	649.373597	4.345618828
665	7.42072679	650	4.343217596
665.598132	7.4224557	650.598132	4.340927193
667.298752	7.42733648	652.298752	4.334428269
667.582188	7.42814491	652.582188	4.333347013
668.806723	7.43162111	653.806723	4.32868176
669.373597	7.43322126	654.373597	4.326525449
670	7.43498278	655	4.324145158
670.598132	7.43665824	655.598132	4.321874731
672.298752	7.44138699	657.298752	4.315432177
672.582188	7.44217008	657.582188	4.31436027
673.806723	7.44553678	658.806723	4.309735249
674.373597	7.44708623	659.373597	4.307597498
675	7.44879168	660	4.305237665
675.598132	7.45041362	660.598132	4.302986684
677.298752	7.45499	662.298752	4.296599184
677.582188	7.4557477	662.582188	4.295536388
678.806723	7.45900447	663.806723	4.290950683
679.373597	7.46050299	664.373597	4.288831056
680	7.46215214	665	4.286491162
680.598132	7.46372024	665.598132	4.284259188
682.298752	7.4681433	667.298752	4.27792546
682.582188	7.46887536	667.582188	4.276871575
683.806723	7.47202132	668.806723	4.272324261
684.373597	7.47346838	669.373597	4.270222309
685	7.47506059	670	4.26790195
685.598132	7.47657419	670.598132	4.265688547
687.298752	7.48084157	672.298752	4.259407356
687.582188	7.48154756	672.582188	4.258362207
688.806723	7.48458042	673.806723	4.25385246
689.373597	7.48597481	674.373597	4.251767841

Final State: j'=2		Final State: j'=1	
E(cm⁻¹)	σ (Å²)	E(cm⁻¹)	σ (Å²)
690	7.48750854	675	4.249466567
690.598132	7.48896601	675.598132	4.247271361
692.298752	7.4930719	677.298752	4.241041681
692.582188	7.49375064	677.582188	4.240005094
693.806723	7.49666433	678.806723	4.235532167
694.373597	7.49800267	679.373597	4.233464555
695	7.49947183	680	4.231182055
695.598132	7.50087017	680.598132	4.229004721
697.298752	7.50479587	682.298752	4.222825744
697.582188	7.50544327	682.582188	4.221797579
698.806723	7.50821517	683.806723	4.21736106
699.373597	7.50948321	684.373597	4.215310251
700	7.51087154	685	4.213046311
700.598132	7.51218268	685.598132	4.210886714
702.298752	7.51579865	687.298752	4.204758117
702.582188	7.51637601	687.582188	4.20373835
703.806723	7.51865026	688.806723	4.199338209
704.373597	7.51927373	689.373597	4.197304294
705	7.51955537	690	4.195059058
705.598132	7.52158521	690.598132	4.192917389
707.298752	7.52640132	692.298752	4.186840198
707.582188	7.52800325	692.582188	4.185829085
708.806723	7.52816347	693.806723	4.181466729
709.373597	7.52951028	694.373597	4.179450576
710	7.53276899	695	4.177225134
710.598132	7.53409448	695.598132	4.175102858
712.298752	7.53602952	697.298752	4.169082802
712.582188	7.53583908	697.582188	4.168081649
713.806723	7.53955199	698.806723	4.163764572
714.373597	7.54287445	699.373597	4.161771048
715	7.54391917	700	4.159572414
715.598132	7.54445305	700.598132	4.157477884
717.298752	7.54748757	702.298752	4.151561225
717.582188	7.54773612	702.582188	4.150583996
718.806723	7.55217984	703.806723	4.1464407
719.373597	7.55277729	704.373597	4.144675613
720	7.55351483	705	4.142847156
720.598132	7.55396055	705.598132	4.140456026
722.298752	7.55610995	707.298752	4.134075844
722.582188	7.55844222	707.582188	4.13276097
723.806723	7.56178701	708.806723	4.129379744
724.373597	7.56246625	709.373597	4.127280419
725	7.5632811	710	4.124444096

Final State: j’=2		Final State: j’=1	
E(cm⁻¹)	σ (Å²)	E(cm⁻¹)	σ (Å²)
725.598132	7.56398546	710.598132	4.122374383
727.298752	7.56681013	712.298752	4.116995744
727.582188	7.56749127	712.582188	4.116208477
728.806723	7.57157925	713.806723	4.111654631
729.373597	7.57195175	714.373597	4.10886384
730	7.57270536	715	4.106819393
730.598132	7.57349006	715.598132	4.104989437
732.298752	7.57527042	717.298752	4.099235662
732.582188	7.57512131	717.582188	4.098393404
733.806723	7.57713999	718.806723	4.093435397
734.373597	7.57987787	719.373597	4.091672913
735	7.58184838	720	4.089687034
735.598132	7.58220202	720.598132	4.087866569
737.298752	7.58377811	722.298752	4.082443959
737.582188	7.58390046	722.582188	4.080702133
738.806723	7.58274274	723.806723	4.076279995
739.373597	7.58544634	724.373597	4.074479683
740	7.58490654	725	4.072461288
740.598132	7.58604261	725.598132	4.070552691
742.298752	7.58948891	727.298752	4.064786244
742.582188	7.59261365	727.582188	4.063865072
743.806723	7.5937333	728.806723	4.059008458
744.373597	7.59107901	729.373597	4.057323851
745	7.59673428	730	4.055325423
745.598132	7.59862553	730.598132	4.053390063
747.298752	7.59441505	732.298752	4.047999176
747.582188	7.59644989	732.582188	4.047210198
748.806723	7.60540508	733.806723	4.043191245
749.373597	7.60459086	734.373597	4.040601768
750	7.60407035	735	4.038241268
750.598132	7.60039808	735.598132	4.036463361
752.298752	7.60914682	737.298752	4.031171565
752.582188	7.60916694	737.582188	4.030336567
753.806723	7.60907913	738.806723	4.027304416
754.373597	7.6071464	739.373597	4.024692913
755	7.60965647	740	4.023066077
755.598132	7.61353011	740.598132	4.021112542
757.298752	7.61293977	742.298752	4.015214703
757.582188	7.6135625	742.582188	4.01335054
758.806723	7.61218718	743.806723	4.009514713
759.373597	7.61509217	744.373597	4.008781528
760	7.62034842	745	4.005093979
760.598132	7.61649215	745.598132	4.002781613

Final State: j'=2		Final State: j'=1	
E(cm⁻¹)	σ (Å²)	E(cm⁻¹)	σ (Å²)
762.298752	7.61987345	747.298752	3.999108653
762.582188	7.61479797	747.582188	3.997723772
763.806723	7.62029883	748.806723	3.991432517
764.373597	7.62482414	749.373597	3.990155983
765	7.622929	750	3.9885558
765.598132	7.62717237	750.598132	3.988023598
767.298752	7.62537588	752.298752	3.980321431
767.582188	7.62676477	752.582188	3.979514411
768.806723	7.62762313	753.806723	3.976051434
769.373597	7.62905179	754.373597	3.974988807
770	7.62932157	755	3.971949462
770.598132	7.62959623	755.598132	3.969556648
772.298752	7.63067168	757.298752	3.96495157
772.582188	7.62993309	757.582188	3.963928359
773.806723	7.63369794	758.806723	3.960711942
774.373597	7.63397319	759.373597	3.958030047
775	7.63444428	760	3.954737903
775.598132	7.6349482	760.598132	3.954330195
777.298752	7.63644957	762.298752	3.948302205
777.582188	7.63675005	762.582188	3.94918364
778.806723	7.63807154	763.806723	3.943429677
779.373597	7.63859976	764.373597	3.940696235
780	7.63913564	765	3.939647706
780.598132	7.63994352	765.598132	3.936520619
782.298752	7.64152216	767.298752	3.932045364
782.582188	7.64175784	767.582188	3.930930145
783.806723	7.64283329	768.806723	3.926840409
784.373597	7.6433348	769.373597	3.925019081
785	7.6438848	770	3.923137722
785.598132	7.64440479	770.598132	3.921314518
787.298752	7.64585319	772.298752	3.916086319
787.582188	7.64609063	772.582188	3.915445269
788.806723	7.64714867	773.806723	3.910746378
789.373597	7.64765006	774.373597	3.909023021
790	7.64816093	775	3.907054855
790.598132	7.64863286	775.598132	3.90515747
792.298752	7.64995483	777.298752	3.899721134
792.582188	7.65017206	777.582188	3.898798066
793.806723	7.65109848	778.806723	3.894833249
794.373597	7.65152047	779.373597	3.8930205
795	7.65198177	780	3.891019042
795.598132	7.65241753	780.598132	3.888949225
797.298752	7.65363252	782.298752	3.8836296

Final State: j’=2		Final State: j’=1	
E(cm⁻¹)	σ (Å²)	E(cm⁻¹)	σ (Å²)
797.582188	7.65383121	782.582188	3.882728482
798.806723	7.65467449	783.806723	3.878821843
799.373597	7.65505648	784.373597	3.877012971
800	7.65547264	785	3.875015172
800.598132	7.6558644	785.598132	3.873108588
802.298752	7.65694901	787.298752	3.867691996
802.582188	7.65712565	787.582188	3.866789185
803.806723	7.65787545	788.806723	3.862876555
804.373597	7.65821525	789.373597	3.861073074
805	7.65858536	790	3.859090199
805.598132	7.65893351	790.598132	3.857196886
807.298752	7.65989525	792.298752	3.851812225
807.582188	7.66005148	792.582188	3.850915175
808.806723	7.6607131	793.806723	3.847041796
809.373597	7.66101202	794.373597	3.845249945
810	7.66133693	795	3.843270847
810.598132	7.66164189	795.598132	3.841381901
812.298752	7.66248085	797.298752	3.836016379
812.582188	7.66261665	797.582188	3.83512311
813.806723	7.66319014	798.806723	3.831267422
814.373597	7.6634484	799.373597	3.829484312
815	7.66372846	800	3.827515125
815.598132	7.6639907	800.598132	3.825635919
817.298752	7.66470864	802.298752	3.820298565
817.582188	7.66482433	802.582188	3.819409793
818.806723	7.66531113	803.806723	3.815572732
819.373597	7.66552936	804.373597	3.813797884
820	7.66576525	805	3.811837743
820.598132	7.66598538	805.598132	3.809967109
822.298752	7.66658392	807.298752	3.804654242
822.582188	7.66667976	807.582188	3.803769585
823.806723	7.66708099	808.806723	3.799950345
824.373597	7.66725969	809.373597	3.798183784
825	7.66745197	810	3.796232797
825.598132	7.66763053	810.598132	3.79437094
827.298752	7.66811127	812.298752	3.789082876
827.582188	7.66818753	812.582188	3.788202342
828.806723	7.66850432	813.806723	3.784400786
829.373597	7.66864402	814.373597	3.782642363
830	7.66879331	815	3.780700349
830.598132	7.66893086	815.598132	3.778847024
832.298752	7.66929542	817.298752	3.77358313
832.582188	7.66935238	817.582188	3.772706611

Final State: j'=2		Final State: j'=1	
E(cm^{-1})	σ (Å^2)	E(cm^{-1})	σ (Å^2)
833.806723	7.66958592	818.806723	3.768922357
834.373597	7.66968721	819.373597	3.767171938
835	7.66979409	820	3.765238748
835.598132	7.66989124	820.598132	3.763393832
837.298752	7.67014123	822.298752	3.758153783
837.582188	7.67017916	822.582188	3.757281221
838.806723	7.67033066	823.806723	3.753514054
839.373597	7.67039404	824.373597	3.751771526
840	7.67045914	825	3.749847038
840.598132	7.67051643	825.598132	3.748010418
842.298752	7.67065356	827.298752	3.742793876
842.582188	7.67067273	827.582188	3.741925215
843.806723	7.67074337	828.806723	3.738174899
844.373597	7.67076942	829.373597	3.736440165
845	7.67079333	830	3.734524253
845.598132	7.67081139	830.598132	3.732695805
847.298752	7.67083732	832.298752	3.727502465
847.582188	7.67083798	832.582188	3.726637673
848.806723	7.670829	833.806723	3.722903993
849.373597	7.67081829	834.373597	3.721176934
850	7.67080164	835	3.719269521
850.598132	7.67078105	835.598132	3.717449179
852.298752	7.67069746	837.298752	3.712278794
852.582188	7.67067996	837.582188	3.711417822
853.806723	7.67059254	838.806723	3.707700613
854.373597	7.67054561	839.373597	3.705981164
855	7.67048906	840	3.704082149
855.598132	7.6704304	840.598132	3.702269812
857.298752	7.67023906	842.298752	3.697122156
857.582188	7.67020362	842.582188	3.696264973
858.806723	7.67003904	843.806723	3.692564074
859.373597	7.6699565	844.373597	3.69085218
860	7.66986063	845	3.688961495
860.598132	7.66976456	845.598132	3.687157104
862.298752	7.66946716	847.298752	3.68203201
862.582188	7.66941413	847.582188	3.681178569
863.806723	7.6691736	848.806723	3.67749388
864.373597	7.66905602	849.373597	3.675789447
865	7.66892151	850	3.673907038
865.598132	7.66878861	850.598132	3.67211054
867.298752	7.6683869	852.298752	3.667007848
867.582188	7.66831655	852.582188	3.666158129
868.806723	7.66800136	853.806723	3.662489539

Final State: j’=2		Final State: j’=1	
E(cm⁻¹)	σ (Å²)	E(cm⁻¹)	σ (Å²)
869.373597	7.66784932	854.373597	3.660792572
870	7.66767684	855	3.658918387
870.598132	7.66750773	855.598132	3.657129745
872.298752	7.6670035	857.298752	3.652049345
872.582188	7.6669161	857.582188	3.651203336
873.806723	7.66652754	858.806723	3.647550785
874.373597	7.66634164	859.373597	3.645861227
875	7.6661318	860	3.643995226
875.598132	7.66592711	860.598132	3.642214408
877.298752	7.66532213	862.298752	3.637156221
877.582188	7.66521799	862.582188	3.636313931
878.806723	7.66475733	863.806723	3.632677354
879.373597	7.66453818	864.373597	3.630995203
880	7.66429163	865	3.629137364
880.598132	7.664052	865.598132	3.627364347
882.298752	7.66334807	867.298752	3.622328337
882.582188	7.66322751	867.582188	3.621489744
883.806723	7.66269607	868.806723	3.617869108
884.373597	7.662444	869.373597	3.616194331
885	7.66216171	870	3.614344665
885.598132	7.66188778	870.598132	3.612579437
887.298752	7.66108674	872.298752	3.60756559
887.582188	7.66095006	872.582188	3.606730675
888.806723	7.66034919	873.806723	3.60312601
889.373597	7.66006534	874.373597	3.601458619
890	7.65974748	875	3.599617101
890.598132	7.65943992	875.598132	3.597859663
892.298752	7.6585437	877.298752	3.592867974
892.582188	7.65839122	877.582188	3.592036746
893.806723	7.6577223	878.806723	3.588448041
894.373597	7.65740709	879.373597	3.586788057
895	7.65705466	880	3.584954691
895.598132	7.65671414	880.598132	3.583205053
897.298752	7.65572477	882.298752	3.578235543
897.582188	7.65555684	882.582188	3.577408033
898.806723	7.65482139	883.806723	3.573835303
899.373597	7.6544755	884.373597	3.572182495
900	7.65408928	885	3.570357563
900.598132	7.65371661	885.598132	3.568615739
902.298752	7.65263635	887.298752	3.563668461
902.582188	7.65245335	887.582188	3.56284464
903.806723	7.65165309	888.806723	3.55928796
904.373597	7.65127736	889.373597	3.557642792

Final State: j’=2		Final State: j’=1	
E(cm^{-1})	σ (Å^2)	E(cm^{-1})	σ (Å^2)
905	7.65085829	890	3.555825852
905.598132	7.65045438	890.598132	3.554091855
907.298752	7.64928601	892.298752	3.549166878
907.582188	7.64908844	892.582188	3.548346778
908.806723	7.64822561	893.806723	3.544806161
909.373597	7.64782115	894.373597	3.543168443
910	7.64737054	895	3.541359715
910.598132	7.64693672	895.598132	3.539633582
912.298752	7.64568451	897.298752	3.534730962
912.582188	7.64547316	897.582188	3.533914592
913.806723	7.64455168	898.806723	3.530390072
914.373597	7.64412057	899.373597	3.528759807
915	7.64364098	900	3.526959327
915.598132	7.64317999	900.598132	3.525241062
917.298752	7.64185435	902.298752	3.520360802
917.582188	7.6416315	902.582188	3.519548164
918.806723	7.64066377	903.806723	3.51603975
919.373597	7.64021396	904.373597	3.514416926
920	7.6397167	905	3.512624674
920.598132	7.6392433	905.598132	3.510914266
922.298752	7.63793667	907.298752	3.506056291
922.582188	7.63773744	907.582188	3.505247354
923.806723	7.63700128	908.806723	3.501754912
924.373597	7.63610808	909.373597	3.500139471
925	7.63526429	910	3.498355336
925.598132	7.63528771	910.598132	3.496652672
927.298752	7.63345899	912.298752	3.491816547
927.582188	7.63352927	912.582188	3.491011226
928.806723	7.6315465	913.806723	3.487534254
929.373597	7.63136782	914.373597	3.485925863
930	7.63055969	915	3.484149438
930.598132	7.63018238	915.598132	3.482454034
932.298752	7.62831048	917.298752	3.477637757
932.582188	7.62844915	917.582188	3.476835571
933.806723	7.62689808	918.806723	3.473371326
934.373597	7.62654779	919.373597	3.471768193
935	7.62623419	920	3.46999682
935.598132	7.62520024	920.598132	3.468305298
937.298752	7.62303057	922.298752	3.463484992
937.582188	7.62296153	922.582188	3.462676699
938.806723	7.62208184	923.806723	3.459130423
939.373597	7.62165162	924.373597	3.457625675
940	7.6212593	925	3.455979397

Final State: j'=2		Final State: j'=1	
E(cm^{-1})	σ (Å^2)	E(cm^{-1})	σ (Å^2)
940.598132	7.61994252	925.598132	3.454147953
942.298752	7.61808703	927.298752	3.449484525
942.582188	7.61791494	927.582188	3.44859941
943.806723	7.61685318	928.806723	3.445400943
944.373597	7.61633623	929.373597	3.443707659
945	7.61580218	930	3.44203426
945.598132	7.61509528	930.598132	3.440334569
947.298752	7.61274824	932.298752	3.435644731
947.582188	7.6123404	932.582188	3.434750051
948.806723	7.6113998	933.806723	3.43146123
949.373597	7.6108396	934.373597	3.429839329
950	7.61021047	935	3.428034867
950.598132	7.60961366	935.598132	3.426475501
952.298752	7.6074397	937.298752	3.421864604
952.582188	7.60758403	937.582188	3.42104192
953.806723	7.60568221	938.806723	3.417571215
954.373597	7.60524145	939.373597	3.415975062
955	7.60464266	940	3.414193604
955.598132	7.60406277	940.598132	3.412685737
957.298752	7.60277548	942.298752	3.408025744
957.582188	7.60254871	942.582188	3.407214513
958.806723	7.60055333	943.806723	3.403793006
959.373597	7.59950408	944.373597	3.402218181
960	7.598352	945	3.400476851
960.598132	7.59851861	945.598132	3.398888688
962.298752	7.59573186	947.298752	3.394271135
962.582188	7.59567892	947.582188	3.393533827
963.806723	7.593304	948.806723	3.390084081
964.373597	7.59267723	949.373597	3.388520055
965	7.59234888	950	3.386795661
965.598132	7.59210693	950.598132	3.38515111
967.298752	7.58858469	952.298752	3.380524662
967.582188	7.58839215	952.582188	3.379641556
968.806723	7.58768671	953.806723	3.376458441
969.373597	7.58778632	954.373597	3.374866455
970	7.58507276	955	3.373136428
970.598132	7.58442527	955.598132	3.371486963
972.298752	7.58307694	957.298752	3.366684982
972.582188	7.5828236	957.582188	3.365864078
973.806723	7.58034001	958.806723	3.362711567
974.373597	7.57979418	959.373597	3.361203157
975	7.57955661	960	3.359639158
975.598132	7.57878091	960.598132	3.35782646

Final State: j'=2		Final State: j'=1	
E(cm⁻¹)	σ (Å²)	E(cm⁻¹)	σ (Å²)
977.298752	7.57673035	962.298752	3.353435367
977.582188	7.57650572	962.582188	3.352606617
978.806723	7.57346719	963.806723	3.34944023
979.373597	7.57394446	964.373597	3.347975939
980	7.57275539	965	3.3462038
980.598132	7.57145716	965.598132	3.344514711
982.298752	7.5694665	967.298752	3.340186434
982.582188	7.56962172	967.582188	3.339411903
983.806723	7.56725704	968.806723	3.335952291
984.373597	7.56549467	969.373597	3.334260759
985	7.56562215	970	3.332990133
985.598132	7.5654898	970.598132	3.331379001
987.298752	7.56227259	972.298752	3.326671139
987.582188	7.56176811	972.582188	3.325896731
988.806723	7.56017111	973.806723	3.322927101
989.373597	7.55940827	974.373597	3.321210972
990	7.55851867	975	3.319431768
990.598132	7.55761813	975.598132	3.317843533
992.298752	7.55491716	977.298752	3.313267117
992.582188	7.55456463	977.582188	3.312569265
993.806723	7.55284049	978.806723	3.309565099
994.373597	7.55200172	979.373597	3.307813605
995	7.55106686	980	3.306146488
995.598132	7.55016432	980.598132	3.304776689
997.298752	7.54755026	982.298752	3.300288241
997.582188	7.54712126	982.582188	3.299325552
998.806723	7.54525111	983.806723	3.296223914
999.373597	7.54429003	984.373597	3.294936554
1000	7.54339331	985	3.293115159
1000.59813	7.54250414	985.598132	3.291406132
1002.29875	7.53987808	987.298752	3.287163055
1002.58219	7.53943596	987.582188	3.28640765
1003.80672	7.53751981	988.806723	3.283154002
1004.3736	7.53662904	989.373597	3.281672844
1005	7.53564172	990	3.280048332
1005.59813	7.53469477	990.598132	3.278520764
1007.29875	7.53198378	992.298752	3.274096573
1007.58219	7.5315296	992.582188	3.273336292
1008.80672	7.5295782	993.806723	3.270160226
1009.3736	7.52866915	994.373597	3.268695077
1010	7.52766032	995	3.267077937
1010.59813	7.52669366	995.598132	3.265536717
1012.29875	7.52392963	997.298752	3.261157487

**Final State: j’=2**		**Final State: j’=1**	
E(cm⁻¹)	σ (Å²)	E(cm⁻¹)	σ (Å²)
1012.58219	7.52346678	997.582188	3.260426434
1013.80672	7.52146012	998.806723	3.257280141
1014.3736	7.52052731	999.373597	3.255857657
1015	7.51949376	1000	3.254188631
1015.59813	7.51850426	1000.59813	3.252659248
1017.29875	7.51567779	1002.29875	3.248304953
1017.58219	7.51520477	1002.58219	3.247579871
1018.80672	7.51315438	1003.80672	3.244449951
1019.3736	7.51220145	1004.3736	3.243002734
1020	7.5111457	1005	3.241404921
1020.59813	7.5101349	1005.59813	3.239879946
1022.29875	7.50724671	1007.29875	3.235554829
1022.58219	7.50676332	1007.58219	3.234833611
1023.80672	7.50466821	1008.80672	3.231719024
1024.3736	7.5036947	1009.3736	3.230281362
1025	7.50261629	1010	3.228694318
1025.59813	7.50158393	1010.59813	3.227180205
1027.29875	7.49863492	1012.29875	3.222882222
1028.80672	7.49600286	1012.58219	3.222166905
1029.3736	7.49500929	1013.80672	3.219079834
1030	7.49390874	1014.3736	3.217652541
1030.59813	7.49285527	1015	3.216076684
1032.29875	7.48984634	1015.59813	3.21457318
1033.80672	7.48716137	1017.29875	3.210305136
1034.3736	7.48614795	1017.58219	3.209594822
1035	7.48502551	1018.80672	3.206529553
1035.59813	7.48395118	1019.3736	3.205112469
1037.29875	7.48088316	1020	3.203547959
1038.80672	7.47814604	1020.59813	3.202055442
1039.3736	7.47711308	1022.29875	3.197819141
1040	7.47596908	1022.58219	3.197114135
1040.59813	7.47487422	1023.80672	3.194071723
1042.29875	7.47174795	1024.3736	3.192665194
1043.80672	7.46895943	1025	3.191112342
1044.3736	7.46790719	1025.59813	3.189630937
1045	7.46674194	1027.29875	3.185426307
1045.59813	7.46562682	1028.80672	3.181707077
1047.29875	7.46244313	1029.3736	3.180311186
1048.80672	7.45960392	1030	3.178770117
1049.3736	7.45853268	1030.59813	3.177299988
1050	7.45734647	1032.29875	3.173127526
1050.59813	7.45621137	1033.80672	3.169436958
1052.29875	7.45297108	1034.3736	3.16805185

Final State: j'=2		Final State: j'=1	
E(cm⁻¹)	σ (Å²)	E(cm⁻¹)	σ (Å²)
1053.80672	7.45008192	1035	3.166522744
1054.3736	7.44899196	1035.59813	3.165064038
1055	7.4477851	1037.29875	3.160924141
1055.59813	7.44663028	1038.80672	3.15726256
1057.29875	7.44333421	1039.3736	3.155888381
1058.80672	7.44039578	1040	3.154371363
1059.3736	7.43928736	1040.59813	3.152924217
1060	7.43806013	1042.29875	3.148817297
1060.59813	7.43688591	1043.80672	3.145185052
1062.29875	7.43353481	1044.3736	3.143821947
1063.80672	7.43054784	1045	3.142317152
1064.3736	7.42942121	1045.59813	3.14088171
1065	7.42817391	1047.29875	3.136808122
1065.59813	7.42698058	1048.80672	3.133205572
1067.29875	7.42357526	1049.3736	3.131853635
1068.80672	7.42054042	1050	3.130361219
1069.3736	7.41939587	1050.59813	3.128937597
1070	7.41812877	1052.29875	3.124897751
1070.59813	7.41691657	1053.80672	3.121325224
1072.29875	7.41345781	1054.3736	3.119984598
1073.80672	7.41037582	1055	3.118504677
1074.3736	7.4092136	1055.59813	3.11709302
1075	7.40792701	1057.29875	3.113087277
1075.59813	7.40669623	1058.80672	3.109545057
1077.29875	7.40318481	1059.3736	3.108215856
1078.80672	7.40005635	1060	3.106748584
1079.3736	7.39887672	1060.59813	3.105349024
1080	7.39757091	1062.29875	3.101377694
1080.59813	7.39632184	1063.80672	3.097866125
1083.80672	7.38958429	1064.3736	3.096548462
1084.3736	7.3883875	1065	3.095093943
1085	7.38706278	1065.59813	3.093706594
1085.59813	7.38579567	1067.29875	3.089770065
1088.80672	7.37896196	1068.80672	3.086289398
1089.3736	7.37774553	1069.3736	3.084983386
1090	7.37640493	1070	3.083541754
1090.59813	7.37512005	1070.59813	3.082166719
1093.80672	7.36819166	1072.29875	3.07826526
1094.3736	7.36696136	1073.80672	3.074815816
1095	7.36559966	1074.3736	3.073521543
1095.59813	7.36429731	1075	3.07209292
1098.80672	7.35727575	1075.59813	3.07073031
1099.3736	7.35602908	1077.29875	3.066864246

Final State: j’=2		Final State: j’=1	
E(cm⁻¹)	σ (Å²)	E(cm⁻¹)	σ (Å²)
1100.59813	7.35332979	1078.80672	3.063446261
1103.80672	7.34621655	1079.3736	3.062163841
1104.3736	7.34495379	1080	3.06074832
1105.59813	7.34221986	1080.59813	3.059398238
1108.80672	7.3350165	1083.80672	3.052181563
1109.3736	7.33373793	1084.3736	3.050911085
1110.59813	7.33096993	1085	3.049508766
1113.80672	7.32367803	1085.59813	3.048171293
1114.3736	7.32238391	1088.80672	3.041022505
1115.59813	7.31958247	1089.3736	3.039769834
1118.80672	7.3122036	1090	3.038375023
1119.3736	7.31089422	1090.59813	3.037050257
1120.59813	7.30805994	1093.80672	3.02996981
1123.80672	7.30059576	1094.3736	3.028723467
1124.3736	7.29927141	1095	3.027347813
1125.59813	7.29640491	1095.59813	3.026035844
1128.80672	7.28885704	1098.80672	3.019024134
1129.3736	7.28751802	1099.3736	3.017789958
1130.59813	7.28461993	1100.59813	3.015128663
1133.80672	7.27699008	1103.80672	3.008186074
1134.3736	7.2756367	1104.3736	3.006964133
1135.59813	7.27270767	1105.59813	3.0043293
1138.80672	7.26499753	1108.80672	2.997456176
1139.3736	7.26363006	1109.3736	2.99624652
1140.59813	7.26067077	1110.59813	2.993638254
1143.80672	7.25288205	1113.80672	2.986834876
1144.3736	7.25150082	1114.3736	2.985637566
1145.59813	7.24851189	1115.59813	2.983055967
1148.80672	7.24064626	1118.80672	2.97632258
1149.3736	7.23925156	1119.3736	2.975137654
1150.59813	7.2362336	1120.59813	2.972582808
1153.80672	7.2282926	1123.80672	2.965919619
1154.3736	7.22688466	1124.3736	2.96474709
1155.59813	7.2238382	1125.59813	2.962219076
1158.80672	7.21582283	1128.80672	2.955626223
1159.3736	7.21440178	1129.3736	2.954466138
1160.59813	7.21132694	1130.59813	2.951964986
1163.80672	7.20323605	1133.80672	2.945442572
1165.59813	7.19869573	1134.3736	2.944294942
1168.80672	7.19049811	1135.59813	2.941820685
1170.59813	7.18598363	1138.80672	2.935368781
1173.80672	7.1776755	1139.3736	2.934233606
1175.59813	7.1731975	1140.59813	2.931786268

Final State: j’=2		Final State: j’=1	
E(cm^{-1})	σ (Å^2)	E(cm^{-1})	σ (Å^2)
1178.80672	7.16488844	1143.80672	2.92540488
1180.59813	7.16029596	1144.3736	2.924282165
1183.80672	7.15203562	1145.59813	2.921861736
1185.59813	7.14727193	1148.80672	2.915550848
1188.80672	7.13893511	1149.3736	2.914440578
1190.59813	7.13417858	1150.59813	2.912047042
1193.80672	7.12580593	1153.80672	2.905806603
1195.59813	7.12098091	1154.3736	2.904708778
1198.80672	7.11254375	1155.59813	2.902342124
1200.59813	7.1076674	1158.80672	2.896172109
1203.80672	7.09893347	1159.3736	2.895086729
1205.59813	7.09442873	1160.59813	2.892746969
1208.80672	7.08568282	1163.80672	2.886647678
1210.59813	7.08105229	1165.59813	2.883262398
1213.80672	7.07246436	1168.80672	2.87723911
1215.59813	7.06728243	1170.59813	2.873909529
1218.80672	7.05870075	1173.80672	2.867942053
1220.59813	7.05383698	1175.59813	2.864622921
1223.80672	7.0450785	1178.80672	2.85873354
1225.59813	7.04009989	1180.59813	2.855437791
1228.80672	7.03139923	1183.80672	2.849603292
1230.59813	7.02675883	1185.59813	2.846379453
1233.80672	7.01777023	1188.80672	2.84061905
1235.59813	7.01282981	1190.59813	2.83742838
1238.80672	7.00398733	1193.80672	2.831731504
1240.59813	6.99905195	1195.59813	2.828586251
1243.80672	6.99019505	1198.80672	2.822962474
1245.59813	6.98525155	1200.59813	2.819851838
1248.80672	6.97633444	1203.80672	2.814351221
1250.59813	6.97136243	1205.59813	2.811243798
1253.80672	6.96244457	1208.80672	2.805730082
1255.59813	6.95745641	1210.59813	2.802661394
1258.80672	6.94851852	1213.80672	2.797231978
1260.59813	6.94352304	1215.59813	2.794252178
1263.80672	6.93456654	1218.80672	2.788876803
1265.59813	6.929562	1220.59813	2.785936725
1268.80672	6.92059198	1223.80672	2.780600706
1270.59813	6.91558059	1225.59813	2.777692175
1275.59813	6.9015828	1228.80672	2.772450312
1280.59813	6.88757362	1230.59813	2.769543743
1285.59813	6.87355773	1233.80672	2.764378957
1290.59813	6.85953984	1235.59813	2.761510211
1295.59813	6.84552466	1238.80672	2.756409877

Final State: j'=2		Final State: j'=1	
E(cm^{-1})	σ (Å^2)	E(cm^{-1})	σ (Å^2)
1300.59813	6.83151687	1240.59813	2.753567048
1305.59813	6.81752106	1243.80672	2.748516812
1310.59813	6.80354186	1245.59813	2.745710925
1315.59813	6.78958378	1248.80672	2.740730611
1320.59813	6.77565129	1250.59813	2.737962531
1325.59813	6.76174882	1253.80672	2.733034827
1330.59813	6.74788072	1255.59813	2.730300575
1335.59813	6.73405124	1258.80672	2.725430576
1340.59813	6.72026458	1260.59813	2.722727826
1345.59813	6.70652487	1263.80672	2.717915763
1350.59813	6.69283607	1265.59813	2.715244843
1355.59813	6.67920216	1268.80672	2.710489032
1360.59813	6.66562693	1270.59813	2.707849382
1365.59813	6.65211414	1275.59813	2.700540296
1370.59813	6.63866739	1280.59813	2.69331616
1375.59813	6.62529021	1285.59813	2.686175611
1380.59813	6.61198601	1290.59813	2.679117292
1385.59813	6.59875808	1295.59813	2.672139884
1390.59813	6.58560963	1300.59813	2.665242066
1395.59813	6.57254368	1305.59813	2.658422571
1400.59813	6.55956339	1310.59813	2.651680156
1405.59813	6.54667145	1315.59813	2.645013592
		1320.59813	2.638421726
		1325.59813	2.631903418
		1330.59813	2.625457605
		1335.59813	2.619083271
		1340.59813	2.612779428
		1345.59813	2.606545177
		1350.59813	2.600379652
		1355.59813	2.594282059
		1360.59813	2.588251652
		1365.59813	2.582287794
		1370.59813	2.576389866
		1375.59813	2.570557292
		1380.59813	2.564789637
		1385.59813	2.559086445
		1390.59813	2.553447379
		1395.59813	2.547872159
		1400.59813	2.542360468
		1405.59813	2.536912201

Inelastic collisions' Cross-Section: Pseudo-Singlet State
Initial rotational state: j=5

**Final State: j'=4**		**Final State: j'=3**		**Final State: j'=2**	
E(cm⁻¹)	σ (Å²)	E(cm⁻¹)	σ (Å²)	E(cm⁻¹)	σ (Å²)
410.374226	20603.5608	410.374226	4891.76638	410.374226	1068.25197
410.374227	14580.7683	410.374227	3461.93531	410.374227	755.999771
410.374228	11915.5538	410.374228	2829.2275	410.374228	617.824766
410.374229	10328.494	410.374229	2452.48132	410.374229	535.547366
410.37423	9246.59327	410.37423	2195.66214	410.37423	479.46006
410.374231	8448.81828	410.374231	2006.29482	410.374231	438.103202
410.374232	7829.44668	410.374232	1859.28025	410.374232	405.995567
410.374233	7330.71472	410.374233	1740.90504	410.374233	380.142378
410.374234	6918.04471	410.374234	1642.96035	410.374234	358.750954
410.374235	6569.30727	410.374235	1560.19263	410.374235	340.674002
410.374236	6269.59516	410.374236	1489.06293	410.374236	325.138663
410.374237	6008.45133	410.374237	1427.08876	410.374237	311.602799
410.374238	5778.29502	410.374238	1372.47046	410.374238	299.673392
410.374239	5573.47796	410.374239	1323.86719	410.374239	289.057615
410.37424	5389.69496	410.37424	1280.25691	410.37424	279.532271
410.374241	5223.59918	410.374241	1240.84512	410.374241	270.923848
410.374242	5072.54358	410.374242	1205.00343	410.374242	263.095116
410.374243	4934.40438	410.374243	1172.22768	410.374243	255.935972
410.374244	4807.4547	410.374244	1142.10795	410.374244	249.356889
410.374245	4690.27425	410.374245	1114.30706	410.374245	243.284238
410.374246	4581.68244	410.374246	1088.54478	410.374246	237.656811
410.374247	4480.68939	410.374247	1064.58612	410.374247	232.423295
410.374248	4386.45721	410.374248	1042.23219	410.374248	227.540249
410.374249	4298.27221	410.374249	1021.31357	410.374249	222.970676
410.37425	4215.52134	410.37425	1001.68473	410.37425	218.682794
410.374251	4137.67496	410.374251	983.219959	410.374251	214.649145
410.374252	4064.27368	410.374252	965.810205	410.374252	210.845917
410.374253	3994.91531	410.374253	949.359998	410.374253	207.252257
410.374254	3929.24701	410.374254	933.785588	410.374254	203.849876
410.374255	3866.95742	410.374255	919.013065	410.374255	200.62263
410.374256	3807.77083	410.374256	904.976987	410.374256	197.55623
410.374257	3751.44179	410.374257	891.619093	410.374257	194.63795
410.374258	3697.75117	410.374258	878.887368	410.374258	191.856431
410.374259	3646.50301	410.374259	866.735301	410.374259	189.201514
410.37426	3597.52082	410.37426	855.120998	410.37426	186.66405
410.374261	3550.64508	410.374261	844.006595	410.374261	184.23577
410.374262	3505.73188	410.374262	833.357936	410.374262	181.909214
410.374263	3462.65065	410.374263	823.144033	410.374263	179.677614
410.374264	3421.2827	410.374264	813.336708	410.374264	177.534816
410.374265	3381.51965	410.374265	803.910239	410.374265	175.475203
410.374266	3343.2626	410.374266	794.841151	410.374266	173.493647
410.374267	3306.42084	410.374267	786.107919	410.374267	171.585447
410.374268	3270.91128	410.374268	777.690818	410.374268	169.746296

Final State: j'=4		Final State: j'=3		Final State: j'=2	
E(cm⁻¹)	σ (Å²)	E(cm⁻¹)	σ (Å²)	E(cm⁻¹)	σ (Å²)
410.374269	3236.65723	410.374269	769.571649	410.374269	167.972218
410.37427	3203.58785	410.37427	761.733594	410.37427	166.259541
410.374271	3171.63874	410.374271	754.161372	410.374271	164.604927
410.374272	3140.74921	410.374272	746.840575	410.374272	163.00523
410.374273	3110.8635	410.374273	739.757975	410.374273	161.45756
410.374274	3081.92979	410.374274	732.901268	410.374274	159.95923
410.374275	3053.9001	410.374275	726.259067	410.374275	158.507753
410.374276	3026.7295	410.374276	719.820714	410.374276	157.100801
410.374277	3000.37618	410.374277	713.576279	410.374277	155.736205
410.374278	2974.80115	410.374278	707.516513	410.374278	154.411946
410.374279	2949.96808	410.374279	701.632792	410.374279	153.12614
410.37428	2925.84265	410.37428	695.916972	410.37428	151.877008
410.374281	2902.39352	410.374281	690.361615	410.374281	150.662926
410.374282	2879.58977	410.374282	684.959383	410.374282	149.482291
410.374283	2857.4038	410.374283	679.703722	410.374283	148.333671
410.374284	2835.80906	410.374284	674.588342	410.374284	147.215693
410.374285	2814.78052	410.374285	669.607292	410.374285	146.127057
410.374286	2794.29481	410.374286	664.75503	410.374286	145.066552
410.374287	2774.32971	410.374287	660.026282	410.374287	144.033027
410.374288	2754.8644	410.374288	655.416108	410.374288	143.025402
410.374289	2735.87913	410.374289	650.919822	410.374289	142.042655
410.37429	2717.35508	410.37429	646.532956	410.37429	141.083809
410.374291	2699.27475	410.374291	642.251358	410.374291	140.147957
410.374292	2681.62132	410.374292	638.071035	410.374292	139.234228
410.374293	2664.3786	410.374293	633.988148	410.374293	138.341782
410.374294	2647.53199	410.374294	629.999231	410.374294	137.469863
410.374295	2631.06716	410.374295	626.100881	410.374295	136.617727
410.374296	2614.97016	410.374296	622.289792	410.374296	135.784653
410.374297	2599.22833	410.374297	618.562955	410.374297	134.969983
410.374298	2583.82925	410.374298	614.917428	410.374298	134.173074
410.374299	2568.76133	410.374299	611.350456	410.374299	133.393326
410.3743	2554.01316	410.3743	607.859336	410.3743	132.630147
410.374301	2539.57417	410.374301	604.441556	410.374301	131.882988
410.374302	2525.43438	410.374302	601.09475	410.374302	131.151334
410.374303	2511.58392	410.374303	597.816574	410.374303	130.434671
410.374304	2498.01348	410.374304	594.604822	410.374304	129.732519
410.374305	2484.71429	410.374305	591.45741	410.374305	129.044421
410.374306	2471.67781	410.374306	588.372313	410.374306	128.369937
410.374307	2458.89604	410.374307	585.34763	410.374307	127.70865
410.374308	2446.36101	410.374308	582.381476	410.374308	127.060148
410.374309	2434.06546	410.374309	579.472123	410.374309	126.424055
410.37431	2422.00219	410.37431	576.617864	410.37431	125.799997
410.374311	2410.16422	410.374311	573.817043	410.374311	125.187613
410.374312	2398.54528	410.374312	571.068176	410.374312	124.586579
410.374313	2387.13888	410.374313	568.369715	410.374313	123.996555

Final State: j'=4		Final State: j'=3		Final State: j'=2	
E(cm^{-1})	σ (Å^2)	E(cm^{-1})	σ (Å^2)	E(cm^{-1})	σ (Å^2)
410.374314	2375.93893	410.374314	565.720221	410.374314	123.41723
410.374315	2364.93966	410.374315	563.118325	410.374315	122.848302
410.374316	2354.13564	410.374316	560.562737	410.374316	122.28949
410.374317	2343.52116	410.374317	558.052101	410.374317	121.740499
410.374318	2333.09141	410.374318	555.585279	410.374318	121.201079
410.374319	2322.84119	410.374319	553.161032	410.374319	120.670961
410.37432	2312.76571	410.37432	550.778231	410.37432	120.149896
410.374321	2302.86056	410.374321	548.435823	410.374321	119.637656
410.374322	2293.121	410.374322	546.132687	410.374322	119.133995
410.374323	2283.5429	410.374323	543.86784	410.374323	118.638699
410.374324	2274.12219	410.374324	541.640321	410.374324	118.151558
410.374325	2264.85471	410.374325	539.449141	410.374325	117.672356
410.374825	1408.5924	410.374825	339.114271	410.374825	73.6960452
410.375225	1450.56859	410.375225	350.901427	410.375225	76.1273017
410.375325	1477.30996	410.375325	357.686615	410.375325	77.5743149
410.375825	1660.67184	410.375825	403.3393	410.375825	87.3710775
410.376225	1846.83413	410.376225	449.15477	410.376225	97.2376203
410.376325	1897.25783	410.376325	461.515124	410.376325	99.9022576
410.376825	2166.33236	410.376825	527.197624	410.376825	114.075367
410.377225	2394.7868	410.377225	582.618842	410.377225	126.048735
410.377325	2452.68883	410.377325	596.612206	410.377325	129.073879
410.377825	2739.96376	410.377825	665.668357	410.377825	144.015477
410.378225	2958.0401	410.378225	717.573275	410.378225	155.262962
410.378325	3009.63294	410.378325	729.76707	410.378325	157.908012
410.378825	3242.50529	410.378825	784.221996	410.378825	169.738616
410.379225	3390.73483	410.379225	818.096865	410.379225	177.122616
410.379325	3421.58727	410.379325	825.011244	410.379325	178.634071
410.379825	3535.13484	410.379825	849.544009	410.379825	184.026177
410.380225	3575.52038	410.380225	856.928009	410.380225	185.692855
410.380325	3578.67216	410.380325	857.116708	410.380325	185.750516
410.380825	3555.2521	410.380825	848.88021	410.380825	184.043231
410.381225	3494.32334	410.381225	832.586077	410.381225	180.562987
410.381325	3474.04318	410.381325	827.374663	410.381325	179.444229
410.381825	3347.91485	410.381825	795.859612	410.381825	172.653859
410.382225	3224.08067	410.382225	765.696081	410.382225	166.132248
410.382325	3190.8375	410.382325	757.676039	410.382325	164.396016
410.382825	3015.77977	410.382825	715.795335	410.382825	155.318446
410.383225	2870.06179	410.383225	681.245026	410.383225	147.820525
410.383325	2833.42952	410.383325	672.58954	410.383325	145.941207
410.383825	2651.76776	410.383825	629.789908	410.383825	136.64466
410.384225	2510.64503	410.384225	596.634275	410.384225	129.440062
410.384325	2476.20427	410.384325	588.549954	410.384325	127.683102
410.384825	2310.04007	410.384825	549.565617	410.384825	119.210352
410.385225	2185.08035	410.385225	520.25101	410.385225	112.839103
410.385325	2155.00754	410.385325	513.194742	410.385325	111.305485

Final State: j'=4		Final State: j'=3		Final State: j'=2	
E(cm⁻¹)	σ (Å²)	E(cm⁻¹)	σ (Å²)	E(cm⁻¹)	σ (Å²)
410.385825	2011.74993	410.385825	479.563531	410.385825	103.996914
410.386225	1905.59761	410.386225	454.618583	410.386225	98.5766833
410.386325	1880.20788	410.386325	448.648185	410.386325	97.2795785
410.386825	1759.89468	410.386825	420.333907	410.386825	91.1285451
410.387225	1671.25188	410.387225	399.44528	410.387225	86.5916045
410.387325	1650.0945	410.387325	394.455851	410.387325	85.5080042
410.387825	1549.98547	410.387825	370.826341	410.387825	80.3769023
410.388225	1476.30269	410.388225	353.411328	410.388225	76.596021
410.388325	1458.71609	410.388325	349.251691	410.388325	75.6929916
410.388825	1375.45982	410.388825	329.542998	410.388825	71.4150689
410.389225	1314.09623	410.389225	314.999072	410.389225	68.2587099
410.389325	1299.43438	410.389325	311.521854	410.389325	67.5040931
410.389825	1229.91904	410.389825	295.022516	410.389825	63.9240726
410.390225	1178.55131	410.390225	282.817299	410.390225	61.2762188
410.390325	1166.25846	410.390325	279.894779	410.390325	60.6422377
410.390825	1107.85977	410.390825	266.001867	410.390825	57.6286403
410.391225	1064.57214	410.391225	255.693918	410.391225	55.3929966
410.391325	1054.19444	410.391325	253.221391	410.391325	54.856766
410.391825	1004.78852	410.391825	241.443953	410.391825	52.3028096
410.392225	968.045285	410.392225	232.677776	410.392225	50.4020226
410.392325	959.220232	410.392325	230.571407	410.392325	49.9453137
410.392825	917.116165	410.392825	220.516851	410.392825	47.7654119
410.393225	885.701622	410.393225	213.009541	410.393225	46.1379425
410.393325	878.142699	410.393325	211.202521	410.393325	45.7461438
410.393825	842.003913	410.393825	202.5592	410.393825	43.8726264
410.394225	814.955711	410.394225	196.085998	410.394225	42.4695784
410.395225	753.761534	410.395225	181.427816	410.395225	39.2927125
410.396225	700.492274	410.396225	168.652899	410.396225	36.524412
410.397225	653.847523	410.397225	157.454829	410.397225	34.0980473
410.398225	612.7809	410.398225	147.586531	410.398225	31.960005
410.399225	576.444531	410.399225	138.847233	410.399225	30.0667387
410.400225	544.146056	410.400225	131.072862	410.400225	28.3825782
410.401225	515.316484	410.401225	124.128185	410.401225	26.8782229
410.402225	489.485053	410.402225	117.901214	410.402225	25.5293798
410.403225	466.259264	410.403225	112.298473	410.403225	24.3157916
410.404225	445.310033	410.404225	107.241426	410.404225	23.2203862
410.405225	426.359654	410.405225	102.663754	410.405225	22.2288801
410.406225	409.172256	410.406225	98.5091797	410.406225	21.3289934
410.407225	393.546621	410.407225	94.7295148	410.407225	20.5102855
410.408225	379.309977	410.408225	91.2834269	410.408225	19.7638084
410.409225	366.313358	410.409225	88.1350812	410.409225	19.0818963
410.410225	354.427318	410.410225	85.2535666	410.410225	18.457742
410.411225	343.538878	410.411225	82.6118059	410.411225	17.8854676
410.412225	333.549462	410.412225	80.1861765	410.412225	17.3599331
410.413225	324.372332	410.413225	77.9557345	410.413225	16.8767545

Final State: j’=4		Final State: j’=3		Final State: j’=2	
E(cm⁻¹)	σ (Å²)	E(cm⁻¹)	σ (Å²)	E(cm⁻¹)	σ (Å²)
410.414225	315.930671	410.414225	75.9020004	410.414225	16.4318579
410.415225	308.156056	410.415225	74.0087497	410.415225	16.0216208
410.416225	300.98817	410.416225	72.2612442	410.416225	15.6430286
410.417225	294.372355	410.417225	70.6465596	410.417225	15.2930907
410.418225	288.260206	410.418225	69.1527914	410.418225	14.9694518
410.419225	282.607258	410.419225	67.7695318	410.419225	14.6696603
410.420225	277.374008	410.420225	66.4871726	410.420225	14.391733
410.421225	272.524478	410.421225	65.2970093	410.421225	14.1337894
410.422225	268.025581	410.422225	64.1911871	410.422225	13.8940817
410.423225	263.84732	410.423225	63.1624881	410.423225	13.6710498
410.424225	259.962469	410.424225	62.204249	410.424225	13.4633562
410.425225	256.345613	410.425225	61.3105241	410.425225	13.2695887
410.426225	252.973873	410.426225	60.4757757	410.426225	13.0885957
410.427225	249.825752	410.427225	59.6948565	410.427225	12.9192395
410.428225	246.881728	410.428225	58.9630478	410.428225	12.7605543
410.429225	244.123899	410.429225	58.2760887	410.429225	12.6115748
410.430225	241.535013	410.430225	57.6298844	410.430225	12.4714128
410.431225	239.100097	410.431225	57.0207895	410.431225	12.3393157
410.432225	236.804773	410.432225	56.4453858	410.432225	12.2144973
410.433225	234.635678	410.433225	55.9005185	410.433225	12.0963451
410.434225	232.580733	410.434225	55.3832744	410.434225	11.9841253
410.435225	230.628622	410.435225	54.8909563	410.435225	11.8773546
410.436225	228.769054	410.436225	54.4211642	410.436225	11.7754465
410.437225	226.992465	410.437225	53.9715157	410.437225	11.6779201
410.438225	225.290158	410.438225	53.5400271	410.438225	11.5843444
410.439225	223.653938	410.439225	53.1246932	410.439225	11.4942517
410.440225	222.076654	410.440225	52.7238424	410.440225	11.4073153
410.441225	220.551421	410.441225	52.3358121	410.441225	11.3231698
410.442225	219.072185	410.442225	51.9591834	410.442225	11.2414886
410.443225	217.633387	410.443225	51.5926262	410.443225	11.1620186
410.444225	216.230044	410.444225	51.2349832	410.444225	11.0844604
410.445225	214.857923	410.445225	50.8851783	410.445225	11.0086479
410.446225	213.512558	410.446225	50.5421392	410.446225	10.9343107
410.447225	212.190733	410.447225	50.205201	410.447225	10.8612696
410.448225	210.889186	410.448225	49.8735072	410.448225	10.7893908
410.449225	209.604965	410.449225	49.5463982	410.449225	10.7185004
410.450225	208.335884	410.450225	49.223249	410.450225	10.6484993
410.451225	207.079905	410.451225	48.9036768	410.451225	10.5792942
410.452225	205.834864	410.452225	48.5870834	410.452225	10.5107295
410.453225	204.599849	410.453225	48.2732304	410.453225	10.4427866
410.454225	203.373029	410.454225	47.9617764	410.454225	10.375326
410.455225	202.153756	410.455225	47.6524213	410.455225	10.3083559
410.456225	200.941015	410.456225	47.3449909	410.456225	10.2418259
410.457225	199.734503	410.457225	47.0393934	410.457225	10.1756783
410.458225	198.533329	410.458225	46.7354836	410.458225	10.1098813

Final State: j’=4		Final State: j’=3		Final State: j’=2	
E(cm⁻¹)	σ (Å²)	E(cm⁻¹)	σ (Å²)	E(cm⁻¹)	σ (Å²)
410.459225	197.337805	410.459225	46.4330894	410.459225	10.0444744
410.460225	196.147264	410.460225	46.1323001	410.460225	9.97936752
410.461225	194.961921	410.461225	45.8329496	410.461225	9.91461668
410.462225	193.782041	410.462225	45.5351879	410.462225	9.8501959
410.463225	192.607512	410.463225	45.2388775	410.463225	9.7861062
410.464225	191.438798	410.464225	44.9442963	410.464225	9.72241732
410.465225	190.276372	410.465225	44.6512355	410.465225	9.65904824
410.466225	189.120338	410.466225	44.3599891	410.466225	9.59608794
410.467225	187.971529	410.467225	44.0706096	410.467225	9.53350796
410.468225	186.830037	410.468225	43.7832206	410.468225	9.47137208
410.469225	185.696746	410.469225	43.4977883	410.469225	9.40969634
410.470225	184.572104	410.470225	43.2145544	410.470225	9.34846497
410.471225	183.456927	410.471225	42.9335463	410.471225	9.28776078
410.472225	182.351474	410.472225	42.6550316	410.472225	9.22756723
410.473225	181.256484	410.473225	42.3791156	410.473225	9.16792057
410.474225	180.172923	410.474225	42.1058501	410.474225	9.10887933
410.574225	586.098577	410.574225	120.700578	410.574225	26.2351654
410.674225	150.073628	410.674225	35.2444042	410.674225	7.61126191
410.774225	103.166058	410.774225	24.0257605	410.774225	5.19109311
410.874225	84.6497415	410.874225	19.5299243	410.874225	4.2198918
410.974225	78.6143254	410.974225	17.8892083	410.974225	3.86350489
411.074225	80.1417258	411.074225	17.9224763	411.074225	3.86668603
411.174225	79.1261388	411.174225	17.3741817	411.174225	3.74576199
411.274225	77.6758249	411.274225	16.3302389	411.274225	3.52304664
411.358282	108.553007	411.358282	20.8544561	411.358282	4.56198417
411.374225	114.201701	411.374225	22.3717167	411.374225	4.84071922
411.474225	74.0842381	411.474225	16.8539434	411.474225	3.58213177
411.574225	61.2736819	411.574225	14.0812921	411.574225	3.01590163
411.582817	60.6895819	411.582817	13.9428795	411.582817	2.98736516
411.674225	56.0373289	411.674225	12.8120461	411.674225	2.75150928
411.774225	52.6428679	411.774225	11.968622	411.774225	2.57305941
411.874225	50.0259139	411.874225	11.3171881	411.874225	2.43439199
411.974225	47.812213	411.974225	10.7689945	411.974225	2.31737631
412	47.2851175	412	10.6391034	412	2.28963434
412.074225	45.8440815	412.074225	10.2848304	412.074225	2.2139428
412.074845	45.8325114	412.074845	10.2820258	412.074845	2.21333397
412.14969	44.4787266	412.14969	9.95016397	412.14969	2.14241319
412.174225	44.054171	412.174225	9.8461139	412.174225	2.12018836
412.274225	42.4146506	412.274225	9.44351186	412.274225	2.03417941
412.358282	41.1481628	412.358282	9.12933433	412.358282	1.96713968
412.374225	40.9199067	412.374225	9.07207332	412.374225	1.95492977
412.474225	39.5861494	412.474225	8.73054616	412.474225	1.88219402
412.574225	38.4657363	412.574225	8.42100604	412.574225	1.81643566
412.674225	37.7156424	412.674225	8.15524075	412.674225	1.76007983
412.774225	37.9834574	412.774225	8.00236649	412.774225	1.72538158

Final State: j'=4		Final State: j'=3		Final State: j'=2	
E(cm⁻¹)	σ (Å²)	E(cm⁻¹)	σ (Å²)	E(cm⁻¹)	σ (Å²)
412.874225	42.4813246	412.874225	8.61468538	412.874225	1.84180466
412.974225	54.4084531	412.974225	10.6197894	412.974225	2.26021796
413.074225	59.4390487	413.074225	11.5811669	413.074225	2.47228914
413.174225	44.2957199	413.174225	9.45002042	413.174225	2.00882315
413.274225	43.5630306	413.274225	9.37543627	413.274225	1.99941907
413.358282	44.8631419	413.358282	9.59936727	413.358282	2.0516313
413.374225	45.06073	413.374225	9.63150546	413.374225	2.05915918
413.474225	45.2627489	413.474225	9.64081244	413.474225	2.06382712
413.574225	43.7756965	413.574225	9.33563	413.574225	1.99940165
413.582817	43.5981837	413.582817	9.29996021	413.582817	1.9918133
413.674225	41.5206543	413.674225	8.88310259	413.674225	1.90289206
413.774225	39.2332283	413.774225	8.42144836	413.774225	1.80439954
413.874225	37.1970634	413.874225	8.00634237	413.874225	1.71590524
413.974225	35.4600494	413.974225	7.64835038	413.974225	1.63962158
414	35.0576788	414	7.56479641	414	1.62180806
414.074225	33.9920057	414.074225	7.34234966	414.074225	1.57440806
414.074845	33.9836921	414.074845	7.3406193	414.074845	1.57404796
414.14969	33.0353965	414.14969	7.14099622	414.14969	1.53152639
414.174225	32.7489635	414.174225	7.08035708	414.174225	1.51861868
414.274225	31.6915976	414.274225	6.85480766	414.274225	1.4705586
414.374225	30.7894767	414.374225	6.65986357	414.374225	1.42905012
414.474225	30.0198173	414.474225	6.4910066	414.474225	1.39307878
414.574225	29.365665	414.574225	6.3449716	414.574225	1.36199208
414.674225	28.8137973	414.674225	6.21904687	414.674225	1.33518326
414.774225	28.3530042	414.774225	6.11100088	414.774225	1.31218426
414.874225	27.9727009	414.874225	6.01880261	414.874225	1.29256824
414.974225	27.661551	414.974225	5.940266	414.974225	1.27588066
415	27.5910622	415	5.92196816	415	1.27199042
415.074225	27.4069704	415.074225	5.87303204	415.074225	1.26158929
415.174225	27.1947697	415.174225	5.81444905	415.174225	1.24915637
415.274225	27.0098607	415.274225	5.76176672	415.274225	1.23800541
415.358282	26.8645126	415.358282	5.71995375	415.358282	1.22912883
415.374225	26.8372268	415.374225	5.71214279	415.374225	1.22747305
415.582817	26.4605604	415.582817	5.60815996	415.582817	1.20542332
416	25.4531143	416	5.36027278	416	1.15282936
416.074845	25.2390518	416.074845	5.30962928	416.074845	1.14209576
416.14969	25.0198618	416.14969	5.25750196	416.14969	1.13105698
416.374225	24.3682368	416.374225	5.09069615	416.374225	1.09576679
417.074845	31.6103019	417.074845	5.90889267	417.074845	1.29000687
417.358282	29.130967	417.358282	5.63481648	417.358282	1.20972878
417.374225	29.314087	417.374225	5.67390221	417.374225	1.21667472
417.582817	36.1949171	417.582817	6.77802901	417.582817	1.44873323
418	25.2931305	418	5.06095664	418	1.0909743
418.074845	24.1831767	418.074845	4.88809389	418.074845	1.05268451
418.14969	23.3833741	418.14969	4.75835055	418.14969	1.02425394

Final State: j'=4		Final State: j'=3		Final State: j'=2	
E(cm⁻¹)	σ (Å²)	E(cm⁻¹)	σ (Å²)	E(cm⁻¹)	σ (Å²)
418.374225	21.8984165	418.374225	4.5030056	418.374225	0.968924003
418.582817	21.0599252	418.582817	4.34883181	418.582817	0.935808715
419.14969	19.6121651	419.14969	4.06530606	419.14969	0.875227172
419.358282	19.2107956	419.358282	3.98354084	419.358282	0.857800153
419.374225	19.1818848	419.374225	3.97761365	419.374225	0.856534973
419.582817	18.8230529	419.582817	3.90343339	419.582817	0.840741728
420	18.2044012	420	3.77270546	420	0.812950053
420.074845	18.1072601	420.074845	3.75165734	420.074845	0.808485138
420.14969	18.0146786	420.14969	3.73138325	420.14969	0.804186795
420.374225	17.7672725	420.374225	3.67557548	420.374225	0.792386373
421.358282	17.6002576	421.358282	3.57335472	421.358282	0.771930674
421.374225	17.6154342	421.374225	3.57440209	421.374225	0.772196696
421.582817	17.8581901	421.582817	3.59509271	421.582817	0.777179453
422	18.2782908	422	3.6299796	422	0.785550387
422.074845	18.2893734	422.074845	3.62734517	422.074845	0.785057942
422.14969	18.2726861	422.14969	3.62078099	422.14969	0.783691132
422.358282	18.0893142	422.358282	3.5829781	422.358282	0.775558773
422.374225	18.0682214	422.374225	3.57907477	422.374225	0.774713357
423.358282	16.3064808	423.358282	3.2724046	423.358282	0.708240068
423.374225	16.280995	423.374225	3.26789615	423.374225	0.70726855
423.582817	15.9675186	423.582817	3.21193396	423.582817	0.695201825
424	15.446649	424	3.11571628	424	0.674545663
424.074845	15.3681229	424.074845	3.10062465	424.074845	0.671321934
424.14969	15.2944902	424.14969	3.0862239	424.14969	0.668252301
424.374225	15.1081534	424.374225	3.04776379	424.374225	0.660114534
425	16.658503	425	3.20669973	425	0.698599343
425.358282	19.323632	425.358282	3.42316989	425.358282	0.78063787
425.374225	18.8027611	425.374225	3.37653157	425.374225	0.762155235
425.582817	20.1927797	425.582817	3.56527283	425.582817	0.779848159
426	14.267279	426	2.84842479	426	0.61886203
426.074845	14.1021183	426.074845	2.82640691	426.074845	0.614069133
426.14969	13.9880703	426.14969	2.81003617	426.14969	0.610513863
426.374225	13.7733657	426.374225	2.7751019	426.374225	0.602979676
427.074845	13.3820952	427.074845	2.69832808	427.074845	0.586649178
427.358282	13.2426474	427.358282	2.66986945	427.358282	0.580618399
427.374225	13.2348462	427.374225	2.66829087	427.374225	0.580279742
427.582817	13.1316352	427.582817	2.64743636	427.582817	0.57586028
428	12.9226258	428	2.60584835	428	0.567025652
428.074845	12.8848617	428.074845	2.59841446	428.074845	0.565444696
428.14969	12.8470697	428.14969	2.59099549	428.14969	0.563866686
428.374225	12.7337655	428.374225	2.56885846	428.374225	0.559156298
428.582817	12.6290531	428.582817	2.54851272	428.582817	0.554825336
429.14969	12.3510184	429.14969	2.49476858	429.14969	0.543385797
429.374225	12.2447696	429.374225	2.47425846	429.374225	0.539023046
429.582817	12.1484957	429.582817	2.45565512	429.582817	0.535068871

Final State: j’=4		Final State: j’=3		Final State: j’=2	
E(cm⁻¹)	σ (Å²)	E(cm⁻¹)	σ (Å²)	E(cm⁻¹)	σ (Å²)
430	11.9637319	430	2.41983033	430	0.527464309
430.074845	11.9317549	430.074845	2.41360538	430.074845	0.526144648
430.14969	11.9001503	430.14969	2.40744367	430.14969	0.52483909
430.374225	11.8076518	430.374225	2.38934311	430.374225	0.521008191
431.358282	11.4522242	431.358282	2.31770611	431.358282	0.505972167
431.374225	11.4473447	431.374225	2.31666618	431.374225	0.505758659
431.582817	11.3878446	431.582817	2.3036684	431.582817	0.503086974
432	11.30133	432	2.28166474	432	0.498733
432.074845	11.2927313	432.074845	2.27854011	432.074845	0.498158149
432.14969	11.287113	432.14969	2.27576162	432.14969	0.497670858
432.358282	11.2914906	432.358282	2.27033426	432.358282	0.496901346
432.374225	11.2932946	432.374225	2.27008796	432.374225	0.496885655
433.358282	11.7738711	433.358282	2.29653878	433.358282	0.506621891
433.374225	11.7740283	433.374225	2.2960964	433.374225	0.50655325
433.582817	11.7039531	433.582817	2.28243961	433.582817	0.503621027
434	11.3457397	434	2.23216933	434	0.49174708
434.074845	11.2769192	434.074845	2.22279196	434.074845	0.489524926
434.14969	11.2102365	434.14969	2.21367873	434.14969	0.487371355
434.374225	11.0268857	434.374225	2.18830245	434.374225	0.481417728
435	10.6400338	435	2.13181486	435	0.46845707
435.358282	10.4737392	435.358282	2.10582752	435.358282	0.46263784
435.374225	10.4669473	435.374225	2.10474265	435.374225	0.46239681
435.582817	10.3818691	435.582817	2.09100161	435.582817	0.459353777
436	10.2285916	436	2.06558022	436	0.453774126
436.074845	10.2029714	436.074845	2.06125499	436.074845	0.452830467
436.14969	10.1778302	436.14969	2.05699129	436.14969	0.451901494
436.374225	10.1050008	436.374225	2.04453656	436.374225	0.449196273
437.074845	9.89765991	437.074845	2.00835692	437.074845	0.44139352
437.358282	9.82057953	437.358282	1.99467129	437.358282	0.43846121
437.374225	9.81636783	437.374225	1.99392629	437.374225	0.438300837
437.582817	9.76185388	437.582817	1.98416599	437.582817	0.436217006
438	9.65777807	438	1.96538344	438	0.432217738
438.074845	9.63977231	438.074845	1.96211049	438.074845	0.431523119
438.14969	9.62196505	438.14969	1.9588661	438.14969	0.430835028
438.374225	9.56973235	438.374225	1.94930661	438.374225	0.428812212
438.582817	9.52280299	438.582817	1.94065497	438.582817	0.426986111
439.14969	9.40324261	439.14969	1.91826571	439.14969	0.422290344
439.358282	9.36230805	439.358282	1.91044768	439.358282	0.420663278
439.374225	9.35925584	439.374225	1.90986316	439.374225	0.420541683
439.582817	9.3201693	439.582817	1.90229101	439.582817	0.418974394
440	9.24727084	440	1.88785235	440	0.41601102
440.074845	9.23492685	440.074845	1.88535926	440.074845	0.415503066
440.14969	9.2227987	440.14969	1.88289484	440.14969	0.415002167
440.374225	9.18764643	440.374225	1.87566757	440.374225	0.413539622
441.358282	9.04789384	441.358282	1.84615127	441.358282	0.407634008

Final State: j’=4		Final State: j’=3		Final State: j’=2	
E(cm⁻¹)	σ (Å²)	E(cm⁻¹)	σ (Å²)	E(cm⁻¹)	σ (Å²)
441.374225	9.04569093	441.374225	1.84568841	441.374225	0.407541343
441.582817	9.01660534	441.582817	1.83963684	441.582817	0.406327678
442	8.95620451	442	1.82745763	442	0.403861554
442.074845	8.94496207	442.074845	1.82525227	442.074845	0.403411133
442.14969	8.93358599	442.14969	1.82303982	442.14969	0.402957982
442.358282	8.90118427	442.358282	1.81683567	442.358282	0.401680835
442.374225	8.89866659	442.374225	1.81635931	442.374225	0.401582363
443.358282	8.73520384	443.358282	1.78665367	443.358282	0.395363306
443.374225	8.73248324	443.374225	1.78617327	443.374225	0.395262292
443.582817	8.69677825	443.582817	1.77989633	443.582817	0.393934219
444	8.6255586	444	1.76748251	444	0.391303103
444.074845	8.61286202	444.074845	1.76527993	444.074845	0.390835889
444.14969	8.60020081	444.14969	1.76308588	444.14969	0.390370359
444.374225	8.56246523	444.374225	1.75655764	444.374225	0.388985139
445	8.45974828	445	1.7388313	445	0.385226431
445.358282	8.40282926	445.358282	1.7290124	445.358282	0.383147873
445.374225	8.40033054	445.374225	1.72858106	445.374225	0.383056671
445.582817	8.36791376	445.582817	1.72298455	445.582817	0.381873687
446	8.30461879	446	1.712042	446	0.379564881
446.074845	8.29348192	446.074845	1.7101139	446.074845	0.379158695
446.14969	8.28241076	446.14969	1.70819664	446.14969	0.378754883
446.374225	8.24959659	446.374225	1.70250752	446.374225	0.377558168
447.074845	8.15099453	447.074845	1.68535551	447.074845	0.373961264
447.358282	8.11270449	447.358282	1.67866815	447.358282	0.372563965
447.374225	8.11058778	447.374225	1.67830005	447.374225	0.372487443
447.582817	8.08301278	447.582817	1.6734696	447.582817	0.37148024
448	8.02929859	448	1.66403996	448	0.369518827
448.074845	8.01985868	448.074845	1.66237864	448.074845	0.369174226
448.14969	8.01047613	448.14969	1.66072686	448.14969	0.368831392
448.374225	7.98268002	448.374225	1.65582523	448.374225	0.367816265
448.582817	7.95730835	448.582817	1.65134377	448.582817	0.366889436
449.14969	7.89045196	449.14969	1.63950007	449.14969	0.364447141
449.358282	7.86657716	449.358282	1.63525943	449.358282	0.363575667
449.374225	7.86476753	449.374225	1.63493932	449.374225	0.363509626
449.582817	7.84128172	449.582817	1.6307625	449.582817	0.36265251
450	7.79532265	450	1.62258403	450	0.360977205
450.074845	7.78721111	450.074845	1.6211401	450.074845	0.360681812
450.14969	7.77913831	450.14969	1.61970296	450.14969	0.360388009
450.374225	7.75514226	450.374225	1.61543203	450.374225	0.359515415
451.358282	7.65343182	451.358282	1.59737085	451.358282	0.355833932
451.374225	7.65182768	451.374225	1.5970879	451.374225	0.355776179
451.582817	7.630904	451.582817	1.59338743	451.582817	0.355023242
452	7.58962058	452	1.58610995	452	0.353543001
452.074845	7.58228863	452.074845	1.58482082	452.074845	0.353280875
452.14969	7.57497824	452.14969	1.5835366	452.14969	0.353019795

Final State: j'=4		Final State: j'=3		Final State: j'=2	
E(cm⁻¹)	σ (Å²)	E(cm⁻¹)	σ (Å²)	E(cm⁻¹)	σ (Å²)
452.358282	7.55471766	452.358282	1.57998289	452.358282	0.352297353
452.374225	7.55317576	452.374225	1.57971281	452.374225	0.352242435
453.358282	7.45976752	453.358282	1.56344622	453.358282	0.348936876
453.374225	7.45828824	453.374225	1.56319116	453.374225	0.348884976
453.582817	7.43892717	453.582817	1.55984298	453.582817	0.348204977
454	7.40066701	454	1.55325147	454	0.3468665
454.074845	7.3938668	454.074845	1.55208303	454.074845	0.346629286
454.14969	7.38708574	454.14969	1.55091886	454.14969	0.346393022
454.374225	7.36686013	454.374225	1.5474519	454.374225	0.345689454
455	7.31142686	455	1.5379895	455	0.343770389
455.358282	7.28031988	455.358282	1.53270227	455.358282	0.342699603
455.374225	7.27894636	455.374225	1.53246917	455.374225	0.342652397
455.582817	7.26106088	455.582817	1.52943694	455.582817	0.342038637
456	7.22576528	456	1.52346735	456	0.340831423
456.074845	7.21950053	456.074845	1.52240958	456.074845	0.340617682
456.14969	7.21325625	456.14969	1.52135594	456.14969	0.340404845
456.374225	7.19464699	456.374225	1.51821885	456.374225	0.33977144
457.074845	7.13777099	457.074845	1.50865884	457.074845	0.337844561
457.358282	7.11527333	457.358282	1.50488808	457.358282	0.337086098
457.374225	7.11401477	457.374225	1.50467657	457.374225	0.337043398
457.582817	7.09765846	457.582817	1.50193994	457.582817	0.336493793
458	7.065414	458	1.49655236	458	0.335412971
458.074845	7.05969524	458.074845	1.49559805	458.074845	0.335221718
458.14969	7.05399647	458.14969	1.49464741	458.14969	0.335031322
458.374225	7.03701902	458.374225	1.49181754	458.374225	0.334464903
458.582817	7.02140588	458.582817	1.48921793	458.582817	0.333945163
459.14969	6.97973476	459.14969	1.48229312	459.14969	0.33256343
459.358282	6.96467536	459.358282	1.47979554	459.358282	0.332066093
459.374225	6.96352893	459.374225	1.47960536	459.374225	0.332028238
459.582817	6.94862637	459.582817	1.47713683	459.582817	0.331537304
460	6.91924329	460	1.47227744	460	0.330572502
460.074845	6.91403069	460.074845	1.47141656	460.074845	0.330401823
460.14969	6.90883593	460.14969	1.47055896	460.14969	0.330231838
460.374225	6.89335608	460.374225	1.46800558	460.374225	0.3297262
461.358282	6.82730863	461.358282	1.45715019	461.358282	0.327583569
461.582817	6.81263176	461.582817	1.45474731	461.582817	0.327110881
462	6.78573333	462	1.45035308	462	0.32624793
462.074845	6.78095759	462.074845	1.44957419	462.074845	0.326095159
462.14969	6.77619671	462.14969	1.44879821	462.14969	0.325943062
462.358282	6.76300646	462.358282	1.44665048	462.358282	0.325522335
462.374225	6.76200303	462.374225	1.44648721	462.374225	0.32549038
463.358282	6.7013146	463.358282	1.43665116	463.358282	0.323569978
463.582817	6.68780062	463.582817	1.43447135	463.582817	0.323145795
464	6.6630074	464	1.43048282	464	0.322370935
464.074845	6.65860196	464.074845	1.42977558	464.074845	0.322233728

Final State: j'=4		Final State: j'=3		Final State: j'=2	
E(cm⁻¹)	σ (Å²)	E(cm⁻¹)	σ (Å²)	E(cm⁻¹)	σ (Å²)
464.14969	6.65420921	464.14969	1.42907096	464.14969	0.322097094
464.374225	6.64110823	464.374225	1.42697158	464.374225	0.321690257
465	6.60518559	465	1.42123649	465	0.320581544
465.358282	6.5850003	465.358282	1.41802751	465.358282	0.319962622
465.374225	6.58410874	465.374225	1.41788696	465.374225	0.31993601
465.582817	6.57249013	465.582817	1.41604503	465.582817	0.319581569
466	6.54952114	466	1.41241261	466	0.318883013
466.074845	6.54543789	466.074845	1.41176877	466.074845	0.318759545
466.374225	6.52922171	466.374225	1.4092156	466.374225	0.318270677
467.074845	6.49196245	467.074845	1.40337442	467.074845	0.317154519
467.358282	6.47716024	467.358282	1.40106418	467.358282	0.31671436
467.582817	6.46554345	467.582817	1.39925567	467.582817	0.3163697
468	6.44421118	468	1.39594384	468	0.315740947
468.074845	6.44041836	468.074845	1.39535634	468.074845	0.315629631
468.14969	6.43663572	468.14969	1.39477091	468.14969	0.315518695
468.374225	6.42535041	468.374225	1.39302637	468.374225	0.315188639
468.582817	6.41494754	468.582817	1.3914221	468.582817	0.314885247
469.14969	6.38707194	469.14969	1.38713739	469.14969	0.314077815
469.358282	6.37695818	469.358282	1.38558847	469.358282	0.313786507
469.582817	6.36615531	469.582817	1.38393744	469.582817	0.313476534
470	6.34631405	470	1.38091412	470	0.312910184
470.074845	6.34278563	470.074845	1.38037769	470.074845	0.312809894
470.14969	6.3392667	470.14969	1.37984312	470.14969	0.312709998
470.374225	6.32876598	470.374225	1.37825046	470.374225	0.312412643
471.358282	6.2837225	471.358282	1.37145643	471.358282	0.311149918
471.582817	6.27366284	471.582817	1.36994816	471.582817	0.31087081
472	6.25518086	472	1.36718563	472	0.310360994
472.074845	6.25189351	472.074845	1.36669552	472.074845	0.310270736
472.14969	6.24861476	472.14969	1.36620698	472.14969	0.310180817
472.358282	6.23952143	472.358282	1.36485371	472.358282	0.309931934
472.374225	6.23882917	472.374225	1.36475073	472.374225	0.309913068
473.358282	6.19682965	473.358282	1.3585402	473.358282	0.308776744
473.582817	6.18744363	473.582817	1.35716085	473.582817	0.308525639
474	6.17019391	474	1.35463423	474	0.308066933
474.074845	6.16712502	474.074845	1.35418586	474.074845	0.307985681
474.14969	6.16406375	474.14969	1.35373897	474.14969	0.307904764
474.374225	6.15492656	474.374225	1.35240725	474.374225	0.307663998
475	6.12982128	475	1.34876455	475	0.307007775
475.358282	6.11568132	475.358282	1.34672384	475.358282	0.306641738
475.374225	6.11505586	475.374225	1.34663372	475.374225	0.306625605
476	6.09076939	476	1.34314792	476	0.306003306
476.074845	6.08789792	476.074845	1.34273738	476.074845	0.305930228
476.14969	6.08503342	476.14969	1.34232821	476.14969	0.305857449
476.374225	6.07648166	476.374225	1.34110857	476.374225	0.305640873
477.074845	6.05019381	477.074845	1.337379	477.074845	0.304981446

Final State: j'=4		Final State: j'=3		Final State: j'=2	
E(cm⁻¹)	σ (Å²)	E(cm⁻¹)	σ (Å²)	E(cm⁻¹)	σ (Å²)
477.358282	6.03972619	477.358282	1.33590221	477.358282	0.304721556
477.582817	6.031501	477.582817	1.33474516	477.582817	0.304518449
478	6.01637352	478	1.33262503	478	0.304147433
478.074845	6.01368069	478.074845	1.33224869	478.074845	0.304081741
478.14969	6.01099423	478.14969	1.3318736	478.14969	0.30401632
478.374225	6.00297263	478.374225	1.33075558	478.374225	0.303821601
478.582817	5.99557116	478.582817	1.32972658	478.582817	0.303642772
479.14969	5.97569892	479.14969	1.32697643	479.14969	0.303166738
479.358282	5.96847421	479.358282	1.32598127	479.358282	0.302995145
479.582817	5.96074944	479.582817	1.32491992	479.582817	0.302812582
480	5.94653766	480	1.32297495	480	0.302479139
480.074845	5.94400732	480.074845	1.32262956	480.074845	0.302420102
480.14969	5.94148264	480.14969	1.32228541	480.14969	0.302361292
480.374225	5.93394319	480.374225	1.32125951	480.374225	0.302186308
481.358282	5.90150012	481.358282	1.31687707	481.358282	0.301443693
481.582817	5.89423144	481.582817	1.31590259	481.582817	0.301279679
482	5.88085496	482	1.31411638	482	0.300980157
482.074845	5.87847275	482.074845	1.31379928	482.074845	0.300927127
482.14969	5.87609579	482.14969	1.31348316	482.14969	0.300874311
482.358282	5.86949905	482.358282	1.31260737	482.358282	0.300728204
482.374225	5.86899646	482.374225	1.31254076	482.374225	0.300717123
483.358282	5.83843049	483.358282	1.30851399	483.358282	0.300050217
483.582817	5.83157834	483.582817	1.30761833	483.582817	0.299902909
484	5.81896582	484	1.3059763	484	0.299633978
484.14969	5.81447686	484.14969	1.30539392	484.14969	0.29953903
484.374225	5.80777941	484.374225	1.30452733	484.374225	0.299397821
485	5.78934051	485	1.30215468	485	0.299013479
485.358282	5.77893043	485.358282	1.30082357	485.358282	0.298799126
485.374225	5.77846997	485.374225	1.30076467	485.374225	0.29878971
485.582817	5.77246087	485.582817	1.29999865	485.582817	0.298666711
486	5.76054636	486	1.29848712	486	0.298425268
486.074845	5.75842352	486.074845	1.29821883	486.074845	0.298382509
486.14969	5.75630553	486.14969	1.29795125	486.14969	0.298339905
486.374225	5.7499761	486.374225	1.29715373	486.374225	0.298213373
487.074845	5.73048015	487.074845	1.29471001	487.074845	0.297827542
487.358282	5.72269837	487.358282	1.29374135	487.358282	0.297675526
487.582817	5.71657698	487.582817	1.29298161	487.582817	0.297556754
488	5.70530189	488	1.29158812	488	0.297339803
488.074845	5.70329248	488.074845	1.29134058	488.074845	0.29730141
488.14969	5.70128706	488.14969	1.29109376	488.14969	0.297263116
488.374225	5.69529525	488.374225	1.29035784	488.374225	0.297149258
488.582817	5.68976104	488.582817	1.28967997	488.582817	0.297044626
489.14969	5.67487565	489.14969	1.28786592	489.14969	0.296766049
489.358282	5.66945404	489.358282	1.28720851	489.358282	0.296665647
489.582817	5.66365142	489.582817	1.28650677	489.582817	0.296558756

Final State: j'=4		Final State: j'=3		Final State: j'=2	
E(cm⁻¹)	σ (Å²)	E(cm⁻¹)	σ (Å²)	E(cm⁻¹)	σ (Å²)
490	5.65296014	490	1.28521928	490	0.296363407
490.074845	5.65105432	490.074845	1.2849905	490.074845	0.29632881
490.14969	5.64915218	490.14969	1.28476237	490.14969	0.296294336
490.374225	5.64346786	490.374225	1.28408189	490.374225	0.296191722
491.358282	5.6189373	491.358282	1.28116755	491.358282	0.295755527
491.582817	5.61342551	491.582817	1.28051753	491.582817	0.295658911
492	5.60326621	492	1.27932426	492	0.295482325
492.074845	5.6014547	492.074845	1.27911214	492.074845	0.295451017
492.14969	5.59964651	492.14969	1.27890053	492.14969	0.295419815
492.358282	5.59462487	492.358282	1.27831396	492.358282	0.295333429
492.374225	5.59424223	492.374225	1.27826934	492.374225	0.295326897
493.358282	5.57090425	493.358282	1.2755623	493.358282	0.294931057
493.582817	5.56565642	493.582817	1.27495765	493.582817	0.294843218
494	5.55597999	494	1.2738469	494	0.294682311
494.074845	5.55425449	494.074845	1.27364923	494.074845	0.294653726
494.14969	5.55253168	494.14969	1.27345216	494.14969	0.294625277
494.374225	5.54738133	494.374225	1.27286394	494.374225	0.294540472
495	5.53316772	495	1.27124687	495	0.294308118
495.358282	5.52512139	495.358282	1.27033593	495.358282	0.294177446
495.374225	5.52476498	495.374225	1.27029562	495.374225	0.294171664
495.582817	5.52011244	495.582817	1.26977025	495.582817	0.294096721
496	5.51087155	496	1.26872937	496	0.293948152
496.074845	5.50922251	496.074845	1.26854407	496.074845	0.293921754
496.14969	5.50757643	496.14969	1.26835926	496.14969	0.293895372
496.374225	5.50265436	496.374225	1.26780673	496.374225	0.293816808
497.074845	5.48744804	497.074845	1.26610607	497.074845	0.293574971
497.358282	5.48136042	497.358282	1.26542736	497.358282	0.293478576
497.582817	5.47656309	497.582817	1.26489314	497.582817	0.293402633
498	5.4677087	498	1.2639087	498	0.293262704
498.074845	5.46612815	498.074845	1.26373317	498.074845	0.293237711
498.14969	5.46454985	498.14969	1.26355789	498.14969	0.29321278
498.374225	5.4598293	498.374225	1.26303395	498.374225	0.29313822
498.582817	5.45546269	498.582817	1.26254968	498.582817	0.293069181
499.14969	5.44368438	499.14969	1.26124418	499.14969	0.292882693
499.358282	5.43938219	499.358282	1.26076749	499.358282	0.292814376
499.582817	5.43476978	499.582817	1.26025636	499.582817	0.292740966
500	5.42623979	500	1.25931046	500	0.292604
500.074845	5.42472774	500.074845	1.25914294	500.074845	0.292580328
500.14969	5.42320775	500.14969	1.25897441	500.14969	0.292555926
500.374225	5.41865972	500.374225	1.25846954	500.374225	0.292482614
501.358282	5.39892667	501.358282	1.25627164	501.358282	0.292159881
501.582817	5.39446667	501.582817	1.25577237	501.582817	0.292085569
502.074845	5.38474447	502.074845	1.25467977	502.074845	0.291921379
502.14969	5.38327145	502.14969	1.25451365	502.14969	0.291896224
502.358282	5.37917392	502.358282	1.25405054	502.358282	0.291825738

Final State: j’=4		Final State: j’=3		Final State: j’=2	
E(cm⁻¹)	σ (Å²)	E(cm⁻¹)	σ (Å²)	E(cm⁻¹)	σ (Å²)
502.374225	5.3788612	502.374225	1.25401508	502.374225	0.291820325
503.358282	5.35967527	503.358282	1.25182295	503.358282	0.29147926
503.582817	5.35532614	503.582817	1.25131963	503.582817	0.291399052
504.074845	5.34582656	504.074845	1.25020951	504.074845	0.291219142
504.14969	5.34438436	504.14969	1.25003948	504.14969	0.291191236
504.374225	5.34006363	504.374225	1.24952795	504.374225	0.291106566
505	5.32803927	505	1.24808201	505	0.290861245
505.358282	5.32118245	505.358282	1.24724137	505.358282	0.290715276
505.374225	5.32087988	505.374225	1.24720261	505.374225	0.29070959
505.582817	5.31688022	505.582817	1.2467065	505.582817	0.290620412
506.074845	5.30744864	506.074845	1.24551215	506.074845	0.290403596
506.14969	5.30601256	506.14969	1.24532744	506.14969	0.290369432
506.374225	5.30170112	506.374225	1.24476773	506.374225	0.29026475
507.074845	5.28819422	507.074845	1.24295914	507.074845	0.289914997
507.358282	5.28269495	507.358282	1.24219514	507.358282	0.289761698
507.582817	5.27831822	507.582817	1.24157406	507.582817	0.289634576
508.074845	5.26864589	508.074845	1.24015498	508.074845	0.289335511
508.14969	5.26716322	508.14969	1.23993122	508.14969	0.289287385
508.374225	5.26269111	508.374225	1.23924531	508.374225	0.289137705
508.582817	5.25850265	508.582817	1.23858726	508.582817	0.288991366
509.14969	5.24690978	509.14969	1.2366755	509.14969	0.288552588
509.358282	5.24254787	509.358282	1.23591681	509.358282	0.28837313
509.582817	5.23777744	509.582817	1.23506081	509.582817	0.288166581
510	5.22865275	510	1.23333686	510	0.287738845
510.074845	5.22698821	510.074845	1.2330085	510.074845	0.287656198
510.14969	5.22529744	510.14969	1.2326708	510.14969	0.287570382
510.374225	5.22012949	510.374225	1.23160875	510.374225	0.287297539
511.358282	5.19490012	511.358282	1.225691	511.358282	0.285707639
511.582817	5.18818424	511.582817	1.22388463	511.582817	0.285206677
512.074845	5.17101125	512.074845	1.21879747	512.074845	0.283782946
512.14969	5.1679599	512.14969	1.21782599	512.14969	0.28351128
512.358282	5.15850661	512.358282	1.21470115	512.358282	0.282643306
513.358282	5.06531962	513.358282	1.1847636	513.358282	0.278237698
513.582817	5.08147464	513.582817	1.19589727	513.582817	0.282995031
514.074845	5.09947992	514.074845	1.21512583	514.074845	0.293480903
514.14969	5.12342908	514.14969	1.22601365	514.14969	0.296264336
514.374225	5.15909542	514.374225	1.23822128	514.374225	0.296496598
515	5.08548297	515	1.21019617	515	0.286293325
515.358282	5.06450722	515.358282	1.19886891	515.358282	0.284432493
515.374225	5.06188259	515.374225	1.19841377	515.374225	0.284666317
515.582817	5.05774637	515.582817	1.20876412	515.582817	0.292412975
516.074845	5.15800381	516.074845	1.24666655	516.074845	0.297936083
516.14969	5.14698141	516.14969	1.24347505	516.14969	0.296753639
516.374225	5.17411993	516.374225	1.25019345	516.374225	0.293954076
517.074845	5.10125989	517.074845	1.2152228	517.074845	0.284567807

Final State: j'=4		Final State: j'=3		Final State: j'=2	
E(cm⁻¹)	σ (Å²)	E(cm⁻¹)	σ (Å²)	E(cm⁻¹)	σ (Å²)
517.358282	5.03074449	517.358282	1.18669108	517.358282	0.278923742
517.582817	5.04061509	517.582817	1.20538603	517.582817	0.289212337
518.074845	5.06726696	518.074845	1.22305787	518.074845	0.296938442
518.14969	5.08741847	518.14969	1.2327422	518.14969	0.299477745
518.374225	5.09779196	518.374225	1.23846894	518.374225	0.298460928
518.582817	5.1222181	518.582817	1.24195713	518.582817	0.294013089
519.14969	5.12283359	519.14969	1.24082204	519.14969	0.295627391
519.358282	5.08280968	519.358282	1.22358138	519.358282	0.290485333
519.582817	5.05645	519.582817	1.225144	519.582817	0.29609624
520	5.11401504	520	1.24190209	520	0.29614677
520.074845	5.10688594	520.074845	1.23868903	520.074845	0.294909321
520.14969	5.09951951	520.14969	1.23550534	520.14969	0.293771149
520.374225	5.07486628	520.374225	1.22549788	520.374225	0.290600502
521.358282	5.03099423	521.358282	1.23230367	521.358282	0.305401073
521.582817	5.05290484	521.582817	1.25242455	521.582817	0.305925523
522.074845	5.11898105	522.074845	1.25176258	522.074845	0.297137193
522.14969	5.10791659	522.14969	1.2456358	522.14969	0.295128709
522.358282	5.05558579	522.358282	1.21995241	522.358282	0.287702134
522.374225	5.04920818	522.374225	1.21697299	522.374225	0.287027342
523.358282	5.10188617	523.358282	1.25272357	523.358282	0.299768911
523.582817	5.08675141	523.582817	1.24636313	523.582817	0.29752069
524.074845	5.05230937	524.074845	1.23264812	524.074845	0.293109786
524.14969	5.04607555	524.14969	1.23020822	524.14969	0.292381217
524.374225	5.02369059	524.374225	1.2214973	524.374225	0.289906489
525	4.9106828	525	1.18768283	525	0.287900165
525.358282	4.99226846	525.358282	1.23681007	525.358282	0.303220481
525.374225	4.99562796	525.374225	1.23843018	525.374225	0.303389361
525.582817	5.05660049	525.582817	1.25979448	525.582817	0.301155321
526.074845	5.04969129	526.074845	1.24503075	526.074845	0.300183628
526.14969	5.02612647	526.14969	1.23569212	526.14969	0.297562263
526.374225	4.99827066	526.374225	1.22164898	526.374225	0.293710714
527.074845	5.10116923	527.074845	1.27177692	527.074845	0.307238633
527.358282	5.07991324	527.358282	1.262118	527.358282	0.303611801
527.582817	5.06699371	527.582817	1.25676158	527.582817	0.301702116
528.074845	5.04239667	528.074845	1.2472482	528.074845	0.298501804
528.14969	5.03870946	528.14969	1.2458648	528.14969	0.298056421
528.374225	5.02723635	528.374225	1.24158199	528.374225	0.296707469
528.582817	5.01552123	528.582817	1.23720901	528.582817	0.295374339
529.14969	4.96948796	529.14969	1.21987891	529.14969	0.290515577
529.582817	4.88502793	529.582817	1.19010351	529.582817	0.284317907
530	4.89991815	530	1.22034506	530	0.303973128
530.074845	4.92311769	530.074845	1.23353031	530.074845	0.307451018
530.14969	4.92846786	530.14969	1.24052198	530.14969	0.308043502
530.374225	5.03182992	530.374225	1.26679099	530.374225	0.306843979
531.582817	5.09214208	531.582817	1.29112933	531.582817	0.315451607

Final State: j’=4		Final State: j’=3		Final State: j’=2	
E(cm⁻¹)	σ (Å²)	E(cm⁻¹)	σ (Å²)	E(cm⁻¹)	σ (Å²)
532.074845	5.04666194	532.074845	1.26817532	532.074845	0.306306632
532.14969	5.04224845	532.14969	1.26632718	532.14969	0.305651419
532.358282	5.03145903	532.358282	1.26198782	532.358282	0.304143416
532.374225	5.03071587	532.374225	1.26169519	532.374225	0.30404363
533.582817	4.98197754	533.582817	1.24410968	533.582817	0.298386324
534.074845	4.95883479	534.074845	1.23586874	534.074845	0.295948629
534.14969	4.95458329	534.14969	1.2343258	534.14969	0.295505446
534.374225	4.93972361	534.374225	1.22887615	534.374225	0.293973777
535	4.85117953	535	1.19704076	535	0.286374046
535.374225	4.80131298	535.374225	1.19654503	535.374225	0.295105708
535.582817	4.85637591	535.582817	1.23158917	535.582817	0.303812839
536.074845	4.96100487	536.074845	1.25218632	536.074845	0.303885237
536.14969	4.97361809	536.14969	1.25331867	536.14969	0.301593064
536.374225	4.88254642	536.374225	1.21308957	536.374225	0.291591442
537.074845	5.0428031	537.074845	1.28931893	537.074845	0.31462704
537.358282	5.01233306	537.358282	1.2746272	537.358282	0.309070982
537.582817	4.99759883	537.582817	1.26837834	537.582817	0.306902203
538.074845	4.9752934	538.074845	1.25978673	538.074845	0.304041238
538.14969	4.97248607	538.14969	1.25877288	538.14969	0.303712729
538.374225	4.96454982	538.374225	1.25597473	538.374225	0.302816268
538.582817	4.9576201	538.582817	1.25359974	538.582817	0.302066873
539.14969	4.93954808	539.14969	1.24757284	539.14969	0.300210172
539.582817	4.925138	539.582817	1.24277754	539.582817	0.2987733
540	4.90914718	540	1.2373194	540	0.297176227
540.074845	4.90589957	540.074845	1.2361849	540.074845	0.296849779
540.14969	4.90246604	540.14969	1.23497925	540.14969	0.296504452
540.374225	4.89080488	540.374225	1.23082523	540.374225	0.295330864
541.582817	4.73267585	541.582817	1.1962962	541.582817	0.297771593
542.074845	4.93896104	542.074845	1.26505126	542.074845	0.308116509
542.14969	4.92287987	542.14969	1.25611372	542.14969	0.305167405
542.358282	4.85954084	542.358282	1.23071996	542.358282	0.299086238
542.374225	4.86619057	542.374225	1.23274173	542.374225	0.2989914
543.582817	4.94074944	543.582817	1.26437847	543.582817	0.305918231
544.074845	4.91436636	544.074845	1.25379514	544.074845	0.302480142
544.14969	4.91113479	544.14969	1.25255534	544.14969	0.302084109
544.374225	4.90201504	544.374225	1.24910112	544.374225	0.300986838
545	4.87842295	545	1.2402774	545	0.29821837
545.374225	4.86383452	545.374225	1.23471887	545.374225	0.296498515
545.582817	4.85496237	545.582817	1.23125581	545.582817	0.295438212
546.074845	4.82946329	546.074845	1.2209036	546.074845	0.292349953
546.14969	4.82460806	546.14969	1.21887539	546.14969	0.291765456
546.374225	4.80711788	546.374225	1.21149377	546.374225	0.289725984
547.074845	4.75326569	547.074845	1.1967395	547.074845	0.290197383
547.358282	4.71695959	547.358282	1.18553772	547.358282	0.289788157
547.582817	4.74317336	547.582817	1.20715557	547.582817	0.298552427

Final State: j'=4		Final State: j'=3		Final State: j'=2	
E(cm⁻¹)	σ (Å²)	E(cm⁻¹)	σ (Å²)	E(cm⁻¹)	σ (Å²)
548.074845	4.68559833	548.074845	1.17905506	548.074845	0.285550138
548.14969	4.64741981	548.14969	1.16590744	548.14969	0.282714773
548.374225	4.59774688	548.374225	1.16378158	548.374225	0.290628575
548.582817	4.66924197	548.582817	1.21193353	548.582817	0.304718352
549.14969	4.77502579	549.14969	1.21862093	549.14969	0.300375175
549.582817	4.79206958	549.582817	1.24865658	549.582817	0.313162744
550	4.85979456	550	1.25432863	550	0.306079078
550.074845	4.83423499	550.074845	1.24399629	550.074845	0.303226942
550.14969	4.80917393	550.14969	1.23477537	550.14969	0.300792818
550.374225	4.76203823	550.374225	1.21547983	550.374225	0.297100036
551.582817	4.86854665	551.582817	1.2686528	551.582817	0.311743366
552.074845	4.81944173	552.074845	1.2443394	552.074845	0.3028387
552.14969	4.80960599	552.14969	1.23980787	552.14969	0.301416416
552.358282	4.77394206	552.358282	1.2241004	552.358282	0.296943543
552.374225	4.77050734	552.374225	1.22264454	552.374225	0.29656432
553.582817	4.87862679	553.582817	1.29054425	553.582817	0.323533154
554.074845	4.83973248	554.074845	1.26700565	554.074845	0.311886537
554.14969	4.83361322	554.14969	1.26389539	554.14969	0.310650559
554.374225	4.81457662	554.374225	1.2546362	554.374225	0.307195493
555	4.74019643	555	1.22140045	555	0.296556805
555.374225	4.63600645	555.374225	1.17912795	555.374225	0.285496731
555.582817	4.4961984	555.582817	1.13161791	555.582817	0.27686864
556.074845	4.68840901	556.074845	1.25533119	556.074845	0.319208144
556.14969	4.7663371	556.14969	1.27045018	556.14969	0.32770667
556.374225	4.8835774	556.374225	1.31696634	556.374225	0.336765985
557.074845	4.97279297	557.074845	1.34367301	557.074845	0.335751582
557.358282	4.90547181	557.358282	1.30404551	557.358282	0.322026321
557.582817	4.87614473	557.582817	1.2908857	557.582817	0.317711335
558.074845	4.83020632	558.074845	1.27028622	558.074845	0.31092818
558.14969	4.82337094	558.14969	1.26722395	558.14969	0.309941811
558.374225	4.80087618	558.374225	1.25716777	558.374225	0.306777973
558.582817	4.77432169	558.582817	1.24541482	558.582817	0.303259265
559.14969	4.57902823	559.14969	1.17665928	559.14969	0.290527384
559.582817	4.67743523	559.582817	1.24048047	559.582817	0.325022537
560	4.887706	560	1.31683477	560	0.331176753
560.074845	4.88001331	560.074845	1.31173635	560.074845	0.328674107
560.14969	4.87284167	560.14969	1.30729908	560.14969	0.326605051
560.374225	4.85429669	560.374225	1.29674515	560.374225	0.322009173
561.582817	4.78293091	561.582817	1.2628773	561.582817	0.309521643
562.074845	4.74937836	562.074845	1.24837122	562.074845	0.304913105
562.14969	4.74301419	562.14969	1.24564303	562.14969	0.304082575
562.358282	4.72192846	562.358282	1.23666242	562.358282	0.301423753
562.374225	4.72006762	562.374225	1.23587119	562.374225	0.301195161
563.582817	4.70696943	563.582817	1.27329316	563.582817	0.324491581
564.074845	4.81176255	564.074845	1.29850863	564.074845	0.322876314

Final State: j'=4		Final State: j'=3		Final State: j'=2	
E(cm⁻¹)	σ (Å²)	E(cm⁻¹)	σ (Å²)	E(cm⁻¹)	σ (Å²)
564.14969	4.83750007	564.14969	1.30093345	564.14969	0.320881914
564.374225	4.74165096	564.374225	1.25810825	564.374225	0.309667088
565	4.8595137	565	1.30669614	565	0.324828818
565.374225	4.75943193	565.374225	1.26201539	565.374225	0.312607853
565.582817	4.74710159	565.582817	1.25540845	565.582817	0.309187082
566.074845	4.76197036	566.074845	1.26815482	566.074845	0.313499659
566.374225	4.69013386	566.374225	1.23432476	566.374225	0.302785303
567.074845	4.68803905	567.074845	1.24043337	567.074845	0.305813979
567.358282	4.54616975	567.358282	1.18162166	567.358282	0.289362578
567.582817	4.48159947	567.582817	1.17404219	567.582817	0.293452896
568.074845	4.88472685	568.074845	1.35501507	568.074845	0.352000429
568.14969	4.87704017	568.14969	1.34651312	568.14969	0.345685958
568.374225	4.84020546	568.374225	1.32082489	568.374225	0.332345495
568.582817	4.80809554	568.582817	1.30193474	568.582817	0.324376847
569.14969	4.67589374	569.14969	1.23553433	569.14969	0.302941322
569.582817	4.79327201	569.582817	1.31604497	569.582817	0.341268246
570	4.77703099	570	1.29550922	570	0.323984125
570.074845	4.7694914	570.074845	1.29108648	570.074845	0.322024051
570.14969	4.76193616	570.14969	1.28684101	570.14969	0.320243068
570.374225	4.73859341	570.374225	1.27446513	570.374225	0.31546374
571.582817	4.68391589	571.582817	1.26344538	571.582817	0.31584903
572.074845	4.37959152	572.074845	1.16007077	572.074845	0.289291234
572.14969	4.36446902	572.14969	1.17262281	572.14969	0.297029737
572.358282	4.55491628	572.358282	1.2387496	572.358282	0.316331408
572.374225	4.54899137	572.374225	1.23497216	572.374225	0.318242212
573.582817	4.78037986	573.582817	1.30773703	573.582817	0.330925145
574.074845	4.75707241	574.074845	1.29037006	574.074845	0.320202222
574.14969	4.64548172	574.14969	1.25140161	574.14969	0.310352142
574.374225	4.78560527	574.374225	1.30766345	574.374225	0.325661096
575	4.66118026	575	1.26107052	575	0.309961655
575.374225	4.5342418	575.374225	1.2100137	575.374225	0.294692751
576.074845	4.61947388	576.074845	1.25972122	576.14969	0.30935229
576.14969	4.61062208	576.14969	1.25587008	577.358282	0.324881645
576.374225	4.58373131	576.374225	1.24474049	577.582817	0.313271513
577.074845	4.33971362	577.074845	1.18439002	578.14969	0.310656398
577.358282	4.66099719	577.358282	1.30214136	578.374225	0.309853807
577.582817	4.58875263	577.582817	1.26962018	578.582817	0.30904679
578.074845	4.57616001	578.074845	1.26195838	579.582817	0.304819882
578.14969	4.5752267	578.14969	1.26115791	580	0.30296757
578.374225	4.5704206	578.374225	1.25848708	580.374225	0.299966778
578.582817	4.56401549	578.582817	1.255805	581.582817	0.304849268
579.14969	4.53996862	579.14969	1.24788603	582.074845	0.30996604
579.582817	4.51736922	579.582817	1.24154132	582.14969	0.310209421
580	4.49307334	580	1.23467538	582.358282	0.309973772
580.074845	4.48776045	580.074845	1.23306926	582.374225	0.309939129

Final State: j'=4		Final State: j'=3		Final State: j'=2	
E(cm⁻¹)	σ (Å²)	E(cm⁻¹)	σ (Å²)	E(cm⁻¹)	σ (Å²)
580.14969	4.48171746	580.14969	1.23120175	583.582817	0.308003553
580.374225	4.45435928	580.374225	1.22249362	585	0.306632049
581.582817	4.47767171	581.582817	1.24765964	585.582817	0.305675023
582.074845	4.51344895	582.074845	1.26302438	587.074845	0.306148358
582.14969	4.51959892	582.14969	1.26325212	587.358282	0.306414682
582.358282	4.52247813	582.358282	1.26152589	587.582817	0.306548738
582.374225	4.52233725	582.374225	1.26136143	588.14969	0.306588625
583.582817	4.49885815	583.582817	1.25329526	589.582817	0.305497516
584.14969	4.488596	584.14969	1.25120561	590	0.310151295
584.374225	4.48439791	584.374225	1.25041548	590.374225	0.309425287
585	4.47064365	585	1.247902	591.582817	0.30895409
585.374225	4.4596559	585.374225	1.24599759	592.074845	0.309029136
585.582817	4.45249349	585.582817	1.24488279	592.358282	0.309098658
586.14969	4.43592802	586.14969	1.24345444	593.582817	0.309486812
586.374225	4.43382863	586.374225	1.24402705	595	0.30993969
587.074845	4.43625451	587.074845	1.24669774	595.374225	0.310040011
587.358282	4.43653812	587.358282	1.24724393	595.582817	0.31008738
587.582817	4.43592277	587.582817	1.24741598	596.14969	0.310161146
588.14969	4.43079013	588.14969	1.24684439	597.074845	0.309893523
588.374225	4.42691029	588.374225	1.24608583	597.358282	0.309858258
588.582817	4.42156036	588.582817	1.24486371	597.582817	0.310034504
589.14969	4.34668275	589.14969	1.22673866	599.582817	0.311928334
589.582817	4.39558429	589.582817	1.24821087	600	0.312140232
590	4.44950046	590	1.26126052	600.14969	0.312214132
590.14969	4.4462544	590.14969	1.25915699	600.374225	0.312322168
590.374225	4.43996026	590.374225	1.25717773	601.582817	0.312881436
591.582817	4.42146863	591.582817	1.25451949	602.074845	0.313102142
592.074845	4.41690621	592.074845	1.25450893	602.358282	0.313228231
592.14969	4.41629143	592.14969	1.25452719	603.582817	0.313759231
592.358282	4.41460445	592.358282	1.25459338	604.14969	0.313995658
592.374225	4.41449063	592.374225	1.25460024	604.374225	0.314088046
593.582817	4.40580853	593.582817	1.25525048	605	0.314344747
594.14969	4.40187338	594.14969	1.25559044	605.582817	0.314592747
594.374225	4.40029056	594.374225	1.25572004	607.074845	0.315296674
595	4.39570647	595	1.25604526	607.358282	0.315438809
595.374225	4.39278242	595.374225	1.25619769	607.582817	0.315552802
595.582817	4.3910341	595.582817	1.25625851	609.582817	0.316562318
596.14969	4.38552094	596.374225	1.25619526	610	0.31677007
596.374225	4.38280067	597.074845	1.25568698	610.374225	0.316956741
597.074845	4.37092242	597.358282	1.25601968	611.582817	0.317553959
597.358282	4.36681219	597.582817	1.25684648	612.074845	0.317795201
597.582817	4.36632423	598.14969	1.25919568	612.14969	0.31783239
598.14969	4.37050743	598.374225	1.25979884	612.358282	0.317934127
598.374225	4.37150332	598.582817	1.26022535	613.582817	0.31853373
598.582817	4.37184524	599.14969	1.26104922	614.374225	0.318921108

Final State: j'=4		Final State: j'=3		Final State: j'=2	
E(cm⁻¹)	σ (Å²)	E(cm⁻¹)	σ (Å²)	E(cm⁻¹)	σ (Å²)
599.14969	4.37096368	599.582817	1.26154316	615	0.319227738
599.582817	4.36938172	600	1.26198316	615.374225	0.319412486
600	4.36752528	600.374225	1.26236794	615.582817	0.319515413
600.14969	4.36682939	601.582817	1.26359086	617.074845	0.320255828
600.374225	4.36574003	602.074845	1.26408501	617.358282	0.320397527
601.582817	4.35953632	602.14969	1.26416062	617.582817	0.320510482
602.074845	4.35690352	602.358282	1.26436883	619.582817	0.321519092
602.14969	4.35651145	602.374225	1.26438532	620	0.321730192
602.358282	4.35537255	603.582817	1.26557977	620.374225	0.32192087
602.374225	4.35529663	604.14969	1.26613429	621.582817	0.322536432
603.582817	4.34856546	604.374225	1.26635549	622.074845	0.322787201
604.14969	4.34528195	605	1.26698897	622.358282	0.322932115
604.374225	4.34396388	605.374225	1.26738995	623.582817	0.323559985
605	4.3402793	605.582817	1.26762224	624.14969	0.323851124
605.374225	4.33814309	606.14969	1.26828641	625	0.324288407
605.582817	4.33697982	606.374225	1.26856063	625.374225	0.324481843
606.14969	4.33397455	607.074845	1.26943802	625.582817	0.324589573
606.374225	4.3328484	607.358282	1.26979658	626.374225	0.324998747
607.074845	4.3295043	607.582817	1.27008092	627.074845	0.325361461
607.358282	4.32820631	608.14969	1.27079249	627.358282	0.325508643
607.582817	4.32719877	608.374225	1.27107248	627.582817	0.325625785
608.14969	4.32464549	608.582817	1.27133102	629.582817	0.326669232
608.374225	4.32363441	609.14969	1.27202803	630	0.326887376
608.582817	4.32269362	609.582817	1.27255609	630.374225	0.32708413
609.14969	4.32012483	610	1.27306042	631.582817	0.327719938
609.582817	4.31814716	610.14969	1.27324144	632.074845	0.327979109
610	4.31622133	610.374225	1.27351161	632.358282	0.328128831
610.14969	4.31554138	611.582817	1.27495757	633.582817	0.328777685
610.374225	4.31450324	612.074845	1.27554329	635	0.329530891
611.582817	4.30887288	612.14969	1.27563306	635.582817	0.32984215
612.074845	4.30655331	612.358282	1.27588076	636.14969	0.330145049
612.14969	4.30621189	613.582817	1.2773417	637.074845	0.330640158
612.358282	4.30521997	614.14969	1.27802113	637.358282	0.330792246
613.582817	4.29945948	614.374225	1.27829117	637.582817	0.330913178
614.14969	4.29679212	615	1.27904621	638.14969	0.331217914
614.374225	4.29573811	615.374225	1.27950051	639.582817	0.331990667
615	4.2928016	615.582817	1.27975433	640	0.332215877
615.374225	4.29107162	616.14969	1.28044679	640.14969	0.332297235
615.582817	4.29010489	616.374225	1.28072176	641.582817	0.333074568
616.14969	4.28749077	617.074845	1.28158363	642.074845	0.333341865
616.374225	4.28646107	617.358282	1.28193367	642.358282	0.333496225
617.074845	4.28325643	617.582817	1.2822119	642.374225	0.33350523
617.358282	4.28197278	618.14969	1.28291586	643.582817	0.334164837
617.582817	4.28096905	618.374225	1.28319381	644.14969	0.334475011
618.14969	4.27842057	618.582817	1.28345349	645	0.334940894

Final State: j'=4		Final State: j'=3		Final State: j'=2	
E(cm⁻¹)	σ (Å²)	E(cm⁻¹)	σ (Å²)	E(cm⁻¹)	σ (Å²)
618.374225	4.27741498	619.14969	1.28415992	645.582817	0.33526139
618.582817	4.27648314	619.582817	1.2847011	647.074845	0.33608325
619.14969	4.27395977	620	1.28522285	647.358282	0.336239784
619.582817	4.27203989	620.14969	1.28541053	647.582817	0.336364308
620	4.27018657	620.374225	1.28569186	649.582817	0.337473245
620.14969	4.26953692	621.582817	1.28720864	650	0.33770508
620.374225	4.26854872	622.074845	1.28782768	650.374225	0.337913787
621.582817	4.26325126	622.14969	1.28792207	651.582817	0.338588393
622.074845	4.26109544	622.358282	1.28818474	652.074845	0.338863425
622.14969	4.26077987	622.374225	1.28820495	652.14969	0.338905597
622.358282	4.25986224	623.582817	1.28973096	652.358282	0.339022176
622.374225	4.25980317	624.14969	1.29044871	653.582817	0.339709661
623.582817	4.25456733	624.374225	1.29073344	654.14969	0.340028571
624.14969	4.25212411	625	1.29152812	654.374225	0.340155019
624.374225	4.25115855	625.374225	1.29200404	655	0.340507658
625	4.24846444	625.582817	1.29226972	655.374225	0.340719163
625.374225	4.24687474	626.14969	1.29299258	655.582817	0.340837029
625.582817	4.2459845	626.374225	1.29327933	657.074845	0.341681778
626.14969	4.24357121	627.074845	1.29417546	657.358282	0.341842705
626.374225	4.24261798	627.358282	1.29453876	657.582817	0.34197047
627.074845	4.2396422	627.582817	1.29482669	658.582817	0.34253942
627.358282	4.23844642	628.14969	1.29555474	659.582817	0.343110004
627.582817	4.23751093	628.374225	1.29584358	660	0.343348231
628.14969	4.23513004	628.582817	1.29611211	660.14969	0.343434139
628.374225	4.23418959	629.14969	1.29684282	660.374225	0.343562644
628.582817	4.23331717	629.582817	1.29740206	661.582817	0.344255639
629.14969	4.23095267	630	1.2979416	662.074845	0.344538282
629.582817	4.22915226	630.14969	1.29813523	662.14969	0.344581487
630	4.22741309	630.374225	1.298426	662.358282	0.344701357
630.14969	4.22680388	631.582817	1.29999484	663.582817	0.345407439
630.374225	4.22587622	632.074845	1.30063541	664.14969	0.34573491
631.582817	4.22090688	632.14969	1.30073271	665	0.346227238
632.074845	4.21888522	632.358282	1.30100473	665.582817	0.346565465
632.14969	4.2185898	632.374225	1.30102533	667.074845	0.347433334
632.358282	4.21772936	633.582817	1.30260381	667.358282	0.347598623
632.374225	4.21767437	634.14969	1.30334606	667.582817	0.347729748
633.582817	4.21276972	634.374225	1.30364034	669.14969	0.348646298
634.14969	4.21048226	635	1.30446195	669.582817	0.348900441
634.374225	4.20957844	635.374225	1.30495355	670	0.349145391
635	4.20705639	635.582817	1.3052279	670.374225	0.349365495
635.374225	4.20556864	636.14969	1.30597434	671.582817	0.350077701
635.582817	4.20473534	636.374225	1.30627032	672.074845	0.350368327
636.14969	4.20247622	637.074845	1.30719536	672.358282	0.350535782
636.374225	4.20158358	637.358282	1.30756997	673.582817	0.351261714
637.074845	4.19879633	637.582817	1.30786659	674.14969	0.351598361

Final State: j'=4		Final State: j'=3		Final State: j'=2	
E(cm⁻¹)	σ (Å²)	E(cm⁻¹)	σ (Å²)	E(cm⁻¹)	σ (Å²)
637.358282	4.19767611	638.14969	1.30861703	675	0.352104755
637.582817	4.19679986	638.374225	1.30891458	675.374225	0.352327955
638.14969	4.19456828	638.582817	1.30919116	676.374225	0.352925667
638.374225	4.19368647	639.14969	1.30994365	677.074845	0.35334563
638.582817	4.19286838	639.582817	1.31051925	677.358282	0.353515716
639.14969	4.19065027	640	1.31107473	677.582817	0.353650602
639.582817	4.18896058	640.14969	1.31127361	679.14969	0.354594633
640	4.18732763	640.374225	1.3115727	680	0.355108855
640.14969	4.18675587	641.582817	1.31318551	680.374225	0.355335603
640.374225	4.18588467	642.074845	1.31384395	682.074845	0.356370005
641.582817	4.18121541	642.14969	1.31394363	682.358282	0.356542968
642.074845	4.17931454	642.358282	1.31422336	684.14969	0.357640659
642.14969	4.17903699	642.374225	1.31424419	685	0.358164275
642.358282	4.17822757	643.582817	1.31586487	686.374225	0.359014333
642.374225	4.17817612	644.14969	1.31662658	687.074845	0.359449951
643.582817	4.17356222	644.374225	1.31692858	687.358282	0.359626463
644.14969	4.17140936	645	1.31777169	688.374225	0.360261044
644.374225	4.1705587	645.374225	1.31827546	689.14969	0.360747923
645	4.16818408	645.582817	1.31855685	690	0.361283905
645.374225	4.16678339	646.14969	1.31932203	692.074845	0.362603213
645.582817	4.16599861	646.374225	1.31962536	692.358282	0.362784841
646.14969	4.16387089	647.074845	1.32057367	694.14969	0.363942109
646.374225	4.16303009	647.358282	1.32095743	695	0.364497853
647.074845	4.16040372	647.582817	1.32126098	697.074845	0.365876747
647.358282	4.15934813	648.14969	1.32202944	697.358282	0.366068228
647.582817	4.15852236	648.374225	1.32233417	699.14969	0.367302794
648.14969	4.15641912	648.582817	1.32261735	700	0.367908723
648.374225	4.15558765	649.14969	1.32338755	702.074845	0.369490678
648.582817	4.15481642	649.582817	1.32397667	702.358282	0.369726873
649.14969	4.15272493	650	1.3245454	704.14969	0.371585809
649.582817	4.15113145	650.14969	1.32474851	705	0.371055753
650	4.14959088	650.374225	1.32505448	706.374225	0.371870907
650.14969	4.14905175	651.582817	1.32670377	707.074845	0.370582441
650.374225	4.14822975	652.074845	1.32737726	707.358282	0.369625881
651.582817	4.14382328	652.358282	1.32776501	708.374225	0.37326161
652.074845	4.1420286	652.374225	1.32778595	709.14969	0.373266364
652.14969	4.14176667	653.582817	1.32944186	710	0.372600908
652.358282	4.14100231	654.14969	1.33021988	710.374225	0.371509076
652.374225	4.14095391	654.374225	1.33052829	712.074845	0.375701419
653.582817	4.13659616	655	1.33138956	712.358282	0.376376019
654.14969	4.13456224	655.374225	1.33190345	713.582817	0.374985443
654.374225	4.13375838	655.582817	1.33219064	714.14969	0.370935028
655	4.1315141	656.14969	1.33297166	714.374225	0.373797098
655.374225	4.13019018	656.374225	1.33328125	715	0.376184548
655.582817	4.12944832	657.074845	1.33424917	715.374225	0.376945498

Final State: j’=4		Final State: j’=3		Final State: j’=2	
E(cm⁻¹)	σ (Å²)	E(cm⁻¹)	σ (Å²)	E(cm⁻¹)	σ (Å²)
656.14969	4.12743657	657.358282	1.33464065	717.074845	0.375438287
656.374225	4.12664148	657.582817	1.33494994	717.358282	0.377338847
657.074845	4.12415759	658.14969	1.33573384	719.14969	0.378592439
657.358282	4.12315899	658.374225	1.33604456	720	0.379980738
657.582817	4.12237809	658.582817	1.33633331	722.074845	0.379725011
658.14969	4.12038803	659.14969	1.33711875	722.358282	0.381497609
658.374225	4.11960145	659.582817	1.33771934	724.14969	0.381870604
658.582817	4.11887163	660	1.33829952	725	0.38304619
659.14969	4.11689218	660.374225	1.33881814	727.074845	0.381446485
659.582817	4.11538386	661.582817	1.34049892	727.358282	0.382651589
660	4.11392561	662.074845	1.3411856	729.14969	0.384777237
660.14969	4.11341503	662.358282	1.34158069	730	0.386039797
660.374225	4.11263687	662.374225	1.34160159	732.074845	0.388788428
661.582817	4.10846431	663.582817	1.34328855	732.358282	0.389350215
662.074845	4.10676441	664.14969	1.34408241	734.14969	0.387545146
662.14969	4.10651641	664.374225	1.34439519	735	0.388917671
662.358282	4.10579234	665	1.34527275	737.074845	0.392260491
662.374225	4.10574645	665.374225	1.34579566	737.358282	0.3927189
663.582817	4.10161799	665.582817	1.34608813	739.14969	0.391410702
664.14969	4.09968192	666.374225	1.3471987	739.582817	0.395098673
664.374225	4.09892886	667.074845	1.34818473	740	0.392112422
665	4.09680183	667.358282	1.34858335	741.582817	0.393992203
665.374225	4.09554702	668.374225	1.35001231	742.074845	0.392142044
665.582817	4.09484377	668.582817	1.35030638	742.358282	0.389964903
666.374225	4.09218291	669.14969	1.35110763	744.14969	0.397861814
667.074845	4.08982797	669.582817	1.35171751	744.374225	0.394438379
667.358282	4.08888116	670	1.35230869	745	0.391065827
667.582817	4.08814073	670.374225	1.3528362	747.074845	0.402055206
668.374225	4.08550777	671.582817	1.35454785	747.358282	0.394175601
668.582817	4.08481554	672.074845	1.35524754	749.14969	0.397553677
669.14969	4.08292995	672.358282	1.3556497	750	0.40021538
669.582817	4.08150797	672.374225	1.3556706	752.074845	0.399273593
670	4.08012478	673.582817	1.35738875	752.358282	0.400116055
670.374225	4.07890263	674.14969	1.35819784	753.582817	0.403020359
671.582817	4.07494488	674.374225	1.35851612	754.14969	0.4048413
672.074845	4.07333263	675	1.35941073	755	0.397227543
672.358282	4.07241077	675.374225	1.35994314	757.074845	0.40454259
672.374225	4.07236709	676.374225	1.36137313	757.358282	0.401399621
673.582817	4.06845088	677.074845	1.36237901	758.582817	0.406123592
674.14969	4.0666149	677.358282	1.36278553	759.14969	0.404815904
674.374225	4.06590076	677.582817	1.36310573	760	0.403586558
675	4.06388348	678.374225	1.36424286	762.074845	0.408393455
675.374225	4.0626935	678.582817	1.36454282	762.358282	0.409966441
676.374225	4.05950376	679.14969	1.36536108	763.582817	0.410990528
677.074845	4.05727119	679.582817	1.36598328	764.14969	0.40490815

Final State: j’=4		Final State: j’=3		Final State: j’=2	
E(cm⁻¹)	σ (Å²)	E(cm⁻¹)	σ (Å²)	E(cm⁻¹)	σ (Å²)
677.358282	4.05637379	680	1.3665875	765	0.402034975
677.582817	4.05567166	680.374225	1.36712579	766.374225	0.409580473
678.374225	4.05317632	681.582817	1.36887514	767.074845	0.398800849
678.582817	4.05252045	682.074845	1.36959131	767.358282	0.408530081
679.14969	4.05073404	682.358282	1.37000303	767.582817	0.409842319
679.582817	4.04938699	682.374225	1.37002388	768.374225	0.413450918
680	4.04807708	683.582817	1.3717834	768.582817	0.409549031
680.374225	4.04691978	684.14969	1.37261315	769.14969	0.408662904
681.582817	4.04317371	684.374225	1.37293884	769.582817	0.409700482
682.074845	4.04164857	685	1.37385733	770	0.410524149
682.358282	4.04077658	685.374225	1.37440351	770.374225	0.411093788
682.374225	4.04073521	685.582817	1.37470965	771.582817	0.412519293
683.582817	4.03703393	686.374225	1.37587349	772.074845	0.413958444
684.14969	4.03529973	687.074845	1.37690957	772.358282	0.412443779
684.374225	4.03462535	687.358282	1.37732836	772.374225	0.412368192
685	4.03272178	687.582817	1.37765785	773.582817	0.41131022
685.374225	4.03159931	688.374225	1.3788314	774.14969	0.412565623
685.582817	4.03097065	688.582817	1.37914143	774.374225	0.412848767
686.374225	4.02859343	689.14969	1.37998844	775	0.41347612
687.074845	4.02649219	689.582817	1.3806323	775.374225	0.413796962
687.358282	4.02564815	690	1.38125949	776.374225	0.414592056
687.582817	4.02498819	690.374225	1.38181791	777.074845	0.415070466
688.374225	4.02264462	691.582817	1.38363964	777.358282	0.41520477
688.582817	4.02202925	692.074845	1.38438865	778.374225	0.415631142
689.14969	4.02035467	692.358282	1.38481963	778.582817	0.41576831
689.582817	4.01909326	692.374225	1.38484085	779.14969	0.416179088
690	4.01786819	693.582817	1.38669011	780	0.416872281
690.374225	4.0167868	694.14969	1.38756784	780.374225	0.417275523
691.582817	4.01329585	694.374225	1.38791245	782.074845	0.417803549
692.074845	4.01187904	695	1.38888971	782.358282	0.41802577
692.358282	4.01107023	695.374225	1.38947243	782.374225	0.418035809
692.374225	4.01103191	695.582817	1.38980031	783.582817	0.41885692
693.582817	4.00761176	696.374225	1.39105336	784.14969	0.419224106
694.14969	4.00601763	697.074845	1.39217983	784.374225	0.419367259
694.374225	4.00539914	697.358282	1.39263835	785	0.419772883
695	4.00365957	697.582817	1.39299992	785.374225	0.420012545
695.374225	4.00263802	698.374225	1.39430232	786.374225	0.42065858
695.582817	4.00206747	698.582817	1.3946502	787.074845	0.42111455
696.374225	3.99992156	699.14969	1.39561034	787.358282	0.421298789
697.074845	3.99804286	699.582817	1.39635031	788.374225	0.42194285
697.358282	3.99729389	700	1.39708316	788.582817	0.422059929
697.582817	3.99671117	700.374225	1.39774577	789.14969	0.422322757
698.374225	3.99466255	701.582817	1.40001143	790	0.422836534
698.582817	3.99413103	702.074845	1.40101168	790.374225	0.423081803
699.14969	3.99270141	702.358282	1.40161505	792.074845	0.424187575

Final State: j'=4		Final State: j'=3		Final State: j'=2	
E(cm⁻¹)	σ (Å²)	E(cm⁻¹)	σ (Å²)	E(cm⁻¹)	σ (Å²)
699.582817	3.99164387	702.374225	1.40164541	792.358282	0.424369303
700	3.99063571	703.582817	1.40468653	792.374225	0.424377439
700.374225	3.98976578	704.14969	1.40673524	793.582817	0.425151039
701.582817	3.98713754	704.374225	1.40782334	794.14969	0.425511849
702.074845	3.98619279	705	1.40656178	794.374225	0.425652646
702.358282	3.9857049	705.374225	1.40729011	795	0.42605222
702.374225	3.98568443	705.582817	1.40104158	795.374225	0.426287433
703.582817	3.98447615	706.374225	1.40691459	796.374225	0.426918799
704.14969	3.98517893	707.074845	1.40513556	797.074845	0.427359939
704.374225	3.98618227	707.358282	1.39919123	797.358282	0.427537066
705	3.98817454	707.582817	1.4031497	798.374225	0.428169921
705.374225	3.98893029	708.374225	1.40954368	798.582817	0.428302735
705.582817	3.98154835	708.582817	1.41141694	799.14969	0.428658512
706.374225	3.97650243	709.14969	1.41106358	800	0.429193863
707.074845	3.98074245	709.582817	1.40389122	800.374225	0.42942771
707.358282	3.96365784	710	1.40536712	802.074845	0.430504393
707.582817	3.96502444	710.374225	1.40555142	802.358282	0.430683758
708.374225	3.96812477	711.582817	1.4114725	802.374225	0.430691531
708.582817	3.97036109	712.074845	1.41570293	803.582817	0.431458267
709.14969	3.97359836	712.358282	1.41790066	804.14969	0.431816827
709.582817	3.96657454	713.582817	1.41089993	804.374225	0.431956529
710	3.95791867	714.14969	1.40476861	805	0.432354573
710.374225	3.95872327	715	1.41507489	805.374225	0.432588858
711.582817	3.95595372	715.374225	1.41729748	806.374225	0.433220952
712.074845	3.9610874	715.582817	1.41853002	807.074845	0.433666078
712.358282	3.96633688	716.374225	1.42494638	807.358282	0.433845184
713.582817	3.94591095	717.074845	1.40974829	808.374225	0.434484762
714.14969	3.9469627	717.358282	1.41771756	808.582817	0.434618999
714.374225	3.9372233	717.582817	1.41663948	809.14969	0.434977277
715	3.94371719	718.374225	1.41232863	810	0.435514812
715.374225	3.9454021	718.582817	1.41604808	810.374225	0.435748971
715.582817	3.94646429	719.14969	1.42010989	812.074845	0.436827163
716.374225	3.95825091	719.582817	1.42216692	812.358282	0.437006514
717.074845	3.92925164	720	1.4240771	813.582817	0.437781562
717.358282	3.93821542	720.374225	1.42598199	814.14969	0.438140449
717.582817	3.93699987	721.582817	1.41922743	814.374225	0.438280048
718.374225	3.92397859	722.074845	1.42152593	815	0.438678886
718.582817	3.92742224	722.358282	1.42792312	815.374225	0.43891329
719.14969	3.93052698	722.374225	1.42833691	816.374225	0.439546671
719.582817	3.93164221	723.582817	1.42445905	817.074845	0.439993165
720	3.93273288	724.14969	1.42732787	817.358282	0.440172751
720.374225	3.9342662	724.374225	1.42822855	818.374225	0.440813851
721.582817	3.93446114	725	1.43059807	818.582817	0.440948737
722.074845	3.92352784	725.374225	1.43205697	819.14969	0.441308033
722.358282	3.92995246	725.582817	1.43294915	820	0.441847058

Final State: j'=4		Final State: j'=3		Final State: j'=2	
E(cm⁻¹)	σ (Å²)	E(cm⁻¹)	σ (Å²)	E(cm⁻¹)	σ (Å²)
722.374225	3.93120866	726.374225	1.43792211	820.374225	0.442081573
723.582817	3.91507892	727.074845	1.42402249	822.074845	0.443162791
724.14969	3.91672222	727.358282	1.42840816	822.358282	0.443342597
724.374225	3.91706815	727.582817	1.43151957	822.374225	0.443349907
725	3.91785513	728.374225	1.42242349	823.582817	0.444119394
725.374225	3.91851118	728.582817	1.42762116	824.14969	0.444479177
725.582817	3.91905478	729.14969	1.43322396	824.374225	0.444618837
726.374225	3.92534566	729.582817	1.43520653	825	0.445018872
727.074845	3.91528964	730	1.4367588	825.374225	0.445253548
727.358282	3.90800826	730.374225	1.43801996	826.374225	0.445888432
727.582817	3.90940513	731.582817	1.44220039	827.074845	0.446336267
728.374225	3.89655308	732.074845	1.44442692	827.358282	0.446516291
728.582817	3.89551358	732.358282	1.4461651	828.374225	0.447158686
729.14969	3.90093769	732.374225	1.44627245	828.582817	0.447294182
729.582817	3.90199579	733.582817	1.4396966	829.14969	0.447654378
730	3.90245429	734.14969	1.43838699	830	0.448194764
730.374225	3.902703	734.374225	1.43742014	830.374225	0.448429592
731.582817	3.90388822	735	1.44238445	832.074845	0.449513875
732.074845	3.90557351	735.374225	1.44473759	832.358282	0.449694131
732.358282	3.90771524	735.582817	1.44574993	832.374225	0.449701177
732.374225	3.90788556	736.374225	1.44903395	833.582817	0.450473048
733.582817	3.89167607	737.074845	1.45195667	834.14969	0.450833713
734.14969	3.88983022	737.358282	1.45329801	834.374225	0.450973429
734.374225	3.89195769	737.582817	1.45448959	835	0.45137482
735	3.88612644	738.374225	1.45918267	835.374225	0.451609815
735.374225	3.88814521	738.582817	1.45629411	836.374225	0.452246374
735.582817	3.88878771	739.14969	1.45133702	837.074845	0.452695696
736.374225	3.8903285	739.582817	1.46096169	837.358282	0.452876209
737.074845	3.89175608	740	1.45101199	838.374225	0.45352
737.358282	3.89264935	740.374225	1.44180568	838.582817	0.453656164
737.582817	3.89363854	741.582817	1.45514805	839.14969	0.454017329
738.374225	3.90332926	742.074845	1.45224265	840	0.454559194
738.582817	3.89879562	742.358282	1.44488963	840.374225	0.454794362
739.14969	3.89403561	742.374225	1.44465019	842.074845	0.455881925
739.582817	3.90274962	743.582817	1.45856733	842.358282	0.45606268
740	3.90366917	744.14969	1.46691143	842.374225	0.456069438
740.374225	3.87476387	745	1.44505675	843.582817	0.456843767
741.582817	3.87790605	745.374225	1.45391357	844.14969	0.457205447
742.074845	3.88415694	745.582817	1.45667862	844.374225	0.457345247
742.358282	3.87191488	746.374225	1.46521018	845	0.457748082
742.374225	3.87065892	747.074845	1.47925612	845.374225	0.457983428
743.582817	3.87296006	747.358282	1.45298342	846.374225	0.45862181
744.14969	3.88982402	747.582817	1.44542827	847.074845	0.459072747
744.374225	3.88793226	748.374225	1.45144771	847.358282	0.459253756
745	3.85273513	748.582817	1.44483795	848.374225	0.459899112

Final State: j'=4		Final State: j'=3		Final State: j'=2	
E(cm⁻¹)	σ (Å²)	E(cm⁻¹)	σ (Å²)	E(cm⁻¹)	σ (Å²)
745.374225	3.85916854	749.14969	1.46008586	848.582817	0.460035991
745.582817	3.86168003	749.582817	1.46417658	849.14969	0.46039821
746.374225	3.8712351	750	1.46829783	850	0.46094165
747.074845	3.90267421	750.374225	1.47370272	850.374225	0.461177188
747.358282	3.88559559	751.582817	1.45656425	852.074845	0.462268298
747.582817	3.86412126	752.074845	1.46358611	852.358282	0.462449601
748.374225	3.84757677	752.358282	1.46600868	852.374225	0.46245605
748.582817	3.82825762	752.374225	1.46612225	853.582817	0.463233027
749.14969	3.84711646	753.582817	1.47451131	854.14969	0.46359579
749.582817	3.85175718	754.14969	1.4803507	854.374225	0.463735686
750	3.85686002	754.374225	1.4843572	855	0.464140073
750.374225	3.86593089	755	1.45883995	855.374225	0.464375819
751.582817	3.83374534	755.374225	1.45698355	856.374225	0.465016139
752.074845	3.84023383	755.582817	1.46674857	857.074845	0.465468803
752.358282	3.84235212	756.374225	1.46778941	857.358282	0.465650379
752.374225	3.84245808	757.074845	1.47766461	858.374225	0.466297405
753.582817	3.85017833	757.358282	1.47083405	858.582817	0.466435043
754.14969	3.85845865	757.582817	1.47564694	859.14969	0.466798405
754.374225	3.86649158	758.374225	1.47323949	860	0.467343541
755	3.85411561	758.582817	1.48173457	860.374225	0.467579506
755.374225	3.82918458	759.14969	1.4833111	862.074845	0.468674421
755.582817	3.83065703	759.582817	1.45224237	862.358282	0.468856295
756.374225	3.84495912	760	1.47150971	862.374225	0.468862428
757.074845	3.8394851	760.374225	1.47975813	863.582817	0.469642249
757.358282	3.83777519	761.582817	1.47776792	864.14969	0.470006197
757.582817	3.83482917	762.074845	1.48521619	864.374225	0.470146203
758.374225	3.83509852	762.358282	1.49028958	865	0.470552251
758.582817	3.84198661	762.374225	1.49064639	865.374225	0.470788415
759.14969	3.86982615	763.582817	1.49327908	866.374225	0.471430854
759.582817	3.80066271	764.14969	1.47421568	867.074845	0.471885344
760	3.81660287	764.374225	1.4798536	867.358282	0.472067535
760.374225	3.82642128	765	1.46429241	868.374225	0.472716351
761.582817	3.81984517	765.374225	1.47613237	868.582817	0.472854814
762.074845	3.82805337	765.582817	1.48614515	869.14969	0.47321939
762.358282	3.83519862	766.374225	1.48686484	870	0.473766373
762.374225	3.83578219	767.074845	1.46228301	870.374225	0.474002759
763.582817	3.86401108	767.358282	1.48373059	872.074845	0.47510176
764.14969	3.80870673	767.582817	1.48766761	872.358282	0.475284264
764.374225	3.81256017	768.374225	1.49943449	872.374225	0.475290053
765	3.79123644	768.582817	1.48942788	873.582817	0.476072922
765.374225	3.79869702	769.14969	1.48355084	874.14969	0.476438139
765.582817	3.8116508	769.582817	1.48638216	874.374225	0.476578255
766.374225	3.81255406	770	1.48862197	875	0.476986088
767.074845	3.81458416	770.374225	1.49017918	875.374225	0.477222704
767.358282	3.80665681	771.582817	1.49410423	876.374225	0.47786738

Final State: j'=4		Final State: j'=3		Final State: j'=2	
E(cm⁻¹)	σ (Å²)	E(cm⁻¹)	σ (Å²)	E(cm⁻¹)	σ (Å²)
767.582817	3.81135209	772.074845	1.49832485	877.074845	0.478323862
768.374225	3.83240655	772.358282	1.49098958	877.358282	0.478506687
768.582817	3.83857583	772.374225	1.48886566	878.374225	0.479157444
769.14969	3.80062653	773.582817	1.4886226	878.582817	0.479296774
769.582817	3.8025746	774.14969	1.49217831	879.14969	0.479662647
770	3.80400688	774.374225	1.49292399	880	0.480211601
770.374225	3.80502385	775	1.49449244	880.374225	0.480448456
771.582817	3.80808421	775.374225	1.49524586	882.074845	0.481551845
772.074845	3.81816896	776.374225	1.49706274	882.358282	0.481735011
772.358282	3.80554669	777.074845	1.498086	882.374225	0.481740438
772.374225	3.80135591	777.358282	1.49830258	883.582817	0.482526567
773.582817	3.79012096	778.374225	1.49884656	884.14969	0.482893133
774.14969	3.79237687	778.582817	1.49912181	884.374225	0.483033382
774.374225	3.79265781	779.14969	1.4999878	885	0.483443109
775	3.79265384	780	1.50153883	885.374225	0.483680237
775.374225	3.79239187	780.374225	1.5025144	886.374225	0.484327338
776.374225	3.79144132	782.074845	1.50280805	887.074845	0.484785956
777.074845	3.79067093	782.358282	1.50330307	887.358282	0.48496947
777.358282	3.79011945	782.374225	1.50331897	888.374225	0.485622287
778.374225	3.78676807	783.582817	1.50499196	888.582817	0.485762549
778.582817	3.78621511	784.14969	1.50571147	889.14969	0.486129823
779.14969	3.78505671	784.374225	1.50598479	890	0.486680902
780	3.78416308	785	1.50678125	890.374225	0.486918277
780.374225	3.78477537	785.374225	1.50724108	892.074845	0.488026415
782.074845	3.77622822	786.374225	1.5084941	892.358282	0.488210299
782.358282	3.7759509	787.074845	1.50938164	892.374225	0.488215307
782.374225	3.77593045	787.358282	1.5097361	893.582817	0.489005016
783.582817	3.77370555	788.374225	1.51091218	894.14969	0.489372995
784.14969	3.7724943	788.582817	1.51109409	894.374225	0.489513417
784.374225	3.77200738	789.14969	1.51142462	895	0.489925283
785	3.7706419	790	1.51241668	895.374225	0.490162927
785.374225	3.76982471	790.374225	1.5129023	896.374225	0.490812684
786.374225	3.76765293	792.074845	1.51505352	897.074845	0.491273607
787.074845	3.7661586	792.358282	1.51539922	897.358282	0.49145796
787.358282	3.76556635	792.374225	1.51540815	898.374225	0.492113078
788.374225	3.7634617	793.582817	1.51687658	898.582817	0.492254324
788.582817	3.762943	794.14969	1.51755399	899.14969	0.49262323
789.14969	3.76103961	794.374225	1.51781065	900	0.493176687
790	3.75868826	795	1.51856303	900.374225	0.493414641
790.374225	3.75785462	795.374225	1.51899305	902.074845	0.494528116
792.074845	3.75410784	796.374225	1.52015663	902.358282	0.494712848
792.358282	3.75347618	797.074845	1.52096977	902.374225	0.494717475
792.374225	3.75344011	797.358282	1.5212925	903.582817	0.495511231
793.582817	3.75073664	798.374225	1.52243957	904.14969	0.495880984
794.14969	3.74946574	798.582817	1.52268952	904.374225	0.496021616

Final State: j’=4		Final State: j’=3		Final State: j’=2	
E(cm⁻¹)	σ (Å²)	E(cm⁻¹)	σ (Å²)	E(cm⁻¹)	σ (Å²)
794.374225	3.74896144	799.14969	1.52334095	905	0.496435787
795	3.74755777	800	1.52432189	905.374225	0.496674194
795.374225	3.74671554	800.374225	1.52474299	906.374225	0.497327209
796.374225	3.74445176	802.074845	1.52671736	907.074845	0.49779094
797.074845	3.74284496	802.358282	1.52704364	907.358282	0.497976164
797.358282	3.74218984	802.374225	1.52705058	908.374225	0.498634389
798.374225	3.73983487	803.582817	1.5284503	908.582817	0.498776866
798.582817	3.73935418	804.14969	1.52909923	909.14969	0.499147797
799.14969	3.73804968	804.374225	1.52934418	910	0.499704353
800	3.73610632	805	1.53006972	910.374225	0.49994942
800.374225	3.73525394	805.374225	1.53048395	912.074845	0.501064114
802.074845	3.73140957	806.374225	1.53161894	912.358282	0.501250054
802.358282	3.73077093	807.074845	1.53242349	913.582817	0.502053994
802.374225	3.73073347	807.358282	1.53274365	914.14969	0.502426555
803.582817	3.72801747	808.374225	1.53387679	915	0.502985884
804.14969	3.72674489	808.582817	1.53412358	915.374225	0.503232257
804.374225	3.72623927	809.14969	1.53476078	917.074845	0.50435395
805	3.72483755	810	1.53571482	917.358282	0.504541302
805.374225	3.7239966	810.374225	1.53612176	918.582817	0.50535254
806.374225	3.72175546	812.074845	1.53803383	919.14969	0.505729333
807.074845	3.7201884	812.358282	1.53834961	920	0.50629684
807.358282	3.71955396	813.582817	1.53971092	920.374225	0.506547799
808.374225	3.71727946	814.14969	1.54033945	922.074845	0.507708609
808.582817	3.71681563	814.374225	1.54057543	922.358282	0.507909499
809.14969	3.71554987	815	1.54128023	923.582817	0.509040968
810	3.71365386	815.374225	1.5416807	924.14969	0.509607306
810.374225	3.712818	816.374225	1.54278254	925	0.509652012
812.074845	3.70904164	817.074845	1.54356542	925.374225	0.510010467
812.358282	3.70841317	817.358282	1.54387644	927.074845	0.5109779
813.582817	3.70570217	818.374225	1.54497588	927.358282	0.511228301
814.14969	3.70444938	818.582817	1.54521697	928.582817	0.512255622
814.374225	3.70395062	819.14969	1.5458358	929.14969	0.512661469
815	3.70257279	820	1.54676196	930	0.513079003
815.374225	3.7017448	820.374225	1.54715547	930.374225	0.513098639
816.374225	3.69954306	822.074845	1.54901161	932.074845	0.514238567
817.074845	3.69800607	822.358282	1.54931779	932.358282	0.514418027
817.358282	3.69738356	822.374225	1.54932148	933.582817	0.515115508
818.374225	3.69515143	823.582817	1.55063744	934.14969	0.515557293
818.582817	3.69469768	824.14969	1.55124664	935	0.516351477
819.14969	3.69345632	824.374225	1.55147389	935.374225	0.517319846
820	3.69159666	825	1.55215841	937.074845	0.518313076
820.374225	3.69077551	825.374225	1.55254503	937.358282	0.51752176
822.074845	3.68707095	826.374225	1.55361277	938.582817	0.518448022
822.358282	3.68645405	827.074845	1.55437285	939.14969	0.518891474
822.374225	3.68641549	827.358282	1.55467422	940	0.520011068

Final State: j’=4		Final State: j’=3		Final State: j’=2	
E(cm⁻¹)	σ (Å²)	E(cm⁻¹)	σ (Å²)	E(cm⁻¹)	σ (Å²)
823.582817	3.68379264	828.374225	1.55573796	940.374225	0.52074615
824.14969	3.68256253	828.582817	1.55597305	942.074845	0.520587776
824.374225	3.6820716	829.14969	1.55657257	942.358282	0.520812112
825	3.68071989	830	1.55746979	943.582817	0.521718108
825.374225	3.67990569	830.374225	1.55784945	944.14969	0.522137149
826.374225	3.67774378	832.074845	1.55964867	945	0.522836898
827.074845	3.67623589	832.358282	1.55994517	945.374225	0.523315414
827.358282	3.67562467	832.374225	1.55994719	947.074845	0.524513888
828.374225	3.67343197	833.582817	1.56122294	947.358282	0.524050802
828.582817	3.67298776	834.14969	1.56181272	948.582817	0.524979275
829.14969	3.67176905	834.374225	1.56203115	949.14969	0.525388321
830	3.66994341	835	1.5626953	950	0.526003255
830.374225	3.66913606	835.374225	1.56306795	950.374225	0.526280763
832.074845	3.6655006	836.374225	1.56410131	952.074845	0.5281374
832.358282	3.66489502	837.074845	1.56483841	952.358282	0.527685399
832.374225	3.66485597	837.358282	1.56513004	953.582817	0.528201861
833.582817	3.66228229	838.374225	1.56615766	954.14969	0.528676184
834.14969	3.66107477	838.582817	1.56638667	955	0.529319513
834.374225	3.66059161	839.14969	1.56696664	955.374225	0.529600888
835	3.65926589	840	1.56783454	957.074845	0.531192744
835.374225	3.65846534	840.374225	1.56820014	957.358282	0.531847215
836.374225	3.65634298	842.074845	1.56994176	958.582817	0.532785863
837.074845	3.65486391	842.358282	1.57022846	959.14969	0.53377769
837.358282	3.65426386	842.374225	1.57022878	960	0.533224986
838.374225	3.65211004	843.582817	1.5714639	960.374225	0.533029537
838.582817	3.65167521	844.14969	1.57203404	962.074845	0.533974664
839.14969	3.65047881	844.374225	1.57224354	962.358282	0.534254198
840	3.64868656	845	1.57288719	963.582817	0.537413648
840.374225	3.64789278	845.374225	1.57324572	964.14969	0.535258947
842.074845	3.64432523	846.374225	1.57424441	965	0.536022332
842.358282	3.64373073	847.074845	1.5749584	965.374225	0.53643922
842.374225	3.64369115	847.358282	1.57524016	967.074845	0.537158417
843.582817	3.64116598	848.374225	1.57623137	967.358282	0.538110374
844.14969	3.63998066	848.582817	1.57645431	968.582817	0.53828888
844.374225	3.63950513	849.14969	1.57701462	969.14969	0.538973512
845	3.63820502	850	1.57785296	970	0.54093078
845.374225	3.63741796	850.374225	1.57820438	970.374225	0.53919391
846.374225	3.63533467	852.074845	1.57988804	972.074845	0.540624601
847.074845	3.63388409	852.358282	1.58016487	972.358282	0.540875256
847.358282	3.63329511	852.374225	1.58016347	973.582817	0.544071077
848.374225	3.63117974	853.582817	1.58135769	974.14969	0.542691049
848.582817	3.63075417	854.14969	1.5819081	975	0.542762473
849.14969	3.62957986	854.374225	1.5821086	975.374225	0.542648216
850	3.62782073	855	1.58273162	977.074845	0.545804628
850.374225	3.62704035	855.374225	1.5830759	977.358282	0.544409855

Final State: j'=4		Final State: j'=3		Final State: j'=2	
E(cm⁻¹)	σ (Å²)	E(cm⁻¹)	σ (Å²)	E(cm⁻¹)	σ (Å²)
852.074845	3.62354004	856.374225	1.58403975	978.582817	0.544620303
852.358282	3.62295655	857.074845	1.58473049	979.14969	0.545237856
852.374225	3.62291644	857.358282	1.58500239	980	0.546056152
853.582817	3.62043933	858.374225	1.58595693	980.374225	0.545969766
854.14969	3.61927601	858.582817	1.58617383	982.074845	0.547503864
854.374225	3.61880801	859.14969	1.58671433	982.358282	0.548598974
855	3.61753333	860	1.587523	983.582817	0.549306399
855.374225	3.6167596	860.374225	1.58786016	984.14969	0.548245017
856.374225	3.61471504	862.074845	1.58948564	985	0.549345936
857.074845	3.61329273	862.358282	1.58975258	985.374225	0.549750508
857.358282	3.61271474	862.374225	1.58974938	987.074845	0.551341668
858.374225	3.61063751	863.582817	1.59090261	987.358282	0.552629239
858.582817	3.61022115	864.14969	1.59143322	988.582817	0.552152853
859.14969	3.60906874	864.374225	1.59162463	989.14969	0.552574797
860	3.60734247	865	1.59222703	990	0.553257927
860.374225	3.60657541	865.374225	1.592557	990.374225	0.553563958
862.074845	3.60314188	866.374225	1.5934859	992.074845	0.554966165
862.358282	3.60256935	867.074845	1.59415338	992.358282	0.554876864
862.374225	3.60252872	867.358282	1.59441536	993.582817	0.555665059
863.582817	3.60009937	868.374225	1.59533315	994.14969	0.55606962
864.14969	3.59895789	868.582817	1.59554399	995	0.55667555
864.374225	3.59849743	869.14969	1.59606468	995.374225	0.55694572
865	3.597248	870	1.59684363	997.074845	0.558155753
865.374225	3.59648756	870.374225	1.59716642	997.358282	0.5583406
866.374225	3.59448154	872.074845	1.59873368	998.582817	0.559169483
867.074845	3.59308735	872.358282	1.59899071	999.14969	0.559580977
867.358282	3.59252025	872.374225	1.59898571	1000	0.560176234
868.374225	3.59048103	873.582817	1.60009791	1000.37423	0.560326009
868.582817	3.59007382	874.14969	1.60060869	1002.07485	0.561527387
869.14969	3.58894324	874.374225	1.60079098	1002.35828	0.56172235
870	3.58724968	875	1.60137276	1003.58282	0.562561029
870.374225	3.58649587	875.374225	1.60168837	1004.14969	0.562948665
872.074845	3.58312889	876.374225	1.60258227	1005	0.563530459
872.358282	3.58256724	877.074845	1.60322653	1005.37423	0.563786652
872.374225	3.5825261	877.358282	1.60347859	1007.07485	0.564959207
873.582817	3.58014431	878.374225	1.60435956	1007.35828	0.565157724
874.14969	3.5790246	878.582817	1.60456438	1008.58282	0.565983211
874.374225	3.57857164	879.14969	1.60506524	1009.14969	0.566364133
875	3.57734736	880	1.60581442	1010	0.56694164
875.374225	3.57660017	880.374225	1.60612286	1010.37423	0.567196456
876.374225	3.57463253	882.074845	1.6076319	1012.07485	0.568356319
877.074845	3.5732663	882.358282	1.60787901	1012.35828	0.568549749
877.358282	3.5727101	882.374225	1.60787216	1013.58282	0.569385744
878.374225	3.57070872	883.582817	1.60894337	1014.14969	0.569772943
878.582817	3.5703106	884.14969	1.60943432	1015	0.570354011

Final State: j'=4		Final State: j'=3		Final State: j'=2	
E(cm⁻¹)	σ (Å²)	E(cm⁻¹)	σ (Å²)	E(cm⁻¹)	σ (Å²)
879.14969	3.56920174	884.374225	1.60960746	1015.37423	0.570609645
880	3.56754076	885	1.61016862	1017.07485	0.57177049
880.374225	3.56680019	885.374225	1.61046987	1017.35828	0.571963774
882.074845	3.56349935	886.374225	1.61132875	1018.58282	0.57279864
882.358282	3.56294853	887.074845	1.61194983	1019.14969	0.573185152
882.374225	3.5629069	887.358282	1.61219197	1020	0.573765096
883.582817	3.56057238	888.374225	1.61303607	1020.37423	0.574020254
884.14969	3.55947432	888.582817	1.61323491	1022.07485	0.575180364
884.374225	3.55902887	889.14969	1.61371593	1022.35828	0.575373743
885	3.55782956	890	1.61443537	1023.58282	0.576209276
885.374225	3.55709554	890.374225	1.61472939	1024.14969	0.576596092
886.374225	3.55516602	892.074845	1.61618022	1025	0.577176374
887.074845	3.55382757	892.358282	1.61641749	1025.37423	0.577431632
887.358282	3.55328216	892.374225	1.61640871	1027.07485	0.578591947
888.374225	3.55131837	893.582817	1.61743894	1027.35828	0.578785391
888.582817	3.55092926	894.14969	1.61790998	1028.58282	0.57962067
889.14969	3.54984196	894.374225	1.61807396	1029.14969	0.580007338
890	3.54821327	895	1.61861454	1030	0.580587412
890.374225	3.54748583	895.374225	1.61890134	1030.37423	0.580842566
892.074845	3.54425052	896.374225	1.6197252	1032.07485	0.582002384
892.358282	3.54371045	897.074845	1.62032305	1032.35828	0.582195737
892.374225	3.54366834	897.358282	1.62055528	1033.58282	0.583030668
893.582817	3.54138059	898.374225	1.62136241	1034.14969	0.583417177
894.14969	3.54030398	898.582817	1.62155526	1035	0.583997046
894.374225	3.53986592	899.14969	1.62201641	1035.37423	0.584252043
895	3.53869123	900	1.62270606	1037.07485	0.585411225
895.374225	3.53797026	900.374225	1.62298555	1037.35828	0.585604495
896.374225	3.53607838	902.074845	1.62437801	1038.58282	0.586438901
897.074845	3.53476731	902.358282	1.62460521	1039.14969	0.586825151
897.358282	3.53423249	902.374225	1.62459455	1040	0.587404535
898.374225	3.53230567	903.582817	1.62558363	1040.37423	0.587659376
898.582817	3.53192546	904.14969	1.62603474	1042.07485	0.588817637
899.14969	3.53085929	904.374225	1.62618946	1042.35828	0.589010717
900	3.5292623	905	1.62670932	1043.58282	0.589844402
900.374225	3.52854768	905.374225	1.6269815	1044.14969	0.590230191
902.074845	3.52537651	906.374225	1.62777003	1045	0.590809069
902.358282	3.52484689	907.074845	1.6283444	1045.37423	0.591063665
902.374225	3.5248043	907.358282	1.62856656	1047.07485	0.5922207
903.582817	3.52256208	908.374225	1.62933627	1047.35828	0.592413571
904.14969	3.5215062	908.582817	1.62952303	1048.58282	0.593246266
904.374225	3.52107527	909.14969	1.62996403	1049.14969	0.593631673
905	3.51992452	910	1.63062323	1050	0.594209776
905.374225	3.51921605	910.374225	1.63091241	1050.37423	0.594464027
906.374225	3.51736035	912.074845	1.63222044	1052.07485	0.595619511
907.074845	3.51607548	912.358282	1.63243738	1052.35828	0.595812127

Final State: j’=4		Final State: j’=3		Final State: j’=2	
E(cm⁻¹)	σ (Å²)	E(cm⁻¹)	σ (Å²)	E(cm⁻¹)	σ (Å²)
907.358282	3.51555084	913.582817	1.63337108	1053.58282	0.596643603
908.374225	3.51365909	914.14969	1.63380133	1054.14969	0.597028474
908.582817	3.51328729	915	1.63444429	1055	0.597605689
909.14969	3.51224107	915.374225	1.63472632	1055.37423	0.597859557
910	3.51067376	917.074845	1.63600052	1057.07485	0.599013148
910.374225	3.50998468	917.358282	1.63621163	1057.35828	0.599205499
912.074845	3.50685867	918.582817	1.6371192	1058.58282	0.600035563
912.358282	3.50633849	919.14969	1.63753668	1059.14969	0.600419729
913.582817	3.50409368	920	1.63815936	1060	0.600995923
914.14969	3.50305575	920.374225	1.63843187	1060.37423	0.601249323
915	3.50150029	922.074845	1.63965569	1062.07485	0.602400804
915.374225	3.5008162	922.358282	1.63985722	1062.35828	0.602592693
917.074845	3.49771004	923.582817	1.64094113	1063.58282	0.603421134
917.358282	3.49719259	924.14969	1.64190538	1064.14969	0.603804526
918.582817	3.49495684	925	1.64216172	1065	0.604379527
919.14969	3.49392108	925.374225	1.64235266	1065.37423	0.604632394
920	3.49236494	927.074845	1.64347349	1067.07485	0.605781356
920.374225	3.49167851	927.358282	1.64362294	1067.35828	0.605972849
922.074845	3.48852818	928.582817	1.64594118	1068.58282	0.606799393
922.358282	3.48799226	929.14969	1.64537829	1069.14969	0.607181904
923.582817	3.48554804	930	1.64680945	1070	0.607755519
924.14969	3.48485401	930.374225	1.64596828	1070.37423	0.608007807
925	3.48364836	932.074845	1.64777103	1072.07485	0.609153958
925.374225	3.48273459	932.358282	1.64754346	1072.35828	0.609344968
927.074845	3.47987153	933.582817	1.64825983	1073.58282	0.610169379
927.358282	3.47920668	934.14969	1.6485329	1074.14969	0.610550937
928.582817	3.47831371	935	1.64915087	1075	0.611122992
929.14969	3.47626624	935.374225	1.65042757	1075.37423	0.611374613
930	3.47585441	937.074845	1.65272264	1077.07485	0.612517569
930.374225	3.47423183	937.358282	1.65101977	1077.35828	0.612708065
932.074845	3.47196679	938.582817	1.65156821	1078.58282	0.613530134
932.358282	3.47093761	939.14969	1.65192741	1079.14969	0.613910524
933.582817	3.46881036	940	1.65294922	1080	0.614480957
934.14969	3.46756837	940.374225	1.65569535	1080.37423	0.614731782
935	3.46575051	942.074845	1.65418787	1082.07485	0.615871371
935.374225	3.46537441	942.358282	1.65423289	1082.35828	0.616061166
937.074845	3.46408798	943.582817	1.65491645	1083.58282	0.616880637
937.358282	3.462579	944.14969	1.65527028	1084.14969	0.617259806
938.582817	3.4599172	945	1.65584935	1085	0.617828394
939.14969	3.45879026	945.374225	1.65627717	1085.37423	0.618078381
940	3.45696799	947.074845	1.65899439	1087.07485	0.619214137
940.374225	3.45880763	947.358282	1.6578992	1087.35828	0.619403281
942.074845	3.45454551	948.582817	1.65818566	1088.58282	0.620219923
942.358282	3.45381425	949.14969	1.65852504	1089.14969	0.620597739
943.582817	3.45138447	950	1.65904769	1090	0.621164293

Final State: j’=4		Final State: j’=3		Final State: j’=2	
E(cm⁻¹)	σ (Å²)	E(cm⁻¹)	σ (Å²)	E(cm⁻¹)	σ (Å²)
944.14969	3.45030998	950.374225	1.65928216	1090.37423	0.621413386
945	3.44863434	952.074845	1.66293631	1092.07485	0.622544972
945.374225	3.44782424	952.358282	1.66104115	1092.35828	0.622733411
947.074845	3.44694894	953.582817	1.66147766	1093.58282	0.623546942
947.358282	3.4459672	954.14969	1.66167128	1094.14969	0.623923323
948.582817	3.44300213	955	1.66214412	1095	0.624487668
949.14969	3.44194881	955.374225	1.66235856	1095.37423	0.624735781
950	3.44038804	957.074845	1.6635019	1097.07485	0.625862844
950.374225	3.43969511	957.358282	1.66481866	1097.35828	0.62605052
952.074845	3.43929355	958.582817	1.66555738	1098.58282	0.626860708
952.358282	3.4370485	959.14969	1.6685425	1099.14969	0.627235525
953.582817	3.43502788	960	1.6664745	1100	0.627797489
954.14969	3.43366149	960.374225	1.66581012	1100.37423	0.628044549
955	3.43203025	962.074845	1.66689446	1102.07485	0.629166751
955.374225	3.43133228	962.358282	1.66694498	1102.35828	0.629353601
957.074845	3.42797698	963.582817	1.67183859	1103.58282	0.63016019
957.358282	3.42798192	964.14969	1.66851075	1104.14969	0.630533322
958.582817	3.42560892	965	1.66856917	1105	0.631092736
959.14969	3.42748249	965.374225	1.66875987	1105.37423	0.63133867
960	3.42464053	967.074845	1.67059864	1107.07485	0.632455643
960.374225	3.42344757	967.358282	1.67151927	1107.35828	0.632641612
962.074845	3.42072321	968.582817	1.67056067	1108.58282	0.633444358
962.358282	3.42002541	969.14969	1.67090627	1109.14969	0.633815694
963.582817	3.41903572	970	1.67701011	1110	0.634372386
964.14969	3.4177464	970.374225	1.67236926	1110.37423	0.634617096
965	3.41563164	972.074845	1.67258258	1112.07485	0.635728504
965.374225	3.41473428	972.358282	1.67272291	1112.35828	0.635913531
967.074845	3.41363452	973.582817	1.67724606	1113.58282	0.636712184
967.358282	3.4133777	974.14969	1.67733568	1114.14969	0.637081594
968.582817	3.41002391	975	1.67477957	1115	0.637635378
969.14969	3.40867346	975.374225	1.67461145	1115.37423	0.637878806
970	3.41173673	977.074845	1.67828913	1117.07485	0.638984291
970.374225	3.40836422	977.358282	1.67624258	1117.35828	0.639168318
972.074845	3.40434014	978.582817	1.67749502	1118.58282	0.639962613
972.358282	3.4037632	979.14969	1.67671786	1119.14969	0.640329985
973.582817	3.40313326	980	1.67937679	1120	0.640880695
974.14969	3.40501163	980.374225	1.67772501	1120.37423	0.641122755
975	3.40031193	982.074845	1.67885341	1122.07485	0.64222196
975.374225	3.40013656	982.358282	1.67986635	1122.35828	0.642404929
977.074845	3.39690654	983.582817	1.6793831	1123.58282	0.643194609
977.358282	3.39611975	984.14969	1.68035876	1124.14969	0.643559825
978.582817	3.39577866	985	1.67981602	1125	0.644107264
979.14969	3.39352764	985.374225	1.68042471	1125.37423	0.644347889
980	3.39369887	987.074845	1.68199725	1127.07485	0.645440457
980.374225	3.3918542	987.358282	1.68363394	1127.35828	0.645622306

Final State: j’=4		Final State: j’=3		Final State: j’=2	
E(cm⁻¹)	σ (Å²)	E(cm⁻¹)	σ (Å²)	E(cm⁻¹)	σ (Å²)
982.074845	3.38863996	988.582817	1.68314182	1128.58282	0.646407114
982.358282	3.38777836	989.14969	1.68339954	1129.14969	0.646770053
983.582817	3.3859636	990	1.68397404	1130	0.647314062
984.14969	3.38695799	990.374225	1.68425538	1130.37423	0.647553154
985	3.38407885	992.074845	1.68608384	1132.07485	0.648638716
985.374225	3.38358744	992.358282	1.68578562	1132.35828	0.648819389
987.074845	3.38083581	993.582817	1.68608014	1133.58282	0.649599059
987.358282	3.38050365	994.14969	1.68640213	1134.14969	0.649959601
988.582817	3.37940528	995	1.68689895	1135	0.650499974
989.14969	3.37837819	995.374225	1.68712845	1135.37423	0.650737489
990	3.37699658	997.074845	1.68822487	1137.07485	0.651815653
990.374225	3.37640243	997.358282	1.68836458	1137.35828	0.651995114
992.074845	3.37485299	998.582817	1.68900183	1138.58282	0.652769385
992.358282	3.37433506	999.14969	1.6893515	1139.14969	0.653127361
993.582817	3.37194213	1000	1.69002769	1140	0.653663928
994.14969	3.37105895	1000.37423	1.68991872	1140.37423	0.653899742
995	3.36976643	1002.07485	1.69085462	1142.07485	0.654970179
995.374225	3.36920964	1002.35828	1.69100956	1142.35828	0.655148275
997.074845	3.36685009	1003.58282	1.6916694	1143.58282	0.655916859
997.358282	3.36642639	1004.14969	1.69197224	1144.14969	0.656272209
998.582817	3.36456955	1005	1.69242591	1145	0.656804757
999.14969	3.36371545	1005.37423	1.69262551	1145.37423	0.657038767
1000	3.36297426	1007.07485	1.69355366	1147.07485	0.658101011
1000.37423	3.36212408	1007.35828	1.69371946	1147.35828	0.658277758
1002.07485	3.35951639	1008.58282	1.69434686	1148.58282	0.659040321
1002.35828	3.35910465	1009.14969	1.69462244	1149.14969	0.659392863
1003.58282	3.35732803	1010	1.6950523	1150	0.65992116
1004.14969	3.35650709	1010.37423	1.69524341	1150.37423	0.660153287
1005	3.35527975	1012.07485	1.69611469	1152.07485	0.661206861
1005.37423	3.35473938	1012.35828	1.69625976	1152.35828	0.661382134
1007.07485	3.35231973	1013.58282	1.69688567	1153.58282	0.662138297
1007.35828	3.35193484	1014.14969	1.69717505	1154.14969	0.662487842
1008.58282	3.35021917	1015	1.69760882	1155	0.663011589
1009.14969	3.34938714	1015.37423	1.69779915	1155.37423	0.663241693
1010	3.34816042	1017.07485	1.69865779	1157.07485	0.66428589
1010.37423	3.3476233	1017.35828	1.69879963	1157.35828	0.66445958
1012.07485	3.3452046	1018.58282	1.69940964	1158.58282	0.665208748
1012.35828	3.34480339	1019.14969	1.69969096	1159.14969	0.665554984
1013.58282	3.34307549	1020	1.70011205	1160	0.66607367
1014.14969	3.34227874	1020.37423	1.70029679	1160.37423	0.666301504
1015	3.341089	1022.07485	1.7011341	1162.07485	0.667334956
1015.37423	3.34056505	1022.35828	1.70127317	1162.35828	0.667506781
1017.07485	3.33819528	1023.58282	1.70187244	1163.58282	0.668247489
1017.35828	3.33780059	1024.14969	1.70214894	1164.14969	0.668589661
1018.58282	3.33609711	1025	1.70256267	1165	0.669101545

Final State: j’=4		Final State: j’=3		Final State: j’=2	
E(cm⁻¹)	σ (Å²)	E(cm⁻¹)	σ (Å²)	E(cm⁻¹)	σ (Å²)
1019.14969	3.33531003	1025.37423	1.70274411	1165.37423	0.669326213
1020	3.33413312	1027.07485	1.70356569	1167.07485	0.670342389
1020.37423	3.33361414	1027.35828	1.70370219	1167.35828	0.670510543
1022.07485	3.33126938	1028.58282	1.70428929	1168.58282	0.671228791
1022.35828	3.3308798	1029.14969	1.70456013	1169.14969	0.671551019
1023.58282	3.32920062	1030	1.70496538	1170	0.671970247
1024.14969	3.32842541	1030.37423	1.70514308	1170.37423	0.67223268
1025	3.32726656	1032.07485	1.705948	1172.07485	0.673218544
1025.37423	3.32675537	1032.35828	1.70608177	1172.35828	0.673661629
1027.07485	3.32444571	1033.58282	1.70665726	1173.58282	0.674232317
1027.35828	3.3240633	1034.14969	1.70692281	1174.14969	0.674599315
1028.58282	3.32240678	1035	1.70731979	1175	0.675181192
1029.14969	3.32164251	1035.37423	1.70749443	1175.37423	0.675307527
1030	3.32049992	1037.07485	1.70828381	1177.07485	0.676318366
1030.37423	3.31999572	1037.35828	1.70841501	1177.35828	0.676437949
1032.07485	3.31771816	1038.58282	1.70897944	1178.58282	0.677287045
1032.35828	3.3173412	1039.14969	1.70923987	1179.14969	0.677667827
1033.58282	3.31570792	1040	1.70962961	1180	0.678120595
1034.14969	3.3149545	1040.37423	1.70980052	1180.37423	0.678315128
1035	3.31382833	1042.07485	1.71057482	1182.07485	0.679451733
1035.37423	3.31333116	1042.35828	1.71070351	1182.35828	0.6795354
1037.07485	3.31108626	1043.58282	1.71125718	1183.58282	0.680291495
1037.35828	3.31071483	1044.14969	1.71151268	1184.14969	0.680566658
1038.58282	3.30910485	1045	1.71189504	1185	0.68102222
1039.14969	3.30836223	1045.37423	1.71206275	1185.37423	0.681220384
1040	3.30725229	1047.07485	1.7128225	1187.07485	0.682326249
1040.37423	3.3067622	1047.35828	1.71294879	1187.35828	0.682328145
1042.07485	3.30454944	1048.58282	1.71349215	1188.58282	0.683173244
1042.35828	3.30418346	1049.14969	1.71374292	1189.14969	0.683469208
1043.58282	3.30259645	1050	1.71411823	1190	0.683930348
1044.14969	3.30186448	1050.37423	1.71428282	1190.37423	0.684132893
1045	3.30077058	1052.07485	1.71502865	1192.07485	0.685164691
1045.37423	3.30028742	1052.35828	1.71515265	1192.35828	0.685352381
1047.07485	3.29810648	1053.58282	1.71568612	1193.58282	0.686132846
1047.35828	3.29774588	1054.14969	1.71593235	1194.14969	0.686360559
1048.58282	3.2961816	1055	1.71630089	1195	0.68681119
1049.14969	3.29546019	1055.37423	1.71646253	1195.37423	0.687013919
1050	3.29438218	1057.07485	1.71719501	1197.07485	0.687926369
1050.37423	3.29390587	1057.35828	1.71731677	1197.35828	0.68806895
1052.07485	3.29175641	1058.58282	1.71784076	1198.58282	0.688895952
1052.35828	3.29140112	1059.14969	1.71808264	1199.14969	0.689237262
1053.58282	3.28985933	1060	1.7184447	1200	0.689668118
1054.14969	3.28914833	1060.37423	1.7186035	1200.37423	0.689849462
1055	3.28808604	1062.07485	1.71932318	1202.07485	0.690732267
1055.37423	3.28761649	1062.35828	1.71944286	1202.35828	0.690876355

Final State: j'=4		Final State: j'=3		Final State: j'=2	
E(cm⁻¹)	σ (Å²)	E(cm⁻¹)	σ (Å²)	E(cm⁻¹)	σ (Å²)
1057.07485	3.28549813	1063.58282	1.71995781	1203.58282	0.691485361
1057.35828	3.28514812	1064.14969	1.72019553	1204.14969	0.691924456
1058.58282	3.2836285	1065	1.72055142	1205	0.692290564
1059.14969	3.2829278	1065.37423	1.72070749	1205.37423	0.692701023
1060	3.28188102	1067.07485	1.721415	1207.07485	0.693335057
1060.37423	3.28141815	1067.35828	1.72153267	1207.35828	0.693839842
1062.07485	3.2793305	1068.58282	1.722039	1208.58282	0.694411293
1062.35828	3.27898566	1069.14969	1.72227278	1209.14969	0.694564853
1063.58282	3.27748796	1070	1.72262278	1210	0.695526326
1064.14969	3.27679743	1070.37423	1.72277628	1210.37423	0.695536195
1065	3.27576594	1072.07485	1.72347219	1212.07485	0.696080854
1065.37423	3.27530965	1072.35828	1.72358793	1212.35828	0.696141177
1067.07485	3.27325226	1073.58282	1.72408606	1213.58282	0.697432758
1067.35828	3.27291258	1074.14969	1.72431608	1214.14969	0.69756885
1068.58282	3.27143645	1075	1.7246605	1215	0.697920648
1069.14969	3.27075595	1075.37423	1.72481153	1215.37423	0.698044957
1070	3.26973955	1077.07485	1.72549647	1217.07485	0.699178952
1070.37423	3.26928976	1077.35828	1.72561043	1217.35828	0.699297434
1072.07485	3.2672622	1078.58282	1.7261008	1218.58282	0.699850637
1072.35828	3.26692759	1079.14969	1.72632725	1219.14969	0.700080575
1073.58282	3.26547275	1080	1.72666635	1220	0.700814001
1074.14969	3.26480209	1080.37423	1.72681509	1220.37423	0.701194512
1075	3.26380057	1082.07485	1.72748978	1222.07485	0.701755481
1075.37423	3.26335717	1082.35828	1.72760188	1222.35828	0.701648708
1077.07485	3.26135902	1083.58282	1.72808492	1223.58282	0.702570515
1077.35828	3.26102939	1084.14969	1.72830801	1224.14969	0.702414921
1078.58282	3.25959551	1085	1.72864211	1225	0.703506712
1079.14969	3.25893458	1085.37423	1.72878868	1225.37423	0.703562475
1080	3.25794768	1087.07485	1.72945357	1227.07485	0.704418076
1080.37423	3.25751057	1087.35828	1.72956406	1227.35828	0.704493745
1082.07485	3.25554387	1088.58282	1.73004019	1228.58282	0.704969763
1082.35828	3.25521662	1089.14969	1.73026012	1229.14969	0.705888355
1083.58282	3.25380335	1090	1.73058953	1230	0.706489895
1084.14969	3.25315198	1090.37423	1.73073405	1230.37423	0.706458809
1085	3.25217951	1092.07485	1.73138975	1232.07485	0.706599135
1085.37423	3.25174857	1092.35828	1.73149871	1232.35828	0.707303199
1087.07485	3.24981042	1093.58282	1.73196838	1233.58282	0.707488525
1087.35828	3.24948789	1094.14969	1.73218536	1234.14969	0.708188778
1088.58282	3.2480949	1095	1.73251037	1235	0.708577983
1089.14969	3.24745291	1095.37423	1.73265296	1235.37423	0.708755349
1090	3.2464946	1097.07485	1.73330006	1237.07485	0.709565763
1090.37423	3.24606975	1097.35828	1.73340762	1237.35828	0.709821795
1092.07485	3.24415965	1098.58282	1.73387123	1238.58282	0.710464438
1092.35828	3.24384177	1099.14969	1.73408545	1239.14969	0.710719328
1093.58282	3.24246867	1100	1.73440646	1240	0.711136317

Final State: j'=4		Final State: j'=3		Final State: j'=2	
E(cm⁻¹)	σ (Å²)	E(cm⁻¹)	σ (Å²)	E(cm⁻¹)	σ (Å²)
1094.14969	3.24183592	1100.37423	1.73454716	1240.37423	0.711323036
1095	3.24089152	1102.07485	1.73518624	1242.07485	0.712176289
1095.37423	3.24047262	1102.35828	1.7352925	1242.35828	0.712320242
1097.07485	3.23859008	1103.58282	1.7357505	1243.58282	0.712948465
1097.35828	3.23827675	1104.14969	1.73596215	1244.14969	0.71323376
1098.58282	3.23692318	1105	1.73627925	1245	0.713657577
1099.14969	3.23629949	1105.37423	1.73641841	1245.37423	0.713841368
1100	3.2353687	1107.07485	1.73705006	1247.07485	0.714710426
1100.37423	3.23495568	1107.35828	1.73715509	1247.35828	0.714850143
1102.07485	3.23310015	1108.58282	1.7376079	1248.58282	0.715458335
1102.35828	3.23279129	1109.14969	1.73781718	1249.14969	0.715740146
1103.58282	3.23145688	1110	1.73813078	1250	0.716161936
1104.14969	3.23084206	1110.37423	1.73826838	1250.37423	0.716347125
1105	3.22992469	1112.07485	1.73889323	1252.07485	0.717186794
1105.37423	3.22951736	1112.35828	1.73899713	1252.35828	0.717326306
1107.07485	3.22768832	1113.58282	1.73944517	1253.58282	0.717928586
1107.35828	3.22738384	1114.14969	1.73965228	1254.14969	0.718209507
1108.58282	3.22606818	1115	1.73996265	1255	0.718626523
1109.14969	3.22546207	1115.37423	1.74009886	1255.37423	0.718808783
1110	3.22455779	1117.07485	1.74071745	1257.07485	0.719635913
1110.37423	3.22415609	1117.35828	1.74082033	1257.35828	0.719773432
1112.07485	3.22235295	1118.58282	1.74126402	1258.58282	0.720366227
1112.35828	3.22205277	1119.14969	1.74146915	1259.14969	0.720640131
1113.58282	3.22075547	1120	1.74177657	1260	0.721049979
1114.14969	3.22015786	1120.37423	1.74191152	1260.37423	0.721229924
1115	3.21926641	1122.07485	1.74252443	1262.07485	0.722046024
1115.37423	3.2188702	1122.35828	1.74262638	1262.35828	0.722181645
1117.07485	3.21709243	1123.58282	1.74306613	1263.58282	0.722766104
1117.35828	3.21679644	1124.14969	1.74326946	1264.14969	0.723036053
1118.58282	3.2155171	1125	1.74357424	1265	0.723439883
1119.14969	3.21492781	1125.37423	1.74370804	1265.37423	0.723617148
1120	3.21404889	1127.07485	1.74431585	1267.07485	0.724420763
1120.37423	3.21365804	1127.35828	1.74441697	1267.35828	0.724554272
1122.07485	3.21190507	1128.58282	1.74485317	1268.58282	0.72512961
1122.35828	3.21161317	1129.14969	1.74505489	1269.14969	0.725395386
1123.58282	3.21035134	1130	1.74535732	1270	0.725793016
1124.14969	3.20977019	1130.37423	1.74549009	1270.37423	0.72596756
1125	3.20890354	1132.07485	1.74609335	1272.07485	0.7267589
1125.37423	3.20851791	1132.35828	1.74619372	1272.35828	0.726890358
1127.07485	3.20678916	1133.58282	1.74662679	1273.58282	0.727456986
1127.35828	3.20650125	1134.14969	1.74682711	1274.14969	0.727718554
1128.58282	3.20525656	1135	1.74712744	1275	0.728110038
1129.14969	3.20468334	1135.37423	1.74725929	1275.37423	0.728281875
1130	3.20382865	1137.07485	1.74785856	1277.07485	0.729060882
1130.37423	3.20344813	1137.35828	1.7479583	1277.35828	0.729190289

Final State: j'=4		Final State: j'=3		Final State: j'=2	
E(cm⁻¹)	σ (Å²)	E(cm⁻¹)	σ (Å²)	E(cm⁻¹)	σ (Å²)
1132.07485	3.20174301	1138.58282	1.74838862	1278.58282	0.72974803
1132.35828	3.20145901	1139.14969	1.7485877	1279.14969	0.730005485
1133.58282	3.20023102	1140	1.74888617	1280	0.730390816
1134.14969	3.19966554	1140.37423	1.74901728	1280.37423	0.730559924
1135	3.19882252	1142.07485	1.74961305	1282.07485	0.73132662
1135.37423	3.19844697	1142.35828	1.74971222	1282.35828	0.731453975
1137.07485	3.19676491	1143.58282	1.75014018	1283.58282	0.732002863
1137.35828	3.19648471	1144.14969	1.75033817	1285	0.7326354
1138.58282	3.195273	1145	1.75063513	1285.37423	0.73280182
1139.14969	3.19471507	1145.37423	1.75076552	1287.07485	0.733556248
1140	3.19388342	1147.07485	1.75135835	1287.35828	0.733681563
1140.37423	3.19351274	1147.35828	1.75145703	1288.58282	0.734221623
1142.07485	3.19185313	1148.58282	1.75188298	1290	0.73484396
1142.35828	3.19157665	1149.14969	1.75208008	1290.37423	0.735007691
1143.58282	3.19038081	1150	1.75237571	1292.07485	0.735749901
1144.14969	3.18983025	1150.37423	1.75250553	1292.35828	0.735873178
1145	3.18900969	1152.07485	1.75309587	1293.58282	0.736404456
1145.37423	3.18864374	1152.35828	1.75319415	1295	0.737016645
1147.07485	3.18700605	1153.58282	1.7536184	1295.37423	0.737177694
1147.35828	3.18673319	1154.14969	1.75381474	1297.07485	0.737907752
1148.58282	3.18555285	1155	1.75410926	1297.35828	0.738029007
1149.14969	3.18500947	1155.37423	1.7542386	1298.58282	0.738551553
1150	3.18419974	1157.07485	1.75482682	1300	0.739153654
1150.37423	3.18383842	1157.35828	1.75492476	1300.37423	0.73931203
1152.07485	3.18222217	1158.58282	1.75534755	1302.07485	0.740030035
1152.35828	3.18195285	1159.14969	1.75554323	1302.35828	0.740149279
1153.58282	3.18078768	1160	1.75583676	1303.58282	0.740663161
1154.14969	3.18025134	1160.37423	1.75596566	1305	0.741255249
1155	3.17945226	1162.07485	1.75655187	1305.37423	0.741411
1155.37423	3.17909548	1162.35828	1.75664948	1307.07485	0.74211702
1157.07485	3.17750034	1163.58282	1.75707071	1307.35828	0.742234276
1157.35828	3.17723453	1164.14969	1.75726576	1308.58282	0.742739561
1158.58282	3.1760844	1165	1.75755785	1310	0.743321727
1159.14969	3.17555508	1165.37423	1.75768613	1310.37423	0.743474862
1160	3.17476664	1167.07485	1.75826848	1312.07485	0.744169017
1160.37423	3.17441444	1167.35828	1.75836516	1312.35828	0.7442843
1162.07485	3.17284075	1168.58282	1.75877969	1313.58282	0.744781074
1162.35828	3.17257856	1169.14969	1.75896711	1315	0.745353417
1163.58282	3.17144417	1170	1.75910003	1315.37423	0.745503964
1164.14969	3.17092647	1170.37423	1.75929886	1317.07485	0.746186385
1165	3.17014567	1172.07485	1.75975032	1317.35828	0.746299724
1165.37423	3.16979867	1172.35828	1.75998479	1318.58282	0.746788079
1167.07485	3.16825188	1173.58282	1.76049859	1320	0.747350714
1167.35828	3.16799489	1174.14969	1.76060087	1320.37423	0.747498701
1168.58282	3.16688978	1175	1.76094439	1322.07485	0.748169529

Final State: j’=4		Final State: j’=3		Final State: j’=2	
E(cm⁻¹)	σ (Å²)	E(cm⁻¹)	σ (Å²)	E(cm⁻¹)	σ (Å²)
1169.14969	3.16639604	1175.37423	1.76112489	1322.35828	0.748280927
1170	3.16578167	1177.07485	1.76168647	1323.58282	0.748760979
1170.37423	3.16536769	1177.35828	1.76174229	1325	0.749314028
1172.07485	3.16392418	1178.58282	1.7622328	1325.37423	0.749459492
1172.35828	3.16332553	1179.14969	1.76239367	1327.07485	0.750118902
1173.58282	3.1623498	1180	1.76273816	1327.35828	0.750228388
1174.14969	3.16184259	1180.37423	1.76285577	1328.58282	0.750700217
1175	3.16090053	1182.07485	1.76345128	1330	0.751243814
1175.37423	3.16076664	1182.35828	1.76357847	1330.37423	0.751386789
1177.07485	3.15919158	1183.58282	1.76400793	1332.07485	0.752034898
1177.35828	3.15897965	1184.14969	1.76418454	1332.35828	0.752142528
1178.58282	3.15777387	1185	1.76445648	1333.58282	0.752606298
1179.14969	3.15726241	1185.37423	1.76457247	1335	0.753140587
1180	3.15651068	1187.07485	1.765169	1335.37423	0.753281103
1180.37423	3.1561954	1187.35828	1.76514021	1337.07485	0.753918113
1182.07485	3.15459704	1188.58282	1.7657441	1337.35828	0.754023899
1182.35828	3.15443345	1189.14969	1.76592358	1338.58282	0.754479721
1183.58282	3.15327292	1190	1.76619956	1340	0.755004854
1184.14969	3.15282279	1190.37423	1.76631976	1340.37423	0.755142964
1185	3.15210728	1192.07485	1.76671778	1342.07485	0.755769071
1185.37423	3.15178986	1192.35828	1.76703761	1342.35828	0.755873041
1187.07485	3.15026377	1193.58282	1.76753321	1343.58282	0.75632106
1187.35828	3.15010436	1194.14969	1.76768498	1345	0.756837211
1188.58282	3.14890421	1195	1.7679578	1345.37423	0.756972944
1189.14969	3.14843933	1195.37423	1.76808056	1347.07485	0.757588344
1190	3.14772151	1197.07485	1.76862802	1347.35828	0.75769054
1190.37423	3.14740126	1197.35828	1.76870863	1348.58282	0.758130905
1192.07485	3.14592041	1198.58282	1.76920677	1350	0.758638236
1192.35828	3.14564671	1199.14969	1.76935916	1350.37423	0.758771658
1193.58282	3.14450157	1200	1.76974579	1352.07485	0.759376558
1194.14969	3.14408928	1200.37423	1.76985582	1352.35828	0.759477008
1195	3.14338209	1202.07485	1.77040927	1353.58282	0.759909873
1195.37423	3.1430629	1202.35828	1.77050186	1355	0.76040857
1197.07485	3.14164426	1203.58282	1.77072069	1355.37423	0.760539716
1197.35828	3.1414131	1204.14969	1.77076738	1357.07485	0.761134354
1198.58282	3.14034871	1205	1.77103074	1357.35828	0.761233098
1199.14969	3.13978985	1205.37423	1.77129794	1358.58282	0.761658625
1200	3.13908998	1207.07485	1.77183554	1360	0.762148885
1200.37423	3.13878751	1207.35828	1.77197127	1360.37423	0.762277811
1202.07485	3.13740414	1208.58282	1.77267612	1362.07485	0.762862402
1202.35828	3.13717685	1209.14969	1.77278758	1362.35828	0.762959489
1203.58282	3.13632304	1210	1.77312234	1363.58282	0.763377843
1204.14969	3.13579153	1210.37423	1.77330864	1364.14969	0.763570927
1205	3.13497752	1212.07485	1.7736826	1365	0.763859859
1205.37423	3.13477835	1212.35828	1.7731138	1365.37423	0.763986616

Final State: j’=4		Final State: j’=3		Final State: j’=2	
E(cm⁻¹)	σ (Å²)	E(cm⁻¹)	σ (Å²)	E(cm⁻¹)	σ (Å²)
1207.07485	3.13348645	1213.58282	1.77445349	1367.07485	0.764561422
1207.35828	3.13310953	1214.14969	1.77472023	1367.35828	0.76465688
1208.58282	3.13185356	1215	1.77495617	1368.58282	0.765068244
1209.14969	3.13152932	1215.37423	1.7750385	1369.14969	0.765258104
1210	3.13041659	1217.07485	1.7757439	1370	0.765542221
1210.37423	3.1302176	1217.35828	1.77583129	1370.37423	0.765666869
1212.07485	3.12911292	1218.58282	1.77620948	1372.07485	0.766232125
1212.35828	3.12899827	1219.14969	1.77635626	1372.35828	0.766326
1213.58282	3.12745612	1220	1.77629428	1373.58282	0.766730552
1214.14969	3.1271242	1220.37423	1.77698761	1374.14969	0.766917276
1215	3.12650593	1222.07485	1.77748932	1375	0.767196701
1215.37423	3.1262599	1222.35828	1.77739623	1375.37423	0.767319285
1217.07485	3.12465743	1223.58282	1.77791884	1377.07485	0.767875262
1217.35828	3.12445145	1224.14969	1.77770658	1377.35828	0.767967613
1218.58282	3.12352117	1225	1.7785978	1378.58282	0.768365539
1219.14969	3.12311675	1225.37423	1.77870467	1379.14969	0.768549215
1220	3.1221191	1227.07485	1.77925342	1380	0.76882409
1220.37423	3.12171461	1227.35828	1.77932714	1380.37423	0.768944676
1222.07485	3.12071876	1228.58282	1.77941577	1382.07485	0.769491608
1222.35828	3.12084564	1229.14969	1.78011473	1382.35828	0.769582514
1223.58282	3.1194035	1230	1.77953106	1383.58282	0.769974021
1224.14969	3.11953718	1230.37423	1.78054685	1384.14969	0.770154633
1225	3.1180902	1232.07485	1.78068224	1385	0.770425129
1225.37423	3.11789829	1232.35828	1.78115203	1385.37423	0.770543805
1227.07485	3.11652575	1233.58282	1.78111153	1387.07485	0.771082031
1227.35828	3.11635927	1234.14969	1.78179714	1387.35828	0.771171328
1228.58282	3.11536239	1235	1.78205333	1388.58282	0.771556741
1229.14969	3.114585	1235.37423	1.78217296	1389.14969	0.771734577
1230	3.11393365	1237.07485	1.78265617	1390	0.772000782
1230.37423	3.11362436	1237.35828	1.7830113	1390.37423	0.772117428
1232.07485	3.11289686	1238.58282	1.78338099	1392.07485	0.772647257
1232.35828	3.11222571	1239.14969	1.7835681	1392.35828	0.772735192
1233.58282	3.11145916	1240	1.78385523	1393.58282	0.773114436
1234.14969	3.11085459	1240.37423	1.78398535	1394.14969	0.773289532
1235	3.11018648	1242.07485	1.78458184	1395	0.773551542
1235.37423	3.10988774	1242.35828	1.78468225	1395.37423	0.773666505
1237.07485	3.10852538	1243.58282	1.78513963	1397.07485	0.774188046
1237.35828	3.10827964	1244.14969	1.78534826	1397.35828	0.774274679
1238.58282	3.10730342	1245	1.78565229	1398.58282	0.774648101
1239.14969	3.1068685	1245.37423	1.78577808	1399.14969	0.774820497
1240	3.10619306	1247.07485	1.78644873	1400	0.775078531
1240.37423	3.10588951	1247.35828	1.786549	1400.37423	0.775191737
1242.07485	3.10453654	1248.58282	1.78699511	1402.07485	0.775705384
1242.35828	3.10430905	1249.14969	1.78720434	1402.35828	0.775790707
1243.58282	3.10332487	1250	1.78751879	1403.58282	0.776158515

Final State: j'=4		Final State: j'=3		Final State: j'=2	
E(cm⁻¹)	σ (Å²)	E(cm⁻¹)	σ (Å²)	E(cm⁻¹)	σ (Å²)
1244.14969	3.10287961	1250.37423	1.78765717	1404.14969	0.776328331
1245	3.10220217	1252.07485	1.78828789	1405	0.776582516
1245.37423	3.10189583	1252.35828	1.78839298	1405.37423	0.77669403
1247.07485	3.10056656	1253.58282	1.78884735	1407.07485	0.777200069
1247.35828	3.10034105	1254.14969	1.78906461	1407.35828	0.777284138
1248.58282	3.09936475	1255	1.78938822	1408.58282	0.777646539
1249.14969	3.09892165	1255.37423	1.78952823	1409.14969	0.777813869
1250	3.0982498	1257.07485	1.79016587	1410	0.778064345
1250.37423	3.09794901	1257.35828	1.79027235	1410.37423	0.778174355
1252.07485	3.09661297	1258.58282	1.79073298	1412.07485	0.778672985
1252.35828	3.09638949	1259.14969	1.79094692	1412.35828	0.778755813
1253.58282	3.0954176	1260	1.79126794	1413.58282	0.779113052
1254.14969	3.09497772	1260.37423	1.79140919	1414.14969	0.779277991
1255	3.09431407	1262.07485	1.79205403	1415	0.779524911
1255.37423	3.09401569	1262.35828	1.79216174	1415.37423	0.779633396
1257.07485	3.0926888	1263.58282	1.79262776	1417.07485	0.780124968
1257.35828	3.09246689	1264.14969	1.79284411	1417.35828	0.780206668
1258.58282	3.09150346	1265	1.79316863	1418.58282	0.780558912
1259.14969	3.09106577	1265.37423	1.79331135	1419.14969	0.780721581
1260	3.09040152	1267.07485	1.79396225	1420	0.780965103
1260.37423	3.0901039	1267.35828	1.79407088	1420.37423	0.781072093
1262.07485	3.0887831	1268.58282	1.7945406	1422.07485	0.781556959
1262.35828	3.08856227	1269.14969	1.79475874	1422.35828	0.781637562
1263.58282	3.08760387	1270	1.79508604	1423.58282	0.781985054
1264.14969	3.08716864	1270.37423	1.79523003	1424.14969	0.782145544
1265	3.08650797	1272.07485	1.79588694	1425	0.782385815
1265.37423	3.08621185	1272.35828	1.79599659	1425.37423	0.782491394
1267.07485	3.08489783	1273.58282	1.796471	1427.07485	0.782969885
1267.35828	3.08467809	1274.14969	1.79669099	1427.35828	0.783049428
1268.58282	3.08372382	1275	1.79702139	1428.58282	0.783392416
1269.14969	3.08329047	1275.37423	1.79716674	1429.14969	0.783550837
1270	3.08263254	1277.07485	1.79782981	1430	0.78378804
1270.37423	3.08233757	1277.35828	1.79794048	1430.37423	0.783892277
1272.07485	3.08102912	1278.58282	1.79841929	1432.07485	0.784364745
1272.35828	3.0808103	1279.14969	1.79864132	1432.35828	0.784443299
1273.58282	3.07986551	1280	1.79897478	1433.58282	0.784782063
1274.14969	3.07942845	1280.37423	1.79912148	1434.14969	0.784938553
1275	3.07877321	1282.07485	1.79979072	1435	0.78517291
1275.37423	3.07847936	1282.35828	1.79990243	1435.37423	0.785275891
1277.07485	3.07717614	1283.58282	1.80038571	1437.07485	0.785742869
1277.35828	3.07695817	1285	1.80094641	1437.35828	0.785820533
1278.58282	3.07601693	1285.37423	1.80109447	1438.58282	0.786155569
1279.14969	3.07558147	1287.07485	1.80176997	1439.14969	0.786310417
1280	3.07492861	1287.35828	1.80188272	1440	0.786542453
1280.37423	3.07463574	1288.58282	1.80237051	1440.37423	0.786644505

Final State: j'=4		Final State: j'=3		Final State: j'=2	
E(cm⁻¹)	σ (Å²)	E(cm⁻¹)	σ (Å²)	E(cm⁻¹)	σ (Å²)
1282.07485	3.07333716	1290	1.80293644	1442.07485	0.787108533
1282.35828	3.07311992	1290.37423	1.80308587	1442.35828	0.787186197
1283.58282	3.07218186	1292.07485	1.80376766	1443.58282	0.787527694
1285	3.07109713	1292.35828	1.80388146	1444.14969	0.787670876
1285.37423	3.07080511	1293.58282	1.80437378	1445	0.787892326
1287.07485	3.0695107	1295	1.80494494	1445.37423	0.78800089
1287.35828	3.06929414	1295.37423	1.80509575	1447.07485	0.788448926
1288.58282	3.06835891	1297.07485	1.80578384	1447.35828	0.78853056
1290	3.06727735	1297.35828	1.80589868	1448.58282	0.788835255
1290.37423	3.0669861	1298.58282	1.80639553	1449.14969	0.788980447
1292.07485	3.06569537	1300	1.80697193	1450	0.789215341
1292.35828	3.06547939	1300.37423	1.80712412	1450.37423	0.789322167
1293.58282	3.06454665	1302.07485	1.80781849	1452.07485	0.789763067
1295	3.06346788	1302.35828	1.80793437	1452.35828	0.789851195
1295.37423	3.06317728	1303.58282	1.80843574	1453.58282	0.790164998
1297.07485	3.06188976	1305	1.80901734	1454.14969	0.790319308
1297.35828	3.06167429	1305.37423	1.80917089	1455	0.790555881
1298.58282	3.06074373	1307.07485	1.80987151	1455.37423	0.790643767
1300	3.05966736	1307.35828	1.80998844	1457.07485	0.791068751
1300.37423	3.05937731	1308.58282	1.81049428	1457.35828	0.791151038
1302.07485	3.05809258	1310	1.81108106	1458.58282	0.791479537
1302.35828	3.05787755	1310.37423	1.81123596	1459.14969	0.791630901
1303.58282	3.05694884	1312.07485	1.81194277	1460	0.791859436
1305	3.05587452	1312.35828	1.81206072	1460.37423	0.791928552
1305.37423	3.05558495	1313.58282	1.81257099	1462.07485	0.792378085
1307.07485	3.05430255	1315	1.81316287	1462.35828	0.79245428
1307.35828	3.05408791	1315.37423	1.81331911	1463.58282	0.792776489
1308.58282	3.05316076	1317.07485	1.81403202	1464.14969	0.792924943
1310	3.05208814	1317.35828	1.81415099	1465	0.793149864
1310.37423	3.05179894	1318.58282	1.81466562	1465.37423	0.793260489
1312.07485	3.05051854	1320	1.81526253	1467.07485	0.79364056
1312.35828	3.05030419	1320.37423	1.81542008	1467.35828	0.793739225
1313.58282	3.04937829	1322.07485	1.81613899	1468.58282	0.794061371
1315	3.04830706	1322.35828	1.81625895	1469.14969	0.794208005
1315.37423	3.04801815	1323.58282	1.81677786	1470	0.794427542
1317.07485	3.04673934	1325	1.8173797	1470.37423	0.794524228
1317.35828	3.04652522	1325.37423	1.81753853	1472.07485	0.794951709
1318.58282	3.04560033	1327.07485	1.8182633	1472.35828	0.795025967
1320	3.04453021	1327.35828	1.81838424	1473.58282	0.795335223
1320.37423	3.04424149	1328.58282	1.81890733	1474.14969	0.795483436
1322.07485	3.04296391	1330	1.81951397	1475	0.795702953
1322.35828	3.04274998	1330.37423	1.81967406	1475.37423	0.795799562
1323.58282	3.04182585	1332.07485	1.82040455	1477.07485	0.796259579
1325	3.04075653	1332.35828	1.82052642	1477.35828	0.79631374
1325.37423	3.04046793	1333.58282	1.82105359	1478.58282	0.796581979

Final State: j’=4		Final State: j’=3		Final State: j’=2	
E(cm⁻¹)	σ (Å²)	E(cm⁻¹)	σ (Å²)	E(cm⁻¹)	σ (Å²)
1327.07485	3.03919127	1335	1.8216649	1479.14969	0.796722441
1327.35828	3.03897748	1335.37423	1.8218262	1480	0.796949073
1328.58282	3.03805387	1337.07485	1.82256224	1480.37423	0.797070549
1330	3.03698507	1337.35828	1.82268503	1482.07485	0.797473208
1330.37423	3.03669653	1338.58282	1.82321613	1482.35828	0.797551878
1332.07485	3.03542047	1340	1.82383196	1483.58282	0.797795481
1332.35828	3.03520676	1340.37423	1.82399443	1484.14969	0.797977797
1333.58282	3.03428346	1342.07485	1.82473583	1485	0.798206508
1335	3.03321495	1342.35828	1.8248595	1485.37423	0.798308713
1335.37423	3.03292639	1343.58282	1.82539439	1487.07485	0.798691304
1337.07485	3.03165066	1345	1.82601457	1487.35828	0.798771852
1337.35828	3.03143697	1345.37423	1.82617816	1488.58282	0.799097557
1338.58282	3.03051378	1347.07485	1.82692469	1489.14969	0.799257249
1340	3.02944532	1347.35828	1.82704921	1490	0.799397498
1340.37423	3.0291567	1348.58282	1.82758774	1490.37423	0.799517162
1342.07485	3.02788102	1350	1.82821206	1492.07485	0.799959259
1342.35828	3.02766732	1350.37423	1.82837674	1492.35828	0.800032176
1343.58282	3.02674405	1352.07485	1.82912817	1493.58282	0.800298244
1345	3.02567545	1352.35828	1.82925351	1494.14969	0.80047091
1345.37423	3.02538669	1353.58282	1.82979549	1495	0.80065142
1347.07485	3.02411084	1355	1.83042377	1495.37423	0.800785641
1347.35828	3.02389708	1355.37423	1.83058946	1497.07485	0.80116256
1348.58282	3.02297357	1357.07485	1.83134555	1497.35828	0.801263952
1350	3.02190464	1357.35828	1.83147164	1498.58282	0.801530101
1350.37423	3.02161569	1358.58282	1.83201691	1499.14969	0.801699315
1352.07485	3.02033944	1360	1.83264892	1500	0.80186968
1352.35828	3.0201256	1360.37423	1.83281555	1500.37423	0.801979926
1353.58282	3.01920168	1362.07485	1.83357603	1502.07485	0.802402812
1355	3.01813226	1362.35828	1.83370285	1502.35828	0.802508748
1355.37423	3.01784307	1363.58282	1.83425118	1503.58282	0.802624616
1357.07485	3.01656625	1364.14969	1.83450526	1504.14969	0.802899584
1357.35828	3.01635229	1365	1.83488668	1505.37423	0.80321838
1358.58282	3.01542783	1365.37423	1.83505422	1507.07485	0.803628532
1360	3.01435776	1367.07485	1.83581879	1507.35828	0.803663489
1360.37423	3.01406829	1367.35828	1.83594627	1508.58282	0.803978193
1362.07485	3.01279072	1368.58282	1.83649746	1509.14969	0.804116331
1362.35828	3.01257661	1369.14969	1.83675285	1510.37423	0.804410773
1363.58282	3.01165152	1370	1.8371362	1512.07485	0.804815113
1364.14969	3.0112232	1370.37423	1.83730458	1512.35828	0.804886171
1365	3.01058065	1372.07485	1.83807292	1513.58282	0.805182478
1365.37423	3.01029087	1372.35828	1.83820103	1514.14969	0.805318609
1367.07485	3.00901241	1373.58282	1.83875485	1515.37423	0.805611947
1367.35828	3.00879814	1374.14969	1.83901144	1517.07485	0.806017802
1368.58282	3.00787229	1375	1.83939657	1517.35828	0.806085729
1369.14969	3.00744362	1375.37423	1.8395657	1518.58282	0.806378417

Final State: j’=4		Final State: j’=3		Final State: j’=2	
E(cm⁻¹)	σ (Å²)	E(cm⁻¹)	σ (Å²)	E(cm⁻¹)	σ (Å²)
1370	3.00680054	1377.07485	1.84033752	1519.14969	0.806512186
1370.37423	3.0065104	1377.35828	1.84046618	1520.37423	0.806807579
1372.07485	3.00523095	1378.58282	1.84102241	1522.07485	0.80721252
1372.35828	3.00501648	1379.14969	1.84128008	1522.35828	0.807280006
1373.58282	3.00408981	1380	1.84166683	1523.58282	0.807571415
1374.14969	3.00366074	1380.37423	1.84183664	1524.14969	0.807706229
1375	3.00301707	1382.07485	1.84261159	1525.37423	0.807997201
1375.37423	3.00272654	1382.35828	1.84274076	1527.07485	0.808400407
1377.07485	3.001446	1383.58282	1.84329915	1527.35828	0.808467506
1377.35828	3.00123133	1384.14969	1.84355778	1528.58282	0.808758397
1378.58282	3.00030376	1385	1.84394596	1529.14969	0.808892821
1379.14969	2.99987427	1385.37423	1.84411638	1530.37423	0.80918279
1380	2.99922996	1387.07485	1.84489411	1532.07485	0.809584954
1380.37423	2.99893903	1387.35828	1.8450237	1533.58282	0.809941105
1382.07485	2.99765731	1388.58282	1.84558401	1534.14969	0.810074877
1382.35828	2.99744243	1389.14969	1.84584351	1535.37423	0.810363667
1383.58282	2.99651392	1390	1.84623296	1537.07485	0.810764389
1384.14969	2.99608398	1390.37423	1.84640386	1538.58282	0.811119345
1385	2.99543902	1392.07485	1.84718402	1539.14969	0.811252684
1385.37423	2.99514765	1392.35828	1.84731401	1540.37423	0.811540527
1387.07485	2.99386472	1393.58282	1.84787592	1542.07485	0.811939889
1387.35828	2.9936496	1394.14969	1.84813617	1543.58282	0.812293636
1388.58282	2.99272014	1395	1.84852666	1544.14969	0.812426521
1389.14969	2.99228975	1395.37423	1.84869804	1545.37423	0.812713427
1390	2.99164408	1397.07485	1.84948022	1547.07485	0.813111513
1390.37423	2.99135224	1397.35828	1.84961054	1548.58282	0.81346418
1392.07485	2.99006811	1398.58282	1.8501738	1549.14969	0.813596644
1392.35828	2.98985276	1399.14969	1.85043465	1550.37423	0.813882712
1393.58282	2.98892226	1400	1.85082604	1552.07485	0.81427963
1394.14969	2.98849142	1400.37423	1.85099777	1553.58282	0.814631262
1395	2.98784506	1402.07485	1.85178158	1554.14969	0.814763367
1395.37423	2.98755278	1402.35828	1.85191217	1555.37423	0.815048607
1397.07485	2.98626738	1403.58282	1.8524765	1557.07485	0.815444409
1397.35828	2.98605181	1404.14969	1.85273783	1558.58282	0.81579508
1398.58282	2.9851203	1405	1.85312989	1559.14969	0.815926829
1399.14969	2.984689	1405.37423	1.85330189	1560.37423	0.816211299
1400	2.98404195	1407.07485	1.85408695	1562.07485	0.816606056
1400.37423	2.98374919	1407.35828	1.85421774	1563.58282	0.816955806
1402.07485	2.98246258	1408.58282	1.85478285	1564.14969	0.817087224
1402.35828	2.98224677	1409.14969	1.85504451	1565.37423	0.817370956
1403.58282	2.98131428	1410	1.85543705	1567.07485	0.817764676
1404.14969	2.98088251	1410.37423	1.85560983	1568.58282	0.81811362
1405	2.98023477	1412.07485	1.85639514	1569.14969	0.818244644
1405.37423	2.97994155	1412.35828	1.85652604	1570.37423	0.818527703
1407.07485	2.97865374	1413.58282	1.85709165	1572.07485	0.818920501

Final State: j'=4		Final State: j'=3		Final State: j'=2	
E(cm⁻¹)	σ (Å²)	E(cm⁻¹)	σ (Å²)	E(cm⁻¹)	σ (Å²)
1407.35828	2.9784377	1414.14969	1.85735351	1573.58282	0.819268511
1408.58282	2.97750424	1415	1.85774632	1574.14969	0.819399284
1409.14969	2.97707202	1415.37423	1.85791921	1575.37423	0.819681623
1410	2.97642361	1417.07485	1.85870492	1577.07485	0.820073474
1410.37423	2.9761382	1417.35828	1.85883587	1578.58282	0.820420664
1412.07485	2.97484097	1418.58282	1.85940166	1579.14969	0.820552776
1412.35828	2.9746247	1419.14969	1.85966358	1580.37423	0.820832773
1413.58282	2.9736903	1420	1.86005645	1582.07485	0.821223677
1414.14969	2.97325765	1420.37423	1.86022935	1583.58282	0.821570032
1415	2.9726086	1422.07485	1.86101503	1584.14969	0.821700168
1415.37423	2.97232291	1422.35828	1.86114597	1585.37423	0.821981147
1417.07485	2.97102439	1423.58282	1.86171163	1588.58282	0.822716614
1417.35828	2.97080792	1424.14969	1.86197346	1589.14969	0.822846426
1418.58282	2.96987262	1425	1.86236617	1590.37423	0.823126733
1419.14969	2.96943955	1425.37423	1.86253899	1593.58282	0.823860382
1420	2.96878988	1427.07485	1.8633242	1594.14969	0.823989874
1420.37423	2.96850393	1427.35828	1.86345504	1595.37423	0.824269464
1422.07485	2.96720416	1428.58282	1.86402024	1598.58282	0.825001233
1422.35828	2.96698752	1429.14969	1.86428183	1599.14969	0.825130394
1423.58282	2.96605133	1430	1.86467414	1600.37423	0.825409254
1424.14969	2.96561787	1430.37423	1.86484677	1603.58282	0.826139062
1425	2.9649676	1432.07485	1.86563102	1604.14969	0.826267867
1425.37423	2.96468138	1432.35828	1.86576169	1605.37423	0.826545967
1427.07485	2.96338044	1433.58282	1.86632606	1608.58282	0.82727372
1427.35828	2.96316357	1434.14969	1.86658723	1609.14969	0.827402158
1428.58282	2.96222653	1435	1.86697887	1610.37423	0.827679441
1429.14969	2.96179266	1435.37423	1.86715118	1613.58282	0.828405029
1430	2.96114178	1437.07485	1.86793384	1614.14969	0.828533072
1430.37423	2.96085529	1437.35828	1.8680642	1615.37423	0.828809493
1432.07485	2.95955309	1438.58282	1.86862716	1618.58282	0.829532776
1432.35828	2.95933602	1439.14969	1.86888759	1619.14969	0.829660395
1433.58282	2.95839799	1440	1.86927796	1620.37423	0.829935908
1434.14969	2.95796367	1440.37423	1.86944964	1623.58282	0.83065672
1435	2.95731205	1442.07485	1.87022808	1624.14969	0.830783894
1435.37423	2.95702522	1442.35828	1.87035728	1625.37423	0.831058434
1437.07485	2.95572126	1443.58282	1.87093861	1628.58282	0.831776601
1437.35828	2.95550384	1444.14969	1.87123446	1629.14969	0.831903302
1438.58282	2.95456415	1445	1.87158047	1630.37423	0.832176795
1439.14969	2.95412888	1445.37423	1.87174185	1633.58282	0.832892134
1440	2.95347548	1447.07485	1.87252641	1634.14969	0.833018317
1440.37423	2.95318767	1447.35828	1.872651	1635.37423	0.833290692
1442.07485	2.95187567	1448.58282	1.87324088	1638.58282	0.834003
1442.35828	2.95165553	1449.14969	1.87354274	1639.14969	0.834128636
1443.58282	2.95064608	1450	1.87387956	1640.37423	0.834399801
1444.14969	2.95020478	1450.37423	1.87404478	1643.58282	0.835108861

Final State: j’=4		Final State: j’=3		Final State: j’=2	
E(cm⁻¹)	σ (Å²)	E(cm⁻¹)	σ (Å²)	E(cm⁻¹)	σ (Å²)
1445	2.94964436	1452.07485	1.87483231	1644.14969	0.835233901
1445.37423	2.94932638	1452.35828	1.87495756	1645.37423	0.835503789
1447.07485	2.94805106	1453.58282	1.8755134	1648.58282	0.836209365
1447.35828	2.9478161	1454.14969	1.87576818	1649.14969	0.836333782
1448.58282	2.94694571	1455	1.876154	1650.37423	0.836602281
1449.14969	2.94646708	1455.37423	1.87637976	1653.58282	0.837304135
1450	2.94584383	1457.07485	1.87711481	1654.14969	0.837427874
1450.37423	2.9455379	1457.35828	1.87723495	1655.37423	0.837694902
1452.07485	2.94426048	1458.58282	1.87778549	1658.58282	0.838392783
1452.35828	2.9440115	1459.14969	1.87804162	1659.14969	0.838515803
1453.58282	2.94309726	1460	1.87847522	1660.37423	0.838781264
1454.14969	2.94264773	1460.37423	1.87859427	1663.58282	0.839474893
1455	2.94195265	1462.07485	1.87937228	1664.14969	0.839597148
1455.37423	2.94163004	1462.35828	1.87949884	1665.37423	0.839860925
1457.07485	2.94045995	1463.58282	1.88005014	1668.58282	0.840549948
1457.35828	2.940221	1464.14969	1.88030582	1670.37423	0.840933493
1458.58282	2.93925593	1465	1.88068998	1673.58282	0.841617831
1459.14969	2.93880919	1465.37423	1.88085926	1675.37423	0.841998514
1460	2.93808271	1467.07485	1.88164564	1678.58282	0.842677787
1460.37423	2.93793069	1467.35828	1.88175413	1680.37423	0.843055565
1462.07485	2.93661151	1468.58282	1.8823018	1683.58282	0.843729473
1462.35828	2.93638577	1469.14969	1.88255615	1685.37423	0.844104176
1463.58282	2.93542952	1470	1.88293766	1688.58282	0.844772437
1464.14969	2.93498773	1470.37423	1.88310561	1690.37423	0.845143905
1465	2.93431442	1472.07485	1.88385273	1693.58282	0.845806223
1465.37423	2.93397571	1472.35828	1.88401276	1695.37423	0.846174293
1467.07485	2.93284942	1473.58282	1.88453665	1698.58282	0.846830372
1467.35828	2.93257018	1474.14969	1.88478857	1700.37423	0.847194876
1468.58282	2.93160522	1475	1.88516585	1703.58282	0.847844431
1469.14969	2.93116551	1475.37423	1.88533133	1705.37423	0.848205205
1470	2.93050615	1477.07485	1.88608324	1708.58282	0.848847937
1470.37423	2.93021513	1477.35828	1.88621668	1710.37423	0.849204823
1472.07485	2.92893486	1478.58282	1.88680475	1713.58282	0.849840447
1472.35828	2.92868309	1479.14969	1.88707259	1715.37423	0.850193279
1473.58282	2.92778478	1480	1.88739703	1718.58282	0.850821502
1474.14969	2.92733634	1480.37423	1.88755217	1720.37423	0.85117013
1475	2.92667284	1482.07485	1.8883283	1723.58282	0.851790669
1475.37423	2.92638092	1482.35828	1.88844598	1725.37423	0.852134925
1477.07485	2.92497139	1483.58282	1.88907462	1728.58282	0.852747502
1477.35828	2.92481154	1484.14969	1.88926259	1730.37423	0.853087224
1478.58282	2.92395677	1485	1.88962105	1733.58282	0.853691533
1479.14969	2.92349755	1485.37423	1.88978073	1735.37423	0.854026554
1480	2.92288567	1487.07485	1.89056279	1738.58282	0.854622242
1480.37423	2.92252336	1487.35828	1.8906595	1740.37423	0.854952279
1482.07485	2.92128	1488.58282	1.89118674	1743.58282	0.855537738

Final State: j'=4		Final State: j'=3		Final State: j'=2	
E(cm⁻¹)	σ (Å²)	E(cm⁻¹)	σ (Å²)	E(cm⁻¹)	σ (Å²)
1482.35828	2.92104238	1489.14969	1.89142596	1745.37423	0.855870406
1483.58282	2.92025313	1490	1.89185436	1748.58282	0.856436419
1484.14969	2.91972905	1490.37423	1.89198838	1750.37423	0.85676459
1485	2.91903123	1492.07485	1.89271347	1753.58282	0.857339981
1485.37423	2.91871574	1492.35828	1.89283556	1755.37423	0.857649304
1487.07485	2.91747769	1493.58282	1.89344065	1758.58282	0.858210934
1487.35828	2.91730948	1494.14969	1.89360881	1760.37423	0.858518587
1488.58282	2.91630337	1495	1.89403348	1763.58282	0.859070564
1489.14969	2.91581015	1495.37423	1.89410968	1765.37423	0.859373765
1490	2.91537157	1497.07485	1.89494291	1768.58282	0.859915531
1490.37423	2.91501205	1497.35828	1.89501538	1770.37423	0.860213083
1492.07485	2.91364587	1498.58282	1.8955587	1773.58282	0.860741695
1492.35828	2.9134195	1499.14969	1.89576873	1775.37423	0.861035749
1493.58282	2.91264191	1500	1.89618915	1780.37423	0.861832292
1494.14969	2.91210365	1500.37423	1.89632317	1785.37423	0.862639694
1495	2.91149369	1502.07485	1.89705776	1790.37423	0.86340181
1495.37423	2.91112272	1502.35828	1.8971161	1795.37423	0.864169071
1497.07485	2.90987156	1503.58282	1.8980124	1800.37423	0.864904298
1497.35828	2.90958806	1504.14969	1.89792622	1805.37423	0.865648355
1498.58282	2.90876877	1505.37423	1.89845137	1810.37423	0.866348826
1499.14969	2.9082568	1507.07485	1.89920001	1815.37423	0.867040909
1500	2.9076998	1507.35828	1.89934963	1820.37423	0.867716643
1500.37423	2.90736732	1508.58282	1.89983337	1825.37423	0.868374481
1502.07485	2.90602016	1509.14969	1.90007449	1830.37423	0.869015658
1502.35828	2.90569581	1510.37423	1.90059716	1835.37423	0.869639906
1503.58282	2.90514769	1512.07485	1.90130611	1840.37423	0.870247116
1504.14969	2.90451694	1512.35828	1.90142605	1845.37423	0.870837422
1505.37423	2.90350108	1513.58282	1.90194356	1850.37423	0.871410877
1507.07485	2.90219034	1514.14969	1.90218199	1855.37423	0.871967588
1507.35828	2.90205699	1515.37423	1.90269588	1860.37423	0.872507671
1508.58282	2.90111575	1517.07485	1.90340528	1865.37423	0.873031268
1509.14969	2.90067978	1517.35828	1.9035233	1870.37423	0.873538534
1510.37423	2.89974595	1518.58282	1.90403309	1875.37423	0.874029657
1512.07485	2.8984897	1519.14969	1.90426921	1880.37423	0.874504816
1512.35828	2.89826846	1520.37423	1.90477335	1885.37423	0.874964232
1513.58282	2.89733329	1522.07485	1.90547683	1890.37423	0.87540812
1514.14969	2.89690415	1522.35828	1.90559366	1895.37423	0.875836727
1515.37423	2.89597886	1523.58282	1.9060975	1900.37423	0.876250304
1517.07485	2.89470022	1524.14969	1.90633027	1905.37423	0.876649106
1517.35828	2.89448666	1525.37423	1.90683208	1910.37423	0.877033414
1518.58282	2.89356367	1527.07485	1.90752629		
1519.14969	2.89313672	1527.35828	1.90764144		
1520.37423	2.89221778	1528.58282	1.90813938		
1522.07485	2.89093842	1529.14969	1.90836969		
1522.35828	2.89072552	1530.37423	1.90886592		

Final State: j’=4		Final State: j’=3		Final State: j’=2	
E(cm⁻¹)	σ (Å²)	E(cm⁻¹)	σ (Å²)	E(cm⁻¹)	σ (Å²)
1523.58282	2.88980612	1532.07485	1.90955256		
1524.14969	2.88938074	1533.58282	1.91015903		
1525.37423	2.88846238	1534.14969	1.91038643		
1527.07485	2.88718848	1535.37423	1.91087655		
1527.35828	2.88697645	1537.07485	1.9115548		
1528.58282	2.88606008	1538.58282	1.91215394		
1529.14969	2.88563591	1539.14969	1.91237859		
1530.37423	2.8847204	1540.37423	1.91286279		
1532.07485	2.88345036	1542.07485	1.91353275		
1533.58282	2.8823255	1543.58282	1.91412438		
1534.14969	2.88190298	1544.14969	1.91434619		
1535.37423	2.88099085	1545.37423	1.91482423		
1537.07485	2.87972545	1547.07485	1.9154856		
1538.58282	2.8786047	1548.58282	1.91606958		
1539.14969	2.87818373	1549.14969	1.91628852		
1540.37423	2.87727498	1550.37423	1.91676032		
1542.07485	2.87601439	1552.07485	1.91741298		
1543.58282	2.87489804	1553.58282	1.91798921		
1544.14969	2.87447873	1554.14969	1.9182052		
1545.37423	2.87357364	1555.37423	1.91867065		
1547.07485	2.8723182	1557.07485	1.91931446		
1548.58282	2.87120649	1558.58282	1.91988279		
1549.14969	2.87078894	1559.14969	1.92009582		
1550.37423	2.86988772	1560.37423	1.92055484		
1552.07485	2.8686377	1562.07485	1.92118968		
1553.58282	2.86753089	1563.58282	1.92175004		
1554.14969	2.86711522	1564.14969	1.92196007		
1555.37423	2.86621805	1565.37423	1.92241258		
1557.07485	2.86497375	1567.07485	1.92303837		
1558.58282	2.8638721	1568.58282	1.92359069		
1559.14969	2.86345838	1569.14969	1.92379767		
1560.37423	2.86256547	1570.37423	1.92424363		
1562.07485	2.86132719	1572.07485	1.9248603		
1563.58282	2.86023094	1573.58282	1.92540451		
1564.14969	2.85981928	1574.14969	1.92560845		
1565.37423	2.85893083	1575.37423	1.92604781		
1567.07485	2.85769882	1577.07485	1.92665531		
1568.58282	2.85660822	1578.58282	1.92719138		
1569.14969	2.85619869	1579.14969	1.92739108		
1570.37423	2.8553149	1580.37423	1.927825		
1572.07485	2.85408944	1582.07485	1.9284233		
1573.58282	2.85300471	1583.58282	1.9289512		
1574.14969	2.85259742	1584.14969	1.92914901		
1575.37423	2.85171846	1585.37423	1.92957513		
1577.07485	2.85049982	1588.58282	1.93068396		

Final State: j’=4		Final State: j’=3		Final State: j’=2	
E(cm⁻¹)	σ (Å²)	E(cm⁻¹)	σ (Å²)	E(cm⁻¹)	σ (Å²)
1578.58282	2.84942122	1589.14969	1.93087871		
1579.14969	2.84902148	1590.37423	1.93129821		
1580.37423	2.84814232	1593.58282	1.93238972		
1582.07485	2.84693072	1594.14969	1.93258141		
1583.58282	2.84585844	1595.37423	1.9329943		
1584.14969	2.84545584	1598.58282	1.93406856		
1585.37423	2.84458714	1599.14969	1.93425721		
1588.58282	2.84231711	1600.37423	1.93466354		
1589.14969	2.84191699	1603.58282	1.93572066		
1590.37423	2.84105366	1604.14969	1.93590629		
1593.58282	2.8387979	1605.37423	1.93630611		
1594.14969	2.83840035	1608.58282	1.93734623		
1595.37423	2.83754255	1609.14969	1.93752887		
1598.58282	2.83530148	1610.37423	1.93792224		
1599.14969	2.83490655	1613.58282	1.93894555		
1600.37423	2.83405445	1614.14969	1.93912523		
1603.58282	2.8318285	1615.37423	1.93951223		
1604.14969	2.83143625	1618.58282	1.94051896		
1605.37423	2.83059001	1619.14969	1.94069573		
1608.58282	2.82837952	1620.37423	1.94107645		
1609.14969	2.82799003	1623.58282	1.94206683		
1610.37423	2.82714976	1624.14969	1.94224073		
1613.58282	2.82495511	1625.37423	1.94261528		
1614.14969	2.82456844	1628.58282	1.94358962		
1615.37423	2.82373428	1629.14969	1.94376071		
1618.58282	2.82155581	1630.37423	1.94412919		
1619.14969	2.82117202	1633.58282	1.9450878		
1620.37423	2.8203441	1634.14969	1.94525613		
1623.58282	2.81818211	1635.37423	1.94561869		
1624.14969	2.81780125	1638.58282	1.94656191		
1625.37423	2.81697969	1639.14969	1.94672754		
1628.58282	2.81483448	1640.37423	1.94708431		
1629.14969	2.8144566	1643.58282	1.94801253		
1630.37423	2.8136415	1644.14969	1.94817555		
1633.58282	2.81151333	1645.37423	1.94852666		
1634.14969	2.81113848	1648.58282	1.94944027		
1635.37423	2.81032995	1649.14969	1.94960073		
1638.58282	2.80821907	1650.37423	1.94994637		
1639.14969	2.8078473	1653.58282	1.9508458		
1640.37423	2.80704541	1654.14969	1.95100378		
1643.58282	2.80495204	1655.37423	1.9513441		
1644.14969	2.8045834	1658.58282	1.95222981		
1645.37423	2.80378823	1659.14969	1.9523854		
1648.58282	2.80171258	1660.37423	1.95272058		
1649.14969	2.80134707	1663.58282	1.95359303		

Final State: j’=4		Final State: j’=3		Final State: j’=2	
E(cm⁻¹)	σ (Å²)	E(cm⁻¹)	σ (Å²)	E(cm⁻¹)	σ (Å²)
1650.37423	2.8005587	1664.14969	1.9537463		
1653.58282	2.79850095	1665.37423	1.95407653		
1654.14969	2.7981386	1668.58282	1.95493635		
1655.37423	2.7973571	1670.37423	1.95541272		
1658.58282	2.79531743	1673.58282	1.95626014		
1659.14969	2.79495827	1675.37423	1.95672995		
1660.37423	2.7941837	1678.58282	1.95756563		
1663.58282	2.7921622	1680.37423	1.95802903		
1664.14969	2.79180626	1683.58282	1.9588535		
1665.37423	2.79103866	1685.37423	1.9593108		
1668.58282	2.78903537	1688.58282	1.96012461		
1670.37423	2.78792217	1690.37423	1.96057612		
1673.58282	2.78593736	1693.58282	1.96137982		
1675.37423	2.78483436	1695.37423	1.96182584		
1678.58282	2.78286801	1698.58282	1.96262		
1680.37423	2.78177533	1700.37423	1.96306084		
1683.58282	2.7798275	1703.58282	1.96384602		
1685.37423	2.77874516	1705.37423	1.96428201		
1688.58282	2.77681587	1708.58282	1.96505878		
1690.37423	2.7757439	1710.37423	1.96549022		
1693.58282	2.77383316	1713.58282	1.96625914		
1695.37423	2.77277155	1715.37423	1.96668637		
1698.58282	2.77087935	1718.58282	1.96744801		
1700.37423	2.76982808	1720.37423	1.96787134		
1703.58282	2.76795441	1723.58282	1.96862628		
1705.37423	2.76691347	1725.37423	1.96904601		
1708.58282	2.76505827	1728.58282	1.96979482		
1710.37423	2.76402763	1730.37423	1.97021129		
1713.58282	2.76219085	1733.58282	1.97095455		
1715.37423	2.76117047	1735.37423	1.97136811		
1718.58282	2.75935204	1738.58282	1.97210653		
1720.37423	2.75834187	1740.37423	1.97251771		
1723.58282	2.75654169	1743.58282	1.97325408		
1725.37423	2.7555417	1745.37423	1.97364639		
1728.58282	2.75375968	1748.58282	1.97438157		
1730.37423	2.75276981	1750.37423	1.97479143		
1733.58282	2.75100585	1753.58282	1.97550393		
1735.37423	2.75002604	1755.37423	1.97592113		
1738.58282	2.7482801	1758.58282	1.97664168		
1740.37423	2.74731037	1760.37423	1.97704615		
1743.58282	2.74558316	1763.58282	1.9777654		
1745.37423	2.74462109	1765.37423	1.97816741		
1748.58282	2.74292969	1768.58282	1.9788853		
1750.37423	2.7419621	1770.37423	1.97928651		
1753.58282	2.74026137	1773.58282	1.98000697		

**Final State: j’=4**		**Final State: j’=3**		**Final State: j’=2**	
E(cm^{-1})	σ (Å^2)	E(cm^{-1})	σ (Å^2)	E(cm^{-1})	σ (Å^2)
1755.37423	2.73932705	1775.37423	1.98040488		
1758.58282	2.73764788	1780.37423	1.98152163		
1760.37423	2.73672038	1785.37423	1.98261918		
1763.58282	2.7350574	1790.37423	1.98372648		
1765.37423	2.73413801	1795.37423	1.98486003		
1768.58282	2.73249245	1800.37423	1.98596989		
1770.37423	2.73158236	1805.37423	1.98705028		
1773.58282	2.7299533	1810.37423	1.98816895		
1775.37423	2.72905343	1815.37423	1.98927911		
1780.37423	2.72656959	1820.37423	1.9903936		
1785.37423	2.72407353	1825.37423	1.99150295		
1790.37423	2.72164683	1830.37423	1.99261706		
1795.37423	2.71918779	1835.37423	1.99373254		
1800.37423	2.7167964	1840.37423	1.99484963		
1805.37423	2.71440822	1845.37423	1.99596824		
1810.37423	2.71205702	1850.37423	1.99708837		
1815.37423	2.70972432	1855.37423	1.99820993		
1820.37423	2.70740928	1860.37423	1.99933278		
1825.37423	2.70512778	1865.37423	2.00045674		
1830.37423	2.70286359	1870.37423	2.00158162		
1835.37423	2.70062146	1875.37423	2.00270714		
1840.37423	2.69840118	1880.37423	2.003833		
1845.37423	2.69620237	1885.37423	2.00495888		
1850.37423	2.69402476	1890.37423	2.0060844		
1855.37423	2.69186805	1895.37423	2.00720914		
1860.37423	2.68973196	1900.37423	2.00833267		
1865.37423	2.68761621	1905.37423	2.00945451		
1870.37423	2.68552053	1910.37423	2.01057416		
1875.37423	2.68344469				
1880.37423	2.68138842				
1885.37423	2.67935151				
1890.37423	2.6773337				
1895.37423	2.67533478				
1900.37423	2.67335458				
1905.37423	2.67139285				
1910.37423	2.66944943				

	Initial rotational state: j=5		
Final State: j’=1		Final State: j’=0	
E(cm⁻¹)	σ (Å²)	E(cm⁻¹)	σ (Å²)
410.374226	667.453852	410.374226	355.951698
410.374227	472.36536	410.374227	251.912428
410.374228	386.038407	410.374228	205.875084
410.374229	334.635413	410.374229	178.462468
410.37423	299.595471	410.37423	159.776152
410.374231	273.758756	410.374231	145.997846
410.374232	253.700691	410.374232	135.301245
410.374233	237.5502	410.374233	126.688517
410.374234	224.187254	410.374234	119.56236
410.374235	212.895054	410.374235	113.540509
410.374236	203.190738	410.374236	108.365458
410.374237	194.735594	410.374237	103.856577
410.374238	187.284078	410.374238	99.8829171
410.374239	180.653242	410.374239	96.3469145
410.37424	174.703639	410.37424	93.1742046
410.374241	169.326868	410.374241	90.3069773
410.374242	164.437193	410.374242	87.6995105
410.374243	159.965826	410.374243	85.3151206
410.374244	155.856835	410.374244	83.1239798
410.374245	152.06422	410.374245	81.1015566
410.374246	148.549743	410.374246	79.2274594
410.374247	145.281345	410.374247	77.484591
410.374248	142.231887	410.374248	75.8584793
410.374249	139.378255	410.374249	74.3367978
410.37425	136.700594	410.37425	72.908958
410.374251	134.181752	410.374251	71.5658121
410.374252	131.806849	410.374252	70.2994264
410.374253	129.56286	410.374253	69.102854
410.374254	127.438359	410.374254	67.9700015
410.374255	125.423261	410.374255	66.8954911
410.374256	123.508638	410.374256	65.8745612
410.374257	121.68654	410.374257	64.902973
410.374258	119.949872	410.374258	63.976942
410.374259	118.292288	410.374259	63.0930839
410.37426	116.70807	410.37426	62.2483505
410.374261	115.192054	410.374261	61.4399872
410.374262	113.73958	410.374262	60.6655085
410.374263	112.346419	410.374263	59.92266
410.374264	111.008728	410.374264	59.2093914
410.374265	109.722996	410.374265	58.5238314
410.374266	108.486021	410.374266	57.8642719
410.374267	107.294866	410.374267	57.2291467
410.374268	106.14684	410.374268	56.6170208

Final State: j'=1		Final State: j'=0	
E(cm⁻¹)	σ (Å²)	E(cm⁻¹)	σ (Å²)
410.374269	105.039461	410.374269	56.0265701
410.37427	103.970433	410.37427	55.4565709
410.374271	102.937671	410.374271	54.9059112
410.374272	101.939211	410.374272	54.3735433
410.374273	100.973246	410.374273	53.8585043
410.374274	100.0381	410.374274	53.3598992
410.374275	99.1322173	410.374275	52.8768994
410.374276	98.2541445	410.374276	52.4087297
410.374277	97.4025271	410.374277	51.9546673
410.374278	96.5761029	410.374278	51.5140396
410.374279	95.7736955	410.374279	51.086219
410.37428	94.9941938	410.37428	50.6706129
410.374281	94.2365826	410.374281	50.2666804
410.374282	93.4998613	410.374282	49.8738874
410.374283	92.7831357	410.374283	49.4917571
410.374284	92.0855472	410.374284	49.119832
410.374285	91.4062845	410.374285	48.7576791
410.374286	90.7445912	410.374286	48.4048951
410.374287	90.0997477	410.374287	48.0610964
410.374288	89.4710802	410.374288	47.7259236
410.374289	88.8579493	410.374289	47.3990356
410.37429	88.2597453	410.37429	47.0801075
410.374291	87.6759019	410.374291	46.7688372
410.374292	87.1058746	410.374292	46.4649343
410.374293	86.5491393	410.374293	46.1681193
410.374294	86.005223	410.374294	45.8781401
410.374295	85.473662	410.374295	45.5947493
410.374296	84.9540048	410.374296	45.3177061
410.374297	84.4458412	410.374297	45.0467918
410.374298	83.9487695	410.374298	44.7817923
410.374299	83.4624139	410.374299	44.522507
410.3743	82.9864059	410.3743	44.2687397
410.374301	82.5204027	410.374301	44.0203072
410.374302	82.0640813	410.374302	43.7770375
410.374303	81.617122	410.374303	43.5387602
410.374304	81.179224	410.374304	43.3053146
410.374305	80.7501029	410.374305	43.0765492
410.374306	80.3294825	410.374306	42.8523168
410.374307	79.9171034	410.374307	42.6324791
410.374308	79.5127084	410.374308	42.4168987
410.374309	79.1160619	410.374309	42.20545
410.37431	78.726931	410.37431	41.998009
410.374311	78.3450895	410.374311	41.794455
410.374312	77.9703351	410.374312	41.5946801
410.374313	77.6024568	410.374313	41.3985716

Final State: j'=1		Final State: j'=0	
E(cm⁻¹)	σ (Å²)	E(cm⁻¹)	σ (Å²)
410.374314	77.2412582	410.374314	41.2060251
410.374315	76.8865523	410.374315	41.0169408
410.374316	76.5381635	410.374316	40.8312249
410.374317	76.1959066	410.374317	40.6487787
410.374318	75.8596263	410.374318	40.4695195
410.374319	75.5291537	410.374319	40.2933571
410.37432	75.2043346	410.37432	40.1202092
410.374321	74.8850255	410.374321	39.9499996
410.374322	74.5710735	410.374322	39.7826464
410.374323	74.2623443	410.374323	39.6180783
410.374324	73.9587069	410.374324	39.4562253
410.374325	73.6600266	410.374325	39.2970154
410.374825	46.4209781	410.374825	24.7947454
410.375225	48.0882485	410.375225	25.6981698
410.375325	49.0281937	410.375325	26.2027364
410.375825	55.3259485	410.375825	29.576304
410.376225	61.629462	410.376225	32.9477038
410.376325	63.3284663	410.376325	33.8557992
410.376825	72.3476843	410.376825	38.6723599
410.377225	79.9457887	410.377225	42.7241914
410.377325	81.8623095	410.377325	43.7452533
410.377825	91.3063726	410.377825	48.7694329
410.378225	98.384908	410.378225	52.5247071
410.378325	100.044463	410.378325	53.403298
410.378825	107.431859	410.378825	57.3018757
410.379225	111.994686	410.379225	59.6924554
410.379325	112.920218	410.379325	60.1742123
410.379825	116.163745	410.379825	61.8409262
410.380225	117.078759	410.380225	62.277571
410.380325	117.081272	410.380325	62.2664783
410.380825	115.847029	410.380825	61.5526113
410.381225	113.549478	410.381225	60.2933286
410.381325	112.822616	410.381325	59.898921
410.381825	108.461086	410.381825	57.550172
410.382225	104.317732	410.382225	55.3349685
410.382325	103.219257	410.382325	54.7492551
410.382825	97.4977034	410.382825	51.7064985
410.383225	92.7907399	410.383225	49.2101115
410.383325	91.6129709	410.383325	48.5861254
410.383825	85.7939687	410.383825	45.5060535
410.384225	81.290362	410.384225	43.1244243
410.384325	80.1926311	410.384325	42.5441105
410.384825	74.8999576	410.384825	39.7464403
410.385225	70.9203992	410.385225	37.6430756
410.385325	69.962462	410.385325	37.1367269

Final State: j'=1		Final State: j'=0	
E(cm⁻¹)	σ (Å²)	E(cm⁻¹)	σ (Å²)
410.385825	65.3961768	410.385825	34.7227582
410.386225	62.0083875	410.386225	32.9313407
410.386325	61.197382	410.386325	32.5024286
410.386825	57.3504266	410.386825	30.4673543
410.387225	54.5113422	410.387225	28.9648333
410.387325	53.8330512	410.387325	28.605776
410.387825	50.6198792	410.387825	26.9045257
410.388225	48.2509129	410.388225	25.6497319
410.388325	47.6849654	410.388325	25.3499181
410.388825	45.0027886	410.388825	23.9285702
410.389225	43.0228011	410.389225	22.8789577
410.389325	42.5493206	410.389325	22.6279013
410.389825	40.302224	410.389825	21.4362416
410.390225	38.6394191	410.390225	20.554118
410.390325	38.2412273	410.390325	20.3428203
410.390825	36.3478235	410.390825	19.3380299
410.391225	34.9426823	410.391225	18.5921217
410.391325	34.6056084	410.391325	18.4131788
410.391825	32.9995917	410.391825	17.5603485
410.392225	31.8039632	410.392225	16.9253134
410.392325	31.5166601	410.392325	16.772674
410.392825	30.1449107	410.392825	16.0439149
410.393225	29.1205248	410.393225	15.499526
410.393325	28.8739228	410.393325	15.3684581
410.393825	27.6942215	410.393825	14.7414
410.394225	26.8105271	410.394225	14.2716033
410.395225	24.809069	410.395225	13.2072609
410.396225	23.064163	410.396225	12.2790075
410.397225	21.5342152	410.397225	11.4648224
410.398225	20.1855837	410.398225	10.7469161
410.399225	18.9910272	410.399225	10.1107915
410.400225	17.9280803	410.400225	9.54462558
410.401225	16.9783834	410.401225	9.03865408
410.402225	16.1267081	410.402225	8.58475275
410.403225	15.3602398	410.403225	8.17616261
410.404225	14.6683386	410.404225	7.80721411
410.405225	14.0418803	410.405225	7.47309133
410.406225	13.4732543	410.406225	7.16969042
410.407225	12.9558357	410.407225	6.89354819
410.408225	12.4839686	410.408225	6.64163426
410.409225	12.0528707	410.409225	6.4113875
410.410225	11.6581957	410.410225	6.2005137
410.411225	11.2963056	410.411225	6.00709161
410.412225	10.9638761	410.412225	5.82938198
410.413225	10.6581742	410.413225	5.66583865

Final State: j'=1		Final State: j'=0	
E(cm⁻¹)	σ (Å²)	E(cm⁻¹)	σ (Å²)
410.414225	10.3766604	410.414225	5.51516018
410.415225	10.1170045	410.415225	5.37614124
410.416225	9.87732235	410.416225	5.24771375
410.417225	9.65575153	410.417225	5.12893576
410.418225	9.45074994	410.418225	5.01895279
410.419225	9.26080526	410.419225	4.91700729
410.420225	9.0846787	410.420225	4.82238302
410.421225	8.92112827	410.421225	4.73446802
410.422225	8.76909526	410.422225	4.65266762
410.423225	8.62757075	410.423225	4.5764838
410.424225	8.49573394	410.424225	4.50542271
410.425225	8.37268081	410.425225	4.4390469
410.426225	8.25770355	410.426225	4.37694577
410.427225	8.15005192	410.427225	4.31877321
410.428225	8.04911446	410.428225	4.26417113
410.429225	7.95431047	410.429225	4.21282349
410.430225	7.86507423	410.430225	4.16445335
410.431225	7.78091419	410.431225	4.11878196
410.432225	7.70135441	410.432225	4.07556752
410.433225	7.6259899	410.433225	4.03457769
410.434225	7.55437591	410.434225	3.99561146
410.435225	7.48617082	410.435225	3.95846773
410.436225	7.42105568	410.436225	3.92295271
410.437225	7.35870516	410.437225	3.88894088
410.438225	7.29883913	410.438225	3.85625288
410.439225	7.24119673	410.439225	3.82476783
410.440225	7.18552387	410.440225	3.79434953
410.441225	7.13163192	410.441225	3.76487532
410.442225	7.07929055	410.442225	3.73626001
410.443225	7.02835503	410.443225	3.70839259
410.444225	6.9786474	410.444225	3.68118713
410.445225	6.93000642	410.445225	3.65458569
410.446225	6.88231188	410.446225	3.62850637
410.447225	6.83547265	410.447225	3.60287846
410.448225	6.78935401	410.448225	3.57766499
410.449225	6.7438879	410.449225	3.55279832
410.450225	6.69896349	410.450225	3.52826632
410.451225	6.65453922	410.451225	3.50398191
410.452225	6.61053118	410.452225	3.47997
410.453225	6.56692316	410.453225	3.45618074
410.454225	6.52365638	410.454225	3.43257555
410.455225	6.48071021	410.455225	3.40915376
410.456225	6.4380202	410.456225	3.38589615
410.457225	6.39560225	410.457225	3.36278151
410.458225	6.35342996	410.458225	3.33980196

Final State: j’=1		Final State: j’=0	
E(cm⁻¹)	σ (Å²)	E(cm⁻¹)	σ (Å²)
410.459225	6.31145999	410.459225	3.31697509
410.460225	6.26972707	410.460225	3.29428647
410.461225	6.22822184	410.461225	3.2716984
410.462225	6.18691111	410.462225	3.24925974
410.463225	6.14583953	410.463225	3.2269483
410.464225	6.10498484	410.464225	3.20475446
410.465225	6.06439011	410.465225	3.18270701
410.466225	6.02402329	410.466225	3.16078871
410.467225	5.98392092	410.467225	3.1390299
410.468225	5.94407921	410.468225	3.11740525
410.469225	5.9045263	410.469225	3.09595645
410.470225	5.86527503	410.470225	3.07466075
410.471225	5.82635893	410.471225	3.05354381
410.472225	5.78776092	410.472225	3.03260591
410.473225	5.74950731	410.473225	3.01185603
410.474225	5.71163161	410.474225	2.991307
410.574225	15.590307	410.574225	7.52964462
410.674225	4.83831212	410.674225	2.53960327
410.774225	3.28520153	410.774225	1.71633937
410.874225	2.66265978	410.874225	1.38381291
410.974225	2.4313618	410.974225	1.25352508
411.074225	2.42649893	411.074225	1.23794661
411.174225	2.33974074	411.174225	1.18087638
411.274225	2.16178127	411.274225	1.06485259
411.358282	2.63451272	411.358282	1.23106864
411.374225	2.84871559	411.374225	1.3492896
411.474225	2.29768237	411.474225	1.18464915
411.574225	1.93099224	411.574225	0.998050303
411.582817	1.91212005	411.582817	0.988138297
411.674225	1.75645954	411.674225	0.90541733
411.774225	1.63930888	411.774225	0.84246124
411.874225	1.54869386	411.874225	0.793702501
411.974225	1.47251858	411.974225	0.752817723
412	1.45447218	412	0.743156934
412.074225	1.4052992	412.074225	0.716871461
412.074845	1.40490263	412.074845	0.716659557
412.14969	1.35884126	412.14969	0.692084169
412.174225	1.34439663	412.174225	0.684383291
412.274225	1.28844609	412.274225	0.65454329
412.358282	1.24464992	412.358282	0.631125194
412.374225	1.23665275	412.374225	0.626832584
412.474225	1.1886351	412.474225	0.600893055
412.574225	1.14424415	412.574225	0.576388282
412.674225	1.10392132	412.674225	0.55292536
412.774225	1.0717038	412.774225	0.530487896

Final State: j’=1		Final State: j’=0	
E(cm⁻¹)	σ (Å²)	E(cm⁻¹)	σ (Å²)
412.874225	1.12238586	412.874225	0.544232424
412.974225	1.36278914	412.974225	0.646923944
413.074225	1.49531072	413.074225	0.710771948
413.174225	1.27294009	413.174225	0.636769296
413.274225	1.27481195	413.274225	0.637472131
413.358282	1.30714058	413.358282	0.649864942
413.374225	1.31168263	413.374225	0.651497184
413.474225	1.31394717	413.474225	0.650137553
413.574225	1.273568	413.574225	0.629894915
413.582817	1.26881489	413.582817	0.627582997
413.674225	1.21290196	413.674225	0.600607053
413.774225	1.15061164	413.774225	0.570641973
413.874225	1.09437392	413.874225	0.543508568
413.974225	1.04573612	413.974225	0.519921919
414	1.034363	414	0.514386182
414.074225	1.00406947	414.074225	0.499596819
414.074845	1.00383526	414.074845	0.499483935
414.14969	0.976625357	414.14969	0.486136876
414.174225	0.968364716	414.174225	0.482066808
414.274225	0.937567514	414.274225	0.466852486
414.374225	0.910939833	414.374225	0.453593236
414.474225	0.887850121	414.474225	0.441997996
414.574225	0.867865993	414.574225	0.431866597
414.674225	0.85060967	414.674225	0.423019145
414.774225	0.835785177	414.774225	0.415313555
414.874225	0.823118007	414.874225	0.408620264
414.974225	0.812326118	414.974225	0.402806956
415	0.809799494	415	0.401434108
415.074225	0.803052562	415.074225	0.397723019
415.174225	0.794973068	415.174225	0.393210564
415.274225	0.787719777	415.274225	0.389103189
415.358282	0.781942619	415.358282	0.385830009
415.374225	0.780865817	415.374225	0.385220121
415.582817	0.766555053	415.582817	0.377227958
416	0.73257678	416	0.359189421
416.074845	0.725629362	416.074845	0.355585442
416.14969	0.718461709	416.14969	0.351870999
416.374225	0.69502862	416.374225	0.33964015
417.074845	0.786597123	417.074845	0.374869196
417.358282	0.75482632	417.358282	0.35820037
417.374225	0.761007513	417.374225	0.360724225
417.582817	0.914750244	417.582817	0.421980206
418	0.692949163	418	0.331158648
418.074845	0.669646812	418.074845	0.321378808
418.14969	0.65208877	418.14969	0.313867876

Final State: j’=1		Final State: j’=0	
E(cm⁻¹)	σ (Å²)	E(cm⁻¹)	σ (Å²)
418.374225	0.6174069	418.374225	0.29858269
418.582817	0.596414629	418.582817	0.28899116
419.14969	0.557797701	419.14969	0.27072267
419.358282	0.546668008	419.358282	0.265345418
419.374225	0.545859966	419.374225	0.264953473
419.582817	0.535768404	419.582817	0.26004093
420	0.517985363	420	0.251288946
420.074845	0.515122469	420.074845	0.249863077
420.14969	0.512364314	420.14969	0.248482537
420.374225	0.504770686	420.374225	0.244630888
421.358282	0.490785704	421.358282	0.235566726
421.374225	0.490925702	421.374225	0.235566573
421.582817	0.493708722	421.582817	0.235930601
422	0.498435777	422	0.236476449
422.074845	0.498087179	422.074845	0.236137953
422.14969	0.497208598	422.14969	0.23560217
422.358282	0.492126015	422.358282	0.233122238
422.374225	0.491600507	422.374225	0.23288118
423.358282	0.450200871	423.358282	0.214647665
423.374225	0.449591011	423.374225	0.214376643
423.582817	0.442019407	423.582817	0.210991624
424	0.428986463	424	0.20504738
424.074845	0.426940145	424.074845	0.204093479
424.14969	0.424987255	424.14969	0.203174506
424.374225	0.419771732	424.374225	0.200650439
425	0.442766802	425	0.205285883
425.358282	0.461286872	425.358282	0.212113054
425.374225	0.456237999	425.374225	0.210464413
425.582817	0.488817742	425.582817	0.224389761
426	0.392632268	426	0.18729571
426.074845	0.389893549	426.074845	0.186132939
426.14969	0.387817868	426.14969	0.185219804
426.374225	0.383276551	426.374225	0.183121508
427.074845	0.373025826	427.074845	0.178128235
427.358282	0.369202988	427.358282	0.176263603
427.374225	0.368990809	427.374225	0.176159192
427.582817	0.366187538	427.582817	0.174804315
428	0.360594926	428	0.172124527
428.074845	0.359595001	428.074845	0.171648604
428.14969	0.358597089	428.14969	0.171174436
428.374225	0.355619439	428.374225	0.169763751
428.582817	0.352882701	428.582817	0.168471725
429.14969	0.345654682	429.14969	0.165070862
429.374225	0.342897244	429.374225	0.163775027
429.582817	0.340396842	429.582817	0.162599711

Final State: j’=1		Final State: j’=0	
E(cm⁻¹)	σ (Å²)	E(cm⁻¹)	σ (Å²)
430	0.335583964	430	0.160333836
430.074845	0.334748064	430.074845	0.159939533
430.14969	0.333920699	430.14969	0.159548967
430.374225	0.331491278	430.374225	0.158400078
431.358282	0.321898037	431.358282	0.153796964
431.374225	0.321758471	431.374225	0.153728925
431.582817	0.320028583	431.582817	0.152866481
432	0.317128203	432	0.151328664
432.074845	0.316724341	432.074845	0.151089846
432.14969	0.316369987	432.14969	0.150866312
432.358282	0.315712547	432.358282	0.150344084
432.374225	0.315686563	432.374225	0.150311413
433.358282	0.319919605	433.358282	0.150111397
433.374225	0.319858506	433.374225	0.150073536
433.582817	0.317931708	433.582817	0.149252795
434	0.310941601	434	0.146669314
434.074845	0.309652993	434.074845	0.146191791
434.14969	0.308403993	434.14969	0.14572557
434.374225	0.304940568	434.374225	0.14441071
435	0.297288473	435	0.1413623
435.358282	0.293787787	435.358282	0.139898743
435.374225	0.293641862	435.374225	0.139836905
435.582817	0.291794758	435.582817	0.139048681
436	0.288383865	436	0.137570254
436.074845	0.28780404	436.074845	0.137316519
436.14969	0.287232877	436.14969	0.137065829
436.374225	0.285565706	436.374225	0.136330756
437.074845	0.280730082	437.074845	0.134176117
437.358282	0.278904531	437.358282	0.133355311
437.374225	0.278804856	437.374225	0.133310535
437.582817	0.277504148	437.582817	0.13272345
438	0.275002971	438	0.131589868
438.074845	0.27456773	438.074845	0.131391878
438.14969	0.274136309	438.14969	0.131195444
438.374225	0.272865777	438.374225	0.130615578
438.582817	0.271716991	438.582817	0.130089442
439.14969	0.268750123	439.14969	0.128720163
439.358282	0.267716691	439.358282	0.128238612
439.374225	0.267639025	439.374225	0.128202277
439.582817	0.2666404	439.582817	0.127733793
440	0.264740757	440	0.126832925
440.074845	0.264413534	440.074845	0.126676309
440.14969	0.264090423	440.14969	0.126521202
440.374225	0.263144077	440.374225	0.126064503
441.358282	0.259296769	441.358282	0.124185108

Final State: j’=1		Final State: j’=0	
E(cm⁻¹)	σ (Å²)	E(cm⁻¹)	σ (Å²)
441.374225	0.259236591	441.374225	0.124155697
441.582817	0.258447909	441.582817	0.123773355
442	0.256856946	442	0.123013477
442.074845	0.256568226	442.074845	0.122877444
442.14969	0.256278297	442.14969	0.122741461
442.358282	0.255464239	442.358282	0.122362668
442.374225	0.255401695	442.374225	0.122333734
443.358282	0.251487585	443.358282	0.120562074
443.374225	0.251424146	443.374225	0.120533865
443.582817	0.250595367	443.582817	0.120165471
444	0.248956115	444	0.119440375
444.074845	0.248665327	444.074845	0.119312081
444.14969	0.248375647	444.14969	0.119184374
444.374225	0.247514009	444.374225	0.118804855
445	0.245176485	445	0.117776809
445.358282	0.24388332	445.358282	0.117208356
445.374225	0.243826601	445.374225	0.117183394
445.582817	0.24309012	445.582817	0.116859639
446	0.241651824	446	0.116226927
446.074845	0.241398609	446.074845	0.116115477
446.14969	0.241146852	446.14969	0.116004663
446.374225	0.240400325	446.374225	0.115675901
447.074845	0.2381532	447.074845	0.114685065
447.358282	0.237278657	447.358282	0.114298853
447.374225	0.237230485	447.374225	0.114277472
447.582817	0.236599778	447.582817	0.113998715
448	0.235369457	448	0.113454328
448.074845	0.235153011	448.074845	0.113358434
448.14969	0.234937739	448.14969	0.113263105
448.374225	0.2342998	448.374225	0.112980265
448.582817	0.233716845	448.582817	0.112721703
449.14969	0.232178684	449.14969	0.112038782
449.358282	0.231628967	449.358282	0.111794493
449.374225	0.231587506	449.374225	0.11177596
449.582817	0.23104643	449.582817	0.111535588
450	0.229988375	450	0.111065285
450.074845	0.22980172	450.074845	0.110982347
450.14969	0.22961605	450.14969	0.110899821
450.374225	0.229064463	450.374225	0.110654777
451.358282	0.226736457	451.358282	0.109622692
451.374225	0.226700003	451.374225	0.10960641
451.582817	0.226223841	451.582817	0.10939619
452	0.225288274	452	0.108983603
452.074845	0.225122667	452.074845	0.108910651
452.14969	0.224957731	452.14969	0.108838085

Final State: j'=1		Final State: j'=0	
E(cm⁻¹)	σ (Å²)	E(cm⁻¹)	σ (Å²)
452.358282	0.224501389	452.358282	0.108637532
452.374225	0.224466699	452.374225	0.108622308
453.358282	0.222380795	453.358282	0.10771009
453.374225	0.222348488	453.374225	0.107695686
453.582817	0.221919421	453.582817	0.107509303
454	0.221076063	454	0.107143313
454.074845	0.220926669	454.074845	0.107078619
454.14969	0.220777846	454.14969	0.107014189
454.374225	0.220334798	454.374225	0.106822645
455	0.219126966	455	0.106302144
455.358282	0.218453241	455.358282	0.106012818
455.374225	0.218423578	455.374225	0.106000088
455.582817	0.218037511	455.582817	0.105834646
456	0.217278178	456	0.105509984
456.074845	0.217143759	456.074845	0.105452619
456.14969	0.217009897	456.14969	0.105395502
456.374225	0.216611528	456.374225	0.105225703
457.074845	0.215399572	457.074845	0.104710637
457.358282	0.214922482	457.358282	0.104508477
457.374225	0.214895451	457.374225	0.104496982
457.582817	0.214549819	457.582817	0.104350833
458	0.213869692	458	0.104063681
458.074845	0.213749351	458.074845	0.104012952
458.14969	0.213629493	458.14969	0.103962451
458.374225	0.213272969	458.374225	0.103812358
458.582817	0.212945762	458.582817	0.103674791
459.14969	0.212075582	459.14969	0.103309838
459.358282	0.211762294	459.358282	0.10317877
459.374225	0.211738443	459.374225	0.103168812
459.582817	0.21142911	459.582817	0.103039568
460	0.210821059	460	0.102786095
460.074845	0.21071345	460.074845	0.102741316
460.14969	0.210606298	460.14969	0.102696743
460.374225	0.210287492	460.374225	0.102564272
461.358282	0.208935988	461.358282	0.102005172
461.582817	0.208637669	461.582817	0.10188236
462	0.20809306	462	0.101658691
462.074845	0.207996649	462.074845	0.101619187
462.14969	0.207900629	462.14969	0.101579853
462.358282	0.207635024	462.358282	0.10147122
462.374225	0.207614853	462.374225	0.10146297
463.358282	0.20640222	463.358282	0.100969685
463.582817	0.206134337	463.582817	0.100861318
464	0.205644929	464	0.100664016
464.074845	0.205558266	464.074845	0.100629163

Final State: j'=1		Final State: j'=0	
E(cm⁻¹)	σ (Å²)	E(cm⁻¹)	σ (Å²)
464.14969	0.205471921	464.14969	0.100594467
464.374225	0.205214989	464.374225	0.100491368
465	0.204514566	465	0.100211624
465.358282	0.204123736	465.358282	0.100056358
465.374225	0.204106514	465.374225	0.100049525
465.582817	0.203882595	465.582817	0.099960838
466	0.203441704	466	0.099786956
466.074845	0.203363645	466.074845	0.099756214
466.374225	0.203054425	466.374225	0.099634841
467.074845	0.202349208	467.074845	0.099359713
467.358282	0.202071109	467.358282	0.099251884
467.582817	0.201853346	467.582817	0.099167924
468	0.201456107	468	0.099015005
468.074845	0.201385809	468.074845	0.098987996
468.14969	0.201315628	468.14969	0.098961161
468.374225	0.201107009	468.374225	0.098881363
468.582817	0.200915222	468.582817	0.098808285
469.14969	0.200404582	469.14969	0.098614711
469.358282	0.200220498	469.358282	0.098545272
469.582817	0.200024485	469.582817	0.098471621
470	0.19966634	470	0.098337707
470.074845	0.199602938	470.074845	0.098314069
470.14969	0.199539743	470.14969	0.098290562
470.374225	0.199351656	470.374225	0.098220741
471.358282	0.198552918	471.358282	0.097927065
471.582817	0.198376184	471.582817	0.097862819
472	0.198053587	472	0.097746098
472.074845	0.197996461	472.074845	0.097725519
472.14969	0.197939525	472.14969	0.097705035
472.358282	0.197782092	472.358282	0.097648471
472.374225	0.197770073	472.374225	0.097644224
473.358282	0.197050696	473.358282	0.097388997
473.582817	0.196891678	473.582817	0.097333269
474	0.196601166	474	0.097232113
474.074845	0.196549693	474.074845	0.097214287
474.14969	0.196498448	474.14969	0.097196579
474.374225	0.196345897	474.374225	0.097143975
475	0.195930146	475	0.097002001
475.358282	0.19569819	475.358282	0.096923699
475.374225	0.195687964	475.374225	0.096920259
476	0.195293537	476	0.096788725
476.074845	0.195247215	476.074845	0.096773409
476.14969	0.195201083	476.14969	0.096758186
476.374225	0.195063807	476.374225	0.096713061
477.074845	0.194645714	477.074845	0.096577324

Final State: j'=1		Final State: j'=0	
E(cm⁻¹)	σ (Å²)	E(cm⁻¹)	σ (Å²)
477.358282	0.194480928	477.358282	0.096524547
477.582817	0.194352134	477.582817	0.096483603
478	0.194116864	478	0.096409498
478.074845	0.194075199	478.074845	0.096396479
478.14969	0.19403371	478.14969	0.09638354
478.374225	0.193910191	478.374225	0.096345196
478.582817	0.193796786	478.582817	0.09631023
479.14969	0.193494818	479.14969	0.096218315
479.358282	0.193385968	479.358282	0.096185617
479.582817	0.193270172	479.582817	0.096151085
480	0.193058633	480	0.096088752
480.074845	0.193021171	480.074845	0.096077808
480.14969	0.192983893	480.14969	0.096066947
480.374225	0.192872861	480.374225	0.096034801
481.358282	0.192401766	481.358282	0.095901612
481.582817	0.192297719	481.582817	0.09587295
482	0.192107694	482	0.095821337
482.074845	0.192074051	482.074845	0.095812301
482.14969	0.192040545	482.14969	0.095803333
482.358282	0.191947885	482.358282	0.095778702
482.374225	0.191940848	482.374225	0.095776841
483.358282	0.191517859	483.358282	0.095667744
483.582817	0.191424432	483.582817	0.095644454
484	0.191253924	484	0.095602602
484.14969	0.191193691	484.14969	0.095588074
484.374225	0.191104142	484.374225	0.095566723
485	0.19086041	485	0.095510165
485.358282	0.190724609	485.358282	0.095479653
485.374225	0.190718668	485.374225	0.095478319
485.582817	0.190640914	485.582817	0.095461208
486	0.190487923	486	0.095428329
486.074845	0.190460856	486.074845	0.095422614
486.14969	0.190433919	486.14969	0.095416963
486.374225	0.190353607	486.374225	0.095400334
487.074845	0.190109523	487.074845	0.095351643
487.358282	0.190013272	487.358282	0.095333301
487.582817	0.189938203	487.582817	0.095319312
488	0.189801029	488	0.095294571
488.074845	0.189776727	488.074845	0.095290276
488.14969	0.18975258	488.14969	0.095286075
488.374225	0.189680588	488.374225	0.095273734
488.582817	0.189614529	488.582817	0.095262666
489.14969	0.189438762	489.14969	0.095234559
489.358282	0.189375388	489.358282	0.095224916
489.582817	0.189307993	489.582817	0.095214962

Final State: j’=1		Final State: j’=0	
E(cm⁻¹)	σ (Å²)	E(cm⁻¹)	σ (Å²)
490	0.189184934	490	0.095197597
490.074845	0.189163147	490.074845	0.09519462
490.14969	0.18914142	490.14969	0.095191717
490.374225	0.189076852	490.374225	0.095183255
491.358282	0.188802714	491.358282	0.095150905
491.582817	0.188742134	491.582817	0.095144588
492	0.188631468	492	0.095133863
492.074845	0.188611849	492.074845	0.095132078
492.14969	0.188592335	492.14969	0.095130329
492.358282	0.188538296	492.358282	0.095125683
492.374225	0.188534186	492.374225	0.095125346
493.358282	0.188287149	493.358282	0.095107739
493.582817	0.188232449	493.582817	0.095104651
494	0.18813253	494	0.095099849
494.074845	0.188114785	494.074845	0.095099112
494.14969	0.188097038	494.14969	0.095098388
494.374225	0.188044271	494.374225	0.09509648
495	0.187900777	495	0.09509285
495.358282	0.187820015	495.358282	0.095091825
495.374225	0.187816474	495.374225	0.0950918
495.582817	0.187770429	495.582817	0.095091619
496	0.18767914	496	0.095091908
496.074845	0.187663011	496.074845	0.095092058
496.14969	0.187646752	496.14969	0.09509225
496.374225	0.187598746	496.374225	0.095092992
497.074845	0.187451304	497.074845	0.095097071
497.358282	0.187392723	497.358282	0.095099428
497.582817	0.187346753	497.582817	0.095101552
498	0.187262265	498	0.095106141
498.074845	0.187247211	498.074845	0.095107045
498.14969	0.18723221	498.14969	0.095107975
498.374225	0.187187386	498.374225	0.095110914
498.582817	0.187146	498.582817	0.095113835
499.14969	0.18703466	499.14969	0.095122631
499.358282	0.186994059	499.358282	0.095126169
499.582817	0.186950541	499.582817	0.095130158
500	0.186869742	500	0.095137879
500.074845	0.186855758	500.074845	0.09513946
500.14969	0.186841403	500.14969	0.095140937
500.374225	0.186798363	500.374225	0.095145506
501.358282	0.186610546	501.358282	0.095166845
501.582817	0.186567827	501.582817	0.09517197
502.074845	0.18647365	502.074845	0.095183487
502.14969	0.186459271	502.14969	0.095185264
502.358282	0.186419122	502.358282	0.095190232

Final State: j’=1		Final State: j’=0	
E(cm⁻¹)	σ (Å²)	E(cm⁻¹)	σ (Å²)
502.374225	0.186416034	502.374225	0.095190613
503.358282	0.186223658	503.358282	0.095214147
503.582817	0.186178846	503.582817	0.095219437
504.074845	0.186078958	504.074845	0.095230758
504.14969	0.186063514	504.14969	0.095232436
504.374225	0.18601682	504.374225	0.095237377
505	0.185882534	505	0.095250097
505.358282	0.185803181	505.358282	0.095256798
505.374225	0.185799102	505.374225	0.095256993
505.582817	0.185751865	505.582817	0.095260526
506.074845	0.185635176	506.074845	0.095267451
506.14969	0.18561683	506.14969	0.095268329
506.374225	0.185560858	506.374225	0.095270642
507.074845	0.185374788	507.074845	0.095274218
507.358282	0.185293729	507.358282	0.09527375
507.582817	0.185226628	507.582817	0.095272389
508.074845	0.185069367	508.074845	0.09526585
508.14969	0.185044006	508.14969	0.095264343
508.374225	0.184965726	508.374225	0.095258993
508.582817	0.184888971	508.582817	0.095252655
509.14969	0.184659471	509.14969	0.095227646
509.358282	0.184565649	509.358282	0.095214936
509.582817	0.184457669	509.582817	0.095198622
510	0.184233784	510	0.095159507
510.074845	0.184190427	510.074845	0.095151196
510.14969	0.184145381	510.14969	0.095142325
510.374225	0.184002037	510.374225	0.095112324
511.358282	0.183157336	511.358282	0.094893534
511.582817	0.182886709	511.582817	0.09481112
512.074845	0.182101425	512.074845	0.094546214
512.14969	0.181948298	512.14969	0.094490688
512.358282	0.181450736	512.358282	0.094302378
513.358282	0.176655625	513.358282	0.091374641
513.582817	0.178661758	513.582817	0.091951088
514.074845	0.182217272	514.074845	0.092510641
514.14969	0.184095407	514.14969	0.093458061
514.374225	0.186179677	514.374225	0.095238149
515	0.181670522	515	0.094290223
515.358282	0.179236116	515.358282	0.092359695
515.374225	0.179204597	515.374225	0.092285506
515.582817	0.18143245	515.582817	0.092546044
516.074845	0.187552847	516.074845	0.0963503
516.14969	0.187119913	516.14969	0.096385061
516.374225	0.186347574	516.374225	0.096245222
517.074845	0.181879756	517.074845	0.094663456

Final State: j'=1		Final State: j'=0	
E(cm⁻¹)	σ (Å²)	E(cm⁻¹)	σ (Å²)
517.358282	0.17726827	517.358282	0.0920217
517.582817	0.18135245	517.582817	0.093465343
518.074845	0.18357665	518.074845	0.093432382
518.14969	0.185403038	518.14969	0.094382595
518.374225	0.186611183	518.374225	0.095835295
518.582817	0.184858266	518.582817	0.095025429
519.14969	0.187019799	519.14969	0.096594158
519.358282	0.184146962	519.358282	0.095459639
519.582817	0.183738431	519.582817	0.094074164
520	0.187368795	520	0.096959805
520.074845	0.186825368	520.074845	0.096819991
520.14969	0.186289145	520.14969	0.096662325
520.374225	0.184616781	520.374225	0.096088688
521.358282	0.186848903	521.358282	0.094622172
521.582817	0.189232943	521.582817	0.097325163
522.074845	0.187742428	522.074845	0.097126722
522.14969	0.186876181	522.14969	0.096787651
522.358282	0.182602461	522.358282	0.094675922
522.374225	0.182100748	522.374225	0.094366643
523.358282	0.189590605	523.358282	0.098336138
523.582817	0.188547875	523.582817	0.09803938
524.074845	0.186278102	524.074845	0.097287908
524.14969	0.185871303	524.14969	0.097138032
524.374225	0.184409761	524.374225	0.096561045
525	0.179257174	525	0.093149294
525.358282	0.186177514	525.358282	0.095895143
525.374225	0.186474526	525.374225	0.096056505
525.582817	0.187627347	525.582817	0.096786041
526.074845	0.188895491	526.074845	0.097975147
526.14969	0.187363355	526.14969	0.097524327
526.374225	0.18292439	526.374225	0.094391747
527.074845	0.193216666	527.074845	0.099999843
527.358282	0.191687855	527.358282	0.099633289
527.582817	0.190824105	527.582817	0.099404374
528.074845	0.189286667	528.074845	0.098967084
528.14969	0.189062754	528.14969	0.098899702
528.374225	0.188367806	528.374225	0.098683208
528.582817	0.187654293	528.582817	0.098448396
529.14969	0.18478352	529.14969	0.097375817
529.582817	0.179698629	529.582817	0.095023067
530	0.185406232	530	0.095217444
530.074845	0.187910669	530.074845	0.096937969
530.14969	0.188917822	530.14969	0.098038697
530.374225	0.190170861	530.374225	0.099023655
531.582817	0.196767103	531.582817	0.101520177

Final State: j'=1		Final State: j'=0	
E(cm⁻¹)	σ (Å²)	E(cm⁻¹)	σ (Å²)
532.074845	0.19324772	532.074845	0.100846779
532.14969	0.1929546	532.14969	0.100771167
532.358282	0.192266166	532.358282	0.100591311
532.374225	0.19221998	532.374225	0.10057916
533.582817	0.189438262	533.582817	0.099818082
534.074845	0.188111213	534.074845	0.099399827
534.14969	0.18785956	534.14969	0.099314889
534.374225	0.186962826	534.374225	0.098998514
535	0.181584004	535	0.096677266
535.374225	0.180349345	535.374225	0.095120097
535.582817	0.18634379	535.582817	0.096665772
536.074845	0.189591705	536.074845	0.099903702
536.14969	0.189299177	536.14969	0.099410889
536.374225	0.182793813	536.374225	0.095064903
537.074845	0.197105247	537.074845	0.102639106
537.358282	0.194838469	537.358282	0.102139572
537.582817	0.193863775	537.582817	0.10188554
538.074845	0.192524394	538.074845	0.101536622
538.14969	0.192366934	538.14969	0.101496428
538.374225	0.191933113	538.374225	0.101386665
538.582817	0.191565574	538.582817	0.101294503
539.14969	0.190632202	539.14969	0.101058483
539.582817	0.18988304	539.582817	0.100858411
540	0.189016869	540	0.100606912
540.074845	0.188835058	540.074845	0.100551087
540.14969	0.188640839	540.14969	0.100490372
540.374225	0.187965771	540.374225	0.100270733
541.582817	0.181528753	541.582817	0.096884246
542.074845	0.19328368	542.074845	0.10141818
542.14969	0.192077779	542.14969	0.101397467
542.358282	0.187044901	542.358282	0.099963042
542.374225	0.186925697	542.374225	0.099759561
543.582817	0.193668683	543.582817	0.102687979
544.074845	0.192016243	544.074845	0.102223262
544.14969	0.191821087	544.14969	0.10216823
544.374225	0.191275384	544.374225	0.102013775
545	0.189864907	545	0.101601524
545.374225	0.188962164	545.374225	0.101317779
545.582817	0.188393939	545.582817	0.101128298
546.074845	0.186675687	546.074845	0.100496253
546.14969	0.186336957	546.14969	0.10036022
546.374225	0.18510391	546.374225	0.099828395
547.074845	0.183168166	547.074845	0.097952091
547.358282	0.181623767	547.358282	0.096691326
547.582817	0.185856944	547.582817	0.098421148

Final State: j'=1		Final State: j'=0	
E(cm⁻¹)	σ (Å²)	E(cm⁻¹)	σ (Å²)
548.074845	0.180009848	548.074845	0.097508899
548.14969	0.177645932	548.14969	0.096455088
548.374225	0.17663162	548.374225	0.0946774
548.582817	0.183388961	548.582817	0.097273202
549.14969	0.186689829	549.14969	0.098737109
549.582817	0.193857855	549.582817	0.102961056
550	0.19246376	550	0.102370399
550.074845	0.190774862	550.074845	0.101717513
550.14969	0.189101547	550.14969	0.101003335
550.374225	0.185341519	550.374225	0.09848788
551.582817	0.195960487	551.582817	0.104228564
552.074845	0.191594229	552.074845	0.102867868
552.14969	0.190802722	552.14969	0.102565697
552.358282	0.188087916	552.358282	0.101420065
552.374225	0.187837971	552.374225	0.101306029
553.582817	0.200578943	553.582817	0.105429676
554.074845	0.196110744	554.074845	0.104820464
554.14969	0.195547195	554.14969	0.104675976
554.374225	0.193892482	554.374225	0.104206836
555	0.188087126	555	0.102195103
555.374225	0.180784071	555.374225	0.099143531
555.582817	0.172621393	555.582817	0.095143819
556.074845	0.188198978	556.074845	0.097947813
556.14969	0.193122427	556.14969	0.097699481
556.374225	0.204490308	556.374225	0.104640331
557.074845	0.206878995	557.074845	0.108010213
557.358282	0.201978609	557.358282	0.107490799
557.582817	0.199898337	557.582817	0.106883488
558.074845	0.196473365	558.074845	0.105806262
558.14969	0.19595338	558.14969	0.105630528
558.374225	0.194234389	558.374225	0.105021169
558.582817	0.192212886	558.582817	0.104246964
559.14969	0.180544153	559.14969	0.097951538
559.582817	0.191871636	559.582817	0.098594866
560	0.205269598	560	0.108492605
560.074845	0.204370132	560.074845	0.108376974
560.14969	0.203585043	560.14969	0.108249267
560.374225	0.20171771	560.374225	0.107871004
561.582817	0.195844131	561.582817	0.106280266
562.074845	0.19337405	562.074845	0.105475032
562.14969	0.192909892	562.14969	0.10531444
562.358282	0.191381481	562.358282	0.104766003
562.374225	0.19124698	562.374225	0.104716225
563.582817	0.19625181	563.582817	0.104717963
564.074845	0.200423708	564.074845	0.108295451

Final State: j’=1		Final State: j’=0	
E(cm⁻¹)	σ (Å²)	E(cm⁻¹)	σ (Å²)
564.14969	0.199930216	564.14969	0.10770675
564.374225	0.194041213	564.374225	0.103650312
565	0.202485329	565	0.108267356
565.374225	0.195838684	565.374225	0.105272398
565.582817	0.194665162	565.582817	0.105325192
566.074845	0.197222845	566.074845	0.106740788
566.374225	0.191383139	566.374225	0.104622873
567.074845	0.192656374	567.074845	0.105279945
567.358282	0.182174028	567.358282	0.1010968
567.582817	0.17780755	567.582817	0.096056
568.074845	0.213408721	568.074845	0.110671405
568.14969	0.211703611	568.14969	0.111069886
568.374225	0.20678342	568.374225	0.110542873
568.582817	0.203289304	568.582817	0.109674817
569.14969	0.191503144	569.14969	0.105072466
569.582817	0.207431698	569.582817	0.108416347
570	0.202867334	570	0.109732994
570.074845	0.202019442	570.074845	0.109544552
570.14969	0.201218693	570.14969	0.109345024
570.374225	0.198940824	570.374225	0.108692501
571.582817	0.197390974	571.582817	0.107777574
572.074845	0.175554263	572.074845	0.099464952
572.14969	0.177715446	572.14969	0.098898319
572.358282	0.187681963	572.358282	0.100992654
572.374225	0.187691948	572.374225	0.100477943
573.582817	0.20449525	573.582817	0.109420248
574.074845	0.201586594	574.074845	0.109968183
574.14969	0.192639075	574.14969	0.104784196
574.374225	0.2047899	574.374225	0.111489199
575	0.195546064	575	0.108027187
575.374225	0.186177786	575.374225	0.104316923
576.074845	0.194728671	576.074845	0.107722037
576.14969	0.19404627	576.14969	0.107509263
576.374225	0.192088885	576.374225	0.106868372
577.074845	0.175600664	577.074845	0.098388536
577.358282	0.199887685	577.358282	0.109178239
577.582817	0.194483854	577.582817	0.10771903
578.074845	0.193747766	578.074845	0.107661287
578.14969	0.193683829	578.14969	0.107661074
578.374225	0.193380965	578.374225	0.107614147
578.582817	0.193001771	578.582817	0.107530099
579.14969	0.191667496	579.14969	0.1071845
579.582817	0.190491189	579.582817	0.106863206
580	0.18926065	580	0.10652017
580.074845	0.188984664	580.074845	0.106439842

Final State: j'=1		Final State: j'=0	
E(cm⁻¹)	σ (Å²)	E(cm⁻¹)	σ (Å²)
580.14969	0.188665898	580.14969	0.106345168
580.374225	0.187172935	580.374225	0.105880179
581.582817	0.189040603	581.582817	0.106769948
582.074845	0.192220593	582.074845	0.107979682
582.14969	0.192553023	582.14969	0.108085399
582.358282	0.192701193	582.358282	0.108143438
582.374225	0.192693995	582.374225	0.108142384
583.582817	0.191768953	583.582817	0.108011829
584.14969	0.191449203	584.14969	0.108001135
584.374225	0.191318554	584.374225	0.107996979
585	0.190849219	585	0.107959285
585.374225	0.19042747	585.374225	0.107902519
585.582817	0.190144335	585.582817	0.107861707
586.14969	0.189580672	586.14969	0.107829159
586.374225	0.189609679	586.374225	0.107888999
587.074845	0.190143212	587.074845	0.10817355
587.358282	0.190304107	587.358282	0.108265417
587.582817	0.190382822	587.582817	0.108323226
588.14969	0.1903833	588.14969	0.108410851
588.374225	0.190282386	588.374225	0.108415716
588.582817	0.190092994	588.582817	0.108392018
589.14969	0.186345417	589.14969	0.107491835
589.582817	0.189218584	589.582817	0.108436801
590	0.19234808	590	0.109401515
590.14969	0.192195862	590.14969	0.109361888
590.374225	0.191961012	590.374225	0.109319401
591.582817	0.191579584	591.582817	0.109411847
592.074845	0.191582712	592.074845	0.109500767
592.14969	0.191587105	592.14969	0.109515344
592.358282	0.191601374	592.358282	0.109556839
592.374225	0.191603076	592.374225	0.109560087
593.582817	0.191739706	593.582817	0.10981554
594.14969	0.191809844	594.14969	0.109937423
594.374225	0.191836247	594.374225	0.109985437
595	0.191900934	595	0.110117361
595.374225	0.191929916	595.374225	0.110194025
595.582817	0.191940181	595.582817	0.110235521
596.14969	0.191929695	596.14969	0.110340641
596.374225	0.191898569	596.374225	0.110377428
597.074845	0.191633719	597.074845	0.110475786
597.358282	0.191567732	597.358282	0.110539426
597.582817	0.1916566	597.582817	0.110617826
598.14969	0.192134278	598.14969	0.11083739
598.374225	0.192285384	598.374225	0.110910395
598.582817	0.192396549	598.582817	0.110971695

Final State: j’=1		Final State: j’=0	
E(cm⁻¹)	σ (Å²)	E(cm⁻¹)	σ (Å²)
599.14969	0.192610185	599.14969	0.111120928
599.582817	0.192729665	599.582817	0.111227421
600	0.192828967	600	0.111327822
600.14969	0.192862951	600.14969	0.111363599
600.374225	0.192911983	600.374225	0.111417111
601.582817	0.193159121	601.582817	0.111703953
602.074845	0.193254471	602.074845	0.111820455
602.14969	0.193269184	602.14969	0.111838164
602.358282	0.193308523	602.358282	0.111887499
602.374225	0.19331189	602.374225	0.111891267
603.582817	0.193531733	603.582817	0.112176342
604.14969	0.193628186	604.14969	0.112309721
604.374225	0.193665438	604.374225	0.112362599
605	0.193768464	605	0.112510781
605.374225	0.193832802	605.374225	0.112600356
605.582817	0.193869999	605.582817	0.112650709
606.14969	0.193978431	606.14969	0.112789132
606.374225	0.194024387	606.374225	0.112844506
607.074845	0.19417588	607.074845	0.11301849
607.358282	0.194239667	607.358282	0.113089094
607.582817	0.194290933	607.582817	0.113145022
608.14969	0.19442019	608.14969	0.113286138
608.374225	0.19447131	608.374225	0.113341952
608.582817	0.194518659	608.582817	0.113393773
609.14969	0.194646648	609.14969	0.113534361
609.582817	0.194743434	609.582817	0.113641606
610	0.194835799	610	0.113744806
610.14969	0.194869132	610.14969	0.11378177
610.374225	0.194918511	610.374225	0.113837274
611.582817	0.195181664	611.582817	0.114135616
612.074845	0.19528735	612.074845	0.11425706
612.14969	0.195303614	612.14969	0.114275483
612.358282	0.195348197	612.358282	0.114326984
613.582817	0.195609616	613.582817	0.114629353
614.14969	0.195730208	614.14969	0.11476953
614.374225	0.195778	614.374225	0.114825114
615	0.195911244	615	0.114980194
615.374225	0.195991683	615.374225	0.115073006
615.582817	0.196036441	615.582817	0.115124795
616.14969	0.196158489	616.14969	0.115265679
616.374225	0.19620706	616.374225	0.115321541
617.074845	0.196358803	617.074845	0.115496068
617.358282	0.196420655	617.358282	0.115566725
617.582817	0.196469997	617.582817	0.115622696
618.14969	0.196594298	618.14969	0.115764188

Final State: j’=1		Final State: j’=0	
E(cm⁻¹)	σ (Å²)	E(cm⁻¹)	σ (Å²)
618.374225	0.196643523	618.374225	0.115820272
618.582817	0.196689427	618.582817	0.115872395
619.14969	0.19681439	619.14969	0.116014101
619.582817	0.196910073	619.582817	0.116122456
620	0.19700211	620	0.116226919
620.14969	0.197035571	620.14969	0.116264357
620.374225	0.197085404	620.374225	0.116320586
621.582817	0.197353705	621.582817	0.116623496
622.074845	0.197462886	622.074845	0.11674697
622.14969	0.197479829	622.14969	0.116765706
622.358282	0.19752599	622.358282	0.116818104
622.374225	0.197529817	622.374225	0.116822052
623.582817	0.197799253	623.582817	0.117125609
624.14969	0.197925858	624.14969	0.117268122
624.374225	0.197976038	624.374225	0.117324595
625	0.198115762	625	0.117482116
625.374225	0.198199879	625.374225	0.117576272
625.582817	0.198246607	625.582817	0.117628805
626.14969	0.198373779	626.14969	0.117771629
626.374225	0.198424225	626.374225	0.117828228
627.074845	0.198581494	627.074845	0.118004985
627.358282	0.198645335	627.358282	0.118076506
627.582817	0.198696183	627.582817	0.118133112
628.14969	0.198824084	628.14969	0.118276247
628.374225	0.198874797	628.374225	0.118332964
628.582817	0.198921945	628.582817	0.118385662
629.14969	0.199050202	629.14969	0.118528955
629.582817	0.199148367	629.582817	0.118638489
630	0.199242779	630	0.118744103
630.14969	0.199277015	630.14969	0.118781909
630.374225	0.199328031	630.374225	0.118838741
631.582817	0.199603181	631.582817	0.119144843
632.074845	0.199715223	632.074845	0.119269641
632.14969	0.199732556	632.14969	0.119288537
632.358282	0.199779976	632.358282	0.119341505
632.374225	0.199783862	632.374225	0.119345471
633.582817	0.20006049	633.582817	0.119652117
634.14969	0.200190538	634.14969	0.119796032
634.374225	0.200242117	634.374225	0.119853045
635	0.200385749	635	0.120012135
635.374225	0.200472093	635.374225	0.120107142
635.582817	0.20052014	635.582817	0.120160168
636.14969	0.200650833	636.14969	0.120304311
636.374225	0.200702648	636.374225	0.12036142
637.074845	0.200864291	637.074845	0.120539775

Final State: j’=1		Final State: j’=0	
E(cm⁻¹)	σ (Å²)	E(cm⁻¹)	σ (Å²)
637.358282	0.200929854	637.358282	0.120611907
637.582817	0.200982028	637.582817	0.120668968
638.14969	0.20111334	638.14969	0.120813311
638.374225	0.201165395	638.374225	0.120870497
638.582817	0.201213805	638.582817	0.12092362
639.14969	0.20134542	639.14969	0.121068062
639.582817	0.201446095	639.582817	0.121178451
640	0.201542973	640	0.1212849
640.14969	0.201578026	640.14969	0.121322968
640.374225	0.201630328	640.374225	0.12138022
641.582817	0.201912334	641.582817	0.121688531
642.074845	0.202027191	642.074845	0.121814205
642.14969	0.202044889	642.14969	0.121833204
642.358282	0.202093524	642.358282	0.121886572
642.374225	0.202097415	642.374225	0.121890536
643.582817	0.202380747	643.582817	0.122199165
644.14969	0.202513888	644.14969	0.12234399
644.374225	0.20256667	644.374225	0.122401367
645	0.202713762	645	0.122561415
645.374225	0.202802092	645.374225	0.12265696
645.582817	0.202851263	645.582817	0.122710288
646.14969	0.202985008	646.14969	0.122855241
646.374225	0.203038049	646.374225	0.122912659
647.074845	0.203203502	647.074845	0.123091998
647.358282	0.203270577	647.358282	0.123164517
647.582817	0.20332389	647.582817	0.123221834
648.14969	0.203458273	648.14969	0.123366895
648.374225	0.203511488	648.374225	0.123424364
648.582817	0.203561012	648.582817	0.123477746
649.14969	0.203695643	649.14969	0.123622863
649.582817	0.20379863	649.582817	0.123733756
650	0.203897771	650	0.123840715
650.14969	0.203933544	650.14969	0.123878901
650.374225	0.203987048	650.374225	0.12393641
651.582817	0.204275418	651.582817	0.124245983
652.074845	0.204392899	652.074845	0.124372185
652.14969	0.204410933	652.14969	0.124391219
652.358282	0.204460706	652.358282	0.124444809
652.374225	0.204464653	652.374225	0.124448751
653.582817	0.204754261	653.582817	0.124758477
654.14969	0.204890371	654.14969	0.124903778
654.374225	0.204944316	654.374225	0.124961329
655	0.205094712	655	0.125121927
655.374225	0.205184939	655.374225	0.125217711
655.582817	0.205235181	655.582817	0.125271191

Final State: j'=1		Final State: j'=0	
E(cm⁻¹)	σ (Å²)	E(cm⁻¹)	σ (Å²)
656.14969	0.205371862	656.14969	0.125416548
656.374225	0.205426053	656.374225	0.125474125
657.074845	0.205595176	657.074845	0.125653959
657.358282	0.205663758	657.358282	0.125726672
657.582817	0.20571812	657.582817	0.125784098
658.14969	0.205855413	658.14969	0.125929481
658.374225	0.20590985	658.374225	0.125987091
658.582817	0.20596038	658.582817	0.126040574
659.14969	0.206098006	659.14969	0.126185995
659.582817	0.206203276	659.582817	0.126297098
660	0.206304479	660	0.126404287
660.14969	0.206341079	660.14969	0.126442539
660.374225	0.206395729	660.374225	0.126500132
661.582817	0.206690414	661.582817	0.126810215
662.074845	0.206810534	662.074845	0.126936643
662.14969	0.206828892	662.14969	0.126955668
662.358282	0.206879791	662.358282	0.127009379
662.374225	0.206883773	662.374225	0.127013303
663.582817	0.207179705	663.582817	0.127323399
664.14969	0.207318756	664.14969	0.127469045
664.374225	0.207373909	664.374225	0.127526497
665	0.207527663	665	0.127687265
665.374225	0.207619842	665.374225	0.127783079
665.582817	0.207671203	665.582817	0.127836608
666.374225	0.207866294	666.374225	0.128039707
667.074845	0.208039288	667.074845	0.128219701
667.358282	0.208109362	667.358282	0.128292442
667.582817	0.208164944	667.582817	0.128349856
668.374225	0.208360957	668.374225	0.128552951
668.582817	0.20841267	668.582817	0.12860649
669.14969	0.208553335	669.14969	0.128752172
669.582817	0.208660993	669.582817	0.128863111
670	0.20876475	670	0.128970384
670.374225	0.208857938	670.374225	0.129066205
671.582817	0.209159449	671.582817	0.129376361
672.074845	0.209282455	672.074845	0.129502873
672.358282	0.209353432	672.358282	0.129575609
672.374225	0.209357401	672.374225	0.12957947
673.582817	0.209660358	673.582817	0.129889651
674.14969	0.209802857	674.14969	0.130035371
674.374225	0.209859414	674.374225	0.130092779
675	0.210017005	675	0.130253616
675.374225	0.210111378	675.374225	0.130349423
676.374225	0.210364038	676.374225	0.130606123
677.074845	0.210541583	677.074845	0.130786224

Final State: j’=1		Final State: j’=0	
E(cm⁻¹)	σ (Å²)	E(cm⁻¹)	σ (Å²)
677.358282	0.210613571	677.358282	0.130859003
677.582817	0.210670488	677.582817	0.130916386
678.374225	0.210871713	678.374225	0.131119605
678.582817	0.210924749	678.582817	0.131173156
679.14969	0.211069414	679.14969	0.131318999
679.582817	0.211179907	679.582817	0.131429966
680	0.211286658	680	0.131537383
680.374225	0.211382413	680.374225	0.131633263
681.582817	0.211692671	681.582817	0.131943798
682.074845	0.211819436	682.074845	0.132070535
682.358282	0.211892546	682.358282	0.132143401
682.374225	0.211896547	682.374225	0.132147223
683.582817	0.212209059	683.582817	0.132458024
684.14969	0.212356221	684.14969	0.13260416
684.374225	0.212414496	684.374225	0.132661655
685	0.212577495	685	0.132823036
685.374225	0.212675028	685.374225	0.132919115
685.582817	0.212729516	685.582817	0.132972848
686.374225	0.212936711	686.374225	0.133176785
687.074845	0.213120883	687.074845	0.133357766
687.358282	0.213195522	687.358282	0.133430901
687.582817	0.213254608	687.582817	0.133488545
688.374225	0.213463851	688.374225	0.133692936
688.582817	0.213519145	688.582817	0.133746846
689.14969	0.213669882	689.14969	0.133893753
689.582817	0.213785166	689.582817	0.134005536
690	0.213896758	690	0.134113915
690.374225	0.213996848	690.374225	0.134210596
691.582817	0.214322318	691.582817	0.134524434
692.074845	0.214455818	692.074845	0.134652822
692.358282	0.214532872	692.358282	0.134726674
692.374225	0.214537048	692.374225	0.13473049
693.582817	0.214867808	693.582817	0.135046202
694.14969	0.215024472	694.14969	0.135195163
694.374225	0.215086527	694.374225	0.135253779
695	0.215261042	695	0.135418847
695.374225	0.215365743	695.374225	0.135517269
695.582817	0.215424436	695.582817	0.135572425
696.374225	0.215648743	696.374225	0.135782453
697.074845	0.215849967	697.074845	0.135969907
697.358282	0.215932031	697.358282	0.136045957
697.582817	0.215997197	697.582817	0.136105982
698.374225	0.216230194	698.374225	0.13632027
698.582817	0.216292364	698.582817	0.136377154
699.14969	0.216463604	699.14969	0.136533172

Final State: j'=1		Final State: j'=0	
E(cm⁻¹)	σ (Å²)	E(cm⁻¹)	σ (Å²)
699.582817	0.216596183	699.582817	0.13665286
700	0.216726582	700	0.136770125
700.374225	0.216845156	700.374225	0.136875713
701.582817	0.217248145	701.582817	0.137228916
702.074845	0.217424931	702.074845	0.137380348
702.358282	0.217531571	702.358282	0.137470251
702.374225	0.217537352	702.374225	0.137474918
703.582817	0.21807112	703.582817	0.137905687
704.14969	0.218427483	704.14969	0.138173137
704.374225	0.218617998	704.374225	0.138310103
705	0.218500455	705	0.138343119
705.374225	0.218638961	705.374225	0.138473979
705.582817	0.217657901	705.582817	0.137909188
706.374225	0.21849932	706.374225	0.138402602
707.074845	0.218380854	707.074845	0.138450337
707.358282	0.217539487	707.358282	0.137934794
707.582817	0.217984317	707.582817	0.138206711
708.374225	0.21906126	708.374225	0.138935543
708.582817	0.219375677	708.582817	0.139141454
709.14969	0.219294586	709.14969	0.139126815
709.582817	0.218374669	709.582817	0.138624454
710	0.218738503	710	0.138870886
710.374225	0.218344451	710.374225	0.138792175
711.582817	0.219425013	711.582817	0.139420551
712.074845	0.220152983	712.074845	0.139877114
712.358282	0.220666681	712.358282	0.140176561
713.582817	0.219742126	713.582817	0.139821096
714.14969	0.218055783	714.14969	0.138875258
714.374225	0.219025727	714.374225	0.13945136
715	0.220113568	715	0.14012957
715.374225	0.22049513	715.374225	0.140380054
715.582817	0.220709911	715.582817	0.140519983
716.374225	0.221939467	716.374225	0.141275764
717.074845	0.219895364	717.074845	0.140253407
717.358282	0.220787048	717.358282	0.140871216
717.582817	0.220319907	717.582817	0.140544064
718.374225	0.219818797	718.374225	0.140280048
718.582817	0.220389961	718.582817	0.14061759
719.14969	0.22106583	719.14969	0.141051851
719.582817	0.221421693	719.582817	0.141290859
720	0.221756109	720	0.141514736
720.374225	0.222094815	720.374225	0.141733699
721.582817	0.221475154	721.582817	0.141427804
722.074845	0.221772765	722.074845	0.141705089
722.358282	0.222736801	722.358282	0.142432394

Final State: j’=1		Final State: j’=0	
E(cm⁻¹)	σ (Å²)	E(cm⁻¹)	σ (Å²)
722.374225	0.222806165	722.374225	0.142481838
723.582817	0.221926855	723.582817	0.141942715
724.14969	0.222416326	724.14969	0.142267512
724.374225	0.222573722	724.374225	0.142374704
725	0.222991033	725	0.142660405
725.374225	0.223251453	725.374225	0.14283476
725.582817	0.223411422	725.582817	0.14293908
726.374225	0.224334386	726.374225	0.143480703
727.074845	0.222724749	727.074845	0.14244327
727.358282	0.222894277	727.358282	0.143030842
727.582817	0.223163934	727.582817	0.143012241
728.374225	0.222214219	728.374225	0.142676227
728.582817	0.222646283	728.582817	0.14279696
729.14969	0.223563777	729.14969	0.143360829
729.582817	0.223905194	729.582817	0.143587119
730	0.224177109	730	0.143773138
730.374225	0.224401463	730.374225	0.143928036
731.582817	0.225152996	731.582817	0.144436571
732.074845	0.225559172	732.074845	0.144689214
732.358282	0.225884072	732.358282	0.144874352
732.374225	0.225905085	732.374225	0.144885521
733.582817	0.225111831	733.582817	0.14471612
734.14969	0.224808525	734.14969	0.144483924
734.374225	0.224789148	734.374225	0.144466338
735	0.225302974	735	0.144854423
735.374225	0.225699927	735.374225	0.145105342
735.582817	0.225874416	735.582817	0.145219575
736.374225	0.226449849	736.374225	0.145606961
737.074845	0.226964568	737.074845	0.145957902
737.358282	0.227199972	737.358282	0.146117745
737.582817	0.227408636	737.582817	0.146258963
738.374225	0.228227451	738.374225	0.14690997
738.582817	0.227763882	738.582817	0.146618576
739.14969	0.226980641	739.14969	0.146103138
739.582817	0.228772607	739.582817	0.147009408
740	0.227762743	740	0.14647283
740.374225	0.226092944	740.374225	0.145855775
741.582817	0.227717191	741.582817	0.146785994
742.074845	0.227658132	742.074845	0.147033169
742.358282	0.226240062	742.358282	0.1461312
742.374225	0.226238741	742.374225	0.146153609
743.582817	0.228341708	743.582817	0.147199662
744.14969	0.229897748	744.14969	0.148191784
744.374225	0.228824621	744.374225	0.147791237
745	0.226132032	745	0.145996685

Final State: j’=1		Final State: j’=0	
E(cm⁻¹)	σ (Å²)	E(cm⁻¹)	σ (Å²)
745.374225	0.2276388	745.374225	0.146856957
745.582817	0.228117967	745.582817	0.147143232
746.374225	0.229623775	746.374225	0.148039276
747.074845	0.232292276	747.074845	0.149475273
747.358282	0.229680705	747.358282	0.147962867
747.582817	0.226891232	747.582817	0.147067549
748.374225	0.227826707	748.374225	0.147217284
748.582817	0.226593506	748.582817	0.146578031
749.14969	0.228837236	749.14969	0.14786625
749.582817	0.229545979	749.582817	0.148290724
750	0.230271525	750	0.148726752
750.374225	0.231241311	750.374225	0.149295837
751.582817	0.228350224	751.582817	0.147708694
752.074845	0.229543807	752.074845	0.148418196
752.358282	0.229966192	752.358282	0.148675676
752.374225	0.229986707	752.374225	0.148688026
753.582817	0.231478947	753.582817	0.149595581
754.14969	0.232533822	754.14969	0.150197533
754.374225	0.233278787	754.374225	0.150598265
755	0.229887279	755	0.148469705
755.374225	0.229121026	755.374225	0.14826995
755.582817	0.230469306	755.582817	0.149483264
756.374225	0.230680508	756.374225	0.149329335
757.074845	0.23210987	757.074845	0.15039624
757.358282	0.230971658	757.358282	0.149907353
757.582817	0.23180975	757.582817	0.150141568
758.374225	0.231445683	758.374225	0.149980541
758.582817	0.232971858	758.582817	0.150756475
759.14969	0.234024074	759.14969	0.151603808
759.582817	0.227935921	759.582817	0.148102215
760	0.231246674	760	0.149898812
760.374225	0.232669402	760.374225	0.150750783
761.582817	0.232380311	761.582817	0.150565779
762.074845	0.233689173	762.074845	0.151343192
762.358282	0.234575404	762.358282	0.15187363
762.374225	0.234638096	762.374225	0.151911102
763.582817	0.236398112	763.582817	0.152275801
764.14969	0.232145018	764.14969	0.150515223
764.374225	0.232508948	764.374225	0.150979203
765	0.230610433	765	0.149838861
765.374225	0.232250069	765.374225	0.150778837
765.582817	0.233966502	765.582817	0.151826826
766.374225	0.234135498	766.374225	0.151992872
767.074845	0.230990854	767.074845	0.15036055
767.358282	0.233677602	767.358282	0.15181168

Final State: j'=1		Final State: j'=0	
E(cm⁻¹)	σ (Å²)	E(cm⁻¹)	σ (Å²)
767.582817	0.234368226	767.582817	0.152201511
768.374225	0.236616382	768.374225	0.153332907
768.582817	0.236014476	768.582817	0.152804357
769.14969	0.233826172	769.14969	0.15207674
769.582817	0.234300272	769.582817	0.152341928
770	0.234697711	770	0.152568825
770.374225	0.234990874	770.374225	0.152738636
771.582817	0.235795742	771.582817	0.153186515
772.074845	0.236770644	772.074845	0.153519466
772.358282	0.235380134	772.358282	0.153044153
772.374225	0.235436518	772.374225	0.153108488
773.582817	0.235021959	773.582817	0.15298748
774.14969	0.235595604	774.14969	0.153317745
774.374225	0.235734579	774.374225	0.153399817
775	0.236040559	775	0.153588725
775.374225	0.236194869	775.374225	0.153686836
776.374225	0.236579443	776.374225	0.153933828
777.074845	0.2368238	777.074845	0.154089077
777.358282	0.236894841	777.358282	0.154136933
778.374225	0.237062712	778.374225	0.154283022
778.582817	0.237117467	778.582817	0.154324824
779.14969	0.237292068	779.14969	0.154447287
780	0.23761568	780	0.154648784
780.374225	0.2378322	780.374225	0.154755033
782.074845	0.237941005	782.074845	0.154970426
782.358282	0.238045948	782.358282	0.155036272
782.374225	0.238050431	782.374225	0.155038954
783.582817	0.238420282	783.582817	0.155287856
784.14969	0.238581338	784.14969	0.155399503
784.374225	0.238643643	784.374225	0.155442663
785	0.238820853	785	0.15556591
785.374225	0.238925142	785.374225	0.155638054
786.374225	0.23920738	786.374225	0.155832577
787.074845	0.239407818	787.074845	0.155969285
787.358282	0.23948917	787.358282	0.156024085
788.374225	0.239776925	788.374225	0.156213551
788.582817	0.239828898	788.582817	0.156248927
789.14969	0.239932186	789.14969	0.156334674
790	0.240141043	790	0.156493297
790.374225	0.240245907	790.374225	0.156565709
792.074845	0.240725061	792.074845	0.156891678
792.358282	0.240803807	792.358282	0.156944972
792.374225	0.240807116	792.374225	0.15694705
793.582817	0.241142868	793.582817	0.157173562
794.14969	0.2412994	794.14969	0.15727863

Final State: j'=1		Final State: j'=0	
E(cm⁻¹)	σ (Å²)	E(cm⁻¹)	σ (Å²)
794.374225	0.241360226	794.374225	0.157319195
795	0.241533911	795	0.157435358
795.374225	0.241635727	795.374225	0.157503042
796.374225	0.241909595	796.374225	0.157685045
797.074845	0.242100926	797.074845	0.157812329
797.358282	0.242177519	797.358282	0.157863241
798.374225	0.242450542	798.374225	0.158044446
798.582817	0.242508194	798.582817	0.158082769
799.14969	0.242661967	799.14969	0.1581843
800	0.242893553	800	0.158336393
800.374225	0.24299455	800.374225	0.158402234
802.074845	0.243461634	802.074845	0.158705395
802.358282	0.243539321	802.358282	0.158755524
802.374225	0.243542482	802.374225	0.158757355
803.582817	0.243875566	803.582817	0.158971185
804.14969	0.244031296	804.14969	0.159070504
804.374225	0.244091717	804.374225	0.15910877
805	0.24426492	805	0.159218897
805.374225	0.244366458	805.374225	0.159282978
806.374225	0.24464119	806.374225	0.159456186
807.074845	0.244834985	807.074845	0.159577963
807.358282	0.244912873	807.358282	0.159626689
808.374225	0.245190794	808.374225	0.159799711
808.582817	0.245249468	808.582817	0.15983634
809.14969	0.245405372	809.14969	0.159932929
810	0.245639362	810	0.160077264
810.374225	0.24574105	810.374225	0.160139549
812.074845	0.246211045	812.074845	0.160426688
812.358282	0.246289221	812.358282	0.160474113
813.582817	0.24662718	813.582817	0.160678151
814.14969	0.246783737	814.14969	0.16077213
814.374225	0.246844375	814.374225	0.160808219
815	0.247018712	815	0.160912535
815.374225	0.247120764	815.374225	0.16097305
816.374225	0.247397342	816.374225	0.161136937
817.074845	0.247592662	817.074845	0.161252262
817.358282	0.247671133	817.358282	0.161298354
818.374225	0.247951075	818.374225	0.16146186
818.582817	0.248010316	818.582817	0.161496606
819.14969	0.248167433	819.14969	0.161587901
820	0.248403221	820	0.16172427
820.374225	0.248505568	820.374225	0.161782978
822.074845	0.248979169	822.074845	0.162054128
822.358282	0.249057902	822.358282	0.162098869
822.374225	0.249060847	822.374225	0.162100282

Final State: j'=1		Final State: j'=0	
E(cm⁻¹)	σ (Å²)	E(cm⁻¹)	σ (Å²)
823.582817	0.249398315	823.582817	0.162291288
824.14969	0.249555958	824.14969	0.162379876
824.374225	0.249616915	824.374225	0.16241377
825	0.2497926	825	0.162512186
825.374225	0.249895262	825.374225	0.162569074
826.374225	0.250173799	826.374225	0.162723422
827.074845	0.250370618	827.074845	0.162832119
827.358282	0.250449652	827.358282	0.1628755
828.374225	0.250731481	828.374225	0.163029227
828.582817	0.250791264	828.582817	0.163062041
829.14969	0.250949505	829.14969	0.163147905
830	0.251186992	830	0.163276121
830.374225	0.251289962	830.374225	0.163331178
832.074845	0.251767076	832.074845	0.163586043
832.358282	0.251846388	832.358282	0.163628054
832.374225	0.251849232	832.374225	0.163629254
833.582817	0.252189212	833.582817	0.163808677
834.14969	0.252348016	834.14969	0.163891791
834.374225	0.252409289	834.374225	0.163923451
835	0.252586342	835	0.164015881
835.374225	0.252689617	835.374225	0.164069094
836.374225	0.252970138	836.374225	0.164213757
837.074845	0.253168474	837.074845	0.16431573
837.358282	0.253248055	837.358282	0.164356369
838.374225	0.253531779	838.374225	0.164500163
838.582817	0.253592081	838.582817	0.16453102
839.14969	0.253751447	839.14969	0.164611374
840	0.253990601	840	0.164731321
840.374225	0.254094179	840.374225	0.164782677
842.074845	0.254574759	842.074845	0.165021038
842.358282	0.25465462	842.358282	0.165060286
842.374225	0.254657365	842.374225	0.165061271
843.582817	0.254999839	843.582817	0.165228954
844.14969	0.255159744	844.14969	0.165306535
844.374225	0.255221326	844.374225	0.165335942
845	0.255399722	845	0.165422318
845.374225	0.255503595	845.374225	0.165471809
846.374225	0.255786063	846.374225	0.165606692
847.074845	0.255985867	847.074845	0.165701856
847.358282	0.256066	847.358282	0.165739716
848.374225	0.25635153	848.374225	0.165873511
848.582817	0.25641238	848.582817	0.16590237
849.14969	0.256572825	849.14969	0.165977167
850	0.256813603	850	0.166088771
850.374225	0.256917762	850.374225	0.166136393

Final State: j'=1		Final State: j'=0	
E(cm⁻¹)	σ (Å²)	E(cm⁻¹)	σ (Å²)
852.074845	0.257401697	852.074845	0.166358096
852.358282	0.257482089	852.358282	0.166394568
852.374225	0.25748473	852.374225	0.166395336
853.582817	0.257829607	853.582817	0.166551185
854.14969	0.257990576	854.14969	0.166623191
854.374225	0.258052442	854.374225	0.166650331
855	0.258232137	855	0.1667306
855.374225	0.258336559	855.374225	0.16677635
856.374225	0.258620871	856.374225	0.166901387
857.074845	0.258822111	857.074845	0.166989701
857.358282	0.258902768	857.358282	0.167024765
858.374225	0.259190014	858.374225	0.167148488
858.582817	0.259251387	858.582817	0.167175343
859.14969	0.259412855	859.14969	0.16724455
860	0.259655184	860	0.167347765
860.374225	0.259759857	860.374225	0.167391637
862.074845	0.260246978	862.074845	0.167596616
862.358282	0.26032788	862.358282	0.16763028
862.374225	0.260330412	862.374225	0.167630841
863.582817	0.260677556	863.582817	0.167774812
864.14969	0.260839518	864.14969	0.167841217
864.374225	0.260901638	864.374225	0.167866087
865	0.261082561	865	0.167940229
865.374225	0.261187501	865.374225	0.167982231
866.374225	0.261473532	866.374225	0.168097386
867.074845	0.26167612	867.074845	0.168178833
867.358282	0.261757252	867.358282	0.168211096
868.374225	0.262046087	868.374225	0.168324728
868.582817	0.262107941	868.582817	0.168349578
869.14969	0.262270372	869.14969	0.168413183
870	0.262514113	870	0.168508
870.374225	0.262619284	870.374225	0.168548126
872.074845	0.263109331	872.074845	0.168736354
872.358282	0.263190695	872.358282	0.168767219
872.374225	0.263193111	872.374225	0.168767569
873.582817	0.263542349	873.582817	0.168899655
874.14969	0.263705216	874.14969	0.168960461
874.374225	0.263767554	874.374225	0.168983066
875	0.263949616	875	0.169051079
875.374225	0.264055002	875.374225	0.169089335
876.374225	0.264342596	876.374225	0.169194619
877.074845	0.264546401	877.074845	0.169269201
877.358282	0.264627981	877.358282	0.169298671
878.374225	0.264918227	878.374225	0.169402231
878.582817	0.264980544	878.582817	0.169425069

Final State: j'=1		Final State: j'=0	
E(cm⁻¹)	σ (Å²)	E(cm⁻¹)	σ (Å²)
879.14969	0.265143828	879.14969	0.169483084
880	0.265388838	880	0.169569511
880.374225	0.265494423	880.374225	0.1696059
882.074845	0.26598711	882.074845	0.169777417
882.358282	0.266068881	882.358282	0.169805489
882.374225	0.266071177	882.374225	0.169805635
883.582817	0.266422288	883.582817	0.16992587
884.14969	0.266585963	884.14969	0.169981088
884.374225	0.26664847	884.374225	0.170001436
885	0.266831546	885	0.170063339
885.374225	0.266937313	885.374225	0.170097865
886.374225	0.267226282	886.374225	0.17019331
887.074845	0.267431179	887.074845	0.17026105
887.358282	0.267513157	887.358282	0.170287731
888.374225	0.267804609	888.374225	0.170381262
888.582817	0.267867352	888.582817	0.170402098
889.14969	0.26803138	889.14969	0.170454544
890	0.268277506	890	0.170532621
890.374225	0.268383426	890.374225	0.170565294
892.074845	0.268878418	892.074845	0.170720169
892.358282	0.268960566	892.358282	0.170745462
892.374225	0.268962734	892.374225	0.170745409
893.582817	0.269315484	893.582817	0.170853836
894.14969	0.269479842	894.14969	0.170903508
894.374225	0.269542479	894.374225	0.170921615
895	0.269726465	895	0.170977425
895.374225	0.269832533	895.374225	0.171008246
896.374225	0.270122697	896.374225	0.171093908
897.074845	0.270328586	897.074845	0.171154829
897.358282	0.270410864	897.358282	0.171178739
898.374225	0.270703355	898.374225	0.171262291
898.582817	0.270766499	898.582817	0.171281127
899.14969	0.270931181	899.14969	0.171328028
900	0.271178262	900	0.171397798
900.374225	0.27128446	900.374225	0.171426763
902.074845	0.271781531	902.074845	0.171565067
902.358282	0.271863975	902.358282	0.171587589
902.374225	0.271866001	902.374225	0.171587341
903.582817	0.27222027	903.582817	0.171683995
904.14969	0.272385277	904.14969	0.171728127
904.374225	0.27244801	904.374225	0.171744003
905	0.272632876	905	0.171793733
905.374225	0.272739173	905.374225	0.171820858
906.374225	0.273030466	906.374225	0.171896734
907.074845	0.273237286	907.074845	0.171950838

Final State: j'=1		Final State: j'=0	
E(cm⁻¹)	σ (Å²)	E(cm⁻¹)	σ (Å²)
907.358282	0.273319908	907.358282	0.171971969
908.374225	0.27361342	908.374225	0.172045527
908.582817	0.273676942	908.582817	0.172062352
909.14969	0.273842306	909.14969	0.172103692
910	0.274090451	910	0.172165096
910.374225	0.274199674	910.374225	0.172191891
912.074845	0.274696431	912.074845	0.172311875
912.358282	0.274779277	912.358282	0.172331587
913.582817	0.27513743	913.582817	0.172415796
914.14969	0.275303349	914.14969	0.172454245
915	0.275552423	915	0.17251128
915.374225	0.275662126	915.374225	0.172536136
917.074845	0.276161482	917.074845	0.172647083
917.358282	0.276244869	917.358282	0.172665243
918.582817	0.276605904	918.582817	0.172742543
919.14969	0.276773619	919.14969	0.172777627
920	0.277026221	920	0.172829323
920.374225	0.27713794	920.374225	0.172851663
922.074845	0.277655342	922.074845	0.172948777
922.358282	0.277745135	922.358282	0.17296391
923.582817	0.278256743	923.582817	0.173038602
924.14969	0.278504558	924.14969	0.173136735
925	0.278506523	925	0.173176411
925.374225	0.27866579	925.374225	0.173173292
927.074845	0.279101971	927.074845	0.173270268
927.358282	0.279214746	927.358282	0.173274525
928.582817	0.27964969	928.582817	0.173532487
929.14969	0.279868885	929.14969	0.173426424
930	0.279985917	930	0.173567769
930.374225	0.280031017	930.374225	0.17347825
932.074845	0.280544393	932.074845	0.173645767
932.358282	0.280613327	932.358282	0.173599109
933.582817	0.280922904	933.582817	0.173656052
934.14969	0.281118267	934.14969	0.173663556
935	0.28146261	935	0.173696698
935.374225	0.281887582	935.374225	0.173793492
937.074845	0.282374632	937.074845	0.174021226
937.358282	0.28198648	937.358282	0.173866043
938.582817	0.282394536	938.582817	0.173876008
939.14969	0.282587058	939.14969	0.173895408
940	0.28306786	940	0.173970441
940.374225	0.283387175	940.374225	0.174248398
942.074845	0.283342004	942.074845	0.174075687
942.358282	0.28344131	942.358282	0.174064958
943.582817	0.283836372	943.582817	0.174093775

Final State: j'=1		Final State: j'=0	
E(cm⁻¹)	σ (Å²)	E(cm⁻¹)	σ (Å²)
944.14969	0.284017762	944.14969	0.174112691
945	0.284315314	945	0.17414569
945.374225	0.284510929	945.374225	0.174177163
947.074845	0.285067468	947.074845	0.174398467
947.358282	0.284868803	947.358282	0.174320749
948.582817	0.285273927	948.582817	0.174288921
949.14969	0.285451028	949.14969	0.174304944
950	0.285716543	950	0.174330872
950.374225	0.285835557	950.374225	0.174342758
952.074845	0.286724019	952.074845	0.174622687
952.358282	0.286475689	952.358282	0.174461691
953.582817	0.286697205	953.582817	0.1744759
954.14969	0.286901188	954.14969	0.174467406
955	0.287179674	955	0.174482314
955.374225	0.287302069	955.374225	0.174489517
957.074845	0.288012344	957.074845	0.174519338
957.358282	0.288302187	957.358282	0.174656968
958.582817	0.288621208	958.582817	0.174704371
959.14969	0.289257888	959.14969	0.175003267
960	0.288950029	960	0.174763514
960.374225	0.28880375	960.374225	0.174674394
962.074845	0.289183335	962.074845	0.17475206
962.358282	0.289308372	962.358282	0.174735028
963.582817	0.29080205	963.582817	0.175228127
964.14969	0.289720237	964.14969	0.174869295
965	0.290053275	965	0.174813111
965.374225	0.290229614	965.374225	0.174809263
967.074845	0.290614612	967.074845	0.174945184
967.358282	0.291047013	967.358282	0.175085172
968.582817	0.291051291	968.582817	0.174872571
969.14969	0.291342095	969.14969	0.174869635
970	0.292182672	970	0.175475608
970.374225	0.291437226	970.374225	0.175032471
972.074845	0.292046266	972.074845	0.174941709
972.358282	0.292151633	972.358282	0.174941133
973.582817	0.293511405	973.582817	0.175289683
974.14969	0.293086507	974.14969	0.175298524
975	0.293061885	975	0.174961548
975.374225	0.292966452	975.374225	0.175004254
977.074845	0.294368153	977.074845	0.17529604
977.358282	0.293649986	977.358282	0.175110819
978.582817	0.293763951	978.582817	0.175231188
979.14969	0.294034365	979.14969	0.175066766
980	0.294351371	980	0.175398626
980.374225	0.294314466	980.374225	0.175146633

Final State: j'=1		Final State: j'=0	
E(cm⁻¹)	σ (Å²)	E(cm⁻¹)	σ (Å²)
982.074845	0.294932184	982.074845	0.175183544
982.358282	0.295362989	982.358282	0.175261024
983.582817	0.295854618	983.582817	0.175047462
984.14969	0.295297607	984.14969	0.175275986
985	0.295789164	985	0.175098225
985.374225	0.295953165	985.374225	0.175154175
987.074845	0.296603625	987.074845	0.175242183
987.358282	0.297108838	987.358282	0.175359088
988.582817	0.296987468	988.582817	0.175291487
989.14969	0.297157392	989.14969	0.175289799
990	0.297436788	990	0.175308167
990.374225	0.297560926	990.374225	0.175320272
992.074845	0.298182379	992.074845	0.175419534
992.358282	0.298161254	992.358282	0.175370898
993.582817	0.298464917	993.582817	0.175344022
994.14969	0.298631576	994.14969	0.175348972
995	0.298882394	995	0.17535738
995.374225	0.298994483	995.374225	0.17536205
997.074845	0.299504373	997.074845	0.175386561
997.358282	0.299581559	997.358282	0.175386251
998.582817	0.299923995	998.582817	0.175388849
999.14969	0.300091834	999.14969	0.175396565
1000	0.300374196	1000	0.175406894
1000.37423	0.300415779	1000.37423	0.175385712
1002.07485	0.30090818	1002.07485	0.175391763
1002.35828	0.300988879	1002.35828	0.175392434
1003.58282	0.301335758	1003.58282	0.175393964
1004.14969	0.301495886	1004.14969	0.1753942
1005	0.301735991	1005	0.175394268
1005.37423	0.301841632	1005.37423	0.175394186
1007.07485	0.302325245	1007.07485	0.17539507
1007.35828	0.302407829	1007.35828	0.17539591
1008.58282	0.302749676	1008.58282	0.175390441
1009.14969	0.302905041	1009.14969	0.17538715
1010	0.30314103	1010	0.175383389
1010.37423	0.303245207	1010.37423	0.175381766
1012.07485	0.303719097	1012.07485	0.175373893
1012.35828	0.303798042	1012.35828	0.175372447
1013.58282	0.30413892	1013.58282	0.175365743
1014.14969	0.304296632	1014.14969	0.175362394
1015	0.304533139	1015	0.175357092
1015.37423	0.304637108	1015.37423	0.175354588
1017.07485	0.3051085	1017.07485	0.175341948
1017.35828	0.305186818	1017.35828	0.175339623
1018.58282	0.305524614	1018.58282	0.175329019

Final State: j’=1		Final State: j’=0	
E(cm⁻¹)	σ (Å²)	E(cm⁻¹)	σ (Å²)
1019.14969	0.305680748	1019.14969	0.175323833
1020	0.305914739	1020	0.175315769
1020.37423	0.306017577	1020.37423	0.175312063
1022.07485	0.306484369	1022.07485	0.175294426
1022.35828	0.306562052	1022.35828	0.175291338
1023.58282	0.306897278	1023.58282	0.175277515
1024.14969	0.307052238	1024.14969	0.175270843
1025	0.307284444	1025	0.175260542
1025.37423	0.307386472	1025.37423	0.175255845
1027.07485	0.307849423	1027.07485	0.175233662
1027.35828	0.307926475	1027.35828	0.175229838
1028.58282	0.308258685	1028.58282	0.175212694
1029.14969	0.308412233	1029.14969	0.175204502
1030	0.308642282	1030	0.175191924
1030.37423	0.308743351	1030.37423	0.175186232
1032.07485	0.309201916	1032.07485	0.175159579
1032.35828	0.309278212	1032.35828	0.175155023
1033.58282	0.309607278	1033.58282	0.175134724
1034.14969	0.309759338	1034.14969	0.175125087
1035	0.309987187	1035	0.175110243
1035.37423	0.310087228	1035.37423	0.175103717
1037.07485	0.310541274	1037.07485	0.175072815
1037.35828	0.31061682	1037.35828	0.175067553
1038.58282	0.31094253	1038.58282	0.175044225
1039.14969	0.311093045	1039.14969	0.175033194
1040	0.311318505	1040	0.175016385
1040.37423	0.311417543	1040.37423	0.175008839
1042.07485	0.311866787	1042.07485	0.174973835
1042.35828	0.311941535	1042.35828	0.174967895
1043.58282	0.312263721	1043.58282	0.174941662
1044.14969	0.312412599	1044.14969	0.174929294
1045	0.312635571	1045	0.174910493
1045.37423	0.312733511	1045.37423	0.174902077
1047.07485	0.313177704	1047.07485	0.174863145
1047.35828	0.313251608	1047.35828	0.174856556
1048.58282	0.313570101	1048.58282	0.174827539
1049.14969	0.313717247	1049.14969	0.174813894
1050	0.313937632	1050	0.174793185
1050.37423	0.314034422	1050.37423	0.174783934
1052.07485	0.314473347	1052.07485	0.174741245
1052.35828	0.314546359	1052.35828	0.174734036
1053.58282	0.314860997	1053.58282	0.174702356
1054.14969	0.315006341	1054.14969	0.174687488
1055	0.315224009	1055	0.174664958
1055.37423	0.315319596	1055.37423	0.174654909

Final State: j’=1		Final State: j’=0	
E(cm⁻¹)	σ (Å²)	E(cm⁻¹)	σ (Å²)
1057.07485	0.315753019	1057.07485	0.174608633
1057.35828	0.315825099	1057.35828	0.174600833
1058.58282	0.316135694	1058.58282	0.17456661
1059.14969	0.316279163	1059.14969	0.174550574
1060	0.316493995	1060	0.174526308
1060.37423	0.31658833	1060.37423	0.174515499
1062.07485	0.317016016	1062.07485	0.174465806
1062.35828	0.317087096	1062.35828	0.174457436
1063.58282	0.317393524	1063.58282	0.174420797
1064.14969	0.317535035	1064.14969	0.17440365
1065	0.317746919	1065	0.174377728
1065.37423	0.31783994	1065.37423	0.174366198
1067.07485	0.318261626	1067.07485	0.174313249
1067.35828	0.318331749	1067.35828	0.174304346
1068.58282	0.318633802	1068.58282	0.174265406
1069.14969	0.318773287	1069.14969	0.174247209
1070	0.318982112	1070	0.174219715
1070.37423	0.319073777	1070.37423	0.174207497
1072.07485	0.319489264	1072.07485	0.174151465
1072.35828	0.319558348	1072.35828	0.174142053
1073.58282	0.319855884	1073.58282	0.174100933
1074.14969	0.319993259	1074.14969	0.174081734
1075	0.320198923	1075	0.174052756
1075.37423	0.320289173	1075.37423	0.174039888
1077.07485	0.32069826	1077.07485	0.173980934
1077.35828	0.320766271	1077.35828	0.173971039
1078.58282	0.321059138	1078.58282	0.17392786
1079.14969	0.32119434	1079.14969	0.173907718
1080	0.321396714	1080	0.173877334
1080.37423	0.321485536	1080.37423	0.173863854
1082.07485	0.321888038	1082.07485	0.17380217
1082.35828	0.321954891	1082.35828	0.173791797
1083.58282	0.322242934	1083.58282	0.173746674
1084.14969	0.322375892	1084.14969	0.173725643
1085	0.322574889	1085	0.173693935
1085.37423	0.322662216	1085.37423	0.173679875
1087.07485	0.32305789	1087.07485	0.1736156
1087.35828	0.3231236	1087.35828	0.173604801
1088.58282	0.323406666	1088.58282	0.17355785
1089.14969	0.323537322	1089.14969	0.173535981
1090	0.323732838	1090	0.173503032
1090.37423	0.323818627	1090.37423	0.17348843
1092.07485	0.324207277	1092.07485	0.173421723
1092.35828	0.324271807	1092.35828	0.173410523
1093.58282	0.324549774	1093.58282	0.173361862

Final State: j'=1		Final State: j'=0	
E(cm⁻¹)	σ (Å²)	E(cm⁻¹)	σ (Å²)
1094.14969	0.324678044	1094.14969	0.17333921
1095	0.324869988	1095	0.173305098
1095.37423	0.324954196	1095.37423	0.173289987
1097.07485	0.32533563	1097.07485	0.173221006
1097.35828	0.325398952	1097.35828	0.173209433
1098.58282	0.325671672	1098.58282	0.173159173
1099.14969	0.325797507	1099.14969	0.173135792
1100	0.325985766	1100	0.173100599
1100.37423	0.326068367	1100.37423	0.173085013
1102.07485	0.326442402	1102.07485	0.173013911
1102.35828	0.326504484	1102.35828	0.173001991
1103.58282	0.326771834	1103.58282	0.172950245
1104.14969	0.326895168	1104.14969	0.172926184
1105	0.327079681	1105	0.17288998
1105.37423	0.327160611	1105.37423	0.172873958
1107.07485	0.327527069	1107.07485	0.172800892
1107.35828	0.327587883	1107.35828	0.17278865
1108.58282	0.327849731	1108.58282	0.172735528
1109.14969	0.327970512	1109.14969	0.17271084
1110	0.328151181	1110	0.172673703
1110.37423	0.328230411	1110.37423	0.172657276
1112.07485	0.328589129	1112.07485	0.172582399
1112.35828	0.328648647	1112.35828	0.172569857
1113.58282	0.328904878	1113.58282	0.172515468
1114.14969	0.329023048	1114.14969	0.172490202
1115	0.329199791	1115	0.172452208
1115.37423	0.32927729	1115.37423	0.172435407
1117.07485	0.329628103	1117.07485	0.172358864
1117.35828	0.329686296	1117.35828	0.172346051
1118.58282	0.329936798	1118.58282	0.172290498
1119.14969	0.330052304	1119.14969	0.172264702
1120	0.330225048	1120	0.172225925
1120.37423	0.330300778	1120.37423	0.172208783
1122.07485	0.330643534	1122.07485	0.17213072
1122.35828	0.330700383	1122.35828	0.172117659
1123.58282	0.330945042	1123.58282	0.172061048
1124.14969	0.331057838	1124.14969	0.17203477
1125	0.331226504	1125	0.171995282
1125.37423	0.331300435	1125.37423	0.17197783
1127.07485	0.331634987	1127.07485	0.171898389
1127.35828	0.331690468	1127.35828	0.171885102
1128.58282	0.331929185	1128.58282	0.171827533
1129.14969	0.332039223	1129.14969	0.171800821
1130	0.332203742	1130	0.171760689
1130.37423	0.332275843	1130.37423	0.17174296

Final State: j'=1		Final State: j'=0	
E(cm⁻¹)	σ (Å²)	E(cm⁻¹)	σ (Å²)
1132.07485	0.332602058	1132.07485	0.17166228
1132.35828	0.332656141	1132.35828	0.171648789
1133.58282	0.332888816	1133.58282	0.171590363
1134.14969	0.332996048	1134.14969	0.17156326
1135	0.333156351	1135	0.171522555
1135.37423	0.333226587	1135.37423	0.171504575
1137.07485	0.333544327	1137.07485	0.171422791
1137.35828	0.33359699	1137.35828	0.171409121
1138.58282	0.33382353	1138.58282	0.171349932
1139.14969	0.333927919	1139.14969	0.171322485
1140	0.334083929	1140	0.171281271
1140.37423	0.334152289	1140.37423	0.171263071
1142.07485	0.334461401	1142.07485	0.171180311
1142.35828	0.334512629	1142.35828	0.171166483
1143.58282	0.334732926	1143.58282	0.171106625
1144.14969	0.334834409	1144.14969	0.171078875
1145	0.334986075	1145	0.171037217
1145.37423	0.335052502	1145.37423	0.171018823
1147.07485	0.335352859	1147.07485	0.170935214
1147.35828	0.335402616	1147.35828	0.170921249
1148.58282	0.335616559	1148.58282	0.170860811
1149.14969	0.335715093	1149.14969	0.170832798
1150	0.335862314	1150	0.170790753
1150.37423	0.335926783	1150.37423	0.170772194
1152.07485	0.336218188	1152.07485	0.170687853
1152.35828	0.336266449	1152.35828	0.170673771
1153.58282	0.33647388	1153.58282	0.170612833
1154.14969	0.336569386	1154.14969	0.170584597
1155	0.336712047	1155	0.170542224
1155.37423	0.336774495	1155.37423	0.170523525
1157.07485	0.337056654	1157.07485	0.170438558
1157.35828	0.337103352	1157.35828	0.170424373
1158.58282	0.337304007	1158.58282	0.170363009
1159.14969	0.337396335	1159.14969	0.170334582
1160	0.337534176	1160	0.170291925
1160.37423	0.337594479	1160.37423	0.170273102
1162.07485	0.337866665	1162.07485	0.170187593
1162.35828	0.337911672	1162.35828	0.170173318
1163.58282	0.338104713	1163.58282	0.17011157
1164.14969	0.338193427	1164.14969	0.170082984
1165	0.338325438	1165	0.170040029
1165.37423	0.338383066	1165.37423	0.170021082
1167.07485	0.338641268	1167.07485	0.169934935
1167.35828	0.338683433	1167.35828	0.169920533
1168.58282	0.338860024	1168.58282	0.169858001

Final State: j’=1		Final State: j’=0	
E(cm⁻¹)	σ (Å²)	E(cm⁻¹)	σ (Å²)
1169.14969	0.338934997	1169.14969	0.169828637
1170	0.339010123	1170	0.169766423
1170.37423	0.339090173	1170.37423	0.169755791
1172.07485	0.339331436	1172.07485	0.169652061
1172.35828	0.339556989	1172.35828	0.169652388
1173.58282	0.339621836	1173.58282	0.169602787
1174.14969	0.339735296	1174.14969	0.169562008
1175	0.339902983	1175	0.169532699
1175.37423	0.339886948	1175.37423	0.169511587
1177.07485	0.340146502	1177.07485	0.169423505
1177.35828	0.34016179	1177.35828	0.169404165
1178.58282	0.340408071	1178.58282	0.169352276
1179.14969	0.340527398	1179.14969	0.169315456
1180	0.340609758	1180	0.169280289
1180.37423	0.340647473	1180.37423	0.169259954
1182.07485	0.340962308	1182.07485	0.169177284
1182.35828	0.340942882	1182.35828	0.169163818
1183.58282	0.341152159	1183.58282	0.169102416
1184.14969	0.34119572	1184.14969	0.169071972
1185	0.3412891	1185	0.169025918
1185.37423	0.341329471	1185.37423	0.169004933
1187.07485	0.341622799	1187.07485	0.168923567
1187.35828	0.341577782	1187.35828	0.168894579
1188.58282	0.341810596	1188.58282	0.168851141
1189.14969	0.34186717	1189.14969	0.168820446
1190	0.341962835	1190	0.168774768
1190.37423	0.342004986	1190.37423	0.168754369
1192.07485	0.342266926	1192.07485	0.168640975
1192.35828	0.342306637	1192.35828	0.168666442
1193.58282	0.34250881	1193.58282	0.168604286
1194.14969	0.342522769	1194.14969	0.168571798
1195	0.342610874	1195	0.168525912
1195.37423	0.342652239	1195.37423	0.168505959
1197.07485	0.342837037	1197.07485	0.168413082
1197.35828	0.342863901	1197.35828	0.168395714
1198.58282	0.343073706	1198.58282	0.168349115
1199.14969	0.343170336	1199.14969	0.16832022
1200	0.343235074	1200	0.168280807
1200.37423	0.343263366	1200.37423	0.168259597
1202.07485	0.343424434	1202.07485	0.168168873
1202.35828	0.343449092	1202.35828	0.168153833
1203.58282	0.343551877	1203.58282	0.168066851
1204.14969	0.343712836	1204.14969	0.168016041
1205	0.343738495	1205	0.167967556
1205.37423	0.343868876	1205.37423	0.167969114

Final State: j’=1		Final State: j’=0	
E(cm⁻¹)	σ (Å²)	E(cm⁻¹)	σ (Å²)
1207.07485	0.34389267	1207.07485	0.167879688
1207.35828	0.344142156	1207.35828	0.167868531
1208.58282	0.344215372	1208.58282	0.167839757
1209.14969	0.344186972	1209.14969	0.167801998
1210	0.344593274	1210	0.167758787
1210.37423	0.344505063	1210.37423	0.16774916
1212.07485	0.344477198	1212.07485	0.167630715
1212.35828	0.344478799	1212.35828	0.167541238
1213.58282	0.344927639	1213.58282	0.167589547
1214.14969	0.344880094	1214.14969	0.167573766
1215	0.344907844	1215	0.167522433
1215.37423	0.344904435	1215.37423	0.167496442
1217.07485	0.345201992	1217.07485	0.167428109
1217.35828	0.345210911	1217.35828	0.167412113
1218.58282	0.345276403	1218.58282	0.167342806
1219.14969	0.345295053	1219.14969	0.167306146
1220	0.34563344	1220	0.167234159
1220.37423	0.345638454	1220.37423	0.167305069
1222.07485	0.345630972	1222.07485	0.167179973
1222.35828	0.345496121	1222.35828	0.16714851
1223.58282	0.345811287	1223.58282	0.167085927
1224.14969	0.345604169	1224.14969	0.166982912
1225	0.346037938	1225	0.167044386
1225.37423	0.345986658	1225.37423	0.167021966
1227.07485	0.346132169	1227.07485	0.166928531
1227.35828	0.346115426	1227.35828	0.166910601
1228.58282	0.346174795	1228.58282	0.166796393
1229.14969	0.346559559	1229.14969	0.166835514
1230	0.346841217	1230	0.166679902
1230.37423	0.34661673	1230.37423	0.166786347
1232.07485	0.34635242	1232.07485	0.166639621
1232.35828	0.346678841	1232.35828	0.166676198
1233.58282	0.346575781	1233.58282	0.166546831
1234.14969	0.346808336	1234.14969	0.166589448
1235	0.346856957	1235	0.166540668
1235.37423	0.34688173	1235.37423	0.16652006
1237.07485	0.347001305	1237.07485	0.166418399
1237.35828	0.347044222	1237.35828	0.166456497
1238.58282	0.347181785	1238.58282	0.166375194
1239.14969	0.347213021	1239.14969	0.166343371
1240	0.347275801	1240	0.166299171
1240.37423	0.347304763	1240.37423	0.166280388
1242.07485	0.347438222	1242.07485	0.166196085
1242.35828	0.347461206	1242.35828	0.166182283
1243.58282	0.347562352	1243.58282	0.166125996

Final State: j'=1		Final State: j'=0	
E(cm^{-1})	σ (Å^2)	E(cm^{-1})	σ (Å^2)
1244.14969	0.347606555	1244.14969	0.166099295
1245	0.347670988	1245	0.166058035
1245.37423	0.347698122	1245.37423	0.166038817
1247.07485	0.347835452	1247.07485	0.165965063
1247.35828	0.347855994	1247.35828	0.165951248
1248.58282	0.347946627	1248.58282	0.16589326
1249.14969	0.347988607	1249.14969	0.165866778
1250	0.348050984	1250	0.165827151
1250.37423	0.348078164	1250.37423	0.165809717
1252.07485	0.348200509	1252.07485	0.165730794
1252.35828	0.348220628	1252.35828	0.165717651
1253.58282	0.34830691	1253.58282	0.165661132
1254.14969	0.348347646	1254.14969	0.165635962
1255	0.348407188	1255	0.165597449
1255.37423	0.348432699	1255.37423	0.165580235
1257.07485	0.348547655	1257.07485	0.165502396
1257.35828	0.348566591	1257.35828	0.165489469
1258.58282	0.348647594	1258.58282	0.165433743
1259.14969	0.348684783	1259.14969	0.165408048
1260	0.348739965	1260	0.165369557
1260.37423	0.34876399	1260.37423	0.165352628
1262.07485	0.348872238	1262.07485	0.165276113
1262.35828	0.348890016	1262.35828	0.165263411
1263.58282	0.348966121	1263.58282	0.16520861
1264.14969	0.34900103	1264.14969	0.165183327
1265	0.349052769	1265	0.165145427
1265.37423	0.349075271	1265.37423	0.165128747
1267.07485	0.349176488	1267.07485	0.165053294
1267.35828	0.349193115	1267.35828	0.16504075
1268.58282	0.349264139	1268.58282	0.164986646
1269.14969	0.349296715	1269.14969	0.164961703
1270	0.349344997	1270	0.16492433
1270.37423	0.349365988	1270.37423	0.16490789
1272.07485	0.349460423	1272.07485	0.164833545
1272.35828	0.349475931	1272.35828	0.164821189
1273.58282	0.349542208	1273.58282	0.164767925
1274.14969	0.349572496	1274.14969	0.164743334
1275	0.349617464	1275	0.164706528
1275.37423	0.349637001	1275.37423	0.164690336
1277.07485	0.349724863	1277.07485	0.16461712
1277.35828	0.34973928	1277.35828	0.164604949
1278.58282	0.349800868	1278.58282	0.164552498
1279.14969	0.349828997	1279.14969	0.164528286
1280	0.349870743	1280	0.164492046
1280.37423	0.349888865	1280.37423	0.164476106

Final State: j’=1		Final State: j’=0	
E(cm⁻¹)	σ (Å²)	E(cm⁻¹)	σ (Å²)
1282.07485	0.349970358	1282.07485	0.164404033
1282.35828	0.349983722	1282.35828	0.164392056
1283.58282	0.350040781	1283.58282	0.164340435
1285	0.350105465	1285	0.16428095
1285.37423	0.350122226	1285.37423	0.164265265
1287.07485	0.350197574	1287.07485	0.16419436
1287.35828	0.35020992	1287.35828	0.164182576
1288.58282	0.350262612	1288.58282	0.164131799
1290	0.350322288	1290	0.164073293
1290.37423	0.350337736	1290.37423	0.164057865
1292.07485	0.350407165	1292.07485	0.16398814
1292.35828	0.350418532	1292.35828	0.163976553
1293.58282	0.350467017	1293.58282	0.163926629
1295	0.350521879	1295	0.16386911
1295.37423	0.350536062	1295.37423	0.163853946
1297.07485	0.350599804	1297.07485	0.16378541
1297.35828	0.350610231	1297.35828	0.163774023
1298.58282	0.350654681	1298.58282	0.163724958
1300	0.350704921	1300	0.163668437
1300.37423	0.350717896	1300.37423	0.163653537
1302.07485	0.350776184	1302.07485	0.163586198
1302.35828	0.350785711	1302.35828	0.163575011
1303.58282	0.350826294	1303.58282	0.16352681
1305	0.350872116	1305	0.163471292
1305.37423	0.35088393	1305.37423	0.163456655
1307.07485	0.35093701	1307.07485	0.16339052
1307.35828	0.350945675	1307.35828	0.163379534
1308.58282	0.350982567	1308.58282	0.163332199
1310	0.351024168	1310	0.163277683
1310.37423	0.351034878	1310.37423	0.163263312
1312.07485	0.351082991	1312.07485	0.163198381
1312.35828	0.351090837	1312.35828	0.163187596
1313.58282	0.351124214	1313.58282	0.163141129
1315	0.351161803	1315	0.163087614
1315.37423	0.351171463	1315.37423	0.163073509
1317.07485	0.351214856	1317.07485	0.163009783
1317.35828	0.351221922	1317.35828	0.162999196
1318.58282	0.351251965	1318.58282	0.162953594
1320	0.351285748	1320	0.162901083
1320.37423	0.351294412	1320.37423	0.16288724
1322.07485	0.351333334	1322.07485	0.162824712
1322.35828	0.351339664	1322.35828	0.162814325
1323.58282	0.35136655	1323.58282	0.162769584
1325	0.351396736	1325	0.162718067
1325.37423	0.35140446	1325.37423	0.162704485

Final State: j'=1		Final State: j'=0	
E(cm⁻¹)	σ (Å²)	E(cm⁻¹)	σ (Å²)
1327.07485	0.351439166	1327.07485	0.162643148
1327.35828	0.351444802	1327.35828	0.162632961
1328.58282	0.351468716	1328.58282	0.162589077
1330	0.351495516	1330	0.162538547
1330.37423	0.351502355	1330.37423	0.162525227
1332.07485	0.351533097	1332.07485	0.162465072
1332.35828	0.351538081	1332.35828	0.16245508
1333.58282	0.351559202	1333.58282	0.162412043
1335	0.351582829	1335	0.162362493
1335.37423	0.35158884	1335.37423	0.162349432
1337.07485	0.351615873	1337.07485	0.162290444
1337.35828	0.351620247	1337.35828	0.162280647
1338.58282	0.351638759	1338.58282	0.16223845
1340	0.351659427	1340	0.162189866
1340.37423	0.351664664	1340.37423	0.162177057
1342.07485	0.351688243	1342.07485	0.162119226
1342.35828	0.35169205	1342.35828	0.162109622
1343.58282	0.35170814	1343.58282	0.16206825
1345	0.351726056	1345	0.162020621
1345.37423	0.351730578	1345.37423	0.162008066
1347.07485	0.351750957	1347.07485	0.161951371
1347.35828	0.351754238	1347.35828	0.161941956
1348.58282	0.351768088	1348.58282	0.161901398
1350	0.351783468	1350	0.161854708
1350.37423	0.35178733	1350.37423	0.161842399
1352.07485	0.351804765	1352.07485	0.161786824
1352.35828	0.351807562	1352.35828	0.161777595
1353.58282	0.351819356	1353.58282	0.16173784
1355	0.351832411	1355	0.16169207
1355.37423	0.351835669	1355.37423	0.161680004
1357.07485	0.351850411	1357.07485	0.161625527
1357.35828	0.351852768	1357.35828	0.16161648
1358.58282	0.351862687	1358.58282	0.161577509
1360	0.351873629	1360	0.161532645
1360.37423	0.351876335	1360.37423	0.161520817
1362.07485	0.35188864	1362.07485	0.161467416
1362.35828	0.3518906	1362.35828	0.161458546
1363.58282	0.351898826	1363.58282	0.161420344
1364.14969	0.351902502	1364.14969	0.161402723
1365	0.351907862	1365	0.161376364
1365.37423	0.351910075	1365.37423	0.161364768
1367.07485	0.351920191	1367.07485	0.161312418
1367.35828	0.351921795	1367.35828	0.161303724
1368.58282	0.351928507	1368.58282	0.161266272
1369.14969	0.351931497	1369.14969	0.161248997

Final State: j'=1		Final State: j'=0	
E(cm⁻¹)	σ (Å²)	E(cm⁻¹)	σ (Å²)
1370	0.351935847	1370	0.161223155
1370.37423	0.35193762	1370.37423	0.161211786
1372.07485	0.351945797	1372.07485	0.161160462
1372.35828	0.351947086	1372.35828	0.161151938
1373.58282	0.351952462	1373.58282	0.161115219
1374.14969	0.351954849	1374.14969	0.16109828
1375	0.351958311	1375	0.161072942
1375.37423	0.351959701	1375.37423	0.161061794
1377.07485	0.351966184	1377.07485	0.161011469
1377.35828	0.351967197	1377.35828	0.161003111
1378.58282	0.351971416	1378.58282	0.160967104
1379.14969	0.351973281	1379.14969	0.160950493
1380	0.351975977	1380	0.160925645
1380.37423	0.351977034	1380.37423	0.160914712
1382.07485	0.351982065	1382.07485	0.160865356
1382.35828	0.351982846	1382.35828	0.160857158
1383.58282	0.351986081	1383.58282	0.160821843
1384.14969	0.351987501	1384.14969	0.160805551
1385	0.351989554	1385	0.160781179
1385.37423	0.351990335	1385.37423	0.160770453
1387.07485	0.351994155	1387.07485	0.16072204
1387.35828	0.35199474	1387.35828	0.160713999
1388.58282	0.351997152	1388.58282	0.160679348
1389.14969	0.351998222	1389.14969	0.160663369
1390	0.351999746	1390	0.160639457
1390.37423	0.3520003	1390.37423	0.160628933
1392.07485	0.352003145	1392.07485	0.160581432
1392.35828	0.352003572	1392.35828	0.160573539
1393.58282	0.352005352	1393.58282	0.160539542
1394.14969	0.352006128	1394.14969	0.160523856
1395	0.352007243	1395	0.16050039
1395.37423	0.35200762	1395.37423	0.160490061
1397.07485	0.352009724	1397.07485	0.160443438
1397.35828	0.352010038	1397.35828	0.160435693
1398.58282	0.352011337	1398.58282	0.160402321
1399.14969	0.352011906	1399.14969	0.160386923
1400	0.352012724	1400	0.160363886
1400.37423	0.352012979	1400.37423	0.160353744
1402.07485	0.352014566	1402.07485	0.16030797
1402.35828	0.352014802	1402.35828	0.160300365
1403.58282	0.352015788	1403.58282	0.160267595
1404.14969	0.352016224	1404.14969	0.160252474
1405	0.352016861	1405	0.16022985
1405.37423	0.352017041	1405.37423	0.16021989
1407.07485	0.352018339	1407.07485	0.160174931

Final State: j’=1		Final State: j’=0	
E(cm⁻¹)	σ (Å²)	E(cm⁻¹)	σ (Å²)
1407.35828	0.352018533	1407.35828	0.16016746
1408.58282	0.352019363	1408.58282	0.160135269
1409.14969	0.352019741	1409.14969	0.160120415
1410	0.352020308	1410	0.160098187
1410.37423	0.352020557	1410.37423	0.160088425
1412.07485	0.35202169	1412.07485	0.160044225
1412.35828	0.35202188	1412.35828	0.160036882
1413.58282	0.352022716	1413.58282	0.160005246
1414.14969	0.352023112	1414.14969	0.159990647
1415	0.352023714	1415	0.1599688
1415.37423	0.352023986	1415.37423	0.159959205
1417.07485	0.352025266	1417.07485	0.159915754
1417.35828	0.352025492	1417.35828	0.159908536
1418.58282	0.352026488	1418.58282	0.159877432
1419.14969	0.352026967	1419.14969	0.159863075
1420	0.352027721	1420	0.159841592
1420.37423	0.352028062	1420.37423	0.159832157
1422.07485	0.35202971	1422.07485	0.159789421
1422.35828	0.352030001	1422.35828	0.159782322
1423.58282	0.352031315	1423.58282	0.159751724
1424.14969	0.352031959	1424.14969	0.1597376
1425	0.352032967	1425	0.159716463
1425.37423	0.352033427	1425.37423	0.159707178
1427.07485	0.352035667	1427.07485	0.159665124
1427.35828	0.352036063	1427.35828	0.159658141
1428.58282	0.352037862	1428.58282	0.159628021
1429.14969	0.352038743	1429.14969	0.159614117
1430	0.352040127	1430	0.159593309
1430.37423	0.35204076	1430.37423	0.159584168
1432.07485	0.352043837	1432.07485	0.159542759
1432.35828	0.352044382	1432.35828	0.159535877
1433.58282	0.352046862	1433.58282	0.159506215
1434.14969	0.35204808	1434.14969	0.15949252
1435	0.352049994	1435	0.159472018
1435.37423	0.35205087	1435.37423	0.159463011
1437.07485	0.352055169	1437.07485	0.159422196
1437.35828	0.352055937	1437.35828	0.159415413
1438.58282	0.35205948	1438.58282	0.159386159
1439.14969	0.352061259	1439.14969	0.159372644
1440	0.352064136	1440	0.159352403
1440.37423	0.352065503	1440.37423	0.159343504
1442.07485	0.352073186	1442.07485	0.159303065
1442.35828	0.352074954	1442.35828	0.1592963
1443.58282	0.352090879	1443.58282	0.15926722
1444.14969	0.35208687	1444.14969	0.159255268

Final State: j’=1		Final State: j’=0	
E(cm⁻¹)	σ (Å²)	E(cm⁻¹)	σ (Å²)
1445	0.352077815	1445	0.15923514
1445.37423	0.35208785	1445.37423	0.15922569
1447.07485	0.352086586	1447.07485	0.159186635
1447.35828	0.352092749	1447.35828	0.159179632
1448.58282	0.352078467	1448.58282	0.159152798
1449.14969	0.352080573	1449.14969	0.159140411
1450	0.352086909	1450	0.159120121
1450.37423	0.35209418	1450.37423	0.159111044
1452.07485	0.352095033	1452.07485	0.159071885
1452.35828	0.352105208	1452.35828	0.159064772
1453.58282	0.352104185	1453.58282	0.159037627
1454.14969	0.35211084	1454.14969	0.159024547
1455	0.352122429	1455	0.15900584
1455.37423	0.352098885	1455.37423	0.159003545
1457.07485	0.352111472	1457.07485	0.158958779
1457.35828	0.352120036	1457.35828	0.15895176
1458.58282	0.35213371	1458.58282	0.158923925
1459.14969	0.352139256	1459.14969	0.15891136
1460	0.352139735	1460	0.158897237
1460.37423	0.352135825	1460.37423	0.158881506
1462.07485	0.352148974	1462.07485	0.158845168
1462.35828	0.352153086	1462.35828	0.158838743
1463.58282	0.352165311	1463.58282	0.158811498
1464.14969	0.352170472	1464.14969	0.158799059
1465	0.352178586	1465	0.158781158
1465.37423	0.352184312	1465.37423	0.158775239
1467.07485	0.352165888	1467.07485	0.158734896
1467.35828	0.352187301	1467.35828	0.158727029
1468.58282	0.352203291	1468.58282	0.158700002
1469.14969	0.352208938	1469.14969	0.158687612
1470	0.352217258	1470	0.158669148
1470.37423	0.352220926	1470.37423	0.158661121
1472.07485	0.352240108	1472.07485	0.158619542
1472.35828	0.352237223	1472.35828	0.158617429
1473.58282	0.352248277	1473.58282	0.158589218
1474.14969	0.352256864	1474.14969	0.158576904
1475	0.35226798	1475	0.15855841
1475.37423	0.352273036	1475.37423	0.15855024
1477.07485	0.352317232	1477.07485	0.158511541
1477.35828	0.352303223	1477.35828	0.158507339
1478.58282	0.352267263	1478.58282	0.158481644
1479.14969	0.352286556	1479.14969	0.158474277
1480	0.352310456	1480	0.158450105
1480.37423	0.352334967	1480.37423	0.15844096
1482.07485	0.352334462	1482.07485	0.158406993

Final State: j'=1		Final State: j'=0	
E(cm⁻¹)	σ (Å²)	E(cm⁻¹)	σ (Å²)
1482.35828	0.352343922	1482.35828	0.158400493
1483.58282	0.352304852	1483.58282	0.158380037
1484.14969	0.3523461	1484.14969	0.158364238
1485	0.352369173	1485	0.158345726
1485.37423	0.352379255	1485.37423	0.158338215
1487.07485	0.352382572	1487.07485	0.158300388
1487.35828	0.352386337	1487.35828	0.158293566
1488.58282	0.352418682	1488.58282	0.158267884
1489.14969	0.3524381	1489.14969	0.158256586
1490	0.352396198	1490	0.158239691
1490.37423	0.352424378	1490.37423	0.158230281
1492.07485	0.352465206	1492.07485	0.158194465
1492.35828	0.352471163	1492.35828	0.158188704
1493.58282	0.352429605	1493.58282	0.158164805
1494.14969	0.352496496	1494.14969	0.158145371
1495	0.352486631	1495	0.15812955
1495.37423	0.352530526	1495.37423	0.158120602
1497.07485	0.352520388	1497.07485	0.158093083
1497.35828	0.352548123	1497.35828	0.158085387
1498.58282	0.35254797	1498.58282	0.158057365
1499.14969	0.352581501	1499.14969	0.158044113
1500	0.352561994	1500	0.15803044
1500.37423	0.352584565	1500.37423	0.158021285
1502.07485	0.352611172	1502.07485	0.157990463
1502.35828	0.352648112	1502.35828	0.157979817
1503.58282	0.352497548	1503.58282	0.157977863
1504.14969	0.352654994	1504.14969	0.157940389
1505.37423	0.352691873	1505.37423	0.157916737
1507.07485	0.352712521	1507.07485	0.157886589
1507.35828	0.352727722	1507.35828	0.157864546
1508.58282	0.352739424	1508.58282	0.1578506
1509.14969	0.352750801	1509.14969	0.157839447
1510.37423	0.352772684	1510.37423	0.157815398
1512.07485	0.352807461	1512.07485	0.157776805
1512.35828	0.352814733	1512.35828	0.157772131
1513.58282	0.352840354	1513.58282	0.157748269
1514.14969	0.352852164	1514.14969	0.157736859
1515.37423	0.352877848	1515.37423	0.157712109
1517.07485	0.35291458	1517.07485	0.15767712
1517.35828	0.352921028	1517.35828	0.157671351
1518.58282	0.352948588	1518.58282	0.157646592
1519.14969	0.352960429	1519.14969	0.157635166
1520.37423	0.352991399	1520.37423	0.157609801
1522.07485	0.353031385	1522.07485	0.157575502
1522.35828	0.353038204	1522.35828	0.157569764

Final State: j'=1		Final State: j'=0	
E(cm⁻¹)	σ (Å²)	E(cm⁻¹)	σ (Å²)
1523.58282	0.353068052	1523.58282	0.15754499
1524.14969	0.353082056	1524.14969	0.157533532
1525.37423	0.353112685	1525.37423	0.157508801
1527.07485	0.353155988	1527.07485	0.157474381
1527.35828	0.353163334	1527.35828	0.157468581
1528.58282	0.353195758	1528.58282	0.15744394
1529.14969	0.35321084	1529.14969	0.15743259
1530.37423	0.353243747	1530.37423	0.157408025
1532.07485	0.353290333	1532.07485	0.157373913
1533.58282	0.353332518	1533.58282	0.157343696
1534.14969	0.353348592	1534.14969	0.157332343
1535.37423	0.353383717	1535.37423	0.157307833
1537.07485	0.353433446	1537.07485	0.157273848
1538.58282	0.353478433	1538.58282	0.157243774
1539.14969	0.353495561	1539.14969	0.157232479
1540.37423	0.353532959	1540.37423	0.1572081
1542.07485	0.353585814	1542.07485	0.157174284
1543.58282	0.353633579	1543.58282	0.157144338
1544.14969	0.353651751	1544.14969	0.157133089
1545.37423	0.35369142	1545.37423	0.15710881
1547.07485	0.353747438	1547.07485	0.157075131
1548.58282	0.353798015	1548.58282	0.15704531
1549.14969	0.353817244	1549.14969	0.15703411
1550.37423	0.353859202	1550.37423	0.157009933
1552.07485	0.353918401	1552.07485	0.156976394
1553.58282	0.3539718	1553.58282	0.156946695
1554.14969	0.353992093	1554.14969	0.156935539
1555.37423	0.354036345	1555.37423	0.156911458
1557.07485	0.354098729	1557.07485	0.156878053
1558.58282	0.354154959	1558.58282	0.156848469
1559.14969	0.354176317	1559.14969	0.156837358
1560.37423	0.354222867	1560.37423	0.156813371
1562.07485	0.354288446	1562.07485	0.156780098
1563.58282	0.354347505	1563.58282	0.15675063
1564.14969	0.354369926	1564.14969	0.156739561
1565.37423	0.354418772	1565.37423	0.156715669
1567.07485	0.354487541	1567.07485	0.156682523
1568.58282	0.354549425	1568.58282	0.15665317
1569.14969	0.35457291	1569.14969	0.156642145
1570.37423	0.354624047	1570.37423	0.156618342
1572.07485	0.354695995	1572.07485	0.156585327
1573.58282	0.354760693	1573.58282	0.156556088
1574.14969	0.354785235	1574.14969	0.156545106
1575.37423	0.354838652	1575.37423	0.156521399
1577.07485	0.354913763	1577.07485	0.156488514

Final State: j’=1		Final State: j’=0	
E(cm⁻¹)	σ (Å²)	E(cm⁻¹)	σ (Å²)
1578.58282	0.35498126	1578.58282	0.15645939
1579.14969	0.355007791	1579.14969	0.156448701
1580.37423	0.355062534	1580.37423	0.156424839
1582.07485	0.355140784	1582.07485	0.156392085
1583.58282	0.355211057	1583.58282	0.156363083
1584.14969	0.355237689	1584.14969	0.156352188
1585.37423	0.355295617	1585.37423	0.156328673
1588.58282	0.355449997	1588.58282	0.156267176
1589.14969	0.35547766	1589.14969	0.156256329
1590.37423	0.355537811	1590.37423	0.156232915
1593.58282	0.355697979	1593.58282	0.156171686
1594.14969	0.355726659	1594.14969	0.156160888
1595.37423	0.355789002	1595.37423	0.156137581
1598.58282	0.355954879	1598.58282	0.156076633
1599.14969	0.355984562	1599.14969	0.156065885
1600.37423	0.356049067	1600.37423	0.156042688
1603.58282	0.356220561	1603.58282	0.155982037
1604.14969	0.35625123	1604.14969	0.155971343
1605.37423	0.356317858	1605.37423	0.155948262
1608.58282	0.35649487	1608.58282	0.155887925
1609.14969	0.356526506	1609.14969	0.155877286
1610.37423	0.356595217	1610.37423	0.155854328
1613.58282	0.356777635	1613.58282	0.155794321
1614.14969	0.356810219	1614.14969	0.155783743
1615.37423	0.356880967	1615.37423	0.155760916
1618.58282	0.357068671	1618.58282	0.15570126
1619.14969	0.357102183	1619.14969	0.155690745
1620.37423	0.357174919	1620.37423	0.155668057
1623.58282	0.357367777	1623.58282	0.155608774
1624.14969	0.35740219	1624.14969	0.155598327
1625.37423	0.357476865	1625.37423	0.155575787
1628.58282	0.357674741	1628.58282	0.155516902
1629.14969	0.357710028	1629.14969	0.155506526
1630.37423	0.357786589	1630.37423	0.155484142
1633.58282	0.357989329	1633.58282	0.15542568
1634.14969	0.358025467	1634.14969	0.155415379
1635.37423	0.358103852	1635.37423	0.155393161
1638.58282	0.358311305	1638.58282	0.155335148
1639.14969	0.358348265	1639.14969	0.155324929
1640.37423	0.358428412	1640.37423	0.155302888
1643.58282	0.358640414	1643.58282	0.155245349
1644.14969	0.358678167	1644.14969	0.155235216
1645.37423	0.358760011	1645.37423	0.155213363
1648.58282	0.358976389	1648.58282	0.15515633
1649.14969	0.359014902	1649.14969	0.155146288

Final State: j’=1		Final State: j’=0	
E(cm⁻¹)	σ (Å²)	E(cm⁻¹)	σ (Å²)
1650.37423	0.359098381	1650.37423	0.155124633
1653.58282	0.359318953	1653.58282	0.155068133
1654.14969	0.359358194	1654.14969	0.155058187
1655.37423	0.359443238	1655.37423	0.155036744
1658.58282	0.359667824	1658.58282	0.154980808
1659.14969	0.359707762	1659.14969	0.154970965
1660.37423	0.359794295	1660.37423	0.154949743
1663.58282	0.360022699	1663.58282	0.154894403
1664.14969	0.3600633	1664.14969	0.154884667
1665.37423	0.360151251	1665.37423	0.154863679
1668.58282	0.360383362	1668.58282	0.154808957
1670.37423	0.360513796	1670.37423	0.154778602
1673.58282	0.360749246	1673.58282	0.15472455
1675.37423	0.360881615	1675.37423	0.154694562
1678.58282	0.361120279	1678.58282	0.154641203
1680.37423	0.361254386	1680.37423	0.154611611
1683.58282	0.361496054	1683.58282	0.154558976
1685.37423	0.361631777	1685.37423	0.154529798
1688.58282	0.361876232	1688.58282	0.154477922
1690.37423	0.362013452	1690.37423	0.154449176
1693.58282	0.362260479	1693.58282	0.15439809
1695.37423	0.362399072	1695.37423	0.154369795
1698.58282	0.362648448	1698.58282	0.154319532
1700.37423	0.362788288	1700.37423	0.154291705
1703.58282	0.363039791	1703.58282	0.154242297
1705.37423	0.363180757	1705.37423	0.15421496
1708.58282	0.363434158	1708.58282	0.154166437
1710.37423	0.363576121	1710.37423	0.154139602
1713.58282	0.363831197	1713.58282	0.154092001
1715.37423	0.363974029	1715.37423	0.154065688
1718.58282	0.364230549	1718.58282	0.154019036
1720.37423	0.364374124	1720.37423	0.153993261
1723.58282	0.364631853	1723.58282	0.15394759
1725.37423	0.364776038	1725.37423	0.153922371
1728.58282	0.365034746	1728.58282	0.153877711
1730.37423	0.365179408	1730.37423	0.153853064
1733.58282	0.365438838	1733.58282	0.153809444
1735.37423	0.365583827	1735.37423	0.153785386
1738.58282	0.365843672	1738.58282	0.153742838
1740.37423	0.365988747	1740.37423	0.153719391
1743.58282	0.366247705	1743.58282	0.153678009
1745.37423	0.366398543	1745.37423	0.153654916
1748.58282	0.366646861	1748.58282	0.153615931
1750.37423	0.366800879	1750.37423	0.153592592
1753.58282	0.367068378	1753.58282	0.15355258

Final State: j'=1		Final State: j'=0	
E(cm⁻¹)	σ (Å²)	E(cm⁻¹)	σ (Å²)
1755.37423	0.367207201	1755.37423	0.153531601
1758.58282	0.367469407	1758.58282	0.153493408
1760.37423	0.367612301	1760.37423	0.15347253
1763.58282	0.367873932	1763.58282	0.153435623
1765.37423	0.368017516	1765.37423	0.153415357
1768.58282	0.36827769	1768.58282	0.153379721
1770.37423	0.3684208	1770.37423	0.153360157
1773.58282	0.368678125	1773.58282	0.153325992
1775.37423	0.368821433	1775.37423	0.153306929
1780.37423	0.369210334	1780.37423	0.153257194
1785.37423	0.369622485	1785.37423	0.15320581
1790.37423	0.370008274	1790.37423	0.153158533
1795.37423	0.370404779	1795.37423	0.153113986
1800.37423	0.370788954	1800.37423	0.153069908
1805.37423	0.371191972	1805.37423	0.153027985
1810.37423	0.371569082	1810.37423	0.152988452
1815.37423	0.371949116	1815.37423	0.152950993
1820.37423	0.372323213	1820.37423	0.152916694
1825.37423	0.372697611	1825.37423	0.152882199
1830.37423	0.373065561	1830.37423	0.152850841
1835.37423	0.373429268	1835.37423	0.152821487
1840.37423	0.373788429	1840.37423	0.152794135
1845.37423	0.374142975	1845.37423	0.152768772
1850.37423	0.374492763	1850.37423	0.15274538
1855.37423	0.374837687	1855.37423	0.152723937
1860.37423	0.375177658	1860.37423	0.152704423
1865.37423	0.375512595	1865.37423	0.152686807
1870.37423	0.375842437	1870.37423	0.152671063
1875.37423	0.376167138	1875.37423	0.152657154
1880.37423	0.376486666	1880.37423	0.152645048
1885.37423	0.376801006	1885.37423	0.152634705
1890.37423	0.377110156	1890.37423	0.152626083
1895.37423	0.377414129	1895.37423	0.15261914
1900.37423	0.377712953	1900.37423	0.152613829
1905.37423	0.378006672	1905.37423	0.152610101
1910.37423	0.378295341	1910.37423	0.152607906

Initial rotational state: j=4

Final State: j'=5		Final State: j'=3		Final State: j'=2	
E(cm⁻¹)	σ (Å²)	E(cm⁻¹)	σ (Å²)	E(cm⁻¹)	σ (Å²)
410.374226	0.000184091	273.582818	21518.1053	273.582818	5455.9775
410.374227	0.000260556	273.582819	15230.033	273.582819	3861.76108
410.374228	0.000319394	273.58282	12447.886	273.58282	3156.42988
410.374229	0.000369138	273.582821	10791.4655	273.582821	2736.50928
410.37423	0.000413088	273.582822	9662.4637	273.582822	2450.3059
410.374231	0.000452938	273.582823	8830.09295	273.582823	2239.30684
410.374232	0.000489689	273.582824	8183.96642	273.582824	2075.52534
410.374233	0.000523996	273.582825	7663.77596	273.582825	1943.6714
410.374234	0.00055631	273.582826	7233.42072	273.582826	1834.59237
410.374235	0.000586963	273.582827	6869.79751	273.582827	1742.43095
410.374236	0.000616203	273.582828	6557.34368	273.582828	1663.24161
410.374237	0.000644221	273.582829	6285.14297	273.582829	1594.25681
410.374238	0.000671173	273.58283	6045.28144	273.58283	1533.4702
410.374239	0.000697181	273.582831	5831.86331	273.582831	1479.38707
410.37424	0.000722348	273.582832	5640.3947	273.582832	1430.86814
410.374241	0.00074676	273.582833	5467.3817	273.582833	1387.02766
410.374242	0.000770488	273.582834	5310.06182	273.582834	1347.16528
410.374243	0.000793594	273.582835	5166.21835	273.582835	1310.71905
410.374244	0.000816131	273.582836	5034.04882	273.582836	1277.23202
410.374245	0.000838146	273.582837	4912.07108	273.582837	1246.32845
410.374246	0.000859678	273.582838	4799.05297	273.582838	1217.69598
410.374247	0.000880762	273.582839	4693.96138	273.582839	1191.07271
410.374248	0.000901432	273.58284	4595.92211	273.58284	1166.23703
410.374249	0.000921715	273.582841	4504.18997	273.582841	1143.00004
410.37425	0.000941635	273.582842	4418.12567	273.582842	1121.19966
410.374251	0.000961216	273.582843	4337.17649	273.582843	1100.6958
410.374252	0.000980478	273.582844	4260.86285	273.582844	1081.36687
410.374253	0.00099944	273.582845	4188.76516	273.582845	1063.10651
410.374254	0.001018119	273.582846	4120.51536	273.582846	1045.82143
410.374255	0.00103653	273.582847	4055.78856	273.582847	1029.42927
410.374256	0.001054687	273.582848	3994.2971	273.582848	1013.85711
410.374257	0.001072604	273.582849	3935.78493	273.582849	999.040038
410.374258	0.001090292	273.58285	3880.02362	273.58285	984.920159
410.374259	0.001107763	273.582851	3826.80835	273.582851	971.44555
410.37426	0.001125026	273.582852	3775.95502	273.582852	958.569551
410.374261	0.001142092	273.582853	3727.29754	273.582853	946.250054
410.374262	0.001158969	273.582854	3680.68592	273.582854	934.449046
410.374263	0.001175665	273.582855	3635.98368	273.582855	923.131929
410.374264	0.001192188	273.582856	3593.06704	273.582856	912.267332
410.374265	0.001208546	273.582857	3551.82302	273.582857	901.826615
410.374266	0.001224745	273.582858	3512.14837	273.582858	891.783605
410.374267	0.001240791	273.582859	3473.94867	273.582859	882.114374
410.374268	0.001256691	273.58286	3437.13703	273.58286	872.796898
410.374269	0.00127245	273.582861	3401.6337	273.582861	863.810961

Final State: j'=5		Final State: j'=3		Final State: j'=2	
E(cm⁻¹)	σ (Å²)	E(cm⁻¹)	σ (Å²)	E(cm⁻¹)	σ (Å²)
410.37427	0.001288073	273.582862	3367.36452	273.582862	855.137768
410.374271	0.001303565	273.582863	3334.26274	273.582863	846.7604
410.374272	0.001318932	273.582864	3302.26482	273.582864	838.66275
410.374273	0.001334177	273.582865	3271.31264	273.582865	830.830091
410.374274	0.001349305	273.582866	3241.35233	273.582866	823.248767
410.374275	0.001364319	273.582867	3212.33347	273.582867	815.905999
410.374276	0.001379225	273.582868	3184.20951	273.582868	808.789991
410.374277	0.001394024	273.582869	3156.93676	273.582869	801.889666
410.374278	0.001408721	273.58287	3130.47466	273.58287	795.194748
410.374279	0.001423319	273.582871	3104.78518	273.582871	788.695595
410.37428	0.001437821	273.582872	3079.83266	273.582872	782.383174
410.374281	0.00145223	273.582873	3055.58401	273.582873	776.249093
410.374282	0.001466549	273.582874	3032.00778	273.582874	770.285386
410.374283	0.001480781	273.582875	3009.07475	273.582875	764.484644
410.374284	0.001494928	273.582876	2986.75723	273.582876	758.839853
410.374285	0.001508992	273.582877	2965.02934	273.582877	753.34445
410.374286	0.001522977	273.582878	2943.86656	273.582878	747.992225
410.374287	0.001536883	273.582879	2923.24564	273.582879	742.777283
410.374288	0.001550715	273.58288	2903.14512	273.58288	737.694186
410.374289	0.001564473	273.582881	2883.54416	273.582881	732.737652
410.37429	0.00157816	273.582882	2864.42337	273.582882	727.902768
410.374291	0.001591777	273.582883	2845.76438	273.582883	723.184879
410.374292	0.001605327	273.582884	2827.54971	273.582884	718.579556
410.374293	0.00161881	273.582885	2809.76274	273.582885	714.082583
410.374294	0.00163223	273.582886	2792.38778	273.582886	709.689991
410.374295	0.001645588	273.582887	2775.41016	273.582887	705.398052
410.374296	0.001658885	273.582888	2758.81526	273.582888	701.20307
410.374297	0.001672122	273.582889	2742.5899	273.582889	697.101703
410.374298	0.001685302	273.58289	2726.72123	273.58289	693.090694
410.374299	0.001698426	273.582891	2711.19699	273.582891	689.166937
410.3743	0.001711495	273.582892	2696.00559	273.582892	685.327494
410.374301	0.00172451	273.582893	2681.13574	273.582893	681.569501
410.374302	0.001737472	273.582894	2666.57713	273.582894	677.890354
410.374303	0.001750384	273.582895	2652.31948	273.582895	674.287438
410.374304	0.001763246	273.582896	2638.35318	273.582896	670.758326
410.374305	0.00177606	273.582897	2624.66899	273.582897	667.300672
410.374306	0.001788825	273.582898	2611.25806	273.582898	663.912234
410.374307	0.001801545	273.582899	2598.11195	273.582899	660.590874
410.374308	0.001814219	273.5829	2585.22249	273.5829	657.334524
410.374309	0.001826849	273.582901	2572.58214	273.582901	654.141268
410.37431	0.001839435	273.582902	2560.1832	273.582902	651.009159
410.374311	0.001851979	273.582903	2548.0187	273.582903	647.936433
410.374312	0.001864482	273.582904	2536.08175	273.582904	644.92134
410.374313	0.001876945	273.582905	2524.36587	273.582905	641.962237
410.374314	0.001889367	273.582906	2512.86471	273.582906	639.05752

Final State: j'=5		Final State: j'=3		Final State: j'=2	
E(cm⁻¹)	σ (Å²)	E(cm⁻¹)	σ (Å²)	E(cm⁻¹)	σ (Å²)
410.374315	0.001901751	273.582907	2501.57216	273.582907	636.205638
410.374316	0.001914097	273.582908	2490.48252	273.582908	633.405147
410.374317	0.001926406	273.582909	2479.59003	273.582909	630.654587
410.374318	0.001938678	273.58291	2468.88943	273.58291	627.952628
410.374319	0.001950915	273.582911	2458.37555	273.582911	625.297955
410.37432	0.001963118	273.582912	2448.04331	273.582912	622.689284
410.374321	0.001975286	273.582913	2437.88793	273.582913	620.125407
410.374322	0.00198742	273.582914	2427.9048	273.582914	617.60515
410.374323	0.001999522	273.582915	2418.08949	273.582915	615.127393
410.374324	0.002011593	273.582916	2408.43763	273.582916	612.691032
410.374325	0.002023631	273.582917	2398.94506	273.582917	610.295008
410.374825	0.007551375	273.583417	1568.20114	273.583417	403.196632
410.375225	0.01296064	273.583817	1654.43921	273.583817	427.247056
410.375325	0.014519517	273.583917	1692.87768	273.583917	437.51444
410.375825	0.023740512	273.584417	1937.09648	273.584417	501.936809
410.376225	0.0330022	273.584817	2173.25332	273.584817	563.655746
410.376325	0.035598388	273.584917	2236.09096	273.584917	580.016535
410.376825	0.05032474	273.585417	2564.59943	273.585417	665.165762
410.377225	0.064190379	273.585817	2834.3651	273.585817	734.569291
410.377325	0.067933761	273.585917	2901.26462	273.585917	751.69535
410.377825	0.088130705	273.586417	3222.82925	273.586417	833.398583
410.378225	0.105716482	273.586817	3452.75008	273.586817	890.936861
410.378325	0.110249271	273.586917	3504.86192	273.586917	903.823109
410.378825	0.133264746	273.587417	3725.28987	273.587417	957.26856
410.379225	0.151474425	273.587817	3846.54282	273.587817	985.168301
410.379325	0.155909637	273.587917	3868.59182	273.587917	989.945434
410.379825	0.176875462	273.588417	3928.64751	273.588417	1000.72878
410.380225	0.191673827	273.588817	3918.8559	273.588817	994.543562
410.380325	0.195040022	273.588917	3908.99622	273.588917	991.139011
410.380825	0.20964511	273.589417	3820.74549	273.589417	964.642524
410.381225	0.218539659	273.589817	3711.07051	273.589817	934.223958
410.381325	0.220375025	273.589917	3679.33402	273.589917	925.640541
410.381825	0.2273292	273.590417	3501.38876	273.590417	878.554004
410.382225	0.23044211	273.590817	3343.15481	273.590817	837.680682
410.382325	0.230916701	273.590917	3302.29316	273.590917	827.231519
410.382825	0.231719257	273.591417	3094.77872	273.591417	774.663533
410.383225	0.230779133	273.591817	2929.20919	273.591817	733.167889
410.383325	0.230364881	273.591917	2888.35284	273.591917	722.97254
410.383825	0.227440393	273.592417	2689.43795	273.592417	673.527729
410.384225	0.224308082	273.592817	2538.39115	273.592817	636.129514
410.384325	0.223443195	273.592917	2501.91116	273.592917	627.109841
410.384825	0.218767639	273.593417	2327.71366	273.593417	584.080847
410.385225	0.214741771	273.593817	2198.44568	273.593817	552.16559
410.385325	0.213711497	273.593917	2167.51398	273.593917	544.532303
410.385825	0.208490616	273.594417	2021.11019	273.594417	508.372612

Final State: j'=5		Final State: j'=3		Final State: j'=2	
E(cm⁻¹)	σ (Å²)	E(cm⁻¹)	σ (Å²)	E(cm⁻¹)	σ (Å²)
410.386225	0.204298748	273.594817	1913.46926	273.594817	481.764754
410.386325	0.203256377	273.594917	1887.81385	273.594917	475.416938
410.386825	0.198110994	273.595417	1766.68347	273.595417	445.431821
410.387225	0.194104384	273.595817	1677.86206	273.595817	423.410603
410.387325	0.193121168	273.595917	1656.69421	273.595917	418.161743
410.387825	0.188327922	273.596417	1556.79717	273.596417	393.349156
410.388225	0.184650444	273.596817	1483.46651	273.596817	375.108768
410.388325	0.183753863	273.596917	1465.97716	273.596917	370.756809
410.388825	0.179409637	273.597417	1383.30546	273.597417	350.154689
410.389225	0.176101138	273.597817	1322.46271	273.597817	334.971433
410.389325	0.175297094	273.597917	1307.92812	273.597917	331.341509
410.389825	0.171412683	273.598417	1239.07773	273.598417	314.130769
410.390225	0.168464748	273.598817	1188.23514	273.598817	301.40512
410.390325	0.167749381	273.598917	1176.06434	273.598917	298.35742
410.390825	0.164297719	273.599417	1118.29039	273.599417	283.873404
410.391225	0.1616819	273.599817	1075.46388	273.599817	273.12669
410.391325	0.161047467	273.599917	1065.20028	273.599917	270.546323
410.391825	0.157987532	273.600417	1016.31084	273.600417	258.265343
410.392225	0.155669091	273.600817	979.957804	273.600817	249.119839
410.392325	0.155106786	273.600917	971.226126	273.600917	246.918628
410.392825	0.152394607	273.601417	929.55145	273.601417	236.422385
410.393225	0.150339145	273.601817	898.436395	273.601817	228.583581
410.393325	0.149840489	273.601917	890.950471	273.601917	226.694854
410.393825	0.147434565	273.602417	855.149285	273.602417	217.659226
410.394225	0.145610222	273.602817	828.333895	273.602817	210.889987
410.395225	0.141409294	273.603817	767.628706	273.603817	195.542169
410.396225	0.137672609	273.604817	714.727717	273.604817	182.146345
410.397225	0.134345356	273.605817	668.348648	273.605817	170.386741
410.398225	0.131380717	273.606817	627.464133	273.606817	160.008133
410.399225	0.128738819	273.607817	591.249885	273.607817	150.804718
410.400225	0.126385616	273.608817	559.023635	273.608817	142.60713
410.401225	0.124292076	273.609817	530.236188	273.609817	135.274997
410.402225	0.122433407	273.610817	504.42143	273.610817	128.694255
410.403225	0.120788271	273.611817	481.189647	273.611817	122.770052
410.404225	0.119338302	273.612817	460.223396	273.612817	117.41582
410.405225	0.118067601	273.613817	441.250419	273.613817	112.566654
410.406225	0.116962308	273.614817	424.037928	273.614817	108.162027
410.407225	0.116010347	273.615817	408.384334	273.615817	104.154544
410.408225	0.115201095	273.616817	394.120401	273.616817	100.49966
410.409225	0.114525198	273.617817	381.099251	273.617817	97.1602819
410.410225	0.113974256	273.618817	369.194676	273.618817	94.1023789
410.411225	0.113540687	273.619817	358.287329	273.619817	91.2994953
410.412225	0.113217763	273.620817	348.289596	273.620817	88.7266508
410.413225	0.112999355	273.621817	339.104297	273.621817	86.3610956
410.414225	0.112879781	273.622817	330.661369	273.622817	84.1848342

Final State: j'=5		Final State: j'=3		Final State: j'=2	
E(cm⁻¹)	σ (Å²)	E(cm⁻¹)	σ (Å²)	E(cm⁻¹)	σ (Å²)
410.415225	0.112853691	273.623817	322.895583	273.623817	82.1784829
410.416225	0.112916328	273.624817	315.739225	273.624817	80.3277667
410.417225	0.113062956	273.625817	309.139946	273.625817	78.6179725
410.418225	0.113289341	273.626817	303.047616	273.626817	77.0385379
410.419225	0.113591107	273.627817	297.420049	273.627817	75.5762417
410.420225	0.113964327	273.628817	292.216339	273.628817	74.2212449
410.421225	0.11440514	273.629817	287.401195	273.629817	72.9649629
410.422225	0.114909642	273.630817	282.940768	273.630817	71.7976907
410.423225	0.115474099	273.631817	278.800761	273.631817	70.7136331
410.424225	0.116094942	273.632817	274.956258	273.632817	69.7041721
410.425225	0.116768455	273.633817	271.384548	273.633817	68.7638602
410.426225	0.117491191	273.634817	268.055562	273.634817	67.8854601
410.427225	0.118259542	273.635817	264.952293	273.635817	67.0634887
410.428225	0.119070082	273.636817	262.053337	273.636817	66.2942075
410.429225	0.119919486	273.637817	259.33898	273.637817	65.5718858
410.430225	0.120804113	273.638817	256.792837	273.638817	64.8923383
410.431225	0.121720866	273.639817	254.399896	273.639817	64.2513691
410.432225	0.122666422	273.640817	252.144523	273.640817	63.645691
410.433225	0.123637478	273.641817	250.012949	273.641817	63.0712579
410.434225	0.124630946	273.642817	247.992762	273.642817	62.5258217
410.435225	0.125643716	273.643817	246.071474	273.643817	62.0055826
410.436225	0.126672844	273.644817	244.241788	273.644817	61.5082932
410.437225	0.127715431	273.645817	242.49054	273.645817	61.0316256
410.438225	0.128768728	273.646817	240.80856	273.646817	60.5731472
410.439225	0.129829966	273.647817	239.190838	273.647817	60.1309428
410.440225	0.130896702	273.648817	237.62684	273.648817	59.7026219
410.441225	0.131966395	273.649817	236.109973	273.649817	59.2870247
410.442225	0.133036763	273.650817	234.636629	273.650817	58.8822655
410.443225	0.134105613	273.651817	233.198879	273.651817	58.4870188
410.444225	0.135170913	273.652817	231.791124	273.652817	58.1000906
410.445225	0.136230929	273.653817	230.411902	273.653817	57.720673
410.446225	0.137283627	273.654817	229.05463	273.654817	57.3475425
410.447225	0.138327626	273.655817	227.715525	273.655817	56.978942
410.448225	0.139361403	273.656817	226.393356	273.656817	56.6154982
410.449225	0.140383522	273.657817	225.086129	273.657817	56.2560289
410.450225	0.141392966	273.658817	223.788807	273.658817	55.8996055
410.451225	0.142388738	273.659817	222.500782	273.659817	55.5462951
410.452225	0.143369683	273.660817	221.221962	273.660817	55.1952471
410.453225	0.144335451	273.661817	219.946606	273.661817	54.8459958
410.454225	0.145285002	273.662817	218.677446	273.662817	54.4987397
410.455225	0.146218088	273.663817	217.414328	273.663817	54.153023
410.456225	0.147134168	273.664817	216.153743	273.664817	53.8085122
410.457225	0.148033193	273.665817	214.896761	273.665817	53.465781
410.458225	0.148914662	273.666817	213.64341	273.666817	53.1237714
410.459225	0.149778954	273.667817	212.392718	273.667817	52.7829401

Final State: j'=5		Final State: j'=3		Final State: j'=2	
E(cm⁻¹)	σ (Å²)	E(cm⁻¹)	σ (Å²)	E(cm⁻¹)	σ (Å²)
410.460225	0.15062571	273.668817	211.145611	273.668817	52.4435193
410.461225	0.151455231	273.669817	209.902524	273.669817	52.1053212
410.462225	0.152267863	273.670817	208.663407	273.670817	51.7682069
410.463225	0.153063666	273.671817	207.428332	273.671817	51.4325633
410.464225	0.153843156	273.672817	206.197414	273.672817	51.0984748
410.465225	0.154606872	273.673817	204.972317	273.673817	50.7659499
410.466225	0.155355069	273.674817	203.752424	273.674817	50.4351224
410.467225	0.156088611	273.675817	202.540387	273.675817	50.1062085
410.468225	0.156807768	273.676817	201.336081	273.676817	49.7793405
410.469225	0.157513487	273.677817	200.138109	273.677817	49.4543506
410.470225	0.158206372	273.678817	198.949986	273.678817	49.1318418
410.471225	0.15888736	273.679817	197.771339	273.679817	48.8118715
410.472225	0.159556935	273.680817	196.602155	273.680817	48.4947096
410.473225	0.160216007	273.681817	195.445917	273.681817	48.1801789
410.474225	0.16086572	273.682817	194.300771	273.682817	47.8689
410.574225	1.04582136	273.782817	581.629764	273.782817	122.09962
410.674225	0.401389098	273.882817	154.255501	273.882817	38.286377
410.774225	0.36763774	273.982817	108.207039	273.982817	26.6217676
410.874225	0.376792679	274	103.836501	274	25.5026973
410.974225	0.41960779	274.074845	90.4057469	274.074845	22.0158374
411.074225	0.498690788	274.082817	89.4298272	274.082817	21.7552316
411.174225	0.562300954	274.14969	84.3618643	274.14969	20.3074168
411.274225	0.620542766	274.182817	83.7910065	274.182817	20.040928
411.358282	0.947633104	274.282817	86.2814461	274.282817	20.1873611
411.374225	1.01297939	274.382817	85.8105768	274.382817	19.5936622
411.474225	0.722323048	274.482817	86.044591	274.482817	18.494436
411.574225	0.651258421	274.582817	117.75566	274.582817	23.4012717
411.582817	0.649628128	274.682817	73.4192017	274.682817	17.5147358
411.674225	0.644769217	274.782817	63.436807	274.782817	15.3580025
411.774225	0.651833549	274.882817	58.7272447	274.882817	14.1613252
411.874225	0.663195035	274.982817	55.4643068	274.982817	13.2977804
411.974225	0.675615921	275	54.9814624	275	13.1690476
412	0.678805045	275.082817	52.8579233	275.082817	12.6029651
412.074225	0.687795835	275.182817	50.605557	275.182817	12.0051287
412.074845	0.687869925	275.282817	48.5760956	275.282817	11.469515
412.14969	0.696554669	275.358282	47.1570556	275.358282	11.0961331
412.174225	0.699315906	275.382817	46.7141526	275.382817	10.9794794
412.274225	0.710182738	275.482817	45.0002004	275.482817	10.5261673
412.358282	0.719021688	275.582817	43.4344587	275.582817	10.1042614
412.374225	0.720696186	275.682817	42.042995	275.682817	9.71187892
412.474225	0.731538772	275.782817	40.900529	275.782817	9.34982672
412.574225	0.744147369	275.882817	40.2577617	275.882817	9.03242275
412.674225	0.76225313	275.982817	41.3882173	275.982817	8.8930851
412.774225	0.800467092	276	42.0450996	276	8.93502569
412.874225	0.931888397	276.074845	46.1585192	276.074845	9.48018804

Final State: j'=5		Final State: j'=3		Final State: j'=2	
E(cm⁻¹)	σ (Å²)	E(cm⁻¹)	σ (Å²)	E(cm⁻¹)	σ (Å²)
412.974225	1.24037755	276.082817	46.7776466	276.082817	9.58444419
413.074225	1.40617163	276.14969	54.2120254	276.14969	10.9094005
413.174225	1.08595363	276.182817	59.3818951	276.182817	11.751419
413.274225	1.10534172	276.282817	56.4637612	276.282817	11.5459042
413.358282	1.17062008	276.382817	45.4311337	276.382817	10.0105535
413.374225	1.18192297	276.482817	46.2860524	276.482817	10.3602387
413.474225	1.22591893	276.582817	48.0053428	276.582817	10.7174827
413.574225	1.22301515	276.682817	48.0586958	276.682817	10.710847
413.582817	1.22125115	276.782817	46.3835003	276.782817	10.3589533
413.674225	1.19540977	276.882817	43.9829404	276.882817	9.86033457
413.774225	1.16295187	276.982817	41.5863013	276.982817	9.35828758
413.874225	1.13421608	277.074845	39.6203727	277.074845	8.94152437
413.974225	1.11135161	277.082817	39.4628324	277.082817	8.9078852
414	1.10640446	277.182817	37.6522912	277.182817	8.51881875
414.074225	1.09415519	277.282817	36.1212526	277.282817	8.18546698
414.074845	1.09406604	277.358282	35.1227594	277.358282	7.9654296
414.14969	1.08446904	277.382817	34.8237167	277.382817	7.89909182
414.174225	1.0818637	277.482817	33.7195219	277.482817	7.65192663
414.274225	1.07372073	277.582817	32.7780653	277.582817	7.43795162
414.374225	1.06914429	277.682817	31.9760462	277.682817	7.25235936
414.474225	1.06772046	277.782817	31.2962271	277.782817	7.09163623
414.574225	1.06916974	277.882817	30.7251875	277.882817	6.95296
414.674225	1.07329365	277.982817	30.2514119	277.982817	6.83407464
414.774225	1.07992517	278	30.1789751	278	6.81547483
414.874225	1.0888833	278.074845	29.8915537	278.074845	6.74013088
414.974225	1.09992105	278.082817	29.8635081	278.082817	6.73261385
415	1.10306451	278.14969	29.6459776	278.14969	6.67332113
415.074225	1.11270232	278.182817	29.5491995	278.182817	6.64621173
415.174225	1.12678199	278.282817	29.294595	278.282817	6.57210718
415.274225	1.14162924	278.382817	29.0838931	278.382817	6.50732602
415.358282	1.15427962	278.482817	28.9004027	278.482817	6.44870462
415.374225	1.15666581	278.582817	28.7275929	278.582817	6.39312374
415.582817	1.18626353	279.14969	27.4349413	279.14969	6.03320089
416	1.22888449	279.358282	26.7866938	279.358282	5.86617178
416.074845	1.23411244	279.582817	26.0988446	279.582817	5.66056079
416.14969	1.23880631	280	26.3261864	280	5.58984164
416.374225	1.25147868	280.074845	27.190604	280.074845	5.64791369
417.074845	1.80412253	280.14969	28.8989375	280.14969	5.91991061
417.358282	1.72952979	280.582817	30.6844273	280.582817	5.99194121
417.374225	1.7441814	281.358282	24.9472202	281.358282	5.29552617
417.582817	2.21455104	281.582817	23.4227892	281.582817	5.02466972
418	1.63236725	282	21.8875857	282	4.72610414
418.074845	1.5752349	282.074845	21.683436	282.074845	4.68444873
418.14969	1.53714534	282.14969	21.49149	282.14969	4.64491542
418.374225	1.47880218	282.358282	21.0066051	282.358282	4.54359928

Final State: j’=5		Final State: j’=3		Final State: j’=2	
E(cm⁻¹)	σ (Å²)	E(cm⁻¹)	σ (Å²)	E(cm⁻¹)	σ (Å²)
418.582817	1.45716137	282.582817	20.546314	282.582817	4.44569185
419.14969	1.44505141	283.358282	19.3005029	283.358282	4.16992868
419.358282	1.44705011	283.582817	19.0408901	283.582817	4.10750497
419.374225	1.44727824	284	18.7408147	284	4.02031427
419.582817	1.45104405	284.14969	18.714235	284.14969	4.00138947
420	1.46275041	284.582817	18.978876	284.582817	3.99759889
420.074845	1.46550874	285	19.5930785	285	4.04923907
420.14969	1.46851468	285.358282	19.7738193	285.358282	4.0485387
420.374225	1.47934784	285.582817	19.5395657	285.582817	3.99997945
421.358282	1.59893065	286	18.7088802	286	3.85396679
421.374225	1.60245937	286.074845	18.5455848	286.074845	3.82573907
421.582817	1.65301559	286.14969	18.3841403	286.14969	3.79777845
422	1.74994131	286.582817	17.5333488	286.582817	3.64806989
422.074845	1.76138679	287.074845	16.7815621	287.074845	3.50913352
422.14969	1.77014422	287.358282	16.4535774	287.358282	3.44391061
422.358282	1.78092165	287.582817	16.2605827	287.582817	3.40096502
422.374225	1.78102071	288	16.3537885	288	3.37423517
423.358282	1.72774698	288.074845	16.6287986	288.074845	3.39949761
423.374225	1.72698103	288.14969	17.2551064	288.14969	3.46651576
423.582817	1.71851316	288.582817	21.6252007	288.582817	3.72416494
424	1.71020516	289.14969	15.623648	289.14969	3.20589837
424.074845	1.71000636	289.358282	15.0493334	289.358282	3.13406831
424.14969	1.71025951	289.582817	14.8011216	289.582817	3.09444469
424.374225	1.71440332	290	14.5250169	290	3.0404855
425	1.96666265	290.074845	14.4828824	290.074845	3.03166166
425.358282	2.3316683	290.14969	14.4415627	290.14969	3.02294901
425.374225	2.27099341	290.582817	14.2079259	290.582817	2.97354038
425.582817	2.46940142	291.358282	13.7820759	291.358282	2.88598723
426	1.78771779	291.582817	13.6572367	291.582817	2.86090873
426.074845	1.77461517	292	13.4275201	292	2.81513004
426.14969	1.76778687	292.074845	13.3868898	292.074845	2.80706296
426.374225	1.76283353	292.14969	13.3464953	292.14969	2.7990472
427.074845	1.77959451	292.358282	13.2353374	292.358282	2.77699824
427.358282	1.78763714	292.582817	13.1183412	292.582817	2.75378145
427.374225	1.78807578	293.358282	12.7398906	293.358282	2.67816835
427.582817	1.79346785	293.582817	12.6387872	293.582817	2.65769741
428	1.80282486	294	12.4628488	294	2.62150632
428.074845	1.80431491	294.074845	12.4331416	294.074845	2.6152858
428.14969	1.80575491	294.14969	12.4040773	294.14969	2.60915493
428.374225	1.80980873	294.582817	12.2512418	294.582817	2.57569245
428.582817	1.81328314	295	12.1415397	295	2.54794378
429.14969	1.82190833	295.358282	12.1046285	295.358282	2.53037994
429.374225	1.82520182	295.582817	12.1318547	295.582817	2.52467274
429.582817	1.82828378	296	12.3655653	296	2.53303307
430	1.83467525	296.074845	12.4369341	296.074845	2.53756352

Final State: j'=5		Final State: j'=3		Final State: j'=2	
E(cm⁻¹)	σ (Å²)	E(cm⁻¹)	σ (Å²)	E(cm⁻¹)	σ (Å²)
430.074845	1.83587104	296.14969	12.5142368	296.14969	2.54269665
430.14969	1.83708582	296.582817	12.8244734	296.582817	2.55819987
430.374225	1.84086291	297.074845	12.4449181	297.074845	2.50327304
431.358282	1.86161555	297.358282	12.1188611	297.358282	2.46196782
431.374225	1.86204803	297.582817	11.8917592	297.582817	2.43259429
431.582817	1.86829929	298	11.5560122	298	2.38689136
432	1.88559806	298.074845	11.505453	298.074845	2.379689
432.074845	1.88979149	298.14969	11.4572389	298.14969	2.37272932
432.14969	1.89447126	298.582817	11.2146257	298.582817	2.3362831
432.358282	1.91084691	299.14969	10.9592588	299.14969	2.29532086
432.374225	1.91234618	299.358282	10.8765956	299.358282	2.28153402
433.358282	2.07007483	299.582817	10.7925386	299.582817	2.26728059
433.374225	2.07133175	300	10.6472774	300	2.24214446
433.582817	2.07496871	300.074845	10.6224928	300.074845	2.23779732
434	2.04229356	300.14969	10.5980554	300.14969	2.23349552
434.074845	2.03538648	300.582817	10.4628351	300.582817	2.20941652
434.14969	2.0287943	301.358282	10.2437734	301.358282	2.16941844
434.374225	2.01164551	301.582817	10.1853735	301.582817	2.1585179
435	1.98396189	302.074845	10.0652655	302.074845	2.13567218
435.358282	1.97697979	302.14969	10.0479674	302.14969	2.13232291
435.374225	1.97676374	302.358282	10.0011742	302.358282	2.12316772
435.582817	1.9745098	302.582817	9.95319887	302.582817	2.11361422
436	1.97247291	303.358282	9.80712458	303.358282	2.0830208
436.074845	1.97236999	303.582817	9.77030571	303.582817	2.07483796
436.14969	1.97233115	304	9.70687511	304	2.06029953
436.374225	1.9725517	304.074845	9.695976	304.074845	2.05776433
437.074845	1.97563843	304.14969	9.68516213	304.14969	2.05524588
437.358282	1.97763296	304.582817	9.62289105	304.582817	2.04088034
437.374225	1.97776026	305	9.560132	305	2.02707798
437.582817	1.97945071	305.358282	9.50176859	305.358282	2.01503764
438	1.98332855	305.582817	9.46281923	305.582817	2.00737569
438.074845	1.98409099	306	9.38619319	306	1.99294274
438.14969	1.98487367	306.074845	9.37197344	306.074845	1.99033574
438.374225	1.98734576	306.14969	9.35763961	306.14969	1.98772537
438.582817	1.98981378	306.582817	9.27312033	306.582817	1.97261523
439.14969	1.99745369	307.074845	9.1758791	307.074845	1.95560943
439.358282	2.00065414	307.358282	9.12019962	307.358282	1.94596455
439.374225	2.00090962	307.582817	9.07654167	307.582817	1.93842532
439.582817	2.00436359	308	8.99687283	308	1.92468421
440	2.01203395	308.074845	8.98280682	308.074845	1.92225773
440.074845	2.01351888	308.14969	8.96881536	308.14969	1.91984306
440.14969	2.01503607	308.582817	8.88936616	308.582817	1.90611275
440.374225	2.01977052	309.14969	8.78947398	309.14969	1.88877012
441.358282	2.04224339	309.358282	8.75390542	309.358282	1.8825668
441.374225	2.04260267	309.582817	8.71633286	309.582817	1.8759953

Final State: j'=5		Final State: j'=3		Final State: j'=2	
E(cm⁻¹)	σ (Å²)	E(cm⁻¹)	σ (Å²)	E(cm⁻¹)	σ (Å²)
441.582817	2.04718988	310	8.64847097	310	1.86407247
442	2.0555543	310.074845	8.6365614	310.074845	1.86197217
442.074845	2.05691846	310.14969	8.62473129	310.14969	1.85988365
442.14969	2.05823842	310.582817	8.55782205	310.582817	1.84802945
442.358282	2.06168447	311.358282	8.44434947	311.358282	1.82771626
442.374225	2.06193396	311.582817	8.41293359	311.582817	1.82204904
443.358282	2.07420825	312	8.35615807	312	1.81175986
443.374225	2.07436973	312.074845	8.34618231	312.074845	1.80994584
443.582817	2.07639564	312.14969	8.33626745	312.14969	1.8081416
444	2.08015801	312.358282	8.30894996	312.358282	1.80316165
444.074845	2.08080546	312.582817	8.28004787	312.582817	1.79787968
444.14969	2.08144728	313.358282	8.1837106	313.358282	1.78022372
444.374225	2.08334836	313.582817	8.15670701	313.582817	1.77526816
445	2.08858793	314	8.10742013	314	1.76622996
445.358282	2.09162945	314.074845	8.09869333	314.074845	1.76463303
445.374225	2.09176627	314.14969	8.08999031	314.14969	1.76303941
445.582817	2.09357044	314.582817	8.04020112	314.582817	1.75394887
446	2.09726665	315	7.99310611	315	1.74537577
446.074845	2.09794359	315.358282	7.95321611	315.358282	1.7381555
446.14969	2.09862478	315.582817	7.9284651	315.582817	1.7336915
446.374225	2.10069617	316	7.8829705	316	1.72552515
447.074845	2.10743726	316.074845	7.87487308	316.074845	1.72407573
447.358282	2.11028892	316.14969	7.86678814	316.14969	1.72262803
447.374225	2.11045417	316.582817	7.82041554	316.582817	1.71436817
447.582817	2.11260073	317.074845	7.76854943	317.074845	1.70517513
448	2.11701516	317.358282	7.73907244	317.358282	1.69997752
448.074845	2.11782364	317.582817	7.71592119	317.582817	1.69590231
448.14969	2.1186367	318	7.67342501	318	1.68844881
448.374225	2.12110619	318.074845	7.66587337	318.074845	1.68712733
448.582817	2.12343682	318.14969	7.658342	318.14969	1.68580931
449.14969	2.12993571	318.582817	7.61519197	318.582817	1.67827372
449.358282	2.13238076	319.14969	7.55985907	319.14969	1.6686387
449.374225	2.13256868	319.358282	7.53983285	319.358282	1.66515761
449.582817	2.13503898	319.582817	7.51847667	319.582817	1.66145043
450	2.14003638	320	7.47935721	320	1.65466686
450.074845	2.14093912	320.074845	7.47241631	320.074845	1.65346346
450.14969	2.14184337	320.14969	7.46549858	320.14969	1.65226525
450.374225	2.14456286	320.582817	7.4259224	320.582817	1.64540988
451.358282	2.15650025	321.358282	7.35701297	321.358282	1.63349678
451.374225	2.15669357	321.582817	7.33751887	321.582817	1.63012854
451.582817	2.15920529	322	7.30182948	322	1.62396543
452	2.16419253	322.074845	7.29549872	322.074845	1.62287265
452.074845	2.16508113	322.14969	7.28918998	322.14969	1.6217838
452.14969	2.16596764	322.358282	7.27172191	322.358282	1.61876927
452.358282	2.16842745	322.582817	7.25312364	322.582817	1.61555795

Final State: j'=5		Final State: j'=3		Final State: j'=2	
E(cm⁻¹)	σ (Å²)	E(cm⁻¹)	σ (Å²)	E(cm⁻¹)	σ (Å²)
452.374225	2.16861477	323.358282	7.19023259	323.358282	1.60471998
453.358282	2.17997969	323.582817	7.17242869	323.582817	1.60165387
453.374225	2.18016248	324	7.13980625	324	1.59604009
453.582817	2.18251928	324.074845	7.1340152	324.074845	1.59504434
454	2.18718891	324.14969	7.12824229	324.14969	1.5940516
454.074845	2.18802042	325	7.06390947	325	1.58300423
454.14969	2.18885002	325.358282	7.03746352	325.358282	1.57847096
454.374225	2.19132851	325.582817	7.0210814	325.582817	1.57566578
455	2.19816185	326	6.99101913	326	1.57052359
455.358282	2.20203385	326.074845	6.98567615	326.074845	1.56961031
455.374225	2.20220556	326.14969	6.98035408	326.14969	1.56870197
455.582817	2.20444792	327.074845	6.91572715	327.074845	1.55768171
456	2.20891261	327.358282	6.89636492	327.358282	1.55438772
456.074845	2.20971122	327.582817	6.88116956	327.582817	1.55180613
456.14969	2.21050918	328	6.85325884	328	1.54706875
456.374225	2.21289964	328.074845	6.8482934	328.074845	1.54622707
457.074845	2.22033359	328.14969	6.84334392	328.14969	1.54538716
457.358282	2.22333418	328.582817	6.81495191	328.582817	1.54057979
457.374225	2.22350234	329.14969	6.77843163	329.14969	1.53440448
457.582817	2.22570958	329.582817	6.75100565	329.582817	1.52977484
458	2.23012117	330	6.72497158	330	1.52538535
458.074845	2.23091255	330.074845	6.72033973	330.074845	1.52460451
458.14969	2.23170394	330.14969	6.7157198	330.14969	1.52382615
458.374225	2.23407816	331.358282	6.64270377	331.358282	1.51154339
458.582817	2.23628407	331.582817	6.62946066	331.582817	1.50931795
459.14969	2.24228113	332	6.60511211	332	1.50522843
459.358282	2.24448881	332.074845	6.60077914	332.074845	1.50450074
459.374225	2.24465714	332.14969	6.59645656	332.14969	1.50377517
459.582817	2.24686577	332.358282	6.58446472	332.358282	1.50176158
460	2.25128323	333.358282	6.52808382	333.358282	1.49229643
460.074845	2.25207584	333.582817	6.51566942	333.582817	1.49021164
460.14969	2.25286852	334	6.49283307	334	1.48637554
460.374225	2.25524639	334.074845	6.48876737	334.074845	1.48569304
461.358282	2.26566269	334.14969	6.48471107	334.14969	1.48501096
461.582817	2.26803667	335	6.43926875	335	1.47736685
462	2.27244322	335.358282	6.42046652	335.358282	1.47420064
462.074845	2.27323308	335.582817	6.40878193	335.582817	1.47222998
462.14969	2.27402266	336	6.38727264	336	1.46859883
462.358282	2.27622189	336.074845	6.38344009	336.074845	1.46795031
462.374225	2.27638992	336.14969	6.37961652	336.14969	1.46730445
463.358282	2.28673229	337.074845	6.33299406	337.074845	1.45940066
463.582817	2.28908361	337.358282	6.31893902	337.358282	1.45700899
464	2.29344282	337.582817	6.30787752	337.582817	1.4551231
464.074845	2.2942235	338	6.28749104	338	1.45163793
464.14969	2.29500371	338.074845	6.28385565	338.074845	1.45101514

Final State: j’=5		Final State: j’=3		Final State: j’=2	
E(cm⁻¹)	σ (Å²)	E(cm⁻¹)	σ (Å²)	E(cm⁻¹)	σ (Å²)
464.374225	2.29734215	338.14969	6.2802271	338.14969	1.45039298
465	2.30383775	338.582817	6.25935286	338.582817	1.44680518
465.358282	2.3075425	339.14969	6.23234286	339.14969	1.44213592
465.374225	2.30770724	339.358282	6.22248674	339.358282	1.44042373
465.582817	2.30985947	339.582817	6.21192469	339.582817	1.43858312
466	2.31415234	340	6.19242388	340	1.43516799
466.074845	2.31492093	340.074845	6.18894216	340.074845	1.43455558
466.374225	2.31799205	340.14969	6.18546447	340.14969	1.43394323
467.074845	2.32514962	341.358282	6.12988734	341.358282	1.42402899
467.358282	2.32803439	341.582817	6.11966302	341.582817	1.42217442
467.582817	2.33031557	342	6.10072893	342	1.41870826
468	2.33454404	342.074845	6.09733929	342.074845	1.4180831
468.074845	2.33530134	342.14969	6.09395119	342.14969	1.41745669
468.14969	2.33605819	342.358282	6.08451756	342.358282	1.41570459
468.374225	2.3383267	343.358282	6.03935441	343.358282	1.40712224
468.582817	2.34043059	343.582817	6.02919478	343.582817	1.40513938
469.14969	2.34613374	344	6.01025635	344	1.40138179
469.358282	2.34822722	344.074845	6.00684659	344.074845	1.40069598
469.582817	2.35047732	344.14969	6.00343197	344.14969	1.40000612
470	2.35464963	345	5.96417682	345	1.39182941
470.074845	2.35539698	345.358282	5.94725045	345.358282	1.38814097
470.14969	2.35614401	345.582817	5.93647172	345.582817	1.38573091
470.374225	2.35838297	346	5.91596789	346	1.38100188
471.358282	2.36816015	346.074845	5.91221032	346.074845	1.38011206
471.582817	2.37038313	346.14969	5.90842028	346.14969	1.37920803
472	2.37450539	347.074845	5.85818646	347.074845	1.36642969
472.074845	2.37524387	347.358282	5.84080056	347.358282	1.36163015
472.14969	2.37598203	347.582817	5.82590755	347.582817	1.35736473
472.358282	2.37803736	348	5.79413755	348	1.34783854
472.374225	2.37819438	348.074845	5.78762345	348.074845	1.34583069
473.358282	2.38785643	348.14969	5.7807712	348.14969	1.34370593
473.582817	2.3900531	348.582817	5.73123583	348.582817	1.32843717
474	2.39412664	349.14969	5.64200816	349.14969	1.31499182
474.074845	2.3948564	349.358282	5.65237423	349.358282	1.33059269
474.14969	2.39558578	349.582817	5.6515039	349.582817	1.33840457
474.374225	2.39777208	350	5.73671316	350	1.38959294
475	2.40384928	350.074845	5.753963	350.074845	1.39378278
475.358282	2.40731812	350.14969	5.76339061	350.14969	1.39401202
475.374225	2.40747225	351.358282	5.62902971	351.358282	1.36723782
476	2.41351165	351.582817	5.73588992	351.582817	1.41195023
476.074845	2.4142324	352	5.73624555	352	1.3966832
476.14969	2.41495279	352.074845	5.71798164	352.074845	1.39678818
476.374225	2.41711187	352.14969	5.7189205	352.14969	1.4061976
477.074845	2.42382875	352.358282	5.7520641	352.358282	1.38401372
477.358282	2.42653734	353.358282	5.56319393	353.358282	1.34367844

Final State: j'=5		Final State: j'=3		Final State: j'=2	
E(cm⁻¹)	σ (Å²)	E(cm⁻¹)	σ (Å²)	E(cm⁻¹)	σ (Å²)
477.582817	2.4286795	353.582817	5.58957116	353.582817	1.35221498
478	2.43265108	354	5.63497636	354	1.38596742
478.074845	2.43336246	354.074845	5.64345862	354.074845	1.38809656
478.14969	2.43407348	354.14969	5.64160897	354.14969	1.38651726
478.374225	2.43620437	355	5.66526513	355	1.3741334
478.582817	2.43818109	355.358282	5.57772107	355.358282	1.36035401
479.14969	2.44353907	355.582817	5.66626935	355.582817	1.39133797
479.358282	2.44550545	356	5.62260913	356	1.36348931
479.582817	2.4476191	356.074845	5.6090371	356.074845	1.35783108
480	2.45153752	356.14969	5.59467416	356.14969	1.35223664
480.074845	2.45223938	357.074845	5.51422692	357.074845	1.37612895
480.14969	2.45294083	357.358282	5.54689723	357.358282	1.40550975
480.374225	2.45504303	357.582817	5.64044802	357.582817	1.44039525
481.358282	2.46421813	358	5.6017625	358	1.36434978
481.582817	2.46630295	358.074845	5.57312255	358.074845	1.35043438
482	2.4701678	358.14969	5.54053332	358.14969	1.33761707
482.074845	2.47086002	358.582817	5.57426614	358.582817	1.42272106
482.14969	2.47155185	359.14969	5.61909586	359.14969	1.3881288
482.358282	2.4734781	359.358282	5.59328417	359.358282	1.37590273
482.374225	2.4736252	359.582817	5.56629791	359.582817	1.36428075
483.358282	2.48267343	360	5.51017965	360	1.34250801
483.582817	2.48472911	360.074845	5.49812542	360.074845	1.33818551
484	2.48854018	360.14969	5.48506932	360.14969	1.33365166
484.14969	2.48990473	361.358282	5.45089063	361.358282	1.40742995
484.374225	2.49194873	361.582817	5.57593258	361.582817	1.42068125
485	2.4976285	362	5.51532905	362	1.37880594
485.358282	2.50086892	362.074845	5.51027814	362.074845	1.3646679
485.374225	2.50101307	362.14969	5.48014103	362.14969	1.34641362
485.582817	2.50289579	362.358282	5.39468822	362.358282	1.34015905
486	2.506652	363.358282	5.53985028	363.358282	1.38347284
486.074845	2.50732467	363.582817	5.5184342	363.582817	1.37398816
486.14969	2.50799715	364	5.48089295	364	1.35862635
486.374225	2.51001154	364.074845	5.47403563	364.074845	1.35594342
487.074845	2.51627672	364.14969	5.46704175	364.14969	1.35323729
487.358282	2.51880162	365	5.35726799	365	1.31441179
487.582817	2.52079825	365.358282	5.26299429	365.358282	1.2904131
488	2.52449879	365.582817	5.2219182	365.582817	1.30374299
488.074845	2.52516143	366	5.36206884	366	1.40063744
488.14969	2.52582366	366.074845	5.36651038	366.074845	1.41035553
488.374225	2.52780819	367.074845	5.36312191	367.074845	1.3686726
488.582817	2.52964867	367.358282	5.55192955	367.358282	1.45479945
489.14969	2.5346353	367.582817	5.52508294	367.582817	1.41575222
489.358282	2.5364645	368	5.47136003	368	1.38645729
489.582817	2.53843023	368.074845	5.46419494	368.074845	1.38309241
490	2.54207307	368.14969	5.45744218	368.14969	1.38000382

Final State: j’=5		Final State: j’=3		Final State: j’=2	
E(cm⁻¹)	σ (Å²)	E(cm⁻¹)	σ (Å²)	E(cm⁻¹)	σ (Å²)
490.074845	2.54272532	368.582817	5.42335038	368.582817	1.36543476
490.14969	2.54337716	369.14969	5.38348109	369.14969	1.34990613
490.374225	2.54533033	369.358282	5.36846559	369.358282	1.34430109
491.358282	2.55384738	369.582817	5.35127984	369.582817	1.33798659
491.582817	2.55578093	370	5.31357026	370	1.32446464
492	2.55936335	370.074845	5.30546755	370.074845	1.32163092
492.074845	2.56000468	370.14969	5.29675248	370.14969	1.31861939
492.14969	2.56064557	371.358282	5.19643896	371.358282	1.36644375
492.358282	2.56242956	371.582817	5.26663251	371.582817	1.43060873
492.374225	2.56256583	372	5.30701737	372	1.34747119
493.358282	2.57093634	372.074845	5.32188255	372.074845	1.35835123
493.582817	2.57283573	372.14969	5.31260943	372.14969	1.35406056
494	2.57635412	372.358282	5.18023891	372.358282	1.32201391
494.074845	2.57698406	373.358282	5.38352096	373.358282	1.38106283
494.14969	2.5776134	373.582817	5.36263028	373.582817	1.37127748
494.374225	2.57949869	374	5.33221564	374	1.35826021
495	2.58473098	374.074845	5.32740215	374.074845	1.35631203
495.358282	2.58771211	374.14969	5.32271273	374.14969	1.35443944
495.374225	2.58784458	375	5.2734041	375	1.33583072
495.582817	2.58957511	375.358282	5.2522558	375.358282	1.32818585
496	2.59302464	376	5.20737591	376	1.31218656
496.074845	2.5936418	376.074845	5.20097819	376.074845	1.30993579
496.14969	2.5942586	376.14969	5.19418273	376.14969	1.30755915
496.374225	2.59610601	377.074845	5.01514098	377.074845	1.26063996
497.074845	2.60184106	377.358282	4.98323764	377.358282	1.2893981
497.358282	2.60414837	377.582817	5.11887452	377.582817	1.37070497
497.582817	2.60597068	378	5.25540346	378	1.36479722
498	2.60934383	378.074845	5.23020004	378.074845	1.34585333
498.074845	2.60994722	378.14969	5.19702855	378.14969	1.32751349
498.14969	2.61054999	378.582817	5.11940049	378.582817	1.36243535
498.374225	2.61235506	379.14969	5.30354997	379.14969	1.38022979
498.582817	2.6140274	379.358282	5.27073266	379.358282	1.36265921
499.14969	2.61854926	379.582817	5.24586629	379.582817	1.35069077
499.358282	2.6202046	380	5.21136057	380	1.33541183
499.582817	2.62198119	380.074845	5.20597415	380.074845	1.33313423
500	2.62526227	380.14969	5.20072957	380.14969	1.33093866
500.074845	2.62585443	381.358282	5.11931761	381.358282	1.29824912
500.14969	2.62644112	381.582817	5.10162248	381.582817	1.29124162
500.374225	2.62819731	382	5.06120687	382	1.27544979
501.358282	2.63582127	382.074845	5.05225254	382.074845	1.2720522
501.582817	2.63754327	382.14969	5.04252449	382.14969	1.26843607
502.074845	2.64129247	382.358282	5.01021426	382.358282	1.25731764
502.14969	2.64185977	383.358282	4.95045016	383.358282	1.27839593
502.358282	2.64343645	383.582817	4.96786565	383.582817	1.29094484
502.374225	2.6435567	384	4.79729414	384	1.23018264

Final State: j'=5		Final State: j'=3		Final State: j'=2	
E(cm⁻¹)	σ (Å²)	E(cm⁻¹)	σ (Å²)	E(cm⁻¹)	σ (Å²)
503.358282	2.65090088	384.14969	4.759773	384.14969	1.2462273
503.582817	2.65255386	385	5.03390009	385	1.31111573
504.074845	2.65614387	385.358282	5.04234705	385.358282	1.37145637
504.14969	2.65668569	385.582817	5.15601231	385.582817	1.41932201
504.374225	2.65830509	386	5.05247664	386	1.326818
505	2.66275641	386.074845	5.03479945	386.074845	1.32017014
505.358282	2.66527225	386.14969	5.01463814	386.14969	1.31402511
505.374225	2.66538471	387.074845	5.15995446	387.074845	1.3784065
505.582817	2.66682924	387.358282	5.12879522	387.358282	1.35486078
506.074845	2.67019305	387.582817	5.0971773	387.582817	1.33712889
506.14969	2.67069851	388	5.02266646	388	1.30292008
506.374225	2.67220457	388.074845	5.00485432	388.074845	1.295907
507.074845	2.67679291	388.14969	4.98473339	388.14969	1.28851676
507.358282	2.67859544	388.582817	4.84232549	388.582817	1.28035841
507.582817	2.67999843	389.14969	5.06642328	389.14969	1.38204109
508.074845	2.6829861	389.358282	5.1300089	389.358282	1.38848962
508.14969	2.68342934	389.582817	5.10991084	389.582817	1.3669951
508.374225	2.68473813	390	5.05697344	390	1.33448173
508.582817	2.68592507	390.074845	5.04698559	390.074845	1.3292691
509.14969	2.6889866	390.14969	5.03672797	390.14969	1.32409361
509.358282	2.69004298	391.358282	4.63747083	391.358282	1.19658874
509.582817	2.69112889	391.582817	4.54446503	391.582817	1.25931334
510	2.69297703	392	4.97089294	392	1.46786742
510.074845	2.69328957	392.074845	5.09728743	392.074845	1.47598474
510.14969	2.6935871	392.14969	5.12673838	392.14969	1.46140893
510.374225	2.69442135	392.358282	5.15035443	392.358282	1.41685554
511.358282	2.69657902	393.358282	5.13345451	393.358282	1.38154693
511.582817	2.69653456	393.582817	5.09562941	393.582817	1.36286618
512.074845	2.69510315	394	5.03421546	394	1.33418693
512.14969	2.6946494	394.074845	5.02258987	394.074845	1.32901069
512.358282	2.69287845	394.14969	5.01035744	394.14969	1.3236619
513.358282	2.65902416	395	4.71471351	395	1.26484751
513.582817	2.67081956	395.358282	4.8548763	395.358282	1.33675144
514.074845	2.68755109	395.582817	5.12889555	395.582817	1.45577916
514.14969	2.70128103	396	5.10655867	396	1.39624741
514.374225	2.72342919	396.074845	5.09677429	396.074845	1.38933679
515	2.69372272	396.14969	5.08755209	396.14969	1.38319677
515.358282	2.68780955	397.074845	4.99797347	397.074845	1.33435975
515.374225	2.68664743	397.358282	4.97231215	397.358282	1.32242762
515.582817	2.68746653	397.582817	4.95031056	397.582817	1.31258621
516.074845	2.74796947	398	4.90016252	398	1.29124889
516.14969	2.74319215	398.074845	4.88890781	398.074845	1.28666602
516.374225	2.7609543	398.14969	4.87656331	398.14969	1.28173132
517.074845	2.73218323	398.582817	4.76388206	398.582817	1.24208698
517.358282	2.69843216	399.14969	4.67645213	399.14969	1.28665693

Final State: j’=5		Final State: j’=3		Final State: j’=2	
E(cm⁻¹)	σ (Å²)	E(cm⁻¹)	σ (Å²)	E(cm⁻¹)	σ (Å²)
517.582817	2.70690788	399.358282	4.81366381	399.358282	1.39319033
518.074845	2.72820789	399.582817	4.83297162	399.582817	1.3914044
518.14969	2.74012208	400	5.04588641	400	1.40311047
518.374225	2.74890587	400.074845	5.00257627	400.074845	1.37306079
518.582817	2.76505581	400.14969	4.94418175	400.14969	1.35075162
519.14969	2.77345799	401.358282	4.93214286	401.358282	1.32876043
519.358282	2.75472637	401.582817	4.86763622	401.582817	1.32244738
519.582817	2.74357977	402.074845	4.8807223	402.074845	1.30463541
520	2.78069825	402.14969	4.8522046	402.14969	1.29377973
520.074845	2.77787396	402.358282	4.77451079	402.358282	1.28966237
520.14969	2.77491695	403.358282	4.49431788	403.358282	1.26542409
520.374225	2.76463262	403.582817	4.53684799	403.582817	1.30034716
521.358282	2.75426859	404	5.09217649	404	1.4495098
521.582817	2.76935069	404.074845	5.07410916	404.074845	1.4319048
522.074845	2.81239826	404.14969	5.05548362	404.14969	1.41657177
522.14969	2.80735419	405	4.78443037	405	1.27835274
522.358282	2.78144393	405.358282	4.93029138	405.358282	1.3991493
522.374225	2.7781526	405.582817	4.97693875	405.582817	1.38806891
523.358282	2.82064433	406	4.91663993	406	1.3431016
523.582817	2.81533493	406.074845	4.90493883	406.074845	1.33639957
524.074845	2.80290924	406.14969	4.89294268	406.14969	1.32979959
524.14969	2.80045688	407.074845	4.54810149	407.074845	1.25563607
524.374225	2.79103483	407.358282	4.81962833	407.358282	1.32819413
525	2.73639877	407.582817	4.7041124	407.582817	1.27131819
525.358282	2.78658521	408	4.45070668	408	1.31431892
525.374225	2.78867046	408.074845	4.55684354	408.074845	1.36486525
525.582817	2.8254859	408.14969	4.5831349	408.14969	1.34399491
526.074845	2.82815377	408.582817	4.95385243	408.582817	1.38576618
526.14969	2.81594218	409.14969	4.69770302	409.14969	1.36054807
526.374225	2.80327449	409.358282	5.04463427	409.358282	1.44730623
527.074845	2.87030973	409.582817	5.05415917	409.582817	1.44782291
527.358282	2.86209151	410	4.77398141	410	1.34595462
527.582817	2.85776342	410.074845	4.99560592	410.074845	1.41519828
528.074845	2.85030773	410.14969	4.97511956	410.14969	1.38845127
528.14969	2.8491967	410.374226	4.88316443	410.374226	1.33161845
528.374225	2.84561871	410.374227	4.88314196	410.374227	1.3316131
528.582817	2.84167961	410.374228	4.88312382	410.374228	1.33160855
529.14969	2.82282528	410.374229	4.88310789	410.374229	1.33160441
529.582817	2.7802562	410.37423	4.88309339	410.37423	1.33160053
530	2.7939372	410.374231	4.88307989	410.374231	1.33159684
530.074845	2.80810228	410.374232	4.88306715	410.374232	1.33159329
530.14969	2.81209112	410.374233	4.88305501	410.374233	1.33158984
530.374225	2.87393463	410.374234	4.88304335	410.374234	1.33158649
531.582817	2.9239126	410.374235	4.8830321	410.374235	1.33158322
532.074845	2.90402283	410.374236	4.8830212	410.374236	1.33158001

**Final State: j'=5**		**Final State: j'=3**		**Final State: j'=2**	
E(cm⁻¹)	σ (Å²)	E(cm⁻¹)	σ (Å²)	E(cm⁻¹)	σ (Å²)
532.14969	2.90242716	410.374237	4.88301059	410.374237	1.33157685
532.358282	2.89883897	410.374238	4.88300025	410.374238	1.33157374
532.374225	2.89861104	410.374239	4.88299015	410.374239	1.33157068
533.582817	2.88549009	410.37424	4.88298025	410.37424	1.33156766
534.074845	2.87810911	410.374241	4.88297053	410.374241	1.33156467
534.14969	2.87655495	410.374242	4.88296098	410.374242	1.33156171
534.374225	2.87065652	410.374243	4.88295159	410.374243	1.33155878
535	2.82664496	410.374244	4.88294232	410.374244	1.33155587
535.374225	2.80197861	410.374245	4.88293318	410.374245	1.331553
535.582817	2.8365818	410.374246	4.88292418	410.374246	1.33155014
536.074845	2.90362903	410.374247	4.88291528	410.374247	1.3315473
536.14969	2.91191443	410.374248	4.88290649	410.374248	1.33154448
536.374225	2.86125088	410.374249	4.88289778	410.374249	1.33154168
537.074845	2.96369458	410.37425	4.88288916	410.37425	1.3315389
537.358282	2.94920456	410.374251	4.88288065	410.374251	1.33153614
537.582817	2.94322921	410.374252	4.88287219	410.374252	1.33153339
538.074845	2.9359543	410.374253	4.88286382	410.374253	1.33153065
538.14969	2.93518687	410.374254	4.88285551	410.374254	1.33152792
538.374225	2.93316254	410.374255	4.88284725	410.374255	1.33152521
538.582817	2.93153224	410.374256	4.88283909	410.374256	1.33152251
539.14969	2.92749808	410.374257	4.88283097	410.374257	1.33151982
539.582817	2.92400651	410.374258	4.8828229	410.374258	1.33151714
540	2.9193446	410.374259	4.88281489	410.374259	1.33151447
540.074845	2.91827798	410.37426	4.88280693	410.37426	1.33151182
540.14969	2.91709911	410.374261	4.88279902	410.374261	1.33150917
540.374225	2.91274205	410.374262	4.88279116	410.374262	1.33150653
541.582817	2.8319424	410.374263	4.88278334	410.374263	1.33150389
542.074845	2.96102586	410.374264	4.88277556	410.374264	1.33150127
542.14969	2.9522391	410.374265	4.88276782	410.374265	1.33149865
542.358282	2.91660263	410.374266	4.88276012	410.374266	1.33149604
542.374225	2.92077321	410.374267	4.88275246	410.374267	1.33149343
543.582817	2.97928108	410.374268	4.88274484	410.374268	1.33149084
544.074845	2.96890743	410.374269	4.88273725	410.374269	1.33148826
544.14969	2.96779484	410.37427	4.8827297	410.37427	1.33148568
544.374225	2.96479543	410.374271	4.88272218	410.374271	1.3314831
545	2.95747105	410.374272	4.88271469	410.374272	1.33148053
545.374225	2.95275226	410.374273	4.88270723	410.374273	1.33147797
545.582817	2.9496564	410.374274	4.88269979	410.374274	1.33147541
546.074845	2.93952444	410.374275	4.88269239	410.374275	1.33147286
546.14969	2.93738207	410.374276	4.88268501	410.374276	1.33147032
546.374225	2.92916049	410.374277	4.88267766	410.374277	1.33146777
547.074845	2.90380921	410.374278	4.88267033	410.374278	1.33146524
547.358282	2.88461481	410.374279	4.88266303	410.374279	1.3314627
547.582817	2.90301926	410.37428	4.88265575	410.37428	1.33146018
548.074845	2.87290583	410.374281	4.8826485	410.374281	1.33145765

Final State: j'=5		Final State: j'=3		Final State: j'=2	
E(cm⁻¹)	σ (Å²)	E(cm⁻¹)	σ (Å²)	E(cm⁻¹)	σ (Å²)
548.14969	2.85026886	410.374282	4.88264126	410.374282	1.33145513
548.374225	2.82209202	410.374283	4.88263405	410.374283	1.33145262
548.582817	2.86813047	410.374284	4.88262686	410.374284	1.33145011
549.14969	2.93908116	410.374285	4.88261969	410.374285	1.3314476
549.582817	2.95413439	410.374286	4.88261254	410.374286	1.3314451
550	3.00032738	410.374287	4.8826054	410.374287	1.3314426
550.074845	2.98533898	410.374288	4.88259829	410.374288	1.3314401
550.14969	2.97064972	410.374289	4.88259119	410.374289	1.33143761
550.374225	2.94386902	410.37429	4.88258412	410.37429	1.33143512
551.582817	3.02249671	410.374291	4.88257706	410.374291	1.33143264
552.074845	2.99713214	410.374292	4.88257002	410.374292	1.33143016
552.14969	2.99179124	410.374293	4.88256296	410.374293	1.33142767
552.358282	2.97175055	410.374294	4.88255594	410.374294	1.33142519
552.374225	2.96977606	410.374295	4.88254894	410.374295	1.33142272
553.582817	3.0497119	410.374296	4.88254197	410.374296	1.33142025
554.074845	3.03046764	410.374297	4.882535	410.374297	1.33141779
554.14969	3.02740455	410.374298	4.88252805	410.374298	1.33141532
554.374225	3.01777569	410.374299	4.88252111	410.374299	1.33141286
555	2.97743029	410.3743	4.88251418	410.3743	1.3314104
555.374225	2.91564377	410.374301	4.88250727	410.374301	1.33140795
555.582817	2.82968992	410.374302	4.88250037	410.374302	1.33140549
556.074845	2.95549936	410.374303	4.88249348	410.374303	1.33140304
556.14969	3.00537119	410.374304	4.8824866	410.374304	1.33140059
556.374225	3.08159028	410.374305	4.88247973	410.374305	1.33139815
557.074845	3.1451521	410.374306	4.88247288	410.374306	1.3313957
557.358282	3.1054629	410.374307	4.88246604	410.374307	1.33139326
557.582817	3.08916838	410.374308	4.88245921	410.374308	1.33139082
558.074845	3.06498303	410.374309	4.8824524	410.374309	1.33138839
558.14969	3.06139121	410.37431	4.88244559	410.37431	1.33138596
558.374225	3.04933763	410.374311	4.88243879	410.374311	1.33138352
558.582817	3.03452258	410.374312	4.88243201	410.374312	1.33138109
559.14969	2.91572786	410.374313	4.88242522	410.374313	1.33137866
559.582817	2.98253642	410.374314	4.88241846	410.374314	1.33137624
560	3.12077597	410.374315	4.88241171	410.374315	1.33137381
560.074845	3.11660841	410.374316	4.88240496	410.374316	1.33137139
560.14969	3.11277097	410.374317	4.88239823	410.374317	1.33136897
560.374225	3.10314175	410.374318	4.8823915	410.374318	1.33136655
561.582817	3.06922173	410.374319	4.88238478	410.374319	1.33136413
562.074845	3.05239324	410.37432	4.88237808	410.37432	1.33136172
562.14969	3.04901596	410.374321	4.88237136	410.374321	1.3313593
562.358282	3.03743728	410.374322	4.88236467	410.374322	1.33135689
562.374225	3.03639114	410.374323	4.88235798	410.374323	1.33135448
563.582817	3.03932173	410.374324	4.88235132	410.374324	1.33135208
564.074845	3.11168598	410.374325	4.88234464	410.374325	1.33134967
564.14969	3.12904716	410.374825	4.87911881	410.374825	1.33018015

Final State: j’=5		Final State: j’=3		Final State: j’=2	
E(cm⁻¹)	σ (Å²)	E(cm⁻¹)	σ (Å²)	E(cm⁻¹)	σ (Å²)
564.374225	3.0691556	410.375225	4.87627934	410.375225	1.32921648
565	3.15144491	410.375325	4.87551935	410.375325	1.32897023
565.374225	3.09004279	410.375825	4.87139145	410.375825	1.32771108
565.582817	3.08398039	410.376225	4.86769705	410.376225	1.32668649
566.074845	3.09822655	410.376325	4.86671923	410.376325	1.32643031
566.374225	3.05422961	410.376825	4.86154941	410.376825	1.32517506
567.074845	3.05925586	410.377225	4.85713518	410.377225	1.3242321
567.358282	2.96917536	410.377325	4.85600536	410.377325	1.32400967
567.582817	2.92895138	410.377825	4.85029229	410.377825	1.32301069
568.074845	3.19705654	410.378225	4.84579408	410.378225	1.32238634
568.14969	3.19272911	410.378325	4.84469924	410.378325	1.32225908
568.374225	3.17070792	410.378825	4.83955446	410.378825	1.32182181
568.582817	3.1516016	410.379225	4.83597384	410.379225	1.32172474
569.14969	3.07002877	410.379325	4.83517621	410.379325	1.32173783
569.582817	3.15106267	410.379825	4.83181803	410.379825	1.32200819
570	3.14418345	410.380225	4.82994798	410.380225	1.32245124
570.074845	3.13990007	410.380325	4.82960329	410.380325	1.32259103
570.14969	3.1356039	410.380825	4.82854668	410.380825	1.32342219
570.374225	3.12225435	410.381225	4.82845624	410.381225	1.32420697
571.582817	3.09692853	410.381325	4.82853098	410.381325	1.32441494
572.074845	2.89976451	410.381825	4.82936788	410.381825	1.32549018
572.14969	2.89036459	410.382225	4.83051092	410.382225	1.32636452
572.358282	3.0182688	410.382325	4.83084858	410.382325	1.3265807
572.374225	3.01447855	410.382825	4.83277095	410.382825	1.32763979
573.582817	3.17858878	410.383225	4.83448611	410.383225	1.3284348
574.074845	3.16743205	410.383325	4.83493799	410.383325	1.32862689
574.14969	3.09377452	410.383825	4.83721869	410.383825	1.32952835
574.374225	3.18908041	410.384225	4.83905257	410.384225	1.33018109
575	3.11154366	410.384325	4.83951373	410.384325	1.33033693
575.374225	3.02992532	410.384825	4.84174721	410.384825	1.33105136
576.074845	3.0928075	410.385225	4.84345614	410.385225	1.33155636
576.14969	3.08751147	410.385325	4.84387954	410.385325	1.33167681
576.374225	3.07138223	410.385825	4.84588192	410.385825	1.33221968
577.074845	2.9134071	410.386225	4.84738224	410.386225	1.33259945
577.358282	3.13149217	410.386325	4.8477379	410.386325	1.33268634
577.582817	3.08481999	410.386825	4.84946435	410.386825	1.3330896
578.074845	3.0804213	410.387225	4.85074078	410.387225	1.33336843
578.14969	3.08041039	410.387325	4.85104262	410.387325	1.33343207
578.374225	3.0790228	410.387825	4.85249129	410.387825	1.33372491
578.582817	3.07641996	410.388225	4.85355579	410.388225	1.33392358
579.14969	3.06482774	410.388325	4.85381319	410.388325	1.33397
579.582817	3.05306991	410.388825	4.85501331	410.388825	1.3341739
580	3.0399918	410.389225	4.85590033	410.389225	1.33431511
580.074845	3.03699508	410.389325	4.85611326	410.389325	1.33434668
580.14969	3.03350248	410.389825	4.85710932	410.389825	1.33448702

Final State: j'=5		Final State: j'=3		Final State: j'=2	
E(cm⁻¹)	σ (Å²)	E(cm⁻¹)	σ (Å²)	E(cm⁻¹)	σ (Å²)
580.374225	3.01676268	410.390225	4.85783743	410.390225	1.33457871
581.582817	3.04212646	410.390325	4.85801673	410.390325	1.33460052
582.074845	3.07034111	410.390825	4.85884125	410.390825	1.33469156
582.14969	3.07511884	410.391225	4.85943763	410.391225	1.33474586
582.358282	3.0787332	410.391325	4.85957988	410.391325	1.33475708
582.374225	3.07876372	410.391825	4.86025673	410.391825	1.33480938
583.582817	3.07227589	410.392225	4.86075283	410.392225	1.33483811
584.14969	3.0696865	410.392325	4.86086981	410.392325	1.33484297
584.374225	3.06855959	410.392825	4.86141995	410.392825	1.33486057
585	3.06398055	410.393225	4.8618297	410.393225	1.33486845
585.374225	3.0593237	410.393325	4.861928	410.393325	1.33486958
585.582817	3.0560065	410.393825	4.86237373	410.393825	1.33486294
586.14969	3.04894769	410.394225	4.86270551	410.394225	1.33485312
586.374225	3.04920717	410.395225	4.86340962	410.395225	1.33479675
587.074845	3.05617488	410.396225	4.86397052	410.396225	1.33470971
587.358282	3.05850754	410.397225	4.86441514	410.397225	1.33460035
587.582817	3.0597735	410.398225	4.8647414	410.398225	1.33446515
588.14969	3.06048456	410.399225	4.86497812	410.399225	1.33431154
588.374225	3.05948289	410.400225	4.86513697	410.400225	1.33414829
588.582817	3.05734056	410.401225	4.86522513	410.401225	1.33396746
589.14969	3.00970984	410.402225	4.86524321	410.402225	1.33377815
589.582817	3.04676202	410.403225	4.86520368	410.403225	1.3335759
590	3.0872374	410.404225	4.86511486	410.404225	1.33336847
590.14969	3.08609604	410.405225	4.86497304	410.405225	1.33315059
590.374225	3.08338936	410.406225	4.86479825	410.406225	1.33292799
591.582817	3.07941619	410.407225	4.86457225	410.407225	1.33269497
592.074845	3.07982611	410.408225	4.86431404	410.408225	1.33246131
592.14969	3.0799421	410.409225	4.86402399	410.409225	1.33221985
592.358282	3.08028164	410.410225	4.86370237	410.410225	1.33197523
592.374225	3.08031801	410.411225	4.8633573	410.411225	1.33172769
593.582817	3.08298671	410.412225	4.862975	410.412225	1.33147663
594.14969	3.08429998	410.413225	4.86257452	410.413225	1.33122153
594.374225	3.08479725	410.414225	4.8621439	410.414225	1.33095936
595	3.0860439	410.415225	4.86170109	410.415225	1.33070321
595.374225	3.08664833	410.416225	4.86123122	410.416225	1.33043962
595.582817	3.08689774	410.417225	4.8607426	410.417225	1.33017579
596.14969	3.08702365	410.418225	4.86023413	410.418225	1.32990741
596.374225	3.08668899	410.419225	4.85971442	410.419225	1.32964115
597.074845	3.08322665	410.420225	4.85917478	410.420225	1.32937012
597.358282	3.08230302	410.421225	4.85861886	410.421225	1.32909911
597.582817	3.0835211	410.422225	4.85805626	410.422225	1.32883103
598.14969	3.09041422	410.423225	4.85747362	410.423225	1.32855891
598.374225	3.09267515	410.424225	4.85688494	410.424225	1.32828661
598.582817	3.09436143	410.425225	4.85628766	410.425225	1.32801405
599.14969	3.09765262	410.426225	4.85567332	410.426225	1.32774203

Final State: j’=5		Final State: j’=3		Final State: j’=2	
E(cm⁻¹)	σ (Å²)	E(cm⁻¹)	σ (Å²)	E(cm⁻¹)	σ (Å²)
599.582817	3.09951267	410.427225	4.85505702	410.427225	1.32746896
600	3.1010585	410.428225	4.85442691	410.428225	1.32719664
600.14969	3.10158963	410.429225	4.85379096	410.429225	1.32692463
600.374225	3.10235161	410.430225	4.85315416	410.430225	1.326655
601.582817	3.10616152	410.431225	4.85251047	410.431225	1.32638428
602.074845	3.10761208	410.432225	4.85185426	410.432225	1.3261124
602.14969	3.10783752	410.433225	4.85120549	410.433225	1.3258426
602.358282	3.10843113	410.434225	4.85054966	410.434225	1.32557645
602.374225	3.10848434	410.435225	4.84988945	410.435225	1.32530756
603.582817	3.11177855	410.436225	4.84922298	410.436225	1.32503768
604.14969	3.11320411	410.437225	4.84856729	410.437225	1.32477658
604.374225	3.11375107	410.438225	4.84789915	410.438225	1.32450971
605	3.11525202	410.439225	4.84723426	410.439225	1.32424518
605.374225	3.11618709	410.440225	4.84656812	410.440225	1.32398092
605.582817	3.11672454	410.441225	4.8459121	410.441225	1.32372213
606.14969	3.11828502	410.442225	4.84524403	410.442225	1.3234623
606.374225	3.11894442	410.443225	4.84458883	410.443225	1.32320287
607.074845	3.12110675	410.444225	4.84392664	410.444225	1.32294363
607.358282	3.12201365	410.445225	4.84326562	410.445225	1.32268361
607.582817	3.12274402	410.446225	4.84261156	410.446225	1.32242835
608.14969	3.12456932	410.447225	4.84195986	410.447225	1.32217297
608.374225	3.12528787	410.448225	4.8413042	410.448225	1.321914
608.582817	3.12595196	410.449225	4.84065122	410.449225	1.32165779
609.14969	3.12773658	410.450225	4.84000577	410.450225	1.32140282
609.582817	3.1290781	410.451225	4.83936111	410.451225	1.32115034
610	3.13034588	410.452225	4.83871823	410.452225	1.32089591
610.14969	3.13080661	410.453225	4.83807392	410.453225	1.32063979
610.374225	3.13148234	410.454225	4.83744136	410.454225	1.32038833
611.582817	3.13504428	410.455225	4.83679639	410.455225	1.32013087
612.074845	3.13645305	410.456225	4.83615777	410.456225	1.31987697
612.14969	3.13667459	410.457225	4.83552114	410.457225	1.31962103
612.358282	3.13726108	410.458225	4.83489549	410.458225	1.31936697
613.582817	3.14070528	410.459225	4.83425888	410.459225	1.31911081
614.14969	3.14227369	410.460225	4.8336305	410.460225	1.31885411
614.374225	3.14289232	410.461225	4.83299749	410.461225	1.31859473
615	3.14460395	410.462225	4.83237121	410.462225	1.31833725
615.374225	3.14563739	410.463225	4.83173406	410.463225	1.31807511
615.582817	3.14620865	410.464225	4.83110492	410.464225	1.31781362
616.14969	3.14775993	410.465225	4.83046262	410.465225	1.31754928
616.374225	3.14837425	410.466225	4.82982762	410.466225	1.31728416
617.074845	3.15028154	410.467225	4.82918977	410.467225	1.31701505
617.358282	3.1510558	410.468225	4.82855212	410.468225	1.31674926
617.582817	3.15167611	410.469225	4.82791517	410.469225	1.31647686
618.14969	3.15322085	410.470225	4.82726579	410.470225	1.31620267
618.374225	3.15383131	410.471225	4.82660864	410.471225	1.31592576

Final State: j’=5		Final State: j’=3		Final State: j’=2	
E(cm⁻¹)	σ (Å²)	E(cm⁻¹)	σ (Å²)	E(cm⁻¹)	σ (Å²)
618.582817	3.15439802	410.472225	4.82596085	410.472225	1.31564707
619.14969	3.15593436	410.473225	4.82530408	410.473225	1.31536435
619.582817	3.15710403	410.474225	4.82464193	410.474225	1.31508142
620	3.15821928	410.574225	4.56651684	410.574225	1.27886526
620.14969	3.15862882	410.674225	4.80185935	410.674225	1.32924817
620.374225	3.15923101	410.774225	4.78354617	410.774225	1.31512469
621.582817	3.16244835	410.874225	4.75798786	410.874225	1.3027305
622.074845	3.16373952	410.974225	4.72218163	410.974225	1.28868354
622.14969	3.16394413	411.074225	4.67438279	411.074225	1.27278658
622.358282	3.16448472	411.174225	4.61571846	411.174225	1.25346837
622.374225	3.16453418	411.274225	4.49446883	411.274225	1.21960907
623.582817	3.16768679	411.358282	4.35938907	411.358282	1.24513076
624.14969	3.16915216	411.374225	4.37878507	411.374225	1.25883016
624.374225	3.16973024	411.474225	4.78759926	411.474225	1.39160022
625	3.17132715	411.574225	4.78179899	411.574225	1.3573309
625.374225	3.17228983	411.582817	4.77970963	411.582817	1.35516736
625.582817	3.17282064	411.674225	4.75667946	411.674225	1.33692724
626.14969	3.17425803	411.774225	4.733964	411.774225	1.32341889
626.374225	3.17482546	411.874225	4.71398706	411.874225	1.31333146
627.074845	3.17658084	411.974225	4.69560005	411.974225	1.30496215
627.358282	3.17729096	412	4.69098647	412	1.302963
627.582817	3.17785989	412.074225	4.67773902	412.074225	1.29737623
628.14969	3.17927224	412.074845	4.67762685	412.074845	1.29733434
628.374225	3.1798298	412.14969	4.66397782	412.14969	1.29178704
628.582817	3.18034678	412.174225	4.65935936	412.174225	1.28994642
629.14969	3.18174716	412.274225	4.63925322	412.274225	1.28212473
629.582817	3.18281261	412.358282	4.61977221	412.358282	1.27481569
630	3.18382755	412.374225	4.61568649	412.374225	1.27331531
630.14969	3.1842011	412.474225	4.58583573	412.474225	1.26272362
630.374225	3.18474923	412.574225	4.54425073	412.574225	1.24917289
631.582817	3.18768142	412.674225	4.47901911	412.674225	1.23110517
632.074845	3.18885881	412.774225	4.36302836	412.774225	1.21052222
632.14969	3.18904618	412.874225	4.25467369	412.874225	1.26412191
632.358282	3.18953911	412.974225	4.38585173	412.974225	1.30183078
632.374225	3.18958486	413.074225	4.60927299	413.074225	1.42762702
633.582817	3.19246616	413.174225	4.78280707	413.174225	1.40920542
634.14969	3.19380695	413.274225	4.72687394	413.274225	1.36563528
634.374225	3.19433613	413.358282	4.68697352	413.358282	1.34656321
635	3.19579785	413.374225	4.68111835	413.374225	1.34409212
635.374225	3.19668005	413.474225	4.65693295	413.474225	1.33407345
635.582817	3.19716628	413.574225	4.64870038	413.574225	1.32944438
636.14969	3.19848313	413.582817	4.6484143	413.582817	1.32916184
636.374225	3.19900286	413.674225	4.64719533	413.674225	1.32658682
637.074845	3.20061032	413.774225	4.64689716	413.774225	1.32407431
637.358282	3.20126076	413.874225	4.64591648	413.874225	1.32157484

Final State: j'=5		Final State: j'=3		Final State: j'=2	
E(cm⁻¹)	σ (Å²)	E(cm⁻¹)	σ (Å²)	E(cm⁻¹)	σ (Å²)
637.582817	3.20178231	413.974225	4.64387529	413.974225	1.31906165
638.14969	3.20307555	414	4.64321252	414	1.31841578
638.374225	3.20358594	414.074225	4.64093597	414.074225	1.31656714
638.582817	3.2040592	414.074845	4.64089794	414.074845	1.31654792
639.14969	3.20534076	414.14969	4.63815424	414.14969	1.31470211
639.582817	3.20631552	414.174225	4.63716646	414.174225	1.3141025
640	3.20724341	414.274225	4.63279288	414.274225	1.31168347
640.14969	3.20758557	414.374225	4.62791093	414.374225	1.30931639
640.374225	3.20808684	414.474225	4.62255364	414.474225	1.30698329
641.582817	3.21076757	414.574225	4.61674284	414.574225	1.30467501
642.074845	3.21184318	414.674225	4.61055174	414.674225	1.30239237
642.14969	3.21201492	414.774225	4.60393662	414.774225	1.30012661
642.358282	3.21246486	414.874225	4.59696646	414.874225	1.29787941
642.374225	3.21250719	414.974225	4.58966327	414.974225	1.29565479
643.582817	3.21514001	415	4.58773842	415	1.29508584
644.14969	3.21636497	415.074225	4.58208714	415.074225	1.29345548
644.374225	3.2168485	415.174225	4.57428973	415.174225	1.29128693
645	3.21818343	415.274225	4.56636332	415.274225	1.28914636
645.374225	3.21898989	415.358282	4.55960545	415.358282	1.28735636
645.582817	3.21943409	415.374225	4.55831551	415.374225	1.28701709
646.14969	3.22063718	415.582817	4.54112492	415.582817	1.28249387
646.374225	3.22111206	416	4.4990356	416	1.2707413
647.074845	3.22258002	416.074845	4.4880415	416.074845	1.26760834
647.358282	3.22317426	416.14969	4.4745107	416.14969	1.26385171
647.582817	3.22365108	416.374225	4.39631796	416.374225	1.24711536
648.14969	3.22483266	417.074845	4.33998037	417.074845	1.25670725
648.374225	3.22529878	417.358282	4.56292379	417.358282	1.33620555
648.582817	3.22573116	417.374225	4.54988913	417.374225	1.33026876
649.14969	3.22690189	417.582817	4.44154214	417.582817	1.31161761
649.582817	3.22779237	418	4.5612619	418	1.32700598
650	3.22863942	418.074845	4.56421489	418.074845	1.32499629
650.14969	3.22895251	418.14969	4.56459432	418.14969	1.32297691
650.374225	3.22941031	418.374225	4.55974871	418.374225	1.31776349
651.582817	3.23185877	418.582817	4.55304058	418.582817	1.3140751
652.074845	3.23284063	419.14969	4.53514727	419.14969	1.30740788
652.14969	3.23299791	419.358282	4.52910867	419.358282	1.30564983
652.358282	3.23340839	419.374225	4.52865881	419.374225	1.30552391
652.374225	3.23344753	419.582817	4.52282966	419.582817	1.3039829
653.582817	3.23585175	420	4.51133473	420	1.30124655
654.14969	3.23697035	420.074845	4.5092358	420.074845	1.30078081
654.374225	3.23741183	420.14969	4.50710919	420.14969	1.30031764
655	3.2386303	420.374225	4.50047861	420.374225	1.29892738
655.374225	3.23936704	421.358282	4.46092374	421.358282	1.29240245
655.582817	3.23977262	421.374225	4.46010781	421.374225	1.29230942
656.14969	3.24087103	421.582817	4.44928462	421.582817	1.29129442

Final State: j'=5		Final State: j'=3		Final State: j'=2	
E(cm⁻¹)	σ (Å²)	E(cm⁻¹)	σ (Å²)	E(cm⁻¹)	σ (Å²)
656.374225	3.24130456	422	4.43292784	422	1.29130519
657.074845	3.24264442	422.074845	4.43147505	422.074845	1.29161403
657.358282	3.24318685	422.14969	4.43051173	422.14969	1.291984
657.582817	3.24362272	422.358282	4.42992954	422.358282	1.29316341
658.14969	3.24470129	422.374225	4.42997971	422.374225	1.2932542
658.374225	3.24512696	423.358282	4.43296672	423.358282	1.29556409
658.582817	3.24552167	423.374225	4.4328775	423.374225	1.29554712
659.14969	3.24659042	423.582817	4.43111064	423.582817	1.29519522
659.582817	3.24740333	424	4.42440867	424	1.29370131
660	3.24817639	424.074845	4.42265505	424.074845	1.2932928
660.14969	3.24846243	424.14969	4.42067782	424.14969	1.29282467
660.374225	3.24888045	424.374225	4.4129184	424.374225	1.29094468
661.582817	3.25111594	425	4.30258943	425	1.27241996
662.074845	3.252012	425.358282	4.38358017	425.358282	1.31241931
662.14969	3.25215602	425.374225	4.38867206	425.374225	1.31120576
662.358282	3.25253044	425.582817	4.35965277	425.582817	1.30855032
662.374225	3.25256649	426	4.4472006	426	1.31272236
663.582817	3.25476184	426.074845	4.44390033	426.074845	1.31111989
664.14969	3.25577636	426.14969	4.4407763	426.14969	1.30985813
664.374225	3.25618646	426.374225	4.43281638	426.374225	1.30731077
665	3.25729887	427.074845	4.41628694	427.074845	1.30408127
665.374225	3.25797213	427.358282	4.41146868	427.358282	1.3035689
665.582817	3.25834253	427.374225	4.41121891	427.374225	1.30354682
666.374225	3.25974178	427.582817	4.40809634	427.582817	1.3033124
667.074845	3.26096544	428	4.40256889	428	1.3030553
667.358282	3.26146101	428.074845	4.40165385	428.074845	1.30302973
667.582817	3.2618596	428.14969	4.40075649	428.14969	1.30300918
668.374225	3.26323409	428.374225	4.39815722	428.374225	1.30297001
668.582817	3.26359469	428.582817	4.3958411	428.582817	1.30295689
669.14969	3.26456476	429.14969	4.38982549	429.14969	1.3029873
669.582817	3.26531461	429.374225	4.38749491	429.374225	1.30301097
670	3.26602087	429.582817	4.38534211	429.582817	1.30303919
670.374225	3.26666502	430	4.38100957	430	1.30309155
671.582817	3.26870931	430.074845	4.38022525	430.074845	1.30310011
672.074845	3.26952878	430.14969	4.37943788	430.14969	1.30310837
672.358282	3.27000326	430.374225	4.37705201	430.374225	1.30312943
672.374225	3.27003644	431.358282	4.3657906	431.358282	1.30309239
673.582817	3.27204541	431.374225	4.36558895	431.374225	1.30308936
674.14969	3.27297426	431.582817	4.36285543	431.582817	1.30302814
674.374225	3.27335022	432	4.35659028	432	1.30280958
675	3.2743689	432.074845	4.35530117	432.074845	1.30275416
675.374225	3.27498611	432.14969	4.35394271	432.14969	1.30269337
676.374225	3.27660857	432.358282	4.3497272	432.358282	1.30250288
677.074845	3.27773078	432.374225	4.34937486	432.374225	1.30248728
677.358282	3.27818565	433.358282	4.32764124	433.358282	1.304108

Final State: j'=5		Final State: j'=3		Final State: j'=2	
E(cm⁻¹)	σ (Å²)	E(cm⁻¹)	σ (Å²)	E(cm⁻¹)	σ (Å²)
677.582817	3.27855155	433.374225	4.3276493	433.374225	1.30421036
678.374225	3.27981374	433.582817	4.32916039	433.582817	1.30551787
678.582817	3.28014508	434	4.33419269	434	1.30734822
679.14969	3.28103643	434.074845	4.33482771	434.074845	1.30754811
679.582817	3.28172618	434.14969	4.33534368	434.14969	1.30771895
680	3.28237573	434.374225	4.3362305	434.374225	1.30810277
680.374225	3.28296886	435	4.33516224	435	1.30868806
681.582817	3.28485238	435.358282	4.33334356	435.358282	1.3089283
682.074845	3.28560815	435.374225	4.33325181	435.374225	1.30893889
682.358282	3.28604601	435.582817	4.33199177	435.582817	1.30907482
682.374225	3.28607682	436	4.32922688	436	1.30935199
683.582817	3.28793385	436.074845	4.32870676	436.074845	1.30940287
684.14969	3.28879341	436.14969	4.32818138	436.14969	1.30945398
684.374225	3.28914182	436.374225	4.32657665	436.374225	1.30961039
685	3.29008642	437.074845	4.32138603	437.074845	1.31012
685.374225	3.29065953	437.358282	4.31923297	437.358282	1.31033383
685.582817	3.290975	437.374225	4.31912954	437.374225	1.3103492
686.374225	3.29216845	437.582817	4.31751003	437.582817	1.31050488
687.074845	3.29321457	438	4.31427509	438	1.31082841
687.358282	3.29363922	438.074845	4.31368929	438.074845	1.31088686
687.582817	3.29398131	438.14969	4.31310187	438.14969	1.31094529
688.374225	3.29516288	438.374225	4.31133077	438.374225	1.3111223
688.582817	3.29547364	438.582817	4.30967168	438.582817	1.31128719
689.14969	3.29631094	439.14969	4.30508639	439.14969	1.3117402
689.582817	3.29696021	439.358282	4.30336836	439.358282	1.3119091
690	3.29757301	439.374225	4.30324168	439.374225	1.31192317
690.374225	3.2981335	439.582817	4.30149966	439.582817	1.31209301
691.582817	3.29992185	440	4.29797808	440	1.31244421
692.074845	3.3006435	440.074845	4.29734082	440.074845	1.31250895
692.358282	3.30106264	440.14969	4.29670227	440.14969	1.31257434
692.374225	3.30109207	440.374225	4.29478241	440.374225	1.31277544
693.582817	3.3028812	441.358282	4.28653502	441.358282	1.31377182
694.14969	3.30371681	441.374225	4.28640562	441.374225	1.31378634
694.374225	3.3040564	441.582817	4.2847598	441.582817	1.31402905
695	3.30498357	442	4.2816161	442	1.3145306
695.374225	3.30554918	442.074845	4.28107328	442.074845	1.31462301
695.582817	3.30586211	442.14969	4.2805368	442.14969	1.314716
696.374225	3.30705586	442.358282	4.27907145	442.358282	1.31497719
697.074845	3.30811816	442.374225	4.27896124	442.374225	1.31499734
697.358282	3.30855392	443.358282	4.27247464	443.358282	1.31623291
697.582817	3.30890662	443.374225	4.27235557	443.374225	1.31625081
698.374225	3.31014515	443.582817	4.27104446	443.582817	1.31650979
698.582817	3.31047625	444	4.26839985	444	1.31701686
699.14969	3.31138354	444.074845	4.26792561	444.074845	1.31710695
699.582817	3.31210087	444.14969	4.26745153	444.14969	1.31719667

Final State: j'=5		Final State: j'=3		Final State: j'=2	
E(cm⁻¹)	σ (Å²)	E(cm⁻¹)	σ (Å²)	E(cm⁻¹)	σ (Å²)
700	3.31279661	444.374225	4.26602819	444.374225	1.3174644
700.374225	3.31344607	445	4.26204318	445	1.3182005
701.582817	3.31567388	445.358282	4.2597469	445.358282	1.31861667
702.074845	3.31667625	445.374225	4.25964436	445.374225	1.31863519
702.358282	3.31729831	445.582817	4.25830183	445.582817	1.31887614
702.374225	3.31733906	446	4.25560579	446	1.31935649
703.582817	3.32070001	446.074845	4.25512071	446.074845	1.31944241
704.14969	3.32332573	446.14969	4.25463543	446.14969	1.31952825
704.374225	3.32496919	446.374225	4.25317578	446.374225	1.31978572
705	3.3288761	447.074845	4.24860149	447.074845	1.32058861
705.374225	3.33084668	447.358282	4.24674349	447.358282	1.32091412
705.582817	3.32542698	447.374225	4.24663578	447.374225	1.32093369
706.374225	3.32402673	447.582817	4.24526786	447.582817	1.32117127
707.074845	3.33005644	448	4.24252362	448	1.32165231
707.358282	3.31676333	448.074845	4.24203014	448.074845	1.32173888
707.582817	3.31869755	448.14969	4.24153741	448.14969	1.32182558
708.374225	3.32407494	448.374225	4.2400565	448.374225	1.32208491
708.582817	3.32668038	448.582817	4.23868131	448.582817	1.3223273
709.14969	3.33138045	449.14969	4.23494538	449.14969	1.32299016
709.582817	3.32700433	449.358282	4.23357195	449.358282	1.32323534
710	3.32119496	449.374225	4.23347867	449.374225	1.32325573
710.374225	3.32316945	449.582817	4.2320954	449.582817	1.32350085
711.582817	3.32502276	450	4.22935903	450	1.32399732
712.074845	3.33103427	450.074845	4.22886911	450.074845	1.32408672
712.358282	3.33642567	450.14969	4.22837968	450.14969	1.32417644
713.582817	3.32342806	450.374225	4.22691392	450.374225	1.32444631
714.14969	3.32624361	451.358282	4.22054338	451.358282	1.32564409
714.374225	3.31879697	451.374225	4.22044765	451.374225	1.32566408
715	3.32639141	451.582817	4.21910386	451.582817	1.32592068
715.374225	3.32907838	452	4.21644374	452	1.32643765
715.582817	3.33067946	452.074845	4.21596855	452.074845	1.32653085
716.374225	3.34330299	452.14969	4.21549395	452.14969	1.32662412
717.074845	3.32115278	452.358282	4.21417452	452.358282	1.3268846
717.358282	3.32967754	452.374225	4.21407388	452.374225	1.32690457
717.582817	3.32939991	453.358282	4.20791468	453.358282	1.32814413
718.374225	3.32101725	453.374225	4.20781595	453.374225	1.32816376
718.582817	3.32462374	453.582817	4.20652284	453.582817	1.32842918
719.14969	3.32913071	454	4.20394948	454	1.32896065
719.582817	3.33150799	454.074845	4.20348942	454.074845	1.32905611
720	3.33380987	454.14969	4.20302985	454.14969	1.32915183
720.374225	3.33634381	454.374225	4.20165379	454.374225	1.32943923
721.582817	3.34048095	455	4.19783994	455	1.33024314
722.074845	3.3328046	455.358282	4.19566872	455.358282	1.33070563
722.358282	3.33918721	455.374225	4.19557234	455.374225	1.33072617
722.374225	3.3403066	455.582817	4.19431256	455.582817	1.330996

Final State: j'=5		Final State: j'=3		Final State: j'=2	
E(cm⁻¹)	σ (Å²)	E(cm⁻¹)	σ (Å²)	E(cm⁻¹)	σ (Å²)
723.582817	3.33051849	456	4.1918015	456	1.33153707
724.14969	3.33374725	456.074845	4.19135204	456.074845	1.33163434
724.374225	3.33476566	456.14969	4.19090301	456.14969	1.33173168
725	3.33744994	456.374225	4.18955769	456.374225	1.332024
725.374225	3.33921092	457.074845	4.18537853	457.074845	1.33293969
725.582817	3.34034344	457.358282	4.183695	457.358282	1.33331161
726.374225	3.34824313	457.374225	4.183593	457.374225	1.33333211
727.074845	3.34189905	457.582817	4.18236466	457.582817	1.33360695
727.358282	3.33658395	458	4.17989942	458	1.33415712
727.582817	3.33848895	458.074845	4.17945818	458.074845	1.33425595
728.374225	3.33001079	458.14969	4.17901717	458.14969	1.33435499
728.582817	3.32977895	458.374225	4.17769567	458.374225	1.33465224
729.14969	3.33619894	458.582817	4.17647026	458.582817	1.33492895
729.582817	3.33846403	459.14969	4.17315136	459.14969	1.33568357
730	3.34016414	459.358282	4.17193431	459.358282	1.33596217
730.374225	3.3415482	459.374225	4.17184528	459.374225	1.33598346
731.582817	3.34633353	459.582817	4.17062656	459.582817	1.33626267
732.074845	3.3493081	460	4.16820369	460	1.33682269
732.358282	3.35202515	460.074845	4.16777	460.074845	1.33692341
732.374225	3.35222074	460.14969	4.16733654	460.14969	1.33702416
733.582817	3.34204216	460.374225	4.166038	460.374225	1.33732689
734.14969	3.34219712	461.358282	4.16037856	461.358282	1.3386611
734.374225	3.3447135	461.582817	4.1590946	461.582817	1.33896731
735	3.34161443	462	4.15671654	462	1.33953777
735.374225	3.34449202	462.074845	4.15629074	462.074845	1.33964031
735.582817	3.34568036	462.14969	4.15586543	462.14969	1.33974301
736.374225	3.34941347	462.358282	4.15468167	462.358282	1.34002947
737.074845	3.35276781	462.374225	4.15459127	462.374225	1.34005137
737.358282	3.35439551	463.358282	4.14904074	463.358282	1.34141052
737.582817	3.35592716	463.582817	4.14778165	463.582817	1.3417223
738.374225	3.36667439	464	4.14545029	464	1.34230326
738.582817	3.36339317	464.074845	4.14503317	464.074845	1.3424077
739.14969	3.36099158	464.14969	4.14461624	464.14969	1.34251232
739.582817	3.3698154	464.374225	4.1433673	464.374225	1.34282624
740	3.37186208	465	4.13990129	465	1.34370464
740.374225	3.34800813	465.358282	4.13792687	465.358282	1.34420898
741.582817	3.35431002	465.374225	4.13783986	465.374225	1.34423282
742.074845	3.36117421	465.582817	4.13669367	465.582817	1.34452761
742.358282	3.35141594	466	4.13440786	466	1.34511696
742.374225	3.35037577	466.074845	4.13399916	466.074845	1.34522354
743.582817	3.35591944	466.374225	4.1323668	466.374225	1.34565008
744.14969	3.37219894	467.074845	4.1285649	467.074845	1.34665049
744.374225	3.37121774	467.358282	4.12703403	467.358282	1.34705655
745	3.34251458	467.582817	4.12582428	467.582817	1.34737792
745.374225	3.34918164	468	4.12358377	468	1.3479786

Final State: j'=5		Final State: j'=3		Final State: j'=2	
E(cm⁻¹)	σ (Å²)	E(cm⁻¹)	σ (Å²)	E(cm⁻¹)	σ (Å²)
745.582817	3.351966	468.074845	4.12318268	468.074845	1.3480867
746.374225	3.36255521	468.14969	4.12278209	468.14969	1.34819475
747.074845	3.3919053	468.374225	4.12158165	468.374225	1.34851915
747.358282	3.37788267	468.582817	4.12046903	468.582817	1.34882109
747.582817	3.35986024	469.14969	4.11745574	469.14969	1.34964459
748.374225	3.34773689	469.358282	4.11635133	469.358282	1.34994816
748.582817	3.33151948	469.582817	4.11516462	469.582817	1.35027559
749.14969	3.34954537	470	4.11296683	470	1.35088551
749.582817	3.35481802	470.074845	4.11257322	470.074845	1.35099531
750	3.36044876	470.14969	4.11218003	470.14969	1.3511049
750.374225	3.36941696	470.374225	4.11100265	470.374225	1.35143448
751.582817	3.34476414	471.358282	4.10586978	471.358282	1.35288483
752.074845	3.35180624	471.582817	4.1047052	471.582817	1.35321721
752.358282	3.35444991	472	4.10254803	472	1.35383627
752.374225	3.35458709	472.074845	4.10216202	472.074845	1.35394749
753.582817	3.3647123	472.14969	4.10177604	472.14969	1.35405887
754.14969	3.37353386	472.358282	4.100702	472.358282	1.35436954
754.374225	3.38118543	472.374225	4.1006197	472.374225	1.35439366
755	3.37210494	473.358282	4.09558213	473.358282	1.35586519
755.374225	3.35132481	473.582817	4.09443917	473.582817	1.35620258
755.582817	3.35318877	474	4.0923217	474	1.35683085
756.374225	3.36789442	474.074845	4.0919426	474.074845	1.35694367
757.074845	3.3650263	474.14969	4.09156395	474.14969	1.35705658
757.358282	3.36430521	474.374225	4.09042926	474.374225	1.35739588
757.582817	3.36233746	475	4.08727929	475	1.3583443
758.374225	3.36473628	475.358282	4.08548416	475.358282	1.3588891
758.582817	3.37134942	475.374225	4.08540435	475.374225	1.35891339
759.14969	3.39733605	476	4.08228341	476	1.35986867
759.582817	3.33778345	476.074845	4.08191149	476.074845	1.35998306
760	3.35290835	476.14969	4.08153971	476.14969	1.36009763
760.374225	3.36254495	476.374225	4.08042591	476.374225	1.36044175
761.582817	3.36001522	477.074845	4.0769652	477.074845	1.36151898
762.074845	3.36855632	477.358282	4.07557166	477.358282	1.36195621
762.358282	3.37560507	477.582817	4.07447031	477.582817	1.36230317
762.374225	3.37616151	478	4.07242992	478	1.36294935
763.582817	3.40426781	478.074845	4.07206472	478.074845	1.36306547
764.14969	3.3570453	478.14969	4.07169974	478.14969	1.36318162
764.374225	3.36103622	478.374225	4.07060655	478.374225	1.36353059
765	3.34388248	478.582817	4.06959277	478.582817	1.36385522
765.374225	3.35144616	479.14969	4.06684783	479.14969	1.36473981
765.582817	3.36342417	479.358282	4.06584154	479.358282	1.36506606
767.074845	3.3699317	479.582817	4.06476036	479.582817	1.36541794
767.358282	3.3636687	480	4.06275758	480	1.36607295
768.582817	3.3950885	480.074845	4.06239893	480.074845	1.36619073
769.14969	3.36299204	480.14969	4.0620409	480.14969	1.36630842

Final State: j’=5		Final State: j’=3		Final State: j’=2	
E(cm⁻¹)	σ (Å²)	E(cm⁻¹)	σ (Å²)	E(cm⁻¹)	σ (Å²)
770	3.36818141	480.374225	4.06096767	480.374225	1.36666221
770.374225	3.3700472	481.358282	4.05629028	481.358282	1.36821871
772.074845	3.38607329	481.582817	4.05522881	481.582817	1.36857523
772.358282	3.37560476	482	4.05326287	482	1.36923923
773.582817	3.36503321	482.074845	4.05291105	482.074845	1.3693586
774.14969	3.36847218	482.14969	4.05255939	482.14969	1.36947793
774.374225	3.36928967	482.358282	4.05158064	482.358282	1.36981097
775	3.37086635	482.374225	4.05150583	482.374225	1.36983648
777.074845	3.37431254	483.358282	4.04691456	483.358282	1.37141398
777.358282	3.37452973	483.582817	4.04587279	483.582817	1.37177545
778.582817	3.37410039	484	4.04394294	484	1.37244834
779.14969	3.37447314	484.14969	4.04325229	484.14969	1.37269042
780	3.37577766	484.374225	4.04221884	484.374225	1.37305381
782.074845	3.37378611	485	4.03934831	485	1.3740697
782.358282	3.37422998	485.358282	4.03771261	485.358282	1.37465307
782.374225	3.3742506	485.374225	4.03763976	485.374225	1.37467903
783.582817	3.37520102	485.582817	4.03668948	485.582817	1.37501828
784.14969	3.37549077	486	4.03479554	486	1.37570035
785	3.37588624	486.074845	4.03445664	486.074845	1.37582298
787.074845	3.37684721	486.14969	4.034118	486.14969	1.37594558
787.358282	3.37699255	486.374225	4.03310367	486.374225	1.37631462
788.374225	3.37752199	487.074845	4.02995055	487.074845	1.37746671
788.582817	3.37755148	487.358282	4.0286814	487.358282	1.37793497
789.14969	3.37718551	487.582817	4.02767815	487.582817	1.37830617
790	3.3770811	488	4.02582	488	1.37899751
790.374225	3.37721307	488.074845	4.02548713	488.074845	1.37912187
792.074845	3.37782932	488.14969	4.02515496	488.14969	1.3792461
792.358282	3.37792224	488.374225	4.02415926	488.374225	1.37961941
793.582817	3.37830332	488.582817	4.02323635	488.582817	1.37996677
794.14969	3.37847138	489.14969	4.02073747	489.14969	1.38091306
795	3.37871498	489.358282	4.01982138	489.358282	1.3812624
795.374225	3.37881725	489.582817	4.01883736	489.582817	1.38163882
797.074845	3.37922267	490	4.01701489	490	1.38233981
797.358282	3.37927793	490.074845	4.01668865	490.074845	1.38246582
798.582817	3.37950129	490.14969	4.01636279	490.14969	1.38259188
799.14969	3.3796063	490.374225	4.01538636	490.374225	1.38297043
800	3.37976905	491.358282	4.01113298	491.358282	1.38463675
802.074845	3.38017289	491.582817	4.01016842	491.582817	1.38501842
802.358282	3.38022701	492	4.00838188	492	1.38572968
803.582817	3.38045566	492.074845	4.00806229	492.074845	1.38585751
804.14969	3.38055777	492.14969	4.00774277	492.14969	1.38598537
805	3.38070571	492.358282	4.00685388	492.358282	1.38634206
806.374225	3.38092847	492.374225	4.00678605	492.374225	1.38636945
807.074845	3.38103803	493.358282	4.00261845	493.358282	1.38806033
807.358282	3.38108039	493.582817	4.00167373	493.582817	1.3884479

Final State: j’=5		Final State: j’=3		Final State: j’=2	
E(cm⁻¹)	σ (Å²)	E(cm⁻¹)	σ (Å²)	E(cm⁻¹)	σ (Å²)
808.582817	3.38125589	494	3.9999242	494	1.38917001
809.14969	3.3813334	494.074845	3.999611	494.074845	1.38929961
810	3.38144562	494.14969	3.99929808	494.14969	1.38942971
810.374225	3.38149128	494.374225	3.99836122	494.374225	1.38981981
812.074845	3.38170024	495	3.99576243	495	1.39091038
812.358282	3.38173294	495.358282	3.99428262	495.358282	1.39153695
813.582817	3.38186841	495.374225	3.99421699	495.374225	1.39156482
814.14969	3.38192785	495.582817	3.99335826	495.582817	1.39193077
814.374225	3.38194815	496	3.9916469	496	1.3926648
815	3.38201313	496.074845	3.99134059	496.074845	1.39279671
817.074845	3.3822013	496.14969	3.99103469	496.14969	1.39292871
817.358282	3.3822248	496.374225	3.99011865	496.374225	1.39332524
818.582817	3.38232023	497.074845	3.98727595	497.074845	1.39456759
819.14969	3.38236111	497.358282	3.9861328	497.358282	1.39507241
820	3.38241855	497.582817	3.98522976	497.582817	1.39547331
820.374225	3.38243906	498	3.98355906	498	1.39622034
822.074845	3.38253945	498.074845	3.98326038	498.074845	1.39635463
822.358282	3.38255389	498.14969	3.98296187	498.14969	1.39648906
823.582817	3.3826106	498.374225	3.98206794	498.374225	1.39689299
824.14969	3.38263364	498.582817	3.98124005	498.582817	1.39726899
825	3.38266465	499.14969	3.97900127	499.14969	1.39829488
827.074845	3.38272211	499.358282	3.97818202	499.358282	1.39867393
827.358282	3.38272796	499.582817	3.97730267	499.582817	1.39908288
828.582817	3.38274783	500	3.97567553	500	1.39984481
829.14969	3.38275403	500.074845	3.97538546	500.074845	1.39998245
830	3.38275984	500.14969	3.9750952	500.14969	1.4001198
832.074845	3.38275646	500.374225	3.97422584	500.374225	1.40053226
832.358282	3.38275409	501.358282	3.97045159	501.358282	1.40235343
833.582817	3.38273862	501.582817	3.96959864	501.582817	1.40277222
834.14969	3.38272859	502.074845	3.96774139	502.074845	1.40369424
835	3.38271021	502.14969	3.96746043	502.14969	1.40383505
837.074845	3.38264859	502.358282	3.96667906	502.358282	1.40422825
837.358282	3.38263833	502.374225	3.96661935	502.374225	1.40425835
838.582817	3.38258912	503.358282	3.96297638	503.358282	1.4061304
839.14969	3.38256359	503.582817	3.96215569	503.582817	1.40656176
840	3.38252209	504.074845	3.96037132	504.074845	1.4075129
840.374225	3.38249714	504.14969	3.96010159	504.14969	1.40765856
842.074845	3.38240491	504.374225	3.9592959	504.374225	1.40809604
842.358282	3.38238715	505	3.95707404	505	1.40932555
842.374225	3.38238043	505.358282	3.95582076	505.358282	1.41003764
843.582817	3.3823057	505.374225	3.95576644	505.374225	1.41007905
844.14969	3.3822654	505.582817	3.95504199	505.582817	1.41048668
845	3.38220188	506.074845	3.95335565	506.074845	1.41147967
847.074845	3.38203167	506.14969	3.95310162	506.14969	1.41163193
847.358282	3.38200674	506.374225	3.95234428	506.374225	1.41209036

Final State: j'=5		Final State: j'=3		Final State: j'=2	
E(cm⁻¹)	σ (Å²)	E(cm⁻¹)	σ (Å²)	E(cm⁻¹)	σ (Å²)
848.582817	3.38189457	507.074845	3.9500271	507.074845	1.41354162
849.14969	3.38184016	507.358282	3.94911148	507.358282	1.41413853
850	3.38175563	507.582817	3.94839649	507.582817	1.41461599
852.074845	3.38153487	508.074845	3.946864	508.074845	1.41567769
852.358282	3.38150312	508.14969	3.94663561	508.14969	1.41584141
853.582817	3.38136168	508.374225	3.94595806	508.374225	1.41633593
854.14969	3.38129386	508.582817	3.9453398	508.582817	1.41680037
855	3.38118933	509.14969	3.94372396	509.14969	1.41809225
857.074845	3.38092044	509.358282	3.94315614	509.358282	1.41857952
857.358282	3.38088221	509.582817	3.94256464	509.582817	1.41911305
858.582817	3.38071292	510	3.94152753	510	1.42013197
859.14969	3.38063229	510.074845	3.94135139	510.074845	1.42031936
860	3.38050874	510.14969	3.94117845	510.14969	1.4205079
862.074845	3.38019412	510.374225	3.94068083	510.374225	1.42108316
862.358282	3.38014972	511.358282	3.93904113	511.358282	1.42383234
862.374225	3.3801393	511.582817	3.93885504	511.582817	1.42453439
863.582817	3.37995398	512.074845	3.93889084	512.074845	1.42623135
864.14969	3.37986123	512.14969	3.93897216	512.14969	1.42651368
865	3.3797196	512.358282	3.93935458	512.358282	1.42734361
867.074845	3.37936158	513.358282	3.94416772	513.358282	1.42707031
867.358282	3.3793113	513.582817	3.93804508	513.582817	1.42372312
868.582817	3.37909045	514.074845	3.92553883	514.074845	1.41472286
869.14969	3.37898618	514.14969	3.9213736	514.14969	1.41400282
870	3.37882742	514.374225	3.9182361	514.374225	1.41809344
872.074845	3.3784282	515	3.92863868	515	1.42726786
872.358282	3.37837237	515.358282	3.92967144	515.358282	1.42577049
873.582817	3.37812771	515.374225	3.92959214	515.374225	1.42539662
874.14969	3.37801252	515.582817	3.92205142	515.582817	1.41787132
874.374225	3.37795733	516.074845	3.90882035	516.074845	1.42127875
875	3.37783751	516.14969	3.91016566	516.14969	1.42259502
877.074845	3.37739922	516.374225	3.90697256	516.374225	1.42447517
877.358282	3.37733815	517.074845	3.9199288	517.074845	1.43367177
878.582817	3.3770709	517.358282	3.92852255	517.358282	1.4345618
879.14969	3.37694537	517.582817	3.9181532	517.582817	1.42688068
880	3.37675495	518.074845	3.90607898	518.074845	1.41873342
882.074845	3.37627967	518.14969	3.90244625	518.14969	1.4183103
882.358282	3.37621357	518.374225	3.90166611	518.374225	1.42278419
883.582817	3.37592493	518.582817	3.89962353	518.582817	1.42499665
884.14969	3.37578958	519.14969	3.90052251	519.14969	1.42986975
885	3.37558458	519.358282	3.90681828	519.358282	1.43313458
887.074845	3.37507417	519.582817	3.89963854	519.582817	1.42285321
887.358282	3.37500336	520	3.89696897	520	1.43145513
888.582817	3.37469444	520.074845	3.89822173	520.074845	1.43257838
889.14969	3.37454979	520.14969	3.89939637	520.14969	1.43355377
890	3.37433089	520.374225	3.90284207	520.374225	1.43596506

Final State: j'=5		Final State: j'=3		Final State: j'=2	
E(cm⁻¹)	σ (Å²)	E(cm⁻¹)	σ (Å²)	E(cm⁻¹)	σ (Å²)
892.074845	3.3737872	521.358282	3.88883589	521.358282	1.41801838
892.358282	3.3737119	521.582817	3.88196282	521.582817	1.42124045
893.582817	3.37338365	522.074845	3.8838402	522.074845	1.43318428
894.14969	3.37323019	522.14969	3.88667243	522.14969	1.43509513
895	3.37299809	522.358282	3.89665267	522.358282	1.4394476
897.074845	3.37242267	522.374225	3.8976847	522.374225	1.43960479
897.358282	3.37234304	523.358282	3.88038599	523.358282	1.43618347
898.582817	3.37199642	523.582817	3.88256752	523.582817	1.43833936
899.14969	3.37183443	524.074845	3.88688852	524.074845	1.44223416
900	3.37158973	524.14969	3.88765737	524.14969	1.44280235
900.374225	3.37146932	524.374225	3.89044441	524.374225	1.44451211
902.074845	3.37098379	525	3.89736206	525	1.43912091
902.358282	3.37090005	525.358282	3.87148285	525.358282	1.42676764
903.582817	3.37053558	525.374225	3.87104346	525.374225	1.42706389
904.14969	3.3703654	525.582817	3.86399613	525.582817	1.4309521
905	3.37010843	526.074845	3.87300965	526.074845	1.43916083
907.074845	3.36947247	526.14969	3.87655689	526.14969	1.44073634
907.358282	3.36938465	526.374225	3.87685566	526.374225	1.43804306
908.582817	3.36900246	527.074845	3.85818609	527.074845	1.43790823
909.14969	3.36882399	527.358282	3.86211703	527.358282	1.44163379
910	3.36855456	527.582817	3.86394861	527.582817	1.44361782
910.374225	3.36843526	528.074845	3.86673549	528.074845	1.44700064
912.074845	3.36788767	528.14969	3.86711561	528.14969	1.44746581
912.358282	3.3677955	528.374225	3.86829537	528.374225	1.44883937
913.582817	3.36739418	528.582817	3.86953604	528.582817	1.45011982
914.14969	3.36720658	529.14969	3.87496901	529.14969	1.45386131
915	3.36692298	529.582817	3.88412054	529.582817	1.45510947
915.374225	3.36679726	530	3.86244643	530	1.43316204
917.074845	3.36621804	530.074845	3.85751531	530.074845	1.43254596
917.358282	3.36612009	530.14969	3.85505895	530.14969	1.43344543
918.582817	3.3656913	530.374225	3.8434415	530.374225	1.43701171
919.14969	3.36548911	531.582817	3.8324219	531.582817	1.43951241
920	3.36517991	532.074845	3.84347205	532.074845	1.44946085
920.374225	3.365041	532.14969	3.84415209	532.14969	1.45016128
922.074845	3.3643697	532.358282	3.84561498	532.358282	1.45181836
922.358282	3.36424571	532.374225	3.84570746	532.374225	1.4519318
923.582817	3.36358038	533.582817	3.85016201	533.582817	1.45865553
924.14969	3.36369167	534.074845	3.85232939	534.074845	1.46125851
925	3.36369655	534.14969	3.85277738	534.14969	1.46168136
925.374225	3.36332746	534.374225	3.85445122	534.374225	1.46301717
927.074845	3.36288546	535	3.86505463	535	1.46658306
927.358282	3.36262886	535.374225	3.85442498	535.374225	1.44960678
928.582817	3.36342876	535.582817	3.83838603	535.582817	1.44482427
929.14969	3.36221619	536.074845	3.83304767	536.074845	1.45381907
930	3.36296616	536.14969	3.83325457	536.14969	1.45713171

Final State: j’=5		Final State: j’=3		Final State: j’=2	
E(cm⁻¹)	σ (Å²)	E(cm⁻¹)	σ (Å²)	E(cm⁻¹)	σ (Å²)
930.374225	3.36190046	536.374225	3.84945953	536.374225	1.46125074
932.074845	3.36199117	537.074845	3.81632563	537.074845	1.45262185
932.358282	3.36137376	537.358282	3.82344554	537.358282	1.45849317
933.582817	3.360947	537.582817	3.82595266	537.582817	1.46082163
934.14969	3.36049744	538.074845	3.8287285	538.074845	1.46419139
935	3.35986341	538.14969	3.82899847	538.14969	1.46460961
935.374225	3.35999413	538.374225	3.82968278	538.374225	1.46578671
937.074845	3.36098992	538.582817	3.83020288	538.582817	1.46680599
937.358282	3.35989842	539.14969	3.83138411	539.14969	1.46939867
938.582817	3.35892028	539.582817	3.83235246	539.582817	1.4713471
939.14969	3.35856688	540	3.83364728	540	1.47331583
940	3.35790543	540.074845	3.83394754	540.074845	1.47368767
940.374225	3.36017935	540.14969	3.83427774	540.14969	1.47406764
942.074845	3.35824231	540.374225	3.83549621	540.374225	1.47526574
942.358282	3.35789751	541.582817	3.8360211	541.582817	1.45979565
943.582817	3.35711217	542.074845	3.81181115	542.074845	1.46522511
944.14969	3.35679487	542.14969	3.81666584	542.14969	1.46872454
945	3.35625357	542.358282	3.82422458	542.358282	1.4705718
945.374225	3.35594343	542.374225	3.82266486	542.374225	1.47049238
947.074845	3.35725758	543.582817	3.80987575	543.582817	1.47461335
947.358282	3.35666124	544.074845	3.81378331	544.074845	1.47847788
948.582817	3.35532289	544.14969	3.81418143	544.14969	1.47895163
949.14969	3.35501176	544.374225	3.81523493	544.374225	1.48029073
950	3.35456073	545	3.81771517	545	1.48372602
950.374225	3.35435519	545.374225	3.81929154	545.374225	1.48577285
952.074845	3.35609314	545.582817	3.82032437	545.582817	1.48696114
952.358282	3.35425605	546.074845	3.82368274	546.074845	1.49002744
953.582817	3.35380771	546.14969	3.82438104	546.14969	1.49053685
954.14969	3.35317683	546.374225	3.82698312	546.374225	1.49211582
955	3.35263592	547.074845	3.82899213	547.074845	1.48843028
955.374225	3.3524162	547.358282	3.83165081	547.358282	1.48645391
957.074845	3.35122961	547.582817	3.82081578	547.582817	1.48022747
957.358282	3.35158202	548.074845	3.83418197	548.074845	1.49278191
958.582817	3.35075924	548.14969	3.83872423	548.14969	1.4930013
959.14969	3.35328358	548.374225	3.83034164	548.374225	1.47606656
960	3.35153772	548.582817	3.80321713	548.582817	1.46281824
960.374225	3.35082458	549.14969	3.8104666	549.14969	1.48073907
962.074845	3.3502149	549.582817	3.79437661	549.582817	1.47382289
962.358282	3.34987324	550	3.7943303	550	1.48511739
963.582817	3.3503767	550.074845	3.7988619	550.074845	1.48691256
964.14969	3.34979307	550.14969	3.80244225	550.14969	1.48777045
965	3.34873733	550.374225	3.80833653	550.374225	1.48615131
965.374225	3.34830421	551.582817	3.78480332	551.582817	1.48608966
967.074845	3.34924894	552.074845	3.79556675	552.074845	1.49431021
967.358282	3.34933316	552.14969	3.79747344	552.14969	1.4954083

Final State: j'=5		Final State: j'=3		Final State: j'=2	
E(cm⁻¹)	σ (Å²)	E(cm⁻¹)	σ (Å²)	E(cm⁻¹)	σ (Å²)
968.582817	3.34749018	552.358282	3.80390224	552.358282	1.49828252
969.14969	3.34683278	552.374225	3.80448189	552.374225	1.49847597
970	3.35084174	553.582817	3.7668359	553.582817	1.47835693
970.374225	3.34796888	554.074845	3.77933937	554.074845	1.49171249
972.074845	3.34600488	554.14969	3.78077689	554.14969	1.49298138
972.358282	3.3457683	554.374225	3.78492459	554.374225	1.49639211
973.582817	3.34657346	555	3.79923356	555	1.50523602
974.14969	3.34907868	555.374225	3.81677531	555.374225	1.51097493
975	3.34543991	555.582817	3.83376635	555.582817	1.50945735
975.374225	3.34569954	556.074845	3.75671018	556.074845	1.4583902
977.074845	3.34447752	556.14969	3.75184758	556.14969	1.45487027
977.358282	3.34402793	556.374225	3.73954913	556.374225	1.46517427
978.582817	3.34509326	557.074845	3.72843506	557.074845	1.4746136
979.14969	3.34352241	557.358282	3.75270289	557.358282	1.49206559
980	3.34465908	557.582817	3.75901928	557.582817	1.4963047
980.374225	3.34326611	558.074845	3.76823044	558.074845	1.5028641
982.074845	3.34202195	558.14969	3.76955864	558.14969	1.50377254
982.358282	3.34149191	558.374225	3.7738961	558.374225	1.50653219
983.582817	3.34107945	558.582817	3.77893984	558.582817	1.50925203
984.14969	3.34269693	559.14969	3.8037264	559.14969	1.50785682
985	3.34080711	559.582817	3.75954158	559.582817	1.46736123
985.374225	3.34074004	560	3.73740474	560	1.48778649
987.074845	3.33991603	560.074845	3.74041827	560.074845	1.49081092
987.358282	3.33990245	560.14969	3.74291785	560.14969	1.49324075
988.582817	3.34017294	560.374225	3.74846901	560.374225	1.49847665
989.14969	3.33978359	561.582817	3.76345563	561.582817	1.5122834
990	3.33935424	562.074845	3.7694691	562.074845	1.51686399
990.374225	3.33917809	562.14969	3.77061794	562.14969	1.51761897
992.074845	3.33950892	562.358282	3.77443506	562.358282	1.51987532
992.358282	3.33930604	562.374225	3.77477374	562.374225	1.52005823
993.582817	3.33827189	563.582817	3.74145826	563.582817	1.4860301
994.14969	3.33801335	564.074845	3.73500501	564.074845	1.50016185
995	3.33765506	564.14969	3.73250707	564.14969	1.50370665
995.374225	3.33750841	564.374225	3.75355629	564.374225	1.51183469
997.074845	3.33700419	565	3.72884693	565	1.50448026
997.358282	3.33688889	565.374225	3.7512631	565.374225	1.51397855
998.582817	3.33636105	565.582817	3.75519085	565.582817	1.51869879
999.14969	3.33612015	566.074845	3.74880106	566.074845	1.51748577
1000	3.33629213	566.374225	3.76460229	566.374225	1.52614316
1000.37423	3.33584713	567.074845	3.76020723	567.074845	1.5246951
1002.07485	3.33506391	567.358282	3.78634305	567.358282	1.53364325
1002.35828	3.33495499	567.582817	3.77784431	567.582817	1.51199033
1003.58282	3.33448313	568.074845	3.69414389	568.074845	1.48225804
1004.14969	3.33426426	568.14969	3.70079323	568.14969	1.4912905
1005	3.33393773	568.374225	3.71639501	568.374225	1.50716912

Final State: j'=5		Final State: j'=3		Final State: j'=2	
E(cm⁻¹)	σ (Å²)	E(cm⁻¹)	σ (Å²)	E(cm⁻¹)	σ (Å²)
1005.37423	3.33379294	568.582817	3.72644688	568.582817	1.51545756
1007.07485	3.33316413	569.14969	3.75767748	569.14969	1.53199783
1007.35828	3.33307657	569.582817	3.71060001	569.582817	1.49363623
1008.58282	3.3326423	570	3.72662878	570	1.51891103
1009.14969	3.33240179	570.074845	3.72894319	570.074845	1.52103008
1010	3.33206	570.14969	3.73110261	570.14969	1.52290236
1010.37423	3.33191148	570.374225	3.73716718	570.374225	1.52769679
1012.07485	3.33125217	571.582817	3.73805894	571.582817	1.52589122
1012.35828	3.33114315	572.074845	3.77430126	572.074845	1.52734579
1013.58282	3.33067424	572.14969	3.7644814	572.14969	1.5147398
1014.14969	3.33045847	572.358282	3.73175446	572.358282	1.49989876
1015	3.330138	572.374225	3.73151065	572.374225	1.49699106
1015.37423	3.32999579	573.582817	3.70801191	573.582817	1.515508
1017.07485	3.32935448	574.074845	3.72025701	574.074845	1.5324707
1017.35828	3.32924686	574.14969	3.73395214	574.14969	1.52837559
1018.58282	3.32878034	574.374225	3.71173585	574.374225	1.52969365
1019.14969	3.32856409	575	3.72640383	575	1.53614426
1020	3.32824128	575.374225	3.7471833	575.374225	1.54369432
1020.37423	3.32809739	576.074845	3.71835814	576.074845	1.53145376
1022.07485	3.32745101	576.14969	3.72011378	576.14969	1.5326021
1022.35828	3.32734351	576.374225	3.7250292	576.374225	1.53551683
1023.58282	3.3268798	577.074845	3.73258734	577.074845	1.51656372
1024.14969	3.32666551	577.358282	3.68259694	577.358282	1.51185235
1025	3.32634597	577.582817	3.69906534	577.582817	1.52221209
1025.37423	3.32620338	578.074845	3.70426989	578.074845	1.52760167
1027.07485	3.32556273	578.14969	3.70487027	578.14969	1.52831126
1027.35828	3.32545753	578.374225	3.70646039	578.374225	1.53014773
1028.58282	3.32499552	578.582817	3.7076858	578.582817	1.53153736
1029.14969	3.32478252	579.14969	3.71018318	579.14969	1.53427181
1030	3.3244648	579.582817	3.71154263	579.582817	1.53566192
1030.37423	3.32432286	580	3.71325478	580	1.53700619
1032.07485	3.32368568	580.074845	3.71377223	580.074845	1.53730963
1032.35828	3.32358118	580.14969	3.71442043	580.14969	1.53764578
1033.58282	3.32312198	580.374225	3.71779779	580.374225	1.53889921
1034.14969	3.3229104	581.582817	3.69402826	581.582817	1.52984502
1035	3.32259502	582.074845	3.6864517	582.074845	1.5287727
1035.37423	3.32245393	582.14969	3.68746413	582.14969	1.53006793
1037.07485	3.32182147	582.358282	3.6897282	582.358282	1.53256127
1037.35828	3.32171788	582.374225	3.68985788	582.374225	1.53270268
1038.58282	3.32126203	583.582817	3.69253751	583.582817	1.53792358
1039.14969	3.32105206	584.14969	3.69220934	584.14969	1.53934204
1040	3.32073918	584.374225	3.69201519	584.374225	1.53985566
1040.37423	3.32059915	585	3.69137904	585	1.54113614
1042.07485	3.31997158	585.374225	3.69084605	585.374225	1.54168893
1042.35828	3.31986892	585.582817	3.69039328	585.582817	1.54185939

Final State: j’=5		Final State: j’=3		Final State: j’=2	
E(cm⁻¹)	σ (Å²)	E(cm⁻¹)	σ (Å²)	E(cm⁻¹)	σ (Å²)
1043.58282	3.31941658	586.14969	3.68811056	586.14969	1.54181447
1044.14969	3.3192083	586.374225	3.68694956	586.374225	1.54183028
1045	3.31889806	587.074845	3.68427843	587.074845	1.54282255
1045.37423	3.31875907	587.358282	3.68361264	587.358282	1.54343931
1047.07485	3.31813674	587.582817	3.6831828	587.582817	1.54394821
1047.35828	3.31803505	588.14969	3.68242204	588.14969	1.54526133
1048.58282	3.31758646	588.374225	3.68230453	588.374225	1.54580064
1049.14969	3.31737999	588.582817	3.68238791	588.582817	1.54632716
1050	3.31707257	589.14969	3.68646657	589.14969	1.5468279
1050.37423	3.31693469	589.582817	3.67263784	589.582817	1.54391563
1052.07485	3.31631789	590	3.66972013	590	1.54542552
1052.35828	3.31621722	590.14969	3.67128011	590.14969	1.54662401
1053.58282	3.3157726	590.374225	3.67200987	590.374225	1.54753425
1054.14969	3.315568	591.582817	3.67040285	591.582817	1.55001217
1055	3.31526355	592.074845	3.66921228	592.074845	1.55083745
1055.37423	3.31512682	592.14969	3.66902435	592.14969	1.55096179
1057.07485	3.31451581	592.358282	3.66849651	592.358282	1.55130821
1057.35828	3.31441622	592.374225	3.66845562	592.374225	1.55133462
1058.58282	3.3139757	593.582817	3.66533365	593.582817	1.55334194
1059.14969	3.31377307	594.14969	3.66386401	594.14969	1.55428269
1060	3.31347167	594.374225	3.66328313	594.374225	1.55465448
1060.37423	3.31333615	595	3.66167177	595	1.55568657
1062.07485	3.31273115	595.374225	3.66071521	595.374225	1.55629907
1062.35828	3.31263263	595.582817	3.66018549	595.582817	1.55663789
1063.58282	3.31219638	596.14969	3.6587537	596.14969	1.55753552
1064.14969	3.31199579	596.374225	3.65817892	596.374225	1.55786909
1065	3.31169753	597.074845	3.656014	597.074845	1.55862135
1065.37423	3.31156326	597.358282	3.6547229	597.358282	1.55875496
1067.07485	3.31096439	597.582817	3.65363831	597.582817	1.5589228
1067.35828	3.31086704	598.14969	3.6516401	598.14969	1.55985806
1068.58282	3.31043514	598.374225	3.65106309	598.374225	1.56030557
1069.14969	3.31023664	598.582817	3.65055657	598.582817	1.56071635
1070	3.3099416	599.14969	3.649187	599.14969	1.56177805
1070.37423	3.30980862	599.582817	3.6481103	599.582817	1.56254534
1072.07485	3.30921604	600	3.64705046	600	1.56326229
1072.35828	3.30911985	600.14969	3.64666509	600.14969	1.56351566
1073.58282	3.30869241	600.374225	3.64608446	600.374225	1.56389312
1074.14969	3.30849601	601.582817	3.6429147	601.582817	1.56588751
1075	3.30820428	602.074845	3.64161252	602.074845	1.56668808
1075.37423	3.3080726	602.14969	3.64141341	602.14969	1.56680928
1077.07485	3.30748644	602.358282	3.64086023	602.358282	1.56714692
1077.35828	3.30739142	602.374225	3.64081722	602.374225	1.56717257
1078.58282	3.30696853	603.582817	3.63759346	603.582817	1.56910993
1079.14969	3.30677428	604.14969	3.63606732	604.14969	1.57000528
1080	3.30648587	604.374225	3.63545863	604.374225	1.57035701

Final State: j'=5		Final State: j'=3		Final State: j'=2	
E(cm^{-1})	σ (Å^2)	E(cm^{-1})	σ (Å^2)	E(cm^{-1})	σ (Å^2)
1080.37423	3.3063555	605	3.63374708	605	1.57132855
1082.07485	3.30577838	605.374225	3.63271143	605.374225	1.57190482
1082.35828	3.30568198	605.582817	3.63213192	605.582817	1.57222556
1083.58282	3.30526366	606.14969	3.63055387	606.14969	1.57309886
1084.14969	3.30507159	606.374225	3.62992978	606.374225	1.5734465
1085	3.30478655	607.074845	3.62799593	607.074845	1.57454033
1085.37423	3.30465751	607.358282	3.62721976	607.358282	1.57498598
1087.07485	3.30408701	607.582817	3.62660671	607.582817	1.57534024
1087.35828	3.3039917	608.14969	3.62506947	608.14969	1.57623709
1088.58282	3.30357799	608.374225	3.62446355	608.374225	1.57659294
1089.14969	3.30338808	608.582817	3.62390188	608.582817	1.57692403
1090	3.30310641	609.14969	3.62238041	609.14969	1.5778239
1090.37423	3.30297871	609.582817	3.62122163	609.582817	1.57851213
1092.07485	3.30241485	610	3.62010846	610	1.57917348
1092.35828	3.30232064	610.14969	3.61970832	610.14969	1.57941078
1093.58282	3.30191152	610.374225	3.61910984	610.374225	1.57976675
1094.14969	3.30172379	611.582817	3.61589403	611.582817	1.58167783
1095	3.30144549	612.074845	3.6145876	612.074845	1.58245379
1095.37423	3.30131912	612.14969	3.61438779	612.14969	1.58257163
1097.07485	3.30076189	612.358282	3.61383472	612.358282	1.58290006
1097.35828	3.30066876	613.582817	3.61058296	613.582817	1.58482304
1098.58282	3.30026422	614.14969	3.60907861	614.14969	1.58571092
1099.14969	3.30007866	614.374225	3.60848296	614.374225	1.58606228
1100	3.29980368	615	3.60682423	615	1.58704009
1100.37423	3.29967865	615.374225	3.60583101	615.374225	1.58762394
1102.07485	3.29912799	615.582817	3.60527829	615.582817	1.58794938
1102.35828	3.29903593	616.14969	3.60377713	616.14969	1.58883339
1103.58282	3.29863593	616.374225	3.60318281	616.374225	1.58918294
1104.14969	3.29845251	617.074845	3.60133234	617.074845	1.59027424
1105	3.29818088	617.358282	3.60058396	617.358282	1.59071551
1105.37423	3.29805713	617.582817	3.5999904	617.582817	1.59106486
1107.07485	3.29751297	618.14969	3.59849646	618.14969	1.59194681
1107.35828	3.29742198	618.374225	3.59790516	618.374225	1.59229572
1108.58282	3.29702644	618.582817	3.59735645	618.582817	1.59262013
1109.14969	3.29684515	619.14969	3.59586655	619.14969	1.59350105
1110	3.29657677	619.582817	3.59472997	619.582817	1.59417393
1110.37423	3.29645432	620	3.59363772	620	1.59482178
1112.07485	3.29591654	620.14969	3.59324437	620.14969	1.59505402
1112.35828	3.2958266	620.374225	3.59265632	620.374225	1.59540224
1113.58282	3.29543546	621.582817	3.58949773	621.582817	1.59727555
1114.14969	3.29525623	622.074845	3.58821605	622.074845	1.59803738
1115	3.29499108	622.14969	3.58801945	622.14969	1.59815296
1115.37423	3.29486988	622.358282	3.58747756	622.358282	1.59847588
1117.07485	3.29433837	622.374225	3.58743444	622.374225	1.59850033
1117.35828	3.29424945	623.582817	3.5842906	623.582817	1.60036773

Final State: j'=5		Final State: j'=3		Final State: j'=2	
E(cm⁻¹)	σ (Å²)	E(cm⁻¹)	σ (Å²)	E(cm⁻¹)	σ (Å²)
1118.58282	3.29386261	624.14969	3.58281885	624.14969	1.60124214
1119.14969	3.29368541	624.374225	3.58223632	624.374225	1.60158848
1120	3.29342338	625	3.58061629	625	1.60255269
1120.37423	3.29330341	625.374225	3.57964561	625.374225	1.60312834
1122.07485	3.29277803	625.582817	3.57910589	625.582817	1.60344959
1122.35828	3.29269009	626.14969	3.57764025	626.14969	1.60432112
1123.58282	3.29230742	626.374225	3.57706032	626.374225	1.60466596
1124.14969	3.2921322	627.074845	3.57525402	627.074845	1.60574201
1125	3.29187324	627.358282	3.57452334	627.358282	1.60617681
1125.37423	3.29175444	627.582817	3.57394291	627.582817	1.60652094
1127.07485	3.291235	628.14969	3.57248343	628.14969	1.6073897
1127.35828	3.29114804	628.374225	3.57190587	628.374225	1.60773355
1128.58282	3.29076944	628.582817	3.57136956	628.582817	1.60805286
1129.14969	3.29059613	629.14969	3.56991328	629.14969	1.60892021
1130	3.29034013	629.582817	3.56880182	629.582817	1.60958235
1130.37423	3.29022248	630	3.56773425	630	1.61021992
1132.07485	3.2897088	630.14969	3.56734863	630.14969	1.6104483
1132.35828	3.28962278	630.374225	3.56677352	630.374225	1.61079106
1133.58282	3.28924809	631.582817	3.56368285	631.582817	1.61263397
1134.14969	3.28907663	632.074845	3.56242911	632.074845	1.61338341
1135	3.2888235	632.14969	3.56223594	632.14969	1.61349709
1135.37423	3.28870696	632.358282	3.56170625	632.358282	1.61381475
1137.07485	3.28819885	632.374225	3.56166331	632.374225	1.61383876
1137.35828	3.28811372	633.582817	3.5585859	633.582817	1.61567569
1138.58282	3.28774281	634.14969	3.55714521	634.14969	1.61653609
1139.14969	3.28757314	634.374225	3.55657501	634.374225	1.61687667
1140	3.28732276	635	3.55498987	635	1.61782535
1140.37423	3.28720728	635.374225	3.55403901	635.374225	1.61839183
1142.07485	3.28670452	635.582817	3.55351067	635.582817	1.61870761
1142.35828	3.28662027	636.14969	3.55207605	636.14969	1.61956516
1143.58282	3.28625298	636.374225	3.55150831	636.374225	1.61990457
1144.14969	3.28608503	637.074845	3.54974104	637.074845	1.62096315
1145	3.28583731	637.358282	3.54902577	637.358282	1.62139098
1145.37423	3.28572286	637.582817	3.5484568	637.582817	1.62172948
1147.07485	3.28522526	638.14969	3.54702816	638.14969	1.62258414
1147.35828	3.28514185	638.374225	3.54646278	638.374225	1.62292247
1148.58282	3.28477807	638.582817	3.54593774	638.582817	1.62323667
1149.14969	3.28461178	639.14969	3.54451206	639.14969	1.62408992
1150	3.28436664	639.582817	3.54342386	639.582817	1.62474135
1150.37423	3.28425318	640	3.54237955	640	1.62536855
1152.07485	3.28376061	640.14969	3.54200114	640.14969	1.62559318
1152.35828	3.28367802	640.374225	3.54143804	640.374225	1.62593036
1153.58282	3.28331768	641.582817	3.53841169	641.582817	1.62774314
1154.14969	3.28315303	642.074845	3.53718485	642.074845	1.62848036
1155	3.28291045	642.14969	3.53699482	642.14969	1.62859212

Final State: j'=5		Final State: j'=3		Final State: j'=2	
E(cm⁻¹)	σ (Å²)	E(cm⁻¹)	σ (Å²)	E(cm⁻¹)	σ (Å²)
1155.37423	3.28279798	642.358282	3.53647695	642.358282	1.62890458
1157.07485	3.28231047	642.374225	3.536434	642.374225	1.62892821
1157.35828	3.28222872	643.582817	3.53342009	643.582817	1.63073488
1158.58282	3.28187194	644.14969	3.53200894	644.14969	1.6315811
1159.14969	3.28170901	644.374225	3.53145044	644.374225	1.63191599
1160	3.28146917	645	3.52989866	645	1.63284904
1160.37423	3.2813578	645.374225	3.52896624	645.374225	1.63340614
1162.07485	3.28087605	645.582817	3.52844879	645.582817	1.6337166
1162.35828	3.28079532	646.14969	3.52704335	646.14969	1.63455997
1163.58282	3.28044307	646.374225	3.52648707	646.374225	1.63489372
1164.14969	3.28028674	647.074845	3.52475663	647.074845	1.63593479
1165	3.28004671	647.358282	3.52405589	647.358282	1.63635549
1165.37423	3.27993716	647.582817	3.52349745	647.582817	1.63668858
1167.07485	3.27946715	648.14969	3.52209781	648.14969	1.63752866
1167.35828	3.27938913	648.374225	3.52154382	648.374225	1.63786158
1168.58282	3.27905567	648.582817	3.52102934	648.582817	1.63817049
1169.14969	3.27891885	649.14969	3.51963239	649.14969	1.63900954
1170	3.27884326	649.582817	3.51856614	649.582817	1.63965008
1170.37423	3.27866084	650	3.51754389	650	1.64026684
1172.07485	3.27828211	650.14969	3.51717202	650.14969	1.64048771
1172.35828	3.27784745	650.374225	3.5166202	650.374225	1.64081925
1173.58282	3.27763678	651.582817	3.51365448	651.582817	1.64260175
1174.14969	3.2774808	652.074845	3.51245325	652.074845	1.64332663
1175	3.27705783	652.14969	3.5122659	652.14969	1.64343655
1175.37423	3.2771623	652.358282	3.51175942	652.358282	1.64374381
1177.07485	3.2766317	652.374225	3.51171632	652.374225	1.64376702
1177.35828	3.27659521	653.582817	3.50876229	653.582817	1.64554347
1178.58282	3.27613496	654.14969	3.50737914	654.14969	1.64637546
1179.14969	3.27596945	654.374225	3.50683178	654.374225	1.64670477
1180	3.27573614	655	3.50531187	655	1.64762213
1180.37423	3.27564921	655.374225	3.50439671	655.374225	1.64816989
1182.07485	3.27507911	655.582817	3.5038894	655.582817	1.6484752
1182.35828	3.27509033	656.14969	3.50251175	656.14969	1.64930436
1183.58282	3.27466604	656.374225	3.50196646	656.374225	1.64963253
1184.14969	3.27455919	657.074845	3.50027131	657.074845	1.6506561
1185	3.27435601	657.358282	3.4995843	657.358282	1.65106961
1185.37423	3.27426357	657.582817	3.49903563	657.582817	1.65139697
1187.07485	3.27375356	658.14969	3.49766331	658.14969	1.65222332
1187.35828	3.27376674	658.374225	3.49712019	658.374225	1.65255038
1188.58282	3.27329051	658.582817	3.49661582	658.582817	1.65285422
1189.14969	3.27316347	659.14969	3.49524619	659.14969	1.65367907
1190	3.27295054	659.582817	3.49420072	659.582817	1.65430882
1190.37423	3.27285193	660	3.49319964	660	1.6549155
1192.07485	3.27237429	660.14969	3.4928338	660.14969	1.65513244
1192.35828	3.27226622	660.374225	3.49229273	660.374225	1.65545851

Final State: j'=5		Final State: j'=3		Final State: j'=2	
E(cm⁻¹)	σ (Å²)	E(cm⁻¹)	σ (Å²)	E(cm⁻¹)	σ (Å²)
1193.58282	3.2718366	661.582817	3.48938461	661.582817	1.6572109
1194.14969	3.27175947	662.074845	3.48820801	662.074845	1.6579236
1195	3.27155038	662.14969	3.48802295	662.14969	1.65803163
1195.37423	3.27144971	662.358282	3.48752763	662.358282	1.65833373
1197.07485	3.27102244	662.374225	3.48748402	662.374225	1.65835655
1197.35828	3.27095627	663.582817	3.48458712	663.582817	1.66010314
1198.58282	3.27060038	664.14969	3.4832363	664.14969	1.66092137
1199.14969	3.27036591	664.374225	3.48269378	664.374225	1.66124493
1200	3.27015739	665	3.48120454	665	1.6621469
1200.37423	3.27007101	665.374225	3.48030555	665.374225	1.66268544
1202.07485	3.26966641	665.582817	3.479808	665.582817	1.66298559
1202.35828	3.26960194	666.374225	3.4779219	666.374225	1.66412356
1203.58282	3.26945546	667.074845	3.47626062	667.074845	1.66512993
1204.14969	3.2692448	667.358282	3.47558682	667.358282	1.66553659
1205	3.26891034	667.582817	3.47504709	667.582817	1.66585839
1205.37423	3.26892865	668.374225	3.47316824	668.374225	1.6669925
1207.07485	3.26860574	668.582817	3.47267351	668.582817	1.66729117
1207.35828	3.26838289	669.14969	3.47133627	669.14969	1.66810255
1208.58282	3.26780709	669.582817	3.47030433	669.582817	1.6687216
1209.14969	3.26780805	670	3.46932383	670	1.66931783
1210	3.26715487	670.374225	3.46843255	670.374225	1.66985192
1210.37423	3.26717045	671.582817	3.46557954	671.582817	1.67157521
1212.07485	3.2670294	672.074845	3.46442665	672.074845	1.67227613
1212.35828	3.267078	672.358282	3.46375921	672.358282	1.67267898
1213.58282	3.26619337	672.374225	3.46371482	672.374225	1.67270156
1214.14969	3.26618194	673.582817	3.46087245	673.582817	1.67441967
1215	3.26603814	674.14969	3.45954864	674.14969	1.67522403
1215.37423	3.26600174	674.374225	3.45901467	674.374225	1.67554227
1217.07485	3.26532747	675	3.45755496	675	1.67642942
1217.35828	3.2652785	675.374225	3.45667121	675.374225	1.67695913
1218.58282	3.2650233	676.374225	3.45433213	676.374225	1.67837364
1219.14969	3.2649318	677.074845	3.45270358	677.074845	1.67936352
1220	3.26438483	677.358282	3.45204227	677.358282	1.67976328
1220.37423	3.26417988	677.582817	3.45151087	677.582817	1.68008003
1222.07485	3.26412664	678.374225	3.44966697	678.374225	1.68119565
1222.35828	3.26442369	678.582817	3.44918138	678.582817	1.6814897
1223.58282	3.26362374	679.14969	3.44787074	679.14969	1.68228773
1224.14969	3.264091	679.582817	3.44685605	679.582817	1.6828968
1225	3.26306711	680	3.44589549	680	1.68348342
1225.37423	3.2630817	680.374225	3.44501885	680.374225	1.68400895
1227.07485	3.26262165	681.582817	3.44221828	681.582817	1.68570476
1227.35828	3.26260975	682.074845	3.44108853	682.074845	1.68639442
1228.58282	3.26226632	682.358282	3.44043332	682.358282	1.68679122
1229.14969	3.26177574	682.374225	3.44038781	682.374225	1.68681344
1230	3.26157799	683.582817	3.43759736	683.582817	1.68850412

Final State: j’=5		Final State: j’=3		Final State: j’=2	
E(cm⁻¹)	σ (Å²)	E(cm⁻¹)	σ (Å²)	E(cm⁻¹)	σ (Å²)
1230.37423	3.26146691	684.14969	3.43629964	684.14969	1.68929619
1232.07485	3.26166998	684.374225	3.43577338	684.374225	1.68960947
1232.35828	3.26112724	685	3.43434221	685	1.69048281
1233.58282	3.26101609	685.374225	3.4334724	685.374225	1.69100441
1234.14969	3.26070219	685.582817	3.43299294	685.582817	1.69129513
1235	3.26048068	686.374225	3.43117553	686.374225	1.69239733
1235.37423	3.26037792	687.074845	3.42957821	687.074845	1.69337237
1237.07485	3.2599034	687.358282	3.42892877	687.358282	1.69376644
1237.35828	3.25980431	687.582817	3.42840488	687.582817	1.69407834
1238.58282	3.25946451	688.374225	3.42659373	688.374225	1.69517761
1239.14969	3.25932431	688.582817	3.4261168	688.582817	1.69546716
1240	3.25908895	689.14969	3.42483147	689.14969	1.69625385
1240.37423	3.25897844	689.582817	3.42383266	689.582817	1.69685422
1242.07485	3.25850151	690	3.42289107	690	1.69743259
1242.35828	3.25841953	690.374225	3.42202763	690.374225	1.69795079
1243.58282	3.25806259	691.582817	3.41927576	691.582817	1.69962357
1244.14969	3.2579075	692.074845	3.41816766	692.074845	1.70030415
1245	3.25766397	692.358282	3.41752368	692.358282	1.70069596
1245.37423	3.25754786	692.374225	3.41747677	692.374225	1.70071783
1247.07485	3.25708375	693.582817	3.41473367	693.582817	1.7023875
1247.35828	3.25700184	694.14969	3.41345986	694.14969	1.7031703
1248.58282	3.25664454	694.374225	3.41294016	694.374225	1.70348008
1249.14969	3.25648773	695	3.41153473	695	1.70434384
1250	3.25624411	695.374225	3.41067687	695.374225	1.70486008
1250.37423	3.25613121	695.582817	3.4102051	695.582817	1.7051479
1252.07485	3.25564813	696.374225	3.40841653	696.374225	1.70623989
1252.35828	3.25556638	697.074845	3.40684641	697.074845	1.70720696
1253.58282	3.2552052	697.358282	3.40620682	697.358282	1.70759828
1254.14969	3.25504786	697.582817	3.40568839	697.582817	1.70790807
1255	3.25480699	698.374225	3.40390372	698.374225	1.70900166
1255.37423	3.25469404	698.582817	3.40343355	698.582817	1.70929023
1257.07485	3.25420891	699.14969	3.40216814	699.14969	1.71007529
1257.35828	3.25412688	699.582817	3.40118012	699.582817	1.71067583
1258.58282	3.25376625	700	3.40025275	700	1.71125555
1259.14969	3.25360736	700.374225	3.39939718	700.374225	1.71177651
1260	3.25336009	701.582817	3.39667318	701.582817	1.71347186
1260.37423	3.2532454	702.074845	3.39557588	702.074845	1.71417138
1262.07485	3.2527552	702.358282	3.39493584	702.358282	1.71457836
1262.35828	3.25267239	702.374225	3.39488679	702.374225	1.7146012
1263.58282	3.25230882	703.582817	3.39215343	703.582817	1.71641688
1264.14969	3.25214874	704.14969	3.39090258	704.14969	1.71741641
1265	3.25189956	704.374225	3.39042205	704.374225	1.71792068
1265.37423	3.25178395	705	3.39058291	705	1.72069163
1267.07485	3.2512896	705.374225	3.39001797	705.374225	1.72166989
1267.35828	3.25120606	705.582817	3.39066908	705.582817	1.72233897

Final State: j’=5		Final State: j’=3		Final State: j’=2	
E(cm⁻¹)	σ (Å²)	E(cm⁻¹)	σ (Å²)	E(cm⁻¹)	σ (Å²)
1268.58282	3.25083875	706.374225	3.38658139	706.374225	1.72064606
1269.14969	3.25067691	707.074845	3.38733428	707.074845	1.72457881
1270	3.25042504	707.358282	3.38630701	707.358282	1.72270155
1270.37423	3.25030818	707.582817	3.38452063	707.582817	1.72202083
1272.07485	3.24980859	708.374225	3.38186458	708.374225	1.72292349
1272.35828	3.24972417	708.582817	3.38135351	708.582817	1.72344725
1273.58282	3.24935889	709.14969	3.38137056	709.14969	1.72572687
1274.14969	3.24918947	709.582817	3.38234717	709.582817	1.72775129
1275	3.24893493	710	3.38026214	710	1.72577697
1275.37423	3.24881683	710.374225	3.37909103	710.374225	1.72700774
1277.07485	3.24831183	711.582817	3.37515736	711.582817	1.72711476
1277.35828	3.24822648	712.074845	3.37403516	712.074845	1.72833122
1278.58282	3.24785711	712.358282	3.37411773	712.358282	1.72981895
1279.14969	3.24768577	713.582817	3.37185271	713.582817	1.73005956
1280	3.24742832	714.14969	3.37249471	714.14969	1.73334882
1280.37423	3.24730887	714.374225	3.37034665	714.374225	1.73070588
1282.07485	3.24679799	715	3.36771082	715	1.73133114
1282.35828	3.24671163	715.374225	3.36676674	715.374225	1.73198981
1283.58282	3.24633791	715.582817	3.36627889	715.582817	1.73239771
1285	3.245904	716.374225	3.36537167	716.374225	1.73560582
1285.37423	3.24578311	717.074845	3.36552377	717.074845	1.73509847
1287.07485	3.24526603	717.358282	3.36323756	717.358282	1.73546564
1287.35828	3.24517863	717.582817	3.36282947	717.582817	1.7356939
1288.58282	3.2448003	718.374225	3.36137083	718.374225	1.73562451
1290	3.244361	718.582817	3.36031411	718.582817	1.73573409
1290.37423	3.2442386	719.14969	3.35875798	719.14969	1.73660885
1292.07485	3.24371497	719.582817	3.35770221	719.582817	1.73730893
1292.35828	3.24362646	720	3.35675829	720	1.73802201
1293.58282	3.24324328	720.374225	3.35590831	720.374225	1.73874537
1295	3.2427983	721.582817	3.3575884	721.582817	1.74417503
1295.37423	3.24267432	722.074845	3.35434613	722.074845	1.74177777
1297.07485	3.2421438	722.358282	3.35248323	722.358282	1.7425926
1297.35828	3.24205411	722.374225	3.35250946	722.374225	1.74288621
1298.58282	3.24166587	723.582817	3.34936976	723.582817	1.74216247
1300	3.24121494	724.14969	3.34800096	724.14969	1.74305097
1300.37423	3.2410893	724.374225	3.34746165	724.374225	1.74339967
1302.07485	3.24055162	725	3.34606512	725	1.74438603
1302.35828	3.24046071	725.374225	3.3452154	725.374225	1.74500867
1303.58282	3.24006717	725.582817	3.34475923	725.582817	1.74537969
1305	3.23961004	726.374225	3.34325816	726.374225	1.74741094
1305.37423	3.23948271	727.074845	3.34693254	727.074845	1.75085444
1307.07485	3.23893752	727.358282	3.34267938	727.358282	1.74877922
1307.35828	3.23884536	727.582817	3.34129279	727.582817	1.74841429
1308.58282	3.23844632	728.374225	3.34221389	728.374225	1.75095945
1310	3.23798276	728.582817	3.33919086	728.582817	1.74843843

Final State: j’=5		Final State: j’=3		Final State: j’=2	
E(cm⁻¹)	σ (Å²)	E(cm⁻¹)	σ (Å²)	E(cm⁻¹)	σ (Å²)
1310.37423	3.23785364	729.14969	3.33745646	729.14969	1.74932673
1312.07485	3.23730073	729.582817	3.33641525	729.582817	1.75000672
1312.35828	3.23720725	730	3.33549374	730	1.7506438
1313.58282	3.2368025	730.374225	3.33464232	730.374225	1.75121187
1315	3.23633229	731.582817	3.33201164	731.582817	1.75312872
1315.37423	3.23620133	732.074845	3.33100231	732.074845	1.75406372
1317.07485	3.23564042	732.358282	3.33046363	732.358282	1.7547659
1317.35828	3.23554558	732.374225	3.33041445	732.374225	1.75481249
1318.58282	3.23513496	733.582817	3.32888124	733.582817	1.75587245
1320	3.23465792	734.14969	3.32850391	734.14969	1.75673073
1320.37423	3.23452505	734.374225	3.32974742	734.374225	1.75843335
1322.07485	3.23395591	735	3.32509075	735	1.75683396
1322.35828	3.23385967	735.374225	3.32415025	735.374225	1.75748362
1323.58282	3.23344301	735.582817	3.32366962	735.582817	1.75782805
1325	3.23295894	736.374225	3.32190426	736.374225	1.75910473
1325.37423	3.23282413	737.074845	3.32039316	737.074845	1.76027747
1327.07485	3.23224654	737.358282	3.31977796	737.358282	1.76079526
1327.35828	3.2321489	737.582817	3.31927294	737.582817	1.76124541
1328.58282	3.23172605	738.374225	3.31857994	738.374225	1.76469433
1330	3.23123475	738.582817	3.31845863	738.582817	1.76478104
1330.37423	3.23109794	739.14969	3.31855979	739.14969	1.76610275
1332.07485	3.23051173	739.582817	3.31644007	739.582817	1.76639112
1332.35828	3.23041262	740	3.32102595	740	1.77082273
1333.58282	3.22998345	740.374225	3.31829784	740.374225	1.76810052
1335	3.2294848	741.582817	3.31195528	741.582817	1.76675141
1335.37423	3.22934595	742.074845	3.3139963	742.074845	1.77192868
1337.07485	3.22875094	742.358282	3.31383663	742.358282	1.77075487
1337.35828	3.22865034	742.374225	3.31367634	742.374225	1.77067035
1338.58282	3.22821473	743.582817	3.30743646	743.582817	1.76876785
1340	3.22770857	744.14969	3.30735998	744.14969	1.77247554
1340.37423	3.22756766	744.374225	3.31022025	744.374225	1.77630351
1342.07485	3.2269637	745	3.30671666	745	1.77084299
1342.35828	3.22686158	745.374225	3.30445167	745.374225	1.77056349
1343.58282	3.22641942	745.582817	3.3037454	745.582817	1.77087803
1345	3.22590567	746.374225	3.30172051	746.374225	1.77273442
1345.37423	3.22576264	747.074845	3.30212764	747.074845	1.77874489
1347.07485	3.2251496	747.358282	3.31087855	747.358282	1.7852401
1347.35828	3.22504595	747.582817	3.3073468	747.582817	1.78207365
1348.58282	3.22459716	748.374225	3.30144032	748.374225	1.77493912
1350	3.2240757	748.582817	3.29976244	748.582817	1.7738071
1350.37423	3.22393053	749.14969	3.29623695	749.14969	1.7745579
1352.07485	3.2233083	749.582817	3.29514847	749.582817	1.77553465
1352.35828	3.22320309	750	3.29424041	750	1.77665264
1353.58282	3.2227476	750.374225	3.29363777	750.374225	1.77841185
1355	3.22221836	751.582817	3.29232033	751.582817	1.77759785

Final State: j’=5		Final State: j’=3		Final State: j’=2	
E(cm⁻¹)	σ (Å²)	E(cm⁻¹)	σ (Å²)	E(cm⁻¹)	σ (Å²)
1355.37423	3.22207102	752.074845	3.29056444	752.074845	1.77829735
1357.07485	3.22143954	752.358282	3.28981021	752.358282	1.77878973
1357.35828	3.22133277	752.374225	3.28974099	752.374225	1.77881826
1358.58282	3.2208705	753.582817	3.28697013	753.582817	1.78118369
1360	3.22033341	754.14969	3.28592136	754.14969	1.78299406
1360.37423	3.22018388	754.374225	3.28578861	754.374225	1.78449068
1362.07485	3.21954307	755	3.29329237	755	1.7909363
1362.35828	3.21943473	755.374225	3.28911424	755.374225	1.7848464
1363.58282	3.21896566	755.582817	3.2843121	755.582817	1.78334654
1364.14969	3.21874793	756.374225	3.28654797	756.374225	1.79061966
1365	3.21842068	757.074845	3.28046139	757.074845	1.78608921
1365.37423	3.21826895	757.358282	3.28200727	757.358282	1.78885993
1367.07485	3.21761878	757.582817	3.27954679	757.582817	1.78629248
1367.35828	3.21750887	758.374225	3.27933859	758.374225	1.78900972
1368.58282	3.21703295	758.582817	3.2776519	758.582817	1.78857569
1369.14969	3.21681208	759.14969	3.28302427	759.14969	1.79927588
1370	3.21648008	759.582817	3.27904433	759.582817	1.78897385
1370.37423	3.21632613	760	3.27510352	760	1.78797271
1372.07485	3.2156666	760.374225	3.27371551	760.374225	1.78909418
1372.35828	3.21555509	761.582817	3.27173998	761.582817	1.7904251
1373.58282	3.21507235	762.074845	3.27029814	762.074845	1.79161133
1374.14969	3.2148483	762.358282	3.26962378	762.358282	1.79276197
1375	3.21451155	762.374225	3.26955924	762.374225	1.79285494
1375.37423	3.21435538	763.582817	3.274529	763.582817	1.80280607
1377.07485	3.21368649	764.14969	3.26912132	764.14969	1.79512973
1377.35828	3.2135734	764.374225	3.26754318	764.374225	1.79471931
1378.58282	3.21308383	765	3.26812518	765	1.79565982
1379.14969	3.21285661	765.374225	3.26454916	765.374225	1.79402913
1380	3.21251513	765.582817	3.26345217	765.582817	1.7952191
1380.37423	3.21235672	766.374225	3.26237986	766.374225	1.79724202
1382.07485	3.2116785	767.074845	3.27187122	767.074845	1.80975242
1382.35828	3.21156384	767.358282	3.26140379	767.358282	1.79898834
1383.58282	3.21106746	767.582817	3.26064663	767.582817	1.7995856
1384.14969	3.21083709	768.374225	3.2597681	768.374225	1.80349623
1385	3.2104909	768.582817	3.26612551	768.582817	1.81071556
1385.37423	3.21033027	769.14969	3.25875822	769.14969	1.8020054
1387.07485	3.20964278	769.582817	3.25757437	769.582817	1.80250375
1387.35828	3.20952654	770	3.25664818	770	1.80310083
1388.58282	3.20902343	770.374225	3.25586996	770.374225	1.80373514
1389.14969	3.20878992	771.582817	3.25388733	771.582817	1.80610429
1390	3.20843903	772.074845	3.25400091	772.074845	1.80840952
1390.37423	3.20827615	772.358282	3.25499824	772.358282	1.80744023
1392.07485	3.2075795	772.374225	3.25573831	772.374225	1.80767047
1392.35828	3.20746171	773.582817	3.25076782	773.582817	1.80792723
1393.58282	3.20695184	774.14969	3.24922935	774.14969	1.80857158

Final State: j’=5		Final State: j’=3		Final State: j’=2	
E(cm⁻¹)	σ (Å²)	E(cm⁻¹)	σ (Å²)	E(cm⁻¹)	σ (Å²)
1394.14969	3.20671524	774.374225	3.2487427	774.374225	1.80890831
1395	3.20635971	775	3.24760028	775	1.80982413
1395.37423	3.20619463	775.374225	3.24687282	775.374225	1.8103608
1397.07485	3.20548886	776.374225	3.24508266	776.374225	1.81180432
1397.35828	3.20536954	777.074845	3.24397765	777.074845	1.81288635
1398.58282	3.20485302	777.358282	3.2435523	777.358282	1.81332953
1399.14969	3.20461337	778.374225	3.24173687	778.374225	1.81458685
1400	3.20425323	778.582817	3.24136959	778.582817	1.81482259
1400.37423	3.20408595	779.14969	3.2402784	779.14969	1.81550809
1402.07485	3.20337121	780	3.23873506	780	1.81667712
1402.35828	3.20325036	780.374225	3.23812291	780.374225	1.81735238
1403.58282	3.20272729	782.074845	3.23479557	782.074845	1.81891607
1404.14969	3.20248459	782.358282	3.23429644	782.358282	1.81931382
1405	3.20211992	782.374225	3.23422604	782.374225	1.8193363
1405.37423	3.20195047	783.582817	3.2321122	783.582817	1.8209227
1407.07485	3.20122687	784.14969	3.23109034	784.14969	1.82164308
1407.35828	3.20110452	784.374225	3.23064169	784.374225	1.82192739
1408.58282	3.20057498	785	3.22955461	785	1.82271548
1409.14969	3.2003293	785.374225	3.22883576	785.374225	1.82318713
1410	3.19996015	786.374225	3.22703701	786.374225	1.82444749
1410.37423	3.19979745	787.074845	3.22583075	787.074845	1.82533511
1412.07485	3.19905627	787.358282	3.22532926	787.358282	1.82569738
1412.35828	3.19893242	788.374225	3.2235477	788.374225	1.82702933
1413.58282	3.19839652	788.582817	3.22325579	788.582817	1.82730468
1414.14969	3.1981479	789.14969	3.22227818	789.14969	1.82797695
1415	3.19777436	790	3.22064591	790	1.82893503
1415.37423	3.19760972	790.374225	3.21990454	790.374225	1.82938551
1417.07485	3.19685978	792.074845	3.21687934	792.074845	1.83147439
1417.35828	3.1967345	792.358282	3.21637306	792.358282	1.83182406
1418.58282	3.19619233	792.374225	3.21629776	792.374225	1.83184434
1419.14969	3.19594082	793.582817	3.21419433	793.582817	1.83333395
1420	3.19556296	794.14969	3.21318997	794.14969	1.83403277
1420.37423	3.19539644	794.374225	3.21274508	794.374225	1.83431031
1422.07485	3.19463787	795	3.21168847	795	1.83508102
1422.35828	3.19451119	795.374225	3.21098144	795.374225	1.83554303
1423.58282	3.19396286	796.374225	3.20922627	796.374225	1.83677443
1424.14969	3.19370852	797.074845	3.20804819	797.074845	1.83763323
1425	3.1933264	797.358282	3.20755167	797.358282	1.83797993
1425.37423	3.193158	798.374225	3.20572029	798.374225	1.83921926
1427.07485	3.19239099	798.582817	3.20540401	798.582817	1.83947222
1427.35828	3.19226287	799.14969	3.20440979	799.14969	1.84016141
1428.58282	3.19170847	800	3.20292075	800	1.84119507
1429.14969	3.19145131	800.374225	3.20221594	800.374225	1.84165076
1430	3.19106497	802.074845	3.19930424	802.074845	1.84371655
1430.37423	3.19089472	802.358282	3.19881218	802.358282	1.84406112

Final State: j'=5		Final State: j'=3		Final State: j'=2	
E(cm⁻¹)	σ (Å²)	E(cm⁻¹)	σ (Å²)	E(cm⁻¹)	σ (Å²)
1432.07485	3.19011925	802.374225	3.1987329	802.374225	1.84408121
1432.35828	3.18998973	803.582817	3.19669154	803.582817	1.84554777
1433.58282	3.18942918	804.14969	3.19571269	804.14969	1.84623549
1434.14969	3.18916918	804.374225	3.19527285	804.374225	1.84650862
1435	3.18877854	805	3.19424758	805	1.84726628
1435.37423	3.18860636	805.374225	3.19355092	805.374225	1.84772046
1437.07485	3.18782199	806.374225	3.19183393	806.374225	1.84893075
1437.35828	3.18769094	807.074845	3.19068765	807.074845	1.84977683
1438.58282	3.18712358	807.358282	3.19020289	807.358282	1.85011922
1439.14969	3.18686023	808.374225	3.18841379	808.374225	1.85134624
1440	3.18646419	808.582817	3.18811269	808.582817	1.85159687
1440.37423	3.18628944	809.14969	3.18714736	809.14969	1.8522801
1442.07485	3.18548948	810	3.18570212	810	1.85330403
1442.35828	3.18535433	810.374225	3.18501168	810.374225	1.85375525
1443.58282	3.18470671	812.074845	3.18218986	812.074845	1.85579812
1444.14969	3.18443456	812.358282	3.18171167	812.358282	1.85613837
1445	3.18413546	813.582817	3.17965021	813.582817	1.857607
1445.37423	3.18392663	814.14969	3.17869839	814.14969	1.85828618
1447.07485	3.18315943	814.374225	3.17826443	814.374225	1.85855604
1447.35828	3.18300715	815	3.17727355	815	1.85930406
1448.58282	3.18250477	815.374225	3.17658975	815.374225	1.85975271
1449.14969	3.18219005	816.374225	3.17491996	816.374225	1.86094796
1450	3.18181975	817.074845	3.1738116	817.074845	1.86178349
1450.37423	3.18162244	817.358282	3.17334031	817.358282	1.8621217
1452.07485	3.18084626	818.374225	3.1715949	818.374225	1.86333406
1452.35828	3.1806777	818.582817	3.17130864	818.582817	1.86358156
1453.58282	3.18012314	819.14969	3.17037056	819.14969	1.86425662
1454.14969	3.17983761	820	3.16896633	820	1.86526831
1455	3.17938637	820.374225	3.16828914	820.374225	1.86571427
1455.37423	3.17916955	822.074845	3.16555459	822.074845	1.86773249
1457.07485	3.17850272	822.358282	3.16509011	822.358282	1.86806864
1457.35828	3.17834388	822.374225	3.16500274	822.374225	1.86808857
1458.58282	3.17772963	823.582817	3.16308798	823.582817	1.8695194
1459.14969	3.1774449	824.14969	3.16216362	824.14969	1.87019032
1460	3.17695638	824.374225	3.16173568	824.374225	1.87045702
1460.37423	3.17692255	825	3.16078001	825	1.87119573
1462.07485	3.17608802	825.374225	3.16010943	825.374225	1.87163898
1462.35828	3.17594236	826.374225	3.15848805	826.374225	1.8728195
1463.58282	3.17533292	827.074845	3.15741854	827.074845	1.87364459
1464.14969	3.17505136	827.358282	3.15696096	827.358282	1.87397863
1465	3.17461705	828.374225	3.15525989	828.374225	1.87517606
1465.37423	3.17437989	828.582817	3.15498862	828.582817	1.87542037
1467.07485	3.17374757	829.14969	3.15407803	829.14969	1.87608704
1467.35828	3.17354293	830	3.15271509	830	1.87708615
1468.58282	3.1729194	830.374225	3.15205122	830.374225	1.87752666

Final State: j'=5		Final State: j'=3		Final State: j'=2	
E(cm⁻¹)	σ (Å²)	E(cm⁻¹)	σ (Å²)	E(cm⁻¹)	σ (Å²)
1469.14969	3.17263794	832.074845	3.14940424	832.074845	1.87951952
1470	3.17221551	832.358282	3.14895357	832.358282	1.87985144
1470.37423	3.17202853	832.374225	3.14886207	832.374225	1.87987127
1472.07485	3.1712232	833.582817	3.14701111	833.582817	1.881284
1472.35828	3.1710472	834.14969	3.14611439	834.14969	1.88194642
1473.58282	3.17049123	834.374225	3.1456925	834.374225	1.88220984
1474.14969	3.17019819	835	3.14477223	835	1.88293912
1475	3.1697681	835.374225	3.14411505	835.374225	1.88337686
1475.37423	3.16957874	836.374225	3.14254251	836.374225	1.88454237
1477.07485	3.16862707	837.074845	3.14151213	837.074845	1.88535682
1477.35828	3.16854957	837.358282	3.14106842	837.358282	1.88568659
1478.58282	3.16803625	838.374225	3.13941213	838.374225	1.88686878
1479.14969	3.16772943	838.582817	3.13915597	838.582817	1.88710984
1480	3.16735214	839.14969	3.13827314	839.14969	1.88776798
1480.37423	3.16708511	840	3.13695185	840	1.88875417
1482.07485	3.16630723	840.374225	3.13630142	840.374225	1.88918908
1482.35828	3.16614444	842.074845	3.13374274	842.074845	1.89115598
1483.58282	3.16569773	842.358282	3.133306	842.358282	1.89148357
1484.14969	3.16531851	842.374225	3.13321038	842.374225	1.89150322
1485	3.16484497	843.582817	3.13142369	843.582817	1.89289739
1485.37423	3.16462731	844.14969	3.13055482	844.14969	1.89355112
1487.07485	3.16384905	844.374225	3.13013904	844.374225	1.89381116
1487.35828	3.16376049	845	3.12925447	845	1.89453075
1488.58282	3.16307425	845.374225	3.12861078	845.374225	1.89496277
1489.14969	3.1627264	846.374225	3.12708747	846.374225	1.89611284
1490	3.16253098	847.074845	3.12609652	847.074845	1.89691642
1490.37423	3.1622642	847.358282	3.12566678	847.358282	1.89724179
1492.07485	3.16134088	848.374225	3.12405567	848.374225	1.89840825
1492.35828	3.16118824	848.582817	3.12381472	848.582817	1.89864604
1493.58282	3.16074555	849.14969	3.12295987	849.14969	1.8992953
1494.14969	3.16034678	850	3.12168055	850	1.90026824
1495	3.15996246	850.374225	3.12104364	850.374225	1.90069732
1495.37423	3.15968188	852.074845	3.11857395	852.074845	1.9026375
1497.07485	3.15887736	852.358282	3.11815123	852.358282	1.90296061
1497.35828	3.15866169	852.374225	3.11805147	852.374225	1.90298003
1498.58282	3.15816951	853.582817	3.11632952	853.582817	1.9043551
1499.14969	3.15779724	854.14969	3.11548871	854.14969	1.90499983
1500	3.15746746	854.374225	3.11507913	854.374225	1.90525629
1500.37423	3.15722732	855	3.1142305	855	1.90596597
1502.07485	3.15631263	855.374225	3.11360041	855.374225	1.90639203
1502.35828	3.15605156	856.374225	3.11212667	856.374225	1.90752611
1503.58282	3.15584973	857.074845	3.11117542	857.074845	1.90831849
1504.14969	3.15534643	857.358282	3.11075975	857.358282	1.90863932
1505.37423	3.15463499	858.374225	3.10919411	858.374225	1.9097894
1507.07485	3.15375396	858.582817	3.1089685	858.582817	1.91002385

Final State: j'=5		Final State: j'=3		Final State: j'=2	
E(cm⁻¹)	σ (Å²)	E(cm⁻¹)	σ (Å²)	E(cm⁻¹)	σ (Å²)
1507.35828	3.15369943	859.14969	3.10814178	859.14969	1.91066399
1508.58282	3.15306637	860	3.10690475	860	1.91162315
1509.14969	3.1527727	860.374225	3.10628146	860.374225	1.91204614
1510.37423	3.1521462	862.074845	3.1039014	862.074845	1.91395865
1512.07485	3.15131862	862.358282	3.10349276	862.358282	1.91427712
1512.35828	3.1511676	862.374225	3.10338881	862.374225	1.91429623
1513.58282	3.15053699	863.582817	3.10173205	863.582817	1.91565153
1514.14969	3.15024883	864.14969	3.1009195	864.14969	1.91628692
1515.37423	3.14962747	864.374225	3.10051612	864.374225	1.91653964
1517.07485	3.14876985	865	3.09970371	865	1.91723901
1517.35828	3.14862623	865.374225	3.09908727	865.374225	1.91765882
1518.58282	3.14800475	866.374225	3.09766342	866.374225	1.9187763
1519.14969	3.14771708	867.074845	3.09675216	867.074845	1.9195571
1520.37423	3.14709856	867.358282	3.09635063	867.358282	1.91987317
1522.07485	3.14623451	868.374225	3.09483073	868.374225	1.92100616
1522.35828	3.14609067	868.582817	3.09462056	868.582817	1.92123723
1523.58282	3.14546907	869.14969	3.09382219	869.14969	1.92186781
1524.14969	3.14518124	870	3.09262767	870	1.92281264
1525.37423	3.14455932	870.374225	3.09201812	870.374225	1.92322917
1527.07485	3.14369564	872.074845	3.08972815	872.074845	1.9251129
1527.35828	3.14355183	872.358282	3.08933373	872.358282	1.92542655
1528.58282	3.14292952	872.374225	3.08922554	872.374225	1.92544524
1529.14969	3.14264116	873.582817	3.08763437	873.582817	1.92677998
1530.37423	3.14201837	874.14969	3.08685025	874.14969	1.92740563
1532.07485	3.1411534	874.374225	3.08645305	874.374225	1.92765433
1533.58282	3.14038628	875	3.08567706	875	1.92834305
1534.14969	3.14009789	875.374225	3.08507434	875.374225	1.92875621
1535.37423	3.13947487	876.374225	3.08370066	876.374225	1.92985634
1537.07485	3.13860951	877.074845	3.08282967	877.074845	1.93062507
1538.58282	3.13784208	877.358282	3.08244237	877.358282	1.93093622
1539.14969	3.13755359	878.374225	3.08096836	878.374225	1.9320512
1540.37423	3.13693037	878.582817	3.08077379	878.582817	1.93227878
1542.07485	3.1360649	879.14969	3.08000391	879.14969	1.93289938
1543.58282	3.13529753	880	3.07885213	880	1.93382918
1544.14969	3.13500907	880.374225	3.07825625	880.374225	1.93423886
1545.37423	3.13438602	882.074845	3.07605695	882.074845	1.93609249
1547.07485	3.13352087	882.358282	3.07567681	882.358282	1.93640107
1548.58282	3.13275389	882.374225	3.07556427	882.374225	1.93641924
1549.14969	3.1324656	883.582817	3.07403908	883.582817	1.93773246
1550.37423	3.13184299	884.14969	3.0732835	884.14969	1.93834788
1552.07485	3.13097854	884.374225	3.07289242	884.374225	1.93859224
1553.58282	3.13021228	885	3.07215313	885	1.9392699
1554.14969	3.12992431	885.374225	3.07156409	885.374225	1.93967592
1555.37423	3.12930239	886.374225	3.07024076	886.374225	1.94075779
1557.07485	3.12843903	887.074845	3.06941031	887.074845	1.94151392

Final State: j'=5		Final State: j'=3		Final State: j'=2	
E(cm⁻¹)	σ (Å²)	E(cm⁻¹)	σ (Å²)	E(cm⁻¹)	σ (Å²)
1558.58282	3.12767385	887.358282	3.06903731	887.358282	1.94181986
1559.14969	3.1273863	888.374225	3.0676093	888.374225	1.94291578
1560.37423	3.12676536	888.582817	3.06743048	888.582817	1.94313979
1562.07485	3.12590347	889.14969	3.06668919	889.14969	1.94374986
1563.58282	3.1251397	890	3.06558031	890	1.94466381
1564.14969	3.12485271	890.374225	3.06499804	890.374225	1.94506613
1565.37423	3.124233	892.074845	3.06288985	892.074845	1.94688806
1567.07485	3.12337293	892.358282	3.06252401	892.358282	1.94719112
1568.58282	3.12261089	892.374225	3.062407	892.374225	1.94720869
1569.14969	3.12232457	893.582817	3.06094811	893.582817	1.94849924
1570.37423	3.12170638	894.14969	3.06022117	894.14969	1.94910391
1572.07485	3.1208485	894.374225	3.05983612	894.374225	1.94934354
1573.58282	3.1200885	895	3.05913378	895	1.95000948
1574.14969	3.11980299	895.374225	3.05855826	895.374225	1.95040791
1575.37423	3.11918654	896.374225	3.05728549	896.374225	1.95147032
1577.07485	3.11833122	897.074845	3.0564958	897.074845	1.95221319
1578.58282	3.11757359	897.358282	3.05613712	897.358282	1.95251355
1579.14969	3.11729471	898.374225	3.0547551	898.374225	1.95358901
1580.37423	3.11667454	898.582817	3.05459222	898.582817	1.95380933
1582.07485	3.1158221	899.14969	3.05387961	899.14969	1.95440814
1583.58282	3.11506711	900	3.05281375	900	1.95530508
1584.14969	3.11478351	900.374225	3.05224493	900.374225	1.95569943
1585.37423	3.11417133	902.074845	3.05022826	902.074845	1.95748737
1588.58282	3.11257007	902.358282	3.04987675	902.358282	1.95778481
1589.14969	3.1122876	902.374225	3.04975502	902.374225	1.95780147
1590.37423	3.1116779	903.582817	3.04836282	903.582817	1.95906766
1593.58282	3.11008339	904.14969	3.04766456	904.14969	1.95966044
1594.14969	3.10980217	904.374225	3.04728533	904.374225	1.95989481
1595.37423	3.10919517	905	3.04662023	905	1.96054824
1598.58282	3.107608	905.374225	3.04605811	905.374225	1.96093827
1599.14969	3.10732812	906.374225	3.04483588	906.374225	1.96197942
1600.37423	3.10672403	907.074845	3.04408729	907.074845	1.96270784
1603.58282	3.10514478	907.358282	3.04374296	907.358282	1.9630021
1604.14969	3.10486632	908.374225	3.04240672	908.374225	1.96405475
1605.37423	3.10426538	908.582817	3.04226005	908.582817	1.96427118
1608.58282	3.10269455	909.14969	3.04157611	909.14969	1.9648573
1609.14969	3.10241761	910	3.04055334	910	1.96573533
1610.37423	3.10181999	910.374225	3.04010437	910.374225	1.9661212
1613.58282	3.10025811	912.074845	3.03807297	912.074845	1.96786994
1614.14969	3.09998278	912.358282	3.03773583	912.358282	1.96816066
1615.37423	3.09938868	913.582817	3.03628398	913.582817	1.96941407
1618.58282	3.09783624	914.14969	3.03561448	914.14969	1.96999282
1619.14969	3.09756261	915	3.03461324	915	1.97085912
1620.37423	3.0969722	915.374225	3.03417376	915.374225	1.97123964
1623.58282	3.09542965	917.074845	3.03218567	917.074845	1.97296252

Final State: j'=5		Final State: j'=3		Final State: j'=2	
E(cm⁻¹)	σ (Å²)	E(cm⁻¹)	σ (Å²)	E(cm⁻¹)	σ (Å²)
1624.14969	3.09515779	917.358282	3.03185576	917.358282	1.97324855
1625.37423	3.09457126	918.582817	3.03043523	918.582817	1.97447992
1628.58282	3.09303903	919.14969	3.0297803	919.14969	1.97504716
1629.14969	3.09276903	920	3.02880109	920	1.97589357
1630.37423	3.09218653	920.374225	3.02837139	920.374225	1.97626401
1633.58282	3.09066502	922.074845	3.02642895	922.074845	1.97791798
1634.14969	3.09039694	922.358282	3.0261071	922.358282	1.97818447
1635.37423	3.08981862	923.582817	3.02471582	923.582817	1.97915479
1638.58282	3.08830824	924.14969	3.02403203	924.14969	1.97977089
1639.14969	3.08804215	925	3.02306409	925	1.98098924
1640.37423	3.08746814	925.374225	3.02262404	925.374225	1.98117456
1643.58282	3.08596923	927.074845	3.0207966	927.074845	1.98304355
1644.14969	3.08570521	927.358282	3.02048168	927.358282	1.98322826
1645.37423	3.08513564	928.582817	3.01898404	928.582817	1.98486891
1648.58282	3.08364853	929.14969	3.01854788	929.14969	1.98504748
1649.14969	3.0833866	930	3.01730472	930	1.98617541
1650.37423	3.0828216	930.374225	3.01710363	930.374225	1.98642207
1653.58282	3.08134659	932.074845	3.01523507	932.074845	1.98841074
1654.14969	3.08108681	932.358282	3.01489713	932.358282	1.98843827
1655.37423	3.08052651	933.582817	3.01360985	933.582817	1.98971971
1658.58282	3.0790639	934.14969	3.01299857	934.14969	1.99013406
1659.14969	3.07880633	935	3.01206336	935	1.99069481
1660.37423	3.0782508	935.374225	3.01164457	935.374225	1.99073181
1663.58282	3.07680081	937.074845	3.00981489	937.074845	1.99319946
1664.14969	3.07654549	937.358282	3.00956596	937.358282	1.9935965
1665.37423	3.07599484	938.582817	3.00826743	938.582817	1.99449556
1668.58282	3.07455762	939.14969	3.00766234	939.14969	1.99494429
1670.37423	3.07375898	940	3.00671972	940	1.99532953
1673.58282	3.07233493	940.374225	3.00619091	940.374225	1.99629627
1675.37423	3.07154354	942.074845	3.00462931	942.074845	1.99822647
1678.58282	3.07013273	942.358282	3.00433296	942.358282	1.99837772
1680.37423	3.06934878	943.582817	3.00305365	943.582817	1.99936053
1683.58282	3.06795136	944.14969	3.0024625	944.14969	1.99983336
1685.37423	3.06717493	945	3.00156499	945	2.00047081
1688.58282	3.06579105	945.374225	3.00114318	945.374225	2.00061861
1690.37423	3.0650222	947.074845	2.99917286	947.074845	2.00285438
1693.58282	3.06365196	947.358282	2.99924193	947.358282	2.00346596
1695.37423	3.06289076	948.582817	2.99797884	948.582817	2.00419708
1698.58282	3.06153422	949.14969	2.99740232	949.14969	2.00467364
1700.37423	3.0607807	950	2.99653986	950	2.0053923
1703.58282	3.05943796	950.374225	2.99615956	950.374225	2.00570072
1705.37423	3.05869216	952.074845	2.99433974	952.074845	2.00774736
1708.58282	3.05736325	952.358282	2.99424105	952.358282	2.00782159
1710.37423	3.05662518	953.582817	2.99308259	953.582817	2.00911794
1713.58282	3.05531013	954.14969	2.99249518	954.14969	2.00942457

Final State: j'=5		Final State: j'=3		Final State: j'=2	
E(cm⁻¹)	σ (Å²)	E(cm⁻¹)	σ (Å²)	E(cm⁻¹)	σ (Å²)
1715.37423	3.05457979	955	2.99165271	955	2.01008238
1718.58282	3.05327862	955.374225	2.99128667	955.374225	2.01037994
1720.37423	3.05255601	957.074845	2.98966491	957.074845	2.01149715
1723.58282	3.0512687	957.358282	2.98930229	957.358282	2.0117915
1725.37423	3.05055383	958.582817	2.98794343	958.582817	2.01252904
1728.58282	3.04928036	959.14969	2.98765203	959.14969	2.0141754
1730.37423	3.04857323	960	2.98690077	960	2.01483665
1733.58282	3.04731357	960.374225	2.98653005	960.374225	2.01511161
1735.37423	3.04661415	962.074845	2.98479585	962.074845	2.01680861
1738.58282	3.04536834	962.358282	2.9845493	962.358282	2.01690887
1740.37423	3.04467671	963.582817	2.98335205	963.582817	2.01779166
1743.58282	3.04344563	964.14969	2.98276726	964.14969	2.01894012
1745.37423	3.04275913	965	2.98202073	965	2.01925323
1748.58282	3.0415618	965.374225	2.98166752	965.374225	2.01936542
1750.37423	3.04086588	967.074845	2.98006722	967.074845	2.02191047
1753.58282	3.03965231	967.358282	2.97993382	967.358282	2.02188662
1755.37423	3.03899001	968.582817	2.97882113	968.582817	2.02263342
1758.58282	3.03779456	969.14969	2.97829726	969.14969	2.0227756
1760.37423	3.03713661	970	2.97700487	970	2.02457848
1763.58282	3.03595339	970.374225	2.97712273	970.374225	2.02495322
1765.37423	3.03530126	972.074845	2.9756307	972.074845	2.02569415
1768.58282	3.0341317	972.358282	2.97537322	972.358282	2.02585376
1770.37423	3.03348675	973.582817	2.97417464	973.582817	2.02599532
1773.58282	3.0323299	974.14969	2.97377189	974.14969	2.02872291
1775.37423	3.0316932	975	2.97301276	975	2.02835414
1780.37423	3.02994116	975.374225	2.97299959	975.374225	2.02913359
1785.37423	3.02816703	977.074845	2.97129405	977.074845	2.0296816
1790.37423	3.02646162	977.358282	2.97088712	977.358282	2.03032766
1795.37423	3.02471196	978.582817	2.96984328	978.582817	2.03248377
1800.37423	3.02302933	979.14969	2.96947632	979.14969	2.03215621
1805.37423	3.0213422	980	2.96846022	980	2.03364337
1810.37423	3.01968822	980.374225	2.96829547	980.374225	2.0333891
1815.37423	3.01804692	982.074845	2.96671843	982.074845	2.03434561
1820.37423	3.01641749	982.358282	2.96626131	982.358282	2.03376007
1825.37423	3.01481771	983.582817	2.96607559	983.582817	2.03511327
1830.37423	3.01322958	984.14969	2.96511995	984.14969	2.03730015
1835.37423	3.01165851	985	2.96458122	985	2.03694132
1840.37423	3.01010434	985.374225	2.96420333	985.374225	2.0372523
1845.37423	3.00856676	987.074845	2.96265294	987.074845	2.03839595
1850.37423	3.00704555	987.358282	2.96212094	987.358282	2.03783102
1855.37423	3.00554046	988.582817	2.96152393	988.582817	2.04022243
1860.37423	3.00405127	989.14969	2.96103742	989.14969	2.0405984
1865.37423	3.00257777	990	2.960303	990	2.04120387
1870.37423	3.00111973	990.374225	2.95997101	990.374225	2.04147062
1875.37423	2.99967697	992.074845	2.95869346	992.074845	2.04317868

Final State: j'=5		Final State: j'=3		Final State: j'=2	
E(cm⁻¹)	σ (Å²)	E(cm⁻¹)	σ (Å²)	E(cm⁻¹)	σ (Å²)
1880.37423	2.99824929	992.358282	2.95852934	992.358282	2.04357898
1885.37423	2.99683652	993.582817	2.95749918	993.582817	2.04438325
1890.37423	2.99543847	994.14969	2.95703635	994.14969	2.0448264
1895.37423	2.99405497	995	2.95634836	995	2.04550292
1900.37423	2.99268591	995.374225	2.9560462	995.374225	2.04580201
1905.37423	2.9913311	997.074845	2.95470913	997.074845	2.04723804
1910.37423	2.98999041	997.358282	2.95449124	997.358282	2.04747621
		998.582817	2.9535324	998.582817	2.04846585
		999.14969	2.95306931	999.14969	2.04890007
		1000	2.95248977	1000	2.04979272
		1000.37423	2.9522472	1000.37423	2.05006062
		1002.07485	2.95090042	1002.07485	2.05136699
		1002.35828	2.95068506	1002.35828	2.05159632
		1003.58282	2.94976144	1003.58282	2.05258716
		1004.14969	2.94933656	1004.14969	2.0530448
		1005	2.94870291	1005	2.0537297
		1005.37423	2.94842334	1005.37423	2.05403032
		1007.07485	2.94716253	1007.07485	2.05539363
		1007.35828	2.94695443	1007.35828	2.05562383
		1008.58282	2.94608166	1008.58282	2.0566255
		1009.14969	2.94567139	1009.14969	2.05707371
		1010	2.94505754	1010	2.05774543
		1010.37423	2.94478679	1010.37423	2.05804091
		1012.07485	2.94356987	1012.07485	2.05938154
		1012.35828	2.94336834	1012.35828	2.0596044
		1013.58282	2.94250172	1013.58282	2.06056508
		1014.14969	2.94210279	1014.14969	2.06100866
		1015	2.94150844	1015	2.06167294
		1015.37423	2.94124631	1015.37423	2.06196479
		1017.07485	2.94006942	1017.07485	2.06328735
		1017.35828	2.93987451	1017.35828	2.06350693
		1018.58282	2.93903611	1018.58282	2.06445239
		1019.14969	2.93864995	1019.14969	2.06488847
		1020	2.93807435	1020	2.06554088
		1020.37423	2.9378202	1020.37423	2.06582723
		1022.07485	2.93667801	1022.07485	2.06712398
		1022.35828	2.93648883	1022.35828	2.06733934
		1023.58282	2.93567532	1023.58282	2.06826724
		1024.14969	2.93530086	1024.14969	2.0686954
		1025	2.93474297	1025	2.06933612
		1025.37423	2.93449656	1025.37423	2.06961736
		1027.07485	2.93338997	1027.07485	2.07089089
		1027.35828	2.933208	1027.35828	2.07110247
		1028.58282	2.93241867	1028.58282	2.07201339
		1029.14969	2.93205595	1029.14969	2.07243368

Final State: j'=5		Final State: j'=3		Final State: j'=2	
E(cm⁻¹)	σ (Å²)	E(cm⁻¹)	σ (Å²)	E(cm⁻¹)	σ (Å²)
		1030	2.93151561	1030	2.07306256
		1030.37423	2.93127682	1030.37423	2.07333853
		1032.07485	2.93020481	1032.07485	2.07458815
		1032.35828	2.9300286	1032.35828	2.07479571
		1033.58282	2.92926396	1033.58282	2.07568938
		1034.14969	2.92891258	1034.14969	2.07610172
		1035	2.92838942	1035	2.07671862
		1035.37423	2.92815796	1035.37423	2.07698923
		1037.07485	2.92711981	1037.07485	2.07821498
		1037.35828	2.92694929	1037.35828	2.07841855
		1038.58282	2.92620879	1038.58282	2.07929496
		1039.14969	2.92586863	1039.14969	2.07969934
		1040	2.92536213	1040	2.08030422
		1040.37423	2.92513804	1040.37423	2.08056965
		1042.07485	2.92413307	1042.07485	2.0817713
		1042.35828	2.92396813	1042.35828	2.0819709
		1043.58282	2.9232512	1043.58282	2.08283
		1044.14969	2.92292195	1044.14969	2.08322635
		1045	2.92243183	1045	2.08381919
		1045.37423	2.92221484	1045.37423	2.08407933
		1047.07485	2.92124221	1047.07485	2.08525688
		1047.35828	2.92108268	1047.35828	2.08545244
		1048.58282	2.92038884	1048.58282	2.08629417
		1049.14969	2.92007022	1049.14969	2.08668245
		1050	2.91959609	1050	2.08726325
		1050.37423	2.91938603	1050.37423	2.08751807
		1052.07485	2.91844497	1052.07485	2.08867148
		1052.35828	2.91829076	1052.35828	2.08886304
		1053.58282	2.91761936	1053.58282	2.08968738
		1054.14969	2.91731112	1054.14969	2.09006759
		1055	2.91685256	1055	2.09063631
		1055.37423	2.91664924	1055.37423	2.09088581
		1057.07485	2.91573895	1057.07485	2.0920151
		1057.35828	2.91558985	1057.35828	2.0922025
		1058.58282	2.91494033	1058.58282	2.09300946
		1059.14969	2.91464219	1059.14969	2.09338163
		1060	2.91419879	1060	2.09393825
		1060.37423	2.91400203	1060.37423	2.09418241
		1062.07485	2.91312163	1062.07485	2.09528745
		1062.35828	2.91297753	1062.35828	2.09547094
		1063.58282	2.91234928	1063.58282	2.09626043
		1064.14969	2.91206094	1064.14969	2.09662452
		1065	2.91163227	1065	2.09716906
		1065.37423	2.91144186	1065.37423	2.09740789
		1067.07485	2.91059046	1067.07485	2.09848874

Final State: j’=5		Final State: j’=3		Final State: j’=2	
E(cm⁻¹)	σ (Å²)	E(cm⁻¹)	σ (Å²)	E(cm⁻¹)	σ (Å²)
		1067.35828	2.91045127	1067.35828	2.09866819
		1068.58282	2.9098436	1068.58282	2.09944027
		1069.14969	2.90956482	1069.14969	2.09979628
		1070	2.9091504	1070	2.10032876
		1070.37423	2.90896617	1070.37423	2.10056228
		1072.07485	2.9081429	1072.07485	2.10161894
		1072.35828	2.90800845	1072.35828	2.10179436
		1073.58282	2.90742074	1073.58282	2.10254901
		1074.14969	2.90715114	1074.14969	2.10289696
		1075	2.90675058	1075	2.1034174
		1075.37423	2.9065723	1075.37423	2.10364561
		1077.07485	2.90577625	1077.07485	2.10467815
		1077.35828	2.9056464	1077.35828	2.10484955
		1078.58282	2.90507797	1078.58282	2.10558688
		1079.14969	2.90481729	1079.14969	2.10592682
		1080	2.90443008	1080	2.10643511
		1080.37423	2.90425758	1080.37423	2.10665805
		1082.07485	2.90348979	1082.07485	2.10766665
		1082.35828	2.90336235	1082.35828	2.10783394
		1083.58282	2.90281257	1083.58282	2.10855396
		1084.14969	2.90256049	1084.14969	2.1088859
		1085	2.90218616	1085	2.10938221
		1085.37423	2.90201921	1085.37423	2.10959984
		1087.07485	2.90127682	1087.07485	2.11058447
		1087.35828	2.90115358	1087.35828	2.11074775
		1088.58282	2.90062173	1088.58282	2.11145053
		1089.14969	2.90037792	1089.14969	2.1117745
		1090	2.90001601	1090	2.11225885
		1090.37423	2.8998544	1090.37423	2.11247123
		1092.07485	2.89913646	1092.07485	2.11343197
		1092.35828	2.89901724	1092.35828	2.11359128
		1093.58282	2.89850262	1093.58282	2.1142769
		1094.14969	2.89826676	1094.14969	2.11459293
		1095	2.89791679	1095	2.11506538
		1095.37423	2.89776027	1095.37423	2.11527252
		1097.07485	2.8970658	1097.07485	2.11620951
		1097.35828	2.89695046	1097.35828	2.11636487
		1098.58282	2.89645234	1098.58282	2.11703341
		1099.14969	2.89622412	1099.14969	2.11734154
		1100	2.89588564	1100	2.11780219
		1100.37423	2.89573396	1100.37423	2.1180041
		1102.07485	2.89506195	1102.07485	2.11891748
		1102.35828	2.8949503	1102.35828	2.11906891
		1103.58282	2.89446799	1103.58282	2.11972048
		1104.14969	2.89424704	1104.14969	2.12002077

Final State: j’=5		Final State: j’=3		Final State: j’=2	
E(cm⁻¹)	σ (Å²)	E(cm⁻¹)	σ (Å²)	E(cm⁻¹)	σ (Å²)
		1105	2.89391943	1105	2.12046965
		1105.37423	2.8937725	1105.37423	2.12066641
		1107.07485	2.89312194	1107.07485	2.12155633
		1107.35828	2.89301382	1107.35828	2.12170386
		1108.58282	2.89254658	1108.58282	2.12233859
		1109.14969	2.89233256	1109.14969	2.12263108
		1110	2.8920154	1110	2.1230683
		1110.37423	2.89187291	1110.37423	2.12325992
		1112.07485	2.89124279	1112.07485	2.12412657
		1112.35828	2.89113802	1112.35828	2.12427024
		1113.58282	2.89068509	1113.58282	2.12488825
		1114.14969	2.8904777	1114.14969	2.12517302
		1115	2.89017043	1115	2.12559866
		1115.37423	2.89003216	1115.37423	2.12578519
		1117.07485	2.88942148	1117.07485	2.12662878
		1117.35828	2.88931991	1117.35828	2.12676859
		1118.58282	2.88888053	1118.58282	2.12737005
		1119.14969	2.88867942	1119.14969	2.12764716
		1120	2.88838154	1120	2.12806134
		1120.37423	2.88824726	1120.37423	2.12824283
		1122.07485	2.88765495	1122.07485	2.12906355
		1122.35828	2.8875564	1122.35828	2.12919957
		1123.58282	2.88712984	1123.58282	2.12978461
		1124.14969	2.88693463	1124.14969	2.13005415
		1125	2.88664565	1125	2.13045697
		1125.37423	2.88651511	1125.37423	2.13063348
		1127.07485	2.88594014	1127.07485	2.13143159
		1127.35828	2.88584443	1127.35828	2.13156385
		1128.58282	2.88542992	1128.58282	2.13213266
		1129.14969	2.88524028	1129.14969	2.13239469
		1130	2.88495967	1130	2.1327863
		1130.37423	2.88483265	1130.37423	2.13295787
		1132.07485	2.88427396	1132.07485	2.13373361
		1132.35828	2.8841809	1132.35828	2.13386215
		1133.58282	2.88377771	1133.58282	2.13441492
		1134.14969	2.88359326	1134.14969	2.13466955
		1135	2.88332049	1135	2.13505008
		1135.37423	2.88319675	1135.37423	2.13521678
		1137.07485	2.88265329	1137.07485	2.13597047
		1137.35828	2.88256272	1137.35828	2.13609527
		1138.58282	2.88217005	1138.58282	2.13663221
		1139.14969	2.88199047	1139.14969	2.1368796
		1140	2.88172499	1140	2.13724911
		1140.37423	2.8816043	1140.37423	2.13741101
		1142.07485	2.88107499	1142.07485	2.13814284

Final State: j’=5		Final State: j’=3		Final State: j’=2	
E(cm⁻¹)	σ (Å²)	E(cm⁻¹)	σ (Å²)	E(cm⁻¹)	σ (Å²)
		1142.35828	2.88098672	1142.35828	2.13826408
		1143.58282	2.88060378	1143.58282	2.1387854
		1144.14969	2.88042868	1144.14969	2.13902553
		1145	2.88016998	1145	2.13938431
		1145.37423	2.88005208	1145.37423	2.13954144
		1147.07485	2.87953584	1147.07485	2.14025185
		1147.35828	2.8794497	1147.35828	2.14036952
		1148.58282	2.87907571	1148.58282	2.14087547
		1149.14969	2.87890472	1149.14969	2.14110849
		1150	2.87865224	1150	2.14145668
		1150.37423	2.87853688	1150.37423	2.14160914
		1152.07485	2.87803258	1152.07485	2.14229846
		1152.35828	2.87794835	1152.35828	2.14241263
		1153.58282	2.87758247	1153.58282	2.1429035
		1154.14969	2.87741524	1154.14969	2.14312956
		1155	2.87716838	1155	2.14346736
		1155.37423	2.8770553	1155.37423	2.14361526
		1157.07485	2.8765617	1157.07485	2.14428394
		1157.35828	2.8764792	1157.35828	2.14439469
		1158.58282	2.87612049	1158.58282	2.14487084
		1159.14969	2.87595652	1159.14969	2.14509014
		1160	2.87571458	1160	2.14541784
		1160.37423	2.87560344	1160.37423	2.14556131
		1162.07485	2.87511897	1162.07485	2.14621009
		1162.35828	2.87503794	1162.35828	2.14631757
		1163.58282	2.87468498	1163.58282	2.14677972
		1164.14969	2.87452705	1164.14969	2.1469928
		1165	2.87428527	1165	2.14731097
		1165.37423	2.87417541	1165.37423	2.14745041
		1167.07485	2.87369577	1167.07485	2.14808197
		1167.35828	2.87361507	1167.35828	2.14818687
		1168.58282	2.8732601	1168.58282	2.1486403
		1169.14969	2.87309696	1169.14969	2.14885265
		1170	2.87288302	1170	2.14917097
		1170.37423	2.87276482	1170.37423	2.14930168
		1172.07485	2.87235107	1172.07485	2.14990116
		1172.35828	2.87239011	1172.35828	2.14991338
		1173.58282	2.87188859	1173.58282	2.15042071
		1174.14969	2.8718145	1174.14969	2.150602
		1175	2.8715795	1175	2.15086333
		1175.37423	2.87140295	1175.37423	2.1510363
		1177.07485	2.87095733	1177.07485	2.15163173
		1177.35828	2.87086639	1177.35828	2.15174623
		1178.58282	2.87059111	1178.58282	2.15211683
		1179.14969	2.87049483	1179.14969	2.15228525

Final State: j’=5		Final State: j’=3		Final State: j’=2	
E(cm⁻¹)	σ (Å²)	E(cm⁻¹)	σ (Å²)	E(cm⁻¹)	σ (Å²)
		1180	2.87019981	1180	2.15259868
		1180.37423	2.87008246	1180.37423	2.15273442
		1182.07485	2.86971212	1182.07485	2.15322184
		1182.35828	2.86957029	1182.35828	2.15335655
		1183.58282	2.86926977	1183.58282	2.15376404
		1184.14969	2.86909192	1184.14969	2.15397265
		1185	2.8688473	1185	2.15426595
		1185.37423	2.86873582	1185.37423	2.15439486
		1187.07485	2.86839569	1187.07485	2.15489629
		1187.35828	2.8682471	1187.35828	2.15504462
		1188.58282	2.86792134	1188.58282	2.15539185
		1189.14969	2.86776163	1189.14969	2.15558419
		1190	2.86752521	1190	2.15586391
		1190.37423	2.86741738	1190.37423	2.15598627
		1192.07485	2.86705417	1192.07485	2.15640802
		1192.35828	2.86694977	1192.35828	2.15657661
		1193.58282	2.86663733	1193.58282	2.1569208
		1194.14969	2.86644877	1194.14969	2.15713443
		1195	2.86621256	1195	2.15740696
		1195.37423	2.86610618	1195.37423	2.15752435
		1197.07485	2.86564721	1197.07485	2.15805734
		1197.35828	2.86556788	1197.35828	2.15814839
		1198.58282	2.86534903	1198.58282	2.15846403
		1199.14969	2.86524223	1199.14969	2.15863211
		1200	2.86490271	1200	2.15889565
		1200.37423	2.86478995	1200.37423	2.15901623
		1202.07485	2.86431979	1202.07485	2.15953924
		1202.35828	2.86423864	1202.35828	2.15962676
		1203.58282	2.86397809	1203.58282	2.15998303
		1204.14969	2.86400674	1204.14969	2.16007946
		1205	2.86362317	1205	2.16027831
		1205.37423	2.86368597	1205.37423	2.16034822
		1207.07485	2.86302846	1207.07485	2.16096789
		1207.35828	2.86326392	1207.35828	2.1609368
		1208.58282	2.86267776	1208.58282	2.16135772
		1209.14969	2.86246456	1209.14969	2.16157582
		1210	2.86258609	1210	2.16159923
		1210.37423	2.86230964	1210.37423	2.16179174
		1212.07485	2.86169066	1212.07485	2.16239376
		1212.35828	2.86182382	1212.35828	2.16236694
		1213.58282	2.86155491	1213.58282	2.16256142
		1214.14969	2.86127744	1214.14969	2.16283009
		1215	2.86100859	1215	2.16309661
		1215.37423	2.86087346	1215.37423	2.1632245
		1217.07485	2.86053845	1217.07485	2.1635858

Final State: j’=5		Final State: j’=3		Final State: j’=2	
E(cm⁻¹)	σ (Å²)	E(cm⁻¹)	σ (Å²)	E(cm⁻¹)	σ (Å²)
		1217.35828	2.86044651	1217.35828	2.16367314
		1218.58282	2.86007311	1218.58282	2.16402999
		1219.14969	2.85990197	1219.14969	2.1642029
		1220	2.85999362	1220	2.16426445
		1220.37423	2.85977284	1220.37423	2.16426226
		1222.07485	2.85919053	1222.07485	2.16490462
		1222.35828	2.8590422	1222.35828	2.16506314
		1223.58282	2.85884829	1223.58282	2.16526977
		1224.14969	2.85853881	1224.14969	2.1654926
		1225	2.8585332	1225	2.16555249
		1225.37423	2.85834012	1225.37423	2.16570179
		1227.07485	2.85789791	1227.07485	2.16612862
		1227.35828	2.85778424	1227.35828	2.16622548
		1228.58282	2.85745222	1228.58282	2.1665237
		1229.14969	2.85756945	1229.14969	2.16645478
		1230	2.85787521	1230	2.16649878
		1230.37423	2.85721361	1230.37423	2.16679796
		1232.07485	2.85644373	1232.07485	2.16745491
		1232.35828	2.85661093	1232.35828	2.16731899
		1233.58282	2.85614051	1233.58282	2.16772832
		1234.14969	2.8561117	1234.14969	2.16774631
		1235	2.85587596	1235	2.16795666
		1235.37423	2.85576696	1235.37423	2.16804613
		1237.07485	2.85532356	1237.07485	2.16842834
		1237.35828	2.85522132	1237.35828	2.16846051
		1238.58282	2.85494441	1238.58282	2.16873243
		1239.14969	2.85478707	1239.14969	2.16887647
		1240	2.85455939	1240	2.16906685
		1240.37423	2.85445369	1240.37423	2.16914858
		1242.07485	2.85400971	1242.07485	2.16951436
		1242.35828	2.85393528	1242.35828	2.16957332
		1243.58282	2.8536052	1243.58282	2.1698291
		1244.14969	2.85346016	1244.14969	2.16994981
		1245	2.85323293	1245	2.17012828
		1245.37423	2.85312656	1245.37423	2.17020464
		1247.07485	2.8526763	1247.07485	2.17056067
		1247.35828	2.85260037	1247.35828	2.17061873
		1248.58282	2.85226471	1248.58282	2.17086768
		1249.14969	2.85211878	1249.14969	2.17098273
		1250	2.85188933	1250	2.17115397
		1250.37423	2.85178135	1250.37423	2.17122866
		1252.07485	2.85132743	1252.07485	2.17156679
		1252.35828	2.85125047	1252.35828	2.17162254
		1253.58282	2.85091023	1253.58282	2.17186017
		1254.14969	2.85076243	1254.14969	2.17196996

Final State: j'=5		Final State: j'=3		Final State: j'=2	
E(cm⁻¹)	σ (Å²)	E(cm⁻¹)	σ (Å²)	E(cm⁻¹)	σ (Å²)
		1255	2.85052994	1255	2.17213657
		1255.37423	2.85042036	1255.37423	2.17220883
		1257.07485	2.84996075	1257.07485	2.17253468
		1257.35828	2.84988274	1257.35828	2.17258841
		1258.58282	2.84953775	1258.58282	2.1728187
		1259.14969	2.84938837	1259.14969	2.172925
		1260	2.84915286	1260	2.17308296
		1260.37423	2.84904158	1260.37423	2.17315175
		1262.07485	2.84857575	1262.07485	2.17346361
		1262.35828	2.84849663	1262.35828	2.17351513
		1263.58282	2.84814645	1263.58282	2.17373605
		1264.14969	2.84799513	1264.14969	2.17383814
		1265	2.84775618	1265	2.17398983
		1265.37423	2.84764308	1265.37423	2.17405588
		1267.07485	2.84717058	1267.07485	2.17435541
		1267.35828	2.8470903	1267.35828	2.17440485
		1268.58282	2.84673472	1268.58282	2.17461667
		1269.14969	2.84658127	1269.14969	2.17471457
		1270	2.84633866	1270	2.17485993
		1270.37423	2.8462236	1270.37423	2.17492318
		1272.07485	2.84574394	1272.07485	2.17521025
		1272.35828	2.84566239	1272.35828	2.17525763
		1273.58282	2.84530922	1273.58282	2.175461
		1274.14969	2.84514526	1274.14969	2.17555443
		1275	2.84489875	1275	2.17569374
		1275.37423	2.84478162	1275.37423	2.17575431
		1277.07485	2.84429436	1277.07485	2.17602938
		1277.35828	2.84421149	1277.35828	2.17607476
		1278.58282	2.8438525	1278.58282	2.17626956
		1279.14969	2.84368584	1279.14969	2.17635903
		1280	2.84343521	1280	2.17649241
		1280.37423	2.84331589	1280.37423	2.17655038
		1282.07485	2.84282065	1282.07485	2.17681374
		1282.35828	2.84273636	1282.35828	2.17685718
		1283.58282	2.84237125	1283.58282	2.17704361
		1285	2.84194681	1285	2.17725687
		1285.37423	2.84182521	1285.37423	2.17731229
		1287.07485	2.84132161	1287.07485	2.17756428
		1287.35828	2.84123585	1287.35828	2.17760583
		1288.58282	2.84086437	1288.58282	2.17778414
		1290	2.84043246	1290	2.17798806
		1290.37423	2.84030849	1290.37423	2.178041
		1292.07485	2.83979621	1292.07485	2.17828192
		1292.35828	2.83970893	1292.35828	2.17832163
		1293.58282	2.83933083	1293.58282	2.17849203

Final State: j’=5		Final State: j’=3		Final State: j’=2	
E(cm⁻¹)	σ (Å²)	E(cm⁻¹)	σ (Å²)	E(cm⁻¹)	σ (Å²)
		1295	2.8388912	1295	2.17868686
		1295.37423	2.83876476	1295.37423	2.17873739
		1297.07485	2.83824351	1297.07485	2.17896754
		1297.35828	2.83815465	1297.35828	2.17900546
		1298.58282	2.83776973	1298.58282	2.17916816
		1300	2.83732214	1300	2.17935415
		1300.37423	2.83719314	1300.37423	2.1794022
		1302.07485	2.83666268	1302.07485	2.17962199
		1302.35828	2.83657221	1302.35828	2.17965817
		1303.58282	2.83618028	1303.58282	2.17981337
		1305	2.83572452	1305	2.17999073
		1305.37423	2.83559292	1305.37423	2.18003662
		1307.07485	2.83505302	1307.07485	2.18024608
		1307.35828	2.83496089	1307.35828	2.18028056
		1308.58282	2.8345618	1308.58282	2.18042846
		1310	2.83409769	1310	2.18059741
		1310.37423	2.83396342	1310.37423	2.18064106
		1312.07485	2.83341391	1312.07485	2.18084057
		1312.35828	2.83332011	1312.35828	2.1808734
		1313.58282	2.83291371	1313.58282	2.18101416
		1315	2.83244113	1315	2.18117494
		1315.37423	2.83230411	1315.37423	2.1812164
		1317.07485	2.83174486	1317.07485	2.1814062
		1317.35828	2.83164933	1317.35828	2.18143741
		1318.58282	2.83123553	1318.58282	2.18157123
		1320	2.83075438	1320	2.18172401
		1320.37423	2.83061459	1320.37423	2.18176333
		1322.07485	2.83004548	1322.07485	2.18194366
		1322.35828	2.82994824	1322.35828	2.1819733
		1323.58282	2.82952697	1323.58282	2.18210033
		1325	2.82903712	1325	2.18224529
		1325.37423	2.82889452	1325.37423	2.18228253
		1327.07485	2.8283155	1327.07485	2.18245363
		1327.35828	2.82821653	1327.35828	2.18248167
		1328.58282	2.82778774	1328.58282	2.18260208
		1330	2.82728916	1330	2.18273943
		1330.37423	2.82714369	1330.37423	2.18277462
		1332.07485	2.82655477	1332.07485	2.18293665
		1332.35828	2.82645403	1332.35828	2.18296324
		1333.58282	2.82601768	1333.58282	2.18307714
		1335	2.82551036	1335	2.18320699
		1335.37423	2.82536202	1335.37423	2.18324018
		1337.07485	2.82476318	1337.07485	2.18339334
		1337.35828	2.82466069	1337.35828	2.18341845
		1338.58282	2.82421679	1338.58282	2.18352599

Final State: j'=5		Final State: j'=3		Final State: j'=2	
E(cm⁻¹)	σ (Å²)	E(cm⁻¹)	σ (Å²)	E(cm⁻¹)	σ (Å²)
		1340	2.82370074	1340	2.18364853
		1340.37423	2.82354948	1340.37423	2.18367976
		1342.07485	2.82294077	1342.07485	2.18382423
		1342.35828	2.82283654	1342.35828	2.18384789
		1343.58282	2.82238511	1343.58282	2.18394919
		1345	2.82186036	1345	2.18406454
		1345.37423	2.82170617	1345.37423	2.18409382
		1347.07485	2.82108769	1347.07485	2.18422979
		1347.35828	2.82098173	1347.35828	2.18425203
		1348.58282	2.82052282	1348.58282	2.1843472
		1350	2.81998945	1350	2.18445549
		1350.37423	2.81983235	1350.37423	2.18448287
		1352.07485	2.81920418	1352.07485	2.18461047
		1352.35828	2.81909651	1352.35828	2.18463131
		1353.58282	2.81863018	1353.58282	2.18472045
		1355	2.81808827	1355	2.18482179
		1355.37423	2.81792824	1355.37423	2.18484731
		1357.07485	2.81729054	1357.07485	2.18496667
		1357.35828	2.81718116	1357.35828	2.18498613
		1358.58282	2.81670755	1358.58282	2.18506935
		1360	2.81615719	1360	2.18516386
		1360.37423	2.81599425	1360.37423	2.18518751
		1362.07485	2.81534719	1362.07485	2.18529876
		1362.35828	2.81523615	1362.35828	2.18531687
		1363.58282	2.81475534	1363.58282	2.18539424
		1364.14969	2.81453216	1364.14969	2.18542957
		1365	2.81419671	1365	2.185482
		1365.37423	2.81403087	1365.37423	2.18550383
		1367.07485	2.81337466	1367.07485	2.18560707
		1367.35828	2.81326197	1367.35828	2.18562385
		1368.58282	2.81277409	1368.58282	2.18569543
		1369.14969	2.81254768	1369.14969	2.1857281
		1370	2.81220735	1370	2.18577654
		1370.37423	2.81203861	1370.37423	2.18579657
		1372.07485	2.8113735	1372.07485	2.18589189
		1372.35828	2.81125922	1372.35828	2.18590734
		1373.58282	2.81076444	1373.58282	2.18597322
		1374.14969	2.81053483	1374.14969	2.18600326
		1375	2.81018975	1375	2.18604773
		1375.37423	2.81001815	1375.37423	2.18606597
		1377.07485	2.80934439	1377.07485	2.18615347
		1377.35828	2.80922854	1377.35828	2.18616761
		1378.58282	2.80872704	1378.58282	2.18622784
		1379.14969	2.80849433	1379.14969	2.18625526
		1380	2.80814462	1380	2.18629582

Final State: j’=5		Final State: j’=3		Final State: j’=2	
E(cm⁻¹)	σ (Å²)	E(cm⁻¹)	σ (Å²)	E(cm⁻¹)	σ (Å²)
		1380.37423	2.8079702	1380.37423	2.18631227
		1382.07485	2.80728806	1382.07485	2.18639207
		1382.35828	2.8071707	1382.35828	2.18640487
		1383.58282	2.80666265	1383.58282	2.18645948
		1384.14969	2.80642696	1384.14969	2.1864843
		1385	2.80607277	1385	2.18652102
		1385.37423	2.80589552	1385.37423	2.18653569
		1387.07485	2.80520534	1387.07485	2.18660775
		1387.35828	2.8050865	1387.35828	2.18661936
		1388.58282	2.80457216	1388.58282	2.18666832
		1389.14969	2.80433354	1389.14969	2.18669059
		1390	2.80397505	1390	2.18672341
		1390.37423	2.80379502	1390.37423	2.18673639
		1392.07485	2.80309715	1392.07485	2.18680078
		1392.35828	2.80297687	1392.35828	2.18681112
		1393.58282	2.80245643	1393.58282	2.1868546
		1394.14969	2.80221502	1394.14969	2.1868743
		1395	2.80185229	1395	2.18690328
		1395.37423	2.80166958	1395.37423	2.18691445
		1397.07485	2.80096433	1397.07485	2.18697128
		1397.35828	2.80084272	1397.35828	2.18698027
		1398.58282	2.80031642	1398.58282	2.18701829
		1399.14969	2.80007231	1399.14969	2.18703543
		1400	2.79970559	1400	2.1870606
		1400.37423	2.79952019	1400.37423	2.18707002
		1402.07485	2.79880797	1402.07485	2.18711929
		1402.35828	2.79868505	1402.35828	2.18712701
		1403.58282	2.79815315	1403.58282	2.18715953
		1404.14969	2.79790647	1404.14969	2.18717412
		1405	2.79753592	1405	2.18719549
		1405.37423	2.79734791	1405.37423	2.18720317
		1407.07485	2.7966291	1407.07485	2.1872449
		1407.35828	2.79650492	1407.35828	2.18725135
		1408.58282	2.79596771	1408.58282	2.18727839
		1409.14969	2.79571857	1409.14969	2.18729046
		1410	2.79534439	1410	2.187308
		1410.37423	2.79517951	1410.37423	2.18731551
		1412.07485	2.79442882	1412.07485	2.18734812
		1412.35828	2.79430346	1412.35828	2.18735331
		1413.58282	2.7937612	1413.58282	2.18737488
		1414.14969	2.79350975	1414.14969	2.18738441
		1415	2.79313212	1415	2.18739816
		1415.37423	2.79296573	1415.37423	2.18740401
		1417.07485	2.79220828	1417.07485	2.187429
		1417.35828	2.79208182	1417.35828	2.18743292

Final State: j’=5		Final State: j’=3		Final State: j’=2	
E(cm⁻¹)	σ (Å²)	E(cm⁻¹)	σ (Å²)	E(cm⁻¹)	σ (Å²)
		1418.58282	2.79153479	1418.58282	2.18744901
		1419.14969	2.79128118	1419.14969	2.187456
		1420	2.79090031	1420	2.18746594
		1420.37423	2.7907325	1420.37423	2.18747012
		1422.07485	2.78996871	1422.07485	2.18748747
		1422.35828	2.78984121	1422.35828	2.18749014
		1423.58282	2.78928974	1423.58282	2.18750075
		1424.14969	2.78903406	1424.14969	2.1875052
		1425	2.78865018	1425	2.18751133
		1425.37423	2.78848107	1425.37423	2.18751382
		1427.07485	2.78771137	1427.07485	2.18752356
		1427.35828	2.78758289	1427.35828	2.18752494
		1428.58282	2.78702728	1428.58282	2.18753002
		1429.14969	2.78676973	1429.14969	2.18753192
		1430	2.78638305	1430	2.18753422
		1430.37423	2.78621271	1430.37423	2.18753502
		1432.07485	2.78543759	1432.07485	2.18753707
		1432.35828	2.78530824	1432.35828	2.18753716
		1433.58282	2.78474883	1433.58282	2.18753667
		1434.14969	2.78448958	1434.14969	2.187536
		1435	2.78410037	1435	2.18753443
		1435.37423	2.78392896	1435.37423	2.1875335
		1437.07485	2.78314903	1437.07485	2.18752772
		1437.35828	2.7830189	1437.35828	2.18752649
		1438.58282	2.78245629	1438.58282	2.18752027
		1439.14969	2.78219563	1439.14969	2.1875169
		1440	2.78180444	1440	2.18751119
		1440.37423	2.78163223	1440.37423	2.18750842
		1442.07485	2.78084986	1442.07485	2.18749345
		1442.35828	2.78071974	1442.35828	2.18749041
		1443.58282	2.78014404	1443.58282	2.18747291
		1444.14969	2.77985694	1444.14969	2.18747332
		1445	2.77948928	1445	2.18746731
		1445.37423	2.77932263	1445.37423	2.18745886
		1447.07485	2.7785286	1447.07485	2.18744212
		1447.35828	2.77840151	1447.35828	2.18743493
		1448.58282	2.77781585	1448.58282	2.18743352
		1449.14969	2.77751485	1449.14969	2.18742561
		1450	2.7771556	1450	2.18740794
		1450.37423	2.77698714	1450.37423	2.18739745
		1452.07485	2.77618495	1452.07485	2.18737469
		1452.35828	2.77606162	1452.35828	2.18736194
		1453.58282	2.7754862	1453.58282	2.18734494
		1454.14969	2.77522468	1454.14969	2.18733111
		1455	2.77483182	1455	2.18730536

Final State: j’=5		Final State: j’=3		Final State: j’=2	
E(cm⁻¹)	σ (Å²)	E(cm⁻¹)	σ (Å²)	E(cm⁻¹)	σ (Å²)
		1455.37423	2.77462471	1455.37423	2.18728812
		1457.07485	2.77384178	1457.07485	2.18728109
		1457.35828	2.77371647	1457.35828	2.18727025
		1458.58282	2.77314791	1458.58282	2.18723711
		1459.14969	2.7728835	1459.14969	2.18722135
		1460	2.77246041	1460	2.18718869
		1460.37423	2.77230403	1460.37423	2.18720307
		1462.07485	2.77150331	1462.07485	2.18715542
		1462.35828	2.77137177	1462.35828	2.18714624
		1463.58282	2.77079838	1463.58282	2.18710932
		1464.14969	2.77053226	1464.14969	2.18709193
		1465	2.77013309	1465	2.18706302
		1465.37423	2.76996209	1465.37423	2.18703855
		1467.07485	2.76913124	1467.07485	2.18702794
		1467.35828	2.76901634	1467.35828	2.18700277
		1468.58282	2.7684426	1468.58282	2.18695869
		1469.14969	2.76817562	1469.14969	2.18693906
		1470	2.76777479	1470	2.18690906
		1470.37423	2.7675983	1470.37423	2.18689547
		1472.07485	2.76680037	1472.07485	2.1868402
		1472.35828	2.76664245	1472.35828	2.18682587
		1473.58282	2.7660833	1473.58282	2.18678477
		1474.14969	2.76581705	1474.14969	2.18676093
		1475	2.76541704	1475	2.18672597
		1475.37423	2.76524122	1475.37423	2.18671031
		1477.07485	2.76444925	1477.07485	2.18662489
		1477.35828	2.76430462	1477.35828	2.1866234
		1478.58282	2.76370114	1478.58282	2.18658177
		1479.14969	2.76339905	1479.14969	2.18656968
		1480	2.763038	1480	2.18653085
		1480.37423	2.76287663	1480.37423	2.18649796
		1482.07485	2.76204885	1482.07485	2.18643751
		1482.35828	2.76192069	1482.35828	2.18641987
		1483.58282	2.7612746	1483.58282	2.18640151
		1484.14969	2.76105083	1484.14969	2.18635022
		1485	2.76066019	1485	2.18629939
		1485.37423	2.76048764	1485.37423	2.18627525
		1487.07485	2.75962028	1487.07485	2.18621221
		1487.35828	2.75953423	1487.35828	2.18619781
		1488.58282	2.75896295	1488.58282	2.1861229
		1489.14969	2.75870395	1489.14969	2.18608071
		1490	2.75825368	1490	2.18608002
		1490.37423	2.7580979	1490.37423	2.18604465
		1492.07485	2.75730331	1492.07485	2.18594003
		1492.35828	2.75716999	1492.35828	2.18592258

Final State: j’=5		Final State: j’=3		Final State: j’=2	
E(cm⁻¹)	σ (Å²)	E(cm⁻¹)	σ (Å²)	E(cm⁻¹)	σ (Å²)
		1493.58282	2.75657001	1493.58282	2.18586257
		1494.14969	2.75632406	1494.14969	2.18582259
		1495	2.75587076	1495	2.18578614
		1495.37423	2.75576193	1495.37423	2.18574729
		1497.07485	2.75488568	1497.07485	2.18567523
		1497.35828	2.75478698	1497.35828	2.18563663
		1498.58282	2.75419	1498.58282	2.18558668
		1499.14969	2.75394664	1499.14969	2.18553285
		1500	2.75350426	1500	2.18550357
		1500.37423	2.75334479	1500.37423	2.18546884
		1502.07485	2.75253633	1502.07485	2.18535324
		1502.35828	2.75245164	1502.35828	2.18530922
		1503.58282	2.75162458	1503.58282	2.18533734
		1504.14969	2.75155977	1504.14969	2.18522927
		1505.37423	2.75098104	1505.37423	2.18513446
		1507.07485	2.75016092	1507.07485	2.18501783
		1507.35828	2.75000407	1507.35828	2.18503375
		1508.58282	2.74944524	1508.58282	2.18492641
		1509.14969	2.74917558	1509.14969	2.18488605
		1510.37423	2.74859121	1510.37423	2.18479978
		1512.07485	2.74778738	1512.07485	2.18468026
		1512.35828	2.74765312	1512.35828	2.18465886
		1513.58282	2.74707115	1513.58282	2.18456798
		1514.14969	2.74680194	1514.14969	2.18452594
		1515.37423	2.74622047	1515.37423	2.18443434
		1517.07485	2.74541415	1517.07485	2.18430543
		1517.35828	2.74527997	1517.35828	2.18428358
		1518.58282	2.74469974	1518.58282	2.18418859
		1519.14969	2.74443027	1519.14969	2.18414504
		1520.37423	2.74385346	1520.37423	2.18404587
		1522.07485	2.74304825	1522.07485	2.18390898
		1522.35828	2.7429142	1522.35828	2.18388586
		1523.58282	2.74233534	1523.58282	2.18378516
		1524.14969	2.74206749	1524.14969	2.18373808
		1525.37423	2.7414891	1525.37423	2.18363538
		1527.07485	2.74068634	1527.07485	2.18349063
		1527.35828	2.74055266	1527.35828	2.1834662
		1528.58282	2.73997555	1528.58282	2.18335951
		1529.14969	2.73970836	1529.14969	2.18330982
		1530.37423	2.73913144	1530.37423	2.18320154
		1532.07485	2.73833083	1532.07485	2.18304893
		1533.58282	2.73762154	1533.58282	2.18291144
		1534.14969	2.73735507	1534.14969	2.18285922
		1535.37423	2.73677975	1535.37423	2.18274544
		1537.07485	2.73598142	1537.07485	2.18258516

Final State: j'=5		Final State: j'=3		Final State: j'=2	
E(cm⁻¹)	σ (Å²)	E(cm⁻¹)	σ (Å²)	E(cm⁻¹)	σ (Å²)
		1538.58282	2.73527422	1538.58282	2.18244089
		1539.14969	2.73500853	1539.14969	2.18238614
		1540.37423	2.73443493	1540.37423	2.1822669
		1542.07485	2.73363904	1542.07485	2.1820991
		1543.58282	2.73293406	1543.58282	2.18194819
		1544.14969	2.73266922	1544.14969	2.18189094
		1545.37423	2.73209746	1545.37423	2.1817663
		1547.07485	2.73130419	1547.07485	2.18159102
		1548.58282	2.73060159	1548.58282	2.18143351
		1549.14969	2.73033762	1549.14969	2.18137377
		1550.37423	2.72976787	1550.37423	2.1812438
		1552.07485	2.72897739	1552.07485	2.18106116
		1553.58282	2.72827726	1553.58282	2.1808971
		1554.14969	2.72801429	1554.14969	2.18083493
		1555.37423	2.72744657	1555.37423	2.18069969
		1557.07485	2.72665901	1557.07485	2.18050973
		1558.58282	2.7259615	1558.58282	2.18033923
		1559.14969	2.72569951	1559.14969	2.18027464
		1560.37423	2.72513396	1560.37423	2.1801342
		1562.07485	2.72434941	1562.07485	2.17993704
		1563.58282	2.72365462	1563.58282	2.17976019
		1564.14969	2.72339365	1564.14969	2.17969323
		1565.37423	2.72283033	1565.37423	2.17954764
		1567.07485	2.72204887	1567.07485	2.17934337
		1568.58282	2.72135685	1568.58282	2.17916031
		1569.14969	2.72109693	1569.14969	2.179091
		1570.37423	2.72053587	1570.37423	2.17894037
		1572.07485	2.71975757	1572.07485	2.17872916
		1573.58282	2.71906838	1573.58282	2.17853992
		1574.14969	2.71880951	1574.14969	2.1784683
		1575.37423	2.71825074	1575.37423	2.17831272
		1577.07485	2.71747566	1577.07485	2.17809467
		1578.58282	2.71678932	1578.58282	2.1778994
		1579.14969	2.71653424	1579.14969	2.1778265
		1580.37423	2.71597507	1580.37423	2.17766509
		1582.07485	2.71520319	1582.07485	2.17744033
		1583.58282	2.71451969	1583.58282	2.17723915
		1584.14969	2.71426296	1584.14969	2.17716307
		1585.37423	2.71370881	1585.37423	2.17699786
		1588.58282	2.71225945	1588.58282	2.17655959
		1589.14969	2.71200378	1589.14969	2.17648135
		1590.37423	2.71145192	1590.37423	2.1763115
		1593.58282	2.7100085	1593.58282	2.17586118
		1594.14969	2.70975389	1594.14969	2.17578083
		1595.37423	2.70920428	1595.37423	2.17560644

Final State: j'=5		Final State: j'=3		Final State: j'=2	
E(cm⁻¹)	σ (Å²)	E(cm⁻¹)	σ (Å²)	E(cm⁻¹)	σ (Å²)
		1598.58282	2.70776669	1598.58282	2.17514438
		1599.14969	2.70751308	1599.14969	2.17506197
		1600.37423	2.70696565	1600.37423	2.17488318
		1603.58282	2.70553374	1603.58282	2.17440968
		1604.14969	2.70528113	1604.14969	2.17432528
		1605.37423	2.70473582	1605.37423	2.1741422
		1608.58282	2.70330939	1608.58282	2.17365761
		1609.14969	2.70305771	1609.14969	2.17357128
		1610.37423	2.70251445	1610.37423	2.17338404
		1613.58282	2.70109321	1613.58282	2.17288872
		1614.14969	2.70084246	1614.14969	2.17280051
		1615.37423	2.70030112	1615.37423	2.17260924
		1618.58282	2.69888482	1618.58282	2.17210354
		1619.14969	2.69863492	1619.14969	2.17201352
		1620.37423	2.69809541	1620.37423	2.17181836
		1623.58282	2.69668371	1623.58282	2.17130265
		1624.14969	2.6964346	1624.14969	2.17121089
		1625.37423	2.69589674	1625.37423	2.171012
		1628.58282	2.69448932	1628.58282	2.17048665
		1629.14969	2.69424093	1629.14969	2.17039321
		1630.37423	2.69370464	1630.37423	2.17019072
		1633.58282	2.69230107	1633.58282	2.16965612
		1634.14969	2.69205334	1634.14969	2.16956108
		1635.37423	2.69151844	1635.37423	2.16935515
		1638.58282	2.6901183	1638.58282	2.1688117
		1639.14969	2.68987114	1639.14969	2.16871512
		1640.37423	2.68933747	1640.37423	2.1685059
		1643.58282	2.68794031	1643.58282	2.16795401
		1644.14969	2.68769368	1644.14969	2.16785593
		1645.37423	2.68716101	1645.37423	2.16764361
		1648.58282	2.68576637	1648.58282	2.16708368
		1649.14969	2.68552012	1649.14969	2.16698424
		1650.37423	2.68498831	1650.37423	2.16676891
		1653.58282	2.68359567	1653.58282	2.16620137
		1654.14969	2.68334975	1654.14969	2.1661006
		1655.37423	2.68281859	1655.37423	2.16588246
		1658.58282	2.68142743	1658.58282	2.16530772
		1659.14969	2.68118172	1659.14969	2.16520571
		1660.37423	2.68065102	1660.37423	2.1649849
		1663.58282	2.67926075	1663.58282	2.16440337
		1664.14969	2.67901517	1664.14969	2.1643002
		1665.37423	2.67848469	1665.37423	2.16407689
		1668.58282	2.67709479	1668.58282	2.16348859
		1670.37423	2.67631873	1670.37423	2.16315909
		1673.58282	2.67492857	1673.58282	2.16256527

Final State: j’=5		Final State: j’=3		Final State: j’=2	
E(cm⁻¹)	σ (Å²)	E(cm⁻¹)	σ (Å²)	E(cm⁻¹)	σ (Å²)
		1675.37423	2.67415222	1675.37423	2.16223216
		1678.58282	2.67276121	1678.58282	2.16163282
		1680.37423	2.67198424	1680.37423	2.16129675
		1683.58282	2.67059176	1683.58282	2.16069231
		1685.37423	2.66981378	1685.37423	2.1603535
		1688.58282	2.66841923	1688.58282	2.15974438
		1690.37423	2.66763993	1690.37423	2.15940308
		1693.58282	2.66624265	1693.58282	2.15878968
		1695.37423	2.66546167	1695.37423	2.15844611
		1698.58282	2.66406107	1698.58282	2.15782885
		1700.37423	2.66327803	1700.37423	2.15748322
		1703.58282	2.66187346	1703.58282	2.15686249
		1705.37423	2.66108803	1705.37423	2.15651504
		1708.58282	2.65967886	1708.58282	2.15589124
		1710.37423	2.6588907	1710.37423	2.15554218
		1713.58282	2.65747629	1713.58282	2.15491568
		1715.37423	2.65668502	1715.37423	2.15456522
		1718.58282	2.65526477	1718.58282	2.1539364
		1720.37423	2.65447006	1720.37423	2.15358476
		1723.58282	2.65304335	1723.58282	2.15295398
		1725.37423	2.65224486	1725.37423	2.15260133
		1728.58282	2.65081106	1728.58282	2.15196897
		1730.37423	2.65000845	1730.37423	2.15161553
		1733.58282	2.64856696	1733.58282	2.15098192
		1735.37423	2.64775988	1735.37423	2.15062789
		1738.58282	2.64630998	1738.58282	2.14999342
		1740.37423	2.64549792	1740.37423	2.14963904
		1743.58282	2.64403735	1743.58282	2.14900471
		1745.37423	2.64323151	1745.37423	2.14864562
		1748.58282	2.64175357	1748.58282	2.14801449
		1750.37423	2.64093549	1750.37423	2.14765794
		1753.58282	2.63946598	1753.58282	2.14701897
		1755.37423	2.63863106	1755.37423	2.14666706
		1758.58282	2.63714482	1758.58282	2.14603121
		1760.37423	2.63630999	1760.37423	2.1456771
		1763.58282	2.63481213	1763.58282	2.14504203
		1765.37423	2.63397194	1765.37423	2.14468806
		1768.58282	2.6324625	1768.58282	2.14405367
		1770.37423	2.63161566	1770.37423	2.14370036
		1773.58282	2.6300884	1773.58282	2.14306834
		1775.37423	2.62923997	1775.37423	2.14271456
		1780.37423	2.62683689	1780.37423	2.14173434
		1785.37423	2.62444326	1785.37423	2.14074541
		1790.37423	2.62200594	1790.37423	2.13976909
		1795.37423	2.61954575	1795.37423	2.13879068

Final State: j'=5		Final State: j'=3		Final State: j'=2	
E(cm⁻¹)	σ (Å²)	E(cm⁻¹)	σ (Å²)	E(cm⁻¹)	σ (Å²)
		1800.37423	2.61707364	1800.37423	2.13781867
		1805.37423	2.61460255	1805.37423	2.1368412
		1810.37423	2.61207964	1810.37423	2.13587573
		1815.37423	2.60954482	1815.37423	2.13491105
		1820.37423	2.6069861	1820.37423	2.13394773
		1825.37423	2.60441139	1825.37423	2.1329924
		1830.37423	2.60181245	1830.37423	2.13203872
		1835.37423	2.59919211	1835.37423	2.13108881
		1840.37423	2.59655034	1840.37423	2.13014276
		1845.37423	2.5938873	1845.37423	2.12920057
		1850.37423	2.59120317	1850.37423	2.12826223
		1855.37423	2.58849817	1855.37423	2.12732772
		1860.37423	2.5857726	1860.37423	2.12639701
		1865.37423	2.58302679	1865.37423	2.12547006
		1870.37423	2.58026112	1870.37423	2.12454678
		1875.37423	2.577476	1875.37423	2.1236271
		1880.37423	2.57467191	1880.37423	2.12271091
		1885.37423	2.57184936	1885.37423	2.12179814
		1890.37423	2.56900889	1890.37423	2.12088865
		1895.37423	2.5661511	1895.37423	2.11998232
		1900.37423	2.56327661	1900.37423	2.11907902
		1905.37423	2.56038606	1905.37423	2.11817862
		1910.37423	2.55748012	1910.37423	2.11728095

		Initial rotational state: j=4	
**Final State: j’=1**		**Final State: j’=0**	
E(cm⁻¹)	σ (Å²)	E(cm⁻¹)	σ (Å²)
273.582818	1668.56988	273.582818	866.893718
273.582819	1181.00696	273.582819	613.592812
273.58282	965.291399	273.58282	501.524996
273.582821	836.863181	273.582821	434.805406
273.582822	749.329955	273.582822	389.331806
273.582823	684.796696	273.582823	355.807215
273.582824	634.704184	273.582824	329.784887
273.582825	594.376245	273.582825	308.835427
273.582826	561.013706	273.582826	291.504599
273.582827	532.825208	273.582827	276.86175
273.582828	508.604073	273.582828	264.279986
273.582829	487.503894	273.582829	253.319584
273.58283	468.911055	273.58283	243.661757
273.582831	452.368425	273.582831	235.069018
273.582832	437.527568	273.582832	227.360351
273.582833	424.117586	273.582833	220.395019
273.582834	411.92429	273.582834	214.06175
273.582835	400.77581	273.582835	208.271256
273.582836	390.532396	273.582836	202.950933
273.582837	381.079129	273.582837	198.041082
273.582838	372.320478	273.582838	193.492074
273.582839	364.176344	273.582839	189.262296
273.58284	356.578948	273.58284	185.316537
273.582841	349.470514	273.582841	181.624783
273.582842	342.801477	273.582842	178.161285
273.582843	336.528984	273.582843	174.90378
273.582844	330.615855	273.582844	171.832954
273.582845	325.029554	273.582845	168.931906
273.582846	319.741552	273.582846	166.185812
273.582847	314.726657	273.582847	163.581588
273.582848	309.962567	273.582848	161.107647
273.582849	305.429432	273.582849	158.753676
273.58285	301.109542	273.58285	156.510478
273.582851	296.987017	273.582851	154.3698
273.582852	293.047587	273.582852	152.324231
273.582853	289.278371	273.582853	150.36708
273.582854	285.667745	273.582854	148.492308
273.582855	282.205125	273.582855	146.694416
273.582856	278.88092	273.582856	144.968421
273.582857	275.686364	273.582857	143.309773
273.582858	272.613457	273.582858	141.714313
273.582859	269.654879	273.582859	140.178239
273.58286	266.803894	273.58286	138.698051
273.582861	264.054318	273.582861	137.270539

Final State: j’=1		Final State: j’=0	
E(cm⁻¹)	σ (Å²)	E(cm⁻¹)	σ (Å²)
273.582862	261.400404	273.582862	135.892716
273.582863	258.836976	273.582863	134.561892
273.582864	256.359107	273.582864	133.275511
273.582865	253.962294	273.582865	132.031231
273.582866	251.642359	273.582866	130.826883
273.582867	249.395395	273.582867	129.660436
273.582868	247.217792	273.582868	128.530017
273.582869	245.106164	273.582869	127.433865
273.58287	243.057367	273.58287	126.370348
273.582871	241.068451	273.582871	125.337934
273.582872	239.136655	273.582872	124.335187
273.582873	237.25941	273.582873	123.360775
273.582874	235.434281	273.582874	122.413431
273.582875	233.659002	273.582875	121.491979
273.582876	231.931427	273.582876	120.595304
273.582877	230.249548	273.582877	119.722363
273.582878	228.611467	273.582878	118.87217
273.582879	227.01538	273.582879	118.043788
273.58288	225.459624	273.58288	117.236353
273.582881	223.942582	273.582881	116.449026
273.582882	222.462755	273.582882	115.681026
273.582883	221.018715	273.582883	114.931614
273.582884	219.609109	273.582884	114.200086
273.582885	218.232648	273.582885	113.485772
273.582886	216.888118	273.582886	112.788041
273.582887	215.574377	273.582887	112.106302
273.582888	214.290296	273.582888	111.439967
273.582889	213.034852	273.582889	110.788505
273.58289	211.80705	273.58289	110.151399
273.582891	210.605938	273.582891	109.528155
273.582892	209.430619	273.582892	108.918306
273.582893	208.280217	273.582893	108.321398
273.582894	207.153934	273.582894	107.737015
273.582895	206.050971	273.582895	107.164745
273.582896	204.970587	273.582896	106.604199
273.582897	203.912062	273.582897	106.055007
273.582898	202.874712	273.582898	105.516811
273.582899	201.857881	273.582899	104.989272
273.5829	200.860938	273.5829	104.472061
273.582901	199.883297	273.582901	103.964874
273.582902	198.924363	273.582902	103.467402
273.582903	197.983595	273.582903	102.979364
273.582904	197.060458	273.582904	102.500482
273.582905	196.15445	273.582905	102.030496
273.582906	195.265079	273.582906	101.56915

Final State: j'=1		Final State: j'=0	
E(cm⁻¹)	σ (Å²)	E(cm⁻¹)	σ (Å²)
273.582907	194.391872	273.582907	101.116198
273.582908	193.534388	273.582908	100.67141
273.582909	192.692178	273.582909	100.234556
273.58291	191.864837	273.58291	99.8054217
273.582911	191.051962	273.582911	99.3838006
273.582912	190.25316	273.582912	98.969488
273.582913	189.468062	273.582913	98.5622913
273.582914	188.69631	273.582914	98.162025
273.582915	187.937559	273.582915	97.7685107
273.582916	187.191473	273.582916	97.381573
273.582917	186.457727	273.582917	97.0010433
273.583417	122.805532	273.583417	64.152466
273.583817	129.972414	273.583817	68.0093048
273.583917	133.06864	273.583917	69.6490488
273.584417	152.568542	273.584417	79.9234943
273.584817	171.307089	273.584817	89.7545831
273.584917	176.281072	273.584917	92.3588932
273.585417	202.210669	273.585417	105.901012
273.585817	223.404449	273.585817	116.918845
273.585917	228.643381	273.585917	119.634127
273.586417	253.707028	273.586417	132.558658
273.586817	271.451856	273.586817	141.616561
273.586917	275.44265	273.586917	143.637422
273.587417	292.106543	273.587417	151.959859
273.587817	300.967002	273.587817	156.219431
273.587917	302.516944	273.587917	156.930998
273.588417	306.279531	273.588417	158.393339
273.588817	304.754624	273.588817	157.209205
273.588917	303.800675	273.588917	156.620751
273.589417	296.078829	273.589417	152.198318
273.589817	287.002788	273.589817	147.238828
273.589917	284.419938	273.589917	145.850015
273.590417	270.16624	273.590417	138.291255
273.590817	257.70168	273.590817	131.782419
273.590917	254.506034	273.590917	130.125428
273.591417	238.375826	273.591417	121.814212
273.591817	225.600937	273.591817	115.279212
273.591917	222.458875	273.591917	113.676839
273.592417	207.199694	273.592417	105.915164
273.592817	195.643232	273.592817	100.053455
273.592917	192.855046	273.592917	98.6404485
273.593417	179.551597	273.593417	91.9040546
273.593817	169.683169	273.593817	86.9084135
273.593917	167.322444	273.593917	85.7133525
273.594417	156.14349	273.594417	80.0540312

Final State: j’=1		Final State: j’=0	
E(cm⁻¹)	σ (Å²)	E(cm⁻¹)	σ (Å²)
273.594817	147.920546	273.594817	75.887779
273.594917	145.961874	273.594917	74.894318
273.595417	136.698799	273.595417	70.1976936
273.595817	129.899949	273.595817	66.7467669
273.595917	128.279574	273.595917	65.9233357
273.596417	120.625962	273.596417	62.0331123
273.596817	115.0006	273.596817	59.1714676
273.596917	113.659258	273.596917	58.4884565
273.597417	107.310192	273.597417	55.254832
273.597817	102.632988	273.597817	52.8704955
273.597917	101.515614	273.597917	52.3005425
273.598417	96.2187632	273.598417	49.5960834
273.598817	92.3022303	273.598817	47.5958035
273.598917	91.3649265	273.598917	47.116876
273.599417	86.9112483	273.599417	44.8392368
273.599817	83.6075113	273.599817	43.1480469
273.599917	82.8154125	273.599917	42.7425833
273.600417	79.0407629	273.600417	40.8088611
273.600817	76.2319008	273.600817	39.3688871
273.600917	75.5572476	273.600917	39.0230615
273.601417	72.3343987	273.601417	37.3701508
273.601817	69.9272535	273.601817	36.1342638
273.601917	69.3477799	273.601917	35.836979
273.602417	66.5764426	273.602417	34.4130804
273.602817	64.499255	273.602817	33.3455348
273.603817	59.7937338	273.603817	30.9254679
273.604817	55.6883408	273.604817	28.8123583
273.605817	52.0854254	273.605817	26.9563871
273.606817	48.9081738	273.606817	25.3176164
273.607817	46.0910722	273.607817	23.8641718
273.608817	43.5825847	273.608817	22.5689979
273.609817	41.3403295	273.609817	21.4105238
273.610817	39.3279178	273.610817	20.3704383
273.611817	37.5159613	273.611817	19.4330346
273.612817	35.8796879	273.612817	18.5864953
273.613817	34.3986061	273.613817	17.819495
273.614817	33.0535529	273.614817	17.1230423
273.615817	31.8294479	273.615817	16.4888925
273.616817	30.713681	273.616817	15.9102393
273.617817	29.6941931	273.617817	15.3813158
273.618817	28.7617401	273.618817	14.8972576
273.619817	27.906733	273.619817	14.4534833
273.620817	27.1219518	273.620817	14.0459037
273.621817	26.4012167	273.621817	13.6710393
273.622817	25.7376765	273.622817	13.3256323

Final State: j'=1		Final State: j'=0	
E(cm⁻¹)	σ (Å²)	E(cm⁻¹)	σ (Å²)
273.623817	25.1266435	273.623817	13.0075389
273.624817	24.5630147	273.624817	12.7139518
273.625817	24.0431287	273.625817	12.4427464
273.626817	23.5623644	273.626817	12.1917415
273.627817	23.1176615	273.627817	11.9595351
273.628817	22.7063414	273.628817	11.744126
273.629817	22.3247553	273.629817	11.544408
273.630817	21.9710561	273.630817	11.3589475
273.631817	21.6421857	273.631817	11.1863155
273.632817	21.3366539	273.632817	11.0254064
273.633817	21.0518979	273.633817	10.8751472
273.634817	20.7861324	273.634817	10.7349477
273.635817	20.5380251	273.635817	10.6038485
273.636817	20.3057408	273.636817	10.4807458
273.637817	20.0875817	273.637817	10.3652314
273.638817	19.8830543	273.638817	10.2562301
273.639817	19.6901135	273.639817	10.1535992
273.640817	19.5081373	273.640817	10.0562674
273.641817	19.3358605	273.641817	9.96411801
273.642817	19.172276	273.642817	9.87624233
273.643817	19.0163758	273.643817	9.79261803
273.644817	18.8675898	273.644817	9.71257849
273.645817	18.7251953	273.645817	9.63556402
273.646817	18.5877996	273.646817	9.56163994
273.647817	18.4558862	273.647817	9.49000661
273.648817	18.3280955	273.648817	9.42085955
273.649817	18.2043327	273.649817	9.35355244
273.650817	18.0835737	273.650817	9.28810987
273.651817	17.9659991	273.651817	9.22412537
273.652817	17.8507576	273.652817	9.16146501
273.653817	17.737866	273.653817	9.09987225
273.654817	17.6267028	273.654817	9.03924707
273.655817	17.5174348	273.655817	8.97969456
273.656817	17.4092616	273.656817	8.92070693
273.657817	17.3021469	273.657817	8.8624416
273.658817	17.196195	273.658817	8.80469357
273.659817	17.0909302	273.659817	8.74731296
273.660817	16.9866559	273.660817	8.6903025
273.661817	16.8826467	273.661817	8.63382198
273.662817	16.779305	273.662817	8.57757119
273.663817	16.6763314	273.663817	8.52148775
273.664817	16.5738715	273.664817	8.46576365
273.665817	16.4716001	273.665817	8.4101763
273.666817	16.369889	273.666817	8.35482793
273.667817	16.2683803	273.667817	8.29969367

Final State: j’=1		Final State: j’=0	
E(cm⁻¹)	σ (Å²)	E(cm⁻¹)	σ (Å²)
273.668817	16.1672029	273.668817	8.24483118
273.669817	16.0663416	273.669817	8.1900985
273.670817	15.9659258	273.670817	8.13553971
273.671817	15.865952	273.671817	8.08127307
273.672817	15.7661148	273.672817	8.02731384
273.673817	15.6668315	273.673817	7.97354456
273.674817	15.5683004	273.674817	7.92003482
273.675817	15.4700387	273.675817	7.86687023
273.676817	15.3724309	273.676817	7.81401909
273.677817	15.2757798	273.677817	7.76151649
273.678817	15.1796579	273.678817	7.70943087
273.679817	15.0842486	273.679817	7.6576936
273.680817	14.9896354	273.680817	7.60641028
273.681817	14.8959134	273.681817	7.55563651
273.682817	14.803306	273.682817	7.50523766
273.782817	40.6467938	273.782817	17.6281223
273.882817	11.7212698	273.882817	6.12306843
273.982817	8.19835271	273.982817	4.23720326
274	7.86102313	274	4.05614263
274.074845	6.81408031	274.074845	3.49139017
274.082817	6.73650174	274.082817	3.44904385
274.14969	6.31510309	274.14969	3.21177943
274.182817	6.2473955	274.182817	3.16568772
274.282817	6.34533118	274.282817	3.1739463
274.382817	6.2167513	274.382817	3.05840428
274.482817	6.00163294	274.482817	2.8105258
274.582817	7.85619899	274.582817	3.29836044
274.682817	5.35239131	274.682817	2.77086535
274.782817	4.71001068	274.782817	2.45788915
274.882817	4.36390199	274.882817	2.26952383
274.982817	4.11424522	274.982817	2.13025723
275	4.07694923	275	2.10936871
275.082817	3.91246506	275.082817	2.01736724
275.182817	3.73777296	275.182817	1.92006457
275.282817	3.58056945	275.282817	1.83281091
275.358282	3.47075448	275.358282	1.7718877
275.382817	3.43644246	275.382817	1.75283795
275.482817	3.3033097	275.482817	1.67864534
275.582817	3.18024678	275.582817	1.60916632
275.682817	3.06753157	275.682817	1.5437502
275.782817	2.96671648	275.782817	1.48182137
275.882817	2.88391993	275.882817	1.42336376
275.982817	2.86340808	275.982817	1.37301739
276	2.88432146	276	1.36723896
276.074845	3.12051935	276.074845	1.39189658

Final State: j'=1		Final State: j'=0	
E(cm⁻¹)	σ (Å²)	E(cm⁻¹)	σ (Å²)
276.082817	3.15797569	276.082817	1.40461574
276.14969	3.59455291	276.14969	1.59566022
276.182817	3.89870367	276.182817	1.71657058
276.282817	3.79002325	276.282817	1.7441986
276.382817	3.15159741	276.382817	1.5528044
276.482817	3.25327245	276.482817	1.63921951
276.582817	3.3808594	276.582817	1.70796163
276.682817	3.39234266	276.682817	1.71248084
276.782817	3.28534918	276.782817	1.65952214
276.882817	3.12648369	276.882817	1.58171562
276.982817	2.96549189	276.982817	1.50237478
277.074845	2.83213942	277.074845	1.43608153
277.082817	2.82139269	277.082817	1.43071755
277.182817	2.69745966	277.182817	1.36854597
277.282817	2.5918441	277.282817	1.31513062
277.358282	2.52247743	277.358282	1.27982331
277.382817	2.50161133	277.382817	1.26917119
277.482817	2.42421745	277.482817	1.22945745
277.582817	2.35761389	277.582817	1.19504578
277.682817	2.30029661	277.682817	1.16518487
277.782817	2.25114789	277.782817	1.13933939
277.882817	2.20923489	277.882817	1.11704882
277.982817	2.1738033	277.982817	1.09793557
278	2.16832723	278	1.09494815
278.074845	2.14630588	278.074845	1.08284758
278.082817	2.14411865	278.082817	1.08164229
278.14969	2.1270853	278.14969	1.07212548
278.182817	2.11939355	278.182817	1.06777433
278.282817	2.09866418	278.282817	1.05589661
278.382817	2.08095046	278.382817	1.045526
278.482817	2.06517268	278.482817	1.03614601
278.582817	2.05025227	278.582817	1.02725173
279.14969	1.94810036	279.14969	0.969339801
279.358282	1.89918361	279.358282	0.941998469
279.582817	1.84155677	279.582817	0.906007781
280	1.83798916	280	0.882484241
280.074845	1.86916885	280.074845	0.885773469
280.14969	1.94504601	280.14969	0.927995319
280.582817	1.96938494	280.582817	0.921712438
281.358282	1.73739697	281.358282	0.856213999
281.582817	1.64261823	281.582817	0.812296437
282	1.54318286	282	0.763900348
282.074845	1.52963581	282.074845	0.757159368
282.14969	1.51683418	282.14969	0.750765444
282.358282	1.48425092	282.358282	0.73439417

Final State: j’=1		Final State: j’=0	
E(cm⁻¹)	σ (Å²)	E(cm⁻¹)	σ (Å²)
282.582817	1.45302355	282.582817	0.718594262
283.358282	1.36678563	283.358282	0.674225755
283.582817	1.34805232	283.582817	0.664231751
284	1.32414114	284	0.650402852
284.14969	1.32035175	284.14969	0.647481248
284.582817	1.32946437	284.582817	0.647458474
285	1.35952996	285	0.656556997
285.358282	1.36569552	285.358282	0.656878738
285.582817	1.34970526	285.582817	0.649116004
286	1.2976141	286	0.625443036
286.074845	1.287488	286.074845	0.620854219
286.14969	1.2774756	286.14969	0.616308512
286.582817	1.22443665	286.582817	0.591965491
287.074845	1.1765596	287.074845	0.569397723
287.358282	1.15489859	287.358282	0.558819527
287.582817	1.14137782	287.582817	0.551872935
288	1.14041548	288	0.547960475
288.074845	1.15420154	288.074845	0.552455857
288.14969	1.18701682	288.14969	0.564169134
288.582817	1.44373854	288.582817	0.587249613
289.14969	1.0820209	289.14969	0.520368699
289.358282	1.05723313	289.358282	0.509302941
289.582817	1.04447177	289.582817	0.503078957
290	1.02779899	290	0.494512511
290.074845	1.0250954	290.074845	0.493106428
290.14969	1.02242246	290.14969	0.491716774
290.582817	1.0071316	290.582817	0.483820749
291.358282	0.979307277	291.358282	0.469776181
291.582817	0.971204313	291.582817	0.465744563
292	0.956335681	292	0.458380486
292.074845	0.953709247	292.074845	0.457082339
292.14969	0.951098912	292.14969	0.455792504
292.358282	0.943916954	292.358282	0.452244275
292.582817	0.93635816	292.582817	0.448509044
293.358282	0.91185299	293.358282	0.436347432
293.582817	0.905274968	293.582817	0.433058377
294	0.893754396	294	0.427250054
294.074845	0.891794764	294.074845	0.426252873
294.14969	0.88987124	294.14969	0.425270581
294.582817	0.879587953	294.582817	0.419922754
295	0.871693127	295	0.41552831
295.358282	0.867926111	295.358282	0.412829594
295.582817	0.868206106	295.582817	0.412056558
296	0.878380544	296	0.41391959
296.074845	0.881760157	296.074845	0.414777652

Final State: j'=1		Final State: j'=0	
E(cm⁻¹)	σ (Å²)	E(cm⁻¹)	σ (Å²)
296.14969	0.885449663	296.14969	0.415736811
296.582817	0.899326063	296.582817	0.418662034
297.074845	0.876982585	297.074845	0.409400228
297.358282	0.859215532	297.358282	0.402515183
297.582817	0.846913614	297.582817	0.397653455
298	0.828534324	298	0.390136467
298.074845	0.825728755	298.074845	0.388956501
298.14969	0.823041256	298.14969	0.387817807
298.582817	0.80932513	298.582817	0.381870943
299.14969	0.794527206	299.14969	0.37521715
299.358282	0.789663862	299.358282	0.372983346
299.582817	0.784686645	299.582817	0.370676364
300	0.776017184	300	0.366613744
300.074845	0.774530048	300.074845	0.365911815
300.14969	0.773062502	300.14969	0.365216966
300.582817	0.764903191	300.582817	0.361333908
301.358282	0.751556958	301.358282	0.354892138
301.582817	0.747968151	301.582817	0.353139662
302.074845	0.740534839	302.074845	0.349471116
302.14969	0.739457204	302.14969	0.348934101
302.358282	0.736528374	302.358282	0.347466959
302.582817	0.733504623	302.582817	0.345936003
303.358282	0.724124902	303.358282	0.341058662
303.582817	0.721710313	303.582817	0.339759057
304	0.717514007	304	0.33745523
304.074845	0.71679156	304.074845	0.337053962
304.14969	0.71607547	304.14969	0.336655463
304.582817	0.711988414	304.582817	0.334381401
305	0.707980202	305	0.332192088
305.358282	0.704362042	305.358282	0.330274696
305.582817	0.701995582	305.582817	0.329050872
306	0.697418868	306	0.326739386
306.074845	0.696578204	306.074845	0.326320994
306.14969	0.695732991	306.14969	0.325902056
306.582817	0.690782492	306.582817	0.323474364
307.074845	0.685129634	307.074845	0.320738832
307.358282	0.681904377	307.358282	0.31918705
307.582817	0.679378791	307.582817	0.317973845
308	0.674772198	308	0.315763497
308.074845	0.673959685	308.074845	0.315373285
308.14969	0.673150504	308.14969	0.314985014
308.582817	0.668556771	308.582817	0.31277763
309.14969	0.662775278	309.14969	0.309991567
309.358282	0.660714867	309.358282	0.308995713
309.582817	0.658537102	309.582817	0.307941088

Final State: j'=1		Final State: j'=0	
E(cm⁻¹)	σ (Å²)	E(cm⁻¹)	σ (Å²)
310	0.654599946	310	0.306028678
310.074845	0.65390852	310.074845	0.305691923
310.14969	0.653221704	310.14969	0.3053571
310.582817	0.649334506	310.582817	0.303457105
311.358282	0.642731482	311.358282	0.300206706
311.582817	0.640902626	311.582817	0.299300591
312	0.637597493	312	0.297656451
312.074845	0.637017059	312.074845	0.29736674
312.14969	0.636440085	312.14969	0.297078538
312.358282	0.634851185	312.358282	0.296283589
312.582817	0.633171603	312.582817	0.295440989
313.358282	0.627587206	313.358282	0.292625455
313.582817	0.626027672	313.582817	0.291835952
314	0.623190066	314	0.290396675
314.074845	0.622688323	314.074845	0.290142045
314.14969	0.622189396	314.14969	0.28988851
314.582817	0.61934111	314.582817	0.288441821
315	0.616658769	315	0.28707802
315.358282	0.614398111	315.358282	0.285929546
315.582817	0.613000325	315.582817	0.285219845
316	0.610439323	316	0.283921027
316.074845	0.609984783	316.074845	0.283690684
316.14969	0.609532315	316.14969	0.283461131
316.582817	0.606937578	316.582817	0.2821484
317.074845	0.604050936	317.074845	0.280688634
317.358282	0.602414273	317.358282	0.279862778
317.582817	0.601133072	317.582817	0.279216352
318	0.598785647	318	0.278033111
318.074845	0.598369028	318.074845	0.277823304
318.14969	0.597954202	318.14969	0.277614361
318.582817	0.595580761	318.582817	0.276419045
319.14969	0.592547928	319.14969	0.274892166
319.358282	0.591452977	319.358282	0.274340788
319.582817	0.590286579	319.582817	0.273753663
320	0.588154536	320	0.272679826
320.074845	0.587776845	320.074845	0.272489503
320.14969	0.587400497	320.14969	0.272299939
320.582817	0.585250357	320.582817	0.271216086
321.358282	0.58152308	321.358282	0.26933407
321.582817	0.580471475	321.582817	0.268802662
322	0.578551467	322	0.267830441
322.074845	0.578211095	322.074845	0.267658088
322.14969	0.577872516	322.14969	0.267486442
322.358282	0.576935317	322.358282	0.267011383
322.582817	0.575938537	322.582817	0.266505557

Final State: j’=1		Final State: j’=0	
E(cm⁻¹)	σ (Å²)	E(cm⁻¹)	σ (Å²)
323.358282	0.572583858	323.358282	0.264800556
323.582817	0.57163775	323.582817	0.264318771
324	0.56990866	324	0.263437299
324.074845	0.569602231	324.074845	0.263280974
324.14969	0.56929713	324.14969	0.263125311
325	0.565909851	325	0.261393932
325.358282	0.564524726	325.358282	0.260684679
325.582817	0.563668603	325.582817	0.260246155
326	0.562102778	326	0.25944302
326.074845	0.56182517	326.074845	0.25930059
326.14969	0.56154842	326.14969	0.259158521
327.074845	0.558207045	327.074845	0.257441564
327.358282	0.557211266	327.358282	0.256929339
327.582817	0.556431313	327.582817	0.256528101
328	0.555002274	328	0.255792643
328.074845	0.554749188	328.074845	0.255661985
328.14969	0.554496354	328.14969	0.255531831
328.582817	0.553050222	328.582817	0.254786644
329.14969	0.551198352	329.14969	0.253831933
329.582817	0.549813034	329.582817	0.253117289
330	0.548502727	330	0.252440895
330.074845	0.548269988	330.074845	0.252320735
330.14969	0.548037796	330.14969	0.252200983
331.358282	0.54439007	331.358282	0.250315964
331.582817	0.54373166	331.582817	0.2499755
332	0.542524579	332	0.249350894
332.074845	0.542310138	332.074845	0.24923997
332.14969	0.542096454	332.14969	0.24912931
332.358282	0.541503746	332.358282	0.248822539
333.358282	0.538729247	333.358282	0.247385446
333.582817	0.538120982	333.582817	0.247069967
334	0.537003874	334	0.2464908
334.074845	0.536805527	334.074845	0.246387778
334.14969	0.536607668	334.14969	0.246285059
335	0.534395676	335	0.245136842
335.358282	0.533483122	335.358282	0.244663058
335.582817	0.532917239	335.582817	0.244369027
336	0.531876639	336	0.243828454
336.074845	0.531691537	336.074845	0.243732254
336.14969	0.531506737	336.14969	0.243636255
337.074845	0.52925938	337.074845	0.24246795
337.358282	0.528582965	337.358282	0.242116449
337.582817	0.528051199	337.582817	0.241839763
338	0.527071442	338	0.241330425
338.074845	0.526896814	338.074845	0.241239645

Final State: j’=1		Final State: j’=0	
E(cm⁻¹)	σ (Å²)	E(cm⁻¹)	σ (Å²)
338.14969	0.526722416	338.14969	0.241149016
338.582817	0.525720195	338.582817	0.240628022
339.14969	0.524422305	339.14969	0.239953951
339.358282	0.523948588	339.358282	0.239707901
339.582817	0.523440588	339.582817	0.239444194
340	0.522501602	340	0.238956924
340.074845	0.522333841	340.074845	0.238869913
340.14969	0.522166043	340.14969	0.238782917
341.358282	0.519473874	341.358282	0.237388701
341.582817	0.518975106	341.582817	0.237130887
342	0.518047581	342	0.236651938
342.074845	0.517880822	342.074845	0.23656602
342.14969	0.51771425	342.14969	0.236479944
342.358282	0.517248661	342.358282	0.236239935
343.358282	0.51499173	343.358282	0.235078843
343.582817	0.514475842	343.582817	0.2348142
344	0.513504491	344	0.234316297
344.074845	0.51332804	344.074845	0.234225926
344.14969	0.513150795	344.14969	0.234135237
345	0.511072349	345	0.23307204
345.358282	0.510148086	345.358282	0.23259944
345.582817	0.509548674	345.582817	0.232292503
346	0.508383253	346	0.231694381
346.074845	0.508165434	346.074845	0.231582422
346.14969	0.507945063	346.14969	0.231468624
347.074845	0.504880535	347.074845	0.229864945
347.358282	0.503756955	347.358282	0.229257217
347.582817	0.502770167	347.582817	0.228709245
348	0.500610646	348	0.227441446
348.074845	0.500163717	348.074845	0.227163057
348.14969	0.499694406	348.14969	0.226862916
348.582817	0.496446974	348.582817	0.224454212
349.14969	0.495110618	349.14969	0.218478443
349.358282	0.499312116	349.358282	0.218583158
349.582817	0.501616383	349.582817	0.218315882
350	0.513739369	350	0.222999532
350.074845	0.514303611	350.074845	0.224511052
350.14969	0.513958391	350.14969	0.225628696
351.358282	0.506923111	351.358282	0.219293085
351.582817	0.512511625	351.582817	0.223512404
352	0.510875563	352	0.227951033
352.074845	0.508795838	352.074845	0.227940386
352.14969	0.505188447	352.14969	0.227820361
352.358282	0.503663481	352.358282	0.227098039
353.358282	0.502635791	353.358282	0.217442742

Final State: j'=1		Final State: j'=0	
E(cm⁻¹)	σ (Å²)	E(cm⁻¹)	σ (Å²)
353.582817	0.500303859	353.582817	0.217627834
354	0.511159546	354	0.220080414
354.074845	0.511434645	354.074845	0.221273634
354.14969	0.510800526	354.14969	0.221920983
355	0.5068239	355	0.22595723
355.358282	0.497289244	355.358282	0.219006666
355.582817	0.513550187	355.582817	0.224170968
356	0.504519027	356	0.225064288
356.074845	0.503098301	356.074845	0.224502504
356.14969	0.501727351	356.14969	0.223859678
357.074845	0.513632305	357.074845	0.21589856
357.358282	0.515610743	357.358282	0.223417764
357.582817	0.503040533	357.582817	0.225370401
358	0.499459677	358	0.222193385
358.074845	0.497695131	358.074845	0.220132981
358.14969	0.497756089	358.14969	0.217299633
358.582817	0.519542492	358.582817	0.223141122
359.14969	0.509473471	359.14969	0.227481867
359.358282	0.506547283	359.358282	0.226619486
359.582817	0.503834004	359.582817	0.225583356
360	0.499020007	360	0.223091258
360.074845	0.498118414	360.074845	0.222499344
360.14969	0.497199354	360.14969	0.221836243
361.358282	0.513722494	361.358282	0.217988218
361.582817	0.51249602	361.582817	0.224377528
362	0.49637582	362	0.221917678
362.074845	0.495364904	362.074845	0.220189454
362.14969	0.494195584	362.14969	0.217577864
362.358282	0.51278781	362.358282	0.208090482
363.358282	0.507862879	363.358282	0.22705006
363.582817	0.505659535	363.582817	0.226326774
364	0.50215562	364	0.224950758
364.074845	0.501553893	364.074845	0.224684261
364.14969	0.50095044	364.14969	0.224407343
365	0.492651287	365	0.219233597
365.358282	0.488870013	365.358282	0.213217472
365.582817	0.496531188	365.582817	0.209674015
366	0.503466248	366	0.21722145
366.074845	0.501437697	366.074845	0.218408715
367.074845	0.512860729	367.074845	0.212085543
367.358282	0.520757298	367.358282	0.228322193
367.582817	0.514632596	367.582817	0.228352506
368	0.508243974	368	0.226906122
368.074845	0.507480378	368.074845	0.226681092
368.14969	0.506779186	368.14969	0.22646542

Final State: j'=1		Final State: j'=0	
E(cm⁻¹)	σ (Å²)	E(cm⁻¹)	σ (Å²)
368.582817	0.50349228	368.582817	0.225336776
369.14969	0.500059709	369.14969	0.223938609
369.358282	0.498842482	369.358282	0.223382382
369.582817	0.497485686	369.582817	0.222719171
370	0.494629901	370	0.221145785
370.074845	0.494039525	370.074845	0.22078467
370.14969	0.493415006	370.14969	0.220387012
371.358282	0.508733794	371.358282	0.212955237
371.582817	0.49610511	371.582817	0.219097032
372	0.501809748	372	0.215291593
372.074845	0.50985687	372.074845	0.217877024
372.14969	0.508058684	372.14969	0.219207899
372.358282	0.500797648	372.358282	0.211829919
373.358282	0.506757537	373.358282	0.226100037
373.582817	0.50461349	373.582817	0.225436978
374	0.501766013	374	0.22444602
374.074845	0.501342106	374.074845	0.224287776
374.14969	0.500935347	374.14969	0.224133306
375	0.496940247	375	0.222473207
375.358282	0.495326507	375.358282	0.221721851
376	0.492006453	376	0.219983722
376.074845	0.491545813	376.074845	0.219716628
376.14969	0.491061393	376.14969	0.219427647
377.074845	0.481253825	377.074845	0.209941327
377.358282	0.487245912	377.358282	0.202148875
377.582817	0.508879267	377.582817	0.213820799
378	0.492297457	378	0.218556473
378.074845	0.49115592	378.074845	0.217425259
378.14969	0.489467411	378.14969	0.215475893
378.582817	0.51260568	378.582817	0.215365766
379.14969	0.505879635	379.14969	0.225168972
379.358282	0.502263064	379.358282	0.224165244
379.582817	0.499738049	379.582817	0.223334437
380	0.49647929	380	0.222131518
380.074845	0.495991428	380.074845	0.221939658
380.14969	0.495521179	380.14969	0.221751778
381.358282	0.488491756	381.358282	0.218602399
381.582817	0.486990225	381.582817	0.217828398
382	0.483659743	382	0.215888326
382.074845	0.482961515	382.074845	0.215421991
382.14969	0.482230423	382.14969	0.214898876
382.358282	0.480117264	382.358282	0.213024853
383.358282	0.490195993	383.358282	0.20917571
383.582817	0.485862964	383.582817	0.210958394
384	0.475983349	384	0.202028044

Final State: j’=1		Final State: j’=0	
E(cm⁻¹)	σ (Å²)	E(cm⁻¹)	σ (Å²)
384.14969	0.475320718	384.14969	0.19598294
385	0.490515981	385	0.207601219
385.358282	0.518640626	385.358282	0.215824548
385.582817	0.511011082	385.582817	0.216557866
386	0.49314939	386	0.214561074
386.074845	0.491475536	386.074845	0.212680886
386.14969	0.491271803	386.14969	0.210804909
387.074845	0.512682737	387.074845	0.220961974
387.358282	0.505129679	387.358282	0.221112872
387.582817	0.500349559	387.582817	0.220056737
388	0.492127903	388	0.21652197
388.074845	0.490574299	388.074845	0.21553567
388.14969	0.488995859	388.14969	0.214365518
388.582817	0.48891296	388.582817	0.205366704
389.14969	0.515114605	389.14969	0.211412895
389.358282	0.51686071	389.358282	0.22006508
389.582817	0.509827903	389.582817	0.221285116
390	0.500942665	390	0.220020002
390.074845	0.499630157	390.074845	0.219657573
390.14969	0.498350805	390.14969	0.219264532
391.358282	0.470948361	391.358282	0.197743253
391.582817	0.474992106	391.582817	0.185017011
392	0.521032657	392	0.204078853
392.074845	0.530344444	392.074845	0.211496324
392.14969	0.530752113	392.14969	0.217185952
392.358282	0.515496225	392.358282	0.219191785
393.358282	0.509623227	393.358282	0.224687402
393.582817	0.505638183	393.582817	0.223298494
394	0.49934032	394	0.220743213
394.074845	0.498204642	394.074845	0.220211008
394.14969	0.497035662	394.14969	0.219633437
395	0.48333252	395	0.202359627
395.358282	0.500818032	395.358282	0.200800481
395.582817	0.533513616	395.582817	0.218787357
396	0.516501299	396	0.224483202
396.074845	0.514653109	396.074845	0.224343339
396.14969	0.51303386	396.14969	0.224164731
397.074845	0.50099321	397.074845	0.221321149
397.358282	0.498270233	397.358282	0.220320135
397.582817	0.496077573	397.582817	0.219416356
398	0.491460246	398	0.21720209
398.074845	0.490489784	398.074845	0.216676313
398.14969	0.489453513	398.14969	0.216088081
398.582817	0.481392622	398.582817	0.210209988
399.14969	0.488318296	399.14969	0.19729

Final State: j'=1		Final State: j'=0	
E(cm⁻¹)	σ (Å²)	E(cm⁻¹)	σ (Å²)
399.358282	0.515337227	399.358282	0.205042423
399.582817	0.505640519	399.582817	0.207441431
400	0.514827988	400	0.222000535
400.074845	0.510288846	400.074845	0.221645204
400.14969	0.504406011	400.14969	0.220516436
401.358282	0.500188033	401.358282	0.217766494
401.582817	0.500835524	401.582817	0.214065002
402.074845	0.495416906	402.074845	0.216805484
402.14969	0.49307264	402.14969	0.215433181
402.358282	0.487680156	402.358282	0.209987693
403.358282	0.476799854	403.358282	0.194532277
403.582817	0.494102148	403.582817	0.185133394
404	0.532207993	404	0.225058243
404.074845	0.527149044	404.074845	0.225354194
404.14969	0.522876063	404.14969	0.225220117
405	0.489557019	405	0.212322845
405.358282	0.526355454	405.358282	0.211451828
405.582817	0.519661159	405.582817	0.22201347
406	0.506387503	406	0.22144929
406.074845	0.504640468	406.074845	0.221028117
406.14969	0.502963415	406.14969	0.220563953
407.074845	0.485616385	407.074845	0.194715747
407.358282	0.50374192	407.358282	0.216164445
407.582817	0.490596759	407.582817	0.211702356
408	0.485997461	408	0.189915553
408.074845	0.490246507	408.074845	0.193840042
408.14969	0.489010923	408.14969	0.192773413
408.582817	0.50893529	408.582817	0.218534861
409.14969	0.499649168	409.14969	0.20144764
409.358282	0.528891283	409.358282	0.223205163
409.582817	0.535275951	409.582817	0.22531033
410	0.496563148	410	0.210933203
410.074845	0.526127753	410.074845	0.224565948
410.14969	0.516922119	410.14969	0.225646832
410.374226	0.501704225	410.374226	0.221741231
410.374227	0.501702004	410.374227	0.221739555
410.374228	0.501700188	410.374228	0.221738214
410.374229	0.501698581	410.374229	0.221737045
410.37423	0.501697105	410.37423	0.221735986
410.374231	0.501695724	410.374231	0.221735004
410.374232	0.501694413	410.374232	0.221734081
410.374233	0.501693159	410.374233	0.221733206
410.374234	0.501691951	410.374234	0.221732367
410.374235	0.501690782	410.374235	0.22173156
410.374236	0.501689645	410.374236	0.221730781

Final State: j’=1		Final State: j’=0	
E(cm⁻¹)	σ (Å²)	E(cm⁻¹)	σ (Å²)
410.374237	0.501688537	410.374237	0.221730024
410.374238	0.501687452	410.374238	0.221729288
410.374239	0.501686389	410.374239	0.22172857
410.37424	0.501685346	410.37424	0.221727868
410.374241	0.501684321	410.374241	0.22172718
410.374242	0.501683311	410.374242	0.221726505
410.374243	0.501682316	410.374243	0.221725843
410.374244	0.501681332	410.374244	0.22172519
410.374245	0.501680363	410.374245	0.221724548
410.374246	0.501679404	410.374246	0.221723915
410.374247	0.501678455	410.374247	0.221723291
410.374248	0.501677516	410.374248	0.221722674
410.374249	0.501676587	410.374249	0.221722064
410.37425	0.501675665	410.37425	0.221721462
410.374251	0.501674751	410.374251	0.221720867
410.374252	0.501673845	410.374252	0.221720277
410.374253	0.501672946	410.374253	0.221719692
410.374254	0.501672053	410.374254	0.221719113
410.374255	0.501671167	410.374255	0.221718538
410.374256	0.501670286	410.374256	0.22171797
410.374257	0.501669411	410.374257	0.221717405
410.374258	0.501668541	410.374258	0.221716845
410.374259	0.501667677	410.374259	0.221716289
410.37426	0.501666817	410.37426	0.221715737
410.374261	0.501665963	410.374261	0.221715188
410.374262	0.501665113	410.374262	0.221714644
410.374263	0.501664266	410.374263	0.221714102
410.374264	0.501663424	410.374264	0.221713563
410.374265	0.501662585	410.374265	0.221713028
410.374266	0.501661751	410.374266	0.221712497
410.374267	0.50166092	410.374267	0.221711965
410.374268	0.501660093	410.374268	0.221711439
410.374269	0.501659268	410.374269	0.221710917
410.37427	0.501658448	410.37427	0.221710396
410.374271	0.501657631	410.374271	0.221709877
410.374272	0.501656816	410.374272	0.22170936
410.374273	0.501656003	410.374273	0.221708847
410.374274	0.501655194	410.374274	0.221708335
410.374275	0.501654388	410.374275	0.221707825
410.374276	0.501653584	410.374276	0.221707318
410.374277	0.501652783	410.374277	0.221706813
410.374278	0.501651984	410.374278	0.221706309
410.374279	0.501651187	410.374279	0.221705808
410.37428	0.501650393	410.37428	0.221705309
410.374281	0.5016496	410.374281	0.22170481

Final State: j’=1		Final State: j’=0	
E(cm⁻¹)	σ (Å²)	E(cm⁻¹)	σ (Å²)
410.374282	0.50164881	410.374282	0.221704314
410.374283	0.501648022	410.374283	0.221703819
410.374284	0.501647237	410.374284	0.221703327
410.374285	0.501646453	410.374285	0.221702835
410.374286	0.501645671	410.374286	0.221702345
410.374287	0.50164489	410.374287	0.221701855
410.374288	0.501644112	410.374288	0.221701368
410.374289	0.501643335	410.374289	0.221700883
410.37429	0.50164256	410.37429	0.221700398
410.374291	0.501641787	410.374291	0.221699915
410.374292	0.501641014	410.374292	0.221699433
410.374293	0.501640248	410.374293	0.221698948
410.374294	0.501639479	410.374294	0.221698469
410.374295	0.501638712	410.374295	0.22169799
410.374296	0.501637946	410.374296	0.221697513
410.374297	0.501637182	410.374297	0.221697037
410.374298	0.501636419	410.374298	0.221696562
410.374299	0.501635658	410.374299	0.221696088
410.3743	0.501634898	410.3743	0.221695615
410.374301	0.501634141	410.374301	0.221695143
410.374302	0.501633383	410.374302	0.221694672
410.374303	0.501632627	410.374303	0.221694202
410.374304	0.501631871	410.374304	0.221693733
410.374305	0.501631118	410.374305	0.221693265
410.374306	0.501630365	410.374306	0.221692797
410.374307	0.501629614	410.374307	0.221692332
410.374308	0.501628863	410.374308	0.221691865
410.374309	0.501628114	410.374309	0.221691401
410.37431	0.501627365	410.37431	0.221690937
410.374311	0.501626618	410.374311	0.221690474
410.374312	0.501625872	410.374312	0.221690012
410.374313	0.501625127	410.374313	0.22168955
410.374314	0.501624382	410.374314	0.22168909
410.374315	0.501623638	410.374315	0.221688629
410.374316	0.501622897	410.374316	0.22168817
410.374317	0.501622154	410.374317	0.221687711
410.374318	0.501621413	410.374318	0.221687253
410.374319	0.501620674	410.374319	0.221686796
410.37432	0.501619935	410.37432	0.22168634
410.374321	0.501619197	410.374321	0.221685883
410.374322	0.501618459	410.374322	0.221685427
410.374323	0.501617723	410.374323	0.221684973
410.374324	0.501616987	410.374324	0.221684519
410.374325	0.501616252	410.374325	0.221684065
410.374825	0.501264288	410.374825	0.221463869

Final State: j’=1		Final State: j’=0	
E(cm⁻¹)	σ (Å²)	E(cm⁻¹)	σ (Å²)
410.375225	0.50096635	410.375225	0.221265106
410.375325	0.500888493	410.375325	0.221211085
410.375825	0.500478074	410.375825	0.220912616
410.376225	0.50012676	410.376225	0.220638731
410.376325	0.500036144	410.376325	0.220565411
410.376825	0.499572514	410.376825	0.220171194
410.377225	0.499196755	410.377225	0.219826951
410.377325	0.499103397	410.377325	0.219737726
410.377825	0.49865105	410.377825	0.219279961
410.378225	0.498320164	410.378225	0.218910488
410.378325	0.498243225	410.378325	0.218819464
410.378825	0.497906393	410.378825	0.218382764
410.379225	0.497702645	410.379225	0.21806873
410.379325	0.497662513	410.379325	0.217996779
410.379825	0.497523918	410.379825	0.217683762
410.380225	0.497489451	410.380225	0.21749525
410.380325	0.497491335	410.380325	0.217457172
410.380825	0.497555204	410.380825	0.217319684
410.381225	0.497663435	410.381225	0.21726961
410.381325	0.49769724	410.381325	0.217264716
410.381825	0.497896566	410.381825	0.217279643
410.382225	0.498083534	410.382225	0.217331535
410.382325	0.498132625	410.382325	0.217349024
410.382825	0.498387801	410.382825	0.217457414
410.383225	0.498594856	410.383225	0.217563011
410.383325	0.498646687	410.383325	0.217591376
410.383825	0.49890046	410.383825	0.217739864
410.384225	0.499094495	410.384225	0.217863547
410.384325	0.499141892	410.384325	0.217894956
410.384825	0.499366742	410.384825	0.21805011
410.385225	0.499533956	410.385225	0.218172256
410.385325	0.499574027	410.385325	0.218202286
410.385825	0.499762766	410.385825	0.218348235
410.386225	0.499900411	410.386225	0.218459502
410.386325	0.499932386	410.386325	0.218486529
410.386825	0.500085315	410.386825	0.218616594
410.387225	0.500196998	410.387225	0.218714622
410.387325	0.500222178	410.387325	0.218738209
410.387825	0.500344733	410.387825	0.218851114
410.388225	0.500431721	410.388225	0.2189357
410.388325	0.500452768	410.388325	0.218955947
410.388825	0.500548504	410.388825	0.21905298
410.389225	0.500617911	410.389225	0.21912518
410.389325	0.500634353	410.389325	0.219142285
410.389825	0.500708848	410.389825	0.219225335

Final State: j’=1		Final State: j’=0	
E(cm⁻¹)	σ (Å²)	E(cm⁻¹)	σ (Å²)
410.390225	0.500762277	410.390225	0.219286829
410.390325	0.500774488	410.390325	0.219301529
410.390825	0.500832655	410.390825	0.219371906
410.391225	0.500874131	410.391225	0.219424163
410.391325	0.500883937	410.391325	0.219436812
410.391825	0.500927484	410.391825	0.219496385
410.392225	0.500958486	410.392225	0.219540935
410.392325	0.500965811	410.392325	0.219551584
410.392825	0.500997936	410.392825	0.219602305
410.393225	0.501020976	410.393225	0.21964012
410.393325	0.501026894	410.393325	0.219648905
410.393825	0.501049665	410.393825	0.219692228
410.394225	0.501066371	410.394225	0.219724246
410.395225	0.501095385	410.395225	0.219795669
410.396225	0.501112445	410.396225	0.219856022
410.397225	0.501119429	410.397225	0.219906946
410.398225	0.501115663	410.398225	0.219949422
410.399225	0.501105037	410.399225	0.219984558
410.400225	0.501088066	410.400225	0.220013298
410.401225	0.501064192	410.401225	0.220036465
410.402225	0.501036399	410.402225	0.220054311
410.403225	0.501001821	410.403225	0.220067387
410.404225	0.500964886	410.404225	0.220076585
410.405225	0.500923113	410.405225	0.220082097
410.406225	0.500879171	410.406225	0.220083734
410.407225	0.500831379	410.407225	0.220082411
410.408225	0.500780488	410.408225	0.220078011
410.409225	0.500726823	410.409225	0.220070962
410.410225	0.500670887	410.410225	0.220061186
410.411225	0.500613351	410.411225	0.220049403
410.412225	0.50055389	410.412225	0.220035605
410.413225	0.500492358	410.413225	0.220019239
410.414225	0.500427834	410.414225	0.220001111
410.415225	0.500363681	410.415225	0.219981313
410.416225	0.500297008	410.416225	0.219959794
410.417225	0.500229149	410.417225	0.219936391
410.418225	0.500159327	410.418225	0.219911726
410.419225	0.500089729	410.419225	0.219885851
410.420225	0.50001818	410.420225	0.219858362
410.421225	0.49994564	410.421225	0.219829788
410.422225	0.499873747	410.422225	0.219800145
410.423225	0.499799166	410.423225	0.219769219
410.424225	0.49972423	410.424225	0.219737512
410.425225	0.499649025	410.425225	0.219704886
410.426225	0.499573417	410.426225	0.219671358

Final State: j’=1		Final State: j’=0	
E(cm⁻¹)	σ (Å²)	E(cm⁻¹)	σ (Å²)
410.427225	0.499497882	410.427225	0.219637038
410.428225	0.499420645	410.428225	0.219602083
410.429225	0.499344146	410.429225	0.21956643
410.430225	0.499267136	410.430225	0.219530461
410.431225	0.499190177	410.431225	0.219493713
410.432225	0.499113245	410.432225	0.219456778
410.433225	0.499035325	410.433225	0.219419165
410.434225	0.498957793	410.434225	0.219381684
410.435225	0.498881341	410.435225	0.219343598
410.436225	0.49880464	410.436225	0.219305123
410.437225	0.498727253	410.437225	0.219266805
410.438225	0.498650129	410.438225	0.219228294
410.439225	0.498573868	410.439225	0.219189652
410.440225	0.498497605	410.440225	0.219151091
410.441225	0.498421644	410.441225	0.219112367
410.442225	0.498345805	410.442225	0.219073612
410.443225	0.498269689	410.443225	0.219035133
410.444225	0.498195602	410.444225	0.218996617
410.445225	0.498119403	410.445225	0.218958296
410.446225	0.498045878	410.446225	0.218920089
410.447225	0.497972267	410.447225	0.218881845
410.448225	0.497897514	410.448225	0.218843926
410.449225	0.49782449	410.449225	0.218805955
410.450225	0.497750267	410.450225	0.218768483
410.451225	0.497678011	410.451225	0.218730593
410.452225	0.497604849	410.452225	0.218693585
410.453225	0.497531657	410.453225	0.218656635
410.454225	0.49745988	410.454225	0.218619575
410.455225	0.497387811	410.455225	0.218582989
410.456225	0.497315976	410.456225	0.218546147
410.457225	0.497244421	410.457225	0.218509752
410.458225	0.497173184	410.458225	0.218473128
410.459225	0.497100759	410.459225	0.218436786
410.460225	0.497029858	410.460225	0.218401319
410.461225	0.496958665	410.461225	0.218364762
410.462225	0.496886529	410.462225	0.218328919
410.463225	0.496814971	410.463225	0.21829344
410.464225	0.496742695	410.464225	0.21825735
410.465225	0.49667068	410.465225	0.21822161
410.466225	0.496598882	410.466225	0.218185585
410.467225	0.496526539	410.467225	0.218150073
410.468225	0.496453939	410.468225	0.218113712
410.469225	0.496380314	410.469225	0.218077719
410.470225	0.49630687	410.470225	0.218041722
410.471225	0.496233471	410.471225	0.21800549

Final State: j’=1		Final State: j’=0	
E(cm⁻¹)	σ (Å²)	E(cm⁻¹)	σ (Å²)
410.472225	0.496159556	410.472225	0.217968691
410.473225	0.496084484	410.473225	0.217932121
410.474225	0.496010346	410.474225	0.217895448
410.574225	0.47670772	410.574225	0.198444809
410.674225	0.497363331	410.674225	0.214906107
410.774225	0.494232422	410.774225	0.214630013
410.874225	0.491225453	410.874225	0.213472571
410.974225	0.487532777	410.974225	0.21154185
411.074225	0.483093171	411.074225	0.208760518
411.174225	0.478265819	411.174225	0.205395928
411.274225	0.470667349	411.274225	0.198475268
411.358282	0.465562167	411.358282	0.183592677
411.374225	0.473665814	411.374225	0.181537739
411.474225	0.509745598	411.474225	0.207765432
411.574225	0.501870038	411.574225	0.211459546
411.582817	0.501341041	411.582817	0.211507127
411.674225	0.496837694	411.674225	0.211350714
411.774225	0.493494762	411.774225	0.21069177
411.874225	0.491010125	411.874225	0.20994251
411.974225	0.488972735	411.974225	0.209179861
412	0.488490856	412	0.208981616
412.074225	0.487161783	412.074225	0.20840083
412.074845	0.4871498	412.074845	0.20839619
412.14969	0.48585202	412.14969	0.207784789
412.174225	0.485428443	412.174225	0.207575608
412.274225	0.483650033	412.274225	0.206650463
412.358282	0.482028098	412.358282	0.205735445
412.374225	0.481698274	412.374225	0.205541099
412.474225	0.479399491	412.474225	0.204093653
412.574225	0.476486977	412.574225	0.202003795
412.674225	0.472541412	412.674225	0.198575432
412.774225	0.467110601	412.774225	0.192098138
412.874225	0.467064763	412.874225	0.183580401
412.974225	0.475465074	412.974225	0.182228897
413.074225	0.510688558	413.074225	0.191788863
413.174225	0.50503578	413.174225	0.208317592
413.274225	0.495554827	413.274225	0.207036842
413.358282	0.490797781	413.358282	0.205400338
413.374225	0.490152789	413.374225	0.205145067
413.474225	0.487604536	413.474225	0.204138855
413.574225	0.486776886	413.574225	0.203995746
413.582817	0.486750354	413.582817	0.204008346
413.674225	0.486623512	413.674225	0.204249136
413.774225	0.486586385	413.774225	0.204564592
413.874225	0.486486105	413.874225	0.204811785

Final State: j’=1		Final State: j’=0	
E(cm^{-1})	σ (Å^2)	E(cm^{-1})	σ (Å^2)
413.974225	0.486300386	413.974225	0.204965239
414	0.486238268	414	0.204990507
414.074225	0.486036919	414.074225	0.205033822
414.074845	0.486034669	414.074845	0.205034052
414.14969	0.485798839	414.14969	0.205038254
414.174225	0.485716567	414.174225	0.205032018
414.274225	0.485349514	414.274225	0.204972665
414.374225	0.484945081	414.374225	0.204865552
414.474225	0.484507232	414.474225	0.204716218
414.574225	0.484039131	414.574225	0.204530088
414.674225	0.483542669	414.674225	0.204308867
414.774225	0.483018079	414.774225	0.204055863
414.874225	0.482465302	414.874225	0.203773264
414.974225	0.481897655	414.974225	0.203464557
415	0.481746667	415	0.203381599
415.074225	0.481309409	415.074225	0.203134604
415.174225	0.480712611	415.174225	0.202788283
415.274225	0.480117512	415.274225	0.202430867
415.358282	0.479614415	415.358282	0.202125706
415.374225	0.479519672	415.374225	0.202067517
415.582817	0.478288544	415.582817	0.201301074
416	0.475503461	416	0.199516591
416.074845	0.474820281	416.074845	0.199053633
416.14969	0.474006658	416.14969	0.198473805
416.374225	0.470135814	416.374225	0.194794191
417.074845	0.466553849	417.074845	0.186278843
417.358282	0.481406525	417.358282	0.200279931
417.374225	0.480111464	417.374225	0.19964846
417.582817	0.471135809	417.582817	0.192619349
418	0.481667502	418	0.200296854
418.074845	0.482009586	418.074845	0.200727557
418.14969	0.482118186	418.14969	0.200972015
418.374225	0.481910638	418.374225	0.201192466
418.582817	0.481528972	418.582817	0.201135251
419.14969	0.480543397	419.14969	0.200720649
419.358282	0.480232221	419.358282	0.200550288
419.374225	0.480208611	419.374225	0.200537319
419.582817	0.47991792	419.582817	0.200364781
420	0.479349259	420	0.200005019
420.074845	0.47924395	420.074845	0.19993616
420.14969	0.47913606	420.14969	0.199865271
420.374225	0.478789486	420.374225	0.199635933
421.358282	0.476279967	421.358282	0.197966331
421.374225	0.476221109	421.374225	0.197927692
421.582817	0.475439544	421.582817	0.197387811

Final State: j’=1		Final State: j’=0	
E(cm⁻¹)	σ (Å²)	E(cm⁻¹)	σ (Å²)
422	0.474314018	422	0.196519981
422.074845	0.474245699	422.074845	0.196441065
422.14969	0.474223272	422.14969	0.196390316
422.358282	0.474362392	422.358282	0.196378136
422.374225	0.474382372	422.374225	0.196383495
423.358282	0.475747639	423.358282	0.196945689
423.374225	0.475758	423.374225	0.196949559
423.582817	0.475853107	423.582817	0.196972605
424	0.475782879	424	0.196873532
424.074845	0.475726567	424.074845	0.196831382
424.14969	0.47565337	424.14969	0.196779586
424.374225	0.47529578	424.374225	0.196546437
425	0.467870744	425	0.191591952
425.358282	0.472426222	425.358282	0.194337162
425.374225	0.47310687	425.374225	0.194579825
425.582817	0.47184502	425.582817	0.192332827
426	0.479047277	426	0.197859541
426.074845	0.478873087	426.074845	0.197789413
426.14969	0.478707357	426.14969	0.197711738
426.374225	0.478303557	426.374225	0.197491489
427.074845	0.477638736	427.074845	0.197008887
427.358282	0.477511585	427.358282	0.196874792
427.374225	0.477506709	427.374225	0.196867826
427.582817	0.477446333	427.582817	0.196785663
428	0.477385789	428	0.196650381
428.074845	0.477381339	428.074845	0.196629373
428.14969	0.477378301	428.14969	0.196609172
428.374225	0.477377135	428.374225	0.19655259
428.582817	0.477384358	428.582817	0.196504416
429.14969	0.477427388	429.14969	0.196385898
429.374225	0.477449366	429.374225	0.196341292
429.582817	0.477469154	429.582817	0.196300085
430	0.477507249	430	0.196216345
430.074845	0.477513387	430.074845	0.196200948
430.14969	0.477519185	430.14969	0.196185374
430.374225	0.477534526	430.374225	0.196137565
431.358282	0.477527526	431.358282	0.195890788
431.374225	0.47752413	431.374225	0.195886799
431.582817	0.477492026	431.582817	0.195817901
432	0.477347554	432	0.195644751
432.074845	0.477305188	432.074845	0.195605799
432.14969	0.477255913	432.14969	0.195563501
432.358282	0.477072697	432.358282	0.195423883
432.374225	0.4770555	432.374225	0.195411621
433.358282	0.475710836	433.358282	0.194514534

Final State: j’=1		Final State: j’=0	
E(cm⁻¹)	σ (Å²)	E(cm⁻¹)	σ (Å²)
433.374225	0.475719858	433.374225	0.194515675
433.582817	0.476014595	433.582817	0.194594858
434	0.476894584	434	0.194902485
434.074845	0.477034866	434.074845	0.194950541
434.14969	0.477164851	434.14969	0.194993958
434.374225	0.477494661	434.374225	0.195096233
435	0.478077448	435	0.195221338
435.358282	0.478290792	435.358282	0.195234468
435.374225	0.478299148	435.374225	0.195234509
435.582817	0.478402105	435.582817	0.195231687
436	0.478581198	436	0.195212747
436.074845	0.478610489	436.074845	0.195207991
436.14969	0.478639498	436.14969	0.195202948
436.374225	0.478722453	436.374225	0.195186117
437.074845	0.478959446	437.074845	0.195122196
437.358282	0.479048532	437.358282	0.195092931
437.374225	0.4790576	437.374225	0.195092467
437.582817	0.479117213	437.582817	0.195068726
438	0.479240017	438	0.195021384
438.074845	0.479261403	438.074845	0.195012589
438.14969	0.479282647	438.14969	0.195003709
438.374225	0.479344997	438.374225	0.19497645
438.582817	0.479401364	438.582817	0.194950315
439.14969	0.479544986	439.14969	0.194874999
439.358282	0.479593893	439.358282	0.194845504
439.374225	0.479596764	439.374225	0.194843141
439.582817	0.47964411	439.582817	0.194812643
440	0.47973037	440	0.19474848
440.074845	0.47974497	440.074845	0.194736571
440.14969	0.479759312	440.14969	0.194724553
440.374225	0.479801205	440.374225	0.194687982
441.358282	0.479987667	441.358282	0.194528725
441.374225	0.479991939	441.374225	0.194526258
441.582817	0.480038224	441.582817	0.194495739
442	0.480146761	442	0.19444047
442.074845	0.480168361	442.074845	0.194431424
442.14969	0.480190532	442.14969	0.194422625
442.358282	0.480255474	442.358282	0.194399444
442.374225	0.480260643	442.374225	0.19439776
443.358282	0.480614797	443.358282	0.194307825
443.374225	0.480619526	443.374225	0.194306562
443.582817	0.480701748	443.582817	0.194289788
444	0.480865543	444	0.19425702
444.074845	0.480895084	444.074845	0.194251173
444.14969	0.480924635	444.14969	0.194245336

Final State: j’=1		Final State: j’=0	
E(cm⁻¹)	σ (Å²)	E(cm⁻¹)	σ (Å²)
444.374225	0.481013268	444.374225	0.194227806
445	0.48125931	445	0.194178228
445.358282	0.48139892	445.358282	0.194149058
445.374225	0.481405142	445.374225	0.194147739
445.582817	0.481485749	445.582817	0.194130526
446	0.481646065	446	0.19409544
446.074845	0.481674645	446.074845	0.194089048
446.14969	0.481703235	446.14969	0.194082641
446.374225	0.481788492	446.374225	0.194063253
447.074845	0.482052064	447.074845	0.19400132
447.358282	0.482157539	447.358282	0.193975668
447.374225	0.482162594	447.374225	0.193974359
447.582817	0.482240854	447.582817	0.193955149
448	0.482394455	448	0.193916516
448.074845	0.482421875	448.074845	0.193909509
448.14969	0.482449265	448.14969	0.193902505
448.374225	0.482531461	448.374225	0.193881387
448.582817	0.482607509	448.582817	0.193861629
449.14969	0.482813565	449.14969	0.193807471
449.358282	0.48288927	449.358282	0.193787407
449.374225	0.482894426	449.374225	0.193785746
449.582817	0.482970731	449.582817	0.193765764
450	0.48312232	450	0.193725479
450.074845	0.483149504	450.074845	0.193718255
450.14969	0.483176755	450.14969	0.193711013
450.374225	0.483258621	450.374225	0.193689353
451.358282	0.483621088	451.358282	0.193595165
451.374225	0.483626766	451.374225	0.193593616
451.582817	0.483704743	451.582817	0.193573971
452	0.483861513	452	0.19353482
452.074845	0.483889822	452.074845	0.193527786
452.14969	0.483918238	452.14969	0.193520823
452.358282	0.483997484	452.358282	0.1935015
452.374225	0.484003513	452.374225	0.193500031
453.358282	0.48438308	453.358282	0.193410266
453.374225	0.484388947	453.374225	0.193408675
453.582817	0.484470892	453.582817	0.193390063
454	0.484635053	454	0.19335288
454.074845	0.484664614	454.074845	0.193346252
454.14969	0.48469431	454.14969	0.193339612
454.374225	0.484783398	454.374225	0.193319785
455	0.485033779	455	0.193264924
455.358282	0.485178061	455.358282	0.193233743
455.374225	0.48518455	455.374225	0.193232363
455.582817	0.485268995	455.582817	0.193214272

Final State: j’=1		Final State: j’=0	
E(cm⁻¹)	σ (Å²)	E(cm⁻¹)	σ (Å²)
456	0.485438497	456	0.19317824
456.074845	0.485469	456.074845	0.193171816
456.14969	0.485499516	456.14969	0.193165369
456.374225	0.485591244	456.374225	0.193146067
457.074845	0.485878931	457.074845	0.193086063
457.358282	0.485995917	457.358282	0.193061848
457.374225	0.486002533	457.374225	0.193060427
457.582817	0.48608879	457.582817	0.193042717
458	0.48626183	458	0.193007213
458.074845	0.486293014	458.074845	0.193000863
458.14969	0.486324109	458.14969	0.192994501
458.374225	0.486417631	458.374225	0.192975433
458.582817	0.486504745	458.582817	0.192957745
459.14969	0.48674214	459.14969	0.192909759
459.358282	0.486829799	459.358282	0.192892129
459.374225	0.486836323	459.374225	0.192890771
459.582817	0.486924316	459.582817	0.192873163
460	0.487100425	460	0.192838001
460.074845	0.487132088	460.074845	0.192831695
460.14969	0.487163809	460.14969	0.192825404
460.374225	0.48725897	460.374225	0.192806507
461.358282	0.487678559	461.358282	0.192724005
461.582817	0.487774787	461.582817	0.19270525
462	0.487954208	462	0.192670455
462.074845	0.487986449	462.074845	0.192664237
462.14969	0.488018758	462.14969	0.192657992
462.358282	0.488108935	462.358282	0.19264066
462.374225	0.488115827	462.374225	0.192639333
463.358282	0.488543664	463.358282	0.192557878
463.582817	0.488641862	463.582817	0.192539364
464	0.488824962	464	0.192505065
464.074845	0.488857917	464.074845	0.192498923
464.14969	0.488890822	464.14969	0.192492783
464.374225	0.488989896	464.374225	0.192474374
465	0.489267009	465	0.192423274
465.358282	0.48942664	465.358282	0.192394108
465.374225	0.489433534	465.374225	0.192392804
465.582817	0.489526707	465.582817	0.192375861
466	0.489713825	466	0.192342074
466.074845	0.48974753	466.074845	0.19233601
466.374225	0.489881876	466.374225	0.19231182
467.074845	0.490198848	467.074845	0.192255465
467.358282	0.490327673	467.358282	0.192232711
467.582817	0.490429503	467.582817	0.192214766
468	0.490620224	468	0.192181437

Final State: j’=1		Final State: j’=0	
E(cm⁻¹)	σ (Å²)	E(cm⁻¹)	σ (Å²)
468.074845	0.490654623	468.074845	0.192175438
468.14969	0.490688848	468.14969	0.192169516
468.374225	0.490791929	468.374225	0.192151625
468.582817	0.490887794	468.582817	0.192135019
469.14969	0.491149241	469.14969	0.192090042
469.358282	0.491245824	469.358282	0.192073501
469.582817	0.491350013	469.582817	0.192055751
470	0.491544093	470	0.192022863
470.074845	0.49157899	470.074845	0.192016953
470.14969	0.491613901	470.14969	0.19201106
470.374225	0.491718812	470.374225	0.191993408
471.358282	0.492181107	471.358282	0.191916219
471.582817	0.492286923	471.582817	0.191898693
472	0.492484402	472	0.191866178
472.074845	0.4925199	472.074845	0.191860348
472.14969	0.492555426	472.14969	0.191854517
472.358282	0.492654914	472.358282	0.191838259
472.374225	0.492662334	472.374225	0.191837038
473.358282	0.493132602	473.358282	0.191760714
473.582817	0.493240435	473.582817	0.19174337
474	0.49344142	474	0.191711191
474.074845	0.493477518	474.074845	0.191705413
474.14969	0.493513621	474.14969	0.191699657
474.374225	0.493622161	474.374225	0.191682354
475	0.493925876	475	0.19163429
475.358282	0.494100475	475.358282	0.191606857
475.374225	0.494108253	475.374225	0.191605629
476	0.494414452	476	0.19155784
476.074845	0.494451172	476.074845	0.191552127
476.14969	0.494487921	476.14969	0.19154643
476.374225	0.494598345	476.374225	0.191529323
477.074845	0.494944143	477.074845	0.191476108
477.358282	0.495084624	477.358282	0.191454614
477.582817	0.49519614	477.582817	0.19143764
478	0.495403858	478	0.191406107
478.074845	0.495441189	478.074845	0.191400464
478.14969	0.495478583	478.14969	0.191394829
478.374225	0.495590769	478.374225	0.191377909
478.582817	0.495695257	478.582817	0.191362204
479.14969	0.495979983	479.14969	0.191319629
479.358282	0.496085099	479.358282	0.191303992
479.582817	0.496198466	479.582817	0.191287184
480	0.496409575	480	0.19125601
480.074845	0.496447463	480.074845	0.191250416
480.14969	0.496485529	480.14969	0.19124483

Final State: j’=1		Final State: j’=0	
E(cm⁻¹)	σ (Å²)	E(cm⁻¹)	σ (Å²)
480.374225	0.496599585	480.374225	0.191228097
481.358282	0.497101875	481.358282	0.191154975
481.582817	0.497217075	481.582817	0.191138358
482	0.497431577	482	0.19110752
482.074845	0.497470192	482.074845	0.191101994
482.14969	0.49750874	482.14969	0.191096466
482.358282	0.497616475	482.358282	0.191081095
482.374225	0.497624729	482.374225	0.191079919
483.358282	0.498135103	483.358282	0.191007613
483.582817	0.498252121	483.582817	0.190991206
484	0.498470177	484	0.190960686
484.14969	0.498548494	484.14969	0.19094977
484.374225	0.49866621	484.374225	0.190933399
485	0.498995462	485	0.190887957
485.358282	0.499184728	485.358282	0.190862003
485.374225	0.499193175	485.374225	0.190860832
485.582817	0.49930383	485.582817	0.190845749
486	0.499525223	486	0.190815647
486.074845	0.499565083	486.074845	0.190810248
486.14969	0.499604908	486.14969	0.190804864
486.374225	0.499724428	486.374225	0.190788717
487.074845	0.500099217	487.074845	0.190738481
487.358282	0.50025132	487.358282	0.190718223
487.582817	0.500372141	487.582817	0.190702206
488	0.500597103	488	0.190672512
488.074845	0.5006375	488.074845	0.190667178
488.14969	0.500678086	488.14969	0.190661881
488.374225	0.500799563	488.374225	0.190645956
488.582817	0.500912736	488.582817	0.190631177
489.14969	0.501221173	489.14969	0.190591159
489.358282	0.501334966	489.358282	0.19057647
489.582817	0.5014577	489.582817	0.190560711
490	0.501686397	490	0.190531481
490.074845	0.501727512	490.074845	0.190526231
490.14969	0.501768618	490.14969	0.190521023
490.374225	0.501892147	490.374225	0.190505378
491.358282	0.502436265	491.358282	0.190437095
491.582817	0.502561042	491.582817	0.190421619
492	0.502793494	492	0.190392947
492.074845	0.502835221	492.074845	0.190387809
492.14969	0.502877099	492.14969	0.190382679
492.358282	0.502993759	492.358282	0.190368417
492.374225	0.503002649	492.374225	0.190367324
493.358282	0.503555972	493.358282	0.19030048
493.582817	0.503682843	493.582817	0.190285324

Final State: j'=1		Final State: j'=0	
E(cm⁻¹)	σ (Å²)	E(cm⁻¹)	σ (Å²)
494	0.503919392	494	0.190257329
494.074845	0.503961816	494.074845	0.190252309
494.14969	0.504004365	494.14969	0.190247309
494.374225	0.504131993	494.374225	0.190232322
495	0.504489654	495	0.190190812
495.358282	0.50469513	495.358282	0.19016722
495.374225	0.504704309	495.374225	0.190166174
495.582817	0.504824283	495.582817	0.190152509
496	0.505065089	496	0.190125317
496.074845	0.505108303	496.074845	0.190120439
496.14969	0.50515157	496.14969	0.190115597
496.374225	0.505281793	496.374225	0.190101054
497.074845	0.505689611	497.074845	0.190056156
497.358282	0.505855347	497.358282	0.19003817
497.582817	0.505986989	497.582817	0.190023982
498	0.506232404	498	0.189997843
498.074845	0.506276535	498.074845	0.189993175
498.14969	0.506320685	498.14969	0.189988517
498.374225	0.506453461	498.374225	0.18997461
498.582817	0.506577008	498.582817	0.189961742
499.14969	0.506914143	499.14969	0.189927172
499.358282	0.50703881	499.358282	0.189914582
499.582817	0.507173268	499.582817	0.18990114
500	0.507423973	500	0.189876376
500.074845	0.507469053	500.074845	0.189871981
500.14969	0.507514183	500.14969	0.189867576
500.374225	0.507649832	500.374225	0.189854517
501.358282	0.508248727	501.358282	0.189798422
501.582817	0.508386548	501.582817	0.189785948
502.074845	0.508689817	502.074845	0.189759143
502.14969	0.508736071	502.14969	0.189755135
502.358282	0.50886541	502.358282	0.189744022
502.374225	0.508875319	502.374225	0.189743178
503.358282	0.509490868	503.358282	0.189692779
503.582817	0.50963268	503.582817	0.18968178
504.074845	0.509945188	504.074845	0.189658422
504.14969	0.509992956	504.14969	0.18965496
504.374225	0.51013667	504.374225	0.189644753
505	0.510540604	505	0.189617643
505.358282	0.510774134	505.358282	0.189603149
505.374225	0.510785139	505.374225	0.189602435
505.582817	0.510921379	505.582817	0.189594467
506.074845	0.511246879	506.074845	0.189576713
506.14969	0.511296629	506.14969	0.189574172
506.374225	0.511446811	506.374225	0.189566826

Final State: j’=1		Final State: j’=0	
E(cm^{-1})	σ (Å^2)	E(cm^{-1})	σ (Å^2)
507.074845	0.511921305	507.074845	0.189546939
507.358282	0.512116276	507.358282	0.189540391
507.582817	0.512272032	507.582817	0.189535895
508.074845	0.512618069	508.074845	0.189528496
508.14969	0.512671121	508.14969	0.189527673
508.374225	0.512831971	508.374225	0.189525919
508.582817	0.512982818	508.582817	0.189525074
509.14969	0.513401021	509.14969	0.189527743
509.358282	0.513558541	509.358282	0.189530825
509.582817	0.513730502	509.582817	0.189535668
510	0.514058055	510	0.189549678
510.074845	0.514117967	510.074845	0.189552913
510.14969	0.514178287	510.14969	0.189556496
510.374225	0.514362237	510.374225	0.189569048
511.358282	0.515232915	511.358282	0.18967014
511.582817	0.51545286	511.582817	0.189709721
512.074845	0.51597985	512.074845	0.189837178
512.14969	0.516066955	512.14969	0.189863733
512.358282	0.516322011	512.358282	0.189952778
513.358282	0.51643931	513.358282	0.190944759
513.582817	0.515545722	513.582817	0.190528129
514.074845	0.513170774	514.074845	0.189801248
514.14969	0.51298514	514.14969	0.189387673
514.374225	0.51410138	514.374225	0.188878154
515	0.517068879	515	0.18965569
515.358282	0.516390448	515.358282	0.190288108
515.374225	0.51629184	515.374225	0.190295153
515.582817	0.514504541	515.582817	0.189785117
516.074845	0.515403948	516.074845	0.188420407
516.14969	0.51589168	516.14969	0.188470579
516.374225	0.516666433	516.374225	0.188673072
517.074845	0.518629776	517.074845	0.189596719
517.358282	0.518710051	517.358282	0.1906116
517.582817	0.516998539	517.582817	0.189638989
518.074845	0.515056272	518.074845	0.189182459
518.14969	0.514950199	518.14969	0.188767605
518.374225	0.516409168	518.374225	0.188409752
518.582817	0.517065357	518.582817	0.188892265
519.14969	0.517699286	519.14969	0.188386
519.358282	0.518659681	519.358282	0.188975417
519.582817	0.51660258	519.582817	0.189028936
520	0.518356845	520	0.188230522
520.074845	0.518689945	520.074845	0.188337223
520.14969	0.518982384	520.14969	0.188442984
520.374225	0.519733876	520.374225	0.188773415

Final State: j'=1		Final State: j'=0	
E(cm⁻¹)	σ (Å²)	E(cm⁻¹)	σ (Å²)
521.358282	0.515000553	521.358282	0.188204932
521.582817	0.517463238	521.582817	0.187432078
522.074845	0.519410705	522.074845	0.188092501
522.14969	0.519730607	522.14969	0.18829577
522.358282	0.520443898	522.358282	0.189293488
522.374225	0.52041286	522.374225	0.189415915
523.358282	0.520078475	523.358282	0.187543156
523.582817	0.520710493	523.582817	0.187751046
524.074845	0.521886699	524.074845	0.18820514
524.14969	0.522062557	524.14969	0.188287334
524.374225	0.522601824	524.374225	0.18858527
525	0.521483397	525	0.189583917
525.358282	0.519840966	525.358282	0.187893617
525.374225	0.519865681	525.374225	0.187835495
525.582817	0.520498396	525.582817	0.187774699
526.074845	0.521165127	526.074845	0.187510763
526.14969	0.521904944	526.14969	0.187791285
526.374225	0.521155878	526.374225	0.188983414
527.074845	0.521036188	527.074845	0.186531837
527.358282	0.522089055	527.358282	0.186846426
527.582817	0.522670349	527.582817	0.187021264
528.074845	0.523686157	528.074845	0.187327701
528.14969	0.523828004	528.14969	0.187372121
528.374225	0.524249931	528.374225	0.187510191
528.582817	0.5246478	528.582817	0.187652661
529.14969	0.525864739	529.14969	0.188233254
529.582817	0.526812521	529.582817	0.18923576
530	0.520810298	530	0.187798684
530.074845	0.521411251	530.074845	0.187088193
530.14969	0.522495592	530.14969	0.186744691
530.374225	0.52377426	530.374225	0.186756226
531.582817	0.522140625	531.582817	0.185476874
532.074845	0.524879908	532.074845	0.186233371
532.14969	0.525083746	532.14969	0.186294637
532.358282	0.525572182	532.358282	0.186436787
532.374225	0.525605744	532.374225	0.186446293
533.582817	0.527667635	533.582817	0.187000038
534.074845	0.528495672	534.074845	0.187265051
534.14969	0.528632481	534.14969	0.187315833
534.374225	0.529071796	534.374225	0.187497789
535	0.53048273	535	0.188589521
535.374225	0.528348726	535.374225	0.188441353
535.582817	0.525672216	535.582817	0.187308278
536.074845	0.528666551	536.074845	0.186664666
536.14969	0.528394953	536.14969	0.186981877

Final State: j’=1		Final State: j’=0	
E(cm⁻¹)	σ (Å²)	E(cm⁻¹)	σ (Å²)
536.374225	0.527169095	536.374225	0.188767899
537.074845	0.52646302	537.074845	0.185091571
537.358282	0.528111754	537.358282	0.185595443
537.582817	0.528784675	537.582817	0.18580699
538.074845	0.529790982	538.074845	0.186088124
538.14969	0.529918583	538.14969	0.186120314
538.374225	0.530280454	538.374225	0.186208008
538.582817	0.530596881	538.582817	0.186281314
539.14969	0.531412378	539.14969	0.186465012
539.582817	0.53203355	539.582817	0.186612496
540	0.532668921	540	0.186785212
540.074845	0.532789884	540.074845	0.186821848
540.14969	0.532914237	540.14969	0.186861067
540.374225	0.533310962	540.374225	0.186998066
541.582817	0.532805956	541.582817	0.187557283
542.074845	0.530163934	542.074845	0.18568685
542.14969	0.531509205	542.14969	0.18591598
542.358282	0.534299903	542.358282	0.18690458
542.374225	0.53416354	542.374225	0.187010288
543.582817	0.53346455	543.582817	0.185569631
544.074845	0.53460993	544.074845	0.185932514
544.14969	0.534753226	544.14969	0.185974586
544.374225	0.535160719	544.374225	0.186091596
545	0.536215995	545	0.186392586
545.374225	0.536845133	545.374225	0.186587501
545.582817	0.537208806	545.582817	0.186712004
546.074845	0.538136644	546.074845	0.187099183
546.14969	0.538288648	546.14969	0.187177603
546.374225	0.538753622	546.374225	0.187469423
547.074845	0.537747507	547.074845	0.188076923
547.358282	0.537207942	547.358282	0.18852091
547.582817	0.53578509	547.582817	0.187466314
548.074845	0.540133217	548.074845	0.188434587
548.14969	0.540552109	548.14969	0.188891072
548.374225	0.537221676	548.374225	0.188654576
548.582817	0.536125747	548.582817	0.187168188
549.14969	0.535832122	549.14969	0.187142816
549.582817	0.536025459	549.582817	0.184849162
550	0.5374204	550	0.185665269
550.074845	0.538053385	550.074845	0.186052052
550.14969	0.538557341	550.14969	0.186432014
550.374225	0.538197997	550.374225	0.187472805
551.582817	0.537780966	551.582817	0.18479123
552.074845	0.540246509	552.074845	0.185769572
552.14969	0.540579594	552.14969	0.185952733

Final State: j'=1		Final State: j'=0	
E(cm⁻¹)	σ (Å²)	E(cm⁻¹)	σ (Å²)
552.358282	0.541487156	552.358282	0.186589113
552.374225	0.541552693	552.374225	0.186648275
553.582817	0.535898789	553.582817	0.183698616
554.074845	0.539906538	554.074845	0.184617539
554.14969	0.54028817	554.14969	0.184740856
554.374225	0.541321194	554.374225	0.185106793
555	0.544102088	555	0.186411094
555.374225	0.546252686	555.374225	0.188038969
555.582817	0.547108362	555.582817	0.189736563
556.074845	0.535950654	556.074845	0.186222225
556.14969	0.530464951	556.14969	0.185813586
556.374225	0.530869853	556.374225	0.182897182
557.074845	0.535494615	557.074845	0.181899702
557.358282	0.540375905	557.358282	0.183087969
557.582817	0.541618929	557.582817	0.183570418
558.074845	0.543581338	558.074845	0.184363292
558.14969	0.543855885	558.14969	0.184484307
558.374225	0.544695557	558.374225	0.184886089
558.582817	0.545539476	558.582817	0.185361431
559.14969	0.546277169	559.14969	0.18812035
559.582817	0.534717762	559.582817	0.185689789
560	0.539810075	560	0.182168035
560.074845	0.54064779	560.074845	0.182373925
560.14969	0.541329345	560.14969	0.182554342
560.374225	0.542823599	560.374225	0.182984863
561.582817	0.546944533	561.582817	0.184333992
562.074845	0.548371949	562.074845	0.184901008
562.14969	0.548610815	562.14969	0.185007895
562.358282	0.549334599	562.358282	0.185360257
562.374225	0.549393875	562.374225	0.185391329
563.582817	0.543358057	563.582817	0.183535812
564.074845	0.546347482	564.074845	0.182690372
564.14969	0.546468807	564.14969	0.183216874
564.374225	0.544700428	564.374225	0.185046045
565	0.545286256	565	0.18252913
565.374225	0.547569152	565.374225	0.184506571
565.582817	0.549005829	565.582817	0.184708452
566.074845	0.548858155	566.074845	0.184010461
566.374225	0.551518317	566.374225	0.185377146
567.074845	0.551458439	567.074845	0.185047994
567.358282	0.554957859	567.358282	0.187400813
567.582817	0.549274664	567.582817	0.188592772
568.074845	0.539093919	568.074845	0.180218713
568.14969	0.54180903	568.14969	0.180496941
568.374225	0.546498927	568.374225	0.181536185

Final State: j’=1		Final State: j’=0	
E(cm⁻¹)	σ (Å²)	E(cm⁻¹)	σ (Å²)
568.582817	0.548916444	568.582817	0.182340746
569.14969	0.553765725	569.14969	0.185240593
569.582817	0.542214142	569.582817	0.181760135
570	0.550114252	570	0.182405173
570.074845	0.550756299	570.074845	0.18259349
570.14969	0.551322311	570.14969	0.18277443
570.374225	0.552767986	570.374225	0.183300768
571.582817	0.553080985	571.582817	0.183599403
572.074845	0.559079221	572.074845	0.187464476
572.14969	0.555101061	572.14969	0.18695102
572.358282	0.548739597	572.358282	0.185393161
572.374225	0.547778679	572.374225	0.185459835
573.582817	0.549953828	573.582817	0.181943125
574.074845	0.554530773	574.074845	0.182567347
574.14969	0.555054017	574.14969	0.184992242
574.374225	0.553860621	574.374225	0.181744366
575	0.555531365	575	0.183321891
575.374225	0.557986305	575.374225	0.185336038
576.074845	0.554384362	576.074845	0.182916143
576.14969	0.55473828	576.14969	0.183069568
576.374225	0.555676151	576.374225	0.183504876
577.074845	0.554740733	577.074845	0.18659113
577.358282	0.549344884	577.358282	0.180731955
577.582817	0.552067287	577.582817	0.181925258
578.074845	0.553621859	578.074845	0.182271186
578.14969	0.553835806	578.14969	0.18231405
578.374225	0.554403772	578.374225	0.182438611
578.582817	0.554850449	578.582817	0.182546079
579.14969	0.555804588	579.14969	0.182804378
579.582817	0.556373748	579.582817	0.182971901
580	0.556983068	580	0.183151852
580.074845	0.557125855	580.074845	0.183197384
580.14969	0.557288686	580.14969	0.183251795
580.374225	0.557988445	580.374225	0.183513534
581.582817	0.555367194	581.582817	0.182230923
582.074845	0.554847679	582.074845	0.181602656
582.14969	0.555161652	582.14969	0.181644339
582.358282	0.555834908	582.358282	0.181777949
582.374225	0.555875682	582.374225	0.181786715
583.582817	0.557613176	583.582817	0.182059386
584.14969	0.558154616	584.14969	0.18208807
584.374225	0.558357683	584.374225	0.182095692
585	0.558898954	585	0.182115542
585.374225	0.559185282	585.374225	0.182124053
585.582817	0.559315358	585.582817	0.182121423

Final State: j’=1		Final State: j’=0	
E(cm⁻¹)	σ (Å²)	E(cm⁻¹)	σ (Å²)
586.14969	0.559512477	586.14969	0.182041415
586.374225	0.559568777	586.374225	0.181983008
587.074845	0.559940423	587.074845	0.181837917
587.358282	0.560155887	587.358282	0.181806086
587.582817	0.560339	587.582817	0.181788522
588.14969	0.560842462	588.14969	0.181770156
588.374225	0.561067221	588.374225	0.18177651
588.582817	0.561304637	588.582817	0.181795931
589.14969	0.56261205	589.14969	0.182126781
589.582817	0.560838448	589.582817	0.181469431
590	0.560986332	590	0.181091913
590.14969	0.561361991	590.14969	0.181181162
590.374225	0.561695334	590.374225	0.181236882
591.582817	0.562717761	591.582817	0.181209752
592.074845	0.563060673	592.074845	0.181163045
592.14969	0.563111804	592.14969	0.181155355
592.358282	0.563254362	592.358282	0.181133733
592.374225	0.563265041	592.374225	0.18113198
593.582817	0.564082805	593.582817	0.181000306
594.14969	0.564466036	594.14969	0.180937592
594.374225	0.56461802	594.374225	0.180912776
595	0.565043025	595	0.180844056
595.374225	0.565298339	595.374225	0.180803352
595.582817	0.56544147	595.582817	0.18078097
596.14969	0.565833406	596.14969	0.180721285
596.374225	0.56598909	596.374225	0.180697758
597.074845	0.566433386	597.074845	0.18060757
597.358282	0.566552866	597.358282	0.180547824
597.582817	0.566630415	597.582817	0.180494292
598.14969	0.566917147	598.14969	0.180391455
598.374225	0.567063157	598.374225	0.180362496
598.582817	0.56720437	598.582817	0.180337828
599.14969	0.567593152	599.14969	0.18027342
599.582817	0.56788792	599.582817	0.180223671
600	0.568169408	600	0.180174882
600.14969	0.568269624	600.14969	0.180157061
600.374225	0.568419832	600.374225	0.180130265
601.582817	0.56922258	601.582817	0.179983386
602.074845	0.56954799	602.074845	0.179922769
602.14969	0.569597223	602.14969	0.17991342
602.358282	0.56973509	602.358282	0.17988763
602.374225	0.569745408	602.374225	0.17988557
603.582817	0.570541454	603.582817	0.179734309
604.14969	0.570913253	604.14969	0.179662307
604.374225	0.571059988	604.374225	0.179633491

Final State: j'=1		Final State: j'=0	
E(cm⁻¹)	σ (Å²)	E(cm⁻¹)	σ (Å²)
605	0.571466874	605	0.179552256
605.374225	0.571708044	605.374225	0.179502753
605.582817	0.57184202	605.582817	0.179474982
606.14969	0.572204813	606.14969	0.179398996
606.374225	0.572348266	606.374225	0.179368812
607.074845	0.572796797	607.074845	0.179274904
607.358282	0.572978767	607.358282	0.179237036
607.582817	0.573122922	607.582817	0.179207026
608.14969	0.573488517	608.14969	0.179131725
608.374225	0.573633715	608.374225	0.179101962
608.582817	0.573768692	608.582817	0.179074358
609.14969	0.574136162	609.14969	0.178999369
609.582817	0.574417276	609.582817	0.178942095
610	0.574688652	610	0.178886998
610.14969	0.574785741	610.14969	0.178867091
610.374225	0.574931795	610.374225	0.178837376
611.582817	0.575718362	611.582817	0.178676955
612.074845	0.576038869	612.074845	0.178611508
612.14969	0.57608742	612.14969	0.178601429
612.358282	0.576223457	612.358282	0.178573678
613.582817	0.577020167	613.582817	0.178409507
614.14969	0.577388766	614.14969	0.178333155
614.374225	0.577534705	614.374225	0.178302851
615	0.577941557	615	0.178218275
615.374225	0.57818441	615.374225	0.178167423
615.582817	0.578319846	615.582817	0.17813909
616.14969	0.578687787	616.14969	0.178061936
616.374225	0.578833569	616.374225	0.178031335
617.074845	0.579288472	617.074845	0.177935753
617.358282	0.579472406	617.358282	0.177896958
617.582817	0.579617873	617.582817	0.177866128
618.14969	0.579985732	618.14969	0.177788371
618.374225	0.580131396	618.374225	0.177757548
618.582817	0.580266762	618.582817	0.177728901
619.14969	0.580634619	619.14969	0.177650875
619.582817	0.580915721	619.582817	0.177591187
620	0.58118675	620	0.177533704
620.14969	0.58128369	620.14969	0.17751294
620.374225	0.581429432	620.374225	0.177481895
621.582817	0.582213972	621.582817	0.177314424
622.074845	0.582533632	622.074845	0.177246127
622.14969	0.582581979	622.14969	0.17723563
622.358282	0.582717632	622.358282	0.17720669
622.374225	0.582727756	622.374225	0.177204368
623.582817	0.583512322	623.582817	0.177035732

Final State: j’=1		Final State: j’=0	
E(cm⁻¹)	σ (Å²)	E(cm⁻¹)	σ (Å²)
624.14969	0.583880307	624.14969	0.176956382
624.374225	0.584026022	624.374225	0.176924892
625	0.584432422	625	0.176837132
625.374225	0.584675058	625.374225	0.176784399
625.582817	0.584810385	625.582817	0.176755031
626.14969	0.585178217	626.14969	0.176675107
626.374225	0.585323925	626.374225	0.176643408
627.074845	0.58577862	627.074845	0.176544434
627.358282	0.585962511	627.358282	0.176504287
627.582817	0.586107894	627.582817	0.176472354
628.14969	0.586475526	628.14969	0.176391881
628.374225	0.58662113	628.374225	0.176359951
628.582817	0.58675639	628.582817	0.176330279
629.14969	0.587123909	629.14969	0.176249532
629.582817	0.587404685	629.582817	0.17618773
630	0.587675365	630	0.176128223
630.14969	0.587772149	630.14969	0.17610671
630.374225	0.587917676	630.374225	0.176074586
631.582817	0.588700828	631.582817	0.175901226
632.074845	0.589019815	632.074845	0.175830565
632.14969	0.58906807	632.14969	0.175819687
632.358282	0.589203431	632.358282	0.175789744
632.374225	0.589213509	632.374225	0.175787351
633.582817	0.58999618	633.582817	0.17561289
634.14969	0.590363233	634.14969	0.175530828
634.374225	0.590508616	634.374225	0.175498286
635	0.590913846	635	0.175407579
635.374225	0.591155797	635.374225	0.175353076
635.582817	0.591290756	635.582817	0.175322738
636.14969	0.591657538	636.14969	0.175240174
636.374225	0.591802797	636.374225	0.175207429
637.074845	0.592256188	637.074845	0.175105229
637.358282	0.592439488	637.358282	0.175063768
637.582817	0.592584449	637.582817	0.175030814
638.14969	0.592950887	638.14969	0.17494775
638.374225	0.59309606	638.374225	0.174914811
638.582817	0.593230892	638.582817	0.174884188
639.14969	0.593597194	639.14969	0.174800887
639.582817	0.593877032	639.582817	0.174737144
640	0.594146765	640	0.174675772
640.14969	0.594243195	640.14969	0.174653596
640.374225	0.594388204	640.374225	0.174620466
641.582817	0.59516851	641.582817	0.17444177
642.074845	0.595486309	642.074845	0.174368942
642.14969	0.595534361	642.14969	0.174357742

Final State: j’=1		Final State: j’=0	
E(cm⁻¹)	σ (Å²)	E(cm⁻¹)	σ (Å²)
642.358282	0.595669189	642.358282	0.174326897
642.374225	0.595679235	642.374225	0.174324433
643.582817	0.596458788	643.582817	0.174144732
644.14969	0.596824251	644.14969	0.174060245
644.374225	0.596968988	644.374225	0.174026744
645	0.597372525	645	0.173933372
645.374225	0.597613365	645.374225	0.17387729
645.582817	0.597747757	645.582817	0.173846069
646.14969	0.598112819	646.14969	0.173761129
646.374225	0.59825741	646.374225	0.173727442
647.074845	0.598708655	647.074845	0.173622324
647.358282	0.598891076	647.358282	0.17357971
647.582817	0.599035241	647.582817	0.173545813
648.14969	0.599400014	648.14969	0.17346043
648.374225	0.599544392	648.374225	0.17342658
648.582817	0.599678515	648.582817	0.173395108
649.14969	0.600042977	649.14969	0.173309507
649.582817	0.600321371	649.582817	0.173244019
650	0.600589686	650	0.173180975
650.14969	0.600685596	650.14969	0.173158191
650.374225	0.600829801	650.374225	0.173124163
651.582817	0.601605847	651.582817	0.172940701
652.074845	0.601921854	652.074845	0.172865958
652.14969	0.601969608	652.14969	0.172854462
652.358282	0.602103696	652.358282	0.172822803
652.374225	0.602113665	652.374225	0.172820268
653.582817	0.602888652	653.582817	0.172635916
654.14969	0.603251948	654.14969	0.172549268
654.374225	0.603395802	654.374225	0.172514913
655	0.603796854	655	0.17241919
655.374225	0.604036185	655.374225	0.172361704
655.582817	0.604169716	655.582817	0.172329701
656.14969	0.604532501	656.14969	0.172242659
656.374225	0.604676166	656.374225	0.172208149
657.074845	0.60512452	657.074845	0.172100464
657.358282	0.605305633	657.358282	0.172056807
657.582817	0.60544892	657.582817	0.172022101
658.14969	0.605811162	658.14969	0.171934664
658.374225	0.605954563	658.374225	0.171900007
658.582817	0.606087887	658.582817	0.171867798
659.14969	0.606449749	659.14969	0.171780158
659.582817	0.60672624	659.582817	0.171713139
660	0.606992843	660	0.171648657
660.14969	0.607087899	660.14969	0.171625337
660.374225	0.607231149	660.374225	0.171590525

Final State: j'=1		Final State: j'=0	
E(cm⁻¹)	σ (Å²)	E(cm⁻¹)	σ (Å²)
661.582817	0.608001565	661.582817	0.171402878
662.074845	0.608315249	662.074845	0.17132646
662.14969	0.608362629	662.14969	0.171314702
662.358282	0.608495728	662.358282	0.171282341
662.374225	0.608505615	662.374225	0.171279763
663.582817	0.609274805	663.582817	0.171091345
664.14969	0.609635539	664.14969	0.171002921
664.374225	0.609777998	664.374225	0.170967731
665	0.610175964	665	0.17086996
665.374225	0.610413444	665.374225	0.170811252
665.582817	0.610545876	665.582817	0.170778572
666.374225	0.611048251	666.374225	0.170654489
667.074845	0.611492955	667.074845	0.170544583
667.358282	0.611672672	667.358282	0.170500043
667.582817	0.611814754	667.582817	0.170464625
668.374225	0.612316221	668.374225	0.170340074
668.582817	0.612448322	668.582817	0.170307227
669.14969	0.612807498	669.14969	0.170217979
669.582817	0.613081319	669.582817	0.170149533
670	0.61334551	670	0.170083763
670.374225	0.613581891	670.374225	0.17002453
671.582817	0.614345608	671.582817	0.169833309
672.074845	0.614656491	672.074845	0.169755468
672.358282	0.614835484	672.358282	0.169710529
672.374225	0.614845249	672.374225	0.169707892
673.582817	0.615607426	673.582817	0.169516029
674.14969	0.615964995	674.14969	0.169426018
674.374225	0.61610612	674.374225	0.169390194
675	0.616500456	675	0.169290677
675.374225	0.616735714	675.374225	0.169230954
676.374225	0.617364734	676.374225	0.169071476
677.074845	0.617805276	677.074845	0.168959688
677.358282	0.61798324	677.358282	0.168914388
677.582817	0.618123983	677.582817	0.168878383
678.374225	0.618620704	678.374225	0.16875175
678.582817	0.618751648	678.582817	0.168718358
679.14969	0.619107334	679.14969	0.168627633
679.582817	0.619378619	679.582817	0.168558081
680	0.619640277	680	0.168491261
680.374225	0.619874412	680.374225	0.168431084
681.582817	0.620630865	681.582817	0.168236853
682.074845	0.620938841	682.074845	0.168157785
682.358282	0.621116048	682.358282	0.16811216
682.374225	0.621125728	682.374225	0.168109483
683.582817	0.621880777	683.582817	0.167914698

Final State: j’=1		Final State: j’=0	
E(cm⁻¹)	σ (Å²)	E(cm⁻¹)	σ (Å²)
684.14969	0.622234893	684.14969	0.167823326
684.374225	0.62237475	684.374225	0.16778697
685	0.622765374	685	0.167685983
685.374225	0.622998458	685.374225	0.167625378
685.582817	0.623128493	685.582817	0.167591645
686.374225	0.623621654	686.374225	0.167463562
687.074845	0.624058227	687.074845	0.167350165
687.358282	0.624234693	687.358282	0.167304218
687.582817	0.624374193	687.582817	0.1672677
688.374225	0.624866629	688.374225	0.167139264
688.582817	0.624996389	688.582817	0.167105389
689.14969	0.625349186	689.14969	0.167013382
689.582817	0.625618211	689.582817	0.166942852
690	0.625877805	690	0.166875081
690.374225	0.626110102	690.374225	0.166814053
691.582817	0.626861061	691.582817	0.166617073
692.074845	0.627166976	692.074845	0.16653688
692.358282	0.627343053	692.358282	0.166490598
692.374225	0.627352708	692.374225	0.166487888
693.582817	0.628103591	693.582817	0.166290288
694.14969	0.628456136	694.14969	0.16619757
694.374225	0.628595443	694.374225	0.166160677
695	0.628984836	695	0.166058155
695.374225	0.629217387	695.374225	0.165996621
695.582817	0.629347212	695.582817	0.16596236
696.374225	0.629840151	696.374225	0.16583222
697.074845	0.630277531	697.074845	0.165716902
697.358282	0.63045458	697.358282	0.165670148
697.582817	0.63059468	697.582817	0.165632975
698.374225	0.63109049	698.374225	0.165502122
698.582817	0.631221483	698.582817	0.16546757
699.14969	0.631578546	699.14969	0.165373625
699.582817	0.631851907	699.582817	0.165301508
700	0.632116793	700	0.165232084
700.374225	0.632354954	700.374225	0.165169471
701.582817	0.633135554	701.582817	0.164966269
702.074845	0.633461024	702.074845	0.164882839
702.358282	0.633651549	702.358282	0.164834414
702.374225	0.633662029	702.374225	0.164831589
703.582817	0.634531532	703.582817	0.164620848
704.14969	0.63503347	704.14969	0.164518225
704.374225	0.635292323	704.374225	0.164477426
705	0.636364729	705	0.164495137
705.374225	0.63680034	705.374225	0.164449932
705.582817	0.636725693	705.582817	0.164546514

Final State: j’=1		Final State: j’=0	
E(cm⁻¹)	σ (Å²)	E(cm⁻¹)	σ (Å²)
706.374225	0.636234017	706.374225	0.16422102
707.074845	0.63776465	707.074845	0.164286567
707.358282	0.636698894	707.358282	0.164229528
707.582817	0.636596654	707.582817	0.164103269
708.374225	0.637361743	708.374225	0.163875494
708.582817	0.637664713	708.582817	0.163827297
709.14969	0.6384063	709.14969	0.163831579
709.582817	0.638936871	709.582817	0.163973207
710	0.63820996	710	0.163718698
710.374225	0.638891766	710.374225	0.163735184
711.582817	0.639026616	711.582817	0.163397774
712.074845	0.639678649	712.074845	0.163287185
712.358282	0.640293935	712.358282	0.163253943
713.582817	0.640240499	713.582817	0.163090499
714.14969	0.641048718	714.14969	0.16335041
714.374225	0.640256337	714.374225	0.163046149
715	0.640860618	715	0.162846462
715.374225	0.641218278	715.374225	0.162765756
715.582817	0.641431651	715.582817	0.162723321
716.374225	0.642953083	716.374225	0.162621228
717.074845	0.642388599	717.074845	0.162652923
717.358282	0.643061934	717.358282	0.162523108
717.582817	0.642738768	717.582817	0.162508257
718.374225	0.64246062	718.374225	0.162409314
718.582817	0.642657464	718.582817	0.162314205
719.14969	0.64317909	719.14969	0.162174709
719.582817	0.64354261	719.582817	0.162086979
720	0.643901373	720	0.162006608
720.374225	0.644259081	720.374225	0.161936265
721.582817	0.646176466	721.582817	0.162137313
722.074845	0.645150739	722.074845	0.161745632
722.358282	0.646379944	722.358282	0.161679083
722.374225	0.646549062	722.374225	0.161688627
723.582817	0.645584543	723.582817	0.161471361
724.14969	0.646053719	724.14969	0.16135282
724.374225	0.646227465	724.374225	0.161309516
725	0.646708361	725	0.161193218
725.374225	0.647006904	725.374225	0.161124968
725.582817	0.647183861	725.582817	0.161087344
726.374225	0.648115919	726.374225	0.160960051
727.074845	0.648429402	727.074845	0.161204527
727.358282	0.649074208	727.358282	0.16098434
727.582817	0.648384406	727.582817	0.160851119
728.374225	0.649520605	728.374225	0.16097148
728.582817	0.648235222	728.582817	0.160712372

Final State: j'=1		Final State: j'=0	
E(cm⁻¹)	σ (Å²)	E(cm⁻¹)	σ (Å²)
729.14969	0.648814632	729.14969	0.160547222
729.582817	0.649159501	729.582817	0.160460569
730	0.649469641	730	0.16038271
730.374225	0.649740535	730.374225	0.16031468
731.582817	0.650639413	731.582817	0.160100307
732.074845	0.651069453	732.074845	0.160015638
732.358282	0.651382219	732.358282	0.159971019
732.374225	0.651402271	732.374225	0.159968671
733.582817	0.652064014	733.582817	0.159849929
734.14969	0.651943586	734.14969	0.159811606
734.374225	0.652356755	734.374225	0.159814881
735	0.652211924	735	0.159579914
735.374225	0.652555752	735.374225	0.159498129
735.582817	0.652729772	735.582817	0.159456714
736.374225	0.653361624	736.374225	0.159307087
737.074845	0.653946166	737.074845	0.159176223
737.358282	0.654209943	737.358282	0.159122369
737.582817	0.654443146	737.582817	0.159078948
738.374225	0.656124272	738.374225	0.158982021
738.582817	0.655953824	738.582817	0.158996847
739.14969	0.655980832	739.14969	0.159040708
739.582817	0.65641793	739.582817	0.158872138
740	0.657902923	740	0.159202595
740.374225	0.656682394	740.374225	0.158943436
741.582817	0.656668023	741.582817	0.158519551
742.074845	0.65906856	742.074845	0.158677732
742.358282	0.657886439	742.358282	0.158662231
742.374225	0.657929493	742.374225	0.15865764
743.582817	0.657461338	743.582817	0.158185236
744.14969	0.659273739	744.14969	0.158092113
744.374225	0.660743717	744.374225	0.158309137
745	0.657545577	745	0.158238573
745.374225	0.657815859	745.374225	0.158007506
745.582817	0.658061754	745.582817	0.157936789
746.374225	0.659125314	746.374225	0.157738379
747.074845	0.661816061	747.074845	0.157723812
747.358282	0.663403578	747.358282	0.158361009
747.582817	0.663041116	747.582817	0.158235484
748.374225	0.659326919	748.374225	0.157578825
748.582817	0.658887358	748.582817	0.157653347
749.14969	0.659768294	749.14969	0.157342798
749.582817	0.660302353	749.582817	0.157240063
750	0.660898004	750	0.157147779
750.374225	0.661783813	750.374225	0.157077254
751.582817	0.660668743	751.582817	0.157088691

Final State: j’=1		Final State: j’=0	
E(cm⁻¹)	σ (Å²)	E(cm⁻¹)	σ (Å²)
752.074845	0.661244058	752.074845	0.156907064
752.358282	0.66153625	752.358282	0.156835584
752.374225	0.661552085	752.374225	0.156831752
753.582817	0.662782403	753.582817	0.156578731
754.14969	0.663658919	754.14969	0.156471962
754.374225	0.664328497	754.374225	0.156447657
755	0.66505418	755	0.15707717
755.374225	0.663105031	755.374225	0.156663006
755.582817	0.664308694	755.582817	0.156421039
756.374225	0.66647047	756.374225	0.156824593
757.074845	0.665126322	757.074845	0.156040535
757.358282	0.666039837	757.358282	0.156174662
757.582817	0.664922473	757.582817	0.155998484
758.374225	0.665778308	758.374225	0.155999563
758.582817	0.66587278	758.582817	0.155836958
759.14969	0.670765385	759.14969	0.156292108
759.582817	0.664944957	759.582817	0.15612049
760	0.665277295	760	0.155696979
760.374225	0.666103153	760.374225	0.155544767
761.582817	0.666329224	761.582817	0.155422046
762.074845	0.667118818	762.074845	0.155267702
762.358282	0.667785292	762.358282	0.155188292
762.374225	0.667837202	762.374225	0.155183719
763.582817	0.671018404	763.582817	0.155666731
764.14969	0.667817241	764.14969	0.155262891
764.374225	0.66823411	764.374225	0.155087861
765	0.667924559	765	0.155257294
765.374225	0.667729848	765.374225	0.154880751
765.582817	0.668718807	765.582817	0.154732115
766.374225	0.669487007	766.374225	0.154647436
767.074845	0.67323675	767.074845	0.155524042
767.358282	0.670000465	767.358282	0.154595006
767.582817	0.670371127	767.582817	0.154517646
768.374225	0.672040934	768.374225	0.154415257
768.582817	0.674322585	768.582817	0.154961219
769.14969	0.671250497	769.14969	0.15439097
769.582817	0.671499008	769.582817	0.154288213
770	0.671777536	770	0.154204928
770.374225	0.672050279	770.374225	0.15414231
771.582817	0.672967177	771.582817	0.153984196
772.074845	0.67364235	772.074845	0.154010161
772.358282	0.673296445	772.358282	0.153881486
772.374225	0.673378351	772.374225	0.153870352
773.582817	0.673678984	773.582817	0.153755895
774.14969	0.673998349	774.14969	0.153624062

Final State: j’=1		Final State: j’=0	
E(cm⁻¹)	σ (Å²)	E(cm⁻¹)	σ (Å²)
774.374225	0.674137464	774.374225	0.153587179
775	0.674507734	775	0.153492221
775.374225	0.674721954	775.374225	0.153437242
776.374225	0.675291775	776.374225	0.153295424
777.074845	0.675705382	777.074845	0.153205898
777.358282	0.675870388	777.358282	0.153173209
778.374225	0.676360763	778.374225	0.153035724
778.582817	0.676457374	778.582817	0.15300375
779.14969	0.676733727	779.14969	0.15291767
780	0.67718434	780	0.152796616
780.374225	0.677417919	780.374225	0.152753531
782.074845	0.678126307	782.074845	0.152487139
782.358282	0.678279389	782.358282	0.152447618
782.374225	0.678287882	782.374225	0.152445258
783.582817	0.678905507	783.582817	0.152275949
784.14969	0.679187632	784.14969	0.1521958
784.374225	0.679298871	784.374225	0.152163864
785	0.679607745	785	0.152075401
785.374225	0.679792112	785.374225	0.152022345
786.374225	0.680283812	786.374225	0.151881496
787.074845	0.680628391	787.074845	0.151783774
787.358282	0.680768201	787.358282	0.15174454
788.374225	0.681275572	788.374225	0.15160846
788.582817	0.681380288	788.582817	0.151582096
789.14969	0.681648319	789.14969	0.151504911
790	0.682034682	790	0.151377934
790.374225	0.682210941	790.374225	0.151323685
792.074845	0.683019448	792.074845	0.151084563
792.358282	0.683154034	792.358282	0.151045126
792.374225	0.683161739	792.374225	0.151042759
793.582817	0.683733988	793.582817	0.150875509
794.14969	0.684001602	794.14969	0.150797377
794.374225	0.684107544	794.374225	0.150766339
795	0.684401931	795	0.150680633
795.374225	0.68457788	795.374225	0.150629282
796.374225	0.685046213	796.374225	0.150492987
797.074845	0.68537264	797.074845	0.150397922
797.358282	0.685504297	797.358282	0.150359408
798.374225	0.685974214	798.374225	0.150221175
798.582817	0.686070159	798.582817	0.150192997
799.14969	0.686330881	799.14969	0.150116063
800	0.686720765	800	0.150000953
800.374225	0.686892125	800.374225	0.149950259
802.074845	0.687666577	802.074845	0.14972187
802.358282	0.687795102	802.358282	0.149683954

Final State: j’=1		Final State: j’=0	
E(cm⁻¹)	σ (Å²)	E(cm⁻¹)	σ (Å²)
802.374225	0.687802548	802.374225	0.149681654
803.582817	0.688348737	803.582817	0.149520694
804.14969	0.688604031	804.14969	0.149445422
804.374225	0.688705204	804.374225	0.149415489
805	0.68898571	805	0.149332863
805.374225	0.689153466	805.374225	0.149283288
806.374225	0.689599606	806.374225	0.149151645
807.074845	0.689910622	807.074845	0.149059917
807.358282	0.690036264	807.358282	0.149022812
808.374225	0.690485403	808.374225	0.148889996
808.582817	0.690576994	808.582817	0.148863012
809.14969	0.690826218	809.14969	0.148789311
810	0.691198739	810	0.148679094
810.374225	0.691362498	810.374225	0.148630533
812.074845	0.692101163	812.074845	0.148411878
812.358282	0.69222372	812.358282	0.148375566
813.582817	0.692751209	813.582817	0.14821923
814.14969	0.692994297	814.14969	0.148147155
814.374225	0.693090742	814.374225	0.148118469
815	0.693357628	815	0.148039401
815.374225	0.693517389	815.374225	0.147991919
816.374225	0.69394186	816.374225	0.147865966
817.074845	0.694237553	817.074845	0.147778272
817.358282	0.694357036	817.358282	0.1477428
818.374225	0.694784201	818.374225	0.147615848
818.582817	0.694871152	818.582817	0.147590102
819.14969	0.695108032	819.14969	0.147519719
820	0.695462014	820	0.147414512
820.374225	0.695617725	820.374225	0.147368143
822.074845	0.69631903	822.074845	0.147159633
822.358282	0.696435362	822.358282	0.147125019
822.374225	0.69644236	822.374225	0.147122864
823.582817	0.696935892	823.582817	0.146976047
824.14969	0.697166446	824.14969	0.146907397
824.374225	0.69725806	824.374225	0.14688005
825	0.697510951	825	0.146804801
825.374225	0.697662546	825.374225	0.146759571
826.374225	0.698064779	826.374225	0.146639721
827.074845	0.698344729	827.074845	0.146556348
827.358282	0.698457876	827.358282	0.146522615
828.374225	0.698862483	828.374225	0.146401905
828.582817	0.698944612	828.582817	0.146377478
829.14969	0.699168801	829.14969	0.146310612
830	0.699503689	830	0.146210693
830.374225	0.699651134	830.374225	0.146166635

Final State: j’=1		Final State: j’=0	
E(cm⁻¹)	σ (Å²)	E(cm⁻¹)	σ (Å²)
832.074845	0.700313964	832.074845	0.145968835
832.358282	0.700423893	832.358282	0.145936009
832.374225	0.700430674	832.374225	0.145933926
833.582817	0.700896691	833.582817	0.145794797
834.14969	0.701114399	834.14969	0.145729758
834.374225	0.701201068	834.374225	0.145703806
835	0.701439582	835	0.145632586
835.374225	0.701582825	835.374225	0.145589722
836.374225	0.701962279	836.374225	0.145476296
837.074845	0.702226118	837.074845	0.145397471
837.358282	0.702332734	837.358282	0.145365574
838.374225	0.702714267	838.374225	0.145251417
838.582817	0.702791458	838.582817	0.145228373
839.14969	0.703002644	839.14969	0.145165195
840	0.703318021	840	0.145070832
840.374225	0.703457015	840.374225	0.145029191
842.074845	0.704080523	842.074845	0.144842604
842.358282	0.704183901	842.358282	0.14481165
842.374225	0.704190478	842.374225	0.144809641
843.582817	0.704628401	843.582817	0.144678548
844.14969	0.704832997	844.14969	0.144617272
844.374225	0.704914619	844.374225	0.144592782
845	0.705138464	845	0.144525766
845.374225	0.705273189	845.374225	0.144485372
846.374225	0.705629398	846.374225	0.14437864
847.074845	0.705876766	847.074845	0.144304554
847.358282	0.705976826	847.358282	0.144274564
848.374225	0.70633484	848.374225	0.144167224
848.582817	0.706406995	848.582817	0.144145626
849.14969	0.706604942	849.14969	0.144086285
850	0.70690044	850	0.143997694
850.374225	0.707030867	850.374225	0.143958559
852.074845	0.707614357	852.074845	0.143783609
852.358282	0.707711077	852.358282	0.143754599
852.374225	0.707717455	852.374225	0.143752661
853.582817	0.70812683	853.582817	0.143629891
854.14969	0.708318088	854.14969	0.143572518
854.374225	0.708394608	854.374225	0.14354954
855	0.708603548	855	0.143486874
855.374225	0.70872964	855.374225	0.143449031
856.374225	0.709062295	856.374225	0.143349221
857.074845	0.70929292	857.074845	0.143280031
857.358282	0.709386306	857.358282	0.143252007
858.374225	0.709720495	858.374225	0.143151707
858.582817	0.709787536	858.582817	0.143131597

Final State: j’=1		Final State: j’=0	
E(cm⁻¹)	σ (Å²)	E(cm⁻¹)	σ (Å²)
859.14969	0.709972072	859.14969	0.143076217
860	0.710247449	860	0.142993567
860.374225	0.710369181	860.374225	0.142957019
862.074845	0.710912153	862.074845	0.142794048
862.358282	0.711002159	862.358282	0.142767031
862.374225	0.711008353	862.374225	0.142765169
863.582817	0.711388809	863.582817	0.142650965
864.14969	0.711566583	864.14969	0.142597595
864.374225	0.711637964	864.374225	0.142576165
865	0.711831819	865	0.142517973
865.374225	0.711949193	865.374225	0.142482741
866.374225	0.712258036	866.374225	0.142390028
867.074845	0.712471759	867.074845	0.142325863
867.358282	0.712558375	867.358282	0.142299861
868.374225	0.712868524	868.374225	0.142206757
868.582817	0.71293039	868.582817	0.142188179
869.14969	0.713101396	869.14969	0.142136845
870	0.713356451	870	0.142060282
870.374225	0.713469448	870.374225	0.142026372
872.074845	0.713971535	872.074845	0.141875646
872.358282	0.714054759	872.358282	0.141850664
872.374225	0.714060771	872.374225	0.141848875
873.582817	0.714412084	873.582817	0.14174341
874.14969	0.714576281	874.14969	0.141694128
874.374225	0.714642485	874.374225	0.141674278
875	0.714821136	875	0.141620643
875.374225	0.714929742	875.374225	0.141588067
876.374225	0.71521459	876.374225	0.141502585
877.074845	0.71541128	877.074845	0.141443539
877.358282	0.715491097	877.358282	0.141419588
878.374225	0.715777079	878.374225	0.141333811
878.582817	0.715833721	878.582817	0.14131679
879.14969	0.715991098	879.14969	0.141269574
880	0.716225712	880	0.141199187
880.374225	0.716329932	880.374225	0.141167955
882.074845	0.716790907	882.074845	0.141029654
882.358282	0.71686731	882.358282	0.14100674
882.374225	0.716873157	882.374225	0.141005024
883.582817	0.717195165	883.582817	0.140908422
884.14969	0.717345715	884.14969	0.140863274
884.374225	0.717406737	884.374225	0.14084502
885	0.717570092	885	0.140796005
885.374225	0.717669907	885.374225	0.140766121
886.374225	0.71793066	886.374225	0.140687952
887.074845	0.718110261	887.074845	0.14063408

Final State: j'=1		Final State: j'=0	
E(cm⁻¹)	σ (Å²)	E(cm⁻¹)	σ (Å²)
887.358282	0.718183245	887.358282	0.140612205
888.374225	0.718444931	888.374225	0.140533819
888.582817	0.718496307	888.582817	0.140518374
889.14969	0.718640017	889.14969	0.140475314
890	0.718854128	890	0.140411161
890.374225	0.718949533	890.374225	0.140382627
892.074845	0.7193692	892.074845	0.140256858
892.358282	0.71943876	892.358282	0.140236025
892.374225	0.719444456	892.374225	0.140234376
893.582817	0.719737012	893.582817	0.140146702
894.14969	0.719873846	894.14969	0.140105719
894.374225	0.719929674	894.374225	0.140089065
895	0.720077651	895	0.140044695
895.374225	0.720168651	895.374225	0.140017516
896.374225	0.720405181	896.374225	0.139946699
897.074845	0.720567552	897.074845	0.139898021
897.358282	0.720633674	897.358282	0.13987823
898.374225	0.720870964	898.374225	0.139807271
898.582817	0.720917047	898.582817	0.139793409
899.14969	0.721047011	899.14969	0.139754513
900	0.721240412	900	0.139696619
900.374225	0.721326973	900.374225	0.139670784
902.074845	0.721704985	902.074845	0.139557564
902.358282	0.721767617	902.358282	0.139538814
902.374225	0.721773171	902.374225	0.139537234
903.582817	0.722035955	903.582817	0.139458485
904.14969	0.722158926	904.14969	0.139421671
904.374225	0.722209481	904.374225	0.139406618
905	0.722341872	905	0.139366891
905.374225	0.722423923	905.374225	0.139342409
906.374225	0.722635847	906.374225	0.139278933
907.074845	0.722780674	907.074845	0.139235446
907.358282	0.722839779	907.358282	0.139217734
908.374225	0.723052115	908.374225	0.139154176
908.582817	0.723092746	908.582817	0.139141889
909.14969	0.723208553	909.14969	0.139107148
910	0.723380709	910	0.13905548
910.374225	0.723455863	910.374225	0.139032906
912.074845	0.723792725	912.074845	0.138931614
912.358282	0.723848108	912.358282	0.138914938
913.582817	0.724084797	913.582817	0.13884356
914.14969	0.724192909	914.14969	0.138810886
915	0.724353297	915	0.138762318
915.374225	0.724423177	915.374225	0.138741106
917.074845	0.724734977	917.074845	0.138646019

Final State: j'=1		Final State: j'=0	
E(cm⁻¹)	σ (Å²)	E(cm⁻¹)	σ (Å²)
917.358282	0.724785939	917.358282	0.138630375
918.582817	0.725002426	918.582817	0.138563482
919.14969	0.725100328	919.14969	0.138532894
920	0.725243707	920	0.138487473
920.374225	0.725305246	920.374225	0.138467669
922.074845	0.725563813	922.074845	0.138379257
922.358282	0.725600657	922.358282	0.138364861
923.582817	0.725659157	923.582817	0.138309415
924.14969	0.725834472	924.14969	0.138285883
925	0.726197098	925	0.13823055
925.374225	0.726145252	925.374225	0.138213505
927.074845	0.726489702	927.074845	0.138127497
927.358282	0.726474268	927.358282	0.13811447
928.582817	0.727080015	928.582817	0.13806023
929.14969	0.726778539	929.14969	0.138039173
930	0.727270695	930	0.137993513
930.374225	0.727113196	930.374225	0.137970892
932.074845	0.727577191	932.074845	0.137904255
932.358282	0.727468262	932.358282	0.137887128
933.582817	0.727679201	933.582817	0.137827827
934.14969	0.727673748	934.14969	0.137802049
935	0.727661167	935	0.137761925
935.374225	0.727585941	935.374225	0.137745738
937.074845	0.728268047	937.074845	0.13770654
937.358282	0.728336452	937.358282	0.13767095
938.582817	0.72832119	938.582817	0.137617794
939.14969	0.728346368	939.14969	0.137592984
940	0.72828554	940	0.137551036
940.374225	0.728839216	940.374225	0.137535015
942.074845	0.728993313	942.074845	0.137488409
942.358282	0.728949409	942.358282	0.137476373
943.582817	0.728994961	943.582817	0.137427561
944.14969	0.729031181	944.14969	0.137404928
945	0.729061724	945	0.137367925
945.374225	0.729037556	945.374225	0.13734599
947.074845	0.729662219	947.074845	0.137333888
947.358282	0.729752316	947.358282	0.137304822
948.582817	0.729620971	948.582817	0.137256934
949.14969	0.729655071	949.14969	0.137236638
950	0.729710494	950	0.13720623
950.374225	0.729732527	950.374225	0.137192496
952.074845	0.730196578	952.074845	0.137155844
952.358282	0.730086402	952.358282	0.137159944
953.582817	0.730257199	953.582817	0.137108345
954.14969	0.730191179	954.14969	0.137087718

Final State: j’=1		Final State: j’=0	
E(cm⁻¹)	σ (Å²)	E(cm⁻¹)	σ (Å²)
955	0.730204204	955	0.13706028
955.374225	0.730213055	955.374225	0.137048717
957.074845	0.730101063	957.074845	0.13700591
957.358282	0.730249038	957.358282	0.137017124
958.582817	0.730316006	958.582817	0.136920376
959.14969	0.730836131	959.14969	0.137029806
960	0.730710433	960	0.136937278
960.374225	0.730754046	960.374225	0.136926885
962.074845	0.731061286	962.074845	0.136871585
962.358282	0.73097769	962.358282	0.136863421
963.582817	0.731181521	963.582817	0.136924204
964.14969	0.731449367	964.14969	0.136818799
965	0.731213831	965	0.136789595
965.374225	0.731125034	965.374225	0.136775154
967.074845	0.731737869	967.074845	0.136781102
967.358282	0.731697316	967.358282	0.136759808
968.582817	0.731510463	968.582817	0.136726146
969.14969	0.731363396	969.14969	0.136707853
970	0.732467967	970	0.136761707
970.374225	0.732117723	970.374225	0.136702252
972.074845	0.73174583	972.074845	0.136652261
972.358282	0.731717421	972.358282	0.136644434
973.582817	0.731558472	973.582817	0.136611473
974.14969	0.732487888	974.14969	0.136662203
975	0.731769366	975	0.136631408
975.374225	0.73211244	975.374225	0.136629182
977.074845	0.731930106	977.074845	0.136648475
977.358282	0.73216315	977.358282	0.136570726
978.582817	0.732825924	978.582817	0.136585424
979.14969	0.732276341	979.14969	0.136555338
980	0.733056291	980	0.136566986
980.374225	0.73253845	980.374225	0.136531397
982.074845	0.732438192	982.074845	0.136475475
982.358282	0.732161036	982.358282	0.13641716
983.582817	0.731843941	983.582817	0.136535713
984.14969	0.733099052	984.14969	0.136519303
985	0.732381655	985	0.136488379
985.374225	0.732456426	985.374225	0.136485744
987.074845	0.732485793	987.074845	0.136453165
987.358282	0.732264805	987.358282	0.136407387
988.582817	0.732778562	988.582817	0.136474218
989.14969	0.732756619	989.14969	0.136463663
990	0.732758096	990	0.136449938
990.374225	0.732764369	990.374225	0.136442828
992.074845	0.732978405	992.074845	0.136451183

Final State: j’=1		Final State: j’=0	
E(cm⁻¹)	σ (Å²)	E(cm⁻¹)	σ (Å²)
992.358282	0.733000021	992.358282	0.136465846
993.582817	0.732944594	993.582817	0.136443562
994.14969	0.732954058	994.14969	0.136438647
995	0.732972627	995	0.136432508
995.374225	0.732981877	995.374225	0.136430009
997.074845	0.733049943	997.074845	0.136425581
997.358282	0.733057635	997.358282	0.13642518
998.582817	0.733081489	998.582817	0.136420469
999.14969	0.733090582	999.14969	0.136415446
1000	0.733164297	1000	0.136436797
1000.37423	0.733152517	1000.37423	0.136429751
1002.07485	0.733163108	1002.07485	0.136422675
1002.35828	0.733168173	1002.35828	0.136422591
1003.58282	0.733188696	1003.58282	0.136423021
1004.14969	0.733197354	1004.14969	0.136423535
1005	0.733209464	1005	0.136424628
1005.37423	0.733214349	1005.37423	0.136425207
1007.07485	0.733235521	1007.07485	0.136428716
1007.35828	0.733239832	1007.35828	0.136429782
1008.58282	0.733255498	1008.58282	0.136436791
1009.14969	0.73325839	1009.14969	0.136438773
1010	0.733262741	1010	0.136442062
1010.37423	0.733264437	1010.37423	0.136443664
1012.07485	0.733270547	1012.07485	0.136452099
1012.35828	0.733271196	1012.35828	0.136453668
1013.58282	0.733272716	1013.58282	0.136460946
1014.14969	0.733272725	1014.14969	0.136464599
1015	0.733272034	1015	0.136470436
1015.37423	0.733271323	1015.37423	0.136473139
1017.07485	0.733265841	1017.07485	0.13648648
1017.35828	0.733264475	1017.35828	0.13648884
1018.58282	0.73325706	1018.58282	0.136499432
1019.14969	0.733252846	1019.14969	0.13650456
1020	0.733245698	1020	0.136512541
1020.37423	0.733242113	1020.37423	0.136516148
1022.07485	0.733223633	1022.07485	0.136533437
1022.35828	0.733220157	1022.35828	0.136536452
1023.58282	0.733203837	1023.58282	0.136549936
1024.14969	0.733195575	1024.14969	0.136556425
1025	0.733182424	1025	0.136566461
1025.37423	0.733176213	1025.37423	0.136570974
1027.07485	0.733145875	1027.07485	0.136592362
1027.35828	0.733140507	1027.35828	0.136596061
1028.58282	0.733115648	1028.58282	0.136612445
1029.14969	0.733103486	1029.14969	0.136620267

Final State: j'=1		Final State: j'=0	
E(cm⁻¹)	σ (Å²)	E(cm⁻¹)	σ (Å²)
1030	0.733084496	1030	0.13663228
1030.37423	0.73307572	1030.37423	0.136637658
1032.07485	0.733033831	1032.07485	0.136662938
1032.35828	0.73302655	1032.35828	0.136667277
1033.58282	0.732993523	1033.58282	0.136686433
1034.14969	0.732977586	1034.14969	0.136695526
1035	0.732952976	1035	0.136709377
1035.37423	0.732941742	1035.37423	0.136715645
1037.07485	0.732888795	1037.07485	0.13674468
1037.35828	0.732879693	1037.35828	0.136749646
1038.58282	0.732838797	1038.58282	0.136771466
1039.14969	0.732819258	1039.14969	0.136781786
1040	0.732789297	1040	0.136797532
1040.37423	0.732775705	1040.37423	0.136804544
1042.07485	0.732712159	1042.07485	0.136837189
1042.35828	0.73270131	1042.35828	0.136842758
1043.58282	0.732652888	1043.58282	0.136867135
1044.14969	0.732629906	1044.14969	0.136878636
1045	0.732594789	1045	0.136896137
1045.37423	0.732578948	1045.37423	0.136903918
1047.07485	0.732505263	1047.07485	0.136940028
1047.35828	0.732492743	1047.35828	0.136946168
1048.58282	0.732437134	1048.58282	0.136973005
1049.14969	0.732410855	1049.14969	0.136985633
1050	0.732370819	1050	0.137004819
1050.37423	0.732352838	1050.37423	0.137013336
1052.07485	0.732269479	1052.07485	0.137052764
1052.35828	0.73225535	1052.35828	0.137059451
1053.58282	0.732192894	1053.58282	0.137088641
1054.14969	0.732163467	1054.14969	0.137102351
1055	0.732118755	1055	0.137123147
1055.37423	0.732098722	1055.37423	0.13713237
1057.07485	0.732006148	1057.07485	0.137174972
1057.35828	0.731990497	1057.35828	0.137182185
1058.58282	0.731921536	1058.58282	0.13721362
1059.14969	0.731889121	1059.14969	0.13722836
1060	0.731839957	1060	0.137250695
1060.37423	0.731817973	1060.37423	0.137260588
1062.07485	0.731716643	1062.07485	0.137306223
1062.35828	0.731699567	1062.35828	0.137313929
1063.58282	0.731624416	1063.58282	0.137347512
1064.14969	0.731589158	1064.14969	0.137363233
1065	0.731535785	1065	0.137387031
1065.37423	0.731511953	1065.37423	0.137397568
1067.07485	0.731402351	1067.07485	0.137446078

Final State: j'=1		Final State: j'=0	
E(cm⁻¹)	σ (Å²)	E(cm⁻¹)	σ (Å²)
1067.35828	0.731383895	1067.35828	0.137454266
1068.58282	0.731302885	1068.58282	0.137489887
1069.14969	0.731264958	1069.14969	0.137506549
1070	0.731207589	1070	0.137531738
1070.37423	0.73118202	1070.37423	0.137542881
1072.07485	0.731064588	1072.07485	0.137594132
1072.35828	0.73104485	1072.35828	0.137602773
1073.58282	0.730958314	1073.58282	0.13764033
1074.14969	0.730917856	1074.14969	0.137657875
1075	0.730856731	1075	0.137684392
1075.37423	0.730829518	1075.37423	0.137696112
1077.07485	0.730704722	1077.07485	0.137749952
1077.35828	0.730683761	1077.35828	0.137759036
1078.58282	0.730592039	1078.58282	0.137798419
1079.14969	0.730549206	1079.14969	0.137816807
1080	0.730484544	1080	0.137844575
1080.37423	0.730455789	1080.37423	0.137856842
1082.07485	0.730324161	1082.07485	0.137913159
1082.35828	0.730301987	1082.35828	0.137922622
1083.58282	0.730205405	1083.58282	0.137963749
1084.14969	0.730160346	1084.14969	0.137982931
1085	0.730092379	1085	0.138011883
1085.37423	0.73006218	1085.37423	0.138024666
1087.07485	0.729924093	1087.07485	0.138083304
1087.35828	0.729900855	1087.35828	0.138093148
1088.58282	0.72979973	1088.58282	0.13813591
1089.14969	0.729752598	1089.14969	0.138155842
1090	0.729681552	1090	0.138185905
1090.37423	0.729650006	1090.37423	0.138199177
1092.07485	0.729505909	1092.07485	0.13826
1092.35828	0.729481687	1092.35828	0.138270205
1093.58282	0.729376346	1093.58282	0.138314506
1094.14969	0.729327284	1094.14969	0.138335143
1095	0.729253385	1095	0.138366255
1095.37423	0.729220593	1095.37423	0.138379983
1097.07485	0.729070925	1097.07485	0.138442858
1097.35828	0.729045787	1097.35828	0.138453401
1098.58282	0.728936542	1098.58282	0.138499146
1099.14969	0.728885707	1099.14969	0.138520446
1100	0.728809176	1100	0.138552544
1100.37423	0.728775226	1100.37423	0.138566698
1102.07485	0.728620421	1102.07485	0.138631494
1102.35828	0.728594441	1102.35828	0.138642353
1103.58282	0.728481619	1103.58282	0.138689452
1104.14969	0.728429139	1104.14969	0.138711367

Final State: j'=1		Final State: j'=0	
E(cm⁻¹)	σ (Å²)	E(cm⁻¹)	σ (Å²)
1105	0.728350183	1105	0.138744381
1105.37423	0.72831519	1105.37423	0.138758939
1107.07485	0.728155681	1107.07485	0.138825535
1107.35828	0.728128937	1107.35828	0.13883669
1108.58282	0.728012831	1108.58282	0.138885048
1109.14969	0.727958866	1109.14969	0.138907544
1110	0.727877696	1110	0.138941413
1110.37423	0.727841743	1110.37423	0.138956346
1112.07485	0.727677962	1112.07485	0.139024614
1112.35828	0.727650513	1112.35828	0.139036042
1113.58282	0.727531434	1113.58282	0.139085577
1114.14969	0.727476119	1114.14969	0.139108609
1115	0.727392954	1115	0.139143276
1115.37423	0.727356125	1115.37423	0.139158555
1117.07485	0.727188487	1117.07485	0.139228379
1117.35828	0.727160411	1117.35828	0.139240062
1118.58282	0.727038655	1118.58282	0.139290684
1119.14969	0.726982122	1119.14969	0.139314212
1120	0.726897166	1120	0.139349621
1120.37423	0.726859562	1120.37423	0.139365221
1122.07485	0.726688481	1122.07485	0.139436484
1122.35828	0.726659843	1122.35828	0.139448404
1123.58282	0.726535704	1123.58282	0.139500037
1124.14969	0.726478092	1124.14969	0.139524024
1125	0.726391545	1125	0.139560114
1125.37423	0.726353249	1125.37423	0.139576011
1127.07485	0.726179112	1127.07485	0.139648603
1127.35828	0.726149984	1127.35828	0.13966074
1128.58282	0.726023752	1128.58282	0.139713298
1129.14969	0.725965201	1129.14969	0.139737712
1130	0.725877267	1130	0.139774429
1130.37423	0.72583837	1130.37423	0.139790599
1132.07485	0.72566159	1132.07485	0.139864413
1132.35828	0.72563203	1132.35828	0.13987675
1133.58282	0.725503987	1133.58282	0.139930164
1134.14969	0.725444614	1134.14969	0.139954961
1135	0.725355486	1135	0.139992256
1135.37423	0.725316075	1135.37423	0.140008675
1137.07485	0.725137037	1137.07485	0.140083609
1137.35828	0.725107109	1137.35828	0.140096127
1138.58282	0.72497754	1138.58282	0.140150318
1139.14969	0.724917475	1139.14969	0.140175472
1140	0.724827343	1140	0.140213292
1140.37423	0.7247875	1140.37423	0.140229941
1142.07485	0.724606589	1142.07485	0.140305889

Final State: j'=1		Final State: j'=0	
E(cm⁻¹)	σ (Å²)	E(cm⁻¹)	σ (Å²)
1142.35828	0.72457636	1142.35828	0.140318574
1143.58282	0.724445529	1143.58282	0.14037347
1144.14969	0.724384915	1144.14969	0.140398946
1145	0.724293975	1145	0.140437243
1145.37423	0.724253787	1145.37423	0.140454095
1147.07485	0.724071374	1147.07485	0.140530961
1147.35828	0.724040915	1147.35828	0.140543798
1148.58282	0.723909106	1148.58282	0.140599331
1149.14969	0.723848061	1149.14969	0.140625093
1150	0.723756514	1150	0.140663814
1150.37423	0.723716058	1150.37423	0.140680853
1152.07485	0.723532561	1152.07485	0.140758533
1152.35828	0.72350193	1152.35828	0.140771504
1153.58282	0.723369441	1153.58282	0.140827597
1154.14969	0.723308106	1154.14969	0.140853614
1155	0.723216157	1155	0.140892707
1155.37423	0.723175544	1155.37423	0.140909904
1157.07485	0.7229914	1157.07485	0.140988287
1157.35828	0.722960683	1157.35828	0.141001368
1158.58282	0.722827878	1158.58282	0.141057932
1159.14969	0.722766429	1159.14969	0.141084156
1160	0.722674363	1160	0.141123544
1160.37423	0.722633721	1160.37423	0.141140873
1162.07485	0.722449623	1162.07485	0.141219771
1162.35828	0.722418946	1162.35828	0.141232935
1163.58282	0.722286474	1163.58282	0.14128979
1164.14969	0.722225379	1164.14969	0.141316137
1165	0.722133781	1165	0.14135562
1165.37423	0.722093465	1165.37423	0.141372968
1167.07485	0.72191194	1167.07485	0.141451656
1167.35828	0.721881982	1167.35828	0.141464691
1168.58282	0.721754958	1168.58282	0.141520219
1169.14969	0.7216996	1169.14969	0.141544763
1170	0.721604477	1170	0.141562972
1170.37423	0.721564672	1170.37423	0.141590382
1172.07485	0.721353961	1172.07485	0.141656647
1172.35828	0.721229117	1172.35828	0.141709264
1173.58282	0.721200231	1173.58282	0.14175766
1174.14969	0.721094732	1174.14969	0.141781143
1175	0.720997835	1175	0.141835425
1175.37423	0.721002094	1175.37423	0.141841211
1177.07485	0.720806298	1177.07485	0.141925882
1177.35828	0.720782314	1177.35828	0.141934558
1178.58282	0.720621769	1178.58282	0.142002181
1179.14969	0.720516311	1179.14969	0.142032751

Final State: j’=1		Final State: j’=0	
E(cm⁻¹)	σ (Å²)	E(cm⁻¹)	σ (Å²)
1180	0.720468165	1180	0.142072888
1180.37423	0.720434859	1180.37423	0.142088601
1182.07485	0.720200979	1182.07485	0.142174055
1182.35828	0.720215357	1182.35828	0.142182806
1183.58282	0.720058236	1183.58282	0.142250324
1184.14969	0.720016528	1184.14969	0.142272939
1185	0.719933956	1185	0.14231066
1185.37423	0.719896432	1185.37423	0.142327252
1187.07485	0.719677715	1187.07485	0.142404774
1187.35828	0.719672032	1187.35828	0.142409454
1188.58282	0.719532969	1188.58282	0.142488523
1189.14969	0.719480339	1189.14969	0.142513329
1190	0.719394357	1190	0.142552288
1190.37423	0.719355665	1190.37423	0.142569537
1192.07485	0.719066213	1192.07485	0.142651548
1192.35828	0.719130466	1192.35828	0.142662824
1193.58282	0.718974157	1193.58282	0.142736562
1194.14969	0.718945106	1194.14969	0.142756021
1195	0.718861173	1195	0.142794683
1195.37423	0.718822733	1195.37423	0.142812129
1197.07485	0.718645108	1197.07485	0.142891948
1197.35828	0.71861407	1197.35828	0.142905101
1198.58282	0.718469729	1198.58282	0.142953173
1199.14969	0.718388302	1199.14969	0.142985534
1200	0.718336138	1200	0.14303816
1200.37423	0.71830278	1200.37423	0.143054018
1202.07485	0.718139736	1202.07485	0.143131252
1202.35828	0.718113881	1202.35828	0.143143798
1203.58282	0.71796253	1203.58282	0.14317722
1204.14969	0.717799941	1204.14969	0.143205619
1205	0.717707265	1205	0.143261246
1205.37423	0.717685562	1205.37423	0.143258911
1207.07485	0.717607622	1207.07485	0.1433344
1207.35828	0.717446049	1207.35828	0.143357158
1208.58282	0.71742358	1208.58282	0.143453909
1209.14969	0.717404203	1209.14969	0.143466733
1210	0.717112603	1210	0.143543627
1210.37423	0.717175031	1210.37423	0.143554184
1212.07485	0.717067031	1212.07485	0.143613409
1212.35828	0.716876297	1212.35828	0.143581158
1213.58282	0.716777676	1213.58282	0.143712853
1214.14969	0.716829305	1214.14969	0.14373554
1215	0.716764087	1215	0.143769616
1215.37423	0.716738454	1215.37423	0.143782393
1217.07485	0.716514753	1217.07485	0.143882459

Final State: j'=1		Final State: j'=0	
E(cm⁻¹)	σ (Å²)	E(cm⁻¹)	σ (Å²)
1217.35828	0.716495224	1217.35828	0.143894083
1218.58282	0.716391622	1218.58282	0.143947637
1219.14969	0.71634189	1219.14969	0.143971359
1220	0.715992257	1220	0.144036982
1220.37423	0.716146181	1220.37423	0.144035519
1222.07485	0.716051087	1222.07485	0.144109682
1222.35828	0.716090478	1222.35828	0.144080912
1223.58282	0.715840424	1223.58282	0.144193751
1224.14969	0.715784465	1224.14969	0.144173144
1225	0.715689958	1225	0.14427628
1225.37423	0.71570002	1225.37423	0.144285669
1227.07485	0.71551907	1227.07485	0.144370541
1227.35828	0.715510242	1227.35828	0.144379375
1228.58282	0.715299228	1228.58282	0.144444528
1229.14969	0.715148307	1229.14969	0.144493509
1230	0.714715845	1230	0.144511825
1230.37423	0.715109798	1230.37423	0.144549018
1232.07485	0.715077955	1232.07485	0.144577356
1232.35828	0.71495447	1232.35828	0.144628274
1233.58282	0.714823113	1233.58282	0.144673299
1234.14969	0.714793009	1234.14969	0.144710578
1235	0.714711488	1235	0.144751444
1235.37423	0.714674403	1235.37423	0.144769628
1237.07485	0.714479737	1237.07485	0.144854194
1237.35828	0.714536248	1237.35828	0.144855754
1238.58282	0.714353822	1238.58282	0.144927316
1239.14969	0.714302907	1239.14969	0.144956591
1240	0.714217668	1240	0.144998887
1240.37423	0.714180252	1240.37423	0.145017482
1242.07485	0.714009967	1242.07485	0.14510209
1242.35828	0.71398112	1242.35828	0.14511615
1243.58282	0.713862948	1243.58282	0.145177216
1244.14969	0.713809104	1244.14969	0.145205652
1245	0.713726195	1245	0.145248272
1245.37423	0.713687695	1245.37423	0.145267063
1247.07485	0.713534417	1247.07485	0.145351978
1247.35828	0.713506772	1247.35828	0.145366133
1248.58282	0.713389312	1248.58282	0.145427409
1249.14969	0.713335654	1249.14969	0.14545585
1250	0.71325533	1250	0.145498503
1250.37423	0.713219962	1250.37423	0.145517259
1252.07485	0.71306027	1252.07485	0.145602695
1252.35828	0.713033679	1252.35828	0.145616934
1253.58282	0.712918698	1253.58282	0.14567831
1254.14969	0.712866838	1254.14969	0.145706658

Final State: j’=1		Final State: j’=0	
E(cm⁻¹)	σ (Å²)	E(cm⁻¹)	σ (Å²)
1255	0.71278931	1255	0.145749653
1255.37423	0.712754765	1255.37423	0.145768542
1257.07485	0.712598413	1257.07485	0.145854423
1257.35828	0.712572419	1257.35828	0.145868731
1258.58282	0.712460318	1258.58282	0.145930567
1259.14969	0.712408744	1259.14969	0.145959247
1260	0.712331427	1260	0.146002252
1260.37423	0.712297368	1260.37423	0.146021161
1262.07485	0.71214393	1262.07485	0.146107299
1262.35828	0.712118362	1262.35828	0.146121663
1263.58282	0.712008482	1263.58282	0.146183755
1264.14969	0.711957947	1264.14969	0.14621257
1265	0.711882169	1265	0.146255783
1265.37423	0.711848786	1265.37423	0.146274783
1267.07485	0.711698244	1267.07485	0.14636138
1267.35828	0.711673229	1267.35828	0.146375822
1268.58282	0.711565355	1268.58282	0.146438231
1269.14969	0.711515743	1269.14969	0.146467192
1270	0.711441374	1270	0.146510619
1270.37423	0.711408618	1270.37423	0.146529713
1272.07485	0.711260955	1272.07485	0.146616748
1272.35828	0.711236425	1272.35828	0.146631265
1273.58282	0.711130759	1273.58282	0.146694035
1274.14969	0.711082019	1274.14969	0.146723122
1275	0.711009127	1275	0.146766789
1275.37423	0.710977002	1275.37423	0.146785991
1277.07485	0.710832277	1277.07485	0.146873521
1277.35828	0.710808228	1277.35828	0.146888123
1278.58282	0.710704671	1278.58282	0.146951261
1279.14969	0.710656907	1279.14969	0.146980522
1280	0.710585468	1280	0.147024449
1280.37423	0.710553992	1280.37423	0.147043768
1282.07485	0.71041219	1282.07485	0.147131838
1282.35828	0.710388633	1282.35828	0.14714653
1283.58282	0.710287183	1283.58282	0.147210066
1285	0.710170423	1285	0.147283727
1285.37423	0.710139593	1285.37423	0.147303171
1287.07485	0.710000725	1287.07485	0.147391827
1287.35828	0.709977655	1287.35828	0.147406618
1288.58282	0.709878316	1288.58282	0.147470588
1290	0.709763996	1290	0.147544761
1290.37423	0.709733811	1290.37423	0.14756434
1292.07485	0.709597864	1292.07485	0.147653633
1292.35828	0.709575285	1292.35828	0.147668531
1293.58282	0.70947805	1293.58282	0.147732972

Final State: j’=1		Final State: j’=0	
E(cm⁻¹)	σ (Å²)	E(cm⁻¹)	σ (Å²)
1295	0.709366155	1295	0.147807699
1295.37423	0.709336608	1295.37423	0.147827429
1297.07485	0.709203571	1297.07485	0.147917405
1297.35828	0.709181477	1297.35828	0.14793242
1298.58282	0.709086335	1298.58282	0.147997365
1300	0.708976855	1300	0.148072693
1300.37423	0.708947928	1300.37423	0.14809258
1302.07485	0.708817791	1302.07485	0.148183297
1302.35828	0.708796181	1302.35828	0.148198436
1303.58282	0.708703107	1303.58282	0.148263928
1305	0.708596023	1305	0.148339898
1305.37423	0.70856774	1305.37423	0.148359957
1307.07485	0.708440448	1307.07485	0.148451464
1307.35828	0.708419311	1307.35828	0.148466739
1308.58282	0.708328288	1308.58282	0.148532815
1310	0.708223563	1310	0.148609473
1310.37423	0.708195898	1310.37423	0.148629714
1312.07485	0.708071438	1312.07485	0.148722071
1312.35828	0.708050762	1312.35828	0.148737488
1313.58282	0.707961763	1313.58282	0.148804188
1315	0.707859374	1315	0.148881579
1315.37423	0.707832322	1315.37423	0.148902015
1317.07485	0.70771064	1317.07485	0.148995277
1317.35828	0.707690423	1317.35828	0.149010845
1318.58282	0.707603413	1318.58282	0.149078209
1320	0.707503317	1320	0.149156383
1320.37423	0.707476868	1320.37423	0.149177028
1322.07485	0.707357917	1322.07485	0.149271248
1322.35828	0.707338159	1322.35828	0.149286979
1323.58282	0.707253103	1323.58282	0.149355047
1325	0.707155254	1325	0.149434048
1325.37423	0.707129398	1325.37423	0.149454909
1327.07485	0.707013124	1327.07485	0.149550148
1327.35828	0.706993811	1327.35828	0.149566049
1328.58282	0.706910675	1328.58282	0.149634863
1330	0.706815034	1330	0.149714736
1330.37423	0.706789751	1330.37423	0.149735831
1332.07485	0.706676106	1332.07485	0.149832142
1332.35828	0.70665723	1332.35828	0.149848224
1333.58282	0.706575963	1333.58282	0.149917821
1335	0.706482469	1335	0.149998616
1335.37423	0.706457758	1335.37423	0.150019957
1337.07485	0.706346679	1337.07485	0.150117392
1337.35828	0.706328225	1337.35828	0.150133663
1338.58282	0.706248781	1338.58282	0.150204086

Final State: j’=1		Final State: j’=0	
E(cm⁻¹)	σ (Å²)	E(cm⁻¹)	σ (Å²)
1340	0.706157392	1340	0.150285846
1340.37423	0.706133228	1340.37423	0.150307441
1342.07485	0.706024646	1342.07485	0.150406058
1342.35828	0.706006612	1342.35828	0.150422528
1343.58282	0.705928947	1343.58282	0.150493813
1345	0.705839603	1345	0.150576586
1345.37423	0.705815973	1345.37423	0.150598448
1347.07485	0.70570982	1347.07485	0.1506983
1347.35828	0.705692185	1347.35828	0.150714977
1348.58282	0.705616251	1348.58282	0.150787161
1350	0.705528895	1350	0.150870989
1350.37423	0.705505791	1350.37423	0.150893131
1352.07485	0.705401993	1352.07485	0.150994268
1352.35828	0.705384751	1352.35828	0.151011162
1353.58282	0.7053105	1353.58282	0.151084286
1355	0.705225064	1355	0.151169207
1355.37423	0.705202463	1355.37423	0.151191638
1357.07485	0.705100949	1357.07485	0.151294113
1357.35828	0.705084078	1357.35828	0.15131123
1358.58282	0.705011451	1358.58282	0.151385327
1360	0.704927876	1360	0.151471386
1360.37423	0.704905767	1360.37423	0.151494118
1362.07485	0.704806462	1362.07485	0.151597976
1362.35828	0.70478996	1362.35828	0.151615327
1363.58282	0.704718903	1363.58282	0.151690431
1364.14969	0.704686135	1364.14969	0.151725285
1365	0.704637128	1365	0.151777666
1365.37423	0.704615492	1365.37423	0.151800708
1367.07485	0.704518308	1367.07485	0.151905997
1367.35828	0.704502159	1367.35828	0.151923586
1368.58282	0.704432612	1368.58282	0.151999729
1369.14969	0.704400544	1369.14969	0.152035066
1370	0.704352579	1370	0.152088177
1370.37423	0.704331389	1370.37423	0.15211154
1372.07485	0.704236269	1372.07485	0.152218302
1372.35828	0.704220453	1372.35828	0.152236137
1373.58282	0.704152365	1373.58282	0.152313353
1374.14969	0.704120957	1374.14969	0.152349186
1375	0.704073994	1375	0.152403047
1375.37423	0.704053241	1375.37423	0.152426737
1377.07485	0.703960089	1377.07485	0.152535016
1377.35828	0.703944605	1377.35828	0.152553104
1378.58282	0.703877909	1378.58282	0.152631418
1379.14969	0.70384715	1379.14969	0.152667764
1380	0.703801145	1380	0.152722393

Final State: j’=1		Final State: j’=0	
E(cm⁻¹)	σ (Å²)	E(cm⁻¹)	σ (Å²)
1380.37423	0.703780811	1380.37423	0.152746422
1382.07485	0.703689543	1382.07485	0.152856251
1382.35828	0.703674374	1382.35828	0.152874599
1383.58282	0.703609014	1383.58282	0.152954039
1384.14969	0.70357888	1384.14969	0.152990909
1385	0.703533787	1385	0.153046324
1385.37423	0.70351385	1385.37423	0.153070698
1387.07485	0.703424395	1387.07485	0.153182115
1387.35828	0.703409521	1387.35828	0.153200729
1388.58282	0.70334545	1388.58282	0.153281316
1389.14969	0.703315898	1389.14969	0.15331872
1390	0.703271695	1390	0.153374941
1390.37423	0.703252139	1390.37423	0.153399668
1392.07485	0.703164421	1392.07485	0.153512703
1392.35828	0.703149821	1392.35828	0.153531587
1393.58282	0.703086977	1393.58282	0.153613348
1394.14969	0.703057985	1394.14969	0.153651298
1395	0.703014609	1395	0.153708334
1395.37423	0.702995415	1395.37423	0.153733424
1397.07485	0.702909342	1397.07485	0.153848104
1397.35828	0.702895026	1397.35828	0.153867263
1398.58282	0.702833343	1398.58282	0.153950216
1399.14969	0.702804881	1399.14969	0.153988715
1400	0.702762299	1400	0.154046586
1400.37423	0.702743456	1400.37423	0.154072035
1402.07485	0.702658952	1402.07485	0.154188394
1402.35828	0.702644888	1402.35828	0.154207831
1403.58282	0.702584318	1403.58282	0.154291991
1404.14969	0.702556364	1404.14969	0.154331052
1405	0.702514532	1405	0.154389765
1405.37423	0.702496024	1405.37423	0.154415584
1407.07485	0.702412992	1407.07485	0.154533635
1407.35828	0.70239918	1407.35828	0.154553357
1408.58282	0.702339649	1408.58282	0.15463874
1409.14969	0.702312178	1409.14969	0.154678369
1410	0.702271066	1410	0.154737934
1410.37423	0.702253014	1410.37423	0.154764194
1412.07485	0.702171234	1412.07485	0.15488389
1412.35828	0.702157656	1412.35828	0.154903895
1413.58282	0.702099115	1413.58282	0.154990515
1414.14969	0.702072104	1414.14969	0.155030717
1415	0.702031659	1415	0.155091142
1415.37423	0.702013898	1415.37423	0.155117782
1417.07485	0.701933448	1417.07485	0.155239202
1417.35828	0.701920085	1417.35828	0.155259496

Final State: j'=1		Final State: j'=0	
E(cm⁻¹)	σ (Å²)	E(cm⁻¹)	σ (Å²)
1418.58282	0.701862475	1418.58282	0.155347361
1419.14969	0.701835876	1419.14969	0.15538814
1420	0.701796072	1420	0.155449432
1420.37423	0.701778585	1420.37423	0.155476454
1422.07485	0.701699365	1422.07485	0.15559961
1422.35828	0.701686207	1422.35828	0.155620195
1423.58282	0.701629467	1423.58282	0.155709313
1424.14969	0.701603265	1424.14969	0.155750672
1425	0.701564047	1425	0.155812837
1425.37423	0.701546821	1425.37423	0.155840243
1427.07485	0.70146875	1427.07485	0.155965146
1427.35828	0.701455774	1427.35828	0.155986024
1428.58282	0.701399829	1428.58282	0.156076402
1429.14969	0.701373991	1429.14969	0.156118346
1430	0.701335315	1430	0.156181388
1430.37423	0.701318317	1430.37423	0.156209179
1432.07485	0.701241281	1432.07485	0.156335846
1432.35828	0.701228474	1432.35828	0.156357014
1433.58282	0.701173233	1433.58282	0.156448667
1434.14969	0.701147722	1434.14969	0.156491203
1435	0.701109506	1435	0.156555135
1435.37423	0.70109271	1435.37423	0.156583322
1437.07485	0.7010165	1437.07485	0.156711787
1437.35828	0.701003824	1437.35828	0.15673326
1438.58282	0.700949076	1438.58282	0.156826239
1439.14969	0.700923743	1439.14969	0.1568694
1440	0.70088572	1440	0.156934295
1440.37423	0.700868966	1440.37423	0.156962917
1442.07485	0.700792178	1442.07485	0.15709363
1442.35828	0.700779108	1442.35828	0.157115582
1443.58282	0.700718487	1443.58282	0.157220824
1444.14969	0.700698934	1444.14969	0.157270527
1445	0.700667736	1445	0.157318537
1445.37423	0.70064553	1445.37423	0.157349982
1447.07485	0.700576421	1447.07485	0.15747909
1447.35828	0.700560748	1447.35828	0.157502394
1448.58282	0.700521115	1448.58282	0.157593333
1449.14969	0.70049768	1449.14969	0.157650874
1450	0.700457722	1450	0.157705074
1450.37423	0.700437885	1450.37423	0.157735514
1452.07485	0.700368628	1452.07485	0.157868416
1452.35828	0.700351028	1452.35828	0.157892911
1453.58282	0.700303037	1453.58282	0.157986468
1454.14969	0.700276475	1454.14969	0.158032193
1455	0.700237239	1455	0.158102343

Final State: j’=1		Final State: j’=0	
E(cm⁻¹)	σ (Å²)	E(cm⁻¹)	σ (Å²)
1455.37423	0.700248132	1455.37423	0.158136948
1457.07485	0.700160969	1457.07485	0.158261088
1457.35828	0.700144289	1457.35828	0.158284764
1458.58282	0.70008849	1458.58282	0.158384137
1459.14969	0.700063603	1459.14969	0.158430179
1460	0.700040744	1460	0.158508671
1460.37423	0.700015649	1460.37423	0.158525197
1462.07485	0.69994421	1462.07485	0.158662426
1462.35828	0.69993097	1462.35828	0.158685847
1463.58282	0.699877552	1463.58282	0.158785877
1464.14969	0.699853408	1464.14969	0.158832161
1465	0.69981818	1465	0.158901962
1465.37423	0.699805239	1465.37423	0.158933943
1467.07485	0.6997461	1467.07485	0.159063914
1467.35828	0.699721763	1467.35828	0.159090551
1468.58282	0.699667486	1468.58282	0.159192449
1469.14969	0.69964352	1469.14969	0.15923919
1470	0.699607901	1470	0.159309341
1470.37423	0.6995924	1470.37423	0.159340263
1472.07485	0.699513883	1472.07485	0.15948094
1472.35828	0.69951146	1472.35828	0.159509689
1473.58282	0.69945769	1473.58282	0.159603467
1474.14969	0.699432695	1474.14969	0.159651397
1475	0.699396126	1475	0.159722661
1475.37423	0.699379927	1475.37423	0.159754041
1477.07485	0.699293472	1477.07485	0.159907815
1477.35828	0.699293272	1477.35828	0.159924513
1478.58282	0.699274276	1478.58282	0.160015058
1479.14969	0.699248424	1479.14969	0.160075706
1480	0.699194768	1480	0.160138768
1480.37423	0.699167508	1480.37423	0.160175816
1482.07485	0.699112574	1482.07485	0.160314779
1482.35828	0.699097023	1482.35828	0.160339801
1483.58282	0.699085559	1483.58282	0.160438437
1484.14969	0.69903797	1484.14969	0.16048791
1485	0.698995838	1485	0.160562008
1485.37423	0.698978324	1485.37423	0.160594947
1487.07485	0.698918278	1487.07485	0.1607473
1487.35828	0.698903874	1487.35828	0.160758206
1488.58282	0.698846155	1488.58282	0.160867031
1489.14969	0.69881767	1489.14969	0.160918446
1490	0.698815843	1490	0.160980279
1490.37423	0.698785377	1490.37423	0.161015748
1492.07485	0.698707324	1492.07485	0.161165334
1492.35828	0.69869516	1492.35828	0.161190183

Final State: j’=1		Final State: j’=0	
E(cm⁻¹)	σ (Å²)	E(cm⁻¹)	σ (Å²)
1493.58282	0.698686906	1493.58282	0.161279285
1494.14969	0.698617098	1494.14969	0.161344349
1495	0.698597913	1495	0.161421783
1495.37423	0.698559276	1495.37423	0.161449912
1497.07485	0.698522352	1497.07485	0.161601275
1497.35828	0.698495699	1497.35828	0.16162488
1498.58282	0.698454623	1498.58282	0.161726415
1499.14969	0.698416388	1499.14969	0.161779022
1500	0.698407417	1500	0.161849527
1500.37423	0.698381126	1500.37423	0.161883603
1502.07485	0.698321749	1502.07485	0.162031099
1502.35828	0.698288433	1502.35828	0.162063641
1503.58282	0.698369121	1503.58282	0.162171759
1504.14969	0.698224495	1504.14969	0.162213585
1505.37423	0.698170282	1505.37423	0.162328579
1507.07485	0.698117264	1507.07485	0.162477907
1507.35828	0.698079439	1507.35828	0.162515124
1508.58282	0.698051482	1508.58282	0.16261078
1509.14969	0.698029624	1509.14969	0.162661234
1510.37423	0.697984359	1510.37423	0.162769764
1512.07485	0.69791129	1512.07485	0.162920874
1512.35828	0.697900905	1512.35828	0.162946385
1513.58282	0.697854448	1513.58282	0.163055797
1514.14969	0.697832489	1514.14969	0.163106445
1515.37423	0.697785	1515.37423	0.163215958
1517.07485	0.697717848	1517.07485	0.163368313
1517.35828	0.697706617	1517.35828	0.163393774
1518.58282	0.697658719	1518.58282	0.163503827
1519.14969	0.697637168	1519.14969	0.16355461
1520.37423	0.697586681	1520.37423	0.163665334
1522.07485	0.69752038	1522.07485	0.163818948
1522.35828	0.697509243	1522.35828	0.163844582
1523.58282	0.697461114	1523.58282	0.163955443
1524.14969	0.697438832	1524.14969	0.164006826
1525.37423	0.697390715	1525.37423	0.164117955
1527.07485	0.697323767	1527.07485	0.164272571
1527.35828	0.697312475	1527.35828	0.164298383
1528.58282	0.697264172	1528.58282	0.164410015
1529.14969	0.697241967	1529.14969	0.164461734
1530.37423	0.697193932	1530.37423	0.164573582
1532.07485	0.697127162	1532.07485	0.16472919
1533.58282	0.697067942	1533.58282	0.164867433
1534.14969	0.697045678	1534.14969	0.164919465
1535.37423	0.69699757	1535.37423	0.165031978
1537.07485	0.696930745	1537.07485	0.165188491

Final State: j’=1		Final State: j’=0	
E(cm⁻¹)	σ (Å²)	E(cm⁻¹)	σ (Å²)
1538.58282	0.696871532	1538.58282	0.165327517
1539.14969	0.696849274	1539.14969	0.165379835
1540.37423	0.696801195	1540.37423	0.165492959
1542.07485	0.696734419	1542.07485	0.165650301
1543.58282	0.696675202	1543.58282	0.165790042
1544.14969	0.696652934	1544.14969	0.165842625
1545.37423	0.696604836	1545.37423	0.16595631
1547.07485	0.696538026	1547.07485	0.166114409
1548.58282	0.696478775	1548.58282	0.166254801
1549.14969	0.696456486	1549.14969	0.166307623
1550.37423	0.696408366	1550.37423	0.166421818
1552.07485	0.696341506	1552.07485	0.1665806
1553.58282	0.696282199	1553.58282	0.166721574
1554.14969	0.696259901	1554.14969	0.166774615
1555.37423	0.696211722	1555.37423	0.166889261
1557.07485	0.696144784	1557.07485	0.167048653
1558.58282	0.6960854	1558.58282	0.167190151
1559.14969	0.696063071	1559.14969	0.167243377
1560.37423	0.696014825	1560.37423	0.167358427
1562.07485	0.695947786	1562.07485	0.167518354
1563.58282	0.695888303	1563.58282	0.167660301
1564.14969	0.695865938	1564.14969	0.167713694
1565.37423	0.695817603	1565.37423	0.16782909
1567.07485	0.695750445	1567.07485	0.167989477
1568.58282	0.695690832	1568.58282	0.16813181
1569.14969	0.695668435	1569.14969	0.168185342
1570.37423	0.695619998	1570.37423	0.168301027
1572.07485	0.695552684	1572.07485	0.168461797
1573.58282	0.695492951	1573.58282	0.168604448
1574.14969	0.69547049	1574.14969	0.168658097
1575.37423	0.695421935	1575.37423	0.16877402
1577.07485	0.695354461	1577.07485	0.168935095
1578.58282	0.695294575	1578.58282	0.169077998
1579.14969	0.695271758	1579.14969	0.169131667
1580.37423	0.695223375	1580.37423	0.16924784
1582.07485	0.695155714	1582.07485	0.169409147
1583.58282	0.695095663	1583.58282	0.169552232
1584.14969	0.695073079	1584.14969	0.169606032
1585.37423	0.695024247	1585.37423	0.169722266
1588.58282	0.694896155	1588.58282	0.170026932
1589.14969	0.694873491	1589.14969	0.17008077
1590.37423	0.694824512	1590.37423	0.170197076
1593.58282	0.694695992	1593.58282	0.170501872
1594.14969	0.694673263	1594.14969	0.170555725
1595.37423	0.694624117	1595.37423	0.170672053

Final State: j’=1		Final State: j’=0	
E(cm⁻¹)	σ (Å²)	E(cm⁻¹)	σ (Å²)
1598.58282	0.69449516	1598.58282	0.170976839
1599.14969	0.694472338	1599.14969	0.171030679
1600.37423	0.694423025	1600.37423	0.171146975
1603.58282	0.694293593	1603.58282	0.171451615
1604.14969	0.69427069	1604.14969	0.171505422
1605.37423	0.694221192	1605.37423	0.171621631
1608.58282	0.694091262	1608.58282	0.171925988
1609.14969	0.69406827	1609.14969	0.171979734
1610.37423	0.694018578	1610.37423	0.172095807
1613.58282	0.693888132	1613.58282	0.172399746
1614.14969	0.693865049	1614.14969	0.17245341
1615.37423	0.693815155	1615.37423	0.172569298
1618.58282	0.69368418	1618.58282	0.172872689
1619.14969	0.693661001	1619.14969	0.172926249
1620.37423	0.693610894	1620.37423	0.173041898
1623.58282	0.693479366	1623.58282	0.173344613
1624.14969	0.693456089	1624.14969	0.173398045
1625.37423	0.693405774	1625.37423	0.17351341
1628.58282	0.693273677	1628.58282	0.173815326
1629.14969	0.6932503	1629.14969	0.173868607
1630.37423	0.693199765	1630.37423	0.173983641
1633.58282	0.693067095	1633.58282	0.174284636
1634.14969	0.693043614	1634.14969	0.174337746
1635.37423	0.692992857	1635.37423	0.174452403
1638.58282	0.692859598	1638.58282	0.174752357
1639.14969	0.692836018	1639.14969	0.174805276
1640.37423	0.692785037	1640.37423	0.174919513
1643.58282	0.692651185	1643.58282	0.175218316
1644.14969	0.692627501	1644.14969	0.175271025
1645.37423	0.692576291	1645.37423	0.175384799
1648.58282	0.692441845	1648.58282	0.175682339
1649.14969	0.692418053	1649.14969	0.175734818
1650.37423	0.692366613	1650.37423	0.175848086
1653.58282	0.692231568	1653.58282	0.17614426
1654.14969	0.692207668	1654.14969	0.176196492
1655.37423	0.692156004	1655.37423	0.176309219
1658.58282	0.692020363	1658.58282	0.176603927
1659.14969	0.691996349	1659.14969	0.176655894
1660.37423	0.691944459	1660.37423	0.17676804
1663.58282	0.691808224	1663.58282	0.177061185
1664.14969	0.691784119	1664.14969	0.177112869
1665.37423	0.691732003	1665.37423	0.177224399
1668.58282	0.691595143	1668.58282	0.177515905
1670.37423	0.691518629	1670.37423	0.177678161
1673.58282	0.691381237	1673.58282	0.177967918

Final State: j'=1		Final State: j'=0	
$E(cm^{-1})$	σ (Å²)	$E(cm^{-1})$	σ (Å²)
1675.37423	0.691304367	1675.37423	0.178129192
1678.58282	0.691166413	1678.58282	0.178417131
1680.37423	0.691089238	1680.37423	0.178577368
1683.58282	0.690950739	1683.58282	0.178863411
1685.37423	0.690873264	1685.37423	0.179022572
1688.58282	0.690734243	1688.58282	0.179306653
1690.37423	0.690656481	1690.37423	0.179464696
1693.58282	0.690516958	1693.58282	0.179746745
1695.37423	0.690438927	1695.37423	0.179903638
1698.58282	0.690298935	1698.58282	0.180183597
1700.37423	0.690220646	1700.37423	0.18033931
1703.58282	0.690080211	1703.58282	0.180617124
1705.37423	0.690001689	1705.37423	0.180771621
1708.58282	0.689860851	1708.58282	0.181047244
1710.37423	0.68978211	1710.37423	0.181200505
1713.58282	0.689640902	1713.58282	0.181473887
1715.37423	0.689561965	1715.37423	0.181625887
1718.58282	0.689420435	1718.58282	0.181896989
1720.37423	0.689341332	1720.37423	0.182047706
1723.58282	0.689199518	1723.58282	0.182316496
1725.37423	0.689120278	1725.37423	0.182465915
1728.58282	0.68897824	1728.58282	0.182732362
1730.37423	0.688898892	1730.37423	0.182880463
1733.58282	0.688756695	1733.58282	0.183144542
1735.37423	0.688677278	1735.37423	0.183291315
1738.58282	0.688535009	1738.58282	0.183553003
1740.37423	0.688455596	1740.37423	0.183698437
1743.58282	0.688313689	1743.58282	0.183957709
1745.37423	0.6882322	1745.37423	0.184100707
1748.58282	0.68809291	1748.58282	0.184351897
1750.37423	0.688011593	1750.37423	0.184500664
1753.58282	0.687865971	1753.58282	0.184755914
1755.37423	0.687789686	1755.37423	0.184896988
1758.58282	0.687646459	1758.58282	0.185149545
1760.37423	0.687568048	1760.37423	0.185289048
1763.58282	0.687425093	1763.58282	0.185539425
1765.37423	0.687346336	1765.37423	0.1856778
1768.58282	0.687204118	1768.58282	0.185925661
1770.37423	0.687125504	1770.37423	0.186062745
1773.58282	0.686984127	1773.58282	0.186308505
1775.37423	0.686905806	1775.37423	0.186443929
1780.37423	0.686688614	1780.37423	0.186815342
1785.37423	0.686466435	1785.37423	0.187193801
1790.37423	0.686250384	1790.37423	0.187557881
1795.37423	0.686036217	1795.37423	0.187932729

Final State: j'=1		Final State: j'=0	
E(cm⁻¹)	σ (Å²)	E(cm⁻¹)	σ (Å²)
1800.37423	0.685821978	1800.37423	0.188292984
1805.37423	0.685601847	1805.37423	0.18865146
1810.37423	0.68539074	1810.37423	0.189006691
1815.37423	0.685180008	1815.37423	0.189358943
1820.37423	0.684973833	1820.37423	0.189707977
1825.37423	0.684764462	1825.37423	0.190053495
1830.37423	0.684559973	1830.37423	0.190395869
1835.37423	0.684357759	1835.37423	0.190735105
1840.37423	0.68415806	1840.37423	0.191071246
1845.37423	0.683961012	1845.37423	0.19140438
1850.37423	0.683766762	1850.37423	0.191734571
1855.37423	0.683575463	1855.37423	0.192061904
1860.37423	0.683387274	1860.37423	0.19238645
1865.37423	0.683202325	1865.37423	0.192708289
1870.37423	0.683020767	1870.37423	0.193027502
1875.37423	0.68284273	1875.37423	0.193344164
1880.37423	0.682668356	1880.37423	0.193658359
1885.37423	0.682497772	1885.37423	0.193970161
1890.37423	0.682331096	1890.37423	0.194279657
1895.37423	0.682168445	1895.37423	0.194586921
1900.37423	0.682009938	1900.37423	0.194892029
1905.37423	0.681855669	1905.37423	0.195195063
1910.37423	0.681705731	1910.37423	0.195496097

Initial rotational state: j=3

Final State: j'=5		Final State: j'=4		Final State: j'=2	
E(cm⁻¹)	σ (Å²)	E(cm⁻¹)	σ (Å²)	E(cm⁻¹)	σ (Å²)
410.374226	3.12197E-05	273.582818	0.000252813	164.149691	20258.8445
410.374227	4.41888E-05	273.582819	0.000357871	164.149692	14343.439
410.374228	5.41692E-05	273.58282	0.000438745	164.149693	11727.055
410.374229	6.26079E-05	273.582821	0.00050715	164.149694	10169.8394
410.37423	7.00646E-05	273.582822	0.000567615	164.149695	9108.80465
410.374231	7.68262E-05	273.582823	0.000622461	164.149696	8326.80101
410.374232	8.30627E-05	273.582824	0.000673066	164.149697	7719.97326
410.374233	8.8885E-05	273.582825	0.000720325	164.149698	7231.58375
410.374234	9.43698E-05	273.582826	0.00076486	164.149699	6827.67243
410.374235	9.9573E-05	273.582827	0.000807123	164.1497	6486.50678
410.374236	0.000104537	273.582828	0.000847454	164.149701	6193.44916
410.374237	0.000109294	273.582829	0.000886119	164.149702	5938.23161
410.374238	0.00011387	273.58283	0.000923327	164.149703	5713.41188
410.374239	0.000118287	273.582831	0.000959248	164.149704	5513.4455
410.37424	0.000122561	273.582832	0.000994023	164.149705	5334.10647
410.374241	0.000126707	273.582833	0.001027768	164.149706	5172.10974
410.374242	0.000130738	273.582834	0.001060582	164.149707	5024.85758
410.374243	0.000134663	273.582835	0.001092549	164.149708	4890.26613
410.374244	0.000138492	273.582836	0.001123742	164.149709	4766.63945
410.374245	0.000142232	273.582837	0.001154224	164.14971	4652.58699
410.374246	0.000145891	273.582838	0.001184051	164.149711	4546.94908
410.374247	0.000149474	273.582839	0.001213271	164.149712	4448.75477
410.374248	0.000152987	273.58284	0.001241927	164.149713	4357.18242
410.374249	0.000156435	273.582841	0.001270058	164.149714	4271.5319
410.37425	0.000159821	273.582842	0.001297698	164.149715	4191.202
410.374251	0.00016315	273.582843	0.001324878	164.149716	4115.67548
410.374252	0.000166425	273.582844	0.001351627	164.149717	4044.49791
410.374253	0.000169649	273.582845	0.001377969	164.149718	3977.27681
410.374254	0.000172826	273.582846	0.001403929	164.149719	3913.66646
410.374255	0.000175957	273.582847	0.001429526	164.14972	3853.36173
410.374256	0.000179045	273.582848	0.001454781	164.149721	3796.09232
410.374257	0.000182093	273.582849	0.001479711	164.149722	3741.61799
410.374258	0.000185102	273.58285	0.001504333	164.149723	3689.72373
410.374259	0.000188074	273.582851	0.001528661	164.149724	3640.2175
410.37426	0.000191011	273.582852	0.00155271	164.149725	3592.92646
410.374261	0.000193915	273.582853	0.001576493	164.149726	3547.69448
410.374262	0.000196787	273.582854	0.001600022	164.149727	3504.38065
410.374263	0.000199629	273.582855	0.001623308	164.149728	3462.857
410.374264	0.000202441	273.582856	0.001646362	164.149729	3423.00725
410.374265	0.000205225	273.582857	0.001669194	164.14973	3384.72534
410.374266	0.000207983	273.582858	0.001691812	164.149731	3347.91422
410.374267	0.000210715	273.582859	0.001714226	164.149732	3312.48542
410.374268	0.000213422	273.58286	0.001736444	164.149733	3278.35722
410.374269	0.000216105	273.582861	0.001758473	164.149734	3245.45489

Final State: j'=5		Final State: j'=4		Final State: j'=2	
E(cm⁻¹)	σ (Å²)	E(cm⁻¹)	σ (Å²)	E(cm⁻¹)	σ (Å²)
410.37427	0.000218766	273.582862	0.00178032	164.149735	3213.70926
410.374271	0.000221404	273.582863	0.001801993	164.149736	3183.05654
410.374272	0.000224021	273.582864	0.001823498	164.149737	3153.43779
410.374273	0.000226618	273.582865	0.00184484	164.149738	3124.79851
410.374274	0.000229195	273.582866	0.001866026	164.149739	3097.08804
410.374275	0.000231753	273.582867	0.001887062	164.14974	3070.25911
410.374276	0.000234292	273.582868	0.001907951	164.149741	3044.26801
410.374277	0.000236814	273.582869	0.0019287	164.149742	3019.07389
410.374278	0.000239318	273.58287	0.001949313	164.149743	2994.63849
[truncated: 442,308 more chars]
