# Supplementary material for: Correction to “The CH– 3Σ– Anion: Inelastic Rate Coefficients from Collisions with He at Interstellar Conditions”
Source: J Phys Chem A. 2023 Jun 6;127(23):5075–81. doi: 10.1021/acs.jpca.3c03005 (PMC10278135; doi:10.1021/acs.jpca.3c03005)
Supplement: Supplementary file 1 — jp3c03005_si_001.zip [file jp3c03005_si_001.zip › erratum-SI.pdf]

CORRECTIONS TO: The CH^- ($^3\Sigma^-$) anion: Inelastic Rate Coefficients from collisions with He at Interstellar Conditions. J. Phys. Chem. A 2023, 127, 765774; https://doi.org/10.1021/acs.jpca.2c08021

Jorge Alonso de la Fuente,[†] Cristina Sanz-Sanz,[†] Lola Gonzalez-Sanchez ,[‡] E. Yurtsever,[¶] R. Wester,[§] and F. A. Gianturco[*,§]

†*Departamento de Quimica Fisica Aplicada, Modulo 14, Universidad Autonoma de Madrid, 28049 Madrid, Spain*

‡*Facultad de Ciencias Quimicas, Universidad de Salamanca, Plaza de los Caidos s/n, 37008 Salamanca, Spain.*

¶*Dept. of Chemistry, Koc University, Rumelifeneri Yolu, Sariyer,TR, 34450 Istanbul,Turkey*

§*Institut fur Ionen Physik und Angewandte Physik, Leopold-Franzens-Universitat, Technikerstrasse 25, 6020, Innsbruck, Austria*

E-mail: francesco.gianturco@uibk.ac.at

PECs MRCI – aV5Z			
**R (Bohr)**	**X $^3\Sigma^-$**	**^{3}A',^{3}A''**	**$^1\Delta$**
**1.500**	-38.35035254	-38.29264738	-38.27632464
**1.608**	-38.39559265	-38.33803553	-38.32036970
**1.715**	-38.42588379	-38.36820148	-38.34879960
**1.823**	-38.44532545	-38.38727534	-38.40360243
**1.930**	-38.45686887	-38.39826644	-38.42076836
**2.038**	-38.46270811	-38.40338829	-38.42891567
**2.146**	-38.46447514	-38.40428111	-38.43175679
**2.253**	-38.46338210	-38.40216662	-38.43113800
**2.361**	-38.46032782	-38.39795592	-38.42821988
**2.468**	-38.45597628	-38.39232292	-38.42379515
**2.576**	-38.45081468	-38.38576040	-38.41842258
**2.684**	-38.44519705	-38.37862328	-38.41249977
**2.791**	-38.43937706	-38.37117869	-38.40630885
**2.899**	-38.43353294	-38.36361446	-38.40005012
**3.006**	-38.42778646	-38.35603894	-38.39385527
**3.114**	-38.42221769	-38.34870970	-38.38781741
**3.222**	-38.41687628	-38.34221779	-38.38199473
**3.329**	-38.41179014	-38.33701352	-38.37642193
**3.437**	-38.40697196	-38.33316657	-38.37111716
**3.544**	-38.40242409	-38.33045805	-38.36608723
**3.652**	-38.39814203	-38.32861228	-38.36133122
**3.759**	-38.39411696	-38.32737134	-38.35684321
**3.867**	-38.39033752	-38.32652119	-38.35261412
**3.975**	-38.38679101	-38.32588635	-38.34863303
**4.082**	-38.38346420	-38.32533749	-38.34489118
**4.190**	-38.38034386	-38.32481987	-38.34137032
**4.297**	-38.37741709	-38.32433380	-38.33806095
**4.405**	-38.37467322	-38.32388262	-38.33495093
**4.513**	-38.37209718	-38.32347714	-38.33202845
**4.620**	-38.36967947	-38.32310719	-38.32928206
**4.728**	-38.36740969	-38.32325678	-38.32670073
**4.835**	-38.36527803	-38.32286834	-38.32427376
**4.943**	-38.36327527	-38.32251157	-38.32199075
**5.051**	-38.36139264	-38.32218423	-38.31984149
**5.158**	-38.35962168	-38.32188353	-38.31781966
**5.266**	-38.35795381	-38.32160687	-38.31590778
**5.373**	-38.35637952	-38.32135195	-38.31409861
**5.481**	-38.35488630	-38.32111679	-38.31238053
**5.589**	-38.35345338	-38.32089827	-38.31074146
**5.696**	-38.35204282	-38.32069776	-38.30916849
**5.804**	-38.35061239	-38.32051237	-38.30764879
**5.911**	-38.34918124	-38.32034084	-38.30617101
**6.019**	-38.34781462	-38.32018204	-38.30473376
**6.127**	-38.34655078	-38.32003493	-38.30332078
**6.234**	-38.34539694	-38.31989853	-38.30193787
**6.342**	-38.34434865	-38.31977201	-38.30058728
**6.449**	-38.34339805	-38.31965457	-38.29927215
**6.557**	-38.34253798	-38.31954545	-38.29800480
**6.665**	-38.34175802	-38.31944404	-38.29676945

R (Bohr)	$X\ ^3\Sigma^-$	$^3A', ^3A''$	$^1\Delta$
**6.772**	-38.34105171	-38.31934974	-38.29557604
**6.880**	-38.34041215	-38.31926200	-38.29443701
**6.987**	-38.33983307	-38.31918032	-38.29333074
**7.095**	-38.33930876	-38.31910425	-38.29226759
**7.203**	-38.33883403	-38.31903337	-38.29124722
**7.310**	-38.33840416	-38.31896730	-38.29026908
**7.418**	-38.33801488	-38.31890568	-38.28933243
**7.525**	-38.33766232	-38.31884819	-38.28843643
**7.633**	-38.33734295	-38.31879452	-38.28758008
**7.741**	-38.33705357	-38.31874440	-38.28677137
**7.848**	-38.33679130	-38.31869758	-38.28599145
**7.956**	-38.33655351	-38.31865381	-38.28524780
**8.063**	-38.33633783	-38.31861288	-38.28453923
**8.171**	-38.33614213	-38.31857458	-38.28386453
**8.278**	-38.33596446	-38.31853874	-38.28322246
**8.386**	-38.33580309	-38.31850517	-38.28261180
**8.494**	-38.33565643	-38.31847374	-38.28203130
**8.601**	-38.33552306	-38.31844427	-38.28147976
**8.709**	-38.33540171	-38.31841666	-38.28095595
**8.816**	-38.33529121	-38.31839076	-38.28045868
**8.924**	-38.33519053	-38.31836646	-38.27998680
**9.032**	-38.33509873	-38.31834367	-38.27953916
**9.139**	-38.33501497	-38.31832227	-38.27911464
**9.247**	-38.33493769	-38.31830219	-38.27871217
**9.354**	-38.33486785	-38.31828333	-38.27833068
**9.462**	-38.33480399	-38.31826563	-38.27796917
**9.570**	-38.33474556	-38.31824901	-38.27762665
**9.677**	-38.33469204	-38.31823340	-38.27730217
**9.785**	-38.33464300	-38.31821875	-38.27699482
**9.892**	-38.33459802	-38.31820500	-38.27670373
**10.000**	-38.33455673	-38.31819210	-38.27642805

PES aV5Z – CH^- (X $^3\Sigma^-$) + He					
**R (Bohr)/θ(º)**	**0.00**	**10.00**	**20.00**	**30.00**	**40.00**
**5.500000**	-41.39438736898	-41.39474040216	-41.39560563712	-41.39663369113	-41.39747393070
**5.655714**	-41.39561100805	-41.39586159324	-41.39651760879	-41.39730259490	-41.39794189458
**5.811429**	-41.39650328570	-41.39672055685	-41.39721811539	-41.39781796259	-41.39830304235
**5.967143**	-41.39721115514	-41.39737880864	-41.39775682565	-41.39821539365	-41.39858177512
**6.122857**	-41.39775028220	-41.39788351416	-41.39817188757	-41.39852194034	-41.39879685058
**6.278571**	-41.39816077782	-41.39827085862	-41.39849215876	-41.39875828171	-41.39896270079
**6.434286**	-41.39847408703	-41.39856845679	-41.39873937341	-41.39894012734	-41.39909047476
**6.590000**	-41.39871423014	-41.39879730911	-41.39892978122	-41.39907952177	-41.39918870431
**6.745714**	-41.39889914463	-41.39897337883	-41.39907572036	-41.39918584828	-41.39926399606
**6.901429**	-41.39904192087	-41.39910855116	-41.39918671466	-41.39926645054	-41.39932147158
**7.057143**	-41.39915200292	-41.39921176292	-41.39927029057	-41.39932714546	-41.39936502841
**7.212857**	-41.39923625387	-41.39928977543	-41.39933251493	-41.39937251563	-41.39939772674
**7.368571**	-41.39929984427	-41.39934788766	-41.39937831202	-41.39940611007	-41.39942190140
**7.524286**	-41.39934694770	-41.39939039774	-41.39941161020	-41.39943065848	-41.39943938203
**7.680000**	-41.39938110016	-41.39942087544	-41.39943546467	-41.39944819434	-41.39945158889
**7.835714**	-41.39940532890	-41.39944226178	-41.39945221313	-41.39946029222	-41.39945964661
**7.991429**	-41.39942212905	-41.39945686164	-41.39946356910	-41.39946815304	-41.39946445563
**8.147143**	-41.39943346980	-41.39946642104	-41.39947080691	-41.39947268441	-41.39946679398
**8.302857**	-41.39944055318	-41.39947222992	-41.39947487150	-41.39947461928	-41.39946717611
**8.458571**	-41.39944498530	-41.39947495121	-41.39947647034	-41.39947454358	-41.39946609797
**8.614286**	-41.39944722590	-41.39947576664	-41.39947582804	-41.39947293365	-41.39946394582
**8.770000**	-41.39944774157	-41.39947483223	-41.39947403650	-41.39947018528	-41.39946103447
**8.925714**	-41.39944703636	-41.39947197819	-41.39947111279	-41.39946634363	-41.39945757760
**9.081429**	-41.39944534151	-41.39946823189	-41.39946705131	-41.39946223971	-41.39945375719
**9.237143**	-41.39944289964	-41.39946099532	-41.39946098422	-41.39945563420	-41.39944825309
**9.392857**	-41.39943991174	-41.39945275779	-41.39945252956	-41.39944955192	-41.39944332582
**9.548571**	-41.39943655188	-41.39944741253	-41.39944551420	-41.39944166112	-41.39943994442
**9.704286**	-41.39943296294	-41.39944229692	-41.39944055003	-41.39943708888	-41.39943491998
**9.860000**	-41.39942926323	-41.39943593426	-41.39943561004	-41.39943260344	-41.39942827842
**10.015714**	-41.39942553497	-41.39942857971	-41.39943076575	-41.39942822240	-41.39942450906
**10.171429**	-41.39942186320	-41.39942421615	-41.39942604310	-41.39942395182	-41.39942083220
**10.327143**	-41.39941829170	-41.39942023812	-41.39942143284	-41.39941980968	-41.39941725355
**10.482857**	-41.39941481257	-41.39941639617	-41.39941739750	-41.39941579528	-41.39941378889
**10.638571**	-41.39941147985	-41.39941274871	-41.39941357122	-41.39941222650	-41.39941043481
**10.794286**	-41.39940828800	-41.39940927779	-41.39940993853	-41.39940881547	-41.39940718078
**10.950000**	-41.39940524259	-41.39940598329	-41.39940650006	-41.39940557197	-41.39940421337
**11.105714**	-41.39940233724	-41.39940287361	-41.39940326024	-41.39940249973	-41.39940137815
**11.261429**	-41.39939956812	-41.39939993749	-41.39939951932	-41.39939959183	-41.39939869522
**11.417143**	-41.39939693157	-41.39939716404	-41.39939679673	-41.39939684567	-41.39939615010
**11.572857**	-41.39939442378	-41.39939455505	-41.39939422242	-41.39939425228	-41.39939373926
**11.728571**	-41.39939204085	-41.39939208531	-41.39939178495	-41.39939180553	-41.39939145897
**11.884286**	-41.39938977882	-41.39938975990	-41.39938948828	-41.39938951498	-41.39938930151
**12.040000**	-41.39938763613	-41.39938749560	-41.39938733348	-41.39938701942	-41.39938726524
**12.195714**	-41.39938560472	-41.39938548769	-41.39938530901	-41.39938504394	-41.39938534576
**12.351429**	-41.39938368327	-41.39938358381	-41.39938339706	-41.39938318437	-41.39938353992
**12.507143**	-41.39938186847	-41.39938177057	-41.39938160023	-41.39938143620	-41.39938140669
**12.662857**	-41.39938015647	-41.39938007065	-41.39937990390	-41.39937979189	-41.39937982791
**12.818571**	-41.39937854416	-41.39937847102	-41.39937831471	-41.39937824222	-41.39937833692
**12.974286**	-41.39937702758	-41.39937696651	-41.39937682575	-41.39937678307	-41.39937692913
**13.130000**	-41.39937560227	-41.39937555251	-41.39937542953	-41.39937541179	-41.39937560333
**13.285714**	-41.39937426339	-41.39937422411	-41.39937411873	-41.39937412349	-41.39937435793
**13.441429**	-41.39937300597	-41.39937297649	-41.39937290300	-41.39937291363	-41.39937318384
**13.597143**	-41.39937182513	-41.39937180669	-41.39937174495	-41.39937177525	-41.39937207878
**13.752857**	-41.39937071834	-41.39937070257	-41.39937065549	-41.39937070581	-41.39937100218
**13.908571**	-41.39936967206	-41.39936966013	-41.39936963091	-41.39936969987	-41.39937002325
**14.064286**	-41.39936869128	-41.39936868333	-41.39936866645	-41.39936873536	-41.39936910293
**14.220000**	-41.39936776728	-41.39936776257	-41.39936775164	-41.39936776221	-41.39936823678
**14.375714**	-41.39936691326	-41.39936690458	-41.39936689369	-41.39936691712	-41.39936742327
**14.531429**	-41.39936610336	-41.39936609659	-41.39936609242	-41.39936612261	-41.39936666014
**14.687143**	-41.39936533688	-41.39936533335	-41.39936533558	-41.39936537249	-41.39936547512
**14.842857**	-41.39936461177	-41.39936461095	-41.39936461881	-41.39936466155	-41.39936476756
**14.998571**	-41.39936392725	-41.39936392849	-41.39936394122	-41.39936398861	-41.39936409654
**15.154286**	-41.39936328097	-41.39936328390	-41.39936330066	-41.39936335168	-41.39936346023
**15.310000**	-41.39936267054	-41.39936267489	-41.39936269495	-41.39936274858	-41.39936285670

R (Bohr)/θ(º)	0.00	10.00	20.00	30.00	40.00
**15.465714**	-41.39936209378	-41.39936209929	-41.39936212204	-41.39936217744	-41.39936228412
**15.621429**	-41.39936154850	-41.39936155496	-41.39936157983	-41.39936163631	-41.39936174065
**15.777143**	-41.39936103279	-41.39936104002	-41.39936106649	-41.39936112347	-41.39936122463
**15.932857**	-41.39936054484	-41.39936055265	-41.39936058029	-41.39936063727	-41.39936073445
**16.088571**	-41.39936008292	-41.39936009116	-41.39936011957	-41.39936017617	-41.39936026857
**16.244286**	-41.39935964543	-41.39935965395	-41.39935968272	-41.39935973861	-41.39935982546
**16.400000**	-41.39935923082	-41.39935923938	-41.39935926778	-41.39935932243	-41.39935940319
**16.555714**	-41.39935882615	-41.39935883396	-41.39935886259	-41.39935891946	-41.39935899722
**16.711429**	-41.39935846436	-41.39935847266	-41.39935849951	-41.39935854869	-41.39935860237
**16.867143**	-41.39935811081	-41.39935811937	-41.39935814757	-41.39935820186	-41.39935826009
**17.022857**	-41.39935777459	-41.39935778298	-41.39935781064	-41.39935786429	-41.39935793638
**17.178571**	-41.39935745478	-41.39935746283	-41.39935748931	-41.39935753957	-41.39935758727
**17.334286**	-41.39935715024	-41.39935715776	-41.39935718252	-41.39935722559	-41.39935726840
**17.490000**	-41.39935685986	-41.39935685826	-41.39935688635	-41.39935691935	-41.39935696684
**17.645714**	-41.39935658262	-41.39935658881	-41.39935660784	-41.39935663843	-41.39935668556
**17.801429**	-41.39935631764	-41.39935632294	-41.39935633919	-41.39935636689	-41.39935641597
**17.957143**	-41.39935606426	-41.39935606869	-41.39935608249	-41.39935610823	-41.39935615754
**18.112857**	-41.39935582202	-41.39935582569	-41.39935583759	-41.39935586210	-41.39935591039
**18.268571**	-41.39935559057	-41.39935559363	-41.39935560414	-41.39935562767	-41.39935567417
**18.424286**	-41.39935536960	-41.39935537219	-41.39935538165	-41.39935540415	-41.39935544842
**18.580000**	-41.39935515874	-41.39935516094	-41.39935516951	-41.39935519080	-41.39935523257
**18.735714**	-41.39935495753	-41.39935495938	-41.39935496708	-41.39935498697	-41.39935502611
**18.891429**	-41.39935476549	-41.39935476700	-41.39935477375	-41.39935479206	-41.39935482854
**19.047143**	-41.39935458209	-41.39935458322	-41.39935458893	-41.39935460551	-41.39935463929
**19.202857**	-41.39935440682	-41.39935440753	-41.39935441205	-41.39935442675	-41.39935445787
**19.358571**	-41.39935423918	-41.39935423939	-41.39935424255	-41.39935425522	-41.39935428370
**19.514286**	-41.39935407867	-41.39935407826	-41.39935407981	-41.39935409024	-41.39935411605
**19.670000**	-41.39935392483	-41.39935392357	-41.39935392299	-41.39935393081	-41.39935395390
**19.825714**	-41.39935374073	-41.39935375754	-41.39935378080	-41.39935378077	-41.39935379809
**19.981429**	-41.39935363586	-41.39935361971	-41.39935360154	-41.39935359892	-41.39935357304
**20.137143**	-41.39935350182	-41.39935348471	-41.39935345080	-41.39935343542	-41.39935345018
**20.292857**	-41.39935337212	-41.39935336727	-41.39935335731	-41.39935335093	-41.39935335255
**20.448571**	-41.39935324747	-41.39935324220	-41.39935323266	-41.39935323035	-41.39935324147
**20.604286**	-41.39935312770	-41.39935312072	-41.39935310834	-41.39935310488	-41.39935311708
**20.760000**	-41.39935301259	-41.39935300280	-41.39935298595	-41.39935298026	-41.39935299210
**20.915714**	-41.39935290192	-41.39935288739	-41.39935286423	-41.39935285631	-41.39935286810
**21.071429**	-41.39935279547	-41.39935277208	-41.39935274039	-41.39935273153	-41.39935274466
**21.227143**	-41.39935269306	-41.39935264993	-41.39935260938	-41.39935260400	-41.39935262105
**21.382857**	-41.39935259448	-41.39935249783	-41.39935246641	-41.39935247393	-41.39935249786
**21.538571**	-41.39935249951	-41.39935232096	-41.39935233187	-41.39935235160	-41.39935238008
**21.694286**	-41.39935240817	-41.39935232843	-41.39935227097	-41.39935226378	-41.39935228066
**21.850000**	-41.39935232010	-41.39935229542	-41.39935224902	-41.39935221816	-41.39935221240
**22.005714**	-41.39935223523	-41.39935222667	-41.39935220507	-41.39935218155	-41.39935216730
**22.161429**	-41.39935215341	-41.39935215065	-41.39935214289	-41.39935213267	-41.39935212438
**22.317143**	-41.39935207451	-41.39935207425	-41.39935207344	-41.39935207261	-41.39935207307
**22.472857**	-41.39935199840	-41.39935199935	-41.39935200210	-41.39935200683	-41.39935201393
**22.628571**	-41.39935192497	-41.39935192653	-41.39935193117	-41.39935193904	-41.39935195031
**22.784286**	-41.39935185410	-41.39935185599	-41.39935186164	-41.39935187123	-41.39935188493
**22.940000**	-41.39935178567	-41.39935178774	-41.39935179392	-41.39935180443	-41.39935181945
**23.095714**	-41.39935171960	-41.39935172175	-41.39935172819	-41.39935173915	-41.39935175481
**23.251429**	-41.39935165578	-41.39935165796	-41.39935166450	-41.39935167562	-41.39935169151
**23.407143**	-41.39935159412	-41.39935159630	-41.39935160284	-41.39935161395	-41.39935162982
**23.562857**	-41.39935153453	-41.39935153669	-41.39935154317	-41.39935155416	-41.39935156987
**23.718571**	-41.39935147693	-41.39935147905	-41.39935148543	-41.39935149626	-41.39935151169
**23.874286**	-41.39935142123	-41.39935142332	-41.39935142957	-41.39935144018	-41.39935145531
**24.030000**	-41.39935136736	-41.39935136941	-41.39935137553	-41.39935138590	-41.39935140067
**24.185714**	-41.39935131525	-41.39935131725	-41.39935132323	-41.39935133336	-41.39935134776
**24.341429**	-41.39935126483	-41.39935126678	-41.39935127262	-41.39935128249	-41.39935129652
**24.497143**	-41.39935121603	-41.39935121793	-41.39935122363	-41.39935123325	-41.39935124690
**24.652857**	-41.39935116880	-41.39935117065	-41.39935117620	-41.39935118556	-41.39935119884
**24.808571**	-41.39935112305	-41.39935112486	-41.39935113027	-41.39935113938	-41.39935115230
**24.964286**	-41.39935107876	-41.39935108052	-41.39935108578	-41.39935109465	-41.39935110721
**25.120000**	-41.39935103585	-41.39935103756	-41.39935104269	-41.39935105132	-41.39935106353
**25.275714**	-41.39935099428	-41.39935099594	-41.39935100094	-41.39935100933	-41.39935102120
**25.431429**	-41.39935095398	-41.39935095561	-41.39935096047	-41.39935096864	-41.39935098018

R (Bohr)/θ(º)	0.00	10.00	20.00	30.00	40.00
**25.587143**	-41.39935091493	-41.39935091652	-41.39935092125	-41.39935092919	-41.39935094041
**25.742857**	-41.39935087707	-41.39935087862	-41.39935088322	-41.39935089095	-41.39935090186
**25.898571**	-41.39935084036	-41.39935084186	-41.39935084634	-41.39935085387	-41.39935086447
**26.054286**	-41.39935080475	-41.39935080621	-41.39935081058	-41.39935081790	-41.39935082821
**26.210000**	-41.39935077021	-41.39935077163	-41.39935077588	-41.39935078301	-41.39935079304
**26.365714**	-41.39935073670	-41.39935073808	-41.39935074222	-41.39935074915	-41.39935075890
**26.521429**	-41.39935070418	-41.39935070552	-41.39935070955	-41.39935071630	-41.39935072578
**26.677143**	-41.39935067261	-41.39935067392	-41.39935067784	-41.39935068441	-41.39935069364
**26.832857**	-41.39935064196	-41.39935064325	-41.39935064706	-41.39935065345	-41.39935066243
**26.988571**	-41.39935061222	-41.39935061346	-41.39935061717	-41.39935062339	-41.39935063213
**27.144286**	-41.39935058333	-41.39935058454	-41.39935058815	-41.39935059420	-41.39935060270
**27.300000**	-41.39935055528	-41.39935055646	-41.39935055997	-41.39935056586	-41.39935057412
**27.455714**	-41.39935052805	-41.39935052919	-41.39935053261	-41.39935053833	-41.39935054636
**27.611429**	-41.39935050163	-41.39935050274	-41.39935050604	-41.39935051159	-41.39935051940
**27.767143**	-41.39935047609	-41.39935047714	-41.39935048030	-41.39935048565	-41.39935049321
**27.922857**	-41.39935045193	-41.39935045283	-41.39935045564	-41.39935046060	-41.39935046782
**28.078571**	-41.39935042838	-41.39935043627	-41.39935043726	-41.39935043796	-41.39935044357
**28.234286**	-41.39935040399	-41.39935040510	-41.39935040852	-41.39935041506	-41.39935042135
**28.390000**	-41.39935037979	-41.39935038079	-41.39935038375	-41.39935038870	-41.39935039565
**28.545714**	-41.39935035722	-41.39935035820	-41.39935036108	-41.39935036582	-41.39935037259
**28.701429**	-41.39935033540	-41.39935033634	-41.39935033916	-41.39935034384	-41.39935035038
**28.857143**	-41.39935031419	-41.39935031512	-41.39935031786	-41.39935032243	-41.39935032880
**29.012857**	-41.39935029356	-41.39935029446	-41.39935029714	-41.39935030160	-41.39935030781
**29.168571**	-41.39935027349	-41.39935027437	-41.39935027698	-41.39935028133	-41.39935028738
**29.324286**	-41.39935025395	-41.39935025481	-41.39935025736	-41.39935026160	-41.39935026750
**29.480000**	-41.39935023493	-41.39935023577	-41.39935023826	-41.39935024239	-41.39935024815
**29.635714**	-41.39935021642	-41.39935021723	-41.39935021966	-41.39935022369	-41.39935022931
**29.791429**	-41.39935019838	-41.39935019918	-41.39935020155	-41.39935020548	-41.39935021096
**29.947143**	-41.39935018082	-41.39935018160	-41.39935018391	-41.39935018775	-41.39935019309
**30.102857**	-41.39935016371	-41.39935016447	-41.39935016673	-41.39935017047	-41.39935017568
**30.258571**	-41.39935014704	-41.39935014779	-41.39935014999	-41.39935015364	-41.39935015872
**30.414286**	-41.39935013080	-41.39935013153	-41.39935013368	-41.39935013724	-41.39935014220
**30.570000**	-41.39935011499	-41.39935011569	-41.39935011779	-41.39935012127	-41.39935012610
**30.725714**	-41.39935009959	-41.39935010027	-41.39935010231	-41.39935010570	-41.39935011041
**30.881429**	-41.39935008519	-41.39935008567	-41.39935008739	-41.39935009058	-41.39935009513
**31.037143**	-41.39935006996	-41.39935007064	-41.39935007266	-41.39935007642	-41.39935008195
**31.192857**	-41.39935005555	-41.39935005620	-41.39935005811	-41.39935006128	-41.39935006568
**31.348571**	-41.39935004161	-41.39935004223	-41.39935004410	-41.39935004719	-41.39935005148
**31.504286**	-41.39935002801	-41.39935002862	-41.39935003044	-41.39935003346	-41.39935003765
**31.660000**	-41.39935001474	-41.39935001534	-41.39935001712	-41.39935002008	-41.39935002417
**31.815714**	-41.39935000180	-41.39935000239	-41.39935000413	-41.39935000701	-41.39935001101
**31.971429**	-41.39934998918	-41.39934998975	-41.39934999145	-41.39934999427	-41.39934999818
**32.127143**	-41.39934997686	-41.39934997742	-41.39934997908	-41.39934998184	-41.39934998565
**32.282857**	-41.39934996484	-41.39934996539	-41.39934996701	-41.39934996971	-41.39934997343
**32.438571**	-41.39934995310	-41.39934995364	-41.39934995523	-41.39934995786	-41.39934996150
**32.594286**	-41.39934994166	-41.39934994218	-41.39934994373	-41.39934994630	-41.39934994986
**32.750000**	-41.39934993047	-41.39934993099	-41.39934993251	-41.39934993502	-41.39934993849
**32.905714**	-41.39934991956	-41.39934992006	-41.39934992154	-41.39934992400	-41.39934992740
**33.061429**	-41.39934990890	-41.39934990939	-41.39934991084	-41.39934991324	-41.39934991656
**33.217143**	-41.39934989848	-41.39934989897	-41.39934990039	-41.39934990274	-41.39934990598
**33.372857**	-41.39934988832	-41.39934988879	-41.39934989018	-41.39934989247	-41.39934989565
**33.528571**	-41.39934987839	-41.39934987884	-41.39934988020	-41.39934988245	-41.39934988555
**33.684286**	-41.39934986868	-41.39934986913	-41.39934987046	-41.39934987266	-41.39934987569
**33.840000**	-41.39934985920	-41.39934985964	-41.39934986094	-41.39934986309	-41.39934986606
**33.995714**	-41.39934984994	-41.39934985037	-41.39934985164	-41.39934985374	-41.39934985665
**34.151429**	-41.39934984089	-41.39934984131	-41.39934984255	-41.39934984461	-41.39934984745
**34.307143**	-41.39934983204	-41.39934983245	-41.39934983367	-41.39934983568	-41.39934983846
**34.462857**	-41.39934982339	-41.39934982379	-41.39934982499	-41.39934982695	-41.39934982967
**34.618571**	-41.39934981494	-41.39934981533	-41.39934981650	-41.39934981842	-41.39934982108
**34.774286**	-41.39934980667	-41.39934980706	-41.39934980820	-41.39934981008	-41.39934981269
**34.930000**	-41.39934979859	-41.39934979897	-41.39934980009	-41.39934980193	-41.39934980448
**35.085714**	-41.39934979069	-41.39934979106	-41.39934979215	-41.39934979396	-41.39934979645
**35.241429**	-41.39934978296	-41.39934978332	-41.39934978439	-41.39934978616	-41.39934978860
**35.397143**	-41.39934977540	-41.39934977575	-41.39934977680	-41.39934977854	-41.39934978092
**35.552857**	-41.39934976800	-41.39934976835	-41.39934976938	-41.39934977108	-41.39934977341

R (Bohr)/θ(º)	0.00	10.00	20.00	30.00	40.00
**35.708571**	-41.39934976077	-41.39934976111	-41.39934976212	-41.39934976378	-41.39934976607
**35.864286**	-41.39934975369	-41.39934975403	-41.39934975501	-41.39934975664	-41.39934975888
**36.020000**	-41.39934974677	-41.39934974710	-41.39934974806	-41.39934974965	-41.39934975185
**36.175714**	-41.39934973999	-41.39934974031	-41.39934974126	-41.39934974282	-41.39934974497
**36.331429**	-41.39934973336	-41.39934973368	-41.39934973460	-41.39934973613	-41.39934973824
**36.487143**	-41.39934972687	-41.39934972718	-41.39934972809	-41.39934972959	-41.39934973165
**36.642857**	-41.39934972052	-41.39934972082	-41.39934972171	-41.39934972318	-41.39934972520
**36.798571**	-41.39934971430	-41.39934971460	-41.39934971547	-41.39934971691	-41.39934971889
**36.954286**	-41.39934970822	-41.39934970851	-41.39934970936	-41.39934971077	-41.39934971271
**37.110000**	-41.39934970226	-41.39934970254	-41.39934970338	-41.39934970476	-41.39934970666
**37.265714**	-41.39934969642	-41.39934969670	-41.39934969752	-41.39934969887	-41.39934970073
**37.421429**	-41.39934969071	-41.39934969098	-41.39934969178	-41.39934969311	-41.39934969493
**37.577143**	-41.39934968511	-41.39934968537	-41.39934968616	-41.39934968746	-41.39934968925
**37.732857**	-41.39934967963	-41.39934967989	-41.39934968066	-41.39934968194	-41.39934968369
**37.888571**	-41.39934967426	-41.39934967451	-41.39934967527	-41.39934967652	-41.39934967824
**38.044286**	-41.39934966900	-41.39934966925	-41.39934966999	-41.39934967122	-41.39934967290
**38.200000**	-41.39934966385	-41.39934966409	-41.39934966482	-41.39934966602	-41.39934966767
**38.355714**	-41.39934965880	-41.39934965904	-41.39934965976	-41.39934966093	-41.39934966255
**38.511429**	-41.39934965385	-41.39934965409	-41.39934965479	-41.39934965594	-41.39934965753
**38.667143**	-41.39934964900	-41.39934964923	-41.39934964992	-41.39934965106	-41.39934965261
**38.822857**	-41.39934964425	-41.39934964448	-41.39934964516	-41.39934964626	-41.39934964779
**38.978571**	-41.39934963959	-41.39934963982	-41.39934964048	-41.39934964157	-41.39934964307
**39.134286**	-41.39934963503	-41.39934963525	-41.39934963590	-41.39934963697	-41.39934963844
**39.290000**	-41.39934963056	-41.39934963077	-41.39934963141	-41.39934963246	-41.39934963390
**39.445714**	-41.39934962617	-41.39934962638	-41.39934962701	-41.39934962804	-41.39934962945
**39.601429**	-41.39934962187	-41.39934962208	-41.39934962270	-41.39934962370	-41.39934962509
**39.757143**	-41.39934961766	-41.39934961786	-41.39934961846	-41.39934961946	-41.39934962082
**39.912857**	-41.39934961352	-41.39934961373	-41.39934961432	-41.39934961529	-41.39934961662
**40.068571**	-41.39934960947	-41.39934960967	-41.39934961025	-41.39934961120	-41.39934961251
**40.224286**	-41.39934960550	-41.39934960569	-41.39934960626	-41.39934960719	-41.39934960848
**40.380000**	-41.39934960160	-41.39934960178	-41.39934960234	-41.39934960326	-41.39934960452
**40.535714**	-41.39934959777	-41.39934959796	-41.39934959851	-41.39934959941	-41.39934960064
**40.691429**	-41.39934959402	-41.39934959420	-41.39934959474	-41.39934959563	-41.39934959684
**40.847143**	-41.39934959034	-41.39934959052	-41.39934959105	-41.39934959192	-41.39934959311
**41.002857**	-41.39934958673	-41.39934958691	-41.39934958742	-41.39934958828	-41.39934958945
**41.158571**	-41.39934958319	-41.39934958336	-41.39934958387	-41.39934958471	-41.39934958586
**41.314286**	-41.39934957971	-41.39934957988	-41.39934958038	-41.39934958120	-41.39934958233
**41.470000**	-41.39934957630	-41.39934957647	-41.39934957696	-41.39934957777	-41.39934957887
**41.625714**	-41.39934957295	-41.39934957311	-41.39934957360	-41.39934957439	-41.39934957548
**41.781429**	-41.39934956967	-41.39934956983	-41.39934957030	-41.39934957108	-41.39934957215
**41.937143**	-41.39934956644	-41.39934956660	-41.39934956707	-41.39934956784	-41.39934956888
**42.092857**	-41.39934956328	-41.39934956343	-41.39934956389	-41.39934956464	-41.39934956567
**42.248571**	-41.39934956017	-41.39934956032	-41.39934956078	-41.39934956151	-41.39934956253
**42.404286**	-41.39934955712	-41.39934955727	-41.39934955772	-41.39934955844	-41.39934955943
**42.560000**	-41.39934955413	-41.39934955427	-41.39934955471	-41.39934955542	-41.39934955640
**42.715714**	-41.39934955119	-41.39934955133	-41.39934955176	-41.39934955246	-41.39934955342
**42.871429**	-41.39934954830	-41.39934954844	-41.39934954886	-41.39934954955	-41.39934955049
**43.027143**	-41.39934954547	-41.39934954560	-41.39934954602	-41.39934954669	-41.39934954762
**43.182857**	-41.39934954268	-41.39934954282	-41.39934954322	-41.39934954389	-41.39934954480
**43.338571**	-41.39934953995	-41.39934954009	-41.39934954048	-41.39934954114	-41.39934954203
**43.494286**	-41.39934953727	-41.39934953740	-41.39934953779	-41.39934953843	-41.39934953931
**43.650000**	-41.39934953463	-41.39934953476	-41.39934953514	-41.39934953577	-41.39934953664
**43.805714**	-41.39934953204	-41.39934953216	-41.39934953254	-41.39934953316	-41.39934953401
**43.961429**	-41.39934952949	-41.39934952962	-41.39934952999	-41.39934953060	-41.39934953143
**44.117143**	-41.39934952699	-41.39934952711	-41.39934952748	-41.39934952808	-41.39934952890
**44.272857**	-41.39934952454	-41.39934952466	-41.39934952502	-41.39934952561	-41.39934952641
**44.428571**	-41.39934952213	-41.39934952224	-41.39934952260	-41.39934952317	-41.39934952397
**44.584286**	-41.39934951975	-41.39934951987	-41.39934952022	-41.39934952079	-41.39934952156
**44.740000**	-41.39934951742	-41.39934951754	-41.39934951788	-41.39934951844	-41.39934951921
**44.895714**	-41.39934951513	-41.39934951525	-41.39934951558	-41.39934951613	-41.39934951689
**45.051429**	-41.39934951288	-41.39934951299	-41.39934951332	-41.39934951387	-41.39934951461
**45.207143**	-41.39934951067	-41.39934951078	-41.39934951110	-41.39934951164	-41.39934951237
**45.362857**	-41.39934950850	-41.39934950860	-41.39934950892	-41.39934950945	-41.39934951016
**45.518571**	-41.39934950636	-41.39934950647	-41.39934950678	-41.39934950730	-41.39934950800
**45.674286**	-41.39934950426	-41.39934950436	-41.39934950467	-41.39934950518	-41.39934950587

R (Bohr)/θ(º)	0.00	10.00	20.00	30.00	40.00
**45.830000**	-41.39934950219	-41.39934950230	-41.39934950260	-41.39934950310	-41.39934950378
**45.985714**	-41.39934950016	-41.39934950027	-41.39934950056	-41.39934950106	-41.39934950173
**46.141429**	-41.39934949817	-41.39934949827	-41.39934949856	-41.39934949904	-41.39934949970
**46.297143**	-41.39934949621	-41.39934949631	-41.39934949659	-41.39934949707	-41.39934949772
**46.452857**	-41.39934949427	-41.39934949437	-41.39934949466	-41.39934949513	-41.39934949576
**46.608571**	-41.39934949238	-41.39934949247	-41.39934949275	-41.39934949321	-41.39934949384
**46.764286**	-41.39934949051	-41.39934949061	-41.39934949088	-41.39934949133	-41.39934949195
**46.920000**	-41.39934948868	-41.39934948877	-41.39934948904	-41.39934948949	-41.39934949010
**47.075714**	-41.39934948687	-41.39934948696	-41.39934948723	-41.39934948767	-41.39934948827
**47.231429**	-41.39934948510	-41.39934948519	-41.39934948545	-41.39934948588	-41.39934948647
**47.387143**	-41.39934948335	-41.39934948344	-41.39934948370	-41.39934948412	-41.39934948470
**47.542857**	-41.39934948163	-41.39934948172	-41.39934948197	-41.39934948239	-41.39934948297
**47.698571**	-41.39934947994	-41.39934948003	-41.39934948028	-41.39934948069	-41.39934948125
**47.854286**	-41.39934947828	-41.39934947837	-41.39934947861	-41.39934947902	-41.39934947957
**48.010000**	-41.39934947665	-41.39934947673	-41.39934947697	-41.39934947737	-41.39934947791
**48.165714**	-41.39934947504	-41.39934947512	-41.39934947536	-41.39934947575	-41.39934947629
**48.321429**	-41.39934947346	-41.39934947354	-41.39934947377	-41.39934947416	-41.39934947469
**48.477143**	-41.39934947190	-41.39934947198	-41.39934947221	-41.39934947259	-41.39934947311
**48.632857**	-41.39934947037	-41.39934947044	-41.39934947067	-41.39934947105	-41.39934947156
**48.788571**	-41.39934946886	-41.39934946893	-41.39934946916	-41.39934946953	-41.39934947003
**48.944286**	-41.39934946737	-41.39934946745	-41.39934946767	-41.39934946803	-41.39934946853
**49.100000**	-41.39934946591	-41.39934946599	-41.39934946621	-41.39934946656	-41.39934946705
**49.255714**	-41.39934946447	-41.39934946455	-41.39934946476	-41.39934946511	-41.39934946560
**49.411429**	-41.39934946306	-41.39934946313	-41.39934946334	-41.39934946369	-41.39934946416
**49.567143**	-41.39934946167	-41.39934946174	-41.39934946195	-41.39934946229	-41.39934946275
**49.722857**	-41.39934946029	-41.39934946036	-41.39934946057	-41.39934946091	-41.39934946136
**49.878571**	-41.39934945895	-41.39934945901	-41.39934945922	-41.39934945955	-41.39934946000
**50.034286**	-41.39934945762	-41.39934945768	-41.39934945788	-41.39934945821	-41.39934945865
**50.190000**	-41.39934945631	-41.39934945637	-41.39934945657	-41.39934945689	-41.39934945733
**50.345714**	-41.39934945502	-41.39934945508	-41.39934945528	-41.39934945559	-41.39934945603
**50.501429**	-41.39934945375	-41.39934945382	-41.39934945401	-41.39934945432	-41.39934945475
**50.657143**	-41.39934945250	-41.39934945257	-41.39934945275	-41.39934945306	-41.39934945348
**50.812857**	-41.39934945127	-41.39934945134	-41.39934945152	-41.39934945182	-41.39934945224
**50.968571**	-41.39934945006	-41.39934945012	-41.39934945031	-41.39934945060	-41.39934945101
**51.124286**	-41.39934944887	-41.39934944893	-41.39934944911	-41.39934944940	-41.39934944981
**51.280000**	-41.39934944769	-41.39934944775	-41.39934944793	-41.39934944822	-41.39934944862
**51.435714**	-41.39934944654	-41.39934944660	-41.39934944677	-41.39934944706	-41.39934944745
**51.591429**	-41.39934944540	-41.39934944546	-41.39934944563	-41.39934944591	-41.39934944629
**51.747143**	-41.39934944428	-41.39934944433	-41.39934944450	-41.39934944478	-41.39934944516
**51.902857**	-41.39934944317	-41.39934944323	-41.39934944340	-41.39934944367	-41.39934944404
**52.058571**	-41.39934944208	-41.39934944214	-41.39934944230	-41.39934944257	-41.39934944294
**52.214286**	-41.39934944101	-41.39934944107	-41.39934944123	-41.39934944149	-41.39934944185
**52.370000**	-41.39934943995	-41.39934944001	-41.39934944017	-41.39934944043	-41.39934944079
**52.525714**	-41.39934943891	-41.39934943896	-41.39934943912	-41.39934943938	-41.39934943973
**52.681429**	-41.39934943789	-41.39934943794	-41.39934943810	-41.39934943835	-41.39934943870
**52.837143**	-41.39934943688	-41.39934943693	-41.39934943708	-41.39934943733	-41.39934943767
**52.992857**	-41.39934943588	-41.39934943593	-41.39934943608	-41.39934943633	-41.39934943667
**53.148571**	-41.39934943490	-41.39934943495	-41.39934943510	-41.39934943534	-41.39934943567
**53.304286**	-41.39934943393	-41.39934943398	-41.39934943413	-41.39934943437	-41.39934943469
**53.460000**	-41.39934943298	-41.39934943303	-41.39934943317	-41.39934943341	-41.39934943373
**53.615714**	-41.39934943204	-41.39934943209	-41.39934943223	-41.39934943247	-41.39934943278
**53.771429**	-41.39934943111	-41.39934943116	-41.39934943130	-41.39934943153	-41.39934943185
**53.927143**	-41.39934943020	-41.39934943025	-41.39934943039	-41.39934943061	-41.39934943092
**54.082857**	-41.39934942930	-41.39934942935	-41.39934942949	-41.39934942971	-41.39934943002
**54.238571**	-41.39934942842	-41.39934942846	-41.39934942860	-41.39934942882	-41.39934942912
**54.394286**	-41.39934942754	-41.39934942759	-41.39934942772	-41.39934942794	-41.39934942824
**54.550000**	-41.39934942668	-41.39934942673	-41.39934942686	-41.39934942708	-41.39934942737
**54.705714**	-41.39934942583	-41.39934942588	-41.39934942601	-41.39934942622	-41.39934942651
**54.861429**	-41.39934942500	-41.39934942504	-41.39934942517	-41.39934942538	-41.39934942566
**55.017143**	-41.39934942417	-41.39934942422	-41.39934942434	-41.39934942455	-41.39934942483
**55.172857**	-41.39934942336	-41.39934942340	-41.39934942353	-41.39934942373	-41.39934942401
**55.328571**	-41.39934942256	-41.39934942260	-41.39934942272	-41.39934942292	-41.39934942320
**55.484286**	-41.39934942177	-41.39934942181	-41.39934942193	-41.39934942213	-41.39934942240
**55.640000**	-41.39934942099	-41.39934942103	-41.39934942115	-41.39934942134	-41.39934942161
**55.795714**	-41.39934942022	-41.39934942026	-41.39934942038	-41.39934942057	-41.39934942083

R (Bohr)/θ(º)	0.00	10.00	20.00	30.00	40.00
**55.951429**	-41.39934941946	-41.39934941950	-41.39934941962	-41.39934941981	-41.39934942007
**56.107143**	-41.39934941872	-41.39934941875	-41.39934941887	-41.39934941905	-41.39934941931
**56.262857**	-41.39934941798	-41.39934941802	-41.39934941813	-41.39934941831	-41.39934941857
**56.418571**	-41.39934941725	-41.39934941729	-41.39934941740	-41.39934941758	-41.39934941783
**56.574286**	-41.39934941653	-41.39934941657	-41.39934941668	-41.39934941686	-41.39934941711
**56.730000**	-41.39934941583	-41.39934941587	-41.39934941597	-41.39934941615	-41.39934941639
**56.885714**	-41.39934941513	-41.39934941517	-41.39934941527	-41.39934941545	-41.39934941569
**57.041429**	-41.39934941444	-41.39934941448	-41.39934941458	-41.39934941476	-41.39934941499
**57.197143**	-41.39934941376	-41.39934941380	-41.39934941390	-41.39934941408	-41.39934941431
**57.352857**	-41.39934941309	-41.39934941313	-41.39934941323	-41.39934941340	-41.39934941363
**57.508571**	-41.39934941243	-41.39934941247	-41.39934941257	-41.39934941274	-41.39934941296
**57.664286**	-41.39934941179	-41.39934941182	-41.39934941192	-41.39934941208	-41.39934941231
**57.820000**	-41.39934941114	-41.39934941117	-41.39934941127	-41.39934941144	-41.39934941166
**57.975714**	-41.39934941051	-41.39934941054	-41.39934941064	-41.39934941080	-41.39934941101
**58.131429**	-41.39934940988	-41.39934940992	-41.39934941001	-41.39934941017	-41.39934941038
**58.287143**	-41.39934940926	-41.39934940930	-41.39934940939	-41.39934940955	-41.39934940976
**58.442857**	-41.39934940866	-41.39934940869	-41.39934940878	-41.39934940894	-41.39934940915
**58.598571**	-41.39934940806	-41.39934940809	-41.39934940818	-41.39934940833	-41.39934940854
**58.754286**	-41.39934940746	-41.39934940749	-41.39934940759	-41.39934940774	-41.39934940794
**58.910000**	-41.39934940688	-41.39934940691	-41.39934940700	-41.39934940715	-41.39934940735
**59.065714**	-41.39934940630	-41.39934940633	-41.39934940642	-41.39934940657	-41.39934940677
**59.221429**	-41.39934940573	-41.39934940576	-41.39934940585	-41.39934940599	-41.39934940619
**59.377143**	-41.39934940517	-41.39934940520	-41.39934940529	-41.39934940543	-41.39934940562
**59.532857**	-41.39934940462	-41.39934940464	-41.39934940473	-41.39934940487	-41.39934940506
**59.688571**	-41.39934940407	-41.39934940409	-41.39934940418	-41.39934940432	-41.39934940451
**59.844286**	-41.39934940353	-41.39934940356	-41.39934940364	-41.39934940378	-41.39934940396
**60.000000**	-41.39934940213	-41.39934940025	-41.39934940230	-41.39934940204	-41.39934940265

PES aV5Z – CH^- ($X\ ^3\Sigma^-$) + He					
**R (Bohr)/θ(º)**	**50.00**	**60.00**	**70.00**	**80.00**	**90.00**
**5.500000**	-41.39796700171	-41.39813167560	-41.39808698560	-41.39791891850	-41.39772202918
**5.655714**	-41.39830784903	-41.39841387970	-41.39836144524	-41.39821659212	-41.39805540289
**5.811429**	-41.39857289415	-41.39863615390	-41.39858044108	-41.39845675359	-41.39832477882
**5.967143**	-41.39877907753	-41.39881152080	-41.39875554525	-41.39865108985	-41.39854317906
**6.122857**	-41.39893943071	-41.39895002206	-41.39889576852	-41.39880863965	-41.39872067389
**6.278571**	-41.39906406519	-41.39905944126	-41.39900815240	-41.39893639376	-41.39886517180
**6.434286**	-41.39916079427	-41.39914583748	-41.39909817091	-41.39903988811	-41.39898286812
**6.590000**	-41.39923566897	-41.39921396800	-41.39917019826	-41.39912351489	-41.39907863085
**6.745714**	-41.39929341805	-41.39926754736	-41.39922766709	-41.39919083953	-41.39915633302
**6.901429**	-41.39933769050	-41.39930948860	-41.39927339449	-41.39924477064	-41.39921907016
**7.057143**	-41.39937137782	-41.39934169412	-41.39930962565	-41.39928771280	-41.39926944906
**7.212857**	-41.39939674488	-41.39936714490	-41.39933818157	-41.39932167081	-41.39930956732
**7.368571**	-41.39941555052	-41.39938686230	-41.39936055084	-41.39934829320	-41.39934121260
**7.524286**	-41.39942918859	-41.39940099534	-41.39937793851	-41.39936896140	-41.39936590837
**7.680000**	-41.39943876865	-41.39941213846	-41.39939132230	-41.39938481877	-41.39938491215
**7.835714**	-41.39944517710	-41.39942049874	-41.39940128651	-41.39939681031	-41.39939925000
**7.991429**	-41.39944911657	-41.39942558534	-41.39940909250	-41.39940572446	-41.39940980613
**8.147143**	-41.39945113188	-41.39942954449	-41.39941455537	-41.39941218183	-41.39941732521
**8.302857**	-41.39945166145	-41.39943218765	-41.39941852810	-41.39941668448	-41.39942241971
**8.458571**	-41.39945107868	-41.39943268492	-41.39942106260	-41.39941960258	-41.39942560002
**8.614286**	-41.39944966415	-41.39943293452	-41.39942257895	-41.39942129431	-41.39942728483
**8.770000**	-41.39944761749	-41.39943245632	-41.39942324130	-41.39942204556	-41.39942780205
**8.925714**	-41.39944414473	-41.39943141487	-41.39942322554	-41.39942205742	-41.39942743107
**9.081429**	-41.39944227832	-41.39942993908	-41.39942267182	-41.39942116617	-41.39942638830
**9.237143**	-41.39943834001	-41.39942813761	-41.39942169524	-41.39942049520	-41.39942485819
**9.392857**	-41.39943578408	-41.39942609724	-41.39942038778	-41.39941891494	-41.39942298179
**9.548571**	-41.39943146407	-41.39942361968	-41.39941882214	-41.39941740977	-41.39942041692
**9.704286**	-41.39942825170	-41.39942127301	-41.39941679433	-41.39941545175	-41.39941834942
**9.860000**	-41.39942517035	-41.39941884761	-41.39941490004	-41.39941363969	-41.39941564506
**10.015714**	-41.39942258834	-41.39941636475	-41.39941289567	-41.39941173757	-41.39941336090
**10.171429**	-41.39941701040	-41.39941385268	-41.39941082705	-41.39940978508	-41.39941106982
**10.327143**	-41.39941400948	-41.39941135477	-41.39940873407	-41.39940780662	-41.39940880632
**10.482857**	-41.39941108056	-41.39940844675	-41.39940662153	-41.39940582489	-41.39940659455
**10.638571**	-41.39940822953	-41.39940603190	-41.39940451148	-41.39940385891	-41.39940442937
**10.794286**	-41.39940545636	-41.39940367193	-41.39940241707	-41.39940192080	-41.39940232779
**10.950000**	-41.39940277412	-41.39940137996	-41.39940038612	-41.39940003263	-41.39940046557
**11.105714**	-41.39940019469	-41.39939916264	-41.39939840343	-41.39939814227	-41.39939851977
**11.261429**	-41.39939771920	-41.39939701355	-41.39939646731	-41.39939629816	-41.39939663415
**11.417143**	-41.39939542676	-41.39939493454	-41.39939458456	-41.39939451001	-41.39939482051
**11.572857**	-41.39939324486	-41.39939292684	-41.39939276903	-41.39939279163	-41.39939308917
**11.728571**	-41.39939116635	-41.39939099262	-41.39939103078	-41.39939113931	-41.39939142739
**11.884286**	-41.39938918993	-41.39938918874	-41.39938936294	-41.39938955209	-41.39938983377
**12.040000**	-41.39938731388	-41.39938747314	-41.39938775819	-41.39938802055	-41.39938831796
**12.195714**	-41.39938553779	-41.39938583869	-41.39938620703	-41.39938637993	-41.39938686977
**12.351429**	-41.39938385581	-41.39938428549	-41.39938467174	-41.39938483547	-41.39938549548
**12.507143**	-41.39938226273	-41.39938276841	-41.39938312323	-41.39938334150	-41.39938388357
**12.662857**	-41.39938075596	-41.39938120085	-41.39938163084	-41.39938189837	-41.39938240352
**12.818571**	-41.39937933661	-41.39937969959	-41.39938019449	-41.39938050610	-41.39938097743
**12.974286**	-41.39937729674	-41.39937826331	-41.39937881377	-41.39937916445	-41.39937960478
**13.130000**	-41.39937602828	-41.39937689050	-41.39937748804	-41.39937787293	-41.39937828486
**13.285714**	-41.39937477160	-41.39937557952	-41.39937621642	-41.39937663091	-41.39937701679
**13.441429**	-41.39937364255	-41.39937432858	-41.39937499787	-41.39937543754	-41.39937579955
**13.597143**	-41.39937257719	-41.39937313583	-41.39937383121	-41.39937429187	-41.39937463199
**13.752857**	-41.39937157284	-41.39937199933	-41.39937271512	-41.39937319285	-41.39937351289
**13.908571**	-41.39937062705	-41.39937091712	-41.39937164822	-41.39937213933	-41.39937244095
**14.064286**	-41.39936973755	-41.39936988721	-41.39937062905	-41.39937113009	-41.39937141481
**14.220000**	-41.39936890015	-41.39936890762	-41.39936965609	-41.39937016386	-41.39937043309
**14.375714**	-41.39936811519	-41.39936797635	-41.39936872781	-41.39936923934	-41.39936949434
**14.531429**	-41.39936737974	-41.39936709144	-41.39936784264	-41.39936835520	-41.39936859715
**14.687143**	-41.39936670779	-41.39936625095	-41.39936699902	-41.39936751010	-41.39936774007
**14.842857**	-41.39936608321	-41.39936545298	-41.39936619538	-41.39936670268	-41.39936692167
**14.998571**	-41.39936551348	-41.39936469567	-41.39936543017	-41.39936593161	-41.39936614051
**15.154286**	-41.39936365077	-41.39936397719	-41.39936470185	-41.39936519554	-41.39936539518
**15.310000**	-41.39936304544	-41.39936329579	-41.39936400892	-41.39936449315	-41.39936468429

R (Bohr)/θ(º)	50.00	60.00	70.00	80.00	90.00
**15.465714**	-41.39936247044	-41.39936264975	-41.39936334990	-41.39936382314	-41.39936400648
**15.621429**	-41.39936192406	-41.39936215196	-41.39936272334	-41.39936318423	-41.39936336040
**15.777143**	-41.39936140478	-41.39936162508	-41.39936186156	-41.39936257517	-41.39936274476
**15.932857**	-41.39936091118	-41.39936112351	-41.39936135106	-41.39936199473	-41.39936215829
**16.088571**	-41.39936044190	-41.39936064593	-41.39936086458	-41.39936144171	-41.39936159973
**16.244286**	-41.39935999566	-41.39936019104	-41.39936040091	-41.39936091496	-41.39936106791
**16.400000**	-41.39935957106	-41.39935975753	-41.39935995881	-41.39936041336	-41.39936056164
**16.555714**	-41.39935916569	-41.39935934368	-41.39935953687	-41.39935993581	-41.39936007981
**16.711429**	-41.39935876922	-41.39935894545	-41.39935913283	-41.39935948126	-41.39935962133
**16.867143**	-41.39935840184	-41.39935855910	-41.39935873909	-41.39935893060	-41.39935911512
**17.022857**	-41.39935806104	-41.39935819715	-41.39935837434	-41.39935854394	-41.39935872468
**17.178571**	-41.39935772071	-41.39935785916	-41.39935801894	-41.39935819080	-41.39935834511
**17.334286**	-41.39935739040	-41.39935752966	-41.39935767877	-41.39935784088	-41.39935799208
**17.490000**	-41.39935707760	-41.39935721186	-41.39935735740	-41.39935750730	-41.39935765548
**17.645714**	-41.39935678443	-41.39935690984	-41.39935704827	-41.39935719261	-41.39935733659
**17.801429**	-41.39935650702	-41.39935662411	-41.39935675529	-41.39935689344	-41.39935703407
**17.957143**	-41.39935624253	-41.39935635247	-41.39935647723	-41.39935661011	-41.39935674661
**18.112857**	-41.39935598999	-41.39935609351	-41.39935621247	-41.39935634044	-41.39935647266
**18.268571**	-41.39935574880	-41.39935584639	-41.39935595984	-41.39935608302	-41.39935621088
**18.424286**	-41.39935551839	-41.39935561044	-41.39935571859	-41.39935583699	-41.39935596041
**18.580000**	-41.39935529818	-41.39935538508	-41.39935548811	-41.39935560176	-41.39935572070
**18.735714**	-41.39935508763	-41.39935516969	-41.39935526783	-41.39935537680	-41.39935549132
**18.891429**	-41.39935488619	-41.39935496373	-41.39935505721	-41.39935516164	-41.39935527182
**19.047143**	-41.39935469331	-41.39935476662	-41.39935485569	-41.39935495576	-41.39935506176
**19.202857**	-41.39935450847	-41.39935457783	-41.39935466274	-41.39935475866	-41.39935486066
**19.358571**	-41.39935433104	-41.39935439675	-41.39935447779	-41.39935456982	-41.39935466803
**19.514286**	-41.39935416036	-41.39935422272	-41.39935430023	-41.39935438866	-41.39935448335
**19.670000**	-41.39935399542	-41.39935405487	-41.39935412930	-41.39935421454	-41.39935430604
**19.825714**	-41.39935383491	-41.39935389187	-41.39935396390	-41.39935404658	-41.39935413541
**19.981429**	-41.39935368293	-41.39935374024	-41.39935380264	-41.39935388346	-41.39935397042
**20.137143**	-41.39935349240	-41.39935354427	-41.39935364978	-41.39935372584	-41.39935380960
**20.292857**	-41.39935336458	-41.39935339800	-41.39935346266	-41.39935349574	-41.39935366022
**20.448571**	-41.39935326581	-41.39935329886	-41.39935333677	-41.39935339015	-41.39935345494
**20.604286**	-41.39935314523	-41.39935318665	-41.39935323655	-41.39935328805	-41.39935333769
**20.760000**	-41.39935302081	-41.39935306379	-41.39935311770	-41.39935317841	-41.39935324054
**20.915714**	-41.39935289714	-41.39935294033	-41.39935299463	-41.39935305684	-41.39935312354
**21.071429**	-41.39935277476	-41.39935281829	-41.39935287238	-41.39935293426	-41.39935300107
**21.227143**	-41.39935265344	-41.39935269784	-41.39935275186	-41.39935281313	-41.39935287918
**21.382857**	-41.39935253353	-41.39935257916	-41.39935263321	-41.39935269386	-41.39935275897
**21.538571**	-41.39935241745	-41.39935246340	-41.39935251696	-41.39935257662	-41.39935264054
**21.694286**	-41.39935231209	-41.39935235435	-41.39935240512	-41.39935246243	-41.39935252425
**21.850000**	-41.39935222789	-41.39935225932	-41.39935230253	-41.39935235426	-41.39935241177
**22.005714**	-41.39935216840	-41.39935218554	-41.39935221646	-41.39935225809	-41.39935230744
**22.161429**	-41.39935212283	-41.39935213135	-41.39935215096	-41.39935218069	-41.39935221857
**22.317143**	-41.39935207673	-41.39935208555	-41.39935210078	-41.39935212269	-41.39935215055
**22.472857**	-41.39935202394	-41.39935203745	-41.39935205475	-41.39935207567	-41.39935209962
**22.628571**	-41.39935196513	-41.39935198346	-41.39935200495	-41.39935202894	-41.39935205453
**22.784286**	-41.39935190278	-41.39935192455	-41.39935194966	-41.39935197723	-41.39935200620
**22.940000**	-41.39935183902	-41.39935186284	-41.39935189028	-41.39935192041	-41.39935195210
**23.095714**	-41.39935177522	-41.39935180008	-41.39935182875	-41.39935186027	-41.39935189350
**23.251429**	-41.39935171221	-41.39935173745	-41.39935176657	-41.39935179859	-41.39935183239
**23.407143**	-41.39935165050	-41.39935167568	-41.39935170474	-41.39935173671	-41.39935177044
**23.562857**	-41.39935159030	-41.39935161518	-41.39935164387	-41.39935167543	-41.39935170869
**23.718571**	-41.39935153176	-41.39935155618	-41.39935158433	-41.39935161526	-41.39935164785
**23.874286**	-41.39935147494	-41.39935149882	-41.39935152631	-41.39935155651	-41.39935158827
**24.030000**	-41.39935141984	-41.39935144311	-41.39935146990	-41.39935149929	-41.39935153021
**24.185714**	-41.39935136643	-41.39935138908	-41.39935141513	-41.39935144370	-41.39935147373
**24.341429**	-41.39935131468	-41.39935133670	-41.39935136201	-41.39935138975	-41.39935141889
**24.497143**	-41.39935126456	-41.39935128595	-41.39935131052	-41.39935133744	-41.39935136569
**24.652857**	-41.39935121601	-41.39935123678	-41.39935126062	-41.39935128673	-41.39935131413
**24.808571**	-41.39935116897	-41.39935118914	-41.39935121227	-41.39935123759	-41.39935126415
**24.964286**	-41.39935112341	-41.39935114299	-41.39935116542	-41.39935118998	-41.39935121572
**25.120000**	-41.39935107926	-41.39935109827	-41.39935112003	-41.39935114384	-41.39935116880
**25.275714**	-41.39935103649	-41.39935105493	-41.39935107605	-41.39935109914	-41.39935112334
**25.431429**	-41.39935099502	-41.39935101293	-41.39935103342	-41.39935105581	-41.39935107928

R (Bohr)/θ(º)	50.00	60.00	70.00	80.00	90.00
**25.587143**	-41.39935095483	-41.39935097222	-41.39935099210	-41.39935101382	-41.39935103658
**25.742857**	-41.39935091587	-41.39935093275	-41.39935095204	-41.39935097312	-41.39935099519
**25.898571**	-41.39935087809	-41.39935089447	-41.39935091320	-41.39935093365	-41.39935095506
**26.054286**	-41.39935084144	-41.39935085736	-41.39935087554	-41.39935089538	-41.39935091616
**26.210000**	-41.39935080589	-41.39935082135	-41.39935083900	-41.39935085826	-41.39935087843
**26.365714**	-41.39935077140	-41.39935078642	-41.39935080356	-41.39935082226	-41.39935084183
**26.521429**	-41.39935073793	-41.39935075253	-41.39935076917	-41.39935078733	-41.39935080632
**26.677143**	-41.39935070545	-41.39935071963	-41.39935073580	-41.39935075343	-41.39935077187
**26.832857**	-41.39935067391	-41.39935068770	-41.39935070341	-41.39935072053	-41.39935073844
**26.988571**	-41.39935064330	-41.39935065669	-41.39935067196	-41.39935068859	-41.39935070599
**27.144286**	-41.39935061356	-41.39935062659	-41.39935064142	-41.39935065758	-41.39935067448
**27.300000**	-41.39935058469	-41.39935059735	-41.39935061177	-41.39935062747	-41.39935064389
**27.455714**	-41.39935055663	-41.39935056894	-41.39935058296	-41.39935059822	-41.39935061418
**27.611429**	-41.39935052938	-41.39935054135	-41.39935055498	-41.39935056981	-41.39935058532
**27.767143**	-41.39935050291	-41.39935051454	-41.39935052779	-41.39935054221	-41.39935055729
**27.922857**	-41.39935047720	-41.39935048849	-41.39935050137	-41.39935051539	-41.39935053005
**28.078571**	-41.39935045231	-41.39935046320	-41.39935047570	-41.39935048933	-41.39935050358
**28.234286**	-41.39935043037	-41.39935043883	-41.39935045078	-41.39935046400	-41.39935047786
**28.390000**	-41.39935040468	-41.39935041921	-41.39935042748	-41.39935043948	-41.39935045287
**28.545714**	-41.39935038113	-41.39935039134	-41.39935040308	-41.39935042490	-41.39935042893
**28.701429**	-41.39935035869	-41.39935036860	-41.39935037986	-41.39935039175	-41.39935040526
**28.857143**	-41.39935033690	-41.39935034655	-41.39935035750	-41.39935036940	-41.39935038185
**29.012857**	-41.39935031570	-41.39935032510	-41.39935033576	-41.39935034734	-41.39935035944
**29.168571**	-41.39935029507	-41.39935030423	-41.39935031461	-41.39935032589	-41.39935033766
**29.324286**	-41.39935027499	-41.39935028391	-41.39935029403	-41.39935030501	-41.39935031647
**29.480000**	-41.39935025545	-41.39935026415	-41.39935027399	-41.39935028469	-41.39935029585
**29.635714**	-41.39935023642	-41.39935024489	-41.39935025449	-41.39935026491	-41.39935027577
**29.791429**	-41.39935021790	-41.39935022615	-41.39935023550	-41.39935024565	-41.39935025623
**29.947143**	-41.39935019985	-41.39935020790	-41.39935021701	-41.39935022689	-41.39935023721
**30.102857**	-41.39935018228	-41.39935019012	-41.39935019900	-41.39935020863	-41.39935021868
**30.258571**	-41.39935016516	-41.39935017280	-41.39935018146	-41.39935019085	-41.39935020064
**30.414286**	-41.39935014847	-41.39935015593	-41.39935016437	-41.39935017352	-41.39935018306
**30.570000**	-41.39935013222	-41.39935013949	-41.39935014772	-41.39935015663	-41.39935016594
**30.725714**	-41.39935011638	-41.39935012347	-41.39935013149	-41.39935014018	-41.39935014925
**30.881429**	-41.39935010094	-41.39935010786	-41.39935011568	-41.39935012415	-41.39935013299
**31.037143**	-41.39935008594	-41.39935009264	-41.39935010026	-41.39935010853	-41.39935011715
**31.192857**	-41.39935007127	-41.39935008727	-41.39935008525	-41.39935009330	-41.39935010170
**31.348571**	-41.39935005690	-41.39935006333	-41.39935007060	-41.39935007857	-41.39935008665
**31.504286**	-41.39935004294	-41.39935004921	-41.39935005630	-41.39935006397	-41.39935007198
**31.660000**	-41.39935002933	-41.39935003545	-41.39935004236	-41.39935004985	-41.39935005765
**31.815714**	-41.39935001605	-41.39935002203	-41.39935002877	-41.39935003608	-41.39935004369
**31.971429**	-41.39935000310	-41.39935000893	-41.39935001552	-41.39935002265	-41.39935003008
**32.127143**	-41.39934999046	-41.39934999616	-41.39935000258	-41.39935000954	-41.39935001679
**32.282857**	-41.39934997813	-41.39934998369	-41.39934998997	-41.39934999676	-41.39935000383
**32.438571**	-41.39934996609	-41.39934997152	-41.39934997765	-41.39934998428	-41.39934999119
**32.594286**	-41.39934995434	-41.39934995965	-41.39934996563	-41.39934997211	-41.39934997885
**32.750000**	-41.39934994287	-41.39934994806	-41.39934995390	-41.39934996022	-41.39934996681
**32.905714**	-41.39934993168	-41.39934993674	-41.39934994245	-41.39934994862	-41.39934995505
**33.061429**	-41.39934992075	-41.39934992569	-41.39934993127	-41.39934993730	-41.39934994358
**33.217143**	-41.39934991007	-41.39934991490	-41.39934992035	-41.39934992624	-41.39934993238
**33.372857**	-41.39934989965	-41.39934990437	-41.39934990969	-41.39934991544	-41.39934992144
**33.528571**	-41.39934988946	-41.39934989408	-41.39934989928	-41.39934990490	-41.39934991075
**33.684286**	-41.39934987951	-41.39934988402	-41.39934988911	-41.39934989460	-41.39934990032
**33.840000**	-41.39934986979	-41.39934987421	-41.39934987917	-41.39934988454	-41.39934989013
**33.995714**	-41.39934986030	-41.39934986461	-41.39934986947	-41.39934987471	-41.39934988018
**34.151429**	-41.39934985102	-41.39934985523	-41.39934985998	-41.39934986511	-41.39934987045
**34.307143**	-41.39934984195	-41.39934984607	-41.39934985072	-41.39934985573	-41.39934986095
**34.462857**	-41.39934983309	-41.39934983712	-41.39934984166	-41.39934984656	-41.39934985167
**34.618571**	-41.39934982442	-41.39934982837	-41.39934983281	-41.39934983760	-41.39934984259
**34.774286**	-41.39934981596	-41.39934981982	-41.39934982416	-41.39934982884	-41.39934983372
**34.930000**	-41.39934980768	-41.39934981145	-41.39934981570	-41.39934982028	-41.39934982505
**35.085714**	-41.39934979958	-41.39934980327	-41.39934980742	-41.39934981191	-41.39934981657
**35.241429**	-41.39934979166	-41.39934979528	-41.39934979934	-41.39934980372	-41.39934980829
**35.397143**	-41.39934978392	-41.39934978746	-41.39934979143	-41.39934979572	-41.39934980018
**35.552857**	-41.39934977635	-41.39934977981	-41.39934978370	-41.39934978789	-41.39934979226

R (Bohr)/θ(º)	50.00	60.00	70.00	80.00	90.00
**35.708571**	-41.39934976894	-41.39934977233	-41.39934977613	-41.39934978024	-41.39934978451
**35.864286**	-41.39934976169	-41.39934976501	-41.39934976873	-41.39934977275	-41.39934977693
**36.020000**	-41.39934975460	-41.39934975785	-41.39934976149	-41.39934976543	-41.39934976952
**36.175714**	-41.39934974767	-41.39934975084	-41.39934975441	-41.39934975826	-41.39934976227
**36.331429**	-41.39934974088	-41.39934974399	-41.39934974748	-41.39934975125	-41.39934975517
**36.487143**	-41.39934973423	-41.39934973728	-41.39934974070	-41.39934974439	-41.39934974823
**36.642857**	-41.39934972773	-41.39934973072	-41.39934973406	-41.39934973768	-41.39934974143
**36.798571**	-41.39934972137	-41.39934972429	-41.39934972757	-41.39934973110	-41.39934973478
**36.954286**	-41.39934971514	-41.39934971800	-41.39934972121	-41.39934972467	-41.39934972827
**37.110000**	-41.39934970904	-41.39934971184	-41.39934971498	-41.39934971837	-41.39934972190
**37.265714**	-41.39934970307	-41.39934970581	-41.39934970889	-41.39934971221	-41.39934971566
**37.421429**	-41.39934969722	-41.39934969991	-41.39934970292	-41.39934970618	-41.39934970956
**37.577143**	-41.39934969149	-41.39934969413	-41.39934969708	-41.39934970027	-41.39934970358
**37.732857**	-41.39934968588	-41.39934968846	-41.39934969136	-41.39934969448	-41.39934969773
**37.888571**	-41.39934968039	-41.39934968292	-41.39934968576	-41.39934968882	-41.39934969199
**38.044286**	-41.39934967501	-41.39934967749	-41.39934968027	-41.39934968327	-41.39934968638
**38.200000**	-41.39934966974	-41.39934967217	-41.39934967490	-41.39934967783	-41.39934968088
**38.355714**	-41.39934966458	-41.39934966696	-41.39934966963	-41.39934967251	-41.39934967549
**38.511429**	-41.39934965952	-41.39934966185	-41.39934966447	-41.39934966729	-41.39934967022
**38.667143**	-41.39934965456	-41.39934965685	-41.39934965942	-41.39934966218	-41.39934966505
**38.822857**	-41.39934964970	-41.39934965194	-41.39934965446	-41.39934965717	-41.39934965998
**38.978571**	-41.39934964494	-41.39934964714	-41.39934964961	-41.39934965226	-41.39934965502
**39.134286**	-41.39934964027	-41.39934964243	-41.39934964485	-41.39934964745	-41.39934965016
**39.290000**	-41.39934963570	-41.39934963782	-41.39934964019	-41.39934964274	-41.39934964539
**39.445714**	-41.39934963122	-41.39934963329	-41.39934963562	-41.39934963812	-41.39934964072
**39.601429**	-41.39934962682	-41.39934962886	-41.39934963114	-41.39934963359	-41.39934963614
**39.757143**	-41.39934962252	-41.39934962451	-41.39934962675	-41.39934962916	-41.39934963165
**39.912857**	-41.39934961829	-41.39934962025	-41.39934962244	-41.39934962480	-41.39934962725
**40.068571**	-41.39934961415	-41.39934961607	-41.39934961822	-41.39934962054	-41.39934962294
**40.224286**	-41.39934961008	-41.39934961197	-41.39934961408	-41.39934961635	-41.39934961871
**40.380000**	-41.39934960610	-41.39934960795	-41.39934961002	-41.39934961225	-41.39934961456
**40.535714**	-41.39934960219	-41.39934960401	-41.39934960604	-41.39934960822	-41.39934961050
**40.691429**	-41.39934959836	-41.39934960014	-41.39934960213	-41.39934960428	-41.39934960651
**40.847143**	-41.39934959460	-41.39934959635	-41.39934959830	-41.39934960041	-41.39934960259
**41.002857**	-41.39934959091	-41.39934959263	-41.39934959455	-41.39934959661	-41.39934959875
**41.158571**	-41.39934958729	-41.39934958898	-41.39934959086	-41.39934959289	-41.39934959499
**41.314286**	-41.39934958374	-41.39934958539	-41.39934958724	-41.39934958923	-41.39934959130
**41.470000**	-41.39934958026	-41.39934958188	-41.39934958370	-41.39934958565	-41.39934958767
**41.625714**	-41.39934957684	-41.39934957843	-41.39934958021	-41.39934958213	-41.39934958412
**41.781429**	-41.39934957348	-41.39934957505	-41.39934957680	-41.39934957868	-41.39934958063
**41.937143**	-41.39934957019	-41.39934957173	-41.39934957345	-41.39934957529	-41.39934957721
**42.092857**	-41.39934956696	-41.39934956847	-41.39934957015	-41.39934957197	-41.39934957385
**42.248571**	-41.39934956379	-41.39934956527	-41.39934956692	-41.39934956871	-41.39934957055
**42.404286**	-41.39934956067	-41.39934956213	-41.39934956376	-41.39934956550	-41.39934956732
**42.560000**	-41.39934955762	-41.39934955904	-41.39934956064	-41.39934956236	-41.39934956414
**42.715714**	-41.39934955462	-41.39934955602	-41.39934955759	-41.39934955927	-41.39934956102
**42.871429**	-41.39934955167	-41.39934955305	-41.39934955459	-41.39934955624	-41.39934955796
**43.027143**	-41.39934954877	-41.39934955013	-41.39934955164	-41.39934955327	-41.39934955496
**43.182857**	-41.39934954594	-41.39934954726	-41.39934954875	-41.39934955035	-41.39934955200
**43.338571**	-41.39934954315	-41.39934954445	-41.39934954591	-41.39934954748	-41.39934954911
**43.494286**	-41.39934954040	-41.39934954169	-41.39934954312	-41.39934954466	-41.39934954626
**43.650000**	-41.39934953771	-41.39934953897	-41.39934954039	-41.39934954190	-41.39934954347
**43.805714**	-41.39934953507	-41.39934953631	-41.39934953769	-41.39934953918	-41.39934954073
**43.961429**	-41.39934953247	-41.39934953369	-41.39934953505	-41.39934953652	-41.39934953803
**44.117143**	-41.39934952992	-41.39934953112	-41.39934953246	-41.39934953389	-41.39934953538
**44.272857**	-41.39934952742	-41.39934952859	-41.39934952991	-41.39934953132	-41.39934953278
**44.428571**	-41.39934952496	-41.39934952611	-41.39934952740	-41.39934952879	-41.39934953023
**44.584286**	-41.39934952254	-41.39934952367	-41.39934952494	-41.39934952631	-41.39934952772
**44.740000**	-41.39934952016	-41.39934952128	-41.39934952252	-41.39934952386	-41.39934952525
**44.895714**	-41.39934951782	-41.39934951892	-41.39934952015	-41.39934952147	-41.39934952283
**45.051429**	-41.39934951553	-41.39934951661	-41.39934951781	-41.39934951911	-41.39934952045
**45.207143**	-41.39934951327	-41.39934951434	-41.39934951552	-41.39934951679	-41.39934951811
**45.362857**	-41.39934951106	-41.39934951210	-41.39934951327	-41.39934951452	-41.39934951582
**45.518571**	-41.39934950888	-41.39934950990	-41.39934951105	-41.39934951228	-41.39934951356
**45.674286**	-41.39934950674	-41.39934950774	-41.39934950887	-41.39934951008	-41.39934951134

R (Bohr)/θ(º)	50.00	60.00	70.00	80.00	90.00
**45.830000**	-41.39934950463	-41.39934950562	-41.39934950673	-41.39934950792	-41.39934950915
**45.985714**	-41.39934950256	-41.39934950354	-41.39934950463	-41.39934950580	-41.39934950701
**46.141429**	-41.39934950053	-41.39934950149	-41.39934950256	-41.39934950371	-41.39934950490
**46.297143**	-41.39934949853	-41.39934949947	-41.39934950052	-41.39934950165	-41.39934950283
**46.452857**	-41.39934949656	-41.39934949749	-41.39934949852	-41.39934949964	-41.39934950079
**46.608571**	-41.39934949462	-41.39934949554	-41.39934949656	-41.39934949765	-41.39934949879
**46.764286**	-41.39934949272	-41.39934949362	-41.39934949462	-41.39934949570	-41.39934949682
**46.920000**	-41.39934949085	-41.39934949174	-41.39934949272	-41.39934949378	-41.39934949488
**47.075714**	-41.39934948901	-41.39934948988	-41.39934949085	-41.39934949190	-41.39934949298
**47.231429**	-41.39934948720	-41.39934948806	-41.39934948902	-41.39934949004	-41.39934949110
**47.387143**	-41.39934948542	-41.39934948627	-41.39934948721	-41.39934948821	-41.39934948926
**47.542857**	-41.39934948367	-41.39934948450	-41.39934948543	-41.39934948642	-41.39934948745
**47.698571**	-41.39934948195	-41.39934948277	-41.39934948368	-41.39934948465	-41.39934948567
**47.854286**	-41.39934948026	-41.39934948106	-41.39934948196	-41.39934948292	-41.39934948392
**48.010000**	-41.39934947859	-41.39934947938	-41.39934948026	-41.39934948121	-41.39934948219
**48.165714**	-41.39934947695	-41.39934947773	-41.39934947860	-41.39934947953	-41.39934948050
**48.321429**	-41.39934947534	-41.39934947611	-41.39934947696	-41.39934947788	-41.39934947883
**48.477143**	-41.39934947375	-41.39934947451	-41.39934947535	-41.39934947625	-41.39934947719
**48.632857**	-41.39934947219	-41.39934947293	-41.39934947376	-41.39934947465	-41.39934947557
**48.788571**	-41.39934947066	-41.39934947138	-41.39934947220	-41.39934947307	-41.39934947398
**48.944286**	-41.39934946914	-41.39934946986	-41.39934947066	-41.39934947152	-41.39934947242
**49.100000**	-41.39934946766	-41.39934946836	-41.39934946915	-41.39934947000	-41.39934947088
**49.255714**	-41.39934946619	-41.39934946689	-41.39934946767	-41.39934946850	-41.39934946937
**49.411429**	-41.39934946475	-41.39934946544	-41.39934946620	-41.39934946702	-41.39934946788
**49.567143**	-41.39934946333	-41.39934946401	-41.39934946476	-41.39934946557	-41.39934946641
**49.722857**	-41.39934946194	-41.39934946260	-41.39934946334	-41.39934946414	-41.39934946497
**49.878571**	-41.39934946056	-41.39934946122	-41.39934946195	-41.39934946273	-41.39934946354
**50.034286**	-41.39934945921	-41.39934945985	-41.39934946057	-41.39934946134	-41.39934946215
**50.190000**	-41.39934945788	-41.39934945851	-41.39934945922	-41.39934945998	-41.39934946077
**50.345714**	-41.39934945656	-41.39934945719	-41.39934945789	-41.39934945864	-41.39934945941
**50.501429**	-41.39934945527	-41.39934945589	-41.39934945658	-41.39934945731	-41.39934945808
**50.657143**	-41.39934945400	-41.39934945461	-41.39934945528	-41.39934945601	-41.39934945677
**50.812857**	-41.39934945275	-41.39934945335	-41.39934945402	-41.39934945473	-41.39934945547
**50.968571**	-41.39934945152	-41.39934945210	-41.39934945276	-41.39934945347	-41.39934945420
**51.124286**	-41.39934945030	-41.39934945088	-41.39934945153	-41.39934945222	-41.39934945295
**51.280000**	-41.39934944911	-41.39934944968	-41.39934945032	-41.39934945100	-41.39934945171
**51.435714**	-41.39934944793	-41.39934944849	-41.39934944912	-41.39934944979	-41.39934945050
**51.591429**	-41.39934944677	-41.39934944733	-41.39934944794	-41.39934944861	-41.39934944930
**51.747143**	-41.39934944563	-41.39934944618	-41.39934944678	-41.39934944744	-41.39934944812
**51.902857**	-41.39934944450	-41.39934944504	-41.39934944565	-41.39934944629	-41.39934944696
**52.058571**	-41.39934944339	-41.39934944393	-41.39934944452	-41.39934944515	-41.39934944581
**52.214286**	-41.39934944230	-41.39934944283	-41.39934944341	-41.39934944404	-41.39934944469
**52.370000**	-41.39934944123	-41.39934944174	-41.39934944232	-41.39934944294	-41.39934944358
**52.525714**	-41.39934944017	-41.39934944068	-41.39934944125	-41.39934944185	-41.39934944248
**52.681429**	-41.39934943912	-41.39934943963	-41.39934944019	-41.39934944078	-41.39934944141
**52.837143**	-41.39934943810	-41.39934943859	-41.39934943914	-41.39934943973	-41.39934944035
**52.992857**	-41.39934943708	-41.39934943757	-41.39934943811	-41.39934943870	-41.39934943930
**53.148571**	-41.39934943609	-41.39934943657	-41.39934943710	-41.39934943768	-41.39934943827
**53.304286**	-41.39934943510	-41.39934943558	-41.39934943610	-41.39934943667	-41.39934943726
**53.460000**	-41.39934943413	-41.39934943460	-41.39934943512	-41.39934943568	-41.39934943626
**53.615714**	-41.39934943318	-41.39934943364	-41.39934943415	-41.39934943470	-41.39934943527
**53.771429**	-41.39934943224	-41.39934943269	-41.39934943320	-41.39934943374	-41.39934943430
**53.927143**	-41.39934943131	-41.39934943176	-41.39934943225	-41.39934943278	-41.39934943334
**54.082857**	-41.39934943039	-41.39934943084	-41.39934943133	-41.39934943185	-41.39934943240
**54.238571**	-41.39934942949	-41.39934942993	-41.39934943041	-41.39934943093	-41.39934943147
**54.394286**	-41.39934942861	-41.39934942903	-41.39934942951	-41.39934943002	-41.39934943056
**54.550000**	-41.39934942773	-41.39934942815	-41.39934942862	-41.39934942913	-41.39934942965
**54.705714**	-41.39934942687	-41.39934942728	-41.39934942775	-41.39934942825	-41.39934942876
**54.861429**	-41.39934942602	-41.39934942643	-41.39934942689	-41.39934942738	-41.39934942789
**55.017143**	-41.39934942518	-41.39934942559	-41.39934942604	-41.39934942652	-41.39934942702
**55.172857**	-41.39934942435	-41.39934942475	-41.39934942520	-41.39934942567	-41.39934942617
**55.328571**	-41.39934942354	-41.39934942393	-41.39934942437	-41.39934942484	-41.39934942533
**55.484286**	-41.39934942273	-41.39934942312	-41.39934942355	-41.39934942402	-41.39934942450
**55.640000**	-41.39934942194	-41.39934942232	-41.39934942275	-41.39934942321	-41.39934942369
**55.795714**	-41.39934942116	-41.39934942154	-41.39934942196	-41.39934942241	-41.39934942288

R (Bohr)/θ(º)	50.00	60.00	70.00	80.00	90.00
**55.951429**	-41.39934942039	-41.39934942076	-41.39934942118	-41.39934942162	-41.39934942209
**56.107143**	-41.39934941963	-41.39934942000	-41.39934942041	-41.39934942085	-41.39934942131
**56.262857**	-41.39934941888	-41.39934941924	-41.39934941965	-41.39934942008	-41.39934942053
**56.418571**	-41.39934941814	-41.39934941850	-41.39934941890	-41.39934941933	-41.39934941977
**56.574286**	-41.39934941741	-41.39934941777	-41.39934941816	-41.39934941858	-41.39934941902
**56.730000**	-41.39934941669	-41.39934941704	-41.39934941743	-41.39934941785	-41.39934941828
**56.885714**	-41.39934941598	-41.39934941633	-41.39934941671	-41.39934941712	-41.39934941755
**57.041429**	-41.39934941529	-41.39934941563	-41.39934941601	-41.39934941641	-41.39934941683
**57.197143**	-41.39934941460	-41.39934941493	-41.39934941530	-41.39934941571	-41.39934941612
**57.352857**	-41.39934941392	-41.39934941425	-41.39934941462	-41.39934941501	-41.39934941542
**57.508571**	-41.39934941325	-41.39934941357	-41.39934941394	-41.39934941433	-41.39934941473
**57.664286**	-41.39934941258	-41.39934941291	-41.39934941327	-41.39934941365	-41.39934941405
**57.820000**	-41.39934941193	-41.39934941225	-41.39934941260	-41.39934941298	-41.39934941338
**57.975714**	-41.39934941129	-41.39934941160	-41.39934941195	-41.39934941233	-41.39934941272
**58.131429**	-41.39934941065	-41.39934941096	-41.39934941131	-41.39934941168	-41.39934941206
**58.287143**	-41.39934941002	-41.39934941033	-41.39934941067	-41.39934941104	-41.39934941142
**58.442857**	-41.39934940941	-41.39934940971	-41.39934941005	-41.39934941041	-41.39934941078
**58.598571**	-41.39934940880	-41.39934940910	-41.39934940943	-41.39934940978	-41.39934941015
**58.754286**	-41.39934940819	-41.39934940849	-41.39934940882	-41.39934940917	-41.39934940953
**58.910000**	-41.39934940760	-41.39934940789	-41.39934940821	-41.39934940856	-41.39934940892
**59.065714**	-41.39934940702	-41.39934940730	-41.39934940762	-41.39934940796	-41.39934940832
**59.221429**	-41.39934940643	-41.39934940672	-41.39934940704	-41.39934940737	-41.39934940773
**59.377143**	-41.39934940587	-41.39934940615	-41.39934940646	-41.39934940679	-41.39934940714
**59.532857**	-41.39934940530	-41.39934940558	-41.39934940589	-41.39934940621	-41.39934940656
**59.688571**	-41.39934940475	-41.39934940502	-41.39934940532	-41.39934940565	-41.39934940598
**59.844286**	-41.39934940420	-41.39934940447	-41.39934940476	-41.39934940508	-41.39934940542
**60.000000**	-41.39934940363	-41.39934940392	-41.39934940421	-41.39934939001	-41.39934939089

PES aV5Z – CH⁻ (X ³Σ⁻) + He					
**R (Bohr)/θ(º)**	**100.00**	**110.00**	**120.00**	**130.00**	**140.00**
**5.500000**	-41.39755126965	-41.39743471850	-41.39737613893	-41.39736165418	-41.39737140910
**5.655714**	-41.39792292782	-41.39784012846	-41.39780742922	-41.39781211764	-41.39783556423
**5.811429**	-41.39822242186	-41.39816523347	-41.39815161045	-41.39816927361	-41.39820185177
**5.967143**	-41.39846441658	-41.39842648846	-41.39842645272	-41.39845285393	-41.39849091285
**6.122857**	-41.39866043420	-41.39863686111	-41.39864625008	-41.39867805758	-41.39871902106
**6.278571**	-41.39881957010	-41.39880653073	-41.39882223037	-41.39885699210	-41.39889905846
**6.434286**	-41.39894884667	-41.39894358649	-41.39896327261	-41.39899922220	-41.39904101977
**6.590000**	-41.39905389598	-41.39905429007	-41.39907638551	-41.39911228772	-41.39915286156
**6.745714**	-41.39913912633	-41.39914369819	-41.39916695871	-41.39920196072	-41.39924065761
**6.901429**	-41.39920804623	-41.39921567439	-41.39923932255	-41.39927289807	-41.39930929886
**7.057143**	-41.39926350931	-41.39927334995	-41.39929685956	-41.39932867254	-41.39936254689
**7.212857**	-41.39930780453	-41.39931925932	-41.39934227563	-41.39937216236	-41.39940343453
**7.368571**	-41.39934283804	-41.39935544945	-41.39937775160	-41.39940565351	-41.39943436118
**7.524286**	-41.39937020994	-41.39938361512	-41.39940506703	-41.39943101859	-41.39945726037
**7.680000**	-41.39939126880	-41.39940517137	-41.39942569444	-41.39944975790	-41.39947369422
**7.835714**	-41.39940715342	-41.39942130742	-41.39944087114	-41.39946313284	-41.39948493194
**7.991429**	-41.39941881439	-41.39943302275	-41.39945160348	-41.39947217846	-41.39949202601
**8.147143**	-41.39942705855	-41.39944114985	-41.39945875266	-41.39947775850	-41.39949581592
**8.302857**	-41.39943255839	-41.39944639026	-41.39946301544	-41.39948055734	-41.39949699464
**8.458571**	-41.39943588226	-41.39944931869	-41.39946495353	-41.39948114028	-41.39949612404
**8.614286**	-41.39943741717	-41.39945038637	-41.39946508934	-41.39948001496	-41.39949364975
**8.770000**	-41.39943761305	-41.39944999504	-41.39946376792	-41.39947753674	-41.39948997863
**8.925714**	-41.39943677077	-41.39944848436	-41.39946133445	-41.39947402927	-41.39948539430
**9.081429**	-41.39943514591	-41.39944612915	-41.39945806956	-41.39946974989	-41.39948010583
**9.237143**	-41.39943295385	-41.39944315973	-41.39945420408	-41.39946492324	-41.39947435837
**9.392857**	-41.39943036586	-41.39943976644	-41.39944992753	-41.39945946073	-41.39946802980
**9.548571**	-41.39942751525	-41.39943610725	-41.39944518968	-41.39945410078	-41.39945932825
**9.704286**	-41.39942363097	-41.39943211961	-41.39944055917	-41.39944674442	-41.39945177336
**9.860000**	-41.39942124396	-41.39942744003	-41.39943480792	-41.39944070987	-41.39944428522
**10.015714**	-41.39941761362	-41.39942445518	-41.39943130659	-41.39943500946	-41.39943801095
**10.171429**	-41.39941471274	-41.39941991583	-41.39942574515	-41.39942969449	-41.39943299008
**10.327143**	-41.39941190427	-41.39941641657	-41.39942168983	-41.39942408824	-41.39942825345
**10.482857**	-41.39940920600	-41.39941306119	-41.39941843960	-41.39942006984	-41.39942380250
**10.638571**	-41.39940656762	-41.39940997694	-41.39941265047	-41.39941628461	-41.39941962831
**10.794286**	-41.39940411404	-41.39940642569	-41.39940950122	-41.39941272635	-41.39941572098
**10.950000**	-41.39940172849	-41.39940385321	-41.39940653164	-41.39940938384	-41.39941206891
**11.105714**	-41.39939958831	-41.39940141396	-41.39940373926	-41.39940625416	-41.39940866705
**11.261429**	-41.39939756151	-41.39939911827	-41.39940112453	-41.39940333212	-41.39940550777
**11.417143**	-41.39939564532	-41.39939696404	-41.39939869491	-41.39940061371	-41.39940257174
**11.572857**	-41.39939379167	-41.39939491956	-41.39939641794	-41.39939810849	-41.39939984378
**11.728571**	-41.39939201930	-41.39939297566	-41.39939427698	-41.39939577884	-41.39939733687
**11.884286**	-41.39939034234	-41.39939115308	-41.39939227075	-41.39939357735	-41.39939495899
**12.040000**	-41.39938875438	-41.39938943563	-41.39939039149	-41.39939153130	-41.39939275534
**12.195714**	-41.39938725018	-41.39938782366	-41.39938863762	-41.39938963603	-41.39939071749
**12.351429**	-41.39938566777	-41.39938631457	-41.39938700325	-41.39938787221	-41.39938883325
**12.507143**	-41.39938409862	-41.39938490450	-41.39938548294	-41.39938622355	-41.39938665997
**12.662857**	-41.39938258826	-41.39938358808	-41.39938406836	-41.39938445429	-41.39938491732
**12.818571**	-41.39938113583	-41.39938196427	-41.39938239867	-41.39938284827	-41.39938329057
**12.974286**	-41.39937974031	-41.39938047667	-41.39938093105	-41.39938131335	-41.39938182161
**13.130000**	-41.39937840053	-41.39937905270	-41.39937952559	-41.39937984710	-41.39938016287
**13.285714**	-41.39937711519	-41.39937769050	-41.39937818034	-41.39937844704	-41.39937859111
**13.441429**	-41.39937588294	-41.39937638819	-41.39937689332	-41.39937711076	-41.39937710245
**13.597143**	-41.39937470232	-41.39937514384	-41.39937566253	-41.39937583583	-41.39937569308
**13.752857**	-41.39937357184	-41.39937395547	-41.39937448601	-41.39937461988	-41.39937435930
**13.908571**	-41.39937248995	-41.39937282112	-41.39937336179	-41.39937346057	-41.39937309750
**14.064286**	-41.39937145510	-41.39937173883	-41.39937228793	-41.39937235562	-41.39937190422
**14.220000**	-41.39937046572	-41.39937070665	-41.39937126251	-41.39937130279	-41.39937077607
**14.375714**	-41.39936952023	-41.39936972264	-41.39937028367	-41.39937029991	-41.39936970983
**14.531429**	-41.39936861706	-41.39936878490	-41.39936934956	-41.39936934485	-41.39936870237
**14.687143**	-41.39936775465	-41.39936789158	-41.39936845838	-41.39936843556	-41.39936775068
**14.842857**	-41.39936693146	-41.39936704083	-41.39936760839	-41.39936757005	-41.39936727212
**14.998571**	-41.39936614598	-41.39936623088	-41.39936679787	-41.39936674639	-41.39936649889
**15.154286**	-41.39936539672	-41.39936545999	-41.39936602519	-41.39936596271	-41.39936576579
**15.310000**	-41.39936468222	-41.39936472647	-41.39936528872	-41.39936489521	-41.39936505996

R (Bohr)/θ(º)	100.00	110.00	120.00	130.00	140.00
**15.465714**	-41.39936400108	-41.39936402869	-41.39936458692	-41.39936425385	-41.39936441213
**15.621429**	-41.39936335189	-41.39936336506	-41.39936391829	-41.39936364018	-41.39936378724
**15.777143**	-41.39936273333	-41.39936273406	-41.39936328138	-41.39936305620	-41.39936319302
**15.932857**	-41.39936214407	-41.39936218624	-41.39936267478	-41.39936250052	-41.39936262816
**16.088571**	-41.39936148489	-41.39936167039	-41.39936209714	-41.39936197162	-41.39936209103
**16.244286**	-41.39936099732	-41.39936117854	-41.39936154718	-41.39936146830	-41.39936158025
**16.400000**	-41.39936053175	-41.39936070985	-41.39936102363	-41.39936098928	-41.39936109447
**16.555714**	-41.39936008720	-41.39936026385	-41.39936041183	-41.39936053375	-41.39936063285
**16.711429**	-41.39935966327	-41.39935984047	-41.39935998348	-41.39936010092	-41.39936019484
**16.867143**	-41.39935926456	-41.39935943900	-41.39935957589	-41.39935968966	-41.39935978003
**17.022857**	-41.39935889877	-41.39935905374	-41.39935918592	-41.39935929752	-41.39935938689
**17.178571**	-41.39935849520	-41.39935867322	-41.39935880843	-41.39935892019	-41.39935901075
**17.334286**	-41.39935814589	-41.39935830323	-41.39935843391	-41.39935855189	-41.39935864525
**17.490000**	-41.39935780751	-41.39935796060	-41.39935809064	-41.39935819539	-41.39935828073
**17.645714**	-41.39935748284	-41.39935761910	-41.39935774594	-41.39935785207	-41.39935793844
**17.801429**	-41.39935717658	-41.39935731425	-41.39935743690	-41.39935754113	-41.39935762704
**17.957143**	-41.39935688391	-41.39935701567	-41.39935713412	-41.39935723548	-41.39935731881
**18.112857**	-41.39935660509	-41.39935673175	-41.39935684629	-41.39935694504	-41.39935702682
**18.268571**	-41.39935633874	-41.39935646096	-41.39935657209	-41.39935666869	-41.39935674914
**18.424286**	-41.39935608386	-41.39935620198	-41.39935630991	-41.39935640436	-41.39935648351
**18.580000**	-41.39935583985	-41.39935595406	-41.39935605887	-41.39935615110	-41.39935622878
**18.735714**	-41.39935560627	-41.39935571673	-41.39935581851	-41.39935590854	-41.39935598470
**18.891429**	-41.39935538271	-41.39935548957	-41.39935558843	-41.39935567629	-41.39935575099
**19.047143**	-41.39935516874	-41.39935527216	-41.39935536821	-41.39935545397	-41.39935552725
**19.202857**	-41.39935496391	-41.39935506403	-41.39935515740	-41.39935524113	-41.39935531302
**19.358571**	-41.39935476774	-41.39935486475	-41.39935495554	-41.39935503732	-41.39935510786
**19.514286**	-41.39935457975	-41.39935467381	-41.39935476216	-41.39935484208	-41.39935491131
**19.670000**	-41.39935439941	-41.39935449075	-41.39935457682	-41.39935465496	-41.39935472293
**19.825714**	-41.39935422613	-41.39935431503	-41.39935439901	-41.39935447551	-41.39935454230
**19.981429**	-41.39935405913	-41.39935414602	-41.39935422821	-41.39935430325	-41.39935436898
**20.137143**	-41.39935389722	-41.39935398287	-41.39935406376	-41.39935413765	-41.39935420251
**20.292857**	-41.39935373927	-41.39935382420	-41.39935390467	-41.39935397802	-41.39935404235
**20.448571**	-41.39935356745	-41.39935368393	-41.39935374951	-41.39935382325	-41.39935388775
**20.604286**	-41.39935340121	-41.39935348335	-41.39935359172	-41.39935368039	-41.39935373769
**20.760000**	-41.39935329716	-41.39935334655	-41.39935340874	-41.39935348951	-41.39935356815
**20.915714**	-41.39935319099	-41.39935325491	-41.39935330999	-41.39935335239	-41.39935339456
**21.071429**	-41.39935307001	-41.39935313849	-41.39935320418	-41.39935326493	-41.39935331874
**21.227143**	-41.39935294756	-41.39935301605	-41.39935308272	-41.39935314583	-41.39935320364
**21.382857**	-41.39935282639	-41.39935289413	-41.39935296041	-41.39935302359	-41.39935308185
**21.538571**	-41.39935270679	-41.39935277356	-41.39935283927	-41.39935290240	-41.39935296120
**21.694286**	-41.39935258871	-41.39935265410	-41.39935271901	-41.39935278212	-41.39935284183
**21.850000**	-41.39935247283	-41.39935253567	-41.39935259896	-41.39935266164	-41.39935272239
**22.005714**	-41.39935236195	-41.39935241960	-41.39935247910	-41.39935253971	-41.39935260071
**22.161429**	-41.39935226252	-41.39935231079	-41.39935236227	-41.39935241664	-41.39935247437
**22.317143**	-41.39935218311	-41.39935221909	-41.39935225776	-41.39935229929	-41.39935234523
**22.472857**	-41.39935212569	-41.39935215279	-41.39935217995	-41.39935220657	-41.39935223306
**22.628571**	-41.39935208068	-41.39935210636	-41.39935213058	-41.39935215253	-41.39935217153
**22.784286**	-41.39935203550	-41.39935206422	-41.39935209174	-41.39935211796	-41.39935214366
**22.940000**	-41.39935198430	-41.39935201612	-41.39935204710	-41.39935207730	-41.39935210744
**23.095714**	-41.39935192734	-41.39935196086	-41.39935199345	-41.39935202484	-41.39935205504
**23.251429**	-41.39935186682	-41.39935190081	-41.39935193360	-41.39935196459	-41.39935199329
**23.407143**	-41.39935180472	-41.39935183847	-41.39935187074	-41.39935190074	-41.39935192775
**23.562857**	-41.39935174246	-41.39935177556	-41.39935180699	-41.39935183587	-41.39935186139
**23.718571**	-41.39935168086	-41.39935171314	-41.39935174362	-41.39935177138	-41.39935179563
**23.874286**	-41.39935162044	-41.39935165179	-41.39935168130	-41.39935170803	-41.39935173119
**24.030000**	-41.39935156145	-41.39935159187	-41.39935162042	-41.39935164617	-41.39935166837
**24.185714**	-41.39935150404	-41.39935153353	-41.39935156114	-41.39935158600	-41.39935160734
**24.341429**	-41.39935144830	-41.39935147685	-41.39935150358	-41.39935152758	-41.39935154816
**24.497143**	-41.39935139420	-41.39935142188	-41.39935144773	-41.39935147094	-41.39935149079
**24.652857**	-41.39935134176	-41.39935136856	-41.39935139360	-41.39935141604	-41.39935143523
**24.808571**	-41.39935129093	-41.39935131690	-41.39935134115	-41.39935136287	-41.39935138143
**24.964286**	-41.39935124167	-41.39935126684	-41.39935129033	-41.39935131137	-41.39935132933
**25.120000**	-41.39935119395	-41.39935121835	-41.39935124111	-41.39935126149	-41.39935127888
**25.275714**	-41.39935114772	-41.39935117136	-41.39935119342	-41.39935121317	-41.39935123002
**25.431429**	-41.39935110292	-41.39935112584	-41.39935114722	-41.39935116636	-41.39935118270

R (Bohr)/θ(º)	100.00	110.00	120.00	130.00	140.00
**25.587143**	-41.39935105950	-41.39935108172	-41.39935110246	-41.39935112102	-41.39935113686
**25.742857**	-41.39935101742	-41.39935103897	-41.39935105908	-41.39935107709	-41.39935109245
**25.898571**	-41.39935097663	-41.39935099753	-41.39935101704	-41.39935103451	-41.39935104941
**26.054286**	-41.39935093708	-41.39935095736	-41.39935097629	-41.39935099324	-41.39935100770
**26.210000**	-41.39935089873	-41.39935091841	-41.39935093678	-41.39935095323	-41.39935096727
**26.365714**	-41.39935086154	-41.39935088064	-41.39935089846	-41.39935091443	-41.39935092806
**26.521429**	-41.39935082545	-41.39935084400	-41.39935086130	-41.39935087680	-41.39935089004
**26.677143**	-41.39935079045	-41.39935080845	-41.39935082525	-41.39935084031	-41.39935085316
**26.832857**	-41.39935075648	-41.39935077396	-41.39935079028	-41.39935080490	-41.39935081739
**26.988571**	-41.39935072351	-41.39935074049	-41.39935075634	-41.39935077054	-41.39935078267
**27.144286**	-41.39935069150	-41.39935070800	-41.39935072340	-41.39935073719	-41.39935074898
**27.300000**	-41.39935066043	-41.39935067645	-41.39935069142	-41.39935070483	-41.39935071628
**27.455714**	-41.39935063025	-41.39935064582	-41.39935066036	-41.39935067340	-41.39935068453
**27.611429**	-41.39935060094	-41.39935061608	-41.39935063021	-41.39935064288	-41.39935065371
**27.767143**	-41.39935057246	-41.39935058718	-41.39935060092	-41.39935061324	-41.39935062377
**27.922857**	-41.39935054480	-41.39935055911	-41.39935057247	-41.39935058445	-41.39935059469
**28.078571**	-41.39935051792	-41.39935053184	-41.39935054483	-41.39935055648	-41.39935056644
**28.234286**	-41.39935049181	-41.39935050534	-41.39935051797	-41.39935052930	-41.39935053899
**28.390000**	-41.39935046642	-41.39935047958	-41.39935049187	-41.39935050289	-41.39935051232
**28.545714**	-41.39935044182	-41.39935045457	-41.39935046651	-41.39935047723	-41.39935048640
**28.701429**	-41.39935042425	-41.39935043053	-41.39935044194	-41.39935045233	-41.39935046123
**28.857143**	-41.39935039444	-41.39935040778	-41.39935042154	-41.39935042851	-41.39935043691
**29.012857**	-41.39935037162	-41.39935038346	-41.39935039467	-41.39935040623	-41.39935042236
**29.168571**	-41.39935034951	-41.39935036100	-41.39935037176	-41.39935038144	-41.39935038984
**29.324286**	-41.39935032800	-41.39935033919	-41.39935034964	-41.39935035903	-41.39935036707
**29.480000**	-41.39935030708	-41.39935031797	-41.39935032814	-41.39935033728	-41.39935034510
**29.635714**	-41.39935028671	-41.39935029732	-41.39935030723	-41.39935031612	-41.39935032374
**29.791429**	-41.39935026689	-41.39935027721	-41.39935028687	-41.39935029553	-41.39935030295
**29.947143**	-41.39935024758	-41.39935025764	-41.39935026705	-41.39935027549	-41.39935028272
**30.102857**	-41.39935022879	-41.39935023859	-41.39935024775	-41.39935025598	-41.39935026302
**30.258571**	-41.39935021049	-41.39935022004	-41.39935022897	-41.39935023698	-41.39935024385
**30.414286**	-41.39935019266	-41.39935020197	-41.39935021067	-41.39935021849	-41.39935022517
**30.570000**	-41.39935017529	-41.39935018436	-41.39935019285	-41.39935020047	-41.39935020699
**30.725714**	-41.39935015837	-41.39935016722	-41.39935017549	-41.39935018291	-41.39935018927
**30.881429**	-41.39935014189	-41.39935015051	-41.39935015857	-41.39935016581	-41.39935017202
**31.037143**	-41.39935012582	-41.39935013423	-41.39935014209	-41.39935014915	-41.39935015520
**31.192857**	-41.39935011016	-41.39935011836	-41.39935012603	-41.39935013291	-41.39935013881
**31.348571**	-41.39935009489	-41.39935010289	-41.39935011037	-41.39935011709	-41.39935012284
**31.504286**	-41.39935008002	-41.39935008781	-41.39935009511	-41.39935010166	-41.39935010728
**31.660000**	-41.39935006550	-41.39935007312	-41.39935008023	-41.39935008662	-41.39935009210
**31.815714**	-41.39935005135	-41.39935005878	-41.39935006572	-41.39935007196	-41.39935007730
**31.971429**	-41.39935003755	-41.39935004479	-41.39935005157	-41.39935005766	-41.39935006288
**32.127143**	-41.39935002408	-41.39935003116	-41.39935003777	-41.39935004371	-41.39935004881
**32.282857**	-41.39935001095	-41.39935001785	-41.39935002431	-41.39935003011	-41.39935003508
**32.438571**	-41.39934999814	-41.39935000487	-41.39935001118	-41.39935001684	-41.39935002170
**32.594286**	-41.39934998563	-41.39934999221	-41.39934999837	-41.39935000390	-41.39935000863
**32.750000**	-41.39934997343	-41.39934997985	-41.39934998586	-41.39934999126	-41.39934999589
**32.905714**	-41.39934996152	-41.39934996779	-41.39934997366	-41.39934997893	-41.39934998345
**33.061429**	-41.39934994989	-41.39934995602	-41.39934996175	-41.39934996690	-41.39934997132
**33.217143**	-41.39934993854	-41.39934994453	-41.39934995012	-41.39934995516	-41.39934995947
**33.372857**	-41.39934992746	-41.39934993331	-41.39934993878	-41.39934994369	-41.39934994791
**33.528571**	-41.39934991664	-41.39934992235	-41.39934992769	-41.39934993249	-41.39934993661
**33.684286**	-41.39934990607	-41.39934991165	-41.39934991687	-41.39934992156	-41.39934992559
**33.840000**	-41.39934989575	-41.39934990120	-41.39934990630	-41.39934991089	-41.39934991482
**33.995714**	-41.39934988567	-41.39934989100	-41.39934989598	-41.39934990046	-41.39934990431
**34.151429**	-41.39934987582	-41.39934988103	-41.39934988590	-41.39934989028	-41.39934989404
**34.307143**	-41.39934986620	-41.39934987129	-41.39934987605	-41.39934988033	-41.39934988401
**34.462857**	-41.39934985679	-41.39934986177	-41.39934986642	-41.39934987061	-41.39934987420
**34.618571**	-41.39934984761	-41.39934985247	-41.39934985702	-41.39934986111	-41.39934986463
**34.774286**	-41.39934983862	-41.39934984338	-41.39934984783	-41.39934985184	-41.39934985527
**34.930000**	-41.39934982985	-41.39934983449	-41.39934983885	-41.39934984276	-41.39934984612
**35.085714**	-41.39934982127	-41.39934982581	-41.39934983007	-41.39934983390	-41.39934983718
**35.241429**	-41.39934981288	-41.39934981732	-41.39934982149	-41.39934982523	-41.39934982845
**35.397143**	-41.39934980467	-41.39934980902	-41.39934981310	-41.39934981676	-41.39934981990
**35.552857**	-41.39934979665	-41.39934980091	-41.39934980489	-41.39934980848	-41.39934981155

R (Bohr)/θ(º)	100.00	110.00	120.00	130.00	140.00
**35.708571**	-41.39934978881	-41.39934979297	-41.39934979687	-41.39934980038	-41.39934980339
**35.864286**	-41.39934978114	-41.39934978521	-41.39934978902	-41.39934979246	-41.39934979540
**36.020000**	-41.39934977363	-41.39934977762	-41.39934978135	-41.39934978471	-41.39934978759
**36.175714**	-41.39934976629	-41.39934977019	-41.39934977385	-41.39934977713	-41.39934977996
**36.331429**	-41.39934975911	-41.39934976293	-41.39934976650	-41.39934976972	-41.39934977248
**36.487143**	-41.39934975208	-41.39934975582	-41.39934975932	-41.39934976247	-41.39934976517
**36.642857**	-41.39934974521	-41.39934974887	-41.39934975229	-41.39934975537	-41.39934975802
**36.798571**	-41.39934973848	-41.39934974206	-41.39934974541	-41.39934974844	-41.39934975103
**36.954286**	-41.39934973189	-41.39934973540	-41.39934973868	-41.39934974164	-41.39934974418
**37.110000**	-41.39934972544	-41.39934972888	-41.39934973210	-41.39934973499	-41.39934973748
**37.265714**	-41.39934971914	-41.39934972250	-41.39934972565	-41.39934972849	-41.39934973092
**37.421429**	-41.39934971296	-41.39934971625	-41.39934971934	-41.39934972212	-41.39934972450
**37.577143**	-41.39934970691	-41.39934971014	-41.39934971316	-41.39934971588	-41.39934971822
**37.732857**	-41.39934970099	-41.39934970415	-41.39934970711	-41.39934970978	-41.39934971206
**37.888571**	-41.39934969519	-41.39934969828	-41.39934970119	-41.39934970380	-41.39934970604
**38.044286**	-41.39934968951	-41.39934969255	-41.39934969539	-41.39934969794	-41.39934970014
**38.200000**	-41.39934968395	-41.39934968692	-41.39934968971	-41.39934969221	-41.39934969436
**38.355714**	-41.39934967850	-41.39934968141	-41.39934968414	-41.39934968660	-41.39934968871
**38.511429**	-41.39934967316	-41.39934967602	-41.39934967869	-41.39934968110	-41.39934968317
**38.667143**	-41.39934966794	-41.39934967073	-41.39934967335	-41.39934967571	-41.39934967774
**38.822857**	-41.39934966281	-41.39934966556	-41.39934966812	-41.39934967044	-41.39934967242
**38.978571**	-41.39934965780	-41.39934966048	-41.39934966300	-41.39934966526	-41.39934966721
**39.134286**	-41.39934965288	-41.39934965551	-41.39934965798	-41.39934966020	-41.39934966211
**39.290000**	-41.39934964806	-41.39934965064	-41.39934965306	-41.39934965524	-41.39934965711
**39.445714**	-41.39934964333	-41.39934964587	-41.39934964824	-41.39934965037	-41.39934965221
**39.601429**	-41.39934963871	-41.39934964119	-41.39934964351	-41.39934964561	-41.39934964741
**39.757143**	-41.39934963417	-41.39934963660	-41.39934963888	-41.39934964094	-41.39934964270
**39.912857**	-41.39934962972	-41.39934963210	-41.39934963434	-41.39934963636	-41.39934963809
**40.068571**	-41.39934962535	-41.39934962770	-41.39934962990	-41.39934963187	-41.39934963357
**40.224286**	-41.39934962108	-41.39934962338	-41.39934962553	-41.39934962747	-41.39934962913
**40.380000**	-41.39934961689	-41.39934961914	-41.39934962125	-41.39934962315	-41.39934962479
**40.535714**	-41.39934961278	-41.39934961499	-41.39934961705	-41.39934961892	-41.39934962052
**40.691429**	-41.39934960874	-41.39934961091	-41.39934961294	-41.39934961477	-41.39934961635
**40.847143**	-41.39934960479	-41.39934960691	-41.39934960891	-41.39934961071	-41.39934961225
**41.002857**	-41.39934960091	-41.39934960300	-41.39934960495	-41.39934960671	-41.39934960823
**41.158571**	-41.39934959711	-41.39934959915	-41.39934960107	-41.39934960280	-41.39934960429
**41.314286**	-41.39934959337	-41.39934959539	-41.39934959727	-41.39934959896	-41.39934960042
**41.470000**	-41.39934958971	-41.39934959169	-41.39934959353	-41.39934959520	-41.39934959663
**41.625714**	-41.39934958612	-41.39934958806	-41.39934958987	-41.39934959150	-41.39934959291
**41.781429**	-41.39934958259	-41.39934958449	-41.39934958628	-41.39934958788	-41.39934958926
**41.937143**	-41.39934957913	-41.39934958100	-41.39934958275	-41.39934958433	-41.39934958568
**42.092857**	-41.39934957574	-41.39934957757	-41.39934957929	-41.39934958083	-41.39934958216
**42.248571**	-41.39934957241	-41.39934957421	-41.39934957589	-41.39934957741	-41.39934957872
**42.404286**	-41.39934956914	-41.39934957091	-41.39934957256	-41.39934957405	-41.39934957533
**42.560000**	-41.39934956593	-41.39934956767	-41.39934956929	-41.39934957075	-41.39934957201
**42.715714**	-41.39934956278	-41.39934956448	-41.39934956608	-41.39934956752	-41.39934956875
**42.871429**	-41.39934955969	-41.39934956136	-41.39934956293	-41.39934956434	-41.39934956555
**43.027143**	-41.39934955665	-41.39934955829	-41.39934955983	-41.39934956122	-41.39934956241
**43.182857**	-41.39934955367	-41.39934955529	-41.39934955679	-41.39934955816	-41.39934955933
**43.338571**	-41.39934955075	-41.39934955233	-41.39934955381	-41.39934955515	-41.39934955630
**43.494286**	-41.39934954787	-41.39934954943	-41.39934955088	-41.39934955220	-41.39934955333
**43.650000**	-41.39934954505	-41.39934954658	-41.39934954801	-41.39934954930	-41.39934955041
**43.805714**	-41.39934954228	-41.39934954378	-41.39934954519	-41.39934954646	-41.39934954755
**43.961429**	-41.39934953956	-41.39934954103	-41.39934954241	-41.39934954366	-41.39934954473
**44.117143**	-41.39934953688	-41.39934953833	-41.39934953969	-41.39934954091	-41.39934954197
**44.272857**	-41.39934953426	-41.39934953568	-41.39934953702	-41.39934953822	-41.39934953925
**44.428571**	-41.39934953168	-41.39934953308	-41.39934953439	-41.39934953557	-41.39934953659
**44.584286**	-41.39934952914	-41.39934953052	-41.39934953181	-41.39934953297	-41.39934953397
**44.740000**	-41.39934952665	-41.39934952800	-41.39934952927	-41.39934953041	-41.39934953140
**44.895714**	-41.39934952421	-41.39934952553	-41.39934952678	-41.39934952790	-41.39934952887
**45.051429**	-41.39934952180	-41.39934952311	-41.39934952433	-41.39934952544	-41.39934952639
**45.207143**	-41.39934951944	-41.39934952072	-41.39934952193	-41.39934952301	-41.39934952395
**45.362857**	-41.39934951712	-41.39934951838	-41.39934951957	-41.39934952063	-41.39934952155
**45.518571**	-41.39934951484	-41.39934951608	-41.39934951725	-41.39934951829	-41.39934951919
**45.674286**	-41.39934951260	-41.39934951382	-41.39934951496	-41.39934951599	-41.39934951688

R (Bohr)/θ(º)	100.00	110.00	120.00	130.00	140.00
**45.830000**	-41.39934951039	-41.39934951159	-41.39934951272	-41.39934951373	-41.39934951460
**45.985714**	-41.39934950823	-41.39934950941	-41.39934951052	-41.39934951151	-41.39934951237
**46.141429**	-41.39934950610	-41.39934950726	-41.39934950835	-41.39934950933	-41.39934951017
**46.297143**	-41.39934950401	-41.39934950515	-41.39934950622	-41.39934950718	-41.39934950801
**46.452857**	-41.39934950195	-41.39934950307	-41.39934950412	-41.39934950507	-41.39934950589
**46.608571**	-41.39934949993	-41.39934950103	-41.39934950206	-41.39934950300	-41.39934950380
**46.764286**	-41.39934949794	-41.39934949902	-41.39934950004	-41.39934950096	-41.39934950175
**46.920000**	-41.39934949598	-41.39934949705	-41.39934949805	-41.39934949895	-41.39934949973
**47.075714**	-41.39934949406	-41.39934949511	-41.39934949610	-41.39934949698	-41.39934949775
**47.231429**	-41.39934949217	-41.39934949320	-41.39934949417	-41.39934949504	-41.39934949580
**47.387143**	-41.39934949031	-41.39934949133	-41.39934949228	-41.39934949314	-41.39934949388
**47.542857**	-41.39934948848	-41.39934948948	-41.39934949042	-41.39934949126	-41.39934949199
**47.698571**	-41.39934948668	-41.39934948767	-41.39934948859	-41.39934948942	-41.39934949014
**47.854286**	-41.39934948491	-41.39934948588	-41.39934948679	-41.39934948761	-41.39934948831
**48.010000**	-41.39934948318	-41.39934948413	-41.39934948502	-41.39934948583	-41.39934948652
**48.165714**	-41.39934948146	-41.39934948240	-41.39934948328	-41.39934948407	-41.39934948475
**48.321429**	-41.39934947978	-41.39934948070	-41.39934948157	-41.39934948235	-41.39934948302
**48.477143**	-41.39934947812	-41.39934947903	-41.39934947988	-41.39934948065	-41.39934948131
**48.632857**	-41.39934947649	-41.39934947739	-41.39934947822	-41.39934947898	-41.39934947963
**48.788571**	-41.39934947489	-41.39934947577	-41.39934947659	-41.39934947734	-41.39934947798
**48.944286**	-41.39934947331	-41.39934947418	-41.39934947499	-41.39934947572	-41.39934947635
**49.100000**	-41.39934947176	-41.39934947261	-41.39934947341	-41.39934947413	-41.39934947475
**49.255714**	-41.39934947023	-41.39934947107	-41.39934947186	-41.39934947257	-41.39934947317
**49.411429**	-41.39934946873	-41.39934946955	-41.39934947033	-41.39934947103	-41.39934947163
**49.567143**	-41.39934946725	-41.39934946806	-41.39934946882	-41.39934946951	-41.39934947010
**49.722857**	-41.39934946579	-41.39934946659	-41.39934946734	-41.39934946802	-41.39934946860
**49.878571**	-41.39934946436	-41.39934946515	-41.39934946589	-41.39934946655	-41.39934946713
**50.034286**	-41.39934946295	-41.39934946372	-41.39934946445	-41.39934946511	-41.39934946567
**50.190000**	-41.39934946156	-41.39934946232	-41.39934946304	-41.39934946369	-41.39934946424
**50.345714**	-41.39934946019	-41.39934946094	-41.39934946165	-41.39934946229	-41.39934946283
**50.501429**	-41.39934945884	-41.39934945959	-41.39934946028	-41.39934946091	-41.39934946145
**50.657143**	-41.39934945752	-41.39934945825	-41.39934945893	-41.39934945955	-41.39934946008
**50.812857**	-41.39934945622	-41.39934945694	-41.39934945761	-41.39934945822	-41.39934945874
**50.968571**	-41.39934945493	-41.39934945564	-41.39934945630	-41.39934945690	-41.39934945742
**51.124286**	-41.39934945367	-41.39934945436	-41.39934945502	-41.39934945561	-41.39934945611
**51.280000**	-41.39934945242	-41.39934945311	-41.39934945375	-41.39934945433	-41.39934945483
**51.435714**	-41.39934945119	-41.39934945187	-41.39934945251	-41.39934945308	-41.39934945357
**51.591429**	-41.39934944999	-41.39934945065	-41.39934945128	-41.39934945184	-41.39934945233
**51.747143**	-41.39934944880	-41.39934944945	-41.39934945007	-41.39934945063	-41.39934945110
**51.902857**	-41.39934944763	-41.39934944827	-41.39934944888	-41.39934944943	-41.39934944990
**52.058571**	-41.39934944647	-41.39934944711	-41.39934944771	-41.39934944825	-41.39934944871
**52.214286**	-41.39934944533	-41.39934944596	-41.39934944655	-41.39934944708	-41.39934944754
**52.370000**	-41.39934944422	-41.39934944484	-41.39934944542	-41.39934944594	-41.39934944639
**52.525714**	-41.39934944311	-41.39934944372	-41.39934944430	-41.39934944481	-41.39934944526
**52.681429**	-41.39934944203	-41.39934944263	-41.39934944319	-41.39934944370	-41.39934944414
**52.837143**	-41.39934944096	-41.39934944155	-41.39934944211	-41.39934944261	-41.39934944304
**52.992857**	-41.39934943990	-41.39934944049	-41.39934944104	-41.39934944153	-41.39934944196
**53.148571**	-41.39934943887	-41.39934943944	-41.39934943998	-41.39934944047	-41.39934944089
**53.304286**	-41.39934943784	-41.39934943841	-41.39934943894	-41.39934943942	-41.39934943984
**53.460000**	-41.39934943683	-41.39934943739	-41.39934943792	-41.39934943839	-41.39934943880
**53.615714**	-41.39934943584	-41.39934943639	-41.39934943691	-41.39934943738	-41.39934943778
**53.771429**	-41.39934943486	-41.39934943541	-41.39934943591	-41.39934943638	-41.39934943677
**53.927143**	-41.39934943390	-41.39934943443	-41.39934943494	-41.39934943539	-41.39934943578
**54.082857**	-41.39934943295	-41.39934943348	-41.39934943397	-41.39934943442	-41.39934943480
**54.238571**	-41.39934943201	-41.39934943253	-41.39934943302	-41.39934943346	-41.39934943384
**54.394286**	-41.39934943109	-41.39934943160	-41.39934943208	-41.39934943252	-41.39934943289
**54.550000**	-41.39934943018	-41.39934943068	-41.39934943116	-41.39934943159	-41.39934943195
**54.705714**	-41.39934942928	-41.39934942978	-41.39934943025	-41.39934943067	-41.39934943103
**54.861429**	-41.39934942840	-41.39934942889	-41.39934942935	-41.39934942977	-41.39934943012
**55.017143**	-41.39934942752	-41.39934942801	-41.39934942846	-41.39934942888	-41.39934942923
**55.172857**	-41.39934942667	-41.39934942714	-41.39934942759	-41.39934942800	-41.39934942835
**55.328571**	-41.39934942582	-41.39934942629	-41.39934942673	-41.39934942713	-41.39934942748
**55.484286**	-41.39934942498	-41.39934942545	-41.39934942588	-41.39934942628	-41.39934942662
**55.640000**	-41.39934942416	-41.39934942462	-41.39934942505	-41.39934942544	-41.39934942577
**55.795714**	-41.39934942335	-41.39934942380	-41.39934942423	-41.39934942461	-41.39934942494

R (Bohr)/θ(º)	100.00	110.00	120.00	130.00	140.00
**55.951429**	-41.39934942255	-41.39934942300	-41.39934942341	-41.39934942379	-41.39934942412
**56.107143**	-41.39934942176	-41.39934942220	-41.39934942261	-41.39934942299	-41.39934942331
**56.262857**	-41.39934942098	-41.39934942142	-41.39934942183	-41.39934942219	-41.39934942251
**56.418571**	-41.39934942021	-41.39934942064	-41.39934942105	-41.39934942141	-41.39934942172
**56.574286**	-41.39934941946	-41.39934941988	-41.39934942028	-41.39934942064	-41.39934942094
**56.730000**	-41.39934941871	-41.39934941913	-41.39934941952	-41.39934941987	-41.39934942018
**56.885714**	-41.39934941798	-41.39934941839	-41.39934941878	-41.39934941912	-41.39934941943
**57.041429**	-41.39934941725	-41.39934941766	-41.39934941804	-41.39934941838	-41.39934941868
**57.197143**	-41.39934941654	-41.39934941694	-41.39934941731	-41.39934941765	-41.39934941795
**57.352857**	-41.39934941583	-41.39934941623	-41.39934941660	-41.39934941694	-41.39934941722
**57.508571**	-41.39934941514	-41.39934941553	-41.39934941589	-41.39934941622	-41.39934941651
**57.664286**	-41.39934941445	-41.39934941483	-41.39934941519	-41.39934941552	-41.39934941580
**57.820000**	-41.39934941377	-41.39934941415	-41.39934941451	-41.39934941483	-41.39934941511
**57.975714**	-41.39934941310	-41.39934941348	-41.39934941383	-41.39934941415	-41.39934941442
**58.131429**	-41.39934941244	-41.39934941281	-41.39934941316	-41.39934941348	-41.39934941375
**58.287143**	-41.39934941179	-41.39934941216	-41.39934941250	-41.39934941281	-41.39934941308
**58.442857**	-41.39934941115	-41.39934941151	-41.39934941185	-41.39934941216	-41.39934941242
**58.598571**	-41.39934941052	-41.39934941088	-41.39934941121	-41.39934941151	-41.39934941177
**58.754286**	-41.39934940989	-41.39934941025	-41.39934941058	-41.39934941088	-41.39934941113
**58.910000**	-41.39934940928	-41.39934940963	-41.39934940995	-41.39934941025	-41.39934941050
**59.065714**	-41.39934940867	-41.39934940901	-41.39934940934	-41.39934940963	-41.39934940988
**59.221429**	-41.39934940807	-41.39934940841	-41.39934940873	-41.39934940901	-41.39934940926
**59.377143**	-41.39934940748	-41.39934940782	-41.39934940813	-41.39934940841	-41.39934940865
**59.532857**	-41.39934940690	-41.39934940723	-41.39934940754	-41.39934940782	-41.39934940805
**59.688571**	-41.39934940632	-41.39934940665	-41.39934940695	-41.39934940723	-41.39934940746
**59.844286**	-41.39934940575	-41.39934940607	-41.39934940638	-41.39934940665	-41.39934940688
**60.000000**	-41.39934939119	-41.39934938997	-41.39934940580	-41.39934940607	-41.39934940629

PES aV5Z – CH^- ($X\ ^3\Sigma^-$) + He				
**R (Bohr)/θ(º)**	**150.00**	**160.00**	**170.00**	**180.00**
**5.500000**	-41.39738736512	-41.39739955112	-41.39740666206	-41.39738587957
**5.655714**	-41.39786173679	-41.39788205822	-41.39789656617	-41.39787699977
**5.811429**	-41.39823469530	-41.39826060212	-41.39828037170	-41.39826042990
**5.967143**	-41.39852782413	-41.39855742777	-41.39858047431	-41.39855937967
**6.122857**	-41.39875806510	-41.39878988747	-41.39881445530	-41.39879187596
**6.278571**	-41.39893875286	-41.39897152785	-41.39899631487	-41.39897224298
**6.434286**	-41.39908033567	-41.39911303307	-41.39913691289	-41.39911163302
**6.590000**	-41.39919090663	-41.39922254690	-41.39924501801	-41.39921875736
**6.745714**	-41.39927679368	-41.39930674763	-41.39932749650	-41.39930044576
**6.901429**	-41.39934307916	-41.39937089375	-41.39938982352	-41.39936215575
**7.057143**	-41.39939371767	-41.39941919496	-41.39943628227	-41.39940817913
**7.212857**	-41.39943191731	-41.39945498474	-41.39947031822	-41.39944190874
**7.368571**	-41.39946019213	-41.39948093656	-41.39949460914	-41.39946602624
**7.524286**	-41.39948059082	-41.39949914220	-41.39951129218	-41.39948268366
**7.680000**	-41.39949471206	-41.39951126361	-41.39952205115	-41.39949356850
**7.835714**	-41.39950385862	-41.39951862613	-41.39952819788	-41.39949999500
**7.991429**	-41.39950906549	-41.39952226497	-41.39952924751	-41.39950300642
**8.147143**	-41.39951119564	-41.39952298833	-41.39952764592	-41.39950341199
**8.302857**	-41.39951089567	-41.39952146875	-41.39952151325	-41.39950185944
**8.458571**	-41.39950869719	-41.39951820087	-41.39951596234	-41.39949884273
**8.614286**	-41.39950503759	-41.39951133305	-41.39951023840	-41.39949475620
**8.770000**	-41.39950023193	-41.39950354602	-41.39950368536	-41.39948991099
**8.925714**	-41.39949467631	-41.39949268595	-41.39949662302	-41.39948455425
**9.081429**	-41.39948591979	-41.39948576464	-41.39948893251	-41.39947856348
**9.237143**	-41.39947663908	-41.39947832875	-41.39948151203	-41.39947278392
**9.392857**	-41.39946666225	-41.39947129389	-41.39947407455	-41.39946689447
**9.548571**	-41.39946022556	-41.39946438213	-41.39946686274	-41.39946111979
**9.704286**	-41.39945396166	-41.39945768982	-41.39945990527	-41.39945546181
**9.860000**	-41.39944793163	-41.39945127496	-41.39945123366	-41.39944997177
**10.015714**	-41.39944218270	-41.39944519582	-41.39944548558	-41.39944468193
**10.171429**	-41.39943674402	-41.39943947058	-41.39944003285	-41.39943962297
**10.327143**	-41.39943163304	-41.39943412083	-41.39943488699	-41.39943478201
**10.482857**	-41.39942684874	-41.39942913512	-41.39943003655	-41.39943016783
**10.638571**	-41.39942239271	-41.39942447765	-41.39942547301	-41.39942576893
**10.794286**	-41.39941822311	-41.39942012853	-41.39942118906	-41.39942159819
**10.950000**	-41.39941434726	-41.39941612872	-41.39941721422	-41.39941764658
**11.105714**	-41.39941075825	-41.39941237469	-41.39941348367	-41.39941390829
**11.261429**	-41.39940742330	-41.39940895246	-41.39940997776	-41.39941037690
**11.417143**	-41.39940432889	-41.39940575061	-41.39940669389	-41.39940703288
**11.572857**	-41.39940146951	-41.39940276257	-41.39940362653	-41.39940390749
**11.728571**	-41.39939879287	-41.39939993155	-41.39940064026	-41.39940092572
**11.884286**	-41.39939616895	-41.39939727795	-41.39939786700	-41.39939812298
**12.040000**	-41.39939378115	-41.39939477062	-41.39939524180	-41.39939547522
**12.195714**	-41.39939156055	-41.39939244440	-41.39939276440	-41.39939297776
**12.351429**	-41.39938948532	-41.39939027926	-41.39939045407	-41.39939064102
**12.507143**	-41.39938753304	-41.39938826417	-41.39938829015	-41.39938845301
**12.662857**	-41.39938569332	-41.39938635088	-41.39938624957	-41.39938639900
**12.818571**	-41.39938398218	-41.39938457402	-41.39938433145	-41.39938446966
**12.974286**	-41.39938231137	-41.39938272061	-41.39938253315	-41.39938265764
**13.130000**	-41.39938057159	-41.39938057975	-41.39938084433	-41.39938095602
**13.285714**	-41.39937892743	-41.39937896826	-41.39937925730	-41.39937935803
**13.441429**	-41.39937737432	-41.39937750451	-41.39937776615	-41.39937785709
**13.597143**	-41.39937590778	-41.39937612791	-41.39937636472	-41.39937644682
**13.752857**	-41.39937452353	-41.39937483290	-41.39937504713	-41.39937512118
**13.908571**	-41.39937321739	-41.39937361425	-41.39937380787	-41.39937387456
**14.064286**	-41.39937220034	-41.39937246698	-41.39937264179	-41.39937270174
**14.220000**	-41.39937112663	-41.39937138649	-41.39937154412	-41.39937159785
**14.375714**	-41.39937013341	-41.39937035414	-41.39937049906	-41.39937054906
**14.531429**	-41.39936919604	-41.39936939622	-41.39936952748	-41.39936957273
**14.687143**	-41.39936831081	-41.39936849239	-41.39936861126	-41.39936865220
**14.842857**	-41.39936747427	-41.39936763907	-41.39936774674	-41.39936778377
**14.998571**	-41.39936668317	-41.39936683287	-41.39936693048	-41.39936696398
**15.154286**	-41.39936593437	-41.39936607066	-41.39936615922	-41.39936618957
**15.310000**	-41.39936521920	-41.39936534866	-41.39936542959	-41.39936545720

R (Bohr)/θ(º)	150.00	160.00	170.00	180.00
**15.465714**	-41.39936455256	-41.39936466480	-41.39936473681	-41.39936476158
**15.621429**	-41.39936391686	-41.39936402055	-41.39936408743	-41.39936411021
**15.777143**	-41.39936331252	-41.39936340778	-41.39936346902	-41.39936348984
**15.932857**	-41.39936273857	-41.39936282636	-41.39936288271	-41.39936290181
**16.088571**	-41.39936219324	-41.39936227440	-41.39936232649	-41.39936234412
**16.244286**	-41.39936167492	-41.39936175012	-41.39936179844	-41.39936181481
**16.400000**	-41.39936118201	-41.39936125171	-41.39936129683	-41.39936131213
**16.555714**	-41.39936071341	-41.39936077766	-41.39936081985	-41.39936083423
**16.711429**	-41.39936026848	-41.39936032675	-41.39936036596	-41.39936037952
**16.867143**	-41.39935984756	-41.39935989848	-41.39935993387	-41.39935994650
**17.022857**	-41.39935945170	-41.39935949474	-41.39935952355	-41.39935953444
**17.178571**	-41.39935907834	-41.39935912042	-41.39935914127	-41.39935914811
**17.334286**	-41.39935871675	-41.39935876626	-41.39935879318	-41.39935880074
**17.490000**	-41.39935835607	-41.39935840999	-41.39935844163	-41.39935845192
**17.645714**	-41.39935800702	-41.39935805727	-41.39935808756	-41.39935809749
**17.801429**	-41.39935769487	-41.39935774453	-41.39935777527	-41.39935778551
**17.957143**	-41.39935738393	-41.39935743110	-41.39935746204	-41.39935747594
**18.112857**	-41.39935709029	-41.39935713596	-41.39935716448	-41.39935718029
**18.268571**	-41.39935681267	-41.39935685904	-41.39935688809	-41.39935689820
**18.424286**	-41.39935654628	-41.39935659189	-41.39935661963	-41.39935662884
**18.580000**	-41.39935629054	-41.39935633537	-41.39935636249	-41.39935637146
**18.735714**	-41.39935604549	-41.39935608972	-41.39935611651	-41.39935612538
**18.891429**	-41.39935581087	-41.39935585460	-41.39935588117	-41.39935588997
**19.047143**	-41.39935558626	-41.39935562954	-41.39935565592	-41.39935566467
**19.202857**	-41.39935537119	-41.39935541402	-41.39935544023	-41.39935544895
**19.358571**	-41.39935516520	-41.39935520760	-41.39935523363	-41.39935524232
**19.514286**	-41.39935496784	-41.39935500982	-41.39935503567	-41.39935504431
**19.670000**	-41.39935477868	-41.39935482023	-41.39935484591	-41.39935485452
**19.825714**	-41.39935459730	-41.39935463846	-41.39935466395	-41.39935467252
**19.981429**	-41.39935442329	-41.39935446407	-41.39935448943	-41.39935449795
**20.137143**	-41.39935425625	-41.39935429673	-41.39935432196	-41.39935433046
**20.292857**	-41.39935409573	-41.39935413603	-41.39935416120	-41.39935416970
**20.448571**	-41.39935394121	-41.39935398159	-41.39935400682	-41.39935401536
**20.604286**	-41.39935379187	-41.39935383285	-41.39935385846	-41.39935386711
**20.760000**	-41.39935366005	-41.39935369216	-41.39935374267	-41.39935372957
**20.915714**	-41.39935346216	-41.39935353216	-41.39935356617	-41.39935357152
**21.071429**	-41.39935336408	-41.39935340111	-41.39935343432	-41.39935345652
**21.227143**	-41.39935325408	-41.39935329437	-41.39935332099	-41.39935333033
**21.382857**	-41.39935313281	-41.39935317332	-41.39935319973	-41.39935320887
**21.538571**	-41.39935301322	-41.39935305502	-41.39935308247	-41.39935309203
**21.694286**	-41.39935289564	-41.39935293975	-41.39935296924	-41.39935297961
**21.850000**	-41.39935277884	-41.39935282668	-41.39935285962	-41.39935287142
**22.005714**	-41.39935266030	-41.39935271388	-41.39935275284	-41.39935276727
**22.161429**	-41.39935253558	-41.39935259696	-41.39935264700	-41.39935266699
**22.317143**	-41.39935239918	-41.39935246517	-41.39935253561	-41.39935257040
**22.472857**	-41.39935226214	-41.39935230310	-41.39935238406	-41.39935247734
**22.628571**	-41.39935218724	-41.39935220011	-41.39935221632	-41.39935238765
**22.784286**	-41.39935217139	-41.39935220760	-41.39935226133	-41.39935230121
**22.940000**	-41.39935213890	-41.39935217247	-41.39935220378	-41.39935221784
**23.095714**	-41.39935208390	-41.39935211012	-41.39935212989	-41.39935213744
**23.251429**	-41.39935201897	-41.39935204022	-41.39935205469	-41.39935205986
**23.407143**	-41.39935195095	-41.39935196914	-41.39935198093	-41.39935198499
**23.562857**	-41.39935188274	-41.39935189900	-41.39935190925	-41.39935191272
**23.718571**	-41.39935181559	-41.39935183054	-41.39935183982	-41.39935184293
**23.874286**	-41.39935175006	-41.39935176405	-41.39935177266	-41.39935177553
**24.030000**	-41.39935168636	-41.39935169961	-41.39935170771	-41.39935171041
**24.185714**	-41.39935162458	-41.39935163722	-41.39935164492	-41.39935164748
**24.341429**	-41.39935156472	-41.39935157683	-41.39935158421	-41.39935158666
**24.497143**	-41.39935150675	-41.39935151841	-41.39935152549	-41.39935152784
**24.652857**	-41.39935145063	-41.39935146188	-41.39935146870	-41.39935147096
**24.808571**	-41.39935139632	-41.39935140718	-41.39935141376	-41.39935141595
**24.964286**	-41.39935134374	-41.39935135423	-41.39935136060	-41.39935136272
**25.120000**	-41.39935129283	-41.39935130299	-41.39935130915	-41.39935131120
**25.275714**	-41.39935124353	-41.39935125338	-41.39935125935	-41.39935126133
**25.431429**	-41.39935119580	-41.39935120534	-41.39935121112	-41.39935121304

R (Bohr)/θ(º)	150.00	160.00	170.00	180.00
**25.587143**	-41.39935114956	-41.39935115881	-41.39935116442	-41.39935116628
**25.742857**	-41.39935110476	-41.39935111374	-41.39935111918	-41.39935112098
**25.898571**	-41.39935106136	-41.39935107007	-41.39935107535	-41.39935107709
**26.054286**	-41.39935101930	-41.39935102775	-41.39935103287	-41.39935103457
**26.210000**	-41.39935097852	-41.39935098672	-41.39935099169	-41.39935099334
**26.365714**	-41.39935093899	-41.39935094695	-41.39935095178	-41.39935095338
**26.521429**	-41.39935090065	-41.39935090839	-41.39935091308	-41.39935091463
**26.677143**	-41.39935086347	-41.39935087098	-41.39935087554	-41.39935087705
**26.832857**	-41.39935082740	-41.39935083470	-41.39935083913	-41.39935084060
**26.988571**	-41.39935079240	-41.39935079949	-41.39935080380	-41.39935080523
**27.144286**	-41.39935075843	-41.39935076533	-41.39935076952	-41.39935077091
**27.300000**	-41.39935072547	-41.39935073218	-41.39935073624	-41.39935073759
**27.455714**	-41.39935069347	-41.39935069999	-41.39935070394	-41.39935070526
**27.611429**	-41.39935066240	-41.39935066874	-41.39935067258	-41.39935067386
**27.767143**	-41.39935063222	-41.39935063838	-41.39935064213	-41.39935064337
**27.922857**	-41.39935060291	-41.39935060891	-41.39935061255	-41.39935061375
**28.078571**	-41.39935057443	-41.39935058027	-41.39935058381	-41.39935058499
**28.234286**	-41.39935054677	-41.39935055245	-41.39935055589	-41.39935055704
**28.390000**	-41.39935051989	-41.39935052541	-41.39935052877	-41.39935052988
**28.545714**	-41.39935049377	-41.39935049914	-41.39935050241	-41.39935050349
**28.701429**	-41.39935046839	-41.39935047362	-41.39935047680	-41.39935047785
**28.857143**	-41.39935044381	-41.39935044887	-41.39935045195	-41.39935045297
**29.012857**	-41.39935042135	-41.39935042540	-41.39935042819	-41.39935042914
**29.168571**	-41.39935039693	-41.39935040324	-41.39935040964	-41.39935041294
**29.324286**	-41.39935037355	-41.39935037831	-41.39935038121	-41.39935038218
**29.480000**	-41.39935035138	-41.39935035597	-41.39935035877	-41.39935035970
**29.635714**	-41.39935032986	-41.39935033433	-41.39935033704	-41.39935033794
**29.791429**	-41.39935030891	-41.39935031327	-41.39935031591	-41.39935031679
**29.947143**	-41.39935028853	-41.39935029277	-41.39935029535	-41.39935029620
**30.102857**	-41.39935026869	-41.39935027282	-41.39935027533	-41.39935027617
**30.258571**	-41.39935024936	-41.39935025340	-41.39935025585	-41.39935025666
**30.414286**	-41.39935023055	-41.39935023448	-41.39935023687	-41.39935023766
**30.570000**	-41.39935021223	-41.39935021607	-41.39935021839	-41.39935021917
**30.725714**	-41.39935019439	-41.39935019812	-41.39935020039	-41.39935020115
**30.881429**	-41.39935017700	-41.39935018065	-41.39935018286	-41.39935018360
**31.037143**	-41.39935016006	-41.39935016362	-41.39935016578	-41.39935016650
**31.192857**	-41.39935014356	-41.39935014703	-41.39935014913	-41.39935014984
**31.348571**	-41.39935012747	-41.39935013086	-41.39935013291	-41.39935013360
**31.504286**	-41.39935011179	-41.39935011510	-41.39935011710	-41.39935011777
**31.660000**	-41.39935009651	-41.39935009973	-41.39935010169	-41.39935010234
**31.815714**	-41.39935008161	-41.39935008475	-41.39935008667	-41.39935008730
**31.971429**	-41.39935006708	-41.39935007015	-41.39935007245	-41.39935007265
**32.127143**	-41.39935005291	-41.39935005590	-41.39935005773	-41.39935005833
**32.282857**	-41.39935003908	-41.39935004201	-41.39935004379	-41.39935004438
**32.438571**	-41.39935002560	-41.39935002846	-41.39935003020	-41.39935003078
**32.594286**	-41.39935001245	-41.39935001524	-41.39935001694	-41.39935001750
**32.750000**	-41.39934999962	-41.39935000234	-41.39935000400	-41.39935000455
**32.905714**	-41.39934998709	-41.39934998976	-41.39934999138	-41.39934999191
**33.061429**	-41.39934997487	-41.39934997747	-41.39934997905	-41.39934997958
**33.217143**	-41.39934996294	-41.39934996548	-41.39934996703	-41.39934996754
**33.372857**	-41.39934995130	-41.39934995378	-41.39934995529	-41.39934995579
**33.528571**	-41.39934993993	-41.39934994236	-41.39934994383	-41.39934994432
**33.684286**	-41.39934992883	-41.39934993120	-41.39934993264	-41.39934993312
**33.840000**	-41.39934991799	-41.39934992031	-41.39934992172	-41.39934992219
**33.995714**	-41.39934990741	-41.39934990967	-41.39934991105	-41.39934991151
**34.151429**	-41.39934989706	-41.39934989928	-41.39934990063	-41.39934990108
**34.307143**	-41.39934988696	-41.39934988913	-41.39934989045	-41.39934989089
**34.462857**	-41.39934987710	-41.39934987922	-41.39934988050	-41.39934988093
**34.618571**	-41.39934986746	-41.39934986953	-41.39934987079	-41.39934987121
**34.774286**	-41.39934985804	-41.39934986006	-41.39934986130	-41.39934986170
**34.930000**	-41.39934984883	-41.39934985081	-41.39934985202	-41.39934985242
**35.085714**	-41.39934983983	-41.39934984177	-41.39934984295	-41.39934984334
**35.241429**	-41.39934983104	-41.39934983293	-41.39934983409	-41.39934983447
**35.397143**	-41.39934982244	-41.39934982429	-41.39934982542	-41.39934982580
**35.552857**	-41.39934981403	-41.39934981585	-41.39934981695	-41.39934981732

R (Bohr)/θ(º)	150.00	160.00	170.00	180.00
**35.708571**	-41.39934980581	-41.39934980759	-41.39934980867	-41.39934980903
**35.864286**	-41.39934979778	-41.39934979951	-41.39934980057	-41.39934980093
**36.020000**	-41.39934978992	-41.39934979162	-41.39934979266	-41.39934979300
**36.175714**	-41.39934978223	-41.39934978390	-41.39934978491	-41.39934978525
**36.331429**	-41.39934977471	-41.39934977634	-41.39934977733	-41.39934977766
**36.487143**	-41.39934976735	-41.39934976895	-41.39934976992	-41.39934977024
**36.642857**	-41.39934976016	-41.39934976172	-41.39934976267	-41.39934976299
**36.798571**	-41.39934975312	-41.39934975465	-41.39934975558	-41.39934975589
**36.954286**	-41.39934974623	-41.39934974772	-41.39934974864	-41.39934974894
**37.110000**	-41.39934973948	-41.39934974095	-41.39934974184	-41.39934974214
**37.265714**	-41.39934973288	-41.39934973432	-41.39934973520	-41.39934973549
**37.421429**	-41.39934972642	-41.39934972783	-41.39934972869	-41.39934972897
**37.577143**	-41.39934972010	-41.39934972148	-41.39934972232	-41.39934972260
**37.732857**	-41.39934971391	-41.39934971526	-41.39934971608	-41.39934971636
**37.888571**	-41.39934970785	-41.39934970917	-41.39934970998	-41.39934971025
**38.044286**	-41.39934970191	-41.39934970321	-41.39934970400	-41.39934970426
**38.200000**	-41.39934969610	-41.39934969737	-41.39934969815	-41.39934969840
**38.355714**	-41.39934969041	-41.39934969166	-41.39934969242	-41.39934969267
**38.511429**	-41.39934968483	-41.39934968606	-41.39934968680	-41.39934968705
**38.667143**	-41.39934967937	-41.39934968057	-41.39934968130	-41.39934968154
**38.822857**	-41.39934967402	-41.39934967520	-41.39934967591	-41.39934967615
**38.978571**	-41.39934966878	-41.39934966994	-41.39934967064	-41.39934967087
**39.134286**	-41.39934966365	-41.39934966478	-41.39934966547	-41.39934966570
**39.290000**	-41.39934965862	-41.39934965973	-41.39934966040	-41.39934966062
**39.445714**	-41.39934965369	-41.39934965478	-41.39934965544	-41.39934965566
**39.601429**	-41.39934964886	-41.39934964992	-41.39934965057	-41.39934965079
**39.757143**	-41.39934964413	-41.39934964517	-41.39934964581	-41.39934964602
**39.912857**	-41.39934963949	-41.39934964051	-41.39934964113	-41.39934964134
**40.068571**	-41.39934963493	-41.39934963594	-41.39934963655	-41.39934963675
**40.224286**	-41.39934963048	-41.39934963146	-41.39934963206	-41.39934963226
**40.380000**	-41.39934962610	-41.39934962707	-41.39934962766	-41.39934962785
**40.535714**	-41.39934962182	-41.39934962276	-41.39934962334	-41.39934962354
**40.691429**	-41.39934961761	-41.39934961855	-41.39934961911	-41.39934961930
**40.847143**	-41.39934961349	-41.39934961441	-41.39934961496	-41.39934961515
**41.002857**	-41.39934960945	-41.39934961035	-41.39934961089	-41.39934961107
**41.158571**	-41.39934960549	-41.39934960637	-41.39934960690	-41.39934960708
**41.314286**	-41.39934960160	-41.39934960246	-41.39934960299	-41.39934960316
**41.470000**	-41.39934959778	-41.39934959863	-41.39934959915	-41.39934959932
**41.625714**	-41.39934959404	-41.39934959488	-41.39934959538	-41.39934959555
**41.781429**	-41.39934959037	-41.39934959119	-41.39934959168	-41.39934959185
**41.937143**	-41.39934958677	-41.39934958757	-41.39934958806	-41.39934958822
**42.092857**	-41.39934958324	-41.39934958402	-41.39934958450	-41.39934958466
**42.248571**	-41.39934957977	-41.39934958054	-41.39934958101	-41.39934958117
**42.404286**	-41.39934957637	-41.39934957712	-41.39934957759	-41.39934957774
**42.560000**	-41.39934957303	-41.39934957377	-41.39934957423	-41.39934957438
**42.715714**	-41.39934956975	-41.39934957048	-41.39934957093	-41.39934957107
**42.871429**	-41.39934956653	-41.39934956725	-41.39934956769	-41.39934956783
**43.027143**	-41.39934956337	-41.39934956408	-41.39934956451	-41.39934956465
**43.182857**	-41.39934956027	-41.39934956097	-41.39934956139	-41.39934956153
**43.338571**	-41.39934955723	-41.39934955791	-41.39934955833	-41.39934955846
**43.494286**	-41.39934955424	-41.39934955491	-41.39934955532	-41.39934955545
**43.650000**	-41.39934955131	-41.39934955196	-41.39934955237	-41.39934955250
**43.805714**	-41.39934954843	-41.39934954907	-41.39934954946	-41.39934954960
**43.961429**	-41.39934954560	-41.39934954623	-41.39934954662	-41.39934954675
**44.117143**	-41.39934954282	-41.39934954344	-41.39934954382	-41.39934954395
**44.272857**	-41.39934954009	-41.39934954070	-41.39934954108	-41.39934954120
**44.428571**	-41.39934953741	-41.39934953801	-41.39934953838	-41.39934953850
**44.584286**	-41.39934953477	-41.39934953537	-41.39934953573	-41.39934953585
**44.740000**	-41.39934953219	-41.39934953277	-41.39934953313	-41.39934953324
**44.895714**	-41.39934952965	-41.39934953022	-41.39934953057	-41.39934953068
**45.051429**	-41.39934952715	-41.39934952771	-41.39934952806	-41.39934952817
**45.207143**	-41.39934952470	-41.39934952525	-41.39934952559	-41.39934952570
**45.362857**	-41.39934952229	-41.39934952283	-41.39934952316	-41.39934952327
**45.518571**	-41.39934951992	-41.39934952046	-41.39934952078	-41.39934952089
**45.674286**	-41.39934951759	-41.39934951812	-41.39934951844	-41.39934951855

R (Bohr)/θ(º)	150.00	160.00	170.00	180.00
**45.830000**	-41.39934951531	-41.39934951582	-41.39934951614	-41.39934951624
**45.985714**	-41.39934951306	-41.39934951357	-41.39934951388	-41.39934951398
**46.141429**	-41.39934951085	-41.39934951135	-41.39934951166	-41.39934951176
**46.297143**	-41.39934950868	-41.39934950917	-41.39934950947	-41.39934950957
**46.452857**	-41.39934950655	-41.39934950703	-41.39934950732	-41.39934950742
**46.608571**	-41.39934950445	-41.39934950492	-41.39934950521	-41.39934950531
**46.764286**	-41.39934950238	-41.39934950285	-41.39934950314	-41.39934950323
**46.920000**	-41.39934950036	-41.39934950082	-41.39934950110	-41.39934950119
**47.075714**	-41.39934949836	-41.39934949882	-41.39934949909	-41.39934949918
**47.231429**	-41.39934949640	-41.39934949685	-41.39934949712	-41.39934949721
**47.387143**	-41.39934949448	-41.39934949491	-41.39934949518	-41.39934949527
**47.542857**	-41.39934949258	-41.39934949301	-41.39934949327	-41.39934949336
**47.698571**	-41.39934949071	-41.39934949114	-41.39934949140	-41.39934949148
**47.854286**	-41.39934948888	-41.39934948930	-41.39934948955	-41.39934948964
**48.010000**	-41.39934948708	-41.39934948749	-41.39934948774	-41.39934948782
**48.165714**	-41.39934948530	-41.39934948571	-41.39934948595	-41.39934948603
**48.321429**	-41.39934948356	-41.39934948396	-41.39934948420	-41.39934948428
**48.477143**	-41.39934948184	-41.39934948223	-41.39934948247	-41.39934948255
**48.632857**	-41.39934948015	-41.39934948054	-41.39934948077	-41.39934948085
**48.788571**	-41.39934947849	-41.39934947887	-41.39934947910	-41.39934947918
**48.944286**	-41.39934947686	-41.39934947723	-41.39934947746	-41.39934947754
**49.100000**	-41.39934947525	-41.39934947562	-41.39934947584	-41.39934947592
**49.255714**	-41.39934947367	-41.39934947403	-41.39934947425	-41.39934947432
**49.411429**	-41.39934947211	-41.39934947247	-41.39934947268	-41.39934947276
**49.567143**	-41.39934947058	-41.39934947093	-41.39934947114	-41.39934947122
**49.722857**	-41.39934946907	-41.39934946942	-41.39934946963	-41.39934946970
**49.878571**	-41.39934946759	-41.39934946793	-41.39934946814	-41.39934946820
**50.034286**	-41.39934946613	-41.39934946646	-41.39934946667	-41.39934946674
**50.190000**	-41.39934946469	-41.39934946502	-41.39934946522	-41.39934946529
**50.345714**	-41.39934946328	-41.39934946360	-41.39934946380	-41.39934946386
**50.501429**	-41.39934946188	-41.39934946220	-41.39934946240	-41.39934946246
**50.657143**	-41.39934946051	-41.39934946083	-41.39934946102	-41.39934946108
**50.812857**	-41.39934945916	-41.39934945947	-41.39934945966	-41.39934945972
**50.968571**	-41.39934945783	-41.39934945814	-41.39934945833	-41.39934945839
**51.124286**	-41.39934945653	-41.39934945683	-41.39934945701	-41.39934945707
**51.280000**	-41.39934945524	-41.39934945553	-41.39934945572	-41.39934945577
**51.435714**	-41.39934945397	-41.39934945426	-41.39934945444	-41.39934945450
**51.591429**	-41.39934945272	-41.39934945301	-41.39934945318	-41.39934945324
**51.747143**	-41.39934945149	-41.39934945177	-41.39934945195	-41.39934945200
**51.902857**	-41.39934945028	-41.39934945056	-41.39934945073	-41.39934945079
**52.058571**	-41.39934944908	-41.39934944936	-41.39934944953	-41.39934944959
**52.214286**	-41.39934944791	-41.39934944818	-41.39934944835	-41.39934944840
**52.370000**	-41.39934944676	-41.39934944702	-41.39934944719	-41.39934944724
**52.525714**	-41.39934944562	-41.39934944588	-41.39934944604	-41.39934944609
**52.681429**	-41.39934944449	-41.39934944475	-41.39934944491	-41.39934944496
**52.837143**	-41.39934944339	-41.39934944364	-41.39934944380	-41.39934944385
**52.992857**	-41.39934944230	-41.39934944255	-41.39934944270	-41.39934944276
**53.148571**	-41.39934944123	-41.39934944147	-41.39934944162	-41.39934944168
**53.304286**	-41.39934944017	-41.39934944041	-41.39934944056	-41.39934944061
**53.460000**	-41.39934943913	-41.39934943937	-41.39934943952	-41.39934943957
**53.615714**	-41.39934943810	-41.39934943834	-41.39934943848	-41.39934943853
**53.771429**	-41.39934943709	-41.39934943733	-41.39934943747	-41.39934943751
**53.927143**	-41.39934943609	-41.39934943633	-41.39934943647	-41.39934943652
**54.082857**	-41.39934943511	-41.39934943534	-41.39934943548	-41.39934943553
**54.238571**	-41.39934943414	-41.39934943437	-41.39934943451	-41.39934943455
**54.394286**	-41.39934943319	-41.39934943341	-41.39934943355	-41.39934943359
**54.550000**	-41.39934943225	-41.39934943247	-41.39934943260	-41.39934943265
**54.705714**	-41.39934943133	-41.39934943154	-41.39934943167	-41.39934943172
**54.861429**	-41.39934943041	-41.39934943063	-41.39934943076	-41.39934943080
**55.017143**	-41.39934942951	-41.39934942972	-41.39934942985	-41.39934942990
**55.172857**	-41.39934942863	-41.39934942883	-41.39934942896	-41.39934942900
**55.328571**	-41.39934942776	-41.39934942796	-41.39934942808	-41.39934942812
**55.484286**	-41.39934942689	-41.39934942710	-41.39934942722	-41.39934942726
**55.640000**	-41.39934942604	-41.39934942624	-41.39934942636	-41.39934942640
**55.795714**	-41.39934942521	-41.39934942540	-41.39934942552	-41.39934942556

R (Bohr)/θ(º)	150.00	160.00	170.00	180.00
**55.951429**	-41.39934942438	-41.39934942457	-41.39934942469	-41.39934942473
**56.107143**	-41.39934942357	-41.39934942376	-41.39934942387	-41.39934942391
**56.262857**	-41.39934942277	-41.39934942295	-41.39934942307	-41.39934942311
**56.418571**	-41.39934942197	-41.39934942216	-41.39934942227	-41.39934942231
**56.574286**	-41.39934942119	-41.39934942138	-41.39934942149	-41.39934942153
**56.730000**	-41.39934942043	-41.39934942060	-41.39934942072	-41.39934942075
**56.885714**	-41.39934941967	-41.39934941984	-41.39934941995	-41.39934941999
**57.041429**	-41.39934941892	-41.39934941909	-41.39934941920	-41.39934941924
**57.197143**	-41.39934941818	-41.39934941836	-41.39934941846	-41.39934941849
**57.352857**	-41.39934941745	-41.39934941763	-41.39934941773	-41.39934941776
**57.508571**	-41.39934941674	-41.39934941690	-41.39934941701	-41.39934941704
**57.664286**	-41.39934941603	-41.39934941619	-41.39934941630	-41.39934941633
**57.820000**	-41.39934941533	-41.39934941550	-41.39934941559	-41.39934941563
**57.975714**	-41.39934941464	-41.39934941480	-41.39934941490	-41.39934941494
**58.131429**	-41.39934941396	-41.39934941412	-41.39934941422	-41.39934941425
**58.287143**	-41.39934941329	-41.39934941345	-41.39934941355	-41.39934941358
**58.442857**	-41.39934941263	-41.39934941279	-41.39934941288	-41.39934941292
**58.598571**	-41.39934941198	-41.39934941214	-41.39934941223	-41.39934941226
**58.754286**	-41.39934941134	-41.39934941149	-41.39934941158	-41.39934941161
**58.910000**	-41.39934941071	-41.39934941085	-41.39934941095	-41.39934941098
**59.065714**	-41.39934941008	-41.39934941023	-41.39934941032	-41.39934941035
**59.221429**	-41.39934940946	-41.39934940960	-41.39934940969	-41.39934940972
**59.377143**	-41.39934940885	-41.39934940900	-41.39934940908	-41.39934940911
**59.532857**	-41.39934940825	-41.39934940839	-41.39934940848	-41.39934940851
**59.688571**	-41.39934940765	-41.39934940780	-41.39934940788	-41.39934940791
**59.844286**	-41.39934940707	-41.39934940721	-41.39934940730	-41.39934940732
**60.000000**	-41.39934940543	-41.39934940554	-41.39934940315	-41.39934940671

PES aV6Z – CH^- (X $^3\Sigma^-$) + He					
**R (Bohr)/θ(º)**	**0.00**	**10.00**	**20.00**	**30.00**	**40.00**
**5.500000**	-41.40085532453	-41.40122702388	-41.40214335452	-41.40320316185	-41.40405168011
**5.655714**	-41.40208462179	-41.40235938548	-41.40305976232	-41.40387282085	-41.40451901697
**5.811429**	-41.40301148024	-41.40323037895	-41.40376530577	-41.40438902169	-41.40487992121
**5.967143**	-41.40371859170	-41.40390090719	-41.40430913179	-41.40478714391	-41.40515848551
**6.122857**	-41.40427395946	-41.40441747615	-41.40472875024	-41.40509395627	-41.40537335427
**6.278571**	-41.40469889605	-41.40481535587	-41.40505238948	-41.40533003188	-41.40553899355
**6.434286**	-41.40502366407	-41.40512121333	-41.40530157095	-41.40551135193	-41.40566663650
**6.590000**	-41.40527184897	-41.40535569191	-41.40549285867	-41.40565035639	-41.40576495715
**6.745714**	-41.40546168969	-41.40553507630	-41.40563917867	-41.40575671598	-41.40584050940
**6.901429**	-41.40560712944	-41.40567205870	-41.40575072876	-41.40583791884	-41.40589828529
**7.057143**	-41.40571861263	-41.40577649411	-41.40583547844	-41.40589969912	-41.40594206449
**7.212857**	-41.40580387041	-41.40585581685	-41.40589959278	-41.40594631535	-41.40597478977
**7.368571**	-41.40586864528	-41.40591564769	-41.40594766541	-41.40598100592	-41.40599878305
**7.524286**	-41.40591725868	-41.40596021060	-41.40598319563	-41.40600625118	-41.40601588917
**7.680000**	-41.40595309328	-41.40599273133	-41.40600885238	-41.40602406148	-41.40602763329
**7.835714**	-41.40597887060	-41.40601583922	-41.40602678927	-41.40603605154	-41.40603525436
**7.991429**	-41.40599687294	-41.40603165670	-41.40603877809	-41.40604356825	-41.40603974751
**8.147143**	-41.40600896977	-41.40604194910	-41.40604620127	-41.40604772218	-41.40604185022
**8.302857**	-41.40601641647	-41.40604805414	-41.40605018576	-41.40604935901	-41.40604220424
**8.458571**	-41.40602088895	-41.40605086293	-41.40605163880	-41.40604918210	-41.40604122382
**8.614286**	-41.40602298511	-41.40605160412	-41.40605090454	-41.40604763187	-41.40603923376
**8.770000**	-41.40602330349	-41.40605032451	-41.40604914289	-41.40604508221	-41.40603648302
**8.925714**	-41.40602244893	-41.40604845058	-41.40604636714	-41.40604149960	-41.40603315261
**9.081429**	-41.40602065185	-41.40604384261	-41.40604285516	-41.40603768843	-41.40602940243
**9.237143**	-41.40601816497	-41.40603825955	-41.40603658872	-41.40603347151	-41.40602504799
**9.392857**	-41.40601517163	-41.40603121887	-41.40602934698	-41.40602764283	-41.40602011726
**9.548571**	-41.40601183427	-41.40602453917	-41.40602218896	-41.40602130719	-41.40601654382
**9.704286**	-41.40600826615	-41.40601969954	-41.40601741847	-41.40601321038	-41.40601058113
**9.860000**	-41.40600458376	-41.40601359585	-41.40601262329	-41.40600869020	-41.40600539166
**10.015714**	-41.40600086015	-41.40600689441	-41.40600788155	-41.40600425553	-41.40599947027
**10.171429**	-41.40599717206	-41.40600095802	-41.40600323343	-41.40599994118	-41.40599572344
**10.327143**	-41.40599356622	-41.40599680265	-41.40599870893	-41.40599575945	-41.40599213505
**10.482857**	-41.40599007601	-41.40599277536	-41.40599433136	-41.40599173115	-41.40598870559
**10.638571**	-41.40598672637	-41.40598889264	-41.40599009747	-41.40598785278	-41.40598543336
**10.794286**	-41.40598352954	-41.40598538323	-41.40598641305	-41.40598446180	-41.40598230818
**10.950000**	-41.40598048956	-41.40598206808	-41.40598294445	-41.40598126900	-41.40597931108
**11.105714**	-41.40597760568	-41.40597894064	-41.40597969871	-41.40597826480	-41.40597662576
**11.261429**	-41.40597487409	-41.40597599027	-41.40597526301	-41.40597543950	-41.40597408565
**11.417143**	-41.40597228934	-41.40597321659	-41.40597259004	-41.40597278004	-41.40597168004
**11.572857**	-41.40596984580	-41.40597061713	-41.40597007395	-41.40597029124	-41.40596939919
**11.728571**	-41.40596753950	-41.40596817602	-41.40596770126	-41.40596794677	-41.40596723743
**11.884286**	-41.40596536590	-41.40596588305	-41.40596546910	-41.40596573568	-41.40596518911
**12.040000**	-41.40596330210	-41.40596312166	-41.40596336701	-41.40596286334	-41.40596325389
**12.195714**	-41.40596134773	-41.40596118661	-41.40596138837	-41.40596097620	-41.40596142821
**12.351429**	-41.40595950388	-41.40595935631	-41.40595952833	-41.40595919647	-41.40595969704
**12.507143**	-41.40595776422	-41.40595763074	-41.40595778027	-41.40595751807	-41.40595807361
**12.662857**	-41.40595612347	-41.40595600211	-41.40595613753	-41.40595593658	-41.40595583087
**12.818571**	-41.40595457794	-41.40595446930	-41.40595458974	-41.40595444660	-41.40595441617
**12.974286**	-41.40595312400	-41.40595302894	-41.40595313732	-41.40595304715	-41.40595308191
**13.130000**	-41.40595175648	-41.40595167310	-41.40595177494	-41.40595173061	-41.40595182362
**13.285714**	-41.40595046688	-41.40595039404	-41.40595049413	-41.40595049036	-41.40595063720
**13.441429**	-41.40594924926	-41.40594918691	-41.40594905587	-41.40594932253	-41.40594951924
**13.597143**	-41.40594810146	-41.40594804884	-41.40594794149	-41.40594822647	-41.40594846610
**13.752857**	-41.40594702134	-41.40594697740	-41.40594689123	-41.40594719753	-41.40594744110
**13.908571**	-41.40594600554	-41.40594597071	-41.40594590235	-41.40594623280	-41.40594651017
**14.064286**	-41.40594505029	-41.40594502095	-41.40594497021	-41.40594531504	-41.40594563758
**14.220000**	-41.40594415179	-41.40594412857	-41.40594408856	-41.40594404868	-41.40594482485
**14.375714**	-41.40594333266	-41.40594330404	-41.40594326090	-41.40594323673	-41.40594406357
**14.531429**	-41.40594253728	-41.40594251394	-41.40594248030	-41.40594246917	-41.40594335253
**14.687143**	-41.40594178759	-41.40594176885	-41.40594174332	-41.40594174337	-41.40594249307
**14.842857**	-41.40594108029	-41.40594106549	-41.40594104730	-41.40594105675	-41.40594112901
**14.998571**	-41.40594041233	-41.40594040100	-41.40594038916	-41.40594040674	-41.40594048536
**15.154286**	-41.40593978099	-41.40593977258	-41.40593976656	-41.40593979096	-41.40593987449
**15.310000**	-41.40593918378	-41.40593917790	-41.40593917710	-41.40593920721	-41.40593929435

R (Bohr)/θ(º)	0.00	10.00	20.00	30.00	40.00
**15.465714**	-41.40593861856	-41.40593861483	-41.40593861861	-41.40593865350	-41.40593874317
**15.621429**	-41.40593808326	-41.40593808138	-41.40593808914	-41.40593812793	-41.40593821911
**15.777143**	-41.40593757613	-41.40593757582	-41.40593758693	-41.40593762881	-41.40593772072
**15.932857**	-41.40593709410	-41.40593709651	-41.40593711040	-41.40593715465	-41.40593724653
**16.088571**	-41.40593663980	-41.40593664195	-41.40593665804	-41.40593670396	-41.40593679526
**16.244286**	-41.40593620760	-41.40593621068	-41.40593622846	-41.40593627546	-41.40593636564
**16.400000**	-41.40593579750	-41.40593580134	-41.40593582036	-41.40593586789	-41.40593595652
**16.555714**	-41.40593540823	-41.40593541265	-41.40593543251	-41.40593548010	-41.40593556680
**16.711429**	-41.40593503856	-41.40593504341	-41.40593506375	-41.40593511095	-41.40593519540
**16.867143**	-41.40593468736	-41.40593469248	-41.40593471297	-41.40593475941	-41.40593484134
**17.022857**	-41.40593435354	-41.40593435880	-41.40593437914	-41.40593442450	-41.40593450367
**17.178571**	-41.40593403610	-41.40593404134	-41.40593406127	-41.40593410526	-41.40593418149
**17.334286**	-41.40593373407	-41.40593373918	-41.40593375844	-41.40593380080	-41.40593387393
**17.490000**	-41.40593344658	-41.40593345143	-41.40593346977	-41.40593351028	-41.40593358020
**17.645714**	-41.40593317277	-41.40593317724	-41.40593319444	-41.40593323290	-41.40593329954
**17.801429**	-41.40593291186	-41.40593291584	-41.40593293166	-41.40593296792	-41.40593303122
**17.957143**	-41.40593266311	-41.40593266648	-41.40593268071	-41.40593271460	-41.40593277456
**18.112857**	-41.40593242584	-41.40593242846	-41.40593244088	-41.40593247228	-41.40593252892
**18.268571**	-41.40593219939	-41.40593220113	-41.40593221149	-41.40593224032	-41.40593229367
**18.424286**	-41.40593198315	-41.40593198384	-41.40593199189	-41.40593201807	-41.40593206825
**18.580000**	-41.40593177656	-41.40593177597	-41.40593178145	-41.40593180491	-41.40593185205
**18.735714**	-41.40593157902	-41.40593157688	-41.40593157946	-41.40593160023	-41.40593164453
**18.891429**	-41.40593138944	-41.40593138535	-41.40593138467	-41.40593140300	-41.40593144491
**19.047143**	-41.40593120670	-41.40593119881	-41.40593118543	-41.40593119103	-41.40593124866
**19.202857**	-41.40593103651	-41.40593102685	-41.40593101859	-41.40593103170	-41.40593106619
**19.358571**	-41.40593087104	-41.40593085708	-41.40593084473	-41.40593085661	-41.40593089150
**19.514286**	-41.40593071239	-41.40593069218	-41.40593067515	-41.40593068455	-41.40593071727
**19.670000**	-41.40593056021	-41.40593053019	-41.40593050745	-41.40593051488	-41.40593054767
**19.825714**	-41.40593041316	-41.40593036934	-41.40593035017	-41.40593036504	-41.40593039890
**19.981429**	-41.40593027353	-41.40593020562	-41.40593018911	-41.40593021340	-41.40593025117
**20.137143**	-41.40593014027	-41.40593004187	-41.40593004697	-41.40593007125	-41.40593010376
**20.292857**	-41.40593001142	-41.40592989696	-41.40592991175	-41.40592993739	-41.40592997392
**20.448571**	-41.40592988773	-41.40592979805	-41.40592979360	-41.40592981391	-41.40592984737
**20.604286**	-41.40592976882	-41.40592971552	-41.40592969131	-41.40592970166	-41.40592972972
**20.760000**	-41.40592965454	-41.40592962533	-41.40592959801	-41.40592959933	-41.40592962087
**20.915714**	-41.40592954467	-41.40592952881	-41.40592950736	-41.40592950416	-41.40592951992
**21.071429**	-41.40592943902	-41.40592943054	-41.40592941665	-41.40592941325	-41.40592942523
**21.227143**	-41.40592933739	-41.40592933316	-41.40592932574	-41.40592932459	-41.40592933507
**21.382857**	-41.40592923963	-41.40592923794	-41.40592923536	-41.40592923729	-41.40592924799
**21.538571**	-41.40592914556	-41.40592914545	-41.40592914632	-41.40592915120	-41.40592916303
**21.694286**	-41.40592905505	-41.40592905594	-41.40592905919	-41.40592906652	-41.40592907979
**21.850000**	-41.40592896796	-41.40592896949	-41.40592897436	-41.40592898356	-41.40592899820
**22.005714**	-41.40592888416	-41.40592888611	-41.40592889207	-41.40592890263	-41.40592891838
**22.161429**	-41.40592880355	-41.40592880576	-41.40592881245	-41.40592882394	-41.40592884050
**22.317143**	-41.40592872595	-41.40592872835	-41.40592873551	-41.40592874765	-41.40592876480
**22.472857**	-41.40592865110	-41.40592865362	-41.40592866114	-41.40592867377	-41.40592869140
**22.628571**	-41.40592857832	-41.40592858095	-41.40592858879	-41.40592860195	-41.40592862022
**22.784286**	-41.40592850636	-41.40592850906	-41.40592851717	-41.40592853093	-41.40592855031
**22.940000**	-41.40592843617	-41.40592843866	-41.40592844624	-41.40592845946	-41.40592847910
**23.095714**	-41.40592837811	-41.40592838010	-41.40592838614	-41.40592839655	-41.40592841186
**23.251429**	-41.40592832670	-41.40592832879	-41.40592833509	-41.40592834584	-41.40592836125
**23.407143**	-41.40592826454	-41.40592826675	-41.40592827338	-41.40592828454	-41.40592830035
**23.562857**	-41.40592819868	-41.40592820086	-41.40592820735	-41.40592821817	-41.40592823326
**23.718571**	-41.40592813562	-41.40592813775	-41.40592814408	-41.40592815464	-41.40592816938
**23.874286**	-41.40592807621	-41.40592807830	-41.40592808451	-41.40592809489	-41.40592810944
**24.030000**	-41.40592801995	-41.40592802199	-41.40592802809	-41.40592803830	-41.40592805265
**24.185714**	-41.40592796627	-41.40592796828	-41.40592797425	-41.40592798428	-41.40592799839
**24.341429**	-41.40592791481	-41.40592791677	-41.40592792262	-41.40592793244	-41.40592794627
**24.497143**	-41.40592786530	-41.40592786722	-41.40592787294	-41.40592788253	-41.40592789606
**24.652857**	-41.40592781756	-41.40592781943	-41.40592782502	-41.40592783438	-41.40592784759
**24.808571**	-41.40592777147	-41.40592777329	-41.40592777874	-41.40592778787	-41.40592780075
**24.964286**	-41.40592772691	-41.40592772869	-41.40592773399	-41.40592774290	-41.40592775544
**25.120000**	-41.40592768380	-41.40592768553	-41.40592769070	-41.40592769938	-41.40592771159
**25.275714**	-41.40592764207	-41.40592764376	-41.40592764880	-41.40592765725	-41.40592766913
**25.431429**	-41.40592760166	-41.40592760331	-41.40592760822	-41.40592761643	-41.40592762799

R (Bohr)/θ(º)	0.00	10.00	20.00	30.00	40.00
**25.587143**	-41.40592756252	-41.40592756412	-41.40592756890	-41.40592757689	-41.40592758814
**25.742857**	-41.40592752458	-41.40592752614	-41.40592753079	-41.40592753857	-41.40592754950
**25.898571**	-41.40592748780	-41.40592748932	-41.40592749384	-41.40592750142	-41.40592751205
**26.054286**	-41.40592745213	-41.40592745361	-41.40592745802	-41.40592746539	-41.40592747573
**26.210000**	-41.40592741753	-41.40592741897	-41.40592742327	-41.40592743044	-41.40592744051
**26.365714**	-41.40592738397	-41.40592738537	-41.40592738955	-41.40592739654	-41.40592740633
**26.521429**	-41.40592735139	-41.40592735276	-41.40592735683	-41.40592736364	-41.40592737316
**26.677143**	-41.40592731977	-41.40592732111	-41.40592732508	-41.40592733170	-41.40592734097
**26.832857**	-41.40592728907	-41.40592729037	-41.40592729424	-41.40592730070	-41.40592730972
**26.988571**	-41.40592725923	-41.40592726050	-41.40592726428	-41.40592727058	-41.40592727937
**27.144286**	-41.40592723019	-41.40592723145	-41.40592723515	-41.40592724131	-41.40592724989
**27.300000**	-41.40592720181	-41.40592720306	-41.40592720675	-41.40592721283	-41.40592722123
**27.455714**	-41.40592717288	-41.40592717438	-41.40592717853	-41.40592718488	-41.40592719329
**27.611429**	-41.40592712797	-41.40592712284	-41.40592711406	-41.40592713723	-41.40592716507
**27.767143**	-41.40592712208	-41.40592712316	-41.40592712633	-41.40592713148	-41.40592713752
**27.922857**	-41.40592709782	-41.40592710047	-41.40592710391	-41.40592710949	-41.40592711534
**28.078571**	-41.40592707356	-41.40592707619	-41.40592707955	-41.40592708500	-41.40592709068
**28.234286**	-41.40592704991	-41.40592705251	-41.40592705446	-41.40592706086	-41.40592706659
**28.390000**	-41.40592702689	-41.40592702946	-41.40592703110	-41.40592703736	-41.40592704316
**28.545714**	-41.40592700449	-41.40592700571	-41.40592700860	-41.40592701474	-41.40592702036
**28.701429**	-41.40592698270	-41.40592698365	-41.40592698671	-41.40592699273	-41.40592699818
**28.857143**	-41.40592696150	-41.40592696242	-41.40592696541	-41.40592697132	-41.40592697611
**29.012857**	-41.40592694087	-41.40592694177	-41.40592694445	-41.40592695024	-41.40592695511
**29.168571**	-41.40592692078	-41.40592692166	-41.40592692403	-41.40592692838	-41.40592693443
**29.324286**	-41.40592690099	-41.40592690185	-41.40592690440	-41.40592690864	-41.40592691453
**29.480000**	-41.40592688196	-41.40592688280	-41.40592688528	-41.40592688942	-41.40592689517
**29.635714**	-41.40592686343	-41.40592686425	-41.40592686667	-41.40592687071	-41.40592687631
**29.791429**	-41.40592684538	-41.40592684618	-41.40592684855	-41.40592685249	-41.40592685795
**29.947143**	-41.40592682781	-41.40592682859	-41.40592683090	-41.40592683474	-41.40592684007
**30.102857**	-41.40592681068	-41.40592681144	-41.40592681370	-41.40592681745	-41.40592682265
**30.258571**	-41.40592679401	-41.40592679475	-41.40592679695	-41.40592680061	-41.40592680568
**30.414286**	-41.40592677775	-41.40592677848	-41.40592678063	-41.40592678419	-41.40592678915
**30.570000**	-41.40592676192	-41.40592676262	-41.40592676472	-41.40592676821	-41.40592677304
**30.725714**	-41.40592674648	-41.40592674717	-41.40592674922	-41.40592675262	-41.40592675734
**30.881429**	-41.40592673143	-41.40592673210	-41.40592673411	-41.40592673743	-41.40592674203
**31.037143**	-41.40592671676	-41.40592671742	-41.40592671937	-41.40592672262	-41.40592672711
**31.192857**	-41.40592670246	-41.40592670310	-41.40592670501	-41.40592670818	-41.40592671256
**31.348571**	-41.40592668851	-41.40592668913	-41.40592669100	-41.40592669410	-41.40592669838
**31.504286**	-41.40592667490	-41.40592667552	-41.40592667734	-41.40592668036	-41.40592668454
**31.660000**	-41.40592666164	-41.40592666224	-41.40592666402	-41.40592666697	-41.40592667105
**31.815714**	-41.40592664869	-41.40592664928	-41.40592665102	-41.40592665390	-41.40592665789
**31.971429**	-41.40592663606	-41.40592663664	-41.40592663833	-41.40592664115	-41.40592664505
**32.127143**	-41.40592662374	-41.40592662430	-41.40592662596	-41.40592662871	-41.40592663252
**32.282857**	-41.40592661171	-41.40592661226	-41.40592661388	-41.40592661657	-41.40592662029
**32.438571**	-41.40592659997	-41.40592660051	-41.40592660210	-41.40592660472	-41.40592660836
**32.594286**	-41.40592658851	-41.40592658904	-41.40592659059	-41.40592659316	-41.40592659671
**32.750000**	-41.40592657733	-41.40592657784	-41.40592657936	-41.40592658187	-41.40592658534
**32.905714**	-41.40592656641	-41.40592656691	-41.40592656839	-41.40592657085	-41.40592657424
**33.061429**	-41.40592655574	-41.40592655623	-41.40592655769	-41.40592656008	-41.40592656340
**33.217143**	-41.40592654533	-41.40592654581	-41.40592654723	-41.40592654958	-41.40592655282
**33.372857**	-41.40592653516	-41.40592653563	-41.40592653702	-41.40592653931	-41.40592654248
**33.528571**	-41.40592652522	-41.40592652568	-41.40592652704	-41.40592652928	-41.40592653238
**33.684286**	-41.40592651552	-41.40592651596	-41.40592651729	-41.40592651949	-41.40592652252
**33.840000**	-41.40592650603	-41.40592650647	-41.40592650777	-41.40592650992	-41.40592651288
**33.995714**	-41.40592649676	-41.40592649719	-41.40592649847	-41.40592650057	-41.40592650347
**34.151429**	-41.40592648771	-41.40592648813	-41.40592648937	-41.40592649143	-41.40592649427
**34.307143**	-41.40592647886	-41.40592647927	-41.40592648049	-41.40592648250	-41.40592648527
**34.462857**	-41.40592647021	-41.40592647061	-41.40592647180	-41.40592647377	-41.40592647648
**34.618571**	-41.40592646175	-41.40592646214	-41.40592646331	-41.40592646524	-41.40592646789
**34.774286**	-41.40592645348	-41.40592645387	-41.40592645501	-41.40592645689	-41.40592645950
**34.930000**	-41.40592644540	-41.40592644578	-41.40592644689	-41.40592644874	-41.40592645128
**35.085714**	-41.40592643749	-41.40592643786	-41.40592643896	-41.40592644077	-41.40592644326
**35.241429**	-41.40592642976	-41.40592643013	-41.40592643120	-41.40592643297	-41.40592643540
**35.397143**	-41.40592642220	-41.40592642256	-41.40592642361	-41.40592642534	-41.40592642772
**35.552857**	-41.40592641480	-41.40592641516	-41.40592641618	-41.40592641788	-41.40592642021

R (Bohr)/θ(º)	0.00	10.00	20.00	30.00	40.00
**35.708571**	-41.40592640757	-41.40592640791	-41.40592640892	-41.40592641058	-41.40592641287
**35.864286**	-41.40592640049	-41.40592640083	-41.40592640182	-41.40592640344	-41.40592640568
**36.020000**	-41.40592639357	-41.40592639389	-41.40592639486	-41.40592639645	-41.40592639865
**36.175714**	-41.40592638679	-41.40592638711	-41.40592638806	-41.40592638962	-41.40592639177
**36.331429**	-41.40592638016	-41.40592638047	-41.40592638140	-41.40592638293	-41.40592638503
**36.487143**	-41.40592637367	-41.40592637398	-41.40592637488	-41.40592637638	-41.40592637844
**36.642857**	-41.40592636732	-41.40592636762	-41.40592636851	-41.40592636998	-41.40592637200
**36.798571**	-41.40592636110	-41.40592636139	-41.40592636227	-41.40592636370	-41.40592636568
**36.954286**	-41.40592635501	-41.40592635530	-41.40592635616	-41.40592635757	-41.40592635950
**37.110000**	-41.40592634905	-41.40592634933	-41.40592635017	-41.40592635155	-41.40592635345
**37.265714**	-41.40592634321	-41.40592634349	-41.40592634431	-41.40592634567	-41.40592634753
**37.421429**	-41.40592633750	-41.40592633777	-41.40592633858	-41.40592633990	-41.40592634173
**37.577143**	-41.40592633190	-41.40592633217	-41.40592633296	-41.40592633426	-41.40592633605
**37.732857**	-41.40592632642	-41.40592632669	-41.40592632746	-41.40592632873	-41.40592633048
**37.888571**	-41.40592632105	-41.40592632131	-41.40592632207	-41.40592632332	-41.40592632503
**38.044286**	-41.40592631579	-41.40592631604	-41.40592631679	-41.40592631801	-41.40592631970
**38.200000**	-41.40592631064	-41.40592631089	-41.40592631162	-41.40592631282	-41.40592631447
**38.355714**	-41.40592630559	-41.40592630583	-41.40592630655	-41.40592630773	-41.40592630935
**38.511429**	-41.40592630064	-41.40592630088	-41.40592630158	-41.40592630274	-41.40592630432
**38.667143**	-41.40592629580	-41.40592629603	-41.40592629672	-41.40592629785	-41.40592629941
**38.822857**	-41.40592629105	-41.40592629127	-41.40592629195	-41.40592629306	-41.40592629459
**38.978571**	-41.40592628639	-41.40592628662	-41.40592628728	-41.40592628837	-41.40592628986
**39.134286**	-41.40592628183	-41.40592628205	-41.40592628270	-41.40592628377	-41.40592628524
**39.290000**	-41.40592627735	-41.40592627757	-41.40592627821	-41.40592627926	-41.40592628070
**39.445714**	-41.40592627297	-41.40592627318	-41.40592627381	-41.40592627484	-41.40592627625
**39.601429**	-41.40592626867	-41.40592626888	-41.40592626949	-41.40592627050	-41.40592627189
**39.757143**	-41.40592626445	-41.40592626466	-41.40592626526	-41.40592626625	-41.40592626762
**39.912857**	-41.40592626032	-41.40592626052	-41.40592626112	-41.40592626209	-41.40592626342
**40.068571**	-41.40592625627	-41.40592625647	-41.40592625705	-41.40592625801	-41.40592625931
**40.224286**	-41.40592625230	-41.40592625249	-41.40592625306	-41.40592625399	-41.40592625528
**40.380000**	-41.40592624840	-41.40592624858	-41.40592624915	-41.40592625007	-41.40592625133
**40.535714**	-41.40592624458	-41.40592624476	-41.40592624531	-41.40592624621	-41.40592624745
**40.691429**	-41.40592624082	-41.40592624101	-41.40592624155	-41.40592624243	-41.40592624365
**40.847143**	-41.40592623714	-41.40592623732	-41.40592623785	-41.40592623872	-41.40592623992
**41.002857**	-41.40592623354	-41.40592623371	-41.40592623423	-41.40592623509	-41.40592623626
**41.158571**	-41.40592622999	-41.40592623016	-41.40592623068	-41.40592623152	-41.40592623267
**41.314286**	-41.40592622652	-41.40592622669	-41.40592622719	-41.40592622801	-41.40592622914
**41.470000**	-41.40592622311	-41.40592622328	-41.40592622377	-41.40592622458	-41.40592622568
**41.625714**	-41.40592621976	-41.40592621993	-41.40592622041	-41.40592622120	-41.40592622229
**41.781429**	-41.40592621648	-41.40592621664	-41.40592621711	-41.40592621790	-41.40592621896
**41.937143**	-41.40592621326	-41.40592621341	-41.40592621388	-41.40592621465	-41.40592621570
**42.092857**	-41.40592621009	-41.40592621025	-41.40592621070	-41.40592621146	-41.40592621249
**42.248571**	-41.40592620698	-41.40592620714	-41.40592620759	-41.40592620833	-41.40592620934
**42.404286**	-41.40592620394	-41.40592620409	-41.40592620453	-41.40592620526	-41.40592620625
**42.560000**	-41.40592620094	-41.40592620109	-41.40592620153	-41.40592620224	-41.40592620322
**42.715714**	-41.40592619800	-41.40592619815	-41.40592619858	-41.40592619928	-41.40592620024
**42.871429**	-41.40592619512	-41.40592619526	-41.40592619568	-41.40592619637	-41.40592619731
**43.027143**	-41.40592619229	-41.40592619243	-41.40592619284	-41.40592619352	-41.40592619444
**43.182857**	-41.40592618950	-41.40592618964	-41.40592619005	-41.40592619071	-41.40592619162
**43.338571**	-41.40592618677	-41.40592618691	-41.40592618730	-41.40592618796	-41.40592618885
**43.494286**	-41.40592618409	-41.40592618422	-41.40592618461	-41.40592618526	-41.40592618613
**43.650000**	-41.40592618145	-41.40592618158	-41.40592618197	-41.40592618260	-41.40592618346
**43.805714**	-41.40592617886	-41.40592617899	-41.40592617937	-41.40592617999	-41.40592618084
**43.961429**	-41.40592617632	-41.40592617644	-41.40592617682	-41.40592617743	-41.40592617826
**44.117143**	-41.40592617382	-41.40592617394	-41.40592617431	-41.40592617491	-41.40592617573
**44.272857**	-41.40592617136	-41.40592617149	-41.40592617185	-41.40592617244	-41.40592617325
**44.428571**	-41.40592616895	-41.40592616907	-41.40592616943	-41.40592617001	-41.40592617080
**44.584286**	-41.40592616658	-41.40592616670	-41.40592616705	-41.40592616762	-41.40592616840
**44.740000**	-41.40592616426	-41.40592616437	-41.40592616471	-41.40592616527	-41.40592616604
**44.895714**	-41.40592616197	-41.40592616208	-41.40592616242	-41.40592616297	-41.40592616372
**45.051429**	-41.40592615972	-41.40592615983	-41.40592616016	-41.40592616070	-41.40592616144
**45.207143**	-41.40592615751	-41.40592615761	-41.40592615794	-41.40592615847	-41.40592615920
**45.362857**	-41.40592615533	-41.40592615544	-41.40592615576	-41.40592615629	-41.40592615701
**45.518571**	-41.40592615320	-41.40592615330	-41.40592615362	-41.40592615414	-41.40592615484
**45.674286**	-41.40592615110	-41.40592615120	-41.40592615151	-41.40592615202	-41.40592615272

R (Bohr)/θ(º)	0.00	10.00	20.00	30.00	40.00
**45.830000**	-41.40592614903	-41.40592614914	-41.40592614944	-41.40592614994	-41.40592615063
**45.985714**	-41.40592614701	-41.40592614711	-41.40592614741	-41.40592614790	-41.40592614857
**46.141429**	-41.40592614501	-41.40592614511	-41.40592614541	-41.40592614589	-41.40592614655
**46.297143**	-41.40592614305	-41.40592614315	-41.40592614344	-41.40592614391	-41.40592614457
**46.452857**	-41.40592614112	-41.40592614122	-41.40592614151	-41.40592614197	-41.40592614261
**46.608571**	-41.40592613923	-41.40592613932	-41.40592613960	-41.40592614007	-41.40592614069
**46.764286**	-41.40592613736	-41.40592613746	-41.40592613773	-41.40592613819	-41.40592613881
**46.920000**	-41.40592613553	-41.40592613562	-41.40592613589	-41.40592613634	-41.40592613695
**47.075714**	-41.40592613373	-41.40592613382	-41.40592613408	-41.40592613452	-41.40592613513
**47.231429**	-41.40592613195	-41.40592613204	-41.40592613230	-41.40592613274	-41.40592613333
**47.387143**	-41.40592613021	-41.40592613030	-41.40592613056	-41.40592613098	-41.40592613156
**47.542857**	-41.40592612849	-41.40592612858	-41.40592612883	-41.40592612925	-41.40592612982
**47.698571**	-41.40592612680	-41.40592612689	-41.40592612714	-41.40592612755	-41.40592612811
**47.854286**	-41.40592612514	-41.40592612523	-41.40592612547	-41.40592612588	-41.40592612643
**48.010000**	-41.40592612351	-41.40592612359	-41.40592612384	-41.40592612423	-41.40592612478
**48.165714**	-41.40592612190	-41.40592612198	-41.40592612222	-41.40592612262	-41.40592612315
**48.321429**	-41.40592612032	-41.40592612040	-41.40592612064	-41.40592612102	-41.40592612155
**48.477143**	-41.40592611876	-41.40592611884	-41.40592611907	-41.40592611946	-41.40592611998
**48.632857**	-41.40592611723	-41.40592611731	-41.40592611754	-41.40592611791	-41.40592611843
**48.788571**	-41.40592611573	-41.40592611580	-41.40592611603	-41.40592611640	-41.40592611690
**48.944286**	-41.40592611424	-41.40592611432	-41.40592611454	-41.40592611490	-41.40592611540
**49.100000**	-41.40592611278	-41.40592611286	-41.40592611308	-41.40592611343	-41.40592611392
**49.255714**	-41.40592611135	-41.40592611142	-41.40592611163	-41.40592611199	-41.40592611247
**49.411429**	-41.40592610993	-41.40592611000	-41.40592611022	-41.40592611056	-41.40592611104
**49.567143**	-41.40592610854	-41.40592610861	-41.40592610882	-41.40592610916	-41.40592610963
**49.722857**	-41.40592610717	-41.40592610724	-41.40592610745	-41.40592610778	-41.40592610824
**49.878571**	-41.40592610582	-41.40592610589	-41.40592610609	-41.40592610643	-41.40592610688
**50.034286**	-41.40592610449	-41.40592610456	-41.40592610476	-41.40592610509	-41.40592610554
**50.190000**	-41.40592610319	-41.40592610325	-41.40592610345	-41.40592610377	-41.40592610421
**50.345714**	-41.40592610190	-41.40592610196	-41.40592610216	-41.40592610248	-41.40592610291
**50.501429**	-41.40592610063	-41.40592610070	-41.40592610089	-41.40592610120	-41.40592610163
**50.657143**	-41.40592609939	-41.40592609945	-41.40592609964	-41.40592609995	-41.40592610037
**50.812857**	-41.40592609816	-41.40592609822	-41.40592609841	-41.40592609871	-41.40592609913
**50.968571**	-41.40592609695	-41.40592609701	-41.40592609719	-41.40592609749	-41.40592609790
**51.124286**	-41.40592609576	-41.40592609582	-41.40592609600	-41.40592609629	-41.40592609670
**51.280000**	-41.40592609458	-41.40592609464	-41.40592609482	-41.40592609511	-41.40592609551
**51.435714**	-41.40592609343	-41.40592609349	-41.40592609366	-41.40592609395	-41.40592609434
**51.591429**	-41.40592609229	-41.40592609235	-41.40592609252	-41.40592609280	-41.40592609319
**51.747143**	-41.40592609117	-41.40592609123	-41.40592609140	-41.40592609167	-41.40592609205
**51.902857**	-41.40592609007	-41.40592609012	-41.40592609029	-41.40592609057	-41.40592609094
**52.058571**	-41.40592608897	-41.40592608903	-41.40592608920	-41.40592608947	-41.40592608984
**52.214286**	-41.40592608790	-41.40592608796	-41.40592608812	-41.40592608839	-41.40592608875
**52.370000**	-41.40592608685	-41.40592608690	-41.40592608706	-41.40592608733	-41.40592608768
**52.525714**	-41.40592608581	-41.40592608587	-41.40592608602	-41.40592608628	-41.40592608663
**52.681429**	-41.40592608479	-41.40592608484	-41.40592608500	-41.40592608525	-41.40592608560
**52.837143**	-41.40592608377	-41.40592608383	-41.40592608398	-41.40592608423	-41.40592608458
**52.992857**	-41.40592608278	-41.40592608283	-41.40592608298	-41.40592608323	-41.40592608357
**53.148571**	-41.40592608180	-41.40592608185	-41.40592608200	-41.40592608225	-41.40592608258
**53.304286**	-41.40592608084	-41.40592608089	-41.40592608103	-41.40592608127	-41.40592608160
**53.460000**	-41.40592607989	-41.40592607993	-41.40592608008	-41.40592608032	-41.40592608064
**53.615714**	-41.40592607895	-41.40592607899	-41.40592607914	-41.40592607937	-41.40592607969
**53.771429**	-41.40592607802	-41.40592607807	-41.40592607821	-41.40592607845	-41.40592607876
**53.927143**	-41.40592607711	-41.40592607716	-41.40592607730	-41.40592607753	-41.40592607784
**54.082857**	-41.40592607621	-41.40592607626	-41.40592607640	-41.40592607662	-41.40592607693
**54.238571**	-41.40592607533	-41.40592607537	-41.40592607551	-41.40592607573	-41.40592607603
**54.394286**	-41.40592607446	-41.40592607450	-41.40592607464	-41.40592607486	-41.40592607515
**54.550000**	-41.40592607360	-41.40592607364	-41.40592607378	-41.40592607399	-41.40592607428
**54.705714**	-41.40592607275	-41.40592607279	-41.40592607292	-41.40592607314	-41.40592607343
**54.861429**	-41.40592607192	-41.40592607196	-41.40592607209	-41.40592607230	-41.40592607258
**55.017143**	-41.40592607109	-41.40592607113	-41.40592607126	-41.40592607147	-41.40592607175
**55.172857**	-41.40592607028	-41.40592607032	-41.40592607045	-41.40592607065	-41.40592607093
**55.328571**	-41.40592606948	-41.40592606952	-41.40592606964	-41.40592606984	-41.40592607012
**55.484286**	-41.40592606869	-41.40592606873	-41.40592606885	-41.40592606905	-41.40592606932
**55.640000**	-41.40592606791	-41.40592606795	-41.40592606807	-41.40592606827	-41.40592606854
**55.795714**	-41.40592606714	-41.40592606719	-41.40592606730	-41.40592606750	-41.40592606776

R (Bohr)/θ(º)	0.00	10.00	20.00	30.00	40.00
**55.951429**	-41.40592606639	-41.40592606643	-41.40592606654	-41.40592606673	-41.40592606700
**56.107143**	-41.40592606564	-41.40592606568	-41.40592606580	-41.40592606599	-41.40592606624
**56.262857**	-41.40592606491	-41.40592606494	-41.40592606506	-41.40592606524	-41.40592606550
**56.418571**	-41.40592606418	-41.40592606422	-41.40592606433	-41.40592606451	-41.40592606476
**56.574286**	-41.40592606346	-41.40592606350	-41.40592606361	-41.40592606379	-41.40592606404
**56.730000**	-41.40592606276	-41.40592606280	-41.40592606290	-41.40592606308	-41.40592606333
**56.885714**	-41.40592606206	-41.40592606210	-41.40592606221	-41.40592606239	-41.40592606262
**57.041429**	-41.40592606137	-41.40592606141	-41.40592606152	-41.40592606169	-41.40592606193
**57.197143**	-41.40592606070	-41.40592606074	-41.40592606084	-41.40592606101	-41.40592606125
**57.352857**	-41.40592606003	-41.40592606007	-41.40592606017	-41.40592606034	-41.40592606057
**57.508571**	-41.40592605937	-41.40592605941	-41.40592605951	-41.40592605968	-41.40592605991
**57.664286**	-41.40592605872	-41.40592605876	-41.40592605886	-41.40592605902	-41.40592605925
**57.820000**	-41.40592605808	-41.40592605811	-41.40592605822	-41.40592605838	-41.40592605860
**57.975714**	-41.40592605745	-41.40592605748	-41.40592605758	-41.40592605775	-41.40592605796
**58.131429**	-41.40592605682	-41.40592605686	-41.40592605696	-41.40592605711	-41.40592605733
**58.287143**	-41.40592605621	-41.40592605624	-41.40592605633	-41.40592605649	-41.40592605671
**58.442857**	-41.40592605560	-41.40592605563	-41.40592605573	-41.40592605588	-41.40592605609
**58.598571**	-41.40592605500	-41.40592605503	-41.40592605513	-41.40592605528	-41.40592605549
**58.754286**	-41.40592605441	-41.40592605444	-41.40592605453	-41.40592605468	-41.40592605489
**58.910000**	-41.40592605382	-41.40592605386	-41.40592605395	-41.40592605410	-41.40592605430
**59.065714**	-41.40592605325	-41.40592605328	-41.40592605337	-41.40592605352	-41.40592605372
**59.221429**	-41.40592605268	-41.40592605271	-41.40592605280	-41.40592605295	-41.40592605315
**59.377143**	-41.40592605212	-41.40592605215	-41.40592605224	-41.40592605238	-41.40592605258
**59.532857**	-41.40592605157	-41.40592605159	-41.40592605168	-41.40592605183	-41.40592605202
**59.688571**	-41.40592605102	-41.40592605105	-41.40592605113	-41.40592605128	-41.40592605147
**59.844286**	-41.40592605048	-41.40592605051	-41.40592605069	-41.40592605073	-41.40592605092
**60.000000**	-41.40592604994	-41.40592604373	-41.40592604970	-41.40592604864	-41.40592604922

PES aV6Z – CH^- ($X\ ^3\Sigma^-$) + He					
**R (Bohr)/θ(º)**	**50.00**	**60.00**	**70.00**	**80.00**	**90.00**
**5.500000**	-41.40454967798	-41.40471124186	-41.40466374826	-41.40449398046	-41.40429731000
**5.655714**	-41.40488916605	-41.40499332941	-41.40494006533	-41.40479455729	-41.40463435000
**5.811429**	-41.40515267512	-41.40521500312	-41.40515982699	-41.40503635814	-41.40490602000
**5.967143**	-41.40535741498	-41.40538956691	-41.40533486399	-41.40523125743	-41.40512546000
**6.122857**	-41.40551662309	-41.40552723423	-41.40547452102	-41.40538858395	-41.40530295000
**6.278571**	-41.40564047465	-41.40563586854	-41.40558601973	-41.40551566483	-41.40544679000
**6.434286**	-41.40573675552	-41.40572164876	-41.40567508620	-41.40561828986	-41.40556346000
**6.590000**	-41.40581149922	-41.40578928335	-41.40574627448	-41.40570103174	-41.40565806000
**6.745714**	-41.40586927515	-41.40584253527	-41.40580314302	-41.40576759024	-41.40573463000
**6.901429**	-41.40591360684	-41.40588434309	-41.40584852893	-41.40582096889	-41.40579643000
**7.057143**	-41.40594731303	-41.40591653194	-41.40588467246	-41.40586361505	-41.40584610000
**7.212857**	-41.40597262678	-41.40594208960	-41.40591330954	-41.40589748591	-41.40588580000
**7.368571**	-41.40599133776	-41.40596202657	-41.40593592496	-41.40592418513	-41.40591725000
**7.524286**	-41.40600490361	-41.40597645090	-41.40595365151	-41.40594506942	-41.40594193000
**7.680000**	-41.40601445719	-41.40598789308	-41.40596738152	-41.40596120634	-41.40596110000
**7.835714**	-41.40602088893	-41.40599652684	-41.40597767318	-41.40597352183	-41.40597570000
**7.991429**	-41.40602487597	-41.40600178828	-41.40598572842	-41.40598273586	-41.40598659000
**8.147143**	-41.40602697529	-41.40600587763	-41.40599134506	-41.40598944455	-41.40599447000
**8.302857**	-41.40602759462	-41.40600858783	-41.40599544603	-41.40599413975	-41.40599990000
**8.458571**	-41.40602703936	-41.40600902451	-41.40599800984	-41.40599724318	-41.40600336000
**8.614286**	-41.40602558554	-41.40600917446	-41.40599953638	-41.40599905093	-41.40600526000
**8.770000**	-41.40602346543	-41.40600855116	-41.40600015892	-41.40599984972	-41.40600592000
**8.925714**	-41.40601984172	-41.40600734007	-41.40600006157	-41.40599942374	-41.40600562000
**9.081429**	-41.40601784450	-41.40600570087	-41.40599939369	-41.40599924016	-41.40600458000
**9.237143**	-41.40601369847	-41.40600374940	-41.40599827900	-41.40599783703	-41.40600300000
**9.392857**	-41.40601088253	-41.40600157017	-41.40599683819	-41.40599663868	-41.40600102000
**9.548571**	-41.40600631772	-41.40599887986	-41.40599514241	-41.40599464923	-41.40599823000
**9.704286**	-41.40600298138	-41.40599644277	-41.40599293548	-41.40599251285	-41.40599604000
**9.860000**	-41.40599983186	-41.40599392985	-41.40599089202	-41.40599051402	-41.40599308000
**10.015714**	-41.40599603596	-41.40599138413	-41.40598874938	-41.40598842021	-41.40599065000
**10.171429**	-41.40599151471	-41.40598883575	-41.40598655651	-41.40598629667	-41.40598822000
**10.327143**	-41.40598856659	-41.40598632921	-41.40598434348	-41.40598415324	-41.40598582000
**10.482857**	-41.40598570106	-41.40598342679	-41.40598213602	-41.40598200488	-41.40598346000
**10.638571**	-41.40598294261	-41.40598104605	-41.40597995200	-41.40597989662	-41.40598116000
**10.794286**	-41.40598028646	-41.40597872143	-41.40597782677	-41.40597791387	-41.40597902000
**10.950000**	-41.40597772801	-41.40597646196	-41.40597576164	-41.40597590575	-41.40597695000
**11.105714**	-41.40597526361	-41.40597427587	-41.40597375187	-41.40597394651	-41.40597494000
**11.261429**	-41.40597288794	-41.40597216713	-41.40597180256	-41.40597206512	-41.40597300000
**11.417143**	-41.40597070417	-41.40597013756	-41.40596992170	-41.40597023417	-41.40597114000
**11.572857**	-41.40596861992	-41.40596818538	-41.40596811545	-41.40596848029	-41.40596934000
**11.728571**	-41.40596663842	-41.40596631217	-41.40596638349	-41.40596680597	-41.40596763000
**11.884286**	-41.40596475794	-41.40596457367	-41.40596472607	-41.40596520548	-41.40596600000
**12.040000**	-41.40596297571	-41.40596292247	-41.40596313782	-41.40596367631	-41.40596445000
**12.195714**	-41.40596128621	-41.40596135599	-41.40596165335	-41.40596221368	-41.40596296000
**12.351429**	-41.40595968981	-41.40595986894	-41.40596024627	-41.40596083224	-41.40596154000
**12.507143**	-41.40595818296	-41.40595846354	-41.40595891312	-41.40595931981	-41.40595999592
**12.662857**	-41.40595676227	-41.40595713527	-41.40595765409	-41.40595787715	-41.40595849129
**12.818571**	-41.40595542205	-41.40595567741	-41.40595584605	-41.40595642446	-41.40595704494
**12.974286**	-41.40595394373	-41.40595431634	-41.40595449214	-41.40595506449	-41.40595565607
**13.130000**	-41.40595260119	-41.40595301346	-41.40595319459	-41.40595375888	-41.40595432369
**13.285714**	-41.40595132245	-41.40595176726	-41.40595195230	-41.40595250672	-41.40595304664
**13.441429**	-41.40595010515	-41.40595057616	-41.40595076400	-41.40595130694	-41.40595182363
**13.597143**	-41.40594894695	-41.40594943849	-41.40594962830	-41.40595015833	-41.40595065326
**13.752857**	-41.40594784550	-41.40594835254	-41.40594854373	-41.40594905956	-41.40594953406
**13.908571**	-41.40594679849	-41.40594731656	-41.40594750871	-41.40594800925	-41.40594846449
**14.064286**	-41.40594580364	-41.40594632879	-41.40594652166	-41.40594700593	-41.40594744297
**14.220000**	-41.40594485874	-41.40594538747	-41.40594558091	-41.40594604810	-41.40594646789
**14.375714**	-41.40594396158	-41.40594449082	-41.40594468482	-41.40594513424	-41.40594553762
**14.531429**	-41.40594311006	-41.40594363712	-41.40594383172	-41.40594426278	-41.40594465054
**14.687143**	-41.40594230210	-41.40594282462	-41.40594301994	-41.40594343220	-41.40594380503
**14.842857**	-41.40594153570	-41.40594205165	-41.40594224785	-41.40594264092	-41.40594299948
**14.998571**	-41.40594080893	-41.40594131653	-41.40594151380	-41.40594188744	-41.40594223231
**15.154286**	-41.40594003731	-41.40594061765	-41.40594081622	-41.40594117022	-41.40594150196
**15.310000**	-41.40593945578	-41.40593995343	-41.40594015352	-41.40594048780	-41.40594080690

R (Bohr)/θ(º)	50.00	60.00	70.00	80.00	90.00
**15.465714**	-41.40593890230	-41.40593932234	-41.40593952420	-41.40593983870	-41.40594014565
**15.621429**	-41.40593837528	-41.40593859342	-41.40593892675	-41.40593922151	-41.40593951675
**15.777143**	-41.40593787343	-41.40593808343	-41.40593833294	-41.40593863485	-41.40593891878
**15.932857**	-41.40593739539	-41.40593759739	-41.40593783581	-41.40593807964	-41.40593835039
**16.088571**	-41.40593693996	-41.40593713405	-41.40593736180	-41.40593759340	-41.40593784000
**16.244286**	-41.40593650595	-41.40593669226	-41.40593690971	-41.40593712963	-41.40593737000
**16.400000**	-41.40593609227	-41.40593627090	-41.40593647841	-41.40593668709	-41.40593691000
**16.555714**	-41.40593569782	-41.40593586893	-41.40593606681	-41.40593626470	-41.40593648000
**16.711429**	-41.40593532160	-41.40593548531	-41.40593567389	-41.40593586146	-41.40593607000
**16.867143**	-41.40593496264	-41.40593511910	-41.40593529868	-41.40593547651	-41.40593568000
**17.022857**	-41.40593462002	-41.40593476938	-41.40593494027	-41.40593510899	-41.40593530000
**17.178571**	-41.40593429287	-41.40593443531	-41.40593459778	-41.40593475812	-41.40593495000
**17.334286**	-41.40593398037	-41.40593411606	-41.40593427042	-41.40593442307	-41.40593460000
**17.490000**	-41.40593368174	-41.40593381087	-41.40593395744	-41.40593410306	-41.40593428000
**17.645714**	-41.40593339624	-41.40593351903	-41.40593365812	-41.40593379733	-41.40593396000
**17.801429**	-41.40593312318	-41.40593323985	-41.40593337181	-41.40593350513	-41.40593366000
**17.957143**	-41.40593286190	-41.40593297269	-41.40593309789	-41.40593322576	-41.40593338000
**18.112857**	-41.40593261178	-41.40593271694	-41.40593283576	-41.40593295857	-41.40593311000
**18.268571**	-41.40593237223	-41.40593247203	-41.40593258485	-41.40593270293	-41.40593284000
**18.424286**	-41.40593214269	-41.40593223740	-41.40593234462	-41.40593245824	-41.40593259000
**18.580000**	-41.40593192262	-41.40593201254	-41.40593211455	-41.40593222393	-41.40593235000
**18.735714**	-41.40593171149	-41.40593179692	-41.40593189411	-41.40593199947	-41.40593212000
**18.891429**	-41.40593150869	-41.40593159002	-41.40593168276	-41.40593178432	-41.40593190000
**19.047143**	-41.40593131291	-41.40593139111	-41.40593147992	-41.40593157792	-41.40593169000
**19.202857**	-41.40593110508	-41.40593119532	-41.40593128449	-41.40593137960	-41.40593149000
**19.358571**	-41.40593094548	-41.40593101139	-41.40593108682	-41.40593118645	-41.40593129000
**19.514286**	-41.40593077005	-41.40593083812	-41.40593091654	-41.40593100233	-41.40593109000
**19.670000**	-41.40593059984	-41.40593066613	-41.40593074242	-41.40593082834	-41.40593093000
**19.825714**	-41.40593044702	-41.40593050741	-41.40593057796	-41.40593065894	-41.40593075000
**19.981429**	-41.40593030031	-41.40593035941	-41.40593042639	-41.40593050192	-41.40593059000
**20.137143**	-41.40593015714	-41.40593021562	-41.40593028121	-41.40593035449	-41.40593044000
**20.292857**	-41.40593002067	-41.40593007313	-41.40593014061	-41.40593021193	-41.40593029000
**20.448571**	-41.40592989167	-41.40592994498	-41.40593000522	-41.40593007420	-41.40593015000
**20.604286**	-41.40592977008	-41.40592981998	-41.40592987766	-41.40592994305	-41.40593001000
**20.760000**	-41.40592965638	-41.40592970224	-41.40592975634	-41.40592981837	-41.40592989000
**20.915714**	-41.40592955036	-41.40592959183	-41.40592964206	-41.40592970049	-41.40592977000
**21.071429**	-41.40592945122	-41.40592948844	-41.40592953475	-41.40592958955	-41.40592965000
**21.227143**	-41.40592935785	-41.40592939135	-41.40592943402	-41.40592948537	-41.40592954000
**21.382857**	-41.40592926893	-41.40592929962	-41.40592933917	-41.40592938744	-41.40592944000
**21.538571**	-41.40592918332	-41.40592921213	-41.40592924928	-41.40592929502	-41.40592935000
**21.694286**	-41.40592910017	-41.40592912794	-41.40592916335	-41.40592920717	-41.40592926000
**21.850000**	-41.40592901901	-41.40592904625	-41.40592908051	-41.40592912294	-41.40592917000
**22.005714**	-41.40592893967	-41.40592896664	-41.40592900007	-41.40592904154	-41.40592909000
**22.161429**	-41.40592886218	-41.40592888887	-41.40592892160	-41.40592896236	-41.40592901000
**22.317143**	-41.40592878667	-41.40592881296	-41.40592884487	-41.40592888508	-41.40592893000
**22.472857**	-41.40592871339	-41.40592873903	-41.40592876985	-41.40592880955	-41.40592886000
**22.628571**	-41.40592864255	-41.40592866742	-41.40592869657	-41.40592873579	-41.40592878000
**22.784286**	-41.40592857402	-41.40592859867	-41.40592862520	-41.40592866393	-41.40592871000
**22.940000**	-41.40592850537	-41.40592853338	-41.40592855617	-41.40592859442	-41.40592864000
**23.095714**	-41.40592843343	-41.40592846576	-41.40592849170	-41.40592852864	-41.40592858000
**23.251429**	-41.40592838121	-41.40592840447	-41.40592842728	-41.40592847309	-41.40592851000
**23.407143**	-41.40592832068	-41.40592834437	-41.40592836531	-41.40592840380	-41.40592843000
**23.562857**	-41.40592825222	-41.40592827391	-41.40592829674	-41.40592832922	-41.40592837000
**23.718571**	-41.40592818802	-41.40592820998	-41.40592823489	-41.40592826563	-41.40592831000
**23.874286**	-41.40592812799	-41.40592815020	-41.40592817579	-41.40592820562	-41.40592824000
**24.030000**	-41.40592807104	-41.40592809319	-41.40592811877	-41.40592814781	-41.40592818000
**24.185714**	-41.40592801651	-41.40592803839	-41.40592806363	-41.40592809187	-41.40592812000
**24.341429**	-41.40592796406	-41.40592798553	-41.40592801026	-41.40592803771	-41.40592807000
**24.497143**	-41.40592791346	-41.40592793446	-41.40592795860	-41.40592798525	-41.40592801000
**24.652857**	-41.40592786457	-41.40592788506	-41.40592790858	-41.40592793445	-41.40592796000
**24.808571**	-41.40592781730	-41.40592783725	-41.40592786013	-41.40592788523	-41.40592791000
**24.964286**	-41.40592777155	-41.40592779096	-41.40592781320	-41.40592783756	-41.40592786000
**25.120000**	-41.40592772726	-41.40592774613	-41.40592776774	-41.40592779138	-41.40592782000
**25.275714**	-41.40592768436	-41.40592770271	-41.40592772369	-41.40592774662	-41.40592777000
**25.431429**	-41.40592764281	-41.40592766064	-41.40592768100	-41.40592770326	-41.40592773000

R (Bohr)/θ(º)	50.00	60.00	70.00	80.00	90.00
**25.587143**	-41.40592760254	-41.40592761986	-41.40592763963	-41.40592766123	-41.40592768000
**25.742857**	-41.40592756351	-41.40592758033	-41.40592759953	-41.40592762048	-41.40592764000
**25.898571**	-41.40592752566	-41.40592754200	-41.40592756065	-41.40592758098	-41.40592760000
**26.054286**	-41.40592748896	-41.40592750484	-41.40592752294	-41.40592754267	-41.40592756000
**26.210000**	-41.40592745337	-41.40592746879	-41.40592748637	-41.40592750552	-41.40592753000
**26.365714**	-41.40592741883	-41.40592743382	-41.40592745089	-41.40592746949	-41.40592749000
**26.521429**	-41.40592738532	-41.40592739988	-41.40592741647	-41.40592743453	-41.40592745000
**26.677143**	-41.40592735279	-41.40592736695	-41.40592738306	-41.40592740060	-41.40592742000
**26.832857**	-41.40592732122	-41.40592733498	-41.40592735064	-41.40592736767	-41.40592739000
**26.988571**	-41.40592729057	-41.40592730395	-41.40592731916	-41.40592733571	-41.40592735000
**27.144286**	-41.40592726079	-41.40592727380	-41.40592728859	-41.40592730468	-41.40592732000
**27.300000**	-41.40592723186	-41.40592724453	-41.40592725891	-41.40592727454	-41.40592729000
**27.455714**	-41.40592720373	-41.40592721608	-41.40592723008	-41.40592724527	-41.40592726000
**27.611429**	-41.40592717620	-41.40592718841	-41.40592720205	-41.40592721683	-41.40592723000
**27.767143**	-41.40592711461	-41.40592716097	-41.40592717477	-41.40592718920	-41.40592720000
**27.922857**	-41.40592712403	-41.40592713501	-41.40592713166	-41.40592716226	-41.40592718000
**28.078571**	-41.40592709990	-41.40592711224	-41.40592712472	-41.40592713836	-41.40592715000
**28.234286**	-41.40592707558	-41.40592708762	-41.40592709979	-41.40592711297	-41.40592713000
**28.390000**	-41.40592705166	-41.40592706366	-41.40592707525	-41.40592708810	-41.40592710000
**28.545714**	-41.40592702863	-41.40592704035	-41.40592705029	-41.40592706412	-41.40592708000
**28.701429**	-41.40592700623	-41.40592701744	-41.40592702731	-41.40592704056	-41.40592705000
**28.857143**	-41.40592698443	-41.40592699514	-41.40592700496	-41.40592701789	-41.40592703000
**29.012857**	-41.40592696298	-41.40592697368	-41.40592698321	-41.40592699582	-41.40592701000
**29.168571**	-41.40592694210	-41.40592695256	-41.40592696290	-41.40592697436	-41.40592698000
**29.324286**	-41.40592692201	-41.40592693223	-41.40592694231	-41.40592695189	-41.40592696000
**29.480000**	-41.40592690245	-41.40592691112	-41.40592692226	-41.40592693156	-41.40592694000
**29.635714**	-41.40592688341	-41.40592689186	-41.40592690141	-41.40592691177	-41.40592692000
**29.791429**	-41.40592686487	-41.40592687310	-41.40592688241	-41.40592689251	-41.40592690000
**29.947143**	-41.40592684682	-41.40592685484	-41.40592686391	-41.40592687374	-41.40592688000
**30.102857**	-41.40592682923	-41.40592683705	-41.40592684590	-41.40592685548	-41.40592687000
**30.258571**	-41.40592681210	-41.40592681972	-41.40592682834	-41.40592683768	-41.40592685000
**30.414286**	-41.40592679541	-41.40592680284	-41.40592681124	-41.40592682035	-41.40592683000
**30.570000**	-41.40592677914	-41.40592678639	-41.40592679459	-41.40592680346	-41.40592681000
**30.725714**	-41.40592676329	-41.40592677036	-41.40592677835	-41.40592678700	-41.40592680000
**30.881429**	-41.40592674785	-41.40592675474	-41.40592676253	-41.40592677097	-41.40592678000
**31.037143**	-41.40592673278	-41.40592673951	-41.40592674711	-41.40592675534	-41.40592676000
**31.192857**	-41.40592671810	-41.40592672466	-41.40592673208	-41.40592674010	-41.40592675000
**31.348571**	-41.40592670378	-41.40592671019	-41.40592671742	-41.40592672524	-41.40592673000
**31.504286**	-41.40592668982	-41.40592669607	-41.40592670313	-41.40592671076	-41.40592672000
**31.660000**	-41.40592667620	-41.40592668230	-41.40592668919	-41.40592669664	-41.40592670000
**31.815714**	-41.40592666292	-41.40592666888	-41.40592667560	-41.40592668286	-41.40592669000
**31.971429**	-41.40592664996	-41.40592665578	-41.40592666233	-41.40592666943	-41.40592668000
**32.127143**	-41.40592663732	-41.40592664300	-41.40592664940	-41.40592665632	-41.40592666000
**32.282857**	-41.40592662498	-41.40592663052	-41.40592663677	-41.40592664353	-41.40592665000
**32.438571**	-41.40592661294	-41.40592661835	-41.40592662446	-41.40592663106	-41.40592664000
**32.594286**	-41.40592660118	-41.40592660647	-41.40592661243	-41.40592661888	-41.40592663000
**32.750000**	-41.40592658971	-41.40592659488	-41.40592660070	-41.40592660699	-41.40592661000
**32.905714**	-41.40592657851	-41.40592658356	-41.40592658925	-41.40592659539	-41.40592660000
**33.061429**	-41.40592656758	-41.40592657251	-41.40592657806	-41.40592658407	-41.40592659000
**33.217143**	-41.40592655690	-41.40592656171	-41.40592656714	-41.40592657301	-41.40592658000
**33.372857**	-41.40592654647	-41.40592655118	-41.40592655648	-41.40592656221	-41.40592657000
**33.528571**	-41.40592653628	-41.40592654088	-41.40592654606	-41.40592655166	-41.40592656000
**33.684286**	-41.40592652633	-41.40592653083	-41.40592653589	-41.40592654136	-41.40592655000
**33.840000**	-41.40592651661	-41.40592652101	-41.40592652596	-41.40592653130	-41.40592654000
**33.995714**	-41.40592650711	-41.40592651141	-41.40592651625	-41.40592652147	-41.40592653000
**34.151429**	-41.40592649783	-41.40592650203	-41.40592650677	-41.40592651187	-41.40592652000
**34.307143**	-41.40592648876	-41.40592649287	-41.40592649750	-41.40592650249	-41.40592651000
**34.462857**	-41.40592647989	-41.40592648392	-41.40592648844	-41.40592649332	-41.40592650000
**34.618571**	-41.40592647123	-41.40592647516	-41.40592647959	-41.40592648436	-41.40592649000
**34.774286**	-41.40592646276	-41.40592646661	-41.40592647093	-41.40592647560	-41.40592648000
**34.930000**	-41.40592645448	-41.40592645824	-41.40592646247	-41.40592646704	-41.40592647000
**35.085714**	-41.40592644638	-41.40592645006	-41.40592645420	-41.40592645867	-41.40592646000
**35.241429**	-41.40592643846	-41.40592644206	-41.40592644611	-41.40592645048	-41.40592646000
**35.397143**	-41.40592643072	-41.40592643424	-41.40592643821	-41.40592644248	-41.40592645000
**35.552857**	-41.40592642314	-41.40592642660	-41.40592643047	-41.40592643465	-41.40592644000

R (Bohr)/θ(º)	50.00	60.00	70.00	80.00	90.00
**35.708571**	-41.40592641573	-41.40592641911	-41.40592642291	-41.40592642700	-41.40592643000
**35.864286**	-41.40592640849	-41.40592641179	-41.40592641551	-41.40592641951	-41.40592642000
**36.020000**	-41.40592640140	-41.40592640463	-41.40592640827	-41.40592641218	-41.40592642000
**36.175714**	-41.40592639446	-41.40592639763	-41.40592640118	-41.40592640502	-41.40592641000
**36.331429**	-41.40592638767	-41.40592639077	-41.40592639425	-41.40592639801	-41.40592640000
**36.487143**	-41.40592638103	-41.40592638406	-41.40592638747	-41.40592639115	-41.40592639000
**36.642857**	-41.40592637452	-41.40592637750	-41.40592638084	-41.40592638443	-41.40592639000
**36.798571**	-41.40592636816	-41.40592637107	-41.40592637434	-41.40592637786	-41.40592638000
**36.954286**	-41.40592636193	-41.40592636478	-41.40592636798	-41.40592637144	-41.40592638000
**37.110000**	-41.40592635583	-41.40592635862	-41.40592636176	-41.40592636514	-41.40592637000
**37.265714**	-41.40592634986	-41.40592635259	-41.40592635567	-41.40592635897	-41.40592636000
**37.421429**	-41.40592634401	-41.40592634669	-41.40592634970	-41.40592635294	-41.40592636000
**37.577143**	-41.40592633828	-41.40592634091	-41.40592634386	-41.40592634704	-41.40592635000
**37.732857**	-41.40592633267	-41.40592633525	-41.40592633814	-41.40592634125	-41.40592634000
**37.888571**	-41.40592632718	-41.40592632971	-41.40592633254	-41.40592633559	-41.40592634000
**38.044286**	-41.40592632180	-41.40592632428	-41.40592632705	-41.40592633003	-41.40592633000
**38.200000**	-41.40592631653	-41.40592631896	-41.40592632167	-41.40592632460	-41.40592633000
**38.355714**	-41.40592631137	-41.40592631375	-41.40592631641	-41.40592631928	-41.40592632000
**38.511429**	-41.40592630631	-41.40592630864	-41.40592631125	-41.40592631406	-41.40592632000
**38.667143**	-41.40592630135	-41.40592630364	-41.40592630620	-41.40592630895	-41.40592631000
**38.822857**	-41.40592629650	-41.40592629874	-41.40592630124	-41.40592630394	-41.40592631000
**38.978571**	-41.40592629174	-41.40592629393	-41.40592629639	-41.40592629904	-41.40592630000
**39.134286**	-41.40592628707	-41.40592628923	-41.40592629163	-41.40592629423	-41.40592630000
**39.290000**	-41.40592628250	-41.40592628461	-41.40592628698	-41.40592628952	-41.40592629000
**39.445714**	-41.40592627802	-41.40592628009	-41.40592628241	-41.40592628490	-41.40592629000
**39.601429**	-41.40592627362	-41.40592627565	-41.40592627793	-41.40592628037	-41.40592628000
**39.757143**	-41.40592626931	-41.40592627130	-41.40592627354	-41.40592627594	-41.40592628000
**39.912857**	-41.40592626509	-41.40592626704	-41.40592626923	-41.40592627158	-41.40592627000
**40.068571**	-41.40592626095	-41.40592626286	-41.40592626501	-41.40592626732	-41.40592627000
**40.224286**	-41.40592625688	-41.40592625877	-41.40592626087	-41.40592626314	-41.40592627000
**40.380000**	-41.40592625290	-41.40592625475	-41.40592625682	-41.40592625904	-41.40592626000
**40.535714**	-41.40592624899	-41.40592625081	-41.40592625283	-41.40592625501	-41.40592626000
**40.691429**	-41.40592624516	-41.40592624694	-41.40592624893	-41.40592625107	-41.40592625000
**40.847143**	-41.40592624141	-41.40592624315	-41.40592624510	-41.40592624720	-41.40592625000
**41.002857**	-41.40592623772	-41.40592623943	-41.40592624135	-41.40592624341	-41.40592625000
**41.158571**	-41.40592623410	-41.40592623578	-41.40592623766	-41.40592623969	-41.40592624000
**41.314286**	-41.40592623055	-41.40592623220	-41.40592623405	-41.40592623603	-41.40592624000
**41.470000**	-41.40592622707	-41.40592622869	-41.40592623050	-41.40592623245	-41.40592623000
**41.625714**	-41.40592622365	-41.40592622524	-41.40592622702	-41.40592622893	-41.40592623000
**41.781429**	-41.40592622030	-41.40592622186	-41.40592622361	-41.40592622548	-41.40592623000
**41.937143**	-41.40592621701	-41.40592621854	-41.40592622025	-41.40592622210	-41.40592622000
**42.092857**	-41.40592621378	-41.40592621528	-41.40592621697	-41.40592621877	-41.40592622000
**42.248571**	-41.40592621060	-41.40592621208	-41.40592621374	-41.40592621552	-41.40592622000
**42.404286**	-41.40592620749	-41.40592620894	-41.40592621057	-41.40592621231	-41.40592621000
**42.560000**	-41.40592620443	-41.40592620586	-41.40592620746	-41.40592620917	-41.40592621000
**42.715714**	-41.40592620143	-41.40592620284	-41.40592620440	-41.40592620609	-41.40592621000
**42.871429**	-41.40592619849	-41.40592619987	-41.40592620140	-41.40592620306	-41.40592620000
**43.027143**	-41.40592619560	-41.40592619695	-41.40592619846	-41.40592620008	-41.40592620000
**43.182857**	-41.40592619276	-41.40592619409	-41.40592619557	-41.40592619717	-41.40592620000
**43.338571**	-41.40592618997	-41.40592619127	-41.40592619273	-41.40592619430	-41.40592620000
**43.494286**	-41.40592618723	-41.40592618851	-41.40592618995	-41.40592619148	-41.40592619000
**43.650000**	-41.40592618454	-41.40592618580	-41.40592618721	-41.40592618872	-41.40592619000
**43.805714**	-41.40592618190	-41.40592618314	-41.40592618452	-41.40592618601	-41.40592619000
**43.961429**	-41.40592617930	-41.40592618052	-41.40592618188	-41.40592618334	-41.40592618000
**44.117143**	-41.40592617676	-41.40592617795	-41.40592617928	-41.40592618072	-41.40592618000
**44.272857**	-41.40592617425	-41.40592617542	-41.40592617674	-41.40592617815	-41.40592618000
**44.428571**	-41.40592617179	-41.40592617294	-41.40592617424	-41.40592617562	-41.40592618000
**44.584286**	-41.40592616937	-41.40592617051	-41.40592617178	-41.40592617314	-41.40592617000
**44.740000**	-41.40592616700	-41.40592616811	-41.40592616936	-41.40592617070	-41.40592617000
**44.895714**	-41.40592616466	-41.40592616576	-41.40592616699	-41.40592616830	-41.40592617000
**45.051429**	-41.40592616237	-41.40592616345	-41.40592616466	-41.40592616595	-41.40592617000
**45.207143**	-41.40592616011	-41.40592616118	-41.40592616236	-41.40592616363	-41.40592616000
**45.362857**	-41.40592615790	-41.40592615894	-41.40592616011	-41.40592616136	-41.40592616000
**45.518571**	-41.40592615572	-41.40592615675	-41.40592615789	-41.40592615912	-41.40592616000
**45.674286**	-41.40592615358	-41.40592615459	-41.40592615572	-41.40592615693	-41.40592616000

R (Bohr)/θ(º)	50.00	60.00	70.00	80.00	90.00
**45.830000**	-41.40592615148	-41.40592615247	-41.40592615358	-41.40592615476	-41.40592616000
**45.985714**	-41.40592614941	-41.40592615038	-41.40592615148	-41.40592615264	-41.40592615000
**46.141429**	-41.40592614737	-41.40592614834	-41.40592614941	-41.40592615056	-41.40592615000
**46.297143**	-41.40592614537	-41.40592614632	-41.40592614738	-41.40592614850	-41.40592615000
**46.452857**	-41.40592614341	-41.40592614434	-41.40592614537	-41.40592614649	-41.40592615000
**46.608571**	-41.40592614148	-41.40592614239	-41.40592614341	-41.40592614451	-41.40592615000
**46.764286**	-41.40592613958	-41.40592614047	-41.40592614148	-41.40592614256	-41.40592614000
**46.920000**	-41.40592613771	-41.40592613859	-41.40592613958	-41.40592614064	-41.40592614000
**47.075714**	-41.40592613587	-41.40592613674	-41.40592613771	-41.40592613875	-41.40592614000
**47.231429**	-41.40592613406	-41.40592613492	-41.40592613587	-41.40592613690	-41.40592614000
**47.387143**	-41.40592613228	-41.40592613313	-41.40592613407	-41.40592613508	-41.40592614000
**47.542857**	-41.40592613054	-41.40592613136	-41.40592613229	-41.40592613328	-41.40592613000
**47.698571**	-41.40592612881	-41.40592612963	-41.40592613054	-41.40592613152	-41.40592613000
**47.854286**	-41.40592612712	-41.40592612793	-41.40592612882	-41.40592612978	-41.40592613000
**48.010000**	-41.40592612546	-41.40592612625	-41.40592612713	-41.40592612808	-41.40592613000
**48.165714**	-41.40592612382	-41.40592612460	-41.40592612547	-41.40592612640	-41.40592613000
**48.321429**	-41.40592612221	-41.40592612297	-41.40592612383	-41.40592612475	-41.40592613000
**48.477143**	-41.40592612062	-41.40592612138	-41.40592612222	-41.40592612312	-41.40592612000
**48.632857**	-41.40592611906	-41.40592611981	-41.40592612063	-41.40592612152	-41.40592612000
**48.788571**	-41.40592611753	-41.40592611826	-41.40592611908	-41.40592611995	-41.40592612000
**48.944286**	-41.40592611602	-41.40592611674	-41.40592611754	-41.40592611840	-41.40592612000
**49.100000**	-41.40592611453	-41.40592611524	-41.40592611603	-41.40592611688	-41.40592612000
**49.255714**	-41.40592611307	-41.40592611377	-41.40592611455	-41.40592611538	-41.40592612000
**49.411429**	-41.40592611163	-41.40592611232	-41.40592611308	-41.40592611390	-41.40592611000
**49.567143**	-41.40592611021	-41.40592611089	-41.40592611164	-41.40592611245	-41.40592611000
**49.722857**	-41.40592610881	-41.40592610948	-41.40592611023	-41.40592611102	-41.40592611000
**49.878571**	-41.40592610744	-41.40592610810	-41.40592610883	-41.40592610961	-41.40592611000
**50.034286**	-41.40592610609	-41.40592610674	-41.40592610746	-41.40592610823	-41.40592611000
**50.190000**	-41.40592610476	-41.40592610540	-41.40592610611	-41.40592610687	-41.40592611000
**50.345714**	-41.40592610345	-41.40592610408	-41.40592610478	-41.40592610553	-41.40592611000
**50.501429**	-41.40592610216	-41.40592610278	-41.40592610347	-41.40592610420	-41.40592610000
**50.657143**	-41.40592610089	-41.40592610150	-41.40592610218	-41.40592610290	-41.40592610000
**50.812857**	-41.40592609964	-41.40592610024	-41.40592610091	-41.40592610162	-41.40592610000
**50.968571**	-41.40592609841	-41.40592609900	-41.40592609966	-41.40592610036	-41.40592610000
**51.124286**	-41.40592609720	-41.40592609778	-41.40592609843	-41.40592609912	-41.40592610000
**51.280000**	-41.40592609600	-41.40592609657	-41.40592609721	-41.40592609790	-41.40592610000
**51.435714**	-41.40592609482	-41.40592609539	-41.40592609602	-41.40592609670	-41.40592610000
**51.591429**	-41.40592609367	-41.40592609422	-41.40592609484	-41.40592609551	-41.40592610000
**51.747143**	-41.40592609252	-41.40592609307	-41.40592609369	-41.40592609434	-41.40592610000
**51.902857**	-41.40592609140	-41.40592609194	-41.40592609255	-41.40592609319	-41.40592609000
**52.058571**	-41.40592609029	-41.40592609083	-41.40592609142	-41.40592609206	-41.40592609000
**52.214286**	-41.40592608921	-41.40592608973	-41.40592609031	-41.40592609094	-41.40592609000
**52.370000**	-41.40592608813	-41.40592608865	-41.40592608923	-41.40592608984	-41.40592609000
**52.525714**	-41.40592608707	-41.40592608758	-41.40592608815	-41.40592608876	-41.40592609000
**52.681429**	-41.40592608603	-41.40592608653	-41.40592608709	-41.40592608770	-41.40592609000
**52.837143**	-41.40592608500	-41.40592608550	-41.40592608605	-41.40592608664	-41.40592609000
**52.992857**	-41.40592608399	-41.40592608448	-41.40592608502	-41.40592608561	-41.40592609000
**53.148571**	-41.40592608299	-41.40592608348	-41.40592608401	-41.40592608459	-41.40592609000
**53.304286**	-41.40592608201	-41.40592608249	-41.40592608302	-41.40592608359	-41.40592608000
**53.460000**	-41.40592608105	-41.40592608151	-41.40592608204	-41.40592608259	-41.40592608000
**53.615714**	-41.40592608009	-41.40592608055	-41.40592608107	-41.40592608162	-41.40592608000
**53.771429**	-41.40592607915	-41.40592607961	-41.40592608011	-41.40592608066	-41.40592608000
**53.927143**	-41.40592607822	-41.40592607867	-41.40592607917	-41.40592607971	-41.40592608000
**54.082857**	-41.40592607731	-41.40592607775	-41.40592607825	-41.40592607878	-41.40592608000
**54.238571**	-41.40592607641	-41.40592607685	-41.40592607733	-41.40592607785	-41.40592608000
**54.394286**	-41.40592607552	-41.40592607596	-41.40592607643	-41.40592607695	-41.40592608000
**54.550000**	-41.40592607465	-41.40592607507	-41.40592607555	-41.40592607605	-41.40592608000
**54.705714**	-41.40592607379	-41.40592607421	-41.40592607467	-41.40592607517	-41.40592608000
**54.861429**	-41.40592607294	-41.40592607335	-41.40592607381	-41.40592607431	-41.40592607000
**55.017143**	-41.40592607210	-41.40592607251	-41.40592607296	-41.40592607345	-41.40592607000
**55.172857**	-41.40592607127	-41.40592607167	-41.40592607212	-41.40592607260	-41.40592607000
**55.328571**	-41.40592607046	-41.40592607086	-41.40592607130	-41.40592607177	-41.40592607000
**55.484286**	-41.40592606966	-41.40592607005	-41.40592607049	-41.40592607095	-41.40592607000
**55.640000**	-41.40592606887	-41.40592606925	-41.40592606968	-41.40592607014	-41.40592607000
**55.795714**	-41.40592606809	-41.40592606847	-41.40592606889	-41.40592606935	-41.40592607000

R (Bohr)/θ(º)	50.00	60.00	70.00	80.00	90.00
**55.951429**	-41.40592606732	-41.40592606770	-41.40592606811	-41.40592606856	-41.40592607000
**56.107143**	-41.40592606656	-41.40592606693	-41.40592606734	-41.40592606778	-41.40592607000
**56.262857**	-41.40592606581	-41.40592606618	-41.40592606659	-41.40592606702	-41.40592607000
**56.418571**	-41.40592606507	-41.40592606544	-41.40592606584	-41.40592606627	-41.40592607000
**56.574286**	-41.40592606435	-41.40592606470	-41.40592606510	-41.40592606552	-41.40592607000
**56.730000**	-41.40592606363	-41.40592606398	-41.40592606437	-41.40592606479	-41.40592607000
**56.885714**	-41.40592606293	-41.40592606327	-41.40592606366	-41.40592606407	-41.40592606000
**57.041429**	-41.40592606223	-41.40592606257	-41.40592606295	-41.40592606336	-41.40592606000
**57.197143**	-41.40592606154	-41.40592606188	-41.40592606225	-41.40592606265	-41.40592606000
**57.352857**	-41.40592606086	-41.40592606119	-41.40592606156	-41.40592606196	-41.40592606000
**57.508571**	-41.40592606019	-41.40592606052	-41.40592606088	-41.40592606128	-41.40592606000
**57.664286**	-41.40592605953	-41.40592605985	-41.40592606021	-41.40592606060	-41.40592606000
**57.820000**	-41.40592605888	-41.40592605920	-41.40592605955	-41.40592605993	-41.40592606000
**57.975714**	-41.40592605823	-41.40592605855	-41.40592605890	-41.40592605928	-41.40592606000
**58.131429**	-41.40592605760	-41.40592605791	-41.40592605826	-41.40592605863	-41.40592606000
**58.287143**	-41.40592605698	-41.40592605728	-41.40592605763	-41.40592605799	-41.40592606000
**58.442857**	-41.40592605636	-41.40592605666	-41.40592605700	-41.40592605736	-41.40592606000
**58.598571**	-41.40592605575	-41.40592605605	-41.40592605638	-41.40592605674	-41.40592606000
**58.754286**	-41.40592605515	-41.40592605544	-41.40592605577	-41.40592605613	-41.40592606000
**58.910000**	-41.40592605455	-41.40592605484	-41.40592605517	-41.40592605552	-41.40592606000
**59.065714**	-41.40592605397	-41.40592605426	-41.40592605458	-41.40592605492	-41.40592606000
**59.221429**	-41.40592605339	-41.40592605368	-41.40592605400	-41.40592605433	-41.40592605000
**59.377143**	-41.40592605282	-41.40592605310	-41.40592605342	-41.40592605375	-41.40592605000
**59.532857**	-41.40592605226	-41.40592605254	-41.40592605285	-41.40592605318	-41.40592605000
**59.688571**	-41.40592605170	-41.40592605198	-41.40592605228	-41.40592605261	-41.40592605000
**59.844286**	-41.40592605116	-41.40592605143	-41.40592605173	-41.40592605205	-41.40592605000
**60.000000**	-41.40592605060	-41.40592605085	-41.40592605118	-41.40592605149	-41.40592603000

PES aV6Z – CH^- (X $^3\Sigma^-$) + He					
**R (Bohr)/θ(º)**	**100.00**	**110.00**	**120.00**	**130.00**	**140.00**
**5.500000**	-41.40412939452	-41.40401700051	-41.40396382456	-41.40395657940	-41.40397391518
**5.655714**	-41.40450478291	-41.40442573848	-41.40439802269	-41.40440859068	-41.40443794076
**5.811429**	-41.40480660910	-41.40475298614	-41.40474391710	-41.40476691538	-41.40480429158
**5.967143**	-41.40504958306	-41.40501508390	-41.40501934286	-41.40505056997	-41.40509278706
**6.122857**	-41.40524554074	-41.40522518995	-41.40523862601	-41.40527484507	-41.40531950362
**6.278571**	-41.40540383577	-41.40539384416	-41.40541329230	-41.40545210931	-41.40549741485
**6.434286**	-41.40553182722	-41.40552940932	-41.40555251305	-41.40559215248	-41.40563679897
**6.590000**	-41.40563541268	-41.40563836579	-41.40566353600	-41.40570273929	-41.40574588447
**6.745714**	-41.40571919368	-41.40572597032	-41.40575198249	-41.40578992668	-41.40583097110
**6.901429**	-41.40578682491	-41.40579629475	-41.40582232736	-41.40585850379	-41.40589710120
**7.057143**	-41.40584120918	-41.40585255393	-41.40587808653	-41.40591216821	-41.40594813206
**7.212857**	-41.40588470459	-41.40589731097	-41.40592202464	-41.40595390195	-41.40598720090
**7.368571**	-41.40591922213	-41.40593270787	-41.40595638774	-41.40598602680	-41.40601669828
**7.524286**	-41.40594633284	-41.40596035839	-41.40598289143	-41.40601035123	-41.40603851948
**7.680000**	-41.40596735967	-41.40598165365	-41.40600299437	-41.40602837771	-41.40605417579
**7.835714**	-41.40598338657	-41.40599771431	-41.40601788135	-41.40604131841	-41.40606492082
**7.991429**	-41.40599530121	-41.40600950490	-41.40602852840	-41.40605014220	-41.40607170878
**8.147143**	-41.40600385032	-41.40601782323	-41.40603572087	-41.40605564797	-41.40607532959
**8.302857**	-41.40600968550	-41.40602330604	-41.40604011761	-41.40605847364	-41.40607641349
**8.458571**	-41.40601329246	-41.40602648115	-41.40604224604	-41.40605914425	-41.40607548501
**8.614286**	-41.40601511405	-41.40602779601	-41.40604255184	-41.40605809721	-41.40607296234
**8.770000**	-41.40601551565	-41.40602762529	-41.40604140234	-41.40605569026	-41.40606920206
**8.925714**	-41.40601481849	-41.40602628213	-41.40603910776	-41.40605222579	-41.40606450435
**9.081429**	-41.40601326884	-41.40602403998	-41.40603593639	-41.40604796382	-41.40605911835
**9.237143**	-41.40601109045	-41.40602113001	-41.40603211645	-41.40604312726	-41.40605324212
**9.392857**	-41.40600847021	-41.40601775099	-41.40602784618	-41.40603761062	-41.40604420157
**9.548571**	-41.40600555610	-41.40601406577	-41.40602300345	-41.40603216743	-41.40603605849
**9.704286**	-41.40600218936	-41.40600993423	-41.40601832230	-41.40602663233	-41.40602713474
**9.860000**	-41.40599836447	-41.40600507293	-41.40601245969	-41.40601896341	-41.40602142696
**10.015714**	-41.40599589523	-41.40600213261	-41.40600895872	-41.40601324047	-41.40601594968
**10.171429**	-41.40599217870	-41.40599758782	-41.40600343166	-41.40600595330	-41.40601074037
**10.327143**	-41.40598927269	-41.40599466355	-41.40599894287	-41.40600160557	-41.40600582840
**10.482857**	-41.40598645836	-41.40599006095	-41.40599338942	-41.40599747329	-41.40600121978
**10.638571**	-41.40598381486	-41.40598732489	-41.40599000448	-41.40599359229	-41.40599690752
**10.794286**	-41.40598106849	-41.40598374081	-41.40598680678	-41.40598995819	-41.40599289168
**10.950000**	-41.40597876898	-41.40598111142	-41.40598379240	-41.40598655817	-41.40598915180
**11.105714**	-41.40597657107	-41.40597863128	-41.40598096848	-41.40598336300	-41.40598567071
**11.261429**	-41.40597447394	-41.40597629211	-41.40597829256	-41.40598040288	-41.40598244531
**11.417143**	-41.40597247290	-41.40597405699	-41.40597579604	-41.40597765429	-41.40597946912
**11.572857**	-41.40597055209	-41.40597193694	-41.40597346558	-41.40597509381	-41.40597672112
**11.728571**	-41.40596872064	-41.40596993517	-41.40597127638	-41.40597270943	-41.40597418385
**11.884286**	-41.40596697676	-41.40596804970	-41.40596922165	-41.40597049060	-41.40597181811
**12.040000**	-41.40596532270	-41.40596627303	-41.40596729759	-41.40596842605	-41.40596961992
**12.195714**	-41.40596375566	-41.40596459335	-41.40596549896	-41.40596650888	-41.40596757104
**12.351429**	-41.40596227031	-41.40596300997	-41.40596381547	-41.40596470099	-41.40596566082
**12.507143**	-41.40596086188	-41.40596151900	-41.40596222517	-41.40596301167	-41.40596344474
**12.662857**	-41.40595953589	-41.40596011566	-41.40596073505	-41.40596105562	-41.40596169002
**12.818571**	-41.40595829108	-41.40595880163	-41.40595934238	-41.40595934567	-41.40596004471
**12.974286**	-41.40595647664	-41.40595717025	-41.40595804332	-41.40595772076	-41.40595833126
**13.130000**	-41.40595512217	-41.40595574165	-41.40595642917	-41.40595617751	-41.40595671728
**13.285714**	-41.40595382303	-41.40595437521	-41.40595501198	-41.40595471254	-41.40595518884
**13.441429**	-41.40595257802	-41.40595306900	-41.40595365871	-41.40595332255	-41.40595374204
**13.597143**	-41.40595138581	-41.40595182107	-41.40595236708	-41.40595200426	-41.40595237305
**13.752857**	-41.40595024501	-41.40595062943	-41.40595113483	-41.40595075449	-41.40595107817
**13.908571**	-41.40594915413	-41.40594949209	-41.40594995971	-41.40594957013	-41.40594985381
**14.064286**	-41.40594811164	-41.40594840707	-41.40594883950	-41.40594844814	-41.40594869649
**14.220000**	-41.40594711598	-41.40594737239	-41.40594777204	-41.40594738559	-41.40594760288
**14.375714**	-41.40594616554	-41.40594638611	-41.40594675517	-41.40594637965	-41.40594656976
**14.531429**	-41.40594525875	-41.40594544630	-41.40594578681	-41.40594542756	-41.40594559403
**14.687143**	-41.40594439399	-41.40594455109	-41.40594486491	-41.40594452669	-41.40594473493
**14.842857**	-41.40594356968	-41.40594369864	-41.40594398750	-41.40594367449	-41.40594391037
**14.998571**	-41.40594278425	-41.40594288714	-41.40594315265	-41.40594286852	-41.40594313003
**15.154286**	-41.40594203616	-41.40594211485	-41.40594235848	-41.40594219897	-41.40594239114
**15.310000**	-41.40594132387	-41.40594138007	-41.40594160318	-41.40594151106	-41.40594169125

R (Bohr)/θ(º)	100.00	110.00	120.00	130.00	140.00
**15.465714**	-41.40594064592	-41.40594068116	-41.40594088502	-41.40594085848	-41.40594102810
**15.621429**	-41.40594000083	-41.40594001651	-41.40594006503	-41.40594023917	-41.40594039848
**15.777143**	-41.40593938721	-41.40593930299	-41.40593948591	-41.40593965114	-41.40593980037
**15.932857**	-41.40593880367	-41.40593876064	-41.40593893532	-41.40593909253	-41.40593923339
**16.088571**	-41.40593805549	-41.40593824460	-41.40593841185	-41.40593856161	-41.40593869498
**16.244286**	-41.40593757197	-41.40593775329	-41.40593791368	-41.40593805682	-41.40593818348
**16.400000**	-41.40593711126	-41.40593728537	-41.40593743946	-41.40593757649	-41.40593769705
**16.555714**	-41.40593667212	-41.40593683956	-41.40593698781	-41.40593711927	-41.40593723428
**16.711429**	-41.40593625340	-41.40593641462	-41.40593655747	-41.40593668382	-41.40593679380
**16.867143**	-41.40593585399	-41.40593600940	-41.40593614724	-41.40593626889	-41.40593637430
**17.022857**	-41.40593547285	-41.40593562282	-41.40593575599	-41.40593587332	-41.40593597460
**17.178571**	-41.40593510900	-41.40593525387	-41.40593538267	-41.40593549602	-41.40593559354
**17.334286**	-41.40593476151	-41.40593490160	-41.40593502630	-41.40593513597	-41.40593523009
**17.490000**	-41.40593442950	-41.40593456510	-41.40593468595	-41.40593479222	-41.40593488326
**17.645714**	-41.40593411216	-41.40593424353	-41.40593436076	-41.40593446388	-41.40593455212
**17.801429**	-41.40593380871	-41.40593393610	-41.40593404992	-41.40593415011	-41.40593423581
**17.957143**	-41.40593351845	-41.40593364207	-41.40593375267	-41.40593385013	-41.40593393354
**18.112857**	-41.40593324067	-41.40593336074	-41.40593346828	-41.40593356319	-41.40593364452
**18.268571**	-41.40593297476	-41.40593309145	-41.40593319608	-41.40593328861	-41.40593336805
**18.424286**	-41.40593272011	-41.40593283357	-41.40593293544	-41.40593302572	-41.40593310346
**18.580000**	-41.40593247617	-41.40593258652	-41.40593268574	-41.40593277391	-41.40593285010
**18.735714**	-41.40593224238	-41.40593234973	-41.40593244642	-41.40593253259	-41.40593260736
**18.891429**	-41.40593201825	-41.40593212269	-41.40593221693	-41.40593230119	-41.40593237466
**19.047143**	-41.40593180326	-41.40593190487	-41.40593199674	-41.40593207917	-41.40593215145
**19.202857**	-41.40593159691	-41.40593169576	-41.40593178534	-41.40593186601	-41.40593193719
**19.358571**	-41.40593139859	-41.40593149484	-41.40593158222	-41.40593166118	-41.40593173134
**19.514286**	-41.40593120678	-41.40593130139	-41.40593138679	-41.40593146415	-41.40593153335
**19.670000**	-41.40593102053	-41.40593110759	-41.40593119782	-41.40593127417	-41.40593134261
**19.825714**	-41.40593084632	-41.40593093393	-41.40593100594	-41.40593108306	-41.40593115743
**19.981429**	-41.40593067625	-41.40593076103	-41.40593084006	-41.40593091256	-41.40593097604
**20.137143**	-41.40593051851	-41.40593059649	-41.40593066975	-41.40593073964	-41.40593080613
**20.292857**	-41.40593037051	-41.40593044498	-41.40593051287	-41.40593057549	-41.40593063540
**20.448571**	-41.40593022729	-41.40593029947	-41.40593036538	-41.40593042578	-41.40593048240
**20.604286**	-41.40593008911	-41.40593015834	-41.40593022148	-41.40593027922	-41.40593033346
**20.760000**	-41.40592995707	-41.40593002175	-41.40593008261	-41.40593013668	-41.40593018676
**20.915714**	-41.40592983207	-41.40592989430	-41.40592995009	-41.40592999856	-41.40593004528
**21.071429**	-41.40592971403	-41.40592977292	-41.40592982558	-41.40592987157	-41.40592991142
**21.227143**	-41.40592960321	-41.40592965907	-41.40592970875	-41.40592975152	-41.40592978726
**21.382857**	-41.40592949942	-41.40592955272	-41.40592960011	-41.40592964070	-41.40592967408
**21.538571**	-41.40592940213	-41.40592945341	-41.40592949926	-41.40592953888	-41.40592957207
**21.694286**	-41.40592931046	-41.40592936023	-41.40592940516	-41.40592944472	-41.40592947919
**21.850000**	-41.40592922337	-41.40592927200	-41.40592931635	-41.40592935616	-41.40592939208
**22.005714**	-41.40592913984	-41.40592918750	-41.40592923130	-41.40592927117	-41.40592930779
**22.161429**	-41.40592905903	-41.40592910574	-41.40592914882	-41.40592918827	-41.40592922456
**22.317143**	-41.40592898040	-41.40592902605	-41.40592906814	-41.40592910668	-41.40592914184
**22.472857**	-41.40592890368	-41.40592894808	-41.40592898891	-41.40592902615	-41.40592905971
**22.628571**	-41.40592882880	-41.40592887173	-41.40592891105	-41.40592894674	-41.40592897856
**22.784286**	-41.40592875583	-41.40592879698	-41.40592883460	-41.40592886865	-41.40592889869
**22.940000**	-41.40592868479	-41.40592872379	-41.40592875958	-41.40592879196	-41.40592882036
**23.095714**	-41.40592861534	-41.40592865185	-41.40592868583	-41.40592871669	-41.40592874361
**23.251429**	-41.40592854566	-41.40592858026	-41.40592861294	-41.40592864259	-41.40592866835
**23.407143**	-41.40592847174	-41.40592850752	-41.40592854016	-41.40592856931	-41.40592859432
**23.562857**	-41.40592840038	-41.40592843443	-41.40592846745	-41.40592849663	-41.40592852134
**23.718571**	-41.40592834487	-41.40592837024	-41.40592839826	-41.40592842586	-41.40592844990
**23.874286**	-41.40592828323	-41.40592831469	-41.40592833830	-41.40592836102	-41.40592838239
**24.030000**	-41.40592821863	-41.40592825477	-41.40592828267	-41.40592830381	-41.40592832181
**24.185714**	-41.40592815711	-41.40592819211	-41.40592822352	-41.40592824799	-41.40592826626
**24.341429**	-41.40592809894	-41.40592813134	-41.40592816241	-41.40592818917	-41.40592820998
**24.497143**	-41.40592804346	-41.40592807355	-41.40592810276	-41.40592812928	-41.40592815132
**24.652857**	-41.40592799020	-41.40592801847	-41.40592804577	-41.40592807089	-41.40592809254
**24.808571**	-41.40592793887	-41.40592796570	-41.40592799139	-41.40592801499	-41.40592803554
**24.964286**	-41.40592788932	-41.40592791495	-41.40592793931	-41.40592796157	-41.40592798094
**25.120000**	-41.40592784140	-41.40592786602	-41.40592788927	-41.40592791040	-41.40592792872
**25.275714**	-41.40592779503	-41.40592781875	-41.40592784105	-41.40592786124	-41.40592787866
**25.431429**	-41.40592775013	-41.40592777303	-41.40592779450	-41.40592781387	-41.40592783052

R (Bohr)/θ(º)	100.00	110.00	120.00	130.00	140.00
**25.587143**	-41.40592770664	-41.40592772878	-41.40592774951	-41.40592776815	-41.40592778414
**25.742857**	-41.40592766451	-41.40592768594	-41.40592770597	-41.40592772396	-41.40592773937
**25.898571**	-41.40592762367	-41.40592764444	-41.40592766382	-41.40592768121	-41.40592769608
**26.054286**	-41.40592758409	-41.40592760422	-41.40592762300	-41.40592763982	-41.40592765421
**26.210000**	-41.40592754571	-41.40592756522	-41.40592758343	-41.40592759974	-41.40592761366
**26.365714**	-41.40592750849	-41.40592752742	-41.40592754508	-41.40592756089	-41.40592757438
**26.521429**	-41.40592747238	-41.40592749076	-41.40592750789	-41.40592752323	-41.40592753631
**26.677143**	-41.40592743735	-41.40592745519	-41.40592747182	-41.40592748670	-41.40592749940
**26.832857**	-41.40592740334	-41.40592742068	-41.40592743683	-41.40592745128	-41.40592746361
**26.988571**	-41.40592737037	-41.40592738719	-41.40592740288	-41.40592741691	-41.40592742889
**27.144286**	-41.40592733835	-41.40592735469	-41.40592736992	-41.40592738356	-41.40592739519
**27.300000**	-41.40592730726	-41.40592732313	-41.40592733793	-41.40592735118	-41.40592736249
**27.455714**	-41.40592727707	-41.40592729250	-41.40592730688	-41.40592731975	-41.40592733074
**27.611429**	-41.40592724774	-41.40592726274	-41.40592727672	-41.40592728924	-41.40592729992
**27.767143**	-41.40592721926	-41.40592723383	-41.40592724743	-41.40592725960	-41.40592726998
**27.922857**	-41.40592719158	-41.40592720576	-41.40592721897	-41.40592723081	-41.40592724091
**28.078571**	-41.40592716698	-41.40592717847	-41.40592719132	-41.40592720283	-41.40592721266
**28.234286**	-41.40592714041	-41.40592715397	-41.40592716652	-41.40592717639	-41.40592718595
**28.390000**	-41.40592711520	-41.40592712824	-41.40592714020	-41.40592714995	-41.40592715927
**28.545714**	-41.40592709026	-41.40592710295	-41.40592711503	-41.40592712429	-41.40592713333
**28.701429**	-41.40592706600	-41.40592707859	-41.40592709034	-41.40592709909	-41.40592710815
**28.857143**	-41.40592704264	-41.40592705356	-41.40592706610	-41.40592707482	-41.40592708364
**29.012857**	-41.40592701992	-41.40592703052	-41.40592704252	-41.40592705121	-41.40592705932
**29.168571**	-41.40592699781	-41.40592700920	-41.40592701982	-41.40592702825	-41.40592703614
**29.324286**	-41.40592697497	-41.40592698714	-41.40592699749	-41.40592700543	-41.40592701335
**29.480000**	-41.40592695380	-41.40592696593	-41.40592697600	-41.40592698369	-41.40592699141
**29.635714**	-41.40592693343	-41.40592694394	-41.40592695375	-41.40592696255	-41.40592697007
**29.791429**	-41.40592691360	-41.40592692384	-41.40592693340	-41.40592694197	-41.40592694929
**29.947143**	-41.40592689430	-41.40592690427	-41.40592691358	-41.40592692193	-41.40592692907
**30.102857**	-41.40592687550	-41.40592688522	-41.40592689429	-41.40592690243	-41.40592690938
**30.258571**	-41.40592685719	-41.40592686666	-41.40592687551	-41.40592688343	-41.40592689021
**30.414286**	-41.40592683936	-41.40592684859	-41.40592685721	-41.40592686494	-41.40592687155
**30.570000**	-41.40592682199	-41.40592683099	-41.40592683939	-41.40592684693	-41.40592685337
**30.725714**	-41.40592680507	-41.40592681384	-41.40592682203	-41.40592682938	-41.40592683566
**30.881429**	-41.40592678858	-41.40592679713	-41.40592680512	-41.40592681228	-41.40592681841
**31.037143**	-41.40592677251	-41.40592678085	-41.40592678864	-41.40592679563	-41.40592680160
**31.192857**	-41.40592675685	-41.40592676498	-41.40592677258	-41.40592677940	-41.40592678523
**31.348571**	-41.40592674159	-41.40592674952	-41.40592675693	-41.40592676358	-41.40592676926
**31.504286**	-41.40592672670	-41.40592673444	-41.40592674166	-41.40592674815	-41.40592675370
**31.660000**	-41.40592671219	-41.40592671973	-41.40592672679	-41.40592673312	-41.40592673853
**31.815714**	-41.40592669804	-41.40592670540	-41.40592671228	-41.40592671846	-41.40592672375
**31.971429**	-41.40592668424	-41.40592669142	-41.40592669814	-41.40592670417	-41.40592670933
**32.127143**	-41.40592667077	-41.40592667779	-41.40592668434	-41.40592669023	-41.40592669526
**32.282857**	-41.40592665764	-41.40592666449	-41.40592667089	-41.40592667663	-41.40592668154
**32.438571**	-41.40592664483	-41.40592665151	-41.40592665776	-41.40592666337	-41.40592666817
**32.594286**	-41.40592663232	-41.40592663885	-41.40592664495	-41.40592665042	-41.40592665511
**32.750000**	-41.40592662012	-41.40592662649	-41.40592663245	-41.40592663779	-41.40592664237
**32.905714**	-41.40592660821	-41.40592661443	-41.40592662025	-41.40592662547	-41.40592662994
**33.061429**	-41.40592659658	-41.40592660266	-41.40592660834	-41.40592661344	-41.40592661781
**33.217143**	-41.40592658523	-41.40592659117	-41.40592659672	-41.40592660170	-41.40592660597
**33.372857**	-41.40592657416	-41.40592657995	-41.40592658537	-41.40592659024	-41.40592659441
**33.528571**	-41.40592656333	-41.40592656900	-41.40592657430	-41.40592657906	-41.40592658313
**33.684286**	-41.40592655277	-41.40592655830	-41.40592656348	-41.40592656813	-41.40592657211
**33.840000**	-41.40592654245	-41.40592654786	-41.40592655291	-41.40592655746	-41.40592656135
**33.995714**	-41.40592653237	-41.40592653765	-41.40592654259	-41.40592654704	-41.40592655084
**34.151429**	-41.40592652252	-41.40592652769	-41.40592653251	-41.40592653686	-41.40592654058
**34.307143**	-41.40592651290	-41.40592651795	-41.40592652267	-41.40592652692	-41.40592653055
**34.462857**	-41.40592650349	-41.40592650843	-41.40592651305	-41.40592651720	-41.40592652075
**34.618571**	-41.40592649430	-41.40592649913	-41.40592650365	-41.40592650771	-41.40592651118
**34.774286**	-41.40592648533	-41.40592649005	-41.40592649446	-41.40592649843	-41.40592650183
**34.930000**	-41.40592647655	-41.40592648117	-41.40592648549	-41.40592648937	-41.40592649269
**35.085714**	-41.40592646797	-41.40592647248	-41.40592647671	-41.40592648050	-41.40592648376
**35.241429**	-41.40592645958	-41.40592646400	-41.40592646813	-41.40592647184	-41.40592647503
**35.397143**	-41.40592645138	-41.40592645570	-41.40592645974	-41.40592646337	-41.40592646649
**35.552857**	-41.40592644336	-41.40592644759	-41.40592645154	-41.40592645510	-41.40592645814

R (Bohr)/θ(º)	100.00	110.00	120.00	130.00	140.00
**35.708571**	-41.40592643552	-41.40592643965	-41.40592644352	-41.40592644700	-41.40592644998
**35.864286**	-41.40592642785	-41.40592643189	-41.40592643568	-41.40592643908	-41.40592644200
**36.020000**	-41.40592642035	-41.40592642431	-41.40592642801	-41.40592643134	-41.40592643420
**36.175714**	-41.40592641301	-41.40592641688	-41.40592642051	-41.40592642377	-41.40592642657
**36.331429**	-41.40592640583	-41.40592640962	-41.40592641317	-41.40592641636	-41.40592641910
**36.487143**	-41.40592639880	-41.40592640251	-41.40592640599	-41.40592640911	-41.40592641179
**36.642857**	-41.40592639193	-41.40592639556	-41.40592639896	-41.40592640202	-41.40592640465
**36.798571**	-41.40592638520	-41.40592638876	-41.40592639209	-41.40592639508	-41.40592639765
**36.954286**	-41.40592637862	-41.40592638210	-41.40592638536	-41.40592638830	-41.40592639081
**37.110000**	-41.40592637217	-41.40592637559	-41.40592637878	-41.40592638165	-41.40592638411
**37.265714**	-41.40592636586	-41.40592636921	-41.40592637234	-41.40592637515	-41.40592637756
**37.421429**	-41.40592635969	-41.40592636296	-41.40592636603	-41.40592636878	-41.40592637115
**37.577143**	-41.40592635364	-41.40592635685	-41.40592635985	-41.40592636255	-41.40592636487
**37.732857**	-41.40592634772	-41.40592635086	-41.40592635380	-41.40592635645	-41.40592635872
**37.888571**	-41.40592634193	-41.40592634501	-41.40592634789	-41.40592635048	-41.40592635270
**38.044286**	-41.40592633625	-41.40592633927	-41.40592634209	-41.40592634463	-41.40592634680
**38.200000**	-41.40592633069	-41.40592633364	-41.40592633641	-41.40592633890	-41.40592634103
**38.355714**	-41.40592632524	-41.40592632814	-41.40592633085	-41.40592633329	-41.40592633538
**38.511429**	-41.40592631991	-41.40592632275	-41.40592632541	-41.40592632779	-41.40592632985
**38.667143**	-41.40592631468	-41.40592631746	-41.40592632007	-41.40592632241	-41.40592632442
**38.822857**	-41.40592630957	-41.40592631229	-41.40592631484	-41.40592631714	-41.40592631911
**38.978571**	-41.40592630455	-41.40592630722	-41.40592630972	-41.40592631197	-41.40592631391
**39.134286**	-41.40592629963	-41.40592630225	-41.40592630470	-41.40592630691	-41.40592630880
**39.290000**	-41.40592629481	-41.40592629738	-41.40592629979	-41.40592630195	-41.40592630381
**39.445714**	-41.40592629009	-41.40592629261	-41.40592629497	-41.40592629709	-41.40592629891
**39.601429**	-41.40592628547	-41.40592628793	-41.40592629025	-41.40592629233	-41.40592629412
**39.757143**	-41.40592628093	-41.40592628335	-41.40592628562	-41.40592628766	-41.40592628941
**39.912857**	-41.40592627648	-41.40592627886	-41.40592628108	-41.40592628309	-41.40592628480
**40.068571**	-41.40592627212	-41.40592627445	-41.40592627664	-41.40592627860	-41.40592628028
**40.224286**	-41.40592626785	-41.40592627013	-41.40592627227	-41.40592627420	-41.40592627586
**40.380000**	-41.40592626366	-41.40592626590	-41.40592626800	-41.40592626989	-41.40592627151
**40.535714**	-41.40592625955	-41.40592626175	-41.40592626381	-41.40592626566	-41.40592626725
**40.691429**	-41.40592625552	-41.40592625768	-41.40592625970	-41.40592626151	-41.40592626307
**40.847143**	-41.40592625157	-41.40592625368	-41.40592625567	-41.40592625745	-41.40592625898
**41.002857**	-41.40592624769	-41.40592624977	-41.40592625171	-41.40592625346	-41.40592625497
**41.158571**	-41.40592624389	-41.40592624592	-41.40592624783	-41.40592624955	-41.40592625103
**41.314286**	-41.40592624016	-41.40592624216	-41.40592624403	-41.40592624572	-41.40592624716
**41.470000**	-41.40592623650	-41.40592623846	-41.40592624030	-41.40592624196	-41.40592624338
**41.625714**	-41.40592623291	-41.40592623484	-41.40592623664	-41.40592623827	-41.40592623966
**41.781429**	-41.40592622938	-41.40592623128	-41.40592623305	-41.40592623465	-41.40592623601
**41.937143**	-41.40592622593	-41.40592622779	-41.40592622952	-41.40592623109	-41.40592623244
**42.092857**	-41.40592622254	-41.40592622436	-41.40592622607	-41.40592622760	-41.40592622892
**42.248571**	-41.40592621920	-41.40592622100	-41.40592622267	-41.40592622418	-41.40592622548
**42.404286**	-41.40592621594	-41.40592621770	-41.40592621934	-41.40592622083	-41.40592622210
**42.560000**	-41.40592621273	-41.40592621446	-41.40592621607	-41.40592621753	-41.40592621878
**42.715714**	-41.40592620958	-41.40592621128	-41.40592621287	-41.40592621430	-41.40592621553
**42.871429**	-41.40592620649	-41.40592620816	-41.40592620972	-41.40592621112	-41.40592621233
**43.027143**	-41.40592620346	-41.40592620509	-41.40592620663	-41.40592620801	-41.40592620919
**43.182857**	-41.40592620048	-41.40592620209	-41.40592620359	-41.40592620495	-41.40592620611
**43.338571**	-41.40592619756	-41.40592619914	-41.40592620061	-41.40592620194	-41.40592620309
**43.494286**	-41.40592619468	-41.40592619623	-41.40592619769	-41.40592619899	-41.40592620012
**43.650000**	-41.40592619186	-41.40592619339	-41.40592619481	-41.40592619610	-41.40592619720
**43.805714**	-41.40592618909	-41.40592619059	-41.40592619199	-41.40592619326	-41.40592619434
**43.961429**	-41.40592618637	-41.40592618784	-41.40592618922	-41.40592619046	-41.40592619153
**44.117143**	-41.40592618370	-41.40592618515	-41.40592618650	-41.40592618772	-41.40592618877
**44.272857**	-41.40592618108	-41.40592618250	-41.40592618383	-41.40592618503	-41.40592618605
**44.428571**	-41.40592617850	-41.40592617990	-41.40592618120	-41.40592618238	-41.40592618339
**44.584286**	-41.40592617597	-41.40592617734	-41.40592617862	-41.40592617978	-41.40592618078
**44.740000**	-41.40592617348	-41.40592617483	-41.40592617609	-41.40592617723	-41.40592617821
**44.895714**	-41.40592617104	-41.40592617236	-41.40592617360	-41.40592617472	-41.40592617568
**45.051429**	-41.40592616864	-41.40592616994	-41.40592617116	-41.40592617226	-41.40592617320
**45.207143**	-41.40592616627	-41.40592616756	-41.40592616876	-41.40592616983	-41.40592617076
**45.362857**	-41.40592616396	-41.40592616521	-41.40592616639	-41.40592616746	-41.40592616837
**45.518571**	-41.40592616168	-41.40592616291	-41.40592616407	-41.40592616512	-41.40592616602
**45.674286**	-41.40592615944	-41.40592616066	-41.40592616179	-41.40592616282	-41.40592616370

R (Bohr)/θ(º)	100.00	110.00	120.00	130.00	140.00
**45.830000**	-41.40592615724	-41.40592615843	-41.40592615955	-41.40592616056	-41.40592616143
**45.985714**	-41.40592615507	-41.40592615625	-41.40592615735	-41.40592615834	-41.40592615920
**46.141429**	-41.40592615294	-41.40592615410	-41.40592615519	-41.40592615616	-41.40592615700
**46.297143**	-41.40592615086	-41.40592615199	-41.40592615306	-41.40592615402	-41.40592615485
**46.452857**	-41.40592614880	-41.40592614992	-41.40592615097	-41.40592615191	-41.40592615273
**46.608571**	-41.40592614678	-41.40592614788	-41.40592614891	-41.40592614984	-41.40592615064
**46.764286**	-41.40592614479	-41.40592614587	-41.40592614689	-41.40592614780	-41.40592614859
**46.920000**	-41.40592614284	-41.40592614391	-41.40592614490	-41.40592614580	-41.40592614657
**47.075714**	-41.40592614092	-41.40592614197	-41.40592614295	-41.40592614383	-41.40592614459
**47.231429**	-41.40592613903	-41.40592614006	-41.40592614102	-41.40592614190	-41.40592614264
**47.387143**	-41.40592613717	-41.40592613819	-41.40592613914	-41.40592613999	-41.40592614073
**47.542857**	-41.40592613534	-41.40592613634	-41.40592613728	-41.40592613812	-41.40592613884
**47.698571**	-41.40592613355	-41.40592613453	-41.40592613545	-41.40592613628	-41.40592613699
**47.854286**	-41.40592613178	-41.40592613274	-41.40592613365	-41.40592613447	-41.40592613517
**48.010000**	-41.40592613004	-41.40592613099	-41.40592613188	-41.40592613269	-41.40592613338
**48.165714**	-41.40592612833	-41.40592612927	-41.40592613014	-41.40592613093	-41.40592613161
**48.321429**	-41.40592612665	-41.40592612757	-41.40592612843	-41.40592612921	-41.40592612988
**48.477143**	-41.40592612499	-41.40592612590	-41.40592612675	-41.40592612752	-41.40592612818
**48.632857**	-41.40592612337	-41.40592612426	-41.40592612509	-41.40592612585	-41.40592612650
**48.788571**	-41.40592612176	-41.40592612264	-41.40592612347	-41.40592612421	-41.40592612485
**48.944286**	-41.40592612019	-41.40592612105	-41.40592612186	-41.40592612259	-41.40592612322
**49.100000**	-41.40592611864	-41.40592611949	-41.40592612029	-41.40592612101	-41.40592612163
**49.255714**	-41.40592611711	-41.40592611795	-41.40592611873	-41.40592611944	-41.40592612005
**49.411429**	-41.40592611561	-41.40592611643	-41.40592611721	-41.40592611791	-41.40592611850
**49.567143**	-41.40592611413	-41.40592611494	-41.40592611570	-41.40592611639	-41.40592611698
**49.722857**	-41.40592611268	-41.40592611348	-41.40592611423	-41.40592611490	-41.40592611548
**49.878571**	-41.40592611124	-41.40592611203	-41.40592611277	-41.40592611344	-41.40592611401
**50.034286**	-41.40592610983	-41.40592611061	-41.40592611134	-41.40592611199	-41.40592611256
**50.190000**	-41.40592610845	-41.40592610921	-41.40592610993	-41.40592611057	-41.40592611113
**50.345714**	-41.40592610708	-41.40592610783	-41.40592610854	-41.40592610918	-41.40592610972
**50.501429**	-41.40592610574	-41.40592610648	-41.40592610717	-41.40592610780	-41.40592610834
**50.657143**	-41.40592610441	-41.40592610514	-41.40592610583	-41.40592610645	-41.40592610698
**50.812857**	-41.40592610311	-41.40592610383	-41.40592610450	-41.40592610511	-41.40592610563
**50.968571**	-41.40592610182	-41.40592610254	-41.40592610320	-41.40592610380	-41.40592610431
**51.124286**	-41.40592610056	-41.40592610126	-41.40592610192	-41.40592610250	-41.40592610301
**51.280000**	-41.40592609932	-41.40592610001	-41.40592610065	-41.40592610123	-41.40592610173
**51.435714**	-41.40592609810	-41.40592609877	-41.40592609941	-41.40592609998	-41.40592610047
**51.591429**	-41.40592609689	-41.40592609755	-41.40592609818	-41.40592609875	-41.40592609923
**51.747143**	-41.40592609570	-41.40592609636	-41.40592609697	-41.40592609753	-41.40592609801
**51.902857**	-41.40592609453	-41.40592609518	-41.40592609579	-41.40592609633	-41.40592609681
**52.058571**	-41.40592609338	-41.40592609402	-41.40592609462	-41.40592609516	-41.40592609562
**52.214286**	-41.40592609224	-41.40592609287	-41.40592609346	-41.40592609400	-41.40592609445
**52.370000**	-41.40592609113	-41.40592609174	-41.40592609233	-41.40592609285	-41.40592609330
**52.525714**	-41.40592609002	-41.40592609064	-41.40592609121	-41.40592609173	-41.40592609217
**52.681429**	-41.40592608894	-41.40592608954	-41.40592609011	-41.40592609062	-41.40592609106
**52.837143**	-41.40592608787	-41.40592608847	-41.40592608902	-41.40592608953	-41.40592608996
**52.992857**	-41.40592608682	-41.40592608740	-41.40592608795	-41.40592608845	-41.40592608887
**53.148571**	-41.40592608578	-41.40592608636	-41.40592608690	-41.40592608739	-41.40592608781
**53.304286**	-41.40592608476	-41.40592608533	-41.40592608586	-41.40592608634	-41.40592608676
**53.460000**	-41.40592608375	-41.40592608431	-41.40592608484	-41.40592608531	-41.40592608572
**53.615714**	-41.40592608276	-41.40592608332	-41.40592608383	-41.40592608430	-41.40592608470
**53.771429**	-41.40592608179	-41.40592608233	-41.40592608284	-41.40592608330	-41.40592608370
**53.927143**	-41.40592608082	-41.40592608136	-41.40592608186	-41.40592608232	-41.40592608271
**54.082857**	-41.40592607987	-41.40592608040	-41.40592608090	-41.40592608134	-41.40592608173
**54.238571**	-41.40592607894	-41.40592607946	-41.40592607995	-41.40592608039	-41.40592608077
**54.394286**	-41.40592607801	-41.40592607853	-41.40592607901	-41.40592607945	-41.40592607982
**54.550000**	-41.40592607711	-41.40592607761	-41.40592607809	-41.40592607852	-41.40592607889
**54.705714**	-41.40592607621	-41.40592607671	-41.40592607718	-41.40592607760	-41.40592607797
**54.861429**	-41.40592607533	-41.40592607582	-41.40592607628	-41.40592607670	-41.40592607706
**55.017143**	-41.40592607446	-41.40592607495	-41.40592607540	-41.40592607581	-41.40592607617
**55.172857**	-41.40592607360	-41.40592607408	-41.40592607453	-41.40592607494	-41.40592607529
**55.328571**	-41.40592607275	-41.40592607323	-41.40592607367	-41.40592607407	-41.40592607442
**55.484286**	-41.40592607192	-41.40592607239	-41.40592607282	-41.40592607322	-41.40592607356
**55.640000**	-41.40592607110	-41.40592607156	-41.40592607199	-41.40592607238	-41.40592607272
**55.795714**	-41.40592607029	-41.40592607074	-41.40592607117	-41.40592607155	-41.40592607188

R (Bohr)/θ(º)	100.00	110.00	120.00	130.00	140.00
**55.951429**	-41.40592606949	-41.40592606994	-41.40592607036	-41.40592607074	-41.40592607106
**56.107143**	-41.40592606870	-41.40592606914	-41.40592606956	-41.40592606993	-41.40592607026
**56.262857**	-41.40592606793	-41.40592606836	-41.40592606877	-41.40592606914	-41.40592606946
**56.418571**	-41.40592606716	-41.40592606759	-41.40592606799	-41.40592606836	-41.40592606867
**56.574286**	-41.40592606640	-41.40592606683	-41.40592606723	-41.40592606759	-41.40592606790
**56.730000**	-41.40592606566	-41.40592606608	-41.40592606647	-41.40592606683	-41.40592606713
**56.885714**	-41.40592606493	-41.40592606534	-41.40592606573	-41.40592606608	-41.40592606638
**57.041429**	-41.40592606420	-41.40592606461	-41.40592606499	-41.40592606534	-41.40592606564
**57.197143**	-41.40592606349	-41.40592606389	-41.40592606427	-41.40592606461	-41.40592606490
**57.352857**	-41.40592606278	-41.40592606318	-41.40592606355	-41.40592606389	-41.40592606418
**57.508571**	-41.40592606209	-41.40592606248	-41.40592606285	-41.40592606318	-41.40592606347
**57.664286**	-41.40592606140	-41.40592606179	-41.40592606215	-41.40592606248	-41.40592606276
**57.820000**	-41.40592606073	-41.40592606111	-41.40592606147	-41.40592606179	-41.40592606207
**57.975714**	-41.40592606006	-41.40592606044	-41.40592606079	-41.40592606111	-41.40592606138
**58.131429**	-41.40592605940	-41.40592605978	-41.40592606012	-41.40592606044	-41.40592606071
**58.287143**	-41.40592605875	-41.40592605912	-41.40592605947	-41.40592605978	-41.40592606005
**58.442857**	-41.40592605811	-41.40592605848	-41.40592605882	-41.40592605912	-41.40592605939
**58.598571**	-41.40592605748	-41.40592605784	-41.40592605818	-41.40592605848	-41.40592605874
**58.754286**	-41.40592605686	-41.40592605721	-41.40592605754	-41.40592605784	-41.40592605810
**58.910000**	-41.40592605624	-41.40592605659	-41.40592605692	-41.40592605722	-41.40592605747
**59.065714**	-41.40592605564	-41.40592605598	-41.40592605631	-41.40592605660	-41.40592605685
**59.221429**	-41.40592605504	-41.40592605538	-41.40592605570	-41.40592605599	-41.40592605623
**59.377143**	-41.40592605445	-41.40592605479	-41.40592605510	-41.40592605538	-41.40592605563
**59.532857**	-41.40592605386	-41.40592605420	-41.40592605451	-41.40592605479	-41.40592605503
**59.688571**	-41.40592605329	-41.40592605362	-41.40592605393	-41.40592605420	-41.40592605444
**59.844286**	-41.40592605272	-41.40592605305	-41.40592605335	-41.40592605362	-41.40592605386
**60.000000**	-41.40592603258	-41.40592605247	-41.40592605278	-41.40592605303	-41.40592605225

PES aV6Z – CH^- ($X\ ^3\Sigma^-$) + He				
**R (Bohr)/θ(º)**	**150.00**	**160.00**	**170.00**	**180.00**
**5.500000**	-41.40399450446	-41.40400673358	-41.40401101212	-41.40398843485
**5.655714**	-41.40446757843	-41.40448722024	-41.40449864249	-41.40447750004
**5.811429**	-41.40483957353	-41.40486418962	-41.40488056304	-41.40485908745
**5.967143**	-41.40513144018	-41.40515926471	-41.40517872791	-41.40515608529
**6.122857**	-41.40535979261	-41.40538958720	-41.40541056265	-41.40538646035
**6.278571**	-41.40553805135	-41.40556876875	-41.40559015255	-41.40556464098
**6.434286**	-41.40567685586	-41.40570771435	-41.40572853831	-41.40570196274
**6.590000**	-41.40578465223	-41.40581476389	-41.40583465576	-41.40580728688
**6.745714**	-41.40586791010	-41.40589677567	-41.40591544793	-41.40588752905
**6.901429**	-41.40593183530	-41.40595902122	-41.40597638515	-41.40594808868
**7.057143**	-41.40598046342	-41.40600575221	-41.40602173876	-41.40599319209
**7.212857**	-41.40601705180	-41.40604032237	-41.40605490154	-41.40602617278
**7.368571**	-41.40604410816	-41.40606532123	-41.40607848217	-41.40604965314
**7.524286**	-41.40606359277	-41.40608280195	-41.40609456094	-41.40606573489
**7.680000**	-41.40607703869	-41.40609437269	-41.40610477535	-41.40607604533
**7.835714**	-41.40608569015	-41.40610125451	-41.40611043051	-41.40608192656
**7.991429**	-41.40609055075	-41.40610445749	-41.40611252797	-41.40608440138
**8.147143**	-41.40609238949	-41.40610478690	-41.40610885969	-41.40608427057
**8.302857**	-41.40609183252	-41.40610227664	-41.40610142680	-41.40608222891
**8.458571**	-41.40608940214	-41.40609924015	-41.40609643967	-41.40607874461
**8.614286**	-41.40608551837	-41.40609220710	-41.40609035494	-41.40607426834
**8.770000**	-41.40608052031	-41.40608597130	-41.40608348965	-41.40606910100
**8.925714**	-41.40607329202	-41.40607601046	-41.40607610742	-41.40606345895
**9.081429**	-41.40606567068	-41.40606553039	-41.40606817831	-41.40605727428
**9.237143**	-41.40605617804	-41.40605778591	-41.40606041573	-41.40605122358
**9.392857**	-41.40604590025	-41.40605038128	-41.40605264320	-41.40604508940
**9.548571**	-41.40603915173	-41.40604311555	-41.40604510148	-41.40603908493
**9.704286**	-41.40603257824	-41.40603608220	-41.40603781749	-41.40603320657
**9.860000**	-41.40602624774	-41.40602934542	-41.40602889225	-41.40602749976
**10.015714**	-41.40602002578	-41.40602296309	-41.40602285933	-41.40602200146
**10.171429**	-41.40601453369	-41.40601695258	-41.40601714367	-41.40601673137
**10.327143**	-41.40600919219	-41.40601133002	-41.40601176685	-41.40601170396
**10.482857**	-41.40600420593	-41.40600609680	-41.40600681026	-41.40600692505
**10.638571**	-41.40599957162	-41.40600125720	-41.40600215656	-41.40600239263
**10.794286**	-41.40599526083	-41.40599687303	-41.40599779917	-41.40599810217
**10.950000**	-41.40599127120	-41.40599280723	-41.40599372281	-41.40599405369
**11.105714**	-41.40598759235	-41.40598902933	-41.40598991329	-41.40599024152
**11.261429**	-41.40598420607	-41.40598554080	-41.40598636388	-41.40598666061
**11.417143**	-41.40598108690	-41.40598229367	-41.40598307057	-41.40598330532
**11.572857**	-41.40597819584	-41.40597927427	-41.40598000593	-41.40598015742
**11.728571**	-41.40597552162	-41.40597646438	-41.40597697399	-41.40597720340
**11.884286**	-41.40597288511	-41.40597384823	-41.40597422177	-41.40597443088
**12.040000**	-41.40597051500	-41.40597140198	-41.40597163440	-41.40597182392
**12.195714**	-41.40596829423	-41.40596910984	-41.40596920145	-41.40596937241
**12.351429**	-41.40596621215	-41.40596696215	-41.40596691354	-41.40596706734
**12.507143**	-41.40596426022	-41.40596495106	-41.40596476233	-41.40596490095
**12.662857**	-41.40596243235	-41.40596306618	-41.40596274070	-41.40596286544
**12.818571**	-41.40596072170	-41.40596129959	-41.40596084034	-41.40596095330
**12.974286**	-41.40595912041	-41.40595964542	-41.40595905147	-41.40595915788
**13.130000**	-41.40595762253	-41.40595749427	-41.40595736955	-41.40595747183
**13.285714**	-41.40595621761	-41.40595555356	-41.40595578874	-41.40595588799
**13.441429**	-41.40595490968	-41.40595409737	-41.40595430206	-41.40595439960
**13.597143**	-41.40595368982	-41.40595272787	-41.40595292152	-41.40595300025
**13.752857**	-41.40595225198	-41.40595143919	-41.40595161764	-41.40595168406
**13.908571**	-41.40595049953	-41.40595022498	-41.40595038740	-41.40595044561
**14.064286**	-41.40594885057	-41.40594908033	-41.40594922799	-41.40594927999
**14.220000**	-41.40594779071	-41.40594800118	-41.40594813568	-41.40594818248
**14.375714**	-41.40594677696	-41.40594697330	-41.40594709978	-41.40594714420
**14.531429**	-41.40594583504	-41.40594601600	-41.40594613252	-41.40594617332
**14.687143**	-41.40594494564	-41.40594511264	-41.40594522014	-41.40594525770
**14.842857**	-41.40594410529	-41.40594425967	-41.40594435902	-41.40594439363
**14.998571**	-41.40594331074	-41.40594345370	-41.40594354566	-41.40594357762
**15.154286**	-41.40594255899	-41.40594269163	-41.40594277689	-41.40594280643
**15.310000**	-41.40594184735	-41.40594197062	-41.40594204977	-41.40594207712

R (Bohr)/θ(º)	150.00	160.00	170.00	180.00
**15.465714**	-41.40594117331	-41.40594128805	-41.40594136161	-41.40594138696
**15.621429**	-41.40594053452	-41.40594064139	-41.40594070994	-41.40594073346
**15.777143**	-41.40593992891	-41.40594002874	-41.40594009248	-41.40594011432
**15.932857**	-41.40593935446	-41.40593944768	-41.40593950713	-41.40593952744
**16.088571**	-41.40593881021	-41.40593889638	-41.40593895192	-41.40593897082
**16.244286**	-41.40593829065	-41.40593837315	-41.40593842468	-41.40593844248
**16.400000**	-41.40593779841	-41.40593787685	-41.40593792475	-41.40593794122
**16.555714**	-41.40593733041	-41.40593740352	-41.40593744934	-41.40593746487
**16.711429**	-41.40593688521	-41.40593695439	-41.40593699760	-41.40593701215
**16.867143**	-41.40593646146	-41.40593652711	-41.40593656796	-41.40593658167
**17.022857**	-41.40593605793	-41.40593612042	-41.40593615917	-41.40593617215
**17.178571**	-41.40593567345	-41.40593573313	-41.40593577003	-41.40593578235
**17.334286**	-41.40593530695	-41.40593536414	-41.40593539940	-41.40593541117
**17.490000**	-41.40593495740	-41.40593501241	-41.40593504626	-41.40593505753
**17.645714**	-41.40593462385	-41.40593467697	-41.40593470959	-41.40593472044
**17.801429**	-41.40593430542	-41.40593435691	-41.40593438850	-41.40593439900
**17.957143**	-41.40593400127	-41.40593405138	-41.40593408210	-41.40593409232
**18.112857**	-41.40593371064	-41.40593375958	-41.40593378960	-41.40593379960
**18.268571**	-41.40593343277	-41.40593348076	-41.40593351026	-41.40593352008
**18.424286**	-41.40593316698	-41.40593321422	-41.40593324333	-41.40593325304
**18.580000**	-41.40593291260	-41.40593295930	-41.40593298817	-41.40593299783
**18.735714**	-41.40593266903	-41.40593271537	-41.40593274416	-41.40593275381
**18.891429**	-41.40593243566	-41.40593248183	-41.40593251068	-41.40593252040
**19.047143**	-41.40593221193	-41.40593225810	-41.40593228719	-41.40593229705
**19.202857**	-41.40593199729	-41.40593204367	-41.40593207317	-41.40593208323
**19.358571**	-41.40593179120	-41.40593183798	-41.40593186811	-41.40593187845
**19.514286**	-41.40593159310	-41.40593164053	-41.40593167151	-41.40593168226
**19.670000**	-41.40593140242	-41.40593145074	-41.40593148291	-41.40593149419
**19.825714**	-41.40593121816	-41.40593126788	-41.40593130166	-41.40593131370
**19.981429**	-41.40593101882	-41.40593107720	-41.40593112150	-41.40593113593
**20.137143**	-41.40593086774	-41.40593092132	-41.40593096003	-41.40593097444
**20.292857**	-41.40593069516	-41.40593075304	-41.40593079846	-41.40593081602
**20.448571**	-41.40593053736	-41.40593059106	-41.40593063775	-41.40593066326
**20.604286**	-41.40593038710	-41.40593044237	-41.40593049341	-41.40593051649
**20.760000**	-41.40593023622	-41.40593028981	-41.40593034619	-41.40593037546
**20.915714**	-41.40593008882	-41.40593013756	-41.40593019915	-41.40593023989
**21.071429**	-41.40592994622	-41.40592998161	-41.40593004552	-41.40593010865
**21.227143**	-41.40592981660	-41.40592984243	-41.40592988108	-41.40592998423
**21.382857**	-41.40592970004	-41.40592971850	-41.40592972955	-41.40592986273
**21.538571**	-41.40592959924	-41.40592962271	-41.40592965624	-41.40592974778
**21.694286**	-41.40592951010	-41.40592954244	-41.40592958972	-41.40592963614
**21.850000**	-41.40592942603	-41.40592946211	-41.40592950358	-41.40592952864
**22.005714**	-41.40592934260	-41.40592937734	-41.40592940981	-41.40592942510
**22.161429**	-41.40592925836	-41.40592928965	-41.40592931495	-41.40592932534
**22.317143**	-41.40592917365	-41.40592920125	-41.40592922151	-41.40592922919
**22.472857**	-41.40592908925	-41.40592911363	-41.40592913042	-41.40592913648
**22.628571**	-41.40592900590	-41.40592902764	-41.40592904201	-41.40592904704
**22.784286**	-41.40592892406	-41.40592894371	-41.40592895634	-41.40592896068
**22.940000**	-41.40592884400	-41.40592886201	-41.40592887336	-41.40592887722
**23.095714**	-41.40592876583	-41.40592878252	-41.40592879292	-41.40592879642
**23.251429**	-41.40592868943	-41.40592870512	-41.40592871480	-41.40592871805
**23.407143**	-41.40592861460	-41.40592862956	-41.40592863874	-41.40592864180
**23.562857**	-41.40592854115	-41.40592855563	-41.40592856446	-41.40592856739
**23.718571**	-41.40592846925	-41.40592848337	-41.40592849193	-41.40592849477
**23.874286**	-41.40592840034	-41.40592841370	-41.40592842187	-41.40592842458
**24.030000**	-41.40592833714	-41.40592834888	-41.40592835620	-41.40592835865
**24.185714**	-41.40592828018	-41.40592829038	-41.40592829669	-41.40592829880
**24.341429**	-41.40592822509	-41.40592823543	-41.40592824150	-41.40592824349
**24.497143**	-41.40592816806	-41.40592817956	-41.40592818623	-41.40592818840
**24.652857**	-41.40592810969	-41.40592812189	-41.40592812911	-41.40592813147
**24.808571**	-41.40592805216	-41.40592806426	-41.40592807155	-41.40592807396
**24.964286**	-41.40592799670	-41.40592800828	-41.40592801533	-41.40592801766
**25.120000**	-41.40592794361	-41.40592795457	-41.40592796126	-41.40592796349
**25.275714**	-41.40592789277	-41.40592790316	-41.40592790950	-41.40592791160
**25.431429**	-41.40592784398	-41.40592785386	-41.40592785989	-41.40592786189

R (Bohr)/θ(º)	150.00	160.00	170.00	180.00
**25.587143**	-41.40592779704	-41.40592780648	-41.40592781223	-41.40592781414
**25.742857**	-41.40592775176	-41.40592776083	-41.40592776634	-41.40592776817
**25.898571**	-41.40592770803	-41.40592771677	-41.40592772207	-41.40592772383
**26.054286**	-41.40592766575	-41.40592767418	-41.40592767930	-41.40592768099
**26.210000**	-41.40592762483	-41.40592763298	-41.40592763794	-41.40592763957
**26.365714**	-41.40592758521	-41.40592759310	-41.40592759789	-41.40592759948
**26.521429**	-41.40592754681	-41.40592755446	-41.40592755910	-41.40592756064
**26.677143**	-41.40592750959	-41.40592751701	-41.40592752151	-41.40592752300
**26.832857**	-41.40592747349	-41.40592748070	-41.40592748507	-41.40592748652
**26.988571**	-41.40592743849	-41.40592744549	-41.40592744973	-41.40592745113
**27.144286**	-41.40592740452	-41.40592741131	-41.40592741544	-41.40592741681
**27.300000**	-41.40592737155	-41.40592737815	-41.40592738217	-41.40592738350
**27.455714**	-41.40592733955	-41.40592734597	-41.40592734987	-41.40592735116
**27.611429**	-41.40592730848	-41.40592731473	-41.40592731852	-41.40592731977
**27.767143**	-41.40592727831	-41.40592728439	-41.40592728807	-41.40592728930
**27.922857**	-41.40592724901	-41.40592725492	-41.40592725851	-41.40592725969
**28.078571**	-41.40592722055	-41.40592722629	-41.40592722978	-41.40592723094
**28.234286**	-41.40592719519	-41.40592720054	-41.40592720370	-41.40592720325
**28.390000**	-41.40592716832	-41.40592717218	-41.40592717657	-41.40592717610
**28.545714**	-41.40592714180	-41.40592714591	-41.40592715021	-41.40592714971
**28.701429**	-41.40592711631	-41.40592712013	-41.40592712460	-41.40592712406
**28.857143**	-41.40592709161	-41.40592709531	-41.40592709836	-41.40592709912
**29.012857**	-41.40592706760	-41.40592707116	-41.40592707389	-41.40592707487
**29.168571**	-41.40592704400	-41.40592704768	-41.40592705033	-41.40592705129
**29.324286**	-41.40592702129	-41.40592702460	-41.40592702742	-41.40592702835
**29.480000**	-41.40592699761	-41.40592700213	-41.40592700512	-41.40592700603
**29.635714**	-41.40592697610	-41.40592698051	-41.40592698319	-41.40592698407
**29.791429**	-41.40592695517	-41.40592695947	-41.40592696208	-41.40592696294
**29.947143**	-41.40592693480	-41.40592693899	-41.40592694153	-41.40592694237
**30.102857**	-41.40592691497	-41.40592691905	-41.40592692153	-41.40592692235
**30.258571**	-41.40592689566	-41.40592689964	-41.40592690206	-41.40592690286
**30.414286**	-41.40592687686	-41.40592688074	-41.40592688310	-41.40592688388
**30.570000**	-41.40592685855	-41.40592686233	-41.40592686463	-41.40592686539
**30.725714**	-41.40592684071	-41.40592684440	-41.40592684665	-41.40592684739
**30.881429**	-41.40592682334	-41.40592682694	-41.40592682912	-41.40592682985
**31.037143**	-41.40592680641	-41.40592680992	-41.40592681205	-41.40592681276
**31.192857**	-41.40592678992	-41.40592679334	-41.40592679542	-41.40592679611
**31.348571**	-41.40592677384	-41.40592677718	-41.40592677921	-41.40592677988
**31.504286**	-41.40592675817	-41.40592676143	-41.40592676341	-41.40592676407
**31.660000**	-41.40592674289	-41.40592674607	-41.40592674801	-41.40592674865
**31.815714**	-41.40592672800	-41.40592673110	-41.40592673299	-41.40592673362
**31.971429**	-41.40592671347	-41.40592671651	-41.40592671835	-41.40592671896
**32.127143**	-41.40592669931	-41.40592670227	-41.40592670407	-41.40592670467
**32.282857**	-41.40592668550	-41.40592668839	-41.40592669015	-41.40592669074
**32.438571**	-41.40592667203	-41.40592667485	-41.40592667657	-41.40592667714
**32.594286**	-41.40592665888	-41.40592666164	-41.40592666332	-41.40592666387
**32.750000**	-41.40592664606	-41.40592664875	-41.40592665039	-41.40592665093
**32.905714**	-41.40592663354	-41.40592663617	-41.40592663777	-41.40592663830
**33.061429**	-41.40592662133	-41.40592662390	-41.40592662546	-41.40592662598
**33.217143**	-41.40592660941	-41.40592661192	-41.40592661345	-41.40592661395
**33.372857**	-41.40592659777	-41.40592660022	-41.40592660171	-41.40592660221
**33.528571**	-41.40592658641	-41.40592658881	-41.40592659027	-41.40592659075
**33.684286**	-41.40592657532	-41.40592657766	-41.40592657909	-41.40592657956
**33.840000**	-41.40592656448	-41.40592656677	-41.40592656817	-41.40592656863
**33.995714**	-41.40592655391	-41.40592655615	-41.40592655751	-41.40592655796
**34.151429**	-41.40592654357	-41.40592654576	-41.40592654709	-41.40592654754
**34.307143**	-41.40592653348	-41.40592653562	-41.40592653692	-41.40592653736
**34.462857**	-41.40592652362	-41.40592652571	-41.40592652699	-41.40592652741
**34.618571**	-41.40592651399	-41.40592651603	-41.40592651728	-41.40592651769
**34.774286**	-41.40592650457	-41.40592650657	-41.40592650779	-41.40592650820
**34.930000**	-41.40592649537	-41.40592649733	-41.40592649852	-41.40592649892
**35.085714**	-41.40592648638	-41.40592648829	-41.40592648946	-41.40592648985
**35.241429**	-41.40592647759	-41.40592647947	-41.40592648061	-41.40592648099
**35.397143**	-41.40592646900	-41.40592647083	-41.40592647195	-41.40592647232
**35.552857**	-41.40592646060	-41.40592646239	-41.40592646349	-41.40592646385

R (Bohr)/θ(º)	150.00	160.00	170.00	180.00
**35.708571**	-41.40592645239	-41.40592645414	-41.40592645521	-41.40592645557
**35.864286**	-41.40592644435	-41.40592644608	-41.40592644712	-41.40592644747
**36.020000**	-41.40592643650	-41.40592643818	-41.40592643921	-41.40592643955
**36.175714**	-41.40592642882	-41.40592643047	-41.40592643147	-41.40592643180
**36.331429**	-41.40592642130	-41.40592642292	-41.40592642390	-41.40592642423
**36.487143**	-41.40592641395	-41.40592641553	-41.40592641650	-41.40592641682
**36.642857**	-41.40592640676	-41.40592640831	-41.40592640925	-41.40592640956
**36.798571**	-41.40592639972	-41.40592640124	-41.40592640216	-41.40592640247
**36.954286**	-41.40592639284	-41.40592639432	-41.40592639523	-41.40592639553
**37.110000**	-41.40592638610	-41.40592638756	-41.40592638844	-41.40592638873
**37.265714**	-41.40592637951	-41.40592638093	-41.40592638180	-41.40592638209
**37.421429**	-41.40592637305	-41.40592637445	-41.40592637530	-41.40592637558
**37.577143**	-41.40592636673	-41.40592636810	-41.40592636893	-41.40592636921
**37.732857**	-41.40592636055	-41.40592636189	-41.40592636271	-41.40592636297
**37.888571**	-41.40592635449	-41.40592635580	-41.40592635660	-41.40592635687
**38.044286**	-41.40592634856	-41.40592634985	-41.40592635063	-41.40592635089
**38.200000**	-41.40592634275	-41.40592634402	-41.40592634479	-41.40592634504
**38.355714**	-41.40592633707	-41.40592633830	-41.40592633905	-41.40592633931
**38.511429**	-41.40592633150	-41.40592633271	-41.40592633345	-41.40592633369
**38.667143**	-41.40592632604	-41.40592632723	-41.40592632795	-41.40592632819
**38.822857**	-41.40592632070	-41.40592632186	-41.40592632257	-41.40592632280
**38.978571**	-41.40592631546	-41.40592631660	-41.40592631730	-41.40592631753
**39.134286**	-41.40592631033	-41.40592631145	-41.40592631213	-41.40592631236
**39.290000**	-41.40592630531	-41.40592630640	-41.40592630707	-41.40592630729
**39.445714**	-41.40592630038	-41.40592630146	-41.40592630211	-41.40592630233
**39.601429**	-41.40592629556	-41.40592629661	-41.40592629725	-41.40592629746
**39.757143**	-41.40592629082	-41.40592629186	-41.40592629249	-41.40592629270
**39.912857**	-41.40592628619	-41.40592628720	-41.40592628782	-41.40592628803
**40.068571**	-41.40592628164	-41.40592628264	-41.40592628324	-41.40592628344
**40.224286**	-41.40592627718	-41.40592627816	-41.40592627876	-41.40592627896
**40.380000**	-41.40592627282	-41.40592627378	-41.40592627436	-41.40592627456
**40.535714**	-41.40592626854	-41.40592626948	-41.40592627005	-41.40592627024
**40.691429**	-41.40592626434	-41.40592626526	-41.40592626582	-41.40592626601
**40.847143**	-41.40592626022	-41.40592626113	-41.40592626168	-41.40592626186
**41.002857**	-41.40592625618	-41.40592625707	-41.40592625761	-41.40592625779
**41.158571**	-41.40592625222	-41.40592625309	-41.40592625362	-41.40592625380
**41.314286**	-41.40592624833	-41.40592624919	-41.40592624971	-41.40592624989
**41.470000**	-41.40592624452	-41.40592624536	-41.40592624588	-41.40592624605
**41.625714**	-41.40592624079	-41.40592624161	-41.40592624212	-41.40592624228
**41.781429**	-41.40592623712	-41.40592623793	-41.40592623842	-41.40592623859
**41.937143**	-41.40592623352	-41.40592623432	-41.40592623480	-41.40592623497
**42.092857**	-41.40592622999	-41.40592623077	-41.40592623125	-41.40592623141
**42.248571**	-41.40592622653	-41.40592622730	-41.40592622776	-41.40592622792
**42.404286**	-41.40592622313	-41.40592622388	-41.40592622434	-41.40592622449
**42.560000**	-41.40592621979	-41.40592622053	-41.40592622098	-41.40592622113
**42.715714**	-41.40592621652	-41.40592621724	-41.40592621769	-41.40592621783
**42.871429**	-41.40592621330	-41.40592621402	-41.40592621445	-41.40592621460
**43.027143**	-41.40592621015	-41.40592621085	-41.40592621128	-41.40592621142
**43.182857**	-41.40592620705	-41.40592620774	-41.40592620816	-41.40592620830
**43.338571**	-41.40592620401	-41.40592620469	-41.40592620510	-41.40592620524
**43.494286**	-41.40592620103	-41.40592620169	-41.40592620209	-41.40592620223
**43.650000**	-41.40592619809	-41.40592619875	-41.40592619915	-41.40592619928
**43.805714**	-41.40592619522	-41.40592619586	-41.40592619625	-41.40592619638
**43.961429**	-41.40592619239	-41.40592619302	-41.40592619340	-41.40592619353
**44.117143**	-41.40592618961	-41.40592619023	-41.40592619061	-41.40592619074
**44.272857**	-41.40592618688	-41.40592618750	-41.40592618787	-41.40592618799
**44.428571**	-41.40592618421	-41.40592618481	-41.40592618517	-41.40592618529
**44.584286**	-41.40592618158	-41.40592618217	-41.40592618253	-41.40592618265
**44.740000**	-41.40592617899	-41.40592617957	-41.40592617993	-41.40592618004
**44.895714**	-41.40592617646	-41.40592617703	-41.40592617737	-41.40592617749
**45.051429**	-41.40592617396	-41.40592617452	-41.40592617486	-41.40592617498
**45.207143**	-41.40592617151	-41.40592617206	-41.40592617240	-41.40592617251
**45.362857**	-41.40592616911	-41.40592616965	-41.40592616998	-41.40592617008
**45.518571**	-41.40592616674	-41.40592616727	-41.40592616760	-41.40592616770
**45.674286**	-41.40592616442	-41.40592616494	-41.40592616526	-41.40592616536

R (Bohr)/θ(º)	150.00	160.00	170.00	180.00
**45.830000**	-41.40592616213	-41.40592616265	-41.40592616296	-41.40592616306
**45.985714**	-41.40592615989	-41.40592616039	-41.40592616070	-41.40592616080
**46.141429**	-41.40592615768	-41.40592615818	-41.40592615848	-41.40592615858
**46.297143**	-41.40592615551	-41.40592615600	-41.40592615630	-41.40592615640
**46.452857**	-41.40592615338	-41.40592615386	-41.40592615416	-41.40592615425
**46.608571**	-41.40592615128	-41.40592615176	-41.40592615205	-41.40592615214
**46.764286**	-41.40592614922	-41.40592614969	-41.40592614997	-41.40592615007
**46.920000**	-41.40592614720	-41.40592614766	-41.40592614794	-41.40592614803
**47.075714**	-41.40592614521	-41.40592614566	-41.40592614593	-41.40592614602
**47.231429**	-41.40592614325	-41.40592614369	-41.40592614396	-41.40592614405
**47.387143**	-41.40592614132	-41.40592614176	-41.40592614203	-41.40592614211
**47.542857**	-41.40592613943	-41.40592613986	-41.40592614012	-41.40592614021
**47.698571**	-41.40592613757	-41.40592613799	-41.40592613825	-41.40592613833
**47.854286**	-41.40592613573	-41.40592613616	-41.40592613641	-41.40592613649
**48.010000**	-41.40592613394	-41.40592613434	-41.40592613459	-41.40592613468
**48.165714**	-41.40592613216	-41.40592613257	-41.40592613281	-41.40592613289
**48.321429**	-41.40592613042	-41.40592613082	-41.40592613106	-41.40592613114
**48.477143**	-41.40592612871	-41.40592612910	-41.40592612933	-41.40592612941
**48.632857**	-41.40592612702	-41.40592612740	-41.40592612764	-41.40592612771
**48.788571**	-41.40592612536	-41.40592612574	-41.40592612597	-41.40592612605
**48.944286**	-41.40592612373	-41.40592612410	-41.40592612433	-41.40592612440
**49.100000**	-41.40592612212	-41.40592612249	-41.40592612271	-41.40592612279
**49.255714**	-41.40592612054	-41.40592612090	-41.40592612112	-41.40592612120
**49.411429**	-41.40592611899	-41.40592611934	-41.40592611956	-41.40592611963
**49.567143**	-41.40592611746	-41.40592611781	-41.40592611802	-41.40592611809
**49.722857**	-41.40592611595	-41.40592611630	-41.40592611651	-41.40592611658
**49.878571**	-41.40592611447	-41.40592611481	-41.40592611502	-41.40592611509
**50.034286**	-41.40592611301	-41.40592611334	-41.40592611355	-41.40592611362
**50.190000**	-41.40592611157	-41.40592611191	-41.40592611211	-41.40592611217
**50.345714**	-41.40592611016	-41.40592611049	-41.40592611068	-41.40592611075
**50.501429**	-41.40592610877	-41.40592610909	-41.40592610929	-41.40592610935
**50.657143**	-41.40592610740	-41.40592610772	-41.40592610791	-41.40592610797
**50.812857**	-41.40592610606	-41.40592610636	-41.40592610655	-41.40592610662
**50.968571**	-41.40592610473	-41.40592610503	-41.40592610522	-41.40592610528
**51.124286**	-41.40592610342	-41.40592610372	-41.40592610391	-41.40592610397
**51.280000**	-41.40592610213	-41.40592610243	-41.40592610261	-41.40592610267
**51.435714**	-41.40592610087	-41.40592610116	-41.40592610134	-41.40592610140
**51.591429**	-41.40592609962	-41.40592609991	-41.40592610009	-41.40592610014
**51.747143**	-41.40592609839	-41.40592609868	-41.40592609885	-41.40592609891
**51.902857**	-41.40592609718	-41.40592609746	-41.40592609763	-41.40592609769
**52.058571**	-41.40592609600	-41.40592609627	-41.40592609644	-41.40592609649
**52.214286**	-41.40592609482	-41.40592609509	-41.40592609526	-41.40592609531
**52.370000**	-41.40592609367	-41.40592609393	-41.40592609409	-41.40592609415
**52.525714**	-41.40592609253	-41.40592609279	-41.40592609295	-41.40592609301
**52.681429**	-41.40592609141	-41.40592609167	-41.40592609183	-41.40592609188
**52.837143**	-41.40592609030	-41.40592609056	-41.40592609072	-41.40592609077
**52.992857**	-41.40592608922	-41.40592608947	-41.40592608962	-41.40592608967
**53.148571**	-41.40592608815	-41.40592608839	-41.40592608855	-41.40592608859
**53.304286**	-41.40592608709	-41.40592608733	-41.40592608748	-41.40592608753
**53.460000**	-41.40592608605	-41.40592608629	-41.40592608644	-41.40592608649
**53.615714**	-41.40592608503	-41.40592608526	-41.40592608541	-41.40592608546
**53.771429**	-41.40592608402	-41.40592608425	-41.40592608440	-41.40592608444
**53.927143**	-41.40592608302	-41.40592608325	-41.40592608340	-41.40592608344
**54.082857**	-41.40592608204	-41.40592608227	-41.40592608241	-41.40592608245
**54.238571**	-41.40592608107	-41.40592608130	-41.40592608144	-41.40592608148
**54.394286**	-41.40592608012	-41.40592608034	-41.40592608048	-41.40592608052
**54.550000**	-41.40592607919	-41.40592607940	-41.40592607954	-41.40592607958
**54.705714**	-41.40592607826	-41.40592607848	-41.40592607861	-41.40592607865
**54.861429**	-41.40592607735	-41.40592607756	-41.40592607769	-41.40592607774
**55.017143**	-41.40592607645	-41.40592607666	-41.40592607679	-41.40592607683
**55.172857**	-41.40592607557	-41.40592607578	-41.40592607590	-41.40592607594
**55.328571**	-41.40592607469	-41.40592607490	-41.40592607502	-41.40592607506
**55.484286**	-41.40592607383	-41.40592607404	-41.40592607416	-41.40592607420
**55.640000**	-41.40592607299	-41.40592607319	-41.40592607331	-41.40592607335
**55.795714**	-41.40592607215	-41.40592607235	-41.40592607247	-41.40592607251

R (Bohr)/θ(º)	150.00	160.00	170.00	180.00
**55.951429**	-41.40592607133	-41.40592607152	-41.40592607164	-41.40592607168
**56.107143**	-41.40592607052	-41.40592607070	-41.40592607082	-41.40592607086
**56.262857**	-41.40592606972	-41.40592606990	-41.40592607002	-41.40592607006
**56.418571**	-41.40592606893	-41.40592606911	-41.40592606922	-41.40592606926
**56.574286**	-41.40592606815	-41.40592606833	-41.40592606844	-41.40592606848
**56.730000**	-41.40592606738	-41.40592606756	-41.40592606767	-41.40592606771
**56.885714**	-41.40592606662	-41.40592606680	-41.40592606691	-41.40592606694
**57.041429**	-41.40592606587	-41.40592606605	-41.40592606616	-41.40592606619
**57.197143**	-41.40592606514	-41.40592606531	-41.40592606542	-41.40592606545
**57.352857**	-41.40592606442	-41.40592606458	-41.40592606469	-41.40592606472
**57.508571**	-41.40592606370	-41.40592606387	-41.40592606397	-41.40592606401
**57.664286**	-41.40592606299	-41.40592606316	-41.40592606326	-41.40592606329
**57.820000**	-41.40592606229	-41.40592606246	-41.40592606256	-41.40592606259
**57.975714**	-41.40592606161	-41.40592606177	-41.40592606187	-41.40592606190
**58.131429**	-41.40592606093	-41.40592606109	-41.40592606119	-41.40592606122
**58.287143**	-41.40592606026	-41.40592606042	-41.40592606052	-41.40592606055
**58.442857**	-41.40592605960	-41.40592605976	-41.40592605985	-41.40592605989
**58.598571**	-41.40592605895	-41.40592605910	-41.40592605920	-41.40592605923
**58.754286**	-41.40592605831	-41.40592605846	-41.40592605855	-41.40592605858
**58.910000**	-41.40592605768	-41.40592605783	-41.40592605792	-41.40592605795
**59.065714**	-41.40592605705	-41.40592605720	-41.40592605729	-41.40592605732
**59.221429**	-41.40592605643	-41.40592605658	-41.40592605667	-41.40592605670
**59.377143**	-41.40592605583	-41.40592605597	-41.40592605606	-41.40592605609
**59.532857**	-41.40592605523	-41.40592605537	-41.40592605546	-41.40592605549
**59.688571**	-41.40592605463	-41.40592605477	-41.40592605486	-41.40592605224
**59.844286**	-41.40592605405	-41.40592605419	-41.40592605428	-41.40615368545
**60.000000**	-41.40592605198	-41.40592605290	-41.40592605020	-41.40615368504

PES CBS – CH^- ($X\ ^3\Sigma^-$) + He					
**R (Bohr)/θ(º)**	**0.00**	**10.00**	**20.00**	**30.00**	**40.00**
**5.5000000**	-41.4076413813	-41.4080327569	-41.4090026503	-41.4100960360	-41.4109529546
**5.6557143**	-41.4088766406	-41.4091765273	-41.4099235289	-41.4107650394	-41.4114194340
**5.8114286**	-41.4098391499	-41.4100601122	-41.4106343645	-41.4112839517	-41.4117804828
**5.9671429**	-41.4105486156	-41.4107441979	-41.4111840235	-41.4116830827	-41.4120587707
**6.1228571**	-41.4111143137	-41.4112733922	-41.4116083855	-41.4119891733	-41.4122731147
**6.2785714**	-41.4115597435	-41.4116816471	-41.4119350935	-41.4122240800	-41.4124384317
**6.4342857**	-41.4118981491	-41.4119954611	-41.4121859518	-41.4124047792	-41.4125661398
**6.5900000**	-41.4121521153	-41.4122357924	-41.4123782253	-41.4125440422	-41.4126647899
**6.7457143**	-41.4123448479	-41.4124192618	-41.4125254708	-41.4126513391	-41.4127407464
**6.9014286**	-41.4124933207	-41.4125589794	-41.4126381865	-41.4127337076	-41.4127987966
**7.0571429**	-41.4126081251	-41.4126651782	-41.4127243843	-41.4127964650	-41.4128426297
**7.2128571**	-41.4126960816	-41.4127457438	-41.4127901101	-41.4128437276	-41.4128751737
**7.3685714**	-41.4127624232	-41.4128066758	-41.4128399030	-41.4128787596	-41.4128988113
**7.5242857**	-41.4128117169	-41.4128524919	-41.4128771813	-41.4129041910	-41.4129155045
**7.6800000**	-41.4128479443	-41.4128865752	-41.4129045507	-41.4129221510	-41.4129268636
**7.8357143**	-41.4128743414	-41.4129114621	-41.4129240364	-41.4129343513	-41.4129341881
**7.9914286**	-41.4128933363	-41.4129290727	-41.4129372489	-41.4129421481	-41.4129384984
**8.1471429**	-41.4129066511	-41.4129408870	-41.4129454959	-41.4129465950	-41.4129405689
**8.3028571**	-41.4129154992	-41.4129480736	-41.4129498556	-41.4129484943	-41.4129409666
**8.4585714**	-41.4129207837	-41.4129515801	-41.4129512219	-41.4129484478	-41.4129400931
**8.6142857**	-41.4129232420	-41.4129521924	-41.4129503338	-41.4129469045	-41.4129382271
**8.7700000**	-41.4129235219	-41.4129505729	-41.4129477962	-41.4129442027	-41.4129355642
**8.9257143**	-41.4129222038	-41.4129472830	-41.4129440957	-41.4129406051	-41.4129322491
**9.0814286**	-41.4129197931	-41.4129427971	-41.4129396154	-41.4129363249	-41.4129284013
**9.2371429**	-41.4129167053	-41.4129375112	-41.4129346502	-41.4129315436	-41.4129241308
**9.3928571**	-41.4129132566	-41.4129317499	-41.4129294210	-41.4129264221	-41.4129195471
**9.5485714**	-41.4129096658	-41.4129257719	-41.4129240889	-41.4129211051	-41.4129147614
**9.7042857**	-41.4129060671	-41.4129197771	-41.4129187684	-41.4129157225	-41.4129098844
**9.8600000**	-41.4129025285	-41.4129139131	-41.4129135392	-41.4129103881	-41.4129050224
**10.0157143**	-41.4128990735	-41.4129082830	-41.4129084555	-41.4129051979	-41.4129002717
**10.1714286**	-41.4128956999	-41.4129029530	-41.4129035538	-41.4129002280	-41.4128957138
**10.3271429**	-41.4128923958	-41.4128979598	-41.4128988584	-41.4128955343	-41.4128914124
**10.4828571**	-41.4128891498	-41.4128933174	-41.4128943860	-41.4128911525	-41.4128874110
**10.6385714**	-41.4128859570	-41.4128890234	-41.4128901471	-41.4128871000	-41.4128837332
**10.7942857**	-41.4128828210	-41.4128850643	-41.4128861488	-41.4128833779	-41.4128803846
**10.9500000**	-41.4128797536	-41.4128814192	-41.4128823943	-41.4128799743	-41.4128773546
**11.1057143**	-41.4128767719	-41.4128780638	-41.4128788842	-41.4128768677	-41.4128746206
**11.2614286**	-41.4128738962	-41.4128749725	-41.4128756159	-41.4128740300	-41.4128721516
**11.4171429**	-41.4128711465	-41.4128721200	-41.4128725841	-41.4128714303	-41.4128699114
**11.5728571**	-41.4128685407	-41.4128694827	-41.4128697806	-41.4128690368	-41.4128678626
**11.7285714**	-41.4128660925	-41.4128670389	-41.4128671948	-41.4128668192	-41.4128659690
**11.8842857**	-41.4128638109	-41.4128647694	-41.4128648140	-41.4128647505	-41.4128641977
**12.0400000**	-41.4128616995	-41.4128626573	-41.4128626237	-41.4128628071	-41.4128625206
**12.1957143**	-41.4128597569	-41.4128606879	-41.4128606085	-41.4128609703	-41.4128609153
**12.3514286**	-41.4128579774	-41.4128588485	-41.4128587523	-41.4128592254	-41.4128593652
**12.5071429**	-41.4128563518	-41.4128571279	-41.4128570390	-41.4128575620	-41.4128578589
**12.6628571**	-41.4128548683	-41.4128555167	-41.4128554530	-41.4128559729	-41.4128563903
**12.8185714**	-41.4128535133	-41.4128540062	-41.4128539797	-41.4128544540	-41.4128549571
**12.9742857**	-41.4128522726	-41.4128525891	-41.4128526056	-41.4128530033	-41.4128535604
**13.1300000**	-41.4128511317	-41.4128512585	-41.4128513189	-41.4128516203	-41.4128522035
**13.2857143**	-41.4128500767	-41.4128500083	-41.4128501090	-41.4128503052	-41.4128508910
**13.4414286**	-41.4128490946	-41.4128488332	-41.4128489673	-41.4128490587	-41.4128496279
**13.5971429**	-41.4128481737	-41.4128477279	-41.4128478865	-41.4128478812	-41.4128484194
**13.7528571**	-41.4128473036	-41.4128466877	-41.4128468608	-41.4128467729	-41.4128472698
**13.9085714**	-41.4128464753	-41.4128457084	-41.4128458856	-41.4128457331	-41.4128461825
**14.0642857**	-41.4128456815	-41.4128447860	-41.4128449575	-41.4128447607	-41.4128451599
**14.2200000**	-41.4128449162	-41.4128439168	-41.4128440740	-41.4128438536	-41.4128442030
**14.3757143**	-41.4128441745	-41.4128430973	-41.4128432330	-41.4128430092	-41.4128433112
**14.5314286**	-41.4128434529	-41.4128423244	-41.4128424333	-41.4128422241	-41.4128424833
**14.6871429**	-41.4128427489	-41.4128415951	-41.4128416737	-41.4128414946	-41.4128417167
**14.8428571**	-41.4128420605	-41.4128409067	-41.4128409533	-41.4128408166	-41.4128410078
**14.9985714**	-41.4128413869	-41.4128402566	-41.4128402712	-41.4128401856	-41.4128403528
**15.1542857**	-41.4128407274	-41.4128396425	-41.4128396267	-41.4128395974	-41.4128397471
**15.3100000**	-41.4128400819	-41.4128390622	-41.4128390186	-41.4128390475	-41.4128391860

R (Bohr)/θ(º)	0.00	10.00	20.00	30.00	40.00
**15.4657143**	-41.4128394507	-41.4128385135	-41.4128384459	-41.4128385319	-41.4128386647
**15.6214286**	-41.4128388342	-41.4128379946	-41.4128379072	-41.4128380465	-41.4128381785
**15.7771429**	-41.4128382330	-41.4128375036	-41.4128374011	-41.4128375878	-41.4128377228
**15.9328571**	-41.4128376475	-41.4128370387	-41.4128369261	-41.4128371524	-41.4128372936
**16.0885714**	-41.4128370786	-41.4128365983	-41.4128364803	-41.4128367377	-41.4128368870
**16.2442857**	-41.4128365267	-41.4128361808	-41.4128360619	-41.4128363410	-41.4128364997
**16.4000000**	-41.4128359924	-41.4128357849	-41.4128356691	-41.4128359603	-41.4128361289
**16.5557143**	-41.4128354762	-41.4128354090	-41.4128353000	-41.4128355940	-41.4128357720
**16.7114286**	-41.4128349785	-41.4128350520	-41.4128349527	-41.4128352405	-41.4128354272
**16.8671429**	-41.4128344994	-41.4128347124	-41.4128346252	-41.4128348990	-41.4128350929
**17.0228571**	-41.4128340391	-41.4128343893	-41.4128343158	-41.4128345686	-41.4128347679
**17.1785714**	-41.4128335978	-41.4128340814	-41.4128340229	-41.4128342487	-41.4128344514
**17.3342857**	-41.4128331753	-41.4128337879	-41.4128337448	-41.4128339391	-41.4128341429
**17.4900000**	-41.4128327716	-41.4128335077	-41.4128334801	-41.4128336394	-41.4128338421
**17.6457143**	-41.4128323864	-41.4128332401	-41.4128332274	-41.4128333496	-41.4128335489
**17.8014286**	-41.4128320193	-41.4128329841	-41.4128329856	-41.4128330698	-41.4128332635
**17.9571429**	-41.4128316702	-41.4128327391	-41.4128327537	-41.4128327999	-41.4128329859
**18.1128571**	-41.4128313384	-41.4128325045	-41.4128325307	-41.4128325400	-41.4128327164
**18.2685714**	-41.4128310237	-41.4128322795	-41.4128323158	-41.4128322902	-41.4128324553
**18.4242857**	-41.4128307254	-41.4128320637	-41.4128321085	-41.4128320506	-41.4128322030
**18.5800000**	-41.4128304430	-41.4128318566	-41.4128319081	-41.4128318211	-41.4128319597
**18.7357143**	-41.4128301760	-41.4128316578	-41.4128317143	-41.4128316019	-41.4128317257
**18.8914286**	-41.4128299237	-41.4128314667	-41.4128315267	-41.4128313929	-41.4128315012
**19.0471429**	-41.4128296857	-41.4128312832	-41.4128313450	-41.4128311939	-41.4128312865
**19.2028571**	-41.4128294612	-41.4128311068	-41.4128311691	-41.4128310048	-41.4128310815
**19.3585714**	-41.4128292497	-41.4128309373	-41.4128309987	-41.4128308255	-41.4128308863
**19.5142857**	-41.4128290507	-41.4128307743	-41.4128308337	-41.4128306558	-41.4128307009
**19.6700000**	-41.4128288634	-41.4128306178	-41.4128306742	-41.4128304953	-41.4128305253
**19.8257143**	-41.4128286874	-41.4128304674	-41.4128305200	-41.4128303437	-41.4128303592
**19.9814286**	-41.4128285220	-41.4128303229	-41.4128303710	-41.4128302007	-41.4128302026
**20.1371429**	-41.4128283668	-41.4128301841	-41.4128302273	-41.4128300659	-41.4128300550
**20.2928571**	-41.4128282211	-41.4128300510	-41.4128300888	-41.4128299390	-41.4128299162
**20.4485714**	-41.4128280845	-41.4128299232	-41.4128299556	-41.4128298195	-41.4128297859
**20.6042857**	-41.4128279565	-41.4128298006	-41.4128298274	-41.4128297070	-41.4128296637
**20.7600000**	-41.4128278365	-41.4128296830	-41.4128297044	-41.4128296010	-41.4128295492
**20.9157143**	-41.4128277241	-41.4128295704	-41.4128295863	-41.4128295012	-41.4128294420
**21.0714286**	-41.4128276190	-41.4128294625	-41.4128294733	-41.4128294071	-41.4128293416
**21.2271429**	-41.4128275206	-41.4128293591	-41.4128293651	-41.4128293182	-41.4128292477
**21.3828571**	-41.4128274285	-41.4128292602	-41.4128292616	-41.4128292343	-41.4128291597
**21.5385714**	-41.4128273424	-41.4128291655	-41.4128291628	-41.4128291548	-41.4128290773
**21.6942857**	-41.4128272620	-41.4128290749	-41.4128290685	-41.4128290794	-41.4128290000
**21.8500000**	-41.4128271868	-41.4128289882	-41.4128289786	-41.4128290078	-41.4128289275
**22.0057143**	-41.4128271165	-41.4128289052	-41.4128288929	-41.4128289396	-41.4128288593
**22.1614286**	-41.4128270509	-41.4128288259	-41.4128288112	-41.4128288744	-41.4128287950
**22.3171429**	-41.4128269896	-41.4128287500	-41.4128287335	-41.4128288120	-41.4128287342
**22.4728571**	-41.4128269324	-41.4128286774	-41.4128286594	-41.4128287521	-41.4128286767
**22.6285714**	-41.4128268790	-41.4128286080	-41.4128285889	-41.4128286945	-41.4128286221
**22.7842857**	-41.4128268292	-41.4128285415	-41.4128285218	-41.4128286388	-41.4128285700
**22.9400000**	-41.4128267827	-41.4128284778	-41.4128284579	-41.4128285849	-41.4128285203
**23.0957143**	-41.4128267393	-41.4128284168	-41.4128283969	-41.4128285327	-41.4128284726
**23.2514286**	-41.4128266989	-41.4128283584	-41.4128283388	-41.4128284818	-41.4128284267
**23.4071429**	-41.4128266611	-41.4128283023	-41.4128282833	-41.4128284322	-41.4128283824
**23.5628571**	-41.4128266259	-41.4128282484	-41.4128282303	-41.4128283837	-41.4128283395
**23.7185714**	-41.4128265931	-41.4128281967	-41.4128281797	-41.4128283362	-41.4128282977
**23.8742857**	-41.4128265625	-41.4128281470	-41.4128281311	-41.4128282896	-41.4128282571
**24.0300000**	-41.4128265340	-41.4128280992	-41.4128280846	-41.4128282438	-41.4128282173
**24.1857143**	-41.4128265074	-41.4128280530	-41.4128280400	-41.4128281987	-41.4128281784
**24.3414286**	-41.4128264827	-41.4128280086	-41.4128279970	-41.4128281543	-41.4128281401
**24.4971429**	-41.4128264596	-41.4128279656	-41.4128279557	-41.4128281105	-41.4128281024
**24.6528571**	-41.4128264381	-41.4128279241	-41.4128279157	-41.4128280672	-41.4128280652
**24.8085714**	-41.4128264180	-41.4128278839	-41.4128278772	-41.4128280245	-41.4128280284
**24.9642857**	-41.4128263993	-41.4128278450	-41.4128278399	-41.4128279823	-41.4128279920
**25.1200000**	-41.4128263819	-41.4128278072	-41.4128278037	-41.4128279405	-41.4128279560
**25.2757143**	-41.4128263657	-41.4128277705	-41.4128277685	-41.4128278992	-41.4128279202
**25.4314286**	-41.4128263506	-41.4128277349	-41.4128277343	-41.4128278583	-41.4128278848

R (Bohr)/θ(º)	0.00	10.00	20.00	30.00	40.00
**25.5871429**	-41.4128263365	-41.4128277001	-41.4128277010	-41.4128278179	-41.4128278495
**25.7428571**	-41.4128263233	-41.4128276663	-41.4128276685	-41.4128277779	-41.4128278146
**25.8985714**	-41.4128263111	-41.4128276333	-41.4128276367	-41.4128277383	-41.4128277798
**26.0542857**	-41.4128262997	-41.4128276010	-41.4128276056	-41.4128276992	-41.4128277453
**26.2100000**	-41.4128262891	-41.4128275695	-41.4128275751	-41.4128276606	-41.4128277110
**26.3657143**	-41.4128262792	-41.4128275387	-41.4128275452	-41.4128276225	-41.4128276769
**26.5214286**	-41.4128262700	-41.4128275085	-41.4128275159	-41.4128275848	-41.4128276431
**26.6771429**	-41.4128262614	-41.4128274789	-41.4128274870	-41.4128275476	-41.4128276095
**26.8328571**	-41.4128262534	-41.4128274498	-41.4128274587	-41.4128275109	-41.4128275762
**26.9885714**	-41.4128262460	-41.4128274214	-41.4128274308	-41.4128274747	-41.4128275431
**27.1442857**	-41.4128262390	-41.4128273934	-41.4128274033	-41.4128274391	-41.4128275104
**27.3000000**	-41.4128262325	-41.4128273660	-41.4128273762	-41.4128274040	-41.4128274780
**27.4557143**	-41.4128262265	-41.4128273390	-41.4128273495	-41.4128273695	-41.4128274458
**27.6114286**	-41.4128262209	-41.4128273124	-41.4128273231	-41.4128273355	-41.4128274141
**27.7671429**	-41.4128262156	-41.4128272863	-41.4128272972	-41.4128273021	-41.4128273826
**27.9228571**	-41.4128262108	-41.4128272607	-41.4128272715	-41.4128272693	-41.4128273516
**28.0785714**	-41.4128262062	-41.4128272354	-41.4128272463	-41.4128272371	-41.4128273209
**28.2342857**	-41.4128262020	-41.4128272106	-41.4128272213	-41.4128272054	-41.4128272906
**28.3900000**	-41.4128261980	-41.4128271861	-41.4128271967	-41.4128271744	-41.4128272608
**28.5457143**	-41.4128261943	-41.4128271621	-41.4128271725	-41.4128271440	-41.4128272314
**28.7014286**	-41.4128261909	-41.4128271384	-41.4128271485	-41.4128271142	-41.4128272024
**28.8571429**	-41.4128261876	-41.4128271151	-41.4128271249	-41.4128270851	-41.4128271738
**29.0128571**	-41.4128261847	-41.4128270922	-41.4128271016	-41.4128270565	-41.4128271458
**29.1685714**	-41.4128261819	-41.4128270696	-41.4128270787	-41.4128270286	-41.4128271182
**29.3242857**	-41.4128261792	-41.4128270474	-41.4128270561	-41.4128270013	-41.4128270911
**29.4800000**	-41.4128261768	-41.4128270256	-41.4128270338	-41.4128269747	-41.4128270645
**29.6357143**	-41.4128261745	-41.4128270041	-41.4128270118	-41.4128269487	-41.4128270384
**29.7914286**	-41.4128261724	-41.4128269830	-41.4128269902	-41.4128269233	-41.4128270127
**29.9471429**	-41.4128261704	-41.4128269622	-41.4128269690	-41.4128268985	-41.4128269876
**30.1028571**	-41.4128261686	-41.4128269418	-41.4128269480	-41.4128268743	-41.4128269630
**30.2585714**	-41.4128261669	-41.4128269217	-41.4128269275	-41.4128268507	-41.4128269389
**30.4142857**	-41.4128261653	-41.4128269020	-41.4128269072	-41.4128268278	-41.4128269154
**30.5700000**	-41.4128261637	-41.4128268826	-41.4128268874	-41.4128268054	-41.4128268923
**30.7257143**	-41.4128261623	-41.4128268636	-41.4128268678	-41.4128267837	-41.4128268698
**30.8814286**	-41.4128261610	-41.4128268450	-41.4128268487	-41.4128267625	-41.4128268477
**31.0371429**	-41.4128261598	-41.4128268267	-41.4128268299	-41.4128267419	-41.4128268262
**31.1928571**	-41.4128261586	-41.4128268087	-41.4128268114	-41.4128267219	-41.4128268052
**31.3485714**	-41.4128261576	-41.4128267911	-41.4128267933	-41.4128267024	-41.4128267847
**31.5042857**	-41.4128261566	-41.4128267738	-41.4128267755	-41.4128266835	-41.4128267647
**31.6600000**	-41.4128261556	-41.4128267568	-41.4128267581	-41.4128266651	-41.4128267452
**31.8157143**	-41.4128261547	-41.4128267402	-41.4128267411	-41.4128266473	-41.4128267262
**31.9714286**	-41.4128261539	-41.4128267240	-41.4128267244	-41.4128266300	-41.4128267077
**32.1271429**	-41.4128261531	-41.4128267080	-41.4128267080	-41.4128266132	-41.4128266897
**32.2828571**	-41.4128261524	-41.4128266925	-41.4128266921	-41.4128265969	-41.4128266721
**32.4385714**	-41.4128261517	-41.4128266772	-41.4128266764	-41.4128265811	-41.4128266550
**32.5942857**	-41.4128261511	-41.4128266623	-41.4128266611	-41.4128265657	-41.4128266384
**32.7500000**	-41.4128261505	-41.4128266476	-41.4128266462	-41.4128265509	-41.4128266223
**32.9057143**	-41.4128261499	-41.4128266334	-41.4128266316	-41.4128265365	-41.4128266065
**33.0614286**	-41.4128261494	-41.4128266194	-41.4128266173	-41.4128265226	-41.4128265913
**33.2171429**	-41.4128261489	-41.4128266057	-41.4128266034	-41.4128265091	-41.4128265764
**33.3728571**	-41.4128261484	-41.4128265924	-41.4128265898	-41.4128264960	-41.4128265620
**33.5285714**	-41.4128261480	-41.4128265794	-41.4128265765	-41.4128264834	-41.4128265480
**33.6842857**	-41.4128261476	-41.4128265666	-41.4128265635	-41.4128264711	-41.4128265344
**33.8400000**	-41.4128261472	-41.4128265542	-41.4128265509	-41.4128264593	-41.4128265212
**33.9957143**	-41.4128261468	-41.4128265421	-41.4128265386	-41.4128264478	-41.4128265084
**34.1514286**	-41.4128261465	-41.4128265302	-41.4128265266	-41.4128264367	-41.4128264960
**34.3071429**	-41.4128261462	-41.4128265187	-41.4128265149	-41.4128264260	-41.4128264840
**34.4628571**	-41.4128261459	-41.4128265074	-41.4128265035	-41.4128264157	-41.4128264723
**34.6185714**	-41.4128261456	-41.4128264964	-41.4128264924	-41.4128264056	-41.4128264610
**34.7742857**	-41.4128261453	-41.4128264857	-41.4128264816	-41.4128263960	-41.4128264500
**34.9300000**	-41.4128261451	-41.4128264753	-41.4128264711	-41.4128263866	-41.4128264394
**35.0857143**	-41.4128261448	-41.4128264651	-41.4128264609	-41.4128263776	-41.4128264291
**35.2414286**	-41.4128261446	-41.4128264552	-41.4128264509	-41.4128263688	-41.4128264191
**35.3971429**	-41.4128261444	-41.4128264455	-41.4128264412	-41.4128263604	-41.4128264095
**35.5528571**	-41.4128261442	-41.4128264361	-41.4128264318	-41.4128263523	-41.4128264001

R (Bohr)/θ(º)	0.00	10.00	20.00	30.00	40.00
**35.7085714**	-41.4128261440	-41.4128264270	-41.4128264227	-41.4128263444	-41.4128263911
**35.8642857**	-41.4128261438	-41.4128264181	-41.4128264138	-41.4128263368	-41.4128263823
**36.0200000**	-41.4128261436	-41.4128264094	-41.4128264051	-41.4128263295	-41.4128263738
**36.1757143**	-41.4128261435	-41.4128264010	-41.4128263967	-41.4128263224	-41.4128263656
**36.3314286**	-41.4128261433	-41.4128263927	-41.4128263886	-41.4128263156	-41.4128263577
**36.4871429**	-41.4128261432	-41.4128263848	-41.4128263806	-41.4128263091	-41.4128263500
**36.6428571**	-41.4128261430	-41.4128263770	-41.4128263729	-41.4128263027	-41.4128263426
**36.7985714**	-41.4128261429	-41.4128263694	-41.4128263655	-41.4128262966	-41.4128263354
**36.9542857**	-41.4128261428	-41.4128263621	-41.4128263582	-41.4128262907	-41.4128263285
**37.1100000**	-41.4128261427	-41.4128263549	-41.4128263512	-41.4128262850	-41.4128263217
**37.2657143**	-41.4128261426	-41.4128263480	-41.4128263443	-41.4128262795	-41.4128263153
**37.4214286**	-41.4128261424	-41.4128263413	-41.4128263377	-41.4128262742	-41.4128263090
**37.5771429**	-41.4128261423	-41.4128263347	-41.4128263313	-41.4128262691	-41.4128263029
**37.7328571**	-41.4128261422	-41.4128263283	-41.4128263250	-41.4128262642	-41.4128262971
**37.8885714**	-41.4128261422	-41.4128263222	-41.4128263190	-41.4128262594	-41.4128262914
**38.0442857**	-41.4128261421	-41.4128263162	-41.4128263131	-41.4128262548	-41.4128262860
**38.2000000**	-41.4128261420	-41.4128263103	-41.4128263074	-41.4128262504	-41.4128262807
**38.3557143**	-41.4128261419	-41.4128263047	-41.4128263019	-41.4128262462	-41.4128262756
**38.5114286**	-41.4128261418	-41.4128262992	-41.4128262966	-41.4128262421	-41.4128262707
**38.6671429**	-41.4128261417	-41.4128262938	-41.4128262914	-41.4128262382	-41.4128262659
**38.8228571**	-41.4128261417	-41.4128262886	-41.4128262864	-41.4128262344	-41.4128262613
**38.9785714**	-41.4128261416	-41.4128262836	-41.4128262815	-41.4128262307	-41.4128262569
**39.1342857**	-41.4128261415	-41.4128262787	-41.4128262768	-41.4128262272	-41.4128262526
**39.2900000**	-41.4128261415	-41.4128262740	-41.4128262722	-41.4128262238	-41.4128262485
**39.4457143**	-41.4128261414	-41.4128262694	-41.4128262678	-41.4128262205	-41.4128262445
**39.6014286**	-41.4128261414	-41.4128262650	-41.4128262635	-41.4128262173	-41.4128262406
**39.7571429**	-41.4128261413	-41.4128262606	-41.4128262594	-41.4128262143	-41.4128262369
**39.9128571**	-41.4128261413	-41.4128262564	-41.4128262554	-41.4128262114	-41.4128262333
**40.0685714**	-41.4128261412	-41.4128262524	-41.4128262515	-41.4128262086	-41.4128262299
**40.2242857**	-41.4128261412	-41.4128262484	-41.4128262477	-41.4128262059	-41.4128262265
**40.3800000**	-41.4128261411	-41.4128262446	-41.4128262441	-41.4128262033	-41.4128262233
**40.5357143**	-41.4128261411	-41.4128262409	-41.4128262406	-41.4128262007	-41.4128262202
**40.6914286**	-41.4128261410	-41.4128262373	-41.4128262371	-41.4128261983	-41.4128262172
**40.8471429**	-41.4128261410	-41.4128262338	-41.4128262338	-41.4128261960	-41.4128262143
**41.0028571**	-41.4128261409	-41.4128262305	-41.4128262307	-41.4128261938	-41.4128262115
**41.1585714**	-41.4128261409	-41.4128262272	-41.4128262276	-41.4128261916	-41.4128262089
**41.3142857**	-41.4128261409	-41.4128262240	-41.4128262246	-41.4128261895	-41.4128262063
**41.4700000**	-41.4128261408	-41.4128262210	-41.4128262217	-41.4128261875	-41.4128262038
**41.6257143**	-41.4128261408	-41.4128262180	-41.4128262189	-41.4128261856	-41.4128262013
**41.7814286**	-41.4128261408	-41.4128262151	-41.4128262162	-41.4128261837	-41.4128261990
**41.9371429**	-41.4128261407	-41.4128262123	-41.4128262136	-41.4128261820	-41.4128261968
**42.0928571**	-41.4128261407	-41.4128262096	-41.4128262110	-41.4128261803	-41.4128261946
**42.2485714**	-41.4128261407	-41.4128262070	-41.4128262086	-41.4128261786	-41.4128261925
**42.4042857**	-41.4128261406	-41.4128262044	-41.4128262062	-41.4128261770	-41.4128261905
**42.5600000**	-41.4128261406	-41.4128262020	-41.4128262039	-41.4128261755	-41.4128261886
**42.7157143**	-41.4128261406	-41.4128261996	-41.4128262017	-41.4128261740	-41.4128261867
**42.8714286**	-41.4128261406	-41.4128261973	-41.4128261996	-41.4128261726	-41.4128261849
**43.0271429**	-41.4128261405	-41.4128261951	-41.4128261975	-41.4128261712	-41.4128261832
**43.1828571**	-41.4128261405	-41.4128261929	-41.4128261955	-41.4128261699	-41.4128261815
**43.3385714**	-41.4128261405	-41.4128261908	-41.4128261936	-41.4128261687	-41.4128261799
**43.4942857**	-41.4128261405	-41.4128261888	-41.4128261917	-41.4128261675	-41.4128261783
**43.6500000**	-41.4128261404	-41.4128261868	-41.4128261899	-41.4128261663	-41.4128261768
**43.8057143**	-41.4128261404	-41.4128261849	-41.4128261881	-41.4128261652	-41.4128261754
**43.9614286**	-41.4128261404	-41.4128261831	-41.4128261865	-41.4128261641	-41.4128261740
**44.1171429**	-41.4128261404	-41.4128261813	-41.4128261848	-41.4128261630	-41.4128261727
**44.2728571**	-41.4128261403	-41.4128261796	-41.4128261832	-41.4128261620	-41.4128261714
**44.4285714**	-41.4128261403	-41.4128261779	-41.4128261817	-41.4128261611	-41.4128261701
**44.5842857**	-41.4128261403	-41.4128261763	-41.4128261803	-41.4128261602	-41.4128261689
**44.7400000**	-41.4128261403	-41.4128261747	-41.4128261788	-41.4128261593	-41.4128261678
**44.8957143**	-41.4128261403	-41.4128261732	-41.4128261775	-41.4128261584	-41.4128261667
**45.0514286**	-41.4128261403	-41.4128261718	-41.4128261761	-41.4128261576	-41.4128261656
**45.2071429**	-41.4128261402	-41.4128261703	-41.4128261748	-41.4128261568	-41.4128261645
**45.3628571**	-41.4128261402	-41.4128261690	-41.4128261736	-41.4128261560	-41.4128261636
**45.5185714**	-41.4128261402	-41.4128261676	-41.4128261724	-41.4128261553	-41.4128261626
**45.6742857**	-41.4128261402	-41.4128261664	-41.4128261713	-41.4128261546	-41.4128261617

R (Bohr)/θ(º)	0.00	10.00	20.00	30.00	40.00
**45.8300000**	-41.4128261402	-41.4128261651	-41.4128261701	-41.4128261539	-41.4128261608
**45.9857143**	-41.4128261402	-41.4128261639	-41.4128261691	-41.4128261533	-41.4128261599
**46.1414286**	-41.4128261401	-41.4128261628	-41.4128261680	-41.4128261526	-41.4128261591
**46.2971429**	-41.4128261401	-41.4128261616	-41.4128261670	-41.4128261520	-41.4128261583
**46.4528571**	-41.4128261401	-41.4128261605	-41.4128261660	-41.4128261515	-41.4128261575
**46.6085714**	-41.4128261401	-41.4128261595	-41.4128261651	-41.4128261509	-41.4128261568
**46.7642857**	-41.4128261401	-41.4128261585	-41.4128261642	-41.4128261504	-41.4128261561
**46.9200000**	-41.4128261401	-41.4128261575	-41.4128261633	-41.4128261499	-41.4128261554
**47.0757143**	-41.4128261401	-41.4128261565	-41.4128261625	-41.4128261494	-41.4128261547
**47.2314286**	-41.4128261400	-41.4128261556	-41.4128261616	-41.4128261489	-41.4128261541
**47.3871429**	-41.4128261400	-41.4128261547	-41.4128261609	-41.4128261484	-41.4128261535
**47.5428571**	-41.4128261400	-41.4128261539	-41.4128261601	-41.4128261480	-41.4128261529
**47.6985714**	-41.4128261400	-41.4128261530	-41.4128261594	-41.4128261476	-41.4128261523
**47.8542857**	-41.4128261400	-41.4128261522	-41.4128261586	-41.4128261472	-41.4128261518
**48.0100000**	-41.4128261400	-41.4128261514	-41.4128261580	-41.4128261468	-41.4128261512
**48.1657143**	-41.4128261400	-41.4128261507	-41.4128261573	-41.4128261464	-41.4128261507
**48.3214286**	-41.4128261400	-41.4128261500	-41.4128261567	-41.4128261460	-41.4128261502
**48.4771429**	-41.4128261400	-41.4128261492	-41.4128261560	-41.4128261457	-41.4128261498
**48.6328571**	-41.4128261400	-41.4128261486	-41.4128261554	-41.4128261453	-41.4128261493
**48.7885714**	-41.4128261399	-41.4128261479	-41.4128261549	-41.4128261450	-41.4128261489
**48.9442857**	-41.4128261399	-41.4128261473	-41.4128261543	-41.4128261447	-41.4128261485
**49.1000000**	-41.4128261399	-41.4128261466	-41.4128261538	-41.4128261444	-41.4128261481
**49.2557143**	-41.4128261399	-41.4128261461	-41.4128261532	-41.4128261441	-41.4128261477
**49.4114286**	-41.4128261399	-41.4128261455	-41.4128261527	-41.4128261439	-41.4128261473
**49.5671429**	-41.4128261399	-41.4128261449	-41.4128261523	-41.4128261436	-41.4128261469
**49.7228571**	-41.4128261399	-41.4128261444	-41.4128261518	-41.4128261433	-41.4128261466
**49.8785714**	-41.4128261399	-41.4128261439	-41.4128261513	-41.4128261431	-41.4128261462
**50.0342857**	-41.4128261399	-41.4128261433	-41.4128261509	-41.4128261429	-41.4128261459
**50.1900000**	-41.4128261399	-41.4128261429	-41.4128261505	-41.4128261426	-41.4128261456
**50.3457143**	-41.4128261399	-41.4128261424	-41.4128261501	-41.4128261424	-41.4128261453
**50.5014286**	-41.4128261398	-41.4128261419	-41.4128261497	-41.4128261422	-41.4128261450
**50.6571429**	-41.4128261398	-41.4128261415	-41.4128261493	-41.4128261420	-41.4128261447
**50.8128571**	-41.4128261398	-41.4128261411	-41.4128261490	-41.4128261418	-41.4128261445
**50.9685714**	-41.4128261398	-41.4128261407	-41.4128261486	-41.4128261416	-41.4128261442
**51.1242857**	-41.4128261398	-41.4128261403	-41.4128261483	-41.4128261414	-41.4128261440
**51.2800000**	-41.4128261398	-41.4128261399	-41.4128261479	-41.4128261413	-41.4128261437
**51.4357143**	-41.4128261398	-41.4128261395	-41.4128261476	-41.4128261411	-41.4128261435
**51.5914286**	-41.4128261398	-41.4128261391	-41.4128261473	-41.4128261409	-41.4128261433
**51.7471429**	-41.4128261398	-41.4128261388	-41.4128261470	-41.4128261408	-41.4128261430
**51.9028571**	-41.4128261398	-41.4128261385	-41.4128261467	-41.4128261406	-41.4128261428
**52.0585714**	-41.4128261398	-41.4128261381	-41.4128261465	-41.4128261405	-41.4128261426
**52.2142857**	-41.4128261398	-41.4128261378	-41.4128261462	-41.4128261404	-41.4128261425
**52.3700000**	-41.4128261398	-41.4128261375	-41.4128261459	-41.4128261402	-41.4128261423
**52.5257143**	-41.4128261398	-41.4128261372	-41.4128261457	-41.4128261401	-41.4128261421
**52.6814286**	-41.4128261398	-41.4128261369	-41.4128261454	-41.4128261400	-41.4128261419
**52.8371429**	-41.4128261398	-41.4128261367	-41.4128261452	-41.4128261399	-41.4128261418
**52.9928571**	-41.4128261397	-41.4128261364	-41.4128261450	-41.4128261398	-41.4128261416
**53.1485714**	-41.4128261397	-41.4128261361	-41.4128261448	-41.4128261397	-41.4128261414
**53.3042857**	-41.4128261397	-41.4128261359	-41.4128261446	-41.4128261395	-41.4128261413
**53.4600000**	-41.4128261397	-41.4128261356	-41.4128261444	-41.4128261394	-41.4128261412
**53.6157143**	-41.4128261397	-41.4128261354	-41.4128261442	-41.4128261394	-41.4128261410
**53.7714286**	-41.4128261397	-41.4128261352	-41.4128261440	-41.4128261393	-41.4128261409
**53.9271429**	-41.4128261397	-41.4128261350	-41.4128261438	-41.4128261392	-41.4128261408
**54.0828571**	-41.4128261397	-41.4128261348	-41.4128261436	-41.4128261391	-41.4128261406
**54.2385714**	-41.4128261397	-41.4128261346	-41.4128261435	-41.4128261390	-41.4128261405
**54.3942857**	-41.4128261397	-41.4128261344	-41.4128261433	-41.4128261389	-41.4128261404
**54.5500000**	-41.4128261397	-41.4128261342	-41.4128261432	-41.4128261389	-41.4128261403
**54.7057143**	-41.4128261397	-41.4128261340	-41.4128261430	-41.4128261388	-41.4128261402
**54.8614286**	-41.4128261397	-41.4128261338	-41.4128261429	-41.4128261387	-41.4128261401
**55.0171429**	-41.4128261397	-41.4128261336	-41.4128261427	-41.4128261386	-41.4128261400
**55.1728571**	-41.4128261397	-41.4128261335	-41.4128261426	-41.4128261386	-41.4128261399
**55.3285714**	-41.4128261397	-41.4128261333	-41.4128261424	-41.4128261385	-41.4128261398
**55.4842857**	-41.4128261397	-41.4128261332	-41.4128261423	-41.4128261385	-41.4128261397
**55.6400000**	-41.4128261397	-41.4128261330	-41.4128261422	-41.4128261384	-41.4128261397
**55.7957143**	-41.4128261397	-41.4128261329	-41.4128261421	-41.4128261383	-41.4128261396

R (Bohr)/θ(º)	0.00	10.00	20.00	30.00	40.00
**55.9514286**	-41.4128261397	-41.4128261327	-41.4128261420	-41.4128261383	-41.4128261395
**56.1071429**	-41.4128261397	-41.4128261326	-41.4128261419	-41.4128261382	-41.4128261394
**56.2628571**	-41.4128261397	-41.4128261325	-41.4128261418	-41.4128261382	-41.4128261394
**56.4185714**	-41.4128261397	-41.4128261323	-41.4128261417	-41.4128261381	-41.4128261393
**56.5742857**	-41.4128261397	-41.4128261322	-41.4128261416	-41.4128261381	-41.4128261392
**56.7300000**	-41.4128261396	-41.4128261321	-41.4128261415	-41.4128261381	-41.4128261392
**56.8857143**	-41.4128261396	-41.4128261320	-41.4128261414	-41.4128261380	-41.4128261391
**57.0414286**	-41.4128261396	-41.4128261319	-41.4128261413	-41.4128261380	-41.4128261390
**57.1971429**	-41.4128261396	-41.4128261318	-41.4128261412	-41.4128261379	-41.4128261390
**57.3528571**	-41.4128261396	-41.4128261317	-41.4128261411	-41.4128261379	-41.4128261389
**57.5085714**	-41.4128261396	-41.4128261316	-41.4128261410	-41.4128261379	-41.4128261389
**57.6642857**	-41.4128261396	-41.4128261315	-41.4128261410	-41.4128261378	-41.4128261388
**57.8200000**	-41.4128261396	-41.4128261314	-41.4128261409	-41.4128261378	-41.4128261388
**57.9757143**	-41.4128261396	-41.4128261313	-41.4128261408	-41.4128261378	-41.4128261387
**58.1314286**	-41.4128261396	-41.4128261312	-41.4128261407	-41.4128261377	-41.4128261387
**58.2871429**	-41.4128261396	-41.4128261311	-41.4128261407	-41.4128261377	-41.4128261386
**58.4428571**	-41.4128261396	-41.4128261311	-41.4128261406	-41.4128261377	-41.4128261386
**58.5985714**	-41.4128261396	-41.4128261310	-41.4128261405	-41.4128261376	-41.4128261385
**58.7542857**	-41.4128261396	-41.4128261309	-41.4128261405	-41.4128261376	-41.4128261385
**58.9100000**	-41.4128261396	-41.4128261308	-41.4128261404	-41.4128261376	-41.4128261385
**59.0657143**	-41.4128261396	-41.4128261308	-41.4128261404	-41.4128261376	-41.4128261384
**59.2214286**	-41.4128261396	-41.4128261307	-41.4128261403	-41.4128261375	-41.4128261384
**59.3771429**	-41.4128261396	-41.4128261306	-41.4128261403	-41.4128261375	-41.4128261384
**59.5328571**	-41.4128261396	-41.4128261306	-41.4128261402	-41.4128261375	-41.4128261383
**59.6885714**	-41.4128261396	-41.4128261305	-41.4128261402	-41.4128261375	-41.4128261383
**59.8442857**	-41.4128261396	-41.4128261304	-41.4128261401	-41.4128261375	-41.4128261383
**60.0000000**	-41.4128261396	-41.4128261304	-41.4128261401	-41.4128261374	-41.4128261382

PES CBS – CH^- ($X\ ^3\Sigma^-$) + He					
**R (Bohr)/θ(º)**	**50.00**	**60.00**	**70.00**	**80.00**	**90.00**
**5.5000000**	-41.4114560836	-41.4116144586	-41.4115640211	-41.4113923919	-41.4111958753
**5.6557143**	-41.4117941866	-41.4118962073	-41.4118420378	-41.4116961139	-41.4115371150
**5.8114286**	-41.4120560282	-41.4121173829	-41.4120628388	-41.4119394192	-41.4118108624
**5.9671429**	-41.4122592628	-41.4122912268	-41.4122379390	-41.4121350144	-41.4120312178
**6.1228571**	-41.4124172735	-41.4124280324	-41.4123768643	-41.4122922378	-41.4122088719
**6.2785714**	-41.4125403153	-41.4125358083	-41.4124873020	-41.4124185970	-41.4123521925
**6.4342857**	-41.4126361828	-41.4126207881	-41.4125753280	-41.4125201960	-41.4124678469
**6.5900000**	-41.4127107593	-41.4126878240	-41.4126456730	-41.4126019372	-41.4125611843
**6.7457143**	-41.4127685083	-41.4127406929	-41.4127019899	-41.4126677070	-41.4126364963
**6.9014286**	-41.4128128792	-41.4127823359	-41.4127470962	-41.4127205686	-41.4126972168
**7.0571429**	-41.4128466055	-41.4128150460	-41.4127831774	-41.4127629419	-41.4127460862
**7.2128571**	-41.4128718988	-41.4128406152	-41.4128119471	-41.4127967566	-41.4127852898
**7.3685714**	-41.4128905644	-41.4128604496	-41.4128347665	-41.4128235705	-41.4128165747
**7.5242857**	-41.4129040675	-41.4128756591	-41.4128527307	-41.4128446558	-41.4128413452
**7.6800000**	-41.4129135769	-41.4128871258	-41.4128667285	-41.4128610598	-41.4128607397
**7.8357143**	-41.4129200032	-41.4128955570	-41.4128774860	-41.4128736470	-41.4128756895
**7.9914286**	-41.4129240391	-41.4129015252	-41.4128855966	-41.4128831302	-41.4128869649
**8.1471429**	-41.4129262016	-41.4129054976	-41.4128915455	-41.4128900942	-41.4128952079
**8.3028571**	-41.4129268750	-41.4129078589	-41.4128957285	-41.4128950162	-41.4129009587
**8.4585714**	-41.4129263496	-41.4129089279	-41.4128984689	-41.4128982843	-41.4129046736
**8.6142857**	-41.4129248536	-41.4129089698	-41.4129000314	-41.4129002141	-41.4129067395
**8.7700000**	-41.4129225772	-41.4129082056	-41.4129006341	-41.4129010629	-41.4129074854
**8.9257143**	-41.4129196874	-41.4129068195	-41.4129004590	-41.4129010429	-41.4129071902
**9.0814286**	-41.4129163365	-41.4129049646	-41.4128996594	-41.4129003309	-41.4129060904
**9.2371429**	-41.4129126648	-41.4129027671	-41.4128983666	-41.4128990764	-41.4129043853
**9.3928571**	-41.4129088003	-41.4129003308	-41.4128966933	-41.4128974073	-41.4129022417
**9.5485714**	-41.4129048565	-41.4128977398	-41.4128947372	-41.4128954332	-41.4128997980
**9.7042857**	-41.4129009298	-41.4128950618	-41.4128925824	-41.4128932481	-41.4128971675
**9.8600000**	-41.4128970979	-41.4128923501	-41.4128903006	-41.4128909319	-41.4128944413
**10.0157143**	-41.4128934193	-41.4128896464	-41.4128879514	-41.4128885504	-41.4128916907
**10.1714286**	-41.4128899337	-41.4128869823	-41.4128855835	-41.4128861572	-41.4128889698
**10.3271429**	-41.4128866638	-41.4128843811	-41.4128832351	-41.4128837931	-41.4128863181
**10.4828571**	-41.4128836174	-41.4128818596	-41.4128809343	-41.4128814882	-41.4128837619
**10.6385714**	-41.4128807908	-41.4128794287	-41.4128787008	-41.4128792621	-41.4128813171
**10.7942857**	-41.4128781717	-41.4128770953	-41.4128765465	-41.4128771259	-41.4128789908
**10.9500000**	-41.4128757420	-41.4128748624	-41.4128744771	-41.4128750835	-41.4128767836
**11.1057143**	-41.4128734804	-41.4128727306	-41.4128724936	-41.4128731332	-41.4128746907
**11.2614286**	-41.4128713648	-41.4128706985	-41.4128705930	-41.4128712696	-41.4128727041
**11.4171429**	-41.4128693738	-41.4128687629	-41.4128687704	-41.4128694847	-41.4128708137
**11.5728571**	-41.4128674880	-41.4128669197	-41.4128670194	-41.4128677695	-41.4128690086
**11.7285714**	-41.4128656906	-41.4128651642	-41.4128653333	-41.4128661147	-41.4128672779
**11.8842857**	-41.4128639679	-41.4128634911	-41.4128637057	-41.4128645120	-41.4128656115
**12.0400000**	-41.4128623093	-41.4128618952	-41.4128621310	-41.4128629545	-41.4128640007
**12.1957143**	-41.4128607071	-41.4128603712	-41.4128606049	-41.4128614367	-41.4128624383
**12.3514286**	-41.4128591562	-41.4128589139	-41.4128591242	-41.4128599550	-41.4128609191
**12.5071429**	-41.4128576536	-41.4128575187	-41.4128576869	-41.4128585075	-41.4128594393
**12.6628571**	-41.4128561980	-41.4128561812	-41.4128562922	-41.4128570940	-41.4128579970
**12.8185714**	-41.4128547895	-41.4128548973	-41.4128549403	-41.4128557153	-41.4128565916
**12.9742857**	-41.4128534288	-41.4128536636	-41.4128536321	-41.4128543733	-41.4128552237
**13.1300000**	-41.4128521173	-41.4128524772	-41.4128523688	-41.4128530705	-41.4128538948
**13.2857143**	-41.4128508563	-41.4128513355	-41.4128511520	-41.4128518099	-41.4128526068
**13.4414286**	-41.4128496472	-41.4128502363	-41.4128499832	-41.4128505942	-41.4128513621
**13.5971429**	-41.4128484913	-41.4128491780	-41.4128488639	-41.4128494262	-41.4128501631
**13.7528571**	-41.4128473892	-41.4128481592	-41.4128477951	-41.4128483080	-41.4128490120
**13.9085714**	-41.4128463413	-41.4128471787	-41.4128467775	-41.4128472414	-41.4128479108
**14.0642857**	-41.4128453474	-41.4128462359	-41.4128458112	-41.4128462275	-41.4128468608
**14.2200000**	-41.4128444070	-41.4128453299	-41.4128448959	-41.4128452667	-41.4128458631
**14.3757143**	-41.4128435190	-41.4128444604	-41.4128440308	-41.4128443587	-41.4128449179
**14.5314286**	-41.4128426821	-41.4128436268	-41.4128432146	-41.4128435027	-41.4128440250
**14.6871429**	-41.4128418946	-41.4128428288	-41.4128424455	-41.4128426973	-41.4128431837
**14.8428571**	-41.4128411544	-41.4128420660	-41.4128417216	-41.4128419405	-41.4128423925
**14.9985714**	-41.4128404593	-41.4128413379	-41.4128410405	-41.4128412302	-41.4128416499
**15.1542857**	-41.4128398070	-41.4128406440	-41.4128403996	-41.4128405638	-41.4128409536
**15.3100000**	-41.4128391951	-41.4128399838	-41.4128397964	-41.4128399385	-41.4128403012

R (Bohr)/θ(º)	50.00	60.00	70.00	80.00	90.00
**15.4657143**	-41.4128386211	-41.4128393566	-41.4128392282	-41.4128393514	-41.4128396900
**15.6214286**	-41.4128380825	-41.4128387616	-41.4128386922	-41.4128387997	-41.4128391173
**15.7771429**	-41.4128375769	-41.4128381980	-41.4128381860	-41.4128382805	-41.4128385802
**15.9328571**	-41.4128371020	-41.4128376647	-41.4128377070	-41.4128377911	-41.4128380758
**16.0885714**	-41.4128366555	-41.4128371608	-41.4128372528	-41.4128373289	-41.4128376015
**16.2442857**	-41.4128362352	-41.4128366849	-41.4128368214	-41.4128368913	-41.4128371544
**16.4000000**	-41.4128358392	-41.4128362361	-41.4128364108	-41.4128364763	-41.4128367320
**16.5557143**	-41.4128354654	-41.4128358128	-41.4128360192	-41.4128360818	-41.4128363321
**16.7114286**	-41.4128351123	-41.4128354139	-41.4128356451	-41.4128357060	-41.4128359525
**16.8671429**	-41.4128347780	-41.4128350380	-41.4128352870	-41.4128353473	-41.4128355911
**17.0228571**	-41.4128344613	-41.4128346838	-41.4128349439	-41.4128350043	-41.4128352463
**17.1785714**	-41.4128341605	-41.4128343498	-41.4128346147	-41.4128346759	-41.4128349165
**17.3342857**	-41.4128338747	-41.4128340347	-41.4128342985	-41.4128343610	-41.4128346004
**17.4900000**	-41.4128336025	-41.4128337373	-41.4128339946	-41.4128340588	-41.4128342968
**17.6457143**	-41.4128333430	-41.4128334563	-41.4128337025	-41.4128337686	-41.4128340049
**17.8014286**	-41.4128330952	-41.4128331905	-41.4128334216	-41.4128334898	-41.4128337238
**17.9571429**	-41.4128328584	-41.4128329386	-41.4128331516	-41.4128332219	-41.4128334529
**18.1128571**	-41.4128326318	-41.4128326998	-41.4128328920	-41.4128329645	-41.4128331915
**18.2685714**	-41.4128324147	-41.4128324728	-41.4128326427	-41.4128327172	-41.4128329394
**18.4242857**	-41.4128322065	-41.4128322569	-41.4128324033	-41.4128324798	-41.4128326960
**18.5800000**	-41.4128320066	-41.4128320510	-41.4128321736	-41.4128322519	-41.4128324613
**18.7357143**	-41.4128318146	-41.4128318545	-41.4128319534	-41.4128320334	-41.4128322349
**18.8914286**	-41.4128316300	-41.4128316666	-41.4128317425	-41.4128318239	-41.4128320167
**19.0471429**	-41.4128314525	-41.4128314865	-41.4128315406	-41.4128316233	-41.4128318066
**19.2028571**	-41.4128312816	-41.4128313138	-41.4128313477	-41.4128314314	-41.4128316044
**19.3585714**	-41.4128311171	-41.4128311479	-41.4128311634	-41.4128312479	-41.4128314100
**19.5142857**	-41.4128309586	-41.4128309884	-41.4128309876	-41.4128310726	-41.4128312233
**19.6700000**	-41.4128308058	-41.4128308347	-41.4128308199	-41.4128309053	-41.4128310441
**19.8257143**	-41.4128306586	-41.4128306867	-41.4128306601	-41.4128307456	-41.4128308724
**19.9814286**	-41.4128305166	-41.4128305438	-41.4128305080	-41.4128305934	-41.4128307081
**20.1371429**	-41.4128303797	-41.4128304059	-41.4128303632	-41.4128304483	-41.4128305508
**20.2928571**	-41.4128302476	-41.4128302728	-41.4128302256	-41.4128303101	-41.4128304006
**20.4485714**	-41.4128301202	-41.4128301441	-41.4128300947	-41.4128301784	-41.4128302573
**20.6042857**	-41.4128299972	-41.4128300198	-41.4128299702	-41.4128300531	-41.4128301205
**20.7600000**	-41.4128298786	-41.4128298996	-41.4128298519	-41.4128299337	-41.4128299902
**20.9157143**	-41.4128297641	-41.4128297835	-41.4128297395	-41.4128298199	-41.4128298660
**21.0714286**	-41.4128296536	-41.4128296712	-41.4128296325	-41.4128297116	-41.4128297478
**21.2271429**	-41.4128295470	-41.4128295628	-41.4128295308	-41.4128296082	-41.4128296353
**21.3828571**	-41.4128294441	-41.4128294580	-41.4128294339	-41.4128295097	-41.4128295283
**21.5385714**	-41.4128293447	-41.4128293568	-41.4128293417	-41.4128294156	-41.4128294266
**21.6942857**	-41.4128292488	-41.4128292591	-41.4128292537	-41.4128293258	-41.4128293297
**21.8500000**	-41.4128291563	-41.4128291648	-41.4128291697	-41.4128292398	-41.4128292376
**22.0057143**	-41.4128290669	-41.4128290738	-41.4128290895	-41.4128291575	-41.4128291499
**22.1614286**	-41.4128289806	-41.4128289861	-41.4128290128	-41.4128290786	-41.4128290663
**22.3171429**	-41.4128288973	-41.4128289016	-41.4128289392	-41.4128290028	-41.4128289867
**22.4728571**	-41.4128288169	-41.4128288202	-41.4128288687	-41.4128289300	-41.4128289108
**22.6285714**	-41.4128287393	-41.4128287417	-41.4128288009	-41.4128288599	-41.4128288383
**22.7842857**	-41.4128286643	-41.4128286662	-41.4128287356	-41.4128287923	-41.4128287690
**22.9400000**	-41.4128285919	-41.4128285936	-41.4128286727	-41.4128287270	-41.4128287027
**23.0957143**	-41.4128285220	-41.4128285237	-41.4128286119	-41.4128286639	-41.4128286392
**23.2514286**	-41.4128284545	-41.4128284564	-41.4128285532	-41.4128286028	-41.4128285782
**23.4071429**	-41.4128283893	-41.4128283918	-41.4128284962	-41.4128285435	-41.4128285196
**23.5628571**	-41.4128283263	-41.4128283296	-41.4128284410	-41.4128284859	-41.4128284632
**23.7185714**	-41.4128282655	-41.4128282699	-41.4128283873	-41.4128284299	-41.4128284089
**23.8742857**	-41.4128282067	-41.4128282124	-41.4128283350	-41.4128283754	-41.4128283564
**24.0300000**	-41.4128281498	-41.4128281572	-41.4128282841	-41.4128283222	-41.4128283056
**24.1857143**	-41.4128280949	-41.4128281041	-41.4128282344	-41.4128282703	-41.4128282564
**24.3414286**	-41.4128280419	-41.4128280530	-41.4128281858	-41.4128282196	-41.4128282086
**24.4971429**	-41.4128279906	-41.4128280039	-41.4128281383	-41.4128281700	-41.4128281622
**24.6528571**	-41.4128279410	-41.4128279567	-41.4128280918	-41.4128281215	-41.4128281170
**24.8085714**	-41.4128278930	-41.4128279112	-41.4128280462	-41.4128280739	-41.4128280730
**24.9642857**	-41.4128278467	-41.4128278674	-41.4128280015	-41.4128280273	-41.4128280300
**25.1200000**	-41.4128278018	-41.4128278253	-41.4128279576	-41.4128279816	-41.4128279879
**25.2757143**	-41.4128277584	-41.4128277846	-41.4128279145	-41.4128279367	-41.4128279468
**25.4314286**	-41.4128277164	-41.4128277454	-41.4128278722	-41.4128278927	-41.4128279065

R (Bohr)/θ(º)	50.00	60.00	70.00	80.00	90.00
**25.5871429**	-41.4128276758	-41.4128277076	-41.4128278306	-41.4128278495	-41.4128278670
**25.7428571**	-41.4128276364	-41.4128276710	-41.4128277898	-41.4128278070	-41.4128278283
**25.8985714**	-41.4128275984	-41.4128276357	-41.4128277496	-41.4128277654	-41.4128277902
**26.0542857**	-41.4128275615	-41.4128276016	-41.4128277100	-41.4128277244	-41.4128277528
**26.2100000**	-41.4128275258	-41.4128275685	-41.4128276711	-41.4128276842	-41.4128277160
**26.3657143**	-41.4128274912	-41.4128275365	-41.4128276329	-41.4128276447	-41.4128276798
**26.5214286**	-41.4128274577	-41.4128275054	-41.4128275953	-41.4128276059	-41.4128276441
**26.6771429**	-41.4128274252	-41.4128274753	-41.4128275583	-41.4128275677	-41.4128276090
**26.8328571**	-41.4128273937	-41.4128274460	-41.4128275220	-41.4128275303	-41.4128275745
**26.9885714**	-41.4128273632	-41.4128274175	-41.4128274862	-41.4128274936	-41.4128275405
**27.1442857**	-41.4128273336	-41.4128273898	-41.4128274511	-41.4128274575	-41.4128275070
**27.3000000**	-41.4128273049	-41.4128273629	-41.4128274166	-41.4128274221	-41.4128274740
**27.4557143**	-41.4128272771	-41.4128273366	-41.4128273827	-41.4128273874	-41.4128274415
**27.6114286**	-41.4128272501	-41.4128273110	-41.4128273494	-41.4128273534	-41.4128274095
**27.7671429**	-41.4128272238	-41.4128272860	-41.4128273167	-41.4128273200	-41.4128273780
**27.9228571**	-41.4128271984	-41.4128272615	-41.4128272846	-41.4128272873	-41.4128273469
**28.0785714**	-41.4128271737	-41.4128272377	-41.4128272531	-41.4128272553	-41.4128273164
**28.2342857**	-41.4128271497	-41.4128272143	-41.4128272223	-41.4128272239	-41.4128272863
**28.3900000**	-41.4128271264	-41.4128271915	-41.4128271920	-41.4128271932	-41.4128272567
**28.5457143**	-41.4128271037	-41.4128271691	-41.4128271624	-41.4128271631	-41.4128272277
**28.7014286**	-41.4128270817	-41.4128271472	-41.4128271334	-41.4128271337	-41.4128271991
**28.8571429**	-41.4128270604	-41.4128271257	-41.4128271049	-41.4128271050	-41.4128271709
**29.0128571**	-41.4128270396	-41.4128271046	-41.4128270771	-41.4128270769	-41.4128271433
**29.1685714**	-41.4128270194	-41.4128270839	-41.4128270499	-41.4128270494	-41.4128271162
**29.3242857**	-41.4128269997	-41.4128270636	-41.4128270233	-41.4128270226	-41.4128270895
**29.4800000**	-41.4128269806	-41.4128270437	-41.4128269973	-41.4128269964	-41.4128270634
**29.6357143**	-41.4128269620	-41.4128270241	-41.4128269718	-41.4128269708	-41.4128270377
**29.7914286**	-41.4128269439	-41.4128270049	-41.4128269470	-41.4128269459	-41.4128270126
**29.9471429**	-41.4128269263	-41.4128269860	-41.4128269227	-41.4128269216	-41.4128269879
**30.1028571**	-41.4128269092	-41.4128269675	-41.4128268990	-41.4128268978	-41.4128269637
**30.2585714**	-41.4128268925	-41.4128269493	-41.4128268759	-41.4128268747	-41.4128269400
**30.4142857**	-41.4128268762	-41.4128269313	-41.4128268534	-41.4128268522	-41.4128269168
**30.5700000**	-41.4128268604	-41.4128269137	-41.4128268314	-41.4128268302	-41.4128268941
**30.7257143**	-41.4128268450	-41.4128268964	-41.4128268100	-41.4128268089	-41.4128268719
**30.8814286**	-41.4128268299	-41.4128268794	-41.4128267891	-41.4128267881	-41.4128268502
**31.0371429**	-41.4128268153	-41.4128268627	-41.4128267688	-41.4128267678	-41.4128268289
**31.1928571**	-41.4128268010	-41.4128268462	-41.4128267490	-41.4128267482	-41.4128268082
**31.3485714**	-41.4128267871	-41.4128268301	-41.4128267297	-41.4128267290	-41.4128267879
**31.5042857**	-41.4128267735	-41.4128268142	-41.4128267110	-41.4128267104	-41.4128267680
**31.6600000**	-41.4128267603	-41.4128267986	-41.4128266927	-41.4128266923	-41.4128267487
**31.8157143**	-41.4128267474	-41.4128267833	-41.4128266750	-41.4128266748	-41.4128267298
**31.9714286**	-41.4128267348	-41.4128267682	-41.4128266577	-41.4128266577	-41.4128267113
**32.1271429**	-41.4128267225	-41.4128267534	-41.4128266410	-41.4128266411	-41.4128266933
**32.2828571**	-41.4128267105	-41.4128267389	-41.4128266247	-41.4128266250	-41.4128266758
**32.4385714**	-41.4128266989	-41.4128267246	-41.4128266088	-41.4128266094	-41.4128266587
**32.5942857**	-41.4128266874	-41.4128267106	-41.4128265935	-41.4128265942	-41.4128266420
**32.7500000**	-41.4128266763	-41.4128266969	-41.4128265785	-41.4128265795	-41.4128266258
**32.9057143**	-41.4128266654	-41.4128266834	-41.4128265640	-41.4128265653	-41.4128266100
**33.0614286**	-41.4128266548	-41.4128266701	-41.4128265500	-41.4128265515	-41.4128265946
**33.2171429**	-41.4128266444	-41.4128266571	-41.4128265363	-41.4128265381	-41.4128265796
**33.3728571**	-41.4128266343	-41.4128266444	-41.4128265231	-41.4128265251	-41.4128265650
**33.5285714**	-41.4128266244	-41.4128266319	-41.4128265103	-41.4128265125	-41.4128265508
**33.6842857**	-41.4128266147	-41.4128266196	-41.4128264978	-41.4128265003	-41.4128265370
**33.8400000**	-41.4128266053	-41.4128266076	-41.4128264858	-41.4128264885	-41.4128265235
**33.9957143**	-41.4128265960	-41.4128265959	-41.4128264741	-41.4128264770	-41.4128265105
**34.1514286**	-41.4128265870	-41.4128265843	-41.4128264627	-41.4128264659	-41.4128264978
**34.3071429**	-41.4128265782	-41.4128265730	-41.4128264518	-41.4128264552	-41.4128264855
**34.4628571**	-41.4128265695	-41.4128265620	-41.4128264411	-41.4128264448	-41.4128264735
**34.6185714**	-41.4128265611	-41.4128265511	-41.4128264308	-41.4128264348	-41.4128264619
**34.7742857**	-41.4128265528	-41.4128265405	-41.4128264209	-41.4128264251	-41.4128264506
**34.9300000**	-41.4128265448	-41.4128265301	-41.4128264112	-41.4128264157	-41.4128264396
**35.0857143**	-41.4128265369	-41.4128265200	-41.4128264019	-41.4128264066	-41.4128264289
**35.2414286**	-41.4128265292	-41.4128265100	-41.4128263928	-41.4128263978	-41.4128264186
**35.3971429**	-41.4128265216	-41.4128265003	-41.4128263841	-41.4128263893	-41.4128264086
**35.5528571**	-41.4128265142	-41.4128264908	-41.4128263756	-41.4128263810	-41.4128263988

R (Bohr)/θ(º)	50.00	60.00	70.00	80.00	90.00
**35.7085714**	-41.4128265070	-41.4128264815	-41.4128263674	-41.4128263731	-41.4128263894
**35.8642857**	-41.4128264999	-41.4128264724	-41.4128263595	-41.4128263654	-41.4128263802
**36.0200000**	-41.4128264930	-41.4128264635	-41.4128263518	-41.4128263580	-41.4128263714
**36.1757143**	-41.4128264863	-41.4128264548	-41.4128263444	-41.4128263508	-41.4128263628
**36.3314286**	-41.4128264796	-41.4128264464	-41.4128263373	-41.4128263438	-41.4128263544
**36.4871429**	-41.4128264731	-41.4128264381	-41.4128263303	-41.4128263371	-41.4128263463
**36.6428571**	-41.4128264668	-41.4128264300	-41.4128263236	-41.4128263307	-41.4128263385
**36.7985714**	-41.4128264606	-41.4128264221	-41.4128263172	-41.4128263244	-41.4128263309
**36.9542857**	-41.4128264545	-41.4128264144	-41.4128263109	-41.4128263184	-41.4128263235
**37.1100000**	-41.4128264485	-41.4128264068	-41.4128263049	-41.4128263125	-41.4128263164
**37.2657143**	-41.4128264427	-41.4128263995	-41.4128262990	-41.4128263069	-41.4128263095
**37.4214286**	-41.4128264370	-41.4128263923	-41.4128262934	-41.4128263015	-41.4128263028
**37.5771429**	-41.4128264314	-41.4128263853	-41.4128262880	-41.4128262962	-41.4128262964
**37.7328571**	-41.4128264259	-41.4128263785	-41.4128262827	-41.4128262912	-41.4128262901
**37.8885714**	-41.4128264205	-41.4128263719	-41.4128262776	-41.4128262863	-41.4128262840
**38.0442857**	-41.4128264153	-41.4128263654	-41.4128262727	-41.4128262815	-41.4128262782
**38.2000000**	-41.4128264101	-41.4128263590	-41.4128262680	-41.4128262770	-41.4128262725
**38.3557143**	-41.4128264050	-41.4128263529	-41.4128262634	-41.4128262726	-41.4128262670
**38.5114286**	-41.4128264001	-41.4128263469	-41.4128262590	-41.4128262683	-41.4128262617
**38.6671429**	-41.4128263953	-41.4128263410	-41.4128262547	-41.4128262643	-41.4128262565
**38.8228571**	-41.4128263905	-41.4128263353	-41.4128262506	-41.4128262603	-41.4128262516
**38.9785714**	-41.4128263858	-41.4128263298	-41.4128262466	-41.4128262565	-41.4128262467
**39.1342857**	-41.4128263813	-41.4128263243	-41.4128262428	-41.4128262528	-41.4128262421
**39.2900000**	-41.4128263768	-41.4128263191	-41.4128262391	-41.4128262493	-41.4128262376
**39.4457143**	-41.4128263724	-41.4128263139	-41.4128262355	-41.4128262459	-41.4128262332
**39.6014286**	-41.4128263681	-41.4128263089	-41.4128262321	-41.4128262426	-41.4128262290
**39.7571429**	-41.4128263639	-41.4128263041	-41.4128262287	-41.4128262394	-41.4128262249
**39.9128571**	-41.4128263598	-41.4128262993	-41.4128262255	-41.4128262363	-41.4128262210
**40.0685714**	-41.4128263558	-41.4128262947	-41.4128262224	-41.4128262334	-41.4128262172
**40.2242857**	-41.4128263518	-41.4128262902	-41.4128262195	-41.4128262305	-41.4128262135
**40.3800000**	-41.4128263479	-41.4128262859	-41.4128262166	-41.4128262278	-41.4128262099
**40.5357143**	-41.4128263441	-41.4128262816	-41.4128262138	-41.4128262251	-41.4128262065
**40.6914286**	-41.4128263403	-41.4128262775	-41.4128262111	-41.4128262226	-41.4128262032
**40.8471429**	-41.4128263367	-41.4128262735	-41.4128262085	-41.4128262201	-41.4128262000
**41.0028571**	-41.4128263331	-41.4128262696	-41.4128262060	-41.4128262177	-41.4128261969
**41.1585714**	-41.4128263296	-41.4128262658	-41.4128262036	-41.4128262154	-41.4128261939
**41.3142857**	-41.4128263261	-41.4128262621	-41.4128262013	-41.4128262132	-41.4128261910
**41.4700000**	-41.4128263227	-41.4128262585	-41.4128261991	-41.4128262111	-41.4128261882
**41.6257143**	-41.4128263194	-41.4128262550	-41.4128261969	-41.4128262090	-41.4128261855
**41.7814286**	-41.4128263161	-41.4128262516	-41.4128261949	-41.4128262071	-41.4128261829
**41.9371429**	-41.4128263129	-41.4128262483	-41.4128261928	-41.4128262052	-41.4128261803
**42.0928571**	-41.4128263098	-41.4128262450	-41.4128261909	-41.4128262033	-41.4128261779
**42.2485714**	-41.4128263067	-41.4128262419	-41.4128261891	-41.4128262016	-41.4128261755
**42.4042857**	-41.4128263037	-41.4128262389	-41.4128261873	-41.4128261999	-41.4128261733
**42.5600000**	-41.4128263007	-41.4128262359	-41.4128261855	-41.4128261982	-41.4128261711
**42.7157143**	-41.4128262978	-41.4128262331	-41.4128261839	-41.4128261966	-41.4128261690
**42.8714286**	-41.4128262950	-41.4128262303	-41.4128261822	-41.4128261951	-41.4128261669
**43.0271429**	-41.4128262922	-41.4128262276	-41.4128261807	-41.4128261936	-41.4128261649
**43.1828571**	-41.4128262894	-41.4128262249	-41.4128261792	-41.4128261922	-41.4128261630
**43.3385714**	-41.4128262868	-41.4128262224	-41.4128261778	-41.4128261909	-41.4128261612
**43.4942857**	-41.4128262841	-41.4128262199	-41.4128261764	-41.4128261896	-41.4128261594
**43.6500000**	-41.4128262815	-41.4128262175	-41.4128261750	-41.4128261883	-41.4128261577
**43.8057143**	-41.4128262790	-41.4128262152	-41.4128261737	-41.4128261871	-41.4128261560
**43.9614286**	-41.4128262765	-41.4128262129	-41.4128261725	-41.4128261859	-41.4128261544
**44.1171429**	-41.4128262740	-41.4128262107	-41.4128261713	-41.4128261848	-41.4128261529
**44.2728571**	-41.4128262716	-41.4128262085	-41.4128261702	-41.4128261837	-41.4128261514
**44.4285714**	-41.4128262693	-41.4128262065	-41.4128261690	-41.4128261826	-41.4128261499
**44.5842857**	-41.4128262670	-41.4128262044	-41.4128261680	-41.4128261816	-41.4128261485
**44.7400000**	-41.4128262647	-41.4128262025	-41.4128261669	-41.4128261807	-41.4128261472
**44.8957143**	-41.4128262625	-41.4128262006	-41.4128261659	-41.4128261797	-41.4128261459
**45.0514286**	-41.4128262603	-41.4128261987	-41.4128261650	-41.4128261788	-41.4128261447
**45.2071429**	-41.4128262581	-41.4128261969	-41.4128261641	-41.4128261779	-41.4128261434
**45.3628571**	-41.4128262560	-41.4128261952	-41.4128261632	-41.4128261771	-41.4128261423
**45.5185714**	-41.4128262540	-41.4128261935	-41.4128261623	-41.4128261763	-41.4128261412
**45.6742857**	-41.4128262519	-41.4128261919	-41.4128261615	-41.4128261755	-41.4128261401

R (Bohr)/θ(º)	50.00	60.00	70.00	80.00	90.00
**45.8300000**	-41.4128262499	-41.4128261903	-41.4128261607	-41.4128261748	-41.4128261390
**45.9857143**	-41.4128262480	-41.4128261887	-41.4128261599	-41.4128261741	-41.4128261380
**46.1414286**	-41.4128262461	-41.4128261872	-41.4128261592	-41.4128261734	-41.4128261370
**46.2971429**	-41.4128262442	-41.4128261858	-41.4128261585	-41.4128261727	-41.4128261361
**46.4528571**	-41.4128262423	-41.4128261844	-41.4128261578	-41.4128261721	-41.4128261352
**46.6085714**	-41.4128262405	-41.4128261830	-41.4128261571	-41.4128261714	-41.4128261343
**46.7642857**	-41.4128262387	-41.4128261817	-41.4128261565	-41.4128261708	-41.4128261335
**46.9200000**	-41.4128262370	-41.4128261804	-41.4128261559	-41.4128261703	-41.4128261326
**47.0757143**	-41.4128262353	-41.4128261791	-41.4128261553	-41.4128261697	-41.4128261319
**47.2314286**	-41.4128262336	-41.4128261779	-41.4128261547	-41.4128261692	-41.4128261311
**47.3871429**	-41.4128262319	-41.4128261767	-41.4128261542	-41.4128261687	-41.4128261304
**47.5428571**	-41.4128262303	-41.4128261756	-41.4128261536	-41.4128261682	-41.4128261297
**47.6985714**	-41.4128262287	-41.4128261745	-41.4128261531	-41.4128261677	-41.4128261290
**47.8542857**	-41.4128262272	-41.4128261734	-41.4128261526	-41.4128261672	-41.4128261283
**48.0100000**	-41.4128262256	-41.4128261724	-41.4128261522	-41.4128261668	-41.4128261277
**48.1657143**	-41.4128262241	-41.4128261714	-41.4128261517	-41.4128261664	-41.4128261271
**48.3214286**	-41.4128262226	-41.4128261704	-41.4128261513	-41.4128261660	-41.4128261265
**48.4771429**	-41.4128262212	-41.4128261694	-41.4128261509	-41.4128261656	-41.4128261259
**48.6328571**	-41.4128262197	-41.4128261685	-41.4128261505	-41.4128261652	-41.4128261254
**48.7885714**	-41.4128262183	-41.4128261676	-41.4128261501	-41.4128261648	-41.4128261248
**48.9442857**	-41.4128262170	-41.4128261668	-41.4128261497	-41.4128261645	-41.4128261243
**49.1000000**	-41.4128262156	-41.4128261659	-41.4128261493	-41.4128261641	-41.4128261238
**49.2557143**	-41.4128262143	-41.4128261651	-41.4128261490	-41.4128261638	-41.4128261233
**49.4114286**	-41.4128262130	-41.4128261643	-41.4128261486	-41.4128261635	-41.4128261229
**49.5671429**	-41.4128262117	-41.4128261635	-41.4128261483	-41.4128261632	-41.4128261224
**49.7228571**	-41.4128262104	-41.4128261628	-41.4128261480	-41.4128261629	-41.4128261220
**49.8785714**	-41.4128262092	-41.4128261621	-41.4128261477	-41.4128261626	-41.4128261216
**50.0342857**	-41.4128262080	-41.4128261614	-41.4128261474	-41.4128261624	-41.4128261212
**50.1900000**	-41.4128262068	-41.4128261607	-41.4128261471	-41.4128261621	-41.4128261208
**50.3457143**	-41.4128262056	-41.4128261600	-41.4128261469	-41.4128261619	-41.4128261205
**50.5014286**	-41.4128262045	-41.4128261594	-41.4128261466	-41.4128261616	-41.4128261201
**50.6571429**	-41.4128262033	-41.4128261588	-41.4128261464	-41.4128261614	-41.4128261198
**50.8128571**	-41.4128262022	-41.4128261582	-41.4128261461	-41.4128261612	-41.4128261194
**50.9685714**	-41.4128262011	-41.4128261576	-41.4128261459	-41.4128261609	-41.4128261191
**51.1242857**	-41.4128262001	-41.4128261571	-41.4128261457	-41.4128261607	-41.4128261188
**51.2800000**	-41.4128261990	-41.4128261565	-41.4128261455	-41.4128261605	-41.4128261185
**51.4357143**	-41.4128261980	-41.4128261560	-41.4128261452	-41.4128261604	-41.4128261182
**51.5914286**	-41.4128261970	-41.4128261555	-41.4128261450	-41.4128261602	-41.4128261179
**51.7471429**	-41.4128261960	-41.4128261550	-41.4128261449	-41.4128261600	-41.4128261177
**51.9028571**	-41.4128261950	-41.4128261545	-41.4128261447	-41.4128261598	-41.4128261174
**52.0585714**	-41.4128261940	-41.4128261540	-41.4128261445	-41.4128261597	-41.4128261172
**52.2142857**	-41.4128261931	-41.4128261536	-41.4128261443	-41.4128261595	-41.4128261169
**52.3700000**	-41.4128261922	-41.4128261531	-41.4128261442	-41.4128261593	-41.4128261167
**52.5257143**	-41.4128261913	-41.4128261527	-41.4128261440	-41.4128261592	-41.4128261165
**52.6814286**	-41.4128261904	-41.4128261523	-41.4128261438	-41.4128261591	-41.4128261162
**52.8371429**	-41.4128261895	-41.4128261519	-41.4128261437	-41.4128261589	-41.4128261160
**52.9928571**	-41.4128261886	-41.4128261515	-41.4128261436	-41.4128261588	-41.4128261158
**53.1485714**	-41.4128261878	-41.4128261511	-41.4128261434	-41.4128261587	-41.4128261156
**53.3042857**	-41.4128261870	-41.4128261508	-41.4128261433	-41.4128261585	-41.4128261155
**53.4600000**	-41.4128261861	-41.4128261504	-41.4128261432	-41.4128261584	-41.4128261153
**53.6157143**	-41.4128261853	-41.4128261501	-41.4128261430	-41.4128261583	-41.4128261151
**53.7714286**	-41.4128261845	-41.4128261498	-41.4128261429	-41.4128261582	-41.4128261149
**53.9271429**	-41.4128261838	-41.4128261494	-41.4128261428	-41.4128261581	-41.4128261148
**54.0828571**	-41.4128261830	-41.4128261491	-41.4128261427	-41.4128261580	-41.4128261146
**54.2385714**	-41.4128261823	-41.4128261488	-41.4128261426	-41.4128261579	-41.4128261145
**54.3942857**	-41.4128261815	-41.4128261485	-41.4128261425	-41.4128261578	-41.4128261143
**54.5500000**	-41.4128261808	-41.4128261482	-41.4128261424	-41.4128261577	-41.4128261142
**54.7057143**	-41.4128261801	-41.4128261480	-41.4128261423	-41.4128261576	-41.4128261141
**54.8614286**	-41.4128261794	-41.4128261477	-41.4128261422	-41.4128261576	-41.4128261139
**55.0171429**	-41.4128261787	-41.4128261474	-41.4128261421	-41.4128261575	-41.4128261138
**55.1728571**	-41.4128261780	-41.4128261472	-41.4128261420	-41.4128261574	-41.4128261137
**55.3285714**	-41.4128261774	-41.4128261470	-41.4128261420	-41.4128261573	-41.4128261136
**55.4842857**	-41.4128261767	-41.4128261467	-41.4128261419	-41.4128261573	-41.4128261135
**55.6400000**	-41.4128261761	-41.4128261465	-41.4128261418	-41.4128261572	-41.4128261134
**55.7957143**	-41.4128261755	-41.4128261463	-41.4128261417	-41.4128261571	-41.4128261132

R (Bohr)/θ(º)	50.00	60.00	70.00	80.00	90.00
**55.9514286**	-41.4128261748	-41.4128261461	-41.4128261417	-41.4128261571	-41.4128261131
**56.1071429**	-41.4128261742	-41.4128261459	-41.4128261416	-41.4128261570	-41.4128261130
**56.2628571**	-41.4128261736	-41.4128261457	-41.4128261415	-41.4128261569	-41.4128261130
**56.4185714**	-41.4128261731	-41.4128261455	-41.4128261415	-41.4128261569	-41.4128261129
**56.5742857**	-41.4128261725	-41.4128261453	-41.4128261414	-41.4128261568	-41.4128261128
**56.7300000**	-41.4128261719	-41.4128261451	-41.4128261414	-41.4128261568	-41.4128261127
**56.8857143**	-41.4128261714	-41.4128261449	-41.4128261413	-41.4128261567	-41.4128261126
**57.0414286**	-41.4128261708	-41.4128261448	-41.4128261413	-41.4128261567	-41.4128261125
**57.1971429**	-41.4128261703	-41.4128261446	-41.4128261412	-41.4128261566	-41.4128261125
**57.3528571**	-41.4128261698	-41.4128261444	-41.4128261412	-41.4128261566	-41.4128261124
**57.5085714**	-41.4128261692	-41.4128261443	-41.4128261411	-41.4128261565	-41.4128261123
**57.6642857**	-41.4128261687	-41.4128261441	-41.4128261411	-41.4128261565	-41.4128261123
**57.8200000**	-41.4128261682	-41.4128261440	-41.4128261410	-41.4128261565	-41.4128261122
**57.9757143**	-41.4128261678	-41.4128261438	-41.4128261410	-41.4128261564	-41.4128261121
**58.1314286**	-41.4128261673	-41.4128261437	-41.4128261409	-41.4128261564	-41.4128261121
**58.2871429**	-41.4128261668	-41.4128261436	-41.4128261409	-41.4128261563	-41.4128261120
**58.4428571**	-41.4128261663	-41.4128261434	-41.4128261409	-41.4128261563	-41.4128261119
**58.5985714**	-41.4128261659	-41.4128261433	-41.4128261408	-41.4128261563	-41.4128261119
**58.7542857**	-41.4128261654	-41.4128261432	-41.4128261408	-41.4128261562	-41.4128261118
**58.9100000**	-41.4128261650	-41.4128261431	-41.4128261407	-41.4128261562	-41.4128261118
**59.0657143**	-41.4128261645	-41.4128261430	-41.4128261407	-41.4128261562	-41.4128261117
**59.2214286**	-41.4128261641	-41.4128261429	-41.4128261407	-41.4128261562	-41.4128261117
**59.3771429**	-41.4128261637	-41.4128261427	-41.4128261407	-41.4128261561	-41.4128261117
**59.5328571**	-41.4128261633	-41.4128261426	-41.4128261406	-41.4128261561	-41.4128261116
**59.6885714**	-41.4128261629	-41.4128261425	-41.4128261406	-41.4128261561	-41.4128261116
**59.8442857**	-41.4128261625	-41.4128261424	-41.4128261406	-41.4128261560	-41.4128261115
**60.0000000**	-41.4128261621	-41.4128261424	-41.4128261405	-41.4128261560	-41.4128261115

PES CBS – CH^- ($X\ ^3\Sigma^-$) + He					
**R (Bohr)/θ(º)**	**100.00**	**110.00**	**120.00**	**130.00**	**140.00**
**5.5000000**	-41.4110309240	-41.4109054298	-41.4108755305	-41.4108759377	-41.4109011454
**5.6557143**	-41.4114105796	-41.4113314276	-41.4113126266	-41.4113292429	-41.4113649232
**5.8114286**	-41.4117146220	-41.4116646258	-41.4116605048	-41.4116890551	-41.4117318064
**5.9671429**	-41.4119584776	-41.4119279636	-41.4119365376	-41.4119729456	-41.4120187880
**6.1228571**	-41.4121543411	-41.4121375210	-41.4121552379	-41.4121961975	-41.4122447168
**6.2785714**	-41.4123118540	-41.4123049450	-41.4123284695	-41.4123715256	-41.4124207441
**6.4342857**	-41.4124386464	-41.4124389521	-41.4124657441	-41.4125091667	-41.4125569830
**6.5900000**	-41.4125407648	-41.4125462507	-41.4125745759	-41.4126171842	-41.4126627019
**6.7457143**	-41.4126230099	-41.4126321106	-41.4126608478	-41.4127018562	-41.4127450556
**6.9014286**	-41.4126891993	-41.4127007223	-41.4127291525	-41.4127680593	-41.4128090113
**7.0571429**	-41.4127423750	-41.4127554356	-41.4127830843	-41.4128195984	-41.4128581100
**7.2128571**	-41.4127849648	-41.4127989294	-41.4128254737	-41.4128594652	-41.4128952013
**7.3685714**	-41.4128189110	-41.4128333417	-41.4128585652	-41.4128900289	-41.4129227837
**7.5242857**	-41.4128457717	-41.4128603743	-41.4128841498	-41.4129131721	-41.4129430259
**7.6800000**	-41.4128668015	-41.4128813806	-41.4129036605	-41.4129303886	-41.4129576762
**7.8357143**	-41.4128830167	-41.4128974396	-41.4129182447	-41.4129428568	-41.4129680147
**7.9914286**	-41.4128952465	-41.4129094164	-41.4129288195	-41.4129514991	-41.4129748951
**8.1471429**	-41.4129041739	-41.4129180126	-41.4129361171	-41.4129570335	-41.4129788566
**8.3028571**	-41.4129103677	-41.4129238054	-41.4129407232	-41.4129600195	-41.4129802563
**8.4585714**	-41.4129143070	-41.4129272778	-41.4129431104	-41.4129608981	-41.4129793825
**8.6142857**	-41.4129164001	-41.4129288408	-41.4129436655	-41.4129600269	-41.4129765285
**8.7700000**	-41.4129169977	-41.4129288493	-41.4129427132	-41.4129577073	-41.4129720232
**8.9257143**	-41.4129164032	-41.4129276131	-41.4129405331	-41.4129542047	-41.4129662299
**9.0814286**	-41.4129148794	-41.4129254038	-41.4129373728	-41.4129497616	-41.4129595255
**9.2371429**	-41.4129126535	-41.4129224590	-41.4129334560	-41.4129446046	-41.4129522728
**9.3928571**	-41.4129099206	-41.4129189858	-41.4129289860	-41.4129389461	-41.4129447966
**9.5485714**	-41.4129068462	-41.4129151620	-41.4129241481	-41.4129329833	-41.4129373660
**9.7042857**	-41.4129035684	-41.4129111380	-41.4129191078	-41.4129268945	-41.4129301864
**9.8600000**	-41.4129002001	-41.4129070382	-41.4129140104	-41.4129208355	-41.4129233999
**10.0157143**	-41.4128968305	-41.4129029622	-41.4129089786	-41.4129149364	-41.4129170905
**10.1714286**	-41.4128935279	-41.4128989872	-41.4129041119	-41.4129092996	-41.4129112945
**10.3271429**	-41.4128903416	-41.4128951695	-41.4128994857	-41.4129039991	-41.4129060113
**10.4828571**	-41.4128873048	-41.4128915476	-41.4128951525	-41.4128990818	-41.4129012147
**10.6385714**	-41.4128844367	-41.4128881443	-41.4128911429	-41.4128945695	-41.4128968625
**10.7942857**	-41.4128817450	-41.4128849692	-41.4128874687	-41.4128904629	-41.4128929046
**10.9500000**	-41.4128792288	-41.4128820216	-41.4128841252	-41.4128867454	-41.4128892892
**11.1057143**	-41.4128768806	-41.4128792927	-41.4128810953	-41.4128833876	-41.4128859669
**11.2614286**	-41.4128746880	-41.4128767683	-41.4128783524	-41.4128803518	-41.4128828932
**11.4171429**	-41.4128726362	-41.4128744303	-41.4128758642	-41.4128775960	-41.4128800299
**11.5728571**	-41.4128707090	-41.4128722585	-41.4128735952	-41.4128750773	-41.4128773455
**11.7285714**	-41.4128688899	-41.4128702324	-41.4128715099	-41.4128727545	-41.4128748149
**11.8842857**	-41.4128671636	-41.4128683322	-41.4128695745	-41.4128705904	-41.4128724186
**12.0400000**	-41.4128655161	-41.4128665394	-41.4128677582	-41.4128685531	-41.4128701419
**12.1957143**	-41.4128639353	-41.4128648375	-41.4128660348	-41.4128666163	-41.4128679742
**12.3514286**	-41.4128624113	-41.4128632126	-41.4128643829	-41.4128647601	-41.4128659076
**12.5071429**	-41.4128609362	-41.4128616531	-41.4128627858	-41.4128629701	-41.4128639366
**12.6628571**	-41.4128595043	-41.4128601499	-41.4128612318	-41.4128612371	-41.4128620573
**12.8185714**	-41.4128581117	-41.4128586964	-41.4128597133	-41.4128595563	-41.4128602665
**12.9742857**	-41.4128567561	-41.4128572878	-41.4128582264	-41.4128579262	-41.4128585619
**13.1300000**	-41.4128554366	-41.4128559213	-41.4128567705	-41.4128563479	-41.4128569414
**13.2857143**	-41.4128541533	-41.4128545955	-41.4128553468	-41.4128548243	-41.4128554027
**13.4414286**	-41.4128529071	-41.4128533102	-41.4128539584	-41.4128533590	-41.4128539437
**13.5971429**	-41.4128516992	-41.4128520658	-41.4128526094	-41.4128519559	-41.4128525621
**13.7528571**	-41.4128505313	-41.4128508635	-41.4128513040	-41.4128506188	-41.4128512553
**13.9085714**	-41.4128494048	-41.4128497047	-41.4128500466	-41.4128493505	-41.4128500205
**14.0642857**	-41.4128483214	-41.4128485906	-41.4128488411	-41.4128481532	-41.4128488550
**14.2200000**	-41.4128472822	-41.4128475225	-41.4128476908	-41.4128470278	-41.4128477557
**14.3757143**	-41.4128462882	-41.4128465015	-41.4128465980	-41.4128459742	-41.4128467194
**14.5314286**	-41.4128453398	-41.4128455283	-41.4128455642	-41.4128449914	-41.4128457430
**14.6871429**	-41.4128444372	-41.4128446030	-41.4128445900	-41.4128440771	-41.4128448234
**14.8428571**	-41.4128435800	-41.4128437256	-41.4128436751	-41.4128432284	-41.4128439573
**14.9985714**	-41.4128427677	-41.4128428954	-41.4128428183	-41.4128424418	-41.4128431416
**15.1542857**	-41.4128419991	-41.4128421115	-41.4128420177	-41.4128417131	-41.4128423734
**15.3100000**	-41.4128412729	-41.4128413725	-41.4128412709	-41.4128410378	-41.4128416496

R (Bohr)/θ(º)	100.00	110.00	120.00	130.00	140.00
**15.4657143**	-41.4128405875	-41.4128406768	-41.4128405750	-41.4128404113	-41.4128409676
**15.6214286**	-41.4128399411	-41.4128400225	-41.4128399268	-41.4128398290	-41.4128403246
**15.7771429**	-41.4128393318	-41.4128394076	-41.4128393229	-41.4128392864	-41.4128397180
**15.9328571**	-41.4128387574	-41.4128388297	-41.4128387597	-41.4128387791	-41.4128391456
**16.0885714**	-41.4128382159	-41.4128382867	-41.4128382337	-41.4128383029	-41.4128386049
**16.2442857**	-41.4128377052	-41.4128377760	-41.4128377416	-41.4128378543	-41.4128380940
**16.4000000**	-41.4128372232	-41.4128372955	-41.4128372800	-41.4128374297	-41.4128376108
**16.5557143**	-41.4128367679	-41.4128368429	-41.4128368458	-41.4128370262	-41.4128371534
**16.7114286**	-41.4128363372	-41.4128364158	-41.4128364364	-41.4128366412	-41.4128367202
**16.8671429**	-41.4128359295	-41.4128360124	-41.4128360490	-41.4128362725	-41.4128363096
**17.0228571**	-41.4128355429	-41.4128356306	-41.4128356815	-41.4128359183	-41.4128359200
**17.1785714**	-41.4128351759	-41.4128352687	-41.4128353317	-41.4128355769	-41.4128355501
**17.3342857**	-41.4128348271	-41.4128349249	-41.4128349979	-41.4128352473	-41.4128351986
**17.4900000**	-41.4128344950	-41.4128345978	-41.4128346787	-41.4128349283	-41.4128348644
**17.6457143**	-41.4128341786	-41.4128342860	-41.4128343728	-41.4128346195	-41.4128345463
**17.8014286**	-41.4128338767	-41.4128339884	-41.4128340790	-41.4128343200	-41.4128342434
**17.9571429**	-41.4128335883	-41.4128337038	-41.4128337965	-41.4128340298	-41.4128339546
**18.1128571**	-41.4128333127	-41.4128334313	-41.4128335246	-41.4128337484	-41.4128336792
**18.2685714**	-41.4128330490	-41.4128331701	-41.4128332628	-41.4128334757	-41.4128334162
**18.4242857**	-41.4128327966	-41.4128329196	-41.4128330105	-41.4128332118	-41.4128331651
**18.5800000**	-41.4128325549	-41.4128326790	-41.4128327673	-41.4128329565	-41.4128329251
**18.7357143**	-41.4128323234	-41.4128324479	-41.4128325331	-41.4128327098	-41.4128326955
**18.8914286**	-41.4128321015	-41.4128322257	-41.4128323075	-41.4128324717	-41.4128324757
**19.0471429**	-41.4128318890	-41.4128320122	-41.4128320903	-41.4128322424	-41.4128322653
**19.2028571**	-41.4128316853	-41.4128318069	-41.4128318814	-41.4128320216	-41.4128320636
**19.3585714**	-41.4128314902	-41.4128316096	-41.4128316807	-41.4128318095	-41.4128318702
**19.5142857**	-41.4128313033	-41.4128314199	-41.4128314878	-41.4128316060	-41.4128316847
**19.6700000**	-41.4128311243	-41.4128312377	-41.4128313028	-41.4128314109	-41.4128315067
**19.8257143**	-41.4128309530	-41.4128310627	-41.4128311255	-41.4128312242	-41.4128313357
**19.9814286**	-41.4128307891	-41.4128308947	-41.4128309556	-41.4128310457	-41.4128311714
**20.1371429**	-41.4128306323	-41.4128307335	-41.4128307931	-41.4128308752	-41.4128310135
**20.2928571**	-41.4128304824	-41.4128305788	-41.4128306376	-41.4128307125	-41.4128308616
**20.4485714**	-41.4128303391	-41.4128304306	-41.4128304891	-41.4128305574	-41.4128307154
**20.6042857**	-41.4128302022	-41.4128302886	-41.4128303473	-41.4128304097	-41.4128305748
**20.7600000**	-41.4128300714	-41.4128301526	-41.4128302119	-41.4128302689	-41.4128304393
**20.9157143**	-41.4128299465	-41.4128300225	-41.4128300828	-41.4128301350	-41.4128303088
**21.0714286**	-41.4128298272	-41.4128298979	-41.4128299596	-41.4128300075	-41.4128301830
**21.2271429**	-41.4128297133	-41.4128297788	-41.4128298421	-41.4128298861	-41.4128300617
**21.3828571**	-41.4128296047	-41.4128296650	-41.4128297301	-41.4128297706	-41.4128299448
**21.5385714**	-41.4128295009	-41.4128295561	-41.4128296232	-41.4128296606	-41.4128298319
**21.6942857**	-41.4128294018	-41.4128294521	-41.4128295212	-41.4128295558	-41.4128297230
**21.8500000**	-41.4128293072	-41.4128293527	-41.4128294239	-41.4128294558	-41.4128296179
**22.0057143**	-41.4128292168	-41.4128292577	-41.4128293310	-41.4128293605	-41.4128295164
**22.1614286**	-41.4128291305	-41.4128291669	-41.4128292421	-41.4128292695	-41.4128294183
**22.3171429**	-41.4128290479	-41.4128290801	-41.4128291571	-41.4128291824	-41.4128293235
**22.4728571**	-41.4128289689	-41.4128289971	-41.4128290758	-41.4128290991	-41.4128292319
**22.6285714**	-41.4128288933	-41.4128289177	-41.4128289978	-41.4128290193	-41.4128291433
**22.7842857**	-41.4128288208	-41.4128288417	-41.4128289230	-41.4128289427	-41.4128290576
**22.9400000**	-41.4128287513	-41.4128287689	-41.4128288511	-41.4128288691	-41.4128289747
**23.0957143**	-41.4128286846	-41.4128286992	-41.4128287819	-41.4128287983	-41.4128288945
**23.2514286**	-41.4128286205	-41.4128286323	-41.4128287153	-41.4128287300	-41.4128288169
**23.4071429**	-41.4128285589	-41.4128285681	-41.4128286510	-41.4128286641	-41.4128287418
**23.5628571**	-41.4128284995	-41.4128285065	-41.4128285890	-41.4128286004	-41.4128286690
**23.7185714**	-41.4128284423	-41.4128284472	-41.4128285289	-41.4128285388	-41.4128285986
**23.8742857**	-41.4128283871	-41.4128283901	-41.4128284707	-41.4128284790	-41.4128285303
**24.0300000**	-41.4128283337	-41.4128283351	-41.4128284143	-41.4128284210	-41.4128284642
**24.1857143**	-41.4128282821	-41.4128282821	-41.4128283595	-41.4128283646	-41.4128284001
**24.3414286**	-41.4128282321	-41.4128282309	-41.4128283062	-41.4128283098	-41.4128283380
**24.4971429**	-41.4128281836	-41.4128281814	-41.4128282543	-41.4128282563	-41.4128282778
**24.6528571**	-41.4128281364	-41.4128281335	-41.4128282037	-41.4128282042	-41.4128282194
**24.8085714**	-41.4128280906	-41.4128280870	-41.4128281544	-41.4128281533	-41.4128281628
**24.9642857**	-41.4128280461	-41.4128280420	-41.4128281061	-41.4128281036	-41.4128281079
**25.1200000**	-41.4128280026	-41.4128279983	-41.4128280590	-41.4128280549	-41.4128280546
**25.2757143**	-41.4128279603	-41.4128279558	-41.4128280129	-41.4128280073	-41.4128280030
**25.4314286**	-41.4128279189	-41.4128279144	-41.4128279677	-41.4128279608	-41.4128279528

R (Bohr)/θ(º)	100.00	110.00	120.00	130.00	140.00
**25.5871429**	-41.4128278785	-41.4128278742	-41.4128279235	-41.4128279151	-41.4128279041
**25.7428571**	-41.4128278390	-41.4128278349	-41.4128278801	-41.4128278704	-41.4128278569
**25.8985714**	-41.4128278003	-41.4128277966	-41.4128278376	-41.4128278265	-41.4128278110
**26.0542857**	-41.4128277624	-41.4128277592	-41.4128277959	-41.4128277835	-41.4128277665
**26.2100000**	-41.4128277253	-41.4128277226	-41.4128277550	-41.4128277414	-41.4128277233
**26.3657143**	-41.4128276889	-41.4128276869	-41.4128277148	-41.4128277000	-41.4128276813
**26.5214286**	-41.4128276532	-41.4128276519	-41.4128276753	-41.4128276594	-41.4128276405
**26.6771429**	-41.4128276182	-41.4128276176	-41.4128276366	-41.4128276196	-41.4128276009
**26.8328571**	-41.4128275838	-41.4128275841	-41.4128275986	-41.4128275805	-41.4128275624
**26.9885714**	-41.4128275500	-41.4128275512	-41.4128275612	-41.4128275422	-41.4128275250
**27.1442857**	-41.4128275168	-41.4128275189	-41.4128275245	-41.4128275046	-41.4128274886
**27.3000000**	-41.4128274842	-41.4128274873	-41.4128274885	-41.4128274678	-41.4128274533
**27.4557143**	-41.4128274521	-41.4128274563	-41.4128274532	-41.4128274316	-41.4128274190
**27.6114286**	-41.4128274206	-41.4128274258	-41.4128274185	-41.4128273962	-41.4128273856
**27.7671429**	-41.4128273896	-41.4128273960	-41.4128273845	-41.4128273615	-41.4128273532
**27.9228571**	-41.4128273592	-41.4128273666	-41.4128273511	-41.4128273275	-41.4128273217
**28.0785714**	-41.4128273293	-41.4128273378	-41.4128273184	-41.4128272942	-41.4128272910
**28.2342857**	-41.4128272999	-41.4128273095	-41.4128272862	-41.4128272616	-41.4128272612
**28.3900000**	-41.4128272710	-41.4128272818	-41.4128272548	-41.4128272296	-41.4128272322
**28.5457143**	-41.4128272427	-41.4128272545	-41.4128272239	-41.4128271984	-41.4128272040
**28.7014286**	-41.4128272148	-41.4128272278	-41.4128271937	-41.4128271678	-41.4128271766
**28.8571429**	-41.4128271874	-41.4128272015	-41.4128271641	-41.4128271379	-41.4128271499
**29.0128571**	-41.4128271605	-41.4128271757	-41.4128271351	-41.4128271087	-41.4128271240
**29.1685714**	-41.4128271341	-41.4128271504	-41.4128271068	-41.4128270801	-41.4128270988
**29.3242857**	-41.4128271082	-41.4128271256	-41.4128270790	-41.4128270522	-41.4128270742
**29.4800000**	-41.4128270827	-41.4128271012	-41.4128270519	-41.4128270249	-41.4128270503
**29.6357143**	-41.4128270578	-41.4128270773	-41.4128270254	-41.4128269983	-41.4128270271
**29.7914286**	-41.4128270333	-41.4128270539	-41.4128269995	-41.4128269724	-41.4128270045
**29.9471429**	-41.4128270093	-41.4128270309	-41.4128269741	-41.4128269470	-41.4128269825
**30.1028571**	-41.4128269858	-41.4128270083	-41.4128269494	-41.4128269223	-41.4128269610
**30.2585714**	-41.4128269627	-41.4128269862	-41.4128269252	-41.4128268982	-41.4128269402
**30.4142857**	-41.4128269401	-41.4128269646	-41.4128269017	-41.4128268748	-41.4128269199
**30.5700000**	-41.4128269180	-41.4128269433	-41.4128268787	-41.4128268519	-41.4128269002
**30.7257143**	-41.4128268963	-41.4128269226	-41.4128268562	-41.4128268296	-41.4128268809
**30.8814286**	-41.4128268751	-41.4128269022	-41.4128268344	-41.4128268079	-41.4128268622
**31.0371429**	-41.4128268543	-41.4128268823	-41.4128268130	-41.4128267868	-41.4128268440
**31.1928571**	-41.4128268340	-41.4128268628	-41.4128267923	-41.4128267663	-41.4128268263
**31.3485714**	-41.4128268141	-41.4128268437	-41.4128267720	-41.4128267463	-41.4128268090
**31.5042857**	-41.4128267947	-41.4128268250	-41.4128267523	-41.4128267269	-41.4128267922
**31.6600000**	-41.4128267757	-41.4128268068	-41.4128267331	-41.4128267080	-41.4128267758
**31.8157143**	-41.4128267571	-41.4128267889	-41.4128267145	-41.4128266896	-41.4128267599
**31.9714286**	-41.4128267389	-41.4128267714	-41.4128266963	-41.4128266717	-41.4128267443
**32.1271429**	-41.4128267212	-41.4128267544	-41.4128266786	-41.4128266544	-41.4128267292
**32.2828571**	-41.4128267039	-41.4128267377	-41.4128266614	-41.4128266376	-41.4128267145
**32.4385714**	-41.4128266870	-41.4128267214	-41.4128266447	-41.4128266212	-41.4128267002
**32.5942857**	-41.4128266705	-41.4128267055	-41.4128266285	-41.4128266054	-41.4128266862
**32.7500000**	-41.4128266545	-41.4128266900	-41.4128266127	-41.4128265900	-41.4128266726
**32.9057143**	-41.4128266388	-41.4128266749	-41.4128265974	-41.4128265750	-41.4128266593
**33.0614286**	-41.4128266235	-41.4128266601	-41.4128265825	-41.4128265605	-41.4128266464
**33.2171429**	-41.4128266085	-41.4128266456	-41.4128265681	-41.4128265465	-41.4128266338
**33.3728571**	-41.4128265940	-41.4128266316	-41.4128265541	-41.4128265328	-41.4128266216
**33.5285714**	-41.4128265798	-41.4128266178	-41.4128265405	-41.4128265196	-41.4128266096
**33.6842857**	-41.4128265660	-41.4128266045	-41.4128265273	-41.4128265068	-41.4128265980
**33.8400000**	-41.4128265526	-41.4128265914	-41.4128265145	-41.4128264944	-41.4128265867
**33.9957143**	-41.4128265395	-41.4128265787	-41.4128265020	-41.4128264824	-41.4128265756
**34.1514286**	-41.4128265267	-41.4128265663	-41.4128264900	-41.4128264708	-41.4128265649
**34.3071429**	-41.4128265143	-41.4128265543	-41.4128264783	-41.4128264595	-41.4128265544
**34.4628571**	-41.4128265022	-41.4128265425	-41.4128264670	-41.4128264486	-41.4128265442
**34.6185714**	-41.4128264905	-41.4128265311	-41.4128264560	-41.4128264380	-41.4128265342
**34.7742857**	-41.4128264790	-41.4128265199	-41.4128264454	-41.4128264278	-41.4128265245
**34.9300000**	-41.4128264679	-41.4128265091	-41.4128264351	-41.4128264179	-41.4128265151
**35.0857143**	-41.4128264571	-41.4128264985	-41.4128264251	-41.4128264083	-41.4128265059
**35.2414286**	-41.4128264466	-41.4128264883	-41.4128264155	-41.4128263990	-41.4128264969
**35.3971429**	-41.4128264364	-41.4128264783	-41.4128264061	-41.4128263901	-41.4128264881
**35.5528571**	-41.4128264264	-41.4128264686	-41.4128263971	-41.4128263814	-41.4128264796

R (Bohr)/θ(º)	100.00	110.00	120.00	130.00	140.00
**35.7085714**	-41.4128264168	-41.4128264591	-41.4128263883	-41.4128263730	-41.4128264713
**35.8642857**	-41.4128264074	-41.4128264499	-41.4128263798	-41.4128263649	-41.4128264632
**36.0200000**	-41.4128263983	-41.4128264410	-41.4128263716	-41.4128263571	-41.4128264553
**36.1757143**	-41.4128263894	-41.4128264323	-41.4128263637	-41.4128263495	-41.4128264476
**36.3314286**	-41.4128263808	-41.4128264239	-41.4128263560	-41.4128263422	-41.4128264401
**36.4871429**	-41.4128263725	-41.4128264157	-41.4128263486	-41.4128263351	-41.4128264328
**36.6428571**	-41.4128263644	-41.4128264077	-41.4128263414	-41.4128263282	-41.4128264257
**36.7985714**	-41.4128263565	-41.4128264000	-41.4128263344	-41.4128263216	-41.4128264187
**36.9542857**	-41.4128263489	-41.4128263925	-41.4128263277	-41.4128263152	-41.4128264119
**37.1100000**	-41.4128263415	-41.4128263852	-41.4128263212	-41.4128263091	-41.4128264053
**37.2657143**	-41.4128263343	-41.4128263781	-41.4128263149	-41.4128263031	-41.4128263989
**37.4214286**	-41.4128263273	-41.4128263712	-41.4128263089	-41.4128262974	-41.4128263926
**37.5771429**	-41.4128263205	-41.4128263645	-41.4128263030	-41.4128262918	-41.4128263865
**37.7328571**	-41.4128263140	-41.4128263580	-41.4128262973	-41.4128262864	-41.4128263805
**37.8885714**	-41.4128263076	-41.4128263517	-41.4128262919	-41.4128262813	-41.4128263747
**38.0442857**	-41.4128263014	-41.4128263456	-41.4128262866	-41.4128262763	-41.4128263690
**38.2000000**	-41.4128262955	-41.4128263397	-41.4128262814	-41.4128262714	-41.4128263635
**38.3557143**	-41.4128262897	-41.4128263339	-41.4128262765	-41.4128262668	-41.4128263581
**38.5114286**	-41.4128262840	-41.4128263284	-41.4128262717	-41.4128262623	-41.4128263528
**38.6671429**	-41.4128262786	-41.4128263229	-41.4128262671	-41.4128262579	-41.4128263477
**38.8228571**	-41.4128262733	-41.4128263177	-41.4128262627	-41.4128262537	-41.4128263426
**38.9785714**	-41.4128262682	-41.4128263126	-41.4128262584	-41.4128262497	-41.4128263378
**39.1342857**	-41.4128262632	-41.4128263077	-41.4128262542	-41.4128262458	-41.4128263330
**39.2900000**	-41.4128262584	-41.4128263029	-41.4128262502	-41.4128262420	-41.4128263284
**39.4457143**	-41.4128262537	-41.4128262982	-41.4128262463	-41.4128262384	-41.4128263238
**39.6014286**	-41.4128262492	-41.4128262937	-41.4128262426	-41.4128262349	-41.4128263194
**39.7571429**	-41.4128262449	-41.4128262894	-41.4128262390	-41.4128262315	-41.4128263151
**39.9128571**	-41.4128262406	-41.4128262851	-41.4128262355	-41.4128262283	-41.4128263109
**40.0685714**	-41.4128262365	-41.4128262810	-41.4128262322	-41.4128262251	-41.4128263068
**40.2242857**	-41.4128262325	-41.4128262771	-41.4128262289	-41.4128262221	-41.4128263028
**40.3800000**	-41.4128262287	-41.4128262732	-41.4128262258	-41.4128262192	-41.4128262989
**40.5357143**	-41.4128262250	-41.4128262695	-41.4128262228	-41.4128262163	-41.4128262951
**40.6914286**	-41.4128262214	-41.4128262659	-41.4128262198	-41.4128262136	-41.4128262914
**40.8471429**	-41.4128262179	-41.4128262624	-41.4128262170	-41.4128262110	-41.4128262878
**41.0028571**	-41.4128262145	-41.4128262590	-41.4128262143	-41.4128262085	-41.4128262842
**41.1585714**	-41.4128262112	-41.4128262557	-41.4128262117	-41.4128262060	-41.4128262808
**41.3142857**	-41.4128262081	-41.4128262525	-41.4128262092	-41.4128262037	-41.4128262774
**41.4700000**	-41.4128262050	-41.4128262494	-41.4128262067	-41.4128262014	-41.4128262741
**41.6257143**	-41.4128262020	-41.4128262464	-41.4128262044	-41.4128261992	-41.4128262709
**41.7814286**	-41.4128261991	-41.4128262435	-41.4128262021	-41.4128261971	-41.4128262678
**41.9371429**	-41.4128261964	-41.4128262407	-41.4128261999	-41.4128261951	-41.4128262648
**42.0928571**	-41.4128261937	-41.4128262380	-41.4128261978	-41.4128261931	-41.4128262618
**42.2485714**	-41.4128261911	-41.4128262354	-41.4128261958	-41.4128261913	-41.4128262589
**42.4042857**	-41.4128261886	-41.4128262328	-41.4128261938	-41.4128261894	-41.4128262561
**42.5600000**	-41.4128261861	-41.4128262304	-41.4128261919	-41.4128261877	-41.4128262533
**42.7157143**	-41.4128261838	-41.4128262280	-41.4128261901	-41.4128261860	-41.4128262506
**42.8714286**	-41.4128261815	-41.4128262257	-41.4128261883	-41.4128261844	-41.4128262480
**43.0271429**	-41.4128261793	-41.4128262235	-41.4128261866	-41.4128261828	-41.4128262454
**43.1828571**	-41.4128261771	-41.4128262213	-41.4128261850	-41.4128261813	-41.4128262429
**43.3385714**	-41.4128261751	-41.4128262192	-41.4128261834	-41.4128261798	-41.4128262405
**43.4942857**	-41.4128261731	-41.4128262172	-41.4128261819	-41.4128261784	-41.4128262381
**43.6500000**	-41.4128261712	-41.4128262152	-41.4128261804	-41.4128261771	-41.4128262358
**43.8057143**	-41.4128261693	-41.4128262133	-41.4128261790	-41.4128261758	-41.4128262335
**43.9614286**	-41.4128261675	-41.4128262115	-41.4128261777	-41.4128261745	-41.4128262313
**44.1171429**	-41.4128261657	-41.4128262097	-41.4128261763	-41.4128261733	-41.4128262292
**44.2728571**	-41.4128261640	-41.4128262080	-41.4128261751	-41.4128261721	-41.4128262271
**44.4285714**	-41.4128261624	-41.4128262063	-41.4128261739	-41.4128261710	-41.4128262250
**44.5842857**	-41.4128261608	-41.4128262047	-41.4128261727	-41.4128261699	-41.4128262230
**44.7400000**	-41.4128261593	-41.4128262031	-41.4128261715	-41.4128261689	-41.4128262210
**44.8957143**	-41.4128261578	-41.4128262016	-41.4128261704	-41.4128261679	-41.4128262191
**45.0514286**	-41.4128261564	-41.4128262002	-41.4128261694	-41.4128261669	-41.4128262173
**45.2071429**	-41.4128261550	-41.4128261988	-41.4128261684	-41.4128261660	-41.4128262154
**45.3628571**	-41.4128261537	-41.4128261974	-41.4128261674	-41.4128261651	-41.4128262137
**45.5185714**	-41.4128261524	-41.4128261961	-41.4128261664	-41.4128261642	-41.4128262119
**45.6742857**	-41.4128261511	-41.4128261948	-41.4128261655	-41.4128261634	-41.4128262102

R (Bohr)/θ(º)	100.00	110.00	120.00	130.00	140.00
**45.8300000**	-41.4128261499	-41.4128261935	-41.4128261647	-41.4128261626	-41.4128262086
**45.9857143**	-41.4128261488	-41.4128261923	-41.4128261638	-41.4128261618	-41.4128262070
**46.1414286**	-41.4128261476	-41.4128261912	-41.4128261630	-41.4128261611	-41.4128262054
**46.2971429**	-41.4128261466	-41.4128261901	-41.4128261622	-41.4128261604	-41.4128262039
**46.4528571**	-41.4128261455	-41.4128261890	-41.4128261615	-41.4128261597	-41.4128262024
**46.6085714**	-41.4128261445	-41.4128261879	-41.4128261607	-41.4128261590	-41.4128262009
**46.7642857**	-41.4128261435	-41.4128261869	-41.4128261600	-41.4128261584	-41.4128261995
**46.9200000**	-41.4128261425	-41.4128261859	-41.4128261593	-41.4128261578	-41.4128261981
**47.0757143**	-41.4128261416	-41.4128261850	-41.4128261587	-41.4128261572	-41.4128261967
**47.2314286**	-41.4128261407	-41.4128261841	-41.4128261581	-41.4128261566	-41.4128261954
**47.3871429**	-41.4128261399	-41.4128261832	-41.4128261575	-41.4128261560	-41.4128261941
**47.5428571**	-41.4128261390	-41.4128261823	-41.4128261569	-41.4128261555	-41.4128261928
**47.6985714**	-41.4128261382	-41.4128261815	-41.4128261563	-41.4128261550	-41.4128261916
**47.8542857**	-41.4128261375	-41.4128261807	-41.4128261558	-41.4128261545	-41.4128261904
**48.0100000**	-41.4128261367	-41.4128261799	-41.4128261552	-41.4128261540	-41.4128261892
**48.1657143**	-41.4128261360	-41.4128261792	-41.4128261547	-41.4128261536	-41.4128261881
**48.3214286**	-41.4128261353	-41.4128261785	-41.4128261543	-41.4128261531	-41.4128261869
**48.4771429**	-41.4128261346	-41.4128261778	-41.4128261538	-41.4128261527	-41.4128261858
**48.6328571**	-41.4128261340	-41.4128261771	-41.4128261533	-41.4128261523	-41.4128261848
**48.7885714**	-41.4128261333	-41.4128261764	-41.4128261529	-41.4128261519	-41.4128261837
**48.9442857**	-41.4128261327	-41.4128261758	-41.4128261525	-41.4128261515	-41.4128261827
**49.1000000**	-41.4128261321	-41.4128261752	-41.4128261521	-41.4128261512	-41.4128261817
**49.2557143**	-41.4128261316	-41.4128261746	-41.4128261517	-41.4128261508	-41.4128261807
**49.4114286**	-41.4128261310	-41.4128261740	-41.4128261513	-41.4128261505	-41.4128261798
**49.5671429**	-41.4128261305	-41.4128261735	-41.4128261510	-41.4128261502	-41.4128261789
**49.7228571**	-41.4128261300	-41.4128261729	-41.4128261506	-41.4128261498	-41.4128261779
**49.8785714**	-41.4128261295	-41.4128261724	-41.4128261503	-41.4128261495	-41.4128261771
**50.0342857**	-41.4128261290	-41.4128261719	-41.4128261500	-41.4128261492	-41.4128261762
**50.1900000**	-41.4128261285	-41.4128261714	-41.4128261496	-41.4128261490	-41.4128261754
**50.3457143**	-41.4128261281	-41.4128261709	-41.4128261493	-41.4128261487	-41.4128261745
**50.5014286**	-41.4128261277	-41.4128261705	-41.4128261491	-41.4128261484	-41.4128261737
**50.6571429**	-41.4128261272	-41.4128261701	-41.4128261488	-41.4128261482	-41.4128261729
**50.8128571**	-41.4128261268	-41.4128261696	-41.4128261485	-41.4128261479	-41.4128261722
**50.9685714**	-41.4128261264	-41.4128261692	-41.4128261482	-41.4128261477	-41.4128261714
**51.1242857**	-41.4128261261	-41.4128261688	-41.4128261480	-41.4128261475	-41.4128261707
**51.2800000**	-41.4128261257	-41.4128261685	-41.4128261478	-41.4128261473	-41.4128261700
**51.4357143**	-41.4128261253	-41.4128261681	-41.4128261475	-41.4128261471	-41.4128261693
**51.5914286**	-41.4128261250	-41.4128261677	-41.4128261473	-41.4128261469	-41.4128261686
**51.7471429**	-41.4128261247	-41.4128261674	-41.4128261471	-41.4128261467	-41.4128261680
**51.9028571**	-41.4128261244	-41.4128261670	-41.4128261469	-41.4128261465	-41.4128261673
**52.0585714**	-41.4128261241	-41.4128261667	-41.4128261467	-41.4128261463	-41.4128261667
**52.2142857**	-41.4128261238	-41.4128261664	-41.4128261465	-41.4128261462	-41.4128261661
**52.3700000**	-41.4128261235	-41.4128261661	-41.4128261463	-41.4128261460	-41.4128261654
**52.5257143**	-41.4128261232	-41.4128261658	-41.4128261461	-41.4128261458	-41.4128261649
**52.6814286**	-41.4128261229	-41.4128261655	-41.4128261460	-41.4128261457	-41.4128261643
**52.8371429**	-41.4128261227	-41.4128261653	-41.4128261458	-41.4128261455	-41.4128261637
**52.9928571**	-41.4128261224	-41.4128261650	-41.4128261456	-41.4128261454	-41.4128261632
**53.1485714**	-41.4128261222	-41.4128261648	-41.4128261455	-41.4128261452	-41.4128261626
**53.3042857**	-41.4128261220	-41.4128261645	-41.4128261453	-41.4128261451	-41.4128261621
**53.4600000**	-41.4128261217	-41.4128261643	-41.4128261452	-41.4128261450	-41.4128261616
**53.6157143**	-41.4128261215	-41.4128261640	-41.4128261451	-41.4128261449	-41.4128261611
**53.7714286**	-41.4128261213	-41.4128261638	-41.4128261449	-41.4128261448	-41.4128261606
**53.9271429**	-41.4128261211	-41.4128261636	-41.4128261448	-41.4128261446	-41.4128261601
**54.0828571**	-41.4128261209	-41.4128261634	-41.4128261447	-41.4128261445	-41.4128261597
**54.2385714**	-41.4128261207	-41.4128261632	-41.4128261446	-41.4128261444	-41.4128261592
**54.3942857**	-41.4128261205	-41.4128261630	-41.4128261445	-41.4128261443	-41.4128261588
**54.5500000**	-41.4128261204	-41.4128261628	-41.4128261443	-41.4128261442	-41.4128261583
**54.7057143**	-41.4128261202	-41.4128261626	-41.4128261442	-41.4128261441	-41.4128261579
**54.8614286**	-41.4128261200	-41.4128261625	-41.4128261441	-41.4128261440	-41.4128261575
**55.0171429**	-41.4128261199	-41.4128261623	-41.4128261440	-41.4128261440	-41.4128261571
**55.1728571**	-41.4128261197	-41.4128261621	-41.4128261439	-41.4128261439	-41.4128261567
**55.3285714**	-41.4128261196	-41.4128261620	-41.4128261439	-41.4128261438	-41.4128261563
**55.4842857**	-41.4128261194	-41.4128261618	-41.4128261438	-41.4128261437	-41.4128261559
**55.6400000**	-41.4128261193	-41.4128261617	-41.4128261437	-41.4128261436	-41.4128261556
**55.7957143**	-41.4128261192	-41.4128261615	-41.4128261436	-41.4128261436	-41.4128261552

R (Bohr)/θ(º)	100.00	110.00	120.00	130.00	140.00
**55.9514286**	-41.4128261190	-41.4128261614	-41.4128261435	-41.4128261435	-41.4128261549
**56.1071429**	-41.4128261189	-41.4128261613	-41.4128261434	-41.4128261434	-41.4128261545
**56.2628571**	-41.4128261188	-41.4128261611	-41.4128261434	-41.4128261434	-41.4128261542
**56.4185714**	-41.4128261187	-41.4128261610	-41.4128261433	-41.4128261433	-41.4128261539
**56.5742857**	-41.4128261186	-41.4128261609	-41.4128261432	-41.4128261433	-41.4128261535
**56.7300000**	-41.4128261184	-41.4128261608	-41.4128261432	-41.4128261432	-41.4128261532
**56.8857143**	-41.4128261183	-41.4128261607	-41.4128261431	-41.4128261431	-41.4128261529
**57.0414286**	-41.4128261182	-41.4128261605	-41.4128261431	-41.4128261431	-41.4128261526
**57.1971429**	-41.4128261181	-41.4128261604	-41.4128261430	-41.4128261430	-41.4128261523
**57.3528571**	-41.4128261180	-41.4128261603	-41.4128261429	-41.4128261430	-41.4128261520
**57.5085714**	-41.4128261180	-41.4128261602	-41.4128261429	-41.4128261429	-41.4128261518
**57.6642857**	-41.4128261179	-41.4128261602	-41.4128261428	-41.4128261429	-41.4128261515
**57.8200000**	-41.4128261178	-41.4128261601	-41.4128261428	-41.4128261428	-41.4128261512
**57.9757143**	-41.4128261177	-41.4128261600	-41.4128261427	-41.4128261428	-41.4128261510
**58.1314286**	-41.4128261176	-41.4128261599	-41.4128261427	-41.4128261428	-41.4128261507
**58.2871429**	-41.4128261175	-41.4128261598	-41.4128261426	-41.4128261427	-41.4128261505
**58.4428571**	-41.4128261175	-41.4128261597	-41.4128261426	-41.4128261427	-41.4128261502
**58.5985714**	-41.4128261174	-41.4128261596	-41.4128261426	-41.4128261427	-41.4128261500
**58.7542857**	-41.4128261173	-41.4128261596	-41.4128261425	-41.4128261426	-41.4128261498
**58.9100000**	-41.4128261173	-41.4128261595	-41.4128261425	-41.4128261426	-41.4128261495
**59.0657143**	-41.4128261172	-41.4128261594	-41.4128261424	-41.4128261426	-41.4128261493
**59.2214286**	-41.4128261171	-41.4128261594	-41.4128261424	-41.4128261425	-41.4128261491
**59.3771429**	-41.4128261171	-41.4128261593	-41.4128261424	-41.4128261425	-41.4128261489
**59.5328571**	-41.4128261170	-41.4128261592	-41.4128261423	-41.4128261425	-41.4128261487
**59.6885714**	-41.4128261170	-41.4128261592	-41.4128261423	-41.4128261424	-41.4128261485
**59.8442857**	-41.4128261169	-41.4128261591	-41.4128261423	-41.4128261424	-41.4128261483
**60.0000000**	-41.4128261169	-41.4128261591	-41.4128261422	-41.4128261424	-41.4128261481

PES CBS – CH^- ($X\ ^3\Sigma^-$) + He				
**R (Bohr)/θ(º)**	**150.00**	**160.00**	**170.00**	**180.00**
**5.5000000**	-41.4109264168	-41.4109388500	-41.4109402475	-41.4109010403
**5.6557143**	-41.4113988447	-41.4114173016	-41.4114251589	-41.4113993725
**5.8114286**	-41.4117691028	-41.4117923349	-41.4118054274	-41.4117822023
**5.9671429**	-41.4120592771	-41.4120859636	-41.4121017036	-41.4120774077
**6.1228571**	-41.4122859644	-41.4123139850	-41.4123312249	-41.4123053173
**6.2785714**	-41.4124621743	-41.4124903440	-41.4125081966	-41.4124811143
**6.4342857**	-41.4125983821	-41.4126264878	-41.4126441100	-41.4126163451
**6.5900000**	-41.4127031057	-41.4127311860	-41.4127480784	-41.4127198961
**6.7457143**	-41.4127832431	-41.4128110881	-41.4128272000	-41.4127986568
**6.9014286**	-41.4128443050	-41.4128714024	-41.4128869302	-41.4128579915
**7.0571429**	-41.4128906112	-41.4129163494	-41.4129314327	-41.4129020905
**7.2128571**	-41.4129254794	-41.4129493706	-41.4129638901	-41.4129342373
**7.3685714**	-41.4129514106	-41.4129732043	-41.4129867589	-41.4129570147
**7.5242857**	-41.4129702666	-41.4129899411	-41.4130019676	-41.4129724636
**7.6800000**	-41.4129834296	-41.4130011100	-41.4130110618	-41.4129822039
**7.8357143**	-41.4129919392	-41.4130078005	-41.4130153054	-41.4129875258
**7.9914286**	-41.4129966014	-41.4130107972	-41.4130157476	-41.4129894596
**8.1471429**	-41.4129980694	-41.4130107013	-41.4130132662	-41.4129888273
**8.3028571**	-41.4129968995	-41.4130080218	-41.4130085959	-41.4129862831
**8.4585714**	-41.4129935859	-41.4130032309	-41.4130023474	-41.4129823450
**8.6142857**	-41.4129885782	-41.4129967865	-41.4129950221	-41.4129774196
**8.7700000**	-41.4129822888	-41.4129891331	-41.4129870245	-41.4129718236
**8.9257143**	-41.4129750924	-41.4129806892	-41.4129786748	-41.4129658013
**9.0814286**	-41.4129673235	-41.4129718311	-41.4129702202	-41.4129595389
**9.2371429**	-41.4129592719	-41.4129628792	-41.4129618464	-41.4129531775
**9.3928571**	-41.4129511800	-41.4129540895	-41.4129536886	-41.4129468231
**9.5485714**	-41.4129432425	-41.4129456518	-41.4129458413	-41.4129405547
**9.7042857**	-41.4129356069	-41.4129376931	-41.4129383667	-41.4129344310
**9.8600000**	-41.4129283776	-41.4129302857	-41.4129313022	-41.4129284954
**10.0157143**	-41.4129216200	-41.4129234570	-41.4129246663	-41.4129227795
**10.1714286**	-41.4129153669	-41.4129172006	-41.4129184634	-41.4129173058
**10.3271429**	-41.4129096246	-41.4129114869	-41.4129126875	-41.4129120895
**10.4828571**	-41.4129043791	-41.4129062720	-41.4129073251	-41.4129071398
**10.6385714**	-41.4128996020	-41.4129015048	-41.4129023576	-41.4129024611
**10.7942857**	-41.4128952560	-41.4128971334	-41.4128977623	-41.4128980532
**10.9500000**	-41.4128912985	-41.4128931080	-41.4128935147	-41.4128939121
**11.1057143**	-41.4128876859	-41.4128893837	-41.4128895888	-41.4128900309
**11.2614286**	-41.4128843752	-41.4128859218	-41.4128859582	-41.4128863999
**11.4171429**	-41.4128813269	-41.4128826896	-41.4128825969	-41.4128830072
**11.5728571**	-41.4128785052	-41.4128796606	-41.4128794800	-41.4128798396
**11.7285714**	-41.4128758787	-41.4128768135	-41.4128765835	-41.4128768829
**11.8842857**	-41.4128734211	-41.4128741313	-41.4128738856	-41.4128741223
**12.0400000**	-41.4128711101	-41.4128716009	-41.4128713662	-41.4128715431
**12.1957143**	-41.4128689279	-41.4128692117	-41.4128690074	-41.4128691310
**12.3514286**	-41.4128668600	-41.4128669553	-41.4128667934	-41.4128668725
**12.5071429**	-41.4128648952	-41.4128648245	-41.4128647105	-41.4128647548
**12.6628571**	-41.4128630246	-41.4128628133	-41.4128627472	-41.4128627664
**12.8185714**	-41.4128612415	-41.4128609161	-41.4128608935	-41.4128608968
**12.9742857**	-41.4128595404	-41.4128591276	-41.4128591414	-41.4128591368
**13.1300000**	-41.4128579173	-41.4128574427	-41.4128574840	-41.4128574783
**13.2857143**	-41.4128563690	-41.4128558565	-41.4128559158	-41.4128559141
**13.4414286**	-41.4128548926	-41.4128543640	-41.4128544320	-41.4128544380
**13.5971429**	-41.4128534859	-41.4128529602	-41.4128530288	-41.4128530447
**13.7528571**	-41.4128521467	-41.4128516401	-41.4128517025	-41.4128517294
**13.9085714**	-41.4128508730	-41.4128503989	-41.4128504500	-41.4128504878
**14.0642857**	-41.4128496630	-41.4128492319	-41.4128492685	-41.4128493162
**14.2200000**	-41.4128485147	-41.4128481343	-41.4128481548	-41.4128482111
**14.3757143**	-41.4128474262	-41.4128471016	-41.4128471063	-41.4128471690
**14.5314286**	-41.4128463954	-41.4128461297	-41.4128461199	-41.4128461869
**14.6871429**	-41.4128454203	-41.4128452143	-41.4128451927	-41.4128452618
**14.8428571**	-41.4128444988	-41.4128443517	-41.4128443214	-41.4128443905
**14.9985714**	-41.4128436288	-41.4128435382	-41.4128435030	-41.4128435701
**15.1542857**	-41.4128428081	-41.4128427704	-41.4128427342	-41.4128427977
**15.3100000**	-41.4128420344	-41.4128420452	-41.4128420118	-41.4128420703

R (Bohr)/θ(º)	150.00	160.00	170.00	180.00
**15.4657143**	-41.4128413056	-41.4128413596	-41.4128413325	-41.4128413851
**15.6214286**	-41.4128406193	-41.4128407110	-41.4128406932	-41.4128407393
**15.7771429**	-41.4128399733	-41.4128400969	-41.4128400909	-41.4128401301
**15.9328571**	-41.4128393654	-41.4128395148	-41.4128395225	-41.4128395550
**16.0885714**	-41.4128387935	-41.4128389629	-41.4128389854	-41.4128390113
**16.2442857**	-41.4128382552	-41.4128384390	-41.4128384767	-41.4128384967
**16.4000000**	-41.4128377485	-41.4128379414	-41.4128379943	-41.4128380090
**16.5557143**	-41.4128372713	-41.4128374686	-41.4128375357	-41.4128375460
**16.7114286**	-41.4128368216	-41.4128370188	-41.4128370991	-41.4128371059
**16.8671429**	-41.4128363975	-41.4128365909	-41.4128366826	-41.4128366869
**17.0228571**	-41.4128359971	-41.4128361835	-41.4128362846	-41.4128362873
**17.1785714**	-41.4128356186	-41.4128357953	-41.4128359036	-41.4128359057
**17.3342857**	-41.4128352605	-41.4128354254	-41.4128355385	-41.4128355408
**17.4900000**	-41.4128349211	-41.4128350726	-41.4128351882	-41.4128351916
**17.6457143**	-41.4128345990	-41.4128347361	-41.4128348518	-41.4128348569
**17.8014286**	-41.4128342929	-41.4128344150	-41.4128345286	-41.4128345359
**17.9571429**	-41.4128340014	-41.4128341085	-41.4128342177	-41.4128342278
**18.1128571**	-41.4128337236	-41.4128338157	-41.4128339188	-41.4128339320
**18.2685714**	-41.4128334582	-41.4128335360	-41.4128336314	-41.4128336478
**18.4242857**	-41.4128332044	-41.4128332687	-41.4128333550	-41.4128333748
**18.5800000**	-41.4128329614	-41.4128330132	-41.4128330893	-41.4128331125
**18.7357143**	-41.4128327283	-41.4128327688	-41.4128328340	-41.4128328606
**18.8914286**	-41.4128325046	-41.4128325351	-41.4128325888	-41.4128326186
**19.0471429**	-41.4128322895	-41.4128323115	-41.4128323536	-41.4128323862
**19.2028571**	-41.4128320827	-41.4128320975	-41.4128321280	-41.4128321633
**19.3585714**	-41.4128318836	-41.4128318926	-41.4128319118	-41.4128319494
**19.5142857**	-41.4128316917	-41.4128316964	-41.4128317049	-41.4128317443
**19.6700000**	-41.4128315069	-41.4128315084	-41.4128315069	-41.4128315479
**19.8257143**	-41.4128313287	-41.4128313284	-41.4128313177	-41.4128313597
**19.9814286**	-41.4128311569	-41.4128311558	-41.4128311369	-41.4128311797
**20.1371429**	-41.4128309912	-41.4128309904	-41.4128309644	-41.4128310074
**20.2928571**	-41.4128308314	-41.4128308317	-41.4128307999	-41.4128308428
**20.4485714**	-41.4128306774	-41.4128306795	-41.4128306430	-41.4128306855
**20.6042857**	-41.4128305290	-41.4128305335	-41.4128304936	-41.4128305353
**20.7600000**	-41.4128303860	-41.4128303934	-41.4128303513	-41.4128303918
**20.9157143**	-41.4128302483	-41.4128302588	-41.4128302158	-41.4128302549
**21.0714286**	-41.4128301156	-41.4128301296	-41.4128300868	-41.4128301243
**21.2271429**	-41.4128299880	-41.4128300055	-41.4128299641	-41.4128299997
**21.3828571**	-41.4128298653	-41.4128298863	-41.4128298472	-41.4128298808
**21.5385714**	-41.4128297473	-41.4128297716	-41.4128297360	-41.4128297674
**21.6942857**	-41.4128296339	-41.4128296614	-41.4128296300	-41.4128296592
**21.8500000**	-41.4128295250	-41.4128295554	-41.4128295291	-41.4128295558
**22.0057143**	-41.4128294204	-41.4128294534	-41.4128294328	-41.4128294572
**22.1614286**	-41.4128293200	-41.4128293553	-41.4128293410	-41.4128293630
**22.3171429**	-41.4128292238	-41.4128292608	-41.4128292533	-41.4128292729
**22.4728571**	-41.4128291315	-41.4128291699	-41.4128291695	-41.4128291867
**22.6285714**	-41.4128290429	-41.4128290822	-41.4128290893	-41.4128291042
**22.7842857**	-41.4128289581	-41.4128289978	-41.4128290125	-41.4128290252
**22.9400000**	-41.4128288768	-41.4128289164	-41.4128289387	-41.4128289494
**23.0957143**	-41.4128287988	-41.4128288379	-41.4128288680	-41.4128288766
**23.2514286**	-41.4128287241	-41.4128287622	-41.4128287999	-41.4128288066
**23.4071429**	-41.4128286525	-41.4128286892	-41.4128287343	-41.4128287393
**23.5628571**	-41.4128285839	-41.4128286187	-41.4128286710	-41.4128286744
**23.7185714**	-41.4128285181	-41.4128285507	-41.4128286099	-41.4128286119
**23.8742857**	-41.4128284549	-41.4128284850	-41.4128285508	-41.4128285514
**24.0300000**	-41.4128283943	-41.4128284215	-41.4128284935	-41.4128284930
**24.1857143**	-41.4128283361	-41.4128283602	-41.4128284380	-41.4128284364
**24.3414286**	-41.4128282802	-41.4128283009	-41.4128283840	-41.4128283816
**24.4971429**	-41.4128282265	-41.4128282436	-41.4128283315	-41.4128283283
**24.6528571**	-41.4128281748	-41.4128281882	-41.4128282803	-41.4128282766
**24.8085714**	-41.4128281250	-41.4128281346	-41.4128282305	-41.4128282263
**24.9642857**	-41.4128280770	-41.4128280827	-41.4128281818	-41.4128281773
**25.1200000**	-41.4128280307	-41.4128280325	-41.4128281342	-41.4128281295
**25.2757143**	-41.4128279861	-41.4128279838	-41.4128280877	-41.4128280829
**25.4314286**	-41.4128279429	-41.4128279367	-41.4128280421	-41.4128280373

R (Bohr)/θ(º)	150.00	160.00	170.00	180.00
**25.5871429**	-41.4128279011	-41.4128278911	-41.4128279975	-41.4128279928
**25.7428571**	-41.4128278607	-41.4128278469	-41.4128279538	-41.4128279493
**25.8985714**	-41.4128278215	-41.4128278041	-41.4128279109	-41.4128279067
**26.0542857**	-41.4128277835	-41.4128277625	-41.4128278688	-41.4128278650
**26.2100000**	-41.4128277465	-41.4128277222	-41.4128278275	-41.4128278241
**26.3657143**	-41.4128277106	-41.4128276831	-41.4128277869	-41.4128277840
**26.5214286**	-41.4128276757	-41.4128276452	-41.4128277471	-41.4128277447
**26.6771429**	-41.4128276417	-41.4128276084	-41.4128277079	-41.4128277062
**26.8328571**	-41.4128276085	-41.4128275727	-41.4128276695	-41.4128276684
**26.9885714**	-41.4128275761	-41.4128275380	-41.4128276317	-41.4128276312
**27.1442857**	-41.4128275445	-41.4128275043	-41.4128275946	-41.4128275948
**27.3000000**	-41.4128275136	-41.4128274715	-41.4128275581	-41.4128275590
**27.4557143**	-41.4128274834	-41.4128274397	-41.4128275222	-41.4128275239
**27.6114286**	-41.4128274538	-41.4128274088	-41.4128274870	-41.4128274894
**27.7671429**	-41.4128274248	-41.4128273788	-41.4128274524	-41.4128274555
**27.9228571**	-41.4128273964	-41.4128273496	-41.4128274184	-41.4128274223
**28.0785714**	-41.4128273686	-41.4128273212	-41.4128273850	-41.4128273897
**28.2342857**	-41.4128273413	-41.4128272936	-41.4128273523	-41.4128273577
**28.3900000**	-41.4128273145	-41.4128272667	-41.4128273202	-41.4128273262
**28.5457143**	-41.4128272882	-41.4128272406	-41.4128272886	-41.4128272954
**28.7014286**	-41.4128272624	-41.4128272151	-41.4128272577	-41.4128272652
**28.8571429**	-41.4128272371	-41.4128271903	-41.4128272274	-41.4128272355
**29.0128571**	-41.4128272122	-41.4128271662	-41.4128271976	-41.4128272065
**29.1685714**	-41.4128271877	-41.4128271428	-41.4128271685	-41.4128271779
**29.3242857**	-41.4128271637	-41.4128271199	-41.4128271399	-41.4128271500
**29.4800000**	-41.4128271401	-41.4128270976	-41.4128271120	-41.4128271226
**29.6357143**	-41.4128271169	-41.4128270760	-41.4128270846	-41.4128270958
**29.7914286**	-41.4128270940	-41.4128270548	-41.4128270579	-41.4128270696
**29.9471429**	-41.4128270716	-41.4128270342	-41.4128270317	-41.4128270439
**30.1028571**	-41.4128270496	-41.4128270142	-41.4128270060	-41.4128270187
**30.2585714**	-41.4128270280	-41.4128269946	-41.4128269810	-41.4128269941
**30.4142857**	-41.4128270067	-41.4128269756	-41.4128269565	-41.4128269701
**30.5700000**	-41.4128269858	-41.4128269570	-41.4128269326	-41.4128269465
**30.7257143**	-41.4128269653	-41.4128269389	-41.4128269093	-41.4128269235
**30.8814286**	-41.4128269451	-41.4128269212	-41.4128268865	-41.4128269011
**31.0371429**	-41.4128269254	-41.4128269039	-41.4128268642	-41.4128268791
**31.1928571**	-41.4128269059	-41.4128268871	-41.4128268425	-41.4128268577
**31.3485714**	-41.4128268869	-41.4128268707	-41.4128268213	-41.4128268367
**31.5042857**	-41.4128268682	-41.4128268547	-41.4128268007	-41.4128268163
**31.6600000**	-41.4128268498	-41.4128268391	-41.4128267805	-41.4128267964
**31.8157143**	-41.4128268318	-41.4128268239	-41.4128267609	-41.4128267769
**31.9714286**	-41.4128268142	-41.4128268090	-41.4128267418	-41.4128267579
**32.1271429**	-41.4128267968	-41.4128267945	-41.4128267232	-41.4128267394
**32.2828571**	-41.4128267799	-41.4128267803	-41.4128267051	-41.4128267214
**32.4385714**	-41.4128267633	-41.4128267665	-41.4128266874	-41.4128267038
**32.5942857**	-41.4128267470	-41.4128267530	-41.4128266703	-41.4128266867
**32.7500000**	-41.4128267310	-41.4128267398	-41.4128266536	-41.4128266701
**32.9057143**	-41.4128267154	-41.4128267269	-41.4128266373	-41.4128266538
**33.0614286**	-41.4128267001	-41.4128267143	-41.4128266216	-41.4128266380
**33.2171429**	-41.4128266851	-41.4128267020	-41.4128266062	-41.4128266227
**33.3728571**	-41.4128266705	-41.4128266900	-41.4128265913	-41.4128266077
**33.5285714**	-41.4128266562	-41.4128266783	-41.4128265768	-41.4128265931
**33.6842857**	-41.4128266422	-41.4128266669	-41.4128265627	-41.4128265790
**33.8400000**	-41.4128266285	-41.4128266557	-41.4128265490	-41.4128265652
**33.9957143**	-41.4128266151	-41.4128266448	-41.4128265358	-41.4128265518
**34.1514286**	-41.4128266020	-41.4128266341	-41.4128265229	-41.4128265388
**34.3071429**	-41.4128265892	-41.4128266236	-41.4128265104	-41.4128265262
**34.4628571**	-41.4128265767	-41.4128266134	-41.4128264982	-41.4128265139
**34.6185714**	-41.4128265645	-41.4128266035	-41.4128264864	-41.4128265020
**34.7742857**	-41.4128265526	-41.4128265937	-41.4128264750	-41.4128264904
**34.9300000**	-41.4128265410	-41.4128265842	-41.4128264639	-41.4128264792
**35.0857143**	-41.4128265297	-41.4128265749	-41.4128264532	-41.4128264683
**35.2414286**	-41.4128265186	-41.4128265658	-41.4128264428	-41.4128264577
**35.3971429**	-41.4128265078	-41.4128265569	-41.4128264327	-41.4128264474
**35.5528571**	-41.4128264973	-41.4128265482	-41.4128264229	-41.4128264374

R (Bohr)/θ(º)	150.00	160.00	170.00	180.00
**35.7085714**	-41.4128264870	-41.4128265397	-41.4128264134	-41.4128264277
**35.8642857**	-41.4128264770	-41.4128265314	-41.4128264042	-41.4128264183
**36.0200000**	-41.4128264673	-41.4128265233	-41.4128263953	-41.4128264092
**36.1757143**	-41.4128264578	-41.4128265153	-41.4128263867	-41.4128264004
**36.3314286**	-41.4128264485	-41.4128265075	-41.4128263783	-41.4128263918
**36.4871429**	-41.4128264395	-41.4128264999	-41.4128263703	-41.4128263835
**36.6428571**	-41.4128264307	-41.4128264925	-41.4128263624	-41.4128263754
**36.7985714**	-41.4128264222	-41.4128264852	-41.4128263549	-41.4128263676
**36.9542857**	-41.4128264139	-41.4128264781	-41.4128263475	-41.4128263601
**37.1100000**	-41.4128264058	-41.4128264712	-41.4128263404	-41.4128263528
**37.2657143**	-41.4128263979	-41.4128264643	-41.4128263336	-41.4128263457
**37.4214286**	-41.4128263902	-41.4128264577	-41.4128263269	-41.4128263388
**37.5771429**	-41.4128263827	-41.4128264512	-41.4128263205	-41.4128263321
**37.7328571**	-41.4128263755	-41.4128264448	-41.4128263143	-41.4128263257
**37.8885714**	-41.4128263684	-41.4128264386	-41.4128263082	-41.4128263194
**38.0442857**	-41.4128263615	-41.4128264325	-41.4128263024	-41.4128263134
**38.2000000**	-41.4128263549	-41.4128264265	-41.4128262968	-41.4128263075
**38.3557143**	-41.4128263484	-41.4128264207	-41.4128262914	-41.4128263019
**38.5114286**	-41.4128263421	-41.4128264150	-41.4128262861	-41.4128262964
**38.6671429**	-41.4128263359	-41.4128264094	-41.4128262810	-41.4128262911
**38.8228571**	-41.4128263300	-41.4128264039	-41.4128262761	-41.4128262859
**38.9785714**	-41.4128263242	-41.4128263985	-41.4128262713	-41.4128262809
**39.1342857**	-41.4128263186	-41.4128263933	-41.4128262667	-41.4128262761
**39.2900000**	-41.4128263131	-41.4128263882	-41.4128262623	-41.4128262715
**39.4457143**	-41.4128263078	-41.4128263831	-41.4128262580	-41.4128262670
**39.6014286**	-41.4128263026	-41.4128263782	-41.4128262539	-41.4128262626
**39.7571429**	-41.4128262976	-41.4128263734	-41.4128262499	-41.4128262584
**39.9128571**	-41.4128262928	-41.4128263687	-41.4128262460	-41.4128262543
**40.0685714**	-41.4128262880	-41.4128263641	-41.4128262422	-41.4128262504
**40.2242857**	-41.4128262835	-41.4128263596	-41.4128262386	-41.4128262465
**40.3800000**	-41.4128262790	-41.4128263552	-41.4128262351	-41.4128262429
**40.5357143**	-41.4128262747	-41.4128263509	-41.4128262318	-41.4128262393
**40.6914286**	-41.4128262705	-41.4128263466	-41.4128262285	-41.4128262358
**40.8471429**	-41.4128262664	-41.4128263425	-41.4128262254	-41.4128262325
**41.0028571**	-41.4128262625	-41.4128263385	-41.4128262223	-41.4128262293
**41.1585714**	-41.4128262587	-41.4128263345	-41.4128262194	-41.4128262262
**41.3142857**	-41.4128262550	-41.4128263306	-41.4128262166	-41.4128262231
**41.4700000**	-41.4128262514	-41.4128263268	-41.4128262138	-41.4128262202
**41.6257143**	-41.4128262479	-41.4128263231	-41.4128262112	-41.4128262174
**41.7814286**	-41.4128262445	-41.4128263194	-41.4128262086	-41.4128262147
**41.9371429**	-41.4128262412	-41.4128263159	-41.4128262062	-41.4128262121
**42.0928571**	-41.4128262380	-41.4128263124	-41.4128262038	-41.4128262095
**42.2485714**	-41.4128262349	-41.4128263089	-41.4128262015	-41.4128262071
**42.4042857**	-41.4128262319	-41.4128263056	-41.4128261993	-41.4128262047
**42.5600000**	-41.4128262290	-41.4128263023	-41.4128261972	-41.4128262024
**42.7157143**	-41.4128262262	-41.4128262991	-41.4128261951	-41.4128262002
**42.8714286**	-41.4128262235	-41.4128262960	-41.4128261931	-41.4128261980
**43.0271429**	-41.4128262208	-41.4128262929	-41.4128261912	-41.4128261960
**43.1828571**	-41.4128262183	-41.4128262899	-41.4128261893	-41.4128261940
**43.3385714**	-41.4128262158	-41.4128262869	-41.4128261875	-41.4128261921
**43.4942857**	-41.4128262134	-41.4128262840	-41.4128261858	-41.4128261902
**43.6500000**	-41.4128262110	-41.4128262812	-41.4128261842	-41.4128261884
**43.8057143**	-41.4128262088	-41.4128262784	-41.4128261825	-41.4128261866
**43.9614286**	-41.4128262066	-41.4128262757	-41.4128261810	-41.4128261850
**44.1171429**	-41.4128262045	-41.4128262730	-41.4128261795	-41.4128261833
**44.2728571**	-41.4128262024	-41.4128262704	-41.4128261781	-41.4128261818
**44.4285714**	-41.4128262004	-41.4128262679	-41.4128261767	-41.4128261803
**44.5842857**	-41.4128261985	-41.4128262654	-41.4128261753	-41.4128261788
**44.7400000**	-41.4128261966	-41.4128262629	-41.4128261740	-41.4128261774
**44.8957143**	-41.4128261948	-41.4128262605	-41.4128261728	-41.4128261760
**45.0514286**	-41.4128261931	-41.4128262581	-41.4128261716	-41.4128261747
**45.2071429**	-41.4128261914	-41.4128262558	-41.4128261704	-41.4128261734
**45.3628571**	-41.4128261897	-41.4128262536	-41.4128261693	-41.4128261722
**45.5185714**	-41.4128261881	-41.4128262514	-41.4128261682	-41.4128261710
**45.6742857**	-41.4128261866	-41.4128262492	-41.4128261671	-41.4128261699

R (Bohr)/θ(º)	150.00	160.00	170.00	180.00
**45.8300000**	-41.4128261851	-41.4128262471	-41.4128261661	-41.4128261688
**45.9857143**	-41.4128261836	-41.4128262450	-41.4128261652	-41.4128261677
**46.1414286**	-41.4128261822	-41.4128262430	-41.4128261642	-41.4128261667
**46.2971429**	-41.4128261809	-41.4128262410	-41.4128261633	-41.4128261657
**46.4528571**	-41.4128261795	-41.4128262390	-41.4128261625	-41.4128261647
**46.6085714**	-41.4128261783	-41.4128262371	-41.4128261616	-41.4128261638
**46.7642857**	-41.4128261770	-41.4128262352	-41.4128261608	-41.4128261629
**46.9200000**	-41.4128261758	-41.4128262334	-41.4128261600	-41.4128261620
**47.0757143**	-41.4128261747	-41.4128262316	-41.4128261593	-41.4128261612
**47.2314286**	-41.4128261736	-41.4128262298	-41.4128261585	-41.4128261604
**47.3871429**	-41.4128261725	-41.4128262281	-41.4128261578	-41.4128261596
**47.5428571**	-41.4128261714	-41.4128262264	-41.4128261572	-41.4128261589
**47.6985714**	-41.4128261704	-41.4128262248	-41.4128261565	-41.4128261581
**47.8542857**	-41.4128261694	-41.4128262231	-41.4128261559	-41.4128261574
**48.0100000**	-41.4128261685	-41.4128262216	-41.4128261553	-41.4128261568
**48.1657143**	-41.4128261676	-41.4128262200	-41.4128261547	-41.4128261561
**48.3214286**	-41.4128261667	-41.4128262185	-41.4128261541	-41.4128261555
**48.4771429**	-41.4128261658	-41.4128262170	-41.4128261536	-41.4128261549
**48.6328571**	-41.4128261650	-41.4128262155	-41.4128261530	-41.4128261543
**48.7885714**	-41.4128261642	-41.4128262141	-41.4128261525	-41.4128261537
**48.9442857**	-41.4128261634	-41.4128262126	-41.4128261521	-41.4128261532
**49.1000000**	-41.4128261626	-41.4128262113	-41.4128261516	-41.4128261526
**49.2557143**	-41.4128261619	-41.4128262099	-41.4128261511	-41.4128261521
**49.4114286**	-41.4128261612	-41.4128262086	-41.4128261507	-41.4128261516
**49.5671429**	-41.4128261605	-41.4128262073	-41.4128261503	-41.4128261512
**49.7228571**	-41.4128261599	-41.4128262060	-41.4128261499	-41.4128261507
**49.8785714**	-41.4128261592	-41.4128262048	-41.4128261495	-41.4128261503
**50.0342857**	-41.4128261586	-41.4128262035	-41.4128261491	-41.4128261498
**50.1900000**	-41.4128261580	-41.4128262023	-41.4128261487	-41.4128261494
**50.3457143**	-41.4128261574	-41.4128262012	-41.4128261484	-41.4128261490
**50.5014286**	-41.4128261568	-41.4128262000	-41.4128261480	-41.4128261487
**50.6571429**	-41.4128261563	-41.4128261989	-41.4128261477	-41.4128261483
**50.8128571**	-41.4128261558	-41.4128261978	-41.4128261474	-41.4128261479
**50.9685714**	-41.4128261553	-41.4128261967	-41.4128261471	-41.4128261476
**51.1242857**	-41.4128261548	-41.4128261956	-41.4128261468	-41.4128261473
**51.2800000**	-41.4128261543	-41.4128261946	-41.4128261465	-41.4128261469
**51.4357143**	-41.4128261538	-41.4128261936	-41.4128261462	-41.4128261466
**51.5914286**	-41.4128261534	-41.4128261926	-41.4128261460	-41.4128261463
**51.7471429**	-41.4128261530	-41.4128261916	-41.4128261457	-41.4128261460
**51.9028571**	-41.4128261525	-41.4128261906	-41.4128261455	-41.4128261458
**52.0585714**	-41.4128261521	-41.4128261897	-41.4128261453	-41.4128261455
**52.2142857**	-41.4128261517	-41.4128261888	-41.4128261450	-41.4128261452
**52.3700000**	-41.4128261514	-41.4128261879	-41.4128261448	-41.4128261450
**52.5257143**	-41.4128261510	-41.4128261870	-41.4128261446	-41.4128261447
**52.6814286**	-41.4128261506	-41.4128261861	-41.4128261444	-41.4128261445
**52.8371429**	-41.4128261503	-41.4128261853	-41.4128261442	-41.4128261443
**52.9928571**	-41.4128261500	-41.4128261844	-41.4128261440	-41.4128261441
**53.1485714**	-41.4128261496	-41.4128261836	-41.4128261438	-41.4128261439
**53.3042857**	-41.4128261493	-41.4128261828	-41.4128261437	-41.4128261437
**53.4600000**	-41.4128261490	-41.4128261820	-41.4128261435	-41.4128261435
**53.6157143**	-41.4128261487	-41.4128261812	-41.4128261433	-41.4128261433
**53.7714286**	-41.4128261485	-41.4128261805	-41.4128261432	-41.4128261431
**53.9271429**	-41.4128261482	-41.4128261797	-41.4128261430	-41.4128261429
**54.0828571**	-41.4128261479	-41.4128261790	-41.4128261429	-41.4128261428
**54.2385714**	-41.4128261477	-41.4128261783	-41.4128261427	-41.4128261426
**54.3942857**	-41.4128261474	-41.4128261776	-41.4128261426	-41.4128261424
**54.5500000**	-41.4128261472	-41.4128261769	-41.4128261425	-41.4128261423
**54.7057143**	-41.4128261470	-41.4128261762	-41.4128261423	-41.4128261421
**54.8614286**	-41.4128261467	-41.4128261756	-41.4128261422	-41.4128261420
**55.0171429**	-41.4128261465	-41.4128261749	-41.4128261421	-41.4128261419
**55.1728571**	-41.4128261463	-41.4128261743	-41.4128261420	-41.4128261417
**55.3285714**	-41.4128261461	-41.4128261737	-41.4128261419	-41.4128261416
**55.4842857**	-41.4128261459	-41.4128261731	-41.4128261418	-41.4128261415
**55.6400000**	-41.4128261457	-41.4128261725	-41.4128261417	-41.4128261414
**55.7957143**	-41.4128261456	-41.4128261719	-41.4128261416	-41.4128261413

R (Bohr)/θ(º)	150.00	160.00	170.00	180.00
**55.9514286**	-41.4128261454	-41.4128261713	-41.4128261415	-41.4128261411
**56.1071429**	-41.4128261452	-41.4128261708	-41.4128261414	-41.4128261410
**56.2628571**	-41.4128261450	-41.4128261702	-41.4128261413	-41.4128261409
**56.4185714**	-41.4128261449	-41.4128261697	-41.4128261412	-41.4128261408
**56.5742857**	-41.4128261447	-41.4128261691	-41.4128261411	-41.4128261407
**56.7300000**	-41.4128261446	-41.4128261686	-41.4128261411	-41.4128261407
**56.8857143**	-41.4128261444	-41.4128261681	-41.4128261410	-41.4128261406
**57.0414286**	-41.4128261443	-41.4128261676	-41.4128261409	-41.4128261405
**57.1971429**	-41.4128261442	-41.4128261671	-41.4128261408	-41.4128261404
**57.3528571**	-41.4128261440	-41.4128261666	-41.4128261408	-41.4128261403
**57.5085714**	-41.4128261439	-41.4128261662	-41.4128261407	-41.4128261402
**57.6642857**	-41.4128261438	-41.4128261657	-41.4128261406	-41.4128261402
**57.8200000**	-41.4128261437	-41.4128261653	-41.4128261406	-41.4128261401
**57.9757143**	-41.4128261435	-41.4128261648	-41.4128261405	-41.4128261400
**58.1314286**	-41.4128261434	-41.4128261644	-41.4128261405	-41.4128261400
**58.2871429**	-41.4128261433	-41.4128261639	-41.4128261404	-41.4128261399
**58.4428571**	-41.4128261432	-41.4128261635	-41.4128261404	-41.4128261398
**58.5985714**	-41.4128261431	-41.4128261631	-41.4128261403	-41.4128261398
**58.7542857**	-41.4128261430	-41.4128261627	-41.4128261403	-41.4128261397
**58.9100000**	-41.4128261429	-41.4128261623	-41.4128261402	-41.4128261397
**59.0657143**	-41.4128261429	-41.4128261619	-41.4128261402	-41.4128261396
**59.2214286**	-41.4128261428	-41.4128261616	-41.4128261401	-41.4128261396
**59.3771429**	-41.4128261427	-41.4128261612	-41.4128261401	-41.4128261395
**59.5328571**	-41.4128261426	-41.4128261608	-41.4128261400	-41.4128261395
**59.6885714**	-41.4128261425	-41.4128261605	-41.4128261400	-41.4128261394
**59.8442857**	-41.4128261424	-41.4128261601	-41.4128261400	-41.4128261394
**60.0000000**	-41.4128261424	-41.4128261598	-41.4128261399	-41.4128261393

Inelastic collisions cross-sections								
J = 0 → J' = 1			J = 0 → J' = 2			J = 0 → J' = 3		
E_{tot} (cm^{-1})	E_{col} (cm^{-1})	σ (Å)	E_{tot} (cm^{-1})	E_{col} (cm^{-1})	σ (Å)	E_{tot} (cm^{-1})	E_{col} (cm^{-1})	σ (Å)
27.3583817	0.0001000	0.0125247	82.0749450	0.0001000	0.0003660	164.1497900	0.0001000	0.0007789
27.3584817	0.0002000	0.0101795	82.0750450	0.0002000	0.0003521	164.1498900	0.0002000	0.0008227
27.3585817	0.0003000	0.0094892	82.0751450	0.0003000	0.0003804	164.1499900	0.0003000	0.0009193
27.3586817	0.0004000	0.0094106	82.0752450	0.0004000	0.0004348	164.1500900	0.0004000	0.0010666
27.3587817	0.0005000	0.0096690	82.0753450	0.0005000	0.0005091	164.1501900	0.0005000	0.0012581
27.3588817	0.0006000	0.0101540	82.0754450	0.0006000	0.0005997	164.1502900	0.0006000	0.0014885
27.3589817	0.0007000	0.0108024	82.0755450	0.0007000	0.0007048	164.1503900	0.0007000	0.0017538
27.3590817	0.0008000	0.0115764	82.0756450	0.0008000	0.0008229	164.1504900	0.0008000	0.0020497
27.3591817	0.0009000	0.0124505	82.0757450	0.0009000	0.0009528	164.1505900	0.0009000	0.0023721
27.3592817	0.0010000	0.0134108	82.0758450	0.0010000	0.0010937	164.1506900	0.0010000	0.0027172
27.3602817	0.0020000	0.0255505	82.0768450	0.0020000	0.0029793	164.1516900	0.0020000	0.0065699
27.3612817	0.0030000	0.0391000	82.0778450	0.0030000	0.0054716	164.1526900	0.0030000	0.0092322
27.3622817	0.0040000	0.0521678	82.0788450	0.0040000	0.0083991	164.1596900	0.0100000	0.0067680
27.3632817	0.0050000	0.0644756	82.0798450	0.0050000	0.0117543	164.1696900	0.0200000	0.0049444
27.3642817	0.0060000	0.0762018	82.0808450	0.0060000	0.0156099	164.1796900	0.0300000	0.0048831
27.3652817	0.0070000	0.0877548	82.0818450	0.0070000	0.0200892	164.1896900	0.0400000	0.0051911
27.3662817	0.0080000	0.0994909	82.0828450	0.0080000	0.0253379	164.1996900	0.0500000	0.0056805
27.3672817	0.0090000	0.1117395	82.0838450	0.0090000	0.0315052	164.2096900	0.0600000	0.0063947
27.3682817	0.0100000	0.1248163	82.0848450	0.0100000	0.0387384	164.2196900	0.0700000	0.0075118
27.3782817	0.0200000	0.2684669	82.0948450	0.0200000	0.0977237	164.2296900	0.0800000	0.0094089
27.3882817	0.0300000	0.3654872	82.1048450	0.0300000	0.0429638	164.2396900	0.0900000	0.0129295
27.3982817	0.0400000	0.4455810	82.1148450	0.0400000	0.0240688	164.2496900	0.1000000	0.0191871
27.4082817	0.0500000	0.5212311	82.1248450	0.0500000	0.0174151	164.3496900	0.2000000	0.0291316
27.4182817	0.0600000	0.5978341	82.1348450	0.0600000	0.0145965	164.4496900	0.3000000	0.0215696
27.4282817	0.0700000	0.6802070	82.1448450	0.0700000	0.0135464	164.5496900	0.4000000	0.0169071
27.4382817	0.0800000	0.7736268	82.1548450	0.0800000	0.0138076	164.6496900	0.5000000	0.0165071
27.4482817	0.0900000	0.8806954	82.1648450	0.0900000	0.0156589	164.7496900	0.6000000	0.0295939
27.4582817	0.1000000	0.9966103	82.1748450	0.1000000	0.0197657	164.8496900	0.7000000	0.0247277
27.5582817	0.2000000	0.9708140	82.2748450	0.2000000	0.0206776	164.9496900	0.8000000	0.0257671
27.6582817	0.3000000	0.9998009	82.3748450	0.3000000	0.0287099	165.0496900	0.9000000	0.0297631
27.7582817	0.4000000	1.3903655	82.4748450	0.4000000	0.0201310	165.1496900	1.0000000	0.0425546
27.8582817	0.5000000	1.6530475	82.5748450	0.5000000	0.0202473	166.1496900	2.0000000	0.0335419
27.9582817	0.6000000	1.9477445	82.6748450	0.6000000	0.0304170	167.1496900	3.0000000	0.0439099
28.0582817	0.7000000	5.9961763	82.7748450	0.7000000	0.1152331	168.1496900	4.0000000	0.0713249
28.1582817	0.8000000	3.5924202	82.8748450	0.8000000	0.0345132	169.1496900	5.0000000	0.0542094
28.2582817	0.9000000	2.9188511	82.9748450	0.9000000	0.0331781	170.1496900	6.0000000	0.0567436
28.3582817	1.0000000	2.9160332	83.0748450	1.0000000	0.0421896	171.1496900	7.0000000	0.0633780
29.3582817	2.0000000	3.2220656	84.0748450	2.0000000	0.0518404	172.0748450	90.0000000	0.0661053
30.3582817	3.0000000	4.4855144	85.0748450	3.0000000	0.0498571	172.1496900	8.0000000	0.0661928
31.3582817	4.0000000	12.9976380	86.0748450	4.0000000	0.0947521	173.1496900	9.0000000	0.0680087
32.3582817	5.0000000	5.4396478	87.0748450	5.0000000	0.0622165	174.1496900	10.0000000	0.0708982
33.3582817	6.0000000	5.5581431	87.3582817	60.0000000	0.0619675	182.0748450	100.0000000	0.0975347
34.3582817	7.0000000	6.7951216	88.0748450	6.0000000	0.0641074	184.1496900	20.0000000	0.1050843
35.3582817	8.0000000	6.7744277	89.0748450	7.0000000	0.0750471	194.1496900	30.0000000	0.1455967
36.3582817	9.0000000	6.3895737	90.0748450	8.0000000	0.0765331	204.1496900	40.0000000	0.1929196
37.3582817	10.0000000	6.4100838	91.0748450	9.0000000	0.0768107	214.1496900	50.0000000	0.2469350
47.3582817	20.0000000	6.8599438	92.0748450	10.0000000	0.0797358	224.1496900	60.0000000	0.3072821
57.3582817	30.0000000	7.0877463	97.3582817	70.0000000	0.0972584	227.3582820	200.0000000	0.3279030
67.3582817	40.0000000	7.2519440	102.0748450	20.0000000	0.1138724	234.1496900	70.0000000	0.3734686
77.3582817	50.0000000	7.2895825	107.3582820	80.0000000	0.1343318	244.1496900	80.0000000	0.4448577
82.0749450	0.0001000	7.2006212	112.0748450	30.0000000	0.1542295	254.1496900	90.0000000	0.5207817
82.0750450	0.0002000	7.2005391	117.3582820	90.0000000	0.1783543	264.1496900	100.0000000	0.6003876
82.0751450	0.0003000	7.2004226	122.0748450	40.0000000	0.2014819	273.5829170	0.0001000	0.6767878
82.0752450	0.0004000	7.2002942	127.3582820	100.0000000	0.2290917	273.5830170	0.0002000	0.6768145
82.0753450	0.0005000	7.2001659	132.0748450	50.0000000	0.2551655	273.5831170	0.0003000	0.6768437
82.0754450	0.0006000	7.2000393	142.0748450	60.0000000	0.3141329	273.5832170	0.0004000	0.6768725
82.0755450	0.0007000	7.1999164	152.0748450	70.0000000	0.7412689	273.5833170	0.0005000	0.6769003
82.0756450	0.0008000	7.1997966	162.0748450	80.0000000	0.4594316	273.5834170	0.0006000	0.6769272
82.0757450	0.0009000	7.1996763	164.1497900	0.0001000	0.5247015	273.5835170	0.0007000	0.6769531
82.0758450	0.0010000	7.1995603	164.1498900	0.0002000	0.5276882	273.5836170	0.0008000	0.6769782
82.0768450	0.0020000	7.1987118	164.1499900	0.0003000	0.5306143	273.5837170	0.0009000	0.6770024
82.0778450	0.0030000	7.1985580	164.1500900	0.0004000	0.5334936	273.5838170	0.0010000	0.6770258
82.0788450	0.0040000	7.1992894	164.1501900	0.0005000	0.5362979	273.5848170	0.0020000	0.6772235
82.0798450	0.0050000	7.2012403	164.1502900	0.0006000	0.5390122	273.5858170	0.0030000	0.6773730
82.0808450	0.0060000	7.2048303	164.1503900	0.0007000	0.5416321	273.5868170	0.0040000	0.6774902
82.0818450	0.0070000	7.2105729	164.1504900	0.0008000	0.5441291	273.5878170	0.0050000	0.6775842
82.0828450	0.0080000	7.2192229	164.1505900	0.0009000	0.5464818	273.5888170	0.0060000	0.6776603
82.0838450	0.0090000	7.2316042	164.1506900	0.0010000	0.5486880	273.5898170	0.0070000	0.6777239
82.0848450	0.0100000	7.2488229	164.1516900	0.0020000	0.5603553	273.5908170	0.0080000	0.6777776
82.0948450	0.0200000	7.6029465	164.1526900	0.0030000	0.5549276	273.5918170	0.0090000	0.6778234
82.1048450	0.0300000	7.4873553	164.1596900	0.0100000	0.4906545	273.5928170	0.0100000	0.6778633
82.1148450	0.0400000	7.4028986	164.1696900	0.0200000	0.4709893	273.6028170	0.0200000	0.6780911
82.1248450	0.0500000	7.3630252	164.1796900	0.0300000	0.4647128	273.6128170	0.0300000	0.6782016
82.1348450	0.0600000	7.3403659	164.1896900	0.0400000	0.4613075	273.6228170	0.0400000	0.6782698
82.1448450	0.0700000	7.3252473	164.1996900	0.0500000	0.4589275	273.6328170	0.0500000	0.6782822
82.1548450	0.0800000	7.3140022	164.2096900	0.0600000	0.4572109	273.6428170	0.0600000	0.6780986
82.1648450	0.0900000	7.3047633	164.2196900	0.0700000	0.4569398	273.6528170	0.0700000	0.6742430

J = 0 → J' = 1			J = 0 → J' = 2			J = 0 → J' = 3		
E_{tot} (cm⁻¹)	E_{col} (cm⁻¹)	σ (Å)	E_{tot} (cm⁻¹)	E_{col} (cm⁻¹)	σ (Å)	E_{tot} (cm⁻¹)	E_{col} (cm⁻¹)	σ (Å)
82.1748450	0.1000000	7.2974776	164.2296900	0.0800000	0.4642829	273.6628170	0.0800000	0.6699538
82.2748450	0.2000000	7.2755901	164.2396900	0.0900000	0.5571768	273.6728170	0.0900000	0.6780202
82.3748450	0.3000000	7.2575540	164.2496900	0.1000000	0.7294428	273.6828170	0.1000000	0.6782496
82.4748450	0.4000000	7.2693062	164.3496900	0.2000000	0.4708663	273.7828170	0.2000000	0.6787839
82.5748450	0.5000000	7.2453933	164.4496900	0.3000000	0.4653602	273.8828170	0.3000000	0.6794942
82.6748450	0.6000000	7.1910002	164.5496900	0.4000000	0.4652439	273.9828170	0.4000000	0.6807874
82.7748450	0.7000000	7.5745244	164.6496900	0.5000000	0.4591093	274.0828170	0.5000000	0.6806013
82.8748450	0.8000000	7.0953502	164.7496900	0.6000000	0.4708581	274.1828170	0.6000000	0.6819288
82.9748450	0.9000000	7.2813573	164.8496900	0.7000000	0.4711178	274.2828170	0.7000000	0.6840106
83.0748450	1.0000000	7.2703582	164.9496900	0.8000000	0.4954745	274.3828170	0.8000000	0.6840820
84.0748450	2.0000000	7.2892677	165.0496900	0.9000000	0.4765744	274.4828170	0.9000000	0.6846222
85.0748450	3.0000000	7.2632643	165.1496900	1.0000000	0.4722558	274.5828170	1.0000000	0.6845332
86.0748450	4.0000000	7.2525750	166.1496900	2.0000000	0.4804025	275.5828170	2.0000000	0.6935351
87.0748450	5.0000000	7.2598602	167.1496900	3.0000000	0.4856420	276.5828170	3.0000000	0.7016086
87.3582817	60.0000000	7.2584336	168.1496900	4.0000000	0.4970011	277.5828170	4.0000000	0.7096342
88.0748450	6.0000000	7.2546804	169.1496900	5.0000000	0.5020340	278.5828170	5.0000000	0.7181900
89.0748450	7.0000000	7.2485349	170.1496900	6.0000000	0.5090236	279.5828170	6.0000000	0.7265463
90.0748450	8.0000000	7.2455441	171.1496900	7.0000000	0.5164598	280.5828170	7.0000000	0.7348472
91.0748450	9.0000000	7.2410521	172.0748450	90.0000000	0.5239528	281.5828170	8.0000000	0.7432545
92.0748450	10.0000000	7.2358214	172.1496900	8.0000000	0.5245235	282.0748450	200.0000000	0.7474003
97.3582817	70.0000000	7.2027378	173.1496900	9.0000000	0.5322248	282.5828170	9.0000000	0.7516807
102.0748450	20.0000000	7.1660467	174.1496900	10.0000000	0.5398944	283.5828170	10.0000000	0.7600962
107.3582820	80.0000000	7.1171160	182.0748450	100.0000000	0.6024567	293.5828170	20.0000000	0.8448471
112.0748450	30.0000000	7.0677409	184.1496900	20.0000000	0.6191246	303.5828170	30.0000000	0.9301956
117.3582820	90.0000000	7.0068739	194.1496900	30.0000000	0.7006719	313.5828170	40.0000000	1.0155862
122.0748450	40.0000000	6.9482780	204.1496900	40.0000000	0.7834962	323.5828170	50.0000000	1.1005747
127.3582820	100.0000000	6.8783326	214.1496900	50.0000000	0.8667058	327.3582820	300.0000000	1.1324811
132.0748450	50.0000000	6.8122292	224.1496900	60.0000000	0.9494624	333.5828170	60.0000000	1.1847863
142.0748450	60.0000000	6.6628969	227.3582820	200.0000000	0.9758057	343.5828170	70.0000000	1.2679254
152.0748450	70.0000000	6.5905127	234.1496900	70.0000000	1.0311114	353.5828170	80.0000000	1.3497670
162.0748450	80.0000000	6.3356673	244.1496900	80.0000000	1.1110520	363.5828170	90.0000000	1.4301358
164.1497900	0.0001000	6.3235904	254.1496900	90.0000000	1.1889332	364.1496900	200.0000000	1.4346388
164.1498900	0.0002000	6.3259911	264.1496900	100.0000000	1.2641841	373.5828170	100.0000000	1.5089097
164.1499900	0.0003000	6.3283856	273.5829170	0.0001000	1.3312822	382.0748450	300.0000000	1.5744920
164.1500900	0.0004000	6.3307720	273.5830170	0.0002000	1.3313070	410.3743250	0.0001000	1.7842691
164.1501900	0.0005000	6.3331532	273.5831170	0.0003000	1.3313340	410.3744250	0.0002000	1.7841950
164.1502900	0.0006000	6.3355234	273.5832170	0.0004000	1.3313610	410.3745250	0.0003000	1.7841313
164.1503900	0.0007000	6.3378588	273.5833170	0.0005000	1.3313876	410.3746250	0.0004000	1.7840763
164.1504900	0.0008000	6.3401417	273.5834170	0.0006000	1.3314135	410.3747250	0.0005000	1.7840294
164.1505900	0.0009000	6.3423677	273.5835170	0.0007000	1.3314386	410.3748250	0.0006000	1.7839903
164.1506900	0.0010000	6.3445248	273.5836170	0.0008000	1.3314632	410.3749250	0.0007000	1.7839587
164.1516900	0.0020000	6.3597509	273.5837170	0.0009000	1.3314870	410.3750250	0.0008000	1.7839343
164.1526900	0.0030000	6.3621135	273.5838170	0.0010000	1.3315104	410.3752250	0.0010000	1.7839050
164.1596900	0.0100000	6.3220510	273.5848170	0.0020000	1.3317129	410.3762250	0.0020000	1.7840149
164.1696900	0.0200000	6.3041719	273.5858170	0.0030000	1.3318719	410.3772250	0.0030000	1.7842932
164.1796900	0.0300000	6.2966692	273.5868170	0.0040000	1.3320008	410.3782250	0.0040000	1.7846102
164.1896900	0.0400000	6.2911800	273.5878170	0.0050000	1.3321068	410.3792250	0.0050000	1.7849697
164.1996900	0.0500000	6.2857176	273.5888170	0.0060000	1.3321961	410.3802250	0.0060000	1.7854345
164.2096900	0.0600000	6.2790299	273.5898170	0.0070000	1.3322724	410.3812250	0.0070000	1.7861541
164.2196900	0.0700000	6.2691874	273.5908170	0.0080000	1.3323381	410.3822250	0.0080000	1.7874237
164.2296900	0.0800000	6.2513063	273.5918170	0.0090000	1.3323957	410.3832250	0.0090000	1.7838026
164.2396900	0.0900000	6.2155613	273.5928170	0.0100000	1.3324468	410.3842250	0.0100000	1.7800988
164.2496900	0.1000000	6.3893914	273.6028170	0.0200000	1.3327682	410.3942250	0.0200000	1.7840910
164.3496900	0.2000000	6.3048424	273.6128170	0.0300000	1.3329573	410.4042250	0.0300000	1.7845946
164.4496900	0.3000000	6.2953166	273.6228170	0.0400000	1.3331108	410.4142250	0.0400000	1.7850151
164.5496900	0.4000000	6.2919367	273.6328170	0.0500000	1.3332563	410.4242250	0.0500000	1.7856394
164.6496900	0.5000000	6.2776322	273.6428170	0.0600000	1.3333729	410.4342250	0.0600000	1.7875302
164.7496900	0.6000000	6.2833113	273.6528170	0.0700000	1.3314433	410.4442250	0.0700000	1.7794150
164.8496900	0.7000000	6.2154416	273.6628170	0.0800000	1.3261210	410.4542250	0.0800000	1.7833059
164.9496900	0.8000000	6.3344286	273.6728170	0.0900000	1.3323869	410.4642250	0.0900000	1.7840726
165.0496900	0.9000000	6.3017910	273.6828170	0.1000000	1.3328451	410.4742250	0.1000000	1.7844432
165.1496900	1.0000000	6.2886476	273.7828170	0.2000000	1.3336551	410.5742250	0.2000000	1.7851553
166.1496900	2.0000000	6.2737107	273.8828170	0.3000000	1.3343273	410.6742250	0.3000000	1.7860500
167.1496900	3.0000000	6.2508569	273.9828170	0.4000000	1.3353387	410.7742250	0.4000000	1.7869763
168.1496900	4.0000000	6.2379887	274.0828170	0.5000000	1.3348034	410.8742250	0.5000000	1.7875239
169.1496900	5.0000000	6.2195624	274.1828170	0.6000000	1.3363433	410.9742250	0.6000000	1.7887810
170.1496900	6.0000000	6.2010501	274.2828170	0.7000000	1.3382465	411.0742250	0.7000000	1.7880786
171.1496900	7.0000000	6.1827773	274.3828170	0.8000000	1.3381661	411.1742250	0.8000000	1.7894868
172.0748450	90.0000000	6.1682154	274.4828170	0.9000000	1.3387481	411.2742250	0.9000000	1.7899264
172.1496900	8.0000000	6.1669697	274.5828170	1.0000000	1.3390135	411.3742250	1.0000000	1.7908791
173.1496900	9.0000000	6.1502415	275.5828170	2.0000000	1.3463101	412.3742250	2.0000000	1.7979539
174.1496900	10.0000000	6.1330453	276.5828170	3.0000000	1.3532221	413.3742250	3.0000000	1.8050644
182.0748450	100.0000000	5.9984526	277.5828170	4.0000000	1.3600893	414.3742250	4.0000000	1.8119282
184.1496900	20.0000000	5.9633096	278.5828170	5.0000000	1.3670954	415.3742250	5.0000000	1.8190922
194.1496900	30.0000000	5.7945595	279.5828170	6.0000000	1.3740107	416.3742250	6.0000000	1.8261357
204.1496900	40.0000000	5.6283166	280.5828170	7.0000000	1.3808606	417.3742250	7.0000000	1.8331237
214.1496900	50.0000000	5.4659039	281.5828170	8.0000000	1.3877240	418.3742250	8.0000000	1.8400930
224.1496900	60.0000000	5.3082830	282.0748450	200.0000000	1.3910897	419.3742250	9.0000000	1.8470717

J = 0 → J' = 1			J = 0 → J' = 2			J = 0 → J' = 3		
E_{tot} (cm^{-1})	E_{col} (cm^{-1})	σ (Å)	E_{tot} (cm^{-1})	E_{col} (cm^{-1})	σ (Å)	E_{tot} (cm^{-1})	E_{col} (cm^{-1})	σ (Å)
227.3582820	200.0000000	5.2589308	282.5828170	9.0000000	1.3945559	420.3742250	10.0000000	1.8540132
234.1496900	70.0000000	5.1565021	283.5828170	10.0000000	1.4013434	427.3582820	400.0000000	1.9019781
244.1496900	80.0000000	5.0111542	293.5828170	20.0000000	1.4675060	430.3742250	20.0000000	1.9224008
254.1496900	90.0000000	4.8728200	303.5828170	30.0000000	1.5304792	440.3742250	30.0000000	1.9888399
264.1496900	100.0000000	4.7418448	313.5828170	40.0000000	1.5902474	450.3742250	40.0000000	2.0533197
273.5829170	0.0001000	4.6254320	323.5828170	50.0000000	1.6468611	460.3742250	50.0000000	2.1158383
273.5830170	0.0002000	4.6254325	327.3582820	300.0000000	1.6674343	464.1496900	300.0000000	2.1389330
273.5831170	0.0003000	4.6254327	333.5828170	60.0000000	1.7004017	470.3742250	60.0000000	2.1763948
273.5832170	0.0004000	4.6254329	343.5828170	70.0000000	1.7509627	473.5828170	200.0000000	2.1954104
273.5833170	0.0005000	4.6254330	353.5828170	80.0000000	1.7986826	480.3742250	70.0000000	2.2349930
273.5834170	0.0006000	4.6254332	363.5828170	90.0000000	1.8436867	482.0748450	400.0000000	2.2447652
273.5835170	0.0007000	4.6254323	364.1496900	200.0000000	1.8461600	490.3742250	80.0000000	2.2916491
273.5836170	0.0008000	4.6254321	373.5828170	100.0000000	1.8860988	500.3742250	90.0000000	2.3463797
273.5837170	0.0009000	4.6254312	382.0748450	300.0000000	1.9202737	510.3742250	100.0000000	2.3992075
273.5838170	0.0010000	4.6254310	410.3743250	0.0001000	2.0238617	527.3582820	500.0000000	2.4846480
273.5848170	0.0020000	4.6254222	410.3744250	0.0002000	2.0238147	564.1496900	400.0000000	2.6522559
273.5858170	0.0030000	4.6254082	410.3745250	0.0003000	2.0237682	573.5828170	300.0000000	2.6913789
273.5868170	0.0040000	4.6253940	410.3746250	0.0004000	2.0237223	582.0748450	500.0000000	2.7255706
273.5878170	0.0050000	4.6253789	410.3747250	0.0005000	2.0236779	610.3742250	200.0000000	2.8318563
273.5888170	0.0060000	4.6253630	410.3748250	0.0006000	2.0236347	627.3582820	600.0000000	2.8905420
273.5898170	0.0070000	4.6253470	410.3749250	0.0007000	2.0235936	664.1496900	500.0000000	3.0067165
273.5908170	0.0080000	4.6253308	410.3750250	0.0008000	2.0235547	673.5828170	400.0000000	3.0344732
273.5918170	0.0090000	4.6253148	410.3752250	0.0010000	2.0234846	682.0748450	600.0000000	3.0588690
273.5928170	0.0100000	4.6252997	410.3762250	0.0020000	2.0232839	710.3742250	300.0000000	3.1367602
273.6028170	0.0200000	4.6251539	410.3772250	0.0030000	2.0232565	727.3582820	700.0000000	3.1815094
273.6128170	0.0300000	4.6250190	410.3782250	0.0040000	2.0233174	764.1496900	600.0000000	3.2750627
273.6228170	0.0400000	4.6248906	410.3792250	0.0050000	2.0234436	773.5828170	500.0000000	3.2985229
273.6328170	0.0500000	4.6247613	410.3802250	0.0060000	2.0236626	782.0748450	700.0000000	3.3195286
273.6428170	0.0600000	4.6245936	410.3812250	0.0070000	2.0240936	810.3742250	400.0000000	3.3888985
273.6528170	0.0700000	4.6236565	410.3822250	0.0080000	2.0252313	827.3582820	800.0000000	3.4301843
273.6628170	0.0800000	4.6203120	410.3832250	0.0090000	2.0266415	864.1496900	700.0000000	3.5188912
273.6728170	0.0900000	4.6237049	410.3842250	0.0100000	2.0223665	873.5828170	600.0000000	3.5414649
273.6828170	0.1000000	4.6238168	410.3942250	0.0200000	2.0229257	882.0748450	800.0000000	3.5617181
273.7828170	0.2000000	4.6227454	410.4042250	0.0300000	2.0231355	910.3742250	500.0000000	3.6286806
273.8828170	0.3000000	4.6215561	410.4142250	0.0400000	2.0233262	927.3582820	900.0000000	3.6684256
273.9828170	0.4000000	4.6204178	410.4242250	0.0500000	2.0236293	964.1496900	800.0000000	3.7532555
274.0828170	0.5000000	4.6192988	410.4342250	0.0600000	2.0247365	973.5828170	700.0000000	3.7747246
274.1828170	0.6000000	4.6181668	410.4442250	0.0700000	2.0213412	982.0748450	900.0000000	3.7939658
274.2828170	0.7000000	4.6147901	410.4542250	0.0800000	2.0226382	1010.3742300	600.0000000	3.8574698
274.3828170	0.8000000	4.6154340	410.4642250	0.0900000	2.0229691	1064.1496900	900.0000000	3.9762365
274.4828170	0.9000000	4.6142951	410.4742250	0.1000000	2.0231802	1073.5828200	800.0000000	3.9968780
274.5828170	1.0000000	4.6130045	410.5742250	0.2000000	2.0235017	1110.3742300	700.0000000	4.0768752
275.5828170	2.0000000	4.6012143	410.6742250	0.3000000	2.0239433	1173.5828200	900.0000000	4.2111023
276.5828170	3.0000000	4.5893908	410.7742250	0.4000000	2.0242995	1210.3742300	800.0000000	4.2854395
277.5828170	4.0000000	4.5775064	410.8742250	0.5000000	2.0245885	1310.3742300	900.0000000	4.4599273
278.5828170	5.0000000	4.5659418	410.9742250	0.6000000	2.0251584			
279.5828170	6.0000000	4.5543671	411.0742250	0.7000000	2.0249215			
280.5828170	7.0000000	4.5428302	411.1742250	0.8000000	2.0255401			
281.5828170	8.0000000	4.5314033	411.2742250	0.9000000	2.0258111			
282.0748450	200.0000000	4.5258183	411.3742250	1.0000000	2.0262625			
282.5828170	9.0000000	4.5200695	412.3742250	2.0000000	2.0295258			
283.5828170	10.0000000	4.5087870	413.3742250	3.0000000	2.0328660			
293.5828170	20.0000000	4.4003156	414.3742250	4.0000000	2.0361244			
303.5828170	30.0000000	4.2996174	415.3742250	5.0000000	2.0394074			
313.5828170	40.0000000	4.2066130	416.3742250	6.0000000	2.0426670			
323.5828170	50.0000000	4.1212291	417.3742250	7.0000000	2.0459073			
327.3582820	300.0000000	4.0909561	418.3742250	8.0000000	2.0491180			
333.5828170	60.0000000	4.0433641	419.3742250	9.0000000	2.0523208			
343.5828170	70.0000000	3.9728712	420.3742250	10.0000000	2.0555052			
353.5828170	80.0000000	3.9096395	427.3582820	400.0000000	2.0772938			
363.5828170	90.0000000	3.8535116	430.3742250	20.0000000	2.0864688			
364.1496900	200.0000000	3.8505424	440.3742250	30.0000000	2.1159428			
373.5828170	100.0000000	3.8043272	450.3742250	40.0000000	2.1440792			
382.0748450	300.0000000	3.7679106	460.3742250	50.0000000	2.1710264			
410.3743250	0.0001000	3.6800432	464.1496900	300.0000000	2.1809216			
410.3744250	0.0002000	3.6800495	470.3742250	60.0000000	2.1969289			
410.3745250	0.0003000	3.6800600	473.5828170	200.0000000	2.2050377			
410.3746250	0.0004000	3.6800737	480.3742250	70.0000000	2.2219190			
410.3747250	0.0005000	3.6800905	482.0748450	400.0000000	2.2260885			
410.3748250	0.0006000	3.6801094	490.3742250	80.0000000	2.2461378			
410.3749250	0.0007000	3.6801303	500.3742250	90.0000000	2.2697074			
410.3750250	0.0008000	3.6801523	510.3742250	100.0000000	2.2927507			
410.3752250	0.0010000	3.6801995	527.3582820	500.0000000	2.3310035			
410.3762250	0.0020000	3.6804235	564.1496900	400.0000000	2.4118781			
410.3772250	0.0030000	3.6805750	573.5828170	300.0000000	2.4331234			
410.3782250	0.0040000	3.6806668	582.0748450	500.0000000	2.4520067			
410.3792250	0.0050000	3.6807191	610.3742250	200.0000000	2.5164419			
410.3802250	0.0060000	3.6807375	627.3582820	600.0000000	2.5564699			

J = 0 → J' = 1			J = 0 → J' = 2			J = 0 → J' = 3		
E_{tot} (cm^{-1})	E_{col} (cm^{-1})	σ (Å)	E_{tot} (cm^{-1})	E_{col} (cm^{-1})	σ (Å)	E_{tot} (cm^{-1})	E_{col} (cm^{-1})	σ (Å)
410.3812250	0.0070000	3.6806815	664.1496900	500.0000000	2.6473791			
410.3822250	0.0080000	3.6802299	673.5828170	400.0000000	2.6716322			
410.3832250	0.0090000	3.6770990	682.0748450	600.0000000	2.6937757			
410.3842250	0.0100000	3.6793069	710.3742250	300.0000000	2.7694018			
410.3942250	0.0200000	3.6807197	727.3582820	700.0000000	2.8158152			
410.4042250	0.0300000	3.6807354	764.1496900	600.0000000	2.9172806			
410.4142250	0.0400000	3.6807199	773.5828170	500.0000000	2.9431516			
410.4242250	0.0500000	3.6806830	782.0748450	700.0000000	2.9662920			
410.4342250	0.0600000	3.6804655	810.3742250	400.0000000	3.0417310			
410.4442250	0.0700000	3.6803927	827.3582820	800.0000000	3.0852573			
410.4542250	0.0800000	3.6806279	864.1496900	700.0000000	3.1732818			
410.4642250	0.0900000	3.6806069	873.5828170	600.0000000	3.1942192			
410.4742250	0.1000000	3.6805473	882.0748450	800.0000000	3.2124322			
410.5742250	0.2000000	3.6803227	910.3742250	500.0000000	3.2685299			
410.6742250	0.3000000	3.6800915	927.3582820	900.0000000	3.2986397			
410.7742250	0.4000000	3.6799528	964.1496900	800.0000000	3.3544816			
410.8742250	0.5000000	3.6796756	973.5828170	700.0000000	3.3667707			
410.9742250	0.6000000	3.6794870	982.0748450	900.0000000	3.3771559			
411.0742250	0.7000000	3.6792244	1010.3742300	600.0000000	3.4074397			
411.1742250	0.8000000	3.6790383	1064.1496900	900.0000000	3.4503543			
411.2742250	0.9000000	3.6787451	1073.5828200	800.0000000	3.4565452			
411.3742250	1.0000000	3.6785545	1110.3742300	700.0000000	3.4791892			
412.3742250	2.0000000	3.6765008	1173.5828200	900.0000000	3.5218161			
413.3742250	3.0000000	3.6744636	1210.3742300	800.0000000	3.5546805			
414.3742250	4.0000000	3.6724698	1310.3742300	900.0000000	3.6956852			
415.3742250	5.0000000	3.6705916						
416.3742250	6.0000000	3.6687558						
417.3742250	7.0000000	3.6669732						
418.3742250	8.0000000	3.6652544						
419.3742250	9.0000000	3.6636066						
420.3742250	10.0000000	3.6620065						
427.3582820	400.0000000	3.6525679						
430.3742250	20.0000000	3.6493925						
440.3742250	30.0000000	3.6427249						
450.3742250	40.0000000	3.6418560						
460.3742250	50.0000000	3.6466552						
464.1496900	300.0000000	3.6499109						
470.3742250	60.0000000	3.6569762						
473.5828170	200.0000000	3.6614363						
480.3742250	70.0000000	3.6726841						
482.0748450	400.0000000	3.6758822						
490.3742250	80.0000000	3.6936587						
500.3742250	90.0000000	3.7197504						
510.3742250	100.0000000	3.7508414						
527.3582820	500.0000000	3.8146952						
564.1496900	400.0000000	3.9984455						
573.5828170	300.0000000	4.0537359						
582.0748450	500.0000000	4.1072753						
610.3742250	200.0000000	4.3040569						
627.3582820	600.0000000	4.4344617						
664.1496900	500.0000000	4.7417587						
673.5828170	400.0000000	4.8247049						
682.0748450	600.0000000	4.9003991						
710.3742250	300.0000000	5.1569883						
727.3582820	700.0000000	5.3118820						
764.1496900	600.0000000	5.6408354						
773.5828170	500.0000000	5.7223276						
782.0748450	700.0000000	5.7942999						
810.3742250	400.0000000	6.0227560						
827.3582820	800.0000000	6.1500368						
864.1496900	700.0000000	6.3962212						
873.5828170	600.0000000	6.4523180						
882.0748450	800.0000000	6.5002427						
910.3742250	500.0000000	6.6419383						
927.3582820	900.0000000	6.7133904						
964.1496900	800.0000000	6.8326052						
973.5828170	700.0000000	6.8553000						
982.0748450	900.0000000	6.8729680						
1010.3742300	600.0000000	6.9132911						
1064.1496900	900.0000000	6.9135373						
1073.5828200	800.0000000	6.9038482						
1110.3742300	700.0000000	6.8410767						
1173.5828200	900.0000000	6.6558138						
1210.3742300	800.0000000	6.5157542						
1310.3742300	900.0000000	6.0868777						

Inelastic collisions cross-sections					
J = 0 → J’ = 4			J = 0 → J’ = 5		
E_{tot} (cm⁻¹)	E_{col} (cm⁻¹)	σ (Å)	E_{tot} (cm⁻¹)	E_{col} (cm⁻¹)	σ (Å)
273.5829170	0.0001000	0.0000655	410.3743250	0.0001000	0.0000222
273.5830170	0.0002000	0.0000641	410.3744250	0.0002000	0.0000227
273.5831170	0.0003000	0.0000608	410.3745250	0.0003000	0.0000222
273.5832170	0.0004000	0.0000576	410.3746250	0.0004000	0.0000214
273.5833170	0.0005000	0.0000548	410.3747250	0.0005000	0.0000205
273.5834170	0.0006000	0.0000523	410.3748250	0.0006000	0.0000198
273.5835170	0.0007000	0.0000501	410.3749250	0.0007000	0.0000190
273.5836170	0.0008000	0.0000482	410.3750250	0.0008000	0.0000184
273.5837170	0.0009000	0.0000465	410.3752250	0.0010000	0.0000172
273.5838170	0.0010000	0.0000449	410.3762250	0.0020000	0.0000133
273.5848170	0.0020000	0.0000354	410.3772250	0.0030000	0.0000115
273.5858170	0.0030000	0.0000320	410.3782250	0.0040000	0.0000108
273.5868170	0.0040000	0.0000315	410.3792250	0.0050000	0.0000108
273.5878170	0.0050000	0.0000328	410.3802250	0.0060000	0.0000112
273.5888170	0.0060000	0.0000351	410.3812250	0.0070000	0.0000120
273.5898170	0.0070000	0.0000382	410.3822250	0.0080000	0.0000129
273.5908170	0.0080000	0.0000419	410.3832250	0.0090000	0.0000140
273.5918170	0.0090000	0.0000458	410.3842250	0.0100000	0.0000151
273.5928170	0.0100000	0.0000500	410.3942250	0.0200000	0.0000268
273.6028170	0.0200000	0.0000918	410.4042250	0.0300000	0.0000368
273.6128170	0.0300000	0.0001262	410.4142250	0.0400000	0.0000454
273.6228170	0.0400000	0.0001564	410.4242250	0.0500000	0.0000542
273.6328170	0.0500000	0.0001943	410.4342250	0.0600000	0.0000650
273.6428170	0.0600000	0.0003049	410.4442250	0.0700000	0.0000810
273.6528170	0.0700000	0.0024079	410.4542250	0.0800000	0.0001054
273.6628170	0.0800000	0.0013042	410.4642250	0.0900000	0.0001548
273.6728170	0.0900000	0.0007240	410.4742250	0.1000000	0.0002642
273.6828170	0.1000000	0.0007757	410.5742250	0.2000000	0.0002585
273.7828170	0.2000000	0.0010376	410.6742250	0.3000000	0.0003296
273.8828170	0.3000000	0.0011125	410.7742250	0.4000000	0.0001915
273.9828170	0.4000000	0.0007415	410.8742250	0.5000000	0.0002249
274.0828170	0.5000000	0.0010179	410.9742250	0.6000000	0.0002400
274.1828170	0.6000000	0.0009042	411.0742250	0.7000000	0.0002555
274.2828170	0.7000000	0.0009572	411.1742250	0.8000000	0.0002791
274.3828170	0.8000000	0.0010309	411.2742250	0.9000000	0.0003863
274.4828170	0.9000000	0.0012161	411.3742250	1.0000000	0.0004418
274.5828170	1.0000000	0.0020119	412.3742250	2.0000000	0.0003739
275.5828170	2.0000000	0.0013686	413.3742250	3.0000000	0.0004612
276.5828170	3.0000000	0.0016783	414.3742250	4.0000000	0.0006075
277.5828170	4.0000000	0.0021243	415.3742250	5.0000000	0.0005589
278.5828170	5.0000000	0.0020087	416.3742250	6.0000000	0.0005930
279.5828170	6.0000000	0.0021266	417.3742250	7.0000000	0.0006515
280.5828170	7.0000000	0.0023161	418.3742250	8.0000000	0.0006874
281.5828170	8.0000000	0.0024425	419.3742250	9.0000000	0.0007200
282.0748450	200.0000000	0.0024972	420.3742250	10.0000000	0.0007593
282.5828170	9.0000000	0.0025593	427.3582820	400.0000000	0.0010820
283.5828170	10.0000000	0.0026953	430.3742250	20.0000000	0.0012446
293.5828170	20.0000000	0.0043445	440.3742250	30.0000000	0.0019017
303.5828170	30.0000000	0.0065215	450.3742250	40.0000000	0.0027622
313.5828170	40.0000000	0.0092974	460.3742250	50.0000000	0.0038541
323.5828170	50.0000000	0.0127328	464.1496900	300.0000000	0.0043320
327.3582820	300.0000000	0.0142128	470.3742250	60.0000000	0.0052040
333.5828170	60.0000000	0.0168813	473.5828170	200.0000000	0.0056960
343.5828170	70.0000000	0.0217872	480.3742250	70.0000000	0.0068369
353.5828170	80.0000000	0.0274824	482.0748450	400.0000000	0.0071445
363.5828170	90.0000000	0.0339854	490.3742250	80.0000000	0.0087756
364.1496900	200.0000000	0.0343782	500.3742250	90.0000000	0.0110402
373.5828170	100.0000000	0.0412984	510.3742250	100.0000000	0.0136477
382.0748450	300.0000000	0.0481334	527.3582820	500.0000000	0.0188977
410.3743250	0.0001000	0.0748817	564.1496900	400.0000000	0.0334194
410.3744250	0.0002000	0.0749280	573.5828170	300.0000000	0.0386216
410.3745250	0.0003000	0.0749679	582.0748450	500.0000000	0.0429573
410.3746250	0.0004000	0.0750019	610.3742250	200.0000000	0.0593515
410.3747250	0.0005000	0.0750302	627.3582820	600.0000000	0.0702611
410.3748250	0.0006000	0.0750531	664.1496900	500.0000000	0.0960108
410.3749250	0.0007000	0.0750708	673.5828170	400.0000000	0.1029682
410.3750250	0.0008000	0.0750836	682.0748450	600.0000000	0.1093236
410.3752250	0.0010000	0.0750962	710.3742250	300.0000000	0.1309750
410.3762250	0.0020000	0.0749952	727.3582820	700.0000000	0.1442064
410.3772250	0.0030000	0.0747977	764.1496900	600.0000000	0.1731908
410.3782250	0.0040000	0.0745850	773.5828170	500.0000000	0.1806645
410.3792250	0.0050000	0.0743510	782.0748450	700.0000000	0.1874079
410.3802250	0.0060000	0.0740546	810.3742250	400.0000000	0.2099821
410.3812250	0.0070000	0.0736044	827.3582820	800.0000000	0.2236273
410.3822250	0.0080000	0.0728488	864.1496900	700.0000000	0.2535345
410.3832250	0.0090000	0.0755410	873.5828170	600.0000000	0.2612919
410.3842250	0.0100000	0.0776554	882.0748450	800.0000000	0.2683079

J = 0 → J' = 4			J = 0 → J' = 5		
E_{tot} (cm⁻¹)	E_{col} (cm⁻¹)	σ (Å)	E_{tot} (cm⁻¹)	E_{col} (cm⁻¹)	σ (Å)
410.3942250	0.0200000	0.0749789	910.3742250	500.0000000	0.2918970
410.4042250	0.0300000	0.0747056	927.3582820	900.0000000	0.3061786
410.4142250	0.0400000	0.0744948	964.1496900	800.0000000	0.3372232
410.4242250	0.0500000	0.0741669	973.5828170	700.0000000	0.3451554
410.4342250	0.0600000	0.0730861	982.0748450	900.0000000	0.3522565
410.4442250	0.0700000	0.0779047	1010.3742300	600.0000000	0.3756689
410.4542250	0.0800000	0.0756506	1064.1496900	900.0000000	0.4179249
410.4642250	0.0900000	0.0752097	1073.5828200	800.0000000	0.4249346
410.4742250	0.1000000	0.0749558	1110.3742300	700.0000000	0.4509487
410.5742250	0.2000000	0.0750762	1173.5828200	900.0000000	0.4909698
410.6742250	0.3000000	0.0750124	1210.3742300	800.0000000	0.5122813
410.7742250	0.4000000	0.0750914	1310.3742300	900.0000000	0.5689555
410.8742250	0.5000000	0.0752947			
410.9742250	0.6000000	0.0750532			
411.0742250	0.7000000	0.0759859			
411.1742250	0.8000000	0.0756573			
411.2742250	0.9000000	0.0758612			
411.3742250	1.0000000	0.0757580			
412.3742250	2.0000000	0.0769022			
413.3742250	3.0000000	0.0778924			
414.3742250	4.0000000	0.0789725			
415.3742250	5.0000000	0.0800468			
416.3742250	6.0000000	0.0811116			
417.3742250	7.0000000	0.0821836			
418.3742250	8.0000000	0.0832824			
419.3742250	9.0000000	0.0843816			
420.3742250	10.0000000	0.0854834			
427.3582820	400.0000000	0.0933622			
430.3742250	20.0000000	0.0968555			
440.3742250	30.0000000	0.1088084			
450.3742250	40.0000000	0.1212927			
460.3742250	50.0000000	0.1342610			
464.1496900	300.0000000	0.1392730			
470.3742250	60.0000000	0.1476650			
473.5828170	200.0000000	0.1520499			
480.3742250	70.0000000	0.1614566			
482.0748450	400.0000000	0.1638372			
490.3742250	80.0000000	0.1755905			
500.3742250	90.0000000	0.1900225			
510.3742250	100.0000000	0.2047096			
527.3582820	500.0000000	0.2301256			
564.1496900	400.0000000	0.2865236			
573.5828170	300.0000000	0.3010024			
582.0748450	500.0000000	0.3140758			
610.3742250	200.0000000	0.3574180			
627.3582820	600.0000000	0.3830265			
664.1496900	500.0000000	0.4367788			
673.5828170	400.0000000	0.4500789			
682.0748450	600.0000000	0.4618565			
710.3742250	300.0000000	0.4996350			
727.3582820	700.0000000	0.5211265			
764.1496900	600.0000000	0.5643598			
773.5828170	500.0000000	0.5747033			
782.0748450	700.0000000	0.5837324			
810.3742250	400.0000000	0.6119655			
827.3582820	800.0000000	0.6275434			
864.1496900	700.0000000	0.6578469			
873.5828170	600.0000000	0.6648800			
882.0748450	800.0000000	0.6709630			
910.3742250	500.0000000	0.6896022			
927.3582820	900.0000000	0.6996506			
964.1496900	800.0000000	0.7188150			
973.5828170	700.0000000	0.7232195			
982.0748450	900.0000000	0.7270566			
1010.3742300	600.0000000	0.7387956			
1064.1496900	900.0000000	0.7583648			
1073.5828200	800.0000000	0.7615740			
1110.3742300	700.0000000	0.7738677			
1173.5828200	900.0000000	0.7952484			
1210.3742300	800.0000000	0.8081288			
1310.3742300	900.0000000	0.8420834			

Inelastic collisions cross-sections								
J = 1 → J' = 0			J = 1 → J' = 2			J = 1 → J' = 3		
E_{tot} (cm^{-1})	E_{col} (cm^{-1})	σ ($Å^2$)	E_{tot} (cm^{-1})	E_{col} (cm^{-1})	σ ($Å^2$)	E_{tot} (cm^{-1})	E_{col} (cm^{-1})	σ ($Å^2$)
27.358382	0.000100	1142.182590	82.074945	0.000100	0.001301	164.149790	0.000100	0.000957
27.358482	0.000200	464.157549	82.075045	0.000200	0.001309	164.149890	0.000200	0.001038
27.358582	0.000300	288.456066	82.075145	0.000300	0.001485	164.149990	0.000300	0.001194
27.358682	0.000400	214.550799	82.075245	0.000400	0.001770	164.150090	0.000400	0.001420
27.358782	0.000500	176.355485	82.075345	0.000500	0.002139	164.150190	0.000500	0.001708
27.358882	0.000600	154.334896	82.075445	0.000600	0.002580	164.150290	0.000600	0.002050
27.358982	0.000700	140.734397	82.075545	0.000700	0.003084	164.150390	0.000700	0.002441
27.359082	0.000800	131.967034	82.075645	0.000800	0.003645	164.150490	0.000800	0.002875
27.359182	0.000900	126.161090	82.075745	0.000900	0.004258	164.150590	0.000900	0.003345
27.359282	0.001000	122.303155	82.075845	0.001000	0.004919	164.150690	0.001000	0.003846
27.360282	0.002000	116.511677	82.076845	0.002000	0.013553	164.151690	0.002000	0.009351
27.361282	0.003000	118.869479	82.077845	0.003000	0.024614	164.152690	0.003000	0.013030
27.362282	0.004000	118.952571	82.078845	0.004000	0.037250	164.159690	0.010000	0.008950
27.363282	0.005000	117.617567	82.079845	0.005000	0.051367	164.169690	0.020000	0.006187
27.364282	0.006000	115.844930	82.080845	0.006000	0.067230	164.179690	0.030000	0.005985
27.365282	0.007000	114.354090	82.081845	0.007000	0.085307	164.189690	0.040000	0.006471
27.366282	0.008000	113.445647	82.082845	0.008000	0.106108	164.199690	0.050000	0.015108
27.367282	0.009000	113.259457	82.083845	0.009000	0.130223	164.209690	0.060000	0.008095
27.368282	0.010000	113.866936	82.084845	0.010000	0.158081	164.219690	0.070000	0.009131
27.378282	0.020000	122.502685	82.094845	0.020000	0.371986	164.229690	0.080000	0.011299
27.388282	0.030000	111.222957	82.104845	0.030000	0.172619	164.239690	0.090000	0.015401
27.398282	0.040000	101.734611	82.114845	0.040000	0.110437	164.249690	0.100000	0.022746
27.408282	0.050000	95.240318	82.124845	0.050000	0.094256	164.349690	0.200000	0.035669
27.418282	0.060000	91.064349	82.134845	0.060000	0.096952	164.449690	0.300000	0.027014
27.428282	0.070000	88.842423	82.144845	0.070000	0.123560	164.549690	0.400000	0.020363
27.438282	0.080000	88.445788	82.154845	0.080000	0.232684	164.649690	0.500000	0.020102
27.448282	0.090000	89.531765	82.164845	0.090000	0.620268	164.749690	0.600000	0.035896
27.458282	0.100000	91.217357	82.174845	0.100000	0.549343	164.849690	0.700000	0.029334
27.558282	0.200000	44.589943	82.274845	0.200000	0.256763	164.949690	0.800000	0.030592
27.658282	0.300000	30.725306	82.374845	0.300000	0.263010	165.049690	0.900000	0.037128
27.758282	0.400000	32.161796	82.474845	0.400000	0.178568	165.149690	1.000000	0.050296
27.858282	0.500000	30.700709	82.574845	0.500000	0.193803	166.149690	2.000000	0.039569
27.958282	0.600000	30.253105	82.674845	0.600000	0.230366	167.149690	3.000000	0.051447
28.058282	0.700000	80.115431	82.774845	0.700000	0.488937	168.149690	4.000000	0.081539
28.158282	0.800000	42.148491	82.874845	0.800000	0.286649	169.149690	5.000000	0.063230
28.258282	0.900000	30.548784	82.974845	0.900000	0.319418	170.149690	6.000000	0.065925
28.358282	1.000000	27.564563	83.074845	1.000000	0.531232	171.149690	7.000000	0.073416
29.358282	2.000000	15.765718	84.074845	2.000000	0.344561	172.074845	90.000000	0.076344
30.358282	3.000000	15.130279	85.074845	3.000000	0.419483	172.149690	8.000000	0.076432
31.358282	4.000000	33.965300	86.074845	4.000000	0.659892	173.149690	9.000000	0.078312
32.358282	5.000000	11.734510	87.074845	5.000000	0.492577	174.149690	10.000000	0.081411
33.358282	6.000000	10.300561	87.358282	60.000000	0.489809	182.074845	100.000000	0.109045
34.358282	7.000000	11.117557	88.074845	6.000000	0.498686	184.149690	20.000000	0.116581
35.358282	8.000000	9.980505	89.074845	7.000000	0.539589	194.149690	30.000000	0.155191
36.358282	9.000000	8.604219	90.074845	8.000000	0.552348	204.149690	40.000000	0.197127
37.358282	10.000000	7.982324	91.074845	9.000000	0.555522	214.149690	50.000000	0.241786
47.358282	20.000000	5.414586	92.074845	10.000000	0.566976	224.149690	60.000000	0.288641
57.358282	30.000000	4.517122	97.358282	70.000000	0.636299	227.358282	200.000000	0.304069
67.358282	40.000000	4.070654	102.074845	20.000000	0.697665	234.149690	70.000000	0.337219
77.358282	50.000000	3.759397	107.358282	80.000000	0.767475	244.149690	80.000000	0.387056
82.074945	0.000100	3.600308	112.074845	30.000000	0.830201	254.149690	90.000000	0.437575
82.075045	0.000200	3.600265	117.358282	90.000000	0.900609	264.149690	100.000000	0.489237
82.075145	0.000300	3.600205	122.074845	40.000000	0.963327	273.582917	0.000100	0.537998
82.075245	0.000400	3.600138	127.358282	100.000000	1.033296	273.583017	0.000200	0.537981
82.075345	0.000500	3.600072	132.074845	50.000000	1.095272	273.583117	0.000300	0.537961
82.075445	0.000600	3.600006	142.074845	60.000000	1.224003	273.583217	0.000400	0.537937
82.075545	0.000700	3.599943	152.074845	70.000000	1.693849	273.583317	0.000500	0.537912
82.075645	0.000800	3.599881	162.074845	80.000000	1.496034	273.583417	0.000600	0.537886
82.075745	0.000900	3.599818	164.149790	0.000100	1.549310	273.583517	0.000700	0.537860
82.075845	0.001000	3.599758	164.149890	0.000200	1.553568	273.583617	0.000800	0.537833
82.076845	0.002000	3.599312	164.149990	0.000300	1.557815	273.583717	0.000900	0.537805
82.077845	0.003000	3.599213	164.150090	0.000400	1.562033	273.583817	0.001000	0.537777
82.078845	0.004000	3.599557	164.150190	0.000500	1.566234	273.584817	0.002000	0.537533
82.079845	0.005000	3.600510	164.150290	0.000600	1.570400	273.585817	0.003000	0.538510
82.080845	0.006000	3.602283	164.150390	0.000700	1.574494	273.586817	0.004000	0.545520
82.081845	0.007000	3.605133	164.150490	0.000800	1.578485	273.587817	0.005000	0.539508
82.082845	0.008000	3.609436	164.150590	0.000900	1.582362	273.588817	0.006000	0.538417
82.083845	0.009000	3.615604	164.150690	0.001000	1.586095	273.589817	0.007000	0.537960
82.084845	0.010000	3.624191	164.151690	0.002000	1.611760	273.590817	0.008000	0.537684
82.094845	0.020000	3.801010	164.152690	0.003000	1.614429	273.591817	0.009000	0.537485
82.104845	0.030000	3.742994	164.159690	0.010000	1.541859	273.592817	0.010000	0.537328
82.114845	0.040000	3.700548	164.169690	0.020000	1.512437	273.602817	0.020000	0.536451
82.124845	0.050000	3.680392	164.179690	0.030000	1.501849	273.612817	0.030000	0.535878
82.134845	0.060000	3.668843	164.189690	0.040000	1.496227	273.622817	0.040000	0.535274
82.144845	0.070000	3.661064	164.199690	0.050000	1.528366	273.632817	0.050000	0.534403
82.154845	0.080000	3.655221	164.209690	0.060000	1.486016	273.642817	0.060000	0.532655
82.164845	0.090000	3.650382	164.219690	0.070000	1.478757	273.652817	0.070000	0.525193

J = 1 → J’ = 0			J = 1 → J’ = 2			J = 1 → J’ = 3		
E_{tot} (cm^{-1})	E_{col} (cm^{-1})	σ (Å^2)	E_{tot} (cm^{-1})	E_{col} (cm^{-1})	σ (Å^2)	E_{tot} (cm^{-1})	E_{col} (cm^{-1})	σ (Å^2)
82.174845	0.100000	3.646520	164.229690	0.080000	1.473937	273.662817	0.080000	0.565853
82.274845	0.200000	3.633379	164.239690	0.090000	1.526890	273.672817	0.090000	0.542970
82.374845	0.300000	3.622181	164.249690	0.100000	1.759665	273.682817	0.100000	0.539734
82.474845	0.400000	3.625860	164.349690	0.200000	1.510529	273.782817	0.200000	0.537384
82.574845	0.500000	3.611762	164.449690	0.300000	1.502016	273.882817	0.300000	0.537440
82.674845	0.600000	3.582500	164.549690	0.400000	1.502155	273.982817	0.400000	0.537978
82.774845	0.700000	3.771316	164.649690	0.500000	1.489005	274.082817	0.500000	0.540481
82.874845	0.800000	3.530634	164.749690	0.600000	1.511747	274.182817	0.600000	0.538219
82.974845	0.900000	3.621041	164.849690	0.700000	1.483096	274.282817	0.700000	0.552982
83.074845	1.000000	3.613431	164.949690	0.800000	1.547936	274.382817	0.800000	0.541133
84.074845	2.000000	3.601794	165.049690	0.900000	1.519714	274.482817	0.900000	0.540555
85.074845	3.000000	3.568710	165.149690	1.000000	1.512179	274.582817	1.000000	0.539584
86.074845	4.000000	3.543942	166.149690	2.000000	1.527741	275.582817	2.000000	0.546232
87.074845	5.000000	3.528620	167.149690	3.000000	1.534859	276.582817	3.000000	0.550744
87.358282	60.000000	3.522690	168.149690	4.000000	1.551374	277.582817	4.000000	0.555707
88.074845	6.000000	3.507856	169.149690	5.000000	1.561363	278.582817	5.000000	0.560834
89.074845	7.000000	3.487244	170.149690	6.000000	1.572543	279.582817	6.000000	0.565775
90.074845	8.000000	3.468734	171.149690	7.000000	1.583957	280.582817	7.000000	0.570677
91.074845	9.000000	3.450059	172.074845	90.000000	1.595982	281.582817	8.000000	0.575726
92.074845	10.000000	3.431564	172.149690	8.000000	1.596999	282.074845	200.000000	0.578202
97.358282	70.000000	3.339268	173.149690	9.000000	1.609261	282.582817	9.000000	0.580741
102.074845	20.000000	3.263324	174.149690	10.000000	1.621310	283.582817	10.000000	0.585729
107.358282	80.000000	3.183672	182.074845	100.000000	1.717526	293.582817	20.000000	0.635224
112.074845	30.000000	3.116730	184.149690	20.000000	1.742525	303.582817	30.000000	0.683863
117.358282	90.000000	3.045610	194.149690	30.000000	1.862059	313.582817	40.000000	0.731470
122.074845	40.000000	2.985081	204.149690	40.000000	1.979879	323.582817	50.000000	0.777918
127.358282	100.000000	2.920042	214.149690	50.000000	2.095736	327.358282	300.000000	0.795131
132.074845	50.000000	2.863998	224.149690	60.000000	2.209602	333.582817	60.000000	0.823105
142.074845	60.000000	2.750635	227.358282	200.000000	2.245750	343.582817	70.000000	0.866973
152.074845	70.000000	2.678744	234.149690	70.000000	2.321553	353.582817	80.000000	0.909484
162.074845	80.000000	2.540772	244.149690	80.000000	2.431541	363.582817	90.000000	0.950636
164.149790	0.000100	2.529436	254.149690	90.000000	2.539680	364.149690	200.000000	0.952928
164.149890	0.000200	2.530396	264.149690	100.000000	2.645762	373.582817	100.000000	0.990446
164.149990	0.000300	2.531353	273.582917	0.000100	2.744444	382.074845	300.000000	1.023199
164.150090	0.000400	2.532308	273.583017	0.000200	2.744533	410.374325	0.000100	1.125180
164.150190	0.000500	2.533260	273.583117	0.000300	2.744628	410.374425	0.000200	1.125145
164.150290	0.000600	2.534207	273.583217	0.000400	2.744726	410.374525	0.000300	1.125120
164.150390	0.000700	2.535141	273.583317	0.000500	2.744825	410.374625	0.000400	1.125102
164.150490	0.000800	2.536054	273.583417	0.000600	2.744928	410.374725	0.000500	1.125092
164.150590	0.000900	2.536944	273.583517	0.000700	2.745033	410.374825	0.000600	1.125088
164.150690	0.001000	2.537807	273.583617	0.000800	2.745143	410.374925	0.000700	1.125090
164.151690	0.002000	2.543894	273.583717	0.000900	2.745256	410.375025	0.000800	1.125098
164.152690	0.003000	2.544836	273.583817	0.001000	2.745374	410.375225	0.001000	1.125126
164.159690	0.010000	2.528790	273.584817	0.002000	2.747036	410.376225	0.002000	1.125395
164.169690	0.020000	2.521607	273.585817	0.003000	2.751183	410.377225	0.003000	1.125691
164.179690	0.030000	2.518576	273.586817	0.004000	2.734387	410.378225	0.004000	1.125953
164.189690	0.040000	2.516349	273.587817	0.005000	2.738897	410.379225	0.005000	1.126203
164.199690	0.050000	2.514134	273.588817	0.006000	2.740809	410.380225	0.006000	1.126482
164.209690	0.060000	2.511428	273.589817	0.007000	2.741688	410.381225	0.007000	1.126840
164.219690	0.070000	2.507461	273.590817	0.008000	2.742194	410.382225	0.008000	1.127172
164.229690	0.080000	2.500279	273.591817	0.009000	2.742526	410.383225	0.009000	1.122532
164.239690	0.090000	2.485952	273.592817	0.010000	2.742764	410.384225	0.010000	1.122317
164.249690	0.100000	2.555445	273.602817	0.020000	2.743633	410.394225	0.020000	1.125741
164.349690	0.200000	2.521323	273.612817	0.030000	2.743881	410.404225	0.030000	1.126043
164.449690	0.300000	2.517208	273.622817	0.040000	2.743987	410.414225	0.040000	1.126266
164.549690	0.400000	2.515552	273.632817	0.050000	2.743929	410.424225	0.050000	1.126571
164.649690	0.500000	2.509529	273.642817	0.060000	2.743385	410.434225	0.060000	1.126909
164.749690	0.600000	2.511495	273.652817	0.070000	2.736181	410.444225	0.070000	1.123240
164.849690	0.700000	2.484067	273.662817	0.080000	2.720450	410.454225	0.080000	1.125417
164.949690	0.800000	2.531316	273.672817	0.090000	2.743075	410.464225	0.090000	1.125799
165.049690	0.900000	2.517970	273.682817	0.100000	2.743991	410.474225	0.100000	1.125944
165.149690	1.000000	2.512416	273.782817	0.200000	2.744620	410.574225	0.200000	1.126248
166.149690	2.000000	2.503457	273.882817	0.300000	2.745497	410.674225	0.300000	1.126665
167.149690	3.000000	2.491400	273.982817	0.400000	2.747103	410.774225	0.400000	1.127203
168.149690	4.000000	2.483380	274.082817	0.500000	2.747248	410.874225	0.500000	1.127373
169.149690	5.000000	2.473204	274.182817	0.600000	2.748581	410.974225	0.600000	1.126909
170.149690	6.000000	2.463049	274.282817	0.700000	2.750299	411.074225	0.700000	1.127732
171.149690	7.000000	2.453045	274.382817	0.800000	2.751202	411.174225	0.800000	1.128383
172.074845	90.000000	2.444767	274.482817	0.900000	2.751934	411.274225	0.900000	1.128474
172.149690	8.000000	2.444072	274.582817	1.000000	2.751791	411.374225	1.000000	1.128950
173.149690	9.000000	2.434785	275.582817	2.000000	2.762864	412.374225	2.000000	1.132390
174.149690	10.000000	2.425364	276.582817	3.000000	2.772663	413.374225	3.000000	1.135782
182.074845	100.000000	2.353050	277.582817	4.000000	2.782257	414.374225	4.000000	1.139035
184.149690	20.000000	2.334613	278.582817	5.000000	2.792737	415.374225	5.000000	1.142493
194.149690	30.000000	2.248341	279.582817	6.000000	2.802800	416.374225	6.000000	1.145869
204.149690	40.000000	2.166431	280.582817	7.000000	2.812707	417.374225	7.000000	1.149204
214.149690	50.000000	2.088821	281.582817	8.000000	2.822763	418.374225	8.000000	1.152536
224.149690	60.000000	2.015417	282.074845	200.000000	2.827718	419.374225	9.000000	1.155868

J = 1 → J’ = 0			J = 1 → J’ = 2			J = 1 → J’ = 3		
E_{tot} (cm⁻¹)	E_{col} (cm⁻¹)	σ (Å²)	E_{tot} (cm⁻¹)	E_{col} (cm⁻¹)	σ (Å²)	E_{tot} (cm⁻¹)	E_{col} (cm⁻¹)	σ (Å²)
227.358282	200.000000	1.992769	282.582817	9.000000	2.832799	420.374225	10.000000	1.159183
234.149690	70.000000	1.946234	283.582817	10.000000	2.842808	427.358282	400.000000	1.181986
244.149690	80.000000	1.881181	293.582817	20.000000	2.941348	430.374225	20.000000	1.191651
254.149690	90.000000	1.820213	303.582817	30.000000	3.037716	440.374225	30.000000	1.222927
264.149690	100.000000	1.763235	313.582817	40.000000	3.131855	450.374225	40.000000	1.253041
273.582917	0.000100	1.713123	323.582817	50.000000	3.223853	460.374225	50.000000	1.282025
273.583017	0.000200	1.713123	327.358282	300.000000	3.258024	464.149690	300.000000	1.292683
273.583117	0.000300	1.713123	333.582817	60.000000	3.313801	470.374225	60.000000	1.309918
273.583217	0.000400	1.713123	343.582817	70.000000	3.401782	473.582817	200.000000	1.318640
273.583317	0.000500	1.713123	353.582817	80.000000	3.487908	480.374225	70.000000	1.336745
273.583417	0.000600	1.713123	363.582817	90.000000	3.572247	482.074845	400.000000	1.341206
273.583517	0.000700	1.713123	364.149690	200.000000	3.576980	490.374225	80.000000	1.362550
273.583617	0.000800	1.713122	373.582817	100.000000	3.654924	500.374225	90.000000	1.387361
273.583717	0.000900	1.713122	382.074845	300.000000	3.723975	510.374225	100.000000	1.411212
273.583817	0.001000	1.713122	410.374325	0.000100	3.947782	527.358282	500.000000	1.449617
273.584817	0.002000	1.713118	410.374425	0.000200	3.947728	564.149690	400.000000	1.525084
273.585817	0.003000	1.713112	410.374525	0.000300	3.947677	573.582817	300.000000	1.541855
273.586817	0.004000	1.713106	410.374625	0.000400	3.947630	582.074845	500.000000	1.557136
273.587817	0.005000	1.713100	410.374725	0.000500	3.947585	610.374225	200.000000	1.604314
273.588817	0.006000	1.713093	410.374825	0.000600	3.947544	627.358282	600.000000	1.630256
273.589817	0.007000	1.713087	410.374925	0.000700	3.947506	664.149690	500.000000	1.681086
273.590817	0.008000	1.713080	410.375025	0.000800	3.947472	673.582817	400.000000	1.693053
273.591817	0.009000	1.713073	410.375225	0.001000	3.947415	682.074845	600.000000	1.703489
273.592817	0.010000	1.713067	410.376225	0.002000	3.947306	710.374225	300.000000	1.736139
273.602817	0.020000	1.713006	410.377225	0.003000	3.947368	727.358282	700.000000	1.754328
273.612817	0.030000	1.712949	410.378225	0.004000	3.947502	764.149690	600.000000	1.790768
273.622817	0.040000	1.712895	410.379225	0.005000	3.947693	773.582817	500.000000	1.799570
273.632817	0.050000	1.712840	410.380225	0.006000	3.947978	782.074845	700.000000	1.807349
273.642817	0.060000	1.712771	410.381225	0.007000	3.948481	810.374225	400.000000	1.832484
273.652817	0.070000	1.712417	410.382225	0.008000	3.949621	827.358282	800.000000	1.847192
273.662817	0.080000	1.711171	410.383225	0.009000	3.949522	864.149690	700.000000	1.878908
273.672817	0.090000	1.712421	410.384225	0.010000	3.945559	873.582817	600.000000	1.887148
273.682817	0.100000	1.712455	410.394225	0.020000	3.947126	882.074845	800.000000	1.894646
273.782817	0.200000	1.711989	410.404225	0.030000	3.947443	910.374225	500.000000	1.920404
273.882817	0.300000	1.711479	410.414225	0.040000	3.947722	927.358282	900.000000	1.936625
273.982817	0.400000	1.710988	410.424225	0.050000	3.948135	964.149690	800.000000	1.974419
274.082817	0.500000	1.710505	410.434225	0.060000	3.949384	973.582817	700.000000	1.984806
274.182817	0.600000	1.710016	410.444225	0.070000	3.945111	982.074845	900.000000	1.994422
274.282817	0.700000	1.708697	410.454225	0.080000	3.946951	1010.374230	600.000000	2.028557
274.382817	0.800000	1.708866	410.464225	0.090000	3.947413	1064.149690	900.000000	2.103319
274.482817	0.900000	1.708376	410.474225	0.100000	3.947698	1073.582820	800.000000	2.117907
274.582817	1.000000	1.707829	410.574225	0.200000	3.948466	1110.374230	700.000000	2.179230
275.582817	2.000000	1.702780	410.674225	0.300000	3.949377	1173.582820	900.000000	2.300762
276.582817	3.000000	1.697728	410.774225	0.400000	3.950203	1210.374230	800.000000	2.379953
277.582817	4.000000	1.692663	410.874225	0.500000	3.950907	1310.374230	900.000000	2.617056
278.582817	5.000000	1.687724	410.974225	0.600000	3.952179			
279.582817	6.000000	1.682790	411.074225	0.700000	3.951901			
280.582817	7.000000	1.677879	411.174225	0.800000	3.953103			
281.582817	8.000000	1.673016	411.274225	0.900000	3.953760			
282.074845	200.000000	1.670641	411.374225	1.000000	3.954695			
282.582817	9.000000	1.668196	412.374225	2.000000	3.962344			
283.582817	10.000000	1.663404	413.374225	3.000000	3.970051			
293.582817	20.000000	1.617503	414.374225	4.000000	3.977668			
303.582817	30.000000	1.575156	415.374225	5.000000	3.985341			
313.582817	40.000000	1.536232	416.374225	6.000000	3.993009			
323.582817	50.000000	1.500617	417.374225	7.000000	4.000647			
327.358282	300.000000	1.488009	418.374225	8.000000	4.008253			
333.582817	60.000000	1.468200	419.374225	9.000000	4.015869			
343.582817	70.000000	1.438862	420.374225	10.000000	4.023473			
353.582817	80.000000	1.412505	427.358282	400.000000	4.076275			
363.582817	90.000000	1.389023	430.374225	20.000000	4.098928			
364.149690	200.000000	1.387777	440.374225	30.000000	4.173448			
373.582817	100.000000	1.368314	450.374225	40.000000	4.247135			
382.074845	300.000000	1.352840	460.374225	50.000000	4.320055			
410.374325	0.000100	1.314301	464.149690	300.000000	4.347422			
410.374425	0.000200	1.314303	470.374225	60.000000	4.392329			
410.374525	0.000300	1.314307	473.582817	200.000000	4.415388			
410.374625	0.000400	1.314312	480.374225	70.000000	4.464010			
410.374725	0.000500	1.314318	482.074845	400.000000	4.476148			
410.374825	0.000600	1.314325	490.374225	80.000000	4.535190			
410.374925	0.000700	1.314332	500.374225	90.000000	4.605924			
410.375025	0.000800	1.314340	510.374225	100.000000	4.676288			
410.375225	0.001000	1.314357	527.358282	500.000000	4.795102			
410.376225	0.002000	1.314437	564.149690	400.000000	5.050400			
410.377225	0.003000	1.314490	573.582817	300.000000	5.115490			
410.378225	0.004000	1.314523	582.074845	500.000000	5.174024			
410.379225	0.005000	1.314541	610.374225	200.000000	5.368299			
410.380225	0.006000	1.314548	627.358282	600.000000	5.484150			

J = 1 → J' = 0			J = 1 → J' = 2			J = 1 → J' = 3		
E_{tot} (cm⁻¹)	E_{col} (cm⁻¹)	σ (Å²)	E_{tot} (cm⁻¹)	E_{col} (cm⁻¹)	σ (Å²)	E_{tot} (cm⁻¹)	E_{col} (cm⁻¹)	σ (Å²)
410.381225	0.007000	1.314527	664.149690	500.000000	5.731732			
410.382225	0.008000	1.314366	673.582817	400.000000	5.794097			
410.383225	0.009000	1.313248	682.074845	600.000000	5.849714			
410.384225	0.010000	1.314036	710.374225	300.000000	6.030528			
410.394225	0.020000	1.314538	727.358282	700.000000	6.134847			
410.404225	0.030000	1.314541	764.149690	600.000000	6.346593			
410.414225	0.040000	1.314534	773.582817	500.000000	6.397193			
410.424225	0.050000	1.314518	782.074845	700.000000	6.441301			
410.434225	0.060000	1.314438	810.374225	400.000000	6.577698			
410.444225	0.070000	1.314410	827.358282	800.000000	6.651215			
410.454225	0.080000	1.314492	864.149690	700.000000	6.787674			
410.464225	0.090000	1.314482	873.582817	600.000000	6.817518			
410.474225	0.100000	1.314458	882.074845	800.000000	6.842575			
410.574225	0.200000	1.314355	910.374225	500.000000	6.913756			
410.674225	0.300000	1.314250	927.358282	900.000000	6.947500			
410.774225	0.400000	1.314177	964.149690	800.000000	6.998301			
410.874225	0.500000	1.314056	973.582817	700.000000	7.006600			
410.974225	0.600000	1.313965	982.074845	900.000000	7.012499			
411.074225	0.700000	1.313849	1010.374230	600.000000	7.021777			
411.174225	0.800000	1.313760	1064.149690	900.000000	7.000260			
411.274225	0.900000	1.313632	1073.582820	800.000000	6.991936			
411.374225	1.000000	1.313541	1110.374230	700.000000	6.949099			
412.374225	2.000000	1.312581	1173.582820	900.000000	6.847953			
413.374225	3.000000	1.311628	1210.374230	800.000000	6.780319			
414.374225	4.000000	1.310693	1310.374230	900.000000	6.591944			
415.374225	5.000000	1.309799						
416.374225	6.000000	1.308923						
417.374225	7.000000	1.308066						
418.374225	8.000000	1.307234						
419.374225	9.000000	1.306428						
420.374225	10.000000	1.305641						
427.358282	400.000000	1.300796						
430.374225	20.000000	1.299042						
440.374225	30.000000	1.294673						
450.374225	40.000000	1.292464						
460.374225	50.000000	1.292351						
464.149690	300.000000	1.292841						
470.374225	60.000000	1.294270						
473.582817	200.000000	1.295307						
480.374225	70.000000	1.298161						
482.074845	400.000000	1.299015						
490.374225	80.000000	1.303969						
500.374225	90.000000	1.311631						
510.374225	100.000000	1.321097						
527.358282	500.000000	1.341141						
564.149690	400.000000	1.400744						
573.582817	300.000000	1.418924						
582.074845	500.000000	1.436615						
610.374225	200.000000	1.502009						
627.358282	600.000000	1.545554						
664.149690	500.000000	1.648492						
673.582817	400.000000	1.676320						
682.074845	600.000000	1.701723						
710.374225	300.000000	1.787851						
727.358282	700.000000	1.839829						
764.149690	600.000000	1.950096						
773.582817	500.000000	1.977374						
782.074845	700.000000	2.001447						
810.374225	400.000000	2.077730						
827.358282	800.000000	2.120118						
864.149690	700.000000	2.201780						
873.582817	600.000000	2.220307						
882.074845	800.000000	2.236102						
910.374225	500.000000	2.282575						
927.358282	900.000000	2.305822						
964.149690	800.000000	2.344049						
973.582817	700.000000	2.351169						
982.074845	900.000000	2.356640						
1010.374230	600.000000	2.368565						
1064.149690	900.000000	2.365323						
1073.582820	800.000000	2.361460						
1110.374230	700.000000	2.337963						
1173.582820	900.000000	2.271559						
1210.374230	800.000000	2.222146						
1310.374230	900.000000	2.072224						

Inelastic collisions cross-sections					
J = 1 → J' = 4			J = 1 → J' = 5		
E_{tot} (cm⁻¹)	E_{col} (cm⁻¹)	σ (Å²)	E_{tot} (cm⁻¹)	E_{col} (cm⁻¹)	σ (Å²)
273.582917	0.000100	0.000115	410.374325	0.000100	0.000023
273.583017	0.000200	0.000113	410.374425	0.000200	0.000024
273.583117	0.000300	0.000107	410.374525	0.000300	0.000023
273.583217	0.000400	0.000102	410.374625	0.000400	0.000022
273.583317	0.000500	0.000097	410.374725	0.000500	0.000021
273.583417	0.000600	0.000092	410.374825	0.000600	0.000020
273.583517	0.000700	0.000088	410.374925	0.000700	0.000020
273.583617	0.000800	0.000085	410.375025	0.000800	0.000019
273.583717	0.000900	0.000082	410.375225	0.001000	0.000018
273.583817	0.001000	0.000079	410.376225	0.002000	0.000014
273.584817	0.002000	0.000064	410.377225	0.003000	0.000012
273.585817	0.003000	0.000059	410.378225	0.004000	0.000011
273.586817	0.004000	0.000060	410.379225	0.005000	0.000011
273.587817	0.005000	0.000064	410.380225	0.006000	0.000012
273.588817	0.006000	0.000070	410.381225	0.007000	0.000012
273.589817	0.007000	0.000076	410.382225	0.008000	0.000013
273.590817	0.008000	0.000084	410.383225	0.009000	0.000014
273.591817	0.009000	0.000093	410.384225	0.010000	0.000016
273.592817	0.010000	0.000101	410.394225	0.020000	0.000028
273.602817	0.020000	0.000186	410.404225	0.030000	0.000038
273.612817	0.030000	0.000256	410.414225	0.040000	0.000047
273.622817	0.040000	0.000317	410.424225	0.050000	0.000057
273.632817	0.050000	0.000389	410.434225	0.060000	0.000228
273.642817	0.060000	0.000566	410.444225	0.070000	0.000085
273.652817	0.070000	0.003564	410.454225	0.080000	0.000109
273.662817	0.080000	0.002093	410.464225	0.090000	0.000159
273.672817	0.090000	0.001334	410.474225	0.100000	0.000272
273.682817	0.100000	0.001562	410.574225	0.200000	0.000268
273.782817	0.200000	0.002114	410.674225	0.300000	0.000336
273.882817	0.300000	0.002218	410.774225	0.400000	0.000197
273.982817	0.400000	0.001495	410.874225	0.500000	0.000230
274.082817	0.500000	0.001942	410.974225	0.600000	0.000536
274.182817	0.600000	0.001823	411.074225	0.700000	0.000261
274.282817	0.700000	0.001944	411.174225	0.800000	0.000285
274.382817	0.800000	0.002127	411.274225	0.900000	0.000394
274.482817	0.900000	0.002501	411.374225	1.000000	0.000451
274.582817	1.000000	0.004021	412.374225	2.000000	0.000380
275.582817	2.000000	0.002865	413.374225	3.000000	0.000467
276.582817	3.000000	0.003591	414.374225	4.000000	0.000607
277.582817	4.000000	0.004987	415.374225	5.000000	0.000564
278.582817	5.000000	0.004395	416.374225	6.000000	0.000598
279.582817	6.000000	0.004646	417.374225	7.000000	0.000655
280.582817	7.000000	0.005143	418.374225	8.000000	0.000692
281.582817	8.000000	0.005419	419.374225	9.000000	0.000725
282.074845	200.000000	0.005521	420.374225	10.000000	0.000764
282.582817	9.000000	0.005646	427.358282	400.000000	0.001087
283.582817	10.000000	0.005938	430.374225	20.000000	0.001249
293.582817	20.000000	0.009486	440.374225	30.000000	0.001905
303.582817	30.000000	0.014021	450.374225	40.000000	0.002764
313.582817	40.000000	0.019635	460.374225	50.000000	0.003852
323.582817	50.000000	0.026381	464.149690	300.000000	0.004328
327.358282	300.000000	0.029230	470.374225	60.000000	0.005196
333.582817	60.000000	0.034295	473.582817	200.000000	0.005685
343.582817	70.000000	0.043399	480.374225	70.000000	0.006819
353.582817	80.000000	0.053701	482.074845	400.000000	0.007125
363.582817	90.000000	0.065195	490.374225	80.000000	0.008744
364.149690	200.000000	0.065882	500.374225	90.000000	0.010990
373.582817	100.000000	0.077865	510.374225	100.000000	0.013574
382.074845	300.000000	0.089521	527.358282	500.000000	0.018772
410.374325	0.000100	0.133878	564.149690	400.000000	0.032926
410.374425	0.000200	0.133922	573.582817	300.000000	0.038342
410.374525	0.000300	0.133961	582.074845	500.000000	0.042616
410.374625	0.000400	0.133996	610.374225	200.000000	0.058990
410.374725	0.000500	0.134026	627.358282	600.000000	0.069980
410.374825	0.000600	0.134052	664.149690	500.000000	0.096300
410.374925	0.000700	0.134073	673.582817	400.000000	0.103516
410.375025	0.000800	0.134091	682.074845	600.000000	0.110150
410.375225	0.001000	0.134114	710.374225	300.000000	0.133058
410.376225	0.002000	0.134077	727.358282	700.000000	0.147284
410.377225	0.003000	0.133934	764.149690	600.000000	0.178945
410.378225	0.004000	0.133763	773.582817	500.000000	0.187179
410.379225	0.005000	0.133563	782.074845	700.000000	0.194624
410.380225	0.006000	0.133298	810.374225	400.000000	0.219530
410.381225	0.007000	0.132873	827.358282	800.000000	0.234479
410.382225	0.008000	0.132065	864.149690	700.000000	0.266591
410.383225	0.009000	0.133707	873.582817	600.000000	0.274720
410.384225	0.010000	0.136224	882.074845	800.000000	0.281986

J = 1 → J' = 4			J = 1 → J' = 5		
E_{tot} (cm⁻¹)	E_{col} (cm⁻¹)	σ (Å²)	E_{tot} (cm⁻¹)	E_{col} (cm⁻¹)	σ (Å²)
410.394225	0.020000	0.134167	910.374225	500.000000	0.305768
410.404225	0.030000	0.133944	927.358282	900.000000	0.319648
410.414225	0.040000	0.133774	964.149690	800.000000	0.348418
410.424225	0.050000	0.133508	973.582817	700.000000	0.355462
410.434225	0.060000	0.132660	982.074845	900.000000	0.361662
410.444225	0.070000	0.136440	1010.374230	600.000000	0.381423
410.454225	0.080000	0.134726	1064.149690	900.000000	0.414532
410.464225	0.090000	0.134369	1073.582820	800.000000	0.419717
410.474225	0.100000	0.134142	1110.374230	700.000000	0.438215
410.574225	0.200000	0.134331	1173.582820	900.000000	0.464383
410.674225	0.300000	0.134341	1210.374230	800.000000	0.477226
410.774225	0.400000	0.134523	1310.374230	900.000000	0.508450
410.874225	0.500000	0.134777			
410.974225	0.600000	0.134449			
411.074225	0.700000	0.135543			
411.174225	0.800000	0.135330			
411.274225	0.900000	0.135574			
411.374225	1.000000	0.135548			
412.374225	2.000000	0.137366			
413.374225	3.000000	0.139030			
414.374225	4.000000	0.140767			
415.374225	5.000000	0.142537			
416.374225	6.000000	0.144290			
417.374225	7.000000	0.146047			
418.374225	8.000000	0.147837			
419.374225	9.000000	0.149631			
420.374225	10.000000	0.151431			
427.358282	400.000000	0.164262			
430.374225	20.000000	0.169938			
440.374225	30.000000	0.189312			
450.374225	40.000000	0.209500			
460.374225	50.000000	0.230446			
464.149690	300.000000	0.238540			
470.374225	60.000000	0.252094			
473.582817	200.000000	0.259180			
480.374225	70.000000	0.274388			
482.074845	400.000000	0.278240			
490.374225	80.000000	0.297271			
500.374225	90.000000	0.320684			
510.374225	100.000000	0.344571			
527.358282	500.000000	0.386065			
564.149690	400.000000	0.479197			
573.582817	300.000000	0.502898			
582.074845	500.000000	0.524684			
610.374225	200.000000	0.597242			
627.358282	600.000000	0.640459			
664.149690	500.000000	0.732188			
673.582817	400.000000	0.755148			
682.074845	600.000000	0.775588			
710.374225	300.000000	0.842005			
727.358282	700.000000	0.880535			
764.149690	600.000000	0.960515			
773.582817	500.000000	0.980294			
782.074845	700.000000	0.997845			
810.374225	400.000000	1.054785			
827.358282	800.000000	1.087934			
864.149690	700.000000	1.157629			
873.582817	600.000000	1.175124			
882.074845	800.000000	1.190768			
910.374225	500.000000	1.242326			
927.358282	900.000000	1.272945			
964.149690	800.000000	1.338764			
973.582817	700.000000	1.355564			
982.074845	900.000000	1.370682			
1010.374230	600.000000	1.420907			
1064.149690	900.000000	1.515974			
1073.582820	800.000000	1.532592			
1110.374230	700.000000	1.597226			
1173.582820	900.000000	1.707688			
1210.374230	800.000000	1.771865			
1310.374230	900.000000	1.948072			

Inelastic collisions cross-sections								
J = 2 → J' = 0			J = 2 → J' = 1			J = 2 → J' = 3		
E_{tot} (cm^{-1})	E_{col} (cm^{-1})	σ (Å²)	E_{tot} (cm^{-1})	E_{col} (cm^{-1})	σ (Å²)	E_{tot} (cm^{-1})	E_{col} (cm^{-1})	σ (Å²)
82.074945	0.000100	60.074212	82.074945	0.000100	427.254140	164.149790	0.000100	0.002620
82.075045	0.000200	28.899611	82.075045	0.000200	214.875816	164.149890	0.000200	0.002664
82.075145	0.000300	20.813165	82.075145	0.000300	162.485652	164.149990	0.000300	0.002818
82.075245	0.000400	17.845180	82.075245	0.000400	145.248442	164.150090	0.000400	0.003085
82.075345	0.000500	16.714402	82.075345	0.000500	140.469189	164.150190	0.000500	0.003448
82.075445	0.000600	16.407506	82.075445	0.000600	141.173085	164.150290	0.000600	0.003895
82.075545	0.000700	16.527272	82.075545	0.000700	144.640660	164.150390	0.000700	0.004416
82.075645	0.000800	16.884922	82.075645	0.000800	149.587651	164.150490	0.000800	0.005001
82.075745	0.000900	17.378850	82.075745	0.000900	155.322476	164.150590	0.000900	0.005642
82.075845	0.001000	17.953715	82.075845	0.001000	161.477269	164.150690	0.001000	0.006331
82.076845	0.002000	24.453455	82.076845	0.002000	222.478144	164.151690	0.002000	0.014195
82.077845	0.003000	29.939563	82.077845	0.003000	269.374050	164.152690	0.003000	0.020031
82.078845	0.004000	34.469438	82.078845	0.004000	305.748771	164.159690	0.010000	0.019962
82.079845	0.005000	38.591519	82.079845	0.005000	337.304156	164.169690	0.020000	0.020461
82.080845	0.006000	42.709003	82.080845	0.006000	367.900398	164.179690	0.030000	0.023046
82.081845	0.007000	47.113007	82.081845	0.007000	400.142241	164.189690	0.040000	0.026959
82.082845	0.008000	51.995084	82.082845	0.008000	435.505106	164.199690	0.050000	0.173199
82.083845	0.009000	57.468228	82.083845	0.009000	475.102500	164.209690	0.060000	0.047256
82.084845	0.010000	63.596708	82.084845	0.010000	519.074850	164.219690	0.070000	0.046851
82.094845	0.020000	80.226096	82.094845	0.020000	610.836254	164.229690	0.080000	0.054573
82.104845	0.030000	23.516926	82.104845	0.030000	189.005917	164.239690	0.090000	0.070549
82.114845	0.040000	9.882019	82.114845	0.040000	90.707366	164.249690	0.100000	0.099787
82.124845	0.050000	5.720863	82.124845	0.050000	61.944652	164.349690	0.200000	0.219172
82.134845	0.060000	3.996267	82.134845	0.060000	53.106948	164.449690	0.300000	0.331998
82.144845	0.070000	3.179345	82.144845	0.070000	58.023639	164.549690	0.400000	0.110292
82.154845	0.080000	2.835895	82.154845	0.080000	95.627048	164.649690	0.500000	0.105956
82.164845	0.090000	2.859131	82.164845	0.090000	226.631897	164.749690	0.600000	0.131402
82.174845	0.100000	3.248494	82.174845	0.100000	180.678610	164.849690	0.700000	0.125648
82.274845	0.200000	1.701243	82.274845	0.200000	42.301658	164.949690	0.800000	0.131412
82.374845	0.300000	1.576648	82.374845	0.300000	28.939829	165.049690	0.900000	0.182777
82.474845	0.400000	0.830152	82.474845	0.400000	14.763069	165.149690	1.000000	0.242294
82.574845	0.500000	0.668767	82.574845	0.500000	12.841366	166.149690	2.000000	0.201748
82.674845	0.600000	0.838240	82.674845	0.600000	12.743074	167.149690	3.000000	0.226850
82.774845	0.700000	2.725258	82.774845	0.700000	23.224481	168.149690	4.000000	0.326732
82.874845	0.800000	0.715068	82.874845	0.800000	11.935317	169.149690	5.000000	0.285186
82.974845	0.900000	0.611766	82.974845	0.900000	11.843292	170.149690	6.000000	0.286555
83.074845	1.000000	0.700978	83.074845	1.000000	17.759041	171.149690	7.000000	0.310496
84.074845	2.000000	0.435847	84.074845	2.000000	5.862697	172.074845	90.000000	0.324991
85.074845	3.000000	0.282772	85.074845	3.000000	4.842221	172.149690	8.000000	0.325371
86.074845	4.000000	0.407788	86.074845	4.000000	5.811988	173.149690	9.000000	0.330573
87.074845	5.000000	0.216700	87.074845	5.000000	3.529802	174.149690	10.000000	0.339466
87.358282	60.000000	0.204919	87.358282	60.000000	3.337434	182.074845	100.000000	0.420255
88.074845	6.000000	0.188208	88.074845	6.000000	3.027853	184.149690	20.000000	0.440436
89.074845	7.000000	0.190995	89.074845	7.000000	2.854421	194.149690	30.000000	0.535905
90.074845	8.000000	0.172343	90.074845	8.000000	2.598102	204.149690	40.000000	0.629630
91.074845	9.000000	0.155456	91.074845	9.000000	2.359729	214.149690	50.000000	0.722624
92.074845	10.000000	0.146833	92.074845	10.000000	2.201563	224.149690	60.000000	0.815259
97.358282	70.000000	0.123911	97.358282	70.000000	1.748595	227.358282	200.000000	0.844854
102.074845	20.000000	0.116235	102.074845	20.000000	1.563814	234.149690	70.000000	0.907342
107.358282	80.000000	0.114080	107.358282	80.000000	1.457033	244.149690	80.000000	0.998567
112.074845	30.000000	0.115235	112.074845	30.000000	1.406635	254.149690	90.000000	1.088081
117.358282	90.000000	0.118647	117.358282	90.000000	1.378349	264.149690	100.000000	1.178480
122.074845	40.000000	0.122979	122.074845	40.000000	1.368645	273.582917	0.000100	1.267113
127.358282	100.000000	0.128863	127.358282	100.000000	1.369105	273.583017	0.000200	1.266923
132.074845	50.000000	0.134804	132.074845	50.000000	1.376318	273.583117	0.000300	1.266717
142.074845	60.000000	0.148768	142.074845	60.000000	1.404134	273.583217	0.000400	1.266506
152.074845	70.000000	0.322081	152.074845	70.000000	1.810723	273.583317	0.000500	1.266295
162.074845	80.000000	0.186156	162.074845	80.000000	1.511554	273.583417	0.000600	1.266086
164.149790	0.000100	0.209880	164.149790	0.000100	1.549309	273.583517	0.000700	1.265879
164.149890	0.000200	0.211075	164.149890	0.000200	1.553566	273.583617	0.000800	1.265676
164.149990	0.000300	0.212245	164.149990	0.000300	1.557813	273.583717	0.000900	1.265476
164.150090	0.000400	0.213397	164.150090	0.000400	1.562030	273.583817	0.001000	1.265281
164.150190	0.000500	0.214518	164.150190	0.000500	1.566231	273.584817	0.002000	1.263851
164.150290	0.000600	0.215604	164.150290	0.000600	1.570396	273.585817	0.003000	1.271407
164.150390	0.000700	0.216652	164.150390	0.000700	1.574489	273.586817	0.004000	1.314060
164.150490	0.000800	0.217651	164.150490	0.000800	1.578479	273.587817	0.005000	1.275115
164.150590	0.000900	0.218592	164.150590	0.000900	1.582355	273.588817	0.006000	1.268458
164.150690	0.001000	0.219474	164.150690	0.001000	1.586087	273.589817	0.007000	1.265786
164.151690	0.002000	0.224139	164.151690	0.002000	1.611745	273.590817	0.008000	1.264240
164.152690	0.003000	0.221967	164.152690	0.003000	1.614405	273.591817	0.009000	1.263174
164.159690	0.010000	0.196250	164.159690	0.010000	1.541784	273.592817	0.010000	1.262363
164.169690	0.020000	0.188373	164.169690	0.020000	1.512290	273.602817	0.020000	1.258412
164.179690	0.030000	0.185851	164.179690	0.030000	1.501630	273.612817	0.030000	1.256280
164.189690	0.040000	0.184478	164.189690	0.040000	1.495935	273.622817	0.040000	1.254263
164.199690	0.050000	0.183515	164.199690	0.050000	1.527994	273.632817	0.050000	1.251613
164.209690	0.060000	0.182818	164.209690	0.060000	1.485582	273.642817	0.060000	1.246952
164.219690	0.070000	0.182698	164.219690	0.070000	1.478253	273.652817	0.070000	1.239248

J = 2 → J' = 0			J = 2 → J' = 1			J = 2 → J' = 3		
E_{tot} (cm⁻¹)	E_{col} (cm⁻¹)	σ (Å²)	E_{tot} (cm⁻¹)	E_{col} (cm⁻¹)	σ (Å²)	E_{tot} (cm⁻¹)	E_{col} (cm⁻¹)	σ (Å²)
164.229690	0.080000	0.185623	164.229690	0.080000	1.473363	273.662817	0.080000	1.398587
164.239690	0.090000	0.222749	164.239690	0.090000	1.526221	273.672817	0.090000	1.282018
164.249690	0.100000	0.291600	164.249690	0.100000	1.758808	273.682817	0.100000	1.270049
164.349690	0.200000	0.188118	164.349690	0.200000	1.509060	273.782817	0.200000	1.261896
164.449690	0.300000	0.185805	164.449690	0.300000	1.499828	273.882817	0.300000	1.261931
164.549690	0.400000	0.185646	164.549690	0.400000	1.499241	273.982817	0.400000	1.261575
164.649690	0.500000	0.183088	164.649690	0.500000	1.485398	274.082817	0.500000	1.272138
164.749690	0.600000	0.187660	164.749690	0.600000	1.507359	274.182817	0.600000	1.262244
164.849690	0.700000	0.187650	164.849690	0.700000	1.478079	274.282817	0.700000	1.305359
164.949690	0.800000	0.197233	164.949690	0.800000	1.541959	274.382817	0.800000	1.268815
165.049690	0.900000	0.189596	165.049690	0.900000	1.513120	274.482817	0.900000	1.266711
165.149690	1.000000	0.187765	165.149690	1.000000	1.504898	274.582817	1.000000	1.265253
166.149690	2.000000	0.189875	166.149690	2.000000	1.513204	275.582817	2.000000	1.277270
167.149690	3.000000	0.190832	167.149690	3.000000	1.513209	276.582817	3.000000	1.284858
168.149690	4.000000	0.194181	168.149690	4.000000	1.522537	277.582817	4.000000	1.293998
169.149690	5.000000	0.195048	169.149690	5.000000	1.525500	278.582817	5.000000	1.302491
170.149690	6.000000	0.196674	170.149690	6.000000	1.529692	279.582817	6.000000	1.310860
171.149690	7.000000	0.198467	171.149690	7.000000	1.534167	280.582817	7.000000	1.319306
172.074845	90.000000	0.200354	172.074845	90.000000	1.539767	281.582817	8.000000	1.327993
172.149690	8.000000	0.200492	172.149690	8.000000	1.540264	282.074845	200.000000	1.332219
173.149690	9.000000	0.202371	173.149690	9.000000	1.545650	282.582817	9.000000	1.336555
174.149690	10.000000	0.204230	174.149690	10.000000	1.550876	283.582817	10.000000	1.345078
182.074845	100.000000	0.219384	182.074845	100.000000	1.594378	293.582817	20.000000	1.429710
184.149690	20.000000	0.223388	184.149690	20.000000	1.605957	303.582817	30.000000	1.513096
194.149690	30.000000	0.242758	194.149690	30.000000	1.662686	313.582817	40.000000	1.595167
204.149690	40.000000	0.262053	204.149690	40.000000	1.720382	323.582817	50.000000	1.676028
214.149690	50.000000	0.281060	214.149690	50.000000	1.778380	327.358282	300.000000	1.706215
224.149690	60.000000	0.299591	224.149690	60.000000	1.836345	333.582817	60.000000	1.755697
227.358282	200.000000	0.305413	227.358282	200.000000	1.854926	343.582817	70.000000	1.834277
234.149690	70.000000	0.317521	234.149690	70.000000	1.894109	353.582817	80.000000	1.911794
244.149690	80.000000	0.334738	244.149690	80.000000	1.951459	363.582817	90.000000	1.988316
254.149690	90.000000	0.351204	254.149690	90.000000	2.008351	364.149690	200.000000	1.992636
264.149690	100.000000	0.366809	264.149690	100.000000	2.064515	373.582817	100.000000	2.063919
273.582917	0.000100	0.380366	273.582917	0.000100	2.117142	382.074845	300.000000	2.127402
273.583017	0.000200	0.380373	273.583017	0.000200	2.117211	410.374325	0.000100	2.332836
273.583117	0.000300	0.380381	273.583117	0.000300	2.117284	410.374425	0.000200	2.332871
273.583217	0.000400	0.380389	273.583217	0.000400	2.117359	410.374525	0.000300	2.332913
273.583317	0.000500	0.380396	273.583317	0.000500	2.117436	410.374625	0.000400	2.332961
273.583417	0.000600	0.380403	273.583417	0.000600	2.117514	410.374725	0.000500	2.333013
273.583517	0.000700	0.380411	273.583517	0.000700	2.117596	410.374825	0.000600	2.333067
273.583617	0.000800	0.380418	273.583617	0.000800	2.117680	410.374925	0.000700	2.333124
273.583717	0.000900	0.380424	273.583717	0.000900	2.117766	410.375025	0.000800	2.333182
273.583817	0.001000	0.380431	273.583817	0.001000	2.117857	410.375225	0.001000	2.333298
273.584817	0.002000	0.380488	273.584817	0.002000	2.119137	410.376225	0.002000	2.333781
273.585817	0.003000	0.380533	273.585817	0.003000	2.122334	410.377225	0.003000	2.334054
273.586817	0.004000	0.380569	273.586817	0.004000	2.109374	410.378225	0.004000	2.334181
273.587817	0.005000	0.380599	273.587817	0.005000	2.112851	410.379225	0.005000	2.334206
273.588817	0.006000	0.380624	273.588817	0.006000	2.114323	410.380225	0.006000	2.334116
273.589817	0.007000	0.380645	273.589817	0.007000	2.114999	410.381225	0.007000	2.333757
273.590817	0.008000	0.380663	273.590817	0.008000	2.115387	410.382225	0.008000	2.332200
273.591817	0.009000	0.380679	273.591817	0.009000	2.115641	410.383225	0.009000	2.325145
273.592817	0.010000	0.380693	273.592817	0.010000	2.115822	410.384225	0.010000	2.332057
273.602817	0.020000	0.380779	273.602817	0.020000	2.116468	410.394225	0.020000	2.334673
273.612817	0.030000	0.380827	273.612817	0.030000	2.116634	410.404225	0.030000	2.334718
273.622817	0.040000	0.380865	273.622817	0.040000	2.116692	410.414225	0.040000	2.334715
273.632817	0.050000	0.380901	273.632817	0.050000	2.116623	410.424225	0.050000	2.334604
273.642817	0.060000	0.380928	273.642817	0.060000	2.116178	410.434225	0.060000	2.332968
273.652817	0.070000	0.380371	273.652817	0.070000	2.110597	410.444225	0.070000	2.335163
273.662817	0.080000	0.378844	273.662817	0.080000	2.098438	410.454225	0.080000	2.335359
273.672817	0.090000	0.380628	273.672817	0.090000	2.115865	410.464225	0.090000	2.335290
273.682817	0.100000	0.380753	273.682817	0.100000	2.116548	410.474225	0.100000	2.335178
273.782817	0.200000	0.380925	273.782817	0.200000	2.116788	410.574225	0.200000	2.335851
273.882817	0.300000	0.381057	273.882817	0.300000	2.117218	410.674225	0.300000	2.336457
273.982817	0.400000	0.381287	273.982817	0.400000	2.118212	410.774225	0.400000	2.337342
274.082817	0.500000	0.381074	274.082817	0.500000	2.118079	410.874225	0.500000	2.337962
274.182817	0.600000	0.381455	274.182817	0.600000	2.118862	410.974225	0.600000	2.336345
274.282817	0.700000	0.381938	274.282817	0.700000	2.119942	411.074225	0.700000	2.339704
274.382817	0.800000	0.381856	274.382817	0.800000	2.120394	411.174225	0.800000	2.340212
274.482817	0.900000	0.381963	274.482817	0.900000	2.120714	411.274225	0.900000	2.340765
274.582817	1.000000	0.381979	274.582817	1.000000	2.120360	411.374225	1.000000	2.341413
275.582817	2.000000	0.383467	275.582817	2.000000	2.126457	412.374225	2.000000	2.348696
276.582817	3.000000	0.384846	276.582817	3.000000	2.131580	413.374225	3.000000	2.355785
277.582817	4.000000	0.386212	277.582817	4.000000	2.136554	414.374225	4.000000	2.362850
278.582817	5.000000	0.387617	278.582817	5.000000	2.142216	415.374225	5.000000	2.370062
279.582817	6.000000	0.388997	279.582817	6.000000	2.147564	416.374225	6.000000	2.377198
280.582817	7.000000	0.390358	280.582817	7.000000	2.152799	417.374225	7.000000	2.384319
281.582817	8.000000	0.391723	281.582817	8.000000	2.158156	418.374225	8.000000	2.391429
282.074845	200.000000	0.392391	282.074845	200.000000	2.160800	419.374225	9.000000	2.398555

J = 2 → J' = 0			J = 2 → J' = 1			J = 2 → J' = 3		
E_{tot} (cm⁻¹)	E_{col} (cm⁻¹)	σ (Å²)	E_{tot} (cm⁻¹)	E_{col} (cm⁻¹)	σ (Å²)	E_{tot} (cm⁻¹)	E_{col} (cm⁻¹)	σ (Å²)
282.582817	9.000000	0.393079	282.582817	9.000000	2.163504	420.374225	10.000000	2.405651
283.582817	10.000000	0.394423	283.582817	10.000000	2.168839	427.358282	400.000000	2.455066
293.582817	20.000000	0.407393	293.582817	20.000000	2.221360	430.374225	20.000000	2.476279
303.582817	30.000000	0.419513	303.582817	30.000000	2.272853	440.374225	30.000000	2.546100
313.582817	40.000000	0.430805	313.582817	40.000000	2.323238	450.374225	40.000000	2.615143
323.582817	50.000000	0.441307	323.582817	50.000000	2.372553	460.374225	50.000000	2.683412
327.358282	300.000000	0.445076	327.358282	300.000000	2.390884	464.149690	300.000000	2.708999
333.582817	60.000000	0.451059	333.582817	60.000000	2.420839	470.374225	60.000000	2.750954
343.582817	70.000000	0.460101	343.582817	70.000000	2.468132	473.582817	200.000000	2.772470
353.582817	80.000000	0.468482	353.582817	80.000000	2.514493	480.374225	70.000000	2.817770
363.582817	90.000000	0.476244	363.582817	90.000000	2.559950	482.074845	400.000000	2.829062
364.149690	200.000000	0.476667	364.149690	200.000000	2.562503	490.374225	80.000000	2.883892
373.582817	100.000000	0.483427	373.582817	100.000000	2.604576	500.374225	90.000000	2.949342
382.074845	300.000000	0.489126	382.074845	300.000000	2.641911	510.374225	100.000000	3.014147
410.374325	0.000100	0.505965	410.374325	0.000100	2.763447	527.358282	500.000000	3.122808
410.374425	0.000200	0.505954	410.374425	0.000200	2.763409	564.149690	400.000000	3.353847
410.374525	0.000300	0.505942	410.374525	0.000300	2.763374	573.582817	300.000000	3.410296
410.374625	0.000400	0.505930	410.374625	0.000400	2.763340	582.074845	500.000000	3.462081
410.374725	0.000500	0.505919	410.374725	0.000500	2.763309	610.374225	200.000000	3.631706
410.374825	0.000600	0.505908	410.374825	0.000600	2.763280	627.358282	600.000000	3.731638
410.374925	0.000700	0.505898	410.374925	0.000700	2.763253	664.149690	500.000000	3.942927
410.375025	0.000800	0.505888	410.375025	0.000800	2.763229	673.582817	400.000000	3.995838
410.375225	0.001000	0.505871	410.375225	0.001000	2.763189	682.074845	600.000000	4.042981
410.376225	0.002000	0.505820	410.376225	0.002000	2.763112	710.374225	300.000000	4.196379
410.377225	0.003000	0.505813	410.377225	0.003000	2.763154	727.358282	700.000000	4.285371
410.378225	0.004000	0.505828	410.378225	0.004000	2.763246	764.149690	600.000000	4.468865
410.379225	0.005000	0.505859	410.379225	0.005000	2.763379	773.582817	500.000000	4.513657
410.380225	0.006000	0.505914	410.380225	0.006000	2.763578	782.074845	700.000000	4.553121
410.381225	0.007000	0.506021	410.381225	0.007000	2.763928	810.374225	400.000000	4.678542
410.382225	0.008000	0.506305	410.382225	0.008000	2.764725	827.358282	800.000000	4.749150
410.383225	0.009000	0.506658	410.383225	0.009000	2.764655	864.149690	700.000000	4.889835
410.384225	0.010000	0.505589	410.384225	0.010000	2.761879	873.582817	600.000000	4.923220
410.394225	0.020000	0.505725	410.394225	0.020000	2.762964	882.074845	800.000000	4.952355
410.404225	0.030000	0.505775	410.404225	0.030000	2.763174	910.374225	500.000000	5.043315
410.414225	0.040000	0.505819	410.414225	0.040000	2.763358	927.358282	900.000000	5.093534
410.424225	0.050000	0.505892	410.424225	0.050000	2.763635	964.149690	800.000000	5.191678
410.434225	0.060000	0.506166	410.434225	0.060000	2.764497	973.582817	700.000000	5.214587
410.444225	0.070000	0.505314	410.444225	0.070000	2.761494	982.074845	900.000000	5.234449
410.454225	0.080000	0.505635	410.454225	0.080000	2.762769	1010.374230	600.000000	5.295471
410.464225	0.090000	0.505715	410.464225	0.090000	2.763081	1064.149690	900.000000	5.390192
410.474225	0.100000	0.505764	410.474225	0.100000	2.763269	1073.582820	800.000000	5.404017
410.574225	0.200000	0.505814	410.574225	0.200000	2.763685	1110.374230	700.000000	5.450380
410.674225	0.300000	0.505893	410.674225	0.300000	2.764203	1173.582820	900.000000	5.505054
410.774225	0.400000	0.505952	410.774225	0.400000	2.764661	1210.374230	800.000000	5.525446
410.874225	0.500000	0.505993	410.874225	0.500000	2.765034	1310.374230	900.000000	5.556512
410.974225	0.600000	0.506105	410.974225	0.600000	2.765804			
411.074225	0.700000	0.506015	411.074225	0.700000	2.765490			
411.174225	0.800000	0.506139	411.174225	0.800000	2.766211			
411.274225	0.900000	0.506176	411.274225	0.900000	2.766551			
411.374225	1.000000	0.506258	411.374225	1.000000	2.767086			
412.374225	2.000000	0.506767	412.374225	2.000000	2.771242			
413.374225	3.000000	0.507296	413.374225	3.000000	2.775441			
414.374225	4.000000	0.507806	414.374225	4.000000	2.779579			
415.374225	5.000000	0.508322	415.374225	5.000000	2.783760			
416.374225	6.000000	0.508834	416.374225	6.000000	2.787939			
417.374225	7.000000	0.509341	417.374225	7.000000	2.792101			
418.374225	8.000000	0.509842	418.374225	8.000000	2.796242			
419.374225	9.000000	0.510342	419.374225	9.000000	2.800393			
420.374225	10.000000	0.510838	420.374225	10.000000	2.804538			
427.358282	400.000000	0.514214	427.358282	400.000000	2.833342			
430.374225	20.000000	0.515627	430.374225	20.000000	2.845713			
440.374225	30.000000	0.520127	440.374225	30.000000	2.886470			
450.374225	40.000000	0.524377	450.374225	40.000000	2.926868			
460.374225	50.000000	0.528409	460.374225	50.000000	2.966940			
464.149690	300.000000	0.529883	464.149690	300.000000	2.982007			
470.374225	60.000000	0.532259	470.374225	60.000000	3.006760			
473.582817	200.000000	0.533459	473.582817	200.000000	3.019486			
480.374225	70.000000	0.535955	480.374225	70.000000	3.046354			
482.074845	400.000000	0.536571	482.074845	400.000000	3.053068			
490.374225	80.000000	0.539530	490.374225	80.000000	3.085773			
500.374225	90.000000	0.543010	500.374225	90.000000	3.125047			
510.374225	100.000000	0.546422	510.374225	100.000000	3.164219			
527.358282	500.000000	0.552131	527.358282	500.000000	3.230595			
564.149690	400.000000	0.564502	564.149690	400.000000	3.374179			
573.582817	300.000000	0.567884	573.582817	300.000000	3.410979			
582.074845	500.000000	0.570901	582.074845	500.000000	3.444140			
610.374225	200.000000	0.581478	610.374225	200.000000	3.554580			
627.358282	600.000000	0.588253	627.358282	600.000000	3.620675			

J = 2 → J' = 0			J = 2 → J' = 1			J = 2 → J' = 3		
E_{tot} (cm⁻¹)	E_{col} (cm⁻¹)	σ (Å²)	E_{tot} (cm⁻¹)	E_{col} (cm⁻¹)	σ (Å²)	E_{tot} (cm⁻¹)	E_{col} (cm⁻¹)	σ (Å²)
664.149690	500.000000	0.604134	664.149690	500.000000	3.762318			
673.582817	400.000000	0.608467	673.582817	400.000000	3.798043			
682.074845	600.000000	0.612452	682.074845	600.000000	3.829904			
710.374225	300.000000	0.626234	710.374225	300.000000	3.933424			
727.358282	700.000000	0.634793	727.358282	700.000000	3.993030			
764.149690	600.000000	0.653664	764.149690	600.000000	4.113433			
773.582817	500.000000	0.658495	773.582817	500.000000	4.142028			
782.074845	700.000000	0.662818	782.074845	700.000000	4.166877			
810.374225	400.000000	0.676903	810.374225	400.000000	4.243125			
827.358282	800.000000	0.685005	827.358282	800.000000	4.283717			
864.149690	700.000000	0.701260	864.149690	700.000000	4.357537			
873.582817	600.000000	0.705088	873.582817	600.000000	4.373286			
882.074845	800.000000	0.708401	882.074845	800.000000	4.386347			
910.374225	500.000000	0.718481	910.374225	500.000000	4.422283			
927.358282	900.000000	0.723786	927.358282	900.000000	4.438334			
964.149690	800.000000	0.733322	964.149690	800.000000	4.459450			
973.582817	700.000000	0.735345	973.582817	700.000000	4.461979			
982.074845	900.000000	0.737027	982.074845	900.000000	4.463299			
1010.374230	600.000000	0.741741	1010.374230	600.000000	4.461396			
1064.149690	900.000000	0.747742	1064.149690	900.000000	4.434169			
1073.582820	800.000000	0.748534	1073.582820	800.000000	4.426672			
1110.374230	700.000000	0.751377	1110.374230	700.000000	4.391320			
1173.582820	900.000000	0.757327	1173.582820	900.000000	4.314742			
1210.374230	800.000000	0.762651	1210.374230	800.000000	4.265478			
1310.374230	900.000000	0.788526	1310.374230	900.000000	4.131356			

Inelastic collisions cross-sections					
J = 2 → J’ = 4			J = 2 → J’ = 5		
E_{tot} (cm⁻¹)	E_{col} (cm⁻¹)	σ (Å²)	E_{tot} (cm⁻¹)	E_{col} (cm⁻¹)	σ (Å²)
273.582917	0.000100	0.000328	410.374325	0.000100	0.000030
273.583017	0.000200	0.000321	410.374425	0.000200	0.000031
273.583117	0.000300	0.000305	410.374525	0.000300	0.000030
273.583217	0.000400	0.000289	410.374625	0.000400	0.000029
273.583317	0.000500	0.000275	410.374725	0.000500	0.000028
273.583417	0.000600	0.000263	410.374825	0.000600	0.000027
273.583517	0.000700	0.000253	410.374925	0.000700	0.000026
273.583617	0.000800	0.000244	410.375025	0.000800	0.000025
273.583717	0.000900	0.000235	410.375225	0.001000	0.000023
273.583817	0.001000	0.000228	410.376225	0.002000	0.000018
273.584817	0.002000	0.000189	410.377225	0.003000	0.000016
273.585817	0.003000	0.000182	410.378225	0.004000	0.000015
273.586817	0.004000	0.000190	410.379225	0.005000	0.000015
273.587817	0.005000	0.000205	410.380225	0.006000	0.000016
273.588817	0.006000	0.000227	410.381225	0.007000	0.000017
273.589817	0.007000	0.000251	410.382225	0.008000	0.000019
273.590817	0.008000	0.000278	410.383225	0.009000	0.000020
273.591817	0.009000	0.000307	410.384225	0.010000	0.000022
273.592817	0.010000	0.000336	410.394225	0.020000	0.000039
273.602817	0.020000	0.000618	410.404225	0.030000	0.000054
273.612817	0.030000	0.000849	410.414225	0.040000	0.000067
273.622817	0.040000	0.001056	410.424225	0.050000	0.000081
273.632817	0.050000	0.001306	410.434225	0.060000	0.000494
273.642817	0.060000	0.001916	410.444225	0.070000	0.000120
273.652817	0.070000	0.011495	410.454225	0.080000	0.000152
273.662817	0.080000	0.006465	410.464225	0.090000	0.000221
273.672817	0.090000	0.004266	410.474225	0.100000	0.000373
273.682817	0.100000	0.005170	410.574225	0.200000	0.000389
273.782817	0.200000	0.006936	410.674225	0.300000	0.000463
273.882817	0.300000	0.007202	410.774225	0.400000	0.000281
273.982817	0.400000	0.004826	410.874225	0.500000	0.000325
274.082817	0.500000	0.006089	410.974225	0.600000	0.001092
274.182817	0.600000	0.005782	411.074225	0.700000	0.000369
274.282817	0.700000	0.006222	411.174225	0.800000	0.000403
274.382817	0.800000	0.006873	411.274225	0.900000	0.000560
274.482817	0.900000	0.007976	411.374225	1.000000	0.000634
274.582817	1.000000	0.012808	412.374225	2.000000	0.000538
275.582817	2.000000	0.009141	413.374225	3.000000	0.000659
276.582817	3.000000	0.011412	414.374225	4.000000	0.000862
277.582817	4.000000	0.015904	415.374225	5.000000	0.000797
278.582817	5.000000	0.013952	416.374225	6.000000	0.000844
279.582817	6.000000	0.014694	417.374225	7.000000	0.000925
280.582817	7.000000	0.016241	418.374225	8.000000	0.000976
281.582817	8.000000	0.017072	419.374225	9.000000	0.001021
282.074845	200.000000	0.017369	420.374225	10.000000	0.001076
282.582817	9.000000	0.017735	427.358282	400.000000	0.001523
283.582817	10.000000	0.018606	430.374225	20.000000	0.001747
293.582817	20.000000	0.028967	440.374225	30.000000	0.002648
303.582817	30.000000	0.041757	450.374225	40.000000	0.003821
313.582817	40.000000	0.057081	460.374225	50.000000	0.005301
323.582817	50.000000	0.074901	464.149690	300.000000	0.005946
327.358282	300.000000	0.082261	470.374225	60.000000	0.007119
333.582817	60.000000	0.095131	473.582817	200.000000	0.007780
343.582817	70.000000	0.117652	480.374225	70.000000	0.009307
353.582817	80.000000	0.142323	482.074845	400.000000	0.009718
363.582817	90.000000	0.168988	490.374225	80.000000	0.011890
364.149690	200.000000	0.170556	500.374225	90.000000	0.014894
373.582817	100.000000	0.197481	510.374225	100.000000	0.018338
382.074845	300.000000	0.222983	527.358282	500.000000	0.025243
410.374325	0.000100	0.314408	564.149690	400.000000	0.043935
410.374425	0.000200	0.314454	573.582817	300.000000	0.051136
410.374525	0.000300	0.314500	582.074845	500.000000	0.056772
410.374625	0.000400	0.314544	610.374225	200.000000	0.078477
410.374725	0.000500	0.314587	627.358282	600.000000	0.093118
410.374825	0.000600	0.314627	664.149690	500.000000	0.128507
410.374925	0.000700	0.314664	673.582817	400.000000	0.138300
410.375025	0.000800	0.314698	682.074845	600.000000	0.147339
410.375225	0.001000	0.314758	710.374225	300.000000	0.178811
410.376225	0.002000	0.314905	727.358282	700.000000	0.198549
410.377225	0.003000	0.314894	764.149690	600.000000	0.242912
410.378225	0.004000	0.314809	773.582817	500.000000	0.254523
410.379225	0.005000	0.314670	782.074845	700.000000	0.265042
410.380225	0.006000	0.314447	810.374225	400.000000	0.300330
410.381225	0.007000	0.314032	827.358282	800.000000	0.321548
410.382225	0.008000	0.313032	864.149690	700.000000	0.367172
410.383225	0.009000	0.312624	873.582817	600.000000	0.378734
410.384225	0.010000	0.316233	882.074845	800.000000	0.389080

J = 2 → J' = 4			J = 2 → J' = 5		
E_{tot} (cm⁻¹)	E_{col} (cm⁻¹)	σ (Å²)	E_{tot} (cm⁻¹)	E_{col} (cm⁻¹)	σ (Å²)
410.394225	0.020000	0.315235	910.374225	500.000000	0.423080
410.404225	0.030000	0.315039	927.358282	900.000000	0.443100
410.414225	0.040000	0.314853	964.149690	800.000000	0.485456
410.424225	0.050000	0.314499	973.582817	700.000000	0.496104
410.434225	0.060000	0.312296	982.074845	900.000000	0.505602
410.444225	0.070000	0.317991	1010.374230	600.000000	0.536934
410.454225	0.080000	0.316154	1064.149690	900.000000	0.595214
410.464225	0.090000	0.315725	1073.582820	800.000000	0.605350
410.474225	0.100000	0.315423	1110.374230	700.000000	0.644875
410.574225	0.200000	0.315703	1173.582820	900.000000	0.713663
410.674225	0.300000	0.315856	1210.374230	800.000000	0.754843
410.774225	0.400000	0.316287	1310.374230	900.000000	0.874286
410.874225	0.500000	0.316631			
410.974225	0.600000	0.316172			
411.074225	0.700000	0.317955			
411.174225	0.800000	0.317782			
411.274225	0.900000	0.318063			
411.374225	1.000000	0.318173			
412.374225	2.000000	0.321773			
413.374225	3.000000	0.325086			
414.374225	4.000000	0.328469			
415.374225	5.000000	0.331967			
416.374225	6.000000	0.335408			
417.374225	7.000000	0.338842			
418.374225	8.000000	0.342325			
419.374225	9.000000	0.345811			
420.374225	10.000000	0.349298			
427.358282	400.000000	0.373876			
430.374225	20.000000	0.384598			
440.374225	30.000000	0.420535			
450.374225	40.000000	0.456960			
460.374225	50.000000	0.493731			
464.149690	300.000000	0.507676			
470.374225	60.000000	0.530717			
473.582817	200.000000	0.542611			
480.374225	70.000000	0.567800			
482.074845	400.000000	0.574107			
490.374225	80.000000	0.604864			
500.374225	90.000000	0.641807			
510.374225	100.000000	0.678534			
527.358282	500.000000	0.740179			
564.149690	400.000000	0.869975			
573.582817	300.000000	0.900747			
582.074845	500.000000	0.928814			
610.374225	200.000000	1.018301			
627.358282	600.000000	1.068879			
664.149690	500.000000	1.169836			
673.582817	400.000000	1.193778			
682.074845	600.000000	1.214650			
710.374225	300.000000	1.279606			
727.358282	700.000000	1.315291			
764.149690	600.000000	1.384741			
773.582817	500.000000	1.400992			
782.074845	700.000000	1.415111			
810.374225	400.000000	1.459120			
827.358282	800.000000	1.483612			
864.149690	700.000000	1.533080			
873.582817	600.000000	1.545207			
882.074845	800.000000	1.555999			
910.374225	500.000000	1.591503			
927.358282	900.000000	1.612785			
964.149690	800.000000	1.659919			
973.582817	700.000000	1.672389			
982.074845	900.000000	1.683808			
1010.374230	600.000000	1.723138			
1064.149690	900.000000	1.804521			
1073.582820	800.000000	1.819725			
1110.374230	700.000000	1.881491			
1173.582820	900.000000	1.994854			
1210.374230	800.000000	2.063151			
1310.374230	900.000000	2.245781			

Inelastic collisions cross-sections								
J = 3 → J’ = 0			J = 3 → J’ = 1			J = 3 → J’ = 2		
E_{tot} (cm⁻¹)	E_{col} (cm⁻¹)	σ (Å²)	E_{tot} (cm⁻¹)	E_{col} (cm⁻¹)	σ (Å²)	E_{tot} (cm⁻¹)	E_{col} (cm⁻¹)	σ (Å²)
164.149790	0.000100	182.657047	164.149790	0.000100	560.984944	164.149790	0.000100	1536.110180
164.149890	0.000200	96.466483	164.149890	0.000200	304.399611	164.149890	0.000200	780.881852
164.149990	0.000300	71.858360	164.149990	0.000300	233.375765	164.149990	0.000300	550.718073
164.150090	0.000400	62.528658	164.150090	0.000400	208.169391	164.150090	0.000400	452.161522
164.150190	0.000500	59.002734	164.150190	0.000500	200.270131	164.150190	0.000500	404.330808
164.150290	0.000600	58.175415	164.150290	0.000600	200.339390	164.150290	0.000600	380.600543
164.150390	0.000700	58.751204	164.150390	0.000700	204.446360	164.150390	0.000700	369.828809
164.150490	0.000800	60.083147	164.150490	0.000800	210.649984	164.150490	0.000800	366.493106
164.150590	0.000900	61.806506	164.150590	0.000900	217.868429	164.150590	0.000900	367.529986
164.150690	0.001000	63.718479	164.150690	0.001000	225.461898	164.150690	0.001000	371.165386
164.151690	0.002000	77.033130	164.151690	0.002000	274.104818	164.151690	0.002000	416.100462
164.152690	0.003000	72.166014	164.152690	0.003000	254.630750	164.152690	0.003000	391.455281
164.159690	0.010000	15.871925	164.159690	0.010000	52.474994	164.159690	0.010000	117.040418
164.169690	0.020000	5.797951	164.169690	0.020000	18.137800	164.169690	0.020000	59.989471
164.179690	0.030000	3.817654	164.179690	0.030000	11.698558	164.179690	0.030000	45.051357
164.189690	0.040000	3.044007	164.189690	0.040000	9.486811	164.189690	0.040000	39.531420
164.199690	0.050000	2.664941	164.199690	0.050000	17.720543	164.199690	0.050000	203.198952
164.209690	0.060000	2.500171	164.209690	0.060000	7.912817	164.209690	0.060000	46.206984
164.219690	0.070000	2.517512	164.219690	0.070000	7.650905	164.219690	0.070000	39.270811
164.229690	0.080000	2.759330	164.229690	0.080000	8.285232	164.229690	0.080000	40.030420
164.239690	0.090000	3.370697	164.239690	0.090000	10.038307	164.239690	0.090000	46.005464
164.249690	0.100000	4.502104	164.249690	0.100000	13.344500	164.249690	0.100000	58.571527
164.349690	0.200000	3.419830	164.349690	0.200000	10.470681	164.349690	0.200000	64.401245
164.449690	0.300000	1.689100	164.449690	0.300000	5.290555	164.449690	0.300000	65.114952
164.549690	0.400000	0.993593	164.549690	0.400000	2.993119	164.549690	0.400000	16.243465
164.649690	0.500000	0.776539	164.649690	0.500000	2.365591	164.649690	0.500000	12.499030
164.749690	0.600000	1.160853	164.749690	0.600000	3.522742	164.749690	0.600000	12.932947
164.849690	0.700000	0.831909	164.849690	0.700000	2.469310	164.849690	0.700000	10.612767
164.949690	0.800000	0.758978	164.949690	0.800000	2.254963	164.949690	0.800000	9.723906
165.049690	0.900000	0.779745	165.049690	0.900000	2.434383	165.049690	0.900000	12.036446
165.149690	1.000000	1.003983	165.149690	1.000000	2.970141	165.149690	1.000000	14.377501
166.149690	2.000000	0.398070	166.149690	2.000000	1.176815	166.149690	2.000000	6.057824
167.149690	3.000000	0.349501	167.149690	3.000000	1.027408	167.149690	3.000000	4.595054
168.149690	4.000000	0.428331	168.149690	4.000000	1.229992	168.149690	4.000000	5.022037
169.149690	5.000000	0.261986	169.149690	5.000000	0.768474	169.149690	5.000000	3.547502
170.149690	6.000000	0.229879	170.149690	6.000000	0.672392	170.149690	6.000000	3.004558
171.149690	7.000000	0.221370	171.149690	7.000000	0.646325	171.149690	7.000000	2.822179
172.074845	90.000000	0.205044	172.074845	90.000000	0.597461	172.074845	90.000000	2.636202
172.149690	8.000000	0.203483	172.149690	8.000000	0.592857	172.149690	8.000000	2.616759
173.149690	9.000000	0.186916	173.149690	9.000000	0.543674	173.149690	9.000000	2.389432
174.149690	10.000000	0.176384	174.149690	10.000000	0.512163	174.149690	10.000000	2.232594
182.074845	100.000000	0.141530	182.074845	100.000000	0.403371	182.074845	100.000000	1.674642
184.149690	20.000000	0.138223	184.149690	20.000000	0.391692	184.149690	20.000000	1.605621
194.149690	30.000000	0.134607	194.149690	30.000000	0.369780	194.149690	30.000000	1.430035
204.149690	40.000000	0.140659	204.149690	40.000000	0.373396	204.149690	40.000000	1.372535
214.149690	50.000000	0.151089	214.149690	50.000000	0.387116	214.149690	50.000000	1.363435
224.149690	60.000000	0.163993	224.149690	60.000000	0.405729	224.149690	60.000000	1.378903
227.358282	200.000000	0.168493	227.358282	200.000000	0.412334	227.358282	200.000000	1.387055
234.149690	70.000000	0.178464	234.149690	70.000000	0.426943	234.149690	70.000000	1.407999
244.149690	80.000000	0.193950	244.149690	80.000000	0.449520	244.149690	80.000000	1.445023
254.149690	90.000000	0.210090	254.149690	90.000000	0.472563	254.149690	90.000000	1.485963
264.149690	100.000000	0.226560	264.149690	100.000000	0.496487	264.149690	100.000000	1.532655
273.582917	0.000100	0.241710	273.582917	0.000100	0.518783	273.582917	0.000100	1.583891
273.583017	0.000200	0.241719	273.583017	0.000200	0.518767	273.583017	0.000200	1.583652
273.583117	0.000300	0.241729	273.583117	0.000300	0.518747	273.583117	0.000300	1.583394
273.583217	0.000400	0.241740	273.583217	0.000400	0.518724	273.583217	0.000400	1.583130
273.583317	0.000500	0.241749	273.583317	0.000500	0.518700	273.583317	0.000500	1.582866
273.583417	0.000600	0.241759	273.583417	0.000600	0.518675	273.583417	0.000600	1.582604
273.583517	0.000700	0.241768	273.583517	0.000700	0.518649	273.583517	0.000700	1.582344
273.583617	0.000800	0.241777	273.583617	0.000800	0.518622	273.583617	0.000800	1.582089
273.583717	0.000900	0.241785	273.583717	0.000900	0.518595	273.583717	0.000900	1.581839
273.583817	0.001000	0.241794	273.583817	0.001000	0.518568	273.583817	0.001000	1.581595
273.584817	0.002000	0.241863	273.584817	0.002000	0.518330	273.584817	0.002000	1.579801
273.585817	0.003000	0.241915	273.585817	0.003000	0.519270	273.585817	0.003000	1.589240
273.586817	0.004000	0.241955	273.586817	0.004000	0.526027	273.586817	0.004000	1.642549
273.587817	0.005000	0.241988	273.587817	0.005000	0.520227	273.587817	0.005000	1.593862
273.588817	0.006000	0.242014	273.588817	0.006000	0.519172	273.588817	0.006000	1.585535
273.589817	0.007000	0.242035	273.589817	0.007000	0.518729	273.589817	0.007000	1.582189
273.590817	0.008000	0.242053	273.590817	0.008000	0.518460	273.590817	0.008000	1.580251
273.591817	0.009000	0.242068	273.591817	0.009000	0.518266	273.591817	0.009000	1.578911
273.592817	0.010000	0.242081	273.592817	0.010000	0.518112	273.592817	0.010000	1.577892
273.602817	0.020000	0.242149	273.602817	0.020000	0.517240	273.602817	0.020000	1.572892
273.612817	0.030000	0.242175	273.612817	0.030000	0.516661	273.612817	0.030000	1.570166
273.622817	0.040000	0.242186	273.622817	0.040000	0.516052	273.622817	0.040000	1.567583
273.632817	0.050000	0.242177	273.632817	0.050000	0.515186	273.632817	0.050000	1.564210
273.642817	0.060000	0.242098	273.642817	0.060000	0.513475	273.642817	0.060000	1.558324
273.652817	0.070000	0.240709	273.652817	0.070000	0.506257	273.652817	0.070000	1.548636

J = 3 → J’ = 0			J = 3 → J’ = 1			J = 3 → J’ = 2		
E_{tot} (cm⁻¹)	E_{col} (cm⁻¹)	σ (Å²)	E_{tot} (cm⁻¹)	E_{col} (cm⁻¹)	σ (Å²)	E_{tot} (cm⁻¹)	E_{col} (cm⁻¹)	σ (Å²)
273.662817	0.080000	0.239164	273.662817	0.080000	0.545422	273.662817	0.080000	1.747686
273.672817	0.090000	0.242031	273.672817	0.090000	0.523339	273.672817	0.090000	1.601958
273.682817	0.100000	0.242099	273.682817	0.100000	0.520194	273.682817	0.100000	1.586940
273.782817	0.200000	0.242157	273.782817	0.200000	0.517666	273.782817	0.200000	1.576137
273.882817	0.300000	0.242278	273.882817	0.300000	0.517458	273.882817	0.300000	1.575565
273.982817	0.400000	0.242607	273.982817	0.400000	0.517714	273.982817	0.400000	1.574507
274.082817	0.500000	0.242409	274.082817	0.500000	0.519861	274.082817	0.500000	1.587073
274.182817	0.600000	0.242749	274.182817	0.600000	0.517425	274.182817	0.600000	1.574118
274.282817	0.700000	0.243358	274.282817	0.700000	0.531350	274.282817	0.700000	1.627254
274.382817	0.800000	0.243251	274.382817	0.800000	0.519703	274.382817	0.800000	1.581085
274.482817	0.900000	0.243311	274.482817	0.900000	0.518887	274.482817	0.900000	1.577853
274.582817	1.000000	0.243148	274.582817	1.000000	0.517695	274.582817	1.000000	1.575428
275.582817	2.000000	0.245024	275.582817	2.000000	0.521472	275.582817	2.000000	1.584307
276.582817	3.000000	0.246563	276.582817	3.000000	0.523202	276.582817	3.000000	1.587706
277.582817	4.000000	0.248079	277.582817	4.000000	0.525363	277.582817	4.000000	1.593053
278.582817	5.000000	0.249772	278.582817	5.000000	0.527676	278.582817	5.000000	1.597626
279.582817	6.000000	0.251388	279.582817	6.000000	0.529814	279.582817	6.000000	1.602074
280.582817	7.000000	0.252978	280.582817	7.000000	0.531916	280.582817	7.000000	1.606641
281.582817	8.000000	0.254598	281.582817	8.000000	0.534154	281.582817	8.000000	1.611526
282.074845	200.000000	0.255395	282.074845	200.000000	0.535246	282.074845	200.000000	1.613879
282.582817	9.000000	0.256217	282.582817	9.000000	0.536358	282.582817	9.000000	1.616283
283.582817	10.000000	0.257825	283.582817	10.000000	0.538537	283.582817	10.000000	1.621014
293.582817	20.000000	0.273757	293.582817	20.000000	0.559954	293.582817	20.000000	1.668788
303.582817	30.000000	0.289326	303.582817	30.000000	0.580615	303.582817	30.000000	1.716966
313.582817	40.000000	0.304456	313.582817	40.000000	0.600454	313.582817	40.000000	1.765213
323.582817	50.000000	0.319101	323.582817	50.000000	0.619441	323.582817	50.000000	1.813452
327.358282	300.000000	0.324499	327.358282	300.000000	0.626383	327.358282	300.000000	1.831601
333.582817	60.000000	0.333232	333.582817	60.000000	0.637559	333.582817	60.000000	1.861551
343.582817	70.000000	0.346836	343.582817	70.000000	0.654819	343.582817	70.000000	1.909498
353.582817	80.000000	0.359912	353.582817	80.000000	0.671242	353.582817	80.000000	1.957220
363.582817	90.000000	0.372465	363.582817	90.000000	0.686862	363.582817	90.000000	2.004706
364.149690	200.000000	0.373159	364.149690	200.000000	0.687724	364.149690	200.000000	2.007401
373.582817	100.000000	0.384509	373.582817	100.000000	0.701724	373.582817	100.000000	2.051964
382.074845	300.000000	0.394352	382.074845	300.000000	0.713769	382.074845	300.000000	2.091874
410.374325	0.000100	0.424826	410.374325	0.000100	0.750120	410.374325	0.000100	2.221748
410.374425	0.000200	0.424808	410.374425	0.000200	0.750096	410.374425	0.000200	2.221781
410.374525	0.000300	0.424793	410.374525	0.000300	0.750079	410.374525	0.000300	2.221822
410.374625	0.000400	0.424780	410.374625	0.000400	0.750068	410.374625	0.000400	2.221867
410.374725	0.000500	0.424769	410.374725	0.000500	0.750061	410.374725	0.000500	2.221916
410.374825	0.000600	0.424759	410.374825	0.000600	0.750058	410.374825	0.000600	2.221967
410.374925	0.000700	0.424752	410.374925	0.000700	0.750060	410.374925	0.000700	2.222021
410.375025	0.000800	0.424746	410.375025	0.000800	0.750064	410.375025	0.000800	2.222076
410.375225	0.001000	0.424739	410.375225	0.001000	0.750083	410.375225	0.001000	2.222186
410.376225	0.002000	0.424764	410.376225	0.002000	0.750261	410.376225	0.002000	2.222644
410.377225	0.003000	0.424830	410.377225	0.003000	0.750457	410.377225	0.003000	2.222902
410.378225	0.004000	0.424904	410.378225	0.004000	0.750631	410.378225	0.004000	2.223021
410.379225	0.005000	0.424989	410.379225	0.005000	0.750797	410.379225	0.005000	2.223042
410.380225	0.006000	0.425099	410.380225	0.006000	0.750981	410.380225	0.006000	2.222954
410.381225	0.007000	0.425270	410.381225	0.007000	0.751219	410.381225	0.007000	2.222610
410.382225	0.008000	0.425572	410.382225	0.008000	0.751439	410.382225	0.008000	2.221124
410.383225	0.009000	0.424709	410.383225	0.009000	0.748345	410.383225	0.009000	2.214404
410.384225	0.010000	0.423826	410.384225	0.010000	0.748201	410.384225	0.010000	2.220984
410.394225	0.020000	0.424770	410.394225	0.020000	0.750472	410.394225	0.020000	2.223453
410.404225	0.030000	0.424883	410.404225	0.030000	0.750663	410.404225	0.030000	2.223473
410.414225	0.040000	0.424976	410.414225	0.040000	0.750801	410.414225	0.040000	2.223447
410.424225	0.050000	0.425118	410.424225	0.050000	0.750993	410.424225	0.050000	2.223320
410.434225	0.060000	0.425561	410.434225	0.060000	0.751207	410.434225	0.060000	2.221739
410.444225	0.070000	0.423622	410.444225	0.070000	0.748751	410.444225	0.070000	2.223806
410.454225	0.080000	0.424541	410.454225	0.080000	0.750191	410.454225	0.080000	2.223971
410.464225	0.090000	0.424717	410.464225	0.090000	0.750435	410.464225	0.090000	2.223882
410.474225	0.100000	0.424798	410.474225	0.100000	0.750520	410.474225	0.100000	2.223753
410.574225	0.200000	0.424899	410.574225	0.200000	0.750614	410.574225	0.200000	2.224168
410.674225	0.300000	0.425043	410.674225	0.300000	0.750784	410.674225	0.300000	2.224520
410.774225	0.400000	0.425195	410.774225	0.400000	0.751033	410.774225	0.400000	2.225138
410.874225	0.500000	0.425256	410.874225	0.500000	0.751038	410.874225	0.500000	2.225502
410.974225	0.600000	0.425486	410.974225	0.600000	0.750620	410.974225	0.600000	2.223739
411.074225	0.700000	0.425250	411.074225	0.700000	0.751060	411.074225	0.700000	2.226711
411.174225	0.800000	0.425516	411.174225	0.800000	0.751385	411.174225	0.800000	2.226969
411.274225	0.900000	0.425552	411.274225	0.900000	0.751338	411.274225	0.900000	2.227271
411.374225	1.000000	0.425710	411.374225	1.000000	0.751546	411.374225	1.000000	2.227662
412.374225	2.000000	0.426705	412.374225	2.000000	0.752754	412.374225	2.000000	2.232348
413.374225	3.000000	0.427708	413.374225	3.000000	0.753933	413.374225	3.000000	2.236853
414.374225	4.000000	0.428653	414.374225	4.000000	0.755021	414.374225	4.000000	2.241340
415.374225	5.000000	0.429669	415.374225	5.000000	0.756248	415.374225	5.000000	2.245971
416.374225	6.000000	0.430657	416.374225	6.000000	0.757422	416.374225	6.000000	2.250534
417.374225	7.000000	0.431632	417.374225	7.000000	0.758572	417.374225	7.000000	2.255087
418.374225	8.000000	0.432603	418.374225	8.000000	0.759722	418.374225	8.000000	2.259634
419.374225	9.000000	0.433576	419.374225	9.000000	0.760874	419.374225	9.000000	2.264200

J = 3 → J' = 0			J = 3 → J' = 1			J = 3 → J' = 2		
E_{tot} (cm^{-1})	E_{col} (cm^{-1})	σ (Å²)	E_{tot} (cm^{-1})	E_{col} (cm^{-1})	σ (Å²)	E_{tot} (cm^{-1})	E_{col} (cm^{-1})	σ (Å²)
420.374225	10.000000	0.434540	420.374225	10.000000	0.762017	420.374225	10.000000	2.268742
427.358282	400.000000	0.441163	427.358282	400.000000	0.769831	427.358282	400.000000	2.300439
430.374225	20.000000	0.443960	430.374225	20.000000	0.773119	430.374225	20.000000	2.314068
440.374225	30.000000	0.452962	440.374225	30.000000	0.783661	440.374225	30.000000	2.359018
450.374225	40.000000	0.461557	450.374225	40.000000	0.793667	450.374225	40.000000	2.403597
460.374225	50.000000	0.469758	460.374225	50.000000	0.803161	460.374225	50.000000	2.447789
464.149690	300.000000	0.472755	464.149690	300.000000	0.806618	464.149690	300.000000	2.464382
470.374225	60.000000	0.477577	470.374225	60.000000	0.812169	470.374225	60.000000	2.491621
473.582817	200.000000	0.480006	473.582817	200.000000	0.814960	473.582817	200.000000	2.505605
480.374225	70.000000	0.485023	480.374225	70.000000	0.820710	480.374225	70.000000	2.535079
482.074845	400.000000	0.486253	482.074845	400.000000	0.822119	482.074845	400.000000	2.542433
490.374225	80.000000	0.492109	490.374225	80.000000	0.828810	490.374225	80.000000	2.578179
500.374225	90.000000	0.498845	500.374225	90.000000	0.836487	500.374225	90.000000	2.620927
510.374225	100.000000	0.505243	510.374225	100.000000	0.843760	510.374225	100.000000	2.663336
527.358282	500.000000	0.515367	527.358282	500.000000	0.855244	527.358282	500.000000	2.734624
564.149690	400.000000	0.534382	564.149690	400.000000	0.877127	564.149690	400.000000	2.887153
573.582817	300.000000	0.538629	573.582817	300.000000	0.881567	573.582817	300.000000	2.924231
582.074845	500.000000	0.542300	582.074845	500.000000	0.885773	582.074845	500.000000	2.958562
610.374225	200.000000	0.553370	610.374225	200.000000	0.898338	610.374225	200.000000	3.071209
627.358282	600.000000	0.559268	627.358282	600.000000	0.905010	627.358282	600.000000	3.137741
664.149690	500.000000	0.570546	664.149690	500.000000	0.917573	664.149690	500.000000	3.278683
673.582817	400.000000	0.573177	673.582817	400.000000	0.920429	673.582817	400.000000	3.314006
682.074845	600.000000	0.575477	682.074845	600.000000	0.922888	682.074845	600.000000	3.345476
710.374225	300.000000	0.582773	710.374225	300.000000	0.930395	710.374225	300.000000	3.447800
727.358282	700.000000	0.586968	727.358282	700.000000	0.934464	727.358282	700.000000	3.507047
764.149690	600.000000	0.595866	764.149690	600.000000	0.942445	764.149690	600.000000	3.628691
773.582817	500.000000	0.598139	773.582817	500.000000	0.944355	773.582817	500.000000	3.658235
782.074845	700.000000	0.600193	782.074845	700.000000	0.946048	782.074845	700.000000	3.684201
810.374225	400.000000	0.607103	810.374225	400.000000	0.951592	810.374225	400.000000	3.766249
827.358282	800.000000	0.611312	827.358282	800.000000	0.954938	827.358282	800.000000	3.812055
864.149690	700.000000	0.620581	864.149690	700.000000	0.962604	864.149690	700.000000	3.902262
873.582817	600.000000	0.622985	873.582817	600.000000	0.964725	873.582817	600.000000	3.923422
882.074845	800.000000	0.625155	882.074845	800.000000	0.966705	882.074845	800.000000	3.941800
910.374225	500.000000	0.632414	910.374225	500.000000	0.973901	910.374225	500.000000	3.998581
927.358282	900.000000	0.636775	927.358282	900.000000	0.978741	927.358282	900.000000	4.029492
964.149690	800.000000	0.646196	964.149690	800.000000	0.990867	964.149690	800.000000	4.088794
973.582817	700.000000	0.648603	973.582817	700.000000	0.994385	973.582817	700.000000	4.102382
982.074845	900.000000	0.650768	982.074845	900.000000	0.997703	982.074845	900.000000	4.114072
1010.374230	600.000000	0.657963	1010.374230	600.000000	1.009916	1010.374230	600.000000	4.149340
1064.149690	900.000000	0.671637	1064.149690	900.000000	1.038430	1064.149690	900.000000	4.201248
1073.582820	800.000000	0.674043	1073.582820	800.000000	1.044201	1073.582820	800.000000	4.208372
1110.374230	700.000000	0.683447	1110.374230	700.000000	1.068974	1110.374230	700.000000	4.230816
1173.582820	900.000000	0.699413	1173.582820	900.000000	1.119662	1173.582820	900.000000	4.251899
1210.374230	800.000000	0.708259	1210.374230	800.000000	1.153340	1210.374230	800.000000	4.256364
1310.374230	900.000000	0.728376	1310.374230	900.000000	1.255448	1310.374230	900.000000	4.253131

Inelastic collisions cross-sections					
J = 3 → J' = 4			J = 3 → J' = 5		
E_{tot} (cm⁻¹)	E_{col} (cm⁻¹)	σ (Å²)	E_{tot} (cm⁻¹)	E_{col} (cm⁻¹)	σ (Å²)
273.582917	0.000100	0.002624	410.374325	0.000100	0.000132
273.583017	0.000200	0.002616	410.374425	0.000200	0.000135
273.583117	0.000300	0.002531	410.374525	0.000300	0.000132
273.583217	0.000400	0.002448	410.374625	0.000400	0.000127
273.583317	0.000500	0.002376	410.374725	0.000500	0.000123
273.583417	0.000600	0.002314	410.374825	0.000600	0.000118
273.583517	0.000700	0.002263	410.374925	0.000700	0.000114
273.583617	0.000800	0.002219	410.375025	0.000800	0.000110
273.583717	0.000900	0.002183	410.375225	0.001000	0.000103
273.583817	0.001000	0.002152	410.376225	0.002000	0.000083
273.584817	0.002000	0.002043	410.377225	0.003000	0.000076
273.585817	0.003000	0.002109	410.378225	0.004000	0.000076
273.586817	0.004000	0.002250	410.379225	0.005000	0.000079
273.587817	0.005000	0.002428	410.380225	0.006000	0.000085
273.588817	0.006000	0.002627	410.381225	0.007000	0.000093
273.589817	0.007000	0.002839	410.382225	0.008000	0.000102
273.590817	0.008000	0.003057	410.383225	0.009000	0.000111
273.591817	0.009000	0.003279	410.384225	0.010000	0.000121
273.592817	0.010000	0.003501	410.394225	0.020000	0.000217
273.602817	0.020000	0.005484	410.404225	0.030000	0.000296
273.612817	0.030000	0.006956	410.414225	0.040000	0.000366
273.622817	0.040000	0.008145	410.424225	0.050000	0.000457
273.632817	0.050000	0.009400	410.434225	0.060000	0.009611
273.642817	0.060000	0.012132	410.444225	0.070000	0.000715
273.652817	0.070000	0.056133	410.454225	0.080000	0.000834
273.662817	0.080000	0.035922	410.464225	0.090000	0.001164
273.672817	0.090000	0.025224	410.474225	0.100000	0.001883
273.682817	0.100000	0.030183	410.574225	0.200000	0.002239
273.782817	0.200000	0.071665	410.674225	0.300000	0.002499
273.882817	0.300000	0.056344	410.774225	0.400000	0.001608
273.982817	0.400000	0.046534	410.874225	0.500000	0.001746
274.082817	0.500000	0.043911	410.974225	0.600000	0.020058
274.182817	0.600000	0.042737	411.074225	0.700000	0.002012
274.282817	0.700000	0.044522	411.174225	0.800000	0.002205
274.382817	0.800000	0.047938	411.274225	0.900000	0.002993
274.482817	0.900000	0.052872	411.374225	1.000000	0.003673
274.582817	1.000000	0.075251	412.374225	2.000000	0.002993
275.582817	2.000000	0.075457	413.374225	3.000000	0.003663
276.582817	3.000000	0.082089	414.374225	4.000000	0.004883
277.582817	4.000000	0.110217	415.374225	5.000000	0.004472
278.582817	5.000000	0.104695	416.374225	6.000000	0.004706
279.582817	6.000000	0.106720	417.374225	7.000000	0.005167
280.582817	7.000000	0.116206	418.374225	8.000000	0.005447
281.582817	8.000000	0.123323	419.374225	9.000000	0.005678
282.074845	200.000000	0.125287	420.374225	10.000000	0.005964
282.582817	9.000000	0.127482	427.358282	400.000000	0.008285
283.582817	10.000000	0.132780	430.374225	20.000000	0.009421
293.582817	20.000000	0.195416	440.374225	30.000000	0.013841
303.582817	30.000000	0.263956	450.374225	40.000000	0.019332
313.582817	40.000000	0.336840	460.374225	50.000000	0.025965
323.582817	50.000000	0.412477	464.149690	300.000000	0.028777
327.358282	300.000000	0.441518	470.374225	60.000000	0.033792
333.582817	60.000000	0.489816	473.582817	200.000000	0.036564
343.582817	70.000000	0.568221	480.374225	70.000000	0.042854
353.582817	80.000000	0.647277	482.074845	400.000000	0.044520
363.582817	90.000000	0.726725	490.374225	80.000000	0.053177
364.149690	200.000000	0.731234	500.374225	90.000000	0.064773
373.582817	100.000000	0.806297	510.374225	100.000000	0.077643
382.074845	300.000000	0.873760	527.358282	500.000000	0.102376
410.374325	0.000100	1.098355	564.149690	400.000000	0.166412
410.374425	0.000200	1.098519	573.582817	300.000000	0.186925
410.374525	0.000300	1.098646	582.074845	500.000000	0.204498
410.374625	0.000400	1.098742	610.374225	200.000000	0.268515
410.374725	0.000500	1.098808	627.358282	600.000000	0.309762
410.374825	0.000600	1.098848	664.149690	500.000000	0.404547
410.374925	0.000700	1.098863	673.582817	400.000000	0.429746
410.375025	0.000800	1.098855	682.074845	600.000000	0.452657
410.375225	0.001000	1.098782	710.374225	300.000000	0.530087
410.376225	0.002000	1.097811	727.358282	700.000000	0.576980
410.377225	0.003000	1.096655	764.149690	600.000000	0.678331
410.378225	0.004000	1.095606	773.582817	500.000000	0.704053
410.379225	0.005000	1.094608	782.074845	700.000000	0.727071
410.380225	0.006000	1.093538	810.374225	400.000000	0.802509
410.381225	0.007000	1.092321	827.358282	800.000000	0.846641
410.382225	0.008000	1.092207	864.149690	700.000000	0.938640
410.383225	0.009000	1.118454	873.582817	600.000000	0.961351
410.384225	0.010000	1.113597	882.074845	800.000000	0.981472

J = 3 → J' = 4			J = 3 → J' = 5		
E_{tot} (cm⁻¹)	E_{col} (cm⁻¹)	σ (Å²)	E_{tot} (cm⁻¹)	E_{col} (cm⁻¹)	σ (Å²)
410.394225	0.020000	1.096468	910.374225	500.000000	1.046246
410.404225	0.030000	1.095077	927.358282	900.000000	1.083416
410.414225	0.040000	1.093912	964.149690	800.000000	1.159627
410.424225	0.050000	1.091952	973.582817	700.000000	1.178256
410.434225	0.060000	1.093454	982.074845	900.000000	1.194726
410.444225	0.070000	1.115210	1010.374230	600.000000	1.247697
410.454225	0.080000	1.101113	1064.149690	900.000000	1.341493
410.464225	0.090000	1.098555	1073.582820	800.000000	1.357212
410.474225	0.100000	1.097208	1110.374230	700.000000	1.417025
410.574225	0.200000	1.097750	1173.582820	900.000000	1.516322
410.674225	0.300000	1.098051	1210.374230	800.000000	1.573271
410.774225	0.400000	1.098413	1310.374230	900.000000	1.727664
410.874225	0.500000	1.099819			
410.974225	0.600000	1.126378			
411.074225	0.700000	1.104068			
411.174225	0.800000	1.102353			
411.274225	0.900000	1.103768			
411.374225	1.000000	1.103248			
412.374225	2.000000	1.111772			
413.374225	3.000000	1.119112			
414.374225	4.000000	1.126998			
415.374225	5.000000	1.134733			
416.374225	6.000000	1.142365			
417.374225	7.000000	1.150019			
418.374225	8.000000	1.157777			
419.374225	9.000000	1.165478			
420.374225	10.000000	1.173158			
427.358282	400.000000	1.226602			
430.374225	20.000000	1.249546			
440.374225	30.000000	1.324990			
450.374225	40.000000	1.399427			
460.374225	50.000000	1.472814			
464.149690	300.000000	1.500242			
470.374225	60.000000	1.545125			
473.582817	200.000000	1.568097			
480.374225	70.000000	1.616349			
482.074845	400.000000	1.628353			
490.374225	80.000000	1.686488			
500.374225	90.000000	1.755552			
510.374225	100.000000	1.823561			
527.358282	500.000000	1.936737			
564.149690	400.000000	2.172108			
573.582817	300.000000	2.231286			
582.074845	500.000000	2.283219			
610.374225	200.000000	2.452908			
627.358282	600.000000	2.552052			
664.149690	500.000000	2.760573			
673.582817	400.000000	2.812745			
682.074845	600.000000	2.859278			
710.374225	300.000000	3.011419			
727.358282	700.000000	3.100559			
764.149690	600.000000	3.287823			
773.582817	500.000000	3.334501			
782.074845	700.000000	3.375994			
810.374225	400.000000	3.510551			
827.358282	800.000000	3.588337			
864.149690	700.000000	3.748292			
873.582817	600.000000	3.787276			
882.074845	800.000000	3.821626			
910.374225	500.000000	3.930842			
927.358282	900.000000	3.992421			
964.149690	800.000000	4.115559			
973.582817	700.000000	4.144910			
982.074845	900.000000	4.170606			
1010.374230	600.000000	4.251166			
1064.149690	900.000000	4.385040			
1073.582820	800.000000	4.406184			
1110.374230	700.000000	4.482779			
1173.582820	900.000000	4.595352			
1210.374230	800.000000	4.651590			
1310.374230	900.000000	4.777572			

Inelastic collisions cross-sections								
J = 4 → J' = 0			J = 4 → J' = 1			J = 4 → J' = 2		
E_{tot} (cm⁻¹)	E_{col} (cm⁻¹)	σ (Å²)	E_{tot} (cm⁻¹)	E_{col} (cm⁻¹)	σ (Å²)	E_{tot} (cm⁻¹)	E_{col} (cm⁻¹)	σ (Å²)
273.582917	0.000100	19.909475	273.582917	0.000100	94.681068	273.582917	0.000100	349.346564
273.583017	0.000200	9.736138	273.583017	0.000200	46.292782	273.583017	0.000200	170.821957
273.583117	0.000300	6.159565	273.583117	0.000300	29.287263	273.583117	0.000300	108.105114
273.583217	0.000400	4.379846	273.583217	0.000400	20.829001	273.583217	0.000400	76.925860
273.583317	0.000500	3.332719	273.583317	0.000500	15.855067	273.583317	0.000500	58.601339
273.583417	0.000600	2.651477	273.583417	0.000600	12.621067	273.583417	0.000600	46.694914
273.583517	0.000700	2.177514	273.583517	0.000700	10.372582	273.583517	0.000700	38.423010
273.583617	0.000800	1.831236	273.583617	0.000800	8.731136	273.583617	0.000800	32.388964
273.583717	0.000900	1.568932	273.583717	0.000900	7.488559	273.583717	0.000900	27.825900
273.583817	0.001000	1.364409	273.583817	0.001000	6.520592	273.583817	0.001000	24.274385
273.584817	0.002000	0.538646	273.584817	0.002000	2.627667	273.584817	0.002000	10.054096
273.585817	0.003000	0.324357	273.585817	0.003000	1.627590	273.585817	0.003000	6.442504
273.586817	0.004000	0.239566	273.586817	0.004000	1.235460	273.586817	0.004000	5.042020
273.587817	0.005000	0.199227	273.587817	0.005000	1.048815	273.587817	0.005000	4.369131
273.588817	0.006000	0.177987	273.588817	0.006000	0.951055	273.588817	0.006000	4.018216
273.589817	0.007000	0.166081	273.589817	0.007000	0.896293	273.589817	0.007000	3.820415
273.590817	0.008000	0.159074	273.590817	0.008000	0.863953	273.590817	0.008000	3.701868
273.591817	0.009000	0.154762	273.591817	0.009000	0.843872	273.591817	0.009000	3.626419
273.592817	0.010000	0.151963	273.592817	0.010000	0.830614	273.592817	0.010000	3.574794
273.602817	0.020000	0.139609	273.602817	0.020000	0.765247	273.602817	0.020000	3.287248
273.612817	0.030000	0.127858	273.612817	0.030000	0.700859	273.612817	0.030000	3.011145
273.622817	0.040000	0.118900	273.622817	0.040000	0.650907	273.622817	0.040000	2.809835
273.632817	0.050000	0.118133	273.632817	0.050000	0.638084	273.632817	0.050000	2.779497
273.642817	0.060000	0.154504	273.642817	0.060000	0.773770	273.642817	0.060000	3.397905
273.652817	0.070000	1.045934	273.652817	0.070000	4.180338	273.652817	0.070000	17.477666
273.662817	0.080000	0.495709	273.662817	0.080000	2.148154	273.662817	0.080000	8.601663
273.672817	0.090000	0.244631	273.672817	0.090000	1.217370	273.672817	0.090000	5.045658
273.682817	0.100000	0.235881	273.682817	0.100000	1.282585	273.682817	0.100000	5.503236
273.782817	0.200000	0.157828	273.782817	0.200000	0.868354	273.782817	0.200000	3.693432
273.882817	0.300000	0.112846	273.882817	0.300000	0.607571	273.882817	0.300000	2.557991
273.982817	0.400000	0.056435	273.982817	0.400000	0.307187	273.982817	0.400000	1.286290
274.082817	0.500000	0.061995	274.082817	0.500000	0.319461	274.082817	0.500000	1.298936
274.182817	0.600000	0.045912	274.182817	0.600000	0.250025	274.182817	0.600000	1.028432
274.282817	0.700000	0.041675	274.282817	0.700000	0.228535	274.282817	0.700000	0.949169
274.382817	0.800000	0.039286	274.382817	0.800000	0.218898	274.382817	0.800000	0.917934
274.482817	0.900000	0.041211	274.482817	0.900000	0.228883	274.482817	0.900000	0.947287
274.582817	1.000000	0.061381	274.582817	1.000000	0.331391	274.582817	1.000000	1.369784
275.582817	2.000000	0.020953	275.582817	2.000000	0.118531	275.582817	2.000000	0.491329
276.582817	3.000000	0.017192	276.582817	3.000000	0.099448	276.582817	3.000000	0.411064
277.582817	4.000000	0.016380	277.582817	4.000000	0.103985	277.582817	4.000000	0.431859
278.582817	5.000000	0.012436	278.582817	5.000000	0.073602	278.582817	5.000000	0.304631
279.582817	6.000000	0.011010	279.582817	6.000000	0.065107	279.582817	6.000000	0.268722
280.582817	7.000000	0.010315	280.582817	7.000000	0.062011	280.582817	7.000000	0.255875
281.582817	8.000000	0.009552	281.582817	8.000000	0.057398	281.582817	8.000000	0.236523
282.074845	200.000000	0.009217	282.074845	200.000000	0.055202	282.074845	200.000000	0.227255
282.582817	9.000000	0.008929	282.582817	9.000000	0.053368	282.582817	9.000000	0.219504
283.582817	10.000000	0.008493	283.582817	10.000000	0.050720	283.582817	10.000000	0.208295
293.582817	20.000000	0.007086	293.582817	20.000000	0.042088	293.582817	20.000000	0.170188
303.582817	30.000000	0.007333	303.582817	30.000000	0.043031	303.582817	30.000000	0.171287
313.582817	40.000000	0.008099	313.582817	40.000000	0.046834	313.582817	40.000000	0.183539
323.582817	50.000000	0.009156	323.582817	50.000000	0.052098	323.582817	50.000000	0.200991
327.358282	300.000000	0.009613	327.358282	300.000000	0.054356	327.358282	300.000000	0.208451
333.582817	60.000000	0.010428	333.582817	60.000000	0.058344	333.582817	60.000000	0.221539
343.582817	70.000000	0.011882	343.582817	70.000000	0.065352	343.582817	70.000000	0.244182
353.582817	80.000000	0.013496	353.582817	80.000000	0.072994	353.582817	80.000000	0.268347
363.582817	90.000000	0.015255	363.582817	90.000000	0.081186	363.582817	90.000000	0.293651
364.149690	200.000000	0.015359	364.149690	200.000000	0.081666	364.149690	200.000000	0.295114
373.582817	100.000000	0.017143	373.582817	100.000000	0.089863	373.582817	100.000000	0.319819
382.074845	300.000000	0.018835	382.074845	300.000000	0.097563	382.074845	300.000000	0.342548
410.374325	0.000100	0.024961	410.374325	0.000100	0.124953	410.374325	0.000100	0.419210
410.374425	0.000200	0.024976	410.374425	0.000200	0.124994	410.374425	0.000200	0.419272
410.374525	0.000300	0.024989	410.374525	0.000300	0.125030	410.374525	0.000300	0.419333
410.374625	0.000400	0.025001	410.374625	0.000400	0.125063	410.374625	0.000400	0.419392
410.374725	0.000500	0.025010	410.374725	0.000500	0.125091	410.374725	0.000500	0.419448
410.374825	0.000600	0.025018	410.374825	0.000600	0.125115	410.374825	0.000600	0.419501
410.374925	0.000700	0.025023	410.374925	0.000700	0.125135	410.374925	0.000700	0.419550
410.375025	0.000800	0.025028	410.375025	0.000800	0.125151	410.375025	0.000800	0.419596
410.375225	0.001000	0.025032	410.375225	0.001000	0.125172	410.375225	0.001000	0.419676
410.376225	0.002000	0.024998	410.376225	0.002000	0.125137	410.376225	0.002000	0.419870
410.377225	0.003000	0.024932	410.377225	0.003000	0.125003	410.377225	0.003000	0.419853
410.378225	0.004000	0.024861	410.378225	0.004000	0.124843	410.378225	0.004000	0.419739
410.379225	0.005000	0.024783	410.379225	0.005000	0.124656	410.379225	0.005000	0.419551
410.380225	0.006000	0.024684	410.380225	0.006000	0.124408	410.380225	0.006000	0.419251
410.381225	0.007000	0.024534	410.381225	0.007000	0.124011	410.381225	0.007000	0.418697
410.382225	0.008000	0.024282	410.382225	0.008000	0.123256	410.382225	0.008000	0.417362
410.383225	0.009000	0.025179	410.383225	0.009000	0.124788	410.383225	0.009000	0.416816
410.384225	0.010000	0.025884	410.384225	0.010000	0.127137	410.384225	0.010000	0.421626

J = 4 → J’ = 0			J = 4 → J’ = 1			J = 4 → J’ = 2		
E_{tot} (cm⁻¹)	E_{col} (cm⁻¹)	σ (Å²)	E_{tot} (cm⁻¹)	E_{col} (cm⁻¹)	σ (Å²)	E_{tot} (cm⁻¹)	E_{col} (cm⁻¹)	σ (Å²)
410.394225	0.020000	0.024991	410.394225	0.020000	0.125211	410.394225	0.020000	0.420277
410.404225	0.030000	0.024898	410.404225	0.030000	0.124997	410.404225	0.030000	0.419998
410.414225	0.040000	0.024827	410.414225	0.040000	0.124833	410.414225	0.040000	0.419732
410.424225	0.050000	0.024716	410.424225	0.050000	0.124579	410.424225	0.050000	0.419242
410.434225	0.060000	0.024355	410.434225	0.060000	0.123781	410.434225	0.060000	0.416288
410.444225	0.070000	0.025959	410.444225	0.070000	0.127302	410.444225	0.070000	0.423861
410.454225	0.080000	0.025207	410.454225	0.080000	0.125697	410.454225	0.080000	0.421395
410.464225	0.090000	0.025059	410.464225	0.090000	0.125358	410.464225	0.090000	0.420805
410.474225	0.100000	0.024973	410.474225	0.100000	0.125140	410.474225	0.100000	0.420385
410.574225	0.200000	0.025001	410.574225	0.200000	0.125258	410.574225	0.200000	0.420579
410.674225	0.300000	0.024968	410.674225	0.300000	0.125209	410.674225	0.300000	0.420604
410.774225	0.400000	0.024982	410.774225	0.400000	0.125320	410.774225	0.400000	0.420998
410.874225	0.500000	0.025037	410.874225	0.500000	0.125497	410.874225	0.500000	0.421278
410.974225	0.600000	0.024945	410.974225	0.600000	0.125134	410.974225	0.600000	0.420488
411.074225	0.700000	0.025243	411.074225	0.700000	0.126092	411.074225	0.700000	0.422680
411.174225	0.800000	0.025121	411.174225	0.800000	0.125836	411.174225	0.800000	0.422272
411.274225	0.900000	0.025177	411.274225	0.900000	0.126004	411.274225	0.900000	0.422467
411.374225	1.000000	0.025130	411.374225	1.000000	0.125921	411.374225	1.000000	0.422435
412.374225	2.000000	0.025388	412.374225	2.000000	0.127021	412.374225	2.000000	0.425424
413.374225	3.000000	0.025593	413.374225	3.000000	0.127971	413.374225	3.000000	0.428021
414.374225	4.000000	0.025826	414.374225	4.000000	0.128983	414.374225	4.000000	0.430700
415.374225	5.000000	0.026055	415.374225	5.000000	0.130019	415.374225	5.000000	0.433518
416.374225	6.000000	0.026280	416.374225	6.000000	0.131033	416.374225	6.000000	0.436248
417.374225	7.000000	0.026505	417.374225	7.000000	0.132045	417.374225	7.000000	0.438959
418.374225	8.000000	0.026738	418.374225	8.000000	0.133080	418.374225	8.000000	0.441722
419.374225	9.000000	0.026970	419.374225	9.000000	0.134114	419.374225	9.000000	0.444477
420.374225	10.000000	0.027200	420.374225	10.000000	0.135146	420.374225	10.000000	0.447223
427.358282	400.000000	0.028829	427.358282	400.000000	0.142426	427.358282	400.000000	0.466384
430.374225	20.000000	0.029540	430.374225	20.000000	0.145602	430.374225	20.000000	0.474640
440.374225	30.000000	0.031920	440.374225	30.000000	0.156261	440.374225	30.000000	0.501883
450.374225	40.000000	0.034332	450.374225	40.000000	0.167093	450.374225	40.000000	0.528866
460.374225	50.000000	0.036767	460.374225	50.000000	0.178072	460.374225	50.000000	0.555516
464.149690	300.000000	0.037691	464.149690	300.000000	0.182249	464.149690	300.000000	0.565478
470.374225	60.000000	0.039217	470.374225	60.000000	0.189171	470.374225	60.000000	0.581770
473.582817	200.000000	0.040005	473.582817	200.000000	0.192754	473.582817	200.000000	0.590101
480.374225	70.000000	0.041674	480.374225	70.000000	0.200367	480.374225	70.000000	0.607575
482.074845	400.000000	0.042092	482.074845	400.000000	0.202278	482.074845	400.000000	0.611915
490.374225	80.000000	0.044131	490.374225	80.000000	0.211634	490.374225	80.000000	0.632881
500.374225	90.000000	0.046583	500.374225	90.000000	0.222949	500.374225	90.000000	0.657647
510.374225	100.000000	0.049025	510.374225	100.000000	0.234290	510.374225	100.000000	0.681837
527.358282	500.000000	0.053135	527.358282	500.000000	0.253548	527.358282	500.000000	0.721525
564.149690	400.000000	0.061811	564.149690	400.000000	0.295089	564.149690	400.000000	0.801867
573.582817	300.000000	0.063944	573.582817	300.000000	0.305217	573.582817	300.000000	0.819860
582.074845	500.000000	0.065846	582.074845	500.000000	0.314488	582.074845	500.000000	0.836339
610.374225	200.000000	0.071973	610.374225	200.000000	0.344626	610.374225	200.000000	0.887407
627.358282	600.000000	0.075470	627.358282	600.000000	0.362071	627.358282	600.000000	0.915273
664.149690	500.000000	0.082526	664.149690	500.000000	0.397927	664.149690	500.000000	0.968581
673.582817	400.000000	0.084213	673.582817	400.000000	0.406663	673.582817	400.000000	0.980735
682.074845	600.000000	0.085687	682.074845	600.000000	0.414362	682.074845	600.000000	0.991166
710.374225	300.000000	0.090287	710.374225	300.000000	0.438884	710.374225	300.000000	1.022578
727.358282	700.000000	0.092813	727.358282	700.000000	0.452775	727.358282	700.000000	1.039104
764.149690	600.000000	0.097677	764.149690	600.000000	0.480872	764.149690	600.000000	1.069621
773.582817	500.000000	0.098796	773.582817	500.000000	0.487679	773.582817	500.000000	1.076441
782.074845	700.000000	0.099755	782.074845	700.000000	0.493675	782.074845	700.000000	1.082260
810.374225	400.000000	0.102651	810.374225	400.000000	0.512871	810.374225	400.000000	1.099823
827.358282	800.000000	0.104174	827.358282	800.000000	0.523887	827.358282	800.000000	1.109266
864.149690	700.000000	0.106955	864.149690	700.000000	0.546759	864.149690	700.000000	1.127902
873.582817	600.000000	0.107561	873.582817	600.000000	0.552455	873.582817	600.000000	1.132448
882.074845	800.000000	0.108070	882.074845	800.000000	0.557536	882.074845	800.000000	1.136507
910.374225	500.000000	0.109542	910.374225	500.000000	0.574230	910.374225	500.000000	1.150072
927.358282	900.000000	0.110270	927.358282	900.000000	0.584120	927.358282	900.000000	1.158451
964.149690	800.000000	0.111510	964.149690	800.000000	0.605369	964.149690	800.000000	1.177916
973.582817	700.000000	0.111764	973.582817	700.000000	0.610794	973.582817	700.000000	1.183292
982.074845	900.000000	0.111979	982.074845	900.000000	0.615680	982.074845	900.000000	1.188304
1010.374230	600.000000	0.112569	1010.374230	600.000000	0.631918	1010.374230	600.000000	1.206121
1064.149690	900.000000	0.113423	1064.149690	900.000000	0.662710	1064.149690	900.000000	1.245361
1073.582820	800.000000	0.113557	1073.582820	800.000000	0.668098	1073.582820	800.000000	1.252966
1110.374230	700.000000	0.114098	1110.374230	700.000000	0.689069	1110.374230	700.000000	1.284494
1173.582820	900.000000	0.115221	1173.582820	900.000000	0.724961	1173.582820	900.000000	1.344073
1210.374230	800.000000	0.116015	1210.374230	800.000000	0.745860	1210.374230	800.000000	1.380511
1310.374230	900.000000	0.118254	1310.374230	900.000000	0.803571	1310.374230	900.000000	1.478113

Inelastic collisions cross-sections					
J = 4 → J' = 3			J = 4 → J' = 5		
E_{tot} (cm⁻¹)	E_{col} (cm⁻¹)	σ (Å²)	E_{tot} (cm⁻¹)	E_{col} (cm⁻¹)	σ (Å²)
273.582917	0.000100	2233.139490	410.374325	0.000100	0.001520
273.583017	0.000200	1113.237410	410.374425	0.000200	0.001582
273.583117	0.000300	718.201179	410.374525	0.000300	0.001563
273.583217	0.000400	520.918747	410.374625	0.000400	0.001528
273.583317	0.000500	404.404175	410.374725	0.000500	0.001490
273.583417	0.000600	328.301074	410.374825	0.000600	0.001454
273.583517	0.000700	275.134941	410.374925	0.000700	0.001421
273.583617	0.000800	236.120755	410.375025	0.000800	0.001390
273.583717	0.000900	206.431868	410.375225	0.001000	0.001335
273.583817	0.001000	183.168887	410.376225	0.002000	0.001171
273.584817	0.002000	86.927746	410.377225	0.003000	0.001106
273.585817	0.003000	59.842070	410.378225	0.004000	0.001092
273.586817	0.004000	47.877845	410.379225	0.005000	0.001109
273.587817	0.005000	41.325785	410.380225	0.006000	0.001145
273.588817	0.006000	37.265666	410.381225	0.007000	0.001196
273.589817	0.007000	34.518557	410.382225	0.008000	0.001255
273.590817	0.008000	32.530863	410.383225	0.009000	0.001321
273.591817	0.009000	31.014444	410.384225	0.010000	0.001389
273.592817	0.010000	29.803002	410.394225	0.020000	0.002078
273.602817	0.020000	23.341826	410.404225	0.030000	0.002627
273.612817	0.030000	19.739988	410.414225	0.040000	0.003086
273.622817	0.040000	17.336851	410.424225	0.050000	0.003623
273.632817	0.050000	16.009151	410.434225	0.060000	0.055807
273.642817	0.060000	17.219115	410.444225	0.070000	0.005227
273.652817	0.070000	68.296574	410.454225	0.080000	0.005874
273.662817	0.080000	38.246886	410.464225	0.090000	0.007714
273.672817	0.090000	23.874280	410.474225	0.100000	0.011737
273.682817	0.100000	25.713939	410.574225	0.200000	0.020962
273.782817	0.200000	30.554247	410.674225	0.300000	0.021400
273.882817	0.300000	16.029474	410.774225	0.400000	0.016757
273.982817	0.400000	9.937940	410.874225	0.500000	0.015020
274.082817	0.500000	7.509150	410.974225	0.600000	0.083241
274.182817	0.600000	6.095801	411.074225	0.700000	0.016320
274.282817	0.700000	5.448217	411.174225	0.800000	0.017381
274.382817	0.800000	5.137520	411.274225	0.900000	0.021977
274.482817	0.900000	5.041358	411.374225	1.000000	0.026651
274.582817	1.000000	6.463449	412.374225	2.000000	0.027041
275.582817	2.000000	3.269916	413.374225	3.000000	0.029805
276.582817	3.000000	2.392828	414.374225	4.000000	0.039130
277.582817	4.000000	2.430990	415.374225	5.000000	0.037544
278.582817	5.000000	1.863654	416.374225	6.000000	0.038711
279.582817	6.000000	1.596904	417.374225	7.000000	0.042259
280.582817	7.000000	1.503361	418.374225	8.000000	0.044787
281.582817	8.000000	1.407995	419.374225	9.000000	0.046567
282.074845	200.000000	1.353184	420.374225	10.000000	0.048752
282.582817	9.000000	1.304769	427.358282	400.000000	0.066706
283.582817	10.000000	1.233424	430.374225	20.000000	0.075297
293.582817	20.000000	0.983628	440.374225	30.000000	0.107698
303.582817	30.000000	0.954183	450.374225	40.000000	0.145853
313.582817	40.000000	0.978738	460.374225	50.000000	0.189227
323.582817	50.000000	1.022972	464.149690	300.000000	0.206829
327.358282	300.000000	1.042227	470.374225	60.000000	0.237177
333.582817	60.000000	1.075810	473.582817	200.000000	0.253424
343.582817	70.000000	1.132862	480.374225	70.000000	0.289043
353.582817	80.000000	1.192098	482.074845	400.000000	0.298206
363.582817	90.000000	1.252507	490.374225	80.000000	0.344193
364.149690	200.000000	1.255950	500.374225	90.000000	0.402065
373.582817	100.000000	1.313396	510.374225	100.000000	0.462166
382.074845	300.000000	1.365077	527.358282	500.000000	0.568193
410.374325	0.000100	1.537697	564.149690	400.000000	0.802006
410.374425	0.000200	1.537925	573.582817	300.000000	0.871956
410.374525	0.000300	1.538103	582.074845	500.000000	0.927401
410.374625	0.000400	1.538236	610.374225	200.000000	1.115922
410.374725	0.000500	1.538329	627.358282	600.000000	1.228244
410.374825	0.000600	1.538384	664.149690	500.000000	1.467318
410.374925	0.000700	1.538404	673.582817	400.000000	1.527448
410.375025	0.000800	1.538393	682.074845	600.000000	1.581125
410.375225	0.001000	1.538290	710.374225	300.000000	1.756772
410.376225	0.002000	1.536925	727.358282	700.000000	1.859753
410.377225	0.003000	1.535301	764.149690	600.000000	2.076652
410.378225	0.004000	1.533829	773.582817	500.000000	2.130844
410.379225	0.005000	1.532426	782.074845	700.000000	2.179223
410.380225	0.006000	1.530923	810.374225	400.000000	2.337355
410.381225	0.007000	1.529214	827.358282	800.000000	2.430020
410.382225	0.008000	1.529050	864.149690	700.000000	2.625029
410.383225	0.009000	1.565790	873.582817	600.000000	2.673747
410.384225	0.010000	1.558986	882.074845	800.000000	2.717145

J = 4 → J' = 3			J = 4 → J' = 5		
E_{tot} (cm⁻¹)	E_{col} (cm⁻¹)	σ (Å²)	E_{tot} (cm⁻¹)	E_{col} (cm⁻¹)	σ (Å²)
410.394225	0.020000	1.534956	910.374225	500.000000	2.858496
410.404225	0.030000	1.532958	927.358282	900.000000	2.940783
410.414225	0.040000	1.531277	964.149690	800.000000	3.111859
410.424225	0.050000	1.528484	973.582817	700.000000	3.154025
410.434225	0.060000	1.530537	982.074845	900.000000	3.191273
410.444225	0.070000	1.560939	1010.374230	600.000000	3.311275
410.454225	0.080000	1.541158	1064.149690	900.000000	3.518934
410.464225	0.090000	1.537527	1073.582820	800.000000	3.552525
410.474225	0.100000	1.535593	1110.374230	700.000000	3.675403
410.574225	0.200000	1.535852	1173.582820	900.000000	3.857399
410.674225	0.300000	1.535776	1210.374230	800.000000	3.947847
410.774225	0.400000	1.535786	1310.374230	900.000000	4.147254
410.874225	0.500000	1.537255			
410.974225	0.600000	1.573869			
411.074225	0.700000	1.542197			
411.174225	0.800000	1.539307			
411.274225	0.900000	1.540786			
411.374225	1.000000	1.539566			
412.374225	2.000000	1.546513			
413.374225	3.000000	1.551813			
414.374225	4.000000	1.557874			
415.374225	5.000000	1.563728			
416.374225	6.000000	1.569444			
417.374225	7.000000	1.575191			
418.374225	8.000000	1.581084			
419.374225	9.000000	1.586902			
420.374225	10.000000	1.592693			
427.358282	400.000000	1.632948			
430.374225	20.000000	1.650188			
440.374225	30.000000	1.706698			
450.374225	40.000000	1.762185			
460.374225	50.000000	1.816635			
464.149690	300.000000	1.836922			
470.374225	60.000000	1.870049			
473.582817	200.000000	1.886971			
480.374225	70.000000	1.922444			
482.074845	400.000000	1.931255			
490.374225	80.000000	1.973846			
500.374225	90.000000	2.024286			
510.374225	100.000000	2.073804			
527.358282	500.000000	2.155920			
564.149690	400.000000	2.325685			
573.582817	300.000000	2.368495			
582.074845	500.000000	2.405790			
610.374225	200.000000	2.527722			
627.358282	600.000000	2.598926			
664.149690	500.000000	2.748712			
673.582817	400.000000	2.786205			
682.074845	600.000000	2.819651			
710.374225	300.000000	2.929030			
727.358282	700.000000	2.993118			
764.149690	600.000000	3.127642			
773.582817	500.000000	3.161131			
782.074845	700.000000	3.190869			
810.374225	400.000000	3.287068			
827.358282	800.000000	3.342452			
864.149690	700.000000	3.455556			
873.582817	600.000000	3.482914			
882.074845	800.000000	3.506937			
910.374225	500.000000	3.582725			
927.358282	900.000000	3.624987			
964.149690	800.000000	3.708247			
973.582817	700.000000	3.727808			
982.074845	900.000000	3.744840			
1010.374230	600.000000	3.797560			
1064.149690	900.000000	3.882692			
1073.582820	800.000000	3.895821			
1110.374230	700.000000	3.942574			
1173.582820	900.000000	4.008753			
1210.374230	800.000000	4.040536			
1310.374230	900.000000	4.108101			

Inelastic collisions cross-sections								
J = 5 → J' = 0			J = 5 → J' = 1			J = 5 → J' = 2		
E_{tot} (cm⁻¹)	E_{col} (cm⁻¹)	σ (Å²)	E_{tot} (cm⁻¹)	E_{col} (cm⁻¹)	σ (Å²)	E_{tot} (cm⁻¹)	E_{col} (cm⁻¹)	σ (Å²)
410.374325	0.000100	8.268102	410.374325	0.000100	23.942126	410.374325	0.000100	45.095056
410.374425	0.000200	4.243274	410.374425	0.000200	12.287203	410.374425	0.000200	23.139224
410.374525	0.000300	2.755347	410.374525	0.000300	7.978543	410.374525	0.000300	15.023670
410.374625	0.000400	1.991561	410.374625	0.000400	5.766822	410.374625	0.000400	10.858575
410.374725	0.000500	1.532259	410.374725	0.000500	4.436812	410.374725	0.000500	8.354467
410.374825	0.000600	1.228476	410.374825	0.000600	3.557147	410.374825	0.000600	6.698672
410.374925	0.000700	1.014278	410.374925	0.000700	2.936894	410.374925	0.000700	5.531496
410.375025	0.000800	0.856139	410.375025	0.000800	2.478979	410.375025	0.000800	4.670063
410.375225	0.001000	0.640325	410.375225	0.001000	1.854059	410.375225	0.001000	3.495026
410.376225	0.002000	0.248109	410.376225	0.002000	0.718395	410.376225	0.002000	1.363570
410.377225	0.003000	0.143070	410.377225	0.003000	0.414291	410.377225	0.003000	0.795316
410.378225	0.004000	0.100934	410.378225	0.004000	0.292319	410.378225	0.004000	0.568323
410.379225	0.005000	0.080706	410.379225	0.005000	0.233773	410.379225	0.005000	0.459760
410.380225	0.006000	0.069942	410.380225	0.006000	0.202621	410.380225	0.006000	0.402171
410.381225	0.007000	0.063814	410.381225	0.007000	0.184887	410.381225	0.007000	0.369471
410.382225	0.008000	0.060154	410.382225	0.008000	0.174295	410.382225	0.008000	0.349983
410.383225	0.009000	0.057975	410.383225	0.009000	0.167988	410.383225	0.009000	0.338507
410.384225	0.010000	0.056242	410.384225	0.010000	0.162966	410.384225	0.010000	0.329106
410.394225	0.020000	0.050026	410.394225	0.020000	0.144903	410.394225	0.020000	0.293855
410.404225	0.030000	0.045708	410.404225	0.030000	0.132378	410.404225	0.030000	0.268295
410.414225	0.040000	0.042394	410.414225	0.040000	0.122874	410.414225	0.040000	0.249056
410.424225	0.050000	0.040460	410.424225	0.050000	0.118122	410.424225	0.050000	0.240789
410.434225	0.060000	0.040450	410.434225	0.060000	0.397250	410.434225	0.060000	1.228918
410.444225	0.070000	0.043167	410.444225	0.070000	0.126498	410.444225	0.070000	0.256039
410.454225	0.080000	0.049143	410.454225	0.080000	0.142227	410.454225	0.080000	0.284403
410.464225	0.090000	0.064191	410.464225	0.090000	0.185079	410.464225	0.090000	0.366861
410.474225	0.100000	0.098583	410.474225	0.100000	0.283798	410.474225	0.100000	0.556663
410.574225	0.200000	0.048252	410.574225	0.200000	0.140294	410.574225	0.200000	0.290214
410.674225	0.300000	0.041020	410.674225	0.300000	0.116982	410.674225	0.300000	0.230530
410.774225	0.400000	0.017875	410.774225	0.400000	0.051618	410.774225	0.400000	0.105122
410.874225	0.500000	0.016800	410.874225	0.500000	0.048193	410.874225	0.500000	0.097047
410.974225	0.600000	0.014942	410.974225	0.600000	0.093485	410.974225	0.600000	0.272010
411.074225	0.700000	0.013638	411.074225	0.700000	0.039084	411.074225	0.700000	0.078754
411.174225	0.800000	0.013041	411.174225	0.800000	0.037333	411.174225	0.800000	0.075405
411.274225	0.900000	0.016046	411.274225	0.900000	0.045872	411.274225	0.900000	0.093028
411.374225	1.000000	0.016523	411.374225	1.000000	0.047214	411.374225	1.000000	0.094832
412.374225	2.000000	0.007009	412.374225	2.000000	0.019962	412.374225	2.000000	0.040386
413.374225	3.000000	0.005778	413.374225	3.000000	0.016385	413.374225	3.000000	0.033097
414.374225	4.000000	0.005721	414.374225	4.000000	0.016027	414.374225	4.000000	0.032560
415.374225	5.000000	0.004221	415.374225	5.000000	0.011935	415.374225	5.000000	0.024162
416.374225	6.000000	0.003741	416.374225	6.000000	0.010575	416.374225	6.000000	0.021382
417.374225	7.000000	0.003531	417.374225	7.000000	0.009959	417.374225	7.000000	0.020148
418.374225	8.000000	0.003268	418.374225	8.000000	0.009218	418.374225	8.000000	0.018651
419.374225	9.000000	0.003050	419.374225	9.000000	0.008608	419.374225	9.000000	0.017401
420.374225	10.000000	0.002902	420.374225	10.000000	0.008189	420.374225	10.000000	0.016547
427.358282	400.000000	0.002475	427.358282	400.000000	0.006979	427.358282	400.000000	0.014070
430.374225	20.000000	0.002435	430.374225	20.000000	0.006865	430.374225	20.000000	0.013827
440.374225	30.000000	0.002538	440.374225	30.000000	0.007154	440.374225	30.000000	0.014376
450.374225	40.000000	0.002827	450.374225	40.000000	0.007971	450.374225	40.000000	0.015992
460.374225	50.000000	0.003226	460.374225	50.000000	0.009098	460.374225	50.000000	0.018229
464.149690	300.000000	0.003399	464.149690	300.000000	0.009587	464.149690	300.000000	0.019202
470.374225	60.000000	0.003709	470.374225	60.000000	0.010462	470.374225	60.000000	0.020942
473.582817	200.000000	0.003880	473.582817	200.000000	0.010945	473.582817	200.000000	0.021902
480.374225	70.000000	0.004265	480.374225	70.000000	0.012036	480.374225	70.000000	0.024071
482.074845	400.000000	0.004367	482.074845	400.000000	0.012323	482.074845	400.000000	0.024642
490.374225	80.000000	0.004890	490.374225	80.000000	0.013802	490.374225	80.000000	0.027584
500.374225	90.000000	0.005580	500.374225	90.000000	0.015753	500.374225	90.000000	0.031466
510.374225	100.000000	0.006332	510.374225	100.000000	0.017881	510.374225	100.000000	0.035702
527.358282	500.000000	0.007745	527.358282	500.000000	0.021882	527.358282	500.000000	0.043675
564.149690	400.000000	0.011146	564.149690	400.000000	0.031346	564.149690	400.000000	0.062607
573.582817	300.000000	0.012339	573.582817	300.000000	0.034997	573.582817	300.000000	0.069998
582.074845	500.000000	0.013239	582.074845	500.000000	0.037550	582.074845	500.000000	0.075147
610.374225	200.000000	0.016467	610.374225	200.000000	0.046899	610.374225	200.000000	0.094226
627.358282	600.000000	0.018468	627.358282	600.000000	0.052775	627.358282	600.000000	0.106366
664.149690	500.000000	0.022843	664.149690	500.000000	0.065902	664.149690	500.000000	0.133978
673.582817	400.000000	0.023955	673.582817	400.000000	0.069314	673.582817	400.000000	0.141274
682.074845	600.000000	0.024950	682.074845	600.000000	0.072390	682.074845	600.000000	0.147896
710.374225	300.000000	0.028194	710.374225	300.000000	0.082619	710.374225	300.000000	0.170223
727.358282	700.000000	0.030082	727.358282	700.000000	0.088705	727.358282	700.000000	0.183721
764.149690	600.000000	0.034008	764.149690	600.000000	0.101640	764.149690	600.000000	0.212878
773.582817	500.000000	0.034981	773.582817	500.000000	0.104881	773.582817	500.000000	0.220265
782.074845	700.000000	0.035847	782.074845	700.000000	0.107774	782.074845	700.000000	0.226881
810.374225	400.000000	0.038674	810.374225	400.000000	0.117202	810.374225	400.000000	0.248557
827.358282	800.000000	0.040337	827.358282	800.000000	0.122688	827.358282	800.000000	0.261231
864.149690	700.000000	0.043893	864.149690	700.000000	0.134076	864.149690	700.000000	0.287643
873.582817	600.000000	0.044798	873.582817	600.000000	0.136876	873.582817	600.000000	0.294164
882.074845	800.000000	0.045612	882.074845	800.000000	0.139352	882.074845	800.000000	0.299944

J = 5 → J' = 0			J = 5 → J' = 1			J = 5 → J' = 2		
E_{tot} (cm⁻¹)	E_{col} (cm⁻¹)	σ (Å²)	E_{tot} (cm⁻¹)	E_{col} (cm⁻¹)	σ (Å²)	E_{tot} (cm⁻¹)	E_{col} (cm⁻¹)	σ (Å²)
910.374225	500.000000	0.048316	910.374225	500.000000	0.147272	910.374225	500.000000	0.318579
927.358282	900.000000	0.049929	927.358282	900.000000	0.151763	927.358282	900.000000	0.329309
964.149690	800.000000	0.053375	964.149690	800.000000	0.160745	964.149690	800.000000	0.351479
973.582817	700.000000	0.054241	973.582817	700.000000	0.162872	973.582817	700.000000	0.356949
982.074845	900.000000	0.055010	982.074845	900.000000	0.164717	982.074845	900.000000	0.361793
1010.374230	600.000000	0.057510	1010.374230	600.000000	0.170429	1010.374230	600.000000	0.377603
1064.149690	900.000000	0.061841	1064.149690	900.000000	0.179287	1064.149690	900.000000	0.406412
1073.582820	800.000000	0.062534	1073.582820	800.000000	0.180576	1073.582820	800.000000	0.411367
1110.374230	700.000000	0.065029	1110.374230	700.000000	0.184907	1110.374230	700.000000	0.430600
1173.582820	900.000000	0.068633	1173.582820	900.000000	0.190209	1173.582820	900.000000	0.463932
1210.374230	800.000000	0.070460	1210.374230	800.000000	0.192466	1210.374230	800.000000	0.483914
1310.374230	900.000000	0.075308	1310.374230	900.000000	0.197682	1310.374230	900.000000	0.542366

Inelastic collisions cross-sections					
J = 5 → J’ = 3			J = 5 → J’ = 4		
E_{tot} (cm⁻¹)	E_{col} (cm⁻¹)	σ (Å²)	E_{tot} (cm⁻¹)	E_{col} (cm⁻¹)	σ (Å²)
410.374325	0.000100	206.763057	410.374325	0.000100	1700.884260
410.374425	0.000200	106.118667	410.374425	0.000200	885.313778
410.374525	0.000300	68.931865	410.374525	0.000300	583.071107
410.374625	0.000400	49.856212	410.374625	0.000400	427.462332
410.374725	0.000500	38.394524	410.374725	0.000500	333.574459
410.374825	0.000600	30.820890	410.374825	0.000600	271.248895
410.374925	0.000700	25.486295	410.374925	0.000700	227.127648
410.375025	0.000800	21.552403	410.375025	0.000800	194.414324
410.375225	0.001000	16.193447	410.375225	0.001000	149.469115
410.376225	0.002000	6.521046	410.376225	0.002000	65.519835
410.377225	0.003000	3.970956	410.377225	0.003000	41.250286
410.378225	0.004000	2.961014	410.378225	0.004000	30.549809
410.379225	0.005000	2.480522	410.379225	0.005000	24.816672
410.380225	0.006000	2.225977	410.380225	0.006000	21.366998
410.381225	0.007000	2.080945	410.381225	0.007000	19.117933
410.382225	0.008000	1.993524	410.382225	0.008000	17.558972
410.383225	0.009000	1.940871	410.383225	0.009000	16.429842
410.384225	0.010000	1.897197	410.384225	0.010000	15.548993
410.394225	0.020000	1.700344	410.394225	0.020000	11.628825
410.404225	0.030000	1.545690	410.404225	0.030000	9.802050
410.414225	0.040000	1.435125	410.414225	0.040000	8.636837
410.424225	0.050000	1.431714	410.424225	0.050000	8.112577
410.434225	0.060000	25.104799	410.434225	0.060000	104.143692
410.444225	0.070000	1.601053	410.444225	0.070000	8.361187
410.454225	0.080000	1.634444	410.454225	0.080000	8.222946
410.464225	0.090000	2.026828	410.464225	0.090000	9.599462
410.474225	0.100000	2.952422	410.474225	0.100000	13.145943
410.574225	0.200000	1.755273	410.574225	0.200000	11.747253
410.674225	0.300000	1.306888	410.674225	0.300000	8.001193
410.774225	0.400000	0.630885	410.774225	0.400000	4.702431
410.874225	0.500000	0.548352	410.874225	0.500000	3.374323
410.974225	0.600000	5.250888	410.974225	0.600000	15.595406
411.074225	0.700000	0.451721	411.074225	0.700000	2.622761
411.174225	0.800000	0.433357	411.174225	0.800000	2.445802
411.274225	0.900000	0.522959	411.274225	0.900000	2.750968
411.374225	1.000000	0.577890	411.374225	1.000000	3.004615
412.374225	2.000000	0.236420	412.374225	2.000000	1.535345
413.374225	3.000000	0.193652	413.374225	3.000000	1.136302
414.374225	4.000000	0.194366	414.374225	4.000000	1.126865
415.374225	5.000000	0.142976	415.374225	5.000000	0.871096
416.374225	6.000000	0.125903	416.374225	6.000000	0.753758
417.374225	7.000000	0.118955	417.374225	7.000000	0.710246
418.374225	8.000000	0.110147	418.374225	8.000000	0.663210
419.374225	9.000000	0.102460	419.374225	9.000000	0.617192
420.374225	10.000000	0.097247	420.374225	10.000000	0.585520
427.358282	400.000000	0.081711	427.358282	400.000000	0.494150
430.374225	20.000000	0.079803	430.374225	20.000000	0.482972
440.374225	30.000000	0.081097	440.374225	30.000000	0.489901
450.374225	40.000000	0.088031	450.374225	40.000000	0.527431
460.374225	50.000000	0.097890	460.374225	50.000000	0.578389
464.149690	300.000000	0.102161	464.149690	300.000000	0.599686
470.374225	60.000000	0.109751	470.374225	60.000000	0.636470
473.582817	200.000000	0.113905	473.582817	200.000000	0.656071
480.374225	70.000000	0.123195	480.374225	70.000000	0.698629
482.074845	400.000000	0.125621	482.074845	400.000000	0.709469
490.374225	80.000000	0.137992	490.374225	80.000000	0.763140
500.374225	90.000000	0.153989	500.374225	90.000000	0.828954
510.374225	100.000000	0.171066	510.374225	100.000000	0.895393
527.358282	500.000000	0.202271	527.358282	500.000000	1.008483
564.149690	400.000000	0.275462	564.149690	400.000000	1.239899
573.582817	300.000000	0.298409	573.582817	300.000000	1.311363
582.074845	500.000000	0.316753	582.074845	500.000000	1.363294
610.374225	200.000000	0.381239	610.374225	200.000000	1.537498
627.358282	600.000000	0.420806	627.358282	600.000000	1.638454
664.149690	500.000000	0.507218	664.149690	500.000000	1.847650
673.582817	400.000000	0.529303	673.582817	400.000000	1.899223
682.074845	600.000000	0.549100	682.074845	600.000000	1.944953
710.374225	300.000000	0.614189	710.374225	300.000000	2.092754
727.358282	700.000000	0.652376	727.358282	700.000000	2.178254
764.149690	600.000000	0.732100	764.149690	600.000000	2.356048
773.582817	500.000000	0.751762	773.582817	500.000000	2.400023
782.074845	700.000000	0.769174	782.074845	700.000000	2.439172
810.374225	400.000000	0.825047	810.374225	400.000000	2.566374
827.358282	800.000000	0.856910	827.358282	800.000000	2.640426
864.149690	700.000000	0.921428	864.149690	700.000000	2.795195
873.582817	600.000000	0.936963	873.582817	600.000000	2.833641
882.074845	800.000000	0.950596	882.074845	800.000000	2.867814

J = 5 → J' = 3			J = 5 → J' = 4		
E_{tot} (cm⁻¹)	E_{col} (cm⁻¹)	σ (Å²)	E_{tot} (cm⁻¹)	E_{col} (cm⁻¹)	σ (Å²)
910.374225	500.000000	0.993662	910.374225	500.000000	2.978617
927.358282	900.000000	1.017810	927.358282	900.000000	3.042736
964.149690	800.000000	1.066056	964.149690	800.000000	3.174986
973.582817	700.000000	1.077598	973.582817	700.000000	3.207331
982.074845	900.000000	1.087723	982.074845	900.000000	3.235788
1010.374230	600.000000	1.119822	1010.374230	600.000000	3.326889
1064.149690	900.000000	1.175189	1064.149690	900.000000	3.481536
1073.582820	800.000000	1.184333	1073.582820	800.000000	3.506120
1110.374230	700.000000	1.218931	1110.374230	700.000000	3.594794
1173.582820	900.000000	1.276236	1173.582820	900.000000	3.721719
1210.374230	800.000000	1.309314	1210.374230	800.000000	3.782362
1310.374230	900.000000	1.400206	1310.374230	900.000000	3.908943

Inelastic collisions' Rate Coefficients: Pseudo-Singlet State					
Initial rotational state: j=0					
T(K)	k ($cm^3mol^{-1}s^{-1}$)				
	**Final State: j'=5**	**Final State: j'=4**	**Final State: j'=3**	**Final State: j'=2**	**Final State: j'=1**
**5**	5.08936049E-65	1.60973166E-47	1.18180791E-32	1.34417588E-22	2.982255E-14
**6**	1.76498723E-56	7.84067521E-42	3.04576184E-29	6.69047017E-21	1.0931509E-13
**7**	2.22768152E-50	9.05451485E-38	8.31538004E-27	1.0899456E-19	2.7415301E-13
**8**	8.41809691E-46	1.01054743E-34	5.58904209E-25	8.85014718E-19	5.4331037E-13
**9**	3.07040934E-42	2.3789282E-32	1.47720393E-23	4.52330606E-18	9.2154359E-13
**10**	2.18153304E-39	1.88623812E-30	2.03300671E-22	1.67362445E-17	1.40310363E-12
**11**	4.71987331E-37	6.78344235E-29	1.74169974E-21	4.89948107E-17	1.9762827E-12
**12**	4.18804751E-35	1.34906562E-27	1.04614333E-20	1.20397408E-16	2.62693177E-12
**13**	1.87282513E-33	1.70173465E-26	4.78314302E-20	2.58703873E-16	3.3406823E-12
**14**	4.88972057E-32	1.50147877E-25	1.76533881E-19	5.00433345E-16	4.10415282E-12
**15**	8.30247204E-31	9.95511255E-25	5.49042783E-19	8.9018944E-16	4.90549046E-12
**16**	9.93972761E-30	5.23356877E-24	1.48605531E-18	1.4794761E-15	5.73452205E-12
**17**	8.92513727E-29	2.27296875E-23	3.5875513E-18	2.32525304E-15	6.58269693E-12
**18**	6.30640105E-28	8.41933947E-23	7.87400002E-18	3.48841839E-15	7.4429322E-12
**19**	3.64187407E-27	2.72754053E-22	1.59504018E-17	5.03234176E-15	8.30942329E-12
**20**	1.77171939E-26	7.88497549E-22	3.01825926E-17	7.02151981E-15	9.17745315E-12
**21**	7.44095291E-26	2.06739765E-21	5.38739717E-17	9.52039229E-15	1.00432161E-11
**22**	2.75246743E-25	4.9821027E-21	9.14313571E-17	1.259233E-14	1.09036628E-11
**23**	9.11706093E-25	1.11565454E-20	1.48510506E-16	1.629883E-14	1.17563673E-11
**24**	2.74160841E-24	2.34283783E-20	2.32134363E-16	2.069879E-14	1.2599416E-11
**25**	7.57170248E-24	4.64922675E-20	3.507798E-16	2.58481E-14	1.34313143E-11
**26**	1.93938865E-23	8.77530563E-20	5.14431108E-16	3.179921E-14	1.42509102E-11
**27**	4.64552594E-23	1.58411142E-19	7.34600601E-16	3.860092E-14	1.50573314E-11
**28**	1.04809833E-22	2.74793787E-19	1.02431828E-15	4.629826E-14	1.58499337E-11
**29**	2.24097472E-22	4.59936363E-19	1.39809364E-15	5.493241E-14	1.66282586E-11
**30**	4.56514065E-22	7.4540757E-19	1.87185326E-15	6.454079E-14	1.73919986E-11
**31**	8.90162987E-22	1.17336317E-18	2.46285835E-15	7.515709E-14	1.81409693E-11
**32**	1.66819611E-21	1.79880439E-18	3.18960614E-15	8.68114E-14	1.88750859E-11
**33**	3.01534686E-21	2.69201027E-18	4.07171912E-15	9.95304E-14	1.95943439E-11
**34**	5.27361294E-21	3.94110774E-18	5.12982569E-15	1.1333747E-13	2.02988043E-11
**35**	8.94895002E-21	5.65474007E-18	6.38543541E-15	1.2825294E-13	2.09885799E-11
**36**	1.47708798E-20	7.96479192E-18	7.86081164E-15	1.4429425E-13	2.16638255E-11
**37**	2.37668921E-20	1.10291294E-17	9.57884411E-15	1.6147614E-13	2.23247288E-11
**38**	3.73534296E-20	1.5034314E-17	1.156292E-14	1.7981082E-13	2.29715035E-11
**39**	5.74450863E-20	2.01982516E-17	1.383682E-14	1.9930822E-13	2.36043833E-11
**40**	8.65834351E-20	2.67727405E-17	1.642456E-14	2.1997606E-13	2.42236171E-11
**41**	1.28086634E-19	3.50458861E-17	1.935032E-14	2.418201E-13	2.48294653E-11
**42**	1.86220652E-19	4.53443529E-17	2.263833E-14	2.6484427E-13	2.5422196E-11
**43**	2.66392615E-19	5.80354313E-17	2.631275E-14	2.890508E-13	2.6002083E-11
**44**	3.75366421E-19	7.35288962E-17	3.039761E-14	3.1444037E-13	2.65694028E-11
**45**	5.21500416E-19	9.22786444E-17	3.491669E-14	3.4101222E-13	2.71244337E-11
**46**	7.15006539E-19	1.14784097E-16	3.989348E-14	3.687643E-13	2.76674535E-11
**47**	9.6823004E-19	1.41591362E-16	4.535108E-14	3.9769334E-13	2.81987389E-11
**48**	1.29594851E-18	1.73294145E-16	5.131217E-14	4.2779497E-13	2.87185642E-11
**49**	1.71568868E-18	2.1053442E-16	5.779892E-14	4.5906381E-13	2.92272008E-11
**50**	2.24805924E-18	2.5400285E-16	6.483296E-14	4.9149356E-13	2.97249163E-11
**51**	2.91709753E-18	3.04438972E-16	7.243533E-14	5.2507707E-13	3.02119741E-11

**52**	3.75062807E-18	3.62631139E-16	8.062646E-14	5.5980639E-13	3.06886332E-11
**53**	4.78063039E-18	4.29416252E-16	8.942612E-14	5.956729E-13	3.11551476E-11
**54**	6.04361386E-18	5.05679262E-16	9.885339E-14	6.3266732E-13	3.16117666E-11
**55**	7.58099686E-18	5.92352486E-16	1.0892666E-13	6.7077977E-13	3.2058734E-11
**56**	9.43948786E-18	6.90414717E-16	1.1966359E-13	7.0999984E-13	3.24962885E-11
**57**	1.16714658E-17	8.00890172E-16	1.3108112E-13	7.5031665E-13	3.29246635E-11
**58**	1.43353575E-17	9.24847271E-16	1.4319545E-13	7.9171887E-13	3.33440869E-11
**59**	1.74960087E-17	1.06339727E-15	1.5602202E-13	8.3419477E-13	3.37547814E-11
**60**	2.12250485E-17	1.21769275E-15	1.6957553E-13	8.7773228E-13	3.41569645E-11
**61**	2.56012426E-17	1.38892602E-15	1.8386991E-13	9.2231901E-13	3.45508482E-11
**62**	3.07108345E-17	1.57832736E-15	1.9891838E-13	9.6794227E-13	3.49366396E-11
**63**	3.66478732E-17	1.78716324E-15	2.1473339E-13	1.01458914E-12	3.53145405E-11
**64**	4.3514524E-17	2.0167344E-15	2.3132665E-13	1.06224648E-12	3.56847479E-11
**65**	5.14213632E-17	2.26837388E-15	2.4870916E-13	1.11090094E-12	3.60474537E-11
**66**	6.04876528E-17	2.54344505E-15	2.668912E-13	1.16053904E-12	3.64028451E-11
**67**	7.08415953E-17	2.8433395E-15	2.8588234E-13	1.21114713E-12	3.67511046E-11
**68**	8.26205683E-17	3.169475E-15	3.0569145E-13	1.26271146E-12	3.70924101E-11
**69**	9.59713355E-17	3.52329337E-15	3.2632673E-13	1.3152182E-12	3.74269351E-11
**70**	1.11050237E-16	3.90625836E-15	3.4779572E-13	1.36865343E-12	3.77548486E-11
**71**	1.28023354E-16	4.31985354E-15	3.7010529E-13	1.42300319E-12	3.80763155E-11
**72**	1.47066653E-16	4.76558018E-15	3.932617E-13	1.47825347E-12	3.83914964E-11
**73**	1.68366102E-16	5.24495514E-15	4.1727059E-13	1.53439026E-12	3.8700548E-11
**74**	1.92117766E-16	5.75950882E-15	4.4213698E-13	1.59139955E-12	3.9003623E-11
**75**	2.18527881E-16	6.31078306E-15	4.6786533E-13	1.64926733E-12	3.93008705E-11
**76**	2.47812894E-16	6.90032916E-15	4.9445954E-13	1.70797964E-12	3.95924354E-11
**77**	2.80199494E-16	7.52970586E-15	5.2192292E-13	1.76752253E-12	3.98784596E-11
**78**	3.15924609E-16	8.20047741E-15	5.5025831E-13	1.82788213E-12	4.0159081E-11
**79**	3.55235382E-16	8.91421169E-15	5.7946799E-13	1.88904463E-12	4.04344344E-11
**80**	3.98389133E-16	9.67247831E-15	6.0955377E-13	1.95099629E-12	4.07046511E-11
**81**	4.45653289E-16	1.047685E-14	6.4051697E-13	2.01372346E-12	4.09698592E-11
**82**	4.97305297E-16	1.132889E-14	6.7235847E-13	2.07721257E-12	4.12301839E-11
**83**	5.53632523E-16	1.223016E-14	7.0507868E-13	2.14145016E-12	4.14857471E-11
**84**	6.14932122E-16	1.318222E-14	7.386776E-13	2.20642289E-12	4.17366677E-11
**85**	6.81510896E-16	1.418663E-14	7.7315483E-13	2.27211753E-12	4.19830619E-11
**86**	7.53685135E-16	1.524493E-14	8.0850957E-13	2.33852097E-12	4.22250432E-11
**87**	8.31780434E-16	1.635866E-14	8.4474063E-13	2.40562022E-12	4.24627219E-11
**88**	9.16131505E-16	1.752933E-14	8.8184648E-13	2.47340245E-12	4.26962063E-11
**89**	1.00708196E-15	1.875847E-14	9.1982523E-13	2.54185495E-12	4.29256016E-11
**90**	1.10498411E-15	2.004756E-14	9.5867467E-13	2.61096515E-12	4.31510107E-11
**91**	1.21019869E-15	2.139808E-14	9.9839229E-13	2.68072064E-12	4.33725341E-11
**92**	1.32309465E-15	2.281152E-14	1.03897523E-12	2.75110916E-12	4.35902699E-11
**93**	1.44404888E-15	2.428931E-14	1.08042041E-12	2.82211859E-12	4.38043138E-11
**94**	1.57344592E-15	2.583288E-14	1.12272442E-12	2.89373699E-12	4.40147595E-11
**95**	1.71167773E-15	2.744366E-14	1.16588363E-12	2.96595256E-12	4.42216982E-11
**96**	1.85914333E-15	2.912302E-14	1.20989414E-12	3.03875367E-12	4.44252191E-11
**97**	2.01624859E-15	3.087235E-14	1.25475182E-12	3.11212884E-12	4.46254094E-11
**98**	2.18340584E-15	3.269298E-14	1.30045234E-12	3.18606677E-12	4.48223543E-11
**99**	2.36103362E-15	3.458625E-14	1.34699113E-12	3.26055631E-12	4.50161368E-11
**100**	2.54955632E-15	3.655347E-14	1.39436343E-12	3.3355865E-12	4.52068383E-11
**101**	2.74940391E-15	3.85959E-14	1.44256431E-12	3.41114651E-12	4.5394538E-11
**102**	2.96101156E-15	4.07148E-14	1.49158864E-12	3.48722571E-12	4.55793135E-11

**103**	3.18481934E-15	4.291141E-14	1.54143113E-12	3.56381362E-12	4.57612406E-11
**104**	3.42127188E-15	4.518693E-14	1.59208635E-12	3.64089991E-12	4.59403933E-11
**105**	3.67081803E-15	4.754253E-14	1.64354869E-12	3.71847446E-12	4.61168439E-11
**106**	3.93391052E-15	4.997937E-14	1.69581244E-12	3.79652726E-12	4.62906632E-11
**107**	4.21100565E-15	5.249857E-14	1.74887173E-12	3.87504851E-12	4.64619202E-11
**108**	4.50256291E-15	5.510123E-14	1.80272057E-12	3.95402855E-12	4.66306823E-11
**109**	4.80904465E-15	5.778841E-14	1.85735289E-12	4.03345788E-12	4.67970157E-11
**110**	5.13091577E-15	6.056116E-14	1.91276247E-12	4.11332717E-12	4.69609847E-11
**111**	5.46864332E-15	6.34205E-14	1.96894302E-12	4.19362725E-12	4.71226524E-11
**112**	5.82269625E-15	6.63674E-14	2.02588815E-12	4.2743491E-12	4.72820803E-11
**113**	6.19354498E-15	6.940282E-14	2.0835914E-12	4.35548386E-12	4.74393286E-11
**114**	6.58166111E-15	7.252768E-14	2.14204621E-12	4.43702282E-12	4.75944561E-11
**115**	6.98751711E-15	7.57429E-14	2.20124597E-12	4.51895743E-12	4.77475204E-11
**116**	7.41158594E-15	7.904933E-14	2.26118399E-12	4.60127929E-12	4.78985775E-11
**117**	7.85434076E-15	8.244781E-14	2.32185353E-12	4.68398012E-12	4.80476825E-11
**118**	8.31625458E-15	8.593916E-14	2.38324779E-12	4.76705184E-12	4.81948889E-11
**119**	8.79779997E-15	8.952416E-14	2.44535992E-12	4.85048647E-12	4.83402492E-11
**120**	9.29944872E-15	9.320356E-14	2.50818304E-12	4.93427618E-12	4.84838146E-11
**121**	9.82167152E-15	9.697808E-14	2.57171021E-12	5.01841329E-12	4.86256353E-11
**122**	1.036494E-14	1.0084842E-13	2.63593448E-12	5.10289025E-12	4.87657601E-11
**123**	1.092971E-14	1.0481524E-13	2.70084885E-12	5.18769965E-12	4.89042369E-11
**124**	1.151647E-14	1.0887917E-13	2.7664463E-12	5.27283421E-12	4.90411124E-11
**125**	1.212566E-14	1.1304083E-13	2.83271978E-12	5.35828676E-12	4.91764323E-11
**126**	1.275776E-14	1.1730079E-13	2.89966224E-12	5.4440503E-12	4.93102411E-11
**127**	1.341321E-14	1.216596E-13	2.9672666E-12	5.53011792E-12	4.94425825E-11
**128**	1.409248E-14	1.2611778E-13	3.03552577E-12	5.61648284E-12	4.9573499E-11
**129**	1.479601E-14	1.3067583E-13	3.10443265E-12	5.7031384E-12	4.97030322E-11
**130**	1.552425E-14	1.353342E-13	3.17398014E-12	5.79007806E-12	4.98312228E-11
**131**	1.627765E-14	1.4009334E-13	3.24416113E-12	5.8772954E-12	4.99581105E-11
**132**	1.705665E-14	1.4495365E-13	3.3149685E-12	5.9647841E-12	5.0083734E-11
**133**	1.786167E-14	1.4991551E-13	3.38639516E-12	6.05253795E-12	5.02081311E-11
**134**	1.869316E-14	1.5497927E-13	3.458434E-12	6.14055087E-12	5.03313389E-11
**135**	1.955154E-14	1.6014527E-13	3.53107792E-12	6.22881686E-12	5.04533934E-11
**136**	2.043722E-14	1.6541379E-13	3.60431982E-12	6.31733002E-12	5.05743298E-11
**137**	2.135063E-14	1.7078512E-13	3.67815264E-12	6.40608457E-12	5.06941827E-11
**138**	2.229217E-14	1.7625949E-13	3.7525693E-12	6.49507481E-12	5.08129855E-11
**139**	2.326225E-14	1.8183712E-13	3.82756274E-12	6.58429515E-12	5.09307711E-11
**140**	2.426126E-14	1.8751821E-13	3.90312592E-12	6.67374008E-12	5.10475714E-11
**141**	2.52896E-14	1.9330293E-13	3.97925182E-12	6.76340419E-12	5.11634177E-11
**142**	2.634765E-14	1.9919141E-13	4.05593343E-12	6.85328214E-12	5.12783404E-11
**143**	2.743579E-14	2.0518378E-13	4.13316376E-12	6.94336869E-12	5.13923694E-11
**144**	2.855439E-14	2.1128012E-13	4.21093584E-12	7.03365868E-12	5.15055335E-11
**145**	2.970383E-14	2.1748051E-13	4.28924272E-12	7.12414704E-12	5.1617861E-11
**146**	3.088445E-14	2.2378498E-13	4.36807747E-12	7.21482877E-12	5.17293795E-11
**147**	3.209661E-14	2.3019355E-13	4.4474332E-12	7.30569894E-12	5.1840116E-11
**148**	3.334066E-14	2.3670623E-13	4.52730302E-12	7.39675271E-12	5.19500967E-11
**149**	3.461692E-14	2.4332298E-13	4.60768007E-12	7.4879853E-12	5.2059347E-11
**150**	3.592573E-14	2.5004375E-13	4.68855753E-12	7.57939199E-12	5.2167892E-11
**151**	3.726742E-14	2.5686846E-13	4.76992858E-12	7.67096817E-12	5.22757558E-11
**152**	3.864229E-14	2.6379703E-13	4.85178646E-12	7.76270925E-12	5.23829622E-11
**153**	4.005065E-14	2.7082933E-13	4.9341244E-12	7.85461073E-12	5.24895342E-11

**154**	4.14928E-14	2.7796523E-13	5.01693569E-12	7.94666816E-12	5.25954942E-11
**155**	4.296902E-14	2.8520456E-13	5.10021362E-12	8.03887717E-12	5.27008641E-11
**156**	4.447961E-14	2.9254714E-13	5.18395154E-12	8.13123343E-12	5.28056651E-11
**157**	4.602483E-14	2.9999276E-13	5.26814279E-12	8.22373267E-12	5.29099181E-11
**158**	4.760496E-14	3.0754121E-13	5.35278078E-12	8.31637067E-12	5.3013643E-11
**159**	4.922023E-14	3.1519225E-13	5.43785891E-12	8.40914329E-12	5.31168596E-11
**160**	5.087091E-14	3.229456E-13	5.52337064E-12	8.50204642E-12	5.32195868E-11
**161**	5.255724E-14	3.3080098E-13	5.60930944E-12	8.595076E-12	5.33218434E-11
**162**	5.427944E-14	3.387581E-13	5.69566883E-12	8.68822803E-12	5.34236472E-11
**163**	5.603774E-14	3.4681663E-13	5.78244234E-12	8.78149855E-12	5.35250158E-11
**164**	5.783236E-14	3.5497624E-13	5.86962354E-12	8.87488364E-12	5.36259663E-11
**165**	5.966349E-14	3.6323657E-13	5.95720603E-12	8.96837945E-12	5.37265153E-11
**166**	6.153134E-14	3.7159724E-13	6.04518344E-12	9.06198214E-12	5.38266787E-11
**167**	6.343609E-14	3.8005787E-13	6.13354943E-12	9.15568794E-12	5.39264722E-11
**168**	6.537792E-14	3.8861804E-13	6.22229769E-12	9.2494931E-12	5.4025911E-11
**169**	6.735701E-14	3.9727733E-13	6.31142195E-12	9.34339393E-12	5.41250098E-11
**170**	6.937351E-14	4.0603531E-13	6.40091596E-12	9.43738676E-12	5.42237829E-11
**171**	7.142759E-14	4.148915E-13	6.49077351E-12	9.53146796E-12	5.4322244E-11
**172**	7.351938E-14	4.2384544E-13	6.5809884E-12	9.62563395E-12	5.44204066E-11
**173**	7.564901E-14	4.3289665E-13	6.67155449E-12	9.71988118E-12	5.45182837E-11
**174**	7.781663E-14	4.4204461E-13	6.76246566E-12	9.81420611E-12	5.46158879E-11
**175**	8.002234E-14	4.5128882E-13	6.85371582E-12	9.90860527E-12	5.47132314E-11
**176**	8.226626E-14	4.6062873E-13	6.9452989E-12	1.00030752E-11	5.4810326E-11
**177**	8.454848E-14	4.7006381E-13	7.03720888E-12	1.00976125E-11	5.49071831E-11
**178**	8.686911E-14	4.7959349E-13	7.12943976E-12	1.01922137E-11	5.50038139E-11
**179**	8.922822E-14	4.892172E-13	7.22198558E-12	1.02868755E-11	5.51002289E-11
**180**	9.162589E-14	4.9893436E-13	7.3148404E-12	1.03815946E-11	5.51964385E-11
**181**	9.406219E-14	5.0874437E-13	7.40799831E-12	1.04763675E-11	5.52924527E-11
**182**	9.653718E-14	5.1864661E-13	7.50145345E-12	1.05711912E-11	5.53882812E-11
**183**	9.905091E-14	5.2864047E-13	7.59519998E-12	1.06660623E-11	5.54839331E-11
**184**	1.0160341E-13	5.3872531E-13	7.68923209E-12	1.07609775E-11	5.55794175E-11
**185**	1.0419473E-13	5.4890049E-13	7.78354399E-12	1.08559337E-11	5.56747431E-11
**186**	1.0682489E-13	5.5916535E-13	7.87812994E-12	1.09509277E-11	5.57699181E-11
**187**	1.0949391E-13	5.6951922E-13	7.97298422E-12	1.10459563E-11	5.58649506E-11
**188**	1.122018E-13	5.7996142E-13	8.06810116E-12	1.11410164E-11	5.59598482E-11
**189**	1.1494856E-13	5.9049128E-13	8.16347509E-12	1.12361049E-11	5.60546185E-11
**190**	1.1773418E-13	6.0110808E-13	8.25910039E-12	1.13312187E-11	5.61492685E-11
**191**	1.2055865E-13	6.1181113E-13	8.35497147E-12	1.14263548E-11	5.6243805E-11
**192**	1.2342196E-13	6.2259971E-13	8.45108277E-12	1.152151E-11	5.63382347E-11
**193**	1.2632406E-13	6.334731E-13	8.54742876E-12	1.16166814E-11	5.64325639E-11
**194**	1.2926493E-13	6.4443055E-13	8.64400395E-12	1.17118659E-11	5.65267984E-11
**195**	1.3224453E-13	6.5547134E-13	8.74080286E-12	1.18070607E-11	5.66209443E-11
**196**	1.3526278E-13	6.665947E-13	8.83782005E-12	1.19022626E-11	5.67150068E-11
**197**	1.3831966E-13	6.7779989E-13	8.93505013E-12	1.19974687E-11	5.68089914E-11
**198**	1.4141507E-13	6.8908614E-13	9.03248772E-12	1.20926762E-11	5.6902903E-11
**199**	1.4454896E-13	7.0045268E-13	9.13012748E-12	1.21878822E-11	5.69967464E-11
**200**	1.4772124E-13	7.1189873E-13	9.22796409E-12	1.22830836E-11	5.70905262E-11
**201**	1.5093183E-13	7.234235E-13	9.32599228E-12	1.23782777E-11	5.71842466E-11
**202**	1.5418062E-13	7.350262E-13	9.42420679E-12	1.24734616E-11	5.72779119E-11
**203**	1.5746753E-13	7.4670604E-13	9.52260241E-12	1.25686325E-11	5.73715259E-11
**204**	1.6079245E-13	7.5846222E-13	9.62117395E-12	1.26637875E-11	5.74650923E-11

**205**	1.6415526E-13	7.7029392E-13	9.71991625E-12	1.27589239E-11	5.75586145E-11
**206**	1.6755584E-13	7.8220033E-13	9.81882419E-12	1.28540388E-11	5.76520958E-11
**207**	1.7099407E-13	7.9418063E-13	9.91789268E-12	1.29491295E-11	5.77455393E-11
**208**	1.7446981E-13	8.0623401E-13	1.00171167E-11	1.30441932E-11	5.78389479E-11
**209**	1.7798293E-13	8.1835962E-13	1.01164911E-11	1.31392272E-11	5.79323243E-11
**210**	1.8153328E-13	8.3055665E-13	1.0216011E-11	1.32342288E-11	5.8025671E-11
**211**	1.8512072E-13	8.4282425E-13	1.03156713E-11	1.33291953E-11	5.81189903E-11
**212**	1.8874508E-13	8.5516158E-13	1.04154673E-11	1.34241239E-11	5.82122844E-11
**213**	1.9240621E-13	8.675678E-13	1.05153938E-11	1.35190121E-11	5.83055554E-11
**214**	1.9610394E-13	8.8004207E-13	1.06154462E-11	1.36138571E-11	5.8398805E-11
**215**	1.998381E-13	8.9258354E-13	1.07156194E-11	1.37086564E-11	5.84920349E-11
**216**	2.0360851E-13	9.0519134E-13	1.08159088E-11	1.38034072E-11	5.85852466E-11
**217**	2.0741499E-13	9.1786463E-13	1.09163096E-11	1.3898107E-11	5.86784416E-11
**218**	2.1125735E-13	9.3060256E-13	1.10168169E-11	1.39927532E-11	5.87716209E-11
**219**	2.1513539E-13	9.4340425E-13	1.11174261E-11	1.40873433E-11	5.88647858E-11
**220**	2.1904893E-13	9.5626886E-13	1.12181325E-11	1.41818745E-11	5.89579371E-11
**221**	2.2299775E-13	9.691955E-13	1.13189315E-11	1.42763445E-11	5.90510756E-11
**222**	2.2698166E-13	9.8218333E-13	1.14198183E-11	1.43707507E-11	5.91442021E-11
**223**	2.3100043E-13	9.9523148E-13	1.15207885E-11	1.44650906E-11	5.92373169E-11
**224**	2.3505387E-13	1.00833907E-12	1.16218375E-11	1.45593616E-11	5.93304207E-11
**225**	2.3914173E-13	1.02150525E-12	1.17229608E-11	1.46535614E-11	5.94235135E-11
**226**	2.4326381E-13	1.03472913E-12	1.18241538E-11	1.47476873E-11	5.95165957E-11
**227**	2.4741987E-13	1.04800986E-12	1.19254121E-11	1.4841737E-11	5.96096673E-11
**228**	2.5160968E-13	1.06134656E-12	1.20267313E-11	1.49357081E-11	5.97027282E-11
**229**	2.5583301E-13	1.07473836E-12	1.2128107E-11	1.50295981E-11	5.97957783E-11
**230**	2.6008961E-13	1.08818439E-12	1.22295348E-11	1.51234046E-11	5.98888172E-11
**231**	2.6437924E-13	1.10168378E-12	1.23310104E-11	1.52171253E-11	5.99818448E-11
**232**	2.6870166E-13	1.11523567E-12	1.24325295E-11	1.53107577E-11	6.00748604E-11
**233**	2.7305661E-13	1.12883918E-12	1.25340877E-11	1.54042996E-11	6.01678635E-11
**234**	2.7744384E-13	1.14249344E-12	1.2635681E-11	1.54977485E-11	6.02608535E-11
**235**	2.818631E-13	1.15619759E-12	1.27373051E-11	1.55911022E-11	6.03538296E-11
**236**	2.8631412E-13	1.16995076E-12	1.28389557E-11	1.56843583E-11	6.04467911E-11
**237**	2.9079664E-13	1.18375209E-12	1.29406289E-11	1.57775145E-11	6.05397369E-11
**238**	2.9531039E-13	1.19760071E-12	1.30423204E-11	1.58705687E-11	6.06326661E-11
**239**	2.9985511E-13	1.21149575E-12	1.31440262E-11	1.59635185E-11	6.07255777E-11
**240**	3.0443052E-13	1.22543636E-12	1.32457422E-11	1.60563616E-11	6.08184705E-11
**241**	3.0903636E-13	1.23942168E-12	1.33474645E-11	1.6149096E-11	6.09113433E-11
**242**	3.1367234E-13	1.25345084E-12	1.34491891E-11	1.62417193E-11	6.10041947E-11
**243**	3.1833818E-13	1.267523E-12	1.3550912E-11	1.63342293E-11	6.10970235E-11
**244**	3.230336E-13	1.2816373E-12	1.36526293E-11	1.6426624E-11	6.11898282E-11
**245**	3.2775833E-13	1.29579289E-12	1.37543372E-11	1.65189011E-11	6.12826073E-11
**246**	3.3251207E-13	1.30998891E-12	1.38560318E-11	1.66110584E-11	6.13753594E-11
**247**	3.3729453E-13	1.32422453E-12	1.39577092E-11	1.6703094E-11	6.14680827E-11
**248**	3.4210543E-13	1.3384989E-12	1.40593657E-11	1.67950056E-11	6.15607757E-11
**249**	3.4694447E-13	1.35281117E-12	1.41609976E-11	1.68867912E-11	6.16534365E-11
**250**	3.5181136E-13	1.36716051E-12	1.42626011E-11	1.69784487E-11	6.17460636E-11
**251**	3.5670581E-13	1.38154609E-12	1.43641726E-11	1.70699761E-11	6.1838655E-11
**252**	3.6162752E-13	1.39596706E-12	1.44657084E-11	1.71613713E-11	6.19312089E-11
**253**	3.6657619E-13	1.41042261E-12	1.45672048E-11	1.72526322E-11	6.20237234E-11
**254**	3.7155152E-13	1.4249119E-12	1.46686584E-11	1.7343757E-11	6.21161965E-11
**255**	3.765532E-13	1.43943412E-12	1.47700655E-11	1.74347436E-11	6.22086263E-11

**256**	3.8158095E-13	1.45398843E-12	1.48714226E-11	1.752559E-11	6.23010107E-11
**257**	3.8663444E-13	1.46857404E-12	1.49727262E-11	1.76162943E-11	6.23933477E-11
**258**	3.9171339E-13	1.48319012E-12	1.5073973E-11	1.77068545E-11	6.24856351E-11
**259**	3.9681748E-13	1.49783587E-12	1.51751594E-11	1.77972688E-11	6.25778709E-11
**260**	4.0194641E-13	1.51251048E-12	1.5276282E-11	1.78875352E-11	6.26700528E-11
**261**	4.0709986E-13	1.52721316E-12	1.53773376E-11	1.79776519E-11	6.27621787E-11
**262**	4.1227754E-13	1.54194309E-12	1.54783227E-11	1.8067617E-11	6.28542463E-11
**263**	4.1747912E-13	1.5566995E-12	1.55792342E-11	1.81574286E-11	6.29462534E-11
**264**	4.2270431E-13	1.5714816E-12	1.56800686E-11	1.8247085E-11	6.30381977E-11
**265**	4.2795279E-13	1.58628859E-12	1.57808229E-11	1.83365843E-11	6.31300769E-11
**266**	4.3322425E-13	1.60111971E-12	1.58814937E-11	1.84259247E-11	6.32218887E-11
**267**	4.3851837E-13	1.61597417E-12	1.5982078E-11	1.85151045E-11	6.33136306E-11
**268**	4.4383486E-13	1.6308512E-12	1.60825725E-11	1.86041219E-11	6.34053005E-11
**269**	4.4917339E-13	1.64575003E-12	1.61829743E-11	1.86929751E-11	6.34968958E-11
**270**	4.5453365E-13	1.66066991E-12	1.62832802E-11	1.87816625E-11	6.35884142E-11
**271**	4.5991534E-13	1.67561008E-12	1.63834871E-11	1.88701823E-11	6.36798532E-11
**272**	4.6531813E-13	1.69056977E-12	1.64835922E-11	1.8958533E-11	6.37712104E-11
**273**	4.7074171E-13	1.70554825E-12	1.65835923E-11	1.90467127E-11	6.38624835E-11
**274**	4.7618578E-13	1.72054477E-12	1.66834846E-11	1.91347198E-11	6.39536698E-11
**275**	4.8165002E-13	1.7355586E-12	1.67832661E-11	1.92225528E-11	6.4044767E-11
**276**	4.8713411E-13	1.75058898E-12	1.68829341E-11	1.931021E-11	6.41357725E-11
**277**	4.9263775E-13	1.76563521E-12	1.69824855E-11	1.93976898E-11	6.42266839E-11
**278**	4.9816063E-13	1.78069655E-12	1.70819176E-11	1.94849907E-11	6.43174987E-11
**279**	5.0370242E-13	1.79577229E-12	1.71812277E-11	1.95721111E-11	6.44082145E-11
**280**	5.0926282E-13	1.8108617E-12	1.72804128E-11	1.96590494E-11	6.44988285E-11
**281**	5.1484153E-13	1.82596408E-12	1.73794705E-11	1.97458042E-11	6.45893385E-11
**282**	5.2043821E-13	1.84107874E-12	1.74783978E-11	1.98323739E-11	6.46797419E-11
**283**	5.2605258E-13	1.85620495E-12	1.75771923E-11	1.99187571E-11	6.47700361E-11
**284**	5.3168431E-13	1.87134205E-12	1.76758512E-11	2.00049522E-11	6.48602186E-11
**285**	5.3733311E-13	1.88648932E-12	1.77743719E-11	2.0090958E-11	6.4950287E-11
**286**	5.4299865E-13	1.9016461E-12	1.78727519E-11	2.01767728E-11	6.50402387E-11
**287**	5.4868063E-13	1.9168117E-12	1.79709886E-11	2.02623953E-11	6.51300711E-11
**288**	5.5437875E-13	1.93198546E-12	1.80690796E-11	2.03478242E-11	6.52197819E-11
**289**	5.600927E-13	1.94716669E-12	1.81670223E-11	2.0433058E-11	6.53093685E-11
**290**	5.6582217E-13	1.96235474E-12	1.82648143E-11	2.05180954E-11	6.53988283E-11
**291**	5.7156686E-13	1.97754896E-12	1.83624531E-11	2.0602935E-11	6.5488159E-11
**292**	5.7732646E-13	1.99274869E-12	1.84599365E-11	2.06875756E-11	6.55773579E-11
**293**	5.8310068E-13	2.00795329E-12	1.85572619E-11	2.07720158E-11	6.56664226E-11
**294**	5.8888921E-13	2.0231621E-12	1.86544271E-11	2.08562543E-11	6.57553507E-11
**295**	5.9469174E-13	2.03837451E-12	1.87514299E-11	2.09402899E-11	6.58441396E-11
**296**	6.0050799E-13	2.05358987E-12	1.88482678E-11	2.10241214E-11	6.59327869E-11
**297**	6.0633764E-13	2.06880757E-12	1.89449387E-11	2.11077474E-11	6.60212902E-11
**298**	6.1218041E-13	2.08402699E-12	1.90414404E-11	2.11911669E-11	6.6109647E-11
**299**	6.18036E-13	2.0992475E-12	1.91377707E-11	2.12743785E-11	6.61978548E-11
**300**	6.239041E-13	2.1144685E-12	1.92339273E-11	2.13573812E-11	6.62859112E-11
**301**	6.2978443E-13	2.1296894E-12	1.93299083E-11	2.14401737E-11	6.63738139E-11
**302**	6.356767E-13	2.14490959E-12	1.94257115E-11	2.1522755E-11	6.64615604E-11
**303**	6.415806E-13	2.16012847E-12	1.95213347E-11	2.16051238E-11	6.65491484E-11
**304**	6.4749586E-13	2.17534547E-12	1.96167761E-11	2.16872792E-11	6.66365754E-11
**305**	6.5342218E-13	2.19056001E-12	1.97120335E-11	2.17692199E-11	6.67238392E-11
**306**	6.5935928E-13	2.2057715E-12	1.9807105E-11	2.1850945E-11	6.68109372E-11

**307**	6.6530686E-13	2.22097937E-12	1.99019886E-11	2.19324533E-11	6.68978674E-11
**308**	6.7126465E-13	2.23618307E-12	1.99966824E-11	2.20137439E-11	6.69846272E-11
**309**	6.7723236E-13	2.25138203E-12	2.00911845E-11	2.20948157E-11	6.70712144E-11
**310**	6.8320971E-13	2.2665757E-12	2.0185493E-11	2.21756678E-11	6.71576267E-11
**311**	6.8919641E-13	2.28176354E-12	2.02796061E-11	2.2256299E-11	6.72438619E-11
**312**	6.9519219E-13	2.29694499E-12	2.03735219E-11	2.23367086E-11	6.73299176E-11
**313**	7.0119677E-13	2.31211952E-12	2.04672387E-11	2.24168954E-11	6.74157917E-11
**314**	7.0720988E-13	2.3272866E-12	2.05607547E-11	2.24968586E-11	6.75014819E-11
**315**	7.1323124E-13	2.3424457E-12	2.0654068E-11	2.25765973E-11	6.7586986E-11
**316**	7.1926057E-13	2.35759631E-12	2.07471772E-11	2.26561106E-11	6.76723019E-11
**317**	7.2529761E-13	2.3727379E-12	2.08400803E-11	2.27353976E-11	6.77574273E-11
**318**	7.3134209E-13	2.38786997E-12	2.09327758E-11	2.28144573E-11	6.78423602E-11
**319**	7.3739373E-13	2.40299201E-12	2.10252621E-11	2.2893289E-11	6.79270983E-11
**320**	7.4345227E-13	2.41810352E-12	2.11175375E-11	2.29718919E-11	6.80116396E-11
**321**	7.4951746E-13	2.43320401E-12	2.12096004E-11	2.30502651E-11	6.80959819E-11
**322**	7.5558901E-13	2.44829299E-12	2.13014493E-11	2.31284077E-11	6.81801233E-11
**323**	7.6166668E-13	2.46336998E-12	2.13930827E-11	2.32063191E-11	6.82640615E-11
**324**	7.677502E-13	2.47843449E-12	2.1484499E-11	2.32839985E-11	6.83477947E-11
**325**	7.7383932E-13	2.49348606E-12	2.15756968E-11	2.3361445E-11	6.84313208E-11
**326**	7.7993377E-13	2.50852421E-12	2.16666745E-11	2.3438658E-11	6.85146377E-11
**327**	7.8603331E-13	2.5235485E-12	2.17574308E-11	2.35156368E-11	6.85977436E-11
**328**	7.9213767E-13	2.53855845E-12	2.18479643E-11	2.35923806E-11	6.86806364E-11
**329**	7.9824662E-13	2.55355362E-12	2.19382736E-11	2.36688888E-11	6.87633143E-11
**330**	8.043599E-13	2.56853355E-12	2.20283572E-11	2.37451606E-11	6.88457752E-11
**331**	8.1047726E-13	2.58349782E-12	2.21182139E-11	2.38211954E-11	6.89280174E-11
**332**	8.1659846E-13	2.59844598E-12	2.22078424E-11	2.38969926E-11	6.90100389E-11
**333**	8.2272325E-13	2.61337761E-12	2.22972413E-11	2.39725516E-11	6.90918379E-11
**334**	8.288514E-13	2.62829227E-12	2.23864095E-11	2.40478716E-11	6.91734126E-11
**335**	8.3498266E-13	2.64318954E-12	2.24753456E-11	2.41229522E-11	6.92547611E-11
**336**	8.4111679E-13	2.65806902E-12	2.25640484E-11	2.41977928E-11	6.93358817E-11
**337**	8.4725355E-13	2.67293029E-12	2.26525167E-11	2.42723927E-11	6.94167725E-11
**338**	8.5339272E-13	2.68777294E-12	2.27407494E-11	2.43467515E-11	6.9497432E-11
**339**	8.5953405E-13	2.70259657E-12	2.28287454E-11	2.44208685E-11	6.95778582E-11
**340**	8.6567733E-13	2.71740079E-12	2.29165034E-11	2.44947433E-11	6.96580497E-11
**341**	8.7182231E-13	2.73218521E-12	2.30040223E-11	2.45683754E-11	6.97380045E-11
**342**	8.7796877E-13	2.74694945E-12	2.30913012E-11	2.46417642E-11	6.98177212E-11
**343**	8.8411649E-13	2.76169311E-12	2.31783388E-11	2.47149093E-11	6.9897198E-11
**344**	8.9026525E-13	2.77641583E-12	2.32651343E-11	2.47878102E-11	6.99764334E-11
**345**	8.9641481E-13	2.79111723E-12	2.33516865E-11	2.48604664E-11	7.00554257E-11
**346**	9.0256496E-13	2.80579696E-12	2.34379944E-11	2.49328776E-11	7.01341734E-11
**347**	9.0871548E-13	2.82045464E-12	2.35240572E-11	2.50050433E-11	7.0212675E-11
**348**	9.1486616E-13	2.83508992E-12	2.36098737E-11	2.5076963E-11	7.02909289E-11
**349**	9.2101679E-13	2.84970245E-12	2.36954432E-11	2.51486364E-11	7.03689336E-11
**350**	9.2716714E-13	2.86429188E-12	2.37807646E-11	2.52200632E-11	7.04466877E-11
**351**	9.3331701E-13	2.87885788E-12	2.38658371E-11	2.52912428E-11	7.05241896E-11
**352**	9.3946619E-13	2.89340009E-12	2.39506598E-11	2.53621751E-11	7.0601438E-11
**353**	9.4561447E-13	2.9079182E-12	2.40352318E-11	2.54328595E-11	7.06784314E-11
**354**	9.5176165E-13	2.92241186E-12	2.41195523E-11	2.55032959E-11	7.07551684E-11
**355**	9.5790753E-13	2.93688077E-12	2.42036206E-11	2.55734839E-11	7.08316477E-11
**356**	9.640519E-13	2.95132459E-12	2.42874357E-11	2.56434231E-11	7.09078679E-11
**357**	9.7019457E-13	2.96574301E-12	2.4370997E-11	2.57131133E-11	7.09838277E-11

**358**	9.7633534E-13	2.98013573E-12	2.44543036E-11	2.57825542E-11	7.10595257E-11
**359**	9.82474E-13	2.99450243E-12	2.45373548E-11	2.58517455E-11	7.11349608E-11
**360**	9.8861038E-13	3.00884282E-12	2.462015E-11	2.59206871E-11	7.12101316E-11
**361**	9.9474427E-13	3.0231566E-12	2.47026883E-11	2.59893785E-11	7.12850369E-11
**362**	1.00087548E-12	3.03744348E-12	2.47849692E-11	2.60578197E-11	7.13596755E-11
**363**	1.00700384E-12	3.05170316E-12	2.48669919E-11	2.61260104E-11	7.14340462E-11
**364**	1.01312914E-12	3.06593537E-12	2.49487559E-11	2.61939503E-11	7.15081478E-11
**365**	1.01925122E-12	3.08013982E-12	2.50302604E-11	2.62616394E-11	7.15819791E-11
**366**	1.02536987E-12	3.09431625E-12	2.51115049E-11	2.63290774E-11	7.1655539E-11
**367**	1.03148494E-12	3.10846437E-12	2.51924889E-11	2.63962641E-11	7.17288265E-11
**368**	1.03759622E-12	3.12258392E-12	2.52732116E-11	2.64631994E-11	7.18018404E-11
**369**	1.04370356E-12	3.13667464E-12	2.53536726E-11	2.65298831E-11	7.18745796E-11
**370**	1.04980676E-12	3.15073628E-12	2.54338712E-11	2.65963151E-11	7.19470431E-11
**371**	1.05590567E-12	3.16476856E-12	2.55138071E-11	2.66624953E-11	7.20192299E-11
**372**	1.0620001E-12	3.17877126E-12	2.55934797E-11	2.67284236E-11	7.20911389E-11
**373**	1.06808989E-12	3.19274411E-12	2.56728885E-11	2.67940998E-11	7.21627693E-11
**374**	1.07417487E-12	3.20668688E-12	2.5752033E-11	2.68595239E-11	7.22341199E-11
**375**	1.08025487E-12	3.22059932E-12	2.58309127E-11	2.69246958E-11	7.23051899E-11
**376**	1.08632973E-12	3.23448121E-12	2.59095273E-11	2.69896154E-11	7.23759784E-11
**377**	1.09239928E-12	3.24833232E-12	2.59878764E-11	2.70542827E-11	7.24464844E-11
**378**	1.09846337E-12	3.26215241E-12	2.60659594E-11	2.71186976E-11	7.2516707E-11
**379**	1.10452184E-12	3.27594126E-12	2.6143776E-11	2.718286E-11	7.25866455E-11
**380**	1.11057452E-12	3.28969866E-12	2.62213258E-11	2.724677E-11	7.26562989E-11
**381**	1.11662127E-12	3.30342438E-12	2.62986085E-11	2.73104276E-11	7.27256664E-11
**382**	1.12266192E-12	3.31711823E-12	2.63756237E-11	2.73738326E-11	7.27947472E-11
**383**	1.12869633E-12	3.33077998E-12	2.64523711E-11	2.74369852E-11	7.28635406E-11
**384**	1.13472434E-12	3.34440944E-12	2.65288504E-11	2.74998853E-11	7.29320458E-11
**385**	1.14074582E-12	3.35800641E-12	2.66050613E-11	2.7562533E-11	7.30002619E-11
**386**	1.1467606E-12	3.37157068E-12	2.66810034E-11	2.76249282E-11	7.30681884E-11
**387**	1.15276855E-12	3.38510208E-12	2.67566765E-11	2.76870711E-11	7.31358245E-11
**388**	1.15876952E-12	3.39860039E-12	2.68320804E-11	2.77489617E-11	7.32031694E-11
**389**	1.16476336E-12	3.41206545E-12	2.69072148E-11	2.78106E-11	7.32702226E-11
**390**	1.17074995E-12	3.42549706E-12	2.69820795E-11	2.78719861E-11	7.33369834E-11
**391**	1.17672914E-12	3.43889506E-12	2.70566743E-11	2.79331202E-11	7.34034511E-11
**392**	1.18270079E-12	3.45225925E-12	2.71309989E-11	2.79940021E-11	7.34696251E-11
**393**	1.18866476E-12	3.46558948E-12	2.72050531E-11	2.80546322E-11	7.35355049E-11
**394**	1.19462094E-12	3.47888557E-12	2.72788369E-11	2.81150105E-11	7.36010898E-11
**395**	1.20056917E-12	3.49214735E-12	2.735235E-11	2.8175137E-11	7.36663794E-11
**396**	1.20650934E-12	3.50537467E-12	2.74255923E-11	2.8235012E-11	7.3731373E-11
**397**	1.21244131E-12	3.51856736E-12	2.74985636E-11	2.82946355E-11	7.37960701E-11
**398**	1.21836496E-12	3.53172528E-12	2.75712638E-11	2.83540077E-11	7.38604702E-11
**399**	1.22428016E-12	3.54484826E-12	2.76436929E-11	2.84131287E-11	7.39245729E-11
**400**	1.23018678E-12	3.55793616E-12	2.77158506E-11	2.84719987E-11	7.39883777E-11
**401**	1.23608471E-12	3.57098883E-12	2.7787737E-11	2.85306179E-11	7.40518841E-11
**402**	1.24197383E-12	3.58400613E-12	2.78593519E-11	2.85889864E-11	7.41150916E-11
**403**	1.24785401E-12	3.59698792E-12	2.79306953E-11	2.86471044E-11	7.41779999E-11
**404**	1.25372514E-12	3.60993407E-12	2.80017672E-11	2.87049722E-11	7.42406086E-11
**405**	1.25958709E-12	3.62284443E-12	2.80725674E-11	2.87625898E-11	7.43029172E-11
**406**	1.26543977E-12	3.63571889E-12	2.8143096E-11	2.88199575E-11	7.43649254E-11
**407**	1.27128305E-12	3.6485573E-12	2.8213353E-11	2.88770755E-11	7.44266329E-11
**408**	1.27711682E-12	3.66135955E-12	2.82833383E-11	2.8933944E-11	7.44880392E-11

**409**	1.28294097E-12	3.67412552E-12	2.8353052E-11	2.89905633E-11	7.45491442E-11
**410**	1.2887554E-12	3.68685508E-12	2.8422494E-11	2.90469336E-11	7.46099474E-11
**411**	1.29455999E-12	3.69954813E-12	2.84916644E-11	2.91030551E-11	7.46704486E-11
**412**	1.30035464E-12	3.71220454E-12	2.85605633E-11	2.91589281E-11	7.47306475E-11
**413**	1.30613926E-12	3.72482421E-12	2.86291907E-11	2.92145528E-11	7.47905438E-11
**414**	1.31191372E-12	3.73740703E-12	2.86975466E-11	2.92699295E-11	7.48501374E-11
**415**	1.31767794E-12	3.74995289E-12	2.87656311E-11	2.93250585E-11	7.4909428E-11
**416**	1.32343182E-12	3.7624617E-12	2.88334444E-11	2.937994E-11	7.49684153E-11
**417**	1.32917525E-12	3.77493336E-12	2.89009864E-11	2.94345743E-11	7.50270992E-11
**418**	1.33490813E-12	3.78736776E-12	2.89682574E-11	2.94889617E-11	7.50854794E-11
**419**	1.34063039E-12	3.79976481E-12	2.90352573E-11	2.95431025E-11	7.51435559E-11
**420**	1.34634191E-12	3.81212443E-12	2.91019864E-11	2.9596997E-11	7.52013285E-11
**421**	1.3520426E-12	3.82444652E-12	2.91684447E-11	2.96506456E-11	7.5258797E-11
**422**	1.35773239E-12	3.836731E-12	2.92346325E-11	2.97040484E-11	7.53159612E-11
**423**	1.36341116E-12	3.84897778E-12	2.93005498E-11	2.97572059E-11	7.53728212E-11
**424**	1.36907885E-12	3.86118679E-12	2.93661968E-11	2.98101183E-11	7.54293767E-11
**425**	1.37473535E-12	3.87335794E-12	2.94315737E-11	2.98627861E-11	7.54856278E-11
**426**	1.38038059E-12	3.88549116E-12	2.94966806E-11	2.99152094E-11	7.55415743E-11
**427**	1.38601448E-12	3.89758638E-12	2.95615178E-11	2.99673888E-11	7.55972161E-11
**428**	1.39163693E-12	3.90964352E-12	2.96260854E-11	3.00193245E-11	7.56525533E-11
**429**	1.39724787E-12	3.92166252E-12	2.96903837E-11	3.00710168E-11	7.57075858E-11
**430**	1.40284721E-12	3.9336433E-12	2.97544128E-11	3.01224662E-11	7.57623136E-11
**431**	1.40843488E-12	3.94558581E-12	2.98181729E-11	3.0173673E-11	7.58167367E-11
**432**	1.41401079E-12	3.95748999E-12	2.98816644E-11	3.02246376E-11	7.5870855E-11
**433**	1.41957487E-12	3.96935577E-12	2.99448874E-11	3.02753604E-11	7.59246687E-11
**434**	1.42512704E-12	3.98118309E-12	3.00078422E-11	3.03258416E-11	7.59781777E-11
**435**	1.43066724E-12	3.9929719E-12	3.0070529E-11	3.03760818E-11	7.6031382E-11
**436**	1.43619538E-12	4.00472216E-12	3.01329482E-11	3.04260813E-11	7.60842819E-11
**437**	1.4417114E-12	4.0164338E-12	3.01950999E-11	3.04758405E-11	7.61368772E-11
**438**	1.44721522E-12	4.02810678E-12	3.02569844E-11	3.05253598E-11	7.61891682E-11
**439**	1.45270677E-12	4.03974105E-12	3.0318602E-11	3.05746397E-11	7.62411548E-11
**440**	1.45818599E-12	4.05133658E-12	3.03799531E-11	3.06236804E-11	7.62928373E-11
**441**	1.46365282E-12	4.06289331E-12	3.04410379E-11	3.06724825E-11	7.63442156E-11
**442**	1.46910717E-12	4.0744112E-12	3.05018567E-11	3.07210464E-11	7.639529E-11
**443**	1.474549E-12	4.08589023E-12	3.05624098E-11	3.07693724E-11	7.64460606E-11
**444**	1.47997823E-12	4.09733035E-12	3.06226976E-11	3.0817461E-11	7.64965276E-11
**445**	1.4853948E-12	4.10873152E-12	3.06827203E-11	3.08653127E-11	7.6546691E-11
**446**	1.49079866E-12	4.12009373E-12	3.07424784E-11	3.09129279E-11	7.65965511E-11
**447**	1.49618973E-12	4.13141692E-12	3.08019721E-11	3.0960307E-11	7.6646108E-11
**448**	1.50156797E-12	4.14270109E-12	3.08612019E-11	3.10074505E-11	7.6695362E-11
**449**	1.50693332E-12	4.1539462E-12	3.09201679E-11	3.10543588E-11	7.67443133E-11
**450**	1.51228572E-12	4.16515223E-12	3.09788707E-11	3.11010324E-11	7.6792962E-11
**451**	1.5176251E-12	4.17631915E-12	3.10373106E-11	3.11474717E-11	7.68413084E-11
**452**	1.52295143E-12	4.18744694E-12	3.10954879E-11	3.11936772E-11	7.68893527E-11
**453**	1.52826464E-12	4.1985356E-12	3.1153403E-11	3.12396494E-11	7.69370953E-11
**454**	1.53356468E-12	4.20958508E-12	3.12110563E-11	3.12853887E-11	7.69845362E-11
**455**	1.53885151E-12	4.2205954E-12	3.12684482E-11	3.13308956E-11	7.70316758E-11
**456**	1.54412506E-12	4.23156652E-12	3.13255792E-11	3.13761705E-11	7.70785145E-11
**457**	1.54938529E-12	4.24249844E-12	3.13824495E-11	3.14212141E-11	7.71250523E-11
**458**	1.55463216E-12	4.25339114E-12	3.14390596E-11	3.14660267E-11	7.71712897E-11
**459**	1.55986562E-12	4.26424463E-12	3.149541E-11	3.15106088E-11	7.7217227E-11

**460**	1.56508561E-12	4.27505888E-12	3.1551501E-11	3.15549609E-11	7.72628644E-11
**461**	1.57029209E-12	4.2858339E-12	3.1607333E-11	3.15990835E-11	7.73082023E-11
**462**	1.57548503E-12	4.29656969E-12	3.16629065E-11	3.16429772E-11	7.7353241E-11
**463**	1.58066437E-12	4.30726623E-12	3.1718222E-11	3.16866424E-11	7.73979809E-11
**464**	1.58583008E-12	4.31792354E-12	3.17732798E-11	3.17300795E-11	7.74424222E-11
**465**	1.59098211E-12	4.32854161E-12	3.18280804E-11	3.17732892E-11	7.74865654E-11
**466**	1.59612042E-12	4.33912044E-12	3.18826243E-11	3.18162719E-11	7.75304109E-11
**467**	1.60124497E-12	4.34966004E-12	3.19369119E-11	3.18590282E-11	7.75739589E-11
**468**	1.60635573E-12	4.36016041E-12	3.19909436E-11	3.19015585E-11	7.76172099E-11
**469**	1.61145265E-12	4.37062157E-12	3.204472E-11	3.19438634E-11	7.76601642E-11
**470**	1.61653571E-12	4.38104352E-12	3.20982415E-11	3.19859434E-11	7.77028223E-11
**471**	1.62160485E-12	4.39142626E-12	3.21515085E-11	3.2027799E-11	7.77451845E-11
**472**	1.62666006E-12	4.40176982E-12	3.22045216E-11	3.20694307E-11	7.77872513E-11
**473**	1.63170129E-12	4.4120742E-12	3.22572812E-11	3.21108391E-11	7.78290231E-11
**474**	1.63672851E-12	4.42233942E-12	3.23097878E-11	3.21520247E-11	7.78705003E-11
**475**	1.64174169E-12	4.4325655E-12	3.23620419E-11	3.2192988E-11	7.79116834E-11
**476**	1.6467408E-12	4.44275245E-12	3.24140441E-11	3.22337296E-11	7.79525727E-11
**477**	1.6517258E-12	4.45290028E-12	3.24657947E-11	3.227425E-11	7.79931688E-11
**478**	1.65669667E-12	4.46300903E-12	3.25172943E-11	3.23145498E-11	7.80334721E-11
**479**	1.66165338E-12	4.47307871E-12	3.25685434E-11	3.23546294E-11	7.8073483E-11
**480**	1.6665959E-12	4.48310934E-12	3.26195425E-11	3.23944895E-11	7.81132021E-11
**481**	1.6715242E-12	4.49310095E-12	3.26702922E-11	3.24341306E-11	7.81526297E-11
**482**	1.67643826E-12	4.50305355E-12	3.27207929E-11	3.24735531E-11	7.81917665E-11
**483**	1.68133805E-12	4.51296719E-12	3.27710451E-11	3.25127578E-11	7.82306128E-11
**484**	1.68622355E-12	4.52284188E-12	3.28210495E-11	3.25517451E-11	7.82691692E-11
**485**	1.69109472E-12	4.53267765E-12	3.28708064E-11	3.25905155E-11	7.83074361E-11
**486**	1.69595156E-12	4.54247453E-12	3.29203165E-11	3.26290698E-11	7.83454142E-11
**487**	1.70079403E-12	4.55223256E-12	3.29695803E-11	3.26674083E-11	7.83831038E-11
**488**	1.70562211E-12	4.56195176E-12	3.30185983E-11	3.27055316E-11	7.84205055E-11
**489**	1.71043579E-12	4.57163217E-12	3.30673711E-11	3.27434404E-11	7.84576199E-11
**490**	1.71523504E-12	4.58127382E-12	3.31158991E-11	3.27811352E-11	7.84944475E-11
**491**	1.72001984E-12	4.59087674E-12	3.3164183E-11	3.28186166E-11	7.85309887E-11
**492**	1.72479018E-12	4.60044099E-12	3.32122233E-11	3.2855885E-11	7.85672442E-11
**493**	1.72954603E-12	4.60996658E-12	3.32600206E-11	3.28929412E-11	7.86032146E-11
**494**	1.73428738E-12	4.61945356E-12	3.33075754E-11	3.29297856E-11	7.86389002E-11
**495**	1.73901422E-12	4.62890197E-12	3.33548882E-11	3.29664188E-11	7.86743018E-11
**496**	1.74372651E-12	4.63831184E-12	3.34019597E-11	3.30028414E-11	7.87094199E-11
**497**	1.74842426E-12	4.64768323E-12	3.34487903E-11	3.3039054E-11	7.8744255E-11
**498**	1.75310745E-12	4.65701617E-12	3.34953807E-11	3.30750572E-11	7.87788078E-11
**499**	1.75777606E-12	4.66631071E-12	3.35417315E-11	3.31108514E-11	7.88130787E-11
**500**	1.76243007E-12	4.67556689E-12	3.35878431E-11	3.31464374E-11	7.88470685E-11

Inelastic collisions' Rate Coefficients: Pseudo-Singlet State					
**Initial rotational state: j=1**					
T(K)	k ($cm^3 mol^{-1} s^{-1}$)				
	**Final State: j'=5**	**Final State: j'=4**	**Final State: j'=3**	**Final State: j'=2**	**Final State: j'=0**
**5**	1.28118621E-61	8.16892992E-44	3.03351523E-29	1.88593678E-18	2.60858733E-11
**6**	1.19160421E-53	1.07734091E-38	2.09928483E-26	2.49072836E-17	2.57460399E-11
**7**	5.87455546E-48	4.89191158E-35	2.2391286E-24	1.56835441E-16	2.52930777E-11
**8**	1.09680628E-43	2.71026151E-32	7.43153061E-23	6.22480427E-16	2.48193504E-11
**9**	2.31180436E-40	3.69939622E-30	1.13368994E-21	1.81813375E-15	2.43685113E-11
**10**	1.0592255E-37	1.89597025E-28	1.00439793E-20	4.28761442E-15	2.39584891E-11
**11**	1.60056128E-35	4.76943381E-27	5.99645694E-20	8.65888974E-15	2.35944839E-11
**12**	1.05305868E-33	7.03934928E-26	2.66361813E-19	1.55727862E-14	2.32757303E-11
**13**	3.65616292E-32	6.89645646E-25	9.42718745E-19	2.56251463E-14	2.29988938E-11
**14**	7.68432241E-31	4.89770048E-24	2.79156302E-18	3.93299454E-14	2.27597361E-11
**15**	1.08115395E-29	2.68935925E-23	7.16824341E-18	5.71019542E-14	2.2553908E-11
**16**	1.09805239E-28	1.19836073E-22	1.63954394E-17	7.92539298E-14	2.23773063E-11
**17**	8.52786872E-28	4.49619081E-22	3.40936072E-17	1.06002979E-13	2.22262149E-11
**18**	5.29653297E-27	1.46177496E-21	6.54896132E-17	1.37481762E-13	2.20973398E-11
**19**	2.72534062E-26	4.21233056E-21	1.17671316E-16	1.73751546E-13	2.1987796E-11
**20**	1.19506862E-25	1.0955327E-20	1.99766239E-16	2.14815251E-13	2.18950726E-11
**21**	4.56907516E-25	2.60941143E-20	3.23034111E-16	2.60629474E-13	2.18169911E-11
**22**	1.55180574E-24	5.76060887E-20	5.00873343E-16	3.1111504E-13	2.17516642E-11
**23**	4.7546083E-24	1.19039481E-19	7.48749215E-16	3.66165963E-13	2.16974562E-11
**24**	1.33117691E-23	2.3215716E-19	1.08405632E-15	4.25656858E-13	2.16529489E-11
**25**	3.44255697E-23	4.30269445E-19	1.5259301E-15	4.89448978E-13	2.16169108E-11
**26**	8.29869254E-23	7.62259981E-19	2.09502239E-15	5.5739505E-13	2.1588271E-11
**27**	1.87928505E-22	1.29727043E-18	2.81325518E-15	6.29343094E-13	2.15660966E-11
**28**	4.02456672E-22	2.12997491E-18	3.70356452E-15	7.05139403E-13	2.15495732E-11
**29**	8.1974609E-22	3.38634635E-18	4.78964467E-15	7.84630836E-13	2.15379888E-11
**30**	1.59599488E-21	5.2297963E-18	6.09570031E-15	8.67666548E-13	2.15307198E-11
**31**	2.98301425E-21	7.86755688E-18	7.64621239E-15	9.54099275E-13	2.15272187E-11
**32**	5.37274002E-21	1.15571689E-17	9.46572178E-15	1.04378625E-12	2.15270043E-11
**33**	9.35618475E-21	1.66129419E-17	1.1578633E-14	1.13658986E-12	2.15296529E-11
**34**	1.5799384E-20	2.341226E-17	1.4009039E-14	1.23237803E-12	2.15347906E-11
**35**	2.5938824E-20	3.24016172E-17	1.67805683E-14	1.33102448E-12	2.15420873E-11
**36**	4.14986688E-20	4.41022841E-17	1.99162525E-14	1.43240882E-12	2.15512511E-11
**37**	6.48318436E-20	5.91155174E-17	2.34384144E-14	1.53641653E-12	2.15620235E-11
**38**	9.90866853E-20	7.81272482E-17	2.73685755E-14	1.64293891E-12	2.15741752E-11
**39**	1.4840046E-19	1.01912193E-16	3.17273806E-14	1.75187292E-12	2.15875031E-11
**40**	2.18120589E-19	1.31337356E-16	3.65345387E-14	1.86312099E-12	2.16018265E-11
**41**	3.15053927E-19	1.6736489E-16	4.18087783E-14	1.97659081E-12	2.1616985E-11
**42**	4.47743953E-19	2.11054325E-16	4.75678157E-14	2.0921951E-12	2.16328361E-11
**43**	6.26775195E-19	2.63564146E-16	5.38283342E-14	2.2098514E-12	2.1649253E-11
**44**	8.65103796E-19	3.26152744E-16	6.06059741E-14	2.32948175E-12	2.1666123E-11
**45**	1.1784126E-18	4.00178758E-16	6.79153301E-14	2.45101253E-12	2.16833459E-11
**46**	1.5854888E-18	4.87100833E-16	7.57699571E-14	2.57437414E-12	2.17008326E-11
**47**	2.10862182E-18	5.88476826E-16	8.41823817E-14	2.69950082E-12	2.1718504E-11
**48**	2.77401875E-18	7.05962494E-16	9.31641195E-14	2.82633039E-12	2.17362897E-11
**49**	3.61223446E-18	8.41309704E-16	1.02725696E-13	2.95480404E-12	2.17541275E-11
**50**	4.65861339E-18	9.96364212E-16	1.1287667E-13	3.0848661E-12	2.17719622E-11
**51**	5.95373968E-18	1.17306304E-15	1.23625666E-13	3.2164639E-12	2.17897451E-11

**52**	7.54389251E-18	1.37343148E-15	1.34980397E-13	3.34954751E-12	2.1807433E-11
**53**	9.48150329E-18	1.59957983E-15	1.46947703E-13	3.48406961E-12	2.18249882E-11
**54**	1.18256114E-17	1.85369983E-15	1.59533579E-13	3.6199853E-12	2.18423774E-11
**55**	1.46423154E-17	2.13806083E-15	1.7274321E-13	3.75725195E-12	2.18595716E-11
**56**	1.80052166E-17	2.45500581E-15	1.86581006E-13	3.89582909E-12	2.18765456E-11
**57**	2.19958517E-17	2.80694725E-15	2.01050635E-13	4.03567821E-12	2.18932774E-11
**58**	2.67041124E-17	3.19636278E-15	2.16155058E-13	4.17676267E-12	2.19097484E-11
**59**	3.22286492E-17	3.62579087E-15	2.31896562E-13	4.31904758E-12	2.19259425E-11
**60**	3.86772559E-17	4.09782631E-15	2.48276794E-13	4.46249969E-12	2.19418463E-11
**61**	4.6167235E-17	4.61511578E-15	2.65296794E-13	4.6070873E-12	2.19574486E-11
**62**	5.48257392E-17	5.18035328E-15	2.82957026E-13	4.75278011E-12	2.19727401E-11
**63**	6.47900904E-17	5.79627566E-15	3.01257409E-13	4.8995492E-12	2.19877136E-11
**64**	7.62080713E-17	6.46565816E-15	3.20197351E-13	5.04736692E-12	2.20023635E-11
**65**	8.9238192E-17	7.19130994E-15	3.39775772E-13	5.19620679E-12	2.20166857E-11
**66**	1.04049928E-16	7.97606972E-15	3.59991138E-13	5.34604347E-12	2.20306774E-11
**67**	1.20823929E-16	8.82280151E-15	3.80841487E-13	5.49685267E-12	2.2044337E-11
**68**	1.39752199E-16	9.73439035E-15	4.02324454E-13	5.64861108E-12	2.20576643E-11
**69**	1.61038251E-16	1.07137383E-14	4.24437296E-13	5.80129634E-12	2.20706599E-11
**70**	1.84897221E-16	1.17637602E-14	4.47176919E-13	5.95488698E-12	2.20833252E-11
**71**	2.11555968E-16	1.28873802E-14	4.70539901E-13	6.10936234E-12	2.20956627E-11
**72**	2.4125313E-16	1.40875276E-14	4.9452251E-13	6.26470256E-12	2.21076755E-11
**73**	2.7423916E-16	1.53671333E-14	5.19120732E-13	6.42088852E-12	2.21193673E-11
**74**	3.10776329E-16	1.67291266E-14	5.44330285E-13	6.5779018E-12	2.21307425E-11
**75**	3.51138703E-16	1.81764314E-14	5.70146643E-13	6.73572467E-12	2.21418059E-11
**76**	3.95612094E-16	1.97119633E-14	5.96565053E-13	6.89433999E-12	2.2152563E-11
**77**	4.44493975E-16	2.13386263E-14	6.23580551E-13	7.05373125E-12	2.21630195E-11
**78**	4.98093386E-16	2.30593099E-14	6.51187983E-13	7.21388249E-12	2.21731817E-11
**79**	5.56730798E-16	2.48768865E-14	6.79382015E-13	7.3747783E-12	2.2183056E-11
**80**	6.20737964E-16	2.67942084E-14	7.08157153E-13	7.53640379E-12	2.21926492E-11
**81**	6.9045774E-16	2.88141054E-14	7.37507755E-13	7.69874452E-12	2.22019684E-11
**82**	7.66243894E-16	3.09393826E-14	7.67428046E-13	7.86178656E-12	2.22110209E-11
**83**	8.48460881E-16	3.31728177E-14	7.9791213E-13	8.02551639E-12	2.22198142E-11
**84**	9.37483611E-16	3.55171595E-14	8.28954001E-13	8.18992091E-12	2.22283559E-11
**85**	1.03369719E-15	3.79751251E-14	8.60547558E-13	8.35498744E-12	2.22366538E-11
**86**	1.13749665E-15	4.05493988E-14	8.92686613E-13	8.52070366E-12	2.22447159E-11
**87**	1.24928664E-15	4.32426297E-14	9.253649E-13	8.68705763E-12	2.225255E-11
**88**	1.36948117E-15	4.60574306E-14	9.58576089E-13	8.85403775E-12	2.22601644E-11
**89**	1.49850324E-15	4.8996376E-14	9.92313794E-13	9.02163274E-12	2.2267567E-11
**90**	1.63678452E-15	5.20620011E-14	1.02657158E-12	9.18983167E-12	2.22747662E-11
**91**	1.78476504E-15	5.52568001E-14	1.06134296E-12	9.35862387E-12	2.22817701E-11
**92**	1.9428928E-15	5.85832255E-14	1.09662144E-12	9.527999E-12	2.22885869E-11
**93**	2.11162341E-15	6.20436864E-14	1.13240048E-12	9.69794695E-12	2.22952248E-11
**94**	2.29141974E-15	6.56405478E-14	1.16867353E-12	9.86845792E-12	2.23016921E-11
**95**	2.48275151E-15	6.93761299E-14	1.20543402E-12	1.00395223E-11	2.23079969E-11
**96**	2.68609495E-15	7.32527067E-14	1.24267538E-12	1.02111309E-11	2.23141473E-11
**97**	2.90193235E-15	7.72725059E-14	1.28039104E-12	1.03832744E-11	2.23201515E-11
**98**	3.1307517E-15	8.14377076E-14	1.31857443E-12	1.05559441E-11	2.23260174E-11
**99**	3.37304627E-15	8.57504441E-14	1.35721899E-12	1.07291313E-11	2.23317532E-11
**100**	3.62931422E-15	9.02127993E-14	1.39631819E-12	1.09028276E-11	2.23373666E-11
**101**	3.90005819E-15	9.48268083E-14	1.43586548E-12	1.10770246E-11	2.23428656E-11
**102**	4.18578489E-15	9.95944567E-14	1.47585438E-12	1.12517142E-11	2.23482579E-11

**103**	4.48700468E-15	1.04517681E-13	1.51627839E-12	1.14268886E-11	2.23535513E-11
**104**	4.80423117E-15	1.09598366E-13	1.55713108E-12	1.16025401E-11	2.23587532E-11
**105**	5.1379808E-15	1.14838349E-13	1.59840603E-12	1.1778661E-11	2.23638713E-11
**106**	5.48877246E-15	1.20239416E-13	1.64009684E-12	1.19552439E-11	2.23689129E-11
**107**	5.85712704E-15	1.25803301E-13	1.68219719E-12	1.21322817E-11	2.23738853E-11
**108**	6.24356705E-15	1.31531689E-13	1.72470076E-12	1.2309767E-11	2.23787958E-11
**109**	6.64861622E-15	1.37426216E-13	1.7676013E-12	1.24876929E-11	2.23836514E-11
**110**	7.0727991E-15	1.43488463E-13	1.81089259E-12	1.26660526E-11	2.23884592E-11
**111**	7.51664064E-15	1.49719964E-13	1.85456846E-12	1.28448393E-11	2.23932261E-11
**112**	7.98066584E-15	1.56122202E-13	1.89862279E-12	1.30240464E-11	2.23979587E-11
**113**	8.46539931E-15	1.62696609E-13	1.9430495E-12	1.32036673E-11	2.24026638E-11
**114**	8.97136494E-15	1.69444567E-13	1.98784258E-12	1.33836956E-11	2.2407348E-11
**115**	9.49908549E-15	1.76367407E-13	2.03299606E-12	1.35641251E-11	2.24120175E-11
**116**	1.00490822E-14	1.83466412E-13	2.07850402E-12	1.37449494E-11	2.24166788E-11
**117**	1.06218746E-14	1.90742814E-13	2.12436061E-12	1.39261625E-11	2.2421338E-11
**118**	1.12179798E-14	1.98197795E-13	2.17056001E-12	1.41077582E-11	2.24260012E-11
**119**	1.18379124E-14	2.05832488E-13	2.21709648E-12	1.42897307E-11	2.24306744E-11
**120**	1.24821842E-14	2.13647979E-13	2.26396432E-12	1.4472074E-11	2.24353632E-11
**121**	1.31513036E-14	2.21645303E-13	2.3111579E-12	1.46547823E-11	2.24400735E-11
**122**	1.38457755E-14	2.29825447E-13	2.35867165E-12	1.48378499E-11	2.24448109E-11
**123**	1.45661009E-14	2.38189351E-13	2.40650003E-12	1.50212711E-11	2.24495807E-11
**124**	1.53127766E-14	2.46737905E-13	2.45463758E-12	1.52050402E-11	2.24543884E-11
**125**	1.60862947E-14	2.55471955E-13	2.50307891E-12	1.53891516E-11	2.2459239E-11
**126**	1.68871427E-14	2.64392296E-13	2.55181866E-12	1.55736E-11	2.24641378E-11
**127**	1.77158027E-14	2.7349968E-13	2.60085154E-12	1.57583796E-11	2.24690897E-11
**128**	1.85727518E-14	2.8279481E-13	2.65017233E-12	1.59434853E-11	2.24740994E-11
**129**	1.9458461E-14	2.92278344E-13	2.69977585E-12	1.61289115E-11	2.24791718E-11
**130**	2.03733955E-14	3.01950895E-13	2.74965697E-12	1.63146529E-11	2.24843115E-11
**131**	2.13180145E-14	3.11813029E-13	2.79981066E-12	1.65007043E-11	2.24895228E-11
**132**	2.22927704E-14	3.21865269E-13	2.8502319E-12	1.66870603E-11	2.24948102E-11
**133**	2.32981091E-14	3.32108094E-13	2.90091574E-12	1.68737157E-11	2.2500178E-11
**134**	2.43344696E-14	3.42541937E-13	2.95185731E-12	1.70606653E-11	2.25056302E-11
**135**	2.54022836E-14	3.53167188E-13	3.00305176E-12	1.72479039E-11	2.25111709E-11
**136**	2.65019755E-14	3.63984196E-13	3.05449432E-12	1.74354264E-11	2.25168038E-11
**137**	2.76339623E-14	3.74993266E-13	3.10618027E-12	1.76232275E-11	2.25225329E-11
**138**	2.8798653E-14	3.86194659E-13	3.15810493E-12	1.78113022E-11	2.25283618E-11
**139**	2.99964487E-14	3.97588597E-13	3.2102637E-12	1.79996453E-11	2.2534294E-11
**140**	3.12277423E-14	4.09175259E-13	3.26265201E-12	1.81882519E-11	2.25403329E-11
**141**	3.24929187E-14	4.20954783E-13	3.31526535E-12	1.83771166E-11	2.25464819E-11
**142**	3.3792354E-14	4.32927269E-13	3.36809926E-12	1.85662346E-11	2.25527441E-11
**143**	3.51264157E-14	4.45092774E-13	3.42114934E-12	1.87556007E-11	2.25591227E-11
**144**	3.64954629E-14	4.57451317E-13	3.47441124E-12	1.894521E-11	2.25656207E-11
**145**	3.78998453E-14	4.70002878E-13	3.52788064E-12	1.91350572E-11	2.25722408E-11
**146**	3.93399038E-14	4.82747397E-13	3.58155329E-12	1.93251374E-11	2.2578986E-11
**147**	4.08159704E-14	4.95684778E-13	3.63542499E-12	1.95154455E-11	2.25858589E-11
**148**	4.23283674E-14	5.08814886E-13	3.68949156E-12	1.97059765E-11	2.2592862E-11
**149**	4.38774081E-14	5.22137549E-13	3.74374891E-12	1.98967254E-11	2.25999978E-11
**150**	4.54633961E-14	5.35652558E-13	3.79819296E-12	2.0087687E-11	2.26072686E-11
**151**	4.70866257E-14	5.49359669E-13	3.85281969E-12	2.02788564E-11	2.26146768E-11
**152**	4.87473813E-14	5.63258601E-13	3.90762513E-12	2.04702285E-11	2.26222245E-11
**153**	5.04459379E-14	5.77349038E-13	3.96260534E-12	2.06617981E-11	2.26299137E-11

**154**	5.21825606E-14	5.91630631E-13	4.01775643E-12	2.08535604E-11	2.26377464E-11
**155**	5.39575046E-14	6.06102993E-13	4.07307457E-12	2.10455101E-11	2.26457245E-11
**156**	5.57710156E-14	6.20765706E-13	4.12855595E-12	2.12376422E-11	2.26538497E-11
**157**	5.7623329E-14	6.35618318E-13	4.1841968E-12	2.14299516E-11	2.26621238E-11
**158**	5.95146705E-14	6.50660345E-13	4.2399934E-12	2.16224332E-11	2.26705483E-11
**159**	6.14452559E-14	6.65891269E-13	4.29594208E-12	2.1815082E-11	2.26791247E-11
**160**	6.34152909E-14	6.81310542E-13	4.35203918E-12	2.20078927E-11	2.26878544E-11
**161**	6.54249711E-14	6.96917582E-13	4.40828112E-12	2.22008603E-11	2.26967389E-11
**162**	6.74744825E-14	7.1271178E-13	4.46466432E-12	2.23939796E-11	2.27057792E-11
**163**	6.95640008E-14	7.28692492E-13	4.52118526E-12	2.25872455E-11	2.27149766E-11
**164**	7.16936917E-14	7.44859049E-13	4.57784044E-12	2.27806527E-11	2.27243321E-11
**165**	7.38637111E-14	7.61210748E-13	4.63462642E-12	2.29741963E-11	2.27338468E-11
**166**	7.60742049E-14	7.77746862E-13	4.69153976E-12	2.31678709E-11	2.27435215E-11
**167**	7.8325309E-14	7.9446663E-13	4.74857709E-12	2.33616713E-11	2.27533571E-11
**168**	8.06171494E-14	8.11369269E-13	4.80573506E-12	2.35555924E-11	2.27633542E-11
**169**	8.29498424E-14	8.28453964E-13	4.86301035E-12	2.3749629E-11	2.27735137E-11
**170**	8.53234943E-14	8.45719876E-13	4.92039967E-12	2.39437757E-11	2.27838361E-11
**171**	8.77382016E-14	8.63166138E-13	4.97789978E-12	2.41380274E-11	2.27943219E-11
**172**	9.01940513E-14	8.80791857E-13	5.03550746E-12	2.43323788E-11	2.28049716E-11
**173**	9.26911205E-14	8.98596117E-13	5.09321952E-12	2.45268247E-11	2.28157855E-11
**174**	9.52294767E-14	9.16577975E-13	5.15103279E-12	2.47213597E-11	2.2826764E-11
**175**	9.7809178E-14	9.34736464E-13	5.20894417E-12	2.49159786E-11	2.28379073E-11
**176**	1.00430273E-13	9.53070593E-13	5.26695054E-12	2.5110676E-11	2.28492157E-11
**177**	1.030928E-13	9.71579349E-13	5.32504885E-12	2.53054467E-11	2.28606891E-11
**178**	1.05796791E-13	9.90261694E-13	5.38323605E-12	2.55002853E-11	2.28723277E-11
**179**	1.08542264E-13	1.00911657E-12	5.44150913E-12	2.56951866E-11	2.28841315E-11
**180**	1.11329231E-13	1.0281429E-12	5.49986512E-12	2.58901451E-11	2.28961004E-11
**181**	1.14157695E-13	1.04733957E-12	5.55830105E-12	2.60851555E-11	2.29082341E-11
**182**	1.1702765E-13	1.06670546E-12	5.61681401E-12	2.62802125E-11	2.29205327E-11
**183**	1.19939078E-13	1.08623944E-12	5.67540108E-12	2.64753107E-11	2.29329957E-11
**184**	1.22891957E-13	1.10594033E-12	5.7340594E-12	2.66704447E-11	2.29456229E-11
**185**	1.25886252E-13	1.12580696E-12	5.79278612E-12	2.68656091E-11	2.29584139E-11
**186**	1.28921923E-13	1.14583811E-12	5.85157842E-12	2.70607986E-11	2.29713684E-11
**187**	1.31998918E-13	1.16603258E-12	5.91043349E-12	2.72560078E-11	2.29844857E-11
**188**	1.3511718E-13	1.18638913E-12	5.96934856E-12	2.74512312E-11	2.29977655E-11
**189**	1.38276641E-13	1.20690649E-12	6.02832089E-12	2.76464635E-11	2.30112071E-11
**190**	1.41477227E-13	1.2275834E-12	6.08734776E-12	2.78416993E-11	2.302481E-11
**191**	1.44718854E-13	1.24841856E-12	6.14642645E-12	2.80369331E-11	2.30385734E-11
**192**	1.48001431E-13	1.26941068E-12	6.20555429E-12	2.82321595E-11	2.30524966E-11
**193**	1.5132486E-13	1.29055842E-12	6.26472864E-12	2.84273732E-11	2.30665789E-11
**194**	1.54689034E-13	1.31186047E-12	6.32394685E-12	2.86225686E-11	2.30808194E-11
**195**	1.58093839E-13	1.33331546E-12	6.38320633E-12	2.88177405E-11	2.30952174E-11
**196**	1.61539154E-13	1.35492203E-12	6.44250448E-12	2.90128834E-11	2.31097719E-11
**197**	1.65024848E-13	1.3766788E-12	6.50183873E-12	2.92079918E-11	2.3124482E-11
**198**	1.68550788E-13	1.39858439E-12	6.56120656E-12	2.94030604E-11	2.31393468E-11
**199**	1.72116828E-13	1.42063739E-12	6.62060543E-12	2.95980837E-11	2.31543651E-11
**200**	1.7572282E-13	1.44283639E-12	6.68003286E-12	2.97930563E-11	2.31695361E-11
**201**	1.79368605E-13	1.46517994E-12	6.73948635E-12	2.99879729E-11	2.31848585E-11
**202**	1.83054021E-13	1.48766663E-12	6.79896345E-12	3.01828281E-11	2.32003313E-11
**203**	1.86778898E-13	1.51029499E-12	6.85846173E-12	3.03776164E-11	2.32159533E-11
**204**	1.90543058E-13	1.53306357E-12	6.91797878E-12	3.05723325E-11	2.32317234E-11

**205**	1.94346318E-13	1.5559709E-12	6.97751219E-12	3.07669709E-11	2.32476403E-11
**206**	1.98188491E-13	1.57901548E-12	7.03705958E-12	3.09615264E-11	2.32637028E-11
**207**	2.02069379E-13	1.60219585E-12	7.09661862E-12	3.11559935E-11	2.32799096E-11
**208**	2.05988783E-13	1.6255105E-12	7.15618695E-12	3.1350367E-11	2.32962594E-11
**209**	2.09946496E-13	1.64895791E-12	7.21576226E-12	3.15446414E-11	2.33127509E-11
**210**	2.13942304E-13	1.67253658E-12	7.27534227E-12	3.17388114E-11	2.33293826E-11
**211**	2.1797599E-13	1.69624499E-12	7.33492468E-12	3.19328718E-11	2.33461533E-11
**212**	2.2204733E-13	1.72008161E-12	7.39450724E-12	3.21268172E-11	2.33630615E-11
**213**	2.26156096E-13	1.74404489E-12	7.45408772E-12	3.23206423E-11	2.33801058E-11
**214**	2.30302053E-13	1.7681333E-12	7.51366389E-12	3.25143419E-11	2.33972847E-11
**215**	2.34484964E-13	1.7923453E-12	7.57323356E-12	3.27079106E-11	2.34145967E-11
**216**	2.38704584E-13	1.81667932E-12	7.63279454E-12	3.29013433E-11	2.34320403E-11
**217**	2.42960664E-13	1.8411338E-12	7.69234466E-12	3.30946346E-11	2.34496141E-11
**218**	2.47252952E-13	1.8657072E-12	7.75188179E-12	3.32877795E-11	2.34673164E-11
**219**	2.51581191E-13	1.89039792E-12	7.81140379E-12	3.34807727E-11	2.34851458E-11
**220**	2.55945117E-13	1.91520442E-12	7.87090856E-12	3.3673609E-11	2.35031005E-11
**221**	2.60344465E-13	1.9401251E-12	7.93039401E-12	3.38662832E-11	2.35211791E-11
**222**	2.64778966E-13	1.96515839E-12	7.98985806E-12	3.40587903E-11	2.35393799E-11
**223**	2.69248343E-13	1.99030272E-12	8.04929867E-12	3.42511251E-11	2.35577013E-11
**224**	2.7375232E-13	2.01555648E-12	8.10871379E-12	3.44432825E-11	2.35761417E-11
**225**	2.78290615E-13	2.0409181E-12	8.16810141E-12	3.46352574E-11	2.35946993E-11
**226**	2.82862941E-13	2.066386E-12	8.22745952E-12	3.48270449E-11	2.36133725E-11
**227**	2.8746901E-13	2.09195856E-12	8.28678615E-12	3.50186398E-11	2.36321597E-11
**228**	2.92108531E-13	2.11763422E-12	8.34607933E-12	3.52100371E-11	2.36510592E-11
**229**	2.96781206E-13	2.14341137E-12	8.40533711E-12	3.5401232E-11	2.36700692E-11
**230**	3.01486737E-13	2.16928842E-12	8.46455756E-12	3.55922193E-11	2.3689188E-11
**231**	3.06224824E-13	2.19526378E-12	8.52373876E-12	3.57829943E-11	2.37084139E-11
**232**	3.1099516E-13	2.22133586E-12	8.58287884E-12	3.5973552E-11	2.37277452E-11
**233**	3.15797438E-13	2.24750305E-12	8.64197589E-12	3.61638875E-11	2.37471801E-11
**234**	3.20631349E-13	2.27376378E-12	8.70102808E-12	3.6353996E-11	2.37667168E-11
**235**	3.2549658E-13	2.30011644E-12	8.76003354E-12	3.65438727E-11	2.37863537E-11
**236**	3.30392814E-13	2.32655945E-12	8.81899046E-12	3.67335128E-11	2.3806089E-11
**237**	3.35319736E-13	2.35309121E-12	8.87789703E-12	3.69229115E-11	2.38259208E-11
**238**	3.40277025E-13	2.37971015E-12	8.93675145E-12	3.7112064E-11	2.38458474E-11
**239**	3.45264359E-13	2.40641467E-12	8.99555194E-12	3.73009658E-11	2.38658671E-11
**240**	3.50281414E-13	2.43320319E-12	9.05429676E-12	3.74896122E-11	2.3885978E-11
**241**	3.55327865E-13	2.46007414E-12	9.11298416E-12	3.76779984E-11	2.39061784E-11
**242**	3.60403383E-13	2.48702592E-12	9.17161241E-12	3.78661199E-11	2.39264664E-11
**243**	3.65507639E-13	2.51405698E-12	9.23017981E-12	3.80539721E-11	2.39468404E-11
**244**	3.70640303E-13	2.54116573E-12	9.28868466E-12	3.82415506E-11	2.39672984E-11
**245**	3.75801042E-13	2.56835062E-12	9.3471253E-12	3.84288507E-11	2.39878387E-11
**246**	3.80989521E-13	2.59561006E-12	9.40550005E-12	3.8615868E-11	2.40084595E-11
**247**	3.86205405E-13	2.62294252E-12	9.4638073E-12	3.88025982E-11	2.4029159E-11
**248**	3.91448358E-13	2.65034642E-12	9.5220454E-12	3.89890366E-11	2.40499354E-11
**249**	3.96718042E-13	2.67782021E-12	9.58021275E-12	3.91751792E-11	2.40707869E-11
**250**	4.02014119E-13	2.70536236E-12	9.63830776E-12	3.93610213E-11	2.40917118E-11
**251**	4.07336247E-13	2.73297131E-12	9.69632885E-12	3.95465589E-11	2.41127081E-11
**252**	4.12684088E-13	2.76064554E-12	9.75427447E-12	3.97317876E-11	2.41337742E-11
**253**	4.18057298E-13	2.7883835E-12	9.81214307E-12	3.99167032E-11	2.41549082E-11
**254**	4.23455537E-13	2.81618367E-12	9.86993313E-12	4.01013015E-11	2.41761083E-11
**255**	4.28878461E-13	2.84404454E-12	9.92764314E-12	4.02855784E-11	2.41973729E-11

**256**	4.34325727E-13	2.87196458E-12	9.9852716E-12	4.04695298E-11	2.42187E-11
**257**	4.39796991E-13	2.89994228E-12	1.0042817E-11	4.06531515E-11	2.42400879E-11
**258**	4.45291909E-13	2.92797615E-12	1.0100278E-11	4.08364396E-11	2.42615349E-11
**259**	4.50810136E-13	2.95606469E-12	1.0157653E-11	4.101939E-11	2.42830391E-11
**260**	4.56351328E-13	2.9842064E-12	1.02149407E-11	4.12019988E-11	2.43045988E-11
**261**	4.61915139E-13	3.0123998E-12	1.02721396E-11	4.13842621E-11	2.43262123E-11
**262**	4.67501226E-13	3.04064342E-12	1.03292483E-11	4.1566176E-11	2.43478778E-11
**263**	4.73109241E-13	3.06893579E-12	1.03862655E-11	4.17477366E-11	2.43695936E-11
**264**	4.78738841E-13	3.09727544E-12	1.04431898E-11	4.19289402E-11	2.43913578E-11
**265**	4.84389681E-13	3.12566091E-12	1.05000198E-11	4.2109783E-11	2.44131689E-11
**266**	4.90061416E-13	3.15409077E-12	1.05567542E-11	4.22902612E-11	2.4435025E-11
**267**	4.95753702E-13	3.18256357E-12	1.06133917E-11	4.24703712E-11	2.44569244E-11
**268**	5.01466194E-13	3.21107787E-12	1.06699309E-11	4.26501094E-11	2.44788655E-11
**269**	5.07198549E-13	3.23963225E-12	1.07263707E-11	4.2829472E-11	2.45008464E-11
**270**	5.12950423E-13	3.2682253E-12	1.07827097E-11	4.30084557E-11	2.45228656E-11
**271**	5.18721473E-13	3.2968556E-12	1.08389466E-11	4.31870567E-11	2.45449214E-11
**272**	5.24511359E-13	3.32552175E-12	1.08950803E-11	4.33652718E-11	2.45670119E-11
**273**	5.30319736E-13	3.35422237E-12	1.09511095E-11	4.35430974E-11	2.45891357E-11
**274**	5.36146266E-13	3.38295606E-12	1.1007033E-11	4.37205301E-11	2.4611291E-11
**275**	5.41990607E-13	3.41172145E-12	1.10628496E-11	4.38975665E-11	2.46334761E-11
**276**	5.4785242E-13	3.44051718E-12	1.11185581E-11	4.40742034E-11	2.46556895E-11
**277**	5.53731366E-13	3.46934187E-12	1.11741574E-11	4.42504375E-11	2.46779295E-11
**278**	5.59627108E-13	3.49819419E-12	1.12296463E-11	4.44262655E-11	2.47001944E-11
**279**	5.6553931E-13	3.5270728E-12	1.12850236E-11	4.46016842E-11	2.47224827E-11
**280**	5.71467634E-13	3.55597635E-12	1.13402883E-11	4.47766906E-11	2.47447927E-11
**281**	5.77411746E-13	3.58490353E-12	1.13954393E-11	4.49512814E-11	2.47671228E-11
**282**	5.83371313E-13	3.61385302E-12	1.14504754E-11	4.51254536E-11	2.47894716E-11
**283**	5.89346002E-13	3.64282352E-12	1.15053957E-11	4.52992042E-11	2.48118373E-11
**284**	5.95335482E-13	3.67181373E-12	1.15601989E-11	4.54725301E-11	2.48342184E-11
**285**	6.01339422E-13	3.70082237E-12	1.16148842E-11	4.56454285E-11	2.48566134E-11
**286**	6.07357493E-13	3.72984815E-12	1.16694504E-11	4.58178964E-11	2.48790207E-11
**287**	6.13389368E-13	3.75888981E-12	1.17238965E-11	4.5989931E-11	2.49014388E-11
**288**	6.19434719E-13	3.78794609E-12	1.17782216E-11	4.61615294E-11	2.49238662E-11
**289**	6.25493224E-13	3.81701574E-12	1.18324246E-11	4.63326889E-11	2.49463014E-11
**290**	6.31564556E-13	3.84609752E-12	1.18865047E-11	4.65034066E-11	2.49687428E-11
**291**	6.37648395E-13	3.87519021E-12	1.19404607E-11	4.667368E-11	2.4991189E-11
**292**	6.4374442E-13	3.90429258E-12	1.19942919E-11	4.68435064E-11	2.50136386E-11
**293**	6.49852311E-13	3.93340341E-12	1.20479972E-11	4.7012883E-11	2.50360899E-11
**294**	6.55971751E-13	3.96252152E-12	1.21015757E-11	4.71818075E-11	2.50585417E-11
**295**	6.62102424E-13	3.99164571E-12	1.21550266E-11	4.73502771E-11	2.50809925E-11
**296**	6.68244015E-13	4.02077479E-12	1.22083489E-11	4.75182894E-11	2.51034407E-11
**297**	6.74396211E-13	4.0499076E-12	1.22615418E-11	4.7685842E-11	2.51258851E-11
**298**	6.805587E-13	4.07904298E-12	1.23146045E-11	4.78529325E-11	2.51483242E-11
**299**	6.86731175E-13	4.10817976E-12	1.2367536E-11	4.80195584E-11	2.51707566E-11
**300**	6.92913326E-13	4.13731682E-12	1.24203355E-11	4.81857174E-11	2.5193181E-11
**301**	6.99104847E-13	4.16645302E-12	1.24730023E-11	4.83514073E-11	2.5215596E-11
**302**	7.05305434E-13	4.19558724E-12	1.25255355E-11	4.85166258E-11	2.52380002E-11
**303**	7.11514784E-13	4.22471836E-12	1.25779343E-11	4.86813706E-11	2.52603923E-11
**304**	7.17732598E-13	4.25384529E-12	1.2630198E-11	4.88456396E-11	2.52827709E-11
**305**	7.23958574E-13	4.28296692E-12	1.26823257E-11	4.90094306E-11	2.53051348E-11
**306**	7.30192418E-13	4.31208219E-12	1.27343168E-11	4.91727416E-11	2.53274826E-11

**307**	7.36433832E-13	4.34119002E-12	1.27861705E-11	4.93355704E-11	2.53498131E-11
**308**	7.42682524E-13	4.37028934E-12	1.2837886E-11	4.94979151E-11	2.5372125E-11
**309**	7.48938203E-13	4.3993791E-12	1.28894627E-11	4.96597738E-11	2.5394417E-11
**310**	7.55200578E-13	4.42845827E-12	1.29408999E-11	4.98211443E-11	2.54166878E-11
**311**	7.61469363E-13	4.4575258E-12	1.29921968E-11	4.99820249E-11	2.54389362E-11
**312**	7.67744271E-13	4.48658068E-12	1.30433529E-11	5.01424136E-11	2.54611611E-11
**313**	7.74025018E-13	4.5156219E-12	1.30943673E-11	5.03023087E-11	2.54833611E-11
**314**	7.80311324E-13	4.54464846E-12	1.31452396E-11	5.04617083E-11	2.55055351E-11
**315**	7.86602908E-13	4.57365935E-12	1.3195969E-11	5.06206108E-11	2.55276819E-11
**316**	7.92899492E-13	4.60265361E-12	1.32465549E-11	5.07790143E-11	2.55498002E-11
**317**	7.99200801E-13	4.63163025E-12	1.32969968E-11	5.09369172E-11	2.55718891E-11
**318**	8.05506561E-13	4.66058832E-12	1.33472939E-11	5.10943179E-11	2.55939472E-11
**319**	8.11816501E-13	4.68952687E-12	1.33974458E-11	5.12512147E-11	2.56159735E-11
**320**	8.1813035E-13	4.71844494E-12	1.34474519E-11	5.14076061E-11	2.56379668E-11
**321**	8.24447842E-13	4.74734162E-12	1.34973115E-11	5.15634906E-11	2.56599261E-11
**322**	8.3076871E-13	4.77621597E-12	1.35470242E-11	5.17188666E-11	2.56818502E-11
**323**	8.37092692E-13	4.80506708E-12	1.35965893E-11	5.18737328E-11	2.5703738E-11
**324**	8.43419526E-13	4.83389406E-12	1.36460065E-11	5.20280876E-11	2.57255884E-11
**325**	8.49748953E-13	4.86269601E-12	1.3695275E-11	5.21819297E-11	2.57474005E-11
**326**	8.56080715E-13	4.89147204E-12	1.37443946E-11	5.23352577E-11	2.57691731E-11
**327**	8.62414559E-13	4.92022129E-12	1.37933646E-11	5.24880704E-11	2.57909052E-11
**328**	8.6875023E-13	4.94894288E-12	1.38421845E-11	5.26403664E-11	2.58125959E-11
**329**	8.75087479E-13	4.97763597E-12	1.3890854E-11	5.27921445E-11	2.5834244E-11
**330**	8.81426056E-13	5.00629971E-12	1.39393725E-11	5.29434035E-11	2.58558486E-11
**331**	8.87765715E-13	5.03493326E-12	1.39877396E-11	5.30941421E-11	2.58774086E-11
**332**	8.94106212E-13	5.0635358E-12	1.40359549E-11	5.32443594E-11	2.58989232E-11
**333**	9.00447303E-13	5.09210652E-12	1.40840179E-11	5.3394054E-11	2.59203914E-11
**334**	9.0678875E-13	5.12064461E-12	1.41319282E-11	5.35432251E-11	2.59418121E-11
**335**	9.13130314E-13	5.14914927E-12	1.41796854E-11	5.36918714E-11	2.59631846E-11
**336**	9.1947176E-13	5.17761972E-12	1.42272892E-11	5.3839992E-11	2.59845077E-11
**337**	9.25812852E-13	5.20605517E-12	1.4274739E-11	5.3987586E-11	2.60057808E-11
**338**	9.32153361E-13	5.23445487E-12	1.43220347E-11	5.41346523E-11	2.60270027E-11
**339**	9.38493056E-13	5.26281805E-12	1.43691757E-11	5.42811901E-11	2.60481727E-11
**340**	9.4483171E-13	5.29114395E-12	1.44161618E-11	5.44271985E-11	2.60692899E-11
**341**	9.51169098E-13	5.31943185E-12	1.44629925E-11	5.45726765E-11	2.60903533E-11
**342**	9.57504997E-13	5.34768101E-12	1.45096677E-11	5.47176235E-11	2.61113622E-11
**343**	9.63839186E-13	5.37589071E-12	1.45561869E-11	5.48620386E-11	2.61323158E-11
**344**	9.70171445E-13	5.40406023E-12	1.46025498E-11	5.50059211E-11	2.61532131E-11
**345**	9.7650156E-13	5.43218887E-12	1.46487562E-11	5.51492701E-11	2.61740534E-11
**346**	9.82829314E-13	5.46027594E-12	1.46948057E-11	5.52920851E-11	2.61948358E-11
**347**	9.89154495E-13	5.48832076E-12	1.4740698E-11	5.54343653E-11	2.62155596E-11
**348**	9.95476894E-13	5.51632263E-12	1.4786433E-11	5.55761102E-11	2.6236224E-11
**349**	1.0017963E-12	5.5442809E-12	1.48320102E-11	5.57173191E-11	2.62568282E-11
**350**	1.00811251E-12	5.57219491E-12	1.48774296E-11	5.58579914E-11	2.62773715E-11
**351**	1.01442532E-12	5.60006401E-12	1.49226907E-11	5.59981265E-11	2.62978531E-11
**352**	1.02073453E-12	5.62788756E-12	1.49677935E-11	5.6137724E-11	2.63182722E-11
**353**	1.02703993E-12	5.65566491E-12	1.50127376E-11	5.62767834E-11	2.63386282E-11
**354**	1.03334133E-12	5.68339546E-12	1.50575228E-11	5.64153042E-11	2.63589204E-11
**355**	1.03963854E-12	5.71107858E-12	1.5102149E-11	5.65532859E-11	2.6379148E-11
**356**	1.04593136E-12	5.73871366E-12	1.51466159E-11	5.66907282E-11	2.63993103E-11
**357**	1.05221959E-12	5.76630012E-12	1.51909234E-11	5.68276306E-11	2.64194066E-11

**358**	1.05850306E-12	5.79383735E-12	1.52350712E-11	5.69639928E-11	2.64394364E-11
**359**	1.06478156E-12	5.82132477E-12	1.52790593E-11	5.70998146E-11	2.64593989E-11
**360**	1.07105493E-12	5.84876182E-12	1.53228873E-11	5.72350955E-11	2.64792935E-11
**361**	1.07732296E-12	5.87614793E-12	1.53665552E-11	5.73698352E-11	2.64991195E-11
**362**	1.08358549E-12	5.90348254E-12	1.54100628E-11	5.75040337E-11	2.65188763E-11
**363**	1.08984234E-12	5.9307651E-12	1.545341E-11	5.76376906E-11	2.65385634E-11
**364**	1.09609332E-12	5.95799507E-12	1.54965967E-11	5.77708056E-11	2.655818E-11
**365**	1.10233826E-12	5.98517192E-12	1.55396227E-11	5.79033788E-11	2.65777257E-11
**366**	1.10857698E-12	6.01229513E-12	1.55824879E-11	5.80354098E-11	2.65971998E-11
**367**	1.11480933E-12	6.03936417E-12	1.56251922E-11	5.81668987E-11	2.66166017E-11
**368**	1.12103512E-12	6.06637854E-12	1.56677355E-11	5.82978452E-11	2.66359309E-11
**369**	1.12725419E-12	6.09333775E-12	1.57101177E-11	5.84282493E-11	2.66551868E-11
**370**	1.13346638E-12	6.12024128E-12	1.57523388E-11	5.85581109E-11	2.66743689E-11
**371**	1.13967152E-12	6.14708867E-12	1.57943986E-11	5.86874301E-11	2.66934766E-11
**372**	1.14586945E-12	6.17387943E-12	1.58362971E-11	5.88162068E-11	2.67125095E-11
**373**	1.15206001E-12	6.20061309E-12	1.58780342E-11	5.89444411E-11	2.67314669E-11
**374**	1.15824304E-12	6.22728919E-12	1.59196099E-11	5.90721329E-11	2.67503485E-11
**375**	1.1644184E-12	6.25390728E-12	1.59610241E-11	5.91992824E-11	2.67691537E-11
**376**	1.17058591E-12	6.2804669E-12	1.60022768E-11	5.93258896E-11	2.6787882E-11
**377**	1.17674544E-12	6.30696762E-12	1.6043368E-11	5.94519547E-11	2.68065329E-11
**378**	1.18289684E-12	6.33340901E-12	1.60842976E-11	5.95774777E-11	2.68251061E-11
**379**	1.18903995E-12	6.35979063E-12	1.61250656E-11	5.97024589E-11	2.68436009E-11
**380**	1.19517463E-12	6.38611208E-12	1.6165672E-11	5.98268983E-11	2.6862017E-11
**381**	1.20130073E-12	6.41237293E-12	1.62061168E-11	5.99507963E-11	2.6880354E-11
**382**	1.20741812E-12	6.43857279E-12	1.62463999E-11	6.00741529E-11	2.68986113E-11
**383**	1.21352665E-12	6.46471126E-12	1.62865215E-11	6.01969685E-11	2.69167886E-11
**384**	1.21962618E-12	6.49078794E-12	1.63264816E-11	6.03192434E-11	2.69348855E-11
**385**	1.22571658E-12	6.51680247E-12	1.636628E-11	6.04409777E-11	2.69529016E-11
**386**	1.23179772E-12	6.54275445E-12	1.6405917E-11	6.05621717E-11	2.69708364E-11
**387**	1.23786945E-12	6.56864352E-12	1.64453924E-11	6.06828259E-11	2.69886896E-11
**388**	1.24393165E-12	6.59446931E-12	1.64847064E-11	6.08029405E-11	2.70064609E-11
**389**	1.24998419E-12	6.62023148E-12	1.6523859E-11	6.09225158E-11	2.70241497E-11
**390**	1.25602693E-12	6.64592967E-12	1.65628503E-11	6.10415524E-11	2.70417559E-11
**391**	1.26205976E-12	6.67156353E-12	1.66016802E-11	6.11600505E-11	2.70592789E-11
**392**	1.26808255E-12	6.69713274E-12	1.6640349E-11	6.12780105E-11	2.70767186E-11
**393**	1.27409518E-12	6.72263697E-12	1.66788565E-11	6.13954329E-11	2.70940745E-11
**394**	1.28009752E-12	6.74807588E-12	1.67172031E-11	6.15123182E-11	2.71113463E-11
**395**	1.28608946E-12	6.77344916E-12	1.67553886E-11	6.16286668E-11	2.71285338E-11
**396**	1.29207088E-12	6.79875651E-12	1.67934132E-11	6.17444791E-11	2.71456365E-11
**397**	1.29804166E-12	6.82399761E-12	1.68312771E-11	6.18597558E-11	2.71626542E-11
**398**	1.30400169E-12	6.84917218E-12	1.68689802E-11	6.19744972E-11	2.71795866E-11
**399**	1.30995086E-12	6.87427991E-12	1.69065227E-11	6.2088704E-11	2.71964334E-11
**400**	1.31588905E-12	6.89932052E-12	1.69439047E-11	6.22023766E-11	2.72131944E-11
**401**	1.32181616E-12	6.92429373E-12	1.69811264E-11	6.23155157E-11	2.72298691E-11
**402**	1.32773208E-12	6.94919927E-12	1.70181878E-11	6.24281219E-11	2.72464575E-11
**403**	1.3336367E-12	6.97403687E-12	1.70550891E-11	6.25401956E-11	2.72629592E-11
**404**	1.33952992E-12	6.99880627E-12	1.70918304E-11	6.26517376E-11	2.7279374E-11
**405**	1.34541164E-12	7.02350721E-12	1.71284119E-11	6.27627485E-11	2.72957017E-11
**406**	1.35128175E-12	7.04813943E-12	1.71648336E-11	6.28732289E-11	2.73119419E-11
**407**	1.35714015E-12	7.0727027E-12	1.72010958E-11	6.29831795E-11	2.73280945E-11
**408**	1.36298675E-12	7.09719678E-12	1.72371985E-11	6.30926009E-11	2.73441593E-11

**409**	1.36882145E-12	7.12162143E-12	1.7273142E-11	6.32014939E-11	2.7360136E-11
**410**	1.37464416E-12	7.14597642E-12	1.73089265E-11	6.33098591E-11	2.73760244E-11
**411**	1.38045477E-12	7.17026153E-12	1.73445519E-11	6.34176973E-11	2.73918244E-11
**412**	1.38625321E-12	7.19447655E-12	1.73800187E-11	6.35250091E-11	2.74075357E-11
**413**	1.39203937E-12	7.21862126E-12	1.74153268E-11	6.36317955E-11	2.74231582E-11
**414**	1.39781318E-12	7.24269545E-12	1.74504766E-11	6.3738057E-11	2.74386916E-11
**415**	1.40357454E-12	7.26669893E-12	1.74854681E-11	6.38437944E-11	2.74541359E-11
**416**	1.40932336E-12	7.29063149E-12	1.75203016E-11	6.39490087E-11	2.74694907E-11
**417**	1.41505956E-12	7.31449295E-12	1.75549773E-11	6.40537005E-11	2.74847561E-11
**418**	1.42078307E-12	7.33828312E-12	1.75894953E-11	6.41578706E-11	2.74999317E-11
**419**	1.42649378E-12	7.36200182E-12	1.76238559E-11	6.426152E-11	2.75150176E-11
**420**	1.43219164E-12	7.38564888E-12	1.76580593E-11	6.43646493E-11	2.75300134E-11
**421**	1.43787655E-12	7.40922412E-12	1.76921056E-11	6.44672596E-11	2.75449192E-11
**422**	1.44354844E-12	7.43272737E-12	1.77259952E-11	6.45693516E-11	2.75597347E-11
**423**	1.44920724E-12	7.45615848E-12	1.77597281E-11	6.46709261E-11	2.75744598E-11
**424**	1.45485286E-12	7.47951729E-12	1.77933047E-11	6.47719842E-11	2.75890945E-11
**425**	1.46048523E-12	7.50280365E-12	1.78267251E-11	6.48725267E-11	2.76036386E-11
**426**	1.46610429E-12	7.52601741E-12	1.78599897E-11	6.49725544E-11	2.7618092E-11
**427**	1.47170996E-12	7.54915843E-12	1.78930985E-11	6.50720684E-11	2.76324546E-11
**428**	1.47730218E-12	7.57222657E-12	1.79260519E-11	6.51710695E-11	2.76467263E-11
**429**	1.48288086E-12	7.5952217E-12	1.79588501E-11	6.52695586E-11	2.7660907E-11
**430**	1.48844596E-12	7.61814369E-12	1.79914933E-11	6.53675368E-11	2.76749966E-11
**431**	1.49399739E-12	7.64099241E-12	1.80239818E-11	6.5465005E-11	2.76889952E-11
**432**	1.4995351E-12	7.66376775E-12	1.80563158E-11	6.55619641E-11	2.77029025E-11
**433**	1.50505903E-12	7.6864696E-12	1.80884956E-11	6.56584152E-11	2.77167185E-11
**434**	1.5105691E-12	7.70909783E-12	1.81205215E-11	6.57543592E-11	2.77304432E-11
**435**	1.51606527E-12	7.73165234E-12	1.81523937E-11	6.58497971E-11	2.77440764E-11
**436**	1.52154747E-12	7.75413304E-12	1.81841124E-11	6.59447299E-11	2.77576183E-11
**437**	1.52701564E-12	7.77653982E-12	1.8215678E-11	6.60391587E-11	2.77710686E-11
**438**	1.53246973E-12	7.79887259E-12	1.82470906E-11	6.61330845E-11	2.77844274E-11
**439**	1.53790968E-12	7.82113125E-12	1.82783507E-11	6.62265082E-11	2.77976946E-11
**440**	1.54333543E-12	7.84331573E-12	1.83094583E-11	6.6319431E-11	2.78108702E-11
**441**	1.54874694E-12	7.86542594E-12	1.8340414E-11	6.6411854E-11	2.78239542E-11
**442**	1.55414415E-12	7.8874618E-12	1.83712178E-11	6.65037781E-11	2.78369465E-11
**443**	1.559527E-12	7.90942324E-12	1.84018701E-11	6.65952044E-11	2.78498471E-11
**444**	1.56489546E-12	7.93131018E-12	1.84323712E-11	6.66861341E-11	2.78626561E-11
**445**	1.57024946E-12	7.95312257E-12	1.84627214E-11	6.67765681E-11	2.78753734E-11
**446**	1.57558897E-12	7.97486033E-12	1.84929209E-11	6.68665077E-11	2.78879989E-11
**447**	1.58091393E-12	7.99652341E-12	1.85229701E-11	6.69559538E-11	2.79005328E-11
**448**	1.5862243E-12	8.01811176E-12	1.85528693E-11	6.70449077E-11	2.7912975E-11
**449**	1.59152004E-12	8.03962531E-12	1.85826187E-11	6.71333704E-11	2.79253255E-11
**450**	1.5968011E-12	8.06106403E-12	1.86122186E-11	6.7221343E-11	2.79375844E-11
**451**	1.60206744E-12	8.08242787E-12	1.86416695E-11	6.73088267E-11	2.79497515E-11
**452**	1.60731901E-12	8.10371679E-12	1.86709715E-11	6.73958226E-11	2.79618271E-11
**453**	1.61255579E-12	8.12493075E-12	1.87001249E-11	6.74823319E-11	2.79738111E-11
**454**	1.61777772E-12	8.14606971E-12	1.87291302E-11	6.75683557E-11	2.79857035E-11
**455**	1.62298477E-12	8.16713365E-12	1.87579876E-11	6.76538952E-11	2.79975043E-11
**456**	1.62817691E-12	8.18812253E-12	1.87866974E-11	6.77389515E-11	2.80092137E-11
**457**	1.63335409E-12	8.20903634E-12	1.88152599E-11	6.78235258E-11	2.80208316E-11
**458**	1.63851628E-12	8.22987506E-12	1.88436755E-11	6.79076193E-11	2.80323582E-11
**459**	1.64366345E-12	8.25063865E-12	1.88719445E-11	6.79912331E-11	2.80437933E-11

**460**	1.64879556E-12	8.27132712E-12	1.89000673E-11	6.80743685E-11	2.80551372E-11
**461**	1.65391259E-12	8.29194044E-12	1.8928044E-11	6.81570266E-11	2.80663898E-11
**462**	1.65901449E-12	8.31247862E-12	1.89558751E-11	6.82392087E-11	2.80775512E-11
**463**	1.66410124E-12	8.33294164E-12	1.8983561E-11	6.83209159E-11	2.80886216E-11
**464**	1.66917282E-12	8.35332951E-12	1.90111018E-11	6.84021495E-11	2.80996008E-11
**465**	1.67422918E-12	8.37364222E-12	1.9038498E-11	6.84829106E-11	2.81104891E-11
**466**	1.67927031E-12	8.39387977E-12	1.906575E-11	6.85632005E-11	2.81212865E-11
**467**	1.68429617E-12	8.41404219E-12	1.9092858E-11	6.86430205E-11	2.81319931E-11
**468**	1.68930675E-12	8.43412946E-12	1.91198223E-11	6.87223717E-11	2.8142609E-11
**469**	1.69430201E-12	8.45414161E-12	1.91466434E-11	6.88012554E-11	2.81531341E-11
**470**	1.69928193E-12	8.47407866E-12	1.91733216E-11	6.88796727E-11	2.81635688E-11
**471**	1.70424649E-12	8.49394061E-12	1.91998571E-11	6.89576251E-11	2.81739129E-11
**472**	1.70919566E-12	8.5137275E-12	1.92262505E-11	6.90351137E-11	2.81841667E-11
**473**	1.71412943E-12	8.53343934E-12	1.92525019E-11	6.91121397E-11	2.81943302E-11
**474**	1.71904777E-12	8.55307616E-12	1.92786118E-11	6.91887044E-11	2.82044035E-11
**475**	1.72395067E-12	8.57263799E-12	1.93045805E-11	6.92648091E-11	2.82143868E-11
**476**	1.7288381E-12	8.59212486E-12	1.93304084E-11	6.93404551E-11	2.82242801E-11
**477**	1.73371004E-12	8.6115368E-12	1.93560958E-11	6.94156436E-11	2.82340835E-11
**478**	1.73856648E-12	8.63087385E-12	1.93816431E-11	6.94903758E-11	2.82437972E-11
**479**	1.74340741E-12	8.65013606E-12	1.94070506E-11	6.95646531E-11	2.82534213E-11
**480**	1.74823279E-12	8.66932345E-12	1.94323187E-11	6.96384767E-11	2.82629559E-11
**481**	1.75304263E-12	8.68843608E-12	1.94574478E-11	6.97118479E-11	2.82724011E-11
**482**	1.7578369E-12	8.70747399E-12	1.94824382E-11	6.97847681E-11	2.8281757E-11
**483**	1.7626156E-12	8.72643723E-12	1.95072902E-11	6.98572384E-11	2.82910238E-11
**484**	1.7673787E-12	8.74532585E-12	1.95320043E-11	6.99292602E-11	2.83002016E-11
**485**	1.7721262E-12	8.76413991E-12	1.95565807E-11	7.00008348E-11	2.83092906E-11
**486**	1.77685808E-12	8.78287945E-12	1.958102E-11	7.00719634E-11	2.83182908E-11
**487**	1.78157434E-12	8.80154454E-12	1.96053224E-11	7.01426474E-11	2.83272024E-11
**488**	1.78627496E-12	8.82013523E-12	1.96294882E-11	7.0212888E-11	2.83360255E-11
**489**	1.79095994E-12	8.8386516E-12	1.9653518E-11	7.02826867E-11	2.83447603E-11
**490**	1.79562926E-12	8.85709369E-12	1.9677412E-11	7.03520446E-11	2.8353407E-11
**491**	1.80028292E-12	8.87546159E-12	1.97011706E-11	7.0420963E-11	2.83619656E-11
**492**	1.80492091E-12	8.89375535E-12	1.97247942E-11	7.04894434E-11	2.83704364E-11
**493**	1.80954322E-12	8.91197506E-12	1.97482831E-11	7.0557487E-11	2.83788195E-11
**494**	1.81414985E-12	8.93012077E-12	1.97716378E-11	7.06250951E-11	2.83871149E-11
**495**	1.81874079E-12	8.94819256E-12	1.97948585E-11	7.06922691E-11	2.8395323E-11
**496**	1.82331604E-12	8.96619052E-12	1.98179457E-11	7.07590102E-11	2.84034438E-11
**497**	1.8278756E-12	8.98411472E-12	1.98408998E-11	7.08253198E-11	2.84114775E-11
**498**	1.83241945E-12	9.00196524E-12	1.98637212E-11	7.08911992E-11	2.84194243E-11
**499**	1.8369476E-12	9.01974216E-12	1.98864101E-11	7.09566497E-11	2.84272843E-11
**500**	1.84146005E-12	9.03744556E-12	1.9908967E-11	7.10216727E-11	2.84350578E-11

Inelastic collisions' Rate Coefficients: Pseudo-Singlet State					
Initial rotational state: j=2					
T(K)	k ($cm^3mol^{-1}s^{-1}$)				
	**Final State: j'=5**	**Final State: j'=4**	**Final State: j'=3**	**Final State: j'=1**	**Final State: j'=0**
**5**	1.08252737E-54	1.39524299E-36	5.83002971E-22	7.79187719E-12	4.85771099E-13
**6**	7.28208584E-48	1.33228466E-32	2.88579311E-20	7.46071954E-12	4.71999224E-13
**7**	5.49872692E-43	9.26914084E-30	4.67046854E-19	7.2086719E-12	4.62194229E-13
**8**	2.51332247E-39	1.25699322E-27	3.76289219E-18	7.01462202E-12	4.55587161E-13
**9**	1.77288019E-36	5.73878649E-26	1.90592748E-17	6.86508331E-12	4.51636539E-13
**10**	3.38354692E-34	1.22403956E-24	6.980119E-17	6.75070037E-12	4.49946416E-13
**11**	2.49709928E-32	1.50215652E-23	2.02021913E-16	6.66467209E-12	4.50223409E-13
**12**	9.04147794E-31	1.21846063E-22	4.90246129E-16	6.60190274E-12	4.52245711E-13
**13**	1.89372114E-29	7.19004029E-22	1.0391433E-15	6.55848681E-12	4.55839542E-13
**14**	2.58068428E-28	3.30509924E-21	1.9808507E-15	6.53137311E-12	4.60862197E-13
**15**	2.49416144E-27	1.2443154E-20	3.46911657E-15	6.51813665E-12	4.67190814E-13
**16**	1.82363174E-26	3.98353067E-20	5.67181846E-15	6.5168199E-12	4.74715536E-13
**17**	1.05975944E-25	1.11595297E-19	8.76323625E-15	6.52582043E-12	4.83335748E-13
**18**	5.08618097E-25	2.79726678E-19	1.29170383E-14	6.54381036E-12	4.92958222E-13
**19**	2.07791785E-24	6.38495132E-19	1.8300504E-14	6.56967772E-12	5.03496354E-13
**20**	7.40315494E-24	1.34589324E-18	2.50701632E-14	6.60248301E-12	5.14869917E-13
**21**	2.34557194E-23	2.64984753E-18	3.33688078E-14	6.6414266E-12	5.27005006E-13
**22**	6.71529722E-23	4.91831938E-18	4.33237067E-14	6.68582371E-12	5.39833997E-13
**23**	1.76033484E-22	8.67208106E-18	5.50458016E-14	6.7350849E-12	5.53295426E-13
**24**	4.27178533E-22	1.46187404E-17	6.86296602E-14	6.78870068E-12	5.67333781E-13
**25**	9.68528319E-22	2.36865427E-17	8.41539779E-14	6.84622899E-12	5.81899204E-13
**26**	2.0677158E-21	3.70565698E-17	1.01682455E-13	6.9072851E-12	5.96947125E-13
**27**	4.18435953E-21	5.61921453E-17	1.2126491E-13	6.9715332E-12	6.12437854E-13
**28**	8.072316E-21	8.28645016E-17	1.42938515E-13	7.03867941E-12	6.28336147E-13
**29**	1.49186166E-20	1.19174034E-16	1.66729084E-13	7.10846591E-12	6.44610772E-13
**30**	2.65256672E-20	1.67566733E-16	1.92652346E-13	7.18066597E-12	6.61234087E-13
**31**	4.55427703E-20	2.30845611E-16	2.20715168E-13	7.25507975E-12	6.78181634E-13
**32**	7.57521753E-20	3.12177172E-16	2.50916707E-13	7.33153066E-12	6.95431765E-13
**33**	1.22413728E-19	4.15093089E-16	2.83249475E-13	7.40986232E-12	7.12965292E-13
**34**	1.92670858E-19	5.43487424E-16	3.17700302E-13	7.48993587E-12	7.30765179E-13
**35**	2.96019187E-19	7.01609769E-16	3.54251215E-13	7.57162775E-12	7.48816256E-13
**36**	4.44837536E-19	8.94054764E-16	3.9288021E-13	7.65482769E-12	7.67104971E-13
**37**	6.54979633E-19	1.12574846E-15	4.33561949E-13	7.73943706E-12	7.8561917E-13
**38**	9.46423461E-19	1.40193203E-15	4.76268363E-13	7.82536737E-12	8.04347901E-13
**39**	1.34397389E-18	1.7281432E-15	5.20969187E-13	7.91253905E-12	8.23281249E-13
**40**	1.8780132E-18	2.11019601E-15	5.67632426E-13	8.00088033E-12	8.42410185E-13
**41**	2.58529315E-18	2.55415915E-15	6.16224751E-13	8.09032627E-12	8.61726444E-13
**42**	3.50976161E-18	3.06633333E-15	6.66711853E-13	8.18081802E-12	8.81222412E-13
**43**	4.70341614E-18	3.65322802E-15	7.19058735E-13	8.27230201E-12	9.00891039E-13
**44**	6.22717682E-18	4.32153787E-15	7.73229974E-13	8.36472939E-12	9.20725751E-13
**45**	8.1517702E-18	5.07811901E-15	8.29189932E-13	8.45805546E-12	9.40720391E-13
**46**	1.05586164E-17	5.92996559E-15	8.86902943E-13	8.55223924E-12	9.60869156E-13
**47**	1.35407116E-17	6.88418656E-15	9.46333468E-13	8.64724301E-12	9.81166549E-13
**48**	1.72034986E-17	7.94798313E-15	1.00744622E-12	8.74303198E-12	1.00160734E-12
**49**	2.16657178E-17	9.12862671E-15	1.07020629E-12	8.83957397E-12	1.02218653E-12
**50**	2.70602321E-17	1.04334378E-14	1.1345792E-12	8.93683916E-12	1.04289933E-12
**51**	3.35348201E-17	1.18697655E-14	1.20053102E-12	9.03479979E-12	1.06374112E-12

**52**	4.12529315E-17	1.34449684E-14	1.26802838E-12	9.13343001E-12	1.08470744E-12
**53**	5.03943993E-17	1.51663959E-14	1.33703855E-12	9.23270568E-12	1.10579397E-12
**54**	6.11561057E-17	1.70413707E-14	1.40752947E-12	9.33260417E-12	1.12699654E-12
**55**	7.37525966E-17	1.9077173E-14	1.47946975E-12	9.43310428E-12	1.14831106E-12
**56**	8.84166427E-17	2.12810243E-14	1.55282874E-12	9.53418605E-12	1.16973358E-12
**57**	1.05399744E-16	2.36600741E-14	1.62757647E-12	9.63583067E-12	1.19126024E-12
**58**	1.24972576E-16	2.62213859E-14	1.70368374E-12	9.73802041E-12	1.21288727E-12
**59**	1.47425377E-16	2.89719252E-14	1.78112205E-12	9.84073847E-12	1.23461098E-12
**60**	1.73068273E-16	3.19185487E-14	1.85986363E-12	9.94396897E-12	1.25642779E-12
**61**	2.02231543E-16	3.50679933E-14	1.93988145E-12	1.00476968E-11	1.27833418E-12
**62**	2.35265826E-16	3.84268675E-14	2.02114918E-12	1.01519077E-11	1.30032671E-12
**63**	2.72542262E-16	4.20016426E-14	2.10364119E-12	1.02565879E-11	1.32240203E-12
**64**	3.1445258E-16	4.57986453E-14	2.18733256E-12	1.03617246E-11	1.34455685E-12
**65**	3.61409121E-16	4.98240509E-14	2.27219903E-12	1.04673052E-11	1.36678796E-12
**66**	4.13844813E-16	5.40838776E-14	2.35821702E-12	1.05733179E-11	1.38909223E-12
**67**	4.72213079E-16	5.85839813E-14	2.44536359E-12	1.06797515E-11	1.41146658E-12
**68**	5.36987708E-16	6.33300509E-14	2.53361643E-12	1.0786595E-11	1.43390802E-12
**69**	6.08662658E-16	6.83276049E-14	2.62295387E-12	1.08938382E-11	1.45641363E-12
**70**	6.87751824E-16	7.35819881E-14	2.7133548E-12	1.10014709E-11	1.47898053E-12
**71**	7.74788751E-16	7.9098369E-14	2.80479873E-12	1.11094837E-11	1.50160595E-12
**72**	8.70326307E-16	8.48817379E-14	2.89726571E-12	1.12178673E-11	1.52428716E-12
**73**	9.74936318E-16	9.09369056E-14	2.99073637E-12	1.13266129E-11	1.5470215E-12
**74**	1.08920915E-15	9.7268502E-14	3.08519183E-12	1.14357121E-11	1.56980638E-12
**75**	1.21375329E-15	1.03880976E-13	3.18061377E-12	1.15451565E-11	1.59263929E-12
**76**	1.34919484E-15	1.10778596E-13	3.27698434E-12	1.16549384E-11	1.61551776E-12
**77**	1.49617701E-15	1.17965447E-13	3.37428619E-12	1.176505E-11	1.63843939E-12
**78**	1.65535961E-15	1.25445436E-13	3.47250241E-12	1.18754841E-11	1.66140187E-12
**79**	1.82741848E-15	1.33222291E-13	3.57161659E-12	1.19862335E-11	1.68440292E-12
**80**	2.01304488E-15	1.4129956E-13	3.6716127E-12	1.20972914E-11	1.70744035E-12
**81**	2.2129449E-15	1.49680614E-13	3.77247519E-12	1.22086512E-11	1.730512E-12
**82**	2.42783883E-15	1.58368652E-13	3.87418887E-12	1.23203064E-11	1.7536158E-12
**83**	2.65846053E-15	1.67366697E-13	3.97673898E-12	1.24322508E-11	1.77674972E-12
**84**	2.90555678E-15	1.76677603E-13	4.08011111E-12	1.25444784E-11	1.79991181E-12
**85**	3.16988655E-15	1.86304056E-13	4.18429124E-12	1.26569834E-11	1.82310016E-12
**86**	3.45222041E-15	1.96248576E-13	4.28926571E-12	1.27697602E-11	1.84631293E-12
**87**	3.75333977E-15	2.06513518E-13	4.39502117E-12	1.28828033E-11	1.86954832E-12
**88**	4.07403621E-15	2.17101079E-13	4.50154463E-12	1.29961072E-11	1.8928046E-12
**89**	4.4151108E-15	2.28013297E-13	4.60882341E-12	1.3109667E-11	1.91608009E-12
**90**	4.77737335E-15	2.39252058E-13	4.71684513E-12	1.32234775E-11	1.93937316E-12
**91**	5.16164172E-15	2.50819093E-13	4.82559773E-12	1.33375339E-11	1.96268224E-12
**92**	5.56874115E-15	2.62715987E-13	4.93506941E-12	1.34518314E-11	1.9860058E-12
**93**	5.99950347E-15	2.74944181E-13	5.04524867E-12	1.35663655E-11	2.00934237E-12
**94**	6.45476648E-15	2.87504971E-13	5.15612424E-12	1.36811316E-11	2.03269053E-12
**95**	6.93537317E-15	3.00399516E-13	5.26768515E-12	1.37961253E-11	2.05604889E-12
**96**	7.44217108E-15	3.13628841E-13	5.37992065E-12	1.39113423E-11	2.07941613E-12
**97**	7.97601155E-15	3.27193837E-13	5.49282024E-12	1.40267785E-11	2.10279095E-12
**98**	8.53774908E-15	3.41095267E-13	5.60637364E-12	1.41424299E-11	2.12617212E-12
**99**	9.12824058E-15	3.55333767E-13	5.7205708E-12	1.42582924E-11	2.14955844E-12
**100**	9.74834475E-15	3.69909854E-13	5.83540188E-12	1.43743621E-11	2.17294875E-12
**101**	1.03989214E-14	3.84823922E-13	5.95085725E-12	1.44906352E-11	2.19634193E-12
**102**	1.10808308E-14	4.00076252E-13	6.06692746E-12	1.46071081E-11	2.21973691E-12

**103**	1.17949328E-14	4.15667012E-13	6.18360328E-12	1.4723777E-11	2.24313266E-12
**104**	1.25420867E-14	4.31596259E-13	6.30087563E-12	1.48406384E-11	2.26652817E-12
**105**	1.33231501E-14	4.47863945E-13	6.41873564E-12	1.49576888E-11	2.28992247E-12
**106**	1.41389785E-14	4.64469917E-13	6.53717458E-12	1.50749247E-11	2.31331464E-12
**107**	1.49904247E-14	4.81413925E-13	6.6561839E-12	1.51923427E-11	2.33670379E-12
**108**	1.58783381E-14	4.98695618E-13	6.77575519E-12	1.53099396E-11	2.36008904E-12
**109**	1.68035643E-14	5.16314552E-13	6.89588023E-12	1.5427712E-11	2.38346958E-12
**110**	1.77669443E-14	5.34270192E-13	7.01655089E-12	1.55456567E-11	2.4068446E-12
**111**	1.87693139E-14	5.52561914E-13	7.13775921E-12	1.56637705E-11	2.43021332E-12
**112**	1.98115036E-14	5.71189007E-13	7.25949737E-12	1.57820503E-11	2.45357502E-12
**113**	2.08943374E-14	5.90150678E-13	7.38175765E-12	1.5900493E-11	2.47692898E-12
**114**	2.20186328E-14	6.09446052E-13	7.50453248E-12	1.60190956E-11	2.50027451E-12
**115**	2.31852E-14	6.29074177E-13	7.62781439E-12	1.61378549E-11	2.52361094E-12
**116**	2.43948415E-14	6.49034025E-13	7.75159603E-12	1.62567681E-11	2.54693765E-12
**117**	2.56483517E-14	6.69324495E-13	7.87587016E-12	1.63758322E-11	2.57025402E-12
**118**	2.69465163E-14	6.89944415E-13	8.00062963E-12	1.64950442E-11	2.59355946E-12
**119**	2.82901117E-14	7.10892545E-13	8.1258674E-12	1.66144013E-11	2.6168534E-12
**120**	2.96799048E-14	7.32167577E-13	8.25157652E-12	1.67339004E-11	2.64013529E-12
**121**	3.11166527E-14	7.53768142E-13	8.37775013E-12	1.68535389E-11	2.66340461E-12
**122**	3.26011018E-14	7.75692808E-13	8.50438147E-12	1.69733138E-11	2.68666085E-12
**123**	3.41339878E-14	7.97940082E-13	8.63146382E-12	1.70932222E-11	2.70990351E-12
**124**	3.57160351E-14	8.20508415E-13	8.75899059E-12	1.72132615E-11	2.73313213E-12
**125**	3.73479566E-14	8.43396201E-13	8.88695523E-12	1.73334287E-11	2.75634625E-12
**126**	3.90304531E-14	8.66601782E-13	9.01535128E-12	1.74537211E-11	2.77954542E-12
**127**	4.07642133E-14	8.90123446E-13	9.14417232E-12	1.75741359E-11	2.80272923E-12
**128**	4.2549913E-14	9.13959433E-13	9.27341203E-12	1.76946703E-11	2.82589726E-12
**129**	4.43882153E-14	9.38107934E-13	9.40306413E-12	1.78153215E-11	2.84904912E-12
**130**	4.62797699E-14	9.62567091E-13	9.53312238E-12	1.79360867E-11	2.87218442E-12
**131**	4.8225213E-14	9.87335006E-13	9.66358064E-12	1.80569633E-11	2.89530278E-12
**132**	5.02251668E-14	1.01240973E-12	9.79443278E-12	1.81779483E-11	2.91840386E-12
**133**	5.22802397E-14	1.03778928E-12	9.92567274E-12	1.82990391E-11	2.94148728E-12
**134**	5.43910255E-14	1.06347164E-12	1.00572945E-11	1.84202328E-11	2.96455273E-12
**135**	5.65581036E-14	1.08945473E-12	1.01892921E-11	1.85415267E-11	2.98759986E-12
**136**	5.87820384E-14	1.11573645E-12	1.03216595E-11	1.8662918E-11	3.01062836E-12
**137**	6.10633796E-14	1.14231466E-12	1.0454391E-11	1.87844039E-11	3.03363791E-12
**138**	6.34026615E-14	1.1691872E-12	1.05874806E-11	1.89059816E-11	3.05662821E-12
**139**	6.58004031E-14	1.19635185E-12	1.07209225E-11	1.90276484E-11	3.07959896E-12
**140**	6.82571076E-14	1.22380638E-12	1.08547109E-11	1.91494013E-11	3.10254986E-12
**141**	7.0773263E-14	1.25154851E-12	1.098884E-11	1.92712376E-11	3.12548064E-12
**142**	7.3349341E-14	1.27957596E-12	1.11233042E-11	1.93931544E-11	3.148391E-12
**143**	7.59857976E-14	1.30788639E-12	1.12580976E-11	1.9515149E-11	3.17128069E-12
**144**	7.86830726E-14	1.33647744E-12	1.13932147E-11	1.96372183E-11	3.19414943E-12
**145**	8.14415897E-14	1.36534675E-12	1.15286497E-11	1.97593597E-11	3.21699695E-12
**146**	8.42617562E-14	1.39449189E-12	1.1664397E-11	1.98815702E-11	3.239823E-12
**147**	8.71439632E-14	1.42391045E-12	1.18004509E-11	2.00038469E-11	3.26262731E-12
**148**	9.00885853E-14	1.45359996E-12	1.1936806E-11	2.01261868E-11	3.28540964E-12
**149**	9.30959806E-14	1.48355796E-12	1.20734565E-11	2.02485872E-11	3.30816973E-12
**150**	9.61664906E-14	1.51378194E-12	1.2210397E-11	2.0371045E-11	3.33090733E-12
**151**	9.93004406E-14	1.54426939E-12	1.23476218E-11	2.04935573E-11	3.3536222E-12
**152**	1.02498139E-13	1.57501778E-12	1.24851254E-11	2.06161212E-11	3.3763141E-12
**153**	1.05759877E-13	1.60602455E-12	1.26229023E-11	2.07387335E-11	3.39898277E-12

**154**	1.09085931E-13	1.63728712E-12	1.2760947E-11	2.08613915E-11	3.42162797E-12
**155**	1.1247656E-13	1.66880292E-12	1.28992539E-11	2.0984092E-11	3.44424948E-12
**156**	1.15932005E-13	1.70056933E-12	1.30378176E-11	2.1106832E-11	3.46684704E-12
**157**	1.19452492E-13	1.73258373E-12	1.31766325E-11	2.12296085E-11	3.48942042E-12
**158**	1.2303823E-13	1.76484349E-12	1.33156932E-11	2.13524185E-11	3.51196937E-12
**159**	1.26689412E-13	1.79734597E-12	1.34549942E-11	2.14752588E-11	3.53449366E-12
**160**	1.30406215E-13	1.8300885E-12	1.359453E-11	2.15981263E-11	3.55699306E-12
**161**	1.34188798E-13	1.86306841E-12	1.37342952E-11	2.1721018E-11	3.57946731E-12
**162**	1.38037306E-13	1.89628301E-12	1.38742843E-11	2.18439308E-11	3.60191617E-12
**163**	1.41951865E-13	1.92972961E-12	1.40144918E-11	2.19668614E-11	3.62433942E-12
**164**	1.45932589E-13	1.96340551E-12	1.41549123E-11	2.20898068E-11	3.6467368E-12
**165**	1.49979571E-13	1.99730799E-12	1.42955404E-11	2.22127639E-11	3.66910807E-12
**166**	1.54092892E-13	2.03143432E-12	1.44363707E-11	2.23357293E-11	3.69145299E-12
**167**	1.58272617E-13	2.06578179E-12	1.45773976E-11	2.24587E-11	3.71377131E-12
**168**	1.62518793E-13	2.10034764E-12	1.47186158E-11	2.25816726E-11	3.73606279E-12
**169**	1.66831454E-13	2.13512913E-12	1.48600198E-11	2.27046441E-11	3.75832718E-12
**170**	1.71210617E-13	2.17012351E-12	1.50016042E-11	2.28276112E-11	3.78056422E-12
**171**	1.75656284E-13	2.20532803E-12	1.51433636E-11	2.29505706E-11	3.80277367E-12
**172**	1.80168443E-13	2.24073991E-12	1.52852926E-11	2.3073519E-11	3.82495527E-12
**173**	1.84747067E-13	2.2763564E-12	1.54273858E-11	2.31964533E-11	3.84710877E-12
**174**	1.89392112E-13	2.31217473E-12	1.55696377E-11	2.33193701E-11	3.86923392E-12
**175**	1.94103523E-13	2.34819211E-12	1.5712043E-11	2.34422661E-11	3.89133044E-12
**176**	1.98881227E-13	2.38440577E-12	1.58545963E-11	2.3565138E-11	3.91339809E-12
**177**	2.03725139E-13	2.42081293E-12	1.59972921E-11	2.36879826E-11	3.9354366E-12
**178**	2.0863516E-13	2.45741081E-12	1.61401252E-11	2.38107965E-11	3.95744571E-12
**179**	2.13611176E-13	2.49419662E-12	1.628309E-11	2.39335764E-11	3.97942514E-12
**180**	2.18653059E-13	2.53116759E-12	1.64261812E-11	2.40563189E-11	4.00137463E-12
**181**	2.23760669E-13	2.56832093E-12	1.65693935E-11	2.41790207E-11	4.02329392E-12
**182**	2.28933852E-13	2.60565385E-12	1.67127214E-11	2.43016784E-11	4.04518272E-12
**183**	2.34172441E-13	2.64316357E-12	1.68561597E-11	2.44242888E-11	4.06704076E-12
**184**	2.39476255E-13	2.68084731E-12	1.69997029E-11	2.45468483E-11	4.08886777E-12
**185**	2.44845102E-13	2.71870229E-12	1.71433457E-11	2.46693538E-11	4.11066347E-12
**186**	2.50278776E-13	2.75672573E-12	1.72870828E-11	2.47918017E-11	4.13242757E-12
**187**	2.5577706E-13	2.79491485E-12	1.74309088E-11	2.49141887E-11	4.1541598E-12
**188**	2.61339724E-13	2.83326688E-12	1.75748184E-11	2.50365115E-11	4.17585987E-12
**189**	2.66966526E-13	2.87177905E-12	1.77188063E-11	2.51587666E-11	4.19752749E-12
**190**	2.72657213E-13	2.91044859E-12	1.78628672E-11	2.52809508E-11	4.21916238E-12
**191**	2.78411521E-13	2.94927274E-12	1.80069958E-11	2.54030604E-11	4.24076424E-12
**192**	2.84229172E-13	2.98824874E-12	1.81511868E-11	2.55250923E-11	4.26233279E-12
**193**	2.90109881E-13	3.02737384E-12	1.82954348E-11	2.5647043E-11	4.28386773E-12
**194**	2.96053349E-13	3.0666453E-12	1.84397347E-11	2.57689092E-11	4.30536877E-12
**195**	3.02059269E-13	3.10606036E-12	1.85840812E-11	2.58906874E-11	4.32683561E-12
**196**	3.0812732E-13	3.14561629E-12	1.87284691E-11	2.60123742E-11	4.34826794E-12
**197**	3.14257176E-13	3.18531037E-12	1.8872893E-11	2.61339663E-11	4.36966548E-12
**198**	3.20448496E-13	3.22513988E-12	1.90173478E-11	2.62554604E-11	4.39102792E-12
**199**	3.26700932E-13	3.26510208E-12	1.91618284E-11	2.63768529E-11	4.41235496E-12
**200**	3.33014127E-13	3.30519429E-12	1.93063294E-11	2.64981407E-11	4.4336463E-12
**201**	3.39387713E-13	3.34541379E-12	1.94508457E-11	2.66193202E-11	4.45490163E-12
**202**	3.45821314E-13	3.38575789E-12	1.95953722E-11	2.67403883E-11	4.47612064E-12
**203**	3.52314545E-13	3.42622392E-12	1.97399037E-11	2.68613414E-11	4.49730303E-12
**204**	3.58867013E-13	3.46680919E-12	1.9884435E-11	2.69821762E-11	4.51844849E-12

**205**	3.65478316E-13	3.50751105E-12	2.00289612E-11	2.71028895E-11	4.53955672E-12
**206**	3.72148044E-13	3.54832683E-12	2.0173477E-11	2.72234779E-11	4.56062739E-12
**207**	3.78875778E-13	3.58925389E-12	2.03179774E-11	2.7343938E-11	4.58166021E-12
**208**	3.85661094E-13	3.63028959E-12	2.04624573E-11	2.74642667E-11	4.60265487E-12
**209**	3.92503559E-13	3.67143131E-12	2.06069118E-11	2.75844605E-11	4.62361104E-12
**210**	3.99402731E-13	3.71267642E-12	2.07513358E-11	2.77045161E-11	4.64452842E-12
**211**	4.06358164E-13	3.75402234E-12	2.08957242E-11	2.78244304E-11	4.6654067E-12
**212**	4.13369404E-13	3.79546646E-12	2.10400722E-11	2.79442001E-11	4.68624557E-12
**213**	4.2043599E-13	3.8370062E-12	2.11843747E-11	2.80638218E-11	4.7070447E-12
**214**	4.27557454E-13	3.87863899E-12	2.13286268E-11	2.81832924E-11	4.7278038E-12
**215**	4.34733325E-13	3.92036227E-12	2.14728237E-11	2.83026085E-11	4.74852254E-12
**216**	4.41963121E-13	3.9621735E-12	2.16169603E-11	2.84217672E-11	4.76920061E-12
**217**	4.49246358E-13	4.00407015E-12	2.1761032E-11	2.8540765E-11	4.78983771E-12
**218**	4.56582547E-13	4.04604968E-12	2.19050338E-11	2.86595988E-11	4.81043351E-12
**219**	4.6397119E-13	4.08810961E-12	2.20489609E-11	2.87782656E-11	4.83098771E-12
**220**	4.71411786E-13	4.13024742E-12	2.21928085E-11	2.8896762E-11	4.85149999E-12
**221**	4.78903831E-13	4.17246064E-12	2.23365718E-11	2.90150851E-11	4.87197004E-12
**222**	4.86446812E-13	4.21474681E-12	2.24802462E-11	2.91332316E-11	4.89239755E-12
**223**	4.94040215E-13	4.25710346E-12	2.2623827E-11	2.92511985E-11	4.91278222E-12
**224**	5.01683519E-13	4.29952815E-12	2.27673093E-11	2.93689827E-11	4.93312372E-12
**225**	5.09376202E-13	4.34201847E-12	2.29106886E-11	2.94865811E-11	4.95342175E-12
**226**	5.17117735E-13	4.384572E-12	2.30539603E-11	2.96039907E-11	4.97367601E-12
**227**	5.24907586E-13	4.42718634E-12	2.31971197E-11	2.97212085E-11	4.99388619E-12
**228**	5.32745221E-13	4.46985911E-12	2.33401624E-11	2.98382314E-11	5.01405197E-12
**229**	5.406301E-13	4.51258794E-12	2.34830836E-11	2.99550566E-11	5.03417306E-12
**230**	5.48561682E-13	4.55537048E-12	2.3625879E-11	3.00716809E-11	5.05424914E-12
**231**	5.56539422E-13	4.59820439E-12	2.37685441E-11	3.01881016E-11	5.07427993E-12
**232**	5.64562772E-13	4.64108736E-12	2.39110743E-11	3.03043156E-11	5.09426511E-12
**233**	5.72631181E-13	4.68401707E-12	2.40534654E-11	3.04203201E-11	5.11420438E-12
**234**	5.80744096E-13	4.72699123E-12	2.41957128E-11	3.05361121E-11	5.13409746E-12
**235**	5.88900962E-13	4.77000757E-12	2.43378123E-11	3.0651689E-11	5.15394403E-12
**236**	5.9710122E-13	4.81306384E-12	2.44797596E-11	3.07670477E-11	5.1737438E-12
**237**	6.05344312E-13	4.85615777E-12	2.46215502E-11	3.08821855E-11	5.19349648E-12
**238**	6.13629676E-13	4.89928716E-12	2.47631801E-11	3.09970997E-11	5.21320178E-12
**239**	6.21956747E-13	4.94244979E-12	2.49046449E-11	3.11117874E-11	5.23285941E-12
**240**	6.30324961E-13	4.98564346E-12	2.50459405E-11	3.12262459E-11	5.25246907E-12
**241**	6.38733752E-13	5.028866E-12	2.51870627E-11	3.13404726E-11	5.27203048E-12
**242**	6.47182551E-13	5.07211525E-12	2.53280075E-11	3.14544647E-11	5.29154335E-12
**243**	6.55670791E-13	5.11538906E-12	2.54687706E-11	3.15682196E-11	5.31100741E-12
**244**	6.64197901E-13	5.1586853E-12	2.56093481E-11	3.16817346E-11	5.33042237E-12
**245**	6.7276331E-13	5.20200187E-12	2.57497359E-11	3.17950071E-11	5.34978795E-12
**246**	6.81366447E-13	5.24533666E-12	2.58899301E-11	3.19080346E-11	5.36910387E-12
**247**	6.9000674E-13	5.28868761E-12	2.60299267E-11	3.20208145E-11	5.38836986E-12
**248**	6.98683616E-13	5.33205266E-12	2.61697218E-11	3.21333443E-11	5.40758565E-12
**249**	7.07396504E-13	5.37542975E-12	2.63093116E-11	3.22456214E-11	5.42675097E-12
**250**	7.1614483E-13	5.41881687E-12	2.64486921E-11	3.23576434E-11	5.44586555E-12
**251**	7.2492802E-13	5.46221201E-12	2.65878597E-11	3.24694078E-11	5.46492913E-12
**252**	7.33745503E-13	5.50561317E-12	2.67268105E-11	3.25809122E-11	5.48394143E-12
**253**	7.42596705E-13	5.54901839E-12	2.68655407E-11	3.26921542E-11	5.50290221E-12
**254**	7.51481055E-13	5.59242571E-12	2.70040468E-11	3.28031314E-11	5.5218112E-12
**255**	7.60397979E-13	5.63583319E-12	2.71423251E-11	3.29138416E-11	5.54066815E-12

**256**	7.69346907E-13	5.67923891E-12	2.72803719E-11	3.30242823E-11	5.55947281E-12
**257**	7.78327268E-13	5.72264096E-12	2.74181837E-11	3.31344513E-11	5.57822493E-12
**258**	7.87338492E-13	5.76603746E-12	2.75557568E-11	3.32443463E-11	5.59692426E-12
**259**	7.9638001E-13	5.80942655E-12	2.76930879E-11	3.33539651E-11	5.61557055E-12
**260**	8.05451254E-13	5.85280637E-12	2.78301735E-11	3.34633055E-11	5.63416357E-12
**261**	8.14551656E-13	5.89617509E-12	2.79670101E-11	3.35723653E-11	5.65270307E-12
**262**	8.23680651E-13	5.9395309E-12	2.81035943E-11	3.36811424E-11	5.67118882E-12
**263**	8.32837674E-13	5.98287199E-12	2.82399228E-11	3.37896346E-11	5.68962059E-12
**264**	8.42022161E-13	6.02619659E-12	2.83759923E-11	3.38978399E-11	5.70799815E-12
**265**	8.51233552E-13	6.06950293E-12	2.85117995E-11	3.40057562E-11	5.72632127E-12
**266**	8.60471284E-13	6.11278928E-12	2.86473411E-11	3.41133815E-11	5.74458972E-12
**267**	8.69734801E-13	6.1560539E-12	2.8782614E-11	3.42207137E-11	5.76280329E-12
**268**	8.79023545E-13	6.19929508E-12	2.8917615E-11	3.43277509E-11	5.78096176E-12
**269**	8.8833696E-13	6.24251114E-12	2.90523409E-11	3.44344911E-11	5.79906491E-12
**270**	8.97674494E-13	6.28570041E-12	2.91867888E-11	3.45409324E-11	5.81711253E-12
**271**	9.07035594E-13	6.32886121E-12	2.93209555E-11	3.46470729E-11	5.83510441E-12
**272**	9.16419713E-13	6.37199193E-12	2.9454838E-11	3.47529108E-11	5.85304034E-12
**273**	9.25826302E-13	6.41509092E-12	2.95884334E-11	3.48584442E-11	5.87092013E-12
**274**	9.35254817E-13	6.45815661E-12	2.97217388E-11	3.49636713E-11	5.88874357E-12
**275**	9.44704714E-13	6.50118738E-12	2.98547512E-11	3.50685904E-11	5.90651047E-12
**276**	9.54175454E-13	6.54418169E-12	2.99874678E-11	3.51731996E-11	5.92422063E-12
**277**	9.63666498E-13	6.58713797E-12	3.01198857E-11	3.52774973E-11	5.94187387E-12
**278**	9.7317731E-13	6.6300547E-12	3.02520023E-11	3.53814818E-11	5.95947E-12
**279**	9.82707358E-13	6.67293036E-12	3.03838147E-11	3.54851514E-11	5.97700883E-12
**280**	9.92256111E-13	6.71576345E-12	3.05153203E-11	3.55885044E-11	5.99449018E-12
**281**	1.00182304E-12	6.75855248E-12	3.06465164E-11	3.56915394E-11	6.01191388E-12
**282**	1.01140762E-12	6.80129601E-12	3.07774004E-11	3.57942546E-11	6.02927975E-12
**283**	1.02100933E-12	6.84399256E-12	3.09079696E-11	3.58966485E-11	6.04658763E-12
**284**	1.03062765E-12	6.88664073E-12	3.10382216E-11	3.59987197E-11	6.06383734E-12
**285**	1.04026206E-12	6.9292391E-12	3.11681538E-11	3.61004666E-11	6.08102872E-12
**286**	1.04991205E-12	6.97178626E-12	3.12977637E-11	3.62018877E-11	6.09816161E-12
**287**	1.05957711E-12	7.01428085E-12	3.1427049E-11	3.63029817E-11	6.11523586E-12
**288**	1.06925673E-12	7.05672149E-12	3.15560071E-11	3.6403747E-11	6.1322513E-12
**289**	1.0789504E-12	7.09910686E-12	3.16846359E-11	3.65041824E-11	6.14920779E-12
**290**	1.08865762E-12	7.14143561E-12	3.18129328E-11	3.66042865E-11	6.16610519E-12
**291**	1.09837789E-12	7.18370643E-12	3.19408957E-11	3.67040579E-11	6.18294334E-12
**292**	1.10811072E-12	7.22591804E-12	3.20685223E-11	3.68034953E-11	6.1997221E-12
**293**	1.11785562E-12	7.26806916E-12	3.21958103E-11	3.69025975E-11	6.21644134E-12
**294**	1.12761209E-12	7.31015851E-12	3.23227577E-11	3.70013632E-11	6.23310093E-12
**295**	1.13737965E-12	7.35218487E-12	3.24493622E-11	3.70997912E-11	6.24970073E-12
**296**	1.14715781E-12	7.39414699E-12	3.25756218E-11	3.71978803E-11	6.26624061E-12
**297**	1.15694611E-12	7.43604367E-12	3.27015343E-11	3.72956294E-11	6.28272045E-12
**298**	1.16674405E-12	7.47787371E-12	3.28270978E-11	3.73930373E-11	6.29914013E-12
**299**	1.17655116E-12	7.51963594E-12	3.29523103E-11	3.74901029E-11	6.31549953E-12
**300**	1.18636699E-12	7.56132918E-12	3.30771697E-11	3.7586825E-11	6.33179853E-12
**301**	1.19619106E-12	7.60295229E-12	3.32016743E-11	3.76832027E-11	6.34803703E-12
**302**	1.2060229E-12	7.64450414E-12	3.3325822E-11	3.7779235E-11	6.36421492E-12
**303**	1.21586207E-12	7.68598361E-12	3.3449611E-11	3.78749207E-11	6.38033208E-12
**304**	1.2257081E-12	7.7273896E-12	3.35730395E-11	3.7970259E-11	6.39638842E-12
**305**	1.23556054E-12	7.76872103E-12	3.36961058E-11	3.80652488E-11	6.41238384E-12
**306**	1.24541894E-12	7.80997683E-12	3.3818808E-11	3.81598893E-11	6.42831824E-12

**307**	1.25528286E-12	7.85115594E-12	3.39411445E-11	3.82541795E-11	6.44419154E-12
**308**	1.26515185E-12	7.89225732E-12	3.40631136E-11	3.83481186E-11	6.46000364E-12
**309**	1.27502549E-12	7.93327995E-12	3.41847135E-11	3.84417057E-11	6.47575445E-12
**310**	1.28490333E-12	7.97422283E-12	3.43059428E-11	3.85349399E-11	6.4914439E-12
**311**	1.29478494E-12	8.01508496E-12	3.44267999E-11	3.86278205E-11	6.5070719E-12
**312**	1.30466989E-12	8.05586536E-12	3.45472831E-11	3.87203467E-11	6.52263838E-12
**313**	1.31455777E-12	8.09656308E-12	3.46673909E-11	3.88125177E-11	6.53814326E-12
**314**	1.32444815E-12	8.13717715E-12	3.4787122E-11	3.89043329E-11	6.55358647E-12
**315**	1.33434062E-12	8.17770665E-12	3.49064748E-11	3.89957914E-11	6.56896795E-12
**316**	1.34423475E-12	8.21815066E-12	3.50254479E-11	3.90868926E-11	6.58428764E-12
**317**	1.35413016E-12	8.25850828E-12	3.514404E-11	3.91776358E-11	6.59954546E-12
**318**	1.36402642E-12	8.29877861E-12	3.52622497E-11	3.92680205E-11	6.61474136E-12
**319**	1.37392313E-12	8.33896078E-12	3.53800756E-11	3.93580459E-11	6.62987529E-12
**320**	1.38381991E-12	8.37905392E-12	3.54975165E-11	3.94477116E-11	6.6449472E-12
**321**	1.39371635E-12	8.4190572E-12	3.56145712E-11	3.95370169E-11	6.65995703E-12
**322**	1.40361207E-12	8.45896977E-12	3.57312383E-11	3.96259613E-11	6.67490474E-12
**323**	1.41350667E-12	8.49879082E-12	3.58475168E-11	3.97145442E-11	6.68979028E-12
**324**	1.42339977E-12	8.53851954E-12	3.59634054E-11	3.98027652E-11	6.70461362E-12
**325**	1.433291E-12	8.57815513E-12	3.6078903E-11	3.98906238E-11	6.71937472E-12
**326**	1.44317998E-12	8.61769683E-12	3.61940085E-11	3.99781195E-11	6.73407355E-12
**327**	1.45306633E-12	8.65714387E-12	3.63087208E-11	4.00652519E-11	6.74871006E-12
**328**	1.46294969E-12	8.69649548E-12	3.6423039E-11	4.01520206E-11	6.76328424E-12
**329**	1.47282969E-12	8.73575095E-12	3.65369619E-11	4.02384252E-11	6.77779605E-12
**330**	1.48270597E-12	8.77490953E-12	3.66504886E-11	4.03244654E-11	6.79224547E-12
**331**	1.49257818E-12	8.81397052E-12	3.67636181E-11	4.04101407E-11	6.80663249E-12
**332**	1.50244596E-12	8.85293322E-12	3.68763496E-11	4.04954508E-11	6.82095708E-12
**333**	1.51230895E-12	8.89179694E-12	3.6988682E-11	4.05803955E-11	6.83521924E-12
**334**	1.52216681E-12	8.93056101E-12	3.71006146E-11	4.06649744E-11	6.84941894E-12
**335**	1.53201921E-12	8.96922477E-12	3.72121465E-11	4.07491873E-11	6.86355618E-12
**336**	1.54186579E-12	9.00778757E-12	3.73232768E-11	4.0833034E-11	6.87763095E-12
**337**	1.55170623E-12	9.04624878E-12	3.74340049E-11	4.09165141E-11	6.89164324E-12
**338**	1.5615402E-12	9.08460776E-12	3.75443299E-11	4.09996276E-11	6.90559306E-12
**339**	1.57136735E-12	9.12286391E-12	3.76542511E-11	4.10823742E-11	6.91948041E-12
**340**	1.58118737E-12	9.16101663E-12	3.77637678E-11	4.11647537E-11	6.93330529E-12
**341**	1.59099994E-12	9.19906533E-12	3.78728793E-11	4.1246766E-11	6.9470677E-12
**342**	1.60080474E-12	9.23700944E-12	3.79815849E-11	4.1328411E-11	6.96076765E-12
**343**	1.61060146E-12	9.27484839E-12	3.80898841E-11	4.14096885E-11	6.97440517E-12
**344**	1.62038978E-12	9.31258163E-12	3.81977763E-11	4.14905985E-11	6.98798025E-12
**345**	1.63016939E-12	9.35020863E-12	3.83052608E-11	4.15711409E-11	7.00149291E-12
**346**	1.63994E-12	9.38772884E-12	3.84123371E-11	4.16513155E-11	7.01494318E-12
**347**	1.6497013E-12	9.42514176E-12	3.85190047E-11	4.17311225E-11	7.02833107E-12
**348**	1.659453E-12	9.46244688E-12	3.8625263E-11	4.18105618E-11	7.04165661E-12
**349**	1.66919481E-12	9.4996437E-12	3.87311117E-11	4.18896333E-11	7.05491983E-12
**350**	1.67892643E-12	9.53673174E-12	3.88365502E-11	4.1968337E-11	7.06812075E-12
**351**	1.68864757E-12	9.57371052E-12	3.89415782E-11	4.20466731E-11	7.08125939E-12
**352**	1.69835796E-12	9.61057959E-12	3.90461951E-11	4.21246415E-11	7.09433581E-12
**353**	1.70805732E-12	9.64733849E-12	3.91504008E-11	4.22022424E-11	7.10735002E-12
**354**	1.71774537E-12	9.68398679E-12	3.92541946E-11	4.22794757E-11	7.12030207E-12
**355**	1.72742183E-12	9.72052404E-12	3.93575765E-11	4.23563416E-11	7.133192E-12
**356**	1.73708644E-12	9.75694984E-12	3.9460546E-11	4.24328402E-11	7.14601984E-12
**357**	1.74673894E-12	9.79326376E-12	3.95631028E-11	4.25089717E-11	7.15878565E-12

**358**	1.75637905E-12	9.82946543E-12	3.96652467E-11	4.25847361E-11	7.17148947E-12
**359**	1.76600653E-12	9.86555443E-12	3.97669775E-11	4.26601337E-11	7.18413134E-12
**360**	1.77562111E-12	9.9015304E-12	3.98682948E-11	4.27351645E-11	7.19671132E-12
**361**	1.78522254E-12	9.93739296E-12	3.99691986E-11	4.28098289E-11	7.20922946E-12
**362**	1.79481057E-12	9.97314176E-12	4.00696886E-11	4.2884127E-11	7.22168581E-12
**363**	1.80438497E-12	1.00087765E-11	4.01697647E-11	4.2958059E-11	7.23408043E-12
**364**	1.81394547E-12	1.00442967E-11	4.02694267E-11	4.30316251E-11	7.24641338E-12
**365**	1.82349186E-12	1.00797021E-11	4.03686746E-11	4.31048257E-11	7.25868472E-12
**366**	1.83302388E-12	1.01149925E-11	4.04675082E-11	4.31776609E-11	7.27089451E-12
**367**	1.8425413E-12	1.01501674E-11	4.05659274E-11	4.32501311E-11	7.28304282E-12
**368**	1.8520439E-12	1.01852266E-11	4.06639322E-11	4.33222365E-11	7.29512971E-12
**369**	1.86153145E-12	1.02201698E-11	4.07615226E-11	4.33939775E-11	7.30715525E-12
**370**	1.87100373E-12	1.02549967E-11	4.08586985E-11	4.34653543E-11	7.31911951E-12
**371**	1.8804605E-12	1.0289707E-11	4.09554599E-11	4.35363673E-11	7.33102256E-12
**372**	1.88990157E-12	1.03243005E-11	4.10518069E-11	4.36070169E-11	7.34286448E-12
**373**	1.89932671E-12	1.03587769E-11	4.11477395E-11	4.36773033E-11	7.35464534E-12
**374**	1.9087357E-12	1.0393136E-11	4.12432578E-11	4.3747227E-11	7.36636522E-12
**375**	1.91812835E-12	1.04273774E-11	4.13383618E-11	4.38167884E-11	7.3780242E-12
**376**	1.92750445E-12	1.04615011E-11	4.14330516E-11	4.38859879E-11	7.38962236E-12
**377**	1.93686379E-12	1.04955067E-11	4.15273274E-11	4.39548257E-11	7.40115979E-12
**378**	1.94620617E-12	1.0529394E-11	4.16211892E-11	4.40233025E-11	7.41263655E-12
**379**	1.95553141E-12	1.05631629E-11	4.17146373E-11	4.40914186E-11	7.42405276E-12
**380**	1.96483929E-12	1.05968131E-11	4.18076717E-11	4.41591744E-11	7.43540848E-12
**381**	1.97412964E-12	1.06303444E-11	4.19002927E-11	4.42265704E-11	7.44670381E-12
**382**	1.98340227E-12	1.06637567E-11	4.19925005E-11	4.42936071E-11	7.45793884E-12
**383**	1.99265699E-12	1.06970498E-11	4.20842952E-11	4.4360285E-11	7.46911366E-12
**384**	2.00189362E-12	1.07302236E-11	4.21756772E-11	4.44266045E-11	7.48022837E-12
**385**	2.01111197E-12	1.07632777E-11	4.22666466E-11	4.44925661E-11	7.49128306E-12
**386**	2.02031188E-12	1.07962122E-11	4.23572037E-11	4.45581704E-11	7.50227784E-12
**387**	2.02949317E-12	1.08290269E-11	4.24473489E-11	4.46234179E-11	7.51321278E-12
**388**	2.03865567E-12	1.08617215E-11	4.25370823E-11	4.46883091E-11	7.52408801E-12
**389**	2.0477992E-12	1.08942961E-11	4.26264043E-11	4.47528445E-11	7.53490362E-12
**390**	2.05692361E-12	1.09267504E-11	4.27153153E-11	4.48170248E-11	7.5456597E-12
**391**	2.06602873E-12	1.09590843E-11	4.28038155E-11	4.48808504E-11	7.55635638E-12
**392**	2.07511441E-12	1.09912979E-11	4.28919054E-11	4.49443219E-11	7.56699374E-12
**393**	2.08418047E-12	1.10233908E-11	4.29795852E-11	4.50074399E-11	7.57757191E-12
**394**	2.09322678E-12	1.10553631E-11	4.30668554E-11	4.50702051E-11	7.58809098E-12
**395**	2.10225317E-12	1.10872147E-11	4.31537164E-11	4.5132618E-11	7.59855107E-12
**396**	2.11125949E-12	1.11189454E-11	4.32401686E-11	4.51946792E-11	7.60895229E-12
**397**	2.12024561E-12	1.11505552E-11	4.33262124E-11	4.52563893E-11	7.61929474E-12
**398**	2.12921137E-12	1.1182044E-11	4.34118482E-11	4.5317749E-11	7.62957855E-12
**399**	2.13815663E-12	1.12134119E-11	4.34970765E-11	4.53787589E-11	7.63980383E-12
**400**	2.14708125E-12	1.12446586E-11	4.35818978E-11	4.54394196E-11	7.64997069E-12
**401**	2.1559851E-12	1.12757842E-11	4.36663125E-11	4.54997318E-11	7.66007926E-12
**402**	2.16486804E-12	1.13067886E-11	4.37503212E-11	4.55596963E-11	7.67012964E-12
**403**	2.17372994E-12	1.13376718E-11	4.38339242E-11	4.56193135E-11	7.68012196E-12
**404**	2.18257067E-12	1.13684337E-11	4.39171223E-11	4.56785842E-11	7.69005634E-12
**405**	2.19139009E-12	1.13990743E-11	4.39999157E-11	4.57375092E-11	7.6999329E-12
**406**	2.2001881E-12	1.14295937E-11	4.40823052E-11	4.5796089E-11	7.70975177E-12
**407**	2.20896455E-12	1.14599917E-11	4.41642913E-11	4.58543244E-11	7.71951306E-12
**408**	2.21771934E-12	1.14902685E-11	4.42458745E-11	4.59122162E-11	7.72921691E-12

**409**	2.22645234E-12	1.15204238E-11	4.43270554E-11	4.59697649E-11	7.73886344E-12
**410**	2.23516344E-12	1.15504579E-11	4.44078345E-11	4.60269714E-11	7.74845277E-12
**411**	2.24385253E-12	1.15803707E-11	4.44882126E-11	4.60838363E-11	7.75798504E-12
**412**	2.25251948E-12	1.16101622E-11	4.45681901E-11	4.61403605E-11	7.76746037E-12
**413**	2.26116421E-12	1.16398324E-11	4.46477677E-11	4.61965446E-11	7.77687889E-12
**414**	2.26978658E-12	1.16693813E-11	4.4726946E-11	4.62523894E-11	7.78624073E-12
**415**	2.27838652E-12	1.1698809E-11	4.48057257E-11	4.63078957E-11	7.79554603E-12
**416**	2.2869639E-12	1.17281156E-11	4.48841075E-11	4.63630642E-11	7.80479492E-12
**417**	2.29551863E-12	1.17573009E-11	4.49620919E-11	4.64178956E-11	7.81398753E-12
**418**	2.30405061E-12	1.17863652E-11	4.50396796E-11	4.64723909E-11	7.823124E-12
**419**	2.31255974E-12	1.18153085E-11	4.51168713E-11	4.65265507E-11	7.83220446E-12
**420**	2.32104594E-12	1.18441307E-11	4.51936678E-11	4.65803758E-11	7.84122904E-12
**421**	2.3295091E-12	1.1872832E-11	4.52700697E-11	4.66338671E-11	7.8501979E-12
**422**	2.33794914E-12	1.19014124E-11	4.53460776E-11	4.66870253E-11	7.85911115E-12
**423**	2.34636598E-12	1.1929872E-11	4.54216924E-11	4.67398512E-11	7.86796894E-12
**424**	2.35475951E-12	1.19582109E-11	4.54969148E-11	4.67923457E-11	7.87677142E-12
**425**	2.36312967E-12	1.19864291E-11	4.55717454E-11	4.68445095E-11	7.88551872E-12
**426**	2.37147636E-12	1.20145268E-11	4.5646185E-11	4.68963435E-11	7.89421097E-12
**427**	2.37979951E-12	1.2042504E-11	4.57202344E-11	4.69478485E-11	7.90284834E-12
**428**	2.38809904E-12	1.20703607E-11	4.57938943E-11	4.69990254E-11	7.91143094E-12
**429**	2.39637488E-12	1.20980972E-11	4.58671656E-11	4.70498749E-11	7.91995894E-12
**430**	2.40462694E-12	1.21257135E-11	4.59400489E-11	4.7100398E-11	7.92843246E-12
**431**	2.41285516E-12	1.21532097E-11	4.6012545E-11	4.71505954E-11	7.93685167E-12
**432**	2.42105946E-12	1.21805859E-11	4.60846548E-11	4.7200468E-11	7.94521669E-12
**433**	2.42923978E-12	1.22078423E-11	4.6156379E-11	4.72500167E-11	7.95352768E-12
**434**	2.43739605E-12	1.22349789E-11	4.62277185E-11	4.72992423E-11	7.96178479E-12
**435**	2.44552821E-12	1.22619958E-11	4.6298674E-11	4.73481457E-11	7.96998815E-12
**436**	2.45363618E-12	1.22888933E-11	4.63692464E-11	4.73967278E-11	7.97813792E-12
**437**	2.46171991E-12	1.23156713E-11	4.64394364E-11	4.74449894E-11	7.98623425E-12
**438**	2.46977934E-12	1.23423301E-11	4.6509245E-11	4.74929314E-11	7.99427728E-12
**439**	2.47781441E-12	1.23688698E-11	4.6578673E-11	4.75405546E-11	8.00226717E-12
**440**	2.48582506E-12	1.23952905E-11	4.66477212E-11	4.758786E-11	8.01020405E-12
**441**	2.49381124E-12	1.24215924E-11	4.67163905E-11	4.76348485E-11	8.01808809E-12
**442**	2.5017729E-12	1.24477756E-11	4.67846816E-11	4.76815209E-11	8.02591943E-12
**443**	2.50970998E-12	1.24738402E-11	4.68525956E-11	4.77278781E-11	8.03369822E-12
**444**	2.51762243E-12	1.24997865E-11	4.69201332E-11	4.77739211E-11	8.04142462E-12
**445**	2.5255102E-12	1.25256145E-11	4.69872954E-11	4.78196506E-11	8.04909878E-12
**446**	2.53337326E-12	1.25513244E-11	4.7054083E-11	4.78650677E-11	8.05672085E-12
**447**	2.54121154E-12	1.25769164E-11	4.71204969E-11	4.79101733E-11	8.06429098E-12
**448**	2.54902502E-12	1.26023907E-11	4.7186538E-11	4.79549682E-11	8.07180932E-12
**449**	2.55681364E-12	1.26277474E-11	4.72522072E-11	4.79994533E-11	8.07927604E-12
**450**	2.56457737E-12	1.26529867E-11	4.73175054E-11	4.80436296E-11	8.08669128E-12
**451**	2.57231616E-12	1.26781088E-11	4.73824335E-11	4.8087498E-11	8.09405519E-12
**452**	2.58002998E-12	1.27031138E-11	4.74469924E-11	4.81310594E-11	8.10136794E-12
**453**	2.58771879E-12	1.27280019E-11	4.75111831E-11	4.81743147E-11	8.10862968E-12
**454**	2.59538256E-12	1.27527734E-11	4.75750065E-11	4.82172649E-11	8.11584056E-12
**455**	2.60302126E-12	1.27774283E-11	4.76384634E-11	4.82599108E-11	8.12300074E-12
**456**	2.61063485E-12	1.2801967E-11	4.7701555E-11	4.83022535E-11	8.13011037E-12
**457**	2.6182233E-12	1.28263895E-11	4.77642819E-11	4.83442938E-11	8.13716962E-12
**458**	2.62578658E-12	1.28506961E-11	4.78266453E-11	4.83860327E-11	8.14417863E-12
**459**	2.63332467E-12	1.28748869E-11	4.78886461E-11	4.84274711E-11	8.15113757E-12

**460**	2.64083753E-12	1.28989622E-11	4.79502852E-11	4.84686099E-11	8.15804659E-12
**461**	2.64832515E-12	1.29229222E-11	4.80115636E-11	4.85094501E-11	8.16490585E-12
**462**	2.6557875E-12	1.29467671E-11	4.80724822E-11	4.85499927E-11	8.17171551E-12
**463**	2.66322455E-12	1.29704971E-11	4.8133042E-11	4.85902385E-11	8.17847572E-12
**464**	2.67063629E-12	1.29941123E-11	4.81932439E-11	4.86301886E-11	8.18518665E-12
**465**	2.6780227E-12	1.30176131E-11	4.8253089E-11	4.86698438E-11	8.19184845E-12
**466**	2.68538375E-12	1.30409996E-11	4.83125782E-11	4.87092051E-11	8.19846128E-12
**467**	2.69271944E-12	1.3064272E-11	4.83717125E-11	4.87482735E-11	8.2050253E-12
**468**	2.70002974E-12	1.30874306E-11	4.84304928E-11	4.87870499E-11	8.21154067E-12
**469**	2.70731463E-12	1.31104755E-11	4.84889202E-11	4.88255353E-11	8.21800755E-12
**470**	2.71457411E-12	1.31334071E-11	4.85469956E-11	4.88637305E-11	8.2244261E-12
**471**	2.72180817E-12	1.31562255E-11	4.86047201E-11	4.89016367E-11	8.23079647E-12
**472**	2.72901678E-12	1.31789309E-11	4.86620946E-11	4.89392547E-11	8.23711882E-12
**473**	2.73619995E-12	1.32015236E-11	4.87191201E-11	4.89765855E-11	8.24339333E-12
**474**	2.74335766E-12	1.32240039E-11	4.87757976E-11	4.901363E-11	8.24962013E-12
**475**	2.7504899E-12	1.32463719E-11	4.88321282E-11	4.90503892E-11	8.25579941E-12
**476**	2.75759667E-12	1.32686279E-11	4.88881128E-11	4.90868641E-11	8.26193131E-12
**477**	2.76467796E-12	1.3290772E-11	4.89437524E-11	4.91230556E-11	8.26801599E-12
**478**	2.77173376E-12	1.33128047E-11	4.8999048E-11	4.91589647E-11	8.27405363E-12
**479**	2.77876408E-12	1.33347261E-11	4.90540007E-11	4.91945923E-11	8.28004437E-12
**480**	2.78576891E-12	1.33565364E-11	4.91086115E-11	4.92299395E-11	8.28598837E-12
**481**	2.79274825E-12	1.33782359E-11	4.91628814E-11	4.92650071E-11	8.2918858E-12
**482**	2.7997021E-12	1.33998248E-11	4.92168113E-11	4.92997962E-11	8.29773682E-12
**483**	2.80663046E-12	1.34213034E-11	4.92704024E-11	4.93343076E-11	8.30354159E-12
**484**	2.81353332E-12	1.34426719E-11	4.93236557E-11	4.93685425E-11	8.30930027E-12
**485**	2.82041071E-12	1.34639307E-11	4.93765721E-11	4.94025016E-11	8.31501302E-12
**486**	2.82726261E-12	1.34850798E-11	4.94291527E-11	4.9436186E-11	8.32067999E-12
**487**	2.83408903E-12	1.35061197E-11	4.94813986E-11	4.94695967E-11	8.32630136E-12
**488**	2.84088997E-12	1.35270505E-11	4.95333107E-11	4.95027347E-11	8.33187728E-12
**489**	2.84766545E-12	1.35478725E-11	4.95848901E-11	4.95356008E-11	8.33740791E-12
**490**	2.85441548E-12	1.35685859E-11	4.96361378E-11	4.95681961E-11	8.34289341E-12
**491**	2.86114005E-12	1.35891911E-11	4.96870549E-11	4.96005215E-11	8.34833395E-12
**492**	2.86783918E-12	1.36096883E-11	4.97376423E-11	4.96325779E-11	8.35372968E-12
**493**	2.87451287E-12	1.36300777E-11	4.97879012E-11	4.96643665E-11	8.35908076E-12
**494**	2.88116115E-12	1.36503596E-11	4.98378326E-11	4.9695888E-11	8.36438736E-12
**495**	2.88778402E-12	1.36705343E-11	4.98874375E-11	4.97271436E-11	8.36964963E-12
**496**	2.89438149E-12	1.3690602E-11	4.99367169E-11	4.9758134E-11	8.37486774E-12
**497**	2.90095358E-12	1.3710563E-11	4.99856719E-11	4.97888604E-11	8.38004184E-12
**498**	2.90750031E-12	1.37304176E-11	5.00343035E-11	4.98193237E-11	8.3851721E-12
**499**	2.91402168E-12	1.37501661E-11	5.00826129E-11	4.98495248E-11	8.39025867E-12
**500**	2.92051771E-12	1.37698086E-11	5.01306009E-11	4.98794647E-11	8.39530172E-12

Inelastic collisions' Rate Coefficients: Pseudo-Singlet State					
Initial rotational state: j=3					
T(K)	k ($cm^3mol^{-1}s^{-1}$)				
	**Final State: j'=5**	**Final State: j'=4**	**Final State: j'=2**	**Final State: j'=1**	**Final State: j'=0**
**5**	8.96574277E-44	1.05449342E-25	7.52468474E-12	1.61762816E-12	5.51239106E-13
**6**	1.15720607E-38	1.96776319E-23	7.2709608E-12	1.5843544E-12	5.41386233E-13
**7**	5.18360183E-35	8.22960838E-22	7.07330057E-12	1.55866131E-12	5.34027311E-13
**8**	2.84587286E-32	1.35373536E-20	6.9180664E-12	1.53964218E-12	5.28958159E-13
**9**	3.85906684E-30	1.1969394E-19	6.79643916E-12	1.52647376E-12	5.25955036E-13
**10**	1.9676749E-28	6.85914276E-19	6.70204871E-12	1.51839119E-12	5.24790974E-13
**11**	4.92866097E-27	2.86917826E-18	6.63007489E-12	1.51471615E-12	5.25256254E-13
**12**	7.24723093E-26	9.48175443E-18	6.57678223E-12	1.51486543E-12	5.27166502E-13
**13**	7.07639991E-25	2.61456131E-17	6.53922793E-12	1.51834504E-12	5.30363306E-13
**14**	5.01026834E-24	6.25472556E-17	6.51506206E-12	1.52473863E-12	5.34711755E-13
**15**	2.74359011E-23	1.33567734E-16	6.50238555E-12	1.53369521E-12	5.40097131E-13
**16**	1.21946896E-22	2.60105943E-16	6.49964713E-12	1.544918E-12	5.46421682E-13
**17**	4.56506589E-22	4.69496414E-16	6.50556745E-12	1.55815504E-12	5.53601787E-13
**18**	1.4811684E-21	7.95506587E-16	6.51908227E-12	1.57319138E-12	5.61565572E-13
**19**	4.26055514E-21	1.27796076E-15	6.53929937E-12	1.58984274E-12	5.7025093E-13
**20**	1.10632348E-20	1.96207274E-15	6.56546544E-12	1.60795035E-12	5.79603895E-13
**21**	2.63147815E-20	2.89758086E-15	6.59694031E-12	1.62737673E-12	5.89577304E-13
**22**	5.8023902E-20	4.13777427E-15	6.63317669E-12	1.64800223E-12	6.00129699E-13
**23**	1.19780174E-19	5.73848639E-15	6.67370404E-12	1.66972222E-12	6.11224419E-13
**24**	2.33398692E-19	7.75711416E-15	6.71811564E-12	1.69244475E-12	6.2282885E-13
**25**	4.32254378E-19	1.02517049E-14	6.76605821E-12	1.71608863E-12	6.34913805E-13
**26**	7.65311251E-19	1.32801375E-14	6.81722335E-12	1.74058181E-12	6.4745301E-13
**27**	1.30181394E-18	1.68994125E-14	6.87134064E-12	1.76586007E-12	6.60422673E-13
**28**	2.13657407E-18	2.11650557E-14	6.9281719E-12	1.7918659E-12	6.73801133E-13
**29**	3.39575558E-18	2.61306328E-14	6.98750645E-12	1.81854757E-12	6.87568557E-13
**30**	5.24304218E-18	3.18473689E-14	7.0491572E-12	1.84585836E-12	7.01706698E-13
**31**	7.88605741E-18	3.83638635E-14	7.11295735E-12	1.87375588E-12	7.16198684E-13
**32**	1.15829027E-17	4.57258887E-14	7.17875762E-12	1.90220154E-12	7.31028846E-13
**33**	1.66486811E-17	5.39762603E-14	7.24642399E-12	1.93116008E-12	7.46182571E-13
**34**	2.34618808E-17	6.315477E-14	7.31583568E-12	1.96059915E-12	7.61646184E-13
**35**	3.24705052E-17	7.3298168E-14	7.38688355E-12	1.990489E-12	7.77406841E-13
**36**	4.41978497E-17	8.44401867E-14	7.45946865E-12	2.02080216E-12	7.93452443E-13
**37**	5.92478409E-17	9.66115972E-14	7.53350102E-12	2.05151323E-12	8.09771563E-13
**38**	7.8309871E-17	1.0984029E-13	7.60889867E-12	2.08259864E-12	8.2635338E-13
**39**	1.02163075E-16	1.24151376E-13	7.68558668E-12	2.11403646E-12	8.43187627E-13
**40**	1.31680013E-16	1.39567299E-13	7.76349644E-12	2.14580625E-12	8.60264544E-13
**41**	1.67829743E-16	1.56107958E-13	7.84256495E-12	2.17788894E-12	8.77574835E-13
**42**	2.11680254E-16	1.73790838E-13	7.92273428E-12	2.21026667E-12	8.95109634E-13
**43**	2.64400286E-16	1.92631139E-13	8.00395101E-12	2.24292271E-12	9.12860474E-13
**44**	3.27260531E-16	2.12641907E-13	8.08616582E-12	2.27584134E-12	9.30819256E-13
**45**	4.01634225E-16	2.33834165E-13	8.1693331E-12	2.30900779E-12	9.48978228E-13
**46**	4.88997182E-16	2.56217042E-13	8.25341057E-12	2.34240816E-12	9.67329958E-13
**47**	5.90927267E-16	2.79797896E-13	8.33835897E-12	2.37602933E-12	9.85867319E-13
**48**	7.09103367E-16	3.04582431E-13	8.42414182E-12	2.40985892E-12	1.00458347E-12
**49**	8.45303876E-16	3.30574819E-13	8.51072513E-12	2.44388523E-12	1.02347182E-12
**50**	1.00140474E-15	3.577778E-13	8.59807723E-12	2.47809719E-12	1.04252606E-12
**51**	1.17937711E-15	3.86192794E-13	8.68616851E-12	2.51248432E-12	1.06174009E-12

**52**	1.38128461E-15	4.15819999E-13	8.77497131E-12	2.54703666E-12	1.08110805E-12
**53**	1.6092803E-15	4.46658481E-13	8.86445969E-12	2.5817448E-12	1.10062429E-12
**54**	1.86560332E-15	4.78706265E-13	8.95460933E-12	2.61659975E-12	1.12028336E-12
**55**	2.15257534E-15	5.11960419E-13	9.04539742E-12	2.651593E-12	1.14008001E-12
**56**	2.47259672E-15	5.4641713E-13	9.13680248E-12	2.68671644E-12	1.16000915E-12
**57**	2.82814254E-15	5.8207178E-13	9.22880428E-12	2.72196234E-12	1.18006589E-12
**58**	3.22175844E-15	6.18919012E-13	9.32138378E-12	2.75732333E-12	1.20024549E-12
**59**	3.6560564E-15	6.56952795E-13	9.41452298E-12	2.79279239E-12	1.22054336E-12
**60**	4.13371031E-15	6.96166487E-13	9.50820488E-12	2.82836282E-12	1.24095509E-12
**61**	4.65745164E-15	7.36552889E-13	9.60241339E-12	2.86402821E-12	1.26147637E-12
**62**	5.23006491E-15	7.78104296E-13	9.69713327E-12	2.89978244E-12	1.28210306E-12
**63**	5.85438322E-15	8.20812549E-13	9.79235006E-12	2.93561965E-12	1.30283115E-12
**64**	6.53328377E-15	8.64669078E-13	9.88805004E-12	2.97153424E-12	1.32365674E-12
**65**	7.26968339E-15	9.09664946E-13	9.98422016E-12	3.00752085E-12	1.34457607E-12
**66**	8.06653409E-15	9.55790887E-13	1.0080848E-11	3.04357433E-12	1.36558547E-12
**67**	8.92681873E-15	1.00303735E-12	1.01779217E-11	3.07968975E-12	1.3866814E-12
**68**	9.85354663E-15	1.05139451E-12	1.027543E-11	3.1158624E-12	1.40786043E-12
**69**	1.08497494E-14	1.10085233E-12	1.03733622E-11	3.15208774E-12	1.42911923E-12
**70**	1.19184767E-14	1.15140058E-12	1.04717079E-11	3.18836142E-12	1.45045457E-12
**71**	1.30627922E-14	1.20302884E-12	1.05704573E-11	3.22467926E-12	1.4718633E-12
**72**	1.42857699E-14	1.25572656E-12	1.0669601E-11	3.26103725E-12	1.49334238E-12
**73**	1.55904895E-14	1.30948307E-12	1.07691299E-11	3.29743154E-12	1.51488886E-12
**74**	1.69800338E-14	1.36428759E-12	1.08690353E-11	3.33385842E-12	1.53649988E-12
**75**	1.8457484E-14	1.42012926E-12	1.0969309E-11	3.37031433E-12	1.55817264E-12
**76**	2.00259169E-14	1.47699717E-12	1.10699429E-11	3.40679585E-12	1.57990445E-12
**77**	2.16884012E-14	1.53488033E-12	1.11709294E-11	3.44329968E-12	1.60169267E-12
**78**	2.34479945E-14	1.59376776E-12	1.12722609E-11	3.47982266E-12	1.62353477E-12
**79**	2.53077399E-14	1.65364843E-12	1.13739304E-11	3.51636174E-12	1.64542827E-12
**80**	2.72706631E-14	1.71451133E-12	1.1475931E-11	3.55291398E-12	1.66737075E-12
**81**	2.93397698E-14	1.77634543E-12	1.15782559E-11	3.58947656E-12	1.6893599E-12
**82**	3.15180425E-14	1.83913973E-12	1.16808989E-11	3.62604676E-12	1.71139343E-12
**83**	3.38084383E-14	1.90288327E-12	1.17838536E-11	3.66262197E-12	1.73346915E-12
**84**	3.62138862E-14	1.9675651E-12	1.18871141E-11	3.69919966E-12	1.75558492E-12
**85**	3.87372847E-14	2.03317433E-12	1.19906744E-11	3.73577742E-12	1.77773866E-12
**86**	4.13814996E-14	2.09970011E-12	1.2094529E-11	3.77235289E-12	1.79992834E-12
**87**	4.41493621E-14	2.16713166E-12	1.21986724E-11	3.80892384E-12	1.82215201E-12
**88**	4.70436663E-14	2.23545825E-12	1.23030991E-11	3.8454881E-12	1.84440777E-12
**89**	5.00671677E-14	2.30466921E-12	1.24078042E-11	3.88204358E-12	1.86669375E-12
**90**	5.32225813E-14	2.37475396E-12	1.25127824E-11	3.91858827E-12	1.88900817E-12
**91**	5.65125796E-14	2.44570198E-12	1.26180289E-11	3.95512024E-12	1.91134927E-12
**92**	5.99397915E-14	2.51750284E-12	1.2723539E-11	3.99163762E-12	1.93371536E-12
**93**	6.35068006E-14	2.59014618E-12	1.28293079E-11	4.02813861E-12	1.95610478E-12
**94**	6.72161434E-14	2.66362174E-12	1.29353311E-11	4.06462149E-12	1.97851594E-12
**95**	7.10703088E-14	2.73791932E-12	1.30416042E-11	4.10108459E-12	2.00094729E-12
**96**	7.50717361E-14	2.81302882E-12	1.31481228E-11	4.13752631E-12	2.02339729E-12
**97**	7.92228143E-14	2.88894024E-12	1.32548826E-11	4.17394511E-12	2.0458645E-12
**98**	8.35258808E-14	2.96564366E-12	1.33618796E-11	4.21033948E-12	2.06834748E-12
**99**	8.79832207E-14	3.04312923E-12	1.34691095E-11	4.24670801E-12	2.09084484E-12
**100**	9.25970656E-14	3.12138723E-12	1.35765685E-11	4.28304932E-12	2.11335523E-12
**101**	9.7369593E-14	3.200408E-12	1.36842525E-11	4.31936206E-12	2.13587735E-12
**102**	1.02302925E-13	3.28018198E-12	1.37921578E-11	4.35564497E-12	2.15840991E-12

**103**	1.0739913E-13	3.3606997E-12	1.39002805E-11	4.39189682E-12	2.18095169E-12
**104**	1.12660216E-13	3.44195178E-12	1.40086168E-11	4.4281164E-12	2.20350148E-12
**105**	1.18088139E-13	3.52392891E-12	1.41171631E-11	4.46430259E-12	2.22605811E-12
**106**	1.23684793E-13	3.60662191E-12	1.42259158E-11	4.50045428E-12	2.24862044E-12
**107**	1.29452019E-13	3.69002164E-12	1.43348713E-11	4.53657041E-12	2.27118736E-12
**108**	1.35391596E-13	3.77411907E-12	1.4444026E-11	4.57264996E-12	2.29375781E-12
**109**	1.41505245E-13	3.85890526E-12	1.45533765E-11	4.60869195E-12	2.31633072E-12
**110**	1.4779463E-13	3.94437134E-12	1.46629192E-11	4.64469541E-12	2.3389051E-12
**111**	1.54261354E-13	4.03050851E-12	1.47726508E-11	4.68065945E-12	2.36147994E-12
**112**	1.60906962E-13	4.11730808E-12	1.48825679E-11	4.71658319E-12	2.38405427E-12
**113**	1.67732939E-13	4.20476142E-12	1.49926671E-11	4.75246576E-12	2.40662717E-12
**114**	1.74740709E-13	4.29285997E-12	1.5102945E-11	4.78830636E-12	2.42919772E-12
**115**	1.81931639E-13	4.38159525E-12	1.52133983E-11	4.82410419E-12	2.45176503E-12
**116**	1.89307034E-13	4.47095886E-12	1.53240239E-11	4.85985849E-12	2.47432823E-12
**117**	1.96868141E-13	4.56094247E-12	1.54348183E-11	4.89556853E-12	2.49688647E-12
**118**	2.04616146E-13	4.65153782E-12	1.55457783E-11	4.9312336E-12	2.51943893E-12
**119**	2.12552176E-13	4.74273669E-12	1.56569006E-11	4.96685301E-12	2.54198481E-12
**120**	2.206773E-13	4.83453097E-12	1.57681822E-11	5.0024261E-12	2.56452332E-12
**121**	2.28992524E-13	4.92691258E-12	1.58796196E-11	5.03795222E-12	2.5870537E-12
**122**	2.37498798E-13	5.01987352E-12	1.59912097E-11	5.07343077E-12	2.6095752E-12
**123**	2.46197013E-13	5.11340583E-12	1.61029493E-11	5.10886114E-12	2.63208709E-12
**124**	2.55087999E-13	5.20750164E-12	1.62148352E-11	5.14424274E-12	2.65458866E-12
**125**	2.64172529E-13	5.30215309E-12	1.63268642E-11	5.17957503E-12	2.67707922E-12
**126**	2.73451319E-13	5.39735242E-12	1.6439033E-11	5.21485744E-12	2.69955807E-12
**127**	2.82925025E-13	5.4930919E-12	1.65513385E-11	5.25008946E-12	2.72202456E-12
**128**	2.92594246E-13	5.58936384E-12	1.66637775E-11	5.28527057E-12	2.74447803E-12
**129**	3.02459526E-13	5.68616063E-12	1.67763467E-11	5.32040027E-12	2.76691784E-12
**130**	3.12521349E-13	5.78347468E-12	1.68890429E-11	5.35547807E-12	2.78934337E-12
**131**	3.22780145E-13	5.88129846E-12	1.7001863E-11	5.3905035E-12	2.811754E-12
**132**	3.33236287E-13	5.97962447E-12	1.71148036E-11	5.4254761E-12	2.83414913E-12
**133**	3.43890093E-13	6.07844527E-12	1.72278616E-11	5.46039542E-12	2.85652817E-12
**134**	3.54741826E-13	6.17775344E-12	1.73410337E-11	5.49526101E-12	2.87889054E-12
**135**	3.65791695E-13	6.27754162E-12	1.74543166E-11	5.53007246E-12	2.90123567E-12
**136**	3.77039854E-13	6.37780248E-12	1.75677071E-11	5.56482934E-12	2.923563E-12
**137**	3.88486404E-13	6.47852872E-12	1.76812019E-11	5.59953123E-12	2.94587197E-12
**138**	4.00131395E-13	6.57971309E-12	1.77947977E-11	5.63417773E-12	2.96816205E-12
**139**	4.11974821E-13	6.68134835E-12	1.79084912E-11	5.66876845E-12	2.9904327E-12
**140**	4.24016628E-13	6.78342732E-12	1.8022279E-11	5.703303E-12	3.01268339E-12
**141**	4.36256709E-13	6.88594284E-12	1.8136158E-11	5.73778098E-12	3.03491361E-12
**142**	4.48694907E-13	6.98888776E-12	1.82501246E-11	5.77220203E-12	3.05712284E-12
**143**	4.61331014E-13	7.092255E-12	1.83641755E-11	5.80656577E-12	3.07931059E-12
**144**	4.74164776E-13	7.19603748E-12	1.84783074E-11	5.84087182E-12	3.10147634E-12
**145**	4.87195888E-13	7.30022815E-12	1.85925169E-11	5.87511983E-12	3.12361961E-12
**146**	5.00423996E-13	7.40481999E-12	1.87068005E-11	5.90930944E-12	3.14573991E-12
**147**	5.13848703E-13	7.50980602E-12	1.88211548E-11	5.94344028E-12	3.16783676E-12
**148**	5.27469561E-13	7.61517926E-12	1.89355765E-11	5.977512E-12	3.18990969E-12
**149**	5.41286079E-13	7.72093277E-12	1.90500619E-11	6.01152425E-12	3.21195821E-12
**150**	5.55297721E-13	7.82705963E-12	1.91646077E-11	6.04547667E-12	3.23398188E-12
**151**	5.69503906E-13	7.93355294E-12	1.92792104E-11	6.07936893E-12	3.25598021E-12
**152**	5.83904009E-13	8.04040582E-12	1.93938664E-11	6.11320066E-12	3.27795276E-12
**153**	5.98497362E-13	8.14761143E-12	1.95085723E-11	6.14697154E-12	3.29989906E-12

**154**	6.13283258E-13	8.25516293E-12	1.96233244E-11	6.1806812E-12	3.32181867E-12
**155**	6.28260944E-13	8.36305351E-12	1.97381193E-11	6.21432931E-12	3.34371114E-12
**156**	6.43429629E-13	8.47127637E-12	1.98529533E-11	6.24791552E-12	3.36557602E-12
**157**	6.58788484E-13	8.57982476E-12	1.99678229E-11	6.2814395E-12	3.38741288E-12
**158**	6.74336637E-13	8.68869192E-12	2.00827245E-11	6.31490088E-12	3.40922127E-12
**159**	6.90073181E-13	8.79787112E-12	2.01976544E-11	6.34829934E-12	3.43100075E-12
**160**	7.0599717E-13	8.90735565E-12	2.03126091E-11	6.38163452E-12	3.45275089E-12
**161**	7.22107622E-13	9.01713882E-12	2.04275849E-11	6.41490608E-12	3.47447127E-12
**162**	7.38403518E-13	9.12721396E-12	2.05425781E-11	6.44811367E-12	3.49616144E-12
**163**	7.54883807E-13	9.23757441E-12	2.06575851E-11	6.48125695E-12	3.51782099E-12
**164**	7.715474E-13	9.34821355E-12	2.07726022E-11	6.51433557E-12	3.53944949E-12
**165**	7.88393176E-13	9.45912475E-12	2.08876257E-11	6.54734917E-12	3.56104651E-12
**166**	8.05419983E-13	9.57030143E-12	2.10026518E-11	6.58029742E-12	3.58261164E-12
**167**	8.22626634E-13	9.68173701E-12	2.1117677E-11	6.61317995E-12	3.60414444E-12
**168**	8.40011914E-13	9.79342494E-12	2.12326974E-11	6.64599641E-12	3.62564452E-12
**169**	8.57574575E-13	9.90535867E-12	2.13477093E-11	6.67874645E-12	3.64711144E-12
**170**	8.75313343E-13	1.00175317E-11	2.1462709E-11	6.71142971E-12	3.66854479E-12
**171**	8.93226911E-13	1.01299375E-11	2.15776928E-11	6.74404584E-12	3.68994416E-12
**172**	9.11313947E-13	1.02425697E-11	2.16926568E-11	6.77659447E-12	3.71130914E-12
**173**	9.29573091E-13	1.03554217E-11	2.18075973E-11	6.80907524E-12	3.73263932E-12
**174**	9.48002958E-13	1.04684871E-11	2.19225106E-11	6.84148779E-12	3.75393428E-12
**175**	9.66602136E-13	1.05817596E-11	2.20373928E-11	6.87383175E-12	3.77519361E-12
**176**	9.85369188E-13	1.06952327E-11	2.21522402E-11	6.90610676E-12	3.79641692E-12
**177**	1.00430265E-12	1.08089001E-11	2.2267049E-11	6.93831245E-12	3.81760379E-12
**178**	1.02340105E-12	1.09227553E-11	2.23818155E-11	6.97044846E-12	3.83875382E-12
**179**	1.04266287E-12	1.10367922E-11	2.24965358E-11	7.0025144E-12	3.85986661E-12
**180**	1.06208659E-12	1.11510043E-11	2.26112061E-11	7.03450991E-12	3.88094176E-12
**181**	1.08167066E-12	1.12653855E-11	2.27258227E-11	7.06643461E-12	3.90197885E-12
**182**	1.1014135E-12	1.13799294E-11	2.28403817E-11	7.09828812E-12	3.92297751E-12
**183**	1.12131354E-12	1.14946298E-11	2.29548794E-11	7.13007008E-12	3.94393731E-12
**184**	1.14136917E-12	1.16094805E-11	2.3069312E-11	7.1617801E-12	3.96485788E-12
**185**	1.16157875E-12	1.17244753E-11	2.31836757E-11	7.1934178E-12	3.98573881E-12
**186**	1.18194066E-12	1.18396082E-11	2.32979667E-11	7.2249828E-12	4.00657971E-12
**187**	1.20245322E-12	1.19548728E-11	2.34121812E-11	7.25647471E-12	4.02738019E-12
**188**	1.22311478E-12	1.20702632E-11	2.35263155E-11	7.28789316E-12	4.04813985E-12
**189**	1.24392363E-12	1.21857733E-11	2.36403658E-11	7.31923776E-12	4.0688583E-12
**190**	1.26487807E-12	1.2301397E-11	2.37543284E-11	7.35050813E-12	4.08953516E-12
**191**	1.28597639E-12	1.24171282E-11	2.38681994E-11	7.38170387E-12	4.11017004E-12
**192**	1.30721686E-12	1.25329611E-11	2.39819751E-11	7.41282461E-12	4.13076255E-12
**193**	1.32859774E-12	1.26488896E-11	2.40956518E-11	7.44386995E-12	4.15131232E-12
**194**	1.35011727E-12	1.27649077E-11	2.42092258E-11	7.47483951E-12	4.17181895E-12
**195**	1.37177368E-12	1.28810097E-11	2.43226933E-11	7.5057329E-12	4.19228206E-12
**196**	1.3935652E-12	1.29971896E-11	2.44360507E-11	7.53654973E-12	4.21270128E-12
**197**	1.41549005E-12	1.31134415E-11	2.45492942E-11	7.56728961E-12	4.23307624E-12
**198**	1.43754644E-12	1.32297597E-11	2.46624201E-11	7.59795216E-12	4.25340655E-12
**199**	1.45973255E-12	1.33461384E-11	2.47754249E-11	7.62853697E-12	4.27369183E-12
**200**	1.48204658E-12	1.34625718E-11	2.48883047E-11	7.65904368E-12	4.29393173E-12
**201**	1.50448671E-12	1.35790542E-11	2.5001056E-11	7.68947188E-12	4.31412587E-12
**202**	1.52705112E-12	1.36955799E-11	2.51136752E-11	7.71982119E-12	4.33427388E-12
**203**	1.54973798E-12	1.38121433E-11	2.52261585E-11	7.75009122E-12	4.3543754E-12
**204**	1.57254545E-12	1.39287388E-11	2.53385025E-11	7.78028159E-12	4.37443006E-12

**205**	1.59547169E-12	1.40453607E-11	2.54507036E-11	7.8103919E-12	4.3944375E-12
**206**	1.61851485E-12	1.41620036E-11	2.55627582E-11	7.84042178E-12	4.41439737E-12
**207**	1.6416731E-12	1.42786618E-11	2.56746627E-11	7.87037083E-12	4.4343093E-12
**208**	1.66494457E-12	1.43953301E-11	2.57864136E-11	7.90023868E-12	4.45417294E-12
**209**	1.68832741E-12	1.45120028E-11	2.58980074E-11	7.93002494E-12	4.47398795E-12
**210**	1.71181976E-12	1.46286746E-11	2.60094406E-11	7.95972923E-12	4.49375396E-12
**211**	1.73541977E-12	1.47453402E-11	2.61207098E-11	7.98935117E-12	4.51347064E-12
**212**	1.75912557E-12	1.48619942E-11	2.62318115E-11	8.01889039E-12	4.53313764E-12
**213**	1.78293531E-12	1.49786313E-11	2.63427423E-11	8.0483465E-12	4.55275462E-12
**214**	1.80684712E-12	1.50952463E-11	2.64534988E-11	8.07771912E-12	4.57232124E-12
**215**	1.83085914E-12	1.5211834E-11	2.65640776E-11	8.1070079E-12	4.59183716E-12
**216**	1.85496952E-12	1.53283891E-11	2.66744753E-11	8.13621245E-12	4.61130204E-12
**217**	1.87917639E-12	1.54449067E-11	2.67846887E-11	8.16533241E-12	4.63071557E-12
**218**	1.90347789E-12	1.55613814E-11	2.68947144E-11	8.19436741E-12	4.65007741E-12
**219**	1.92787218E-12	1.56778084E-11	2.70045491E-11	8.22331708E-12	4.66938723E-12
**220**	1.95235739E-12	1.57941826E-11	2.71141896E-11	8.25218106E-12	4.68864471E-12
**221**	1.97693168E-12	1.59104991E-11	2.72236327E-11	8.280959E-12	4.70784954E-12
**222**	2.0015932E-12	1.60267528E-11	2.73328751E-11	8.30965053E-12	4.7270014E-12
**223**	2.02634011E-12	1.61429389E-11	2.74419136E-11	8.33825529E-12	4.74609998E-12
**224**	2.05117056E-12	1.62590526E-11	2.75507452E-11	8.36677295E-12	4.76514496E-12
**225**	2.07608273E-12	1.63750891E-11	2.76593667E-11	8.39520314E-12	4.78413604E-12
**226**	2.10107477E-12	1.64910436E-11	2.77677749E-11	8.42354553E-12	4.80307292E-12
**227**	2.12614487E-12	1.66069113E-11	2.78759669E-11	8.45179976E-12	4.8219553E-12
**228**	2.15129121E-12	1.67226877E-11	2.79839396E-11	8.4799655E-12	4.84078288E-12
**229**	2.17651197E-12	1.6838368E-11	2.80916899E-11	8.50804241E-12	4.85955536E-12
**230**	2.20180534E-12	1.69539477E-11	2.81992149E-11	8.53603015E-12	4.87827247E-12
**231**	2.22716952E-12	1.70694222E-11	2.83065117E-11	8.5639284E-12	4.8969339E-12
**232**	2.25260272E-12	1.71847871E-11	2.84135773E-11	8.59173684E-12	4.91553939E-12
**233**	2.27810314E-12	1.73000378E-11	2.85204088E-11	8.61945512E-12	4.93408864E-12
**234**	2.30366902E-12	1.74151699E-11	2.86270034E-11	8.64708295E-12	4.95258139E-12
**235**	2.32929856E-12	1.75301791E-11	2.87333582E-11	8.67461999E-12	4.97101736E-12
**236**	2.35499001E-12	1.76450611E-11	2.88394704E-11	8.70206594E-12	4.98939629E-12
**237**	2.38074161E-12	1.77598115E-11	2.89453373E-11	8.72942049E-12	5.00771791E-12
**238**	2.40655161E-12	1.78744261E-11	2.90509561E-11	8.75668333E-12	5.02598195E-12
**239**	2.43241827E-12	1.79889008E-11	2.91563242E-11	8.78385416E-12	5.04418816E-12
**240**	2.45833986E-12	1.81032314E-11	2.92614387E-11	8.81093269E-12	5.06233629E-12
**241**	2.48431466E-12	1.82174137E-11	2.93662972E-11	8.83791862E-12	5.08042609E-12
**242**	2.51034094E-12	1.83314437E-11	2.94708969E-11	8.86481167E-12	5.09845731E-12
**243**	2.53641701E-12	1.84453174E-11	2.95752353E-11	8.89161155E-12	5.11642971E-12
**244**	2.56254118E-12	1.85590308E-11	2.96793099E-11	8.91831798E-12	5.13434304E-12
**245**	2.58871175E-12	1.867258E-11	2.97831181E-11	8.94493068E-12	5.15219708E-12
**246**	2.61492706E-12	1.87859612E-11	2.98866575E-11	8.97144939E-12	5.1699916E-12
**247**	2.64118543E-12	1.88991704E-11	2.99899255E-11	8.99787383E-12	5.18772636E-12
**248**	2.66748523E-12	1.90122039E-11	3.00929199E-11	9.02420375E-12	5.20540115E-12
**249**	2.69382479E-12	1.91250579E-11	3.01956381E-11	9.05043888E-12	5.22301574E-12
**250**	2.7202025E-12	1.92377288E-11	3.02980779E-11	9.07657897E-12	5.24056992E-12
**251**	2.74661673E-12	1.93502129E-11	3.04002369E-11	9.10262377E-12	5.25806348E-12
**252**	2.77306587E-12	1.94625065E-11	3.05021128E-11	9.12857305E-12	5.27549621E-12
**253**	2.79954832E-12	1.95746062E-11	3.06037035E-11	9.15442655E-12	5.2928679E-12
**254**	2.82606249E-12	1.96865083E-11	3.07050066E-11	9.18018404E-12	5.31017837E-12
**255**	2.85260681E-12	1.97982094E-11	3.080602E-11	9.2058453E-12	5.3274274E-12

**256**	2.87917972E-12	1.99097062E-11	3.09067415E-11	9.2314101E-12	5.34461482E-12
**257**	2.90577965E-12	2.00209951E-11	3.1007169E-11	9.25687821E-12	5.36174043E-12
**258**	2.93240508E-12	2.01320729E-11	3.11073004E-11	9.28224942E-12	5.37880405E-12
**259**	2.95905447E-12	2.02429362E-11	3.12071337E-11	9.30752352E-12	5.39580549E-12
**260**	2.9857263E-12	2.03535818E-11	3.13066669E-11	9.33270031E-12	5.41274459E-12
**261**	3.01241908E-12	2.04640066E-11	3.14058979E-11	9.35777958E-12	5.42962118E-12
**262**	3.0391313E-12	2.05742073E-11	3.15048248E-11	9.38276113E-12	5.44643507E-12
**263**	3.0658615E-12	2.06841807E-11	3.16034457E-11	9.40764478E-12	5.46318612E-12
**264**	3.09260819E-12	2.0793924E-11	3.17017586E-11	9.43243033E-12	5.47987416E-12
**265**	3.11936994E-12	2.09034339E-11	3.17997619E-11	9.45711761E-12	5.49649903E-12
**266**	3.14614529E-12	2.10127076E-11	3.18974535E-11	9.48170643E-12	5.51306058E-12
**267**	3.17293282E-12	2.1121742E-11	3.19948317E-11	9.50619663E-12	5.52955867E-12
**268**	3.1997311E-12	2.12305343E-11	3.20918949E-11	9.53058805E-12	5.54599315E-12
**269**	3.22653875E-12	2.13390817E-11	3.21886411E-11	9.55488051E-12	5.56236388E-12
**270**	3.25335436E-12	2.14473812E-11	3.22850688E-11	9.57907386E-12	5.57867073E-12
**271**	3.28017655E-12	2.15554302E-11	3.23811763E-11	9.60316794E-12	5.59491356E-12
**272**	3.30700397E-12	2.16632259E-11	3.2476962E-11	9.62716262E-12	5.61109224E-12
**273**	3.33383526E-12	2.17707656E-11	3.25724243E-11	9.65105775E-12	5.62720666E-12
**274**	3.36066908E-12	2.18780467E-11	3.26675615E-11	9.67485319E-12	5.64325668E-12
**275**	3.3875041E-12	2.19850666E-11	3.27623722E-11	9.69854882E-12	5.6592422E-12
**276**	3.41433901E-12	2.20918227E-11	3.28568549E-11	9.72214449E-12	5.6751631E-12
**277**	3.44117252E-12	2.21983125E-11	3.29510082E-11	9.7456401E-12	5.69101927E-12
**278**	3.46800333E-12	2.23045336E-11	3.30448304E-11	9.76903552E-12	5.70681061E-12
**279**	3.49483017E-12	2.24104835E-11	3.31383204E-11	9.79233064E-12	5.72253702E-12
**280**	3.52165178E-12	2.25161597E-11	3.32314767E-11	9.81552535E-12	5.73819839E-12
**281**	3.54846692E-12	2.262156E-11	3.33242979E-11	9.83861956E-12	5.75379464E-12
**282**	3.57527434E-12	2.27266821E-11	3.34167828E-11	9.86161317E-12	5.76932567E-12
**283**	3.60207283E-12	2.28315237E-11	3.350893E-11	9.88450607E-12	5.78479141E-12
**284**	3.62886118E-12	2.29360825E-11	3.36007384E-11	9.90729819E-12	5.80019176E-12
**285**	3.65563819E-12	2.30403563E-11	3.36922067E-11	9.92998945E-12	5.81552665E-12
**286**	3.68240268E-12	2.31443431E-11	3.37833336E-11	9.95257976E-12	5.83079601E-12
**287**	3.70915348E-12	2.32480407E-11	3.38741182E-11	9.97506905E-12	5.84599976E-12
**288**	3.73588945E-12	2.3351447E-11	3.39645592E-11	9.99745725E-12	5.86113784E-12
**289**	3.76260942E-12	2.345456E-11	3.40546555E-11	1.00197443E-11	5.87621017E-12
**290**	3.78931228E-12	2.35573778E-11	3.4144406E-11	1.00419301E-11	5.89121672E-12
**291**	3.81599691E-12	2.36598982E-11	3.42338098E-11	1.00640147E-11	5.9061574E-12
**292**	3.8426622E-12	2.37621196E-11	3.43228658E-11	1.0085998E-11	5.92103218E-12
**293**	3.86930707E-12	2.38640398E-11	3.44115731E-11	1.01078799E-11	5.935841E-12
**294**	3.89593043E-12	2.39656572E-11	3.44999306E-11	1.01296604E-11	5.95058382E-12
**295**	3.92253122E-12	2.406697E-11	3.45879375E-11	1.01513395E-11	5.9652606E-12
**296**	3.9491084E-12	2.41679762E-11	3.46755928E-11	1.01729172E-11	5.97987129E-12
**297**	3.97566091E-12	2.42686743E-11	3.47628957E-11	1.01943933E-11	5.99441586E-12
**298**	4.00218773E-12	2.43690626E-11	3.48498454E-11	1.0215768E-11	6.00889428E-12
**299**	4.02868786E-12	2.44691393E-11	3.49364411E-11	1.02370411E-11	6.02330652E-12
**300**	4.05516028E-12	2.45689029E-11	3.50226819E-11	1.02582127E-11	6.03765255E-12
**301**	4.08160401E-12	2.46683517E-11	3.51085671E-11	1.02792827E-11	6.05193236E-12
**302**	4.10801808E-12	2.47674843E-11	3.5194096E-11	1.03002512E-11	6.06614593E-12
**303**	4.13440152E-12	2.48662991E-11	3.52792679E-11	1.03211182E-11	6.08029323E-12
**304**	4.16075338E-12	2.49647947E-11	3.5364082E-11	1.03418836E-11	6.09437425E-12
**305**	4.18707272E-12	2.50629695E-11	3.54485378E-11	1.03625475E-11	6.108389E-12
**306**	4.21335862E-12	2.51608222E-11	3.55326347E-11	1.03831098E-11	6.12233746E-12

**307**	4.23961016E-12	2.52583515E-11	3.5616372E-11	1.04035707E-11	6.13621963E-12
**308**	4.26582644E-12	2.53555559E-11	3.56997491E-11	1.04239301E-11	6.15003551E-12
**309**	4.29200658E-12	2.54524342E-11	3.57827655E-11	1.04441881E-11	6.16378511E-12
**310**	4.31814969E-12	2.55489851E-11	3.58654208E-11	1.04643447E-11	6.17746843E-12
**311**	4.34425491E-12	2.56452073E-11	3.59477143E-11	1.04843999E-11	6.19108548E-12
**312**	4.37032139E-12	2.57410997E-11	3.60296456E-11	1.05043537E-11	6.20463627E-12
**313**	4.39634829E-12	2.5836661E-11	3.61112144E-11	1.05242063E-11	6.21812083E-12
**314**	4.42233477E-12	2.59318902E-11	3.61924201E-11	1.05439577E-11	6.23153916E-12
**315**	4.44828002E-12	2.6026786E-11	3.62732623E-11	1.05636078E-11	6.2448913E-12
**316**	4.47418324E-12	2.61213475E-11	3.63537407E-11	1.05831568E-11	6.25817727E-12
**317**	4.50004364E-12	2.62155736E-11	3.6433855E-11	1.06026048E-11	6.27139709E-12
**318**	4.52586042E-12	2.63094632E-11	3.65136047E-11	1.06219517E-11	6.2845508E-12
**319**	4.55163282E-12	2.64030154E-11	3.65929897E-11	1.06411977E-11	6.29763843E-12
**320**	4.57736008E-12	2.64962292E-11	3.66720096E-11	1.06603428E-11	6.31066001E-12
**321**	4.60304145E-12	2.65891037E-11	3.67506641E-11	1.06793872E-11	6.32361559E-12
**322**	4.62867619E-12	2.6681638E-11	3.68289531E-11	1.06983308E-11	6.33650521E-12
**323**	4.65426359E-12	2.67738311E-11	3.69068763E-11	1.07171738E-11	6.34932891E-12
**324**	4.67980292E-12	2.68656824E-11	3.69844334E-11	1.07359163E-11	6.36208673E-12
**325**	4.70529348E-12	2.69571909E-11	3.70616245E-11	1.07545583E-11	6.37477874E-12
**326**	4.73073459E-12	2.70483559E-11	3.71384492E-11	1.07731E-11	6.38740497E-12
**327**	4.75612555E-12	2.71391765E-11	3.72149075E-11	1.07915414E-11	6.3999655E-12
**328**	4.7814657E-12	2.72296522E-11	3.72909992E-11	1.08098827E-11	6.41246036E-12
**329**	4.80675439E-12	2.73197822E-11	3.73667244E-11	1.08281239E-11	6.42488964E-12
**330**	4.83199095E-12	2.74095658E-11	3.74420828E-11	1.08462651E-11	6.43725337E-12
**331**	4.85717476E-12	2.74990023E-11	3.75170745E-11	1.08643066E-11	6.44955165E-12
**332**	4.88230518E-12	2.75880912E-11	3.75916994E-11	1.08822483E-11	6.46178452E-12
**333**	4.9073816E-12	2.76768319E-11	3.76659576E-11	1.09000905E-11	6.47395206E-12
**334**	4.93240341E-12	2.77652237E-11	3.7739849E-11	1.09178332E-11	6.48605434E-12
**335**	4.95737002E-12	2.78532662E-11	3.78133736E-11	1.09354765E-11	6.49809144E-12
**336**	4.98228083E-12	2.79409588E-11	3.78865316E-11	1.09530206E-11	6.51006343E-12
**337**	5.00713528E-12	2.80283011E-11	3.7959323E-11	1.09704656E-11	6.5219704E-12
**338**	5.03193279E-12	2.81152925E-11	3.80317479E-11	1.09878117E-11	6.53381242E-12
**339**	5.05667281E-12	2.82019326E-11	3.81038064E-11	1.1005059E-11	6.54558958E-12
**340**	5.08135479E-12	2.82882211E-11	3.81754986E-11	1.10222076E-11	6.55730196E-12
**341**	5.1059782E-12	2.83741575E-11	3.82468246E-11	1.10392577E-11	6.56894966E-12
**342**	5.13054251E-12	2.84597414E-11	3.83177847E-11	1.10562094E-11	6.58053276E-12
**343**	5.15504721E-12	2.85449725E-11	3.83883789E-11	1.10730629E-11	6.59205135E-12
**344**	5.17949177E-12	2.86298505E-11	3.84586076E-11	1.10898183E-11	6.60350554E-12
**345**	5.20387571E-12	2.87143751E-11	3.85284709E-11	1.11064758E-11	6.61489541E-12
**346**	5.22819854E-12	2.87985459E-11	3.8597969E-11	1.11230355E-11	6.62622107E-12
**347**	5.25245978E-12	2.88823629E-11	3.86671021E-11	1.11394976E-11	6.63748261E-12
**348**	5.27665895E-12	2.89658256E-11	3.87358707E-11	1.11558623E-11	6.64868015E-12
**349**	5.3007956E-12	2.90489339E-11	3.88042748E-11	1.11721298E-11	6.65981377E-12
**350**	5.32486927E-12	2.91316876E-11	3.88723149E-11	1.11883001E-11	6.67088359E-12
**351**	5.34887952E-12	2.92140866E-11	3.89399911E-11	1.12043736E-11	6.68188972E-12
**352**	5.37282591E-12	2.92961306E-11	3.9007304E-11	1.12203502E-11	6.69283227E-12
**353**	5.39670803E-12	2.93778197E-11	3.90742537E-11	1.12362304E-11	6.70371135E-12
**354**	5.42052545E-12	2.94591535E-11	3.91408406E-11	1.12520141E-11	6.71452707E-12
**355**	5.44427776E-12	2.95401322E-11	3.92070652E-11	1.12677016E-11	6.72527954E-12
**356**	5.46796456E-12	2.96207555E-11	3.92729278E-11	1.12832931E-11	6.7359689E-12
**357**	5.49158547E-12	2.97010235E-11	3.93384287E-11	1.12987888E-11	6.74659524E-12

**358**	5.51514009E-12	2.97809361E-11	3.94035685E-11	1.13141888E-11	6.7571587E-12
**359**	5.53862806E-12	2.98604933E-11	3.94683475E-11	1.13294933E-11	6.7676594E-12
**360**	5.56204901E-12	2.99396952E-11	3.95327661E-11	1.13447027E-11	6.77809746E-12
**361**	5.58540257E-12	3.00185417E-11	3.95968249E-11	1.13598169E-11	6.788473E-12
**362**	5.60868841E-12	3.00970329E-11	3.96605243E-11	1.13748363E-11	6.79878616E-12
**363**	5.63190617E-12	3.01751689E-11	3.97238647E-11	1.13897611E-11	6.80903706E-12
**364**	5.65505552E-12	3.02529497E-11	3.97868468E-11	1.14045914E-11	6.81922583E-12
**365**	5.67813613E-12	3.03303755E-11	3.98494708E-11	1.14193274E-11	6.8293526E-12
**366**	5.7011477E-12	3.04074464E-11	3.99117375E-11	1.14339694E-11	6.83941751E-12
**367**	5.72408989E-12	3.04841624E-11	3.99736473E-11	1.14485176E-11	6.84942069E-12
**368**	5.74696241E-12	3.05605238E-11	4.00352008E-11	1.14629721E-11	6.85936228E-12
**369**	5.76976497E-12	3.06365308E-11	4.00963984E-11	1.14773333E-11	6.8692424E-12
**370**	5.79249727E-12	3.07121834E-11	4.01572409E-11	1.14916012E-11	6.87906121E-12
**371**	5.81515903E-12	3.0787482E-11	4.02177287E-11	1.15057762E-11	6.88881885E-12
**372**	5.83774998E-12	3.08624266E-11	4.02778624E-11	1.15198584E-11	6.89851544E-12
**373**	5.86026985E-12	3.09370177E-11	4.03376427E-11	1.15338481E-11	6.90815114E-12
**374**	5.88271838E-12	3.10112553E-11	4.03970701E-11	1.15477455E-11	6.91772609E-12
**375**	5.90509532E-12	3.10851398E-11	4.04561453E-11	1.15615508E-11	6.92724043E-12
**376**	5.92740041E-12	3.11586714E-11	4.05148689E-11	1.15752643E-11	6.93669432E-12
**377**	5.94963343E-12	3.12318505E-11	4.05732416E-11	1.15888861E-11	6.94608789E-12
**378**	5.97179413E-12	3.13046774E-11	4.0631264E-11	1.16024165E-11	6.95542129E-12
**379**	5.99388229E-12	3.13771523E-11	4.06889367E-11	1.16158558E-11	6.96469469E-12
**380**	6.01589769E-12	3.14492756E-11	4.07462604E-11	1.16292041E-11	6.97390822E-12
**381**	6.03784012E-12	3.15210477E-11	4.08032359E-11	1.16424618E-11	6.98306204E-12
**382**	6.05970938E-12	3.15924689E-11	4.08598638E-11	1.16556289E-11	6.99215629E-12
**383**	6.08150525E-12	3.16635395E-11	4.09161448E-11	1.16687059E-11	7.00119115E-12
**384**	6.10322755E-12	3.17342601E-11	4.09720796E-11	1.16816928E-11	7.01016675E-12
**385**	6.1248761E-12	3.1804631E-11	4.10276689E-11	1.169459E-11	7.01908327E-12
**386**	6.1464507E-12	3.18746525E-11	4.10829135E-11	1.17073977E-11	7.02794084E-12
**387**	6.16795118E-12	3.19443252E-11	4.11378142E-11	1.17201162E-11	7.03673964E-12
**388**	6.18937738E-12	3.20136495E-11	4.11923715E-11	1.17327456E-11	7.04547981E-12
**389**	6.21072912E-12	3.20826258E-11	4.12465864E-11	1.17452863E-11	7.05416153E-12
**390**	6.23200626E-12	3.21512546E-11	4.13004595E-11	1.17577384E-11	7.06278495E-12
**391**	6.25320865E-12	3.22195364E-11	4.13539916E-11	1.17701023E-11	7.07135023E-12
**392**	6.27433612E-12	3.22874716E-11	4.14071835E-11	1.17823781E-11	7.07985753E-12
**393**	6.29538855E-12	3.23550608E-11	4.1460036E-11	1.17945662E-11	7.08830703E-12
**394**	6.31636581E-12	3.24223045E-11	4.15125499E-11	1.18066668E-11	7.09669887E-12
**395**	6.33726775E-12	3.24892032E-11	4.1564726E-11	1.18186801E-11	7.10503324E-12
**396**	6.35809426E-12	3.25557574E-11	4.16165651E-11	1.18306063E-11	7.11331028E-12
**397**	6.37884522E-12	3.26219676E-11	4.16680679E-11	1.18424459E-11	7.12153017E-12
**398**	6.39952051E-12	3.26878345E-11	4.17192354E-11	1.18541989E-11	7.12969308E-12
**399**	6.42012004E-12	3.27533586E-11	4.17700683E-11	1.18658657E-11	7.13779918E-12
**400**	6.44064369E-12	3.28185405E-11	4.18205675E-11	1.18774465E-11	7.14584862E-12
**401**	6.46109137E-12	3.28833807E-11	4.18707339E-11	1.18889416E-11	7.15384159E-12
**402**	6.48146298E-12	3.29478799E-11	4.19205681E-11	1.19003512E-11	7.16177825E-12
**403**	6.50175845E-12	3.30120386E-11	4.19700712E-11	1.19116756E-11	7.16965877E-12
**404**	6.52197768E-12	3.30758575E-11	4.2019244E-11	1.1922915E-11	7.17748333E-12
**405**	6.5421206E-12	3.31393372E-11	4.20680874E-11	1.19340698E-11	7.18525209E-12
**406**	6.56218714E-12	3.32024783E-11	4.21166021E-11	1.19451402E-11	7.19296522E-12
**407**	6.58217722E-12	3.32652815E-11	4.21647891E-11	1.19561264E-11	7.20062291E-12
**408**	6.6020908E-12	3.33277473E-11	4.22126493E-11	1.19670288E-11	7.20822532E-12

**409**	6.6219278E-12	3.33898766E-11	4.22601836E-11	1.19778475E-11	7.21577262E-12
**410**	6.64168817E-12	3.34516699E-11	4.23073928E-11	1.19885829E-11	7.223265E-12
**411**	6.66137186E-12	3.35131279E-11	4.23542779E-11	1.19992352E-11	7.23070262E-12
**412**	6.68097884E-12	3.35742513E-11	4.24008397E-11	1.20098047E-11	7.23808567E-12
**413**	6.70050905E-12	3.36350408E-11	4.24470791E-11	1.20202916E-11	7.24541431E-12
**414**	6.71996247E-12	3.36954971E-11	4.24929972E-11	1.20306963E-11	7.25268873E-12
**415**	6.73933905E-12	3.37556209E-11	4.25385947E-11	1.2041019E-11	7.25990909E-12
**416**	6.75863877E-12	3.38154129E-11	4.25838726E-11	1.20512599E-11	7.26707559E-12
**417**	6.77786162E-12	3.38748738E-11	4.26288319E-11	1.20614194E-11	7.27418839E-12
**418**	6.79700756E-12	3.39340044E-11	4.26734734E-11	1.20714977E-11	7.28124767E-12
**419**	6.81607658E-12	3.39928054E-11	4.27177981E-11	1.2081495E-11	7.28825362E-12
**420**	6.83506868E-12	3.40512775E-11	4.27618069E-11	1.20914118E-11	7.2952064E-12
**421**	6.85398383E-12	3.41094215E-11	4.28055009E-11	1.21012481E-11	7.3021062E-12
**422**	6.87282205E-12	3.41672382E-11	4.28488808E-11	1.21110044E-11	7.3089532E-12
**423**	6.89158333E-12	3.42247283E-11	4.28919477E-11	1.21206808E-11	7.31574758E-12
**424**	6.91026767E-12	3.42818925E-11	4.29347026E-11	1.21302777E-11	7.32248952E-12
**425**	6.92887508E-12	3.43387317E-11	4.29771463E-11	1.21397953E-11	7.3291792E-12
**426**	6.94740558E-12	3.43952466E-11	4.30192798E-11	1.21492339E-11	7.33581679E-12
**427**	6.96585917E-12	3.44514381E-11	4.30611042E-11	1.21585938E-11	7.34240248E-12
**428**	6.98423588E-12	3.45073068E-11	4.31026203E-11	1.21678752E-11	7.34893645E-12
**429**	7.00253572E-12	3.45628537E-11	4.31438292E-11	1.21770785E-11	7.35541888E-12
**430**	7.02075873E-12	3.46180795E-11	4.31847318E-11	1.21862039E-11	7.36184996E-12
**431**	7.03890492E-12	3.4672985E-11	4.3225329E-11	1.21952517E-11	7.36822985E-12
**432**	7.05697434E-12	3.4727571E-11	4.32656219E-11	1.22042221E-11	7.37455875E-12
**433**	7.07496702E-12	3.47818384E-11	4.33056115E-11	1.22131155E-11	7.38083684E-12
**434**	7.092883E-12	3.4835788E-11	4.33452987E-11	1.22219321E-11	7.3870643E-12
**435**	7.11072232E-12	3.48894206E-11	4.33846845E-11	1.22306722E-11	7.39324131E-12
**436**	7.12848502E-12	3.4942737E-11	4.34237699E-11	1.22393361E-11	7.39936805E-12
**437**	7.14617115E-12	3.49957381E-11	4.34625559E-11	1.2247924E-11	7.40544471E-12
**438**	7.16378076E-12	3.50484248E-11	4.35010434E-11	1.22564362E-11	7.41147147E-12
**439**	7.18131391E-12	3.51007978E-11	4.35392336E-11	1.22648731E-11	7.41744851E-12
**440**	7.19877066E-12	3.5152858E-11	4.35771273E-11	1.22732348E-11	7.42337601E-12
**441**	7.21615106E-12	3.52046064E-11	4.36147256E-11	1.22815218E-11	7.42925415E-12
**442**	7.23345518E-12	3.52560437E-11	4.36520294E-11	1.22897341E-11	7.43508313E-12
**443**	7.25068308E-12	3.53071708E-11	4.36890398E-11	1.22978722E-11	7.44086312E-12
**444**	7.26783484E-12	3.53579886E-11	4.37257578E-11	1.23059363E-11	7.4465943E-12
**445**	7.28491052E-12	3.54084979E-11	4.37621844E-11	1.23139267E-11	7.45227686E-12
**446**	7.30191019E-12	3.54586996E-11	4.37983205E-11	1.23218436E-11	7.45791098E-12
**447**	7.31883394E-12	3.55085947E-11	4.38341673E-11	1.23296874E-11	7.46349685E-12
**448**	7.33568184E-12	3.5558184E-11	4.38697256E-11	1.23374583E-11	7.46903464E-12
**449**	7.35245398E-12	3.56074683E-11	4.39049966E-11	1.23451566E-11	7.47452454E-12
**450**	7.36915043E-12	3.56564486E-11	4.39399812E-11	1.23527826E-11	7.47996673E-12
**451**	7.3857713E-12	3.57051257E-11	4.39746804E-11	1.23603365E-11	7.4853614E-12
**452**	7.40231666E-12	3.57535007E-11	4.40090952E-11	1.23678187E-11	7.49070872E-12
**453**	7.4187866E-12	3.58015742E-11	4.40432268E-11	1.23752293E-11	7.49600889E-12
**454**	7.43518123E-12	3.58493473E-11	4.4077076E-11	1.23825688E-11	7.50126208E-12
**455**	7.45150064E-12	3.58968209E-11	4.41106439E-11	1.23898374E-11	7.50646847E-12
**456**	7.46774492E-12	3.59439958E-11	4.41439315E-11	1.23970352E-11	7.51162825E-12
**457**	7.48391418E-12	3.59908731E-11	4.41769398E-11	1.24041628E-11	7.51674161E-12
**458**	7.50000853E-12	3.60374535E-11	4.42096699E-11	1.24112202E-11	7.52180871E-12
**459**	7.51602806E-12	3.6083738E-11	4.42421228E-11	1.24182078E-11	7.52682975E-12

**460**	7.53197288E-12	3.61297275E-11	4.42742994E-11	1.24251258E-11	7.53180491E-12
**461**	7.54784311E-12	3.61754229E-11	4.43062009E-11	1.24319746E-11	7.53673437E-12
**462**	7.56363886E-12	3.62208252E-11	4.43378282E-11	1.24387545E-11	7.54161831E-12
**463**	7.57936023E-12	3.62659353E-11	4.43691823E-11	1.24454656E-11	7.54645691E-12
**464**	7.59500735E-12	3.63107541E-11	4.44002643E-11	1.24521082E-11	7.55125036E-12
**465**	7.61058033E-12	3.63552826E-11	4.44310751E-11	1.24586828E-11	7.55599883E-12
**466**	7.6260793E-12	3.63995216E-11	4.44616159E-11	1.24651894E-11	7.56070251E-12
**467**	7.64150437E-12	3.6443472E-11	4.44918876E-11	1.24716284E-11	7.56536157E-12
**468**	7.65685567E-12	3.64871349E-11	4.45218913E-11	1.24780002E-11	7.56997621E-12
**469**	7.67213332E-12	3.65305112E-11	4.45516279E-11	1.24843048E-11	7.57454659E-12
**470**	7.68733745E-12	3.65736018E-11	4.45810985E-11	1.24905427E-11	7.5790729E-12
**471**	7.7024682E-12	3.66164076E-11	4.46103041E-11	1.24967141E-11	7.58355533E-12
**472**	7.71752568E-12	3.66589295E-11	4.46392457E-11	1.25028192E-11	7.58799404E-12
**473**	7.73251003E-12	3.67011686E-11	4.46679244E-11	1.25088584E-11	7.59238922E-12
**474**	7.74742139E-12	3.67431257E-11	4.46963411E-11	1.25148319E-11	7.59674105E-12
**475**	7.7622599E-12	3.67848018E-11	4.4724497E-11	1.252074E-11	7.60104971E-12
**476**	7.77702568E-12	3.68261978E-11	4.47523929E-11	1.2526583E-11	7.60531538E-12
**477**	7.79171887E-12	3.68673147E-11	4.47800299E-11	1.25323611E-11	7.60953824E-12
**478**	7.80633963E-12	3.69081534E-11	4.48074091E-11	1.25380746E-11	7.61371846E-12
**479**	7.82088809E-12	3.69487149E-11	4.48345314E-11	1.25437238E-11	7.61785622E-12
**480**	7.83536439E-12	3.69890001E-11	4.48613979E-11	1.2549309E-11	7.6219517E-12
**481**	7.84976867E-12	3.70290099E-11	4.48880096E-11	1.25548303E-11	7.62600509E-12
**482**	7.86410109E-12	3.70687454E-11	4.49143675E-11	1.25602882E-11	7.63001655E-12
**483**	7.87836179E-12	3.71082074E-11	4.49404726E-11	1.25656829E-11	7.63398626E-12
**484**	7.89255093E-12	3.71473969E-11	4.49663259E-11	1.25710145E-11	7.63791441E-12
**485**	7.90666864E-12	3.71863148E-11	4.49919284E-11	1.25762835E-11	7.64180116E-12
**486**	7.92071508E-12	3.72249622E-11	4.50172812E-11	1.258149E-11	7.6456467E-12
**487**	7.93469041E-12	3.72633399E-11	4.50423852E-11	1.25866344E-11	7.64945119E-12
**488**	7.94859478E-12	3.73014489E-11	4.50672415E-11	1.25917169E-11	7.65321483E-12
**489**	7.96242835E-12	3.73392902E-11	4.5091851E-11	1.25967378E-11	7.65693777E-12
**490**	7.97619126E-12	3.73768646E-11	4.51162148E-11	1.26016973E-11	7.6606202E-12
**491**	7.98988368E-12	3.74141732E-11	4.51403339E-11	1.26065957E-11	7.66426229E-12
**492**	8.00350577E-12	3.74512169E-11	4.51642093E-11	1.26114333E-11	7.66786421E-12
**493**	8.01705769E-12	3.74879967E-11	4.51878419E-11	1.26162103E-11	7.67142615E-12
**494**	8.0305396E-12	3.75245134E-11	4.52112329E-11	1.26209271E-11	7.67494827E-12
**495**	8.04395166E-12	3.75607682E-11	4.52343831E-11	1.26255838E-11	7.67843074E-12
**496**	8.05729403E-12	3.75967618E-11	4.52572936E-11	1.26301807E-11	7.68187374E-12
**497**	8.07056688E-12	3.76324952E-11	4.52799654E-11	1.26347182E-11	7.68527745E-12
**498**	8.08377038E-12	3.76679695E-11	4.53023994E-11	1.26391963E-11	7.68864203E-12
**499**	8.09690468E-12	3.77031856E-11	4.53245967E-11	1.26436156E-11	7.69196766E-12
**500**	8.10996997E-12	3.77381443E-11	4.53465583E-11	1.2647976E-11	7.6952545E-12

Inelastic collisions' Rate Coefficients: Pseudo-Singlet State					
Initial rotational state: j=4					
T(K)	k (cm³mol⁻¹s⁻¹)				
	**Final State: j'=5**	**Final State: j'=3**	**Final State: j'=2**	**Final State: j'=1**	**Final State: j'=0**
**5**	1.85141302E-29	3.88890151E-12	6.6412598E-13	1.6065001E-13	2.769043E-14
**6**	1.27302468E-26	3.81447389E-12	6.5070698E-13	1.5761442E-13	2.701634E-14
**7**	1.35614982E-24	3.75624898E-12	6.4073191E-13	1.5542702E-13	2.654127E-14
**8**	4.50982186E-23	3.71401809E-12	6.3402464E-13	1.5405045E-13	2.623922E-14
**9**	6.90757104E-22	3.68693273E-12	6.3035933E-13	1.5343306E-13	2.609051E-14
**10**	6.15317964E-21	3.67357695E-12	6.294475E-13	1.5350718E-13	2.607734E-14
**11**	3.6973748E-20	3.67234742E-12	6.309884E-13	1.5420185E-13	2.61839E-14
**12**	1.65428656E-19	3.68169132E-12	6.3470141E-13	1.5545091E-13	2.639662E-14
**13**	5.90098272E-19	3.70021099E-12	6.403391E-13	1.5719618E-13	2.67042E-14
**14**	1.76199674E-18	3.72669304E-12	6.4768924E-13	1.5938798E-13	2.709731E-14
**15**	4.56413354E-18	3.76010175E-12	6.5657198E-13	1.619844E-13	2.756829E-14
**16**	1.05340768E-17	3.7995587E-12	6.6683519E-13	1.6495019E-13	2.811084E-14
**17**	2.21098486E-17	3.84431916E-12	6.7834982E-13	1.6825563E-13	2.871971E-14
**18**	4.28756526E-17	3.89375004E-12	6.910058E-13	1.7187549E-13	2.939053E-14
**19**	7.77852408E-17	3.947311E-12	7.0470863E-13	1.7578825E-13	3.011961E-14
**20**	1.33345467E-16	4.00453889E-12	7.1937673E-13	1.7997546E-13	3.090384E-14
**21**	2.17748807E-16	4.06503523E-12	7.3493935E-13	1.8442128E-13	3.174056E-14
**22**	3.40949838E-16	4.1284561E-12	7.513349E-13	1.8911202E-13	3.262748E-14
**23**	5.14685921E-16	4.19450396E-12	7.6850965E-13	1.9403591E-13	3.356267E-14
**24**	7.52446321E-16	4.26292096E-12	7.8641661E-13	1.991828E-13	3.454448E-14
**25**	1.06939655E-15	4.33348332E-12	8.0501466E-13	2.0454394E-13	3.557148E-14
**26**	1.48226611E-15	4.40599665E-12	8.2426775E-13	2.1011182E-13	3.664248E-14
**27**	2.0092083E-15	4.48029188E-12	8.4414421E-13	2.1587999E-13	3.775645E-14
**28**	2.66964039E-15	4.55622183E-12	8.6461618E-13	2.2184289E-13	3.89125E-14
**29**	3.48407179E-15	4.63365819E-12	8.8565905E-13	2.2799578E-13	4.01099E-14
**30**	4.47392681E-15	4.71248891E-12	9.0725105E-13	2.3433455E-13	4.134799E-14
**31**	5.6613676E-15	4.79261594E-12	9.2937276E-13	2.4085565E-13	4.262622E-14
**32**	7.06912143E-15	4.87395325E-12	9.5200681E-13	2.47556E-13	4.39441E-14
**33**	8.72031589E-15	4.95642514E-12	9.7513757E-13	2.5443292E-13	4.530121E-14
**34**	1.063832E-14	5.03996474E-12	9.9875083E-13	2.6148401E-13	4.669714E-14
**35**	1.284662E-14	5.12451277E-12	1.02283361E-12	2.6870717E-13	4.813156E-14
**36**	1.536866E-14	5.21001641E-12	1.04737395E-12	2.7610048E-13	4.960413E-14
**37**	1.822773E-14	5.29642838E-12	1.07236072E-12	2.8366218E-13	5.111454E-14
**38**	2.14469E-14	5.38370615E-12	1.09778351E-12	2.9139064E-13	5.26625E-14
**39**	2.504888E-14	5.47181124E-12	1.12363248E-12	2.9928433E-13	5.42477E-14
**40**	2.905595E-14	5.56070866E-12	1.14989829E-12	3.0734179E-13	5.586987E-14
**41**	3.348991E-14	5.6503664E-12	1.176572E-12	3.1556162E-13	5.752871E-14
**42**	3.837203E-14	5.74075504E-12	1.203645E-12	3.2394245E-13	5.922393E-14
**43**	4.372295E-14	5.83184739E-12	1.23110896E-12	3.3248293E-13	6.095524E-14
**44**	4.95627E-14	5.92361819E-12	1.25895581E-12	3.4118174E-13	6.272233E-14
**45**	5.591062E-14	6.01604388E-12	1.28717765E-12	3.5003756E-13	6.452491E-14
**46**	6.278538E-14	6.10910236E-12	1.31576677E-12	3.5904906E-13	6.636267E-14
**47**	7.020493E-14	6.20277286E-12	1.34471563E-12	3.6821492E-13	6.823528E-14
**48**	7.818648E-14	6.29703577E-12	1.3740168E-12	3.7753383E-13	7.014243E-14
**49**	8.674654E-14	6.39187251E-12	1.40366299E-12	3.8700444E-13	7.208379E-14
**50**	9.590085E-14	6.48726544E-12	1.43364701E-12	3.9662542E-13	7.405902E-14
**51**	1.0566444E-13	6.58319777E-12	1.46396179E-12	4.0639542E-13	7.606778E-14

**52**	1.1605161E-13	6.67965346E-12	1.49460034E-12	4.1631307E-13	7.810973E-14
**53**	1.2707591E-13	6.7766172E-12	1.5255558E-12	4.2637703E-13	8.018452E-14
**54**	1.387502E-13	6.87407434E-12	1.55682137E-12	4.3658591E-13	8.22918E-14
**55**	1.5108662E-13	6.97201081E-12	1.58839037E-12	4.4693833E-13	8.443121E-14
**56**	1.6409662E-13	7.07041314E-12	1.62025619E-12	4.5743292E-13	8.66024E-14
**57**	1.7779098E-13	7.16926836E-12	1.65241233E-12	4.6806827E-13	8.880499E-14
**58**	1.921798E-13	7.26856402E-12	1.68485236E-12	4.7884301E-13	9.103862E-14
**59**	2.0727254E-13	7.36828814E-12	1.71756995E-12	4.8975573E-13	9.330293E-14
**60**	2.2307803E-13	7.46842919E-12	1.75055887E-12	5.0080504E-13	9.559755E-14
**61**	2.3960449E-13	7.56897605E-12	1.78381297E-12	5.1198953E-13	9.792211E-14
**62**	2.5685955E-13	7.66991801E-12	1.81732619E-12	5.2330781E-13	1.0027623E-13
**63**	2.7485023E-13	7.77124477E-12	1.85109258E-12	5.3475848E-13	1.0265956E-13
**64**	2.9358304E-13	7.87294637E-12	1.88510626E-12	5.4634014E-13	1.050717E-13
**65**	3.130639E-13	7.97501322E-12	1.91936144E-12	5.5805139E-13	1.0751231E-13
**66**	3.3329825E-13	8.07743605E-12	1.95385244E-12	5.6989085E-13	1.0998099E-13
**67**	3.54291E-13	8.18020594E-12	1.98857365E-12	5.8185713E-13	1.1247739E-13
**68**	3.7604658E-13	8.28331424E-12	2.02351958E-12	5.9394883E-13	1.1500112E-13
**69**	3.9856895E-13	8.38675264E-12	2.05868479E-12	6.0616458E-13	1.1755183E-13
**70**	4.2186162E-13	8.49051309E-12	2.09406398E-12	6.1850302E-13	1.2012914E-13
**71**	4.4592768E-13	8.59458781E-12	2.12965189E-12	6.3096275E-13	1.2273268E-13
**72**	4.7076978E-13	8.69896928E-12	2.16544338E-12	6.4354244E-13	1.2536209E-13
**73**	4.9639019E-13	8.80365024E-12	2.20143338E-12	6.562407E-13	1.2801701E-13
**74**	5.2279077E-13	8.90862367E-12	2.23761693E-12	6.6905621E-13	1.3069706E-13
**75**	5.4997304E-13	9.01388277E-12	2.27398915E-12	6.8198761E-13	1.3340189E-13
**76**	5.7793815E-13	9.11942096E-12	2.31054522E-12	6.9503358E-13	1.3613114E-13
**77**	6.0668692E-13	9.22523186E-12	2.34728043E-12	7.0819277E-13	1.3888444E-13
**78**	6.3621983E-13	9.33130931E-12	2.38419016E-12	7.2146387E-13	1.4166145E-13
**79**	6.6653706E-13	9.43764733E-12	2.42126985E-12	7.3484558E-13	1.4446179E-13
**80**	6.9763849E-13	9.54424011E-12	2.45851504E-12	7.4833658E-13	1.4728514E-13
**81**	7.2952371E-13	9.65108203E-12	2.49592134E-12	7.6193558E-13	1.5013112E-13
**82**	7.6219205E-13	9.75816763E-12	2.53348444E-12	7.756413E-13	1.5299939E-13
**83**	7.9564256E-13	9.8654916E-12	2.57120013E-12	7.8945245E-13	1.5588961E-13
**84**	8.2987405E-13	9.97304878E-12	2.60906423E-12	8.0336776E-13	1.5880142E-13
**85**	8.648851E-13	1.00808342E-11	2.64707269E-12	8.1738597E-13	1.6173449E-13
**86**	9.0067404E-13	1.01888429E-11	2.6852215E-12	8.3150584E-13	1.6468848E-13
**87**	9.3723898E-13	1.02970701E-11	2.72350674E-12	8.4572611E-13	1.6766304E-13
**88**	9.7457786E-13	1.04055113E-11	2.76192454E-12	8.6004554E-13	1.7065784E-13
**89**	1.01268836E-12	1.0514162E-11	2.80047113E-12	8.7446292E-13	1.7367254E-13
**90**	1.05156801E-12	1.06230176E-11	2.83914279E-12	8.8897702E-13	1.7670681E-13
**91**	1.09121415E-12	1.07320738E-11	2.87793587E-12	9.0358663E-13	1.7976033E-13
**92**	1.13162393E-12	1.08413266E-11	2.9168468E-12	9.1829054E-13	1.8283276E-13
**93**	1.17279434E-12	1.09507717E-11	2.95587206E-12	9.3308757E-13	1.8592378E-13
**94**	1.2147222E-12	1.1060405E-11	2.99500819E-12	9.4797652E-13	1.8903306E-13
**95**	1.25740418E-12	1.11702227E-11	3.03425182E-12	9.6295621E-13	1.9216028E-13
**96**	1.30083681E-12	1.12802208E-11	3.07359962E-12	9.7802548E-13	1.9530513E-13
**97**	1.34501647E-12	1.13903955E-11	3.11304831E-12	9.9318316E-13	1.9846729E-13
**98**	1.38993939E-12	1.1500743E-11	3.15259469E-12	1.0084281E-12	2.0164643E-13
**99**	1.43560168E-12	1.16112596E-11	3.19223561E-12	1.02375913E-12	2.0484225E-13
**100**	1.48199933E-12	1.17219416E-11	3.23196797E-12	1.03917513E-12	2.0805444E-13
**101**	1.52912819E-12	1.18327855E-11	3.27178873E-12	1.05467495E-12	2.1128269E-13
**102**	1.57698402E-12	1.19437876E-11	3.3116949E-12	1.07025746E-12	2.1452669E-13

**103**	1.62556242E-12	1.20549444E-11	3.35168355E-12	1.08592154E-12	2.1778614E-13
**104**	1.67485894E-12	1.21662525E-11	3.39175179E-12	1.10166608E-12	2.2106073E-13
**105**	1.72486897E-12	1.22777083E-11	3.43189678E-12	1.11748995E-12	2.2435016E-13
**106**	1.77558784E-12	1.23893084E-11	3.47211573E-12	1.13339206E-12	2.2765414E-13
**107**	1.82701076E-12	1.25010495E-11	3.51240589E-12	1.14937131E-12	2.3097237E-13
**108**	1.87913284E-12	1.26129281E-11	3.55276457E-12	1.16542658E-12	2.3430456E-13
**109**	1.93194912E-12	1.27249408E-11	3.5931891E-12	1.18155681E-12	2.376504E-13
**110**	1.98545453E-12	1.28370843E-11	3.63367688E-12	1.19776089E-12	2.4100961E-13
**111**	2.03964393E-12	1.29493552E-11	3.67422533E-12	1.21403774E-12	2.4438191E-13
**112**	2.09451209E-12	1.30617501E-11	3.71483192E-12	1.23038629E-12	2.47767E-13
**113**	2.15005369E-12	1.31742659E-11	3.75549414E-12	1.24680547E-12	2.5116459E-13
**114**	2.20626335E-12	1.3286899E-11	3.79620955E-12	1.26329419E-12	2.5457441E-13
**115**	2.26313559E-12	1.33996461E-11	3.83697572E-12	1.27985139E-12	2.5799617E-13
**116**	2.32066489E-12	1.3512504E-11	3.87779026E-12	1.29647601E-12	2.614296E-13
**117**	2.37884562E-12	1.36254693E-11	3.91865082E-12	1.31316698E-12	2.648744E-13
**118**	2.43767211E-12	1.37385386E-11	3.95955508E-12	1.32992325E-12	2.6833032E-13
**119**	2.49713861E-12	1.38517086E-11	4.00050075E-12	1.34674376E-12	2.7179706E-13
**120**	2.5572393E-12	1.39649758E-11	4.04148556E-12	1.36362745E-12	2.7527436E-13
**121**	2.6179683E-12	1.4078337E-11	4.08250729E-12	1.38057326E-12	2.7876195E-13
**122**	2.67931968E-12	1.41917887E-11	4.12356374E-12	1.39758016E-12	2.8225955E-13
**123**	2.74128742E-12	1.43053275E-11	4.16465274E-12	1.41464709E-12	2.857669E-13
**124**	2.80386548E-12	1.44189499E-11	4.20577212E-12	1.43177299E-12	2.8928373E-13
**125**	2.86704773E-12	1.45326526E-11	4.24691978E-12	1.44895683E-12	2.9280977E-13
**126**	2.930828E-12	1.46464321E-11	4.28809361E-12	1.46619756E-12	2.9634477E-13
**127**	2.99520007E-12	1.47602848E-11	4.32929153E-12	1.48349414E-12	2.9988844E-13
**128**	3.06015765E-12	1.48742073E-11	4.37051151E-12	1.50084552E-12	3.0344055E-13
**129**	3.12569442E-12	1.4988196E-11	4.4117515E-12	1.51825065E-12	3.0700081E-13
**130**	3.191804E-12	1.51022474E-11	4.4530095E-12	1.5357085E-12	3.1056899E-13
**131**	3.25847995E-12	1.52163579E-11	4.49428353E-12	1.55321803E-12	3.1414481E-13
**132**	3.32571582E-12	1.53305239E-11	4.53557161E-12	1.57077819E-12	3.1772803E-13
**133**	3.39350508E-12	1.54447418E-11	4.57687179E-12	1.58838794E-12	3.2131839E-13
**134**	3.46184117E-12	1.5559008E-11	4.61818215E-12	1.60604624E-12	3.2491564E-13
**135**	3.5307175E-12	1.56733187E-11	4.65950077E-12	1.62375206E-12	3.2851952E-13
**136**	3.60012741E-12	1.57876702E-11	4.70082575E-12	1.64150435E-12	3.3212979E-13
**137**	3.67006423E-12	1.5902059E-11	4.74215523E-12	1.65930206E-12	3.357462E-13
**138**	3.74052125E-12	1.60164811E-11	4.78348733E-12	1.67714418E-12	3.393685E-13
**139**	3.81149171E-12	1.61309328E-11	4.8248202E-12	1.69502964E-12	3.4299645E-13
**140**	3.88296882E-12	1.62454104E-11	4.86615203E-12	1.71295742E-12	3.466298E-13
**141**	3.95494576E-12	1.635991E-11	4.90748098E-12	1.73092647E-12	3.5026831E-13
**142**	4.02741569E-12	1.64744278E-11	4.94880527E-12	1.74893575E-12	3.5391173E-13
**143**	4.10037173E-12	1.65889598E-11	4.99012308E-12	1.76698423E-12	3.5755983E-13
**144**	4.17380696E-12	1.67035022E-11	5.03143266E-12	1.78507086E-12	3.6121237E-13
**145**	4.24771445E-12	1.68180512E-11	5.07273224E-12	1.80319461E-12	3.6486911E-13
**146**	4.32208725E-12	1.69326026E-11	5.11402007E-12	1.82135443E-12	3.6852981E-13
**147**	4.39691837E-12	1.70471527E-11	5.15529441E-12	1.83954929E-12	3.7219424E-13
**148**	4.4722008E-12	1.71616973E-11	5.19655353E-12	1.85777815E-12	3.7586217E-13
**149**	4.54792752E-12	1.72762326E-11	5.23779573E-12	1.87603997E-12	3.7953337E-13
**150**	4.6240915E-12	1.73907544E-11	5.27901929E-12	1.89433371E-12	3.832076E-13
**151**	4.70068566E-12	1.75052588E-11	5.32022254E-12	1.91265834E-12	3.8688463E-13
**152**	4.77770292E-12	1.76197416E-11	5.36140378E-12	1.93101281E-12	3.9056425E-13
**153**	4.85513621E-12	1.77341988E-11	5.40256136E-12	1.9493961E-12	3.9424621E-13

**154**	4.93297841E-12	1.78486264E-11	5.44369361E-12	1.96780717E-12	3.979303E-13
**155**	5.01122241E-12	1.79630201E-11	5.48479889E-12	1.98624497E-12	4.016163E-13
**156**	5.08986109E-12	1.80773759E-11	5.52587556E-12	2.00470849E-12	4.0530398E-13
**157**	5.16888731E-12	1.81916897E-11	5.56692199E-12	2.02319668E-12	4.0899312E-13
**158**	5.24829393E-12	1.83059572E-11	5.60793657E-12	2.04170852E-12	4.126835E-13
**159**	5.32807381E-12	1.84201744E-11	5.6489177E-12	2.06024298E-12	4.163749E-13
**160**	5.4082198E-12	1.85343371E-11	5.68986378E-12	2.07879902E-12	4.2006712E-13
**161**	5.48872475E-12	1.8648441E-11	5.73077322E-12	2.09737562E-12	4.2375993E-13
**162**	5.56958152E-12	1.8762482E-11	5.77164445E-12	2.11597176E-12	4.2745312E-13
**163**	5.65078294E-12	1.88764559E-11	5.81247591E-12	2.1345864E-12	4.3114649E-13
**164**	5.73232188E-12	1.89903585E-11	5.85326604E-12	2.15321854E-12	4.3483981E-13
**165**	5.8141912E-12	1.91041856E-11	5.89401329E-12	2.17186715E-12	4.3853288E-13
**166**	5.89638374E-12	1.92179329E-11	5.93471614E-12	2.19053121E-12	4.4222551E-13
**167**	5.9788924E-12	1.93315963E-11	5.97537305E-12	2.20920971E-12	4.4591747E-13
**168**	6.06171003E-12	1.94451716E-11	6.01598252E-12	2.22790164E-12	4.4960856E-13
**169**	6.14482953E-12	1.95586545E-11	6.05654304E-12	2.24660599E-12	4.532986E-13
**170**	6.2282438E-12	1.96720408E-11	6.09705311E-12	2.26532174E-12	4.5698736E-13
**171**	6.31194575E-12	1.97853263E-11	6.13751125E-12	2.28404791E-12	4.6067466E-13
**172**	6.39592831E-12	1.98985068E-11	6.177916E-12	2.30278348E-12	4.643603E-13
**173**	6.48018441E-12	2.00115781E-11	6.21826588E-12	2.32152746E-12	4.6804409E-13
**174**	6.56470701E-12	2.0124536E-11	6.25855944E-12	2.34027886E-12	4.7172582E-13
**175**	6.64948908E-12	2.02373763E-11	6.29879525E-12	2.35903669E-12	4.7540531E-13
**176**	6.73452363E-12	2.03500947E-11	6.33897187E-12	2.37779995E-12	4.7908236E-13
**177**	6.81980367E-12	2.04626872E-11	6.37908787E-12	2.39656768E-12	4.8275679E-13
**178**	6.90532223E-12	2.05751496E-11	6.41914186E-12	2.41533888E-12	4.8642841E-13
**179**	6.99107237E-12	2.06874777E-11	6.45913243E-12	2.43411259E-12	4.9009704E-13
**180**	7.07704717E-12	2.07996673E-11	6.49905818E-12	2.45288784E-12	4.9376248E-13
**181**	7.16323976E-12	2.09117144E-11	6.53891776E-12	2.47166365E-12	4.9742457E-13
**182**	7.24964326E-12	2.10236148E-11	6.57870978E-12	2.49043908E-12	5.0108311E-13
**183**	7.33625084E-12	2.11353643E-11	6.6184329E-12	2.50921316E-12	5.0473794E-13
**184**	7.42305568E-12	2.1246959E-11	6.65808577E-12	2.52798495E-12	5.0838887E-13
**185**	7.51005103E-12	2.13583948E-11	6.69766705E-12	2.54675349E-12	5.1203572E-13
**186**	7.59723012E-12	2.14696675E-11	6.73717544E-12	2.56551785E-12	5.1567834E-13
**187**	7.68458625E-12	2.15807732E-11	6.77660961E-12	2.58427709E-12	5.1931653E-13
**188**	7.77211274E-12	2.16917079E-11	6.81596828E-12	2.60303029E-12	5.2295014E-13
**189**	7.85980294E-12	2.18024675E-11	6.85525015E-12	2.62177651E-12	5.26579E-13
**190**	7.94765024E-12	2.19130481E-11	6.89445395E-12	2.64051484E-12	5.3020294E-13
**191**	8.03564807E-12	2.20234457E-11	6.93357843E-12	2.65924437E-12	5.3382179E-13
**192**	8.1237899E-12	2.21336565E-11	6.97262233E-12	2.67796419E-12	5.374354E-13
**193**	8.21206922E-12	2.22436765E-11	7.01158441E-12	2.6966734E-12	5.410436E-13
**194**	8.30047958E-12	2.23535018E-11	7.05046346E-12	2.71537111E-12	5.4464623E-13
**195**	8.38901455E-12	2.24631287E-11	7.08925825E-12	2.73405643E-12	5.4824315E-13
**196**	8.47766777E-12	2.25725532E-11	7.12796759E-12	2.75272847E-12	5.5183418E-13
**197**	8.56643288E-12	2.26817716E-11	7.16659029E-12	2.77138637E-12	5.5541919E-13
**198**	8.65530361E-12	2.27907801E-11	7.20512518E-12	2.79002925E-12	5.5899801E-13
**199**	8.7442737E-12	2.2899575E-11	7.2435711E-12	2.80865625E-12	5.6257051E-13
**200**	8.83333693E-12	2.30081526E-11	7.28192689E-12	2.82726652E-12	5.6613652E-13
**201**	8.92248716E-12	2.31165092E-11	7.32019142E-12	2.84585921E-12	5.6969591E-13
**202**	9.01171826E-12	2.32246412E-11	7.35836357E-12	2.86443349E-12	5.7324853E-13
**203**	9.10102416E-12	2.33325449E-11	7.39644223E-12	2.88298851E-12	5.7679424E-13
**204**	9.19039883E-12	2.34402168E-11	7.43442629E-12	2.90152345E-12	5.803329E-13

**205**	9.27983631E-12	2.35476533E-11	7.47231469E-12	2.9200375E-12	5.8386437E-13
**206**	9.36933065E-12	2.3654851E-11	7.51010634E-12	2.93852983E-12	5.8738852E-13
**207**	9.45887599E-12	2.37618063E-11	7.5478002E-12	2.95699966E-12	5.909052E-13
**208**	9.54846648E-12	2.38685158E-11	7.58539521E-12	2.97544618E-12	5.9441429E-13
**209**	9.63809635E-12	2.3974976E-11	7.62289036E-12	2.99386861E-12	5.9791566E-13
**210**	9.72775986E-12	2.40811837E-11	7.66028461E-12	3.01226615E-12	6.0140918E-13
**211**	9.81745133E-12	2.41871355E-11	7.69757698E-12	3.03063805E-12	6.0489471E-13
**212**	9.90716513E-12	2.42928281E-11	7.73476646E-12	3.04898354E-12	6.0837214E-13
**213**	9.99689568E-12	2.43982582E-11	7.77185209E-12	3.06730185E-12	6.1184134E-13
**214**	1.00866375E-11	2.45034226E-11	7.80883291E-12	3.08559224E-12	6.1530219E-13
**215**	1.0176385E-11	2.46083181E-11	7.84570797E-12	3.10385397E-12	6.1875458E-13
**216**	1.02661328E-11	2.47129416E-11	7.88247632E-12	3.12208631E-12	6.2219837E-13
**217**	1.03558756E-11	2.48172899E-11	7.91913706E-12	3.14028853E-12	6.2563346E-13
**218**	1.0445608E-11	2.492136E-11	7.95568928E-12	3.15845991E-12	6.2905973E-13
**219**	1.05353248E-11	2.50251489E-11	7.99213208E-12	3.17659975E-12	6.3247707E-13
**220**	1.06250207E-11	2.51286536E-11	8.02846459E-12	3.19470734E-12	6.3588537E-13
**221**	1.07146906E-11	2.5231871E-11	8.06468594E-12	3.212782E-12	6.3928452E-13
**222**	1.08043294E-11	2.53347984E-11	8.10079529E-12	3.23082303E-12	6.4267442E-13
**223**	1.08939321E-11	2.54374328E-11	8.13679178E-12	3.24882977E-12	6.4605495E-13
**224**	1.09834936E-11	2.55397714E-11	8.17267461E-12	3.26680155E-12	6.4942602E-13
**225**	1.1073009E-11	2.56418114E-11	8.20844296E-12	3.28473771E-12	6.5278752E-13
**226**	1.11624735E-11	2.57435501E-11	8.24409604E-12	3.30263759E-12	6.5613935E-13
**227**	1.12518821E-11	2.58449847E-11	8.27963307E-12	3.32050056E-12	6.5948142E-13
**228**	1.13412301E-11	2.59461127E-11	8.31505328E-12	3.33832599E-12	6.6281363E-13
**229**	1.14305129E-11	2.60469312E-11	8.35035591E-12	3.35611325E-12	6.6613589E-13
**230**	1.15197257E-11	2.61474379E-11	8.38554023E-12	3.37386171E-12	6.6944809E-13
**231**	1.16088638E-11	2.62476301E-11	8.42060552E-12	3.39157079E-12	6.7275016E-13
**232**	1.16979229E-11	2.63475053E-11	8.45555105E-12	3.40923986E-12	6.7604201E-13
**233**	1.17868983E-11	2.64470612E-11	8.49037614E-12	3.42686836E-12	6.7932353E-13
**234**	1.18757857E-11	2.65462951E-11	8.52508009E-12	3.44445568E-12	6.8259466E-13
**235**	1.19645805E-11	2.66452049E-11	8.55966225E-12	3.46200126E-12	6.858553E-13
**236**	1.20532786E-11	2.67437881E-11	8.59412195E-12	3.47950454E-12	6.8910538E-13
**237**	1.21418756E-11	2.68420424E-11	8.62845854E-12	3.49696495E-12	6.9234481E-13
**238**	1.22303672E-11	2.69399657E-11	8.66267141E-12	3.51438194E-12	6.9557351E-13
**239**	1.23187494E-11	2.70375557E-11	8.69675993E-12	3.53175498E-12	6.9879141E-13
**240**	1.2407018E-11	2.71348102E-11	8.7307235E-12	3.54908354E-12	7.0199843E-13
**241**	1.2495169E-11	2.7231727E-11	8.76456153E-12	3.56636709E-12	7.051945E-13
**242**	1.25831983E-11	2.73283042E-11	8.79827346E-12	3.58360511E-12	7.0837954E-13
**243**	1.2671102E-11	2.74245397E-11	8.83185871E-12	3.60079711E-12	7.1155348E-13
**244**	1.27588762E-11	2.75204315E-11	8.86531673E-12	3.61794258E-12	7.1471627E-13
**245**	1.28465171E-11	2.76159775E-11	8.898647E-12	3.63504102E-12	7.1786781E-13
**246**	1.29340208E-11	2.7711176E-11	8.93184899E-12	3.65209197E-12	7.2100807E-13
**247**	1.30213837E-11	2.78060249E-11	8.96492219E-12	3.66909495E-12	7.2413695E-13
**248**	1.3108602E-11	2.79005225E-11	8.9978661E-12	3.68604948E-12	7.2725442E-13
**249**	1.31956722E-11	2.79946671E-11	9.03068025E-12	3.70295513E-12	7.3036039E-13
**250**	1.32825907E-11	2.80884567E-11	9.06336416E-12	3.71981142E-12	7.3345482E-13
**251**	1.33693539E-11	2.81818897E-11	9.09591737E-12	3.73661794E-12	7.3653765E-13
**252**	1.34559583E-11	2.82749645E-11	9.12833945E-12	3.75337423E-12	7.3960882E-13
**253**	1.35424007E-11	2.83676794E-11	9.16062995E-12	3.77007989E-12	7.4266827E-13
**254**	1.36286775E-11	2.84600327E-11	9.19278847E-12	3.78673449E-12	7.4571595E-13
**255**	1.37147856E-11	2.8552023E-11	9.22481458E-12	3.80333762E-12	7.4875181E-13

**256**	1.38007216E-11	2.86436487E-11	9.25670791E-12	3.81988888E-12	7.5177579E-13
**257**	1.38864823E-11	2.87349084E-11	9.28846806E-12	3.83638789E-12	7.5478786E-13
**258**	1.39720647E-11	2.88258006E-11	9.32009467E-12	3.85283425E-12	7.5778796E-13
**259**	1.40574655E-11	2.89163239E-11	9.35158739E-12	3.8692276E-12	7.6077604E-13
**260**	1.41426818E-11	2.90064769E-11	9.38294585E-12	3.88556755E-12	7.6375206E-13
**261**	1.42277105E-11	2.90962584E-11	9.41416974E-12	3.90185375E-12	7.6671598E-13
**262**	1.43125486E-11	2.9185667E-11	9.44525873E-12	3.91808585E-12	7.6966775E-13
**263**	1.43971934E-11	2.92747015E-11	9.47621252E-12	3.9342635E-12	7.7260734E-13
**264**	1.44816419E-11	2.93633607E-11	9.5070308E-12	3.95038636E-12	7.755347E-13
**265**	1.45658914E-11	2.94516434E-11	9.53771328E-12	3.9664541E-12	7.784498E-13
**266**	1.4649939E-11	2.95395485E-11	9.5682597E-12	3.9824664E-12	7.8135259E-13
**267**	1.47337821E-11	2.96270748E-11	9.59866979E-12	3.99842294E-12	7.8424305E-13
**268**	1.48174181E-11	2.97142214E-11	9.6289433E-12	4.01432341E-12	7.8712114E-13
**269**	1.49008443E-11	2.98009872E-11	9.65907999E-12	4.03016751E-12	7.8998683E-13
**270**	1.49840581E-11	2.98873712E-11	9.68907963E-12	4.04595495E-12	7.9284008E-13
**271**	1.50670571E-11	2.99733724E-11	9.718942E-12	4.06168544E-12	7.9568087E-13
**272**	1.51498388E-11	3.005899E-11	9.74866689E-12	4.0773587E-12	7.9850916E-13
**273**	1.52324007E-11	3.0144223E-11	9.77825411E-12	4.09297446E-12	8.0132493E-13
**274**	1.53147406E-11	3.02290707E-11	9.80770348E-12	4.10853246E-12	8.0412816E-13
**275**	1.5396856E-11	3.03135321E-11	9.83701481E-12	4.12403242E-12	8.0691881E-13
**276**	1.54787447E-11	3.03976065E-11	9.86618794E-12	4.13947411E-12	8.0969686E-13
**277**	1.55604045E-11	3.04812932E-11	9.89522272E-12	4.15485727E-12	8.1246229E-13
**278**	1.56418331E-11	3.05645914E-11	9.92411901E-12	4.17018168E-12	8.1521508E-13
**279**	1.57230285E-11	3.06475006E-11	9.95287666E-12	4.18544709E-12	8.179552E-13
**280**	1.58039885E-11	3.07300199E-11	9.98149557E-12	4.20065329E-12	8.2068264E-13
**281**	1.58847111E-11	3.08121488E-11	1.00099756E-11	4.21580005E-12	8.2339739E-13
**282**	1.59651942E-11	3.08938868E-11	1.00383167E-11	4.23088717E-12	8.2609941E-13
**283**	1.60454359E-11	3.09752333E-11	1.00665187E-11	4.24591443E-12	8.2878869E-13
**284**	1.61254342E-11	3.10561877E-11	1.00945815E-11	4.26088165E-12	8.3146523E-13
**285**	1.62051873E-11	3.11367496E-11	1.01225052E-11	4.27578862E-12	8.34129E-13
**286**	1.62846933E-11	3.12169185E-11	1.01502895E-11	4.29063517E-12	8.3678E-13
**287**	1.63639504E-11	3.1296694E-11	1.01779345E-11	4.30542111E-12	8.394182E-13
**288**	1.64429568E-11	3.13760757E-11	1.02054402E-11	4.32014627E-12	8.4204361E-13
**289**	1.65217109E-11	3.14550632E-11	1.02328064E-11	4.33481048E-12	8.446562E-13
**290**	1.66002109E-11	3.15336563E-11	1.02600331E-11	4.34941358E-12	8.4725598E-13
**291**	1.66784553E-11	3.16118545E-11	1.02871204E-11	4.36395542E-12	8.4984293E-13
**292**	1.67564423E-11	3.16896576E-11	1.03140681E-11	4.37843584E-12	8.5241704E-13
**293**	1.68341704E-11	3.17670654E-11	1.03408764E-11	4.39285471E-12	8.5497831E-13
**294**	1.69116381E-11	3.18440777E-11	1.03675453E-11	4.40721188E-12	8.5752674E-13
**295**	1.6988844E-11	3.19206941E-11	1.03940746E-11	4.42150722E-12	8.6006232E-13
**296**	1.70657865E-11	3.19969147E-11	1.04204645E-11	4.43574061E-12	8.6258505E-13
**297**	1.71424643E-11	3.20727392E-11	1.0446715E-11	4.44991192E-12	8.6509492E-13
**298**	1.72188759E-11	3.21481676E-11	1.04728262E-11	4.46402105E-12	8.6759193E-13
**299**	1.729502E-11	3.22231997E-11	1.0498798E-11	4.47806788E-12	8.7007609E-13
**300**	1.73708954E-11	3.22978355E-11	1.05246305E-11	4.4920523E-12	8.7254739E-13
**301**	1.74465008E-11	3.2372075E-11	1.05503239E-11	4.50597422E-12	8.7500583E-13
**302**	1.75218349E-11	3.24459181E-11	1.05758781E-11	4.51983354E-12	8.7745142E-13
**303**	1.75968965E-11	3.25193649E-11	1.06012932E-11	4.53363018E-12	8.7988416E-13
**304**	1.76716844E-11	3.25924154E-11	1.06265694E-11	4.54736406E-12	8.8230405E-13
**305**	1.77461976E-11	3.26650696E-11	1.06517068E-11	4.56103508E-12	8.847111E-13
**306**	1.7820435E-11	3.27373278E-11	1.06767054E-11	4.57464319E-12	8.8710531E-13

**307**	1.78943954E-11	3.28091899E-11	1.07015653E-11	4.58818831E-12	8.8948669E-13
**308**	1.79680779E-11	3.28806562E-11	1.07262866E-11	4.60167038E-12	8.9185524E-13
**309**	1.80414815E-11	3.29517268E-11	1.07508696E-11	4.61508934E-12	8.9421098E-13
**310**	1.81146051E-11	3.30224019E-11	1.07753142E-11	4.62844514E-12	8.9655391E-13
**311**	1.81874479E-11	3.30926816E-11	1.07996207E-11	4.64173772E-12	8.9888403E-13
**312**	1.82600089E-11	3.31625663E-11	1.08237892E-11	4.65496706E-12	9.0120137E-13
**313**	1.83322874E-11	3.32320561E-11	1.08478199E-11	4.66813309E-12	9.0350593E-13
**314**	1.84042824E-11	3.33011514E-11	1.08717128E-11	4.6812358E-12	9.0579772E-13
**315**	1.84759931E-11	3.33698524E-11	1.08954682E-11	4.69427514E-12	9.0807675E-13
**316**	1.85474188E-11	3.34381595E-11	1.09190862E-11	4.7072511E-12	9.1034304E-13
**317**	1.86185587E-11	3.3506073E-11	1.0942567E-11	4.72016365E-12	9.125966E-13
**318**	1.86894121E-11	3.35735932E-11	1.09659108E-11	4.73301277E-12	9.1483744E-13
**319**	1.87599783E-11	3.36407205E-11	1.09891178E-11	4.74579845E-12	9.1706558E-13
**320**	1.88302567E-11	3.37074554E-11	1.10121881E-11	4.75852069E-12	9.1928103E-13
**321**	1.89002465E-11	3.37737983E-11	1.1035122E-11	4.77117947E-12	9.214838E-13
**322**	1.89699473E-11	3.38397495E-11	1.10579197E-11	4.78377479E-12	9.2367393E-13
**323**	1.90393583E-11	3.39053096E-11	1.10805813E-11	4.79630667E-12	9.2585141E-13
**324**	1.91084792E-11	3.39704789E-11	1.11031072E-11	4.8087751E-12	9.2801627E-13
**325**	1.91773093E-11	3.40352581E-11	1.11254974E-11	4.82118009E-12	9.3016854E-13
**326**	1.92458481E-11	3.40996476E-11	1.11477523E-11	4.83352167E-12	9.3230821E-13
**327**	1.93140952E-11	3.41636479E-11	1.11698721E-11	4.84579985E-12	9.3443532E-13
**328**	1.93820501E-11	3.42272596E-11	1.1191857E-11	4.85801465E-12	9.3654989E-13
**329**	1.94497124E-11	3.42904833E-11	1.12137072E-11	4.8701661E-12	9.3865193E-13
**330**	1.95170817E-11	3.43533195E-11	1.12354231E-11	4.88225423E-12	9.4074147E-13
**331**	1.95841576E-11	3.44157688E-11	1.12570048E-11	4.89427907E-12	9.4281853E-13
**332**	1.96509399E-11	3.44778318E-11	1.12784526E-11	4.90624065E-12	9.4488312E-13
**333**	1.9717428E-11	3.45395092E-11	1.12997668E-11	4.91813903E-12	9.4693528E-13
**334**	1.97836219E-11	3.46008016E-11	1.13209476E-11	4.92997424E-12	9.4897502E-13
**335**	1.98495211E-11	3.46617097E-11	1.13419953E-11	4.94174632E-12	9.5100237E-13
**336**	1.99151254E-11	3.47222341E-11	1.13629102E-11	4.95345534E-12	9.5301735E-13
**337**	1.99804346E-11	3.47823756E-11	1.13836926E-11	4.96510134E-12	9.5501999E-13
**338**	2.00454485E-11	3.48421347E-11	1.14043427E-11	4.97668437E-12	9.5701031E-13
**339**	2.01101668E-11	3.49015123E-11	1.14248608E-11	4.98820451E-12	9.5898833E-13
**340**	2.01745895E-11	3.49605091E-11	1.14452473E-11	4.9996618E-12	9.6095409E-13
**341**	2.02387164E-11	3.50191258E-11	1.14655024E-11	5.01105633E-12	9.629076E-13
**342**	2.03025473E-11	3.50773632E-11	1.14856264E-11	5.02238815E-12	9.648489E-13
**343**	2.03660821E-11	3.51352221E-11	1.15056197E-11	5.03365734E-12	9.6677801E-13
**344**	2.04293208E-11	3.51927031E-11	1.15254825E-11	5.04486397E-12	9.6869496E-13
**345**	2.04922633E-11	3.52498073E-11	1.15452151E-11	5.05600812E-12	9.7059978E-13
**346**	2.05549096E-11	3.53065352E-11	1.15648179E-11	5.06708988E-12	9.7249249E-13
**347**	2.06172596E-11	3.53628879E-11	1.15842912E-11	5.07810931E-12	9.7437312E-13
**348**	2.06793133E-11	3.5418866E-11	1.16036352E-11	5.08906652E-12	9.762417E-13
**349**	2.07410707E-11	3.54744705E-11	1.16228504E-11	5.09996159E-12	9.7809826E-13
**350**	2.08025319E-11	3.55297022E-11	1.16419371E-11	5.1107946E-12	9.7994284E-13
**351**	2.08636969E-11	3.5584562E-11	1.16608955E-11	5.12156566E-12	9.8177545E-13
**352**	2.09245659E-11	3.56390507E-11	1.16797261E-11	5.13227486E-12	9.8359614E-13
**353**	2.09851387E-11	3.56931693E-11	1.16984291E-11	5.14292229E-12	9.8540492E-13
**354**	2.10454157E-11	3.57469186E-11	1.17170049E-11	5.15350806E-12	9.8720184E-13
**355**	2.11053969E-11	3.58002996E-11	1.17354539E-11	5.16403228E-12	9.8898692E-13
**356**	2.11650823E-11	3.58533132E-11	1.17537764E-11	5.17449504E-12	9.9076019E-13
**357**	2.12244723E-11	3.59059603E-11	1.17719727E-11	5.18489646E-12	9.9252169E-13

**358**	2.12835669E-11	3.59582418E-11	1.17900432E-11	5.19523664E-12	9.9427145E-13
**359**	2.13423664E-11	3.60101588E-11	1.18079883E-11	5.2055157E-12	9.960095E-13
**360**	2.14008709E-11	3.60617121E-11	1.18258083E-11	5.21573376E-12	9.9773587E-13
**361**	2.14590807E-11	3.61129028E-11	1.18435036E-11	5.22589092E-12	9.9945059E-13
**362**	2.15169959E-11	3.61637318E-11	1.18610745E-11	5.23598732E-12	1.00115371E-12
**363**	2.15746169E-11	3.62142001E-11	1.18785214E-11	5.24602306E-12	1.00284524E-12
**364**	2.16319439E-11	3.62643087E-11	1.18958446E-11	5.25599828E-12	1.00452524E-12
**365**	2.16889772E-11	3.63140586E-11	1.19130447E-11	5.26591309E-12	1.00619372E-12
**366**	2.17457171E-11	3.63634508E-11	1.19301218E-11	5.27576763E-12	1.00785073E-12
**367**	2.18021638E-11	3.64124864E-11	1.19470764E-11	5.28556202E-12	1.00949629E-12
**368**	2.18583177E-11	3.64611663E-11	1.19639089E-11	5.29529639E-12	1.01113045E-12
**369**	2.19141792E-11	3.65094916E-11	1.19806196E-11	5.30497088E-12	1.01275323E-12
**370**	2.19697485E-11	3.65574634E-11	1.1997209E-11	5.31458562E-12	1.01436468E-12
**371**	2.20250261E-11	3.66050826E-11	1.20136773E-11	5.32414074E-12	1.01596483E-12
**372**	2.20800122E-11	3.66523504E-11	1.20300251E-11	5.33363638E-12	1.01755371E-12
**373**	2.21347073E-11	3.66992678E-11	1.20462527E-11	5.34307269E-12	1.01913136E-12
**374**	2.21891119E-11	3.67458359E-11	1.20623604E-11	5.3524498E-12	1.02069781E-12
**375**	2.22432262E-11	3.67920558E-11	1.20783487E-11	5.36176786E-12	1.0222531E-12
**376**	2.22970507E-11	3.68379284E-11	1.20942179E-11	5.371027E-12	1.02379727E-12
**377**	2.23505859E-11	3.6883455E-11	1.21099685E-11	5.38022738E-12	1.02533035E-12
**378**	2.24038321E-11	3.69286366E-11	1.21256008E-11	5.38936914E-12	1.02685238E-12
**379**	2.24567899E-11	3.69734743E-11	1.21411153E-11	5.39845243E-12	1.0283634E-12
**380**	2.25094597E-11	3.70179693E-11	1.21565123E-11	5.4074774E-12	1.02986344E-12
**381**	2.2561842E-11	3.70621226E-11	1.21717922E-11	5.41644419E-12	1.03135255E-12
**382**	2.26139373E-11	3.71059353E-11	1.21869555E-11	5.42535298E-12	1.03283074E-12
**383**	2.2665746E-11	3.71494085E-11	1.22020025E-11	5.43420389E-12	1.03429808E-12
**384**	2.27172687E-11	3.71925435E-11	1.22169337E-11	5.4429971E-12	1.03575458E-12
**385**	2.27685059E-11	3.72353413E-11	1.22317494E-11	5.45173276E-12	1.03720029E-12
**386**	2.28194582E-11	3.7277803E-11	1.22464501E-11	5.46041103E-12	1.03863525E-12
**387**	2.28701259E-11	3.73199298E-11	1.22610361E-11	5.46903206E-12	1.04005949E-12
**388**	2.29205098E-11	3.73617229E-11	1.22755079E-11	5.47759601E-12	1.04147306E-12
**389**	2.29706104E-11	3.74031833E-11	1.22898659E-11	5.48610305E-12	1.04287598E-12
**390**	2.30204281E-11	3.74443123E-11	1.23041105E-11	5.49455334E-12	1.04426831E-12
**391**	2.30699637E-11	3.74851109E-11	1.23182421E-11	5.50294704E-12	1.04565007E-12
**392**	2.31192177E-11	3.75255804E-11	1.23322611E-11	5.51128431E-12	1.0470213E-12
**393**	2.31681906E-11	3.75657219E-11	1.23461679E-11	5.51956532E-12	1.04838205E-12
**394**	2.32168831E-11	3.76055366E-11	1.2359963E-11	5.52779025E-12	1.04973235E-12
**395**	2.32652958E-11	3.76450256E-11	1.23736467E-11	5.53595924E-12	1.05107224E-12
**396**	2.33134293E-11	3.76841901E-11	1.23872195E-11	5.54407248E-12	1.05240176E-12
**397**	2.33612842E-11	3.77230312E-11	1.24006817E-11	5.55213013E-12	1.05372094E-12
**398**	2.34088612E-11	3.77615503E-11	1.24140339E-11	5.56013236E-12	1.05502983E-12
**399**	2.34561608E-11	3.77997483E-11	1.24272764E-11	5.56807935E-12	1.05632846E-12
**400**	2.35031838E-11	3.78376266E-11	1.24404097E-11	5.57597126E-12	1.05761688E-12
**401**	2.35499307E-11	3.78751863E-11	1.24534341E-11	5.58380826E-12	1.05889512E-12
**402**	2.35964024E-11	3.79124285E-11	1.246635E-11	5.59159054E-12	1.06016322E-12
**403**	2.36425993E-11	3.79493546E-11	1.2479158E-11	5.59931826E-12	1.06142123E-12
**404**	2.36885222E-11	3.79859656E-11	1.24918584E-11	5.6069916E-12	1.06266917E-12
**405**	2.37341718E-11	3.80222628E-11	1.25044516E-11	5.61461073E-12	1.06390709E-12
**406**	2.37795487E-11	3.80582473E-11	1.25169381E-11	5.62217584E-12	1.06513503E-12
**407**	2.38246537E-11	3.80939204E-11	1.25293182E-11	5.62968709E-12	1.06635302E-12
**408**	2.38694874E-11	3.81292832E-11	1.25415924E-11	5.63714466E-12	1.06756111E-12

**409**	2.39140506E-11	3.8164337E-11	1.25537612E-11	5.64454874E-12	1.06875934E-12
**410**	2.39583439E-11	3.81990829E-11	1.25658249E-11	5.65189951E-12	1.06994774E-12
**411**	2.40023681E-11	3.82335221E-11	1.25777839E-11	5.65919713E-12	1.07112636E-12
**412**	2.40461239E-11	3.8267656E-11	1.25896388E-11	5.66644179E-12	1.07229522E-12
**413**	2.4089612E-11	3.83014855E-11	1.26013898E-11	5.67363368E-12	1.07345438E-12
**414**	2.41328331E-11	3.83350121E-11	1.26130375E-11	5.68077296E-12	1.07460388E-12
**415**	2.41757881E-11	3.83682368E-11	1.26245822E-11	5.68785983E-12	1.07574374E-12
**416**	2.42184776E-11	3.84011609E-11	1.26360244E-11	5.69489447E-12	1.07687402E-12
**417**	2.42609023E-11	3.84337855E-11	1.26473645E-11	5.70187705E-12	1.07799474E-12
**418**	2.43030631E-11	3.8466112E-11	1.26586029E-11	5.70880776E-12	1.07910596E-12
**419**	2.43449606E-11	3.84981414E-11	1.266974E-11	5.71568679E-12	1.0802077E-12
**420**	2.43865957E-11	3.85298751E-11	1.26807763E-11	5.72251431E-12	1.08130001E-12
**421**	2.44279691E-11	3.85613141E-11	1.26917122E-11	5.72929052E-12	1.08238293E-12
**422**	2.44690816E-11	3.85924598E-11	1.2702548E-11	5.73601559E-12	1.0834565E-12
**423**	2.45099339E-11	3.86233134E-11	1.27132843E-11	5.7426897E-12	1.08452075E-12
**424**	2.45505269E-11	3.8653876E-11	1.27239215E-11	5.74931306E-12	1.08557573E-12
**425**	2.45908612E-11	3.86841488E-11	1.27344599E-11	5.75588583E-12	1.08662147E-12
**426**	2.46309378E-11	3.87141331E-11	1.27449E-11	5.7624082E-12	1.08765802E-12
**427**	2.46707573E-11	3.87438301E-11	1.27552422E-11	5.76888037E-12	1.08868541E-12
**428**	2.47103205E-11	3.87732409E-11	1.27654869E-11	5.77530251E-12	1.08970369E-12
**429**	2.47496284E-11	3.88023668E-11	1.27756346E-11	5.78167482E-12	1.09071288E-12
**430**	2.47886816E-11	3.88312091E-11	1.27856856E-11	5.78799747E-12	1.09171304E-12
**431**	2.48274809E-11	3.88597688E-11	1.27956404E-11	5.79427066E-12	1.0927042E-12
**432**	2.48660272E-11	3.88880472E-11	1.28054994E-11	5.80049457E-12	1.09368639E-12
**433**	2.49043213E-11	3.89160456E-11	1.28152631E-11	5.80666939E-12	1.09465967E-12
**434**	2.49423639E-11	3.89437651E-11	1.28249318E-11	5.81279531E-12	1.09562406E-12
**435**	2.49801559E-11	3.89712069E-11	1.28345059E-11	5.81887251E-12	1.09657961E-12
**436**	2.50176982E-11	3.89983722E-11	1.28439859E-11	5.82490118E-12	1.09752635E-12
**437**	2.50549914E-11	3.90252623E-11	1.28533722E-11	5.83088151E-12	1.09846433E-12
**438**	2.50920365E-11	3.90518783E-11	1.28626653E-11	5.83681368E-12	1.09939358E-12
**439**	2.51288342E-11	3.90782214E-11	1.28718654E-11	5.84269789E-12	1.10031415E-12
**440**	2.51653855E-11	3.91042929E-11	1.28809731E-11	5.84853432E-12	1.10122606E-12
**441**	2.5201691E-11	3.91300939E-11	1.28899887E-11	5.85432316E-12	1.10212937E-12
**442**	2.52377517E-11	3.91556256E-11	1.28989127E-11	5.86006459E-12	1.1030241E-12
**443**	2.52735683E-11	3.91808893E-11	1.29077455E-11	5.86575881E-12	1.1039103E-12
**444**	2.53091418E-11	3.92058861E-11	1.29164874E-11	5.871406E-12	1.10478801E-12
**445**	2.53444729E-11	3.92306171E-11	1.2925139E-11	5.87700635E-12	1.10565726E-12
**446**	2.53795624E-11	3.92550837E-11	1.29337005E-11	5.88256004E-12	1.10651809E-12
**447**	2.54144113E-11	3.9279287E-11	1.29421725E-11	5.88806728E-12	1.10737054E-12
**448**	2.54490203E-11	3.93032281E-11	1.29505554E-11	5.89352823E-12	1.10821465E-12
**449**	2.54833903E-11	3.93269083E-11	1.29588494E-11	5.89894309E-12	1.10905046E-12
**450**	2.55175222E-11	3.93503287E-11	1.29670552E-11	5.90431205E-12	1.109878E-12
**451**	2.55514167E-11	3.93734906E-11	1.2975173E-11	5.9096353E-12	1.11069732E-12
**452**	2.55850747E-11	3.93963951E-11	1.29832032E-11	5.91491302E-12	1.11150844E-12
**453**	2.56184971E-11	3.94190434E-11	1.29911463E-11	5.9201454E-12	1.11231142E-12
**454**	2.56516846E-11	3.94414366E-11	1.29990027E-11	5.92533263E-12	1.11310629E-12
**455**	2.56846383E-11	3.9463576E-11	1.30067728E-11	5.93047489E-12	1.11389308E-12
**456**	2.57173588E-11	3.94854626E-11	1.30144569E-11	5.93557237E-12	1.11467183E-12
**457**	2.57498471E-11	3.95070978E-11	1.30220556E-11	5.94062526E-12	1.11544258E-12
**458**	2.5782104E-11	3.95284826E-11	1.30295691E-11	5.94563375E-12	1.11620537E-12
**459**	2.58141304E-11	3.95496182E-11	1.30369979E-11	5.95059801E-12	1.11696024E-12

**460**	2.5845927E-11	3.95705058E-11	1.30443424E-11	5.95551824E-12	1.11770722E-12
**461**	2.58774948E-11	3.95911465E-11	1.3051603E-11	5.96039463E-12	1.11844634E-12
**462**	2.59088346E-11	3.96115416E-11	1.30587801E-11	5.96522735E-12	1.11917766E-12
**463**	2.59399473E-11	3.96316921E-11	1.30658741E-11	5.9700166E-12	1.1199012E-12
**464**	2.59708337E-11	3.96515992E-11	1.30728853E-11	5.97476256E-12	1.120617E-12
**465**	2.60014947E-11	3.9671264E-11	1.30798142E-11	5.97946542E-12	1.1213251E-12
**466**	2.60319311E-11	3.96906878E-11	1.30866612E-11	5.98412536E-12	1.12202554E-12
**467**	2.60621437E-11	3.97098716E-11	1.30934267E-11	5.98874256E-12	1.12271835E-12
**468**	2.60921335E-11	3.97288167E-11	1.3100111E-11	5.99331721E-12	1.12340357E-12
**469**	2.61219013E-11	3.9747524E-11	1.31067145E-11	5.9978495E-12	1.12408123E-12
**470**	2.61514479E-11	3.97659949E-11	1.31132377E-11	6.00233961E-12	1.12475137E-12
**471**	2.61807742E-11	3.97842304E-11	1.31196809E-11	6.00678771E-12	1.12541404E-12
**472**	2.6209881E-11	3.98022316E-11	1.31260446E-11	6.01119401E-12	1.12606926E-12
**473**	2.62387692E-11	3.98199997E-11	1.3132329E-11	6.01555867E-12	1.12671706E-12
**474**	2.62674397E-11	3.98375358E-11	1.31385346E-11	6.01988189E-12	1.1273575E-12
**475**	2.62958933E-11	3.98548411E-11	1.31446618E-11	6.02416384E-12	1.1279906E-12
**476**	2.63241309E-11	3.98719166E-11	1.3150711E-11	6.0284047E-12	1.1286164E-12
**477**	2.63521532E-11	3.98887635E-11	1.31566825E-11	6.03260467E-12	1.12923493E-12
**478**	2.63799613E-11	3.99053829E-11	1.31625767E-11	6.03676392E-12	1.12984623E-12
**479**	2.64075558E-11	3.99217759E-11	1.31683941E-11	6.04088263E-12	1.13045034E-12
**480**	2.64349377E-11	3.99379436E-11	1.31741349E-11	6.04496098E-12	1.1310473E-12
**481**	2.64621079E-11	3.99538872E-11	1.31797996E-11	6.04899916E-12	1.13163712E-12
**482**	2.64890671E-11	3.99696077E-11	1.31853886E-11	6.05299734E-12	1.13221987E-12
**483**	2.65158162E-11	3.99851062E-11	1.31909021E-11	6.0569557E-12	1.13279556E-12
**484**	2.65423561E-11	4.00003838E-11	1.31963407E-11	6.06087443E-12	1.13336423E-12
**485**	2.65686875E-11	4.00154417E-11	1.32017046E-11	6.06475371E-12	1.13392592E-12
**486**	2.65948115E-11	4.00302809E-11	1.32069943E-11	6.0685937E-12	1.13448066E-12
**487**	2.66207288E-11	4.00449026E-11	1.32122101E-11	6.0723946E-12	1.13502849E-12
**488**	2.66464402E-11	4.00593077E-11	1.32173524E-11	6.07615657E-12	1.13556944E-12
**489**	2.66719466E-11	4.00734974E-11	1.32224215E-11	6.0798798E-12	1.13610355E-12
**490**	2.66972489E-11	4.00874728E-11	1.32274179E-11	6.08356447E-12	1.13663085E-12
**491**	2.67223478E-11	4.01012349E-11	1.32323419E-11	6.08721074E-12	1.13715138E-12
**492**	2.67472443E-11	4.01147848E-11	1.32371938E-11	6.09081881E-12	1.13766517E-12
**493**	2.67719391E-11	4.01281237E-11	1.32419741E-11	6.09438884E-12	1.13817225E-12
**494**	2.67964332E-11	4.01412524E-11	1.3246683E-11	6.097921E-12	1.13867266E-12
**495**	2.68207272E-11	4.01541722E-11	1.3251321E-11	6.10141549E-12	1.13916643E-12
**496**	2.68448222E-11	4.01668841E-11	1.32558884E-11	6.10487246E-12	1.13965359E-12
**497**	2.68687189E-11	4.01793891E-11	1.32603856E-11	6.10829209E-12	1.14013419E-12
**498**	2.68924182E-11	4.01916883E-11	1.3264813E-11	6.11167457E-12	1.14060825E-12
**499**	2.69159208E-11	4.02037827E-11	1.32691708E-11	6.11502006E-12	1.14107581E-12
**500**	2.69392276E-11	4.02156734E-11	1.32734595E-11	6.11832873E-12	1.14153689E-12

Inelastic collisions' Rate Coefficients: Pseudo-Singlet State					
Initial rotational state: j=5					
T(K)	k ($cm^3mol^{-1}s^{-1}$)				
	**Final State: j'=4**	**Final State: j'=3**	**Final State: j'=2**	**Final State: j'=1**	**Final State: j'=0**
**5**	1.88478763E-12	3.36611047E-13	5.24564059E-14	2.56499712E-14	8.91247753E-15
**6**	1.83419027E-12	3.23206439E-13	5.12450263E-14	2.51178413E-14	8.76238131E-15
**7**	1.80221661E-12	3.14417048E-13	5.05124319E-14	2.48040267E-14	8.6777859E-15
**8**	1.78372343E-12	3.08811745E-13	5.01405957E-14	2.46575421E-14	8.64522273E-15
**9**	1.77560767E-12	3.05560318E-13	5.00574949E-14	2.46468142E-14	8.65603703E-15
**10**	1.77584094E-12	3.04142471E-13	5.0215835E-14	2.47508449E-14	8.70428437E-15
**11**	1.78302164E-12	3.04213352E-13	5.05830599E-14	2.49550346E-14	8.78572532E-15
**12**	1.79613691E-12	3.05533918E-13	5.11359174E-14	2.52488731E-14	8.89724499E-15
**13**	1.81442608E-12	3.07932077E-13	5.18572675E-14	2.56245614E-14	9.03649264E-15
**14**	1.8372983E-12	3.11279766E-13	5.27341075E-14	2.60761491E-14	9.20164799E-15
**15**	1.86428054E-12	3.15478789E-13	5.37562945E-14	2.65989685E-14	9.39126392E-15
**16**	1.89498361E-12	3.20451705E-13	5.49156897E-14	2.71892529E-14	9.60415753E-15
**17**	1.92907934E-12	3.26135824E-13	5.62055781E-14	2.78438755E-14	9.83933455E-15
**18**	1.96628508E-12	3.32479171E-13	5.76202759E-14	2.85601723E-14	1.00959376E-14
**19**	2.00635309E-12	3.39437767E-13	5.91548721E-14	2.93358261E-14	1.03732125E-14
**20**	2.04906344E-12	3.46973792E-13	6.08050631E-14	3.01687926E-14	1.06704869E-14
**21**	2.09421907E-12	3.5505434E-13	6.25670505E-14	3.10572557E-14	1.09871582E-14
**22**	2.14164247E-12	3.63650571E-13	6.44374797E-14	3.19996019E-14	1.13226866E-14
**23**	2.19117338E-12	3.72737109E-13	6.64134011E-14	3.29944038E-14	1.1676591E-14
**24**	2.24266696E-12	3.82291606E-13	6.84922438E-14	3.40404096E-14	1.20484466E-14
**25**	2.29599249E-12	3.92294397E-13	7.06717933E-14	3.51365341E-14	1.24378823E-14
**26**	2.35103211E-12	4.02728217E-13	7.29501699E-14	3.6281849E-14	1.28445781E-14
**27**	2.40767982E-12	4.13577949E-13	7.53258053E-14	3.74755728E-14	1.32682621E-14
**28**	2.4658404E-12	4.24830398E-13	7.77974171E-14	3.87170587E-14	1.37087064E-14
**29**	2.52542848E-12	4.3647408E-13	8.03639822E-14	4.00057819E-14	1.4165723E-14
**30**	2.58636759E-12	4.4849902E-13	8.30247085E-14	4.13413267E-14	1.46391592E-14
**31**	2.64858927E-12	4.60896569E-13	8.57790068E-14	4.27233719E-14	1.51288933E-14
**32**	2.71203222E-12	4.73659233E-13	8.86264634E-14	4.41516786E-14	1.56348299E-14
**33**	2.77664153E-12	4.86780512E-13	9.15668134E-14	4.56260768E-14	1.61568954E-14
**34**	2.84236792E-12	5.00254761E-13	9.45999167E-14	4.71464535E-14	1.66950345E-14
**35**	2.9091671E-12	5.14077057E-13	9.77257349E-14	4.87127421E-14	1.72492057E-14
**36**	2.97699912E-12	5.28243087E-13	1.00944311E-13	5.03249119E-14	1.78193787E-14
**37**	3.04582788E-12	5.42749043E-13	1.04255752E-13	5.198296E-14	1.84055309E-14
**38**	3.11562059E-12	5.57591537E-13	1.07660212E-13	5.36869032E-14	1.90076452E-14
**39**	3.18634736E-12	5.72767518E-13	1.11157878E-13	5.54367714E-14	1.96257075E-14
**40**	3.25798085E-12	5.88274213E-13	1.14748959E-13	5.72326019E-14	2.02597051E-14
**41**	3.33049587E-12	6.04109064E-13	1.18433675E-13	5.90744347E-14	2.0909625E-14
**42**	3.40386915E-12	6.20269682E-13	1.2221225E-13	6.09623082E-14	2.15754525E-14
**43**	3.47807909E-12	6.36753807E-13	1.26084903E-13	6.28962565E-14	2.22571705E-14
**44**	3.55310549E-12	6.53559272E-13	1.30051843E-13	6.48763061E-14	2.29547585E-14
**45**	3.62892942E-12	6.70683977E-13	1.34113264E-13	6.69024739E-14	2.36681917E-14
**46**	3.70553303E-12	6.88125862E-13	1.3826934E-13	6.89747658E-14	2.43974411E-14
**47**	3.78289943E-12	7.05882893E-13	1.42520227E-13	7.10931754E-14	2.51424728E-14
**48**	3.86101255E-12	7.23953043E-13	1.46866054E-13	7.32576829E-14	2.59032479E-14
**49**	3.93985707E-12	7.42334282E-13	1.51306925E-13	7.54682545E-14	2.66797223E-14
**50**	4.01941833E-12	7.61024563E-13	1.55842918E-13	7.7724842E-14	2.74718471E-14
**51**	4.09968221E-12	7.8002182E-13	1.60474083E-13	8.0027383E-14	2.82795679E-14

**52**	4.18063515E-12	7.99323961E-13	1.65200443E-13	8.23758001E-14	2.91028255E-14
**53**	4.26226404E-12	8.18928861E-13	1.70021991E-13	8.47700018E-14	2.99415559E-14
**54**	4.34455617E-12	8.38834362E-13	1.74938693E-13	8.72098821E-14	3.07956903E-14
**55**	4.42749924E-12	8.5903827E-13	1.79950489E-13	8.96953213E-14	3.16651555E-14
**56**	4.5110813E-12	8.79538356E-13	1.85057288E-13	9.22261859E-14	3.25498741E-14
**57**	4.59529071E-12	9.00332352E-13	1.90258975E-13	9.48023293E-14	3.34497644E-14
**58**	4.68011614E-12	9.21417957E-13	1.95555408E-13	9.7423592E-14	3.43647412E-14
**59**	4.76554654E-12	9.42792831E-13	2.0094642E-13	1.00089802E-13	3.52947155E-14
**60**	4.85157111E-12	9.64454602E-13	2.0643182E-13	1.02800777E-13	3.6239595E-14
**61**	4.93817932E-12	9.86400864E-13	2.1201139E-13	1.05556321E-13	3.71992843E-14
**62**	5.02536086E-12	1.00862918E-12	2.17684893E-13	1.08356228E-13	3.81736853E-14
**63**	5.11310565E-12	1.03113708E-12	2.23452068E-13	1.11200283E-13	3.91626971E-14
**64**	5.20140381E-12	1.05392207E-12	2.29312631E-13	1.14088259E-13	4.01662163E-14
**65**	5.29024568E-12	1.07698163E-12	2.35266281E-13	1.17019921E-13	4.11841373E-14
**66**	5.3796218E-12	1.10031321E-12	2.41312692E-13	1.19995024E-13	4.22163526E-14
**67**	5.46952287E-12	1.12391424E-12	2.47451524E-13	1.23013316E-13	4.32627528E-14
**68**	5.55993979E-12	1.14778213E-12	2.53682417E-13	1.26074535E-13	4.43232268E-14
**69**	5.65086365E-12	1.17191427E-12	2.60004991E-13	1.29178411E-13	4.53976619E-14
**70**	5.74228567E-12	1.19630803E-12	2.66418852E-13	1.32324669E-13	4.64859443E-14
**71**	5.83419726E-12	1.22096077E-12	2.72923589E-13	1.35513025E-13	4.75879586E-14
**72**	5.92658999E-12	1.24586983E-12	2.79518774E-13	1.38743189E-13	4.87035887E-14
**73**	6.01945554E-12	1.27103253E-12	2.86203966E-13	1.42014863E-13	4.98327174E-14
**74**	6.11278579E-12	1.29644619E-12	2.92978707E-13	1.45327745E-13	5.09752264E-14
**75**	6.20657272E-12	1.32210812E-12	2.99842528E-13	1.48681526E-13	5.2130997E-14
**76**	6.30080846E-12	1.3480156E-12	3.06794943E-13	1.52075891E-13	5.32999097E-14
**77**	6.39548526E-12	1.37416593E-12	3.13835456E-13	1.55510522E-13	5.44818443E-14
**78**	6.49059551E-12	1.40055637E-12	3.20963556E-13	1.58985092E-13	5.56766801E-14
**79**	6.58613169E-12	1.42718421E-12	3.28178721E-13	1.62499271E-13	5.6884296E-14
**80**	6.68208642E-12	1.45404669E-12	3.35480417E-13	1.66052725E-13	5.81045705E-14
**81**	6.77845241E-12	1.48114107E-12	3.42868097E-13	1.69645114E-13	5.93373815E-14
**82**	6.87522249E-12	1.50846461E-12	3.50341204E-13	1.73276094E-13	6.05826069E-14
**83**	6.97238956E-12	1.53601455E-12	3.5789917E-13	1.76945317E-13	6.1840124E-14
**84**	7.06994664E-12	1.56378813E-12	3.65541414E-13	1.80652429E-13	6.31098098E-14
**85**	7.16788682E-12	1.59178259E-12	3.73267347E-13	1.84397075E-13	6.43915413E-14
**86**	7.26620328E-12	1.61999516E-12	3.81076369E-13	1.88178892E-13	6.5685195E-14
**87**	7.36488926E-12	1.64842306E-12	3.88967867E-13	1.91997517E-13	6.6990647E-14
**88**	7.4639381E-12	1.67706353E-12	3.96941221E-13	1.95852581E-13	6.83077735E-14
**89**	7.56334317E-12	1.70591377E-12	4.049958E-13	1.99743711E-13	6.96364502E-14
**90**	7.66309794E-12	1.734971E-12	4.13130961E-13	2.03670531E-13	7.09765524E-14
**91**	7.76319591E-12	1.76423244E-12	4.21346054E-13	2.07632661E-13	7.23279554E-14
**92**	7.86363063E-12	1.79369529E-12	4.29640418E-13	2.11629717E-13	7.36905341E-14
**93**	7.96439573E-12	1.82335676E-12	4.38013381E-13	2.15661313E-13	7.5064163E-14
**94**	8.06548484E-12	1.85321404E-12	4.46464264E-13	2.19727059E-13	7.64487162E-14
**95**	8.16689166E-12	1.88326433E-12	4.54992376E-13	2.23826559E-13	7.78440676E-14
**96**	8.2686099E-12	1.91350483E-12	4.63597017E-13	2.27959418E-13	7.92500908E-14
**97**	8.37063333E-12	1.94393271E-12	4.72277478E-13	2.32125233E-13	8.06666587E-14
**98**	8.47295572E-12	1.97454516E-12	4.81033041E-13	2.36323601E-13	8.20936441E-14
**99**	8.57557087E-12	2.00533935E-12	4.89862976E-13	2.40554115E-13	8.35309191E-14
**100**	8.67847261E-12	2.03631245E-12	4.98766547E-13	2.44816364E-13	8.49783555E-14
**101**	8.78165476E-12	2.06746164E-12	5.07743005E-13	2.49109934E-13	8.64358245E-14
**102**	8.88511117E-12	2.09878407E-12	5.16791596E-13	2.53434408E-13	8.79031969E-14

**103**	8.9888357E-12	2.13027689E-12	5.25911552E-13	2.57789366E-13	8.9380343E-14
**104**	9.09282221E-12	2.16193726E-12	5.35102099E-13	2.62174385E-13	9.08671324E-14
**105**	9.19706455E-12	2.19376232E-12	5.44362452E-13	2.66589039E-13	9.23634342E-14
**106**	9.3015566E-12	2.22574921E-12	5.53691818E-13	2.71032898E-13	9.3869117E-14
**107**	9.40629222E-12	2.25789505E-12	5.63089395E-13	2.7550553E-13	9.53840488E-14
**108**	9.51126525E-12	2.29019698E-12	5.7255437E-13	2.800065E-13	9.69080967E-14
**109**	9.61646955E-12	2.32265211E-12	5.82085924E-13	2.8453537E-13	9.84411277E-14
**110**	9.72189895E-12	2.35525757E-12	5.91683227E-13	2.890917E-13	9.99830076E-14
**111**	9.82754729E-12	2.38801045E-12	6.0134544E-13	2.93675046E-13	1.01533602E-13
**112**	9.93340837E-12	2.42090786E-12	6.11071717E-13	2.98284963E-13	1.03092776E-13
**113**	1.0039476E-11	2.4539469E-12	6.20861203E-13	3.02921002E-13	1.04660393E-13
**114**	1.0145744E-11	2.48712465E-12	6.30713034E-13	3.07582711E-13	1.06236317E-13
**115**	1.0252206E-11	2.52043821E-12	6.40626338E-13	3.12269639E-13	1.0782041E-13
**116**	1.0358856E-11	2.55388464E-12	6.50600234E-13	3.16981328E-13	1.09412535E-13
**117**	1.04656875E-11	2.58746104E-12	6.60633835E-13	3.21717321E-13	1.11012554E-13
**118**	1.05726943E-11	2.62116446E-12	6.70726244E-13	3.26477159E-13	1.12620327E-13
**119**	1.06798702E-11	2.65499197E-12	6.80876559E-13	3.31260378E-13	1.14235714E-13
**120**	1.07872087E-11	2.68894064E-12	6.91083868E-13	3.36066516E-13	1.15858575E-13
**121**	1.08947036E-11	2.72300751E-12	7.01347252E-13	3.40895105E-13	1.17488769E-13
**122**	1.10023484E-11	2.75718964E-12	7.11665786E-13	3.45745679E-13	1.19126154E-13
**123**	1.11101369E-11	2.79148409E-12	7.22038539E-13	3.50617768E-13	1.20770589E-13
**124**	1.12180626E-11	2.82588789E-12	7.32464571E-13	3.55510902E-13	1.2242193E-13
**125**	1.1326119E-11	2.86039809E-12	7.42942938E-13	3.60424607E-13	1.24080033E-13
**126**	1.14342998E-11	2.89501173E-12	7.53472687E-13	3.65358412E-13	1.25744756E-13
**127**	1.15425984E-11	2.92972586E-12	7.64052861E-13	3.7031184E-13	1.27415954E-13
**128**	1.16510085E-11	2.9645375E-12	7.74682498E-13	3.75284416E-13	1.29093481E-13
**129**	1.17595235E-11	2.9994437E-12	7.85360629E-13	3.80275664E-13	1.30777193E-13
**130**	1.1868137E-11	3.03444149E-12	7.96086281E-13	3.85285105E-13	1.32466943E-13
**131**	1.19768423E-11	3.0695279E-12	8.06858475E-13	3.90312262E-13	1.34162586E-13
**132**	1.20856331E-11	3.10469999E-12	8.17676229E-13	3.95356656E-13	1.35863975E-13
**133**	1.21945026E-11	3.13995478E-12	8.28538554E-13	4.00417807E-13	1.37570963E-13
**134**	1.23034445E-11	3.17528933E-12	8.39444461E-13	4.05495235E-13	1.39283402E-13
**135**	1.2412452E-11	3.21070066E-12	8.50392953E-13	4.10588461E-13	1.41001146E-13
**136**	1.25215187E-11	3.24618582E-12	8.61383033E-13	4.15697005E-13	1.42724047E-13
**137**	1.2630638E-11	3.28174188E-12	8.72413699E-13	4.20820386E-13	1.44451956E-13
**138**	1.27398033E-11	3.31736588E-12	8.83483945E-13	4.25958125E-13	1.46184726E-13
**139**	1.2849008E-11	3.35305487E-12	8.94592767E-13	4.31109743E-13	1.47922208E-13
**140**	1.29582455E-11	3.38880593E-12	9.05739153E-13	4.3627476E-13	1.49664253E-13
**141**	1.30675092E-11	3.42461613E-12	9.16922094E-13	4.41452699E-13	1.51410714E-13
**142**	1.31767926E-11	3.46048254E-12	9.28140576E-13	4.4664308E-13	1.53161441E-13
**143**	1.3286089E-11	3.49640225E-12	9.39393586E-13	4.51845428E-13	1.54916287E-13
**144**	1.33953919E-11	3.53237235E-12	9.50680108E-13	4.57059266E-13	1.56675103E-13
**145**	1.35046947E-11	3.56838993E-12	9.61999126E-13	4.62284119E-13	1.5843774E-13
**146**	1.36139908E-11	3.60445212E-12	9.73349624E-13	4.67519514E-13	1.60204051E-13
**147**	1.37232738E-11	3.64055604E-12	9.84730587E-13	4.72764978E-13	1.61973886E-13
**148**	1.3832537E-11	3.6766988E-12	9.96140998E-13	4.7802004E-13	1.63747098E-13
**149**	1.39417739E-11	3.71287756E-12	1.00757984E-12	4.83284231E-13	1.65523541E-13
**150**	1.4050978E-11	3.74908947E-12	1.0190461E-12	4.88557082E-13	1.67303065E-13
**151**	1.41601428E-11	3.7853317E-12	1.03053877E-12	4.93838129E-13	1.69085524E-13
**152**	1.42692619E-11	3.82160142E-12	1.04205683E-12	4.99126906E-13	1.70870771E-13
**153**	1.43783288E-11	3.85789583E-12	1.05359926E-12	5.04422951E-13	1.72658661E-13

**154**	1.4487337E-11	3.89421213E-12	1.06516508E-12	5.09725805E-13	1.74449046E-13
**155**	1.45962801E-11	3.93054755E-12	1.07675326E-12	5.1503501E-13	1.76241782E-13
**156**	1.47051519E-11	3.96689933E-12	1.0883628E-12	5.2035011E-13	1.78036724E-13
**157**	1.48139458E-11	4.00326471E-12	1.0999927E-12	5.25670653E-13	1.79833726E-13
**158**	1.49226556E-11	4.03964097E-12	1.11164197E-12	5.30996188E-13	1.81632646E-13
**159**	1.5031275E-11	4.07602539E-12	1.12330961E-12	5.36326268E-13	1.83433339E-13
**160**	1.51397978E-11	4.11241528E-12	1.13499463E-12	5.41660447E-13	1.85235664E-13
**161**	1.52482176E-11	4.14880796E-12	1.14669604E-12	5.46998283E-13	1.87039479E-13
**162**	1.53565284E-11	4.18520077E-12	1.15841287E-12	5.52339337E-13	1.88844641E-13
**163**	1.54647239E-11	4.22159107E-12	1.17014412E-12	5.57683173E-13	1.90651011E-13
**164**	1.55727981E-11	4.25797624E-12	1.18188883E-12	5.63029357E-13	1.92458449E-13
**165**	1.56807449E-11	4.29435369E-12	1.19364603E-12	5.68377461E-13	1.94266816E-13
**166**	1.57885582E-11	4.33072082E-12	1.20541476E-12	5.73727057E-13	1.96075973E-13
**167**	1.5896232E-11	4.36707509E-12	1.21719405E-12	5.79077721E-13	1.97885784E-13
**168**	1.60037605E-11	4.40341395E-12	1.22898295E-12	5.84429035E-13	1.99696112E-13
**169**	1.61111378E-11	4.43973489E-12	1.24078053E-12	5.89780581E-13	2.01506822E-13
**170**	1.62183579E-11	4.47603542E-12	1.25258582E-12	5.95131947E-13	2.03317779E-13
**171**	1.63254151E-11	4.51231306E-12	1.26439791E-12	6.00482724E-13	2.05128849E-13
**172**	1.64323036E-11	4.54856537E-12	1.27621586E-12	6.05832506E-13	2.06939901E-13
**173**	1.65390177E-11	4.58478992E-12	1.28803875E-12	6.11180892E-13	2.08750802E-13
**174**	1.66455519E-11	4.62098432E-12	1.29986566E-12	6.16527483E-13	2.10561423E-13
**175**	1.67519004E-11	4.65714618E-12	1.31169568E-12	6.21871887E-13	2.12371633E-13
**176**	1.68580578E-11	4.69327316E-12	1.32352792E-12	6.27213712E-13	2.14181305E-13
**177**	1.69640186E-11	4.72936292E-12	1.33536148E-12	6.32552573E-13	2.15990311E-13
**178**	1.70697773E-11	4.76541317E-12	1.34719547E-12	6.37888088E-13	2.17798525E-13
**179**	1.71753286E-11	4.80142162E-12	1.35902902E-12	6.43219879E-13	2.19605823E-13
**180**	1.72806672E-11	4.83738603E-12	1.37086124E-12	6.48547572E-13	2.2141208E-13
**181**	1.73857878E-11	4.87330417E-12	1.38269128E-12	6.53870797E-13	2.23217174E-13
**182**	1.74906853E-11	4.90917383E-12	1.39451829E-12	6.5918919E-13	2.25020985E-13
**183**	1.75953545E-11	4.94499284E-12	1.4063414E-12	6.64502388E-13	2.26823391E-13
**184**	1.76997903E-11	4.98075907E-12	1.41815979E-12	6.69810036E-13	2.28624273E-13
**185**	1.78039878E-11	5.01647037E-12	1.42997262E-12	6.7511178E-13	2.30423516E-13
**186**	1.7907942E-11	5.05212467E-12	1.44177907E-12	6.80407273E-13	2.32221001E-13
**187**	1.8011648E-11	5.08771988E-12	1.45357833E-12	6.8569617E-13	2.34016614E-13
**188**	1.8115101E-11	5.12325398E-12	1.46536958E-12	6.90978132E-13	2.35810241E-13
**189**	1.82182962E-11	5.15872494E-12	1.47715204E-12	6.96252824E-13	2.3760177E-13
**190**	1.83212291E-11	5.19413079E-12	1.4889249E-12	7.01519915E-13	2.3939109E-13
**191**	1.84238949E-11	5.22946955E-12	1.50068741E-12	7.0677908E-13	2.41178091E-13
**192**	1.85262891E-11	5.26473931E-12	1.51243877E-12	7.12029996E-13	2.42962664E-13
**193**	1.86284073E-11	5.29993815E-12	1.52417824E-12	7.17272345E-13	2.44744703E-13
**194**	1.87302449E-11	5.3350642E-12	1.53590505E-12	7.22505817E-13	2.46524101E-13
**195**	1.88317977E-11	5.37011561E-12	1.54761847E-12	7.27730101E-13	2.48300755E-13
**196**	1.89330614E-11	5.40509056E-12	1.55931777E-12	7.32944895E-13	2.50074562E-13
**197**	1.90340317E-11	5.43998725E-12	1.57100221E-12	7.38149898E-13	2.51845419E-13
**198**	1.91347045E-11	5.47480392E-12	1.58267108E-12	7.43344817E-13	2.53613226E-13
**199**	1.92350758E-11	5.50953884E-12	1.59432368E-12	7.48529361E-13	2.55377885E-13
**200**	1.93351414E-11	5.54419028E-12	1.6059593E-12	7.53703245E-13	2.57139299E-13
**201**	1.94348976E-11	5.57875657E-12	1.61757727E-12	7.58866186E-13	2.5889737E-13
**202**	1.95343403E-11	5.61323606E-12	1.6291769E-12	7.64017909E-13	2.60652006E-13
**203**	1.96334658E-11	5.64762711E-12	1.64075752E-12	7.69158141E-13	2.62403111E-13
**204**	1.97322704E-11	5.68192813E-12	1.65231848E-12	7.74286614E-13	2.64150595E-13

**205**	1.98307504E-11	5.71613755E-12	1.66385913E-12	7.79403065E-13	2.65894367E-13
**206**	1.99289021E-11	5.75025382E-12	1.67537882E-12	7.84507236E-13	2.67634337E-13
**207**	2.00267221E-11	5.78427541E-12	1.68687692E-12	7.89598871E-13	2.69370419E-13
**208**	2.01242068E-11	5.81820086E-12	1.69835282E-12	7.94677722E-13	2.71102526E-13
**209**	2.0221353E-11	5.85202868E-12	1.7098059E-12	7.99743542E-13	2.72830573E-13
**210**	2.03181572E-11	5.88575745E-12	1.72123556E-12	8.04796091E-13	2.74554476E-13
**211**	2.04146162E-11	5.91938576E-12	1.73264121E-12	8.09835132E-13	2.76274154E-13
**212**	2.05107268E-11	5.95291223E-12	1.74402226E-12	8.14860433E-13	2.77989526E-13
**213**	2.06064859E-11	5.9863355E-12	1.75537815E-12	8.19871766E-13	2.79700512E-13
**214**	2.07018904E-11	6.01965425E-12	1.76670829E-12	8.24868906E-13	2.81407035E-13
**215**	2.07969373E-11	6.05286717E-12	1.77801215E-12	8.29851636E-13	2.83109018E-13
**216**	2.08916237E-11	6.085973E-12	1.78928918E-12	8.3481974E-13	2.84806386E-13
**217**	2.09859468E-11	6.11897048E-12	1.80053883E-12	8.39773007E-13	2.86499065E-13
**218**	2.10799037E-11	6.15185839E-12	1.81176059E-12	8.44711231E-13	2.88186983E-13
**219**	2.11734918E-11	6.18463553E-12	1.82295393E-12	8.4963421E-13	2.89870069E-13
**220**	2.12667083E-11	6.21730074E-12	1.83411835E-12	8.54541746E-13	2.91548252E-13
**221**	2.13595507E-11	6.24985288E-12	1.84525334E-12	8.59433644E-13	2.93221465E-13
**222**	2.14520165E-11	6.28229082E-12	1.85635842E-12	8.64309716E-13	2.94889641E-13
**223**	2.15441031E-11	6.31461347E-12	1.86743311E-12	8.69169775E-13	2.96552714E-13
**224**	2.16358082E-11	6.34681976E-12	1.87847694E-12	8.7401364E-13	2.98210619E-13
**225**	2.17271294E-11	6.37890865E-12	1.88948943E-12	8.78841134E-13	2.99863294E-13
**226**	2.18180645E-11	6.41087912E-12	1.90047015E-12	8.83652083E-13	3.01510676E-13
**227**	2.19086113E-11	6.44273018E-12	1.91141863E-12	8.88446317E-13	3.03152706E-13
**228**	2.19987675E-11	6.47446086E-12	1.92233446E-12	8.93223672E-13	3.04789324E-13
**229**	2.20885311E-11	6.50607022E-12	1.9332172E-12	8.97983984E-13	3.06420472E-13
**230**	2.21779001E-11	6.53755732E-12	1.94406642E-12	9.02727098E-13	3.08046095E-13
**231**	2.22668726E-11	6.56892129E-12	1.95488174E-12	9.07452858E-13	3.09666135E-13
**232**	2.23554465E-11	6.60016123E-12	1.96566273E-12	9.12161116E-13	3.1128054E-13
**233**	2.24436201E-11	6.63127631E-12	1.97640901E-12	9.16851723E-13	3.12889257E-13
**234**	2.25313915E-11	6.66226569E-12	1.9871202E-12	9.21524539E-13	3.14492234E-13
**235**	2.26187591E-11	6.69312858E-12	1.99779592E-12	9.26179424E-13	3.1608942E-13
**236**	2.27057212E-11	6.72386418E-12	2.00843581E-12	9.30816242E-13	3.17680767E-13
**237**	2.27922761E-11	6.75447175E-12	2.01903949E-12	9.35434864E-13	3.19266227E-13
**238**	2.28784223E-11	6.78495054E-12	2.02960664E-12	9.4003516E-13	3.20845752E-13
**239**	2.29641583E-11	6.81529984E-12	2.04013689E-12	9.44617006E-13	3.22419298E-13
**240**	2.30494826E-11	6.84551896E-12	2.05062993E-12	9.49180281E-13	3.2398682E-13
**241**	2.31343939E-11	6.87560723E-12	2.06108541E-12	9.53724869E-13	3.25548275E-13
**242**	2.32188909E-11	6.90556399E-12	2.07150303E-12	9.58250654E-13	3.27103621E-13
**243**	2.33029722E-11	6.93538863E-12	2.08188247E-12	9.62757528E-13	3.28652816E-13
**244**	2.33866365E-11	6.96508052E-12	2.09222344E-12	9.67245382E-13	3.30195822E-13
**245**	2.34698829E-11	6.99463909E-12	2.10252563E-12	9.71714113E-13	3.31732599E-13
**246**	2.355271E-11	7.02406376E-12	2.11278876E-12	9.7616362E-13	3.3326311E-13
**247**	2.3635117E-11	7.053354E-12	2.12301255E-12	9.80593806E-13	3.34787319E-13
**248**	2.37171026E-11	7.08250927E-12	2.13319674E-12	9.85004578E-13	3.36305189E-13
**249**	2.3798666E-11	7.11152907E-12	2.14334104E-12	9.89395844E-13	3.37816688E-13
**250**	2.38798063E-11	7.1404129E-12	2.15344521E-12	9.93767517E-13	3.3932178E-13
**251**	2.39605225E-11	7.16916032E-12	2.163509E-12	9.98119512E-13	3.40820436E-13
**252**	2.40408139E-11	7.19777085E-12	2.17353217E-12	1.00245175E-12	3.42312622E-13
**253**	2.41206796E-11	7.22624408E-12	2.18351448E-12	1.00676415E-12	3.4379831E-13
**254**	2.42001191E-11	7.25457958E-12	2.19345569E-12	1.01105663E-12	3.4527747E-13
**255**	2.42791315E-11	7.28277698E-12	2.2033556E-12	1.01532913E-12	3.46750073E-13

**256**	2.43577162E-11	7.31083589E-12	2.21321399E-12	1.01958157E-12	3.48216093E-13
**257**	2.44358727E-11	7.33875595E-12	2.22303064E-12	1.0238139E-12	3.49675504E-13
**258**	2.45136005E-11	7.36653682E-12	2.23280535E-12	1.02802603E-12	3.51128281E-13
**259**	2.45908989E-11	7.39417819E-12	2.24253795E-12	1.03221792E-12	3.52574398E-13
**260**	2.46677676E-11	7.42167975E-12	2.25222822E-12	1.0363895E-12	3.54013834E-13
**261**	2.47442062E-11	7.44904119E-12	2.261876E-12	1.04054072E-12	3.55446565E-13
**262**	2.48202143E-11	7.47626227E-12	2.27148111E-12	1.04467153E-12	3.5687257E-13
**263**	2.48957915E-11	7.50334271E-12	2.28104338E-12	1.04878188E-12	3.58291829E-13
**264**	2.49709377E-11	7.53028228E-12	2.29056264E-12	1.05287171E-12	3.59704322E-13
**265**	2.50456524E-11	7.55708076E-12	2.30003875E-12	1.05694098E-12	3.6111003E-13
**266**	2.51199357E-11	7.58373793E-12	2.30947155E-12	1.06098966E-12	3.62508936E-13
**267**	2.51937871E-11	7.61025361E-12	2.3188609E-12	1.06501769E-12	3.63901021E-13
**268**	2.52672068E-11	7.63662762E-12	2.32820666E-12	1.06902505E-12	3.65286271E-13
**269**	2.53401945E-11	7.66285978E-12	2.3375087E-12	1.07301169E-12	3.6666467E-13
**270**	2.54127502E-11	7.68894997E-12	2.34676689E-12	1.07697759E-12	3.68036204E-13
**271**	2.54848739E-11	7.71489804E-12	2.35598111E-12	1.08092271E-12	3.69400857E-13
**272**	2.55565656E-11	7.74070387E-12	2.36515124E-12	1.08484703E-12	3.70758619E-13
**273**	2.56278255E-11	7.76636736E-12	2.37427719E-12	1.08875052E-12	3.72109476E-13
**274**	2.56986535E-11	7.79188842E-12	2.38335883E-12	1.09263316E-12	3.73453418E-13
**275**	2.57690498E-11	7.81726697E-12	2.39239608E-12	1.09649492E-12	3.74790433E-13
**276**	2.58390146E-11	7.84250295E-12	2.40138883E-12	1.10033578E-12	3.76120512E-13
**277**	2.59085481E-11	7.86759631E-12	2.41033701E-12	1.10415573E-12	3.77443646E-13
**278**	2.59776506E-11	7.89254701E-12	2.41924053E-12	1.10795476E-12	3.78759826E-13
**279**	2.60463222E-11	7.91735502E-12	2.4280993E-12	1.11173285E-12	3.80069045E-13
**280**	2.61145633E-11	7.94202033E-12	2.43691327E-12	1.11548998E-12	3.81371296E-13
**281**	2.61823742E-11	7.96654295E-12	2.44568235E-12	1.11922616E-12	3.82666573E-13
**282**	2.62497552E-11	7.99092288E-12	2.45440649E-12	1.12294137E-12	3.83954869E-13
**283**	2.63167068E-11	8.01516015E-12	2.46308563E-12	1.1266356E-12	3.85236182E-13
**284**	2.63832294E-11	8.0392548E-12	2.47171971E-12	1.13030887E-12	3.86510505E-13
**285**	2.64493235E-11	8.06320687E-12	2.48030869E-12	1.13396115E-12	3.87777836E-13
**286**	2.65149894E-11	8.08701642E-12	2.48885252E-12	1.13759246E-12	3.89038172E-13
**287**	2.65802278E-11	8.11068352E-12	2.49735116E-12	1.1412028E-12	3.9029151E-13
**288**	2.66450391E-11	8.13420826E-12	2.50580458E-12	1.14479217E-12	3.91537849E-13
**289**	2.67094239E-11	8.15759072E-12	2.51421274E-12	1.14836057E-12	3.92777188E-13
**290**	2.67733827E-11	8.180831E-12	2.52257561E-12	1.15190802E-12	3.94009526E-13
**291**	2.68369164E-11	8.20392923E-12	2.53089318E-12	1.15543453E-12	3.95234864E-13
**292**	2.69000253E-11	8.22688552E-12	2.53916543E-12	1.15894009E-12	3.96453203E-13
**293**	2.69627103E-11	8.2497E-12	2.54739234E-12	1.16242474E-12	3.97664543E-13
**294**	2.70249719E-11	8.27237282E-12	2.5555739E-12	1.16588847E-12	3.98868887E-13
**295**	2.7086811E-11	8.29490413E-12	2.5637101E-12	1.16933131E-12	4.00066236E-13
**296**	2.71482283E-11	8.31729409E-12	2.57180094E-12	1.17275328E-12	4.01256595E-13
**297**	2.72092244E-11	8.33954287E-12	2.57984643E-12	1.17615439E-12	4.02439967E-13
**298**	2.72698003E-11	8.36165066E-12	2.58784656E-12	1.17953466E-12	4.03616355E-13
**299**	2.73299566E-11	8.38361763E-12	2.59580135E-12	1.18289411E-12	4.04785764E-13
**300**	2.73896943E-11	8.405444E-12	2.60371081E-12	1.18623277E-12	4.059482E-13
**301**	2.74490142E-11	8.42712996E-12	2.61157495E-12	1.18955066E-12	4.07103668E-13
**302**	2.75079171E-11	8.44867572E-12	2.61939379E-12	1.19284782E-12	4.08252173E-13
**303**	2.7566404E-11	8.47008152E-12	2.62716736E-12	1.19612425E-12	4.09393724E-13
**304**	2.76244757E-11	8.49134759E-12	2.63489568E-12	1.19938E-12	4.10528325E-13
**305**	2.76821332E-11	8.51247415E-12	2.64257878E-12	1.20261509E-12	4.11655986E-13
**306**	2.77393774E-11	8.53346146E-12	2.65021669E-12	1.20582955E-12	4.12776713E-13

**307**	2.77962093E-11	8.55430977E-12	2.65780945E-12	1.20902342E-12	4.13890516E-13
**308**	2.78526299E-11	8.57501934E-12	2.6653571E-12	1.21219673E-12	4.14997402E-13
**309**	2.79086402E-11	8.59559044E-12	2.67285968E-12	1.21534951E-12	4.16097382E-13
**310**	2.79642411E-11	8.61602335E-12	2.68031723E-12	1.2184818E-12	4.17190465E-13
**311**	2.80194339E-11	8.63631834E-12	2.6877298E-12	1.22159364E-12	4.18276661E-13
**312**	2.80742194E-11	8.65647572E-12	2.69509744E-12	1.22468506E-12	4.1935598E-13
**313**	2.81285988E-11	8.67649576E-12	2.70242021E-12	1.2277561E-12	4.20428433E-13
**314**	2.81825732E-11	8.69637879E-12	2.70969816E-12	1.2308068E-12	4.21494032E-13
**315**	2.82361436E-11	8.71612509E-12	2.71693135E-12	1.23383721E-12	4.22552787E-13
**316**	2.82893112E-11	8.735735E-12	2.72411985E-12	1.23684737E-12	4.23604712E-13
**317**	2.83420772E-11	8.75520882E-12	2.73126371E-12	1.23983731E-12	4.24649818E-13
**318**	2.83944425E-11	8.77454689E-12	2.738363E-12	1.24280709E-12	4.25688118E-13
**319**	2.84464086E-11	8.79374954E-12	2.7454178E-12	1.24575675E-12	4.26719625E-13
**320**	2.84979763E-11	8.8128171E-12	2.75242817E-12	1.24868633E-12	4.27744352E-13
**321**	2.85491471E-11	8.83174993E-12	2.75939419E-12	1.25159588E-12	4.28762313E-13
**322**	2.85999221E-11	8.85054836E-12	2.76631594E-12	1.25448545E-12	4.29773523E-13
**323**	2.86503024E-11	8.86921276E-12	2.77319348E-12	1.25735508E-12	4.30777994E-13
**324**	2.87002894E-11	8.88774347E-12	2.78002692E-12	1.26020484E-12	4.31775743E-13
**325**	2.87498843E-11	8.90614088E-12	2.78681632E-12	1.26303476E-12	4.32766784E-13
**326**	2.87990882E-11	8.92440534E-12	2.79356178E-12	1.26584489E-12	4.33751132E-13
**327**	2.88479025E-11	8.94253723E-12	2.80026338E-12	1.2686353E-12	4.34728803E-13
**328**	2.88963285E-11	8.96053693E-12	2.80692121E-12	1.27140603E-12	4.35699813E-13
**329**	2.89443673E-11	8.97840481E-12	2.81353537E-12	1.27415713E-12	4.36664177E-13
**330**	2.89920204E-11	8.99614128E-12	2.82010594E-12	1.27688866E-12	4.37621912E-13
**331**	2.9039289E-11	9.01374671E-12	2.82663302E-12	1.27960068E-12	4.38573035E-13
**332**	2.90861744E-11	9.0312215E-12	2.83311672E-12	1.28229324E-12	4.39517562E-13
**333**	2.9132678E-11	9.04856606E-12	2.83955712E-12	1.28496639E-12	4.40455511E-13
**334**	2.9178801E-11	9.06578079E-12	2.84595434E-12	1.28762019E-12	4.41386899E-13
**335**	2.92245449E-11	9.08286609E-12	2.85230847E-12	1.2902547E-12	4.42311743E-13
**336**	2.92699109E-11	9.09982238E-12	2.85861961E-12	1.29286998E-12	4.43230063E-13
**337**	2.93149004E-11	9.11665006E-12	2.86488789E-12	1.29546609E-12	4.44141874E-13
**338**	2.93595148E-11	9.13334957E-12	2.87111339E-12	1.29804308E-12	4.45047196E-13
**339**	2.94037555E-11	9.14992131E-12	2.87729624E-12	1.30060102E-12	4.45946048E-13
**340**	2.94476238E-11	9.16636571E-12	2.88343654E-12	1.30313996E-12	4.46838448E-13
**341**	2.94911211E-11	9.1826832E-12	2.88953441E-12	1.30565996E-12	4.47724414E-13
**342**	2.95342488E-11	9.19887422E-12	2.89558995E-12	1.30816109E-12	4.48603967E-13
**343**	2.95770083E-11	9.21493919E-12	2.9016033E-12	1.31064341E-12	4.49477125E-13
**344**	2.9619401E-11	9.23087855E-12	2.90757456E-12	1.31310699E-12	4.50343908E-13
**345**	2.96614284E-11	9.24669274E-12	2.91350384E-12	1.31555187E-12	4.51204335E-13
**346**	2.97030917E-11	9.26238221E-12	2.91939128E-12	1.31797813E-12	4.52058426E-13
**347**	2.97443926E-11	9.27794739E-12	2.925237E-12	1.32038583E-12	4.52906202E-13
**348**	2.97853323E-11	9.29338874E-12	2.9310411E-12	1.32277504E-12	4.53747682E-13
**349**	2.98259123E-11	9.3087067E-12	2.93680373E-12	1.32514581E-12	4.54582888E-13
**350**	2.98661341E-11	9.32390173E-12	2.942525E-12	1.32749822E-12	4.55411838E-13
**351**	2.99059992E-11	9.33897427E-12	2.94820504E-12	1.32983233E-12	4.56234555E-13
**352**	2.99455088E-11	9.3539248E-12	2.95384397E-12	1.3321482E-12	4.57051058E-13
**353**	2.99846646E-11	9.36875375E-12	2.95944193E-12	1.3344459E-12	4.57861369E-13
**354**	3.00234679E-11	9.3834616E-12	2.96499904E-12	1.33672549E-12	4.58665509E-13
**355**	3.00619202E-11	9.39804881E-12	2.97051543E-12	1.33898705E-12	4.59463499E-13
**356**	3.0100023E-11	9.41251584E-12	2.97599124E-12	1.34123064E-12	4.6025536E-13
**357**	3.01377777E-11	9.42686315E-12	2.9814266E-12	1.34345633E-12	4.61041114E-13

**358**	3.01751859E-11	9.44109122E-12	2.98682163E-12	1.34566418E-12	4.61820783E-13
**359**	3.02122489E-11	9.45520051E-12	2.99217648E-12	1.34785427E-12	4.62594387E-13
**360**	3.02489683E-11	9.46919149E-12	2.99749128E-12	1.35002665E-12	4.6336195E-13
**361**	3.02853456E-11	9.48306464E-12	3.00276617E-12	1.35218141E-12	4.64123492E-13
**362**	3.03213821E-11	9.49682043E-12	3.00800127E-12	1.3543186E-12	4.64879036E-13
**363**	3.03570795E-11	9.51045933E-12	3.01319674E-12	1.35643829E-12	4.65628604E-13
**364**	3.03924391E-11	9.52398181E-12	3.0183527E-12	1.35854057E-12	4.66372219E-13
**365**	3.04274626E-11	9.53738837E-12	3.0234693E-12	1.36062549E-12	4.67109902E-13
**366**	3.04621512E-11	9.55067947E-12	3.02854668E-12	1.36269312E-12	4.67841675E-13
**367**	3.04965067E-11	9.56385559E-12	3.03358497E-12	1.36474353E-12	4.68567562E-13
**368**	3.05305303E-11	9.57691722E-12	3.03858432E-12	1.3667768E-12	4.69287586E-13
**369**	3.05642237E-11	9.58986483E-12	3.04354486E-12	1.368793E-12	4.70001767E-13
**370**	3.05975883E-11	9.6026989E-12	3.04846675E-12	1.37079218E-12	4.7071013E-13
**371**	3.06306257E-11	9.61541993E-12	3.05335013E-12	1.37277443E-12	4.71412697E-13
**372**	3.06633373E-11	9.62802839E-12	3.05819513E-12	1.37473982E-12	4.72109492E-13
**373**	3.06957245E-11	9.64052477E-12	3.06300191E-12	1.37668841E-12	4.72800536E-13
**374**	3.0727789E-11	9.65290955E-12	3.0677706E-12	1.37862028E-12	4.73485852E-13
**375**	3.07595322E-11	9.66518322E-12	3.07250135E-12	1.38053549E-12	4.74165465E-13
**376**	3.07909556E-11	9.67734626E-12	3.07719431E-12	1.38243412E-12	4.74839397E-13
**377**	3.08220607E-11	9.68939916E-12	3.08184962E-12	1.38431624E-12	4.7550767E-13
**378**	3.0852849E-11	9.7013424E-12	3.08646743E-12	1.38618192E-12	4.76170309E-13
**379**	3.0883322E-11	9.71317648E-12	3.09104789E-12	1.38803123E-12	4.76827337E-13
**380**	3.09134811E-11	9.72490189E-12	3.09559114E-12	1.38986425E-12	4.77478776E-13
**381**	3.0943328E-11	9.7365191E-12	3.10009734E-12	1.39168104E-12	4.78124651E-13
**382**	3.0972864E-11	9.7480286E-12	3.10456662E-12	1.39348167E-12	4.78764984E-13
**383**	3.10020907E-11	9.75943089E-12	3.10899914E-12	1.39526622E-12	4.79399799E-13
**384**	3.10310095E-11	9.77072646E-12	3.11339505E-12	1.39703476E-12	4.80029119E-13
**385**	3.1059622E-11	9.78191578E-12	3.11775449E-12	1.39878736E-12	4.80652968E-13
**386**	3.10879297E-11	9.79299936E-12	3.12207761E-12	1.40052409E-12	4.81271369E-13
**387**	3.1115934E-11	9.80397767E-12	3.12636457E-12	1.40224502E-12	4.81884345E-13
**388**	3.11436364E-11	9.81485121E-12	3.13061552E-12	1.40395023E-12	4.82491921E-13
**389**	3.11710385E-11	9.82562046E-12	3.13483059E-12	1.40563978E-12	4.8309412E-13
**390**	3.11981416E-11	9.83628591E-12	3.13900995E-12	1.40731376E-12	4.83690965E-13
**391**	3.12249473E-11	9.84684805E-12	3.14315375E-12	1.40897222E-12	4.8428248E-13
**392**	3.12514571E-11	9.85730737E-12	3.14726212E-12	1.41061524E-12	4.84868688E-13
**393**	3.12776724E-11	9.86766435E-12	3.15133524E-12	1.4122429E-12	4.85449613E-13
**394**	3.13035948E-11	9.87791947E-12	3.15537323E-12	1.41385526E-12	4.86025279E-13
**395**	3.13292256E-11	9.88807324E-12	3.15937627E-12	1.4154524E-12	4.86595708E-13
**396**	3.13545664E-11	9.89812613E-12	3.16334449E-12	1.41703439E-12	4.87160926E-13
**397**	3.13796187E-11	9.90807863E-12	3.16727805E-12	1.4186013E-12	4.87720955E-13
**398**	3.14043839E-11	9.91793122E-12	3.1711771E-12	1.42015319E-12	4.88275819E-13
**399**	3.14288634E-11	9.92768438E-12	3.17504179E-12	1.42169015E-12	4.88825541E-13
**400**	3.14530588E-11	9.93733861E-12	3.17887228E-12	1.42321224E-12	4.89370146E-13
**401**	3.14769715E-11	9.94689439E-12	3.1826687E-12	1.42471954E-12	4.89909656E-13
**402**	3.1500603E-11	9.95635219E-12	3.18643123E-12	1.42621211E-12	4.90444095E-13
**403**	3.15239547E-11	9.9657125E-12	3.19015999E-12	1.42769003E-12	4.90973488E-13
**404**	3.15470281E-11	9.97497581E-12	3.19385516E-12	1.42915337E-12	4.91497856E-13
**405**	3.15698246E-11	9.98414259E-12	3.19751687E-12	1.43060219E-12	4.92017224E-13
**406**	3.15923457E-11	9.99321332E-12	3.20114529E-12	1.43203658E-12	4.92531616E-13
**407**	3.16145927E-11	1.00021885E-11	3.20474056E-12	1.4334566E-12	4.93041054E-13
**408**	3.16365673E-11	1.00110686E-11	3.20830282E-12	1.43486232E-12	4.93545563E-13

**409**	3.16582708E-11	1.0019854E-11	3.21183225E-12	1.43625381E-12	4.94045166E-13
**410**	3.16797045E-11	1.00285454E-11	3.21532897E-12	1.43763114E-12	4.94539885E-13
**411**	3.17008701E-11	1.0037143E-11	3.21879316E-12	1.43899438E-12	4.95029745E-13
**412**	3.17217688E-11	1.00456475E-11	3.22222495E-12	1.44034361E-12	4.95514769E-13
**413**	3.17424022E-11	1.00540593E-11	3.22562449E-12	1.4416789E-12	4.9599498E-13
**414**	3.17627716E-11	1.00623789E-11	3.22899195E-12	1.4430003E-12	4.96470402E-13
**415**	3.17828785E-11	1.00706067E-11	3.23232746E-12	1.4443079E-12	4.96941058E-13
**416**	3.18027242E-11	1.00787432E-11	3.23563118E-12	1.44560176E-12	4.9740697E-13
**417**	3.18223102E-11	1.00867889E-11	3.23890326E-12	1.44688195E-12	4.97868163E-13
**418**	3.18416379E-11	1.00947442E-11	3.24214385E-12	1.44814855E-12	4.98324659E-13
**419**	3.18607087E-11	1.01026096E-11	3.2453531E-12	1.44940161E-12	4.98776482E-13
**420**	3.1879524E-11	1.01103857E-11	3.24853115E-12	1.45064122E-12	4.99223654E-13
**421**	3.18980852E-11	1.01180728E-11	3.25167817E-12	1.45186744E-12	4.99666199E-13
**422**	3.19163936E-11	1.01256714E-11	3.25479428E-12	1.45308033E-12	5.00104139E-13
**423**	3.19344508E-11	1.0133182E-11	3.25787966E-12	1.45427998E-12	5.00537498E-13
**424**	3.19522579E-11	1.0140605E-11	3.26093444E-12	1.45546643E-12	5.00966299E-13
**425**	3.19698165E-11	1.01479409E-11	3.26395877E-12	1.45663978E-12	5.01390564E-13
**426**	3.19871279E-11	1.01551903E-11	3.26695281E-12	1.45780007E-12	5.01810317E-13
**427**	3.20041934E-11	1.01623534E-11	3.26991669E-12	1.45894739E-12	5.0222558E-13
**428**	3.20210145E-11	1.01694308E-11	3.27285057E-12	1.46008179E-12	5.02636375E-13
**429**	3.20375925E-11	1.0176423E-11	3.2757546E-12	1.46120335E-12	5.03042726E-13
**430**	3.20539288E-11	1.01833303E-11	3.27862892E-12	1.46231213E-12	5.03444655E-13
**431**	3.20700247E-11	1.01901533E-11	3.28147367E-12	1.46340821E-12	5.03842184E-13
**432**	3.20858815E-11	1.01968924E-11	3.28428902E-12	1.46449164E-12	5.04235337E-13
**433**	3.21015006E-11	1.02035481E-11	3.28707509E-12	1.4655625E-12	5.04624136E-13
**434**	3.21168834E-11	1.02101208E-11	3.28983205E-12	1.46662085E-12	5.05008603E-13
**435**	3.21320312E-11	1.02166109E-11	3.29256002E-12	1.46766676E-12	5.0538876E-13
**436**	3.21469453E-11	1.02230189E-11	3.29525917E-12	1.4687003E-12	5.05764631E-13
**437**	3.2161627E-11	1.02293453E-11	3.29792963E-12	1.46972152E-12	5.06136236E-13
**438**	3.21760777E-11	1.02355905E-11	3.30057156E-12	1.47073051E-12	5.06503599E-13
**439**	3.21902986E-11	1.02417549E-11	3.30318508E-12	1.47172732E-12	5.06866741E-13
**440**	3.22042912E-11	1.0247839E-11	3.30577036E-12	1.47271202E-12	5.07225685E-13
**441**	3.22180566E-11	1.02538432E-11	3.30832753E-12	1.47368467E-12	5.07580452E-13
**442**	3.22315963E-11	1.0259768E-11	3.31085673E-12	1.47464534E-12	5.07931065E-13
**443**	3.22449114E-11	1.02656137E-11	3.31335811E-12	1.4755941E-12	5.08277546E-13
**444**	3.22580034E-11	1.02713809E-11	3.31583182E-12	1.47653101E-12	5.08619916E-13
**445**	3.22708734E-11	1.027707E-11	3.31827798E-12	1.47745613E-12	5.08958196E-13
**446**	3.22835229E-11	1.02826814E-11	3.32069676E-12	1.47836954E-12	5.0929241E-13
**447**	3.22959529E-11	1.02882155E-11	3.32308828E-12	1.47927128E-12	5.09622578E-13
**448**	3.23081649E-11	1.02936728E-11	3.32545269E-12	1.48016144E-12	5.09948723E-13
**449**	3.23201601E-11	1.02990537E-11	3.32779014E-12	1.48104007E-12	5.10270864E-13
**450**	3.23319398E-11	1.03043586E-11	3.33010075E-12	1.48190723E-12	5.10589025E-13
**451**	3.23435052E-11	1.0309588E-11	3.33238467E-12	1.48276299E-12	5.10903227E-13
**452**	3.23548576E-11	1.03147422E-11	3.33464205E-12	1.48360741E-12	5.1121349E-13
**453**	3.23659982E-11	1.03198217E-11	3.33687301E-12	1.48444056E-12	5.11519836E-13
**454**	3.23769282E-11	1.0324827E-11	3.3390777E-12	1.48526249E-12	5.11822286E-13
**455**	3.2387649E-11	1.03297583E-11	3.34125626E-12	1.48607328E-12	5.12120862E-13
**456**	3.23981618E-11	1.03346163E-11	3.34340883E-12	1.48687297E-12	5.12415584E-13
**457**	3.24084677E-11	1.03394012E-11	3.34553554E-12	1.48766164E-12	5.12706474E-13
**458**	3.24185681E-11	1.03441135E-11	3.34763653E-12	1.48843935E-12	5.12993552E-13
**459**	3.2428464E-11	1.03487536E-11	3.34971193E-12	1.48920616E-12	5.13276839E-13

**460**	3.24381569E-11	1.0353322E-11	3.35176189E-12	1.48996212E-12	5.13556355E-13
**461**	3.24476477E-11	1.03578189E-11	3.35378654E-12	1.49070731E-12	5.13832123E-13
**462**	3.24569379E-11	1.03622449E-11	3.35578602E-12	1.49144178E-12	5.14104162E-13
**463**	3.24660285E-11	1.03666004E-11	3.35776045E-12	1.49216559E-12	5.14372492E-13
**464**	3.24749208E-11	1.03708857E-11	3.35970998E-12	1.4928788E-12	5.14637135E-13
**465**	3.24836159E-11	1.03751012E-11	3.36163474E-12	1.49358148E-12	5.14898111E-13
**466**	3.24921151E-11	1.03792474E-11	3.36353487E-12	1.49427368E-12	5.1515544E-13
**467**	3.25004195E-11	1.03833247E-11	3.36541048E-12	1.49495546E-12	5.15409142E-13
**468**	3.25085303E-11	1.03873334E-11	3.36726173E-12	1.49562688E-12	5.15659238E-13
**469**	3.25164487E-11	1.0391274E-11	3.36908874E-12	1.49628801E-12	5.15905747E-13
**470**	3.25241758E-11	1.03951468E-11	3.37089164E-12	1.4969389E-12	5.1614869E-13
**471**	3.25317128E-11	1.03989523E-11	3.37267057E-12	1.49757961E-12	5.16388086E-13
**472**	3.25390609E-11	1.04026909E-11	3.37442566E-12	1.49821019E-12	5.16623957E-13
**473**	3.25462211E-11	1.04063628E-11	3.37615703E-12	1.49883072E-12	5.1685632E-13
**474**	3.25531947E-11	1.04099686E-11	3.37786482E-12	1.49944123E-12	5.17085197E-13
**475**	3.25599828E-11	1.04135086E-11	3.37954915E-12	1.50004181E-12	5.17310607E-13
**476**	3.25665866E-11	1.04169832E-11	3.38121016E-12	1.50063249E-12	5.17532569E-13
**477**	3.2573007E-11	1.04203928E-11	3.38284797E-12	1.50121334E-12	5.17751103E-13
**478**	3.25792454E-11	1.04237377E-11	3.38446272E-12	1.50178442E-12	5.17966228E-13
**479**	3.25853028E-11	1.04270184E-11	3.38605453E-12	1.50234577E-12	5.18177964E-13
**480**	3.25911802E-11	1.04302352E-11	3.38762352E-12	1.50289747E-12	5.1838633E-13
**481**	3.25968789E-11	1.04333886E-11	3.38916983E-12	1.50343957E-12	5.18591346E-13
**482**	3.26024E-11	1.04364788E-11	3.39069358E-12	1.50397211E-12	5.18793029E-13
**483**	3.26077444E-11	1.04395063E-11	3.3921949E-12	1.50449517E-12	5.18991401E-13
**484**	3.26129134E-11	1.04424714E-11	3.39367391E-12	1.50500878E-12	5.19186478E-13
**485**	3.26179081E-11	1.04453745E-11	3.39513074E-12	1.50551302E-12	5.19378282E-13
**486**	3.26227294E-11	1.0448216E-11	3.39656551E-12	1.50600793E-12	5.19566829E-13
**487**	3.26273785E-11	1.04509962E-11	3.39797834E-12	1.50649357E-12	5.19752139E-13
**488**	3.26318564E-11	1.04537156E-11	3.39936936E-12	1.50697E-12	5.19934231E-13
**489**	3.26361643E-11	1.04563744E-11	3.4007387E-12	1.50743727E-12	5.20113123E-13
**490**	3.26403032E-11	1.04589731E-11	3.40208647E-12	1.50789543E-12	5.20288834E-13
**491**	3.26442741E-11	1.04615119E-11	3.40341279E-12	1.50834453E-12	5.20461382E-13
**492**	3.26480781E-11	1.04639914E-11	3.40471779E-12	1.50878464E-12	5.20630786E-13
**493**	3.26517163E-11	1.04664118E-11	3.40600159E-12	1.50921581E-12	5.20797064E-13
**494**	3.26551897E-11	1.04687734E-11	3.40726432E-12	1.50963808E-12	5.20960234E-13
**495**	3.26584994E-11	1.04710767E-11	3.40850608E-12	1.51005151E-12	5.21120314E-13
**496**	3.26616463E-11	1.0473322E-11	3.40972699E-12	1.51045616E-12	5.21277322E-13
**497**	3.26646315E-11	1.04755096E-11	3.41092719E-12	1.51085208E-12	5.21431276E-13
**498**	3.2667456E-11	1.04776399E-11	3.41210679E-12	1.51123932E-12	5.21582195E-13
**499**	3.26701209E-11	1.04797133E-11	3.41326589E-12	1.51161793E-12	5.21730095E-13
**500**	3.26726272E-11	1.048173E-11	3.41440463E-12	1.51198796E-12	5.21874996E-13
